# Assessing the Effectiveness of Correlative Ecological Niche Model Temporal Projection through Floristic Data

**DOI:** 10.3390/biology11081219

**Published:** 2022-08-14

**Authors:** David Dolci, Lorenzo Peruzzi

**Affiliations:** 1 Department of Biology, University of Pisa, Via Derna 1, 56126 Pisa, Italy; 2 Centro Interuniversitario per la Biodiversità Vegetale Big Data-PLANT DATA, Department of Biological, Geological and Environmental Sciences, Alma Mater Studiorum University of Bologna, Via Irnerio 42, 40126 Bologna, Italy; 3 CIRSEC, Centre for Climatic Change Impact, University of Pisa, Via del Borghetto 80, 56124 Pisa, Italy

**Keywords:** algorithms, distribution, endemic species, ENM, plant species, projection, SDM

## Abstract

**Simple Summary:**

Climate change is the main threat for conservation in the 21st century. Reliable methodologies and tools for the evaluation of its impact are urgently needed. Correlative ecological niche models (ENMs) are effective tools for predicting the future distribution of species under climate change scenarios. Despite this, many alternative different methods have been proposed, and objective reasons for a proper selection are unclear. Therefore, a comparative study to evaluate the consistency of predictions of the main ENM algorithms was performed. To test the effectiveness of correlative ENM temporal projection, we compared predictions generated using historical data and projected to the modern climate with predictions generated using modern distribution and climate data. In total, 600 case studies were generated, by using 25 Italian endemic plant species, 12 algorithms and 2 alternative sets of environmental variables. As a result, we highlighted the similarity of eight algorithms and the poor performance of four.

**Abstract:**

Correlative ecological niche modelling (ENM) is a method widely used to study the geographic distribution of species. In recent decades, it has become a leading approach for evaluating the most likely impacts of changing climate. When used to predict future distributions, ENM applications involve transferring models calibrated with modern environmental data to future conditions, usually derived from Global Climate Models (GCMs). The number of algorithms and software packages available to estimate distributions is quite high. To experimentally assess the effectiveness of correlative ENM temporal projection, we evaluated the transferability of models produced using 12 different algorithms on historical and modern data. In particular, we compared predictions generated using historical data and projected to the modern climate (simulating a “future” condition) with predictions generated using modern distribution and climate data. The models produced with the 12 ENM algorithms were evaluated in geographic (range size and coherence of predictions) and environmental space (Schoener’s D index). None of the algorithms shows an overall superior capability to correctly predict future distributions. On the contrary, a few algorithms revealed an inadequate predictive ability. Finally, we provide hints that can be used as guideline to plan further studies based on the adopted general workflow, useful for all studies involving future projections.

## 1. Introduction

Correlative ecological niche models (ENMs) [1] are methods used to achieve insights on niche and potential distribution areas of living organisms. Modern ENMs rely on the possibility of using large databases of primary biodiversity occurrence data together with geospatial environmental variables, to estimate coarse-grained aspects of niche dimensions [2], by exploiting the statistical association between spatial environmental data and occurrence records. These methods experienced a remarkable growth in the latest years, due to ready-to-use data availability [3]. Global-scale databases such as the Global Biodiversity Information Facility (GBIF) [4] and WorldClim [5] boosted the use of ENMs for different purposes. Further reasons for the acceleration in the use of ENM techniques is that these techniques proved their predictive capacity in many situations [6,7,8,9,10]. The climate change debate and the need to find ways to answer questions about future scenarios further increased the expansion of these methods [11], and led to an explosion of studies in the field of ENMs in the last decade [12,13,14,15,16]. In fact, correlative models are one of the most important tools currently available to assess the potential impacts of climate change on species distribution [17]. These studies have been boosted by the availability of ready-to-use datasets for future climatic conditions, generated by applying Global Climate Models (GCMs, also known as General Circulation Models) to the modern climate [18]. Commonly, models trained on modern conditions are transferred to future scenarios to assess changes in future potential distribution caused by different emission scenarios (e.g., [19]). Although this approach is widely adopted, potential distributions may be over- or under-predicted [11], depending on the modelling algorithm used. Numerous different algorithms were used to estimate the ecological niche of a species as a function of a suite of environmental variables [20], as summarized by Franklin [21] based on the underlying theories. Other differences rely on the three main possible types of biological input data available: presence-absence, presence-background, and presence-only methods [20]. Presence-absence methods use observations of species occurrences and absences (i.e. places where a species is actually missing). These methods discern the environmental conditions between occupied and non-occupied habitats, providing the probability of finding a species at each place in the study area [22]. Presence-background methods compare the available environmental conditions in the study area (i.e., background) with the conditions used by the species [23,24]. Sometimes presence-background methods are considered presence-only methods, but there are actually very few true presence-only methods [25], and the most common are envelope- and distance-based approaches. Presence-background methods are neither presence-only nor pseudoabsence methods [26]. Background records do not imply species pseudoabsences, but rather a spectrum of the overall available conditions [27,28,29]. The background is the whole study area, including those cells with presences. Since absence data are very difficult to obtain, presence-only methods have often been used [30], by creating artificial absence data (usually called pseudoabsence data). To cope with this conceptual mistake, different strategies have been proposed to improve the selection of an appropriate pseudoabsence dataset [25].

A large variety of algorithms (presence only, presence-absence, and presence -background) have been used for ENM studies [20,21,31,32]. Over the years, a few of them became widely used and have been considered as best practices. Modern ENM studies began with BIOCLIM, released in 1984 [33]. Then, the GARP Modelling System (GMS) [34] was developed, and in the following years its use became widespread [32,35]. More recently, the MAXENT approach to ENM studies [35] was proposed and, since then, it became the standard for correlative ENM development.

However, the variety of existing algorithms produce different results because of the diversity in how they represent the relationship between environmental conditions and species occurrences [20,36]. For this reason, there is still a lack of consensus on the “best” algorithm to use [37]. Nevertheless, most ENM studies lack true absence data [38,39,40,41]. Additional issues in algorithm selection are related to model transferability, i.e. the projection of a model to different places or times. Multiple studies compared the transferability of different ENM algorithms based on occurrence data of real [42,43,44,45,46] and virtual species [47]. However, most of these studies were focused on a few algorithms or a few species and, as a consequence, comparative data available for inferences was limited. Qiao et al. [47] performed the most exhaustive evaluation of transferability by using virtual species, in order to overcome all common problems that affect real species (e.g., sampling bias or limited sample size) (Table 1).

Further issues in ENM studies are related to environmental variable selection. Different methods and practices were proposed, and the recommended best practices changed over the years. Since the pioneering work by Busby [48,49], the use of a standard set of 19 variables soon became a common practice [33]. Over the years, different strategies have been developed for selecting variables. Some authors proposed to use large datasets [50], others to use a few preselected and uncorrelated variables [51]. In recent years, the use of a high number of variables received support [52], as well as the use of dimensionality reduction via principal component analysis (PCA) on a set of variables, followed by selection of the most important PCA axes as input for modelling [47].

The first objective in this paper is to evaluate the effectiveness of several, commonly used, ENM algorithms when applied to the task of predicting future distributions, taking advantage of historical and modern distribution and climatic data. In particular, to evaluate differences in algorithm responses, we planned an analysis based on the comparison of models constructed with historical data projected to modern climatic conditions (i.e. a real “future” condition), and models constructed with modern data. In this way, we addressed an experimental check of the effectiveness of model transferability to future conditions, here simulated by the modern climate. Differently from other studies [42,43,44,45,46,47], this approach enables evaluating transferability by direct comparison of habitat suitability maps, that represent the focus of most investigations related with forecasting future distribution under climate change scenarios. We expect that “best” algorithms should return distributions as similar as possible among the two models. The second objective of this study is to evaluate the impact of variable selection on transferability. To achieve this, we repeated the analyses with two alternative sets of variables, the first based on the use of a few uncorrelated and biologically meaningful variables and the second based on the use of dimensionality reduction via PCA, starting from a large set of variables. We expect slight differences in results between the two sets of variables.

## 2. Materials and Methods

The transferability of models produced by 12 different ENM algorithms was tested by using historical and modern data. Biological data consisted of a selection of plant species endemic to Italy, showing a well-known distribution. In total, 25 species were selected to be modelled with each algorithm. Environmental data consisted of monthly climate time series used to obtain derived variables. Two sets of variables were used to generate models: the first was composed of 3 a priori selected variables, and the second of 35 variables subjected to PCA. These environmental variables were composed of 19 bioclimatic variables [48,49], plus 16 complementary variables [53], particularly relevant in order to predict more accurate potential distributions [54]. For each algorithm and species a couple of models was produced (historical-projected and modern), and then compared. In all cases, a standardized workflow was applied. Tests of statistical significance were used to check for significant differences.

### 2.1. Study Area and Environmental Variables

Climate time series were collected from CHELSAcruts (1901–2016) [18,55], a delta change monthly climate dataset covering the years 1901–2016 for mean monthly maximum temperatures, mean monthly minimum temperatures, and monthly precipitation sum. Both historical and modern time series were collected to gather 30 years of data. We considered the “historical climate” as that for the period 1901–1930, and the “modern climate” as that for the period 1981–2010. Cumulative monthly precipitation and monthly maximum and minimum temperature at 30″ (DMS) spatial resolution were used to create geospatial environmental variables by using DISMO [56] and ENVIREM [53] packages. A total of 35 climatic variables were obtained for historical and modern times (Supplementary Material, Table S1A).

**Figure 1:**
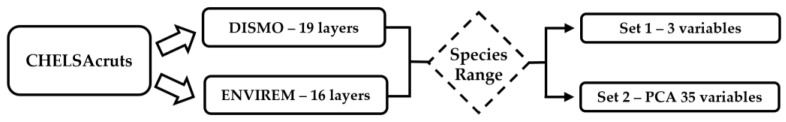
Schematization of the workflow used to generate the two sets of environmental layers. Climate time series from CHELSAcruts were used to generate 35 layers. Then, two sets of variables were organized for two groups of experiments carried out in parallel. The first set was composed of only 3 variables (annual mean temperature, annual precipitation and annual potential evapotranspiration), and the second set was composed of all 35 variables and then converted in 3 summary layers by applying a PCA ordination. Each of the two sets of variables was tailored to fit the known distribution of each species. The procedure was applied to both historical and a modern set of environmental layers.

Environmental layers were tailored based on the distribution of the modelled species (see below), obtaining 25 sets of climatic variables. The 25 study areas included different portions of Italy: 16 the main portion of Italian peninsula, 6 the southern portion and the island of Sicily, 2 the northern portion, and only 1 the whole of Italy. Each study area was selected based on each species range. The main modelling experiment was replicated with two combinations of environmental layers. The first group was composed of only 3 environmental layers (annual mean temperature, annual precipitation, and annual potential evapotranspiration) a priori selected for their biological relevance, according to Barbet-Massin and Jetz [54] and Warren et al. [52]. The second group was composed of the first three axes of a PCA of the 19 + 16 environmental variables calculated with DISMO and ENVIREM (Figure 1). PCA was used to reduce dimensionality and collinearity among variables and to improve transferability of models [47,57,58]. The PCA was performed by using the RSTOOLBOX package [59]. A standardization was applied to equally weight all layers. All cells of the starting raster layers were sampled to compute PCA. The two sets of climatic variables (related to historical and modern times) were transformed by PCA independently from each other. Overall, 50 sets of raster layers were obtained for both historical and modern times.

### 2.2. Study Species and Occurrence Data

We selected 25 plant species endemic to Italy [60,61,62,63,64,65,66,67,68,69,70] (Supplementary Material, Table S1B). These species were preferred for their limited and well-known distribution. The occurrence records were collected from herbarium specimens, literature, and field observations. Most of the records were obtained consulting Wikiplantbase #Italia [71], the Global Biodiversity Information Facility [72], JACQ-Virtual Herbaria [73], and additional botanical literature. Only those records dating back before 1930 or dating after 1980 were retained, in order to obtain two sets of data for each species: one related to historical times, and another related to modern times (Supplementary Material, Table S1C). On average, 52% of records came from literature data, 22% from herbarium specimens, and 26% from observations. Only those observations judged reliable were taken into account, considering the (well-known) distribution range of the selected species and the source of information (expert botanists). For each species and temporal dataset, only one record per grid cell was retained. Environmental outliers were detected and removed by modelling the ellipsoid niche and calculating the Mahalanobis distance from centroids with the NTBOX package [74]. Moreover, the two datasets were also uniform in terms of the number of records by selecting a subset of occurrence records who maximized the niche overlap between historical and modern datasets, calculated with the ELLIPSENM package [75]. The modern occurrence data discarded by the last cleaning step were used as test data, to evaluate the model prediction in modern times. Since the filtration in the E-space depended on the selected environmental variables, two different datasets were obtained for each species (Supplementary Material, Table S1D).

### 2.3. Algorithms and Packages

Habitat suitability maps were calculated using 12 different algorithms. These algorithms are: Bioclim [33,48,49], Domain [76], Generalized Linear Models (GLM) [77,78], Generalized Additive Models (GAM) [78,79], Multivariate Adaptive Regression Splines (MARS) [80,81,82], Flexible Discriminant Analysis (FDA) [83,84,85], Classification Tree Analysis (CTA) [86], Artificial Neural Network (ANN) [87,88], Random Forest (RF) [89,90], Support Vector Machine (SVM) [91,92,93], Maximum Entropy (Maxent) [24,35,94], and Kernel Density Estimation (KDE) [95,96]. All algorithms were used in R [97]. Bioclim, Domain, and Maxent were run by using the DISMO package [56]. GLM, GAM, MARS, RF, and SVM were run by using the SSDM package [6]. FDA and CTA were run by using the SDM package [98]. KDE was run by using the HYPERVOLUME package [95]. A nine-algorithm ensemble approach (GLM, GBM, GAM, MARS, MAXENT, RF, CTA, ANN, and FDA) was also performed by using the Biomod2 package [99,100,101].

### 2.4. Modelling Procedures

**Figure 2:**
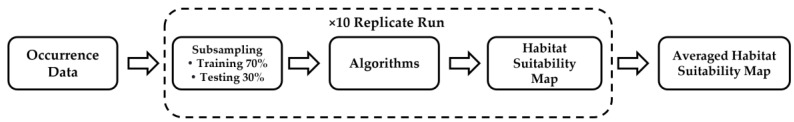
General scheme of the adopted modelling workflow.

A single workflow was adopted in order to obtain comparable results among different algorithms. For each species and for each set of data (‘historical’ and ‘modern’ datasets), the workflow drafted in Figure 2 was applied.

Each set of data was initially split in a training set and a testing set [20], and then a habitat suitability map was calculated by using one of the algorithms. By repeating this step 10 times, an averaged habitat suitability map was obtained. The basic workflow was applied to 12 different procedures, 11 of them based on single algorithms and 1 on ensemble algorithms (Table 2). To cope with the lack of real absence data where needed (see Table 2), we randomly generated pseudoabsence occurrences in the study area [25,34]. For each procedure, the default or commonly used settings were used, because our goal was to compare different methods and algorithms under those commonly adopted parameters widely used by the scientific community (Table 2). All averaged habitat suitability maps were converted into binary maps by applying a probabilistic approach, in which a logistic function was used to convert environmental suitability into a probability of occurrence [102]. For each species, the appropriate conversion curve was automatically determined based on of the calculated species prevalence.

**Figure 3:**
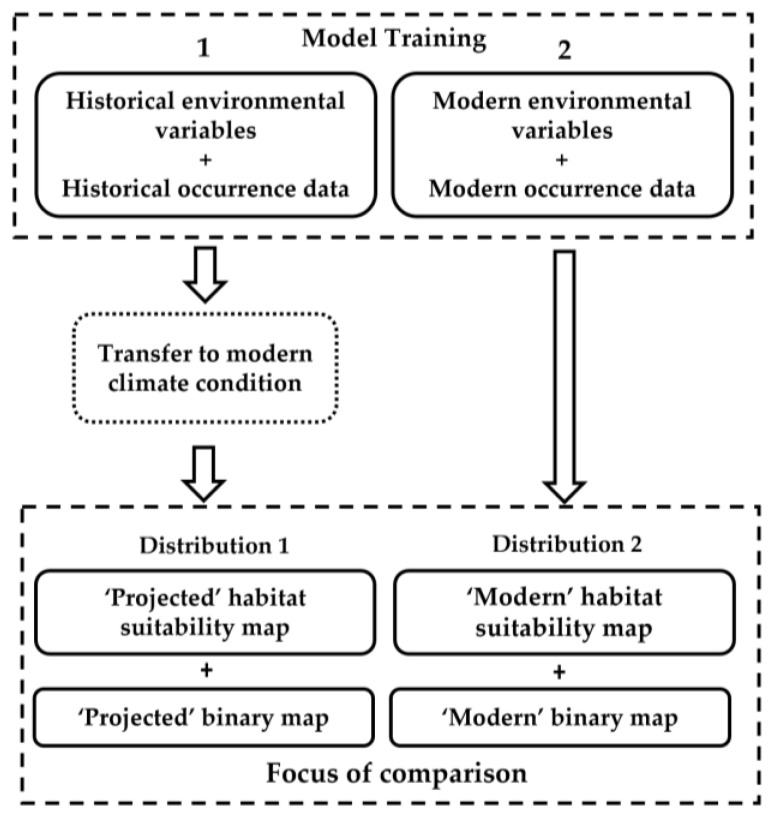
General scheme of the comparison of potential distributions.

For each species and for each modelling procedure, a pair of present potential distributions was obtained and then compared (Figure 3: Distributions 1 and 2). The first was obtained with historical biological data (1910–1930) and historical environmental variables projected to modern climatic conditions. It represents a predicted ‘future’ distribution of a species based on historical data and climate. The second was obtained with modern biological data (1980–present) and modern environmental variables, and represents the modern potential distribution of a species.

The comparison of each pair of potential distribution was carried out on both habitat suitability and binary maps. To compare each pair, three different values were calculated: the Schoener’s D index [103], the percentage of stable cells, and the range size variation.

The Schoener’s D index was calculated from habitat suitability maps according to Warren et al. [104], and is a measure of similarity. The percentage of stable cells and the range size variation were calculated from binary maps. The percentage of stable cells was calculated by considering the number of ‘presence’ cells found in both potential distributions with respect to the total. It represents a measure of the stability of a given method. The range size variation was calculated by comparing the ‘modern’ potential distribution (Figure 3: Distribution 2) with the ‘projected’ potential distribution (Figure 3: Distribution 1), and shows the percentage of sites interested by changes (either gain or loss). It represents the tendency of a given procedure to overestimate or underestimate the results, when used to forecast future potential distributions.

All modelling applications involving the transfer of model predictions should be accompanied by precautionary and exploratory analyses to identify regions of strict extrapolation and better-characterize the degree of novelty of areas to which model rules are to be transferred [105]. To characterize the extrapolation risk associated with model transfer to different scenarios (i.e. the projection of a model built with historical data to modern conditions) the Mobility-Oriented Parity analysis (MOP) [106] was performed on each set of environmental layers and for each species. The analysis was performed by using the Kuenm package [107] with the default parametrization (percentage of values sampled from the calibration region = 10%, distance matrix for each fixed number of rows = 2000).

### 2.5. Model Evaluation Metrics

Because no consensus currently exists regarding the most appropriate method to validate and interpret a model, we used five different evaluation metrics: the area under the curve of the receiver operating characteristic plot based on the training (AUC_TRAIN_) and testing data (AUC_TEST_) [20]; the difference between training and testing AUC (AUC_DIFF_) [108]; the 10% omission rate [109]; the partial ROC (pROC) (threshold 5%) [110].

The training and testing AUC are a measure of statistical significance of a model and determine whether observed predictions of evaluation data differ from null expectations [20]. The difference between training and testing AUC quantifies overfitting [108]. Omission rates at 10% is a threshold-dependent test to measure performance, which indicates how well models created with training data predict test occurrences [20,107]. Partial ROC is another measure of statistical significance of a model [110], proposed to solve the flaws highlighted by Lobo et al. [111] and Jiménez-Valverde [112].

To evaluate the quality of predictions related to the present (‘projected’ and ‘modern’ distributions, Figure 3), the Continuous Boyce Index (CBI) value [113] was calculated by using independent modern occurrence cells for each species and for each procedure.

### 2.6. Final Evaluations of Procedures Response

The application of 12 modelling procedures to 25 species led to 300 case studies (i.e., comparisons of model pairs). The results related to Schoener’s D index, percentage of stable cells and range size variation were used altogether to generate a scatter plot. For the output of each procedure (represented as a single point in the scatter plot) a final evaluation value was obtained by calculating the Euclidean distance (d) from a point that represents the best theoretical performance, i.e. a performance characterized by D index equal to 1, a range size variation equal to 0, and a percentage of stable cells equal to 100.

### 2.7. Data Analysis

To check for statistically significant differences among procedures concerning D index, percentage of stable cells, range size variation and distance (d), the Kruskal-Wallis test [114] followed by the post hoc Mann-Whitney test [115,116] and Bonferroni correction were used. The same approach was also used to compare differences between CBI calculated on the ‘modern’ and ‘projected’ potential distributions. Only differences showing *p* < 0.01 were considered statistically significant.

## 3. Results

Multiple results were collected by applying a single workflow on two different datasets, so that we will show them separately based on the different environmental variables used each time. Each result was obtained by modelling 25 plant species with 12 different procedures, for a total of 600 cases (Supplementary Material S2 and S3).

### 3.1. Performance of the Predictions

#### 3.1.1. Set I: Three Environmental Variables

Based on the three main values used to evaluate the overall performance of each procedure (D index, range size variation, and stability) and on the final evaluation value (distance), only a few procedures showed significant differences in predictive ability. The Kruskal-Wallis analysis showed statistically significant differences concerning D index (*p* = 1.983 × 10^−19^), stability (*p* = 1.026 × 10^−13^), and distance (*p* = 9.937 × 10^−14^), but not concerning range size variation (*p* = 0.089). The pairwise Mann-Whitney tests identified 17 cases of significant differences concerning stability and distance, and 21 cases concerning D index (Table 3 and Supplementary Material, Table S4A,B).

**Figure 4:**
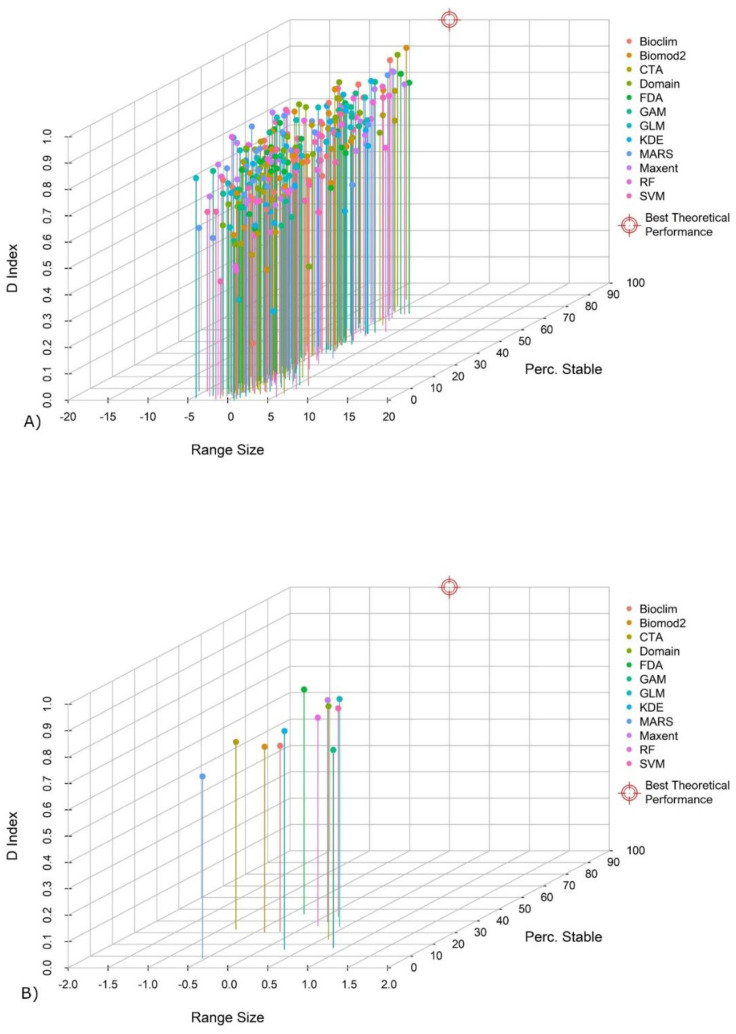
Distribution of the single results for each of the 25 species and procedure (3 variables) (**A**). Mean values for each procedure (**B**).

The values of D index, range size variation, stability, and distance (d) calculated for each procedure are summarized in Supplementary Material, Table S4C–F. The scatter plots show the single results obtained for each species and procedure (Figure 4A) and the mean results for each procedure (Figure 4B). Most of the procedures are characterized by a range size variation near to 0 (<|2|%). In 51 cases, a variation between |2|% and |5|% was observed. In only seven cases, the variation was higher than |5|%. The stability is variable, with FDA, SVM, and Maxent that scored high values (>40%). RF, GLM, CTA, Bioclim, and Biomod2 scored intermediate values (between 30% and 40%). Low values (<30%) were obtained by Domain, GAM, KDE, and MARS. The D index values are similar: five procedures obtained values higher than 0.8, and six procedures between 0.7 and 0.8. Only MARS obtained values lower than 0.7. Overall, the values of distance (d) range from 99.97 to 13.96. The mean values calculated for each procedure describe three main cases: low-distance procedures (d < 60) (FDA, SVM), intermediate-distance procedures (60 ≤ d ≤ 70) (Maxent, RF, GLM, CTA, Bioclim, and Biomod2) and high distance procedure (d > 70) (Domain, GAM, KDE, and MARS).

Summary CBI values (for both ‘projected’ and ‘modern’ procedures) are reported in Supplementary Material, Table S4G−H. The Kruskal-Wallis analysis on the projected CBI values shows a statistically significant difference among procedures (*p* = 8.3 × 10^−4^) and the pairwise Mann-Whitney tests identified only three cases of significant differences related to Maxent, RF, and SVM versus Bioclim.

The comparison of the CBI values for each pair of habitat suitability maps (‘modern’ and ‘projected’) highlighted a significant difference for three procedures (KDE, MARS, and RF), characterized by higher CBI score for the ‘modern’ procedure. The procedures that obtained mean CBI scores greater than 0.7 for both ‘projected’ and ‘modern’ maps were 9 (Biomod2, CTA, Domain, FDA, GAM, GLM, Maxent, RF, and SVM).

#### 3.1.2. Set II: 35 Environmental Variables and Dimensionality Reduction via PCA

The Kruskal-Wallis analysis on the three main values used to evaluate the overall performance of each procedure (D index, range size variation, and stability), and on the final evaluation value (distance) showed statistically significant differences for D index (2.47 × 10^−17^), stability (*p* = 1.74 × 10^−10^), and distance (*p* = 1.73 × 10^−10^). The pairwise Mann-Whitney tests identified 10 cases of significant differences concerning stability and distance, and 19 cases concerning D index (Table 4 and Supplementary Material, Table S5A,B).

**Figure 5:**
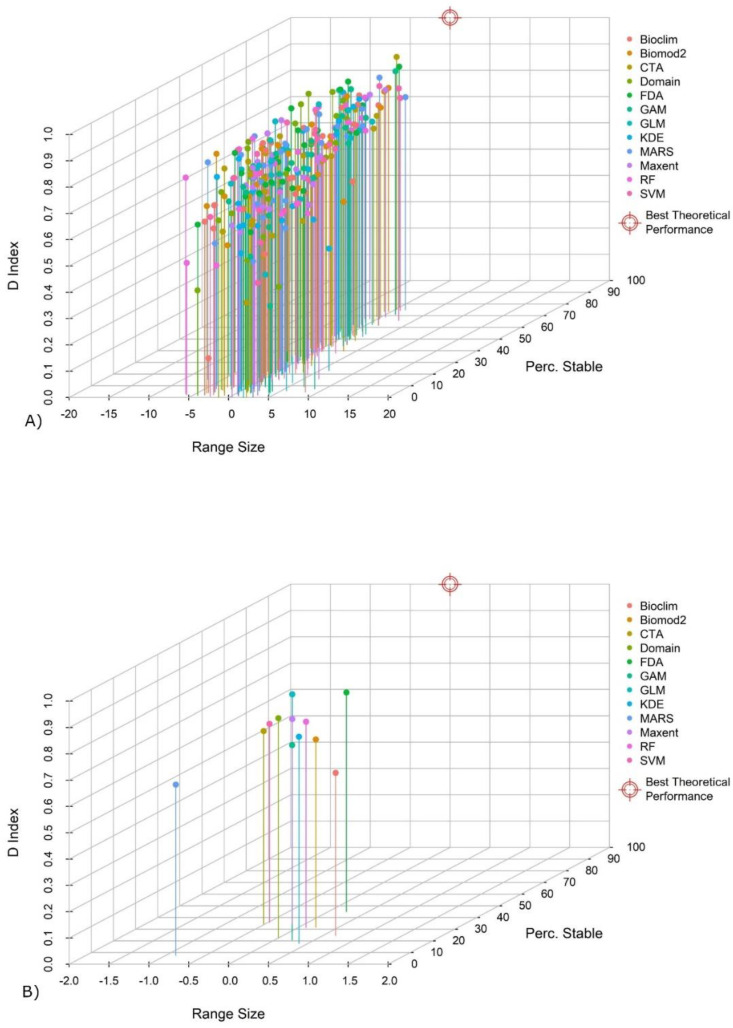
Distribution of the single results for each of the 25 species and each procedure (first three axes of PCA [35 variables]) (**A**). Mean values for each procedure (**B**).

The values of range size variation, stability, D index, and distance calculated for all procedures are summarized in Supplementary Material, Table S5C–F. The scatter plots show the single results (Figure 5A) and the mean results (Figure 5B) for all procedures. Most of the procedures are characterized by range size variation near to 0 (<|2|%). In 57 cases, variations between |2|% and |5|% were observed. In only 14 cases the variation was >|5|%. Several procedures scored similar results concerning stability. Only FDA scored a high value (>40%). GLM, SVM, Maxent, CTA, Biomod2, and RF scored intermediate values (between 30% and 40%). Low values (<30%) were obtained by Bioclim, Domain, GAM, KDE, and MARS. The D index values are similar: three procedures obtained values higher than 0.8, and seven procedures values between 0.7 and 0.8. Only MARS and Bioclim obtained values lower than 0.7. Overall, the values of distance (d) range from 100.02 to 22.72. The mean values calculated for each procedure describe three main cases: low-distance procedures (d < 60) (FDA), intermediate-distance procedures (60 ≤ d ≤ 70) (GLM, SVM, Maxent, CTA, Biomod2, and RF), and high-distance procedures (d > 70) (Bioclim, Domain, GAM, KDE, and MARS).

Summary CBI values (for both ‘projected’ and ‘modern’ procedures) are reported in Supplementary Material, Table S5G. No statistically significant difference among procedures concerning CBI was found. The comparison of the CBI values for each pair of habitat suitability maps (‘modern’ and ‘projected’) highlighted a significant difference for six procedures (Biomod2, CTA, KDE, MARS, RF, and SVM), characterized by higher CBI score for the ‘modern’ procedure. The procedures that obtained mean CBI scores greater than 0.7 for both ‘projected’ and ‘modern’ maps were 7 (Biomod2, CTA, GAM, KDE, Maxent, RF, and SVM).

### 3.2. Evaluation Metrics of the Predictions

The complete list of evaluation metrics acquired in all modelling procedures is reported in Supplementary Material S6 (only three environmental variables) and in Supplementary Material S7 (first three axes of PCA [35 variables]). The evaluation metrics calculated for each pair of procedures (‘present’ and ‘projected’) were comparable, with no evident difference. The AUC values did not highlight significant differences among historical and modern models, in all procedures.

### 3.3. Evaluation of the Transferability

MOP analyses confirmed the transferability of models through the different scenarios selected for this study, albeit differences between the procedures based on three environmental layers and those based on PCA were observed. In general, the extrapolation risk (i.e., the lack of similar environmental combinations) across historical and modern times was low for all selected study areas. When only three environmental layers were used, the areas of strict extrapolation identified by MOP analysis represented less than 1% of the entire surface in 14 cases, 1–2% in 6 cases, and 2–4% in 5 cases. When the first three axes of a PCA based on 35 environmental layers were used, the areas of strict extrapolation identified by MOP analysis represented less than 1% of the entire surface in 18 cases, 1–2% in 2 cases, and 4–9% in 5 cases. MOP analysis confirms that all potential distributions obtained with the different modelling procedures were calculated inside the environmental ranges on which models were originally calibrated. The complete list of results obtained with MOP analysis is reported in Supplementary Material S8.

## 4. Discussion

The application of a standardized modelling protocol to 25 Italian endemic plant species, 12 different procedures and 2 sets of environmental variables allowed to experimentally show that ENM transfers to future conditions are effective. This is of particular relevance, because model transfers are widely used in investigations focused on the study of the effects of climate change on species distribution [11,12,13,14,15,16,17,18,19].

However, no algorithm included in this study showed optimal projection abilities when applied to the task of predict future distribution. Several algorithms had comparably good results (CTA, FDA, GLM, Maxent, RF, SVM, and Biomod2), and a few relatively bad results (Bioclim, Domain, GAM, KDE, and MARS). Similar results were achieved for both tested sets of environmental variables (only three variables vs. first three axes of PCA). Statistical significance tests applied to the summary values of distance (d) highlighted a few differences among the different procedures. Only MARS was always different from other procedures, obtaining extraordinarily low scores in all tests aimed to assess the quality of future projections. In no case significant differences concerning range size variation were highlighted. This means that all procedures are equally capable to estimate changes of range size. On the contrary, statistically significant differences were highlighted concerning stability and D index scores. This means that not all procedures identify the same areas and the same niche. Statistical differences between projected CBI scores were found only when models were based on 3 environmental variables, but not when they were based on the first 3 axes of a PCA from 35 environmental variables. These differences concerned only Maxent, RF, SVM vs. Bioclim. Projected and modern CBI scores for each procedure showed significant differences for RF, KDE, and MARS (for both sets of variables), and for Biomod2, CTA, and SVM (PCA), in all cases characterized by markedly higher CBI scores in modern models. RF, Biomod2, and SVM achieved particularly high CBI scores in modern models, albeit only RF showed significantly higher CBI scores, using both sets of environmental variables. This means that this algorithm is particularly well suited in research aimed to study species potential distribution in current times (e.g., to discover new populations) [117,118]. The differences between the mean values calculated for each procedure (Supplementary Material S9) allow only a rough comparison of results. In general, two algorithms returned overall better results (SVM and FDA), five returned intermediate results (Maxent, RF, CTA, Biomod2, and GLM) and five the worst results (Bioclim, Domain, GAM, KDE, and especially MARS). FDA is the procedure showing the lowest distance, d = 53.91/55.01 when models were built with three environmental variables or PCA, respectively. This is mostly due to the high percentage of stability and to a range size variation near 0. When models were built using three environmental variables, the mean value of distance calculated with SVM was d = 56.25, the second lowest distance among all procedures. When models were built using the first three axes of PCA, the performance of SVM slightly decreased, as a consequence of a lower level of stability. Maxent and GLM were characterized by only relatively higher values of distance, in a range between d = 60–65. All other procedures scored high values of distance (d > 65), particularly high (d > 80) with GAM, KDE, and MARS.

Previous research [119] highlighted the impossibility to find a single ‘best’ algorithm, and our results fully confirm this view. Previous studies on transferability [42,43,44,45,46] highlighted some better-performing algorithms, but comparing a few algorithms and/or a few species. A recent investigation [47] performed with 11 algorithms and 16 virtual species highlighted that none of the investigated algorithms accurately estimated the fundamental niche of species. Our results, albeit based on potential distribution and not on fundamental niche, are fully congruent with this study.

Three main factors are responsible for the similarity among procedures we documented in this study: (1) the decision to average results across 10 replicate runs; (2) the application of subsampling as resampling method in all procedures, and (3) the absence of extrapolation risks in the investigated study areas.

Contrarily to the results provided by Generalized Linear Models (GLM), Ecological Niche Factor Analyses (ENFA), or BIOCLIM, which are always identical for the same dataset [23,33], machine learning methods always return slightly different results [24,94], so that replication is crucial.

The use of subsampling selection together with replications enables avoiding deterministic results and to obtain stochastic results, in order to quantify the variability and uncertainty in model outputs [20,120,121]. According to the latter authors, this topic is neglected in many studies. Indeed, our results suggest that differences among different algorithms can be reduced by adopting strategies aimed to achieve stochastic outcomes. Thus, the recommendation by Sillero and Barbosa [31] about the need to replicate models should be extended to algorithms and methods, when strategies to achieve stochastic results are adopted.

The impact of interpolation and extrapolation on transferability was highlighted in several studies, as the fact that interpolation causes less problems than extrapolation [122]. Owens et al. [106] suggested to perform a series of preliminary and exploratory analyses (MESS, MOP) [105,106] in all niche modelling applications involving transfer of model predictions. In this study, MOP analysis was performed and confirmed the absence of relevant extrapolation risks due to the emergence of non-analog climates in future scenarios for both sets of environmental variables. Some algorithms deal with issues related to extrapolation better than others [123], and possible differences in model responses could be related to extrapolation, absent in our study.

Both sets of environmental variables generated comparable outcomes. Consequently, the two alternative strategies for environmental variables selection can be considered equally good. Despite this, many authors highlighted the need to incorporate biological realism into the ENM process [52,58,124], so that the use of a few variables selected for their biological relevance should be preferred. Warren et al. [52] also highlighted the discrepancy between abstract evaluation metrics and biological plausibility of models, especially when built starting from a high number of environmental variables. A systematic review of 201 studies [125] further pointed out that model prediction and biology of real populations lack of correlation, especially when predictions are not tested with independent data to evaluate the reliability of the distribution hypotheses.

Our study provides a new method to evaluate the transferability of models, useful to compare predictions in geographical space, and further stresses that there is not a single ‘best’ performing algorithm to forecast the future distribution of living organisms under climate change scenarios. The possible use of a diverse suite of algorithms to achieve similar results was confirmed. However, several factors (replication, resampling method, absence of extrapolation risk in the study area) could be equally important as the selection of the algorithm itself. In our experiment, since the aim was to assess the effectiveness of temporal projection in commonly used approaches, we did not optimize or tune each algorithm, albeit this practice is surely possible. Our results are useful as empirical guidelines to select the best modelling settings, especially for studies related to stenochorous plant species [126].

Although we did our utmost to plan a modelling experiment aimed to test the predictive ability of ENM algorithms, multiple factors could have influenced the results. All species used in this study are Italian endemic plants. This type of species is characterized by pros and cons. An important favorable aspect is represented by the limited and well-defined range of each taxon, investigated through long-term floristic studies. On the other hand, most of these species are also characterized by small population size and, as a consequence, the availability of occurrence data is scarce when compared to widespread species [126]. All collected distribution data was obtained from herbarium specimens, literature, and observations, in most of the cases without original georeferencing. Additionally, this “second-step” georeferentiation could be a source of bias. For practical reasons, the number of tested combinations of environmental variables was limited, because an increment in combinations leads quickly to a prohibitive number of cases.

To overcome these limitations, further analyses aimed to test the predictive ability of ENMs under different scenarios could take advantage from the use of virtual species with known niche properties. This type of data could provide abundant and controlled occurrence [47,102,127], and reduce possible problems by eliminating errors that affect data of real species, as, e.g., sampling bias, limited sample size, and complex species interactions. Another source of error could be generated by the need to convert habitat suitability maps into binary potential presence/absence maps. Although binary conversion simplifies the interpretation of distribution maps, this step could deeply alter the outcomes of a model [128]. Further improvements could be achieved by testing different combinations of algorithms and resampling methods. Wenger and Olden [43] already investigated the influence of different resampling methods on the transferability of models. Thus, an exhaustive evaluation of several resampling methods [129,130,131] could provide new best practices for a correct evaluation of future potential distributions. Finally, major improvements for enhancing temporal projection could be achieved by developing proper methods to quantify and evaluate the uncertainty associated with prediction [12,132,133], a topic not addressed in this study.

## 5. Conclusions

The aim of our study was to experimentally check the effectiveness of model transferability of historical occurrence and climatic data to modern conditions, simulating a real “future”. We confirm that all the 12 investigated algorithms are effective in model transferability. Most of the algorithms (Biomod2, CTA, FDA, GLM, Maxent, RF, and SVM) performed in a similar way. Only five algorithms (Bioclim, Domain, GAM, KDE, and MARS) showed a worse predictive ability. Incidentally, RF resulted particularly good in modelling niches *in absence* of temporal projection. These findings highlight that a diverse suite of algorithms is capable to generate similar predictions and, as a consequence, that a stringent prior choice of algorithm is not needed. However, the overall similarity highlighted here is certainly due also to the standardized workflow adopted to build the models: averaging several replicated models, using subsampling as resampling method, checking for absence of extrapolation risk in the selected study areas. These “additional settings” in modelling experiments could be equally important as algorithm selection for the quality of predictions.

## Figures and Tables

**Table 1:** Previous studies focused on analysis of ENM transferability.

Reference	Algorithms	Species/Data	Transferability Test
Randin et al. [42]	GLM, GAM	54 species with more than 30 occurrences from vegetation plots	Evaluation metrics; Kulczynski’s coefficient
Wenger and Olden [43]	GLMM, ANN, R	*Salvelinus fontinalis* (Mitchill, 1814); *Salmo trutta* Linnaeus, 1758	Evaluation metrics combined with resampling methods
Roberts and Hamann [44]	RF	Modern ecosystem types	Validation based on palaeoecological records
Veloz et al. [45]	BRT, MARS, MARS-COM, GAM, GLM	Fossil-pollen data	Tests of niche equivalency (D) and niche similarity (I)
Duque-Lazo et al. [46]	ANN, BRT, CART, FDA, GAM, GLM, MaxEnt, MARS, RF, SRE	Presence-absence data for *Phytophthora cinnamomi* Rands (presence *n* = 599; absence *n* = 1193)	Evaluation metrics; transferability index
Qiao et al. [47]	BIOCLIM, ENFA, CONVEXHULL, MVE, GLM, GAM, BRT, GARP, Maxent, KDE, MA	16 virtual species distributed across mainland Eurasia	Sensitivity, specificity and TSS plus volume ratio of estimated niches

**Table 2:** Summary of the different procedures used in the present work. Commonly adopted parameters were selected in all procedures.

Procedure	Type and Algorithm	Data Input	Package (Version)	Background/Pseudoabsence Cells
BIOCLIM	Single (BIOCLIM)	Presence only	Dismo (1.1-4)	1000 background cells
Domain	Single (Domain)	Presence only	Dismo (1.1-4)	1000 background cells
GLM	Single (GLM)	Presence-pseudoabsence	SSDM (0.2.8)	1000 pseudoabsence cells
GAM	Single (GAM)	Presence-pseudoabsence	SSDM (0.2.8)	1000 pseudoabsence cells
MARS	Single (MARS)	Presence-pseudoabsence	SSDM (0.2.8)	1000 pseudoabsence cells
FDA	Single (FDA)	Presence-pseudoabsence	sdm (1.0-89)	Pseudoabsences = presence cells
CTA	Single (CTA)	Presence-pseudoabsence	sdm (1.0-89)	Pseudoabsences = presence cells
RF	Single (RF)	Presence-pseudoabsence	SSDM (0.2.8)	Pseudoabsences = presence cells
SVM	Single (SVM)	Presence-pseudoabsence	SSDM (0.2.8)	Pseudoabsences = presence cells
Maxent	Single (Maxent)	Presence background	Dismo (1.1-4)	10,000 background cells
Biomod2	Ensemble (GLM, GBM, GAM, MARS, Maxent, RF, CTA, ANN, and FDA)	Presence-pseudoabsence	biomod2 (3.4.11)	Pseudoabsences = presence cells × 10
KDE	Single (KDE)	Presence only	Hypervolume (2.0.12)	1000 background cells

**Table 3:** Results of pairwise Mann-Whitney tests for the values of distance (three variables). A total of 17 cases of statistically significant differences were highlighted. × = statistically significant difference.

	Bioclim	Biomod2	CTA	Domain	FDA	GAM	GLM	KDE	MARS	Maxent	RF	SVM
**Bioclim**												
**Biomod2**	-											
**CTA**	-	-										
**Domain**	-	-	-									
**FDA**	-	-	-	-								
**GAM**	-	-	-	-	×							
**GLM**	-	-	-	-	-	×						
**KDE**	-	-	-	-	×	-	×					
**MARS**	×	×	×	×	×	×	×	-				
**Maxent**	-	-	-	-	-	×	-	×	×			
**RF**	-	-	-	-	-	-	-	-	×	-		
**SVM**	-	-	-	-	-	-	-	×	×	-	-	

**Table 4:** Results of pairwise Mann-Whitney tests for the summary values of distance (first three axes of PCA [35 variables]). A total of 10 cases of statistically significant differences were highlighted. × = statistically significant difference.

	Bioclim	Biomod2	CTA	Domain	FDA	GAM	GLM	KDE	MARS	Maxent	RF	SVM
**Bioclim**												
**Biomod2**	-											
**CTA**	-	-										
**Domain**	-	-	-									
**FDA**	-	-	-	-								
**GAM**	-	-	-	-	-							
**GLM**	-	-	-	-	-	-						
**KDE**	-	-	-	-	×	-	-					
**MARS**	×	×	×	×	×	×	×	-				
**Maxent**	-	-	-	-	-	-	-	-	×			
**RF**	-	-	-	-	-	-	-	-	×	-		
**SVM**	-	-	-	-	-	-	-	-	-	-	-	

## Data Availability

Occurrences, bioclimatic data and other geospatial data used in this study can be downloaded at: https://drive.google.com/file/d/1x6zcPJ8_SLlFpAi1FBtVZQ9O03P2EMZi/view?usp=sharing.

## Supplementary Materials

The following supporting information can be downloaded at: https://www.mdpi.com/article/10.3390/biology11081219/s1: S.1: Supplementary information about species, study areas and environmental variables. S.2: Results of all modelling procedures obtained by using three bioclimatic variables. S.3: Results of all modelling procedures obtained by using the first three axes of a principal component analysis (PCA) on the 35 bioclimatic variables. S.4: Results of all comparisons based on procedures obtained by using three bioclimatic variables. S.5: Results of all comparisons based on procedures obtained by using the first three axes of a principal component analysis (PCA) of the 35 bioclimatic variables. S.6: Complete list of evaluation metrics calculated for all modelling procedures based on the use of three environmental variables. S.7: Complete list of evaluation metrics calculated for all modelling procedures based on the use of 35 environmental variables converted into 3 layers by applying dimensionality reduction technique (PCA). S.8: Results of MOP analysis conducted on each set of environmental variables. S.9: Summary values (range size variation, stability, D index, distance, CBI Projected, CBI Present) grouped according to procedures. Table S1A: Complete list of environmental variables used in this study; Table S1B: List of the endemic species selected in this study; Table S1C,D: Occurrence records available for each species; Table S4A: Results of pairwise Mann-Whitney tests for the values of Schoener’s D-index (3 variables); Table S4B: Results of pairwise Mann-Whitney tests for the values of stability (3 variables); Table S4C: Summary values of ‘Range Size variation’ (3 variables); Table S4D: Summary values of ‘stability’ (3 variables); Table S4E: Summary values of ‘D-index’ (3 variables); Table S4F: Summary values of ‘distance’ (3 variables); Table S4G: Summary results of CBI for present and projected potential distributions (3 variables); Table S4H: Results of pairwise Mann-Whitney tests for the values of CBI when projected (3 variables); Table S5A: Results of pairwise Mann-Whitney tests for the values of Schoener’s D-index (first 3 axes of PCA); Table S5B: Results of pairwise Mann-Whitney tests for the values of stability (first 3 axes of PCA); Table S5C: Summary values of ‘Range Size variation’ (first 3 axes of PCA); Table S5D: Summary values of ‘stability’ (first 3 axes of PCA); Table S5E: Summary values of ‘D-index’ (first 3 axes of PCA); Table S5F: Summary values of ‘distance’ (first 3 axes of PCA); Table S5G: Summary results of CBI for present and projected potential distributions (first 3 axes of PCA)
