# Supplementary material for: Assessing the Effectiveness of Correlative Ecological Niche Model Temporal Projection through Floristic Data
Source: Biology (Basel). 2022 Aug 14;11(8):1219. doi: 10.3390/biology11081219 (PMC9405103; doi:10.3390/biology11081219)
Supplement: Supplementary file 1 [file biology-11-01219-s001.zip › Dolci & Peruzzi - DATA.zip!/Bio_data/Biodata_E_space_b01.b12.v01/New_data_bio01_bio12_var01_Adenostyles australis/rgl_file/Overlap_post-filter_Adnstylsastr.html]

 
RGL model


You must enable Javascript to view this page properly.

  
Drag mouse to rotate model. Use mouse wheel or middle button
to zoom it.

---

  
Object written from rgl 0.100.54 by writeWebGL.
