# Supplementary material for: Assessing the Effectiveness of Correlative Ecological Niche Model Temporal Projection through Floristic Data
Source: Biology (Basel). 2022 Aug 14;11(8):1219. doi: 10.3390/biology11081219 (PMC9405103; doi:10.3390/biology11081219)
Supplement: Supplementary file 1 [file biology-11-01219-s001.zip › Dolci & Peruzzi Supplementary Material S.1-9.zip!/Supplementary material S.1.pdf]

Supplementary material S.1

Supplementary information about species, study areas and environmental variables.

Table S.1.A. Complete list of environmental variables used in this study. All variables were calculated by using DISMO [56] and ENVIREM [53] packages, and environmental data from CHELSAcruts for the two periods 1901–1930 and 1981–2010.

Bioclimatic variable	Package
Annual Mean Temperature	DISMO
Mean Diurnal Range	DISMO
Isothermality	DISMO
Temperature Seasonality	DISMO
Max Temperature of Warmest Month	DISMO
Min Temperature of Coldest Month	DISMO
Temperature Annual Range	DISMO
Mean Temperature of Wettest Quarter	DISMO
Mean Temperature of Driest Quarter	DISMO
Mean Temperature of Warmest Quarter	DISMO
Mean Temperature of Coldest Quarter	DISMO
Annual Precipitation	DISMO
Precipitation of Wettest Month	DISMO
Precipitation of Driest Month	DISMO
Precipitation Seasonality (Coefficient of Variation)	DISMO
Precipitation of Wettest Quarter	DISMO
Precipitation of Driest Quarter	DISMO
Precipitation of Warmest Quarter	DISMO
Precipitation of Coldest Quarter	DISMO
Annual potential evapotranspiration	ENVIREM
Thornthwaite aridity index	ENVIREM
Relative wetness and aridity	ENVIREM
Continentality	ENVIREM
Emberger's pluviothermic quotient	ENVIREM
Sum of mean monthly T. for month with mean T. greater than 0	ENVIREM
Sum of mean monthly temp. for months with mean T. greater than 5	ENVIREM
Max. temp. of the coldest month	ENVIREM
Min. temp. of the warmest month	ENVIREM
Number of months with mean temp greater than 10	ENVIREM
Potential evapotranspiration of coldest quarter	ENVIREM
Potential evapotranspiration of driest quarter	ENVIREM
Monthly variability in potential evapotranspiration	ENVIREM
Mean monthly potential evapotranspiration of warmest quarter	ENVIREM
Mean monthly potential evapotranspiration of wettest quarter	ENVIREM
Compensated thermicity index	ENVIREM

Table S.1.B. – List of the Italian endemic species selected for the modeling experiments, their life form and distribution.

	Species	Raunkiaer's Life Form	Distribution	References
-	*Adenostyles australis* (Ten.) Iamonico & Pignatti	Scapose Hemicryptophyte	Peninsular Italy, on stony ground and beech-fir forests of Apennine mountains (1100–1900 m)	[65]
-	*Aubrieta columnae* Guss. subsp. *columnae*	Suffruticose Chamephyte	Mountains of central and southern Italy, on calcareous rocks (800–2300 m)	[64]
-	*Bellevalia webbiana* Parl.	Bulbous Geophyte	North-central Italy, in grasslands, pastures and wood-margins (100–700 m)	[63]
-	*Cardamine apennina* Lihová & Marhold	Scapose Hemicryptophyte	Central Italy, in wet grassland, peat grasslands, and hygrophilous forest (200–1400 m)	[64, 66]
-	*Carduus affinis* Guss. subsp. *affinis*	Scapose Hemicryptophyte	Central and southern Italy, in mountain woods and grassland (1500–2200 m)	[65, 68]
-	*Crocus biflorus* Mill.	Bulbous Geophyte	Northern and peninsular Italy, Sicily. In grassland, pastures, and dry grassland (0–1200 m)	[63]
-	*Crocus etruscus* Parl.	Bulbous Geophyte	Southern Tuscany, Umbria, and Emilia-Romagna in deciduous woods and pastures (0–700 m)	[63, 67]
-	*Crocus imperati* Ten.	Bulbous Geophyte	Central and southern Italy, in mountain grassland and pastures near the sea (0–1400 m)	[63]
-	*Erysimum pseudorhaeticum* Polatschek	Scapose Hemicryptophyte	North-central and South-central Italy, in grasslands, pastures and fields, along roadsides (100–1850 m)	[64]
-	*Galium magellense* Ten.	Scapose Hemicryptophyte	Apennine mountains in Central Italy, and in Pollino massif on calcareous screes (1800–2600 m)	[65, 67]
-	*Gypsophila arrostoi* Guss. subsp. *arrostoi*	Suffruticose Chamephyte	Southern Italy, on dry, sandy and stony plans (0–800 m).	[64]
-	*Helleborus viridis* L. subsp. *bocconei* (Ten.) Peruzzi	Rhizomatous Geophyte	Mountains and hills of peninsular Italy and Sicily, in woods and shady places (0–1200 m)	[63, 67]
-	*Klasea flavescens* (L.) Holub subsp. *cichoracea* (L.) Greuter & Wagenitz	Scapose Hemicryptophyte	Central and southern Italy, in brushland, pastures and coppice woodland (0–800 m)	[65]
-	*Koeleria splendens* C.Presl	Caespitose hemicryptophytes	Peninsular Italy and Sicily, in dry grasslands, scrublands, grassy slopes and pastures (0–1800 m)	[63]
-	*Leontodon anomalus* Ball	Rosulate Hemicryptophytes	Western part of North Central Italy, on calcareous rocks, cliff, and gravels (0–1800 m)	[65]

-	*Linaria purpurea* (L.) Mill.	Scapose Hemicryptophyte	Peninsular Italy and Sicily, on rocks, screes, and waste places (0–1900 m)	[65]
-	*Ornithogalum etruscum* Parl. subsp. *etruscum*	Bulbous Geophyte	Peninsular Italy, on dry hillsides (0–1200 m)	[63]
-	*Phyteuma ovatum* Honck. subsp. *pseudospicatum* Pignatti	Scapose Hemicryptophyte	Apennine mountains in North Central Italy, and Mount Amiata, in mountain meadows (1000–2300 m)	[65]
-	*Polygala flavescens* DC. subsp. *flavescens*	Scapose Hemicryptophyte	Peninsular Italy, in dry grasslands, pastures and roadsides (0–1200 m)	[64, 69]
-	*Ranunculus pollinensis* (N.Terracc.) Chiov.	Scapose Hemicryptophyte	Peninsular Italy, in grasslands, mountain woods and nitrophilous soils (1100–2400 m)	[63]
-	*Saxifraga porophylla* Bertol. subsp. *porophylla*	Pulvinate Chamaephyte	Central and southern Italy, on limestone rocks and screes above 1000 m	[63]
-	*Sedum magellense* Ten. subsp. *magellense*	Succulent Chamaephyte	Central and southern Italy, in rocky and shady places, especially near beech moss-covered boulder (1300–2900 m)	[63]
-	*Siler montanum* Crantz subsp. *siculum* (Spreng.) Iamonico, Bartolucci &	Scapose Hemicryptophyte	Peninsular Italy, in rocky places and dry grasslands (200–2400 m)	[65]
-	*Viola eugeniae* Parl. subsp. *eugeniae*	Scapose Hemicryptophyte	Peninsular Italy, on calcareous soils and rocky grasslands, usually above 1000 m	[64]
-	*Viola ferrarinii* Moraldo & Ricceri	Scapose Hemicryptophyte	North-Central Italy, in meadows, pastures and screes, mostly above 1500 m	[64]

*This species undergone several taxonomic changes in recent times [70] and was split in multiple groups. In this study the traditional taxonomic delimitation was applied.

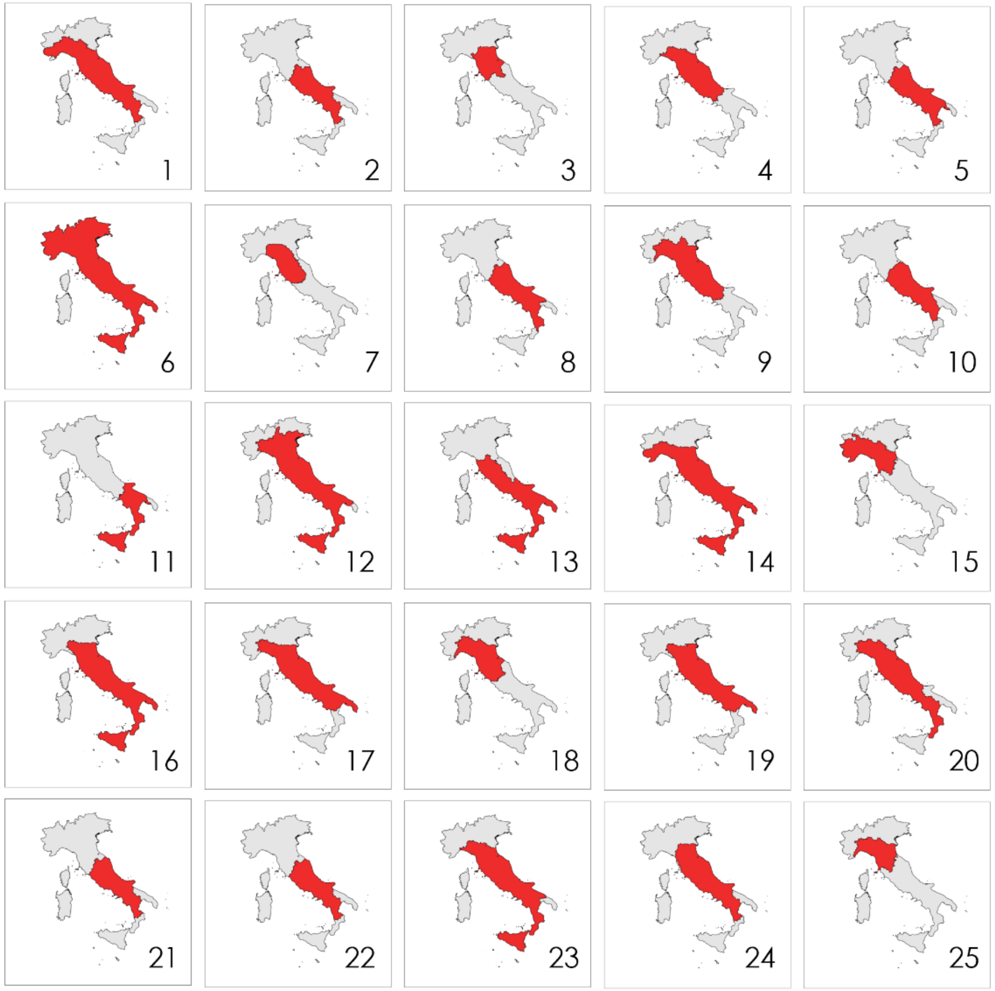

Figure S.1.A. Study areas used to model the selected species. All study areas were selected to include all known occurrence points, regardless time limit. Additional portions were also integrated to include areas where species could spread, based on their ecological requirements. 1) *Adenostyles australis*; 2) *Aubrieta columnae* subsp. *columnae*; 3) *Bellevalia webbiana*; 4) *Cardamine apennina*; 5) *Carduus affinis* subsp. *affinis*; 6) *Crocus biflorus*; 7) *C. etruscus*; 8) *C. imperati*; 9) *Erysimum pseudorhaeticum*; 10) *Galium magellense*; 11) *Gypsophila arrostoi* subsp. *arrostoi*; 12) *Helleborus viridis* subsp. *bocconei*; 13) *Klasea flavescens* subsp. *cichoracea*; 14) *Koeleria splendens*; 15) *Leontodon anomalus*; 16) *Linaria purpurea*; 17) *Ornithogalum etruscum* subsp. *etruscum*; 18) *Phyteuma ovatum* subsp. *pseudospicatum*; 19) *Polygala flavescens* subsp. *flavescens*; 20) *Ranunculus pollinensis*; 21) *Saxifraga porophylla* subsp. *porophylla*; 22) *Sedum magellense* subsp. *magellense*; 23) *Siler montanum* subsp. *siculum*; 24) *Viola eugeniae* subsp. *eugeniae*; 25) *V. ferrarinii*.

Table S.1.C. Number of occurrence records and their abundance (referred to the source of information). Lit = literature; Herb = herbarium specimen; Obs = observation; His = historical; Mod = modern.

	Species	Lit	Herb (percentage)	Obs	His	Mod (number)	Total
-	*Adenostyles australis* (Ten.) Iamonico & Pignatti	60%	13%	27%	61	191	252
-	*Aubrieta columnae* Guss. subsp. *columnae*	64%	28%	8%	19	57	76
-	*Bellevalia webbiana* Parl.	85%	5%	10%	28	25	53
-	*Cardamine apennina* Lihová & Marhold	78%	21%	1%	28	37	65
-	*Carduus affinis* Guss. subsp. *affinis*	40%	53%	7%	60	73	133
-	*Crocus biflorus* Mill.	42%	16%	42%	73	257	330
-	*Crocus etruscus* Parl.	23%	6%	71%	7	107	114
-	*Crocus imperati* Ten.	12%	77%	9%	26	25	51
-	*Erysimum pseudorhaeticum* Polatschek	31%	16%	52%	45	285	330
-	*Galium magellense* Ten.	62%	29%	9%	39	89	128
-	*Gypsophila arrostoi* Guss. subsp. *arrostoi*	20%	23%	57%	23	105	128
-	*Helleborus viridis* L. subsp. *bocconei* (Ten.) Peruzzi	51%	15%	34%	36	220	256
-	*Klasea flavescens* (L.) Holub subsp. *cichoracea* (L.) Greuter & Wagenitz	44%	18%	38%	21	105	126
-	*Koeleria splendens* C.Presl	50%	27%	23%	83	285	368
-	*Leontodon anomalus* Ball	40%	1%	59%	18	88	106
-	*Linaria purpurea* (L.) Mill.	41%	19%	40%	78	394	472
-	*Ornithogalum etruscum* Parl. subsp. *etruscum*	87%	8%	5%	19	63	82
-	*Phyteuma ovatum* Honck. subsp. *pseudospicatum* Pignatti	75%	4%	21%	31	47	78
-	*Polygala flavescens* DC. subsp. *flavescens*	40%	48%	12%	143	251	394
-	*Ranunculus pollinensis* (N.Terracc.) Chiov.	69%	22%	9%	29	120	149
-	*Saxifraga porophylla* Bertol. subsp. *porophylla*	62%	27%	11%	43	75	118
-	*Sedum magellense* Ten. subsp. *magellense*	68%	28%	4%	47	49	96
-	*Siler montanum* Crantz subsp. *siculum* (Spreng.) Iamonico, Bartolucci & F.Conti	52%	36%	12%	60	165	225
-	*Viola eugeniae* Parl. subsp. *eugeniae*	67%	25%	8%	37	145	182
-	*Viola ferrarinii* Moraldo & Ricceri	50%	3%	47%	17	69	86

Table S.1.D. Number of occurrence records after cleaning steps. The two dataset share strong similarities in terms of number of occurrence records. His = historical; Mod = modern.

Species	His	Mod	Test	His	Mod	Test
	Set I: 3 variables			Set II: 3 axes of PCA (35 variables)		
- *Adenostyles australis* (Ten.) Iamonico & Pignatti	61	61	252	61	61	252
- *Aubrieta columnae* Guss. subsp. *columnae*	19	19	76	19	19	76
- *Bellevalia webbiana* Parl.	20	20	45	20	20	45
- *Cardamine apennina* Lihová & Marhold	28	28	65	28	28	65
- *Carduus affinis* Guss. subsp. *affinis*	60	60	133	60	60	133
- *Crocus biflorus* Mill.	73	73	330	73	73	330
- *Crocus etruscus* Parl.	7	7	114	7	7	112
- *Crocus imperati* Ten.	20	20	45	20	20	45
- *Erysimum pseudorhaeticum* Polatschek	45	45	330	45	45	328
- *Galium magellense* Ten.	39	39	128	39	39	128
- *Gypsophila arrostoi* Guss. subsp. *arrostoi*	23	23	128	23	23	126
- *Helleborus viridis* L. subsp. *bocconei* (Ten.) Peruzzi	36	36	256	36	36	256
- *Klasea flavescens* (L.) Holub subsp. *cichoracea* (L.) Greuter & Wagenitz	21	21	126	21	21	126
- *Koeleria splendens* C.Presl	83	83	368	83	83	368
- *Leontodon anomalus* Ball	18	18	106	18	18	106
- *Linaria purpurea* (L.) Mill.	78	78	472	78	78	472
- *Ornithogalum etruscum* Parl. subsp. *etruscum*	19	19	82	19	19	79
- *Phyteuma ovatum* Honck. subsp. *pseudospicatum* Pignatti	31	31	78	31	31	78
- *Polygala flavescens* DC. subsp. *flavescens*	143	143	394	143	143	394
- *Ranunculus pollinensis* (N.Terracc.) Chiov.	29	29	149	29	29	149
- *Saxifraga porophylla* Bertol. subsp. *porophylla*	43	43	118	43	43	118
- *Sedum magellense* Ten. subsp. *magellense*	40	40	89	40	40	89
- *Siler montanum* Crantz subsp. *siculum* (Spreng.) Iamonico, Bartolucci & F.Conti	60	60	225	60	60	225
- *Viola eugeniae* Parl. subsp. *eugeniae*	37	37	182	37	37	182
- *Viola ferrarinii* Moraldo & Ricceri	17	17	86	17	17	86
