# Supplementary material for: Assessing the Effectiveness of Correlative Ecological Niche Model Temporal Projection through Floristic Data
Source: Biology (Basel). 2022 Aug 14;11(8):1219. doi: 10.3390/biology11081219 (PMC9405103; doi:10.3390/biology11081219)
Supplement: Supplementary file 1 [file biology-11-01219-s001.zip › Dolci & Peruzzi Supplementary Material S.1-9.zip!/Supplementary material S.2.pdf]

Supplementary material S.2

Results of all modeling procedures obtained by using 3 bioclimatic variables (annual mean temperature, annual precipitation, and annual potential evapotranspiration).

S.2.1 *Adenostyles australis* Biomod2

Species: *Adenostyles australis* (Ten.) Iamonico & Pignatti

Procedure: Biomod2

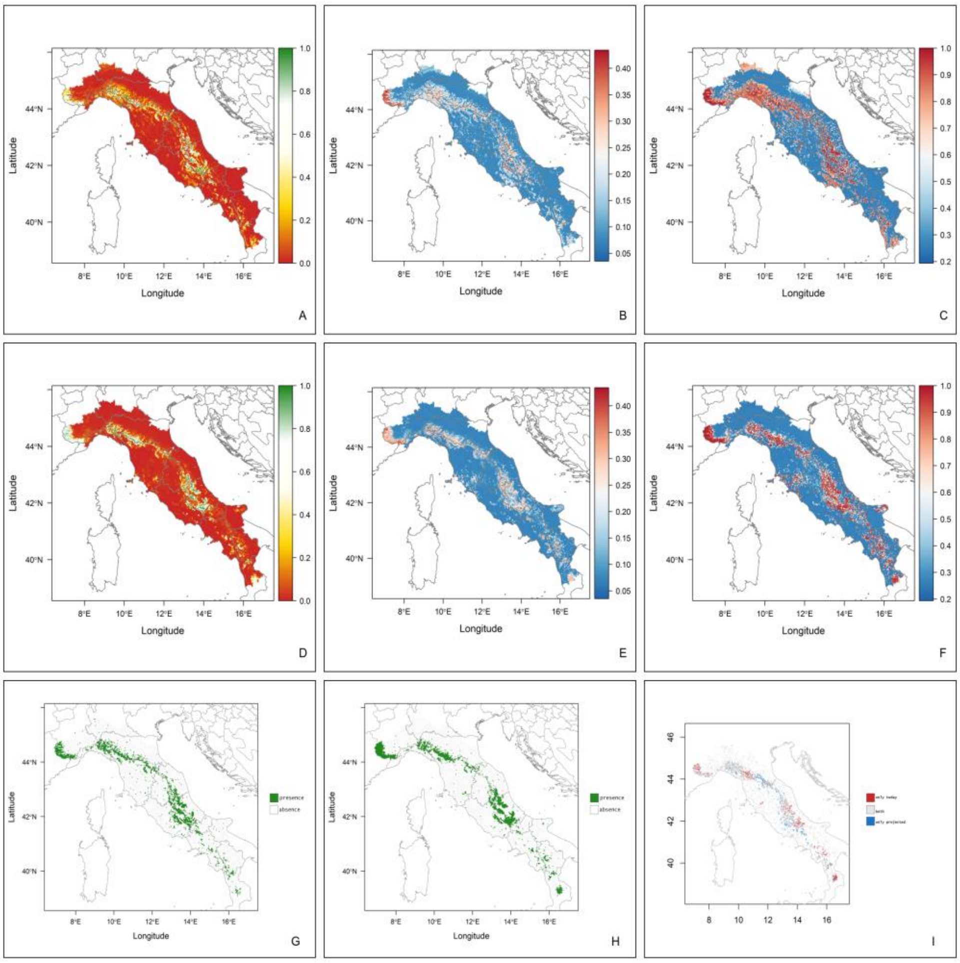

Fig. S.2.1-A) 'Projected' base output; B) 'Projected' uncertainty standard deviation; C) 'Projected' uncertainty range of values; D) 'Present' base output; E) 'Present' uncertainty standard deviation; F) 'Present' uncertainty range of values; G) 'Projected' binary distribution; H) 'Present' binary distribution; I) Range size variation.

Value	Present	Projected
AUC.train	0.985	0.982
AUC.test	0.932	0.873
AUC.diff	0.109	0.109
pROC	1.682	1.375
OR.p10	0.400	0.417
CBI	0.865	0.976
perc.stable	55.589	55.589
range.size	-0.008	-0.008

S.2.2 *Adenostyles australis* Bioclim

Species: *Adenostyles australis* (Ten.) Iamonico & Pignatti

Procedure: Bioclim

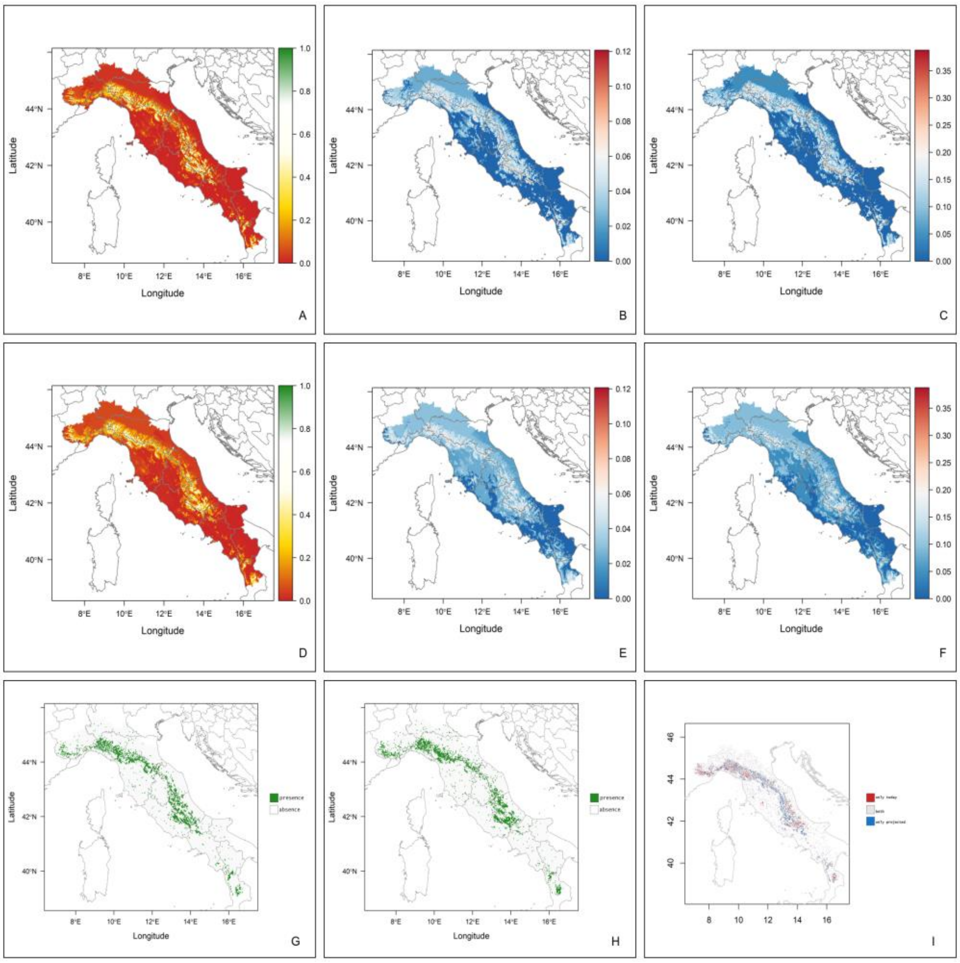

Fig. S.2.2-A) ‘Projected’ base output; B) ‘Projected’ uncertainty standard deviation; C) ‘Projected’ uncertainty range of values; D) ’Present’ base output; E) ’Present’ uncertainty standard deviation; F) ’Present’ uncertainty range of values; G) ‘Projected’ binary distribution; H) ‘Present’ binary distribution; I) Range size variation.

Value	Present	Projected
AUC.train	0.868	0.848
AUC.test	0.822	0.795
AUC.diff	0.063	0.063
pROC	1.212	1.154
OR.p10	0.156	0.206
CBI	0.725	0.787
perc.stable	39.528	39.528
range.size	-2.421	-2.421

S.2.3 *Adenostyles australis* Domain

Species: *Adenostyles australis* (Ten.) Iamonico & Pignatti

Procedure: Domain

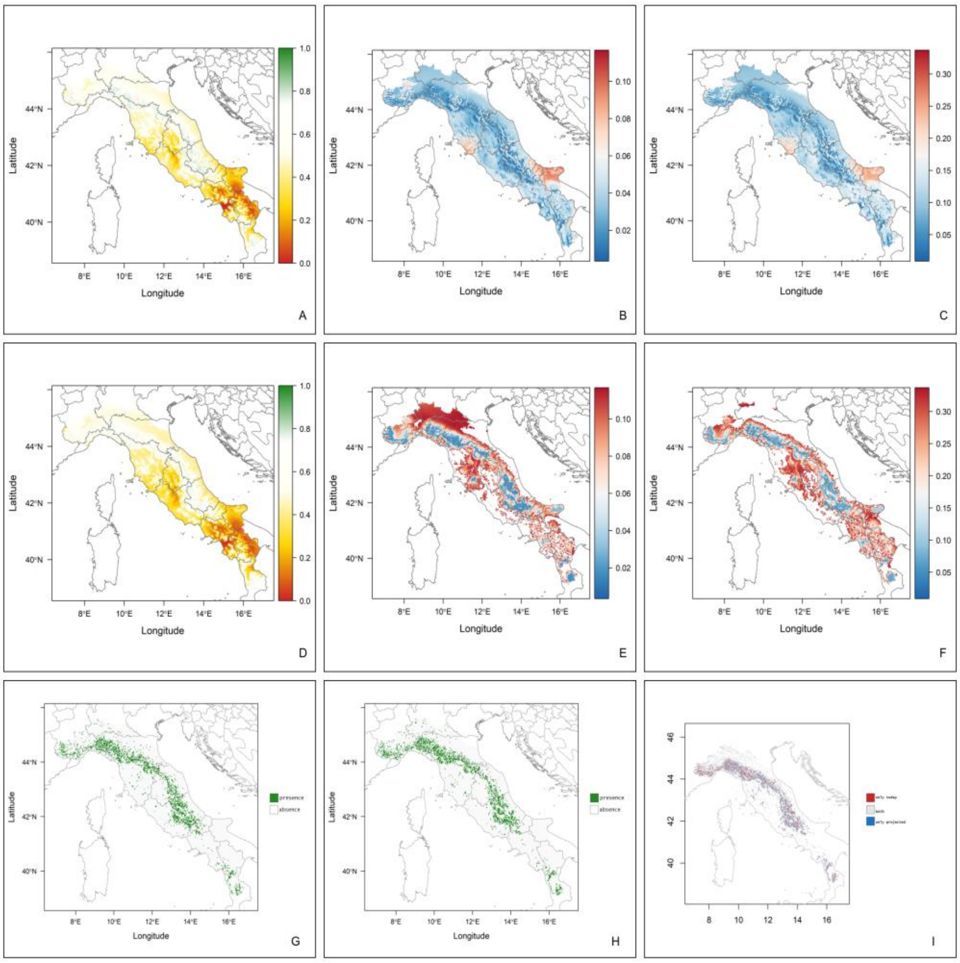

Fig. S.2.3-A) 'Projected' base output; B) 'Projected' uncertainty standard deviation; C) 'Projected' uncertainty range of values; D) 'Present' base output; E) 'Present' uncertainty standard deviation; F) 'Present' uncertainty range of values; G) 'Projected' binary distribution; H) 'Present' binary distribution; I) Range size variation.

Value	Present	Projected
AUC.train	0.850	0.830
AUC.test	0.813	0.825
AUC.diff	0.029	0.029
pROC	1.322	1.352
OR.p10	0.178	0.128
CBI	0.750	0.845
perc.stable	33.285	33.285
range.size	-0.306	-0.306

S.2.4 *Adenostyles australis* Maxent

Species: *Adenostyles australis* (Ten.) Iamonico & Pignatti

Procedure: Maxent

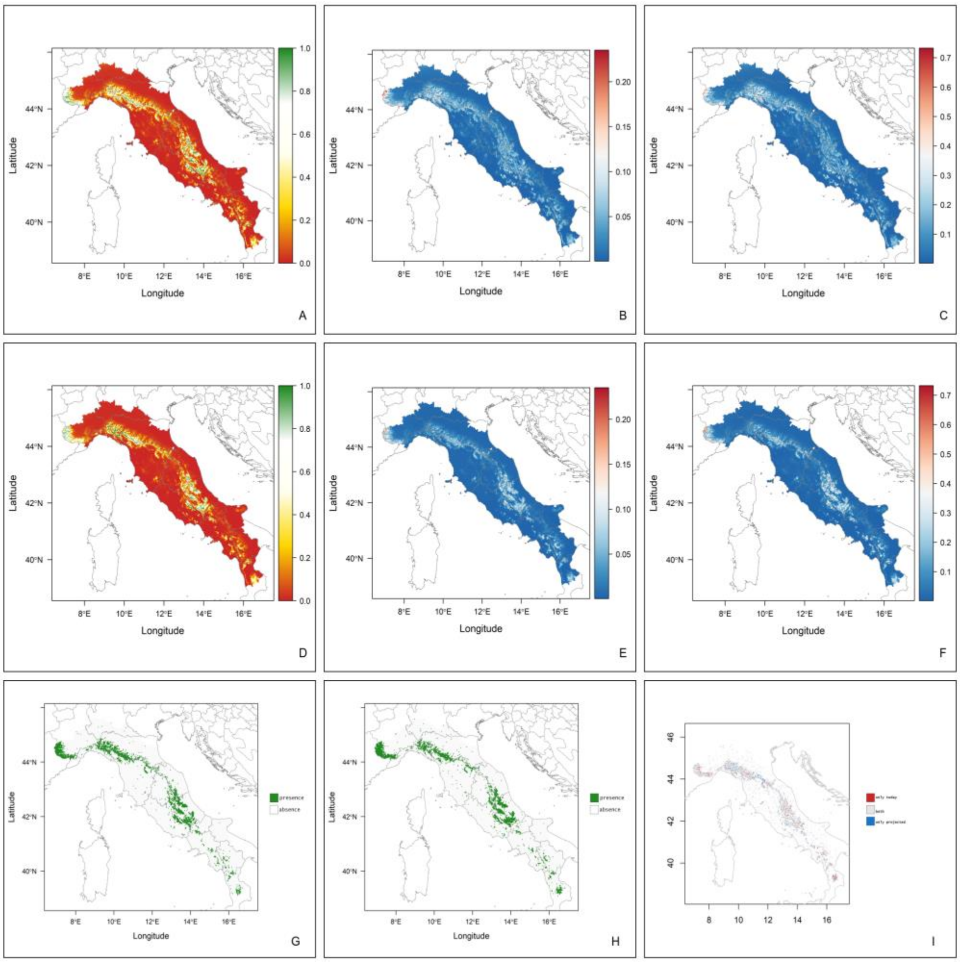

Fig. S.2.4-A) 'Projected' base output; B) 'Projected' uncertainty standard deviation; C) 'Projected' uncertainty range of values; D) 'Present' base output; E) 'Present' uncertainty standard deviation; F) 'Present' uncertainty range of values; G) 'Projected' binary distribution; H) 'Present' binary distribution; I) Range size variation.

Value	Present	Projected
AUC.train	0.929	0.903
AUC.test	0.922	0.906
AUC.diff	0.020	0.020
pROC	1.556	1.550
OR.p10	0.144	0.122
CBI	0.969	0.975
perc.stable	65.162	65.162
range.size	0.285	0.285

S.2.5 *Adenostyles australis* KDE

Species: *Adenostyles australis* (Ten.) Iamonico & Pignatti

Procedure: KDE

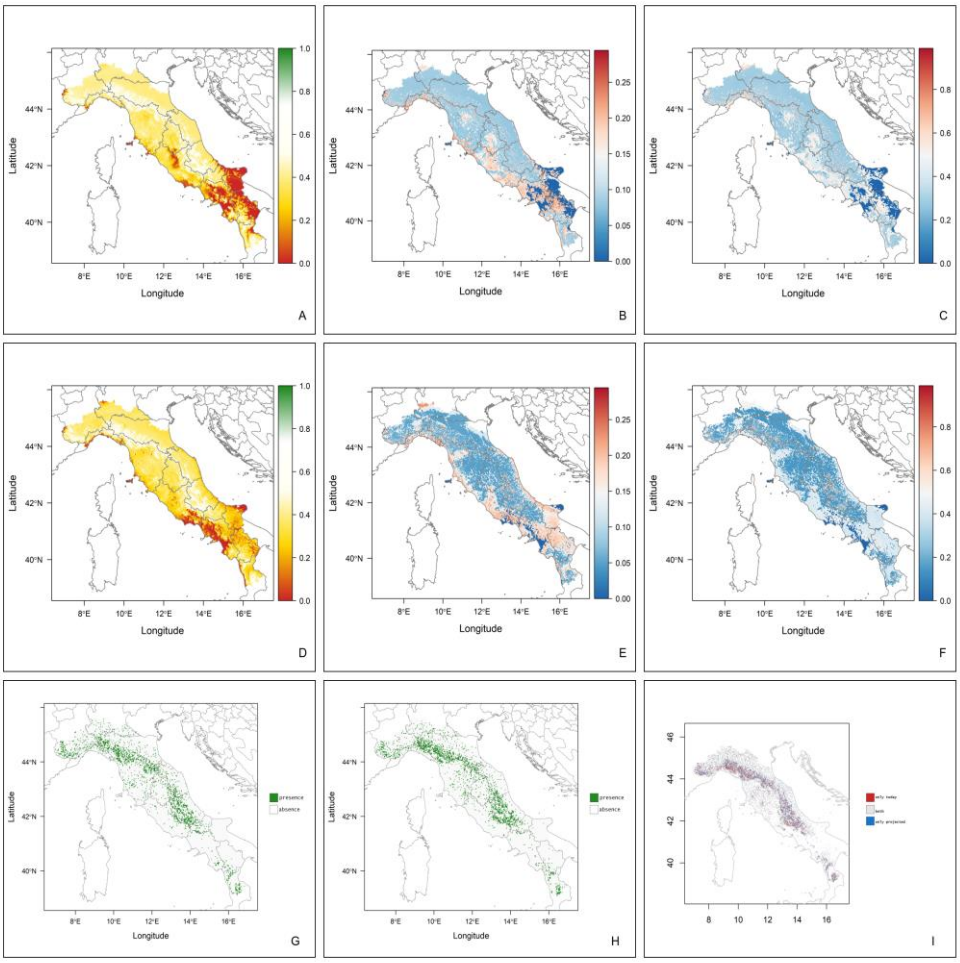

Fig. S.2.5-A) 'Projected' base output; B) 'Projected' uncertainty standard deviation; C) 'Projected' uncertainty range of values; D) 'Present' base output; E) 'Present' uncertainty standard deviation; F) 'Present' uncertainty range of values; G) 'Projected' binary distribution; H) 'Present' binary distribution; I) Range size variation.

Value	Present	Projected
AUC.train	0.878	0.869
AUC.test	0.893	0.845
AUC.diff	0.019	0.036
pROC	1.522	1.343
OR.p10	0.133	0.161
CBI	0.954	0.920
perc.stable	20.442	20.442
range.size	1.342	1.342

S.2.6 *Adenostyles australis* CTA

Species: *Adenostyles australis* (Ten.) Iamonico & Pignatti

Procedure: CTA

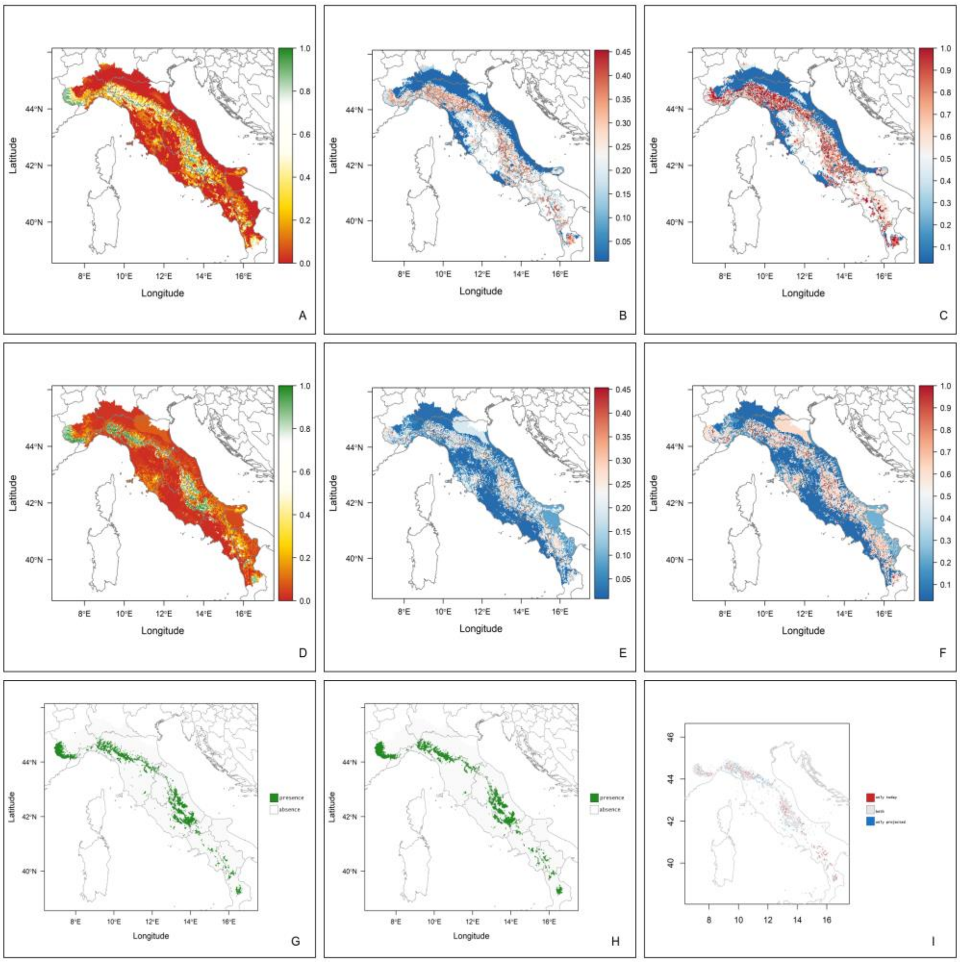

Fig. S.2.6-A) 'Projected' base output; B) 'Projected' uncertainty standard deviation; C) 'Projected' uncertainty range of values; D) 'Present' base output; E) 'Present' uncertainty standard deviation; F) 'Present' uncertainty range of values; G) 'Projected' binary distribution; H) 'Present' binary distribution; I) Range size variation.

Value	Present	Projected
AUC.train	0.956	0.957
AUC.test	0.900	0.866
AUC.diff	0.091	0.091
pROC	1.255	1.145
OR.p10	0.211	0.222
CBI	0.883	0.987
perc.stable	76.247	76.247
range.size	-0.258	-0.258

S.2.7 *Adenostyles australis* FDA

Species: *Adenostyles australis* (Ten.) Iamonico & Pignatti

Procedure: FDA

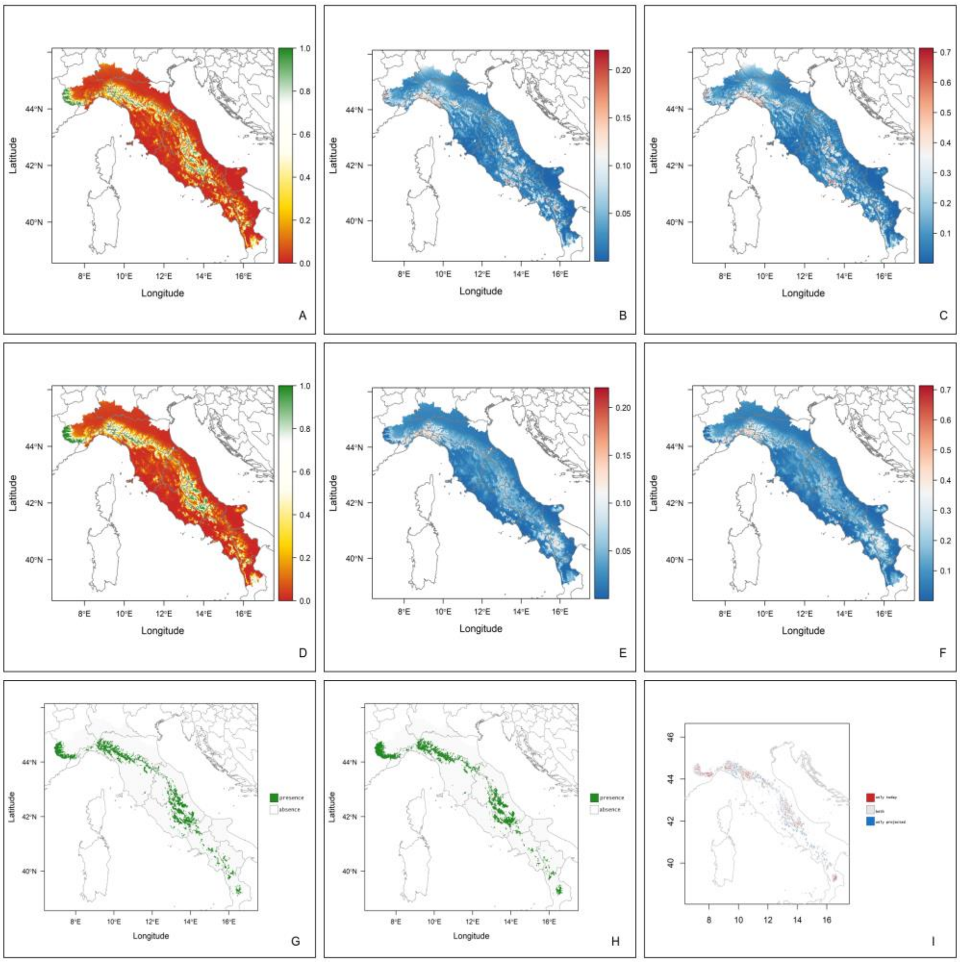

Fig. S.2.7-A) ‘Projected’ base output; B) ‘Projected’ uncertainty standard deviation; C) ‘Projected’ uncertainty range of values; D) ’Present’ base output; E) ’Present’ uncertainty standard deviation; F) ’Present’ uncertainty range of values; G) ‘Projected’ binary distribution; H) ‘Present’ binary distribution; I) Range size variation.

Value	Present	Projected
AUC.train	0.921	0.902
AUC.test	0.898	0.920
AUC.diff	0.006	0.006
pROC	1.476	1.535
OR.p10	0.194	0.078
CBI	0.936	0.979
perc.stable	73.982	73.982
range.size	1.134	1.134

S.2.8 *Adenostyles australis* GAM

Species: *Adenostyles australis* (Ten.) Iamonico & Pignatti

Procedure: GAM

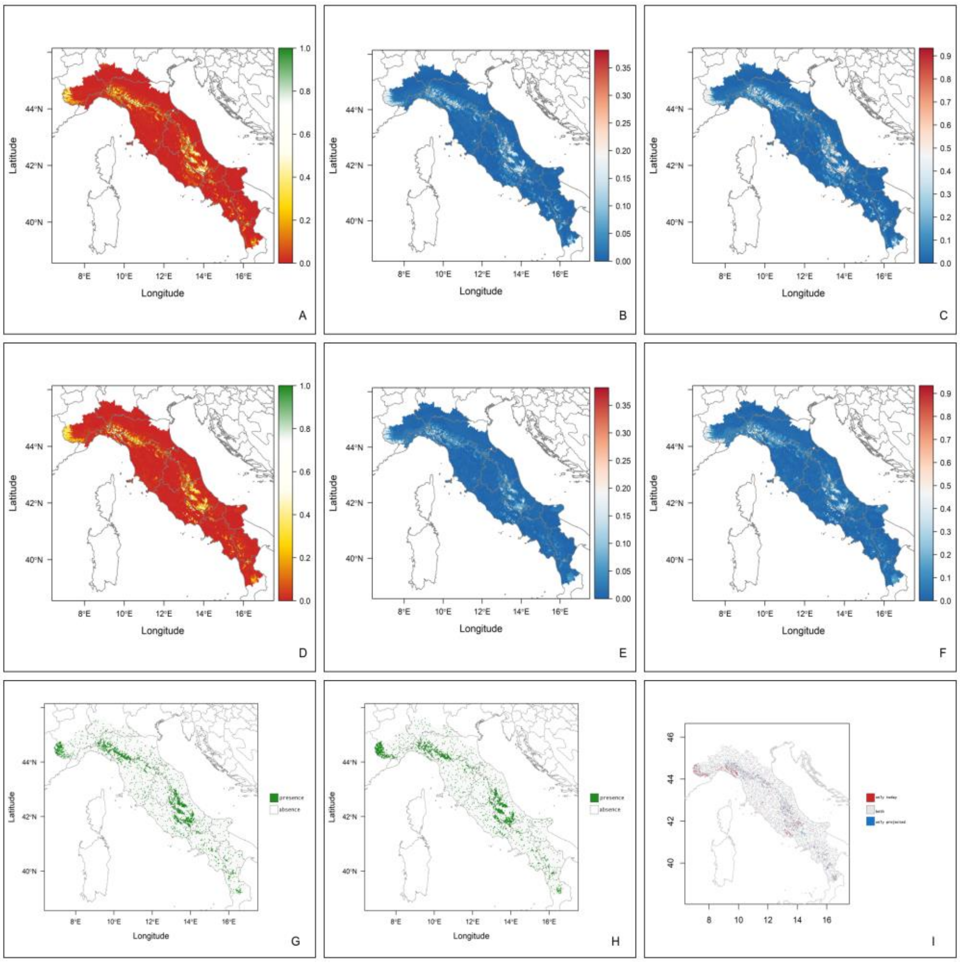

Fig. S.2.8-A) 'Projected' base output; B) 'Projected' uncertainty standard deviation; C) 'Projected' uncertainty range of values; D) 'Present' base output; E) 'Present' uncertainty standard deviation; F) 'Present' uncertainty range of values; G) 'Projected' binary distribution; H) 'Present' binary distribution; I) Range size variation.

Value	Present	Projected
AUC.train	0.940	0.916
AUC.test	0.926	0.886
AUC.diff	0.037	0.037
pROC	1.551	1.402
OR.p10	0.139	0.156
CBI	0.990	0.895
perc.stable	30.990	30.990
range.size	0.004	0.004

S.2.9 *Adenostyles australis* GLM

Species: *Adenostyles australis* (Ten.) Iamonico & Pignatti

Procedure: GLM

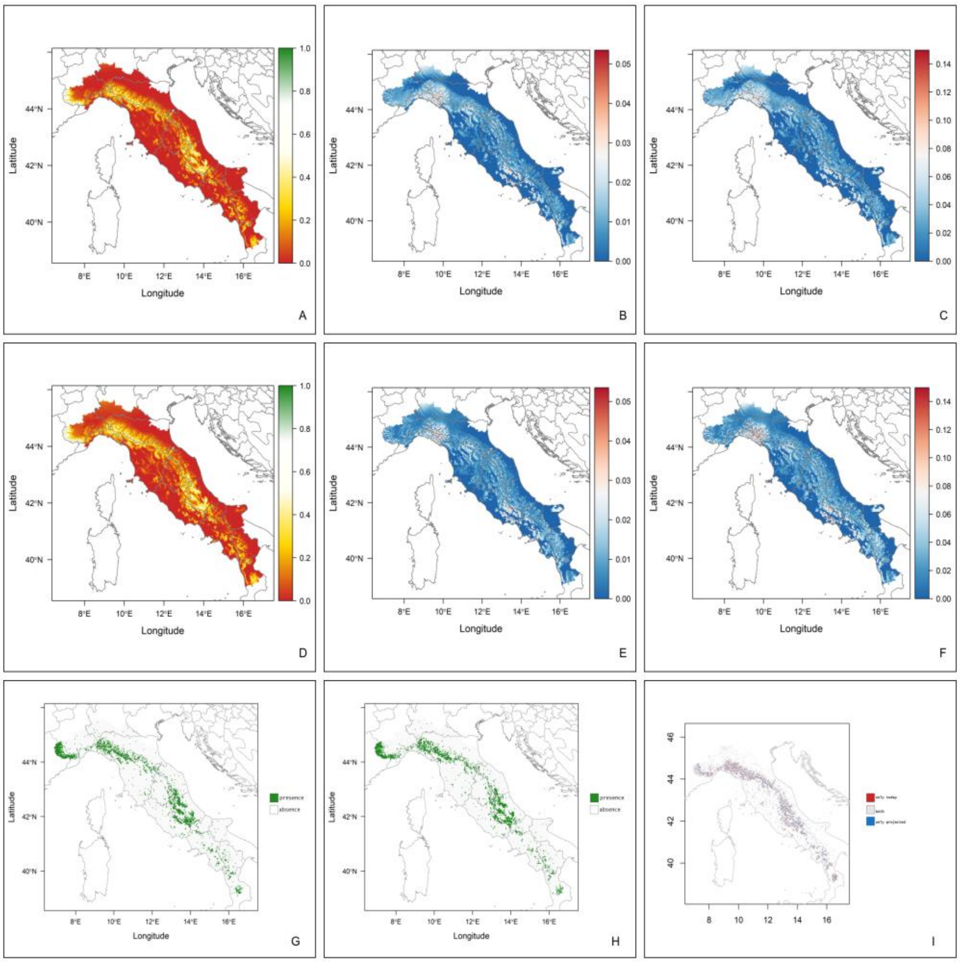

Fig. S.2.9-A) 'Projected' base output; B) 'Projected' uncertainty standard deviation; C) 'Projected' uncertainty range of values; D) 'Present' base output; E) 'Present' uncertainty standard deviation; F) 'Present' uncertainty range of values; G) 'Projected' binary distribution; H) 'Present' binary distribution; I) Range size variation.

Value	Present	Projected
AUC.train	0.915	0.895
AUC.test	0.915	0.901
AUC.diff	0.012	0.012
pROC	1.519	1.509
OR.p10	0.128	0.128
CBI	0.603	0.538
perc.stable	46.015	46.015
range.size	-1.452	-1.452

S.2.10 *Adenostyles australis* MARS

Species: *Adenostyles australis* (Ten.) Iamonico & Pignatti

Procedure: MARS

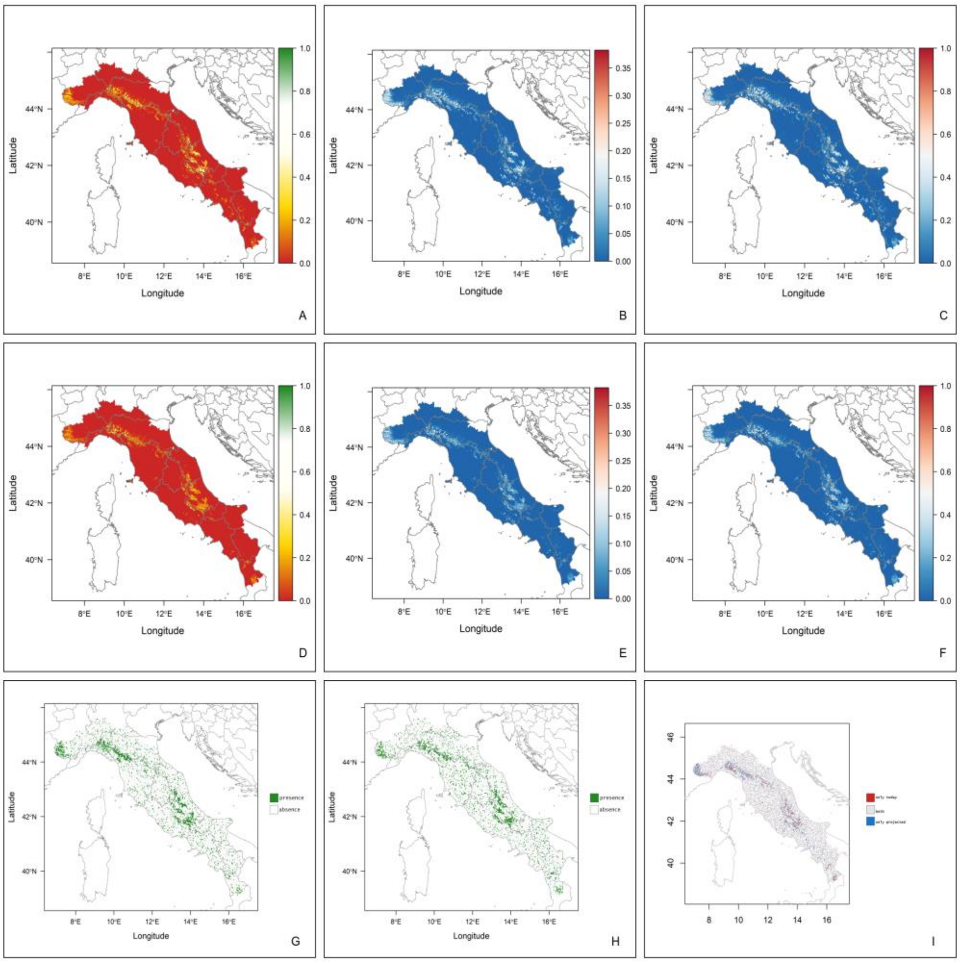

Fig. S.2.10-A) 'Projected' base output; B) 'Projected' uncertainty standard deviation; C) 'Projected' uncertainty range of values; D) 'Present' base output; E) 'Present' uncertainty standard deviation; F) 'Present' uncertainty range of values; G) 'Projected' binary distribution; H) 'Present' binary distribution; I) Range size variation.

Value	Present	Projected
AUC.train	0.924	0.906
AUC.test	0.931	0.843
AUC.diff	0.063	0.063
pROC	1.550	1.211
OR.p10	0.128	0.211
CBI	0.894	0.849
perc.stable	16.903	16.903
range.size	-0.206	-0.206

S.2.11 *Adenostyles australis* RF

Species: *Adenostyles australis* (Ten.) Iamonico & Pignatti

Procedure: RF

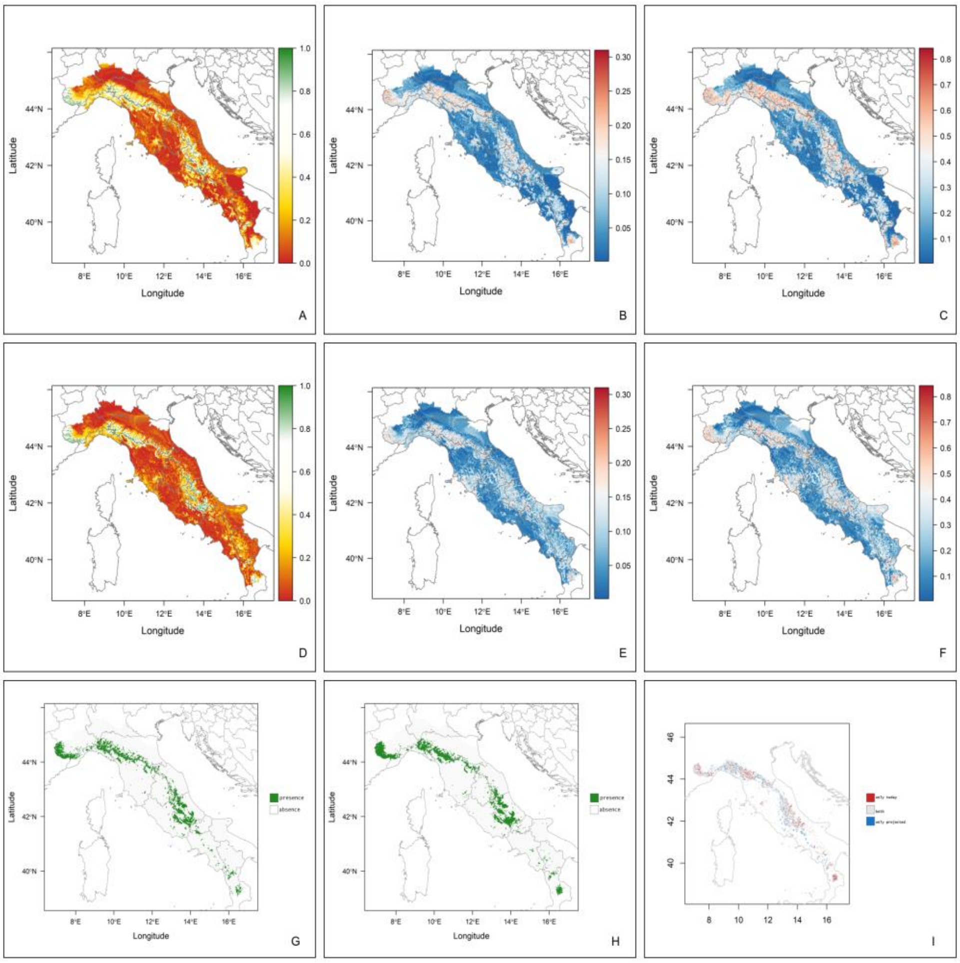

Fig. S.2.11-A) 'Projected' base output; B) 'Projected' uncertainty standard deviation; C) 'Projected' uncertainty range of values; D) 'Present' base output; E) 'Present' uncertainty standard deviation; F) 'Present' uncertainty range of values; G) 'Projected' binary distribution; H) 'Present' binary distribution; I) Range size variation.

Value	Present	Projected
AUC.train	1.000	1.000
AUC.test	0.966	0.953
AUC.diff	0.047	0.047
pROC	1.549	1.407
OR.p10	0.367	0.411
CBI	0.955	0.971
perc.stable	65.780	65.780
range.size	-0.329	-0.329

S.2.12 *Adenostyles australis* SVM

Species: *Adenostyles australis* (Ten.) Iamonico & Pignatti

Procedure: SVM

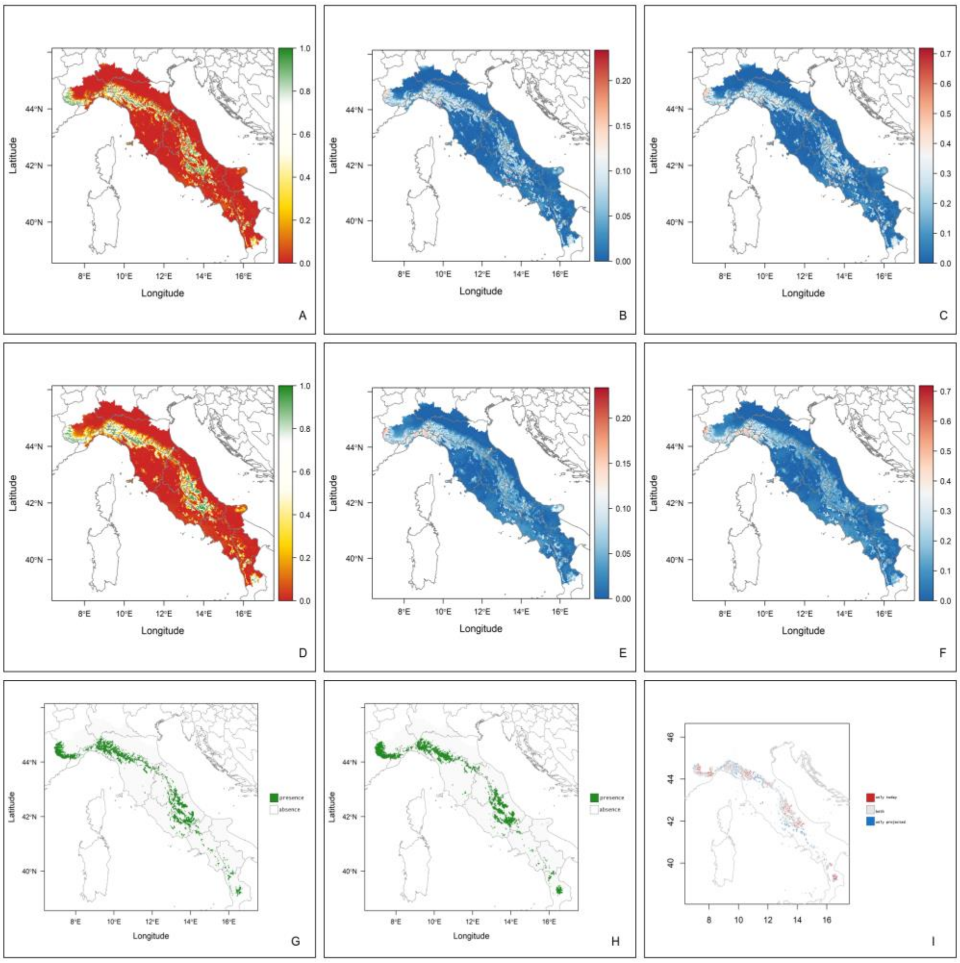

Fig. S.2.12-A) 'Projected' base output; B) 'Projected' uncertainty standard deviation; C) 'Projected' uncertainty range of values; D) 'Present' base output; E) 'Present' uncertainty standard deviation; F) 'Present' uncertainty range of values; G) 'Projected' binary distribution; H) 'Present' binary distribution; I) Range size variation.

Value	Present	Projected
AUC.train	0.939	0.908
AUC.test	0.924	0.892
AUC.diff	0.031	0.031
pROC	1.553	1.417
OR.p10	0.128	0.144
CBI	0.898	0.964
perc.stable	70.976	70.976
range.size	-0.253	-0.253

S.2.13 *Aubrieta columnae* Biomod2

Species: *Aubrieta columnae* Guss. subsp. columnae

Procedure: Biomod2

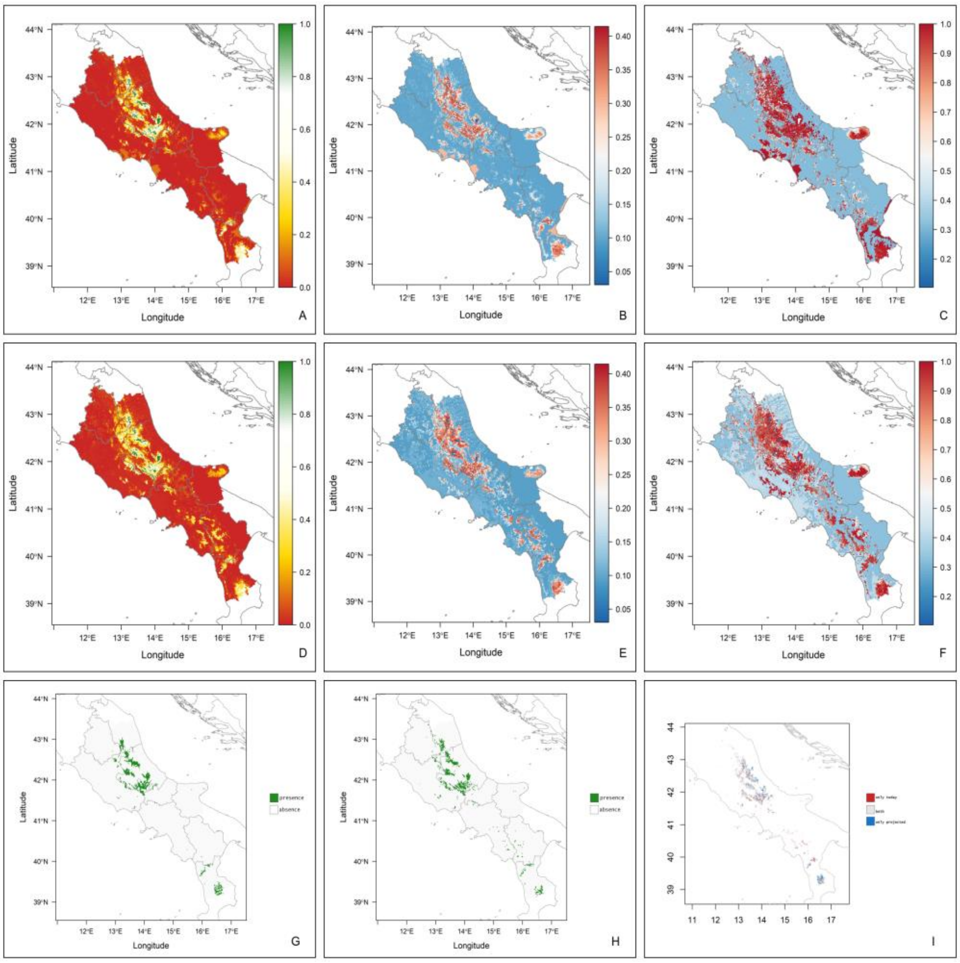

Fig. S.2.13-A) ‘Projected’ base output; B) ‘Projected’ uncertainty standard deviation; C) ‘Projected’ uncertainty range of values; D) ’Present’ base output; E) ’Present’ uncertainty standard deviation; F) ’Present’ uncertainty range of values; G) ‘Projected’ binary distribution; H) ‘Present’ binary distribution; I) Range size variation.

Value	Present	Projected
AUC.train	0.994	0.993
AUC.test	0.881	0.945
AUC.diff	0.047	0.047
pROC	1.628	1.783
OR.p10	0.667	0.383
CBI	0.922	0.876
perc.stable	49.154	49.154
range.size	1.952	1.952

S.2.14 *Aubrieta columnae* Bioclim

Species: *Aubrieta columnae* Guss. subsp. columnae

Procedure: Bioclim

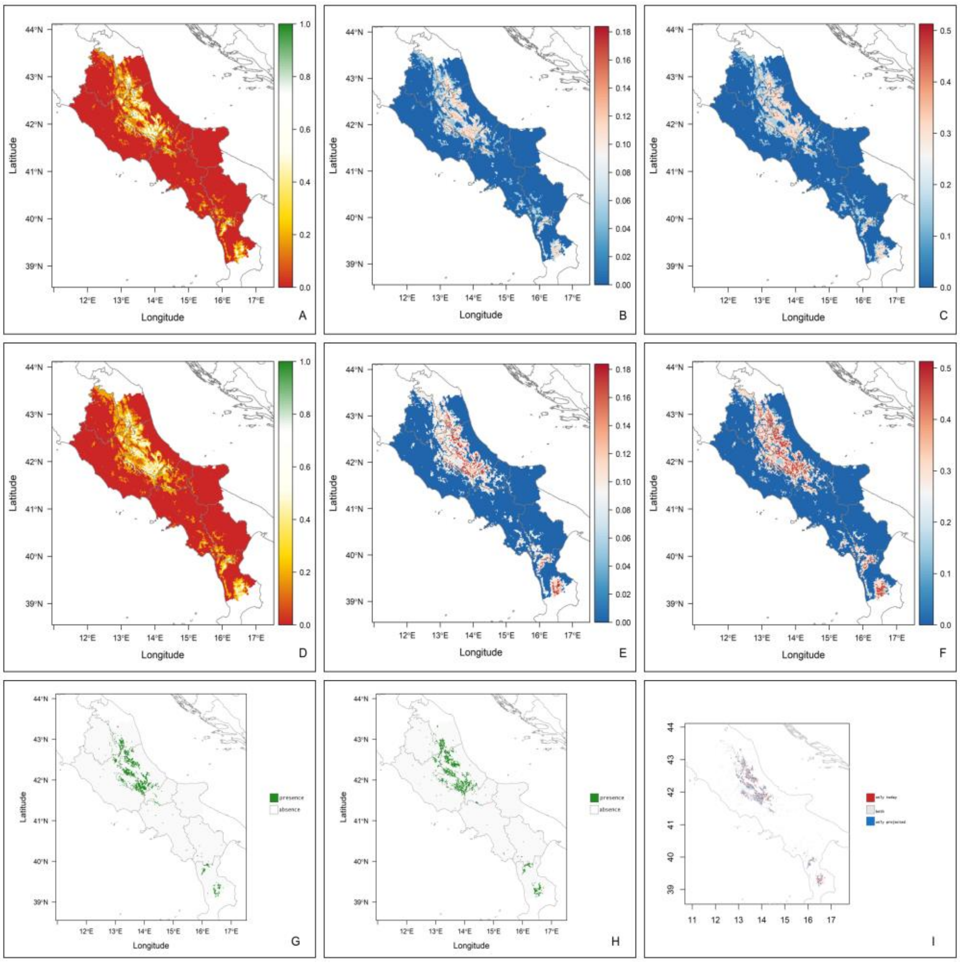

Fig. S.2.14-A) 'Projected' base output; B) 'Projected' uncertainty standard deviation; C) 'Projected' uncertainty range of values; D) 'Present' base output; E) 'Present' uncertainty standard deviation; F) 'Present' uncertainty range of values; G) 'Projected' binary distribution; H) 'Present' binary distribution; I) Range size variation.

Value	Present	Projected
AUC.train	0.906	0.909
AUC.test	0.785	0.818
AUC.diff	0.098	0.098
pROC	1.338	1.395
OR.p10	0.233	0.200
CBI	0.473	0.842
perc.stable	44.840	44.840
range.size	-0.637	-0.637

S.2.15 *Aubrieta columnae* Domain

Species: *Aubrieta columnae* Guss. subsp. columnae

Procedure: Domain

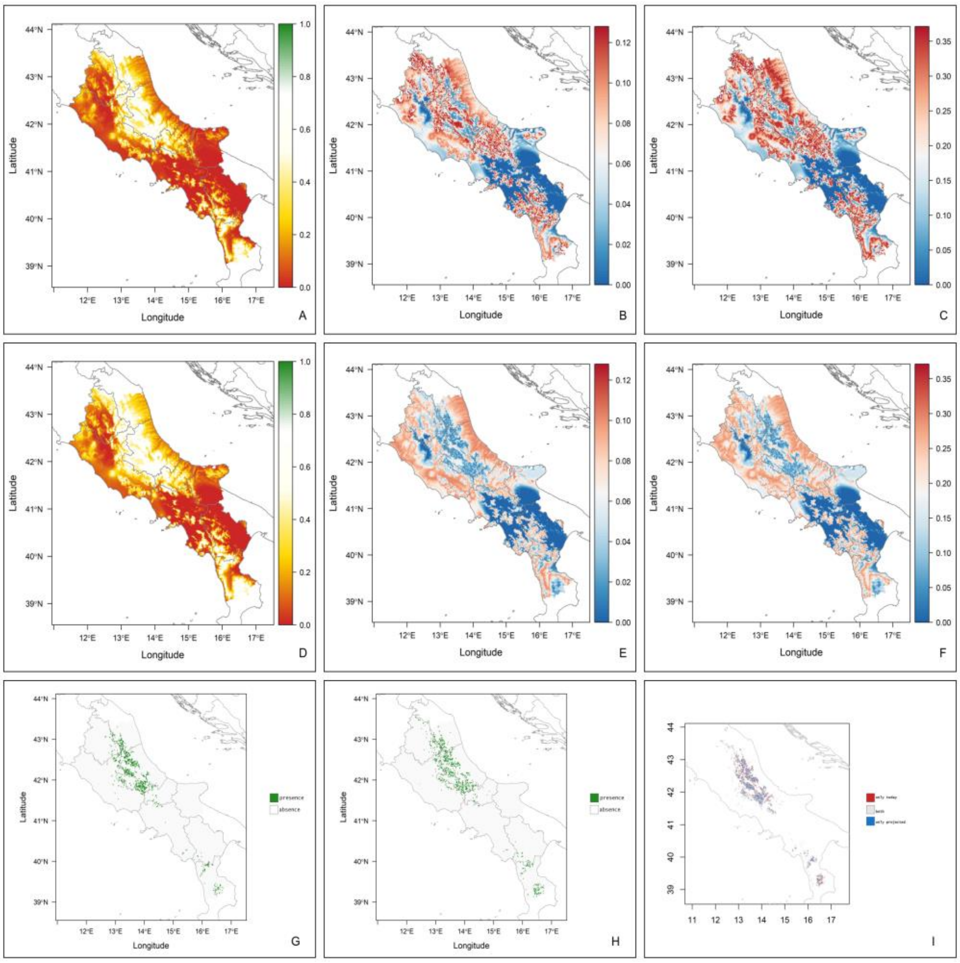

Fig. S.2.15-A) ‘Projected’ base output; B) ‘Projected’ uncertainty standard deviation; C) ‘Projected’ uncertainty range of values; D) ’Present’ base output; E) ’Present’ uncertainty standard deviation; F) ’Present’ uncertainty range of values; G) ‘Projected’ binary distribution; H) ‘Present’ binary distribution; I) Range size variation.

Value	Present	Projected
AUC.train	0.895	0.918
AUC.test	0.869	0.903
AUC.diff	0.022	0.022
pROC	1.668	1.747
OR.p10	0.450	0.317
CBI	0.916	0.780
perc.stable	21.087	21.087
range.size	0.000	0.000

S.2.16 *Aubrieta columnae* Maxent

Species: *Aubrieta columnae* Guss. subsp. columnae

Procedure: Maxent

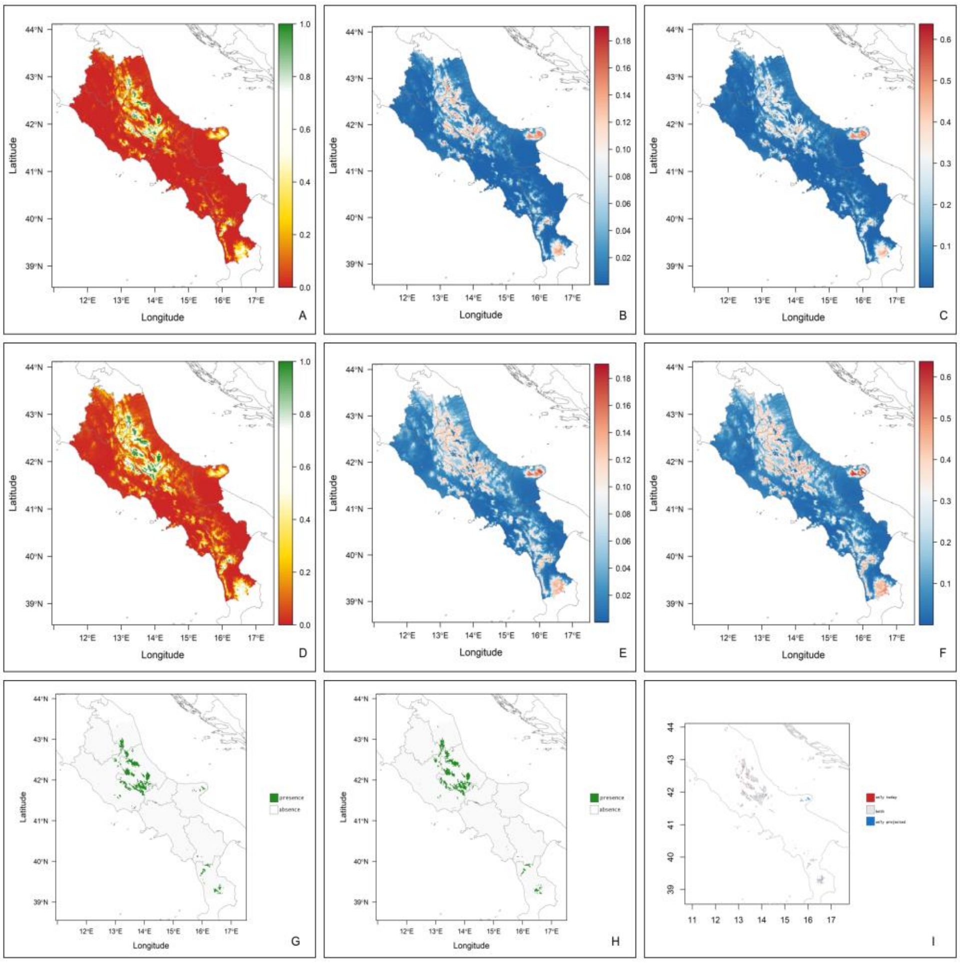

Fig. S.2.16-A) ‘Projected’ base output; B) ‘Projected’ uncertainty standard deviation; C) ‘Projected’ uncertainty range of values; D) ’Present’ base output; E) ’Present’ uncertainty standard deviation; F) ’Present’ uncertainty range of values; G) ‘Projected’ binary distribution; H) ‘Present’ binary distribution; I) Range size variation.

Value	Present	Projected
AUC.train	0.934	0.947
AUC.test	0.938	0.946
AUC.diff	0.020	0.020
pROC	1.796	1.803
OR.p10	0.183	0.200
CBI	0.827	0.869
perc.stable	68.083	68.083
range.size	1.293	1.293

S.2.17 *Aubrieta columnae* KDE

Species: *Aubrieta columnae* Guss. subsp. columnae

Procedure: KDE

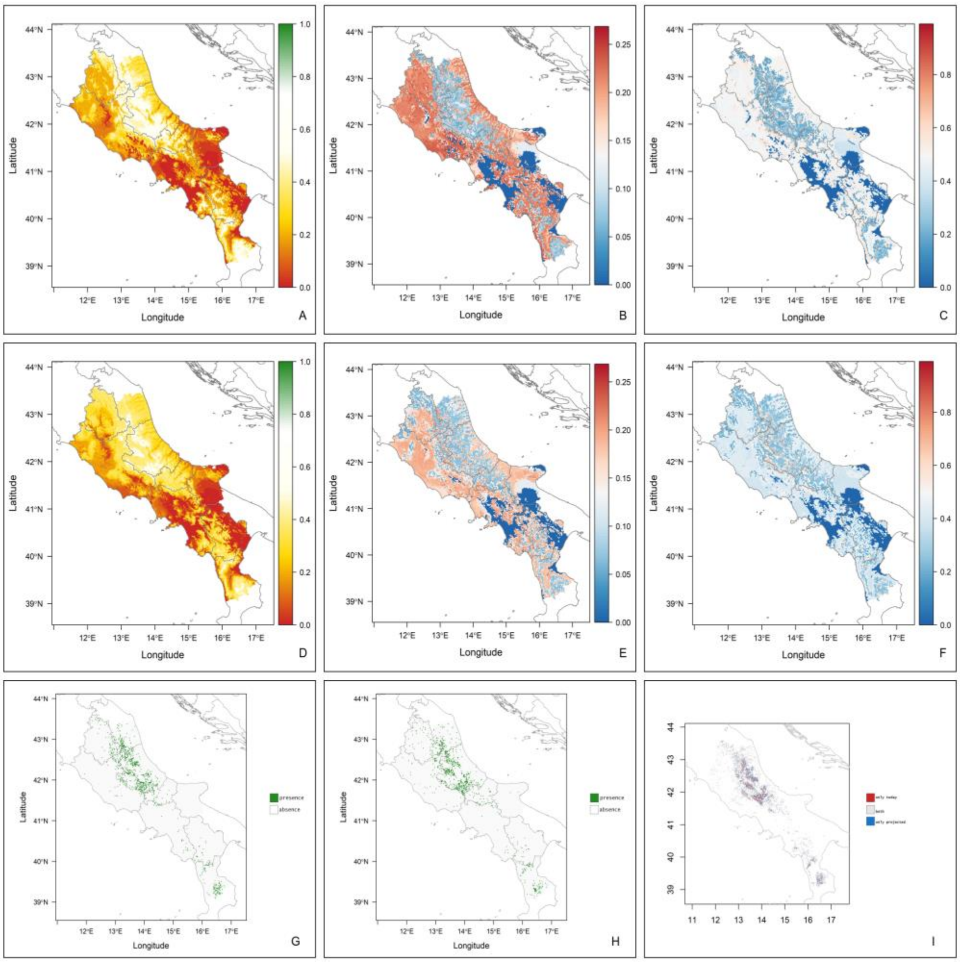

Fig. S.2.17-A) 'Projected' base output; B) 'Projected' uncertainty standard deviation; C) 'Projected' uncertainty range of values; D) 'Present' base output; E) 'Present' uncertainty standard deviation; F) 'Present' uncertainty range of values; G) 'Projected' binary distribution; H) 'Present' binary distribution; I) Range size variation.

Value	Present	Projected
AUC.train	0.918	0.933
AUC.test	0.895	0.850
AUC.diff	0.042	0.084
pROC	1.703	1.586
OR.p10	0.267	0.383
CBI	0.960	0.948
perc.stable	13.102	13.102
range.size	4.530	4.530

S.2.18 *Aubrieta columnae* CTA

Species: *Aubrieta columnae* Guss. subsp. columnae

Procedure: CTA

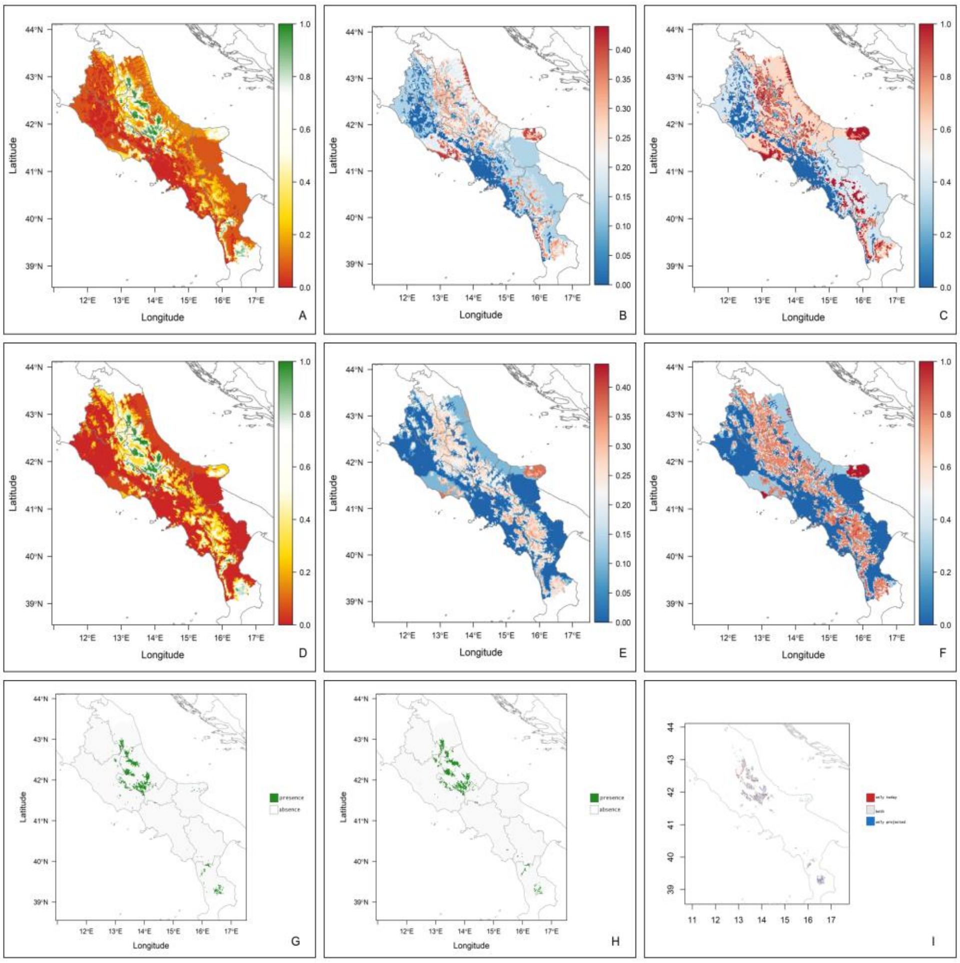

Fig. S.2.18-A) 'Projected' base output; B) 'Projected' uncertainty standard deviation; C) 'Projected' uncertainty range of values; D) 'Present' base output; E) 'Present' uncertainty standard deviation; F) 'Present' uncertainty range of values; G) 'Projected' binary distribution; H) 'Present' binary distribution; I) Range size variation.

Value	Present	Projected
AUC.train	0.959	0.973
AUC.test	0.902	0.936
AUC.diff	0.041	0.041
pROC	1.289	1.255
OR.p10	0.200	0.117
CBI	0.863	0.914
perc.stable	50.043	50.043
range.size	-0.568	-0.568

S.2.19 *Aubrieta columnae* FDA

Species: *Aubrieta columnae* Guss. subsp. columnae

Procedure: FDA

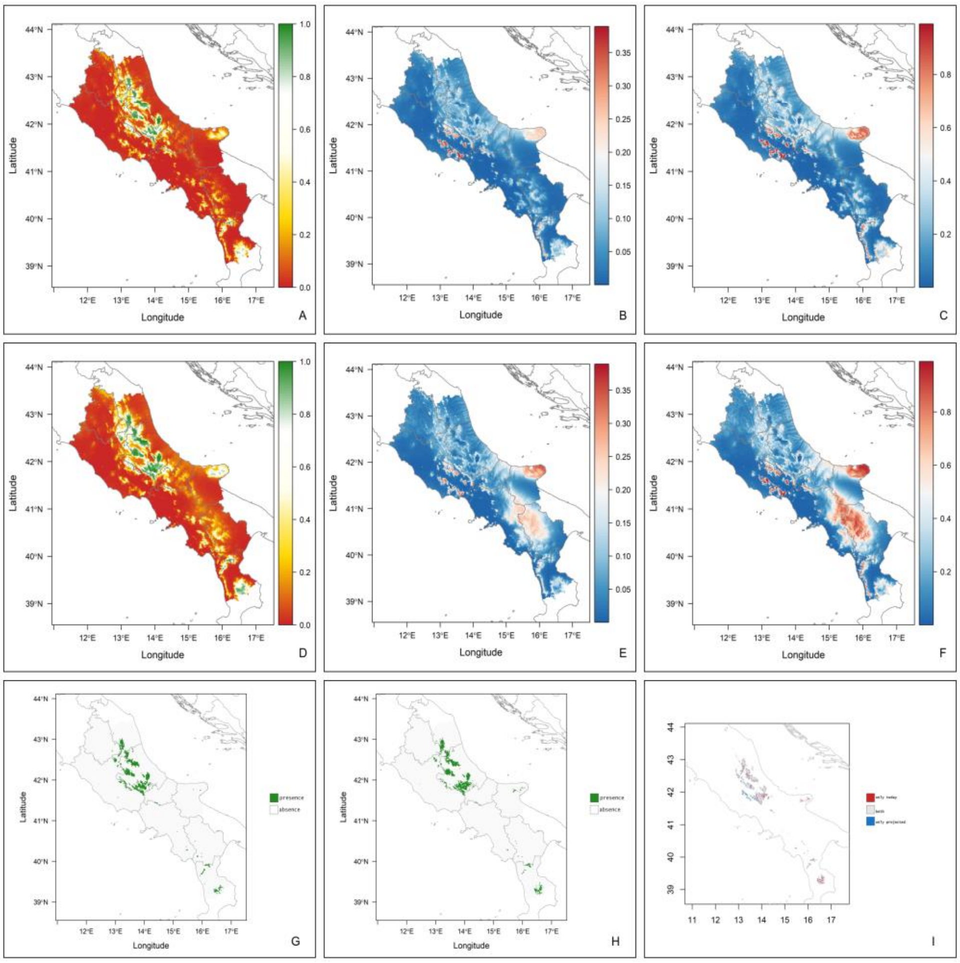

Fig. S.2.19-A) 'Projected' base output; B) 'Projected' uncertainty standard deviation; C) 'Projected' uncertainty range of values; D) 'Present' base output; E) 'Present' uncertainty standard deviation; F) 'Present' uncertainty range of values; G) 'Projected' binary distribution; H) 'Present' binary distribution; I) Range size variation.

Value	Present	Projected
AUC.train	0.951	0.945
AUC.test	0.931	0.930
AUC.diff	0.032	0.032
pROC	1.771	1.776
OR.p10	0.183	0.233
CBI	0.650	0.897
perc.stable	57.554	57.554
range.size	-0.544	-0.544

S.2.20 *Aubrieta columnae* GAM

Species: *Aubrieta columnae* Guss. subsp. columnae

Procedure: GAM

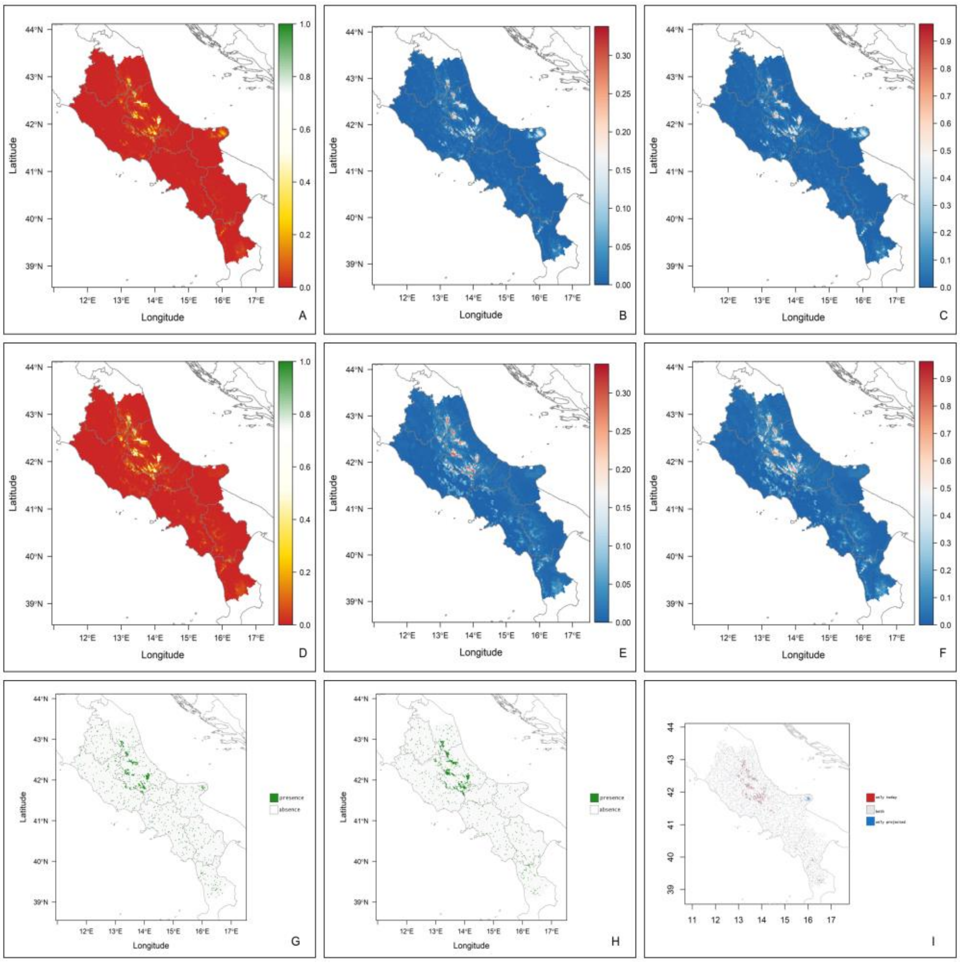

Fig. S.2.20-A) ‘Projected’ base output; B) ‘Projected’ uncertainty standard deviation; C) ‘Projected’ uncertainty range of values; D) ’Present’ base output; E) ’Present’ uncertainty standard deviation; F) ’Present’ uncertainty range of values; G) ‘Projected’ binary distribution; H) ‘Present’ binary distribution; I) Range size variation.

Value	Present	Projected
AUC.train	0.956	0.955
AUC.test	0.830	0.884
AUC.diff	0.076	0.076
pROC	1.507	1.621
OR.p10	0.383	0.283
CBI	0.873	0.785
perc.stable	17.196	17.196
range.size	2.957	2.957

S.2.21 *Aubrieta columnae* GLM

Species: *Aubrieta columnae* Guss. subsp. columnae

Procedure: GLM

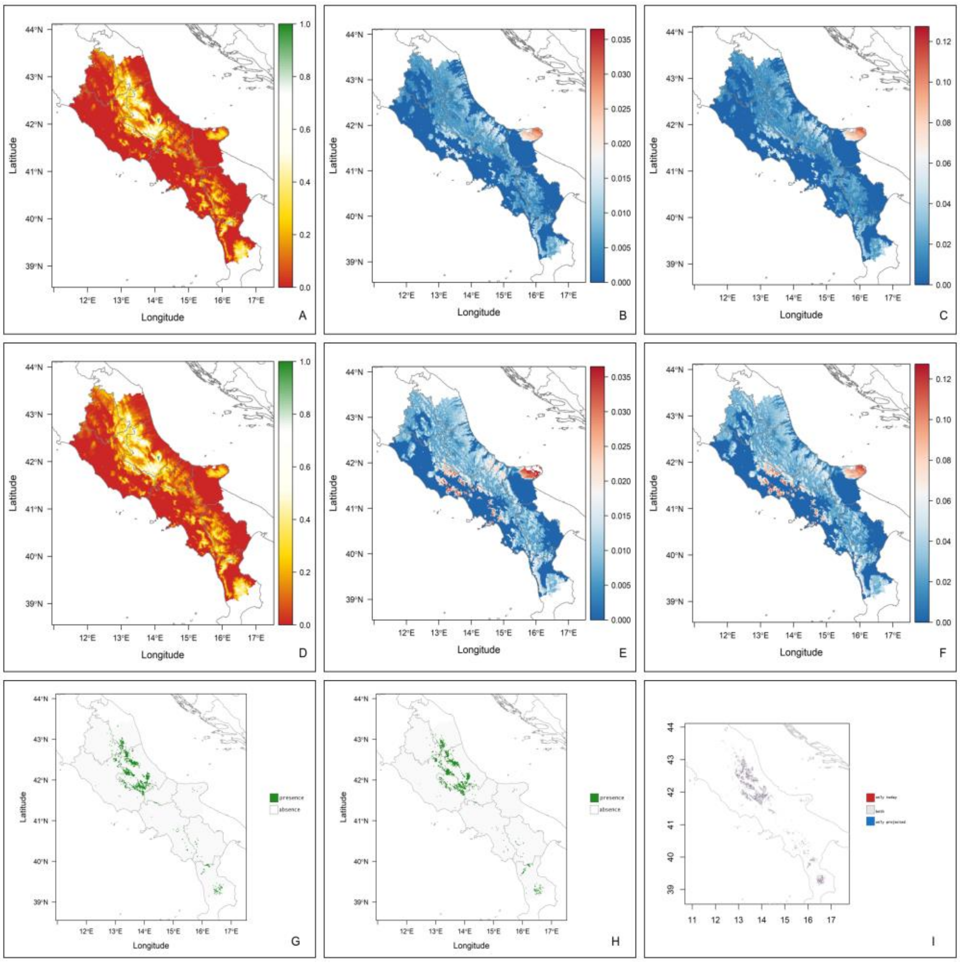

Fig. S.2.21-A) 'Projected' base output; B) 'Projected' uncertainty standard deviation; C) 'Projected' uncertainty range of values; D) 'Present' base output; E) 'Present' uncertainty standard deviation; F) 'Present' uncertainty range of values; G) 'Projected' binary distribution; H) 'Present' binary distribution; I) Range size variation.

Value	Present	Projected
AUC.train	0.944	0.941
AUC.test	0.920	0.954
AUC.diff	0.008	0.008
pROC	1.742	1.837
OR.p10	0.350	0.167
CBI	0.989	0.988
perc.stable	43.315	43.315
range.size	0.380	0.380

S.2.22 *Aubrieta columnae* MARS

Species: *Aubrieta columnae* Guss. subsp. columnae

Procedure: MARS

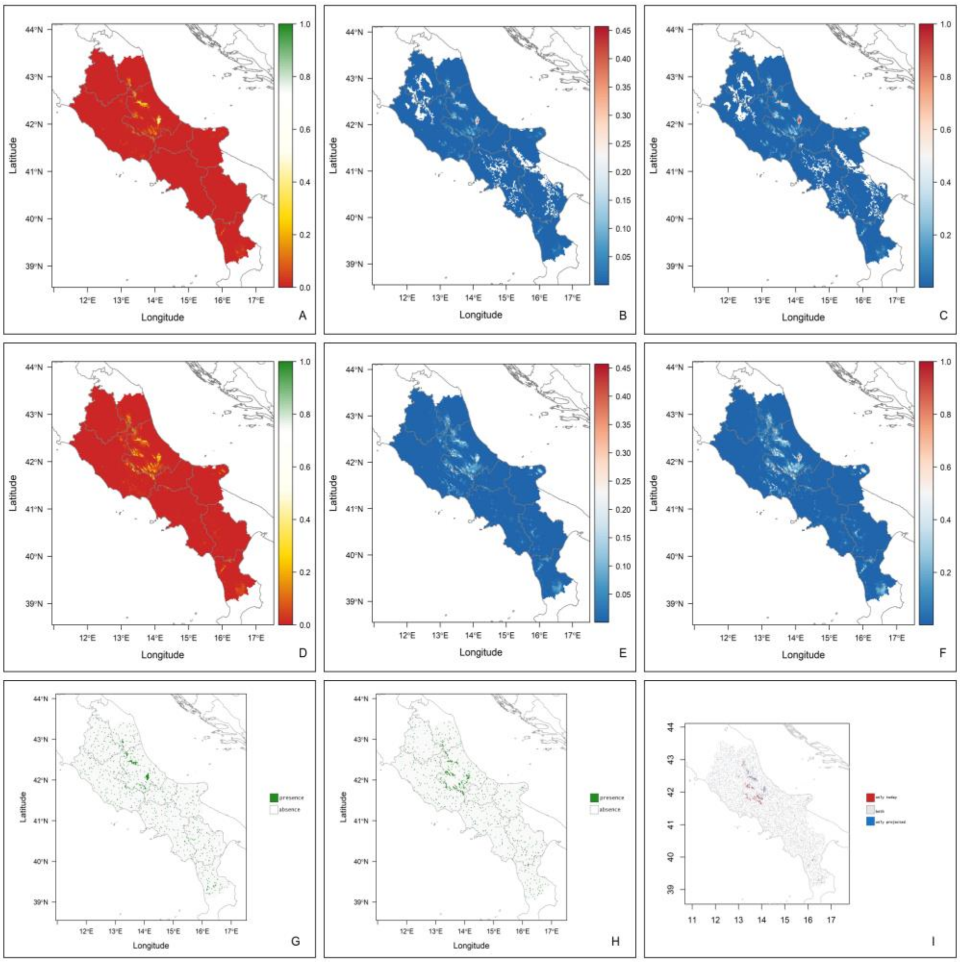

Fig. S.2.22-A) ‘Projected’ base output; B) ‘Projected’ uncertainty standard deviation; C) ‘Projected’ uncertainty range of values; D) ’Present’ base output; E) ’Present’ uncertainty standard deviation; F) ’Present’ uncertainty range of values; G) ‘Projected’ binary distribution; H) ‘Present’ binary distribution; I) Range size variation.

Value	Present	Projected
AUC.train	0.920	0.952
AUC.test	0.746	0.811
AUC.diff	0.144	0.144
pROC	1.348	1.461
OR.p10	0.350	0.333
CBI	0.839	0.734
perc.stable	6.433	6.433
range.size	0.381	0.381

S.2.23 *Aubrieta columnae* RF

Species: *Aubrieta columnae* Guss. subsp. columnae

Procedure: RF

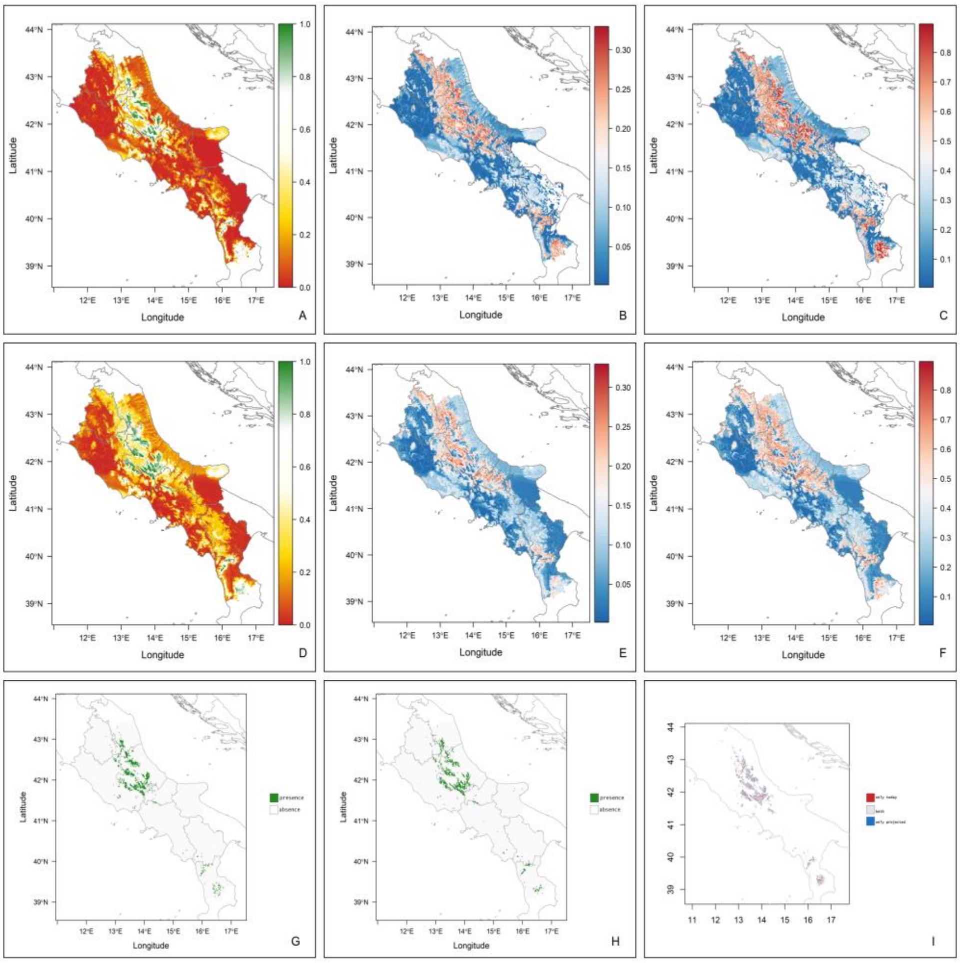

Fig. S.2.23-A) 'Projected' base output; B) 'Projected' uncertainty standard deviation; C) 'Projected' uncertainty range of values; D) 'Present' base output; E) 'Present' uncertainty standard deviation; F) 'Present' uncertainty range of values; G) 'Projected' binary distribution; H) 'Present' binary distribution; I) Range size variation.

Value	Present	Projected
AUC.train	1.000	1.000
AUC.test	0.973	0.932
AUC.diff	0.068	0.068
pROC	1.722	1.661
OR.p10	0.283	0.300
CBI	0.897	0.823
perc.stable	41.818	41.818
range.size	1.691	1.691

S.2.24 *Aubrieta columnae* SVM

Species: *Aubrieta columnae* Guss. subsp. columnae

Procedure: SVM

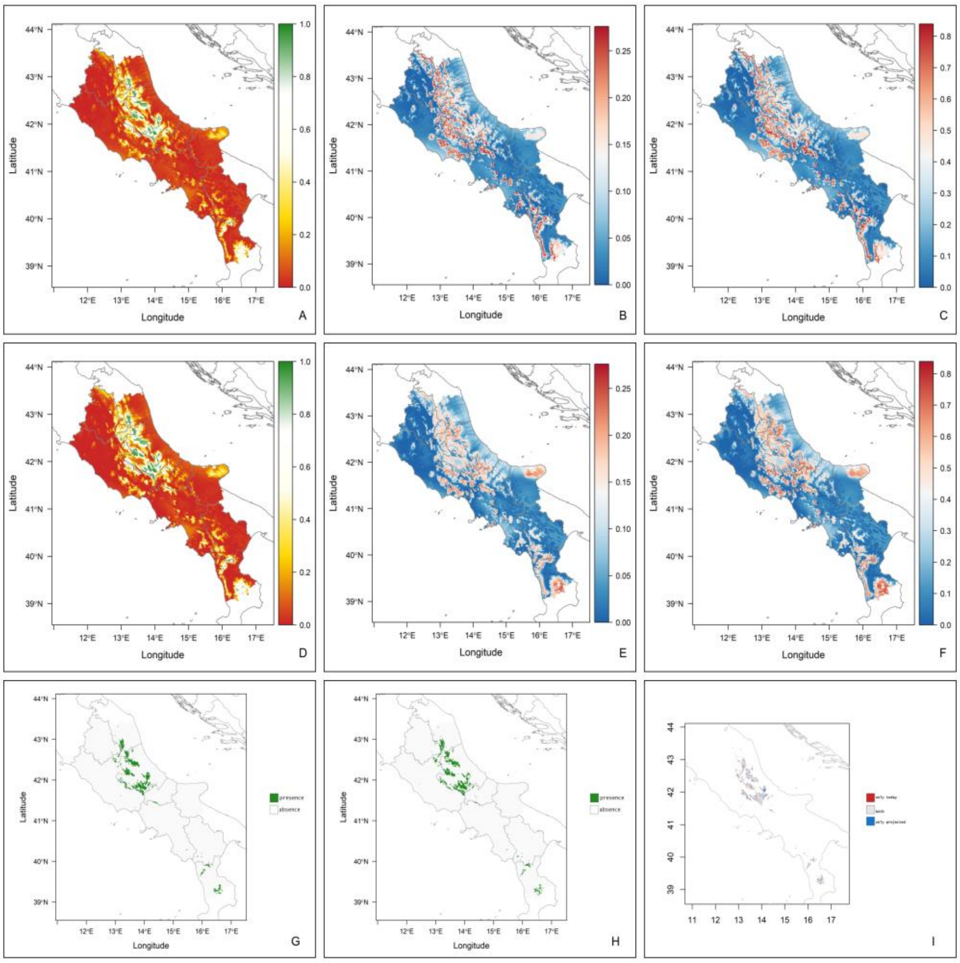

Fig. S.2.24-A) ‘Projected’ base output; B) ‘Projected’ uncertainty standard deviation; C) ‘Projected’ uncertainty range of values; D) ’Present’ base output; E) ’Present’ uncertainty standard deviation; F) ’Present’ uncertainty range of values; G) ‘Projected’ binary distribution; H) ‘Present’ binary distribution; I) Range size variation.

Value	Present	Projected
AUC.train	0.950	0.929
AUC.test	0.935	0.939
AUC.diff	0.016	0.016
pROC	1.711	1.790
OR.p10	0.300	0.133
CBI	0.903	0.957
perc.stable	61.412	61.412
range.size	-0.694	-0.694

S.2.25 *Bellevalia webbiana* Biomod2

Species: *Bellevalia webbiana* Parl.

Procedure: Biomod2

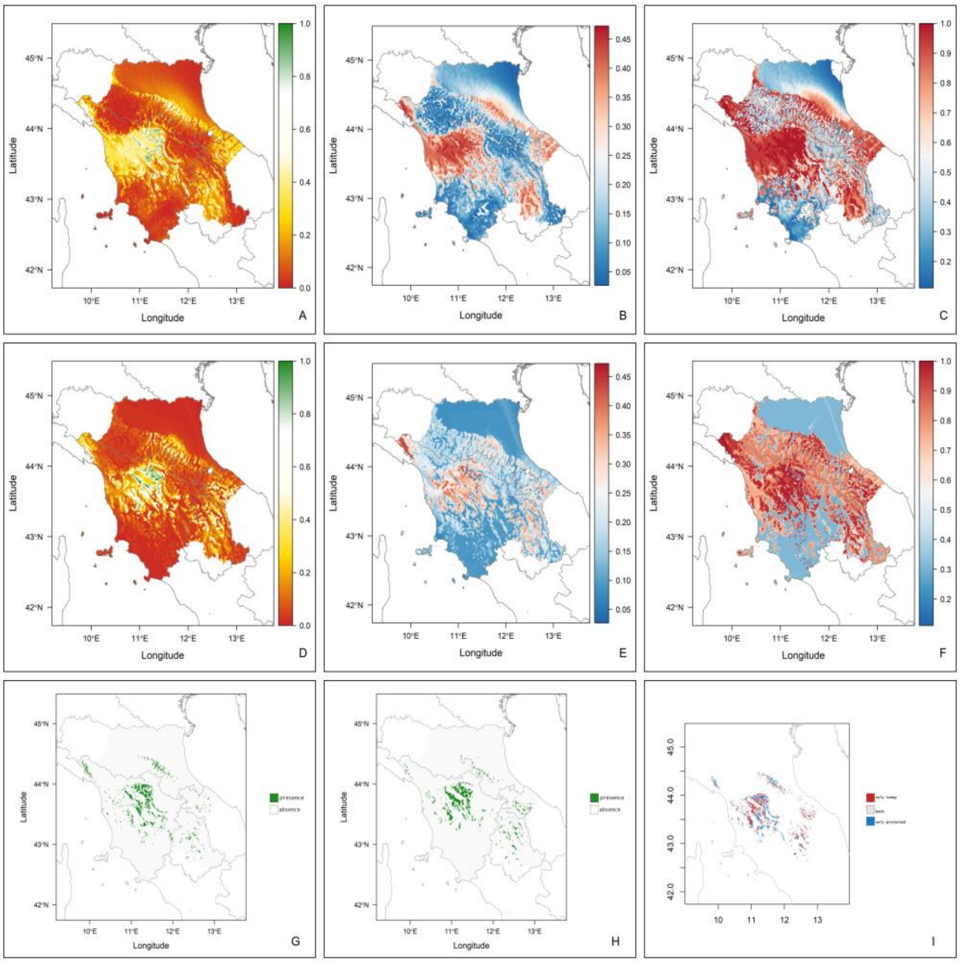

Fig. S.2.25-A) 'Projected' base output; B) 'Projected' uncertainty standard deviation; C) 'Projected' uncertainty range of values; D) 'Present' base output; E) 'Present' uncertainty standard deviation; F) 'Present' uncertainty range of values; G) 'Projected' binary distribution; H) 'Present' binary distribution; I) Range size variation.

Value	Present	Projected
AUC.train	0.994	0.999
AUC.test	0.908	0.936
AUC.diff	0.063	0.063
pROC	1.731	1.782
OR.p10	0.800	0.700
CBI	0.927	0.809
perc.stable	14.460	14.460
range.size	-2.568	-2.568

S.2.26 *Bellevalia webbiana* Bioclim

Species: *Bellevalia webbiana* Parl.

Procedure: Bioclim

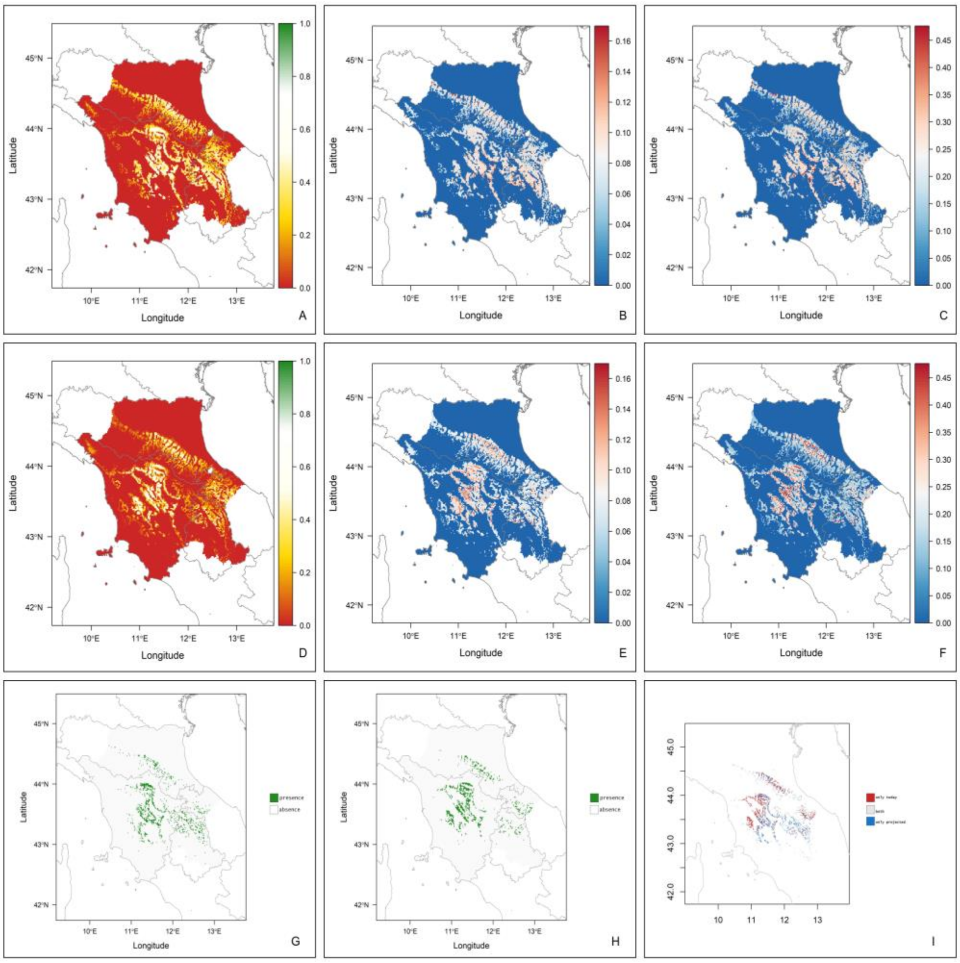

Fig. S.2.26-A) 'Projected' base output; B) 'Projected' uncertainty standard deviation; C) 'Projected' uncertainty range of values; D) 'Present' base output; E) 'Present' uncertainty standard deviation; F) 'Present' uncertainty range of values; G) 'Projected' binary distribution; H) 'Present' binary distribution; I) Range size variation.

Value	Present	Projected
AUC.train	0.922	0.866
AUC.test	0.810	0.736
AUC.diff	0.136	0.136
pROC	1.398	1.307
OR.p10	0.350	0.250
CBI	0.476	-0.033
perc.stable	22.762	22.762
range.size	0.569	0.569

S.2.27 *Bellevalia webbiana* Domain

Species: *Bellevalia webbiana* Parl.

Procedure: Domain

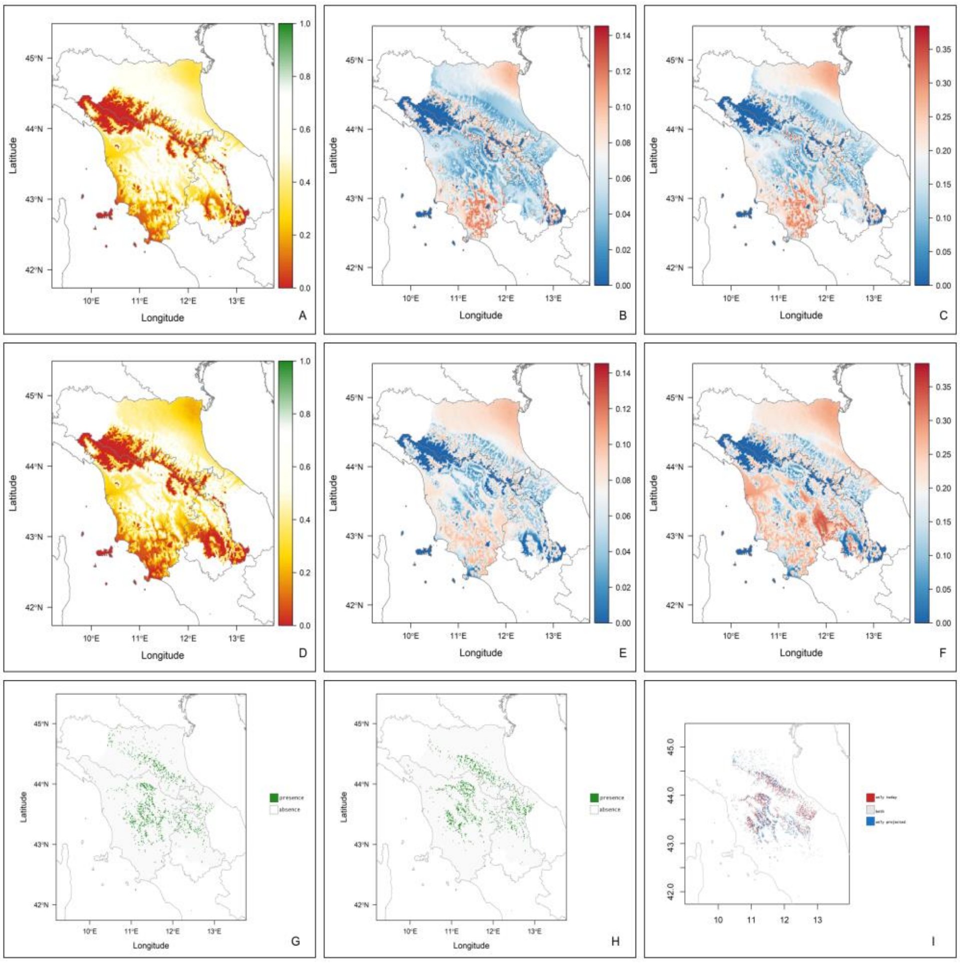

Fig. S.2.27-A) 'Projected' base output; B) 'Projected' uncertainty standard deviation; C) 'Projected' uncertainty range of values; D) 'Present' base output; E) 'Present' uncertainty standard deviation; F) 'Present' uncertainty range of values; G) 'Projected' binary distribution; H) 'Present' binary distribution; I) Range size variation.

Value	Present	Projected
AUC.train	0.893	0.826
AUC.test	0.850	0.748
AUC.diff	0.088	0.088
pROC	1.610	1.416
OR.p10	0.267	0.233
CBI	0.865	0.609
perc.stable	9.665	9.665
range.size	-0.353	-0.353

S.2.28 *Bellevalia webbiana* Maxent

Species: *Bellevalia webbiana* Parl.

Procedure: Maxent

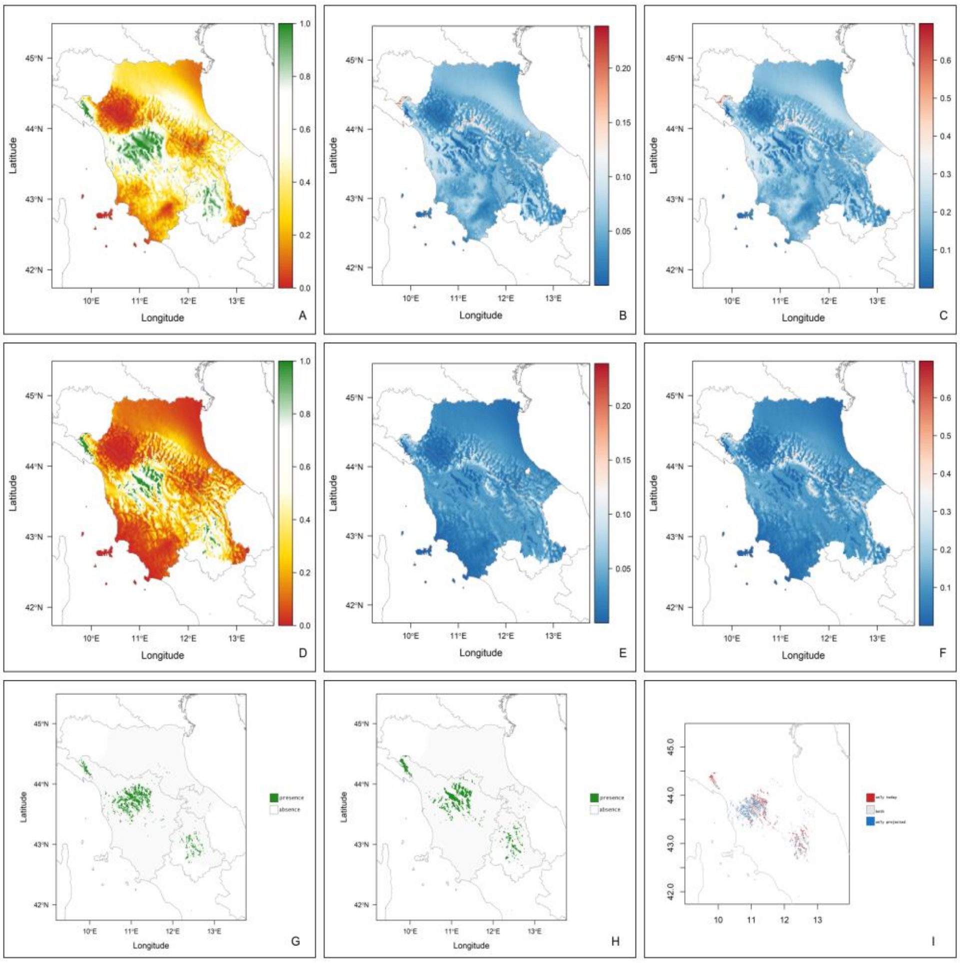

Fig. S.2.28-A) 'Projected' base output; B) 'Projected' uncertainty standard deviation; C) 'Projected' uncertainty range of values; D) 'Present' base output; E) 'Present' uncertainty standard deviation; F) 'Present' uncertainty range of values; G) 'Projected' binary distribution; H) 'Present' binary distribution; I) Range size variation.

Value	Present	Projected
AUC.train	0.910	0.881
AUC.test	0.911	0.863
AUC.diff	0.029	0.029
pROC	1.728	1.658
OR.p10	0.183	0.150
CBI	0.882	0.865
perc.stable	40.202	40.202
range.size	0.637	0.637

S.2.29 *Bellevalia webbiana* KDE

Species: *Bellevalia webbiana* Parl.

Procedure: KDE

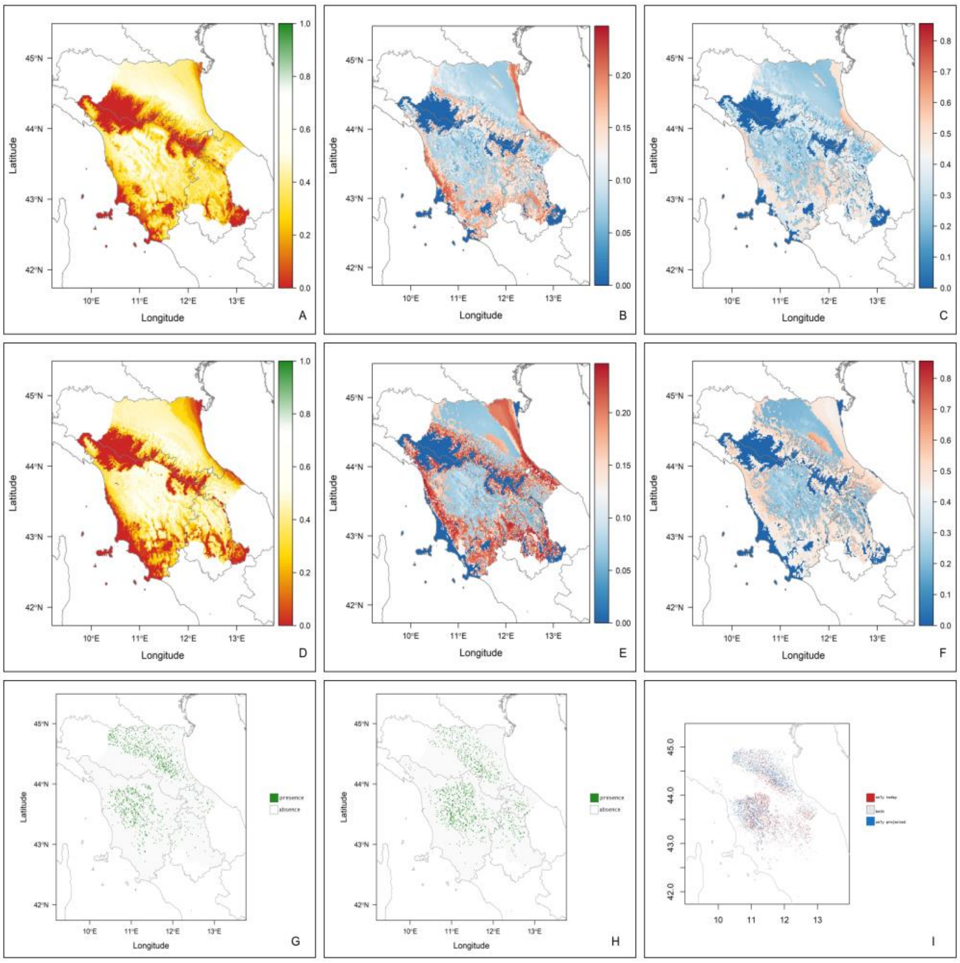

Fig. S.2.29-A) 'Projected' base output; B) 'Projected' uncertainty standard deviation; C) 'Projected' uncertainty range of values; D) 'Present' base output; E) 'Present' uncertainty standard deviation; F) 'Present' uncertainty range of values; G) 'Projected' binary distribution; H) 'Present' binary distribution; I) Range size variation.

Value	Present	Projected
AUC.train	0.858	0.775
AUC.test	0.818	0.757
AUC.diff	0.049	0.059
pROC	1.567	1.427
OR.p10	0.267	0.250
CBI	0.902	0.390
perc.stable	4.374	4.374
range.size	-2.411	-2.411

S.2.30 *Bellevalia webbiana* CTA

Species: *Bellevalia webbiana* Parl.

Procedure: CTA

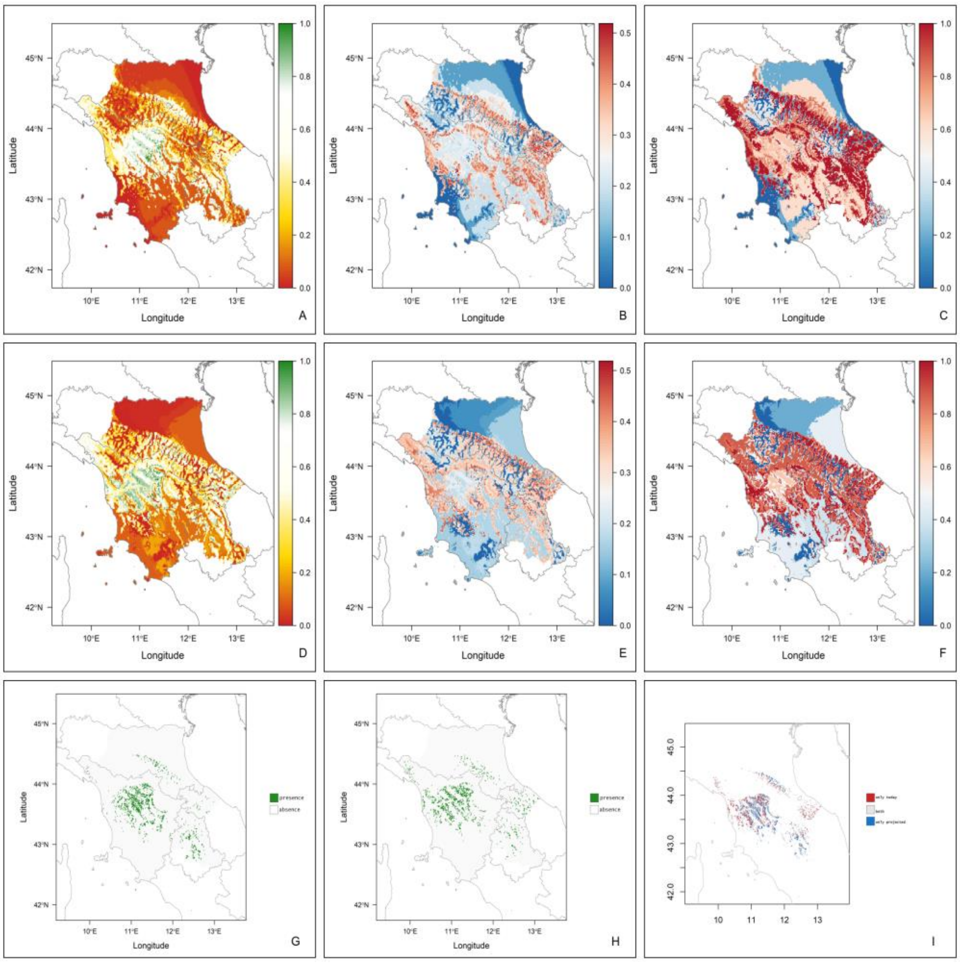

Fig. S.2.30-A) 'Projected' base output; B) 'Projected' uncertainty standard deviation; C) 'Projected' uncertainty range of values; D) 'Present' base output; E) 'Present' uncertainty standard deviation; F) 'Present' uncertainty range of values; G) 'Projected' binary distribution; H) 'Present' binary distribution; I) Range size variation.

Value	Present	Projected
AUC.train	0.935	0.948
AUC.test	0.880	0.874
AUC.diff	0.074	0.074
pROC	1.294	1.385
OR.p10	0.167	0.250
CBI	0.929	0.615
perc.stable	13.596	13.596
range.size	-0.687	-0.687

S.2.31 *Bellevalia webbiana* FDA

Species: *Bellevalia webbiana* Parl.

Procedure: FDA

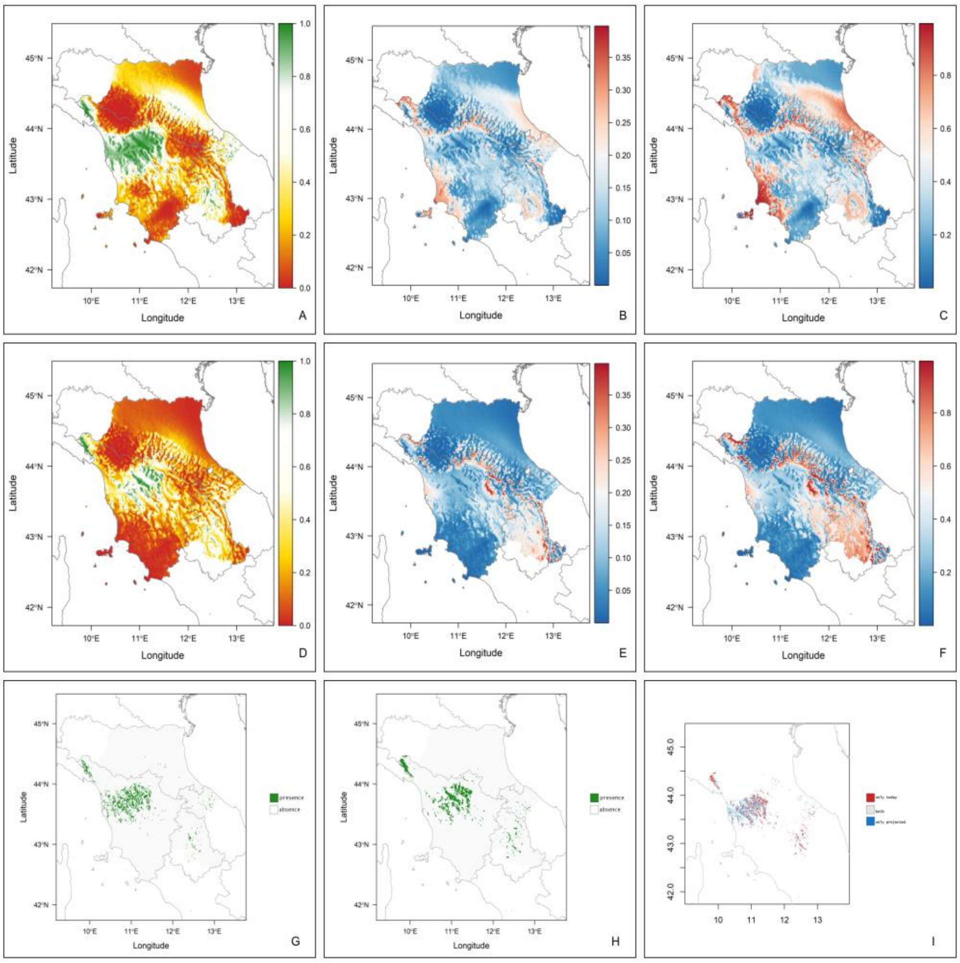

Fig. S.2.31-A) 'Projected' base output; B) 'Projected' uncertainty standard deviation; C) 'Projected' uncertainty range of values; D) 'Present' base output; E) 'Present' uncertainty standard deviation; F) 'Present' uncertainty range of values; G) 'Projected' binary distribution; H) 'Present' binary distribution; I) Range size variation.

Value	Present	Projected
AUC.train	0.904	0.902
AUC.test	0.907	0.897
AUC.diff	0.029	0.029
pROC	1.723	1.704
OR.p10	0.267	0.233
CBI	0.947	0.785
perc.stable	30.891	30.891
range.size	-4.940	-4.940

S.2.32 *Bellevalia webbiana* GAM

Species: *Bellevalia webbiana* Parl.

Procedure: GAM

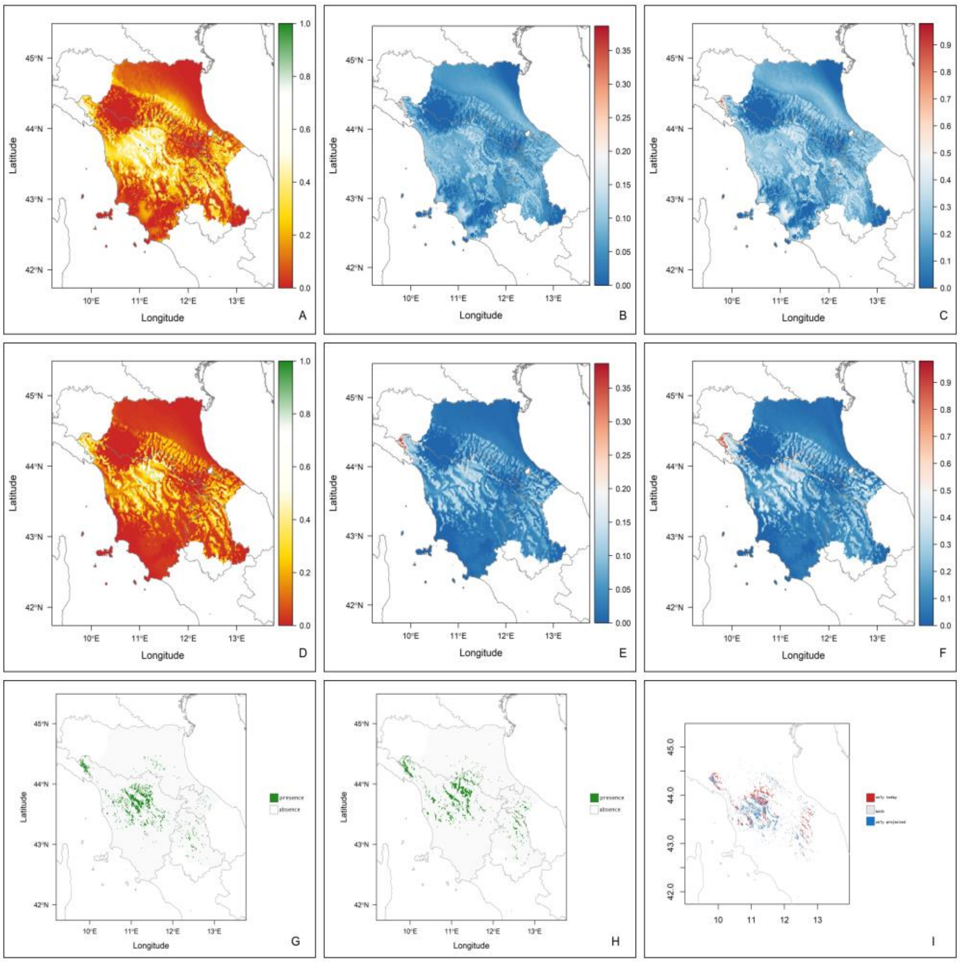

Fig. S.2.32-A) ‘Projected’ base output; B) ‘Projected’ uncertainty standard deviation; C) ‘Projected’ uncertainty range of values; D) ’Present’ base output; E) ’Present’ uncertainty standard deviation; F) ’Present’ uncertainty range of values; G) ‘Projected’ binary distribution; H) ‘Present’ binary distribution; I) Range size variation.

Value	Present	Projected
AUC.train	0.915	0.933
AUC.test	0.897	0.884
AUC.diff	0.052	0.052
pROC	1.714	1.701
OR.p10	0.300	0.333
CBI	0.862	0.897
perc.stable	18.685	18.685
range.size	3.213	3.213

S.2.33 *Bellevalia webbiana* GLM

Species: *Bellevalia webbiana* Parl.

Procedure: GLM

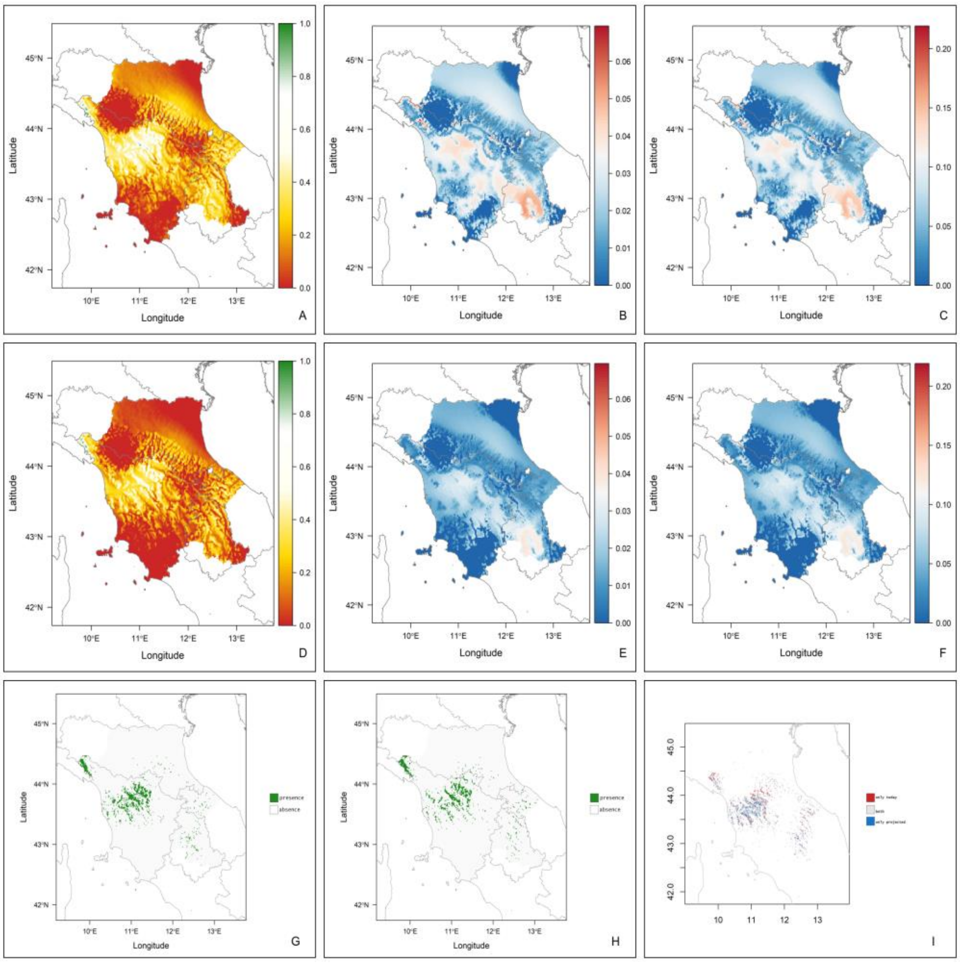

Fig. S.2.33-A) ‘Projected’ base output; B) ‘Projected’ uncertainty standard deviation; C) ‘Projected’ uncertainty range of values; D) ’Present’ base output; E) ’Present’ uncertainty standard deviation; F) ’Present’ uncertainty range of values; G) ‘Projected’ binary distribution; H) ‘Present’ binary distribution; I) Range size variation.

Value	Present	Projected
AUC.train	0.909	0.870
AUC.test	0.893	0.869
AUC.diff	0.018	0.018
pROC	1.712	1.648
OR.p10	0.250	0.183
CBI	0.897	0.894
perc.stable	35.832	35.832
range.size	3.254	3.254

S.2.34 *Bellevalia webbiana* MARS

Species: *Bellevalia webbiana* Parl.

Procedure: MARS

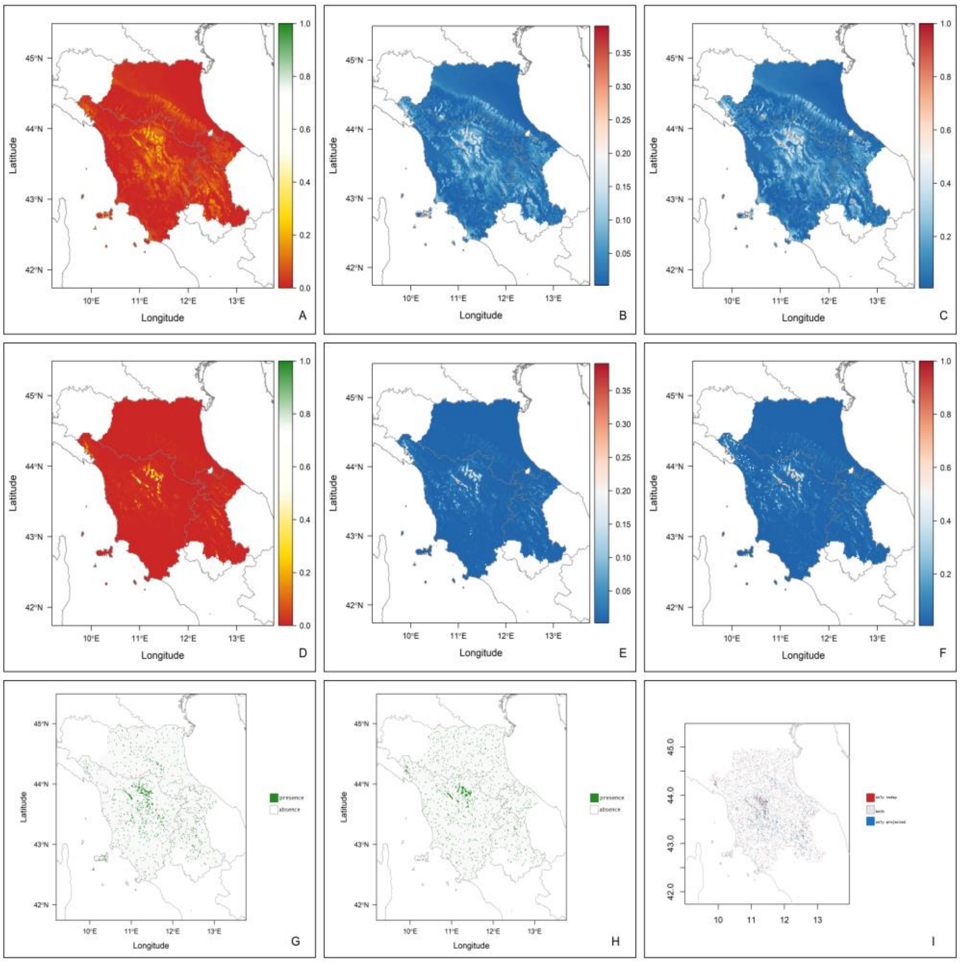

Fig. S.2.34-A) 'Projected' base output; B) 'Projected' uncertainty standard deviation; C) 'Projected' uncertainty range of values; D) 'Present' base output; E) 'Present' uncertainty standard deviation; F) 'Present' uncertainty range of values; G) 'Projected' binary distribution; H) 'Present' binary distribution; I) Range size variation.

Value	Present	Projected
AUC.train	0.837	0.922
AUC.test	0.743	0.833
AUC.diff	0.089	0.089
pROC	1.386	1.575
OR.p10	0.133	0.483
CBI	0.786	0.848
perc.stable	5.830	5.830
range.size	1.692	1.692

S.2.35 *Bellevalia webbiana* RF

Species: *Bellevalia webbiana* Parl.

Procedure: RF

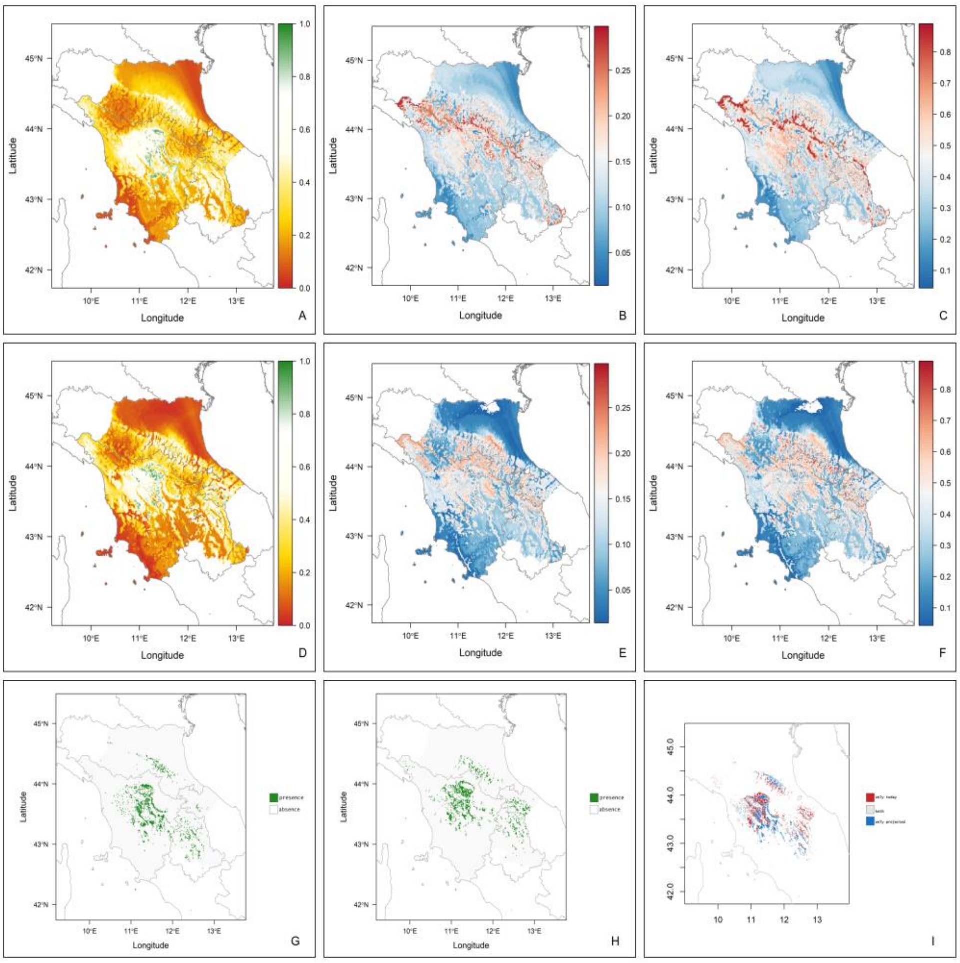

Fig. S.2.35-A) 'Projected' base output; B) 'Projected' uncertainty standard deviation; C) 'Projected' uncertainty range of values; D) 'Present' base output; E) 'Present' uncertainty standard deviation; F) 'Present' uncertainty range of values; G) 'Projected' binary distribution; H) 'Present' binary distribution; I) Range size variation.

Value	Present	Projected
AUC.train	1.000	1.000
AUC.test	0.978	0.983
AUC.diff	0.017	0.017
pROC	1.678	1.634
OR.p10	0.567	0.617
CBI	0.966	0.784
perc.stable	5.908	5.908
range.size	-2.207	-2.207

S.2.36 *Bellevalia webbiana* SVM

Species: *Bellevalia webbiana* Parl.

Procedure: SVM

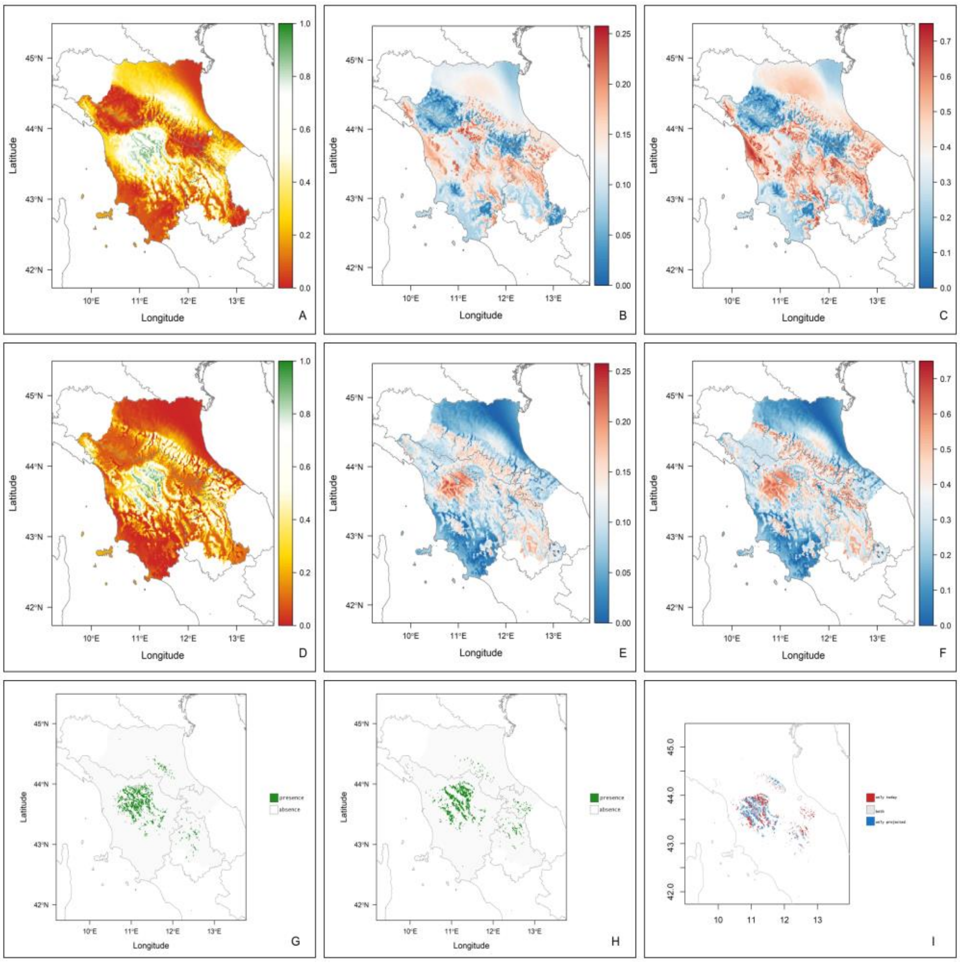

Fig. S.2.36-A) 'Projected' base output; B) 'Projected' uncertainty standard deviation; C) 'Projected' uncertainty range of values; D) 'Present' base output; E) 'Present' uncertainty standard deviation; F) 'Present' uncertainty range of values; G) 'Projected' binary distribution; H) 'Present' binary distribution; I) Range size variation.

Value	Present	Projected
AUC.train	0.962	0.936
AUC.test	0.929	0.926
AUC.diff	0.018	0.018
pROC	1.691	1.747
OR.p10	0.283	0.200
CBI	0.931	0.901
perc.stable	19.617	19.617
range.size	1.732	1.732

S.2.37 *Cardamine apennina* Biomod2

Species: *Cardamine apennina* LihovÃ¡ & Marhold

Procedure: Biomod2

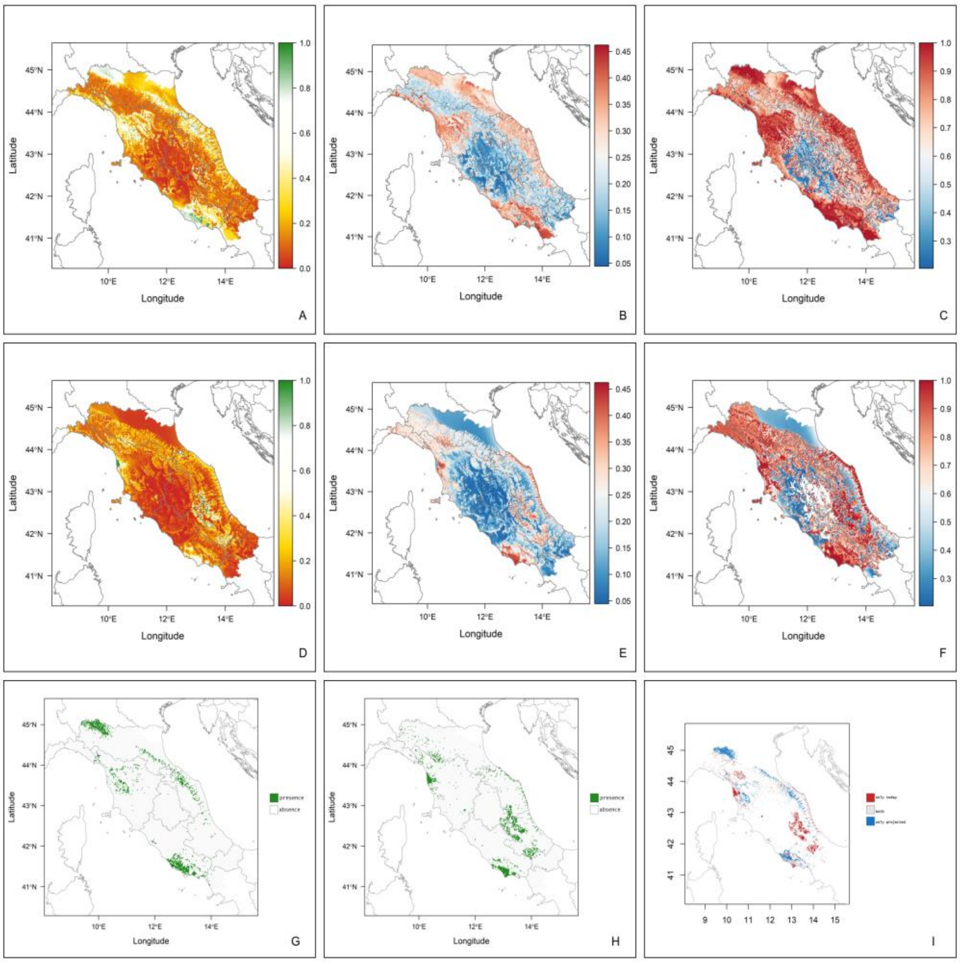

Fig. S.2.37-A) 'Projected' base output; B) 'Projected' uncertainty standard deviation; C) 'Projected' uncertainty range of values; D) 'Present' base output; E) 'Present' uncertainty standard deviation; F) 'Present' uncertainty range of values; G) 'Projected' binary distribution; H) 'Present' binary distribution; I) Range size variation.

Value	Present	Projected
AUC.train	0.998	0.995
AUC.test	0.820	0.901
AUC.diff	0.094	0.094
pROC	1.435	1.598
OR.p10	0.713	0.588
CBI	0.920	0.467
perc.stable	7.459	7.459
range.size	1.328	1.328

S.2.38 *Cardamine apennina* Bioclim

Species: *Cardamine apennina* LihovÃ¡ & Marhold

Procedure: Bioclim

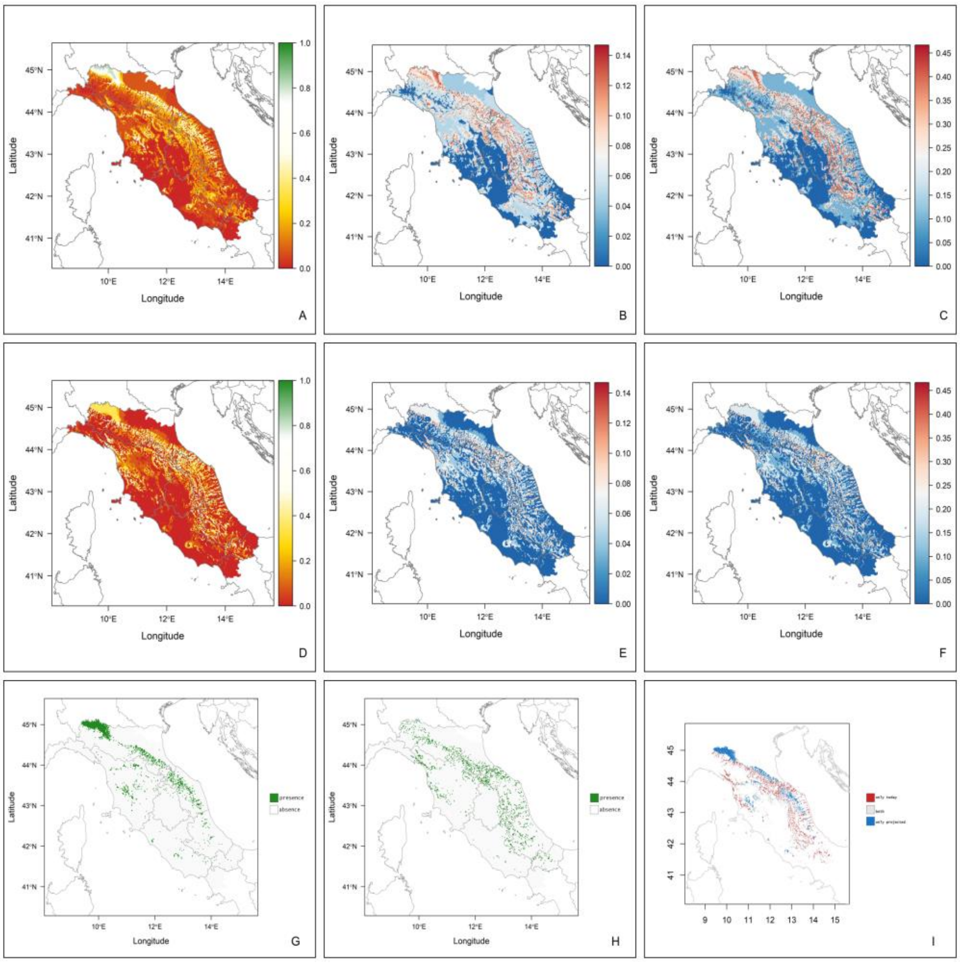

Fig. S.2.38-A) 'Projected' base output; B) 'Projected' uncertainty standard deviation; C) 'Projected' uncertainty range of values; D) 'Present' base output; E) 'Present' uncertainty standard deviation; F) 'Present' uncertainty range of values; G) 'Projected' binary distribution; H) 'Present' binary distribution; I) Range size variation.

Value	Present	Projected
AUC.train	0.811	0.773
AUC.test	0.755	0.642
AUC.diff	0.142	0.142
pROC	1.309	1.117
OR.p10	0.188	0.238
CBI	0.541	0.200
perc.stable	6.991	6.991
range.size	1.396	1.396

S.2.39 *Cardamine apennina* Domain

Species: *Cardamine apennina* LihovÃ¡ & Marhold

Procedure: Domain

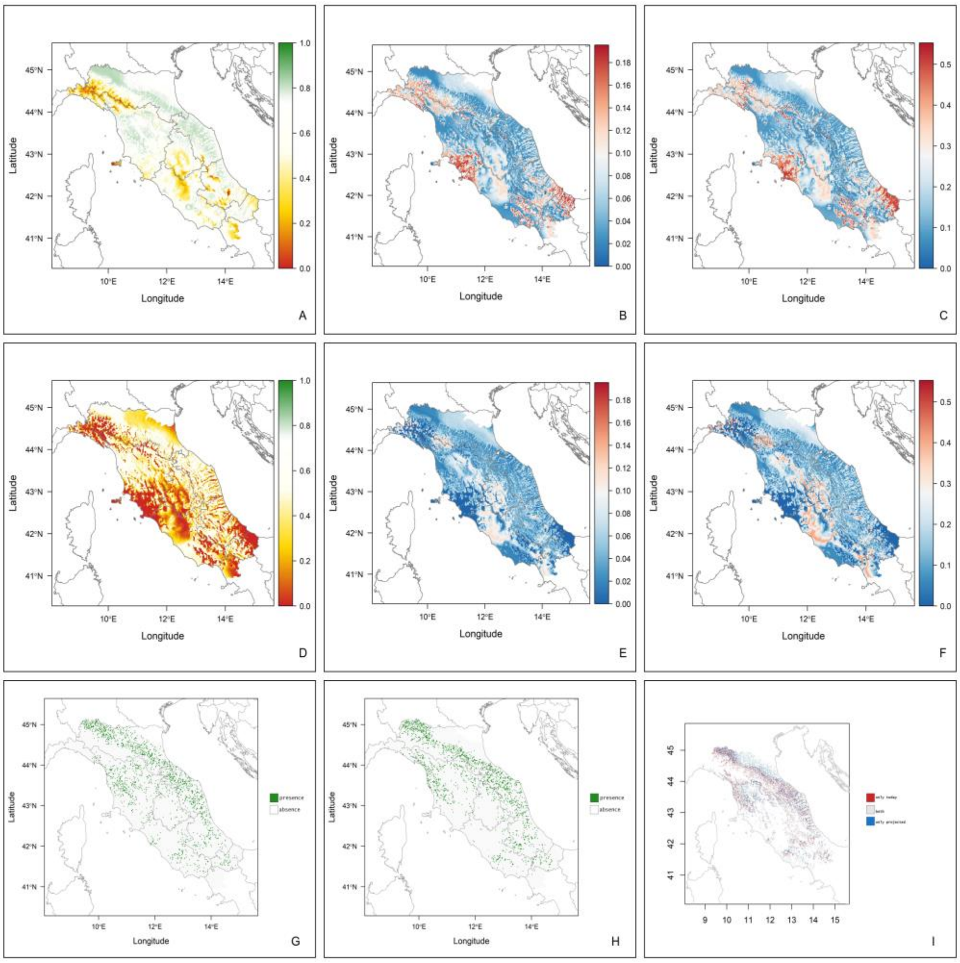

Fig. S.2.39-A) 'Projected' base output; B) 'Projected' uncertainty standard deviation; C) 'Projected' uncertainty range of values; D) 'Present' base output; E) 'Present' uncertainty standard deviation; F) 'Present' uncertainty range of values; G) 'Projected' binary distribution; H) 'Present' binary distribution; I) Range size variation.

Value	Present	Projected
AUC.train	0.752	0.731
AUC.test	0.704	0.803
AUC.diff	0.010	0.010
pROC	1.408	1.468
OR.p10	0.188	0.088
CBI	0.581	-0.074
perc.stable	9.423	9.423
range.size	0.128	0.128

S.2.40 *Cardamine apennina* Maxent

Species: *Cardamine apennina* LihovÃ¡ & Marhold

Procedure: Maxent

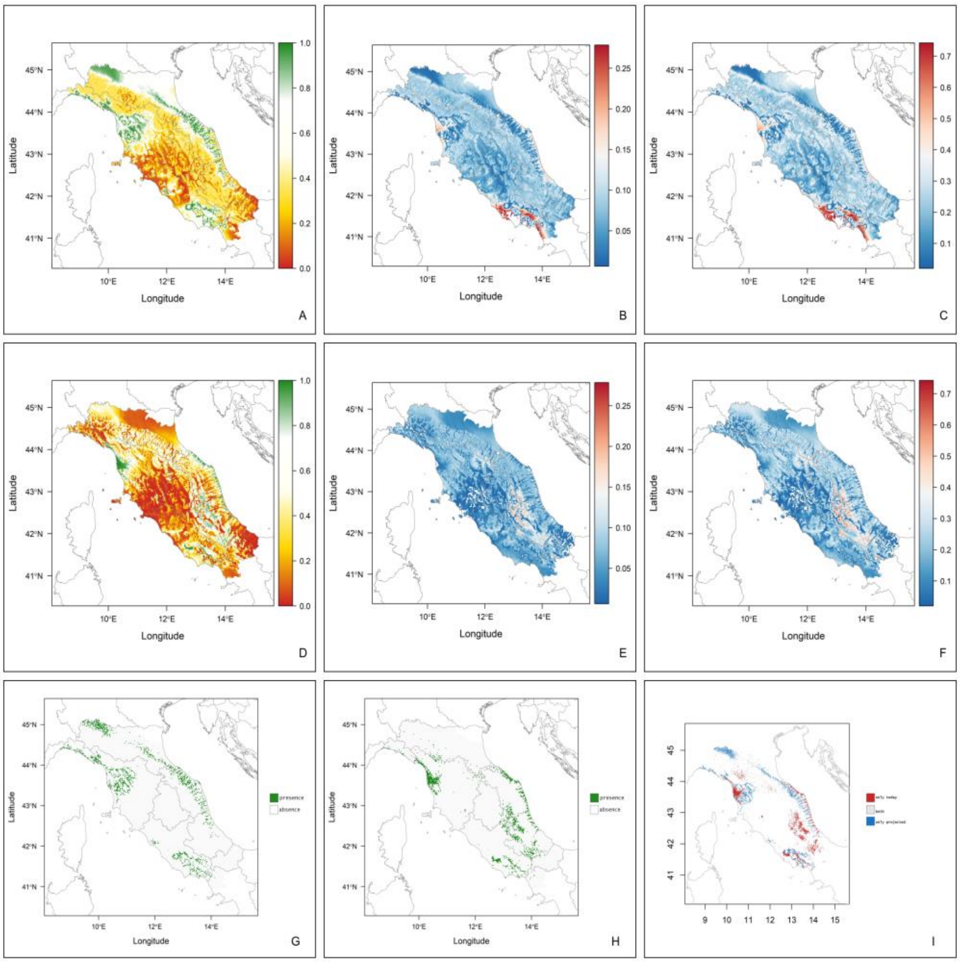

Fig. S.2.40-A) ‘Projected’ base output; B) ‘Projected’ uncertainty standard deviation; C) ‘Projected’ uncertainty range of values; D) ’Present’ base output; E) ’Present’ uncertainty standard deviation; F) ’Present’ uncertainty range of values; G) ‘Projected’ binary distribution; H) ‘Present’ binary distribution; I) Range size variation.

Value	Present	Projected
AUC.train	0.887	0.832
AUC.test	0.873	0.788
AUC.diff	0.069	0.069
pROC	1.589	1.404
OR.p10	0.162	0.188
CBI	0.915	0.265
perc.stable	6.812	6.812
range.size	2.954	2.954

S.2.41 *Cardamine apennina* KDE

Species: *Cardamine apennina* LihovÃ¡ & Marhold

Procedure: KDE

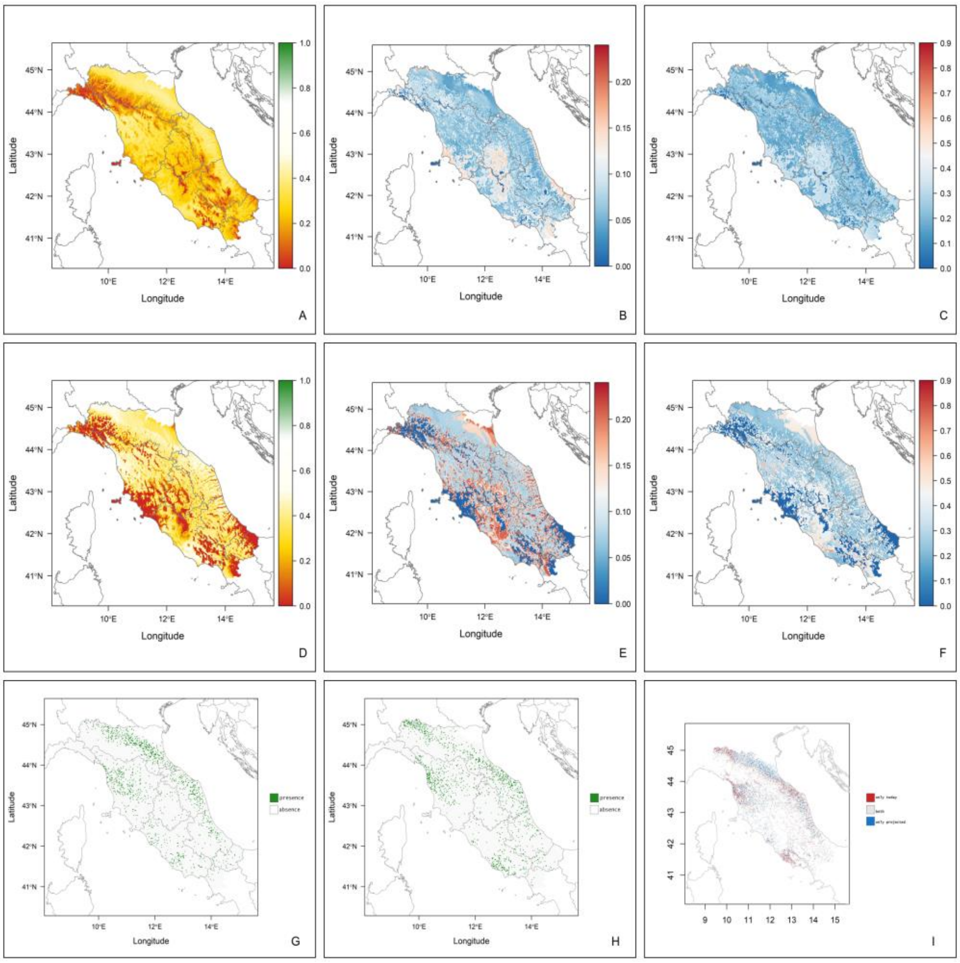

Fig. S.2.41-A) ‘Projected’ base output; B) ‘Projected’ uncertainty standard deviation; C) ‘Projected’ uncertainty range of values; D) ’Present’ base output; E) ’Present’ uncertainty standard deviation; F) ’Present’ uncertainty range of values; G) ‘Projected’ binary distribution; H) ‘Present’ binary distribution; I) Range size variation.

Value	Present	Projected
AUC.train	0.792	0.748
AUC.test	0.720	0.739
AUC.diff	0.088	0.040
pROC	1.368	1.316
OR.p10	0.250	0.088
CBI	0.835	0.131
perc.stable	5.287	5.287
range.size	-2.240	-2.240

S.2.42 *Cardamine apennina* CTA

Species: *Cardamine apennina* LihovÃ¡ & Marhold

Procedure: CTA

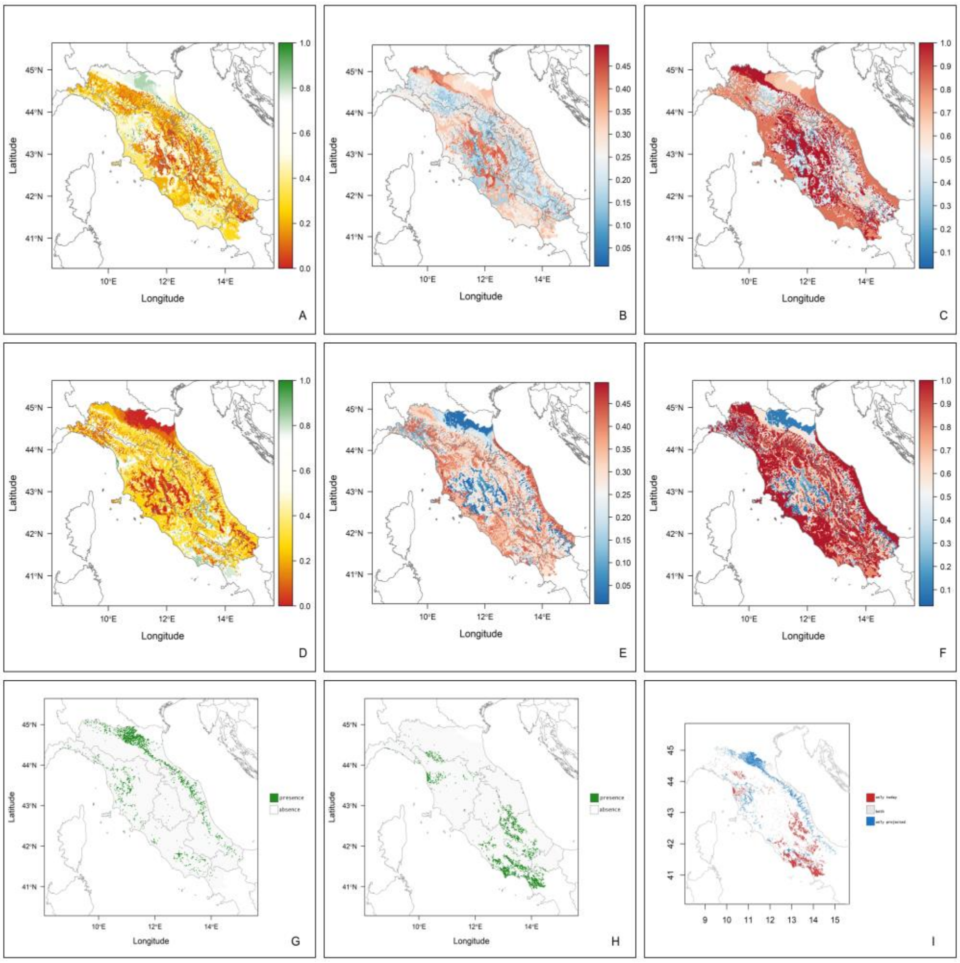

Fig. S.2.42-A) 'Projected' base output; B) 'Projected' uncertainty standard deviation; C) 'Projected' uncertainty range of values; D) 'Present' base output; E) 'Present' uncertainty standard deviation; F) 'Present' uncertainty range of values; G) 'Projected' binary distribution; H) 'Present' binary distribution; I) Range size variation.

Value	Present	Projected
AUC.train	0.939	0.907
AUC.test	0.845	0.784
AUC.diff	0.125	0.125
pROC	1.258	1.164
OR.p10	0.238	0.200
CBI	0.898	0.419
perc.stable	0.030	0.030
range.size	0.791	0.791

S.2.43 *Cardamine apennina* FDA

Species: *Cardamine apennina* LihovÃ¡ & Marhold

Procedure: FDA

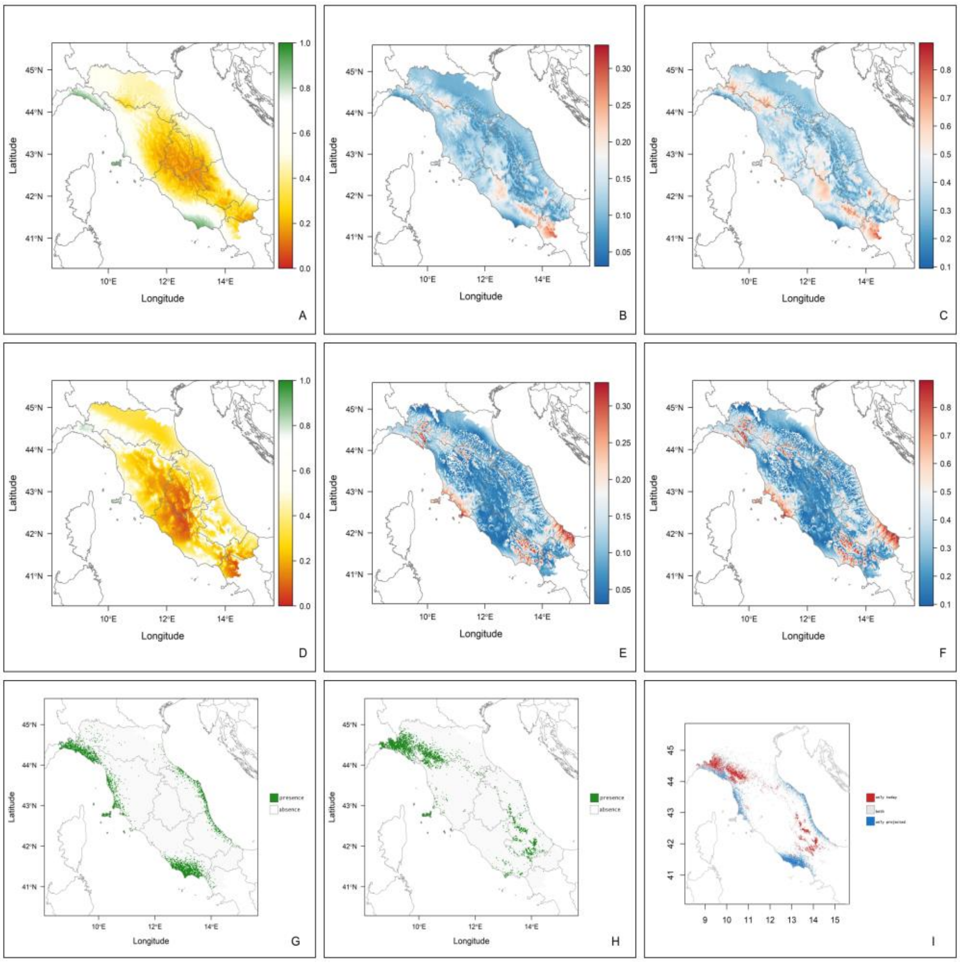

Fig. S.2.43-A) 'Projected' base output; B) 'Projected' uncertainty standard deviation; C) 'Projected' uncertainty range of values; D) 'Present' base output; E) 'Present' uncertainty standard deviation; F) 'Present' uncertainty range of values; G) 'Projected' binary distribution; H) 'Present' binary distribution; I) Range size variation.

Value	Present	Projected
AUC.train	0.698	0.729
AUC.test	0.640	0.654
AUC.diff	0.086	0.086
pROC	1.258	1.218
OR.p10	0.238	0.238
CBI	0.487	0.212
perc.stable	14.278	14.278
range.size	0.707	0.707

S.2.44 *Cardamine apennina* GAM

Species: *Cardamine apennina* LihovÃ¡ & Marhold

Procedure: GAM

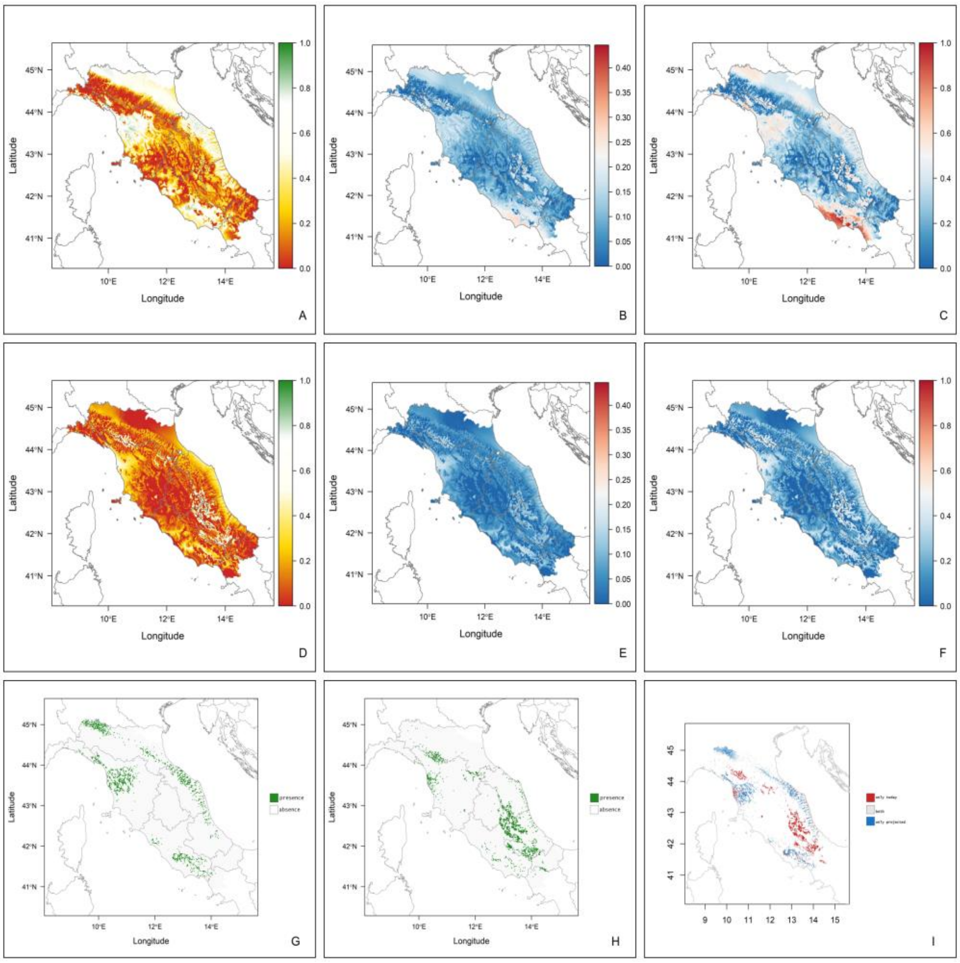

Fig. S.2.44-A) 'Projected' base output; B) 'Projected' uncertainty standard deviation; C) 'Projected' uncertainty range of values; D) 'Present' base output; E) 'Present' uncertainty standard deviation; F) 'Present' uncertainty range of values; G) 'Projected' binary distribution; H) 'Present' binary distribution; I) Range size variation.

Value	Present	Projected
AUC.train	0.909	0.849
AUC.test	0.881	0.765
AUC.diff	0.095	0.095
pROC	1.605	1.383
OR.p10	0.162	0.288
CBI	0.824	0.528
perc.stable	3.418	3.418
range.size	-2.810	-2.810

S.2.45 *Cardamine apennina* GLM

Species: *Cardamine apennina* LihovÃ¡ & Marhold

Procedure: GLM

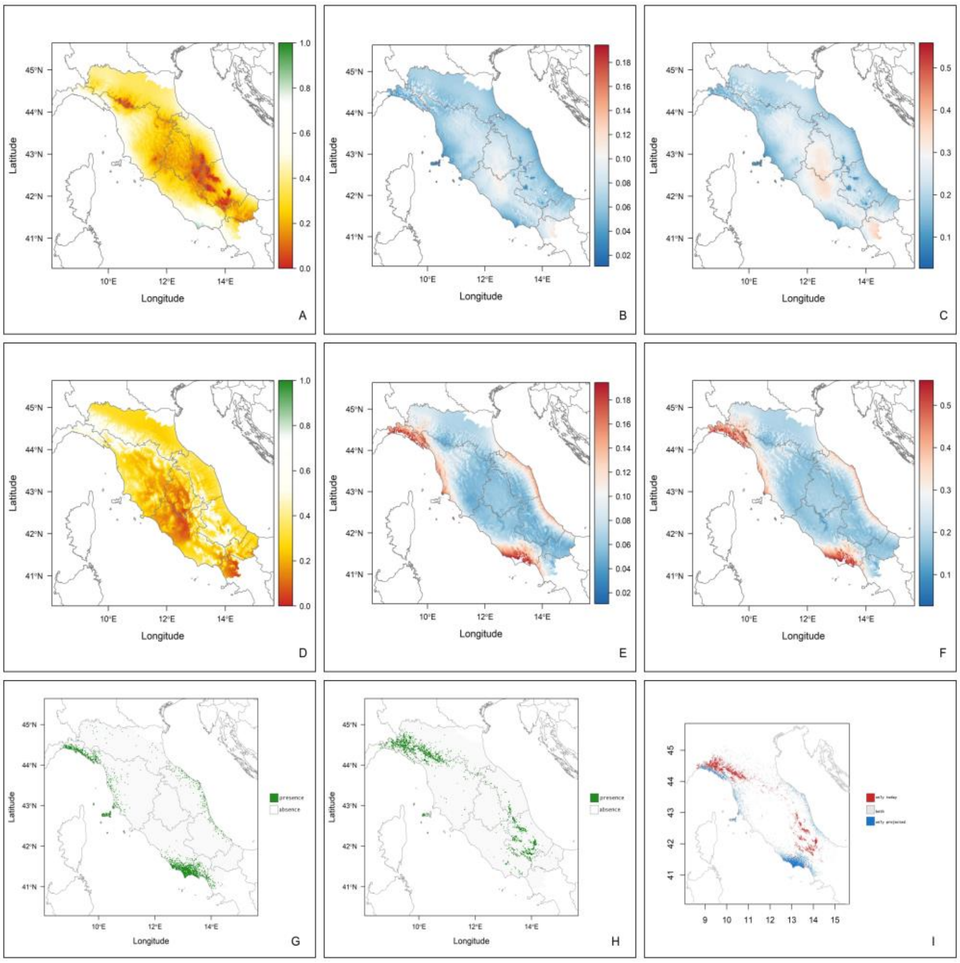

Fig. S.2.45-A) ‘Projected’ base output; B) ‘Projected’ uncertainty standard deviation; C) ‘Projected’ uncertainty range of values; D) ’Present’ base output; E) ’Present’ uncertainty standard deviation; F) ’Present’ uncertainty range of values; G) ‘Projected’ binary distribution; H) ‘Present’ binary distribution; I) Range size variation.

Value	Present	Projected
AUC.train	0.652	0.690
AUC.test	0.572	0.616
AUC.diff	0.086	0.086
pROC	1.253	1.214
OR.p10	0.162	0.200
CBI	0.718	0.160
perc.stable	3.518	3.518
range.size	-3.235	-3.235

S.2.46 *Cardamine apennina* MARS

Species: *Cardamine apennina* LihovÃ¡ & Marhold

Procedure: MARS

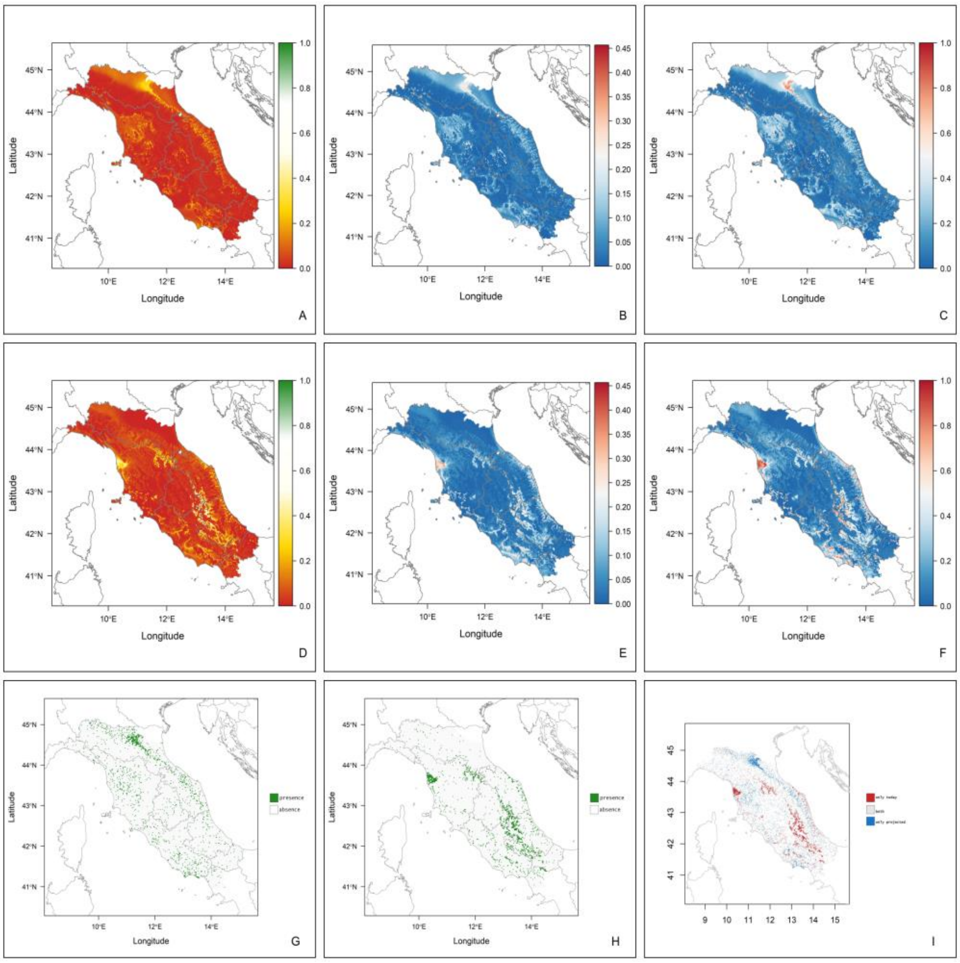

Fig. S.2.46-A) ‘Projected’ base output; B) ‘Projected’ uncertainty standard deviation; C) ‘Projected’ uncertainty range of values; D) ’Present’ base output; E) ’Present’ uncertainty standard deviation; F) ’Present’ uncertainty range of values; G) ‘Projected’ binary distribution; H) ‘Present’ binary distribution; I) Range size variation.

Value	Present	Projected
AUC.train	0.868	0.840
AUC.test	0.805	0.715
AUC.diff	0.126	0.126
pROC	1.431	1.249
OR.p10	0.188	0.200
CBI	0.823	-0.238
perc.stable	1.587	1.587
range.size	-1.361	-1.361

S.2.47 *Cardamine apennina* RF

Species: *Cardamine apennina* LihovÃ¡ & Marhold

Procedure: RF

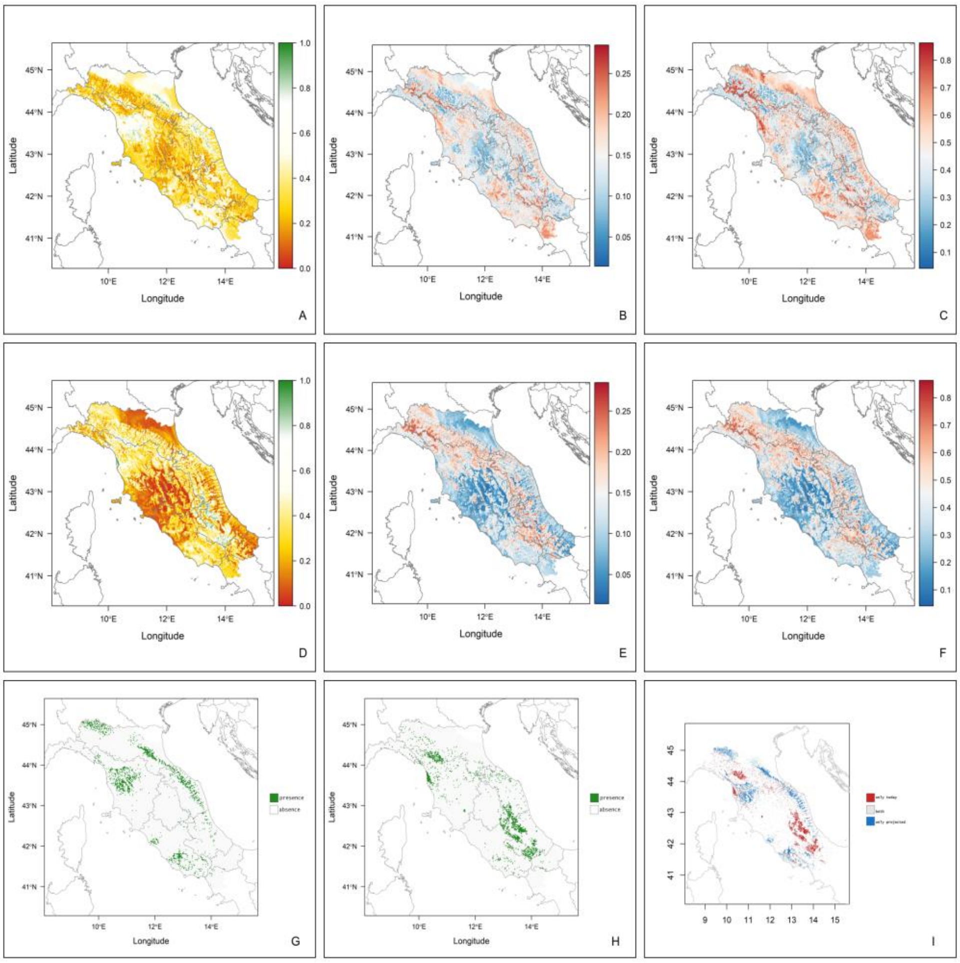

Fig. S.2.47-A) ‘Projected’ base output; B) ‘Projected’ uncertainty standard deviation; C) ‘Projected’ uncertainty range of values; D) ’Present’ base output; E) ’Present’ uncertainty standard deviation; F) ’Present’ uncertainty range of values; G) ‘Projected’ binary distribution; H) ‘Present’ binary distribution; I) Range size variation.

Value	Present	Projected
AUC.train	1.000	1.000
AUC.test	0.963	0.947
AUC.diff	0.053	0.053
pROC	1.488	1.400
OR.p10	0.475	0.475
CBI	0.943	0.643
perc.stable	4.568	4.568
range.size	-0.187	-0.187

S.2.48 *Cardamine apennina* SVM

Species: *Cardamine apennina* LihovÃ¡ & Marhold

Procedure: SVM

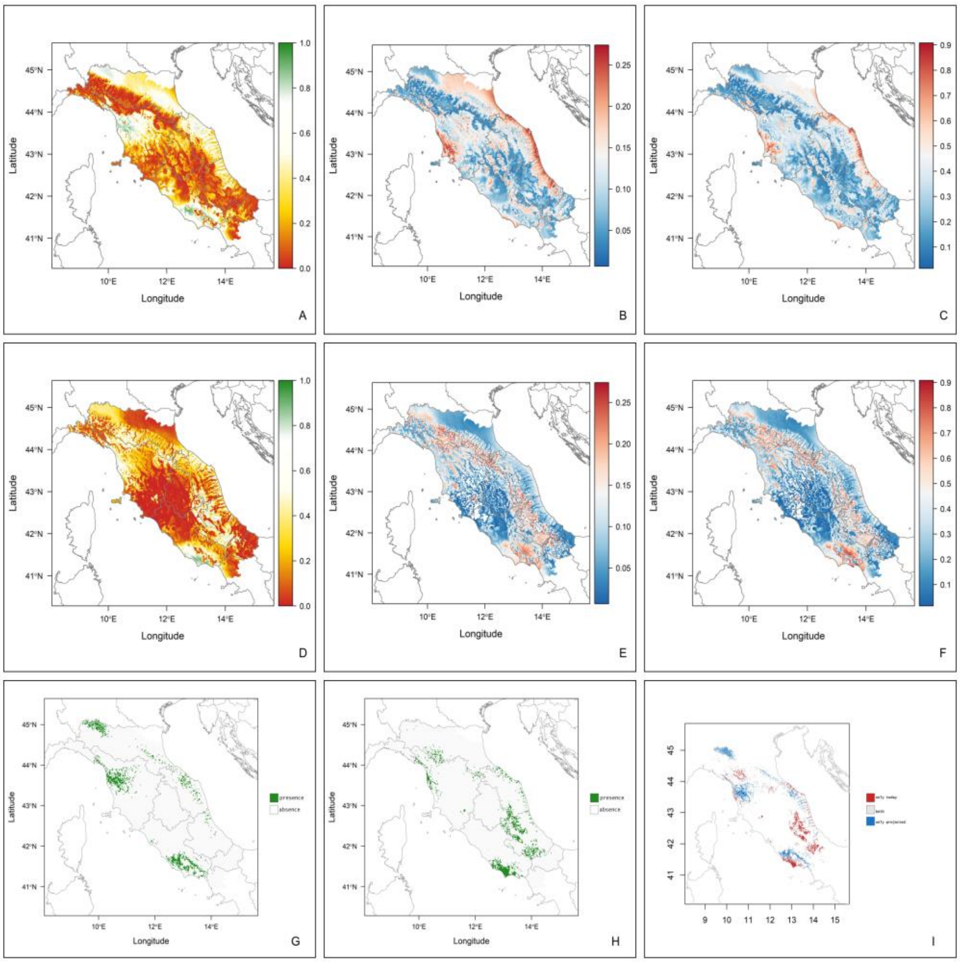

Fig. S.2.48-A) 'Projected' base output; B) 'Projected' uncertainty standard deviation; C) 'Projected' uncertainty range of values; D) 'Present' base output; E) 'Present' uncertainty standard deviation; F) 'Present' uncertainty range of values; G) 'Projected' binary distribution; H) 'Present' binary distribution; I) Range size variation.

Value	Present	Projected
AUC.train	0.910	0.871
AUC.test	0.862	0.825
AUC.diff	0.062	0.062
pROC	1.496	1.381
OR.p10	0.188	0.238
CBI	0.892	0.435
perc.stable	10.568	10.568
range.size	0.780	0.780

S.2.49 *Carduus affinis* Biomod2

Species: *Carduus affinis* Guss. subsp. affinis

Procedure: Biomod2

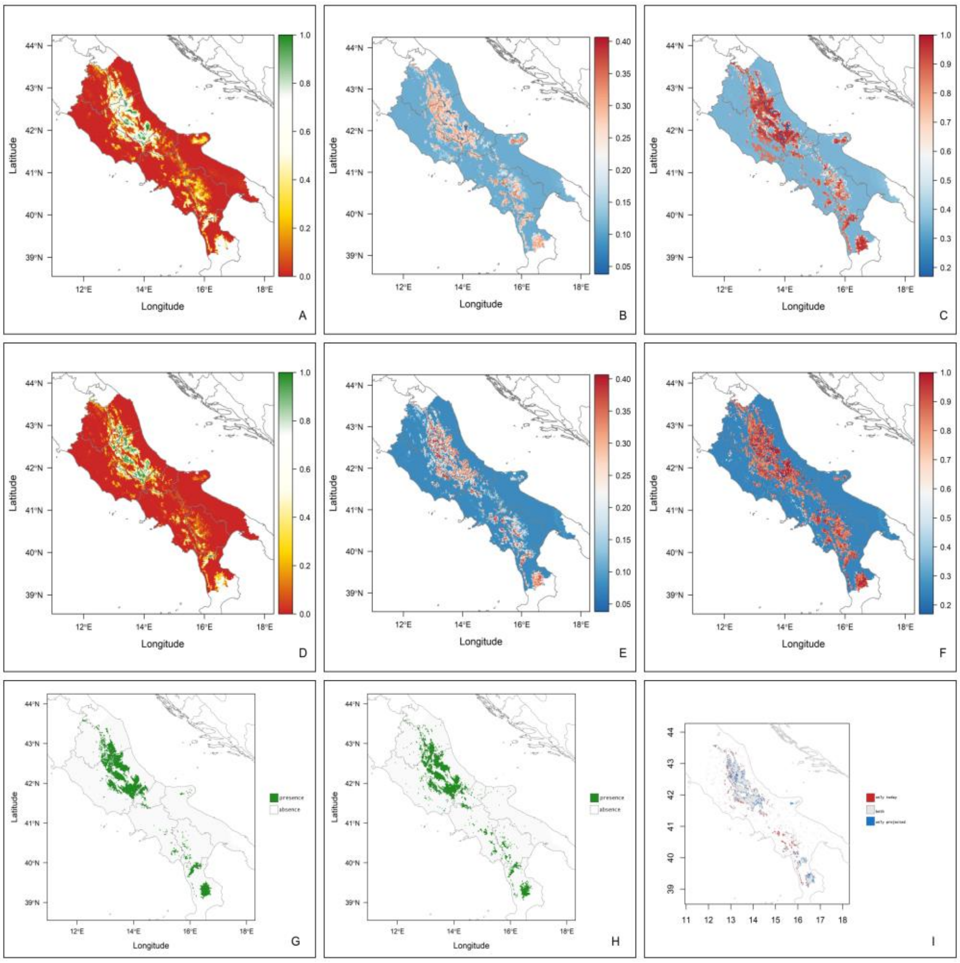

Fig. S.2.49-A) 'Projected' base output; B) 'Projected' uncertainty standard deviation; C) 'Projected' uncertainty range of values; D) 'Present' base output; E) 'Present' uncertainty standard deviation; F) 'Present' uncertainty range of values; G) 'Projected' binary distribution; H) 'Present' binary distribution; I) Range size variation.

Value	Present	Projected
AUC.train	0.991	0.980
AUC.test	0.962	0.968
AUC.diff	0.012	0.012
pROC	1.778	1.768
OR.p10	0.417	0.167
CBI	0.923	0.901
perc.stable	60.249	60.249
range.size	0.532	0.532

S.2.50 *Carduus affinis* Bioclim

Species: *Carduus affinis* Guss. subsp. affinis

Procedure: Bioclim

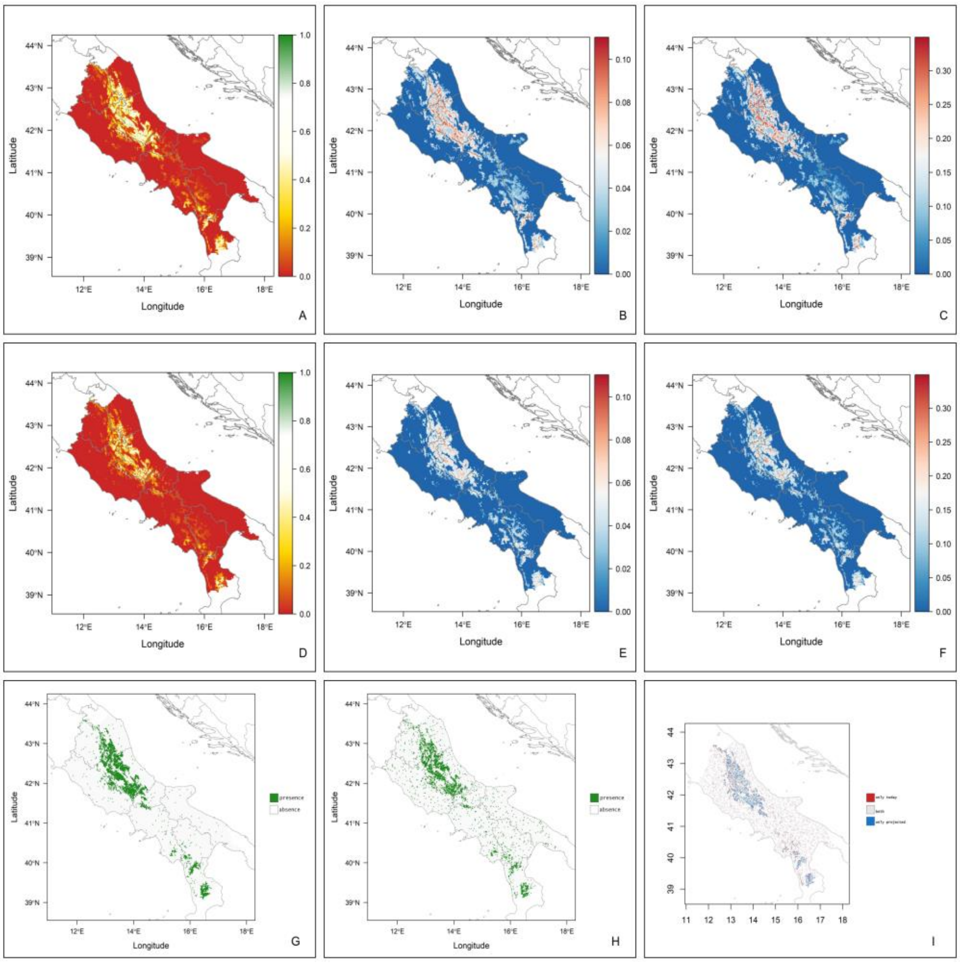

Fig. S.2.50-A) ‘Projected’ base output; B) ‘Projected’ uncertainty standard deviation; C) ‘Projected’ uncertainty range of values; D) ’Present’ base output; E) ’Present’ uncertainty standard deviation; F) ’Present’ uncertainty range of values; G) ‘Projected’ binary distribution; H) ‘Present’ binary distribution; I) Range size variation.

Value	Present	Projected
AUC.train	0.934	0.877
AUC.test	0.892	0.842
AUC.diff	0.042	0.042
pROC	1.383	1.303
OR.p10	0.211	0.161
CBI	0.724	0.908
perc.stable	44.188	44.188
range.size	1.442	1.442

S.2.51 *Carduus affinis* Domain

Species: *Carduus affinis* Guss. subsp. affinis

Procedure: Domain

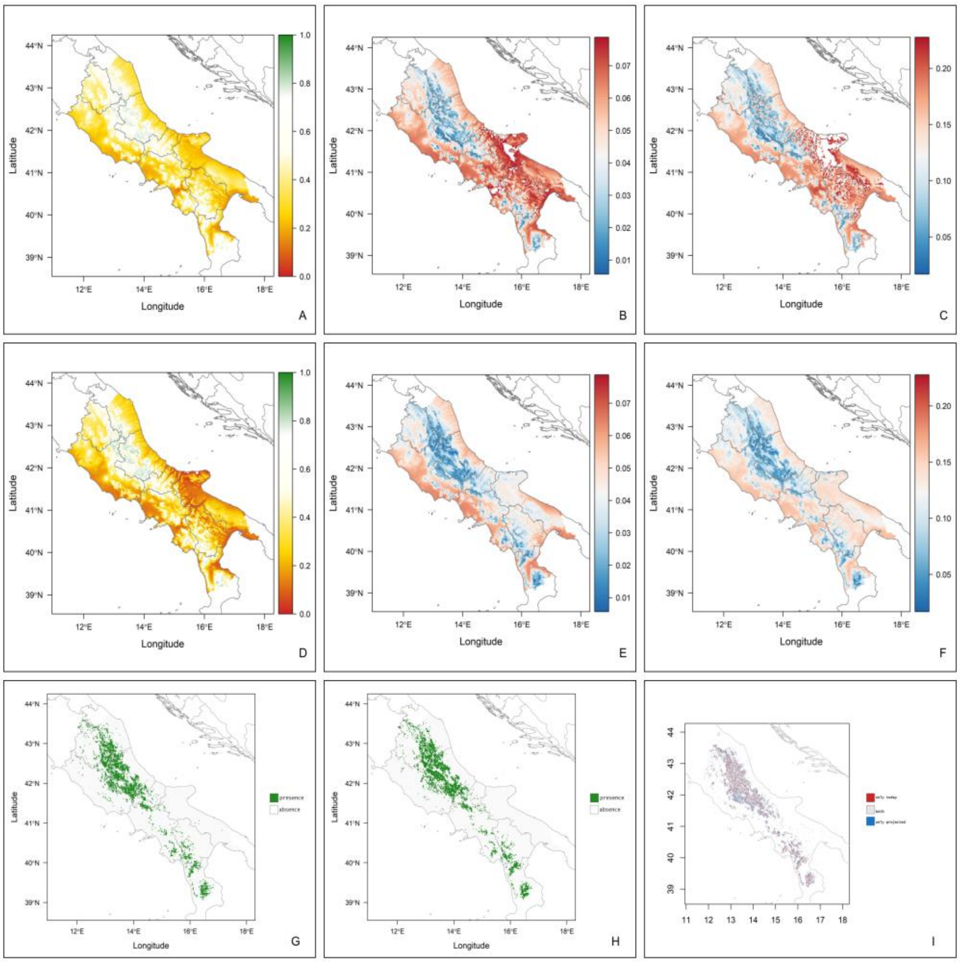

Fig. S.2.51-A) ‘Projected’ base output; B) ‘Projected’ uncertainty standard deviation; C) ‘Projected’ uncertainty range of values; D) ’Present’ base output; E) ’Present’ uncertainty standard deviation; F) ’Present’ uncertainty range of values; G) ‘Projected’ binary distribution; H) ‘Present’ binary distribution; I) Range size variation.

Value	Present	Projected
AUC.train	0.931	0.874
AUC.test	0.924	0.833
AUC.diff	0.052	0.052
pROC	1.624	1.395
OR.p10	0.172	0.222
CBI	0.917	0.910
perc.stable	49.312	49.312
range.size	0.792	0.792

S.2.52 *Carduus affinis* Maxent

Species: *Carduus affinis* Guss. subsp. affinis

Procedure: Maxent

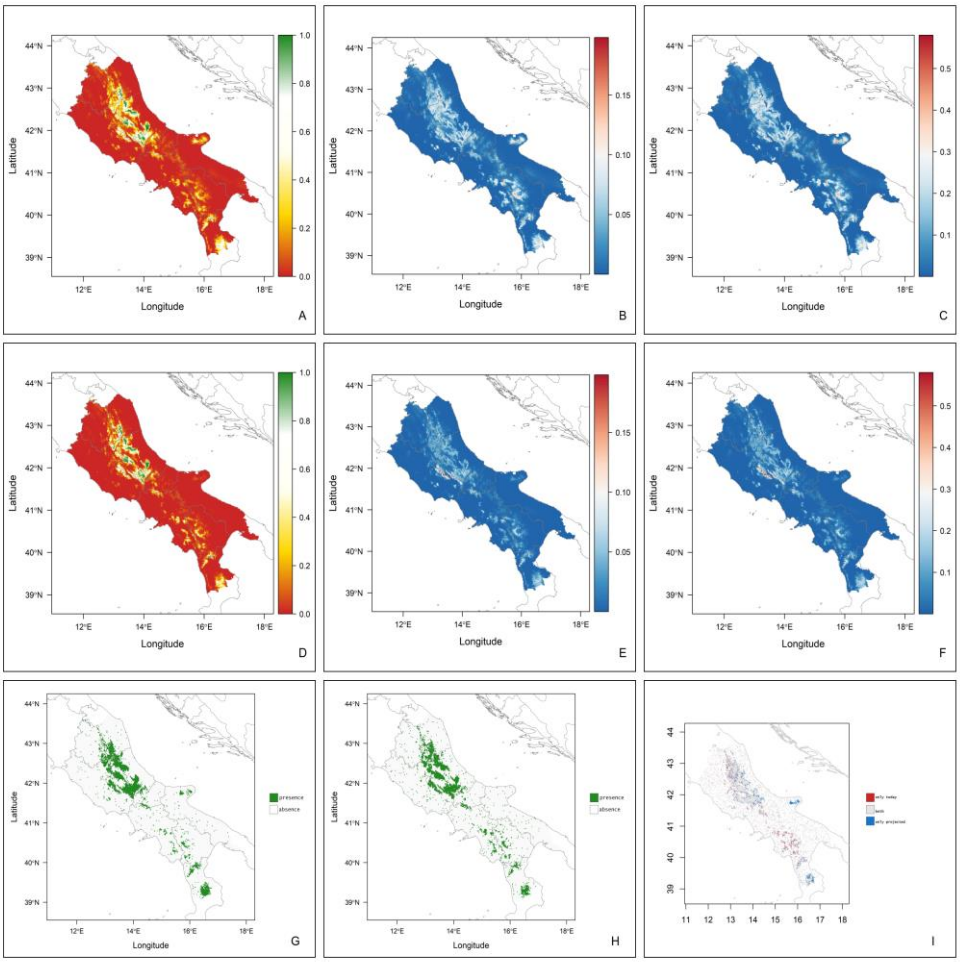

Fig. S.2.52-A) 'Projected' base output; B) 'Projected' uncertainty standard deviation; C) 'Projected' uncertainty range of values; D) 'Present' base output; E) 'Present' uncertainty standard deviation; F) 'Present' uncertainty range of values; G) 'Projected' binary distribution; H) 'Present' binary distribution; I) Range size variation.

Value	Present	Projected
AUC.train	0.962	0.935
AUC.test	0.958	0.936
AUC.diff	0.016	0.016
pROC	1.764	1.661
OR.p10	0.139	0.139
CBI	0.966	0.955
perc.stable	53.662	53.662
range.size	-0.321	-0.321

S.2.53 *Carduus affinis* KDE

Species: *Carduus affinis* Guss. subsp. affinis

Procedure: KDE

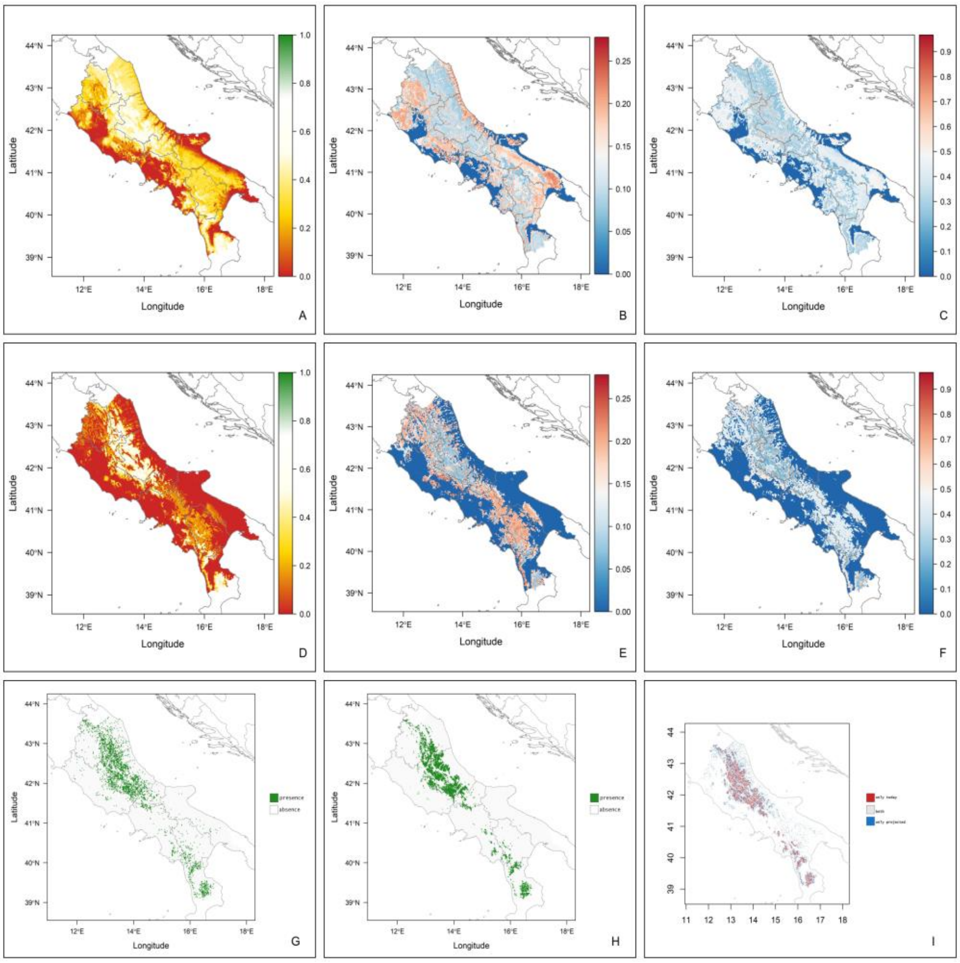

Fig. S.2.53-A) 'Projected' base output; B) 'Projected' uncertainty standard deviation; C) 'Projected' uncertainty range of values; D) 'Present' base output; E) 'Present' uncertainty standard deviation; F) 'Present' uncertainty range of values; G) 'Projected' binary distribution; H) 'Present' binary distribution; I) Range size variation.

Value	Present	Projected
AUC.train	0.948	0.902
AUC.test	0.893	0.860
AUC.diff	0.055	0.047
pROC	1.380	1.387
OR.p10	0.250	0.206
CBI	0.973	0.907
perc.stable	37.876	37.876
range.size	-0.893	-0.893

S.2.54 *Carduus affinis* CTA

Species: *Carduus affinis* Guss. subsp. affinis

Procedure: CTA

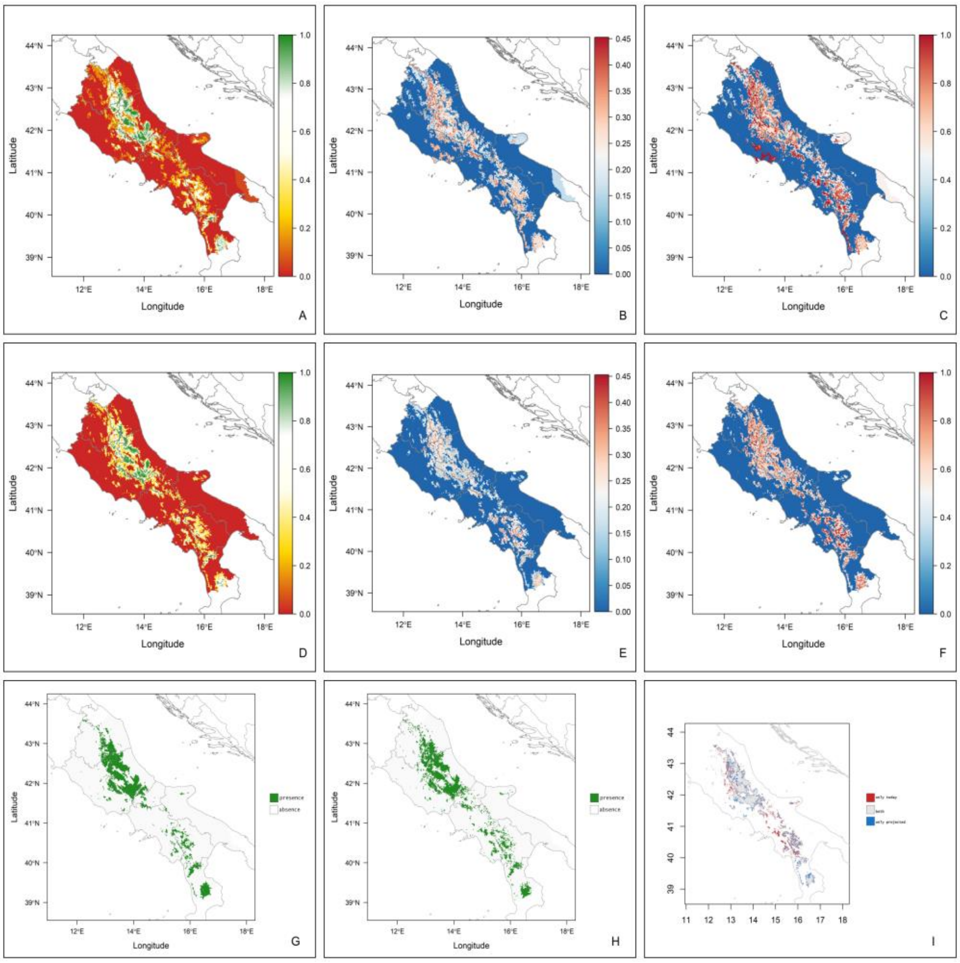

Fig. S.2.54-A) 'Projected' base output; B) 'Projected' uncertainty standard deviation; C) 'Projected' uncertainty range of values; D) 'Present' base output; E) 'Present' uncertainty standard deviation; F) 'Present' uncertainty range of values; G) 'Projected' binary distribution; H) 'Present' binary distribution; I) Range size variation.

Value	Present	Projected
AUC.train	0.970	0.965
AUC.test	0.942	0.918
AUC.diff	0.047	0.047
pROC	1.576	1.344
OR.p10	0.178	0.167
CBI	0.928	0.786
perc.stable	57.191	57.191
range.size	-0.490	-0.490

S.2.55 *Carduus affinis* FDA

Species: *Carduus affinis* Guss. subsp. affinis

Procedure: FDA

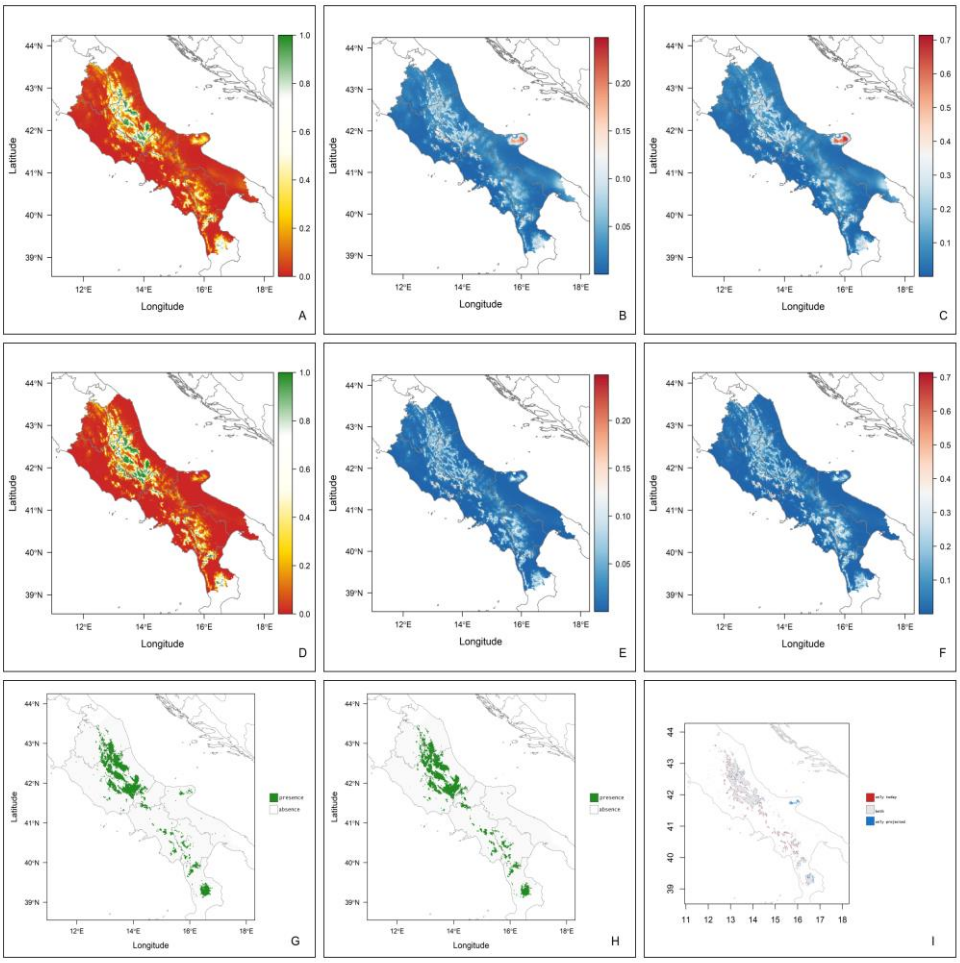

Fig. S.2.55-A) 'Projected' base output; B) 'Projected' uncertainty standard deviation; C) 'Projected' uncertainty range of values; D) 'Present' base output; E) 'Present' uncertainty standard deviation; F) 'Present' uncertainty range of values; G) 'Projected' binary distribution; H) 'Present' binary distribution; I) Range size variation.

Value	Present	Projected
AUC.train	0.949	0.932
AUC.test	0.958	0.932
AUC.diff	0.010	0.010
pROC	1.824	1.609
OR.p10	0.072	0.122
CBI	0.913	0.971
perc.stable	76.328	76.328
range.size	-0.441	-0.441

S.2.56 *Carduus affinis* GAM

Species: *Carduus affinis* Guss. subsp. affinis

Procedure: GAM

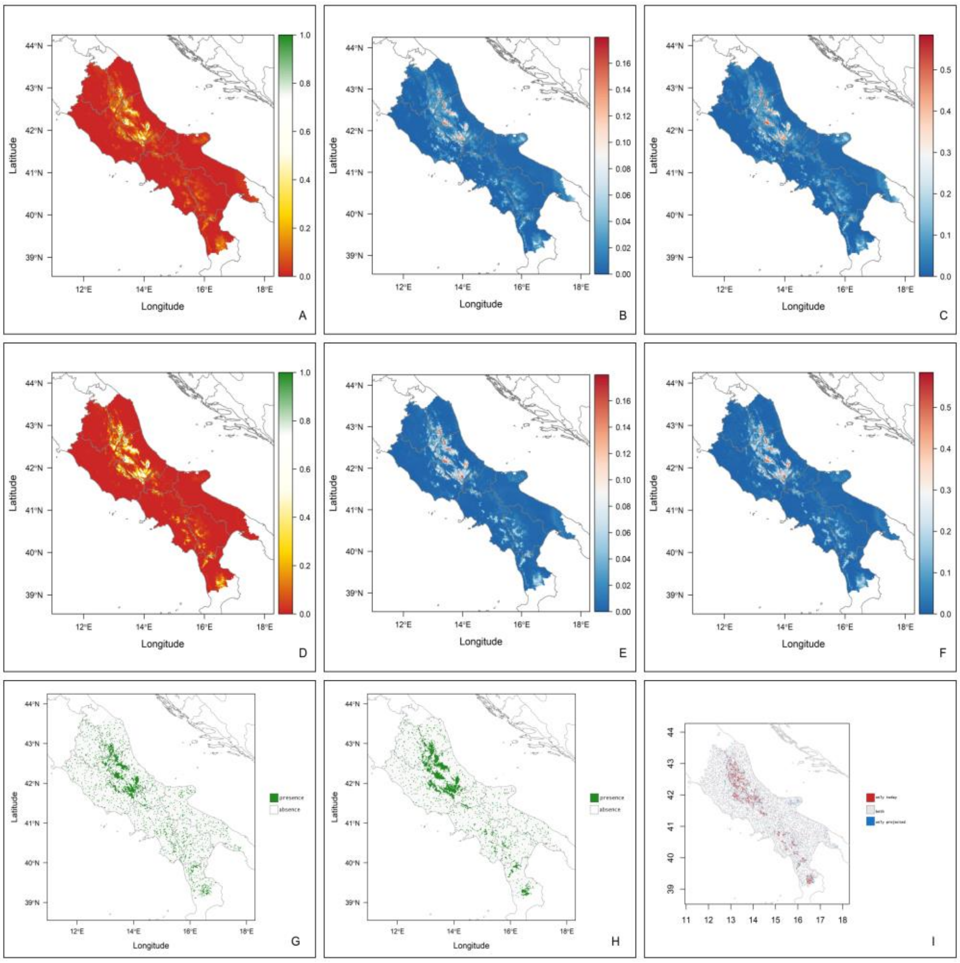

Fig. S.2.56-A) ‘Projected’ base output; B) ‘Projected’ uncertainty standard deviation; C) ‘Projected’ uncertainty range of values; D) ’Present’ base output; E) ’Present’ uncertainty standard deviation; F) ’Present’ uncertainty range of values; G) ‘Projected’ binary distribution; H) ‘Present’ binary distribution; I) Range size variation.

Value	Present	Projected
AUC.train	0.961	0.925
AUC.test	0.961	0.931
AUC.diff	0.011	0.011
pROC	1.764	1.635
OR.p10	0.117	0.150
CBI	0.863	0.758
perc.stable	23.141	23.141
range.size	0.073	0.073

S.2.57 *Carduus affinis* GLM

Species: *Carduus affinis* Guss. subsp. affinis

Procedure: GLM

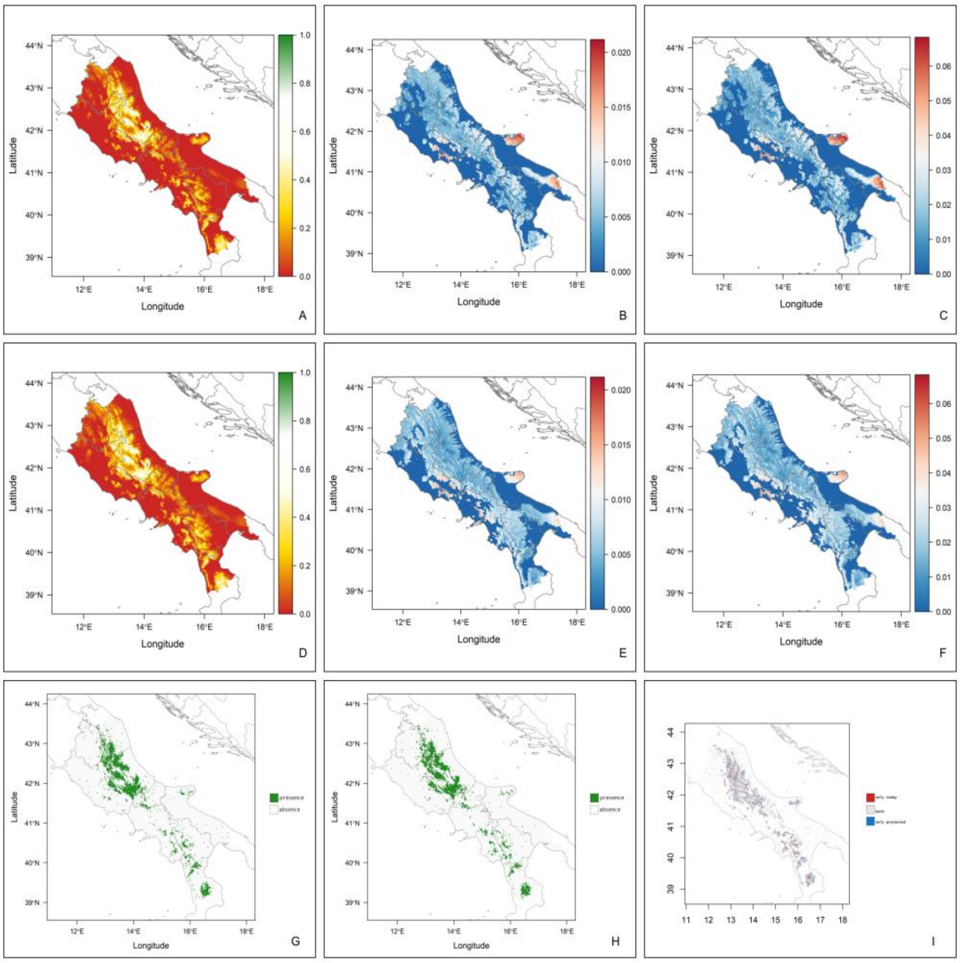

Fig. S.2.57-A) 'Projected' base output; B) 'Projected' uncertainty standard deviation; C) 'Projected' uncertainty range of values; D) 'Present' base output; E) 'Present' uncertainty standard deviation; F) 'Present' uncertainty range of values; G) 'Projected' binary distribution; H) 'Present' binary distribution; I) Range size variation.

Value	Present	Projected
AUC.train	0.955	0.932
AUC.test	0.964	0.942
AUC.diff	0.009	0.009
pROC	1.798	1.675
OR.p10	0.089	0.117
CBI	0.894	0.862
perc.stable	50.755	50.755
range.size	1.669	1.669

S.2.58 *Carduus affinis* MARS

Species: *Carduus affinis* Guss. subsp. affinis

Procedure: MARS

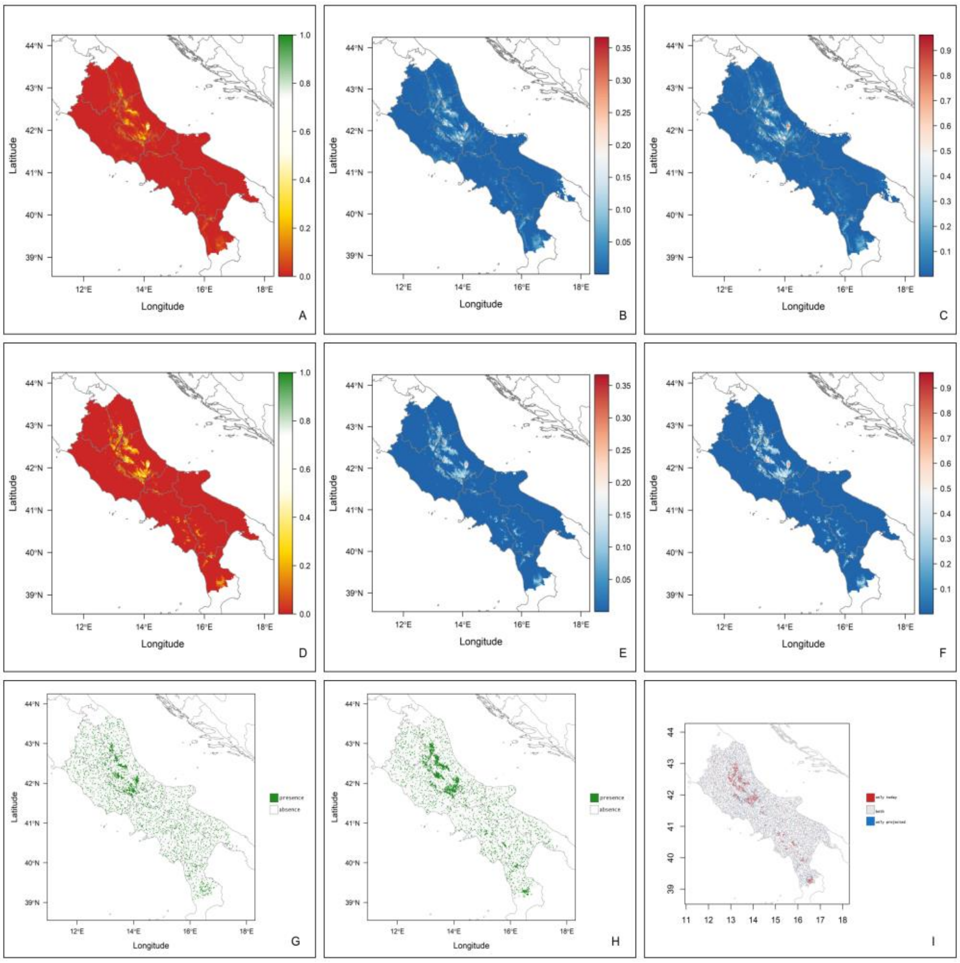

Fig. S.2.58-A) 'Projected' base output; B) 'Projected' uncertainty standard deviation; C) 'Projected' uncertainty range of values; D) 'Present' base output; E) 'Present' uncertainty standard deviation; F) 'Present' uncertainty range of values; G) 'Projected' binary distribution; H) 'Present' binary distribution; I) Range size variation.

Value	Present	Projected
AUC.train	0.936	0.934
AUC.test	0.899	0.904
AUC.diff	0.039	0.039
pROC	1.413	1.453
OR.p10	0.206	0.228
CBI	0.836	0.690
perc.stable	12.480	12.480
range.size	-0.427	-0.427

S.2.59 *Carduus affinis* RF

Species: *Carduus affinis* Guss. subsp. affinis

Procedure: RF

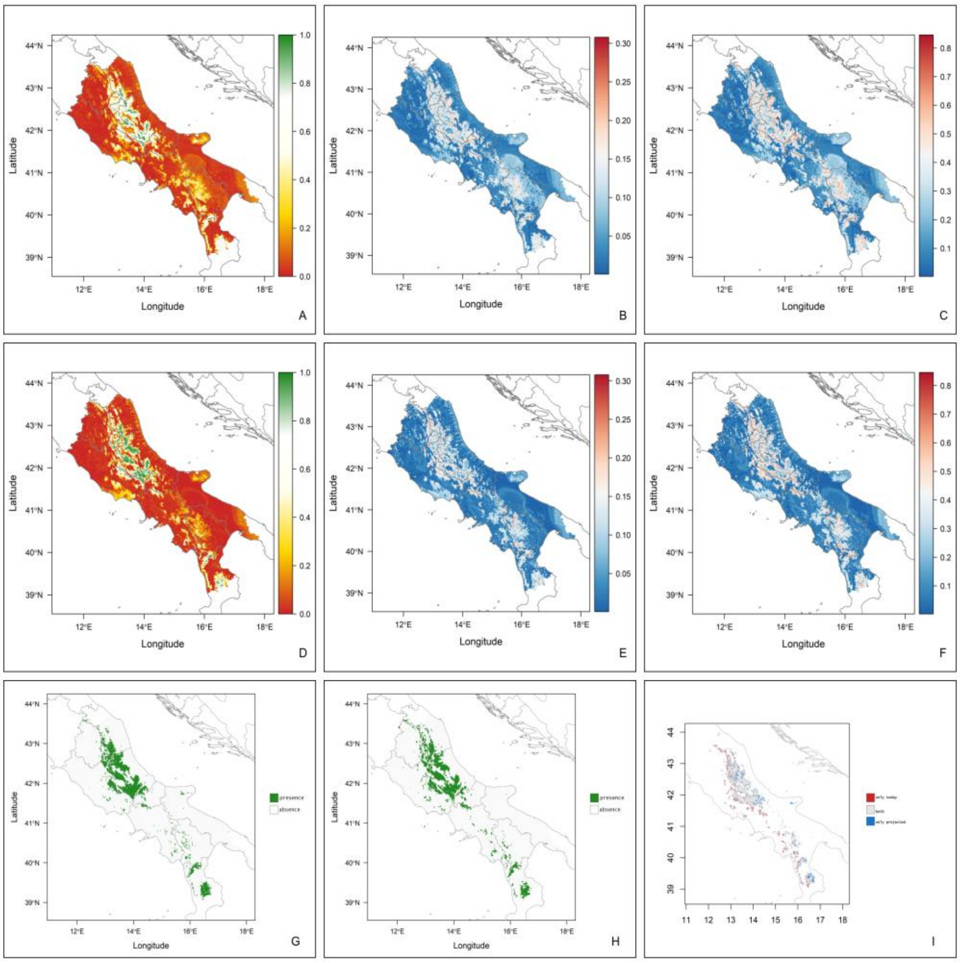

Fig. S.2.59-A) 'Projected' base output; B) 'Projected' uncertainty standard deviation; C) 'Projected' uncertainty range of values; D) 'Present' base output; E) 'Present' uncertainty standard deviation; F) 'Present' uncertainty range of values; G) 'Projected' binary distribution; H) 'Present' binary distribution; I) Range size variation.

Value	Present	Projected
AUC.train	1.000	1.000
AUC.test	0.971	0.968
AUC.diff	0.032	0.032
pROC	1.631	1.561
OR.p10	0.333	0.439
CBI	0.958	0.945
perc.stable	64.296	64.296
range.size	1.566	1.566

S.2.60 *Carduus affinis* SVM

Species: *Carduus affinis* Guss. subsp. affinis

Procedure: SVM

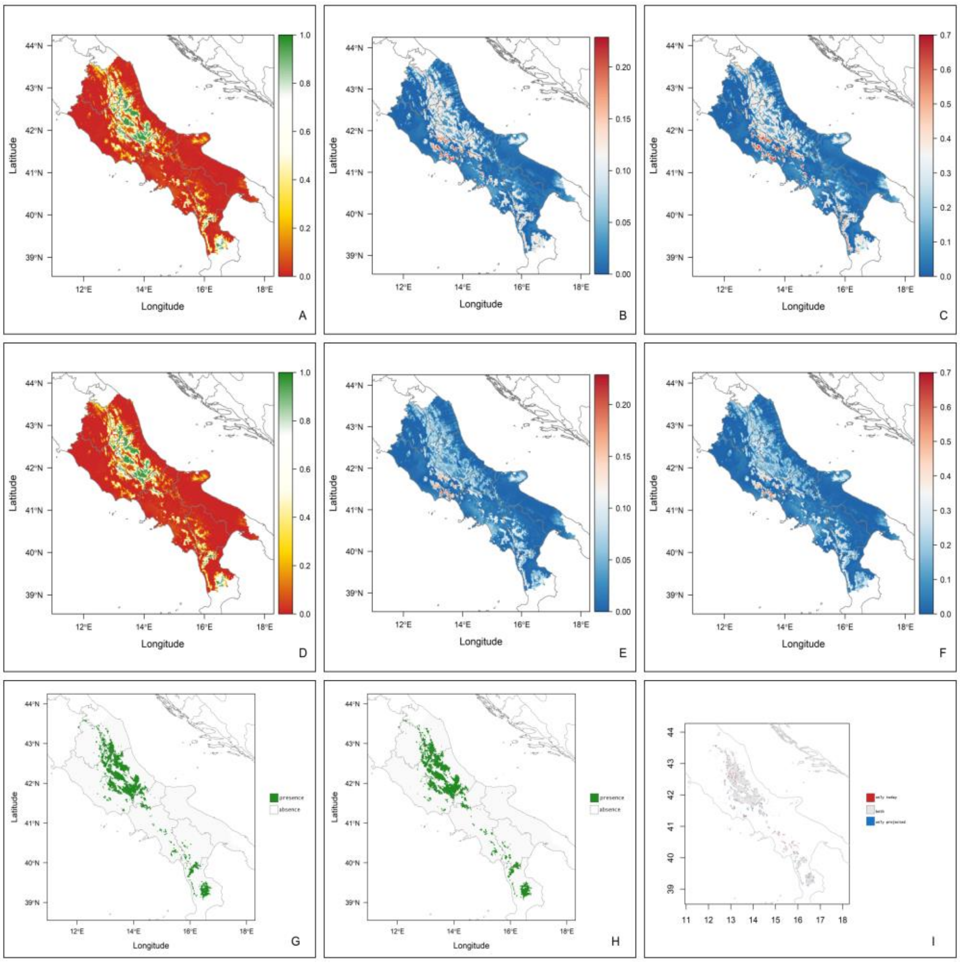

Fig. S.2.60-A) ‘Projected’ base output; B) ‘Projected’ uncertainty standard deviation; C) ‘Projected’ uncertainty range of values; D) ’Present’ base output; E) ’Present’ uncertainty standard deviation; F) ’Present’ uncertainty range of values; G) ‘Projected’ binary distribution; H) ‘Present’ binary distribution; I) Range size variation.

Value	Present	Projected
AUC.train	0.953	0.938
AUC.test	0.951	0.922
AUC.diff	0.023	0.023
pROC	1.716	1.547
OR.p10	0.178	0.178
CBI	0.890	0.921
perc.stable	80.764	80.764
range.size	-1.159	-1.159

S.2.61 *Crocus biflorus* Biomod2

Species: *Crocus biflorus* Mill.

Procedure: Biomod2

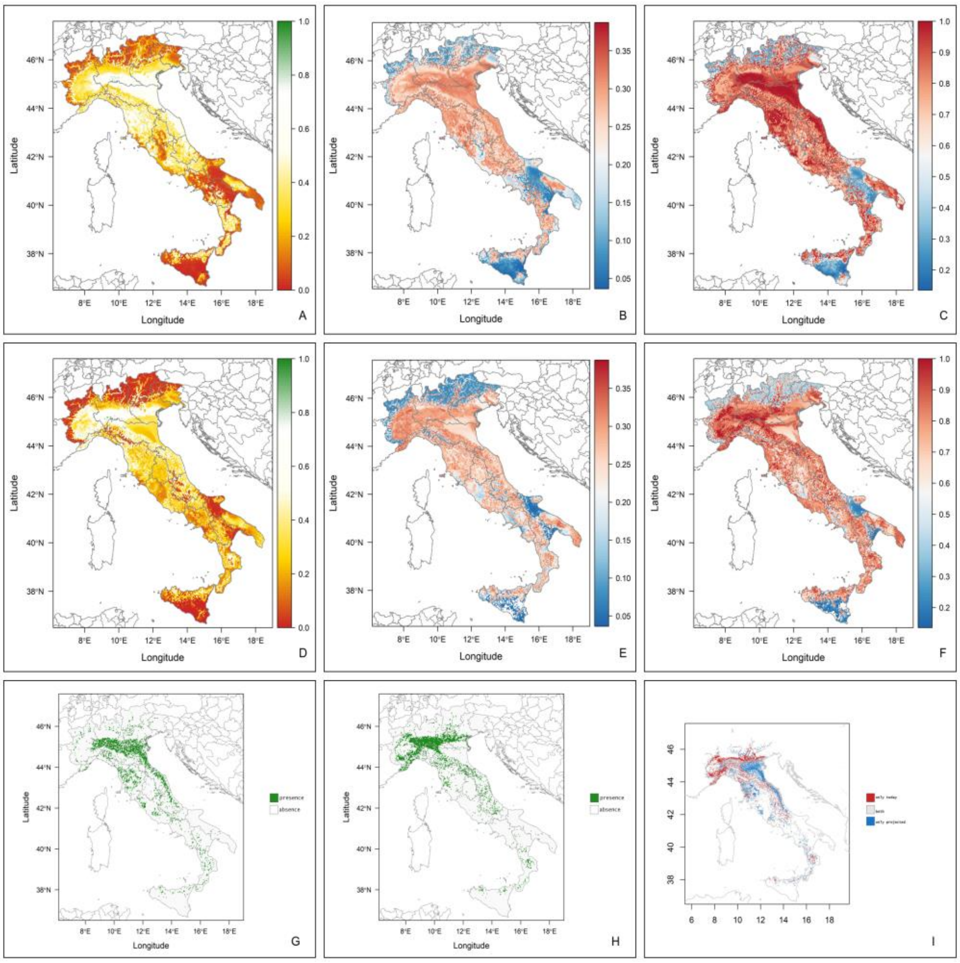

Fig. S.2.61-A) 'Projected' base output; B) 'Projected' uncertainty standard deviation; C) 'Projected' uncertainty range of values; D) 'Present' base output; E) 'Present' uncertainty standard deviation; F) 'Present' uncertainty range of values; G) 'Projected' binary distribution; H) 'Present' binary distribution; I) Range size variation.

Value	Present	Projected
AUC.train	0.985	0.985
AUC.test	0.714	0.714
AUC.diff	0.270	0.270
pROC	1.151	1.195
OR.p10	0.924	0.841
CBI	0.965	0.624
perc.stable	16.364	16.364
range.size	-0.946	-0.946

S.2.62 *Crocus biflorus* Bioclim

Species: *Crocus biflorus* Mill.

Procedure: Bioclim

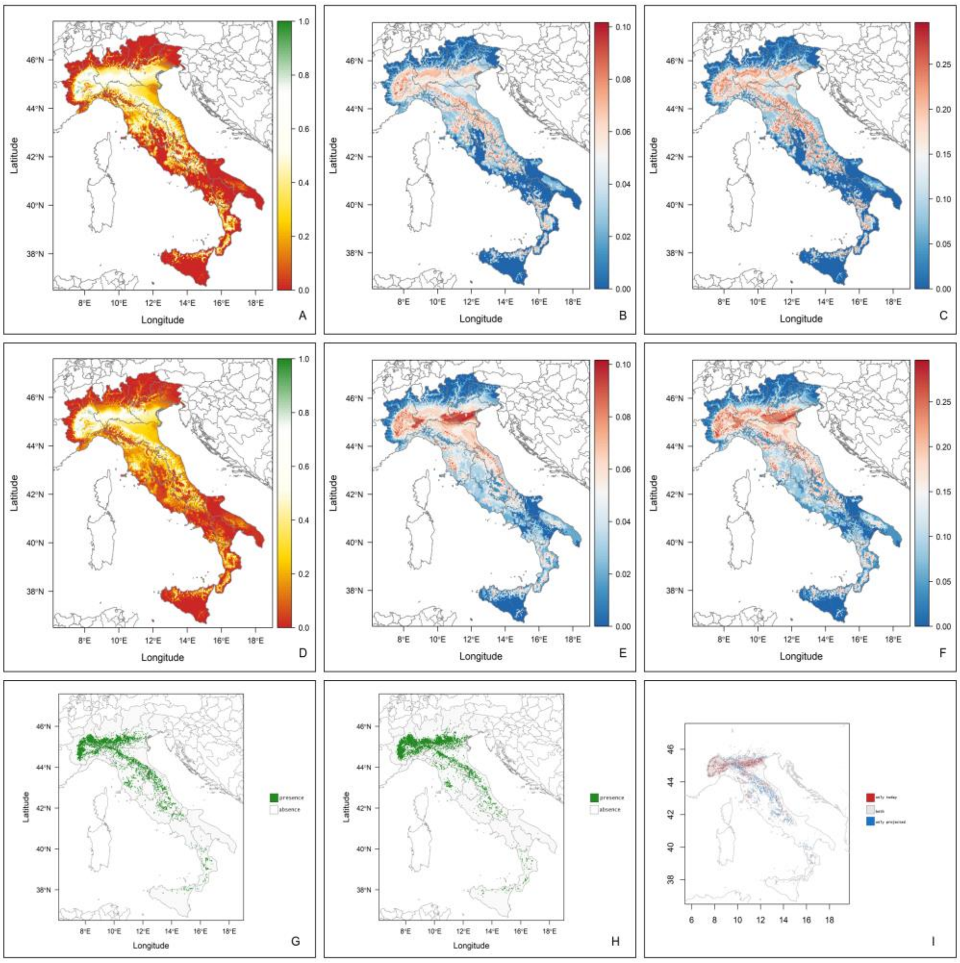

Fig. S.2.62-A) 'Projected' base output; B) 'Projected' uncertainty standard deviation; C) 'Projected' uncertainty range of values; D) 'Present' base output; E) 'Present' uncertainty standard deviation; F) 'Present' uncertainty range of values; G) 'Projected' binary distribution; H) 'Present' binary distribution; I) Range size variation.

Value	Present	Projected
AUC.train	0.705	0.660
AUC.test	0.649	0.628
AUC.diff	0.036	0.036
pROC	1.088	1.092
OR.p10	0.180	0.173
CBI	0.549	0.674
perc.stable	51.618	51.618
range.size	-0.201	-0.201

S.2.63 *Crocus biflorus* Domain

Species: *Crocus biflorus* Mill.

Procedure: Domain

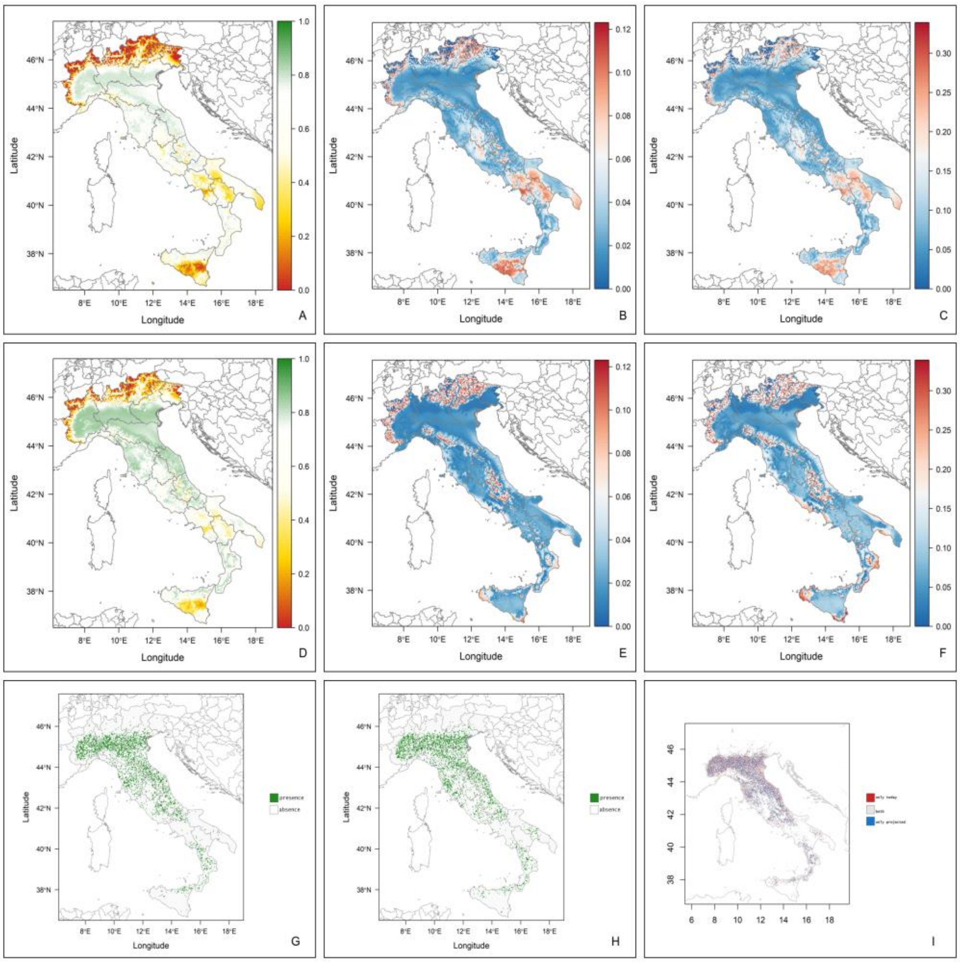

Fig. S.2.63-A) ‘Projected’ base output; B) ‘Projected’ uncertainty standard deviation; C) ‘Projected’ uncertainty range of values; D) ’Present’ base output; E) ’Present’ uncertainty standard deviation; F) ’Present’ uncertainty range of values; G) ‘Projected’ binary distribution; H) ‘Present’ binary distribution; I) Range size variation.

Value	Present	Projected
AUC.train	0.675	0.639
AUC.test	0.686	0.641
AUC.diff	0.021	0.021
pROC	1.182	1.174
OR.p10	0.096	0.145
CBI	0.678	0.758
perc.stable	18.396	18.396
range.size	-2.098	-2.098

S.2.64 *Crocus biflorus* Maxent

Species: *Crocus biflorus* Mill.

Procedure: Maxent

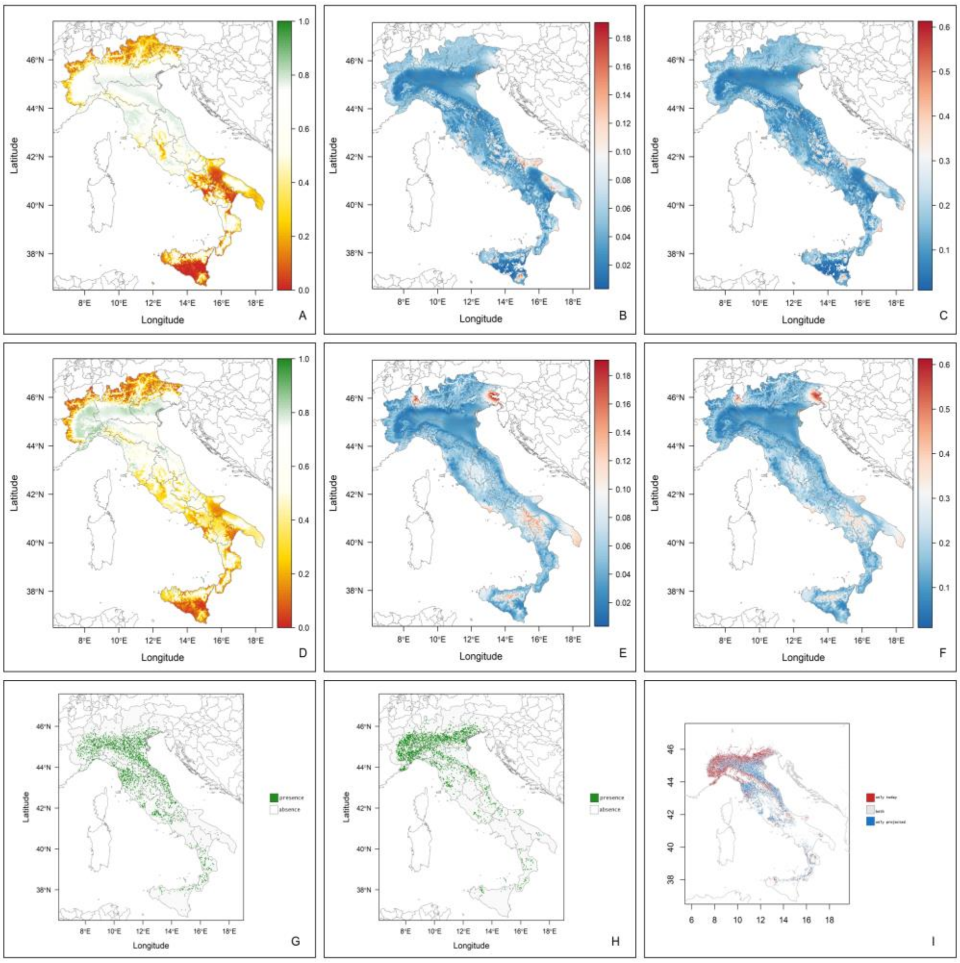

Fig. S.2.64-A) 'Projected' base output; B) 'Projected' uncertainty standard deviation; C) 'Projected' uncertainty range of values; D) 'Present' base output; E) 'Present' uncertainty standard deviation; F) 'Present' uncertainty range of values; G) 'Projected' binary distribution; H) 'Present' binary distribution; I) Range size variation.

Value	Present	Projected
AUC.train	0.720	0.705
AUC.test	0.675	0.661
AUC.diff	0.049	0.049
pROC	1.152	1.193
OR.p10	0.183	0.200
CBI	0.958	0.936
perc.stable	14.610	14.610
range.size	0.063	0.063

S.2.65 *Crocus biflorus* KDE

Species: *Crocus biflorus* Mill.

Procedure: KDE

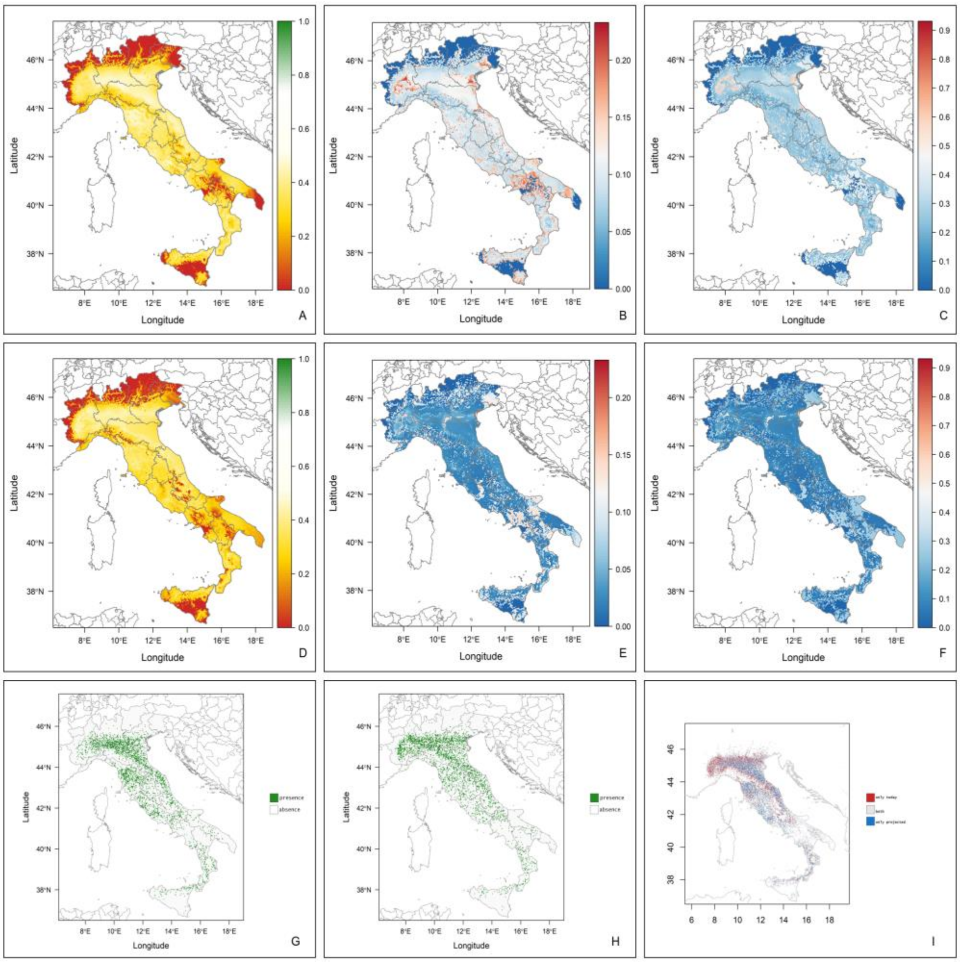

Fig. S.2.65-A) 'Projected' base output; B) 'Projected' uncertainty standard deviation; C) 'Projected' uncertainty range of values; D) 'Present' base output; E) 'Present' uncertainty standard deviation; F) 'Present' uncertainty range of values; G) 'Projected' binary distribution; H) 'Present' binary distribution; I) Range size variation.

Value	Present	Projected
AUC.train	0.675	0.652
AUC.test	0.660	0.602
AUC.diff	0.040	0.057
pROC	1.100	1.101
OR.p10	0.141	0.150
CBI	0.794	0.779
perc.stable	15.701	15.701
range.size	0.195	0.195

S.2.66 *Crocus biflorus* CTA

Species: *Crocus biflorus* Mill.

Procedure: CTA

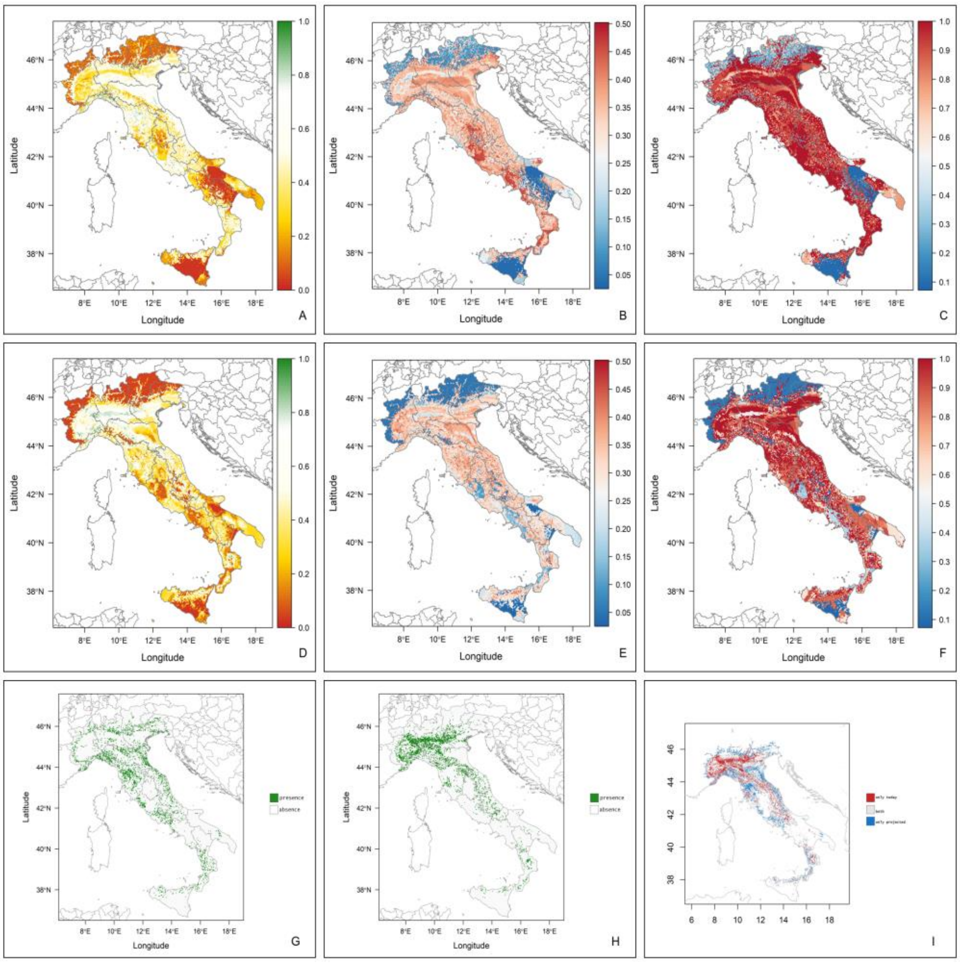

Fig. S.2.66-A) 'Projected' base output; B) 'Projected' uncertainty standard deviation; C) 'Projected' uncertainty range of values; D) 'Present' base output; E) 'Present' uncertainty standard deviation; F) 'Present' uncertainty range of values; G) 'Projected' binary distribution; H) 'Present' binary distribution; I) Range size variation.

Value	Present	Projected
AUC.train	0.899	0.923
AUC.test	0.711	0.714
AUC.diff	0.209	0.209
pROC	1.039	1.032
OR.p10	0.370	0.400
CBI	0.994	0.328
perc.stable	6.887	6.887
range.size	-1.454	-1.454

S.2.67 *Crocus biflorus* FDA

Species: *Crocus biflorus* Mill.

Procedure: FDA

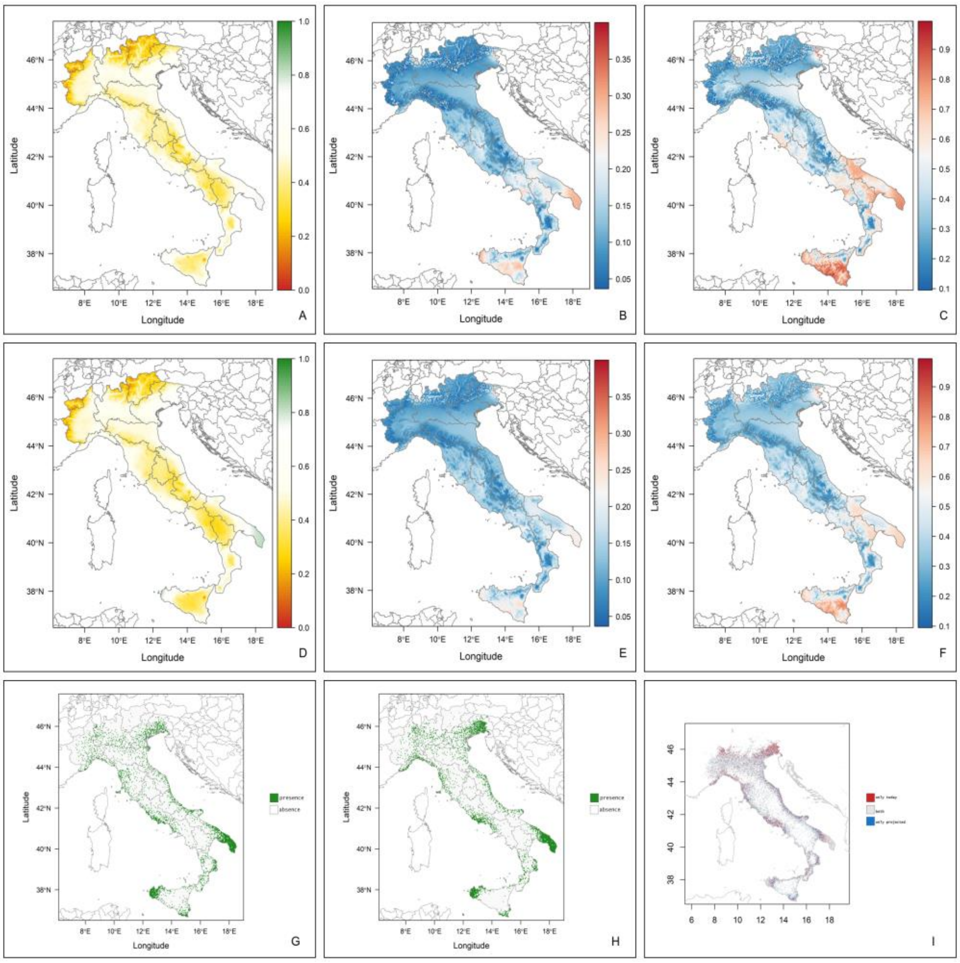

Fig. S.2.67-A) 'Projected' base output; B) 'Projected' uncertainty standard deviation; C) 'Projected' uncertainty range of values; D) 'Present' base output; E) 'Present' uncertainty standard deviation; F) 'Present' uncertainty range of values; G) 'Projected' binary distribution; H) 'Present' binary distribution; I) Range size variation.

Value	Present	Projected
AUC.train	0.609	0.590
AUC.test	0.559	0.569
AUC.diff	0.041	0.041
pROC	1.098	1.099
OR.p10	0.145	0.195
CBI	0.734	0.740
perc.stable	26.420	26.420
range.size	0.136	0.136

S.2.68 *Crocus biflorus* GAM

Species: *Crocus biflorus* Mill.

Procedure: GAM

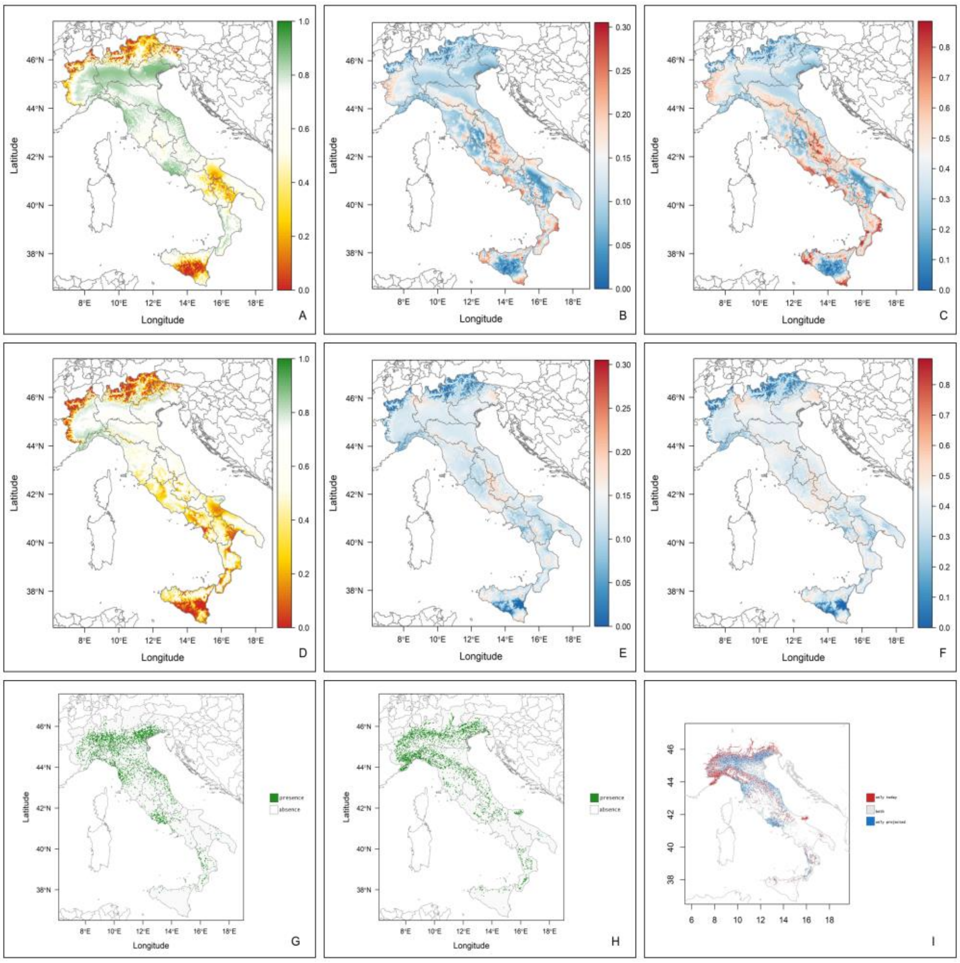

Fig. S.2.68-A) 'Projected' base output; B) 'Projected' uncertainty standard deviation; C) 'Projected' uncertainty range of values; D) 'Present' base output; E) 'Present' uncertainty standard deviation; F) 'Present' uncertainty range of values; G) 'Projected' binary distribution; H) 'Present' binary distribution; I) Range size variation.

Value	Present	Projected
AUC.train	0.703	0.662
AUC.test	0.655	0.616
AUC.diff	0.048	0.048
pROC	1.176	1.168
OR.p10	0.166	0.182
CBI	0.955	0.883
perc.stable	14.093	14.093
range.size	-1.327	-1.327

S.2.69 *Crocus biflorus* GLM

Species: *Crocus biflorus* Mill.

Procedure: GLM

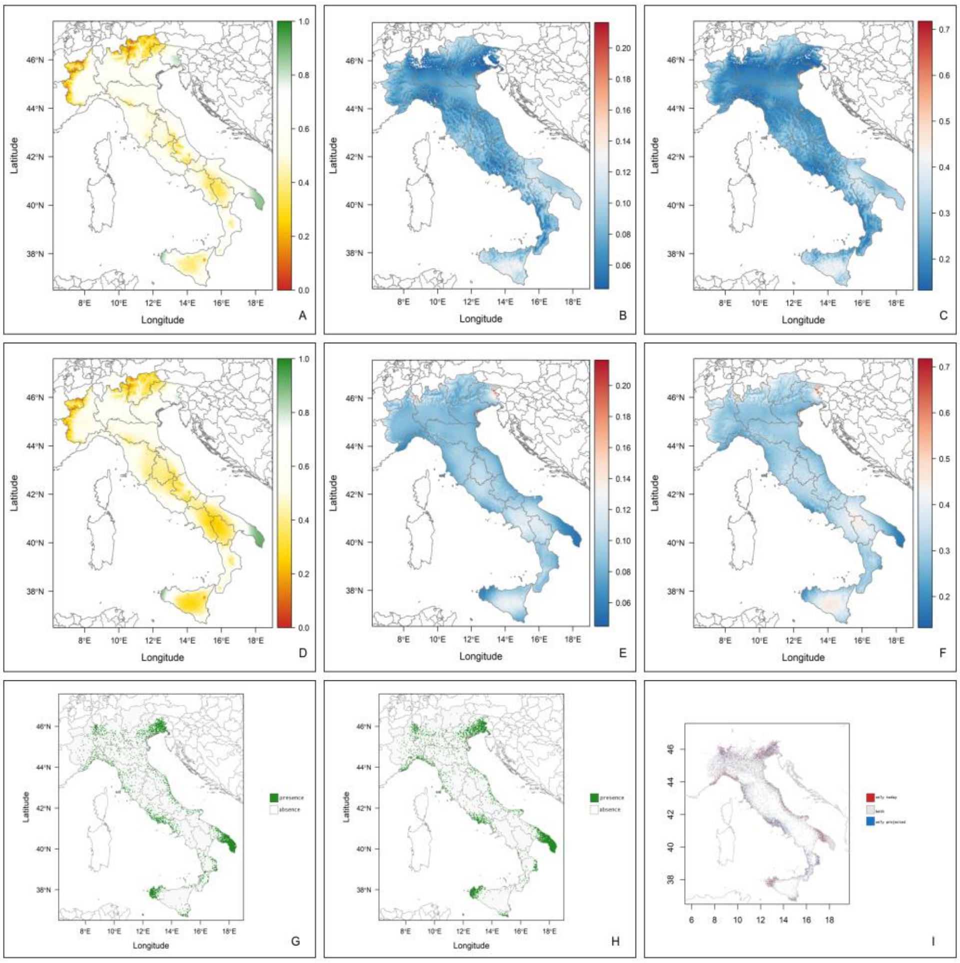

Fig. S.2.69-A) ‘Projected’ base output; B) ‘Projected’ uncertainty standard deviation; C) ‘Projected’ uncertainty range of values; D) ’Present’ base output; E) ’Present’ uncertainty standard deviation; F) ’Present’ uncertainty range of values; G) ‘Projected’ binary distribution; H) ‘Present’ binary distribution; I) Range size variation.

Value	Present	Projected
AUC.train	0.597	0.600
AUC.test	0.585	0.573
AUC.diff	0.032	0.032
pROC	1.120	1.101
OR.p10	0.114	0.155
CBI	0.691	0.681
perc.stable	29.377	29.377
range.size	-0.851	-0.851

S.2.70 *Crocus biflorus* MARS

Species: *Crocus biflorus* Mill.

Procedure: MARS

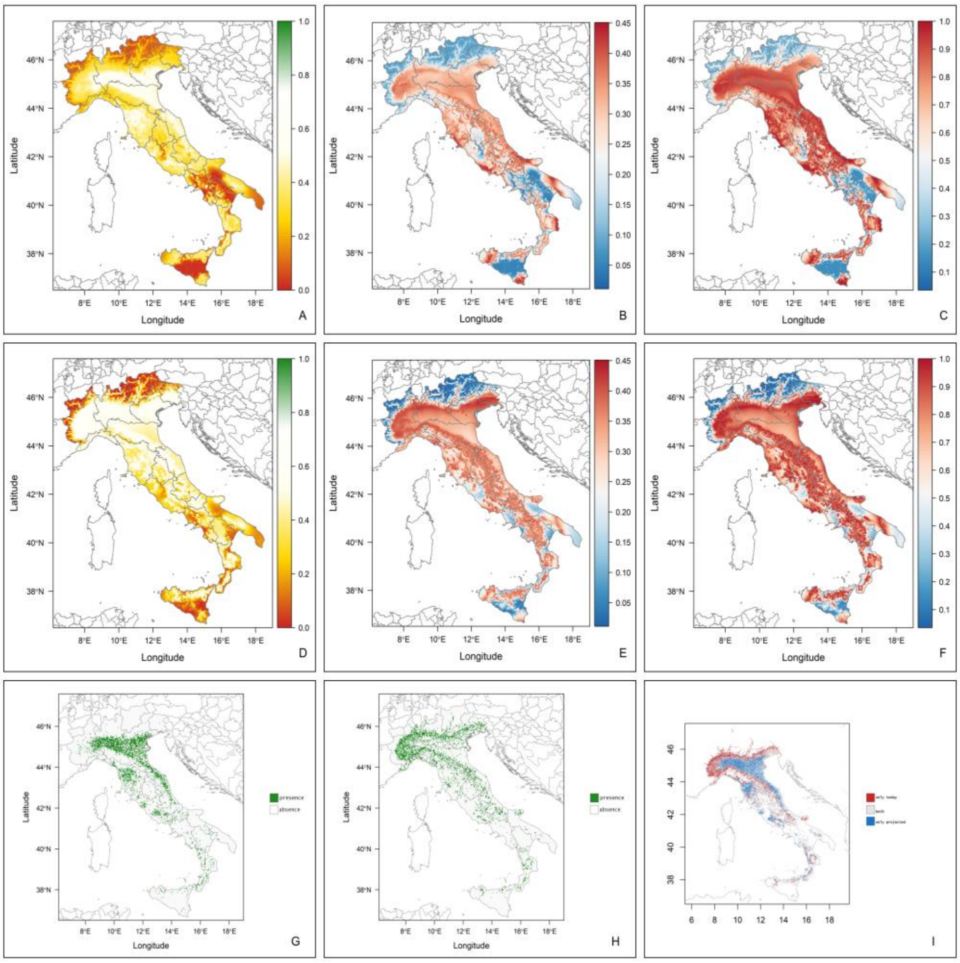

Fig. S.2.70-A) ‘Projected’ base output; B) ‘Projected’ uncertainty standard deviation; C) ‘Projected’ uncertainty range of values; D) ’Present’ base output; E) ’Present’ uncertainty standard deviation; F) ’Present’ uncertainty range of values; G) ‘Projected’ binary distribution; H) ‘Present’ binary distribution; I) Range size variation.

Value	Present	Projected
AUC.train	0.699	0.689
AUC.test	0.623	0.579
AUC.diff	0.113	0.113
pROC	1.125	1.121
OR.p10	0.221	0.245
CBI	0.937	0.916
perc.stable	6.972	6.972
range.size	-0.109	-0.109

S.2.71 *Crocus biflorus* RF

Species: *Crocus biflorus* Mill.

Procedure: RF

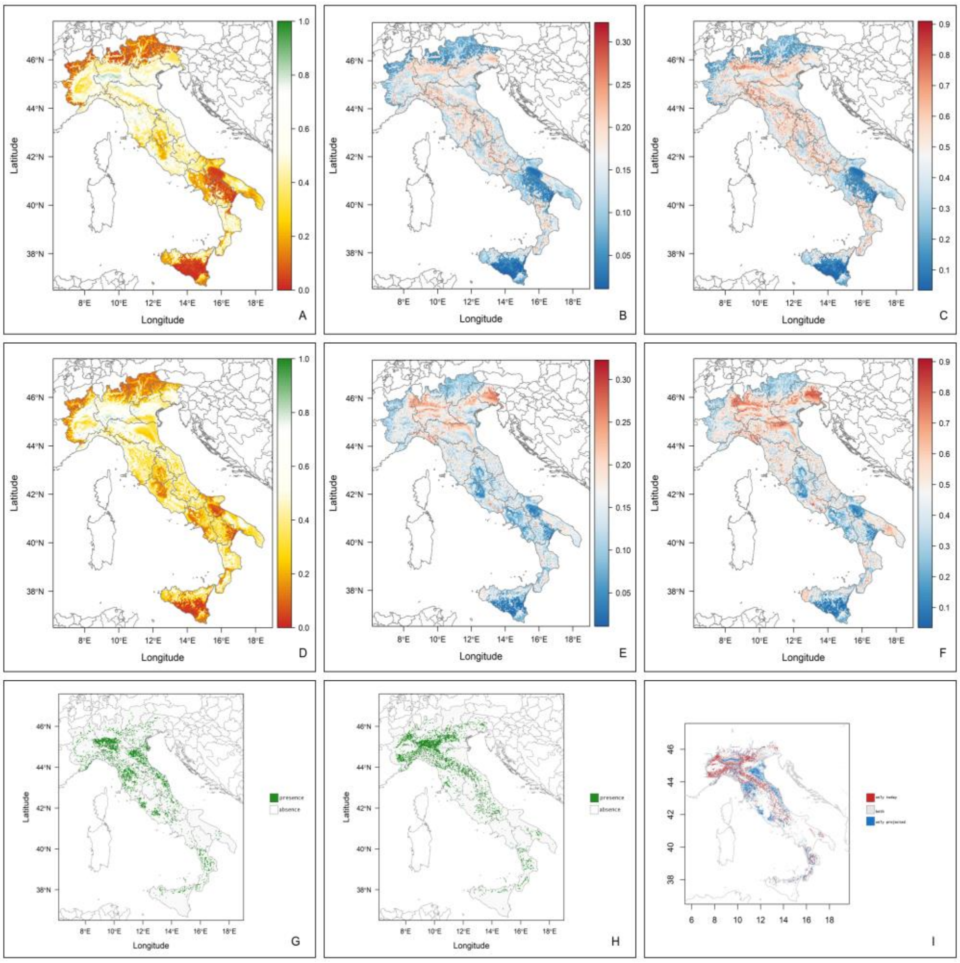

Fig. S.2.71-A) 'Projected' base output; B) 'Projected' uncertainty standard deviation; C) 'Projected' uncertainty range of values; D) 'Present' base output; E) 'Present' uncertainty standard deviation; F) 'Present' uncertainty range of values; G) 'Projected' binary distribution; H) 'Present' binary distribution; I) Range size variation.

Value	Present	Projected
AUC.train	1.000	1.000
AUC.test	0.932	0.932
AUC.diff	0.068	0.068
pROC	1.142	1.156
OR.p10	0.833	0.800
CBI	0.998	0.716
perc.stable	14.035	14.035
range.size	0.277	0.277

S.2.72 *Crocus biflorus* SVM

Species: *Crocus biflorus* Mill.

Procedure: SVM

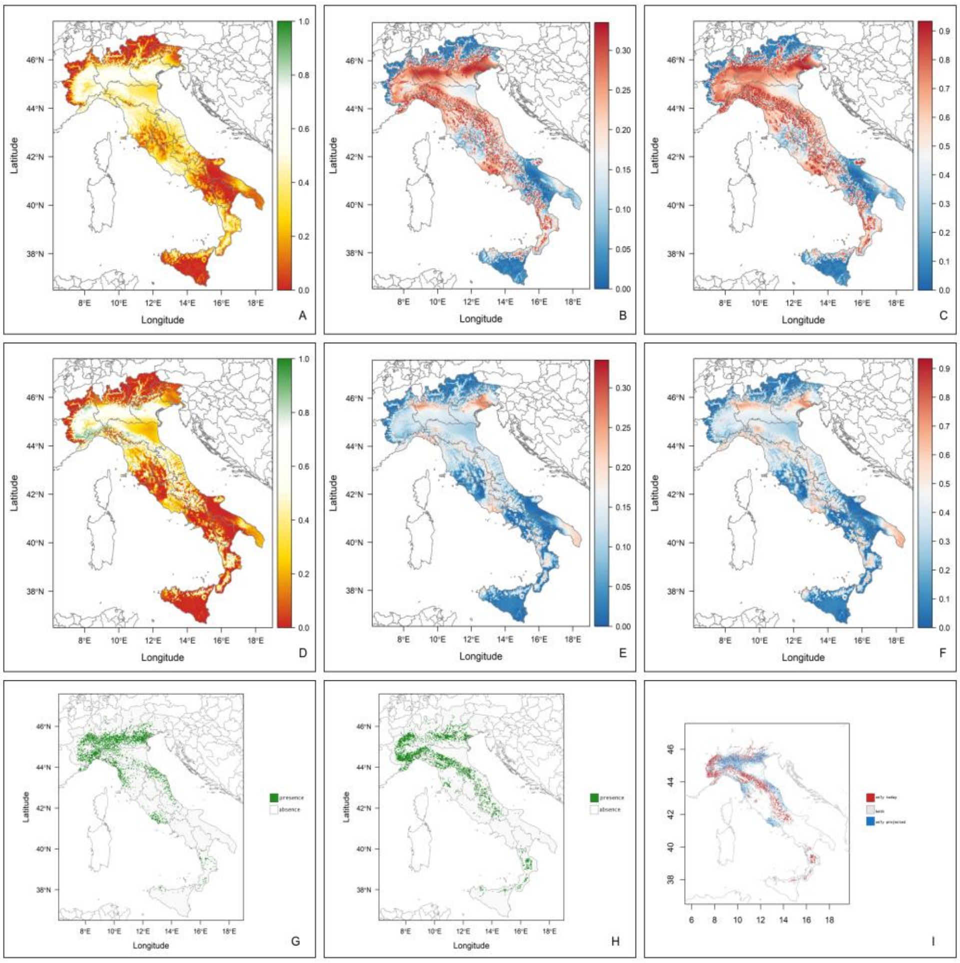

Fig. S.2.72-A) ‘Projected’ base output; B) ‘Projected’ uncertainty standard deviation; C) ‘Projected’ uncertainty range of values; D) ’Present’ base output; E) ’Present’ uncertainty standard deviation; F) ’Present’ uncertainty range of values; G) ‘Projected’ binary distribution; H) ‘Present’ binary distribution; I) Range size variation.

Value	Present	Projected
AUC.train	0.742	0.752
AUC.test	0.705	0.675
AUC.diff	0.077	0.077
pROC	1.101	1.122
OR.p10	0.156	0.191
CBI	0.944	0.831
perc.stable	24.247	24.247
range.size	-0.799	-0.799

S.2.73 *Crocus etruscus* Biomod2

Species: *Crocus etruscus* Parl.

Procedure: Biomod2

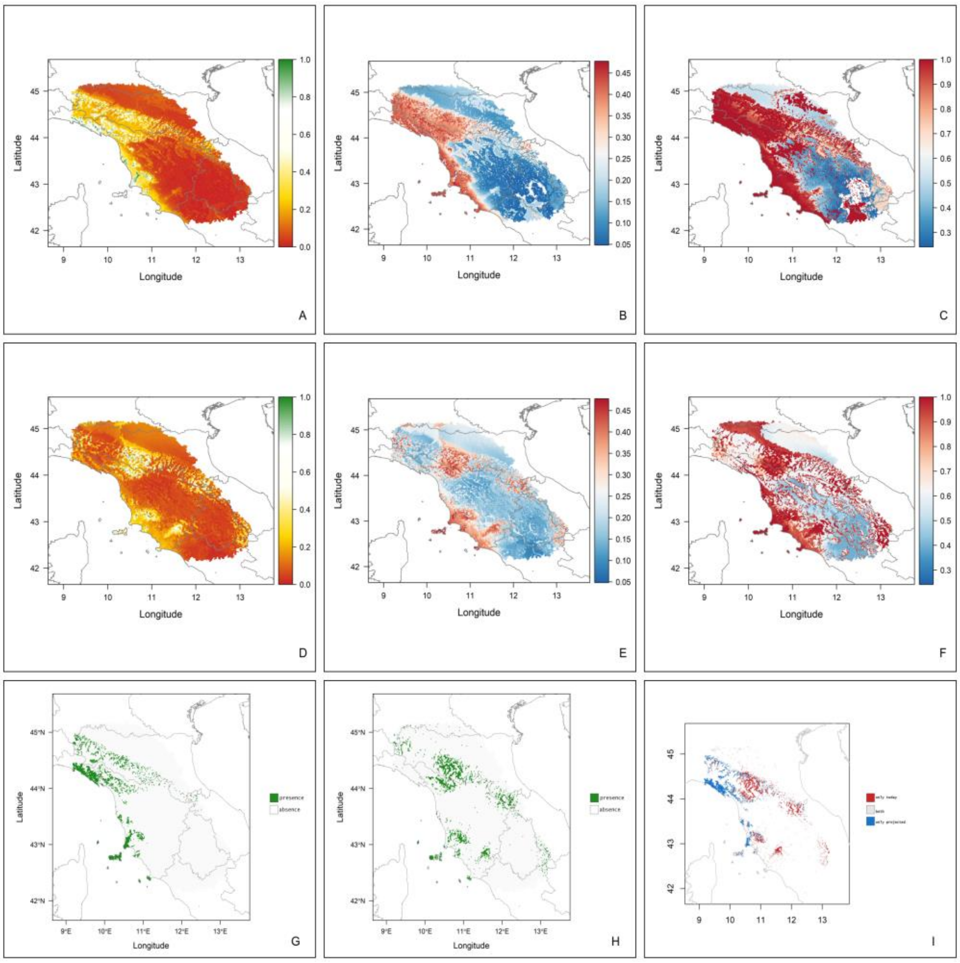

Fig. S.2.73-A) 'Projected' base output; B) 'Projected' uncertainty standard deviation; C) 'Projected' uncertainty range of values; D) 'Present' base output; E) 'Present' uncertainty standard deviation; F) 'Present' uncertainty range of values; G) 'Projected' binary distribution; H) 'Present' binary distribution; I) Range size variation.

Value	Present	Projected
AUC.train	1.000	1.000
AUC.test	0.988	0.905
AUC.diff	0.095	0.095
pROC	1.943	1.861
OR.p10	1.000	0.500
CBI	0.957	0.661
perc.stable	14.634	14.634
range.size	-0.643	-0.643

S.2.74 *Crocus etruscus* Bioclim

Species: *Crocus etruscus* Parl.

Procedure: Bioclim

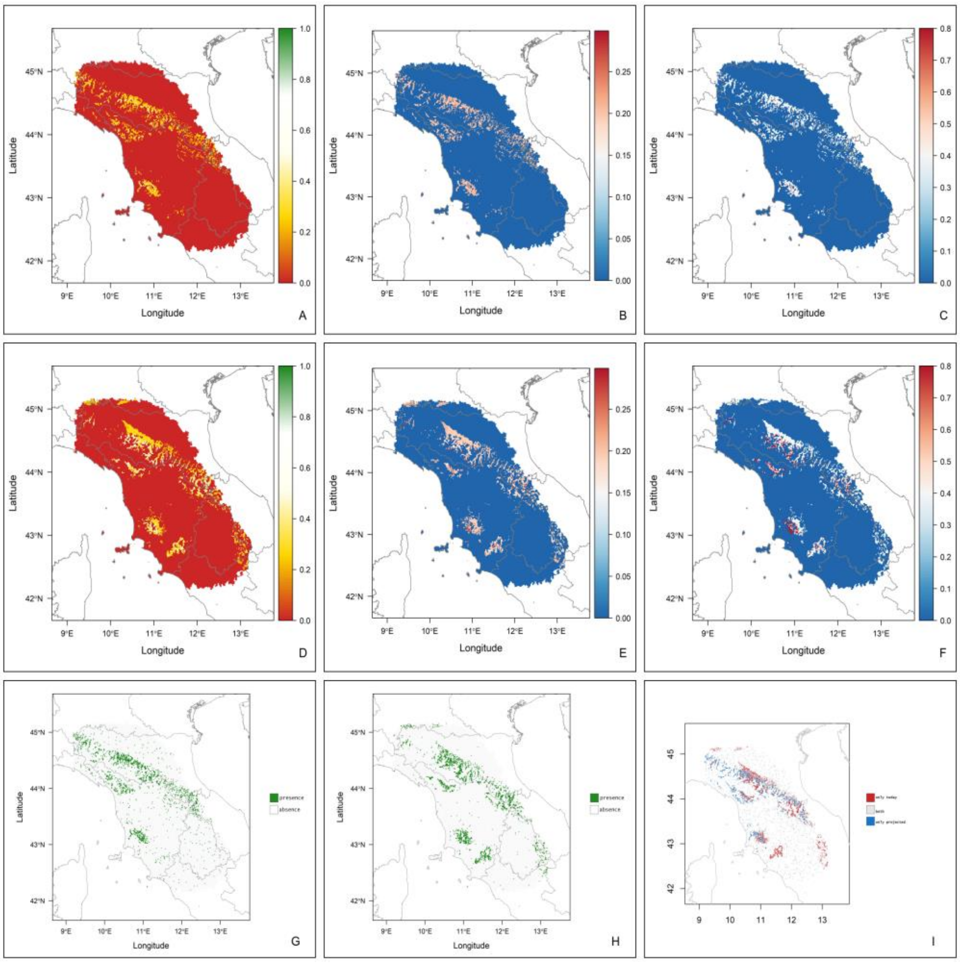

Fig. S.2.74-A) 'Projected' base output; B) 'Projected' uncertainty standard deviation; C) 'Projected' uncertainty range of values; D) 'Present' base output; E) 'Present' uncertainty standard deviation; F) 'Present' uncertainty range of values; G) 'Projected' binary distribution; H) 'Present' binary distribution; I) Range size variation.

Value	Present	Projected
AUC.train	0.954	0.973
AUC.test	0.693	0.607
AUC.diff	0.368	0.368
pROC	1.298	1.225
OR.p10	0.550	0.750
CBI	0.061	0.387
perc.stable	14.910	14.910
range.size	-2.735	-2.735

S.2.75 *Crocus etruscus* Domain

Species: *Crocus etruscus* Parl.

Procedure: Domain

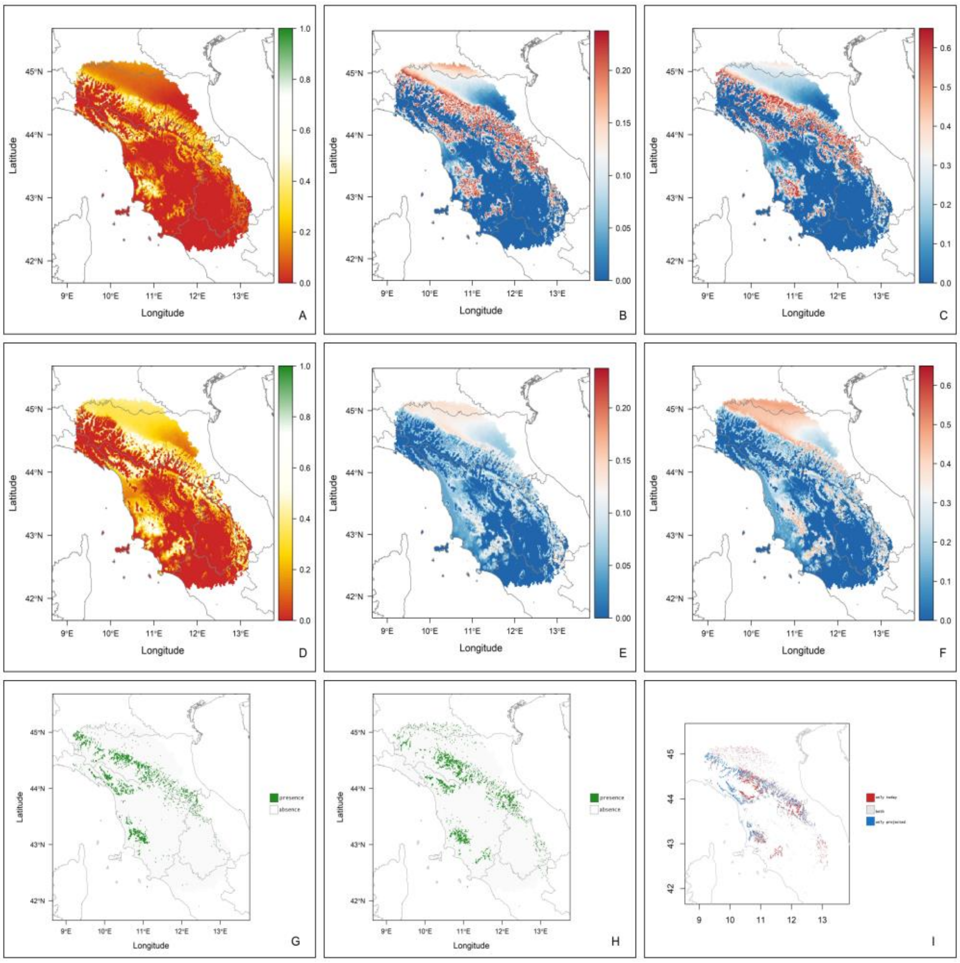

Fig. S.2.75-A) 'Projected' base output; B) 'Projected' uncertainty standard deviation; C) 'Projected' uncertainty range of values; D) 'Present' base output; E) 'Present' uncertainty standard deviation; F) 'Present' uncertainty range of values; G) 'Projected' binary distribution; H) 'Present' binary distribution; I) Range size variation.

Value	Present	Projected
AUC.train	0.916	0.962
AUC.test	0.865	0.656
AUC.diff	0.305	0.305
pROC	1.779	1.451
OR.p10	0.300	0.650
CBI	0.776	0.941
perc.stable	22.922	22.922
range.size	1.511	1.511

S.2.76 *Crocus etruscus* Maxent

Species: *Crocus etruscus* Parl.

Procedure: Maxent

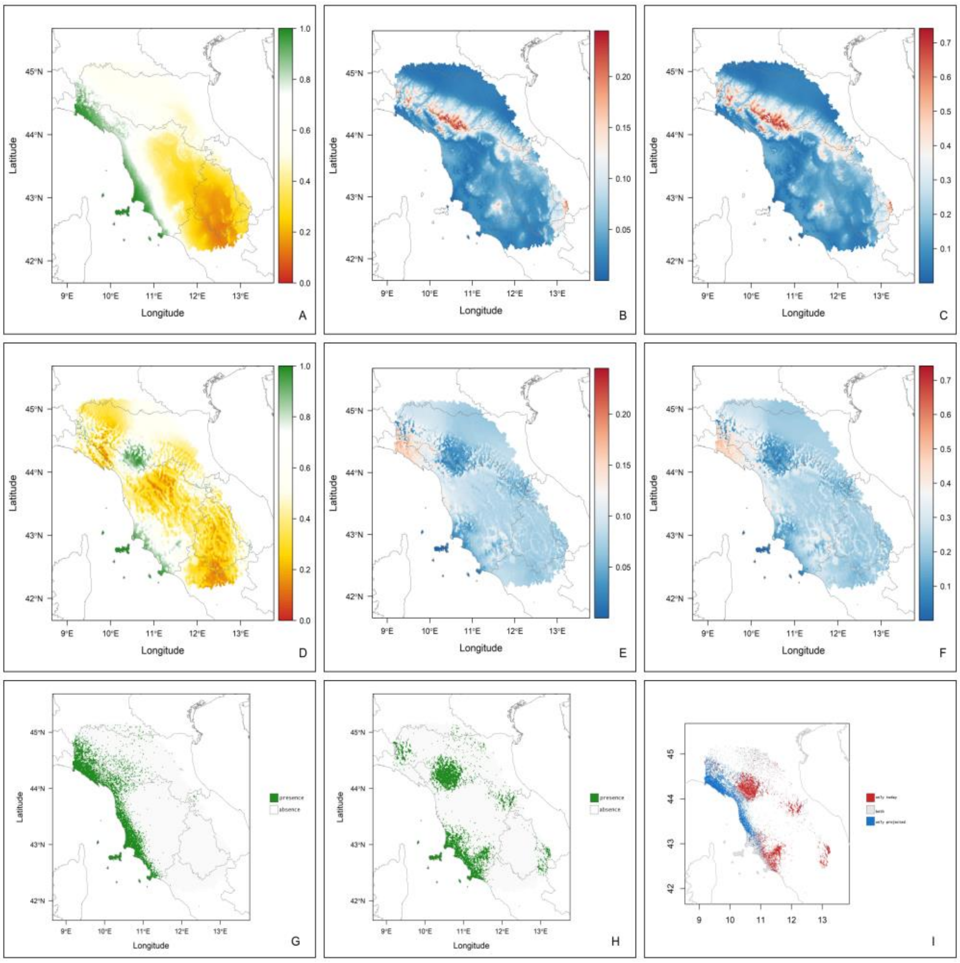

Fig. S.2.76-A) 'Projected' base output; B) 'Projected' uncertainty standard deviation; C) 'Projected' uncertainty range of values; D) 'Present' base output; E) 'Present' uncertainty standard deviation; F) 'Present' uncertainty range of values; G) 'Projected' binary distribution; H) 'Present' binary distribution; I) Range size variation.

Value	Present	Projected
AUC.train	0.903	0.932
AUC.test	0.842	0.903
AUC.diff	0.063	0.063
pROC	1.744	1.842
OR.p10	0.500	0.350
CBI	0.782	0.464
perc.stable	23.171	23.171
range.size	2.077	2.077

S.2.77 *Crocus etruscus* KDE

Species: *Crocus etruscus* Parl.

Procedure: KDE

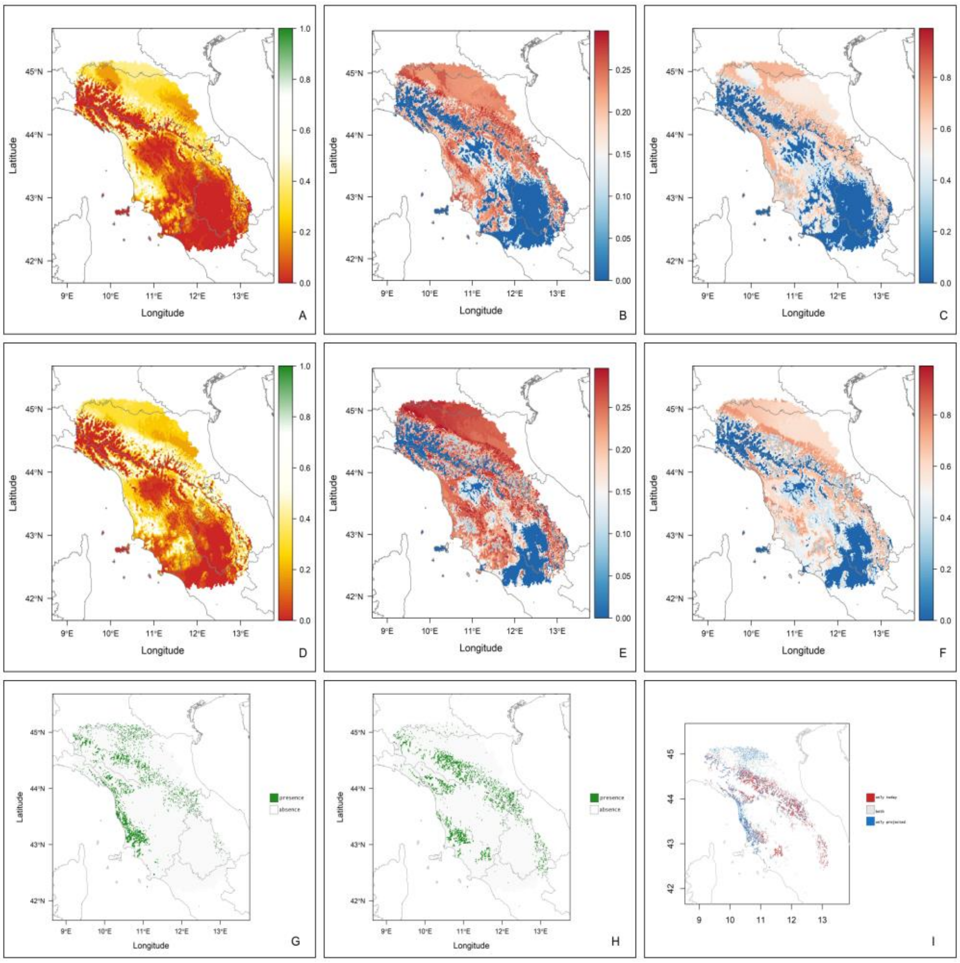

Fig. S.2.77-A) ‘Projected’ base output; B) ‘Projected’ uncertainty standard deviation; C) ‘Projected’ uncertainty range of values; D) ’Present’ base output; E) ’Present’ uncertainty standard deviation; F) ’Present’ uncertainty range of values; G) ‘Projected’ binary distribution; H) ‘Present’ binary distribution; I) Range size variation.

Value	Present	Projected
AUC.train	0.945	0.951
AUC.test	0.713	0.750
AUC.diff	0.234	0.201
pROC	1.537	1.592
OR.p10	0.750	0.650
CBI	0.714	0.644
perc.stable	21.106	21.106
range.size	-0.719	-0.719

S.2.78 *Crocus etruscus* CTA

Species: *Crocus etruscus* Parl.

Procedure: CTA

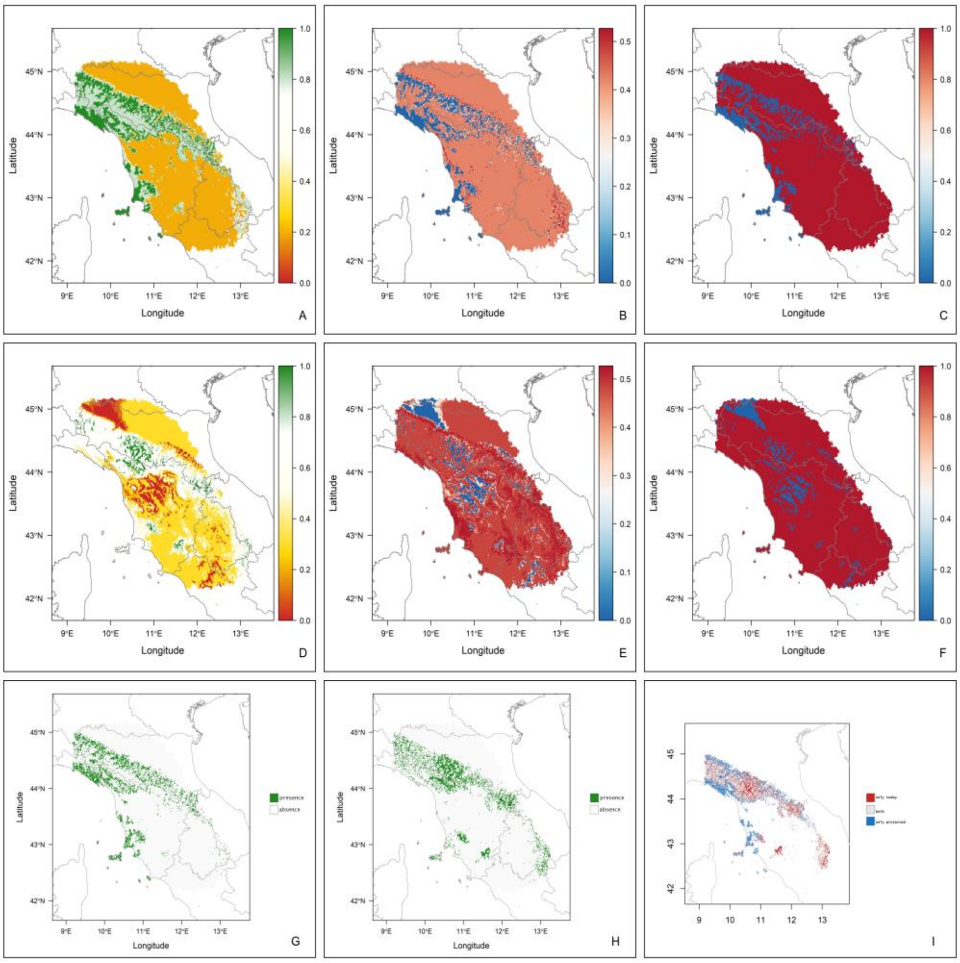

Fig. S.2.78-A) 'Projected' base output; B) 'Projected' uncertainty standard deviation; C) 'Projected' uncertainty range of values; D) 'Present' base output; E) 'Present' uncertainty standard deviation; F) 'Present' uncertainty range of values; G) 'Projected' binary distribution; H) 'Present' binary distribution; I) Range size variation.

Value	Present	Projected
AUC.train	0.787	0.921
AUC.test	0.657	0.871
AUC.diff	0.050	0.050
pROC	1.000	1.000
OR.p10	0.400	0.100
CBI	0.933	0.220
perc.stable	18.983	18.983
range.size	-0.137	-0.137

S.2.79 *Crocus etruscus* FDA

Species: *Crocus etruscus* Parl.

Procedure: FDA

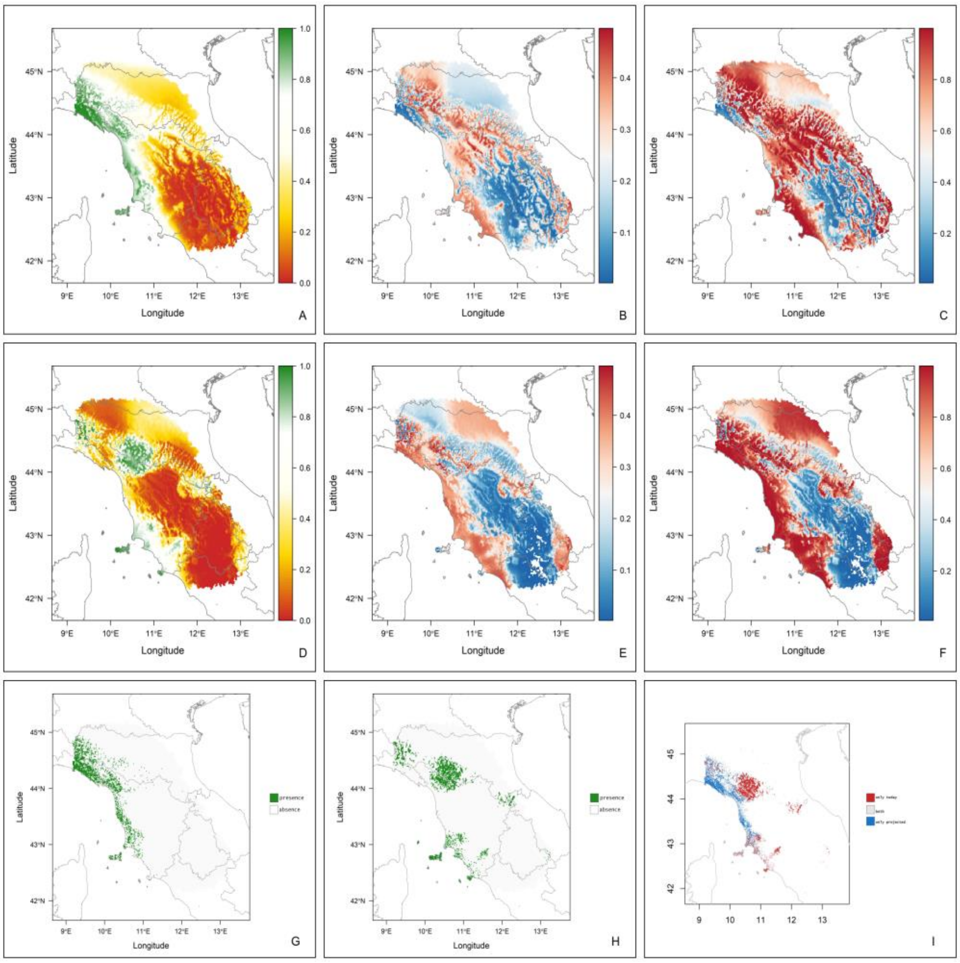

Fig. S.2.79-A) 'Projected' base output; B) 'Projected' uncertainty standard deviation; C) 'Projected' uncertainty range of values; D) 'Present' base output; E) 'Present' uncertainty standard deviation; F) 'Present' uncertainty range of values; G) 'Projected' binary distribution; H) 'Present' binary distribution; I) Range size variation.

Value	Present	Projected
AUC.train	0.966	0.937
AUC.test	0.843	0.914
AUC.diff	0.067	0.067
pROC	1.599	1.699
OR.p10	0.500	0.400
CBI	0.895	0.479
perc.stable	12.192	12.192
range.size	0.254	0.254

S.2.80 *Crocus etruscus* GAM

Species: *Crocus etruscus* Parl.

Procedure: GAM

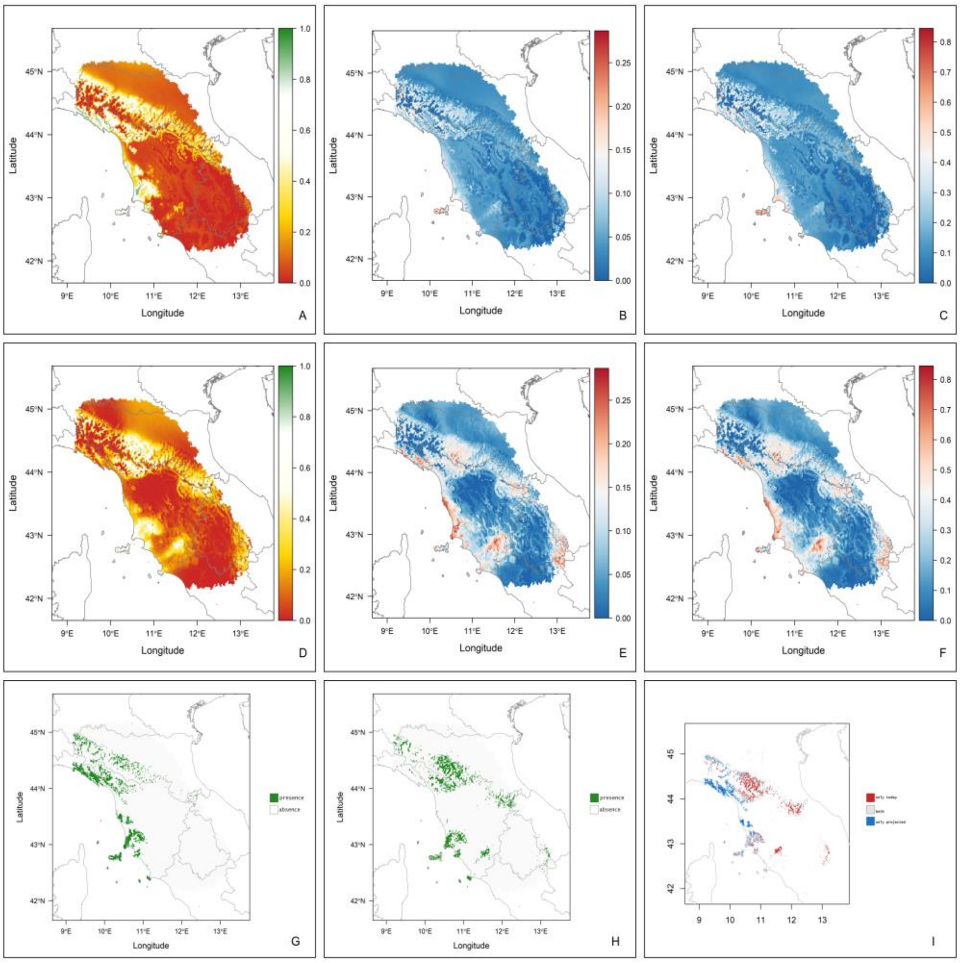

Fig. S.2.80-A) 'Projected' base output; B) 'Projected' uncertainty standard deviation; C) 'Projected' uncertainty range of values; D) 'Present' base output; E) 'Present' uncertainty standard deviation; F) 'Present' uncertainty range of values; G) 'Projected' binary distribution; H) 'Present' binary distribution; I) Range size variation.

Value	Present	Projected
AUC.train	0.957	0.977
AUC.test	0.802	0.940
AUC.diff	0.044	0.044
pROC	1.709	1.887
OR.p10	0.750	0.650
CBI	0.847	0.899
perc.stable	26.866	26.866
range.size	2.750	2.750

S.2.81 *Crocus etruscus* GLM

Species: *Crocus etruscus* Parl.

Procedure: GLM

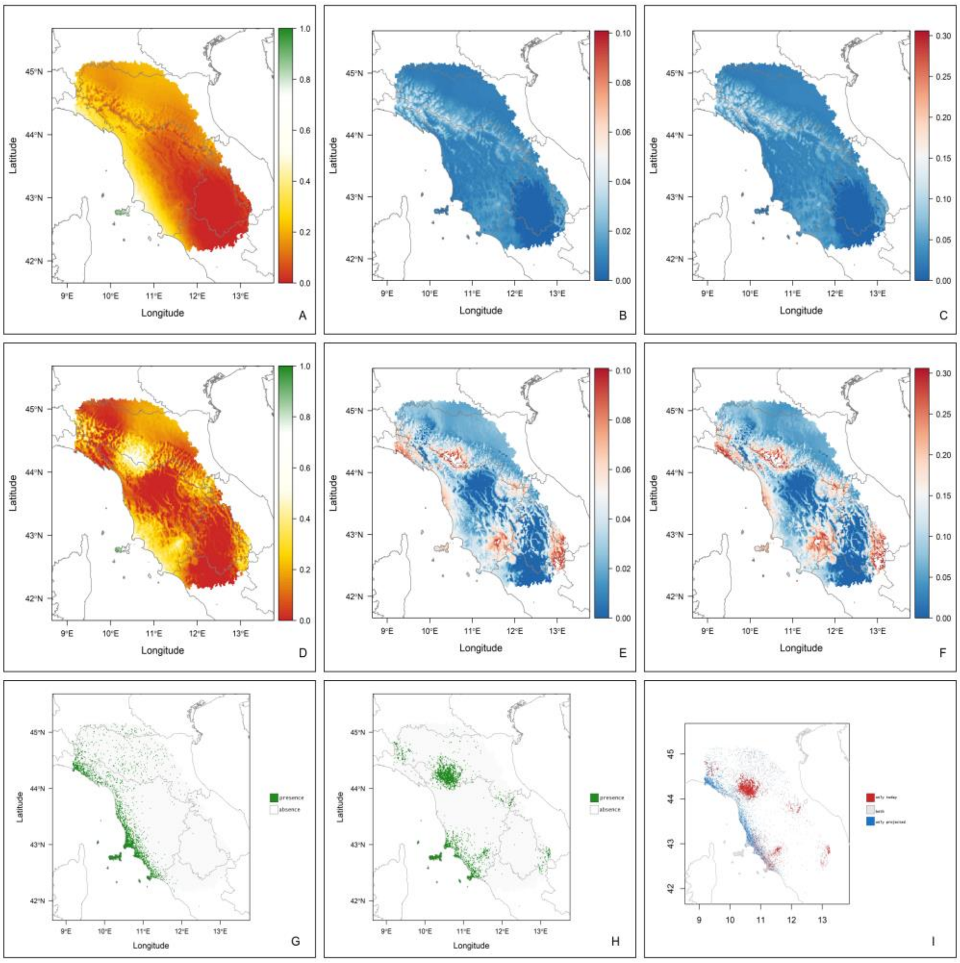

Fig. S.2.81-A) 'Projected' base output; B) 'Projected' uncertainty standard deviation; C) 'Projected' uncertainty range of values; D) 'Present' base output; E) 'Present' uncertainty standard deviation; F) 'Present' uncertainty range of values; G) 'Projected' binary distribution; H) 'Present' binary distribution; I) Range size variation.

Value	Present	Projected
AUC.train	0.884	0.924
AUC.test	0.881	0.917
AUC.diff	0.046	0.046
pROC	1.800	1.867
OR.p10	0.250	0.250
CBI	0.712	0.800
perc.stable	16.513	16.513
range.size	1.216	1.216

S.2.82 *Crocus etruscus* MARS

Species: *Crocus etruscus* Parl.

Procedure: MARS

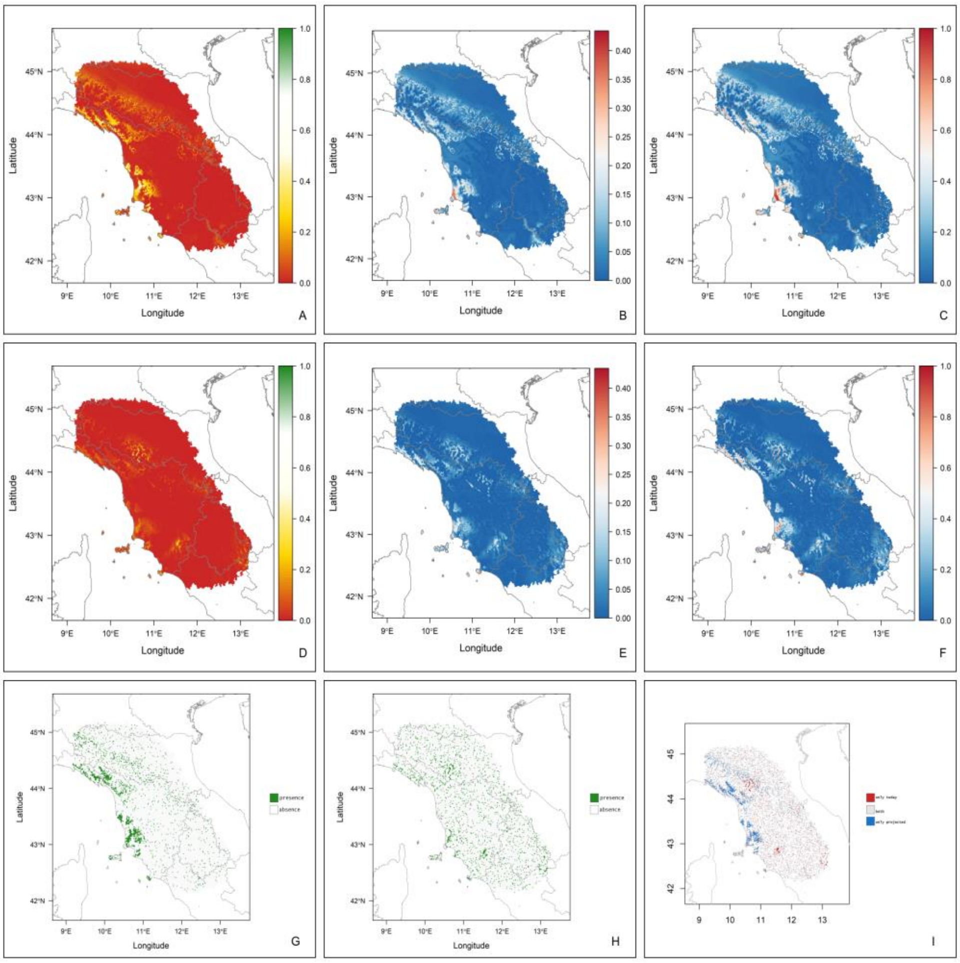

Fig. S.2.82-A) 'Projected' base output; B) 'Projected' uncertainty standard deviation; C) 'Projected' uncertainty range of values; D) 'Present' base output; E) 'Present' uncertainty standard deviation; F) 'Present' uncertainty range of values; G) 'Projected' binary distribution; H) 'Present' binary distribution; I) Range size variation.

Value	Present	Projected
AUC.train	0.915	0.943
AUC.test	0.825	0.822
AUC.diff	0.127	0.127
pROC	1.694	1.707
OR.p10	0.600	0.350
CBI	0.799	0.697
perc.stable	6.463	6.463
range.size	-0.114	-0.114

S.2.83 *Crocus etruscus* RF

Species: *Crocus etruscus* Parl.

Procedure: RF

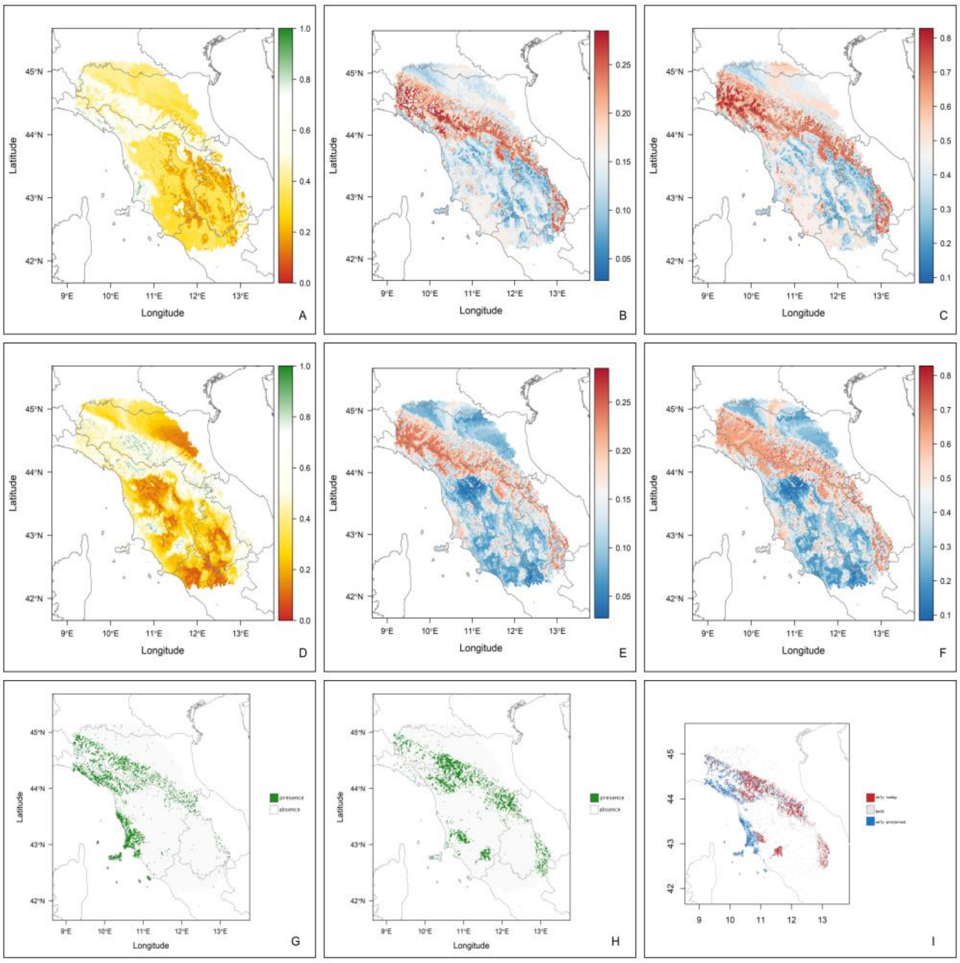

Fig. S.2.83-A) ‘Projected’ base output; B) ‘Projected’ uncertainty standard deviation; C) ‘Projected’ uncertainty range of values; D) ’Present’ base output; E) ’Present’ uncertainty standard deviation; F) ’Present’ uncertainty range of values; G) ‘Projected’ binary distribution; H) ‘Present’ binary distribution; I) Range size variation.

Value	Present	Projected
AUC.train	1.000	1.000
AUC.test	1.000	0.943
AUC.diff	0.057	0.057
pROC	1.810	1.729
OR.p10	0.750	0.750
CBI	0.921	0.642
perc.stable	15.043	15.043
range.size	-0.135	-0.135

S.2.84 *Crocus etruscus* SVM

Species: *Crocus etruscus* Parl.

Procedure: SVM

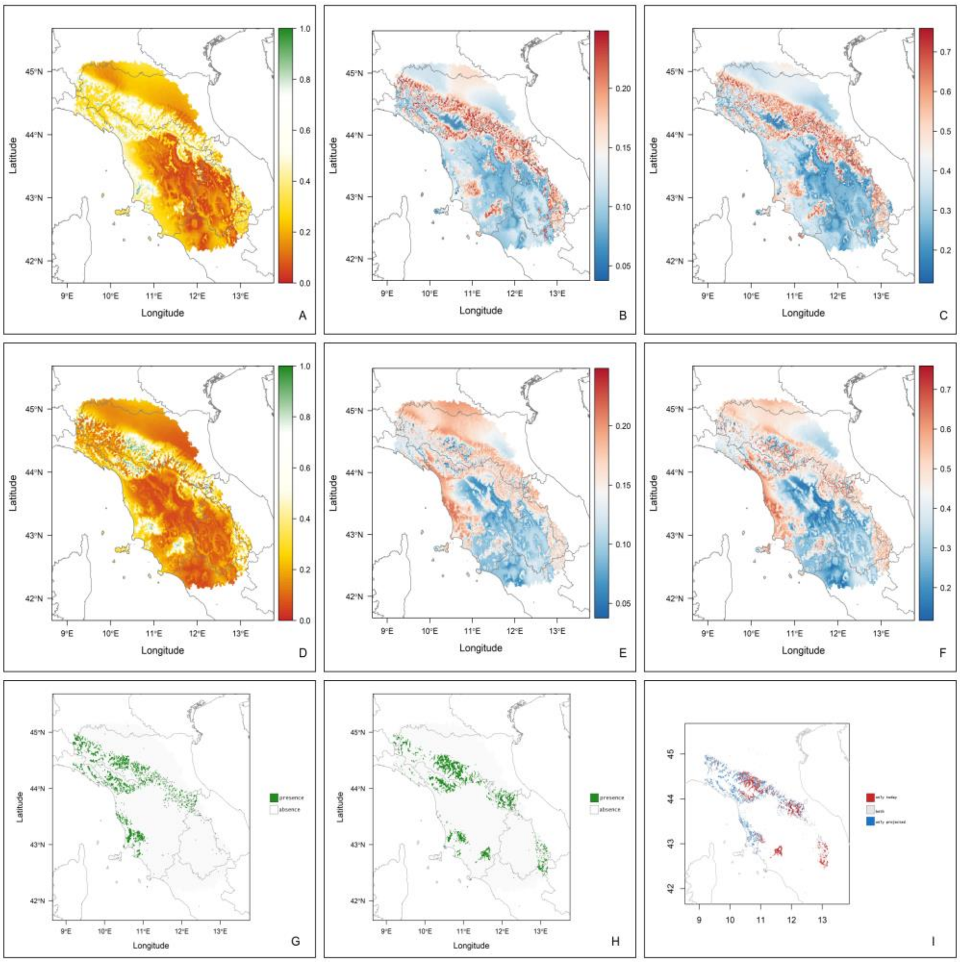

Fig. S.2.84-A) ‘Projected’ base output; B) ‘Projected’ uncertainty standard deviation; C) ‘Projected’ uncertainty range of values; D) ’Present’ base output; E) ’Present’ uncertainty standard deviation; F) ’Present’ uncertainty range of values; G) ‘Projected’ binary distribution; H) ‘Present’ binary distribution; I) Range size variation.

Value	Present	Projected
AUC.train	0.989	0.991
AUC.test	0.971	0.950
AUC.diff	0.047	0.047
pROC	1.753	1.780
OR.p10	0.750	0.500
CBI	0.915	0.933
perc.stable	24.572	24.572
range.size	-0.222	-0.222

S.2.85 *Crocus imperati* Biomod2

Species: *Crocus imperati* Ten.

Procedure: Biomod2

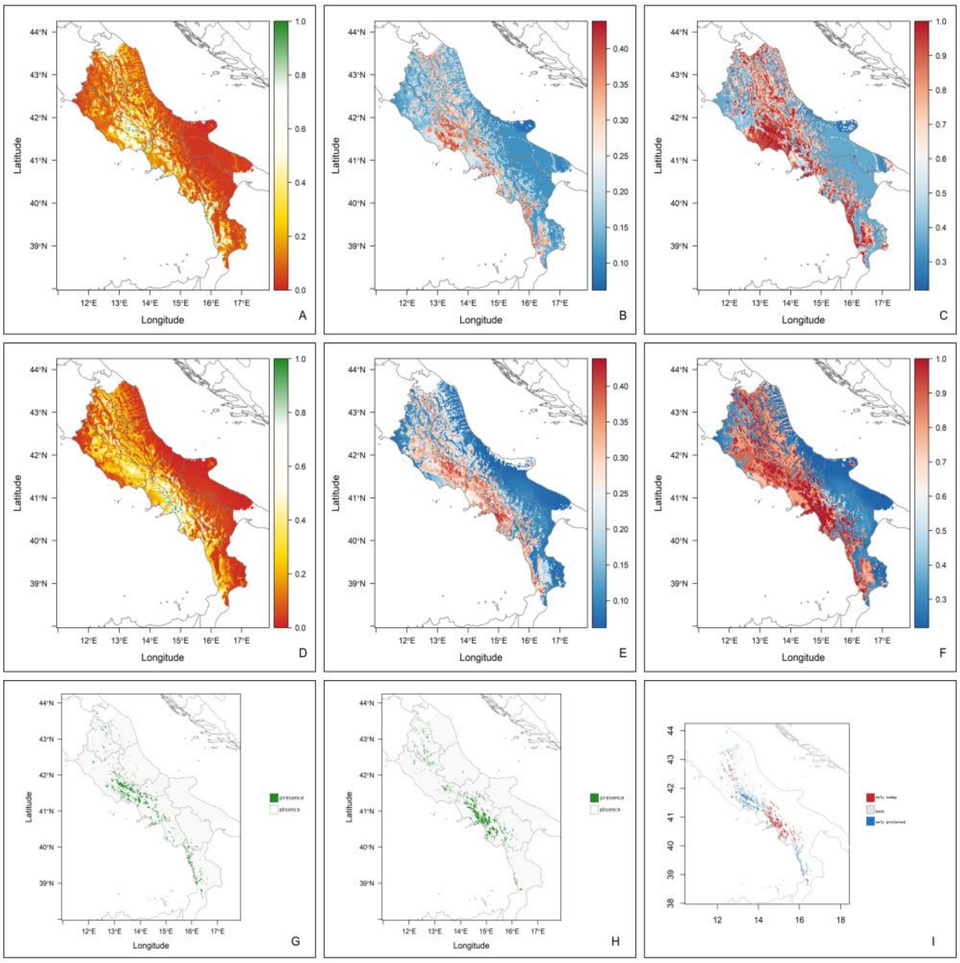

Fig. S.2.85-A) 'Projected' base output; B) 'Projected' uncertainty standard deviation; C) 'Projected' uncertainty range of values; D) 'Present' base output; E) 'Present' uncertainty standard deviation; F) 'Present' uncertainty range of values; G) 'Projected' binary distribution; H) 'Present' binary distribution; I) Range size variation.

Value	Present	Projected
AUC.train	0.998	0.997
AUC.test	0.858	0.747
AUC.diff	0.250	0.250
pROC	1.615	1.430
OR.p10	0.817	1.000
CBI	0.914	0.820
perc.stable	13.881	13.881
range.size	-1.786	-1.786

S.2.86 *Crocus imperati* Bioclim

Species: *Crocus imperati* Ten.

Procedure: Bioclim

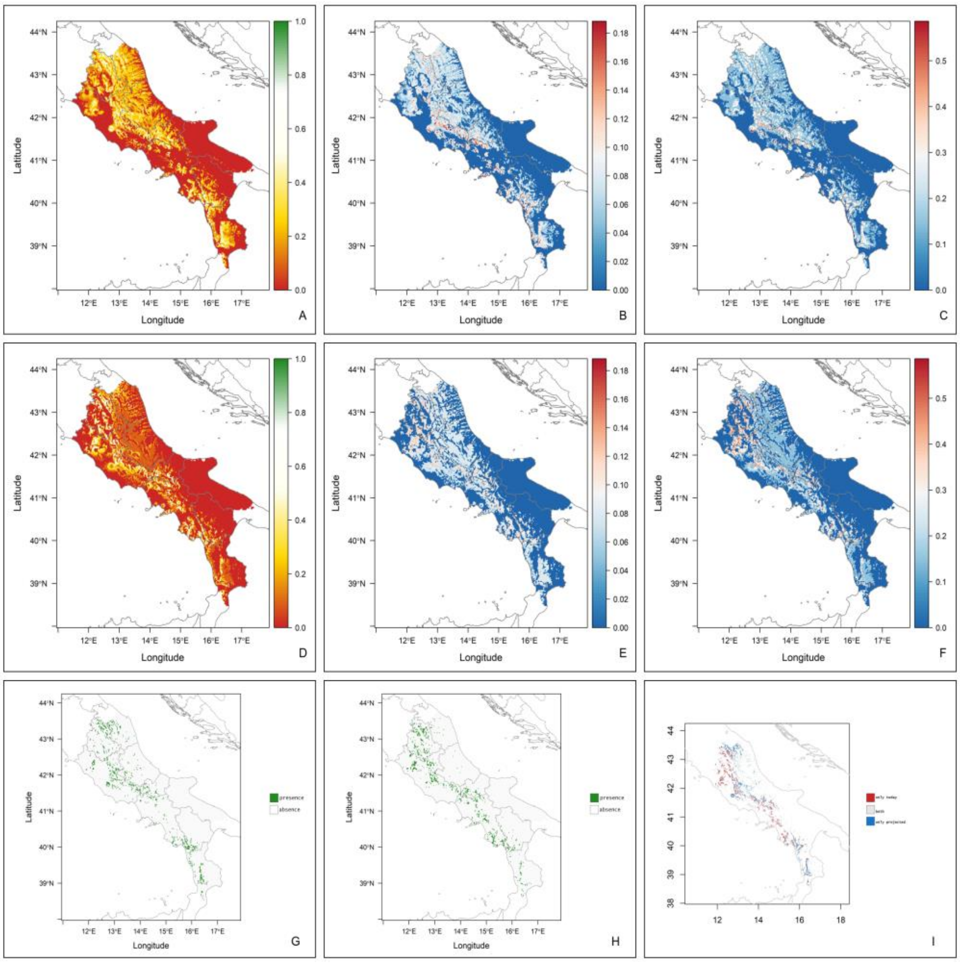

Fig. S.2.86-A) 'Projected' base output; B) 'Projected' uncertainty standard deviation; C) 'Projected' uncertainty range of values; D) 'Present' base output; E) 'Present' uncertainty standard deviation; F) 'Present' uncertainty range of values; G) 'Projected' binary distribution; H) 'Present' binary distribution; I) Range size variation.

Value	Present	Projected
AUC.train	0.844	0.722
AUC.test	0.698	0.564
AUC.diff	0.160	0.160
pROC	1.243	1.156
OR.p10	0.303	0.283
CBI	0.367	0.606
perc.stable	15.375	15.375
range.size	1.594	1.594

S.2.87 *Crocus imperati* Domain

Species: *Crocus imperati* Ten.

Procedure: Domain

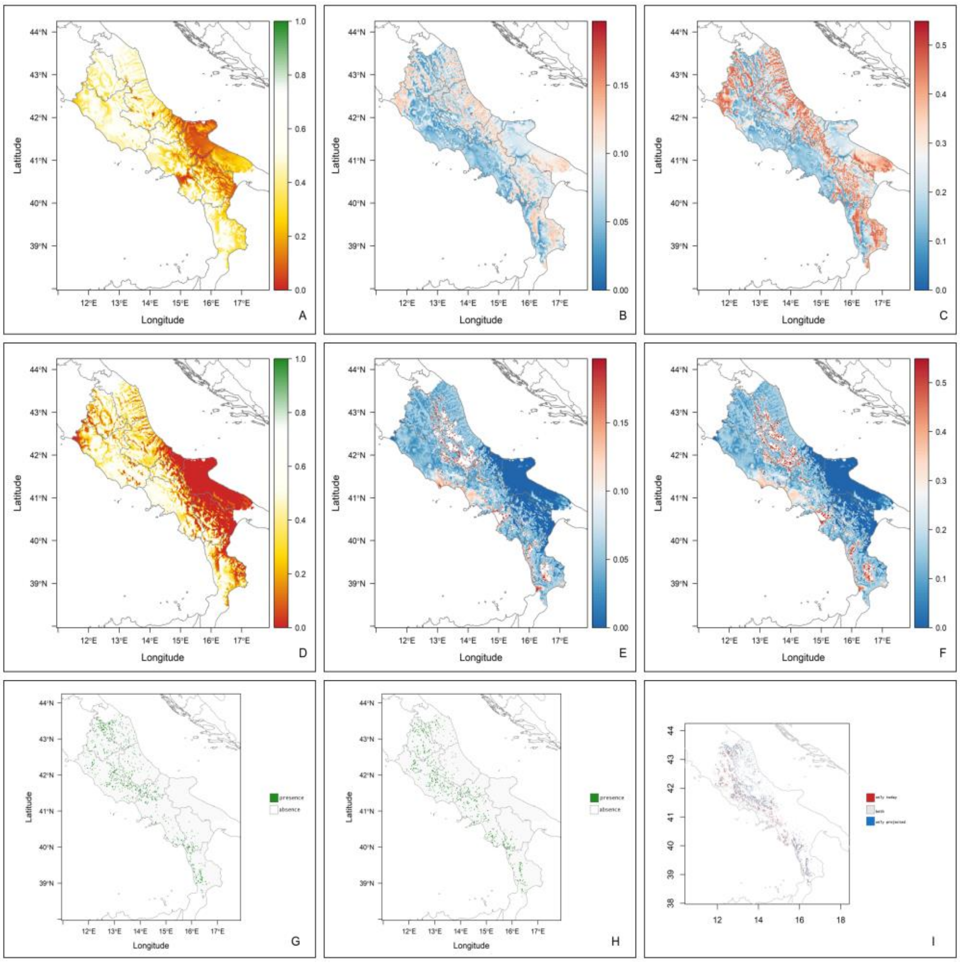

Fig. S.2.87-A) 'Projected' base output; B) 'Projected' uncertainty standard deviation; C) 'Projected' uncertainty range of values; D) 'Present' base output; E) 'Present' uncertainty standard deviation; F) 'Present' uncertainty range of values; G) 'Projected' binary distribution; H) 'Present' binary distribution; I) Range size variation.

Value	Present	Projected
AUC.train	0.818	0.720
AUC.test	0.747	0.608
AUC.diff	0.113	0.113
pROC	1.421	1.267
OR.p10	0.273	0.433
CBI	0.749	0.788
perc.stable	9.549	9.549
range.size	5.938	5.938

S.2.88 *Crocus imperati* Maxent

Species: *Crocus imperati* Ten.

Procedure: Maxent

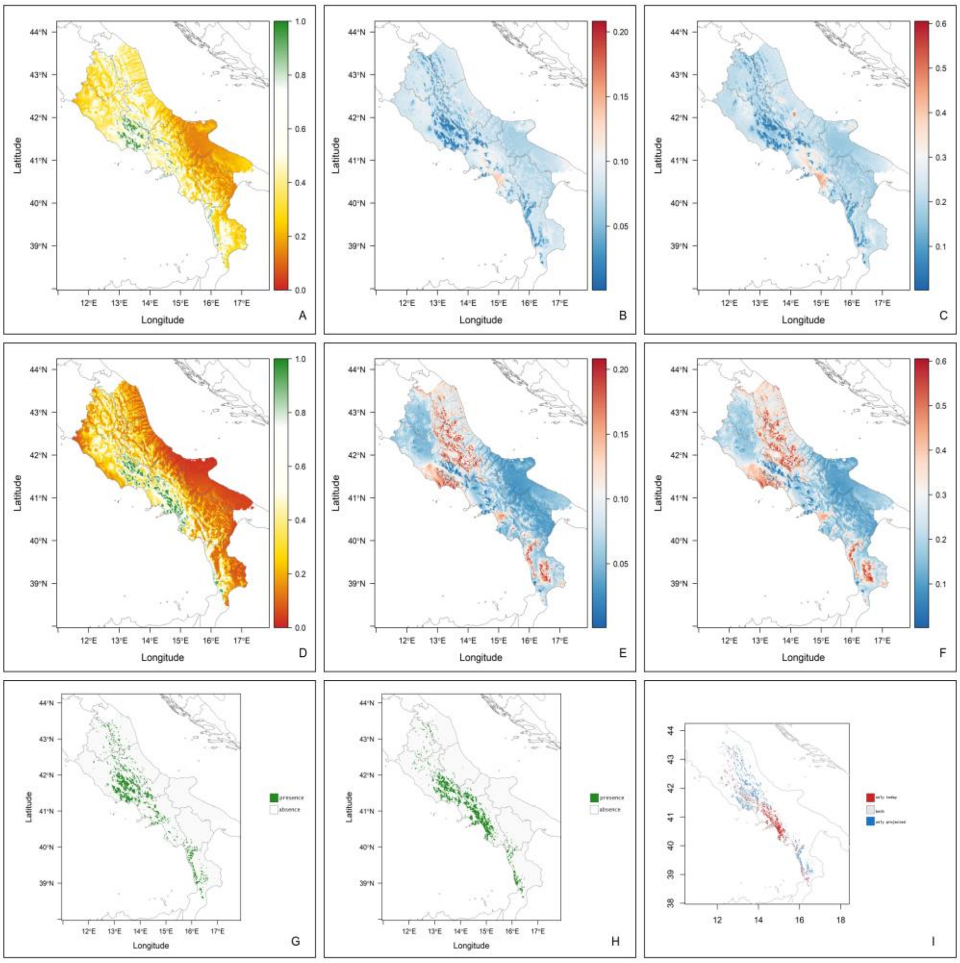

Fig. S.2.88-A) 'Projected' base output; B) 'Projected' uncertainty standard deviation; C) 'Projected' uncertainty range of values; D) 'Present' base output; E) 'Present' uncertainty standard deviation; F) 'Present' uncertainty range of values; G) 'Projected' binary distribution; H) 'Present' binary distribution; I) Range size variation.

Value	Present	Projected
AUC.train	0.865	0.799
AUC.test	0.787	0.788
AUC.diff	0.067	0.067
pROC	1.490	1.534
OR.p10	0.303	0.267
CBI	0.724	0.792
perc.stable	36.960	36.960
range.size	-1.059	-1.059

S.2.89 *Crocus imperati* KDE

Species: *Crocus imperati* Ten.

Procedure: KDE

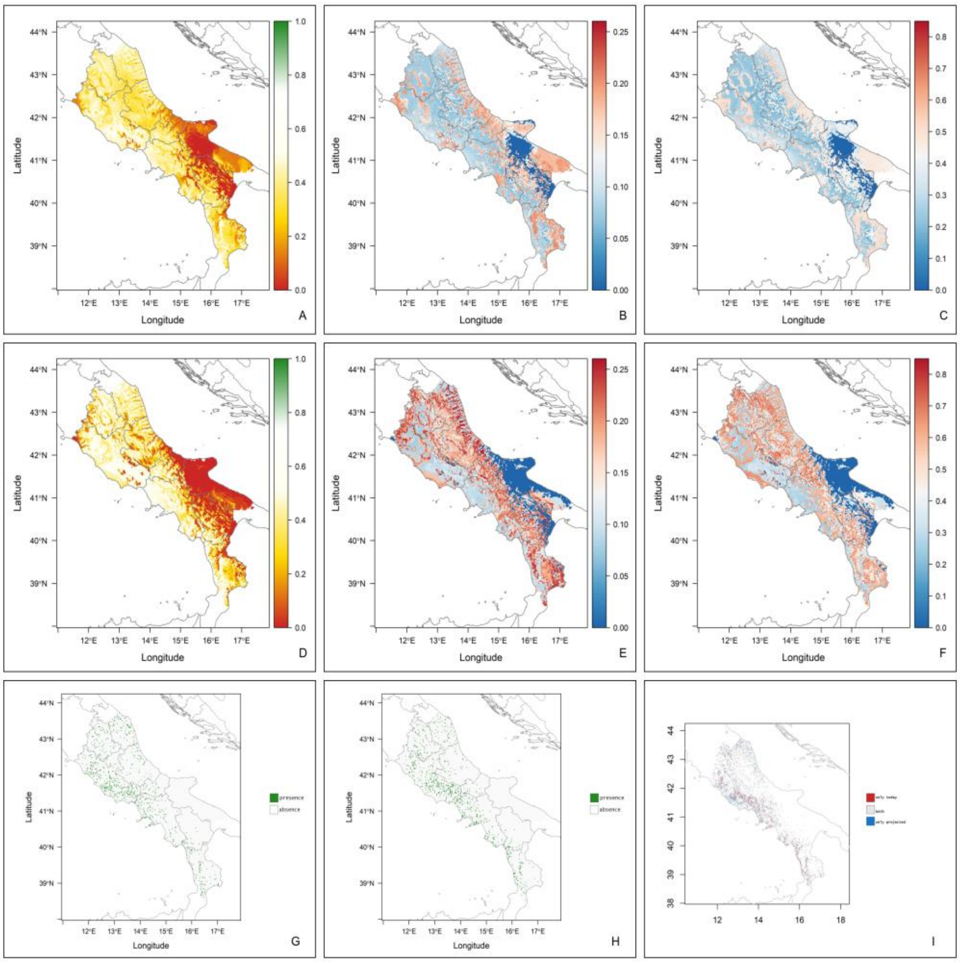

Fig. S.2.89-A) 'Projected' base output; B) 'Projected' uncertainty standard deviation; C) 'Projected' uncertainty range of values; D) 'Present' base output; E) 'Present' uncertainty standard deviation; F) 'Present' uncertainty range of values; G) 'Projected' binary distribution; H) 'Present' binary distribution; I) Range size variation.

Value	Present	Projected
AUC.train	0.819	0.776
AUC.test	0.740	0.599
AUC.diff	0.096	0.177
pROC	1.408	1.209
OR.p10	0.250	0.400
CBI	0.836	0.671
perc.stable	6.086	6.086
range.size	5.395	5.395

S.2.90 *Crocus imperati* CTA

Species: *Crocus imperati* Ten.

Procedure: CTA

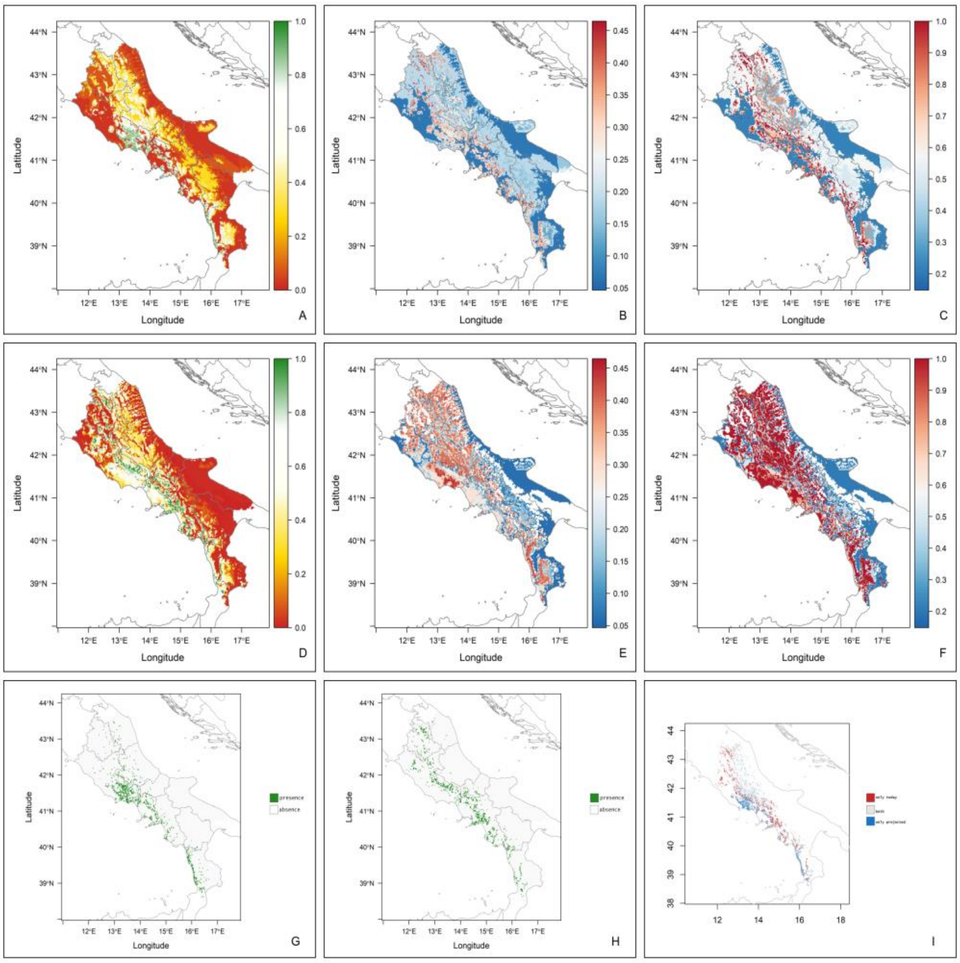

Fig. S.2.90-A) 'Projected' base output; B) 'Projected' uncertainty standard deviation; C) 'Projected' uncertainty range of values; D) 'Present' base output; E) 'Present' uncertainty standard deviation; F) 'Present' uncertainty range of values; G) 'Projected' binary distribution; H) 'Present' binary distribution; I) Range size variation.

Value	Present	Projected
AUC.train	0.959	0.911
AUC.test	0.864	0.823
AUC.diff	0.093	0.093
pROC	1.221	1.283
OR.p10	0.247	0.233
CBI	0.678	0.698
perc.stable	13.614	13.614
range.size	2.173	2.173

S.2.91 *Crocus imperati* FDA

Species: *Crocus imperati* Ten.

Procedure: FDA

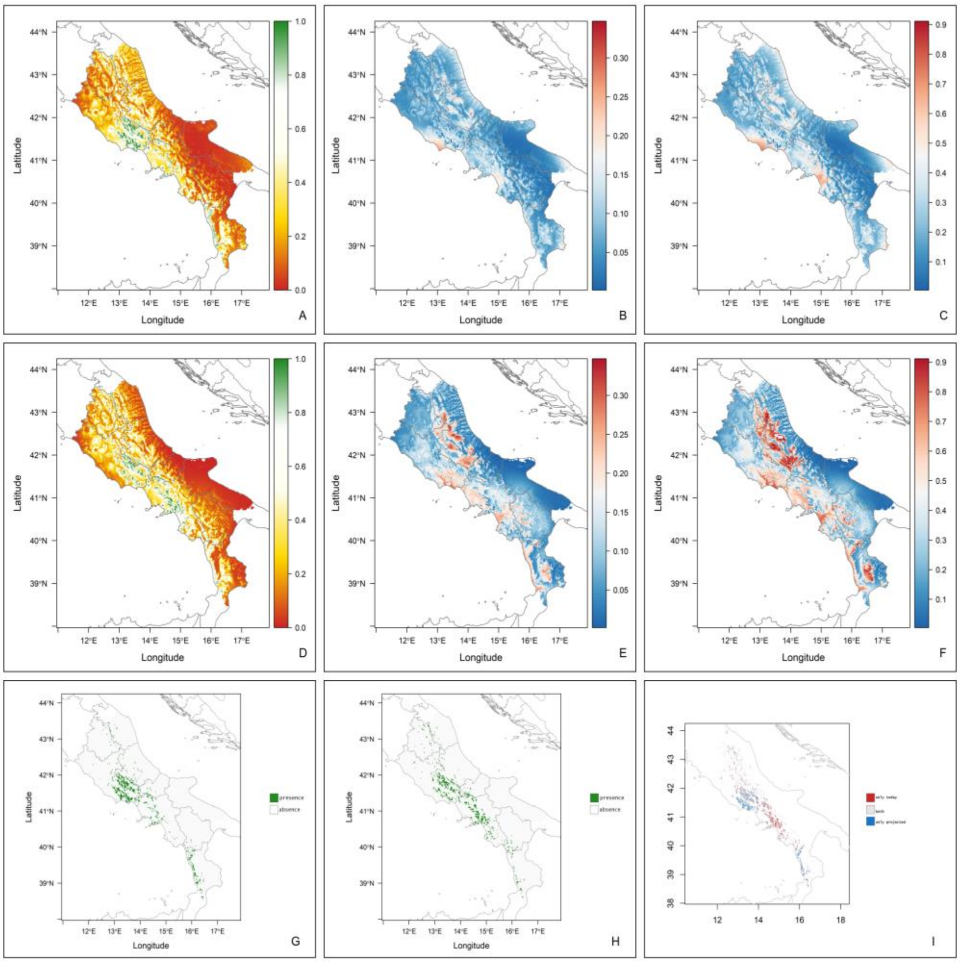

Fig. S.2.91-A) 'Projected' base output; B) 'Projected' uncertainty standard deviation; C) 'Projected' uncertainty range of values; D) 'Present' base output; E) 'Present' uncertainty standard deviation; F) 'Present' uncertainty range of values; G) 'Projected' binary distribution; H) 'Present' binary distribution; I) Range size variation.

Value	Present	Projected
AUC.train	0.874	0.844
AUC.test	0.819	0.780
AUC.diff	0.082	0.082
pROC	1.508	1.438
OR.p10	0.313	0.250
CBI	0.604	0.750
perc.stable	30.959	30.959
range.size	0.150	0.150

S.2.92 *Crocus imperati* GAM

Species: *Crocus imperati* Ten.

Procedure: GAM

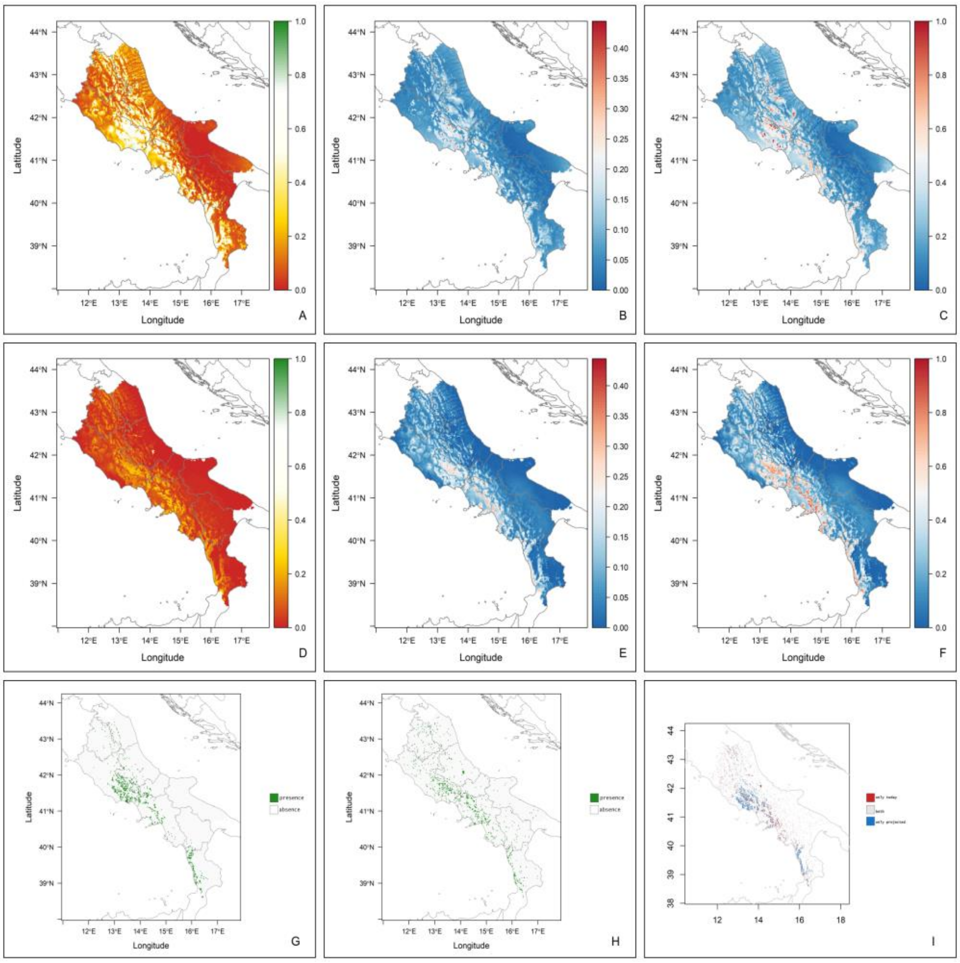

Fig. S.2.92-A) 'Projected' base output; B) 'Projected' uncertainty standard deviation; C) 'Projected' uncertainty range of values; D) 'Present' base output; E) 'Present' uncertainty standard deviation; F) 'Present' uncertainty range of values; G) 'Projected' binary distribution; H) 'Present' binary distribution; I) Range size variation.

Value	Present	Projected
AUC.train	0.912	0.800
AUC.test	0.788	0.781
AUC.diff	0.053	0.053
pROC	1.466	1.529
OR.p10	0.387	0.133
CBI	0.835	0.775
perc.stable	12.454	12.454
range.size	6.644	6.644

S.2.93 *Crocus imperati* GLM

Species: *Crocus imperati* Ten.

Procedure: GLM

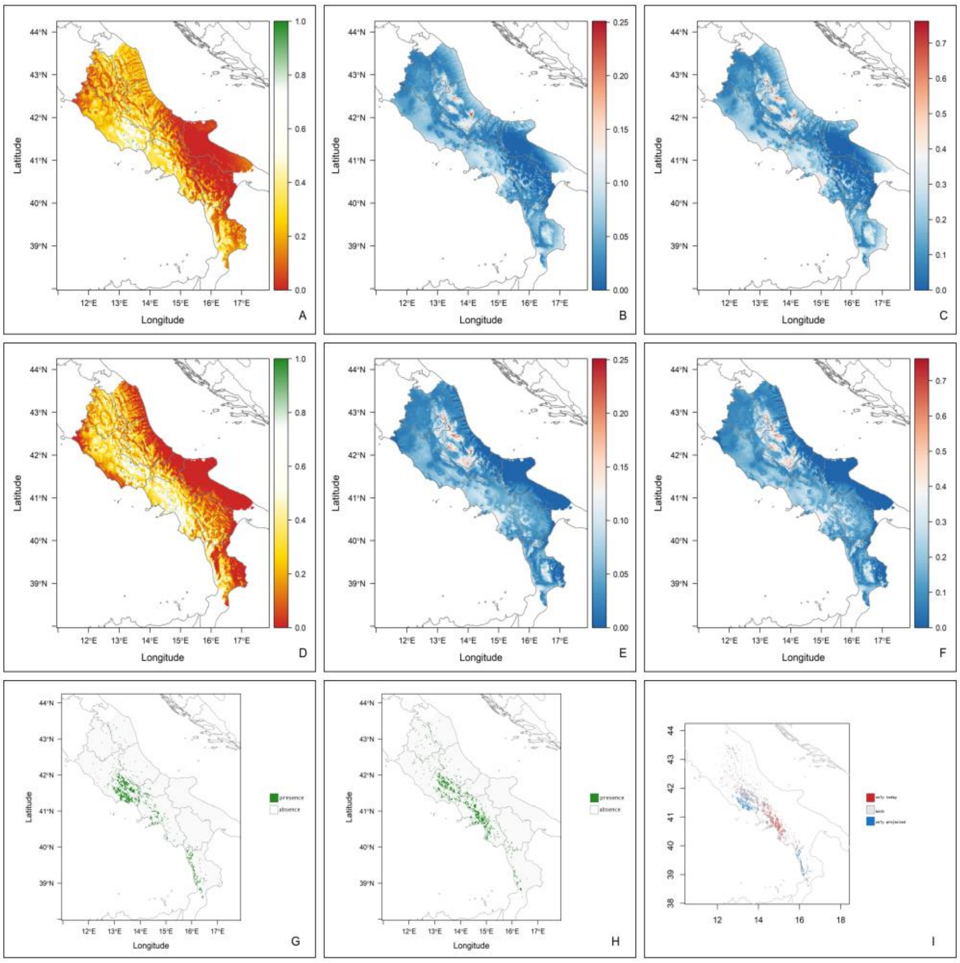

Fig. S.2.93-A) 'Projected' base output; B) 'Projected' uncertainty standard deviation; C) 'Projected' uncertainty range of values; D) 'Present' base output; E) 'Present' uncertainty standard deviation; F) 'Present' uncertainty range of values; G) 'Projected' binary distribution; H) 'Present' binary distribution; I) Range size variation.

Value	Present	Projected
AUC.train	0.872	0.788
AUC.test	0.801	0.780
AUC.diff	0.043	0.043
pROC	1.524	1.527
OR.p10	0.377	0.183
CBI	0.834	0.824
perc.stable	26.266	26.266
range.size	0.802	0.802

S.2.94 *Crocus imperati* MARS

Species: *Crocus imperati* Ten.

Procedure: MARS

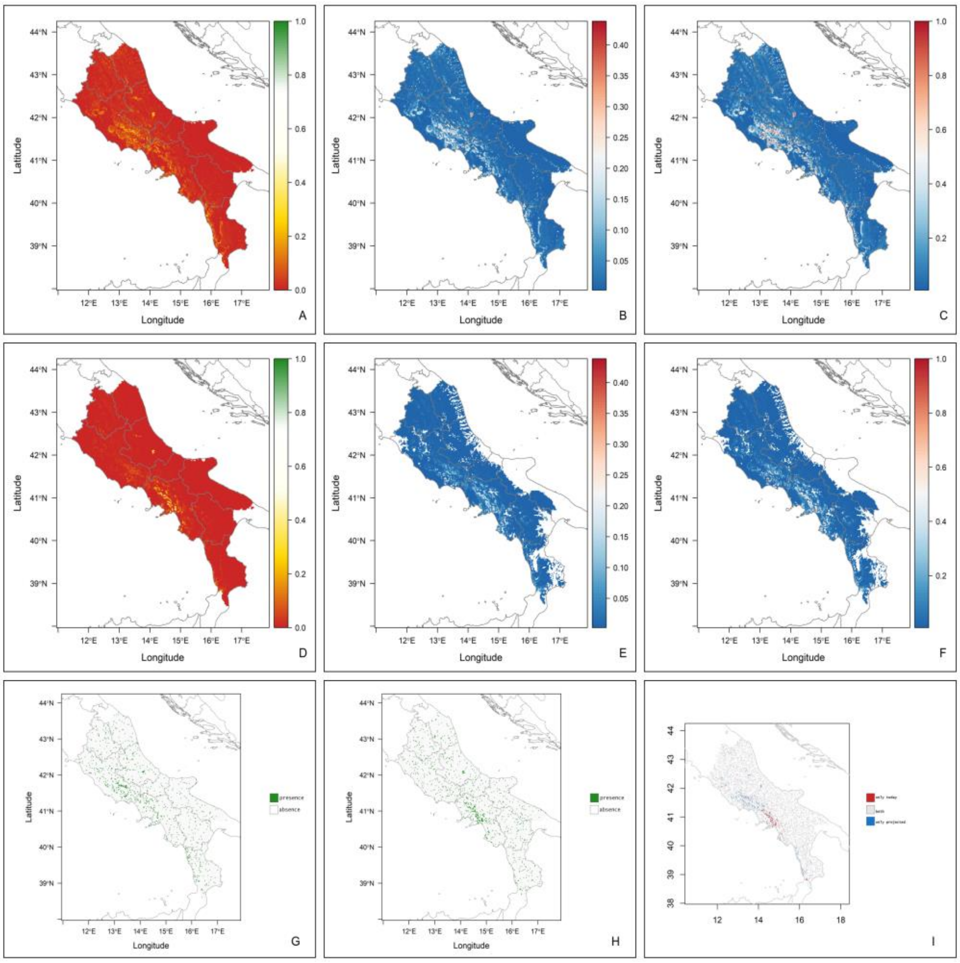

Fig. S.2.94-A) 'Projected' base output; B) 'Projected' uncertainty standard deviation; C) 'Projected' uncertainty range of values; D) 'Present' base output; E) 'Present' uncertainty standard deviation; F) 'Present' uncertainty range of values; G) 'Projected' binary distribution; H) 'Present' binary distribution; I) Range size variation.

Value	Present	Projected
AUC.train	0.812	0.818
AUC.test	0.690	0.672
AUC.diff	0.156	0.156
pROC	1.184	1.290
OR.p10	0.207	0.250
CBI	0.798	0.739
perc.stable	3.607	3.607
range.size	-1.620	-1.620

S.2.95 *Crocus imperati* RF

Species: *Crocus imperati* Ten.

Procedure: RF

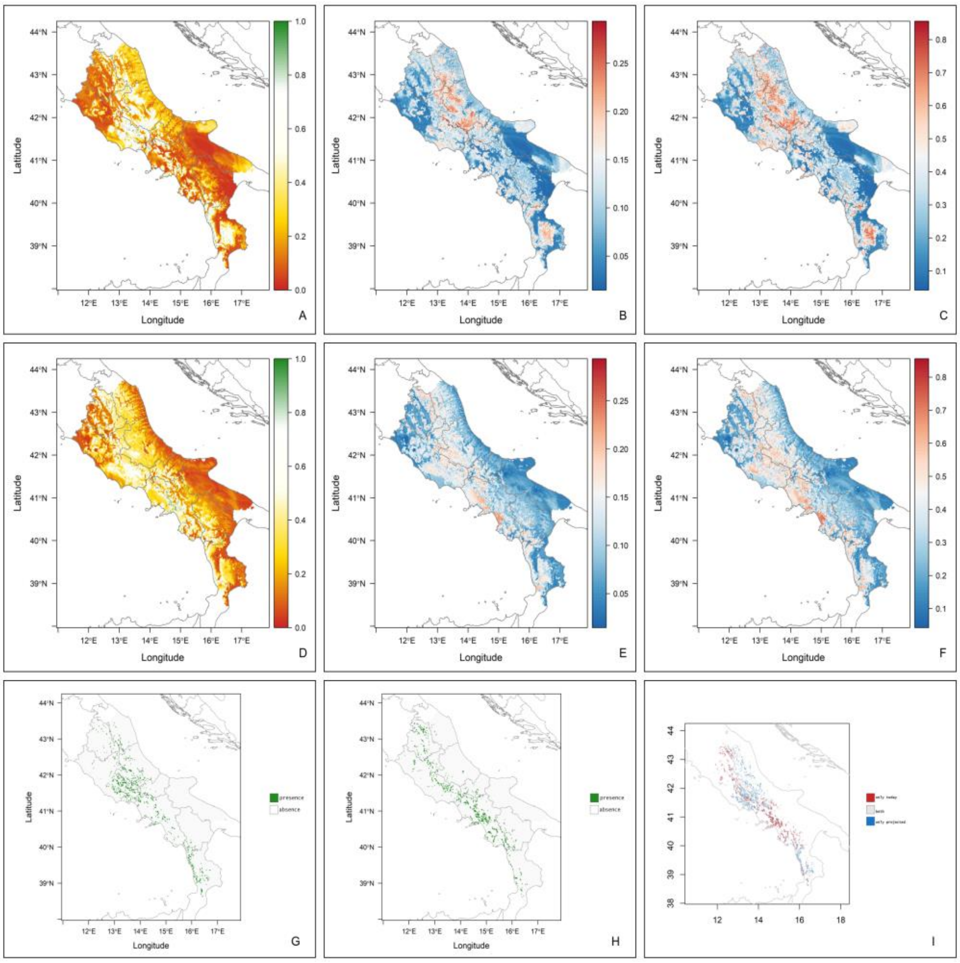

Fig. S.2.95-A) ‘Projected’ base output; B) ‘Projected’ uncertainty standard deviation; C) ‘Projected’ uncertainty range of values; D) ’Present’ base output; E) ’Present’ uncertainty standard deviation; F) ’Present’ uncertainty range of values; G) ‘Projected’ binary distribution; H) ‘Present’ binary distribution; I) Range size variation.

Value	Present	Projected
AUC.train	1.000	1.000
AUC.test	0.973	0.868
AUC.diff	0.132	0.132
pROC	1.570	1.344
OR.p10	0.797	0.767
CBI	0.952	0.777
perc.stable	9.452	9.452
range.size	-3.191	-3.191

S.2.96 *Crocus imperati* SVM

Species: *Crocus imperati* Ten.

Procedure: SVM

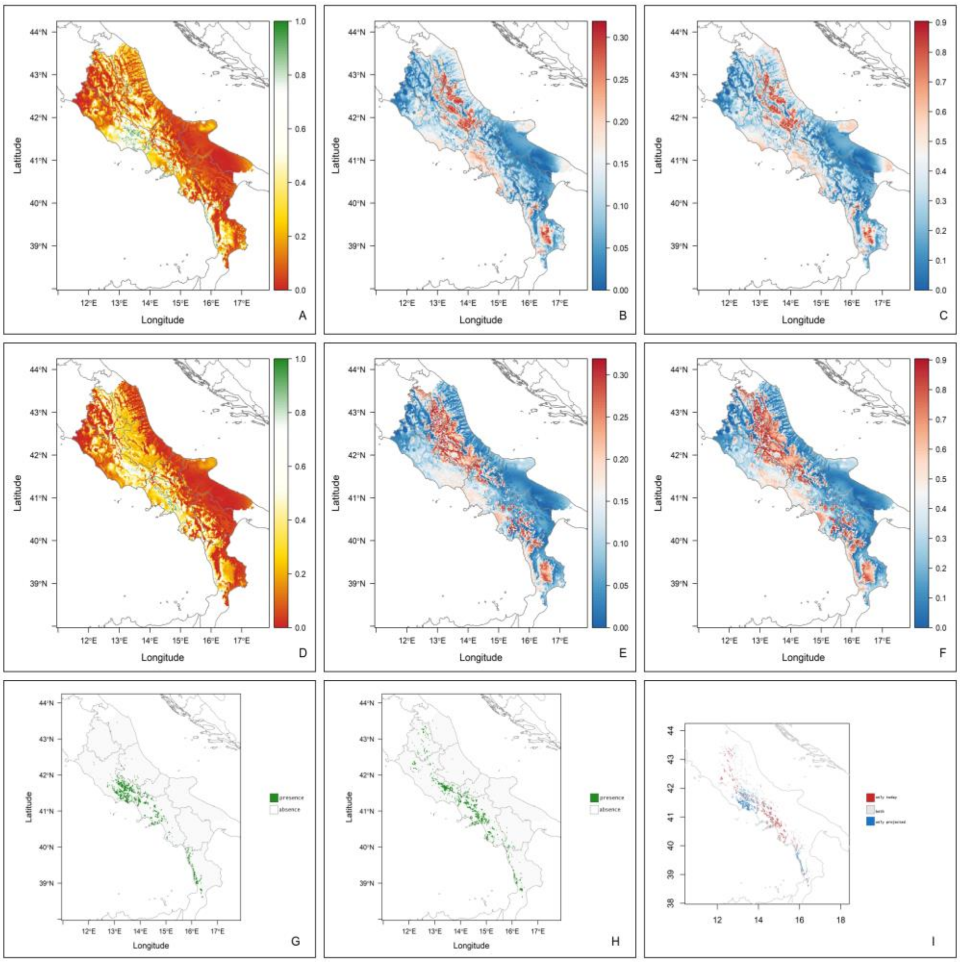

Fig. S.2.96-A) 'Projected' base output; B) 'Projected' uncertainty standard deviation; C) 'Projected' uncertainty range of values; D) 'Present' base output; E) 'Present' uncertainty standard deviation; F) 'Present' uncertainty range of values; G) 'Projected' binary distribution; H) 'Present' binary distribution; I) Range size variation.

Value	Present	Projected
AUC.train	0.948	0.888
AUC.test	0.890	0.863
AUC.diff	0.045	0.045
pROC	1.572	1.493
OR.p10	0.397	0.217
CBI	0.821	0.789
perc.stable	26.675	26.675
range.size	-0.393	-0.393

S.2.97 *Erysimum pseudorhaeticum* Biomod2

Species: *Erysimum pseudorhaeticum* Polatschek

Procedure: Biomod2

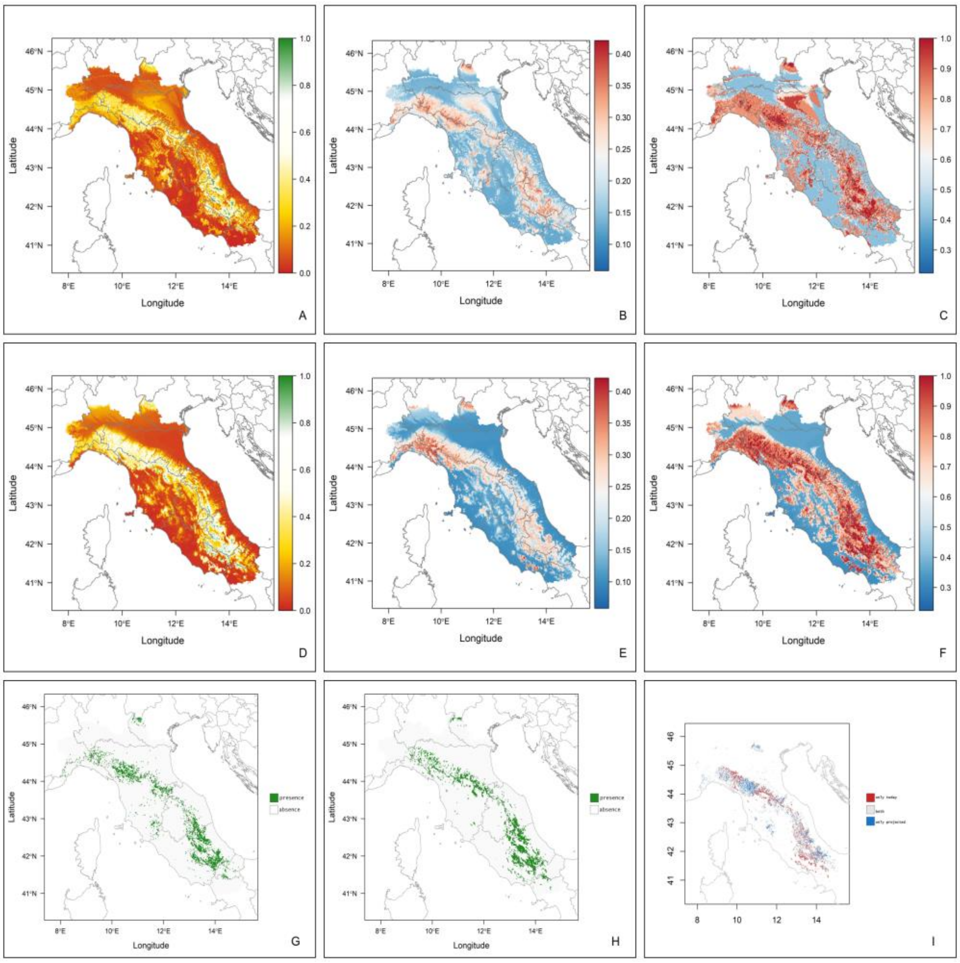

Fig. S.2.97-A) ‘Projected’ base output; B) ‘Projected’ uncertainty standard deviation; C) ‘Projected’ uncertainty range of values; D) ’Present’ base output; E) ’Present’ uncertainty standard deviation; F) ’Present’ uncertainty range of values; G) ‘Projected’ binary distribution; H) ‘Present’ binary distribution; I) Range size variation.

Value	Present	Projected
AUC.train	0.960	0.978
AUC.test	0.853	0.838
AUC.diff	0.140	0.140
pROC	1.476	1.367
OR.p10	0.414	0.621
CBI	0.989	0.919
perc.stable	35.891	35.891
range.size	-2.033	-2.033

S.2.98 *Erysimum pseudorhaeticum* Bioclim

Species: *Erysimum pseudorhaeticum* Polatschek

Procedure: Bioclim

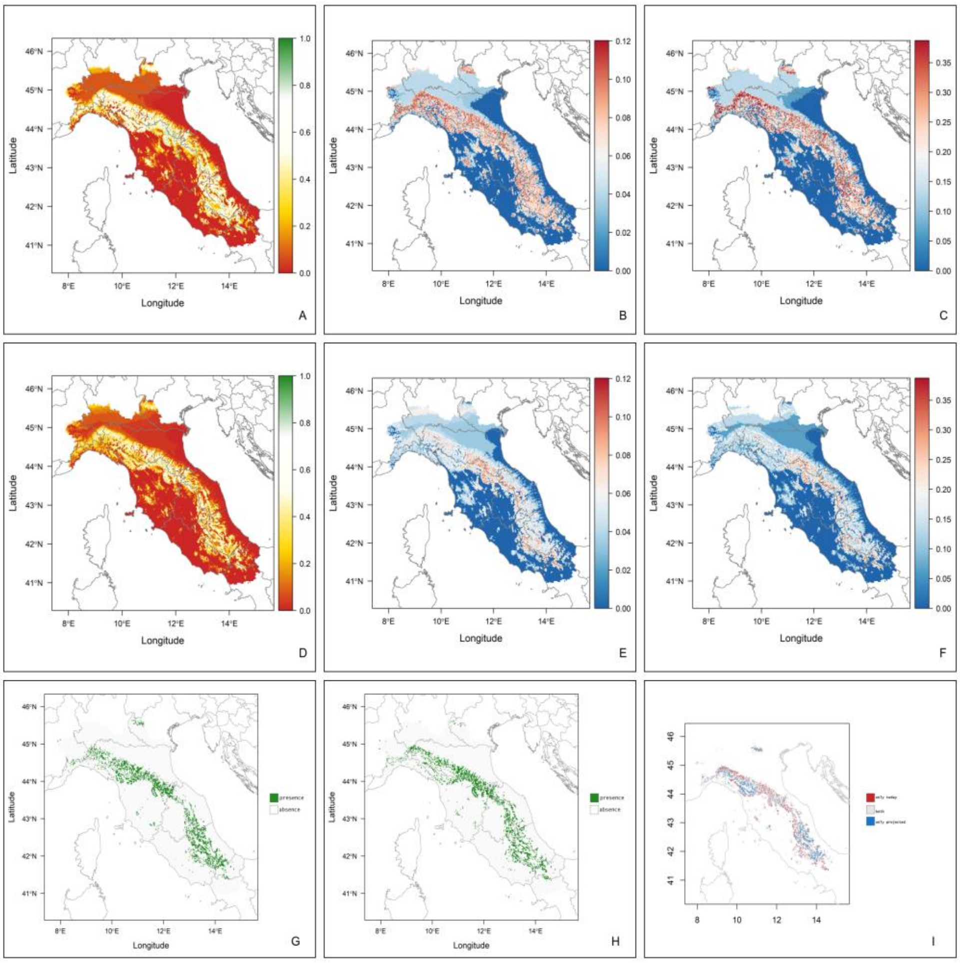

Fig. S.2.98-A) ‘Projected’ base output; B) ‘Projected’ uncertainty standard deviation; C) ‘Projected’ uncertainty range of values; D) ’Present’ base output; E) ’Present’ uncertainty standard deviation; F) ’Present’ uncertainty range of values; G) ‘Projected’ binary distribution; H) ‘Present’ binary distribution; I) Range size variation.

Value	Present	Projected
AUC.train	0.785	0.644
AUC.test	0.725	0.629
AUC.diff	0.033	0.033
pROC	1.177	1.084
OR.p10	0.250	0.186
CBI	0.448	0.831
perc.stable	41.870	41.870
range.size	-1.071	-1.071

S.2.99 *Erysimum pseudorhaeticum* Domain

Species: *Erysimum pseudorhaeticum* Polatschek

Procedure: Domain

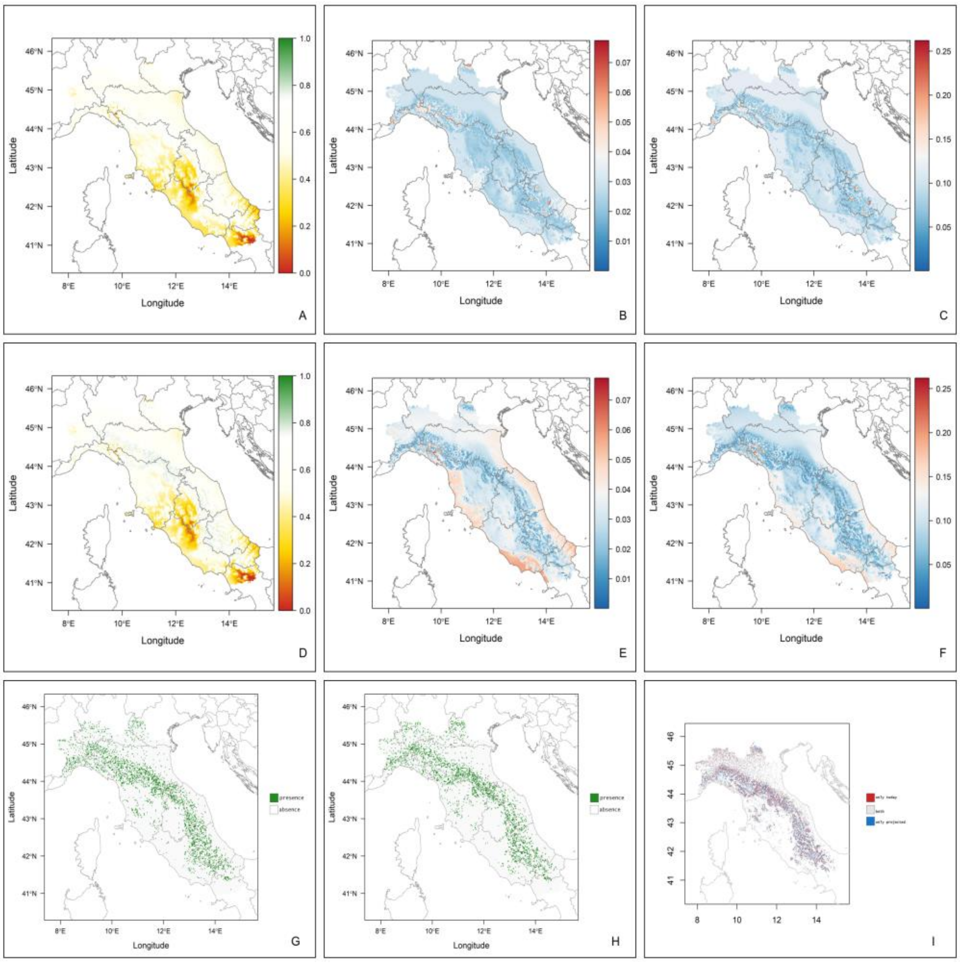

Fig. S.2.99-A) ‘Projected’ base output; B) ‘Projected’ uncertainty standard deviation; C) ‘Projected’ uncertainty range of values; D) ’Present’ base output; E) ’Present’ uncertainty standard deviation; F) ’Present’ uncertainty range of values; G) ‘Projected’ binary distribution; H) ‘Present’ binary distribution; I) Range size variation.

Value	Present	Projected
AUC.train	0.699	0.582
AUC.test	0.688	0.564
AUC.diff	0.043	0.043
pROC	1.192	1.152
OR.p10	0.186	0.214
CBI	0.933	0.982
perc.stable	16.644	16.644
range.size	1.418	1.418

S.2.100 *Erysimum pseudorhaeticum* Maxent

Species: *Erysimum pseudorhaeticum* Polatschek

Procedure: Maxent

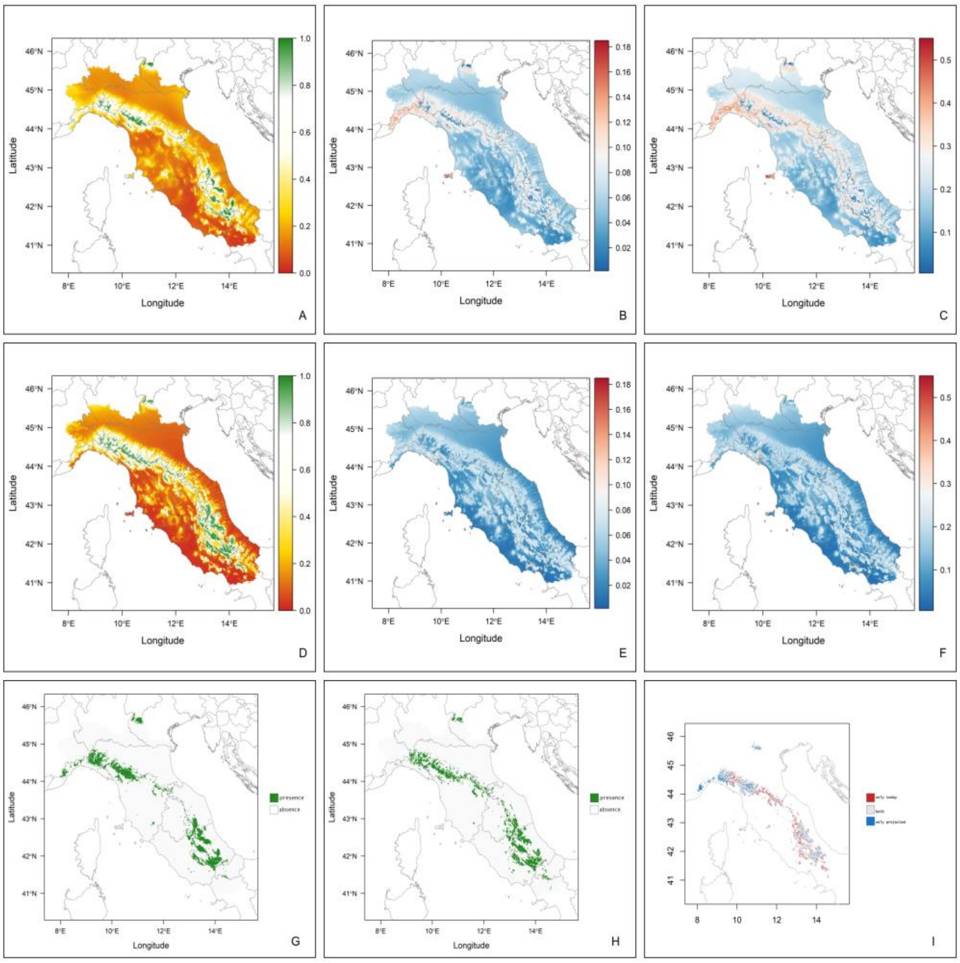

Fig. S.2.100-A) 'Projected' base output; B) 'Projected' uncertainty standard deviation; C) 'Projected' uncertainty range of values; D) 'Present' base output; E) 'Present' uncertainty standard deviation; F) 'Present' uncertainty range of values; G) 'Projected' binary distribution; H) 'Present' binary distribution; I) Range size variation.

Value	Present	Projected
AUC.train	0.847	0.789
AUC.test	0.816	0.791
AUC.diff	0.034	0.034
pROC	1.429	1.337
OR.p10	0.193	0.143
CBI	0.965	0.891
perc.stable	53.992	53.992
range.size	-1.063	-1.063

S.2.101 *Erysimum pseudorhaeticum* KDE

Species: *Erysimum pseudorhaeticum* Polatschek

Procedure: KDE

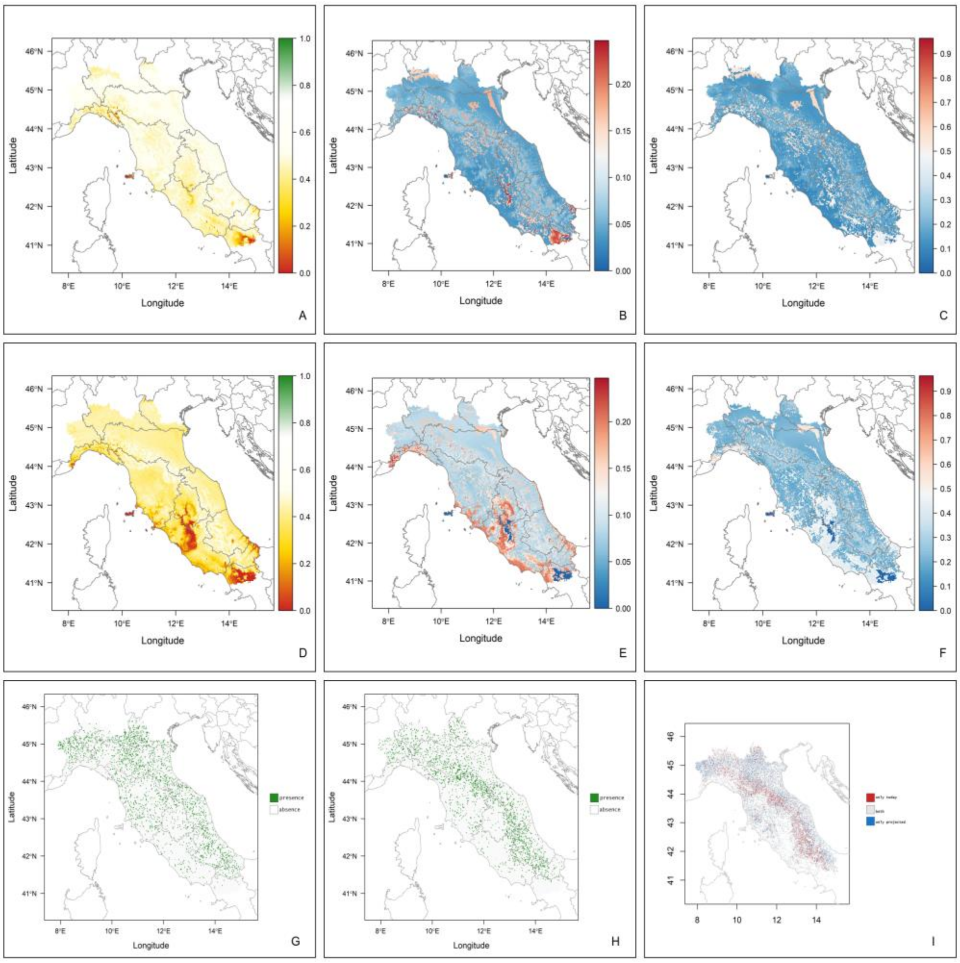

Fig. S.2.101-A) 'Projected' base output; B) 'Projected' uncertainty standard deviation; C) 'Projected' uncertainty range of values; D) 'Present' base output; E) 'Present' uncertainty standard deviation; F) 'Present' uncertainty range of values; G) 'Projected' binary distribution; H) 'Present' binary distribution; I) Range size variation.

Value	Present	Projected
AUC.train	0.687	0.533
AUC.test	0.641	0.468
AUC.diff	0.062	0.066
pROC	1.139	1.100
OR.p10	0.207	0.136
CBI	0.910	0.223
perc.stable	4.967	4.967
range.size	0.924	0.924

S.2.102 *Erysimum pseudorhaeticum* CTA

Species: *Erysimum pseudorhaeticum* Polatschek

Procedure: CTA

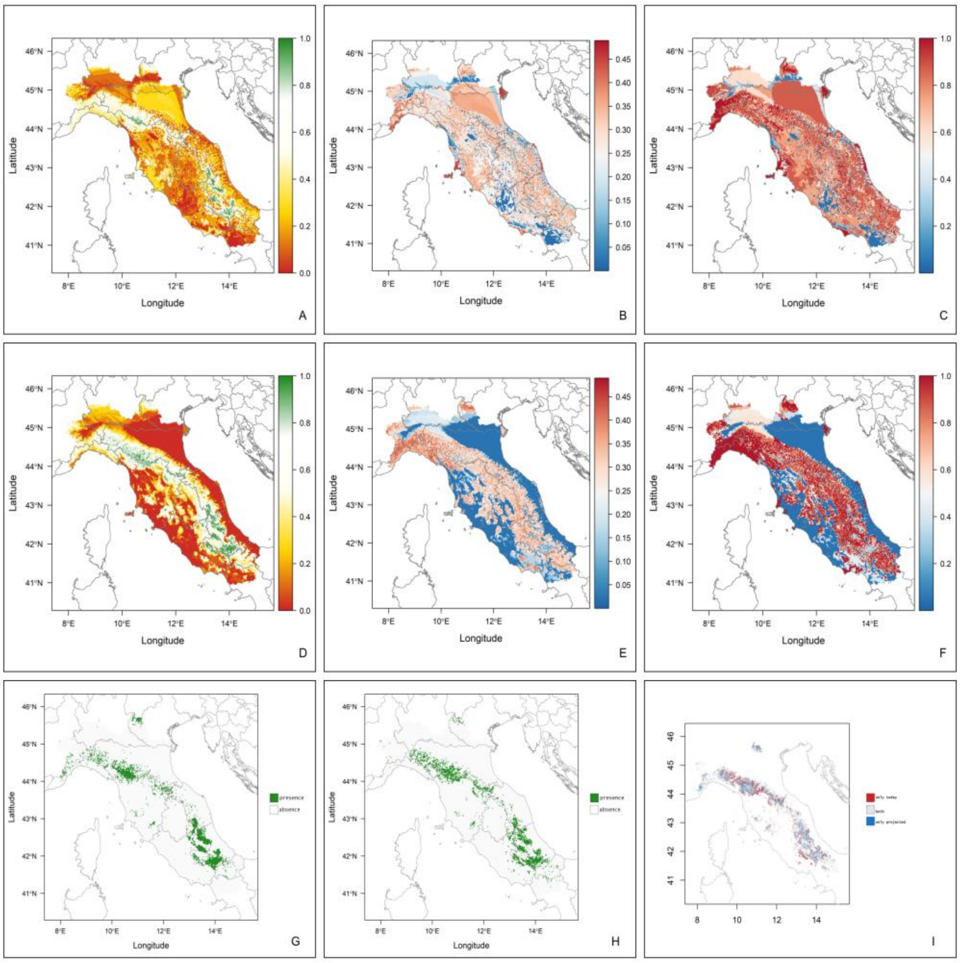

Fig. S.2.102-A) 'Projected' base output; B) 'Projected' uncertainty standard deviation; C) 'Projected' uncertainty range of values; D) 'Present' base output; E) 'Present' uncertainty standard deviation; F) 'Present' uncertainty range of values; G) 'Projected' binary distribution; H) 'Present' binary distribution; I) Range size variation.

Value	Present	Projected
AUC.train	0.933	0.923
AUC.test	0.827	0.782
AUC.diff	0.142	0.142
pROC	1.186	1.114
OR.p10	0.264	0.357
CBI	0.986	0.916
perc.stable	34.480	34.480
range.size	1.606	1.606

S.2.103 *Erysimum pseudorhaeticum* FDA

Species: *Erysimum pseudorhaeticum* Polatschek

Procedure: FDA

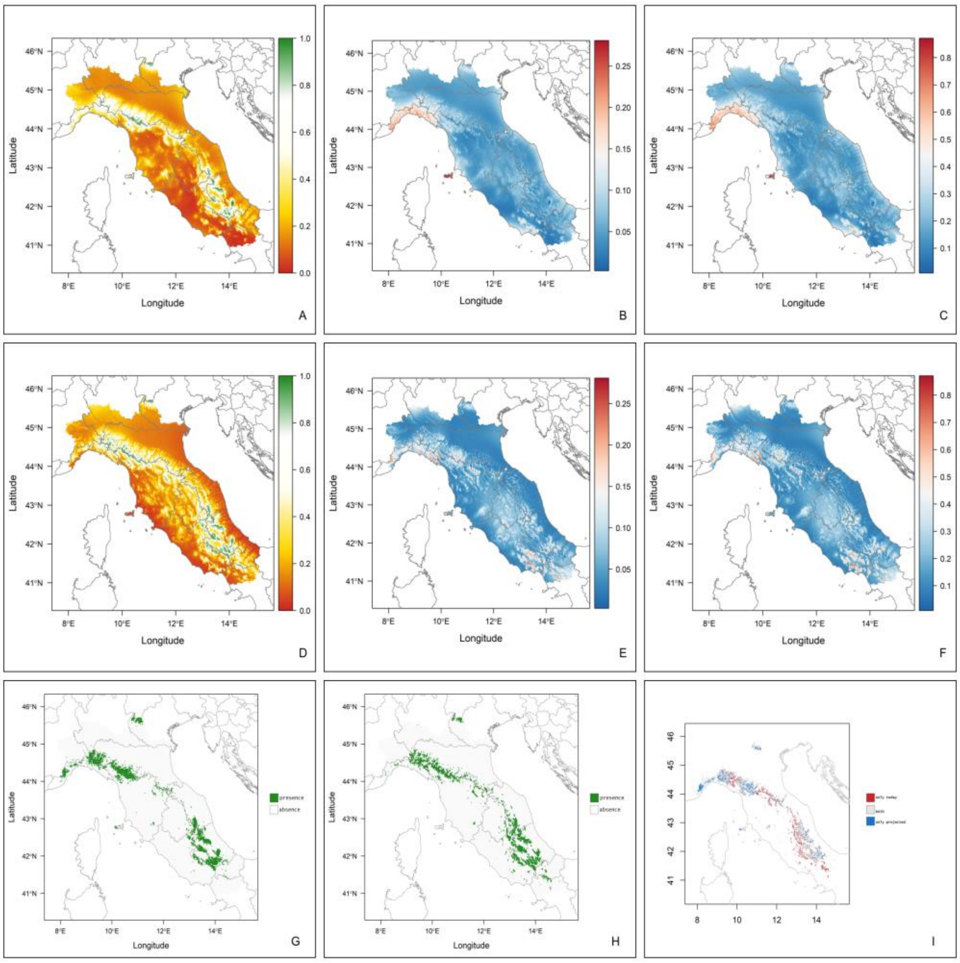

Fig. S.2.103-A) 'Projected' base output; B) 'Projected' uncertainty standard deviation; C) 'Projected' uncertainty range of values; D) 'Present' base output; E) 'Present' uncertainty standard deviation; F) 'Present' uncertainty range of values; G) 'Projected' binary distribution; H) 'Present' binary distribution; I) Range size variation.

Value	Present	Projected
AUC.train	0.823	0.792
AUC.test	0.822	0.764
AUC.diff	0.052	0.052
pROC	1.393	1.242
OR.p10	0.129	0.221
CBI	0.946	0.950
perc.stable	49.546	49.546
range.size	-0.436	-0.436

S.2.104 *Erysimum pseudorhaeticum* GAM

Species: *Erysimum pseudorhaeticum* Polatschek

Procedure: GAM

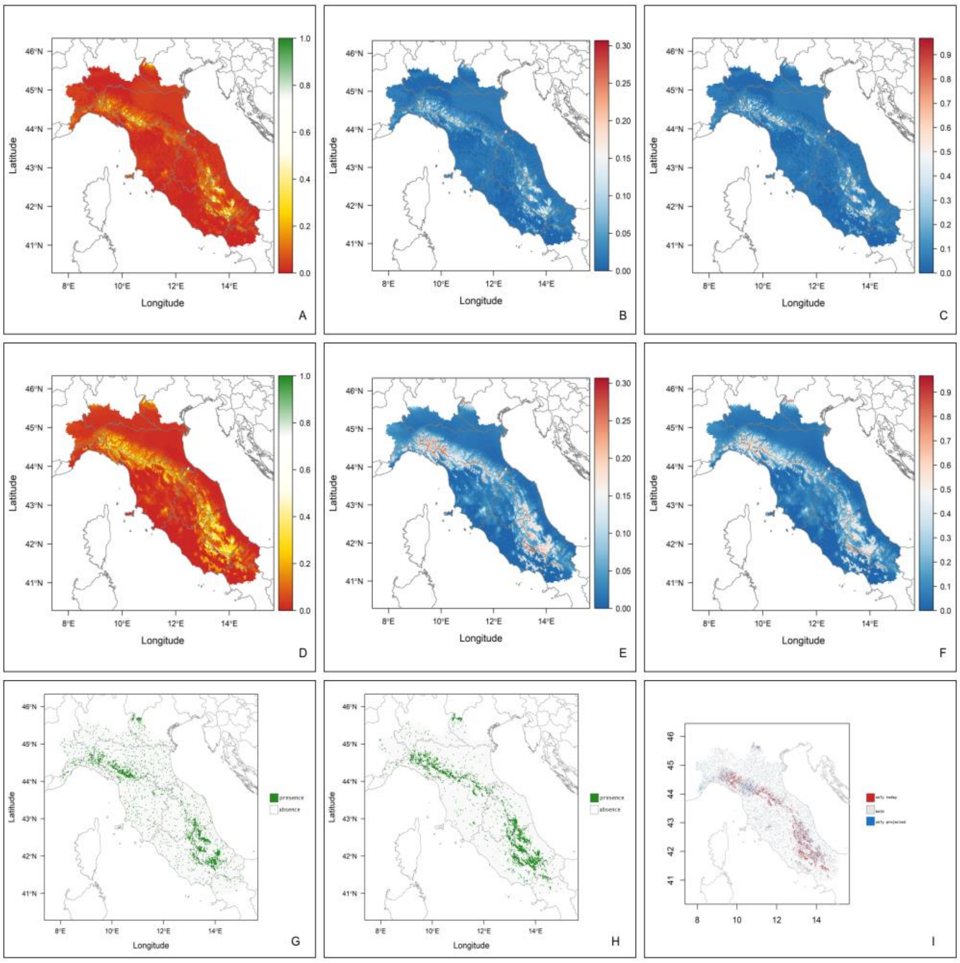

Fig. S.2.104-A) 'Projected' base output; B) 'Projected' uncertainty standard deviation; C) 'Projected' uncertainty range of values; D) 'Present' base output; E) 'Present' uncertainty standard deviation; F) 'Present' uncertainty range of values; G) 'Projected' binary distribution; H) 'Present' binary distribution; I) Range size variation.

Value	Present	Projected
AUC.train	0.858	0.809
AUC.test	0.824	0.771
AUC.diff	0.067	0.067
pROC	1.400	1.250
OR.p10	0.179	0.221
CBI	0.758	0.848
perc.stable	23.052	23.052
range.size	0.073	0.073

S.2.105 *Erysimum pseudorhaeticum* GLM

Species: *Erysimum pseudorhaeticum* Polatschek

Procedure: GLM

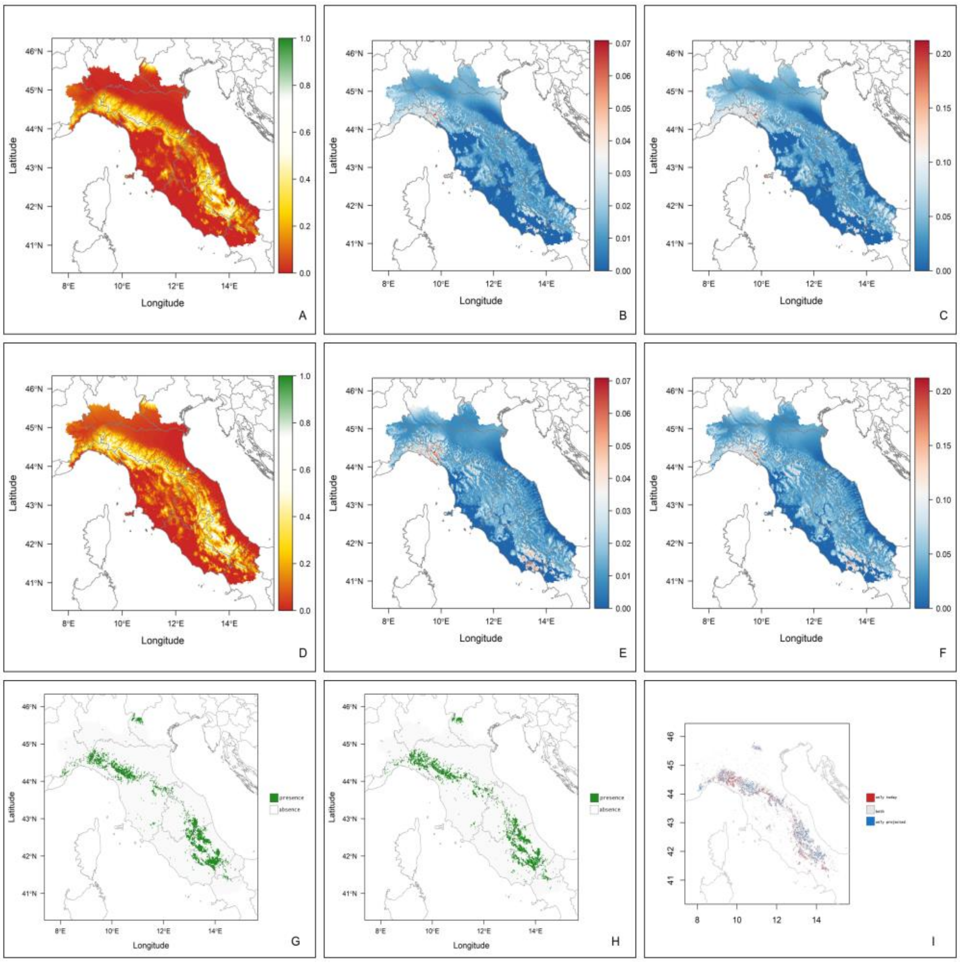

Fig. S.2.105-A) ‘Projected’ base output; B) ‘Projected’ uncertainty standard deviation; C) ‘Projected’ uncertainty range of values; D) ’Present’ base output; E) ’Present’ uncertainty standard deviation; F) ’Present’ uncertainty range of values; G) ‘Projected’ binary distribution; H) ‘Present’ binary distribution; I) Range size variation.

Value	Present	Projected
AUC.train	0.842	0.792
AUC.test	0.807	0.772
AUC.diff	0.035	0.035
pROC	1.384	1.247
OR.p10	0.193	0.150
CBI	0.909	0.919
perc.stable	47.159	47.159
range.size	0.349	0.349

S.2.106 *Erysimum pseudorhaeticum* MARS

Species: *Erysimum pseudorhaeticum* Polatschek

Procedure: MARS

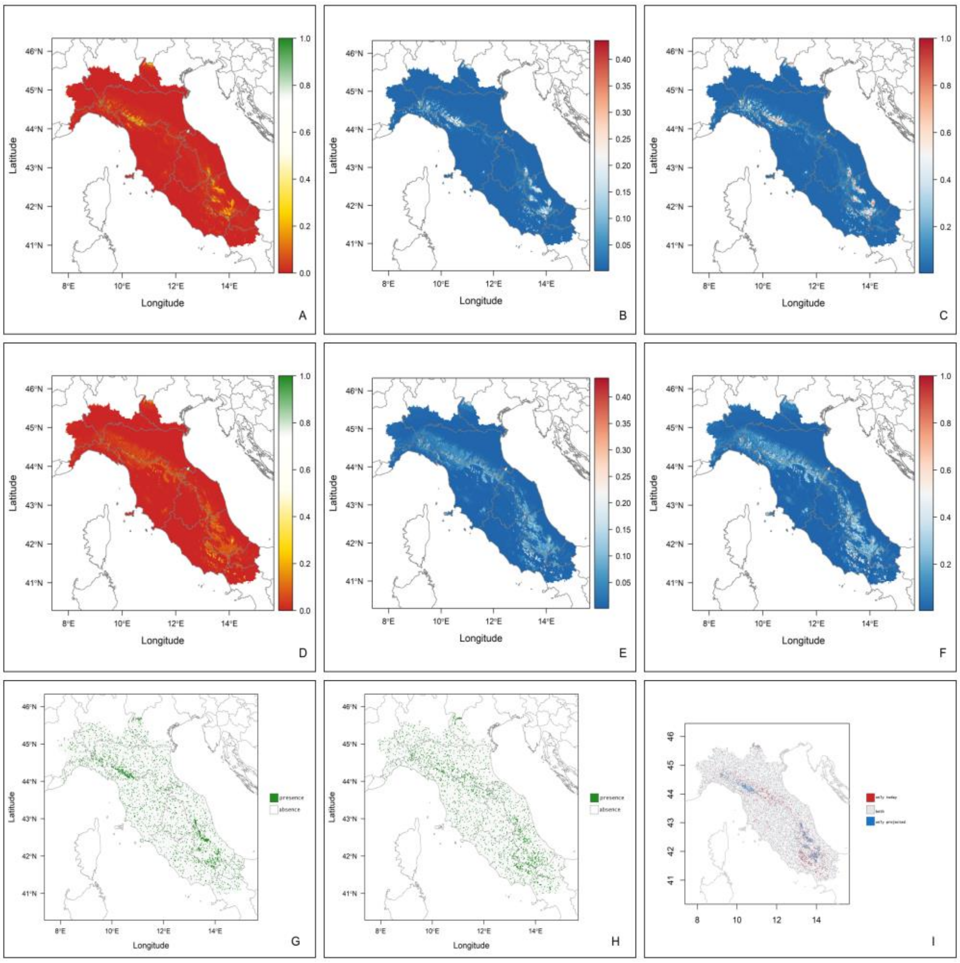

Fig. S.2.106-A) 'Projected' base output; B) 'Projected' uncertainty standard deviation; C) 'Projected' uncertainty range of values; D) 'Present' base output; E) 'Present' uncertainty standard deviation; F) 'Present' uncertainty range of values; G) 'Projected' binary distribution; H) 'Present' binary distribution; I) Range size variation.

Value	Present	Projected
AUC.train	0.880	0.801
AUC.test	0.726	0.721
AUC.diff	0.080	0.080
pROC	1.160	1.115
OR.p10	0.386	0.179
CBI	0.735	0.714
perc.stable	6.514	6.514
range.size	0.905	0.905

S.2.107 *Erysimum pseudorhaeticum* RF

Species: *Erysimum pseudorhaeticum* Polatschek

Procedure: RF

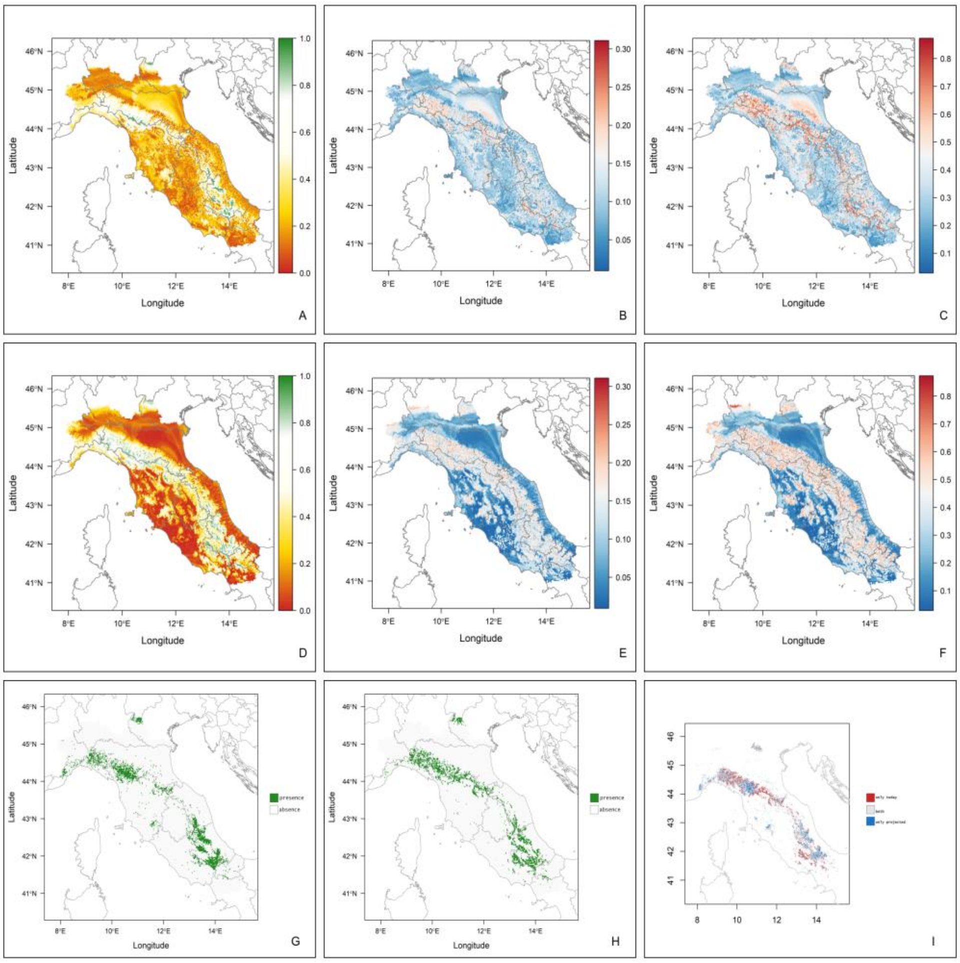

Fig. S.2.107-A) 'Projected' base output; B) 'Projected' uncertainty standard deviation; C) 'Projected' uncertainty range of values; D) 'Present' base output; E) 'Present' uncertainty standard deviation; F) 'Present' uncertainty range of values; G) 'Projected' binary distribution; H) 'Present' binary distribution; I) Range size variation.

Value	Present	Projected
AUC.train	1.000	1.000
AUC.test	0.938	0.943
AUC.diff	0.057	0.057
pROC	1.337	1.306
OR.p10	0.686	0.557
CBI	0.980	0.984
perc.stable	36.074	36.074
range.size	-0.757	-0.757

S.2.108 *Erysimum pseudorhaeticum* SVM

Species: *Erysimum pseudorhaeticum* Polatschek

Procedure: SVM

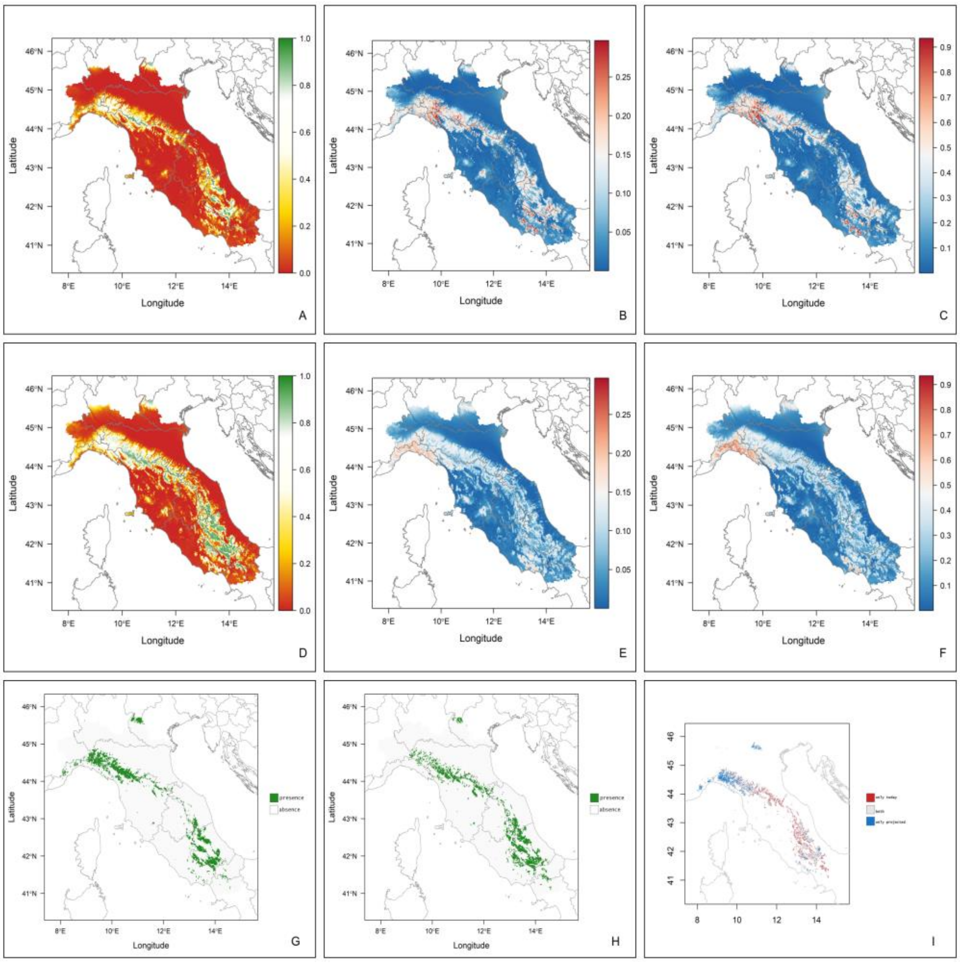

Fig. S.2.108-A) 'Projected' base output; B) 'Projected' uncertainty standard deviation; C) 'Projected' uncertainty range of values; D) 'Present' base output; E) 'Present' uncertainty standard deviation; F) 'Present' uncertainty range of values; G) 'Projected' binary distribution; H) 'Present' binary distribution; I) Range size variation.

Value	Present	Projected
AUC.train	0.882	0.836
AUC.test	0.846	0.801
AUC.diff	0.048	0.048
pROC	1.390	1.217
OR.p10	0.171	0.186
CBI	0.914	0.784
perc.stable	49.546	49.546
range.size	0.043	0.043

S.2.109 *Galium magellense* Biomod2

Species: *Galium magellense* Ten.

Procedure: Biomod2

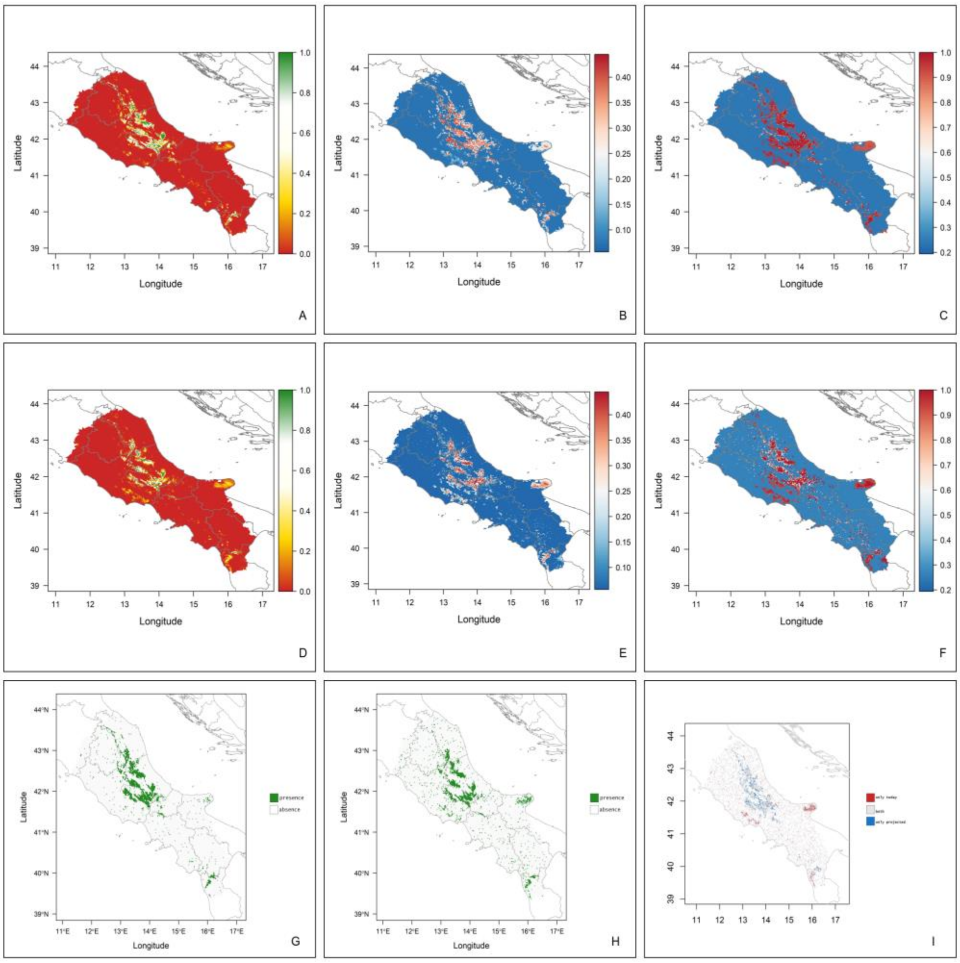

Fig. S.2.109-A) 'Projected' base output; B) 'Projected' uncertainty standard deviation; C) 'Projected' uncertainty range of values; D) 'Present' base output; E) 'Present' uncertainty standard deviation; F) 'Present' uncertainty range of values; G) 'Projected' binary distribution; H) 'Present' binary distribution; I) Range size variation.

Value	Present	Projected
AUC.train	0.997	0.998
AUC.test	0.987	0.994
AUC.diff	0.004	0.004
pROC	1.898	1.948
OR.p10	0.333	0.267
CBI	0.922	0.958
perc.stable	41.705	41.705
range.size	-2.431	-2.431

S.2.110 *Galium magellense* Bioclim

Species: *Galium magellense* Ten.

Procedure: Bioclim

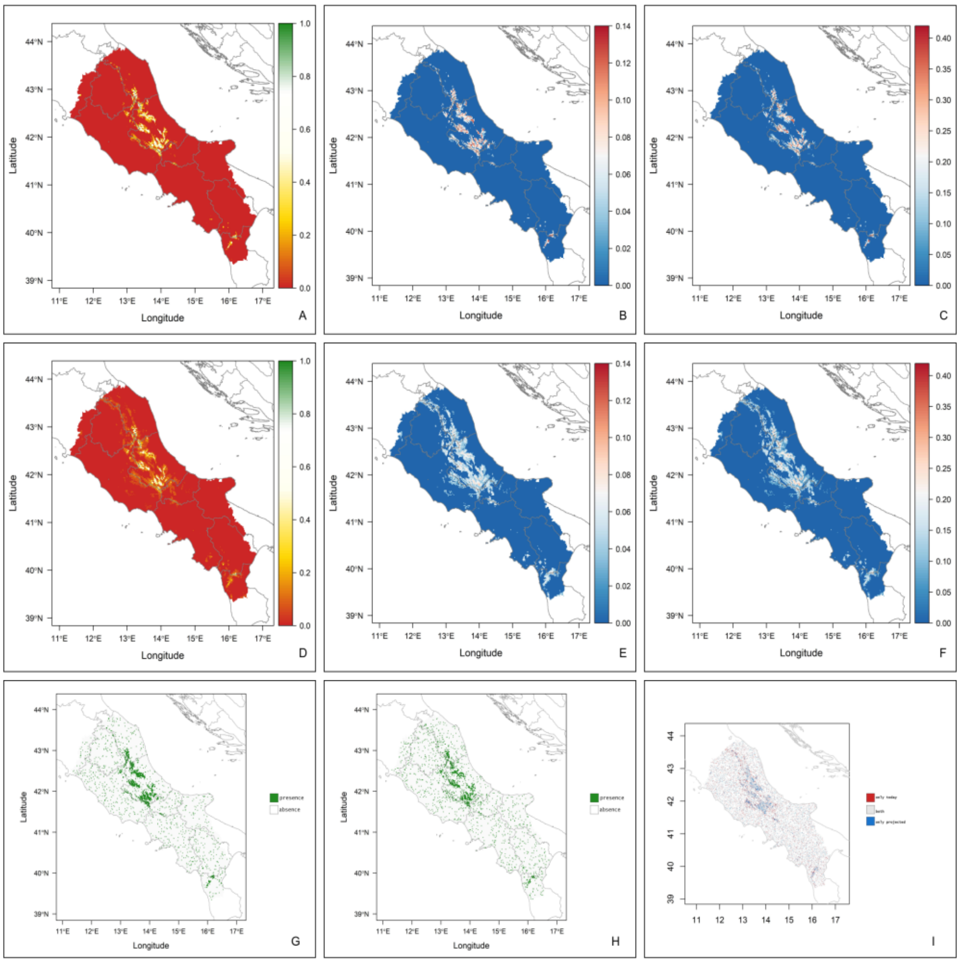

Fig. S.2.110-A) 'Projected' base output; B) 'Projected' uncertainty standard deviation; C) 'Projected' uncertainty range of values; D) 'Present' base output; E) 'Present' uncertainty standard deviation; F) 'Present' uncertainty range of values; G) 'Projected' binary distribution; H) 'Present' binary distribution; I) Range size variation.

Value	Present	Projected
AUC.train	0.975	0.974
AUC.test	0.919	0.914
AUC.diff	0.061	0.061
pROC	1.485	1.486
OR.p10	0.150	0.125
CBI	0.662	0.675
perc.stable	20.238	20.238
range.size	-2.289	-2.289

S.2.111 *Galium magellense* Domain

Species: *Galium magellense* Ten.

Procedure: Domain

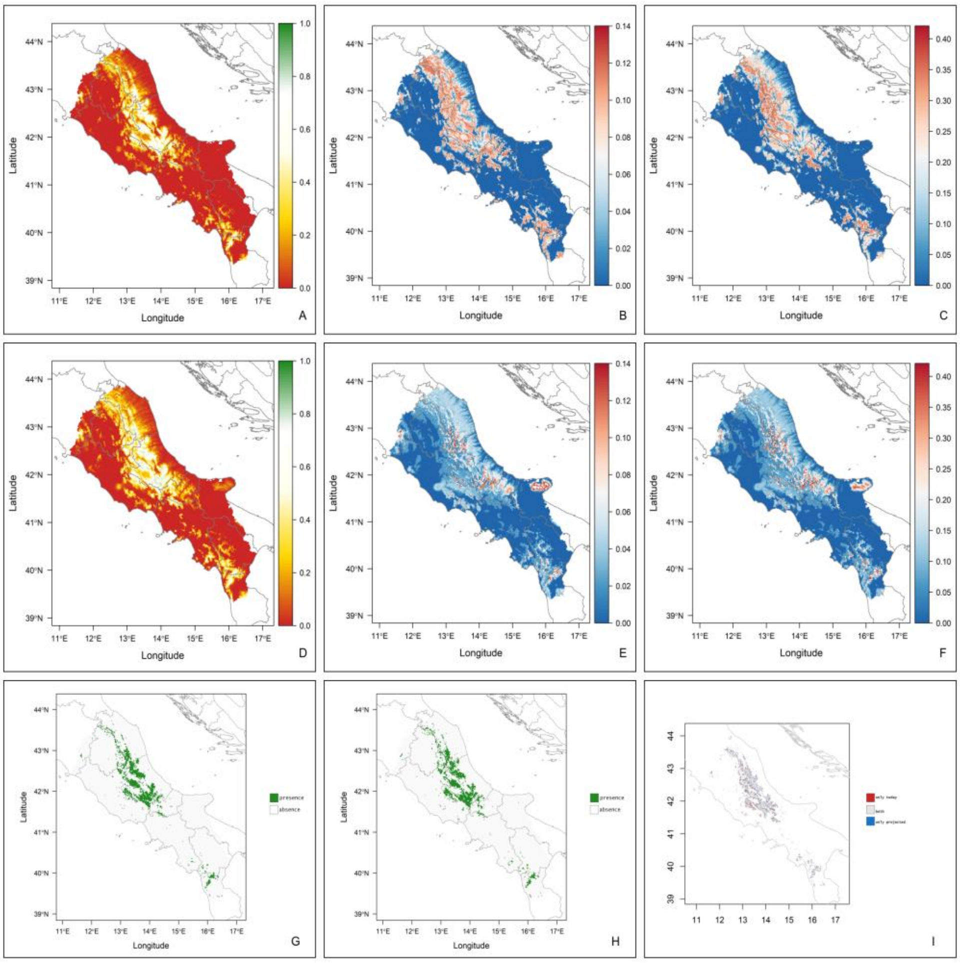

Fig. S.2.111-A) 'Projected' base output; B) 'Projected' uncertainty standard deviation; C) 'Projected' uncertainty range of values; D) 'Present' base output; E) 'Present' uncertainty standard deviation; F) 'Present' uncertainty range of values; G) 'Projected' binary distribution; H) 'Present' binary distribution; I) Range size variation.

Value	Present	Projected
AUC.train	0.977	0.968
AUC.test	0.970	0.968
AUC.diff	0.004	0.004
pROC	1.827	1.903
OR.p10	0.108	0.133
CBI	0.995	0.922
perc.stable	59.439	59.439
range.size	0.495	0.495

S.2.112 *Galium magellense* Maxent

Species: *Galium magellense* Ten.

Procedure: Maxent

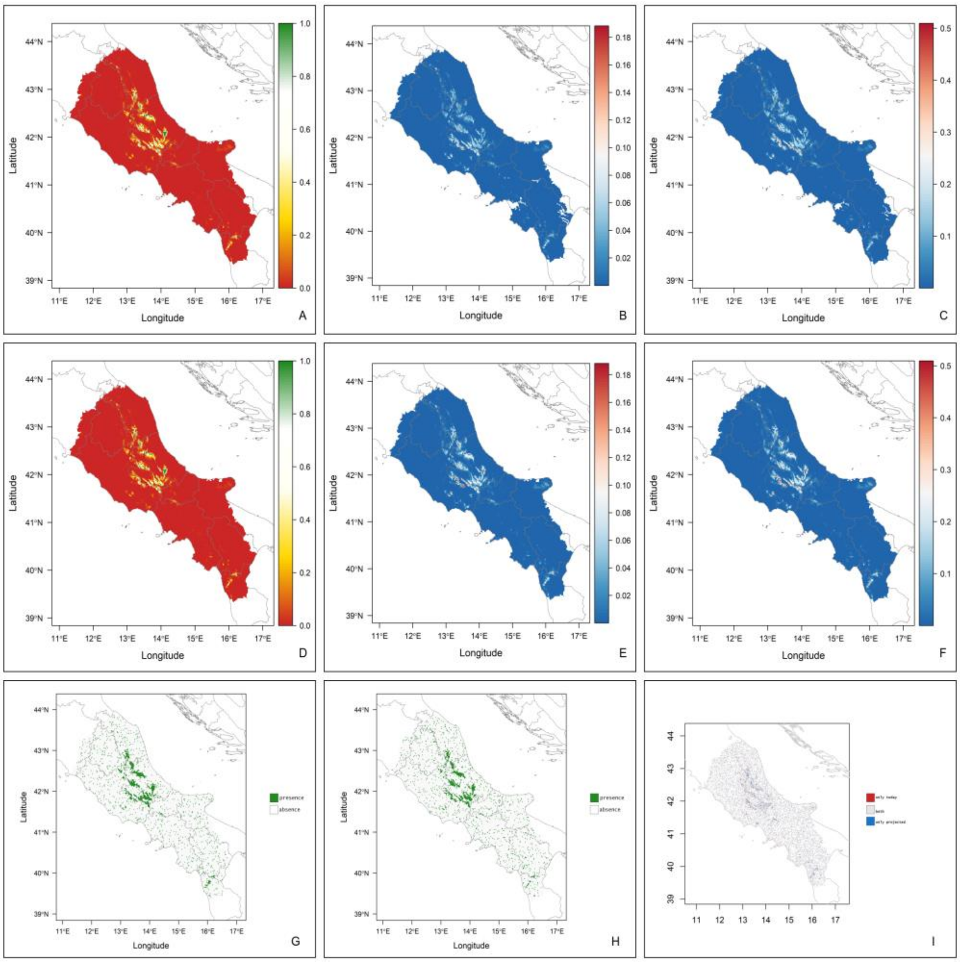

Fig. S.2.112-A) 'Projected' base output; B) 'Projected' uncertainty standard deviation; C) 'Projected' uncertainty range of values; D) 'Present' base output; E) 'Present' uncertainty standard deviation; F) 'Present' uncertainty range of values; G) 'Projected' binary distribution; H) 'Present' binary distribution; I) Range size variation.

Value	Present	Projected
AUC.train	0.984	0.988
AUC.test	0.984	0.985
AUC.diff	0.003	0.003
pROC	1.896	1.917
OR.p10	0.150	0.150
CBI	0.955	0.958
perc.stable	23.262	23.262
range.size	2.114	2.114

S.2.113 *Galium magellense* KDE

Species: *Galium magellense* Ten.

Procedure: KDE

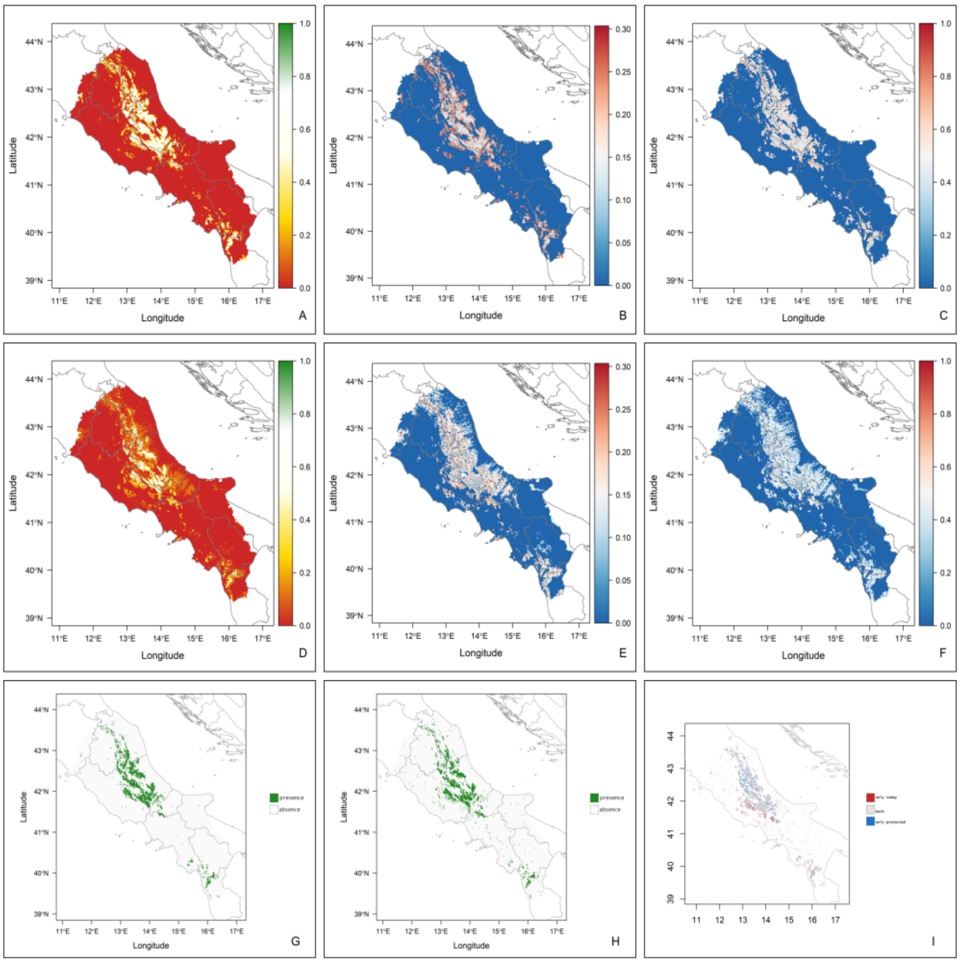

Fig. S.2.113-A) 'Projected' base output; B) 'Projected' uncertainty standard deviation; C) 'Projected' uncertainty range of values; D) 'Present' base output; E) 'Present' uncertainty standard deviation; F) 'Present' uncertainty range of values; G) 'Projected' binary distribution; H) 'Present' binary distribution; I) Range size variation.

Value	Present	Projected
AUC.train	0.979	0.969
AUC.test	0.913	0.965
AUC.diff	0.067	0.012
pROC	1.424	1.822
OR.p10	0.267	0.158
CBI	0.979	0.943
perc.stable	54.786	54.786
range.size	-0.447	-0.447

S.2.114 *Galium magellense* CTA

Species: *Galium magellense* Ten.

Procedure: CTA

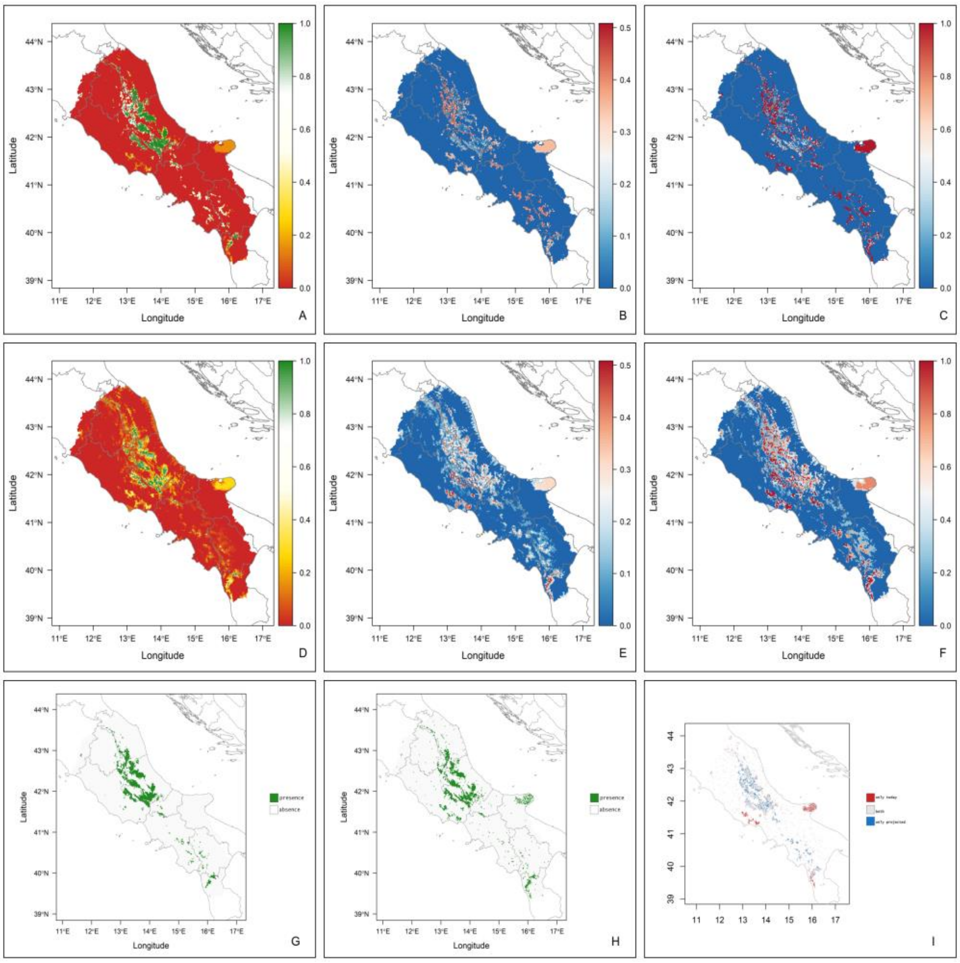

Fig. S.2.114-A) 'Projected' base output; B) 'Projected' uncertainty standard deviation; C) 'Projected' uncertainty range of values; D) 'Present' base output; E) 'Present' uncertainty standard deviation; F) 'Present' uncertainty range of values; G) 'Projected' binary distribution; H) 'Present' binary distribution; I) Range size variation.

Value	Present	Projected
AUC.train	0.984	0.992
AUC.test	0.928	0.978
AUC.diff	0.014	0.014
pROC	1.169	1.202
OR.p10	0.133	0.033
CBI	0.929	0.411
perc.stable	52.725	52.725
range.size	-1.184	-1.184

S.2.115 *Galium magellense* FDA

Species: *Galium magellense* Ten.

Procedure: FDA

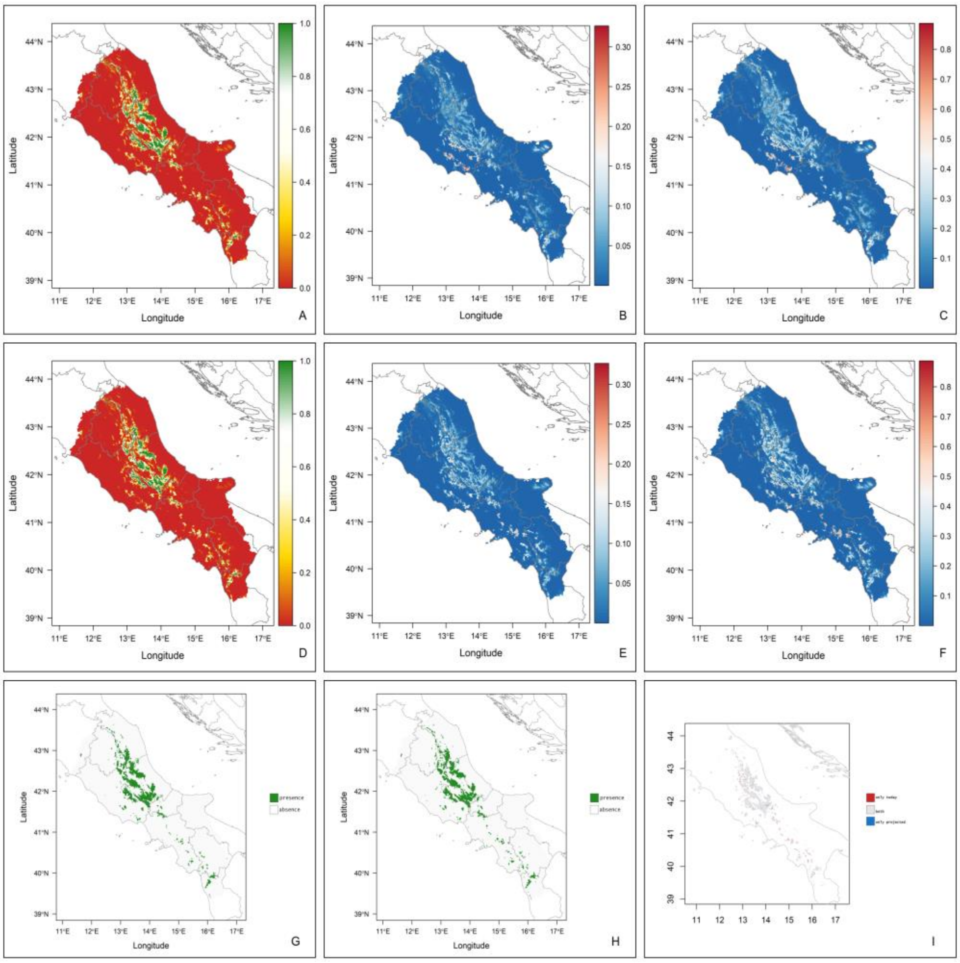

Fig. S.2.115-A) 'Projected' base output; B) 'Projected' uncertainty standard deviation; C) 'Projected' uncertainty range of values; D) 'Present' base output; E) 'Present' uncertainty standard deviation; F) 'Present' uncertainty range of values; G) 'Projected' binary distribution; H) 'Present' binary distribution; I) Range size variation.

Value	Present	Projected
AUC.train	0.982	0.986
AUC.test	0.982	0.986
AUC.diff	0.004	0.004
pROC	1.812	1.914
OR.p10	0.150	0.117
CBI	0.595	0.568
perc.stable	86.124	86.124
range.size	-1.544	-1.544

S.2.116 *Galium magellense* GAM

Species: *Galium magellense* Ten.

Procedure: GAM

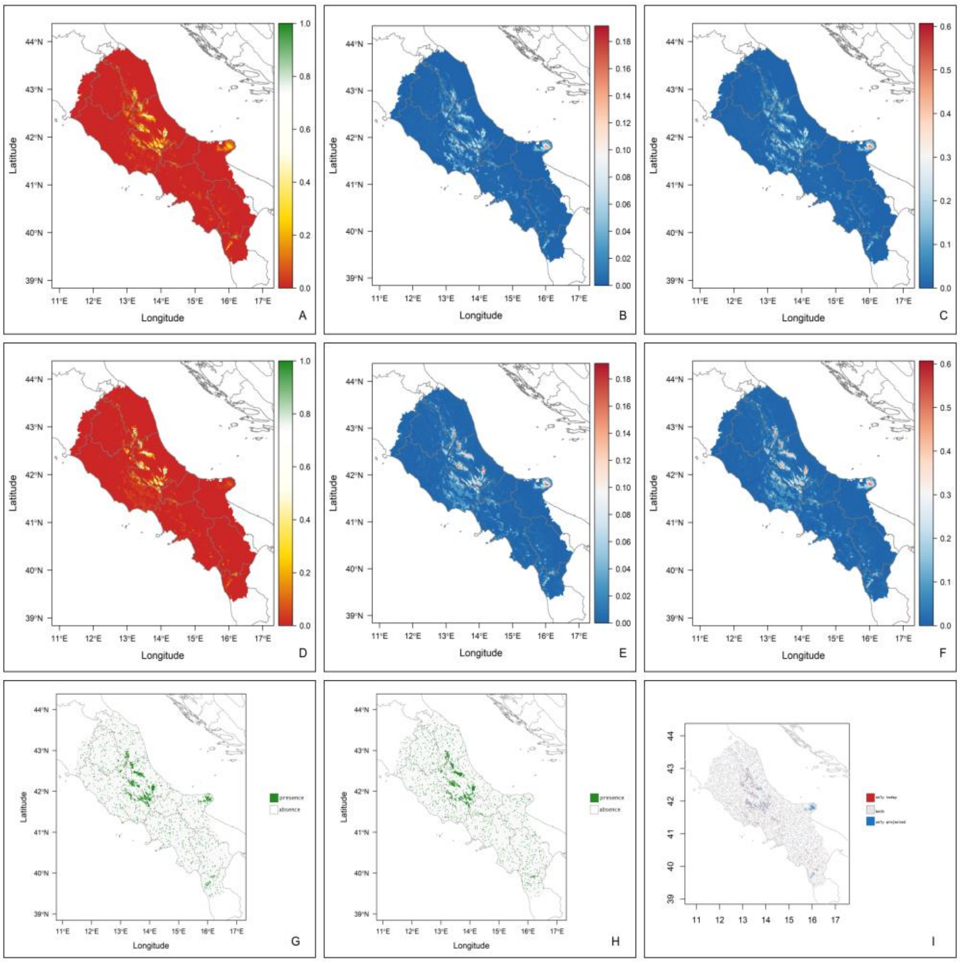

Fig. S.2.116-A) 'Projected' base output; B) 'Projected' uncertainty standard deviation; C) 'Projected' uncertainty range of values; D) 'Present' base output; E) 'Present' uncertainty standard deviation; F) 'Present' uncertainty range of values; G) 'Projected' binary distribution; H) 'Present' binary distribution; I) Range size variation.

Value	Present	Projected
AUC.train	0.978	0.983
AUC.test	0.961	0.983
AUC.diff	0.003	0.003
pROC	1.760	1.903
OR.p10	0.167	0.117
CBI	0.955	0.987
perc.stable	16.518	16.518
range.size	-0.687	-0.687

S.2.117 *Galium magellense* GLM

Species: *Galium magellense* Ten.

Procedure: GLM

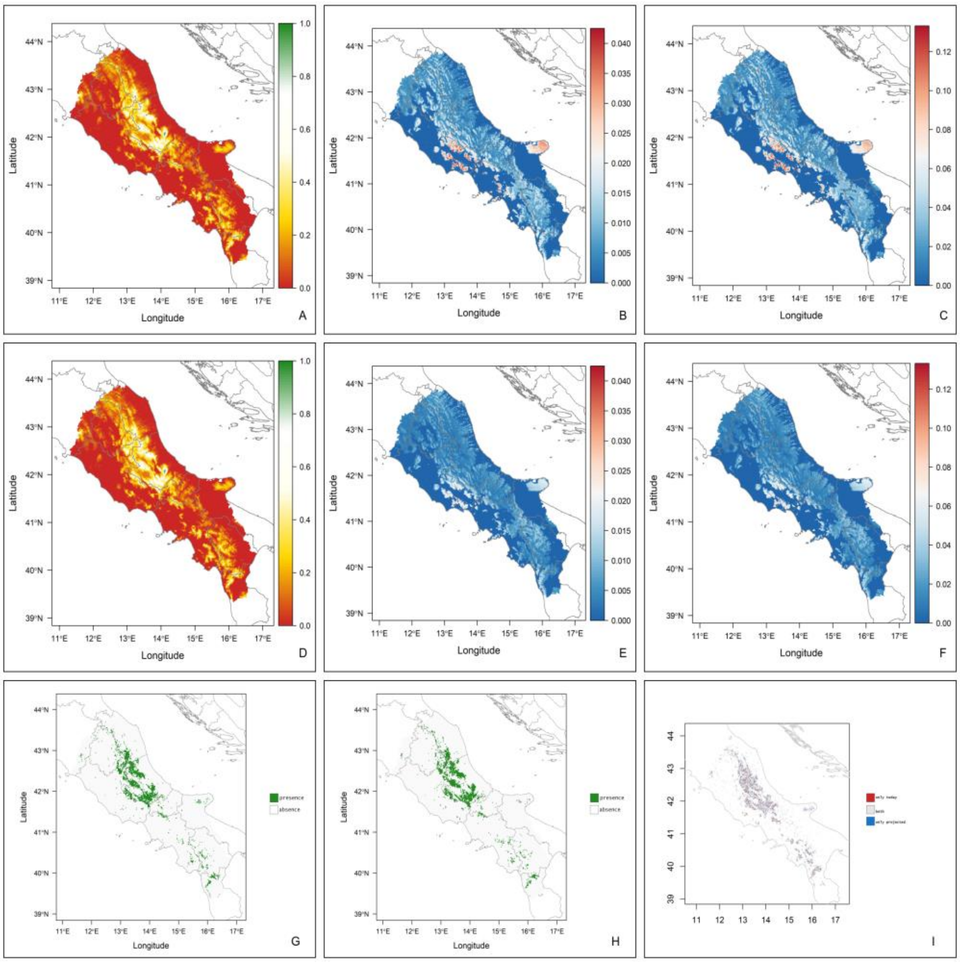

Fig. S.2.117-A) 'Projected' base output; B) 'Projected' uncertainty standard deviation; C) 'Projected' uncertainty range of values; D) 'Present' base output; E) 'Present' uncertainty standard deviation; F) 'Present' uncertainty range of values; G) 'Projected' binary distribution; H) 'Present' binary distribution; I) Range size variation.

Value	Present	Projected
AUC.train	0.982	0.987
AUC.test	0.979	0.985
AUC.diff	0.004	0.004
pROC	1.878	1.916
OR.p10	0.200	0.167
CBI	0.990	0.993
perc.stable	49.266	49.266
range.size	0.286	0.286

S.2.118 *Galium magellense* MARS

Species: *Galium magellense* Ten.

Procedure: MARS

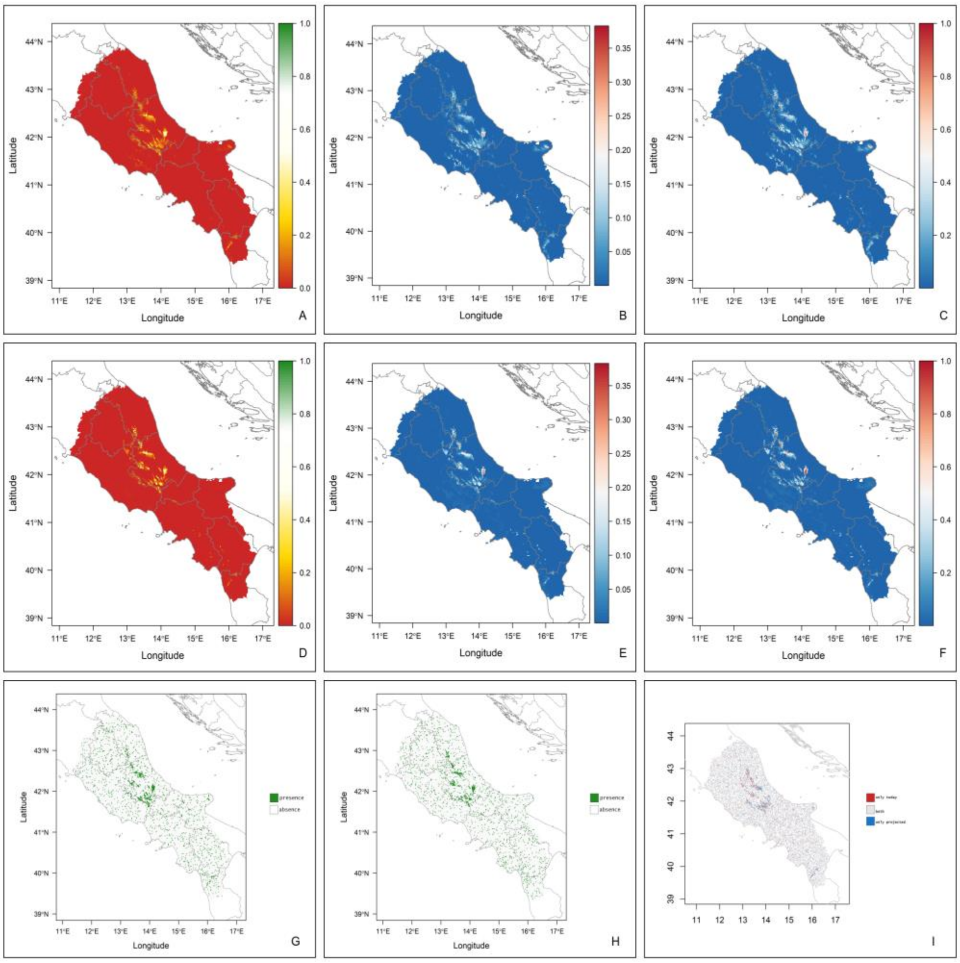

Fig. S.2.118-A) 'Projected' base output; B) 'Projected' uncertainty standard deviation; C) 'Projected' uncertainty range of values; D) 'Present' base output; E) 'Present' uncertainty standard deviation; F) 'Present' uncertainty range of values; G) 'Projected' binary distribution; H) 'Present' binary distribution; I) Range size variation.

Value	Present	Projected
AUC.train	0.976	0.987
AUC.test	0.953	0.937
AUC.diff	0.051	0.051
pROC	1.676	1.627
OR.p10	0.192	0.267
CBI	0.979	0.953
perc.stable	8.876	8.876
range.size	-0.561	-0.561

S.2.119 *Galium magellense* RF

Species: *Galium magellense* Ten.

Procedure: RF

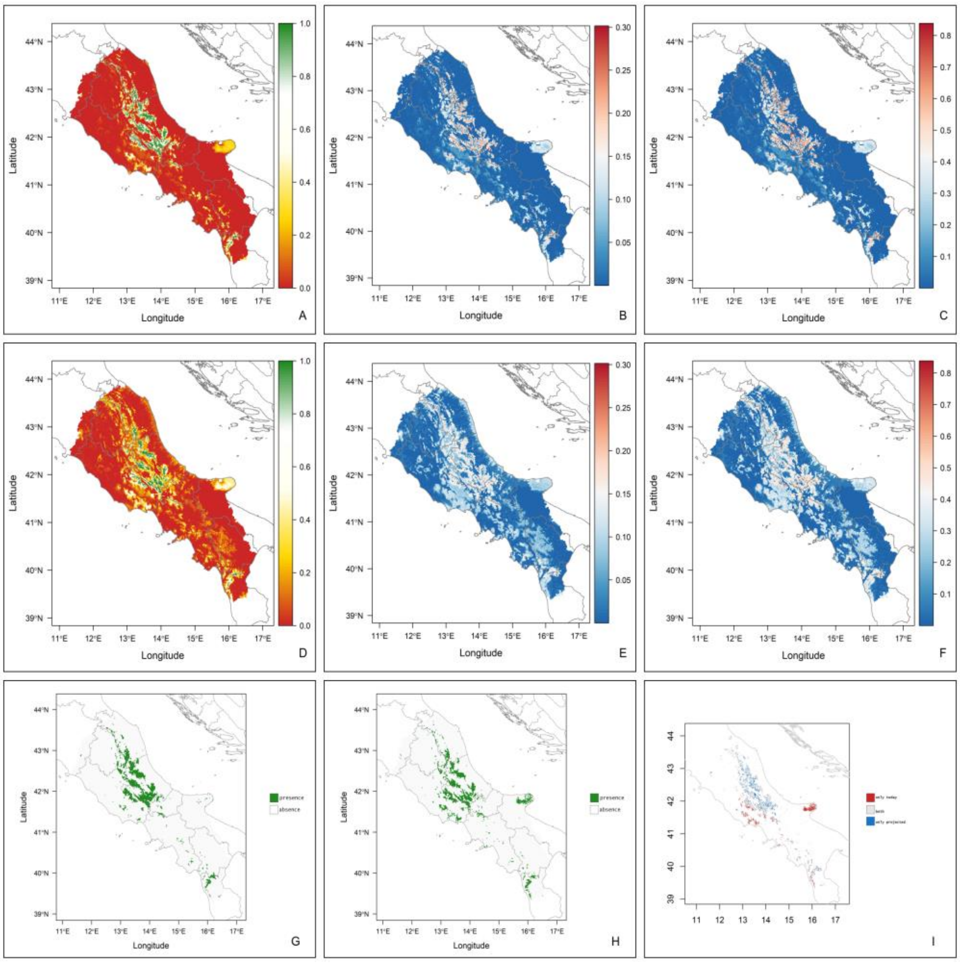

Fig. S.2.119-A) ‘Projected’ base output; B) ‘Projected’ uncertainty standard deviation; C) ‘Projected’ uncertainty range of values; D) ’Present’ base output; E) ’Present’ uncertainty standard deviation; F) ’Present’ uncertainty range of values; G) ‘Projected’ binary distribution; H) ‘Present’ binary distribution; I) Range size variation.

Value	Present	Projected
AUC.train	1.000	1.000
AUC.test	0.998	0.999
AUC.diff	0.001	0.001
pROC	1.888	1.900
OR.p10	0.233	0.292
CBI	0.932	0.859
perc.stable	56.895	56.895
range.size	1.755	1.755

S.2.120 *Galium magellense* SVM

Species: *Galium magellense* Ten.

Procedure: SVM

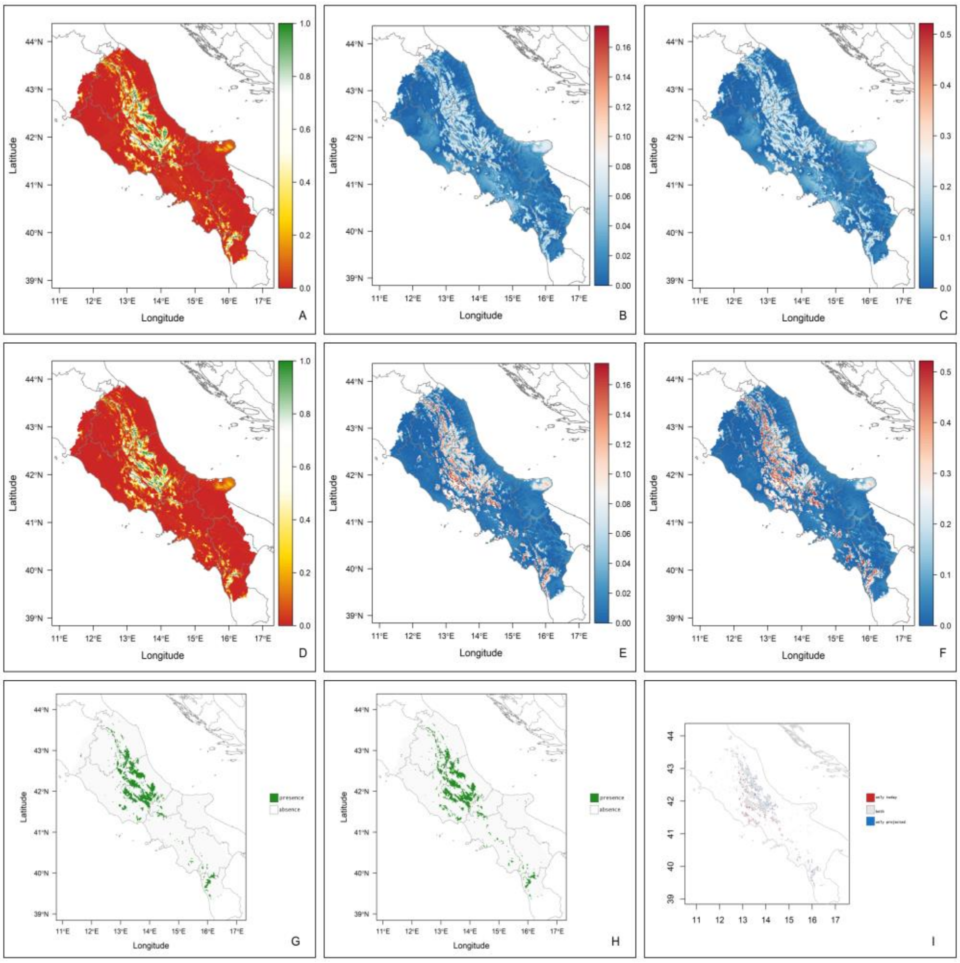

Fig. S.2.120-A) 'Projected' base output; B) 'Projected' uncertainty standard deviation; C) 'Projected' uncertainty range of values; D) 'Present' base output; E) 'Present' uncertainty standard deviation; F) 'Present' uncertainty range of values; G) 'Projected' binary distribution; H) 'Present' binary distribution; I) Range size variation.

Value	Present	Projected
AUC.train	0.984	0.987
AUC.test	0.984	0.986
AUC.diff	0.003	0.003
pROC	1.900	1.925
OR.p10	0.158	0.150
CBI	0.885	0.895
perc.stable	72.955	72.955
range.size	-0.215	-0.215

S.2.121 *Gypsophila arrostoi* Biomod2

Species: *Gypsophila arrostoi* Guss. subsp. *arrostoi*

Procedure: Biomod2

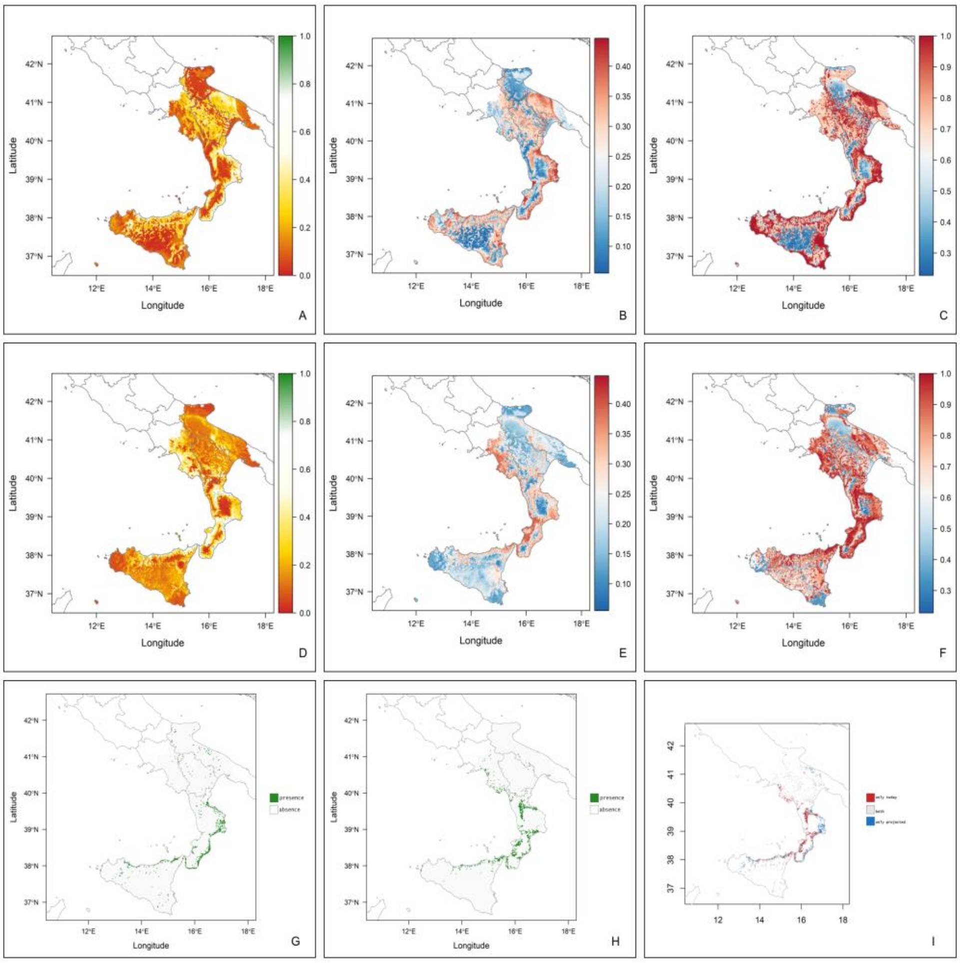

Fig. S.2.121-A) ‘Projected’ base output; B) ‘Projected’ uncertainty standard deviation; C) ‘Projected’ uncertainty range of values; D) ’Present’ base output; E) ’Present’ uncertainty standard deviation; F) ’Present’ uncertainty range of values; G) ‘Projected’ binary distribution; H) ‘Present’ binary distribution; I) Range size variation.

Value	Present	Projected
AUC.train	0.993	1.000
AUC.test	0.822	0.743
AUC.diff	0.257	0.257
pROC	1.470	1.399
OR.p10	0.871	1.000
CBI	0.952	0.544
perc.stable	7.729	7.729
range.size	-4.699	-4.699

S.2.122 *Gypsophila arrostoi* Bioclim

Species: *Gypsophila arrostoi* Guss. subsp. *arrostoi*

Procedure: Bioclim

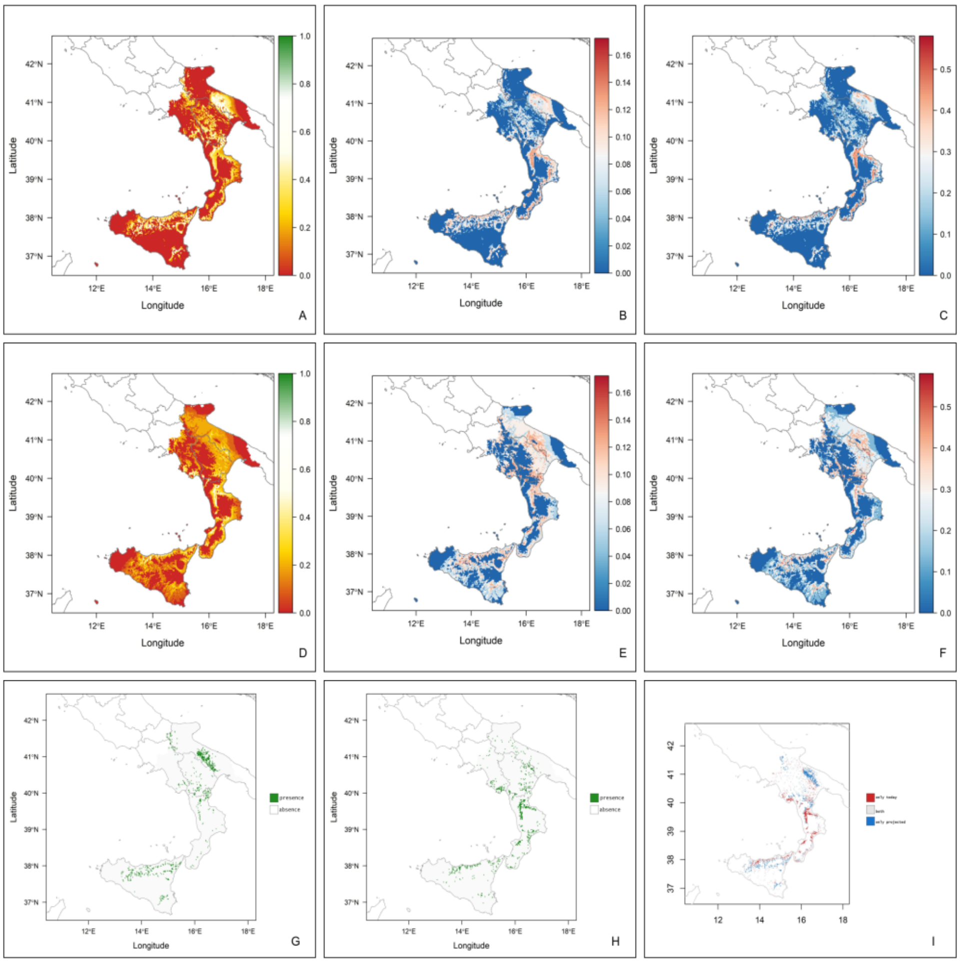

Fig. S.2.122-A) 'Projected' base output; B) 'Projected' uncertainty standard deviation; C) 'Projected' uncertainty range of values; D) 'Present' base output; E) 'Present' uncertainty standard deviation; F) 'Present' uncertainty range of values; G) 'Projected' binary distribution; H) 'Present' binary distribution; I) Range size variation.

Value	Present	Projected
AUC.train	0.773	0.794
AUC.test	0.628	0.681
AUC.diff	0.128	0.128
pROC	1.140	1.226
OR.p10	0.324	0.311
CBI	0.914	0.623
perc.stable	8.604	8.604
range.size	-1.404	-1.404

S.2.123 *Gypsophila arrostoi* Domain

Species: *Gypsophila arrostoi* Guss. subsp. *arrostoi*

Procedure: Domain

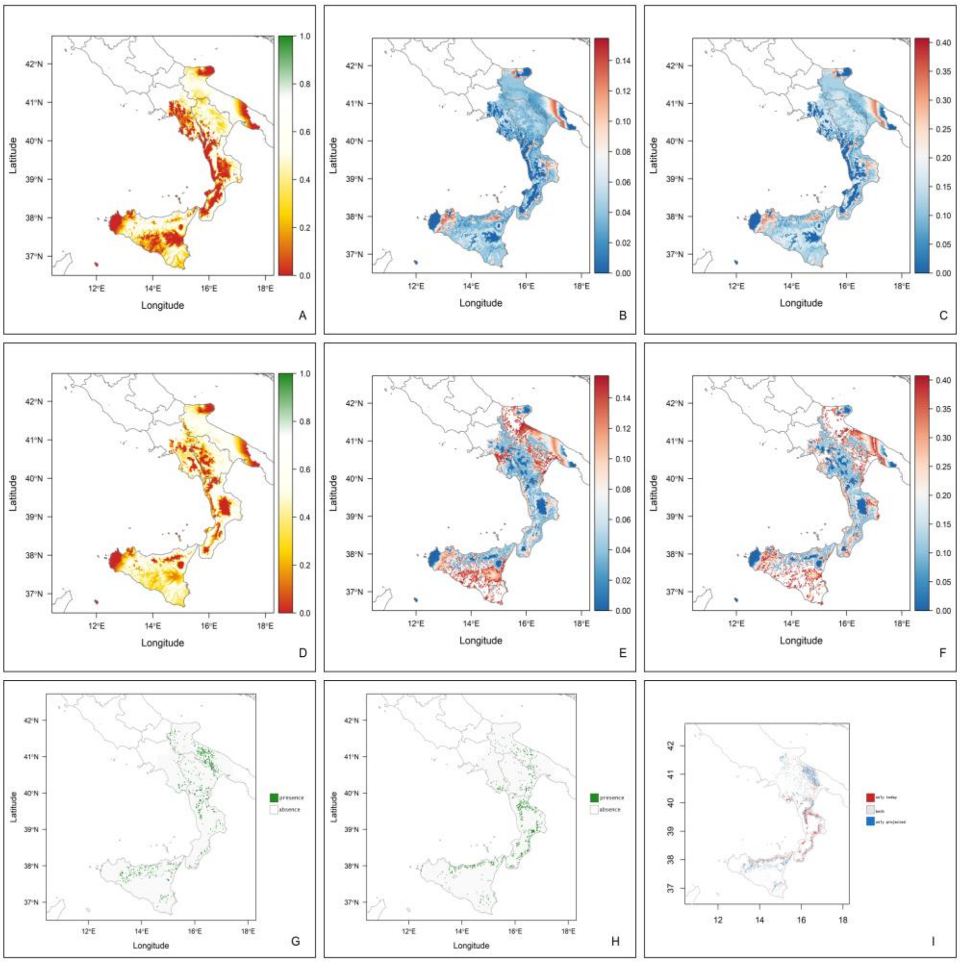

Fig. S.2.123-A) 'Projected' base output; B) 'Projected' uncertainty standard deviation; C) 'Projected' uncertainty range of values; D) 'Present' base output; E) 'Present' uncertainty standard deviation; F) 'Present' uncertainty range of values; G) 'Projected' binary distribution; H) 'Present' binary distribution; I) Range size variation.

Value	Present	Projected
AUC.train	0.760	0.761
AUC.test	0.658	0.708
AUC.diff	0.054	0.054
pROC	1.306	1.360
OR.p10	0.333	0.328
CBI	0.908	0.243
perc.stable	7.314	7.314
range.size	0.728	0.728

S.2.124 *Gypsophila arrostoi* Maxent

Species: *Gypsophila arrostoi* Guss. subsp. *arrostoi*

Procedure: Maxent

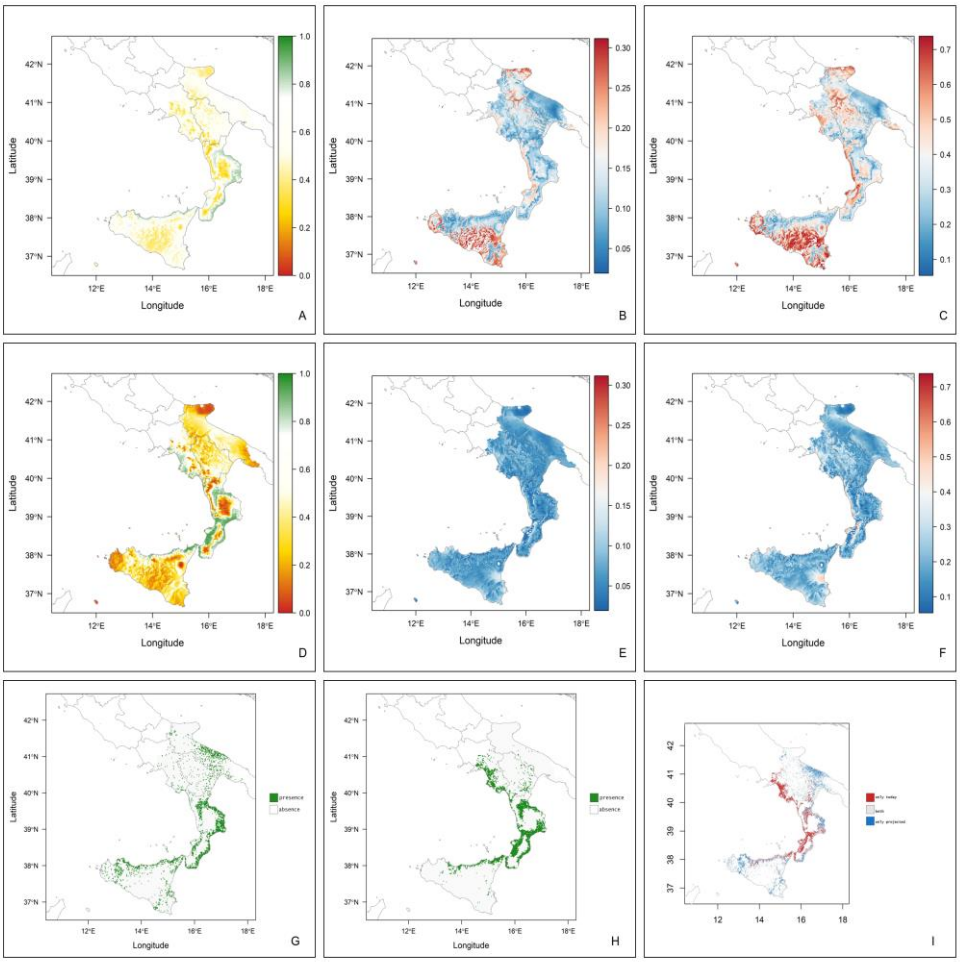

Fig. S.2.124-A) 'Projected' base output; B) 'Projected' uncertainty standard deviation; C) 'Projected' uncertainty range of values; D) 'Present' base output; E) 'Present' uncertainty standard deviation; F) 'Present' uncertainty range of values; G) 'Projected' binary distribution; H) 'Present' binary distribution; I) Range size variation.

Value	Present	Projected
AUC.train	0.805	0.720
AUC.test	0.754	0.526
AUC.diff	0.194	0.194
pROC	1.412	1.261
OR.p10	0.167	0.376
CBI	0.951	0.957
perc.stable	27.618	27.618
range.size	-0.351	-0.351

S.2.125 *Gypsophila arrostoi* KDE

Species: *Gypsophila arrostoi* Guss. subsp. *arrostoi*

Procedure: KDE

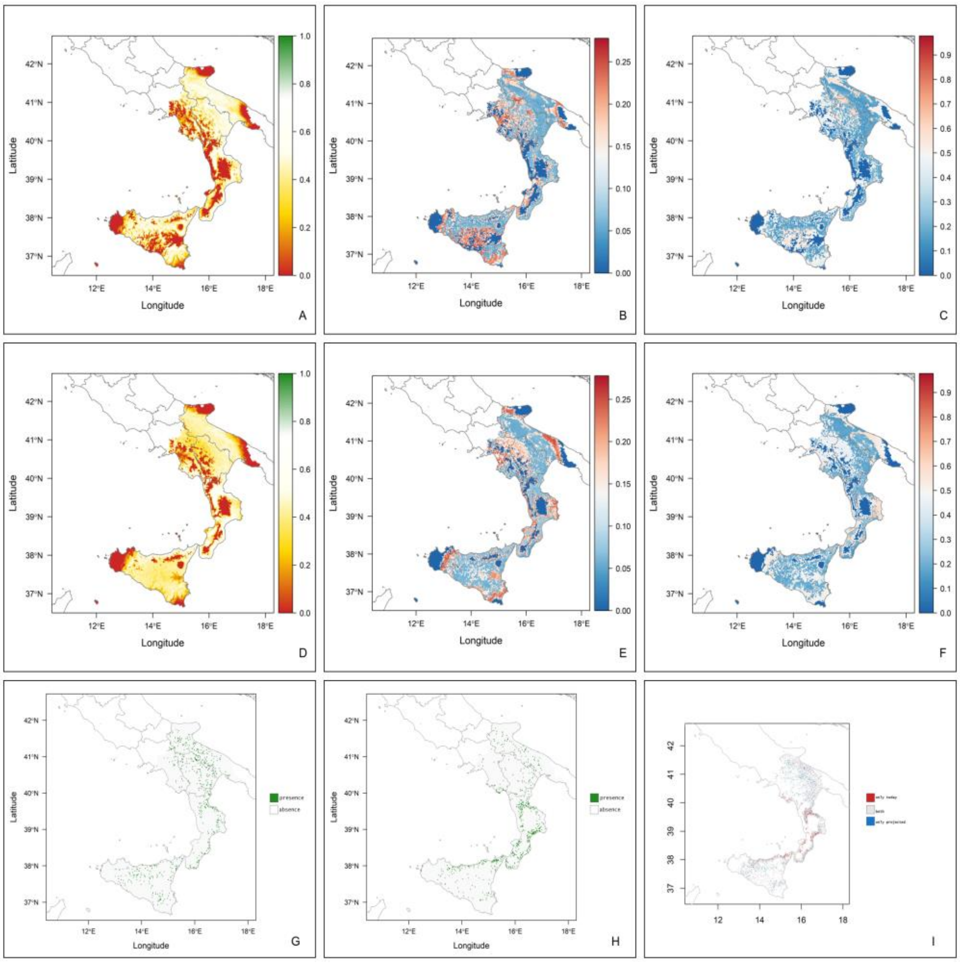

Fig. S.2.125-A) 'Projected' base output; B) 'Projected' uncertainty standard deviation; C) 'Projected' uncertainty range of values; D) 'Present' base output; E) 'Present' uncertainty standard deviation; F) 'Present' uncertainty range of values; G) 'Projected' binary distribution; H) 'Present' binary distribution; I) Range size variation.

Value	Present	Projected
AUC.train	0.830	0.742
AUC.test	0.728	0.694
AUC.diff	0.114	0.050
pROC	1.322	1.291
OR.p10	0.314	0.186
CBI	0.878	0.729
perc.stable	4.396	4.396
range.size	-3.064	-3.064

S.2.126 *Gypsophila arrostoi* CTA

Species: *Gypsophila arrostoi* Guss. subsp. *arrostoi*

Procedure: CTA

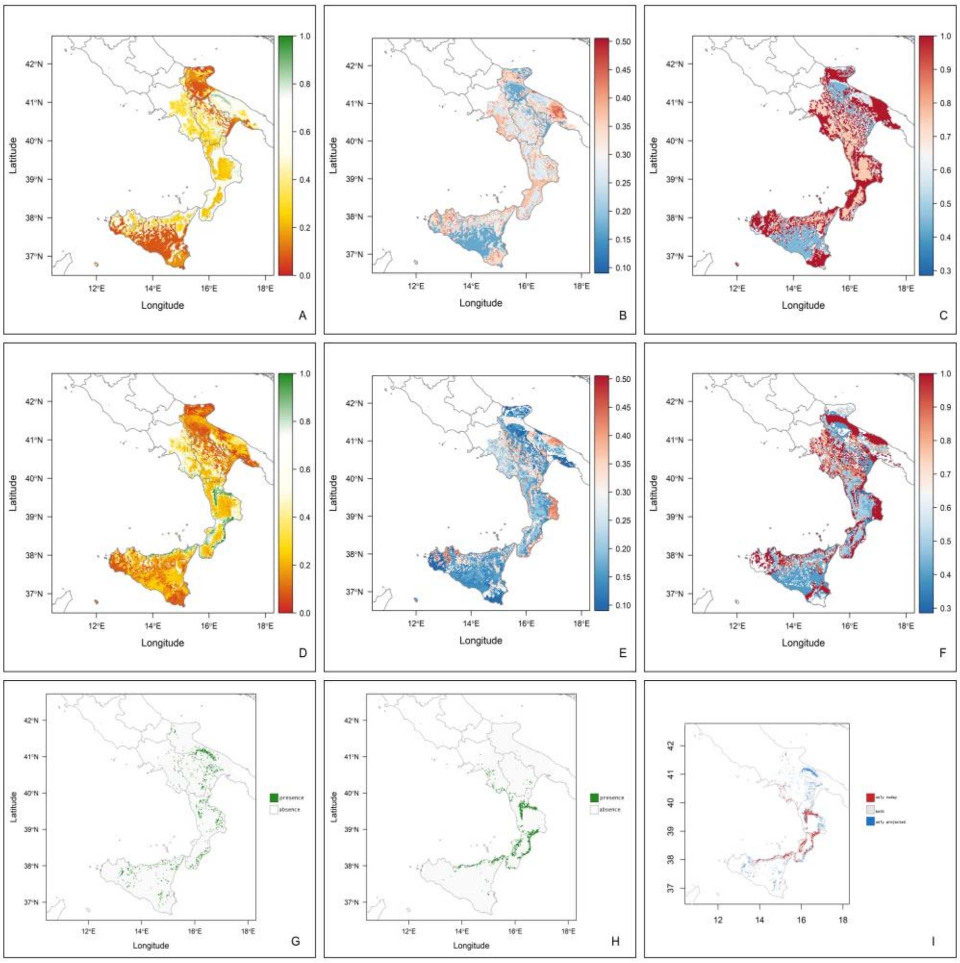

Fig. S.2.126-A) 'Projected' base output; B) 'Projected' uncertainty standard deviation; C) 'Projected' uncertainty range of values; D) 'Present' base output; E) 'Present' uncertainty standard deviation; F) 'Present' uncertainty range of values; G) 'Projected' binary distribution; H) 'Present' binary distribution; I) Range size variation.

Value	Present	Projected
AUC.train	0.912	0.917
AUC.test	0.796	0.764
AUC.diff	0.155	0.155
pROC	1.163	1.180
OR.p10	0.300	0.291
CBI	0.891	0.860
perc.stable	5.191	5.191
range.size	2.634	2.634

S.2.127 *Gypsophila arrostoi* FDA

Species: *Gypsophila arrostoi* Guss. subsp. *arrostoi*

Procedure: FDA

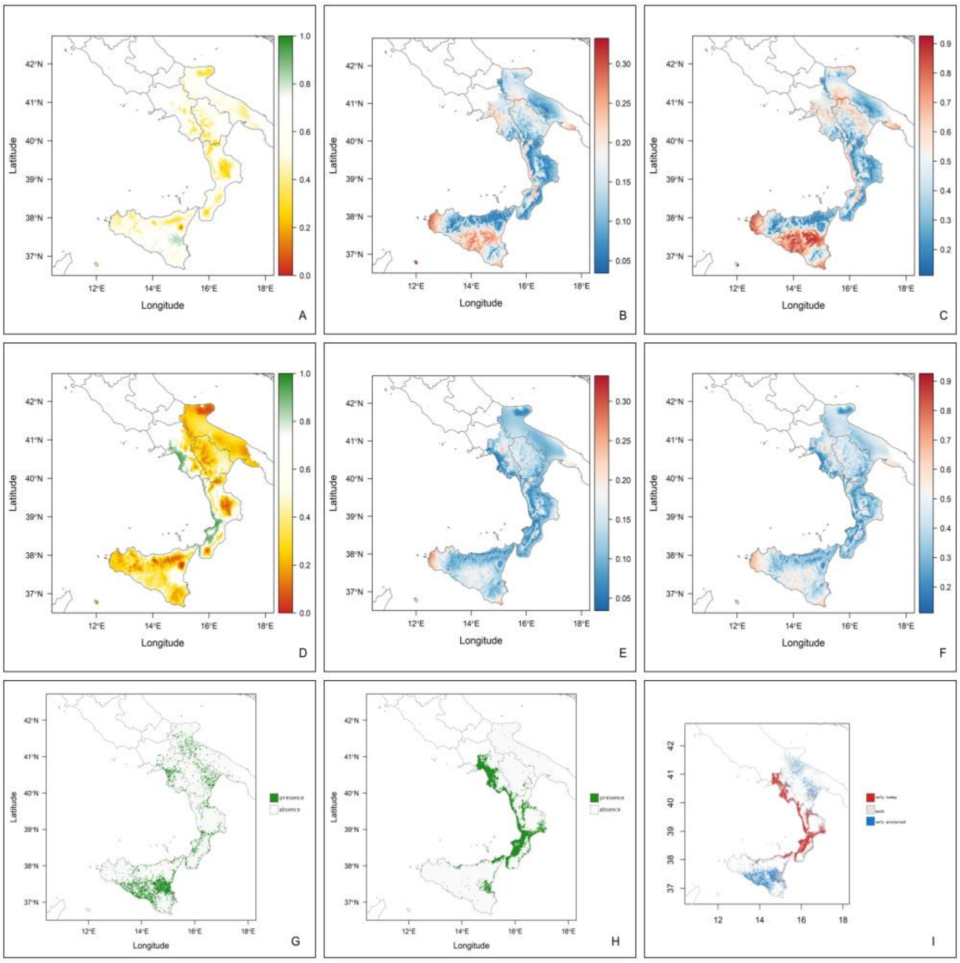

Fig. S.2.127-A) 'Projected' base output; B) 'Projected' uncertainty standard deviation; C) 'Projected' uncertainty range of values; D) 'Present' base output; E) 'Present' uncertainty standard deviation; F) 'Present' uncertainty range of values; G) 'Projected' binary distribution; H) 'Present' binary distribution; I) Range size variation.

Value	Present	Projected
AUC.train	0.753	0.581
AUC.test	0.696	0.578
AUC.diff	0.047	0.047
pROC	1.322	1.226
OR.p10	0.274	0.145
CBI	0.745	0.620
perc.stable	15.049	15.049
range.size	0.640	0.640

S.2.128 *Gypsophila arrostoi* GAM

Species: *Gypsophila arrostoi* Guss. subsp. *arrostoi*

Procedure: GAM

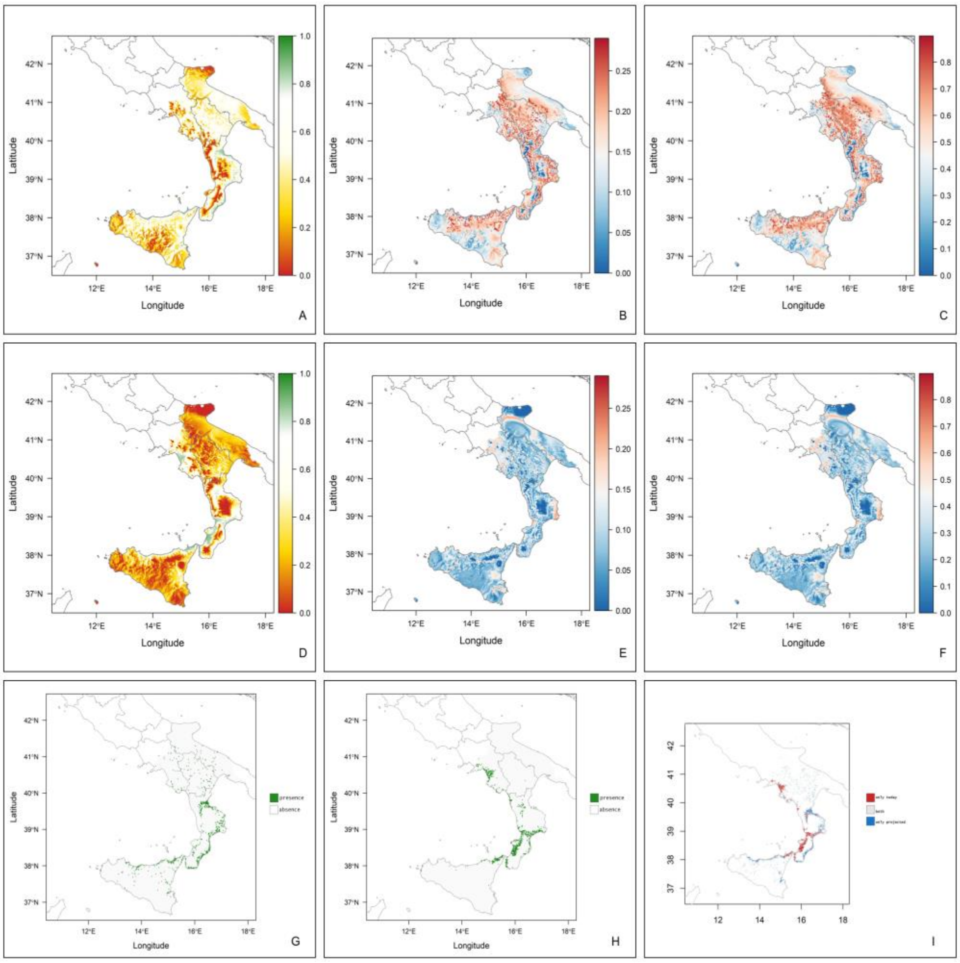

Fig. S.2.128-A) 'Projected' base output; B) 'Projected' uncertainty standard deviation; C) 'Projected' uncertainty range of values; D) 'Present' base output; E) 'Present' uncertainty standard deviation; F) 'Present' uncertainty range of values; G) 'Projected' binary distribution; H) 'Present' binary distribution; I) Range size variation.

Value	Present	Projected
AUC.train	0.799	0.801
AUC.test	0.724	0.711
AUC.diff	0.096	0.096
pROC	1.365	1.379
OR.p10	0.300	0.382
CBI	0.823	0.913
perc.stable	8.978	8.978
range.size	1.307	1.307

S.2.129 *Gypsophila arrostoi* GLM

Species: *Gypsophila arrostoi* Guss. subsp. *arrostoi*

Procedure: GLM

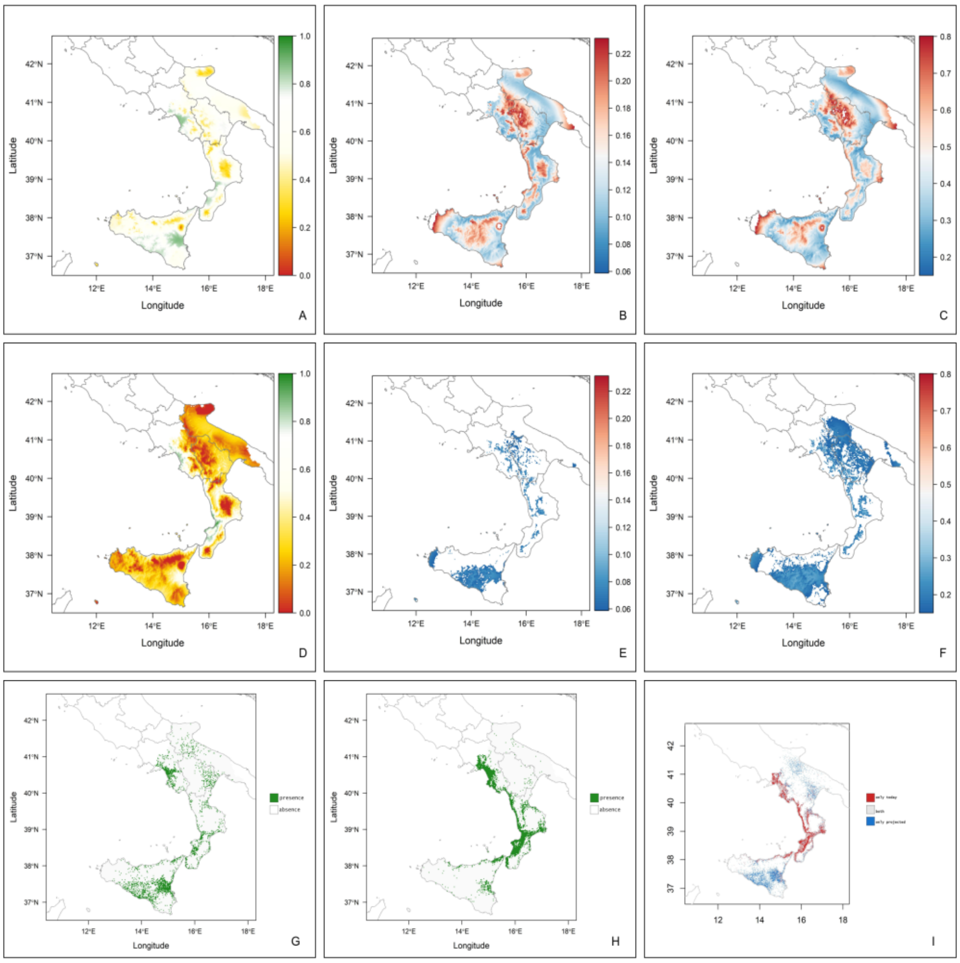

Fig. S.2.129-A) 'Projected' base output; B) 'Projected' uncertainty standard deviation; C) 'Projected' uncertainty range of values; D) 'Present' base output; E) 'Present' uncertainty standard deviation; F) 'Present' uncertainty range of values; G) 'Projected' binary distribution; H) 'Present' binary distribution; I) Range size variation.

Value	Present	Projected
AUC.train	0.723	0.584
AUC.test	0.722	0.436
AUC.diff	0.153	0.153
pROC	1.381	1.156
OR.p10	0.186	0.360
CBI	0.727	0.706
perc.stable	21.744	21.744
range.size	-0.413	-0.413

S.2.130 *Gypsophila arrostoi* MARS

Species: *Gypsophila arrostoi* Guss. subsp. *arrostoi*

Procedure: MARS

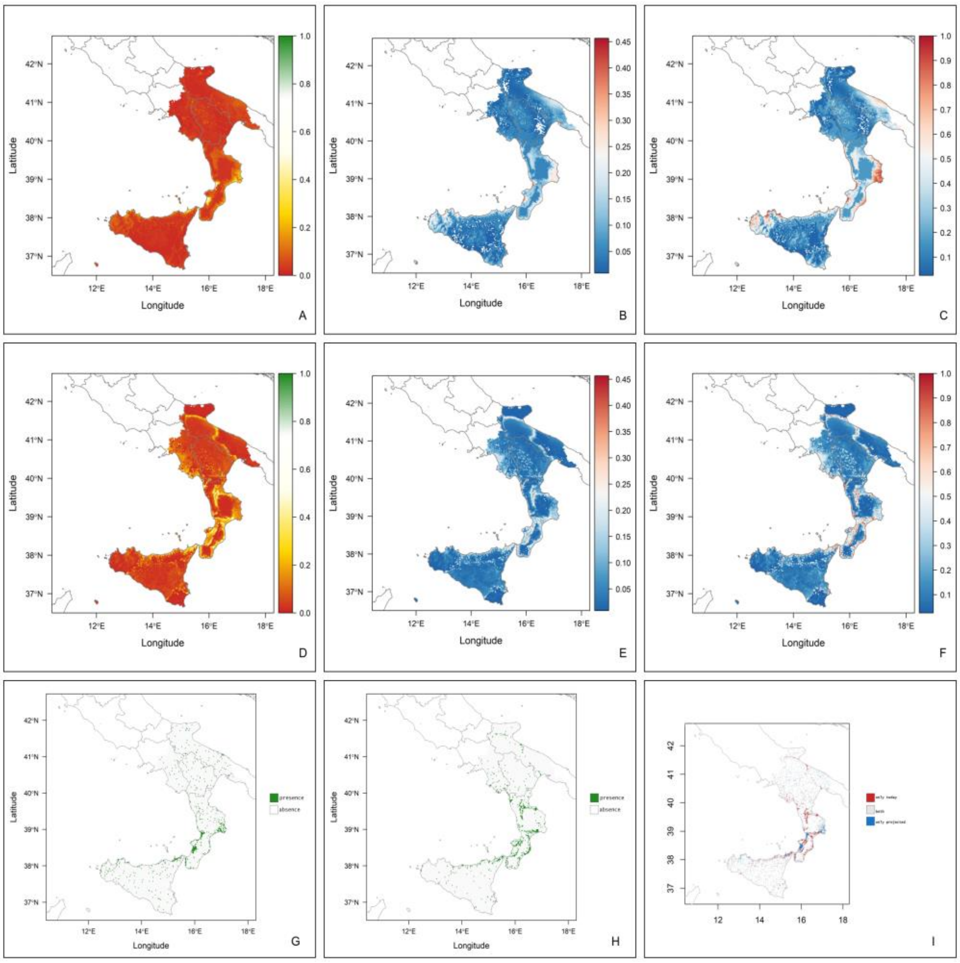

Fig. S.2.130-A) ‘Projected’ base output; B) ‘Projected’ uncertainty standard deviation; C) ‘Projected’ uncertainty range of values; D) ’Present’ base output; E) ’Present’ uncertainty standard deviation; F) ’Present’ uncertainty range of values; G) ‘Projected’ binary distribution; H) ‘Present’ binary distribution; I) Range size variation.

Value	Present	Projected
AUC.train	0.812	0.742
AUC.test	0.647	0.584
AUC.diff	0.167	0.167
pROC	1.209	1.158
OR.p10	0.283	0.289
CBI	0.914	0.480
perc.stable	5.605	5.605
range.size	-1.294	-1.294

S.2.131 *Gypsophila arrostoi* RF

Species: *Gypsophila arrostoi* Guss. subsp. *arrostoi*

Procedure: RF

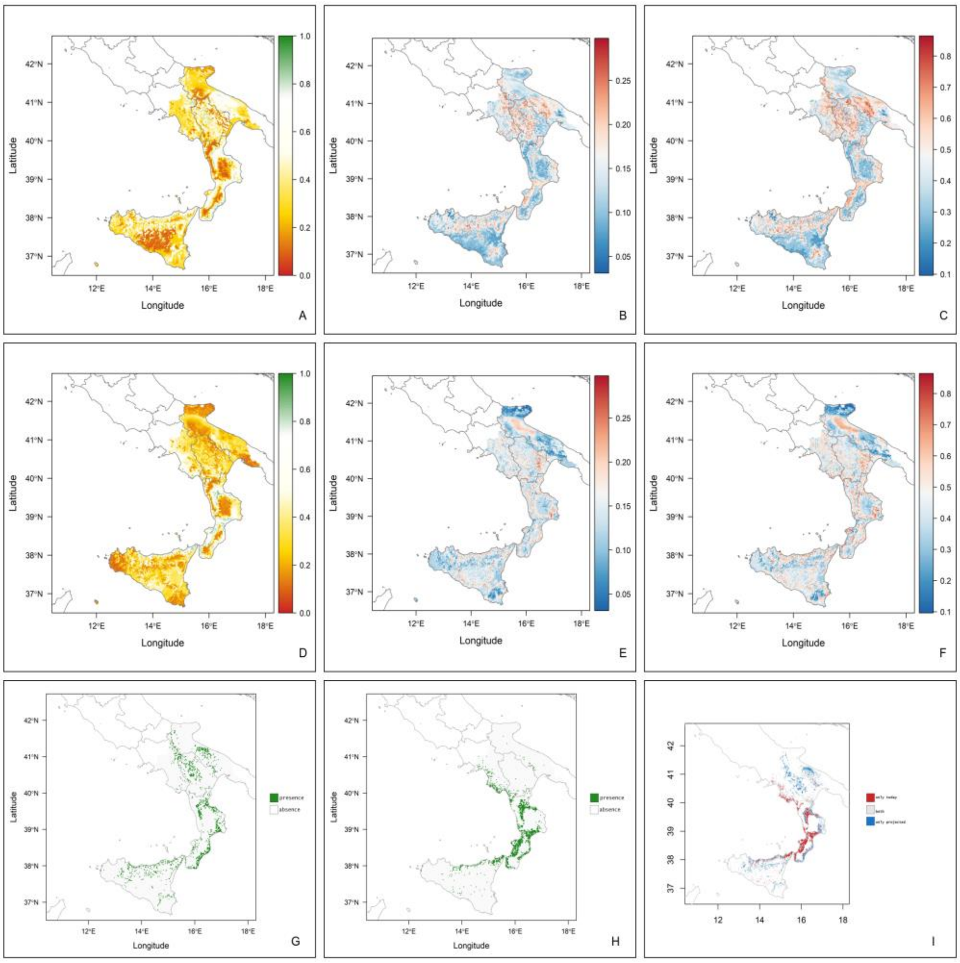

Fig. S.2.131-A) ‘Projected’ base output; B) ‘Projected’ uncertainty standard deviation; C) ‘Projected’ uncertainty range of values; D) ’Present’ base output; E) ’Present’ uncertainty standard deviation; F) ’Present’ uncertainty range of values; G) ‘Projected’ binary distribution; H) ‘Present’ binary distribution; I) Range size variation.

Value	Present	Projected
AUC.train	1.000	1.000
AUC.test	0.931	0.953
AUC.diff	0.047	0.047
pROC	1.338	1.364
OR.p10	0.743	0.874
CBI	0.974	0.771
perc.stable	11.513	11.513
range.size	-0.033	-0.033

S.2.132 *Gypsophila arrostoi* SVM

Species: *Gypsophila arrostoi* Guss. subsp. *arrostoi*

Procedure: SVM

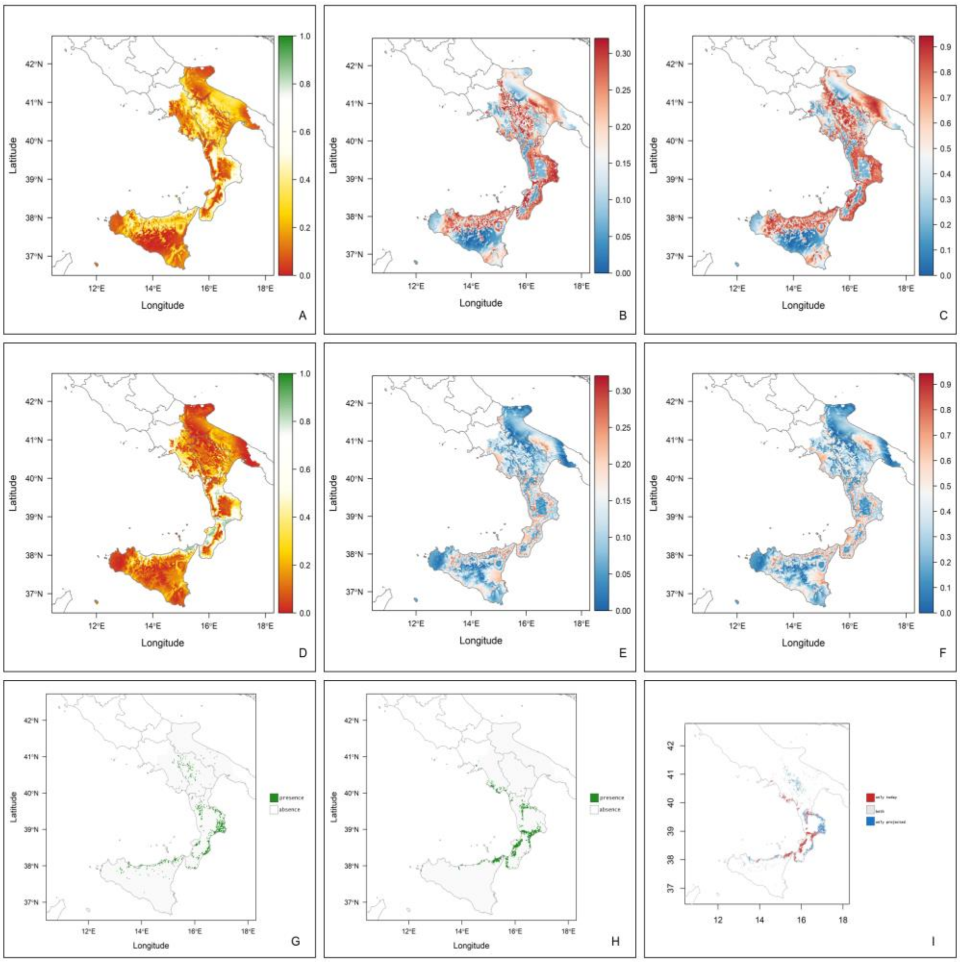

Fig. S.2.132-A) 'Projected' base output; B) 'Projected' uncertainty standard deviation; C) 'Projected' uncertainty range of values; D) 'Present' base output; E) 'Present' uncertainty standard deviation; F) 'Present' uncertainty range of values; G) 'Projected' binary distribution; H) 'Present' binary distribution; I) Range size variation.

Value	Present	Projected
AUC.train	0.877	0.870
AUC.test	0.754	0.770
AUC.diff	0.100	0.100
pROC	1.261	1.262
OR.p10	0.348	0.352
CBI	0.946	0.900
perc.stable	14.682	14.682
range.size	2.549	2.549

S.2.133 *Helleborus viridis* Biomod2

Species: *Helleborus viridis* L. subsp. *bocconei* (Ten.) Peruzzi

Procedure: Biomod2

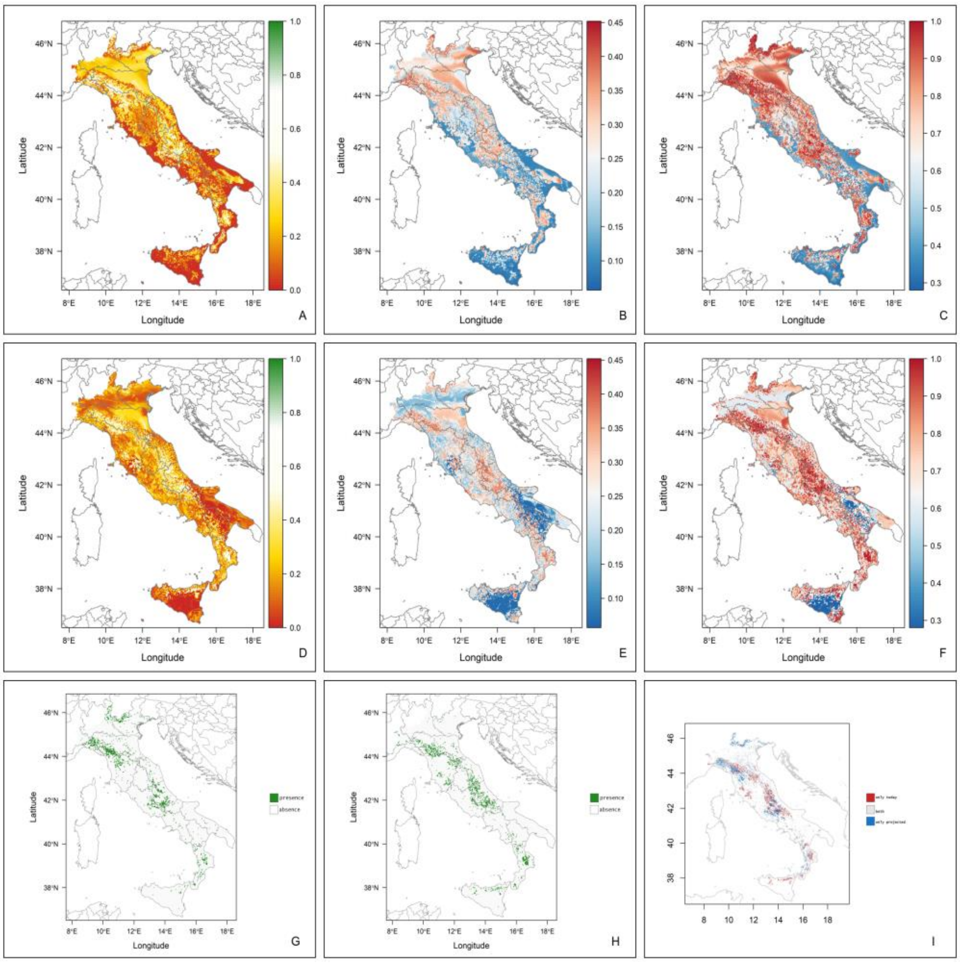

Fig. S.2.133-A) 'Projected' base output; B) 'Projected' uncertainty standard deviation; C) 'Projected' uncertainty range of values; D) 'Present' base output; E) 'Present' uncertainty standard deviation; F) 'Present' uncertainty range of values; G) 'Projected' binary distribution; H) 'Present' binary distribution; I) Range size variation.

Value	Present	Projected
AUC.train	0.996	0.995
AUC.test	0.587	0.753
AUC.diff	0.242	0.242
pROC	1.100	1.316
OR.p10	0.945	0.936
CBI	0.983	0.828
perc.stable	12.584	12.584
range.size	1.794	1.794

S.2.134 *Helleborus viridis* Bioclim

Species: *Helleborus viridis* L. subsp. *bocconei* (Ten.) Peruzzi

Procedure: Bioclim

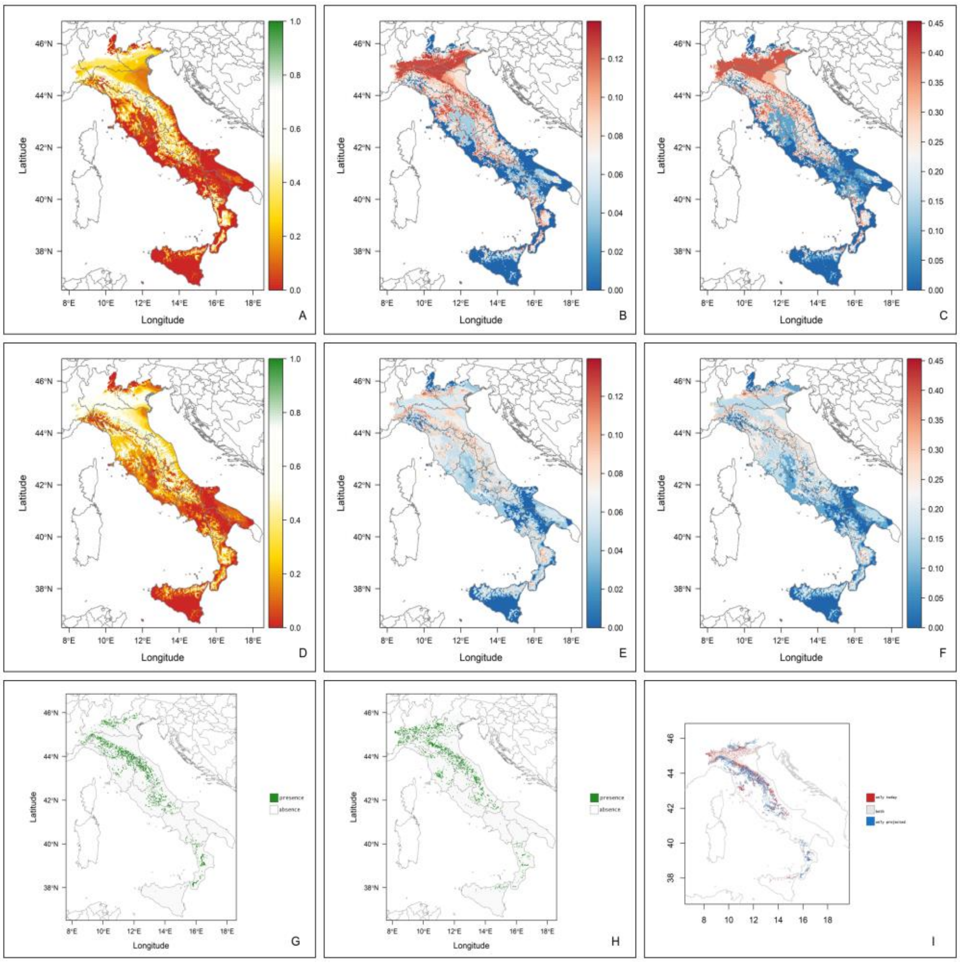

Fig. S.2.134-A) ‘Projected’ base output; B) ‘Projected’ uncertainty standard deviation; C) ‘Projected’ uncertainty range of values; D) ’Present’ base output; E) ’Present’ uncertainty standard deviation; F) ’Present’ uncertainty range of values; G) ‘Projected’ binary distribution; H) ‘Present’ binary distribution; I) Range size variation.

Value	Present	Projected
AUC.train	0.629	0.632
AUC.test	0.614	0.595
AUC.diff	0.067	0.067
pROC	1.134	1.117
OR.p10	0.118	0.182
CBI	0.654	0.807
perc.stable	10.288	10.288
range.size	0.923	0.923

S.2.135 *Helleborus viridis* Domain

Species: *Helleborus viridis* L. subsp. *bocconei* (Ten.) Peruzzi

Procedure: Domain

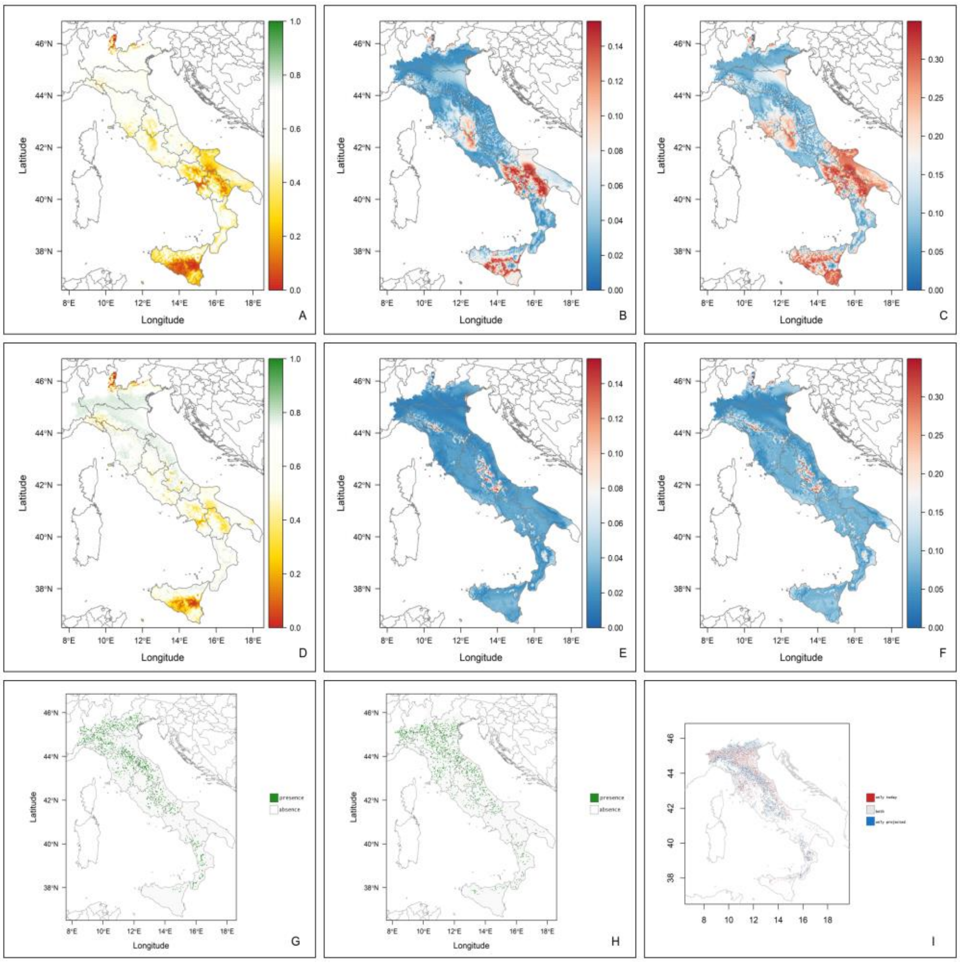

Fig. S.2.135-A) 'Projected' base output; B) 'Projected' uncertainty standard deviation; C) 'Projected' uncertainty range of values; D) 'Present' base output; E) 'Present' uncertainty standard deviation; F) 'Present' uncertainty range of values; G) 'Projected' binary distribution; H) 'Present' binary distribution; I) Range size variation.

Value	Present	Projected
AUC.train	0.612	0.627
AUC.test	0.559	0.546
AUC.diff	0.090	0.090
pROC	1.137	1.143
OR.p10	0.218	0.218
CBI	0.833	0.895
perc.stable	5.977	5.977
range.size	1.740	1.740

S.2.136 *Helleborus viridis* Maxent

Species: *Helleborus viridis* L. subsp. *bocconei* (Ten.) Peruzzi

Procedure: Maxent

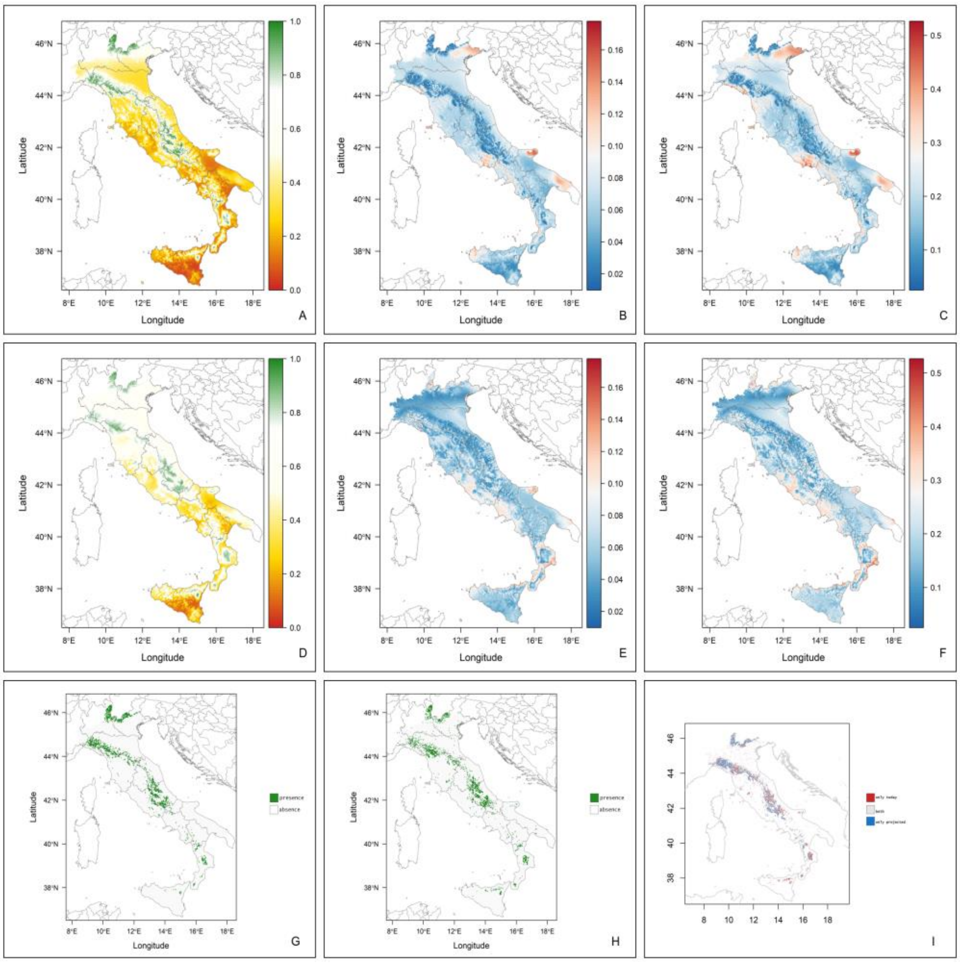

Fig. S.2.136-A) 'Projected' base output; B) 'Projected' uncertainty standard deviation; C) 'Projected' uncertainty range of values; D) 'Present' base output; E) 'Present' uncertainty standard deviation; F) 'Present' uncertainty range of values; G) 'Projected' binary distribution; H) 'Present' binary distribution; I) Range size variation.

Value	Present	Projected
AUC.train	0.700	0.741
AUC.test	0.667	0.680
AUC.diff	0.072	0.072
pROC	1.195	1.222
OR.p10	0.145	0.236
CBI	0.945	0.949
perc.stable	29.192	29.192
range.size	-1.302	-1.302

S.2.137 *Helleborus viridis* KDE

Species: *Helleborus viridis* L. subsp. *bocconei* (Ten.) Peruzzi

Procedure: KDE

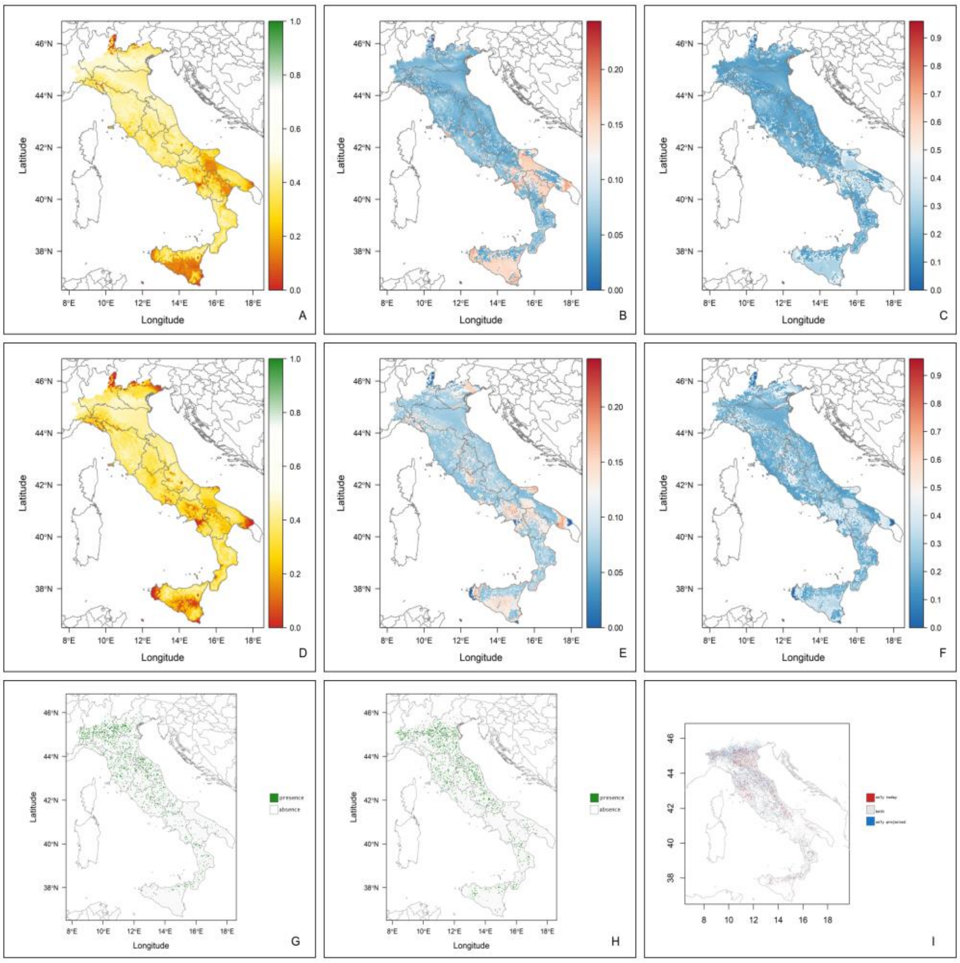

Fig. S.2.137-A) 'Projected' base output; B) 'Projected' uncertainty standard deviation; C) 'Projected' uncertainty range of values; D) 'Present' base output; E) 'Present' uncertainty standard deviation; F) 'Present' uncertainty range of values; G) 'Projected' binary distribution; H) 'Present' binary distribution; I) Range size variation.

Value	Present	Projected
AUC.train	0.637	0.598
AUC.test	0.501	0.496
AUC.diff	0.149	0.102
pROC	1.076	1.118
OR.p10	0.309	0.209
CBI	0.932	0.854
perc.stable	5.829	5.829
range.size	-0.079	-0.079

S.2.138 *Helleborus viridis* CTA

Species: *Helleborus viridis* L. subsp. *bocconei* (Ten.) Peruzzi

Procedure: CTA

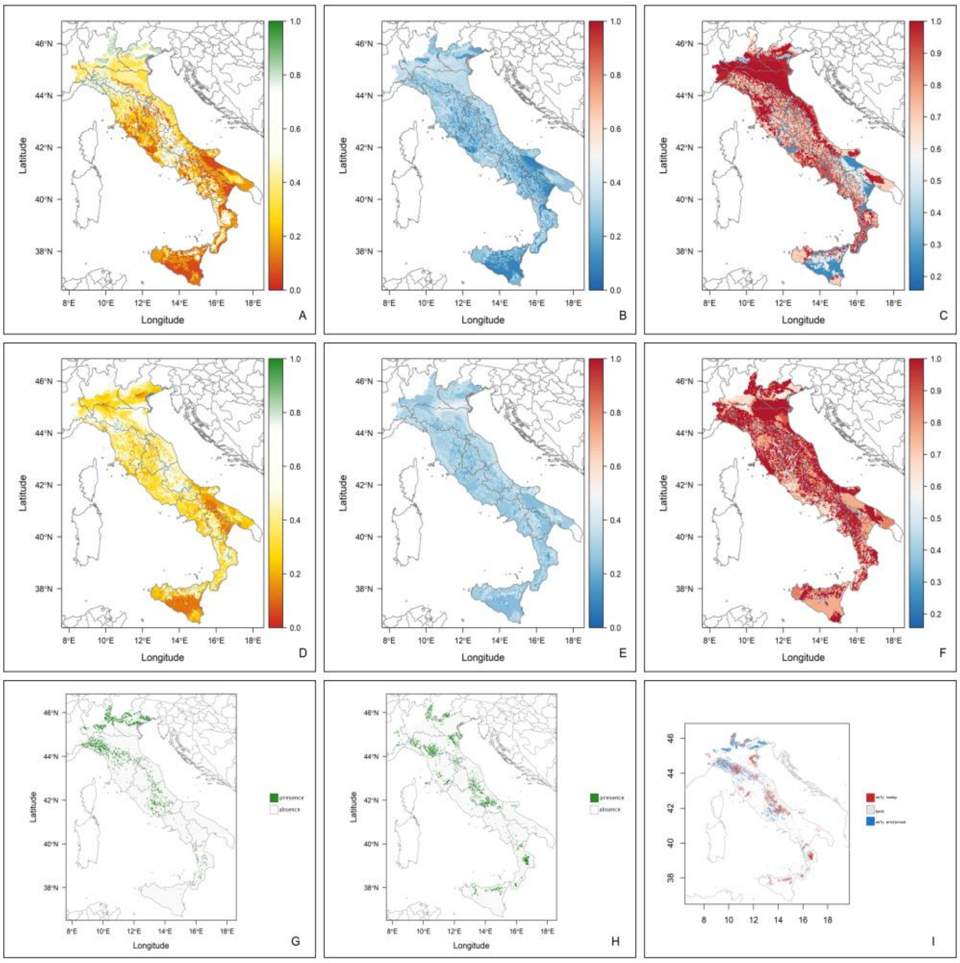

Fig. S.2.138-A) 'Projected' base output; B) 'Projected' uncertainty standard deviation; C) 'Projected' uncertainty range of values; D) 'Present' base output; E) 'Present' uncertainty standard deviation; F) 'Present' uncertainty range of values; G) 'Projected' binary distribution; H) 'Present' binary distribution; I) Range size variation.

Value	Present	Projected
AUC.train	0.890	0.886
AUC.test	0.701	0.750
AUC.diff	0.136	0.136
pROC	1.071	1.133
OR.p10	0.373	0.273
CBI	0.958	0.794
perc.stable	7.256	7.256
range.size	-0.744	-0.744

S.2.139 *Helleborus viridis* FDA

Species: *Helleborus viridis* L. subsp. *bocconei* (Ten.) Peruzzi

Procedure: FDA

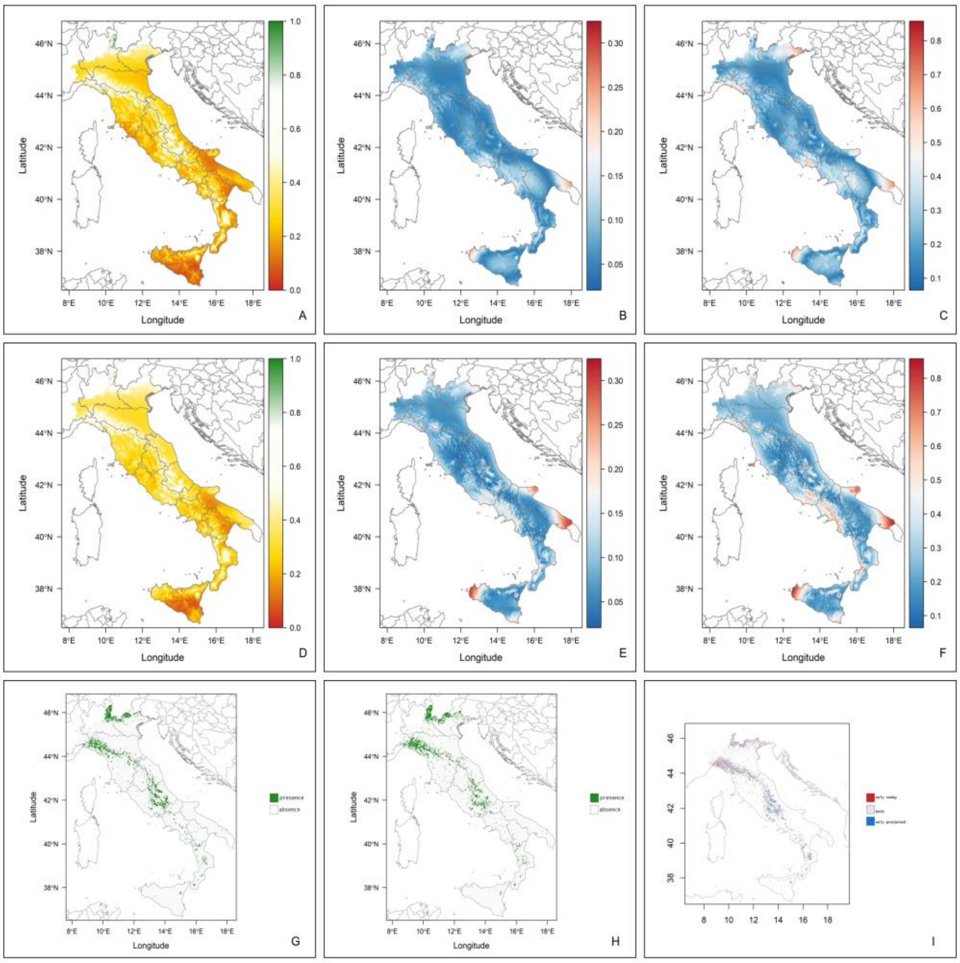

Fig. S.2.139-A) 'Projected' base output; B) 'Projected' uncertainty standard deviation; C) 'Projected' uncertainty range of values; D) 'Present' base output; E) 'Present' uncertainty standard deviation; F) 'Present' uncertainty range of values; G) 'Projected' binary distribution; H) 'Present' binary distribution; I) Range size variation.

Value	Present	Projected
AUC.train	0.634	0.699
AUC.test	0.659	0.672
AUC.diff	0.049	0.049
pROC	1.196	1.205
OR.p10	0.127	0.236
CBI	0.590	0.475
perc.stable	38.288	38.288
range.size	-0.853	-0.853

S.2.140 *Helleborus viridis* GAM

Species: *Helleborus viridis* L. subsp. *bocconei* (Ten.) Peruzzi

Procedure: GAM

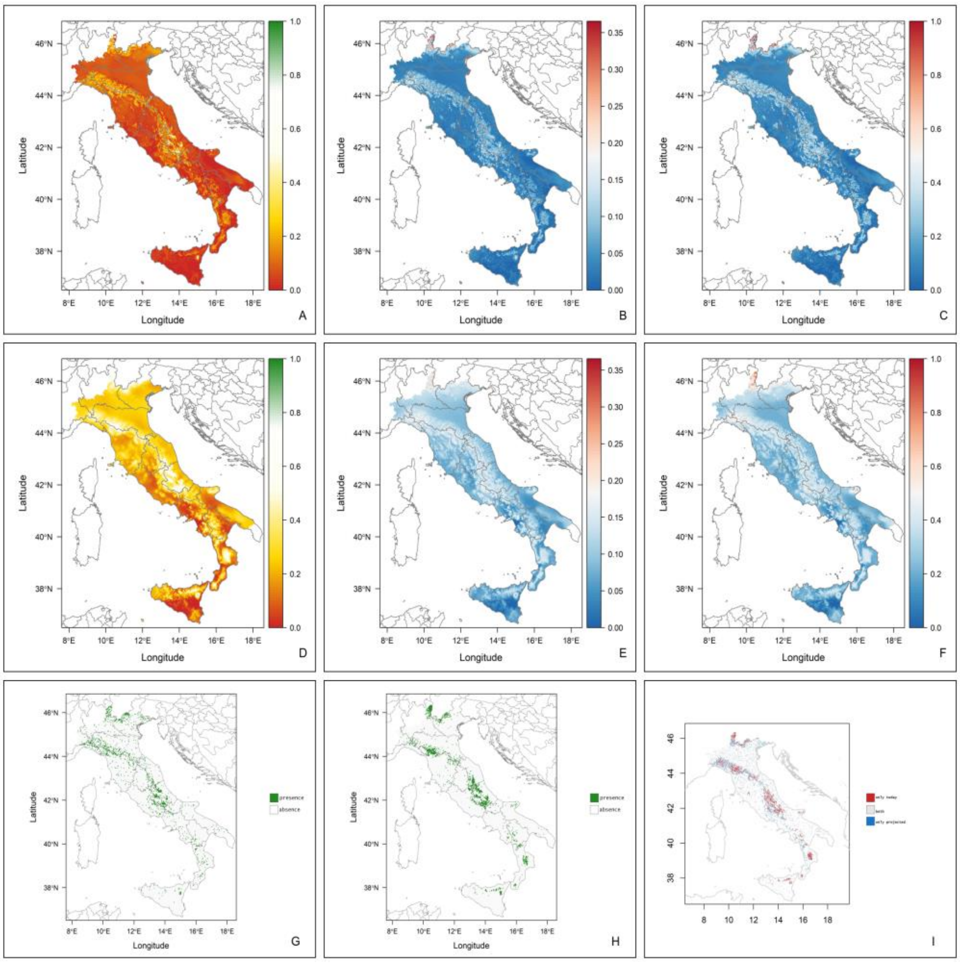

Fig. S.2.140-A) ‘Projected’ base output; B) ‘Projected’ uncertainty standard deviation; C) ‘Projected’ uncertainty range of values; D) ’Present’ base output; E) ’Present’ uncertainty standard deviation; F) ’Present’ uncertainty range of values; G) ‘Projected’ binary distribution; H) ‘Present’ binary distribution; I) Range size variation.

Value	Present	Projected
AUC.train	0.684	0.800
AUC.test	0.673	0.641
AUC.diff	0.159	0.159
pROC	1.178	1.186
OR.p10	0.164	0.355
CBI	0.668	0.451
perc.stable	19.002	19.002
range.size	1.792	1.792

S.2.141 *Helleborus viridis* GLM

Species: *Helleborus viridis* L. subsp. *bocconei* (Ten.) Peruzzi

Procedure: GLM

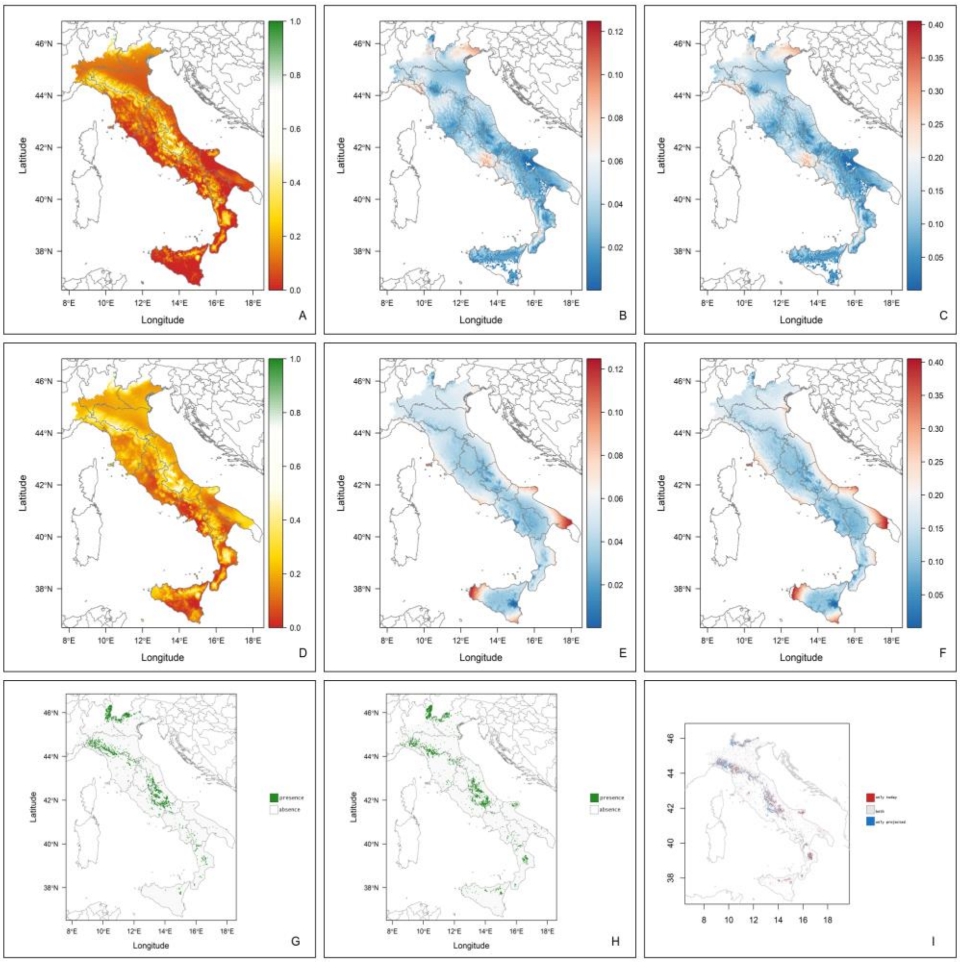

Fig. S.2.141-A) 'Projected' base output; B) 'Projected' uncertainty standard deviation; C) 'Projected' uncertainty range of values; D) 'Present' base output; E) 'Present' uncertainty standard deviation; F) 'Present' uncertainty range of values; G) 'Projected' binary distribution; H) 'Present' binary distribution; I) Range size variation.

Value	Present	Projected
AUC.train	0.670	0.728
AUC.test	0.675	0.673
AUC.diff	0.072	0.072
pROC	1.184	1.211
OR.p10	0.118	0.200
CBI	0.533	0.120
perc.stable	31.524	31.524
range.size	0.243	0.243

S.2.142 *Helleborus viridis* MARS

Species: *Helleborus viridis* L. subsp. *bocconei* (Ten.) Peruzzi

Procedure: MARS

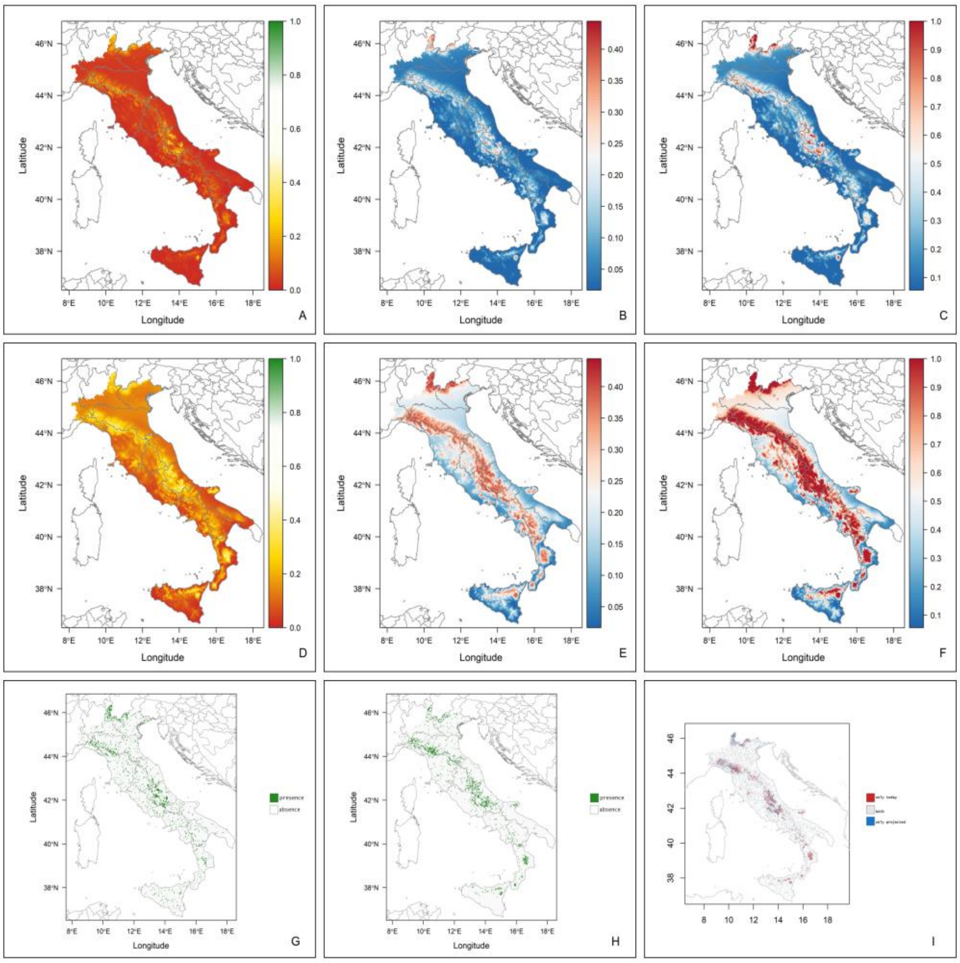

Fig. S.2.142-A) 'Projected' base output; B) 'Projected' uncertainty standard deviation; C) 'Projected' uncertainty range of values; D) 'Present' base output; E) 'Present' uncertainty standard deviation; F) 'Present' uncertainty range of values; G) 'Projected' binary distribution; H) 'Present' binary distribution; I) Range size variation.

Value	Present	Projected
AUC.train	0.745	0.728
AUC.test	0.628	0.628
AUC.diff	0.102	0.102
pROC	1.148	1.106
OR.p10	0.255	0.200
CBI	0.735	0.415
perc.stable	10.610	10.610
range.size	0.209	0.209

S.2.143 *Helleborus viridis* RF

Species: *Helleborus viridis* L. subsp. *bocconei* (Ten.) Peruzzi

Procedure: RF

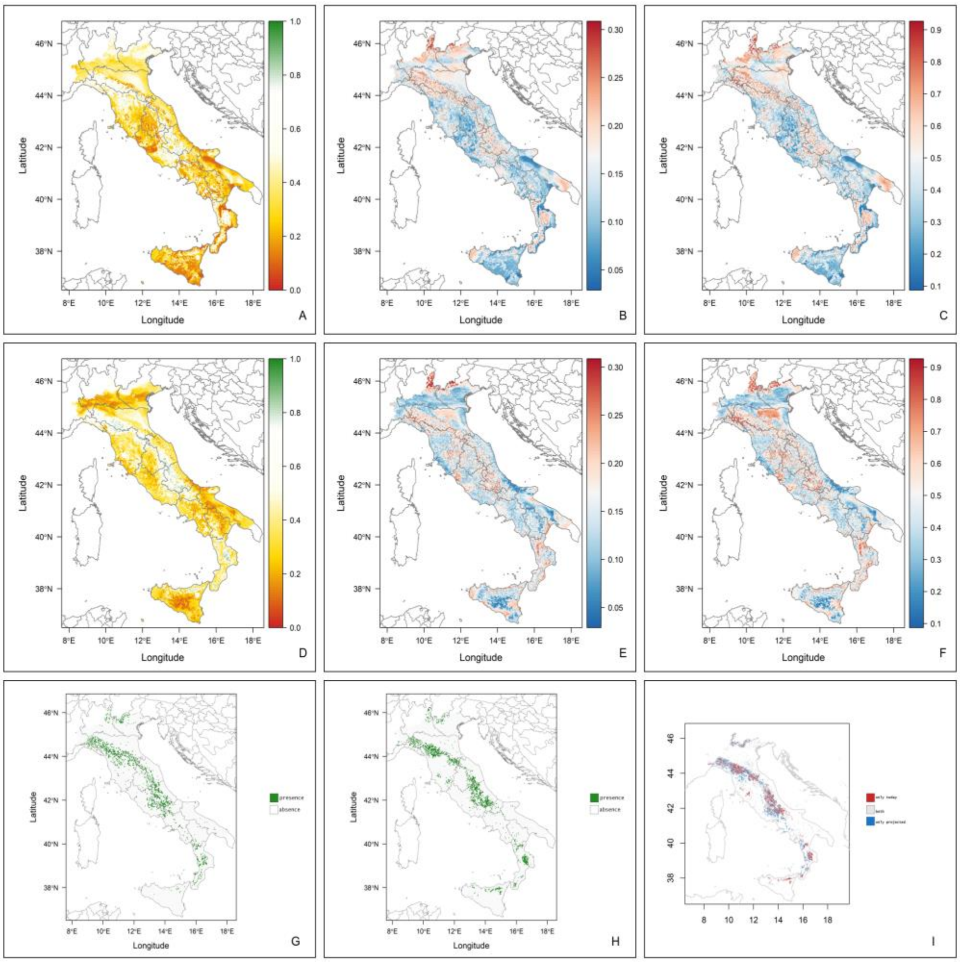

Fig. S.2.143-A) 'Projected' base output; B) 'Projected' uncertainty standard deviation; C) 'Projected' uncertainty range of values; D) 'Present' base output; E) 'Present' uncertainty standard deviation; F) 'Present' uncertainty range of values; G) 'Projected' binary distribution; H) 'Present' binary distribution; I) Range size variation.

Value	Present	Projected
AUC.train	1.000	1.000
AUC.test	0.926	0.943
AUC.diff	0.057	0.057
pROC	1.188	1.245
OR.p10	0.791	0.764
CBI	0.991	0.930
perc.stable	16.630	16.630
range.size	0.797	0.797

S.2.144 *Helleborus viridis* SVM

Species: *Helleborus viridis* L. subsp. *bocconei* (Ten.) Peruzzi

Procedure: SVM

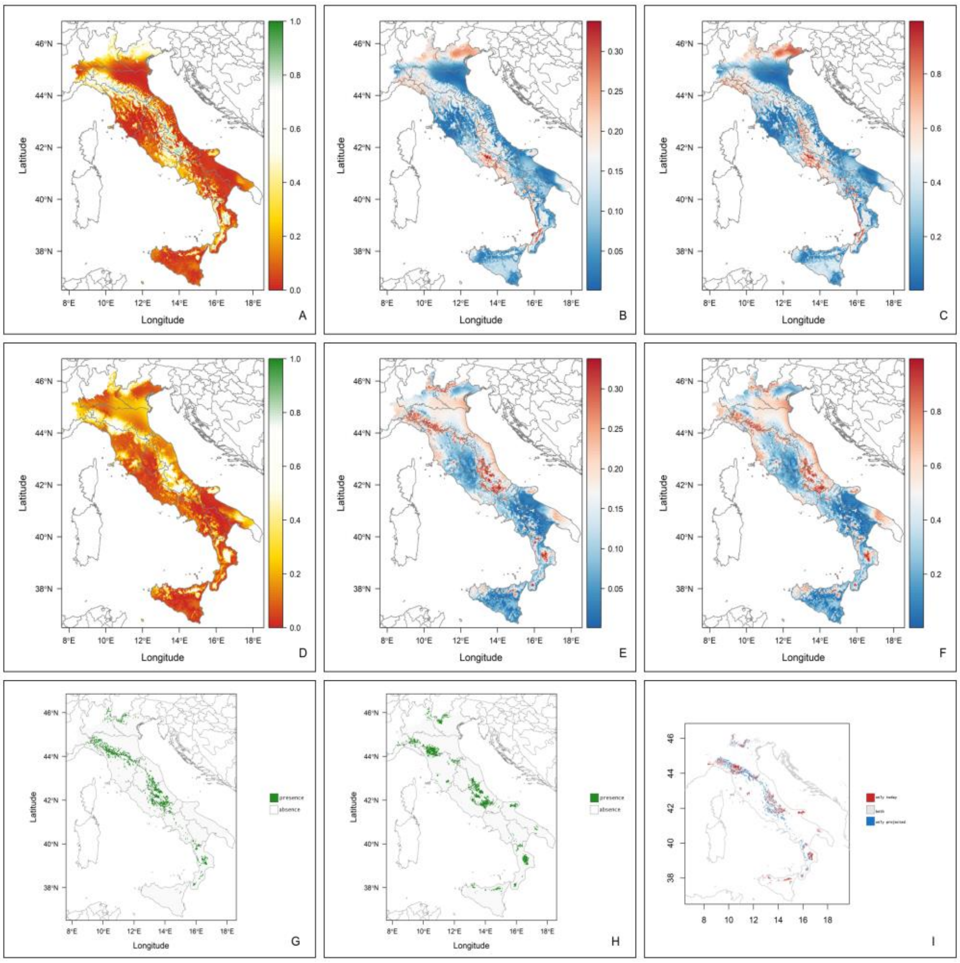

Fig. S.2.144-A) 'Projected' base output; B) 'Projected' uncertainty standard deviation; C) 'Projected' uncertainty range of values; D) 'Present' base output; E) 'Present' uncertainty standard deviation; F) 'Present' uncertainty range of values; G) 'Projected' binary distribution; H) 'Present' binary distribution; I) Range size variation.

Value	Present	Projected
AUC.train	0.798	0.802
AUC.test	0.738	0.762
AUC.diff	0.053	0.053
pROC	1.195	1.228
OR.p10	0.209	0.173
CBI	0.866	0.809
perc.stable	27.394	27.394
range.size	-0.170	-0.170

S.2.145 *Klasea flavescens* Biomod2

Species: *Klasea flavescens* (L.) Holub subsp. *cichoracea* (L.) Greuter & Wagenitz

Procedure: Biomod2

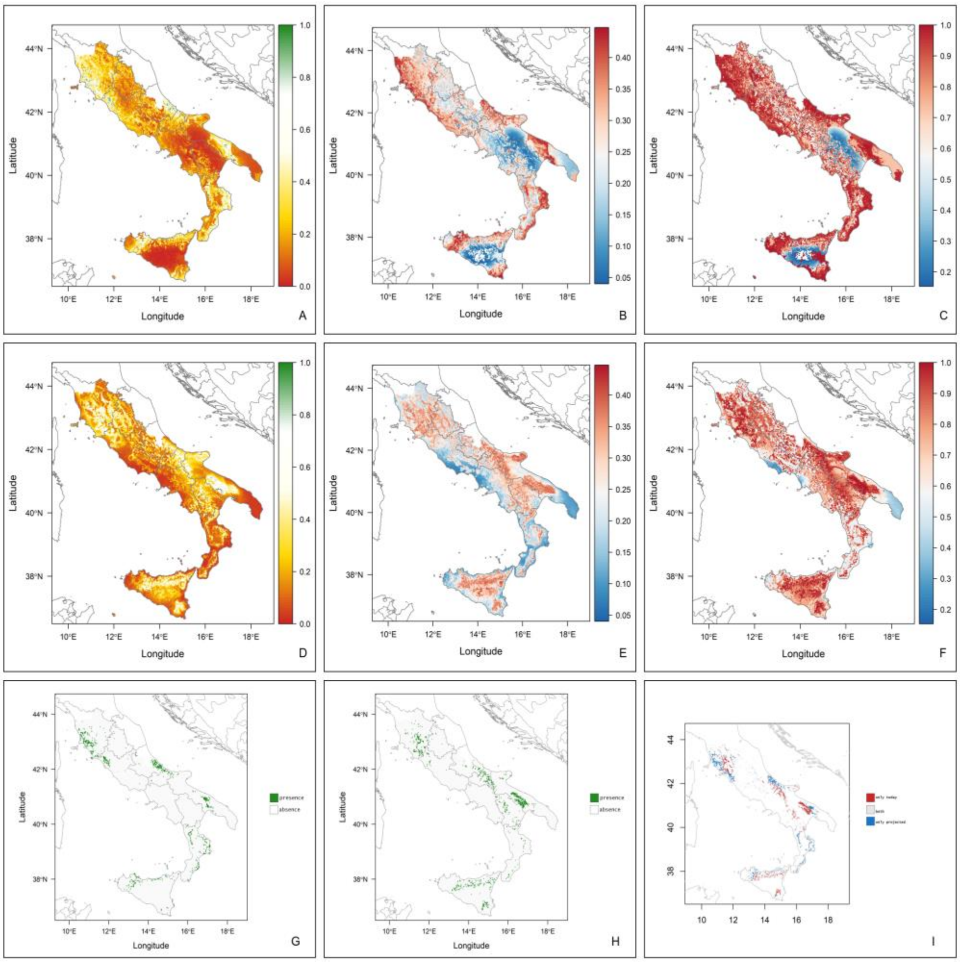

Fig. S.2.145-A) 'Projected' base output; B) 'Projected' uncertainty standard deviation; C) 'Projected' uncertainty range of values; D) 'Present' base output; E) 'Present' uncertainty standard deviation; F) 'Present' uncertainty range of values; G) 'Projected' binary distribution; H) 'Present' binary distribution; I) Range size variation.

Value	Present	Projected
AUC.train	0.996	0.999
AUC.test	0.599	0.727
AUC.diff	0.272	0.272
pROC	1.250	1.352
OR.p10	1.000	0.850
CBI	0.984	0.356
perc.stable	1.660	1.660
range.size	0.288	0.288

S.2.146 *Klasea flavescens* Bioclim

Species: *Klasea flavescens* (L.) Holub subsp. *cichoracea* (L.) Greuter & Wagenitz

Procedure: Bioclim

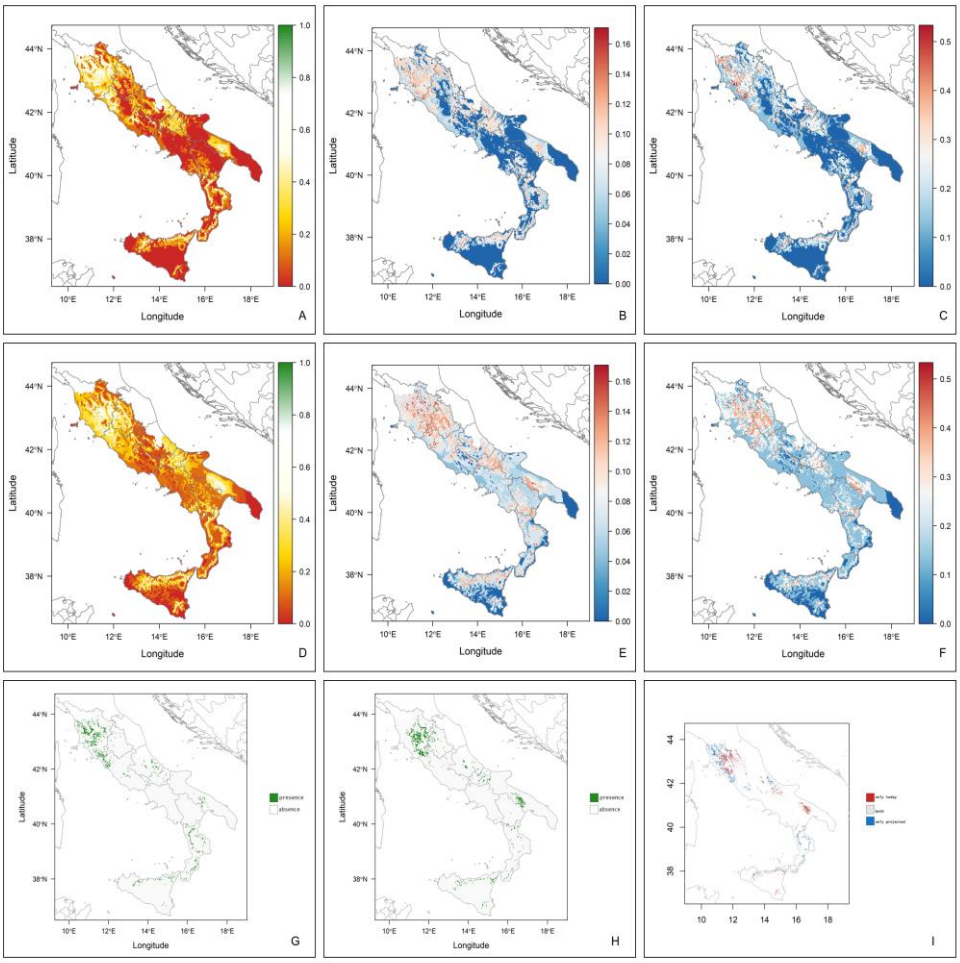

Fig. S.2.146-A) 'Projected' base output; B) 'Projected' uncertainty standard deviation; C) 'Projected' uncertainty range of values; D) 'Present' base output; E) 'Present' uncertainty standard deviation; F) 'Present' uncertainty range of values; G) 'Projected' binary distribution; H) 'Present' binary distribution; I) Range size variation.

Value	Present	Projected
AUC.train	0.666	0.746
AUC.test	0.589	0.702
AUC.diff	0.084	0.084
pROC	1.165	1.275
OR.p10	0.167	0.200
CBI	0.762	0.568
perc.stable	20.509	20.509
range.size	-2.813	-2.813

S.2.147 *Klasea flavescens* Domain

Species: *Klasea flavescens* (L.) Holub subsp. *cichoracea* (L.) Greuter & Wagenitz

Procedure: Domain

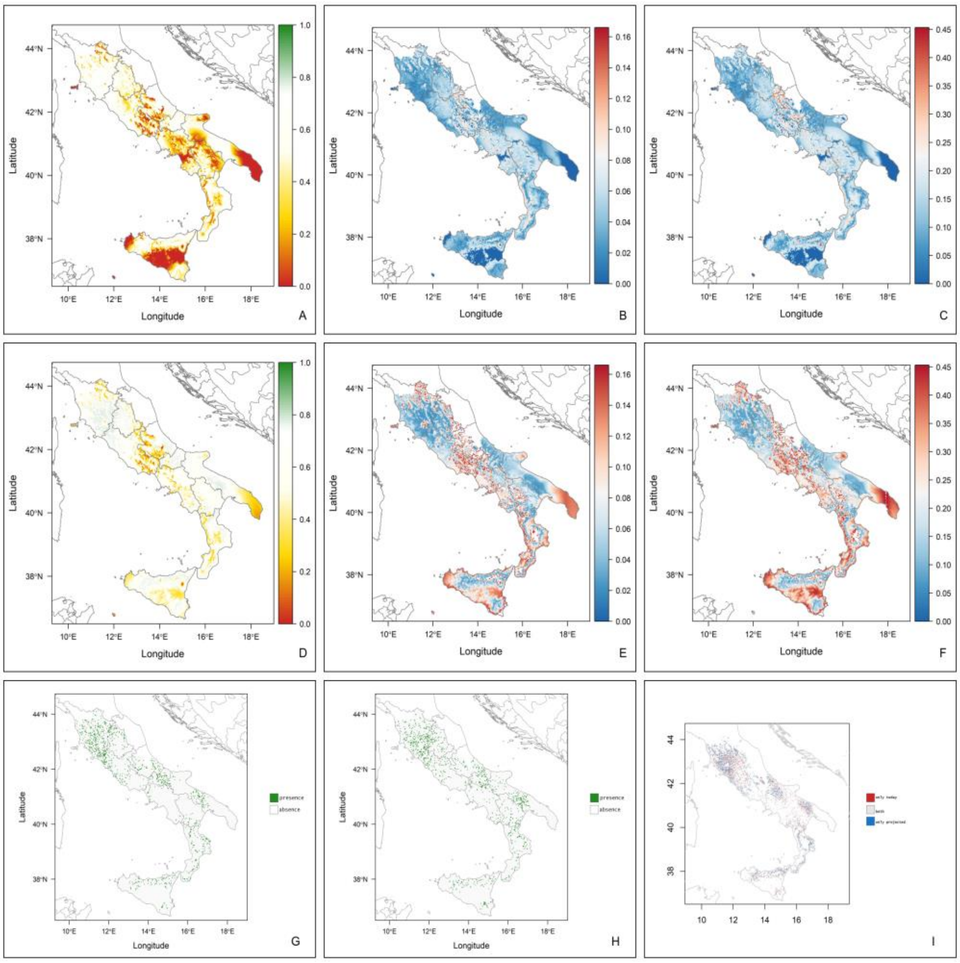

Fig. S.2.147-A) 'Projected' base output; B) 'Projected' uncertainty standard deviation; C) 'Projected' uncertainty range of values; D) 'Present' base output; E) 'Present' uncertainty standard deviation; F) 'Present' uncertainty range of values; G) 'Projected' binary distribution; H) 'Present' binary distribution; I) Range size variation.

Value	Present	Projected
AUC.train	0.618	0.723
AUC.test	0.585	0.672
AUC.diff	0.072	0.072
pROC	1.250	1.338
OR.p10	0.283	0.300
CBI	0.046	0.538
perc.stable	7.348	7.348
range.size	2.003	2.003

S.2.148 *Klasea flavescens* Maxent

Species: *Klasea flavescens* (L.) Holub subsp. *cichoracea* (L.) Greuter & Wagenitz

Procedure: Maxent

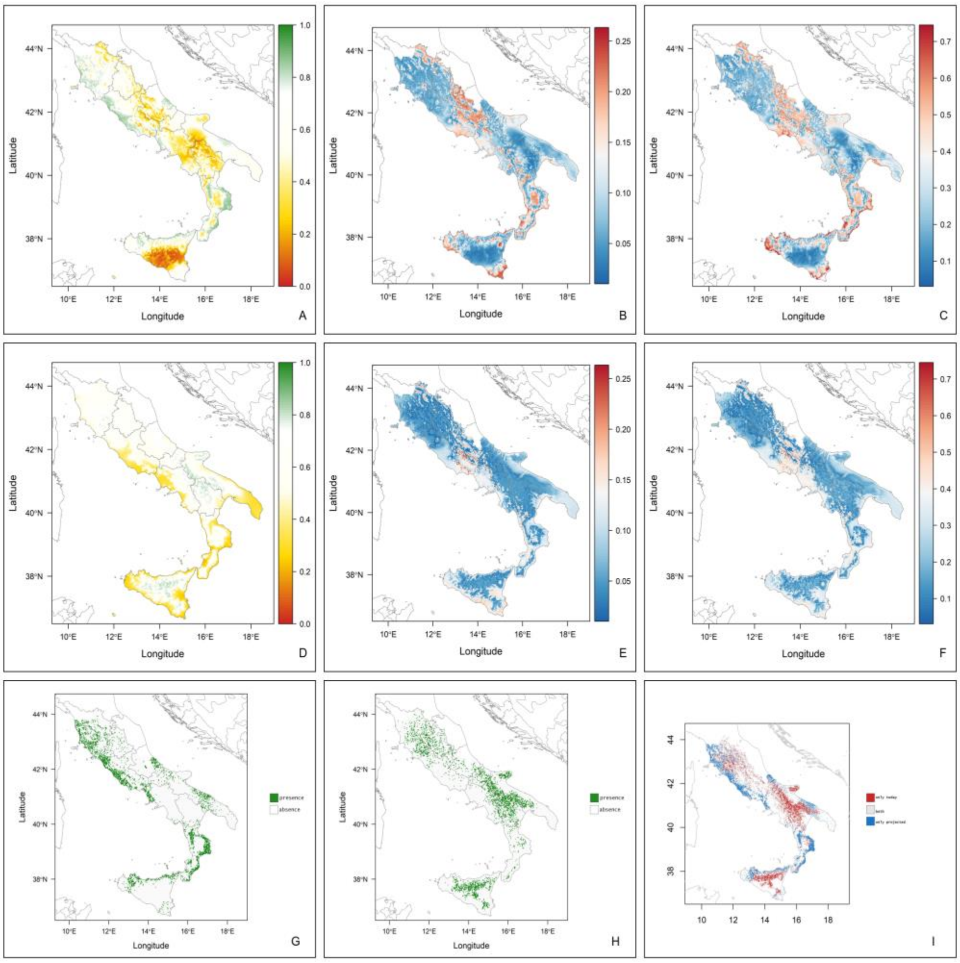

Fig. S.2.148-A) 'Projected' base output; B) 'Projected' uncertainty standard deviation; C) 'Projected' uncertainty range of values; D) 'Present' base output; E) 'Present' uncertainty standard deviation; F) 'Present' uncertainty range of values; G) 'Projected' binary distribution; H) 'Present' binary distribution; I) Range size variation.

Value	Present	Projected
AUC.train	0.684	0.796
AUC.test	0.639	0.603
AUC.diff	0.199	0.199
pROC	1.309	1.274
OR.p10	0.267	0.400
CBI	0.732	0.426
perc.stable	4.579	4.579
range.size	-0.078	-0.078

S.2.149 *Klasea flavescens* KDE

Species: *Klasea flavescens* (L.) Holub subsp. *cichoracea* (L.) Greuter & Wagenitz

Procedure: KDE

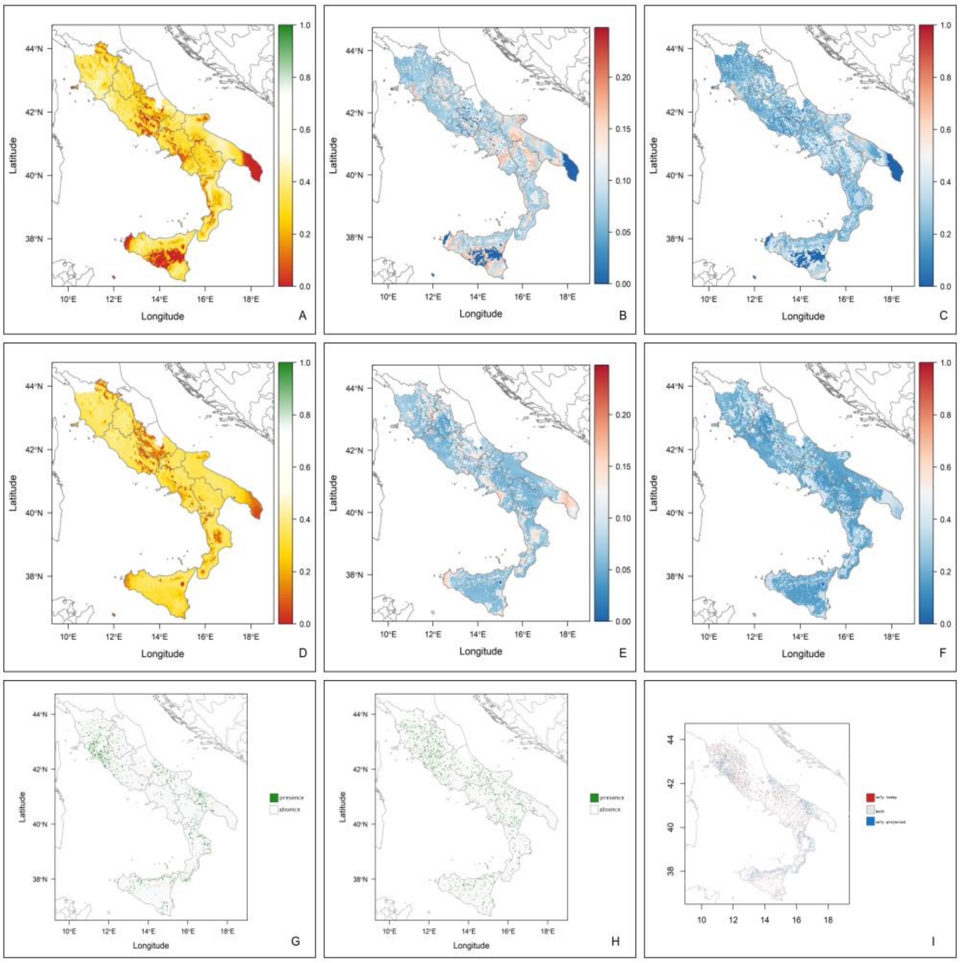

Fig. S.2.149-A) 'Projected' base output; B) 'Projected' uncertainty standard deviation; C) 'Projected' uncertainty range of values; D) 'Present' base output; E) 'Present' uncertainty standard deviation; F) 'Present' uncertainty range of values; G) 'Projected' binary distribution; H) 'Present' binary distribution; I) Range size variation.

Value	Present	Projected
AUC.train	0.645	0.735
AUC.test	0.604	0.720
AUC.diff	0.054	0.038
pROC	1.259	1.371
OR.p10	0.250	0.183
CBI	0.476	0.459
perc.stable	3.369	3.369
range.size	1.332	1.332

S.2.150 *Klasea flavescens* CTA

Species: *Klasea flavescens* (L.) Holub subsp. *cichoracea* (L.) Greuter & Wagenitz

Procedure: CTA

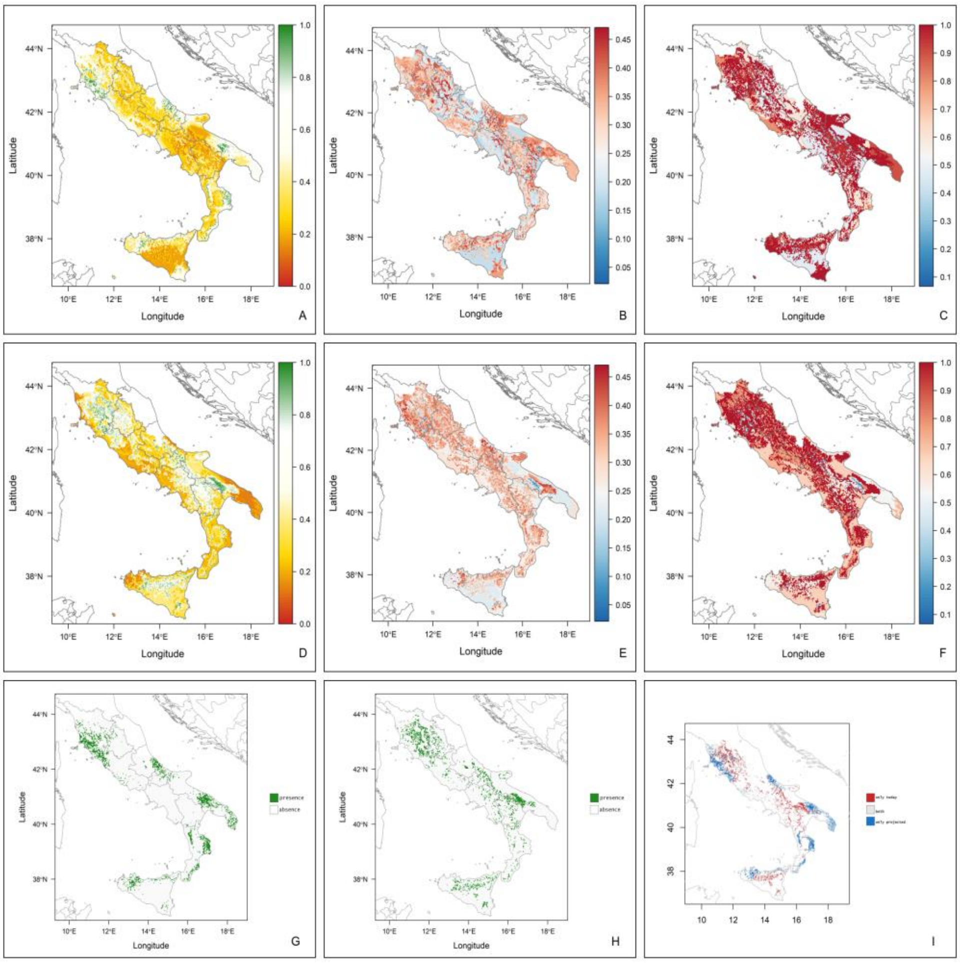

Fig. S.2.150-A) 'Projected' base output; B) 'Projected' uncertainty standard deviation; C) 'Projected' uncertainty range of values; D) 'Present' base output; E) 'Present' uncertainty standard deviation; F) 'Present' uncertainty range of values; G) 'Projected' binary distribution; H) 'Present' binary distribution; I) Range size variation.

Value	Present	Projected
AUC.train	0.876	0.860
AUC.test	0.701	0.693
AUC.diff	0.180	0.180
pROC	1.119	1.110
OR.p10	0.317	0.317
CBI	0.877	0.604
perc.stable	13.251	13.251
range.size	0.818	0.818

S.2.151 *Klasea flavescens* FDA

Species: *Klasea flavescens* (L.) Holub subsp. *cichoracea* (L.) Greuter & Wagenitz

Procedure: FDA

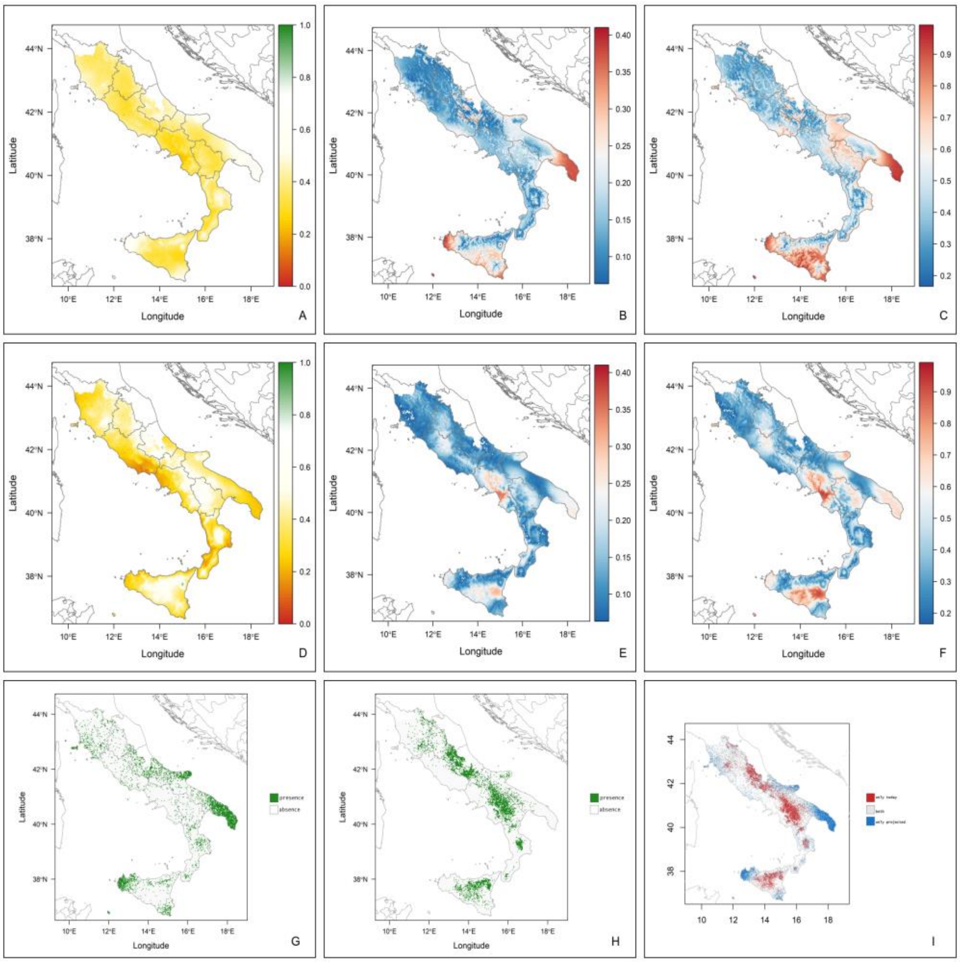

Fig. S.2.151-A) 'Projected' base output; B) 'Projected' uncertainty standard deviation; C) 'Projected' uncertainty range of values; D) 'Present' base output; E) 'Present' uncertainty standard deviation; F) 'Present' uncertainty range of values; G) 'Projected' binary distribution; H) 'Present' binary distribution; I) Range size variation.

Value	Present	Projected
AUC.train	0.638	0.608
AUC.test	0.623	0.539
AUC.diff	0.078	0.078
pROC	1.229	1.199
OR.p10	0.233	0.317
CBI	0.590	0.840
perc.stable	9.358	9.358
range.size	-0.311	-0.311

S.2.152 *Klasea flavescens* GAM

Species: *Klasea flavescens* (L.) Holub subsp. *cichoracea* (L.) Greuter & Wagenitz

Procedure: GAM

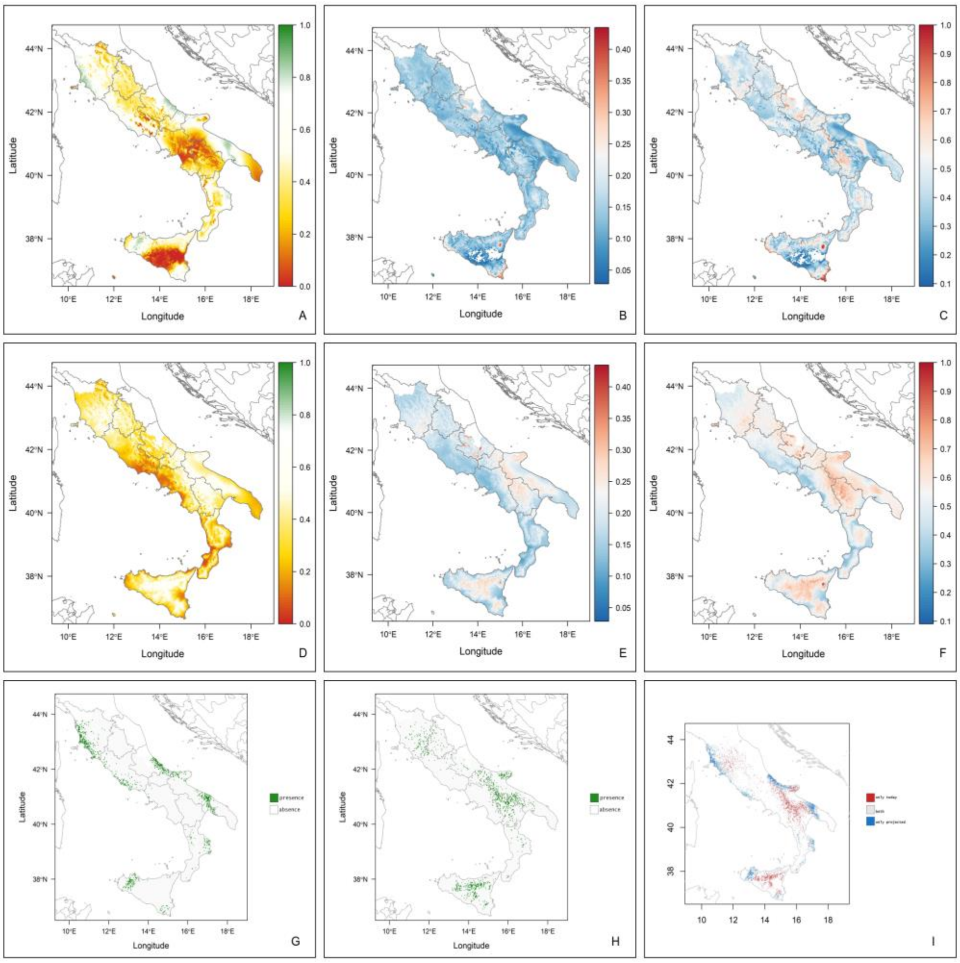

Fig. S.2.152-A) 'Projected' base output; B) 'Projected' uncertainty standard deviation; C) 'Projected' uncertainty range of values; D) 'Present' base output; E) 'Present' uncertainty standard deviation; F) 'Present' uncertainty range of values; G) 'Projected' binary distribution; H) 'Present' binary distribution; I) Range size variation.

Value	Present	Projected
AUC.train	0.670	0.777
AUC.test	0.554	0.662
AUC.diff	0.120	0.120
pROC	1.233	1.324
OR.p10	0.333	0.333
CBI	0.935	0.252
perc.stable	1.209	1.209
range.size	0.785	0.785

S.2.153 *Klasea flavescens* GLM

Species: *Klasea flavescens* (L.) Holub subsp. *cichoracea* (L.) Greuter & Wagenitz

Procedure: GLM

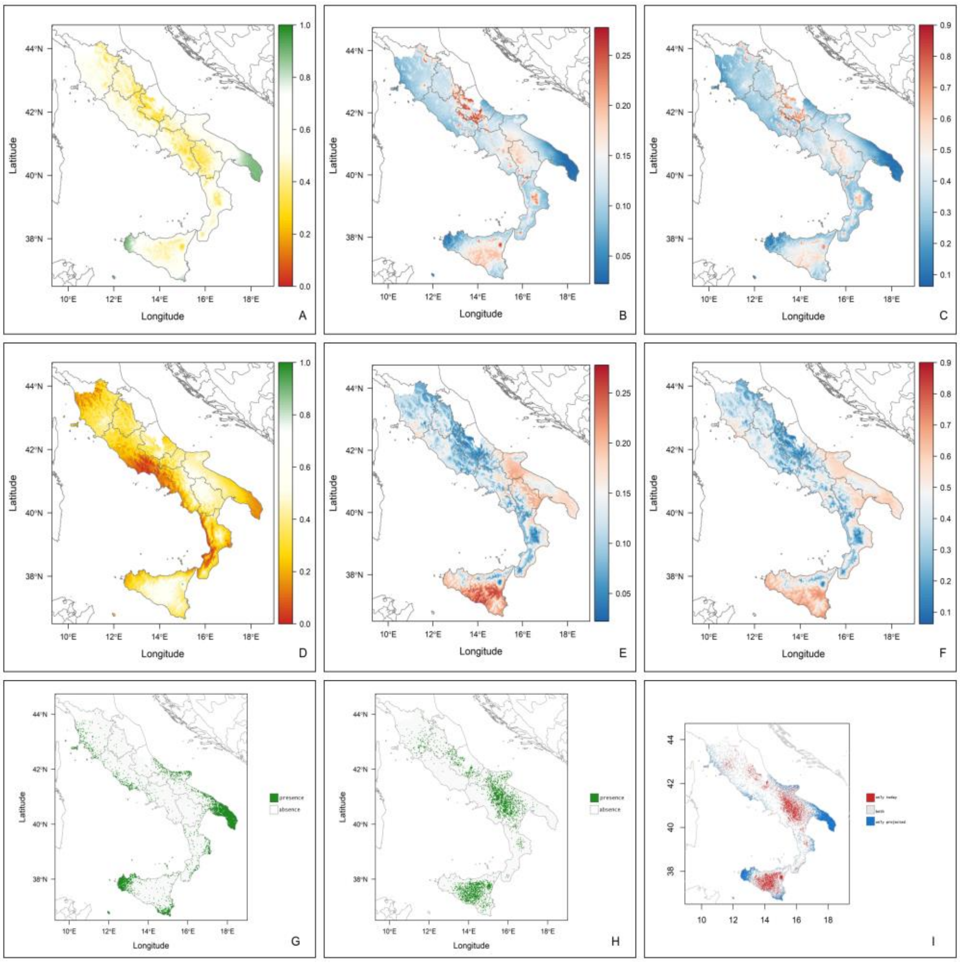

Fig. S.2.153-A) 'Projected' base output; B) 'Projected' uncertainty standard deviation; C) 'Projected' uncertainty range of values; D) 'Present' base output; E) 'Present' uncertainty standard deviation; F) 'Present' uncertainty range of values; G) 'Projected' binary distribution; H) 'Present' binary distribution; I) Range size variation.

Value	Present	Projected
AUC.train	0.616	0.621
AUC.test	0.491	0.597
AUC.diff	0.072	0.072
pROC	1.191	1.288
OR.p10	0.283	0.183
CBI	0.664	0.315
perc.stable	1.862	1.862
range.size	0.202	0.202

S.2.154 *Klasea flavescens* MARS

Species: *Klasea flavescens* (L.) Holub subsp. *cichoracea* (L.) Greuter & Wagenitz

Procedure: MARS

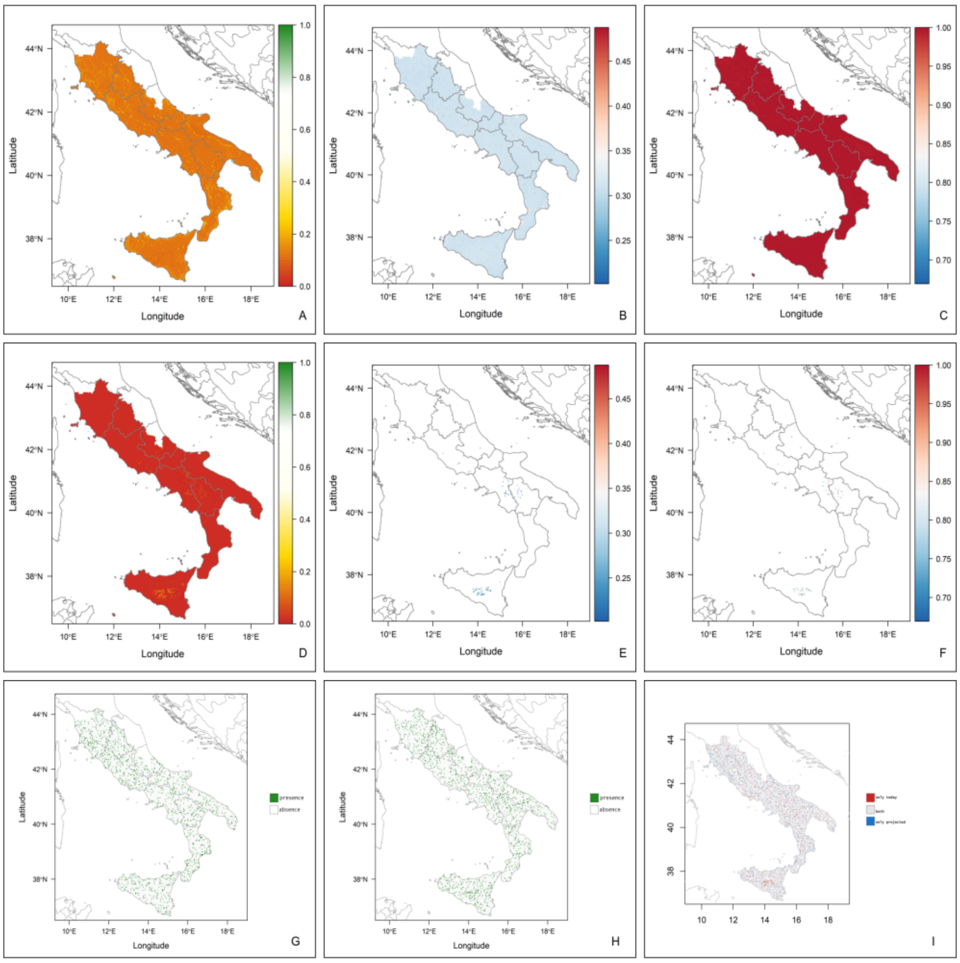

Fig. S.2.154-A) 'Projected' base output; B) 'Projected' uncertainty standard deviation; C) 'Projected' uncertainty range of values; D) 'Present' base output; E) 'Present' uncertainty standard deviation; F) 'Present' uncertainty range of values; G) 'Projected' binary distribution; H) 'Present' binary distribution; I) Range size variation.

Value	Present	Projected
AUC.train	0.618	0.716
AUC.test	0.487	0.511
AUC.diff	0.205	0.205
pROC	1.050	1.335
OR.p10	0.250	0.265
CBI	0.748	-0.921
perc.stable	3.787	3.787
range.size	-1.150	-1.150

S.2.155 *Klasea flavescens* RF

Species: *Klasea flavescens* (L.) Holub subsp. *cichoracea* (L.) Greuter & Wagenitz

Procedure: RF

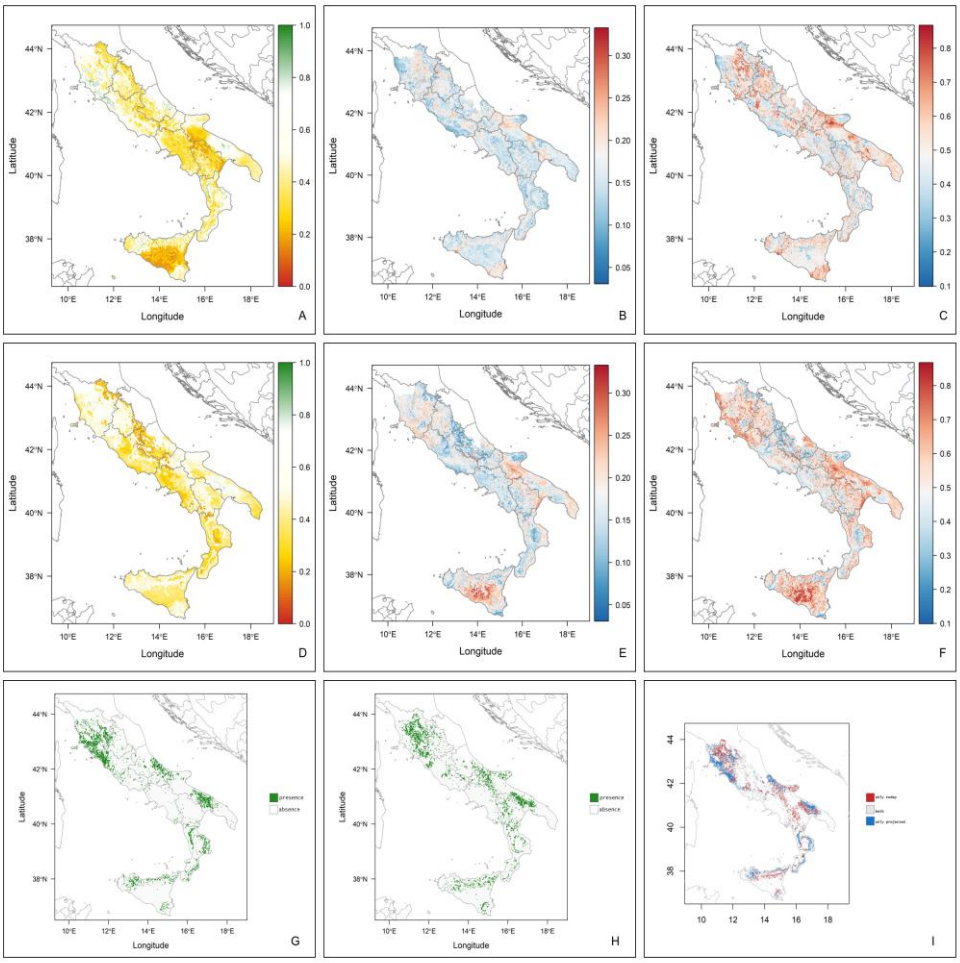

Fig. S.2.155-A) 'Projected' base output; B) 'Projected' uncertainty standard deviation; C) 'Projected' uncertainty range of values; D) 'Present' base output; E) 'Present' uncertainty standard deviation; F) 'Present' uncertainty range of values; G) 'Projected' binary distribution; H) 'Present' binary distribution; I) Range size variation.

Value	Present	Projected
AUC.train	1.000	1.000
AUC.test	0.875	0.898
AUC.diff	0.102	0.102
pROC	1.188	1.323
OR.p10	0.900	0.650
CBI	0.909	0.687
perc.stable	15.156	15.156
range.size	0.778	0.778

S.2.156 *Klasea flavescens* SVM

Species: *Klasea flavescens* (L.) Holub subsp. *cichoracea* (L.) Greuter & Wagenitz

Procedure: SVM

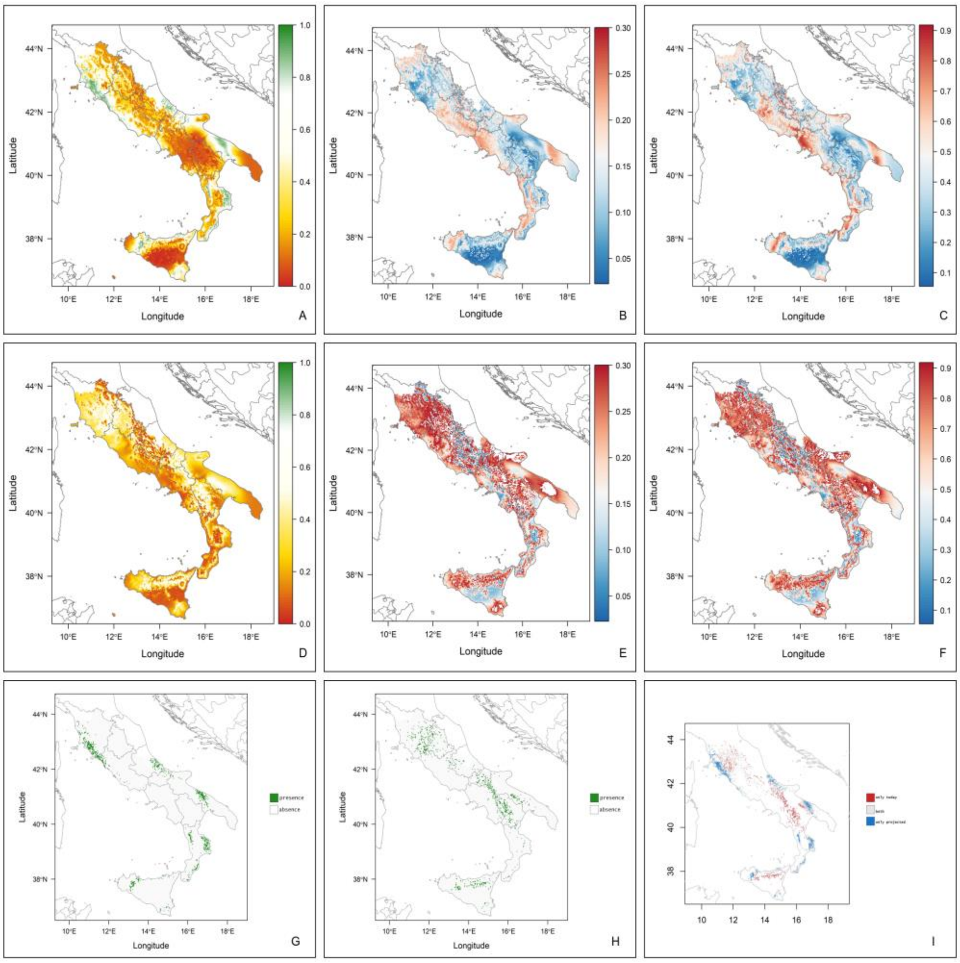

Fig. S.2.156-A) 'Projected' base output; B) 'Projected' uncertainty standard deviation; C) 'Projected' uncertainty range of values; D) 'Present' base output; E) 'Present' uncertainty standard deviation; F) 'Present' uncertainty range of values; G) 'Projected' binary distribution; H) 'Present' binary distribution; I) Range size variation.

Value	Present	Projected
AUC.train	0.803	0.832
AUC.test	0.619	0.789
AUC.diff	0.062	0.062
pROC	1.182	1.402
OR.p10	0.383	0.267
CBI	0.840	0.319
perc.stable	0.882	0.882
range.size	-1.733	-1.733

S.2.157 *Koeleria splendens* Biomod2

Species: *Koeleria splendens* C.Presl

Procedure: Biomod2

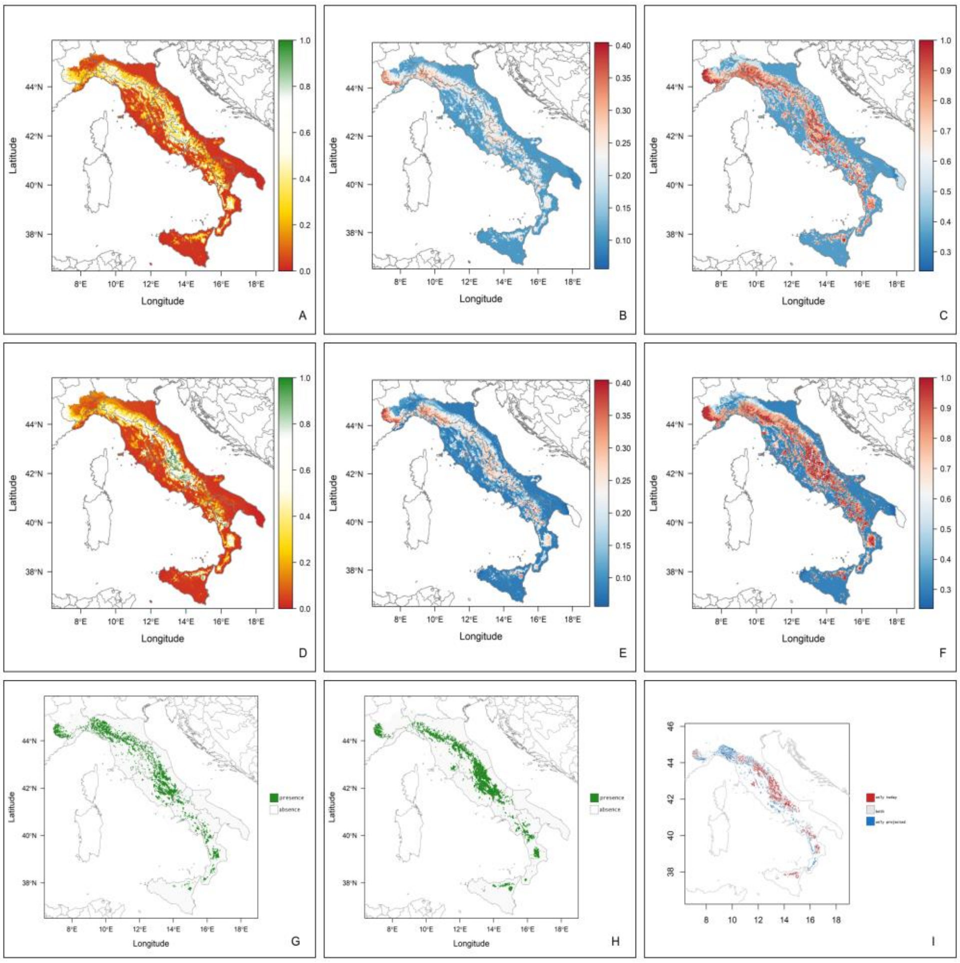

Fig. S.2.157-A) 'Projected' base output; B) 'Projected' uncertainty standard deviation; C) 'Projected' uncertainty range of values; D) 'Present' base output; E) 'Present' uncertainty standard deviation; F) 'Present' uncertainty range of values; G) 'Projected' binary distribution; H) 'Present' binary distribution; I) Range size variation.

Value	Present	Projected
AUC.train	0.964	0.955
AUC.test	0.842	0.890
AUC.diff	0.065	0.065
pROC	1.229	1.391
OR.p10	0.316	0.280
CBI	0.953	0.984
perc.stable	45.844	45.844
range.size	-0.103	-0.103

S.2.158 *Koeleria splendens* Bioclim

Species: *Koeleria splendens* C.Presl

Procedure: Bioclim

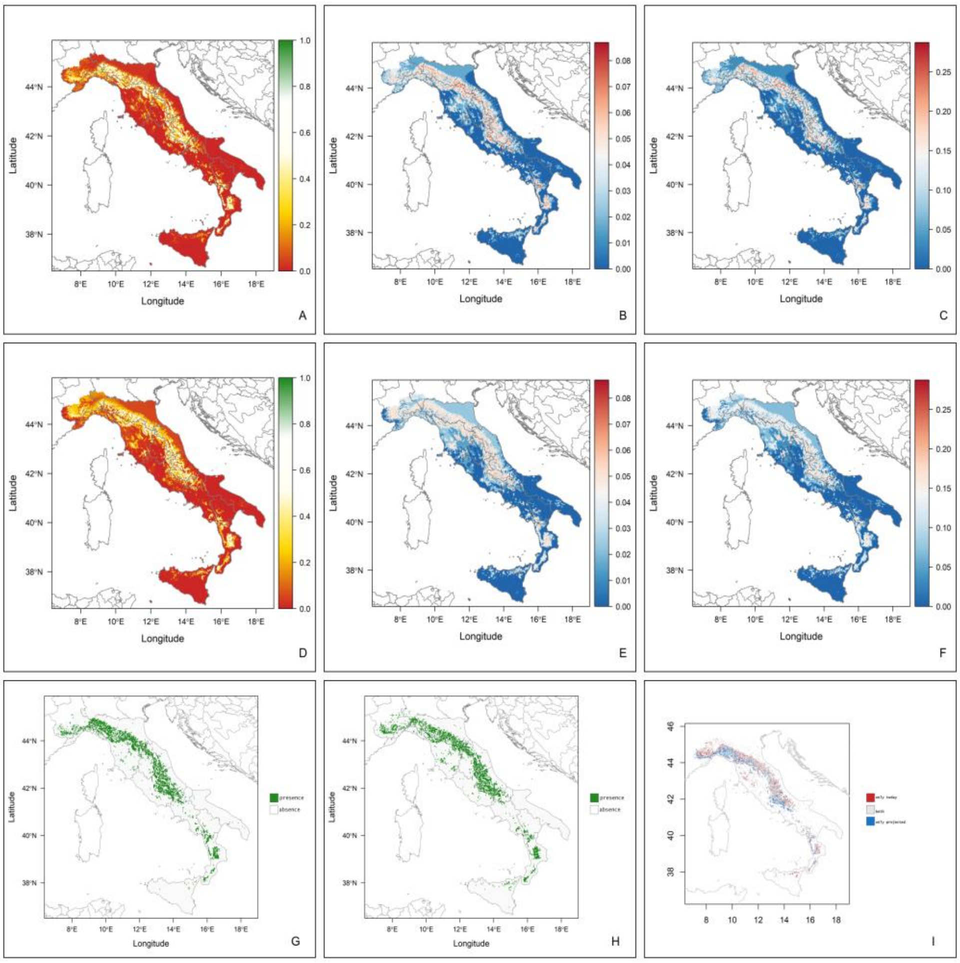

Fig. S.2.158-A) 'Projected' base output; B) 'Projected' uncertainty standard deviation; C) 'Projected' uncertainty range of values; D) 'Present' base output; E) 'Present' uncertainty standard deviation; F) 'Present' uncertainty range of values; G) 'Projected' binary distribution; H) 'Present' binary distribution; I) Range size variation.

Value	Present	Projected
AUC.train	0.818	0.775
AUC.test	0.788	0.752
AUC.diff	0.030	0.030
pROC	1.179	1.155
OR.p10	0.120	0.128
CBI	0.958	0.379
perc.stable	50.687	50.687
range.size	0.786	0.786

S.2.159 *Koeleria splendens* Domain

Species: *Koeleria splendens* C.Presl

Procedure: Domain

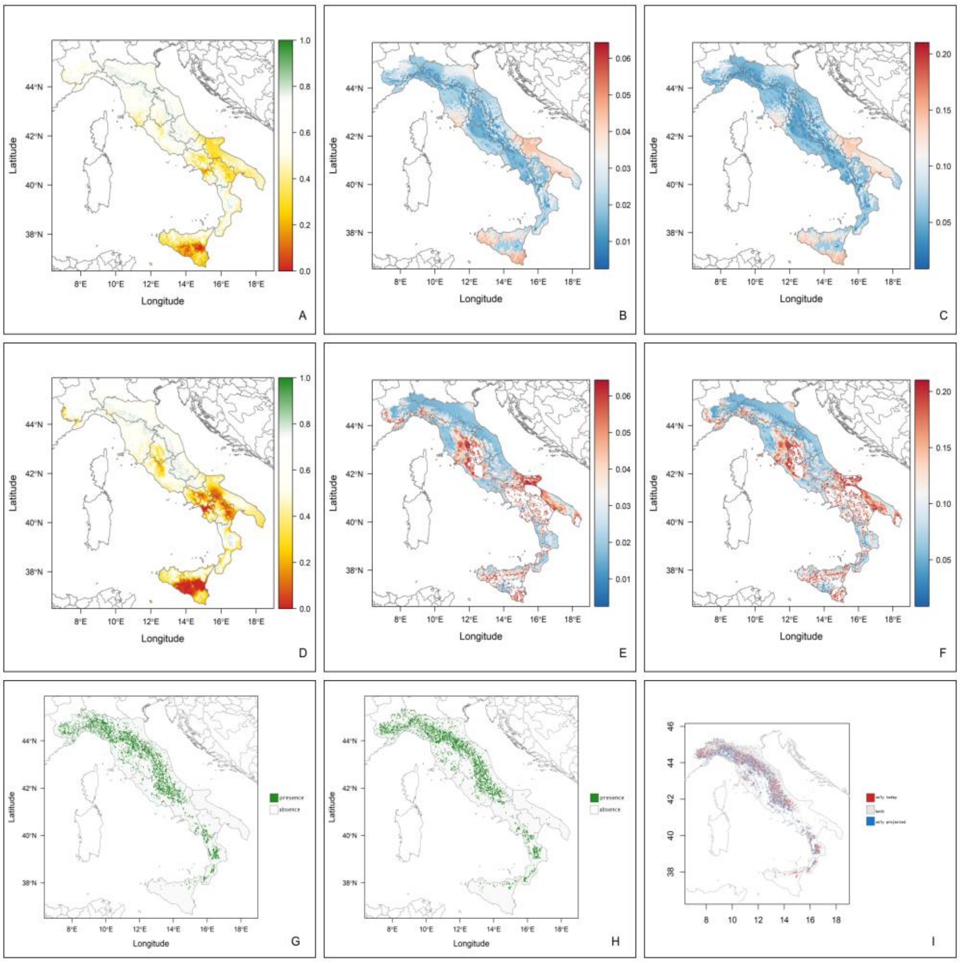

Fig. S.2.159-A) 'Projected' base output; B) 'Projected' uncertainty standard deviation; C) 'Projected' uncertainty range of values; D) 'Present' base output; E) 'Present' uncertainty standard deviation; F) 'Present' uncertainty range of values; G) 'Projected' binary distribution; H) 'Present' binary distribution; I) Range size variation.

Value	Present	Projected
AUC.train	0.812	0.731
AUC.test	0.799	0.740
AUC.diff	0.014	0.014
pROC	1.280	1.231
OR.p10	0.132	0.104
CBI	0.978	0.998
perc.stable	26.845	26.845
range.size	-0.925	-0.925

S.2.160 *Koeleria splendens* Maxent

Species: *Koeleria splendens* C.Presl

Procedure: Maxent

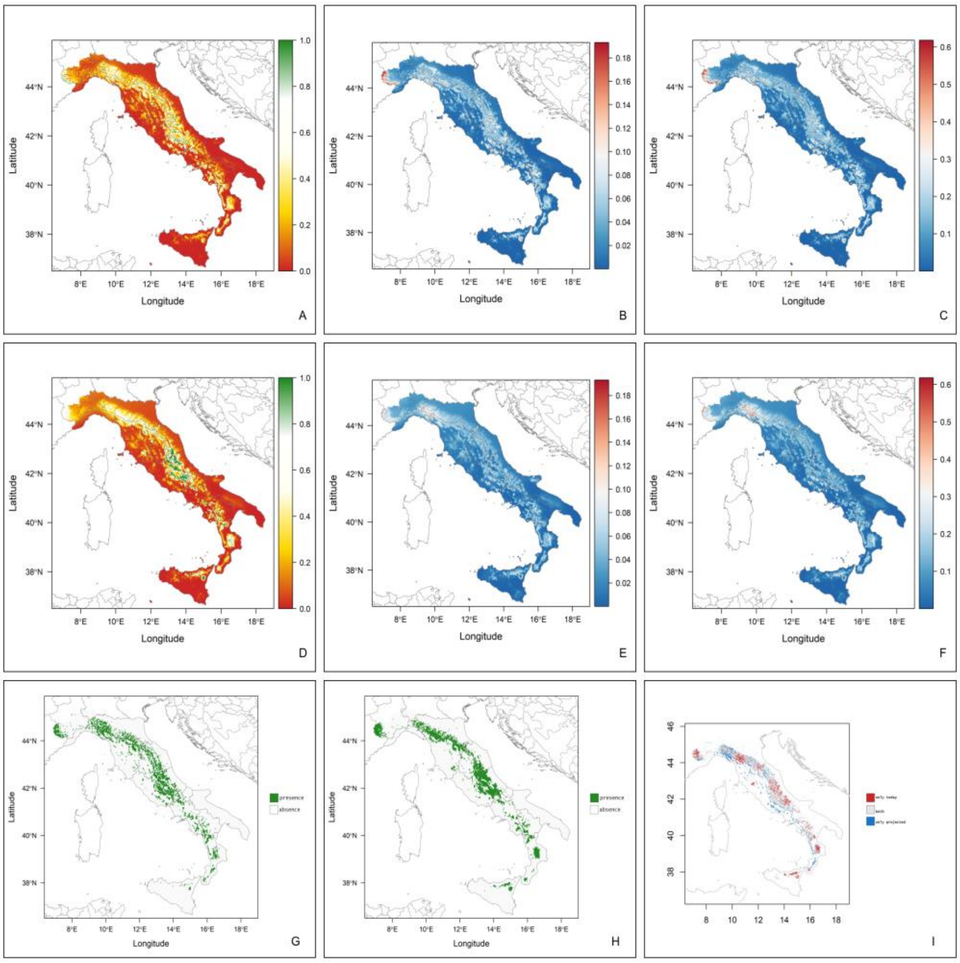

Fig. S.2.160-A) 'Projected' base output; B) 'Projected' uncertainty standard deviation; C) 'Projected' uncertainty range of values; D) 'Present' base output; E) 'Present' uncertainty standard deviation; F) 'Present' uncertainty range of values; G) 'Projected' binary distribution; H) 'Present' binary distribution; I) Range size variation.

Value	Present	Projected
AUC.train	0.893	0.880
AUC.test	0.876	0.852
AUC.diff	0.031	0.031
pROC	1.379	1.384
OR.p10	0.152	0.200
CBI	0.988	0.944
perc.stable	50.160	50.160
range.size	0.353	0.353

S.2.161 *Koeleria splendens* KDE

Species: *Koeleria splendens* C.Presl

Procedure: KDE

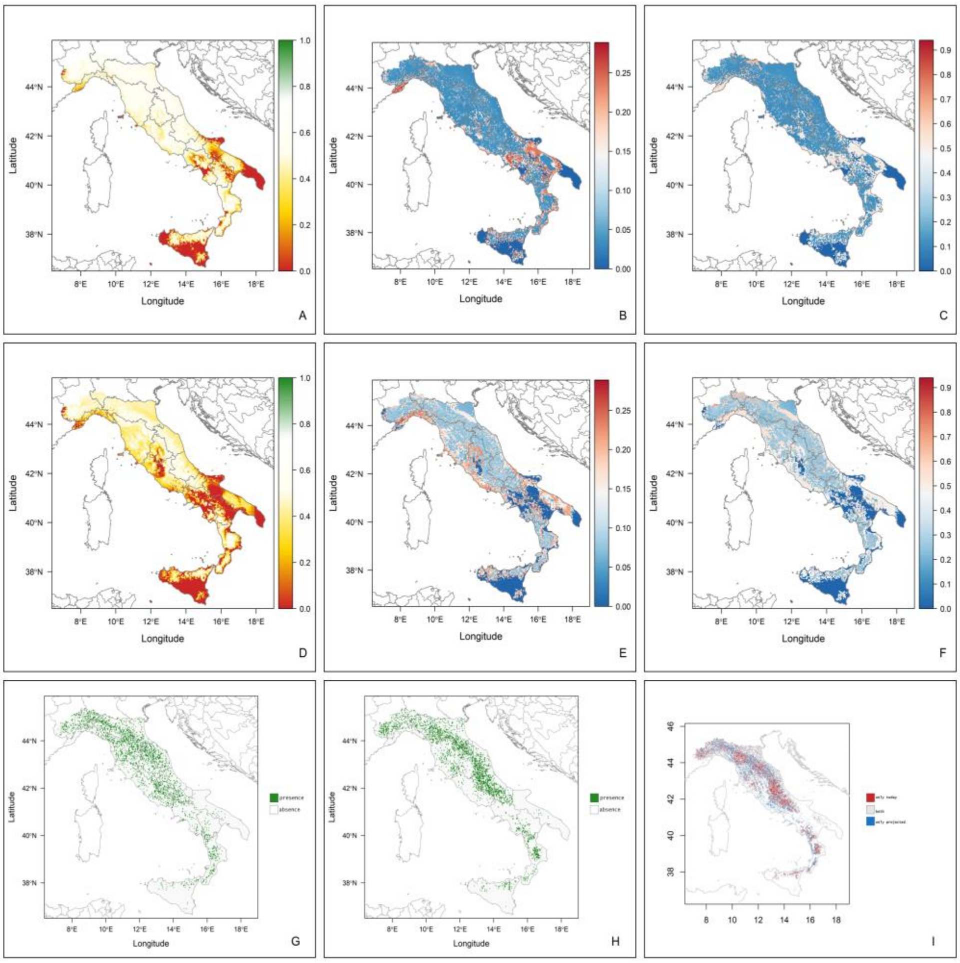

Fig. S.2.161-A) 'Projected' base output; B) 'Projected' uncertainty standard deviation; C) 'Projected' uncertainty range of values; D) 'Present' base output; E) 'Present' uncertainty standard deviation; F) 'Present' uncertainty range of values; G) 'Projected' binary distribution; H) 'Present' binary distribution; I) Range size variation.

Value	Present	Projected
AUC.train	0.840	0.703
AUC.test	0.817	0.667
AUC.diff	0.031	0.043
pROC	1.260	1.124
OR.p10	0.140	0.168
CBI	0.895	0.762
perc.stable	16.352	16.352
range.size	-0.960	-0.960

S.2.162 *Koeleria splendens* CTA

Species: *Koeleria splendens* C.Presl

Procedure: CTA

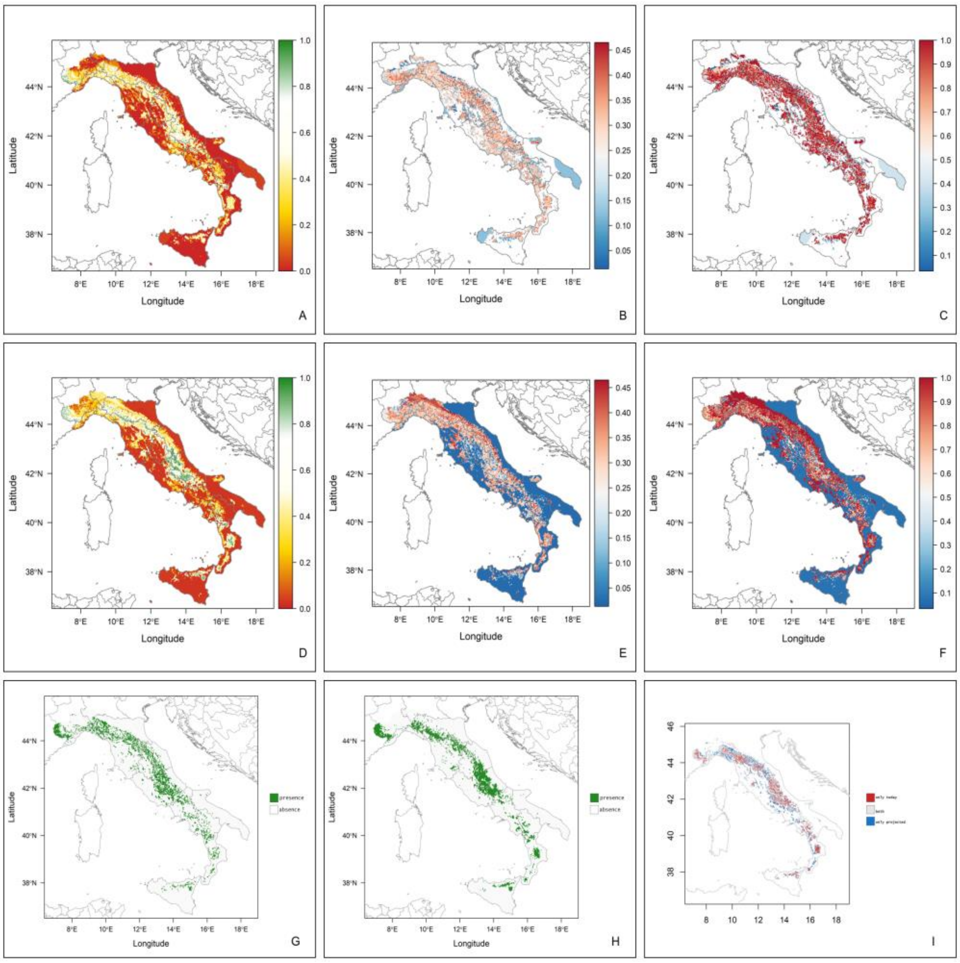

Fig. S.2.162-A) 'Projected' base output; B) 'Projected' uncertainty standard deviation; C) 'Projected' uncertainty range of values; D) 'Present' base output; E) 'Present' uncertainty standard deviation; F) 'Present' uncertainty range of values; G) 'Projected' binary distribution; H) 'Present' binary distribution; I) Range size variation.

Value	Present	Projected
AUC.train	0.941	0.939
AUC.test	0.880	0.838
AUC.diff	0.101	0.101
pROC	1.193	1.128
OR.p10	0.224	0.284
CBI	0.973	0.965
perc.stable	38.783	38.783
range.size	-0.096	-0.096

S.2.163 *Koeleria splendens* FDA

Species: *Koeleria splendens* C.Presl

Procedure: FDA

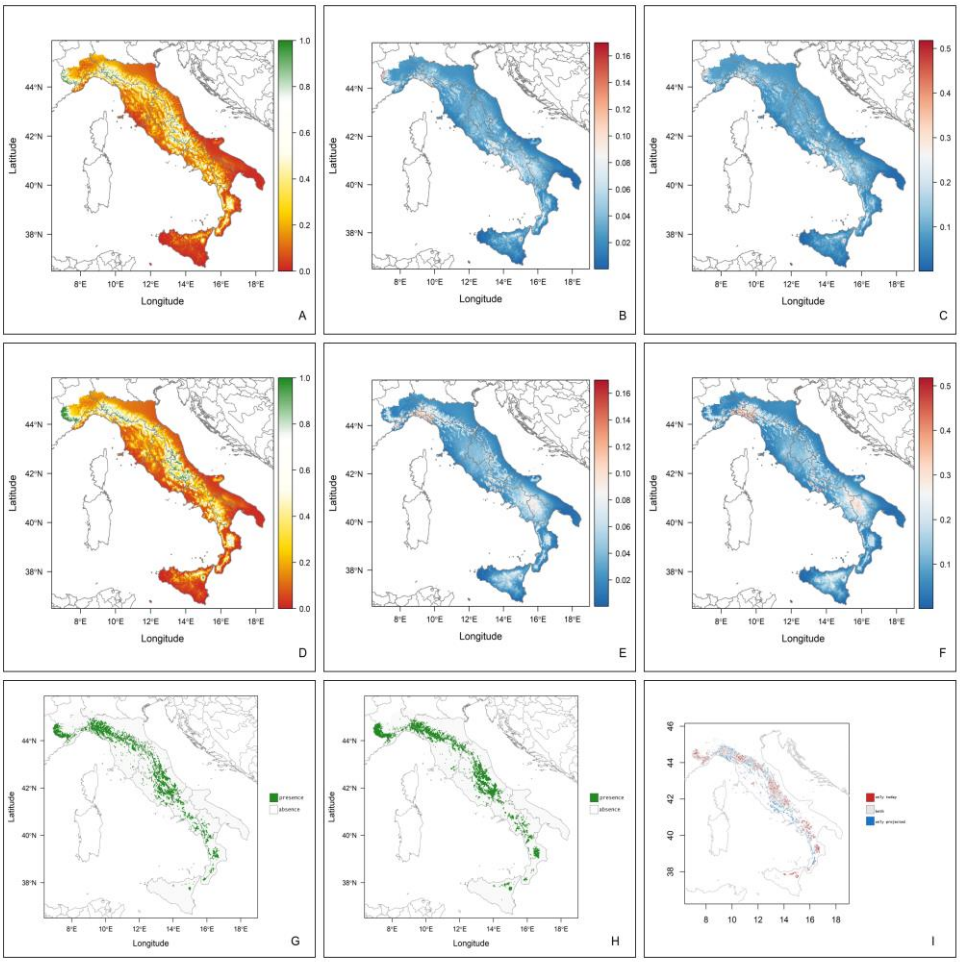

Fig. S.2.163-A) 'Projected' base output; B) 'Projected' uncertainty standard deviation; C) 'Projected' uncertainty range of values; D) 'Present' base output; E) 'Present' uncertainty standard deviation; F) 'Present' uncertainty range of values; G) 'Projected' binary distribution; H) 'Present' binary distribution; I) Range size variation.

Value	Present	Projected
AUC.train	0.864	0.840
AUC.test	0.859	0.854
AUC.diff	0.018	0.018
pROC	1.382	1.399
OR.p10	0.104	0.116
CBI	0.988	0.988
perc.stable	60.899	60.899
range.size	-0.897	-0.897

S.2.164 *Koeleria splendens* GAM

Species: *Koeleria splendens* C.Presl

Procedure: GAM

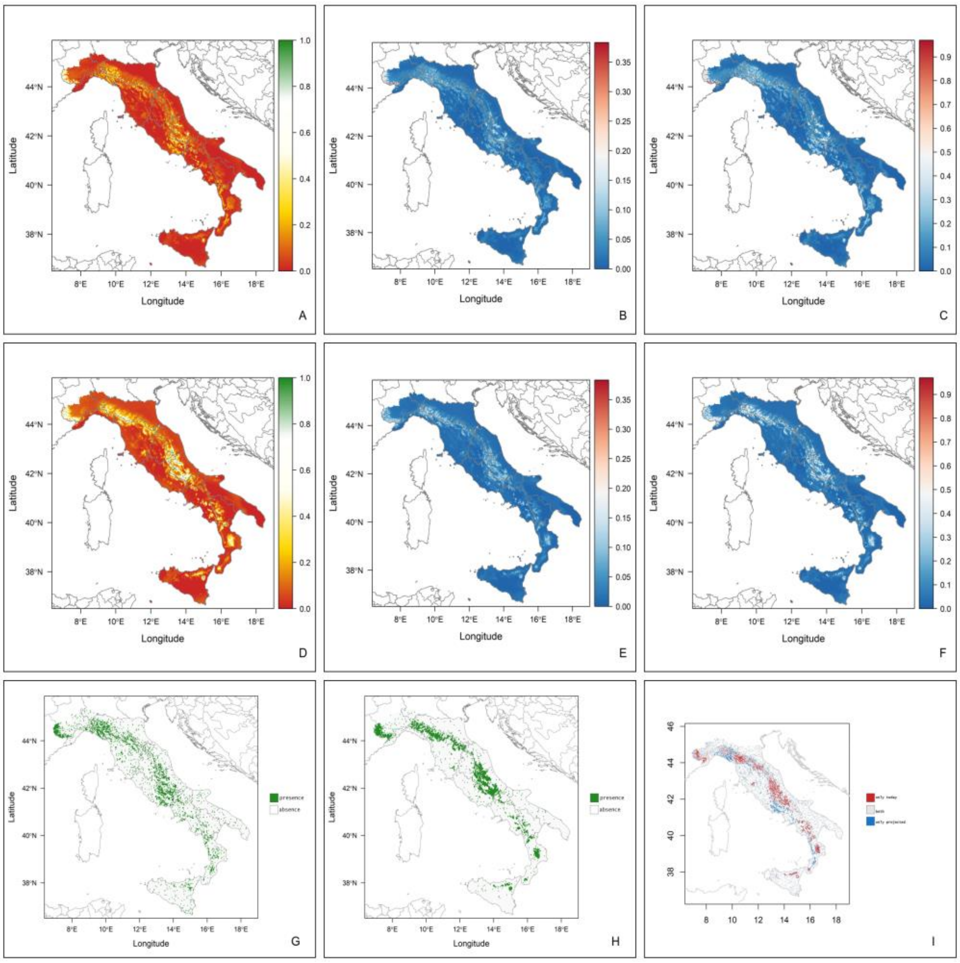

Fig. S.2.164-A) ‘Projected’ base output; B) ‘Projected’ uncertainty standard deviation; C) ‘Projected’ uncertainty range of values; D) ’Present’ base output; E) ’Present’ uncertainty standard deviation; F) ’Present’ uncertainty range of values; G) ‘Projected’ binary distribution; H) ‘Present’ binary distribution; I) Range size variation.

Value	Present	Projected
AUC.train	0.867	0.874
AUC.test	0.857	0.837
AUC.diff	0.045	0.045
pROC	1.317	1.294
OR.p10	0.108	0.188
CBI	0.986	0.933
perc.stable	28.000	28.000
range.size	0.778	0.778

S.2.165 *Koeleria splendens* GLM

Species: *Koeleria splendens* C.Presl

Procedure: GLM

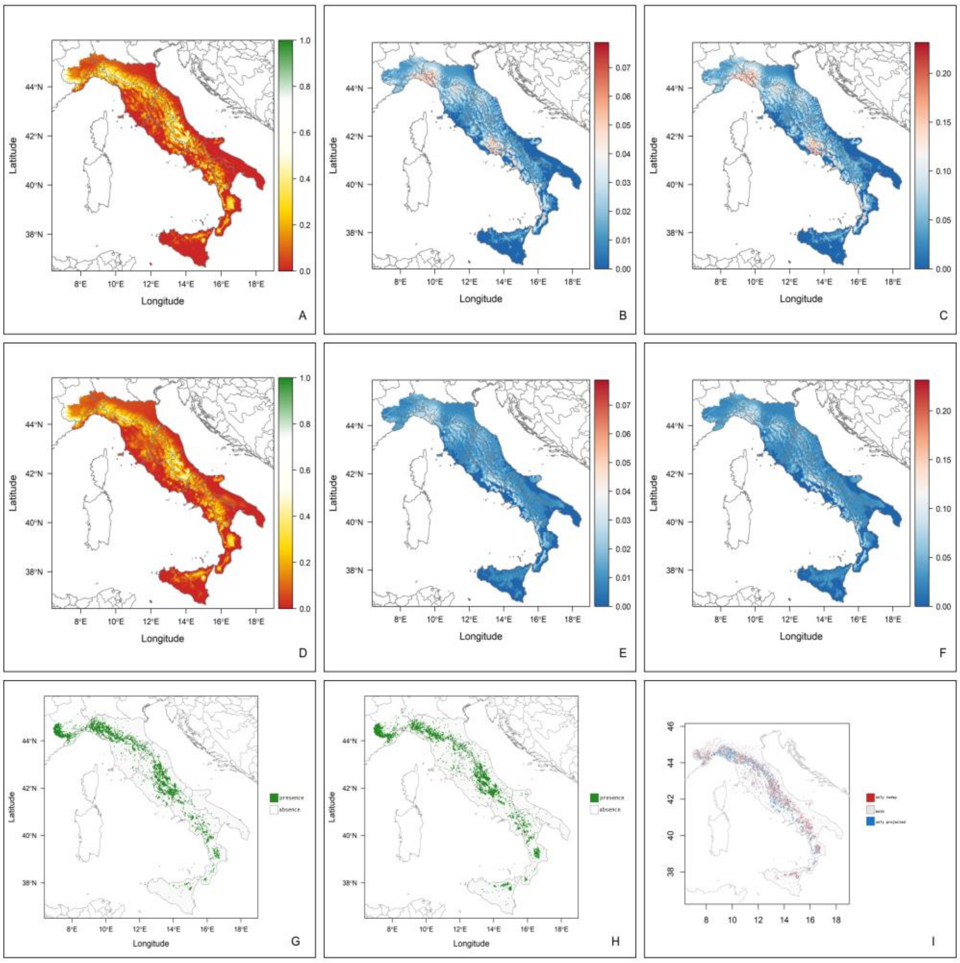

Fig. S.2.165-A) 'Projected' base output; B) 'Projected' uncertainty standard deviation; C) 'Projected' uncertainty range of values; D) 'Present' base output; E) 'Present' uncertainty standard deviation; F) 'Present' uncertainty range of values; G) 'Projected' binary distribution; H) 'Present' binary distribution; I) Range size variation.

Value	Present	Projected
AUC.train	0.865	0.859
AUC.test	0.868	0.832
AUC.diff	0.032	0.032
pROC	1.363	1.355
OR.p10	0.108	0.148
CBI	0.616	0.740
perc.stable	42.650	42.650
range.size	1.008	1.008

S.2.166 *Koeleria splendens* MARS

Species: *Koeleria splendens* C.Presl

Procedure: MARS

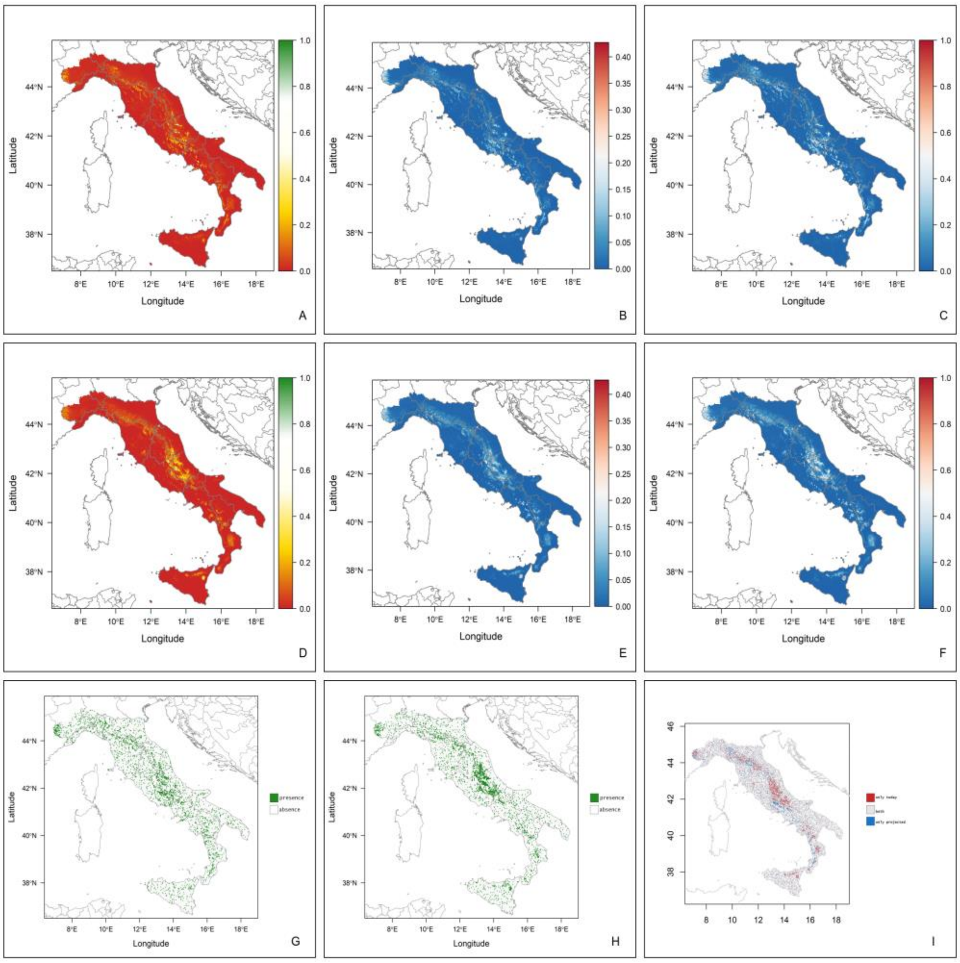

Fig. S.2.166-A) ‘Projected’ base output; B) ‘Projected’ uncertainty standard deviation; C) ‘Projected’ uncertainty range of values; D) ’Present’ base output; E) ’Present’ uncertainty standard deviation; F) ’Present’ uncertainty range of values; G) ‘Projected’ binary distribution; H) ‘Present’ binary distribution; I) Range size variation.

Value	Present	Projected
AUC.train	0.900	0.880
AUC.test	0.881	0.853
AUC.diff	0.035	0.035
pROC	1.325	1.319
OR.p10	0.104	0.140
CBI	0.924	0.867
perc.stable	12.685	12.685
range.size	-0.999	-0.999

S.2.167 *Koeleria splendens* RF

Species: *Koeleria splendens* C.Presl

Procedure: RF

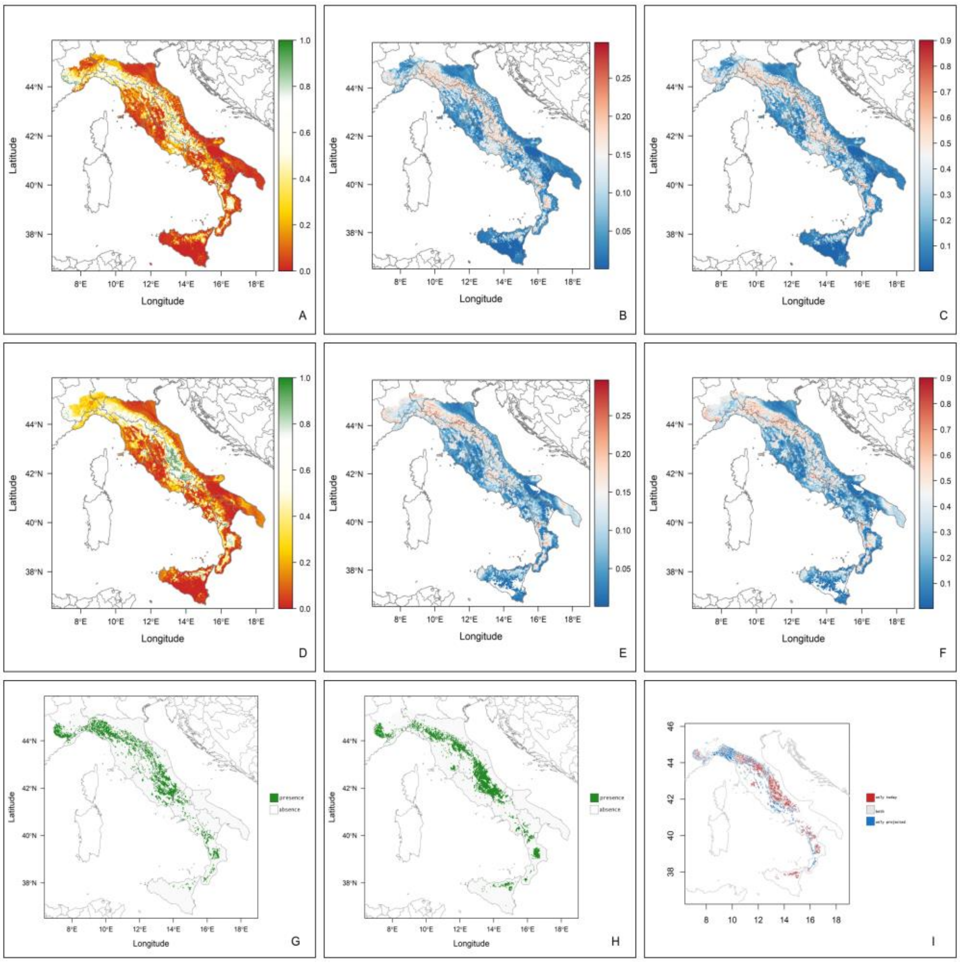

Fig. S.2.167-A) 'Projected' base output; B) 'Projected' uncertainty standard deviation; C) 'Projected' uncertainty range of values; D) 'Present' base output; E) 'Present' uncertainty standard deviation; F) 'Present' uncertainty range of values; G) 'Projected' binary distribution; H) 'Present' binary distribution; I) Range size variation.

Value	Present	Projected
AUC.train	1.000	1.000
AUC.test	0.967	0.962
AUC.diff	0.038	0.038
pROC	1.399	1.335
OR.p10	0.424	0.540
CBI	0.982	0.987
perc.stable	39.141	39.141
range.size	0.029	0.029

S.2.168 *Koeleria splendens* SVM

Species: *Koeleria splendens* C.Presl

Procedure: SVM

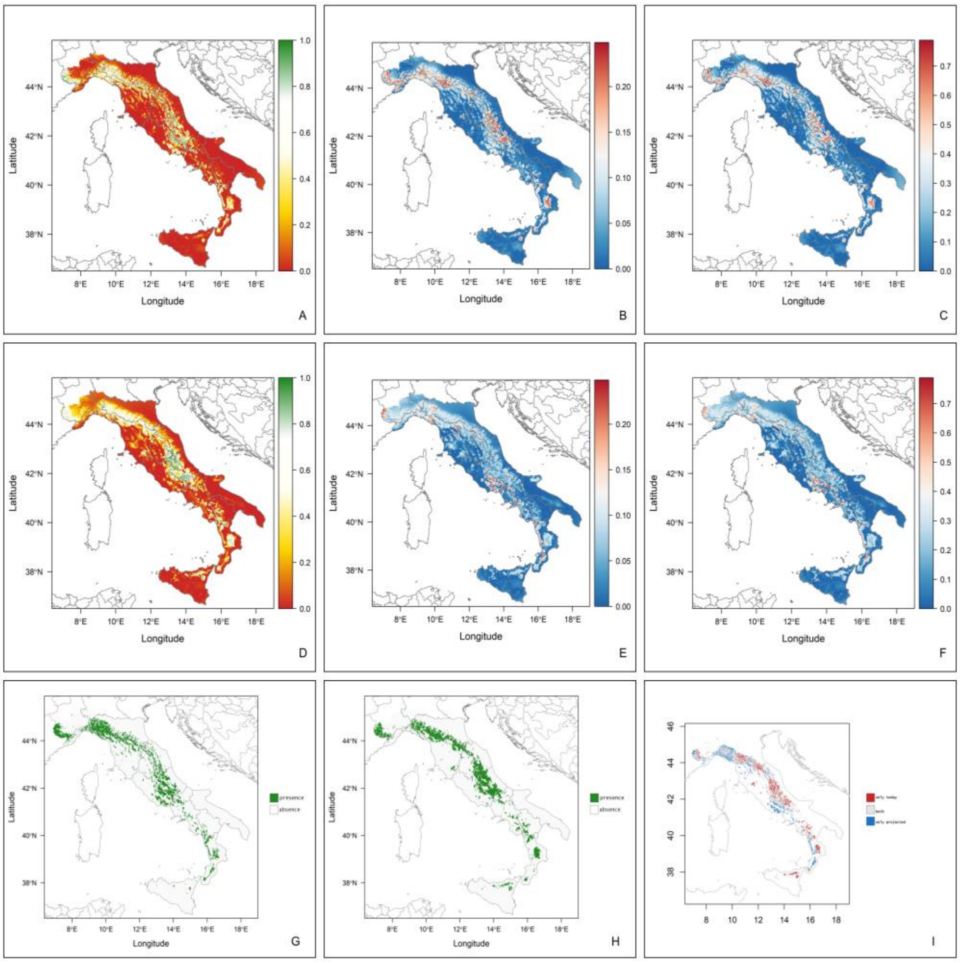

Fig. S.2.168-A) 'Projected' base output; B) 'Projected' uncertainty standard deviation; C) 'Projected' uncertainty range of values; D) 'Present' base output; E) 'Present' uncertainty standard deviation; F) 'Present' uncertainty range of values; G) 'Projected' binary distribution; H) 'Present' binary distribution; I) Range size variation.

Value	Present	Projected
AUC.train	0.903	0.866
AUC.test	0.875	0.873
AUC.diff	0.013	0.013
pROC	1.288	1.266
OR.p10	0.156	0.116
CBI	0.950	0.936
perc.stable	48.711	48.711
range.size	-1.132	-1.132

S.2.169 *Leontodon anomalus* Biomod2

Species: *Leontodon anomalus* Ball

Procedure: Biomod2

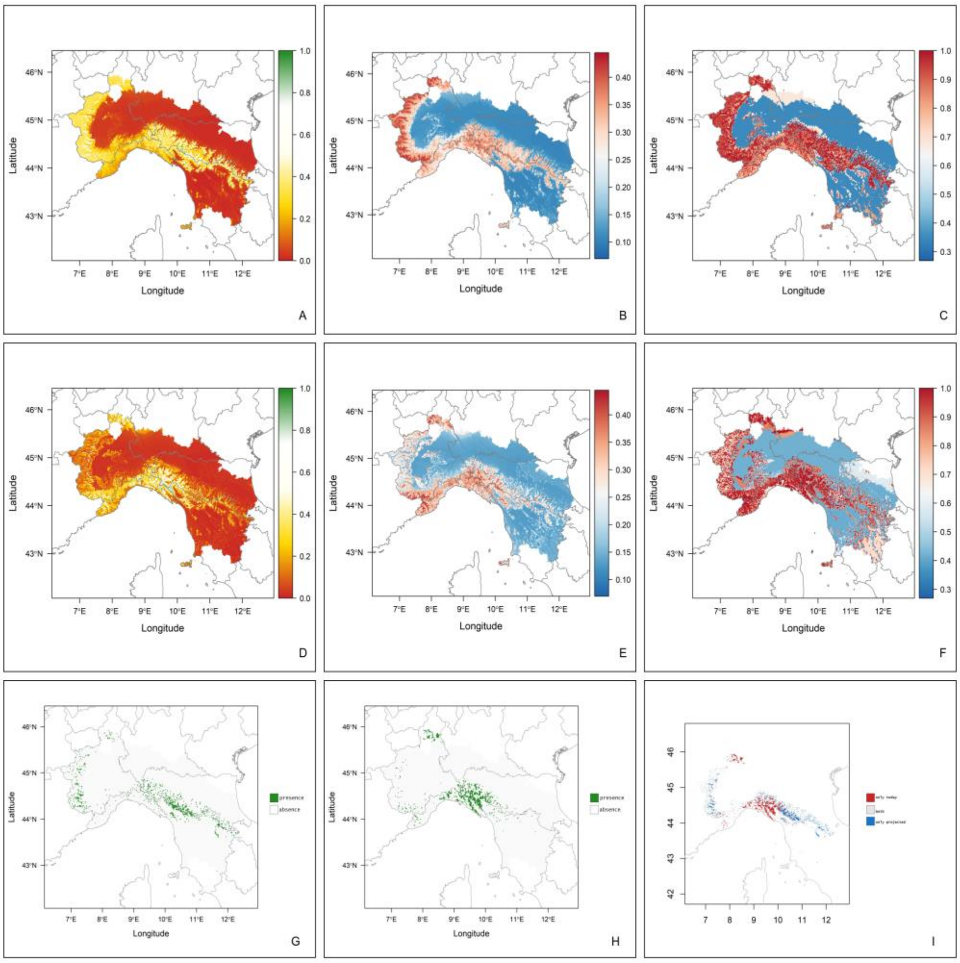

Fig. S.2.169-A) 'Projected' base output; B) 'Projected' uncertainty standard deviation; C) 'Projected' uncertainty range of values; D) 'Present' base output; E) 'Present' uncertainty standard deviation; F) 'Present' uncertainty range of values; G) 'Projected' binary distribution; H) 'Present' binary distribution; I) Range size variation.

Value	Present	Projected
AUC.train	0.997	0.999
AUC.test	0.954	0.860
AUC.diff	0.139	0.139
pROC	1.861	1.557
OR.p10	0.980	0.780
CBI	0.931	0.843
perc.stable	5.683	5.683
range.size	-0.317	-0.317

S.2.170 *Leontodon anomalus* Bioclim

Species: *Leontodon anomalus* Ball

Procedure: Bioclim

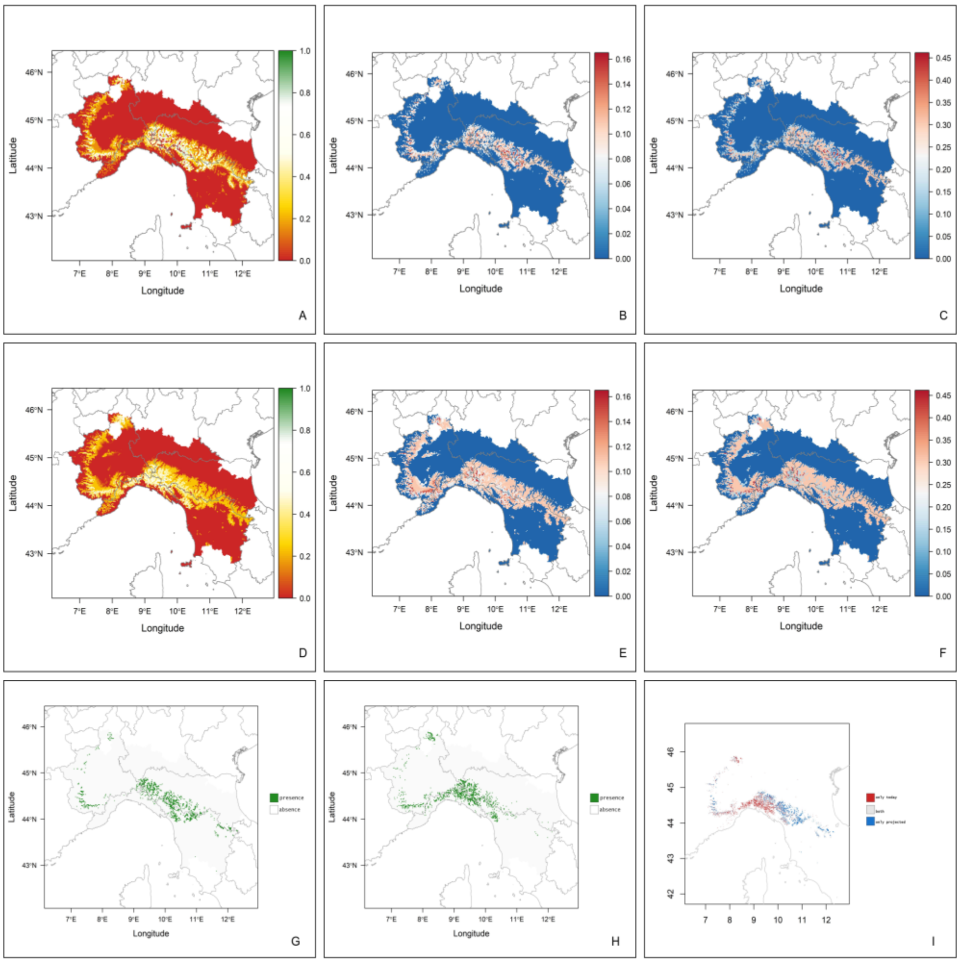

Fig. S.2.170-A) 'Projected' base output; B) 'Projected' uncertainty standard deviation; C) 'Projected' uncertainty range of values; D) 'Present' base output; E) 'Present' uncertainty standard deviation; F) 'Present' uncertainty range of values; G) 'Projected' binary distribution; H) 'Present' binary distribution; I) Range size variation.

Value	Present	Projected
AUC.train	0.867	0.842
AUC.test	0.745	0.803
AUC.diff	0.073	0.073
pROC	1.256	1.390
OR.p10	0.300	0.140
CBI	0.682	0.061
perc.stable	21.608	21.608
range.size	-1.581	-1.581

S.2.171 *Leontodon anomalus* Domain

Species: *Leontodon anomalus* Ball

Procedure: Domain

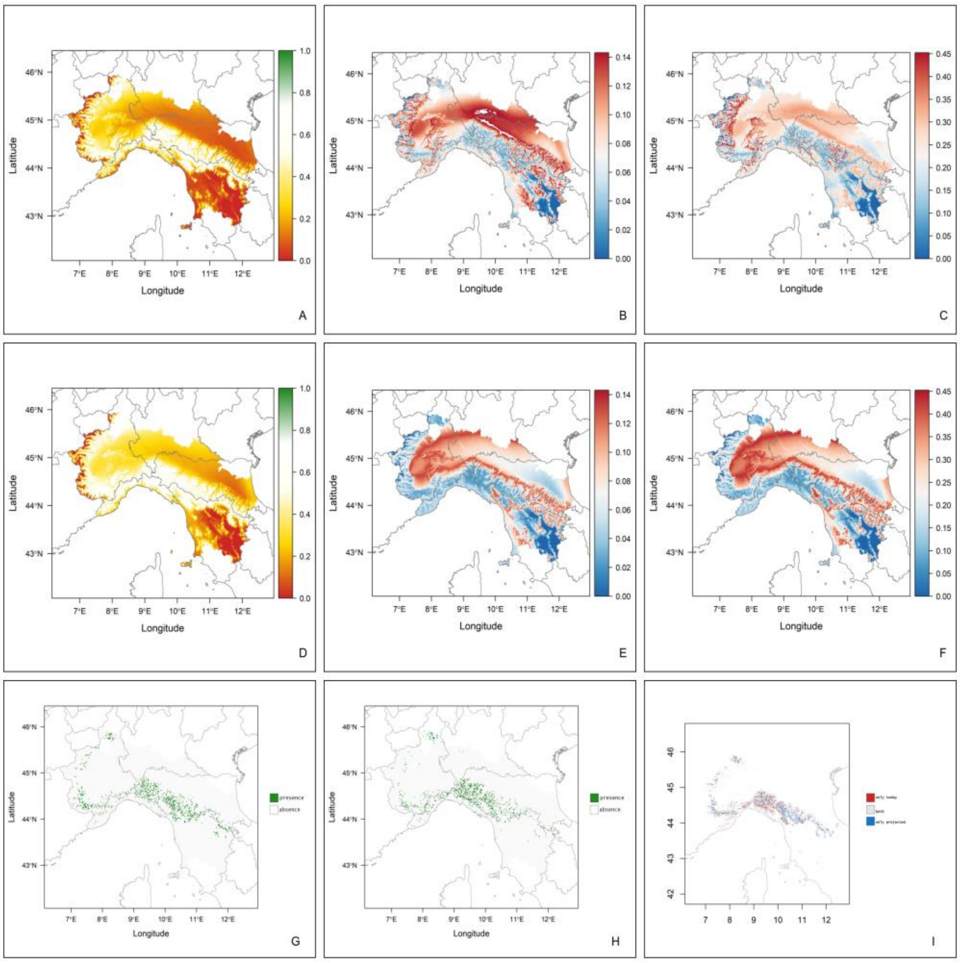

Fig. S.2.171-A) ‘Projected’ base output; B) ‘Projected’ uncertainty standard deviation; C) ‘Projected’ uncertainty range of values; D) ’Present’ base output; E) ’Present’ uncertainty standard deviation; F) ’Present’ uncertainty range of values; G) ‘Projected’ binary distribution; H) ‘Present’ binary distribution; I) Range size variation.

Value	Present	Projected
AUC.train	0.829	0.876
AUC.test	0.852	0.802
AUC.diff	0.088	0.088
pROC	1.625	1.532
OR.p10	0.180	0.340
CBI	0.985	0.868
perc.stable	12.946	12.946
range.size	5.424	5.424

S.2.172 *Leontodon anomalus* Maxent

Species: *Leontodon anomalus* Ball

Procedure: Maxent

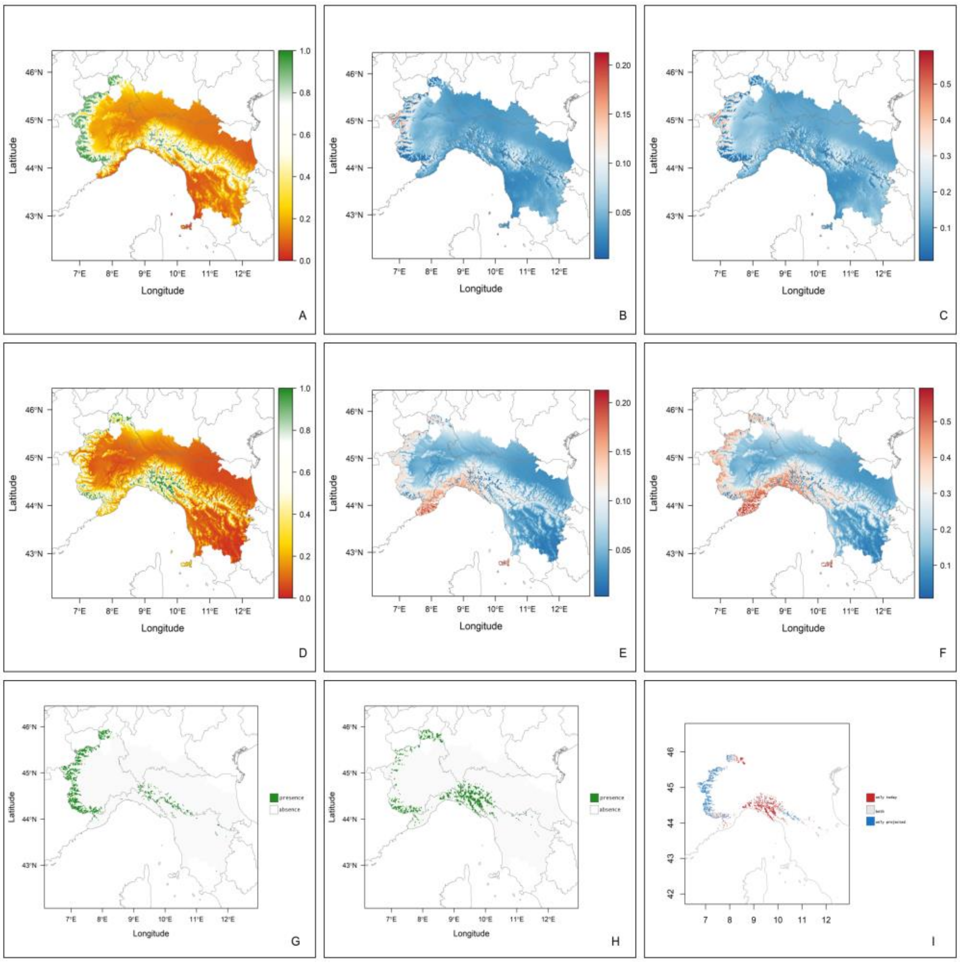

Fig. S.2.172-A) ‘Projected’ base output; B) ‘Projected’ uncertainty standard deviation; C) ‘Projected’ uncertainty range of values; D) ’Present’ base output; E) ’Present’ uncertainty standard deviation; F) ’Present’ uncertainty range of values; G) ‘Projected’ binary distribution; H) ‘Present’ binary distribution; I) Range size variation.

Value	Present	Projected
AUC.train	0.864	0.827
AUC.test	0.788	0.803
AUC.diff	0.044	0.044
pROC	1.486	1.540
OR.p10	0.300	0.220
CBI	0.949	0.689
perc.stable	27.109	27.109
range.size	1.128	1.128

S.2.173 *Leontodon anomalus* KDE

Species: *Leontodon anomalus* Ball

Procedure: KDE

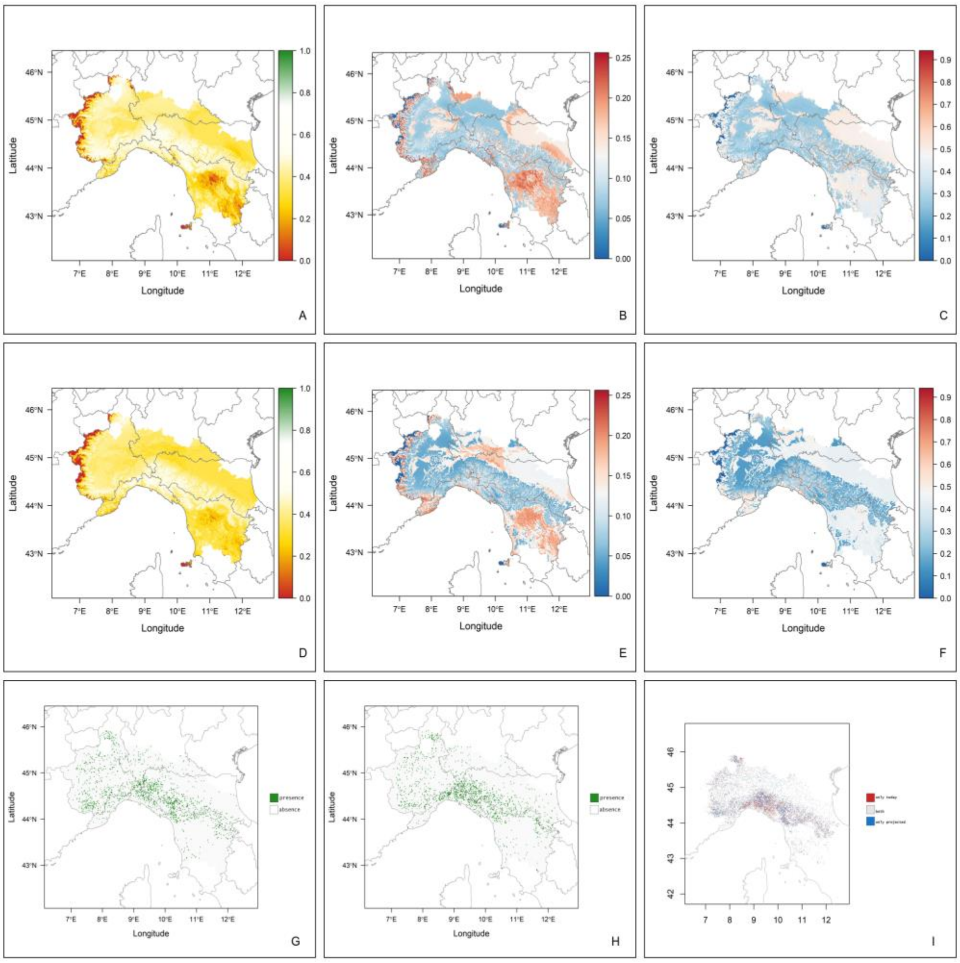

Fig. S.2.173-A) 'Projected' base output; B) 'Projected' uncertainty standard deviation; C) 'Projected' uncertainty range of values; D) 'Present' base output; E) 'Present' uncertainty standard deviation; F) 'Present' uncertainty range of values; G) 'Projected' binary distribution; H) 'Present' binary distribution; I) Range size variation.

Value	Present	Projected
AUC.train	0.834	0.812
AUC.test	0.785	0.799
AUC.diff	0.074	0.059
pROC	1.552	1.547
OR.p10	0.220	0.280
CBI	0.994	0.662
perc.stable	7.065	7.065
range.size	-0.788	-0.788

S.2.174 *Leontodon anomalus* CTA

Species: *Leontodon anomalus* Ball

Procedure: CTA

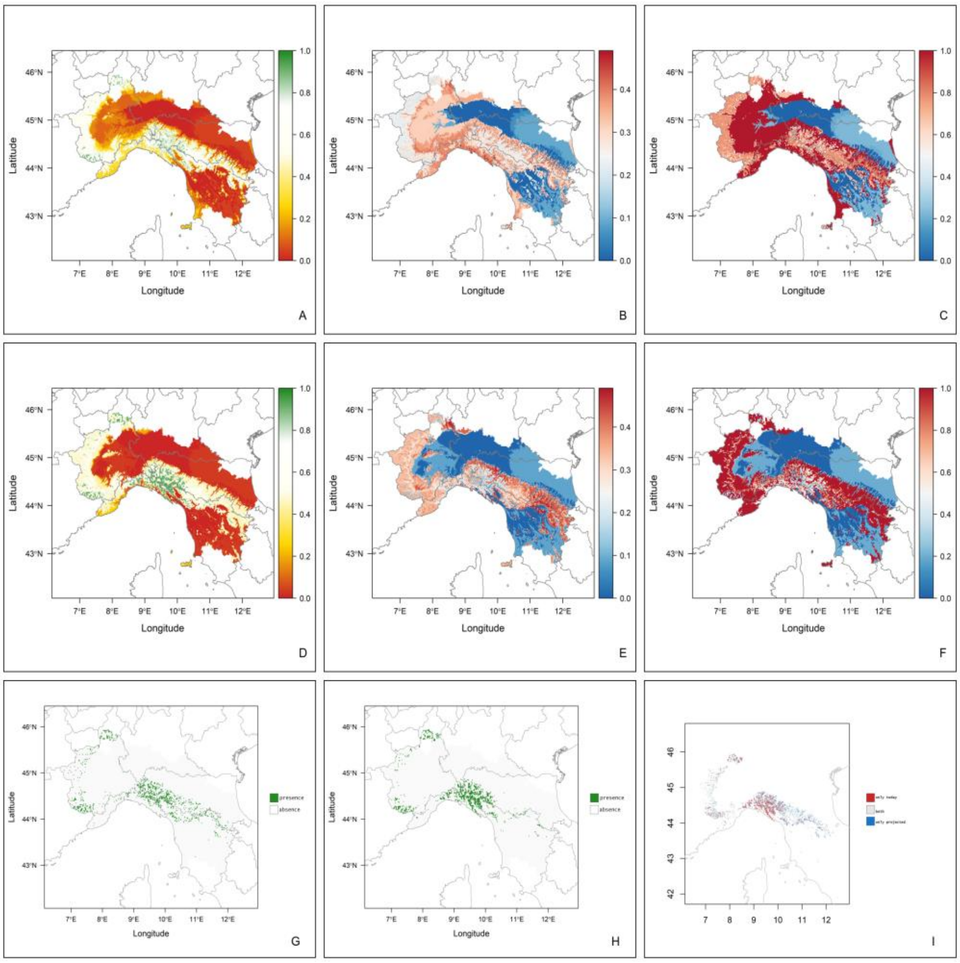

Fig. S.2.174-A) 'Projected' base output; B) 'Projected' uncertainty standard deviation; C) 'Projected' uncertainty range of values; D) 'Present' base output; E) 'Present' uncertainty standard deviation; F) 'Present' uncertainty range of values; G) 'Projected' binary distribution; H) 'Present' binary distribution; I) Range size variation.

Value	Present	Projected
AUC.train	0.953	0.924
AUC.test	0.895	0.812
AUC.diff	0.122	0.122
pROC	1.333	1.271
OR.p10	0.220	0.340
CBI	0.965	0.901
perc.stable	27.761	27.761
range.size	-2.302	-2.302

S.2.175 *Leontodon anomalus* FDA

Species: *Leontodon anomalus* Ball

Procedure: FDA

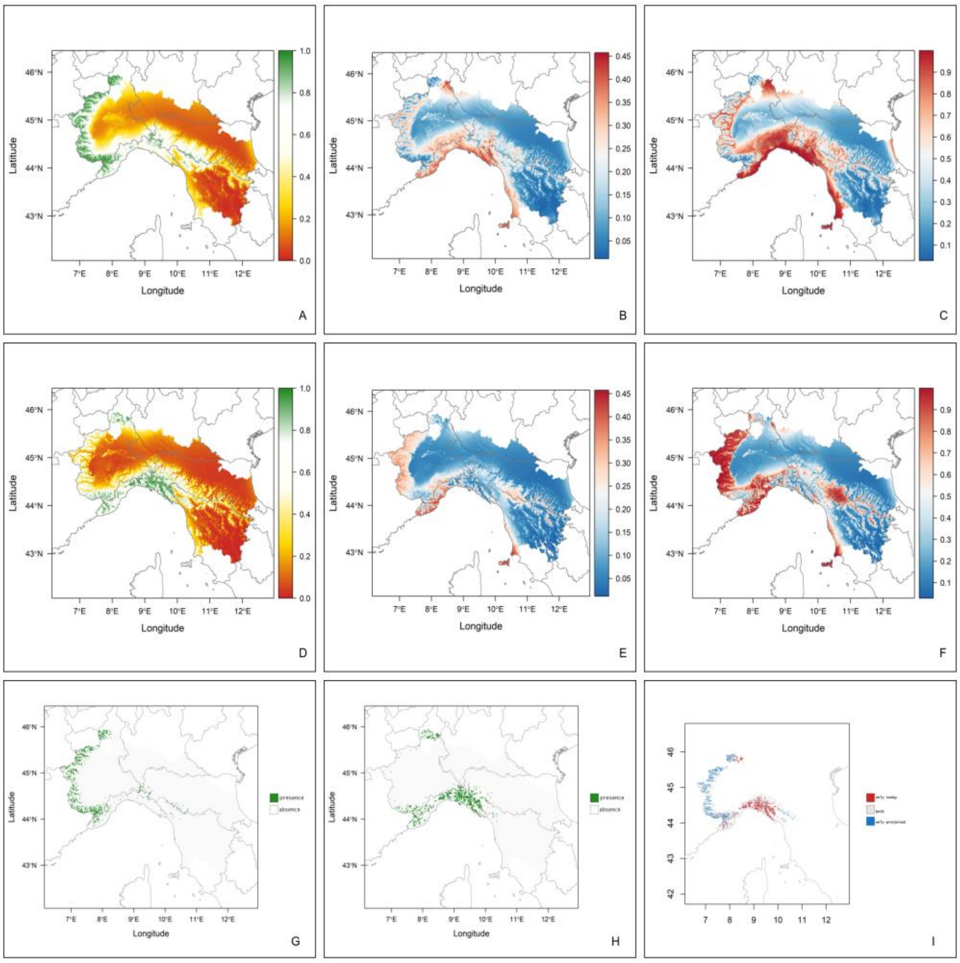

Fig. S.2.175-A) 'Projected' base output; B) 'Projected' uncertainty standard deviation; C) 'Projected' uncertainty range of values; D) 'Present' base output; E) 'Present' uncertainty standard deviation; F) 'Present' uncertainty range of values; G) 'Projected' binary distribution; H) 'Present' binary distribution; I) Range size variation.

Value	Present	Projected
AUC.train	0.865	0.853
AUC.test	0.877	0.877
AUC.diff	0.022	0.022
pROC	1.577	1.622
OR.p10	0.260	0.100
CBI	0.942	0.892
perc.stable	8.863	8.863
range.size	-0.237	-0.237

S.2.176 *Leontodon anomalus* GAM

Species: *Leontodon anomalus* Ball

Procedure: GAM

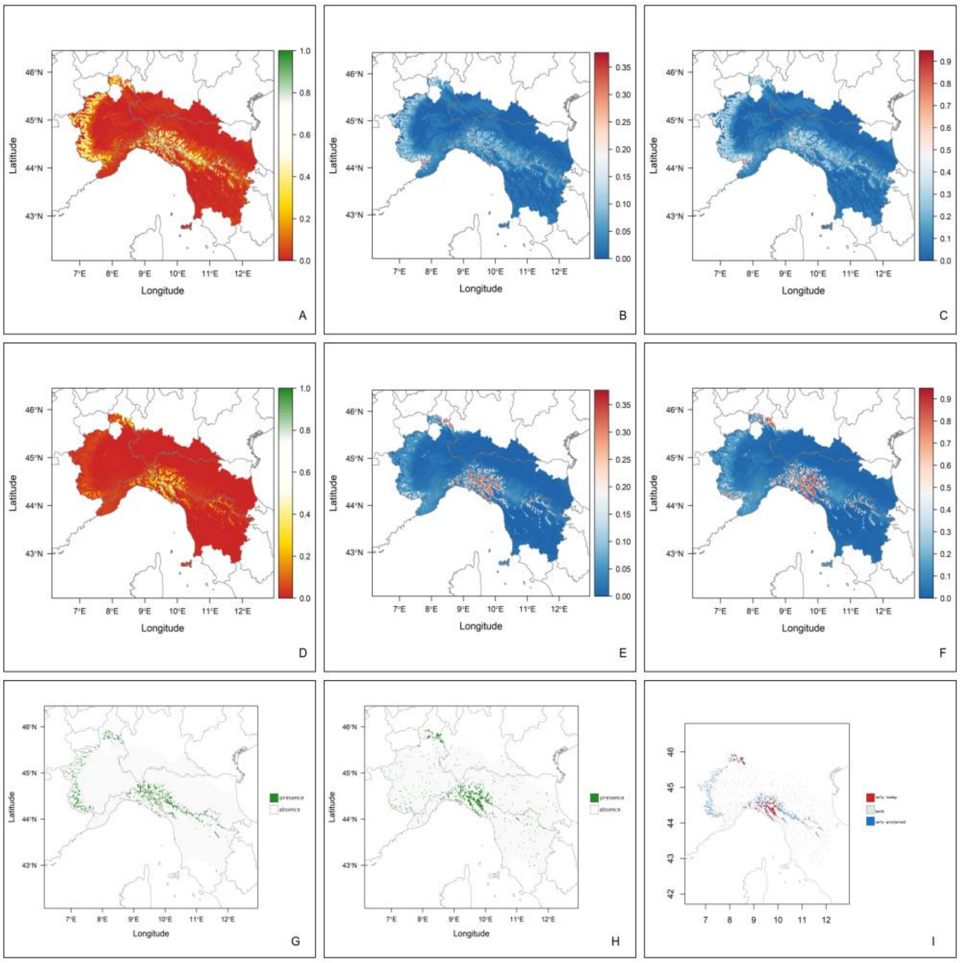

Fig. S.2.176-A) ‘Projected’ base output; B) ‘Projected’ uncertainty standard deviation; C) ‘Projected’ uncertainty range of values; D) ’Present’ base output; E) ’Present’ uncertainty standard deviation; F) ’Present’ uncertainty range of values; G) ‘Projected’ binary distribution; H) ‘Present’ binary distribution; I) Range size variation.

Value	Present	Projected
AUC.train	0.897	0.892
AUC.test	0.849	0.776
AUC.diff	0.116	0.116
pROC	1.582	1.443
OR.p10	0.340	0.400
CBI	0.797	0.748
perc.stable	15.778	15.778
range.size	0.427	0.427

S.2.177 *Leontodon anomalus* GLM

Species: *Leontodon anomalus* Ball

Procedure: GLM

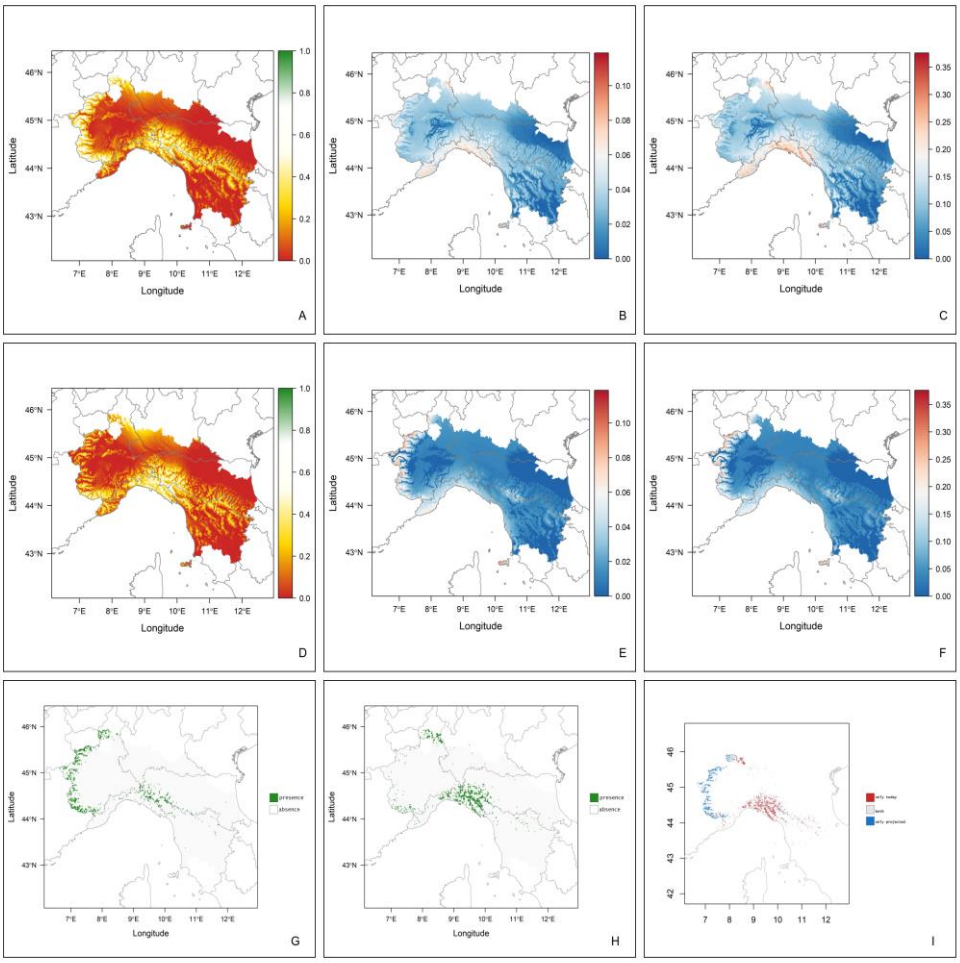

Fig. S.2.177-A) ‘Projected’ base output; B) ‘Projected’ uncertainty standard deviation; C) ‘Projected’ uncertainty range of values; D) ’Present’ base output; E) ’Present’ uncertainty standard deviation; F) ’Present’ uncertainty range of values; G) ‘Projected’ binary distribution; H) ‘Present’ binary distribution; I) Range size variation.

Value	Present	Projected
AUC.train	0.811	0.797
AUC.test	0.854	0.773
AUC.diff	0.074	0.074
pROC	1.591	1.484
OR.p10	0.160	0.240
CBI	0.914	0.873
perc.stable	19.031	19.031
range.size	-1.430	-1.430

S.2.178 *Leontodon anomalus* MARS

Species: *Leontodon anomalus* Ball

Procedure: MARS

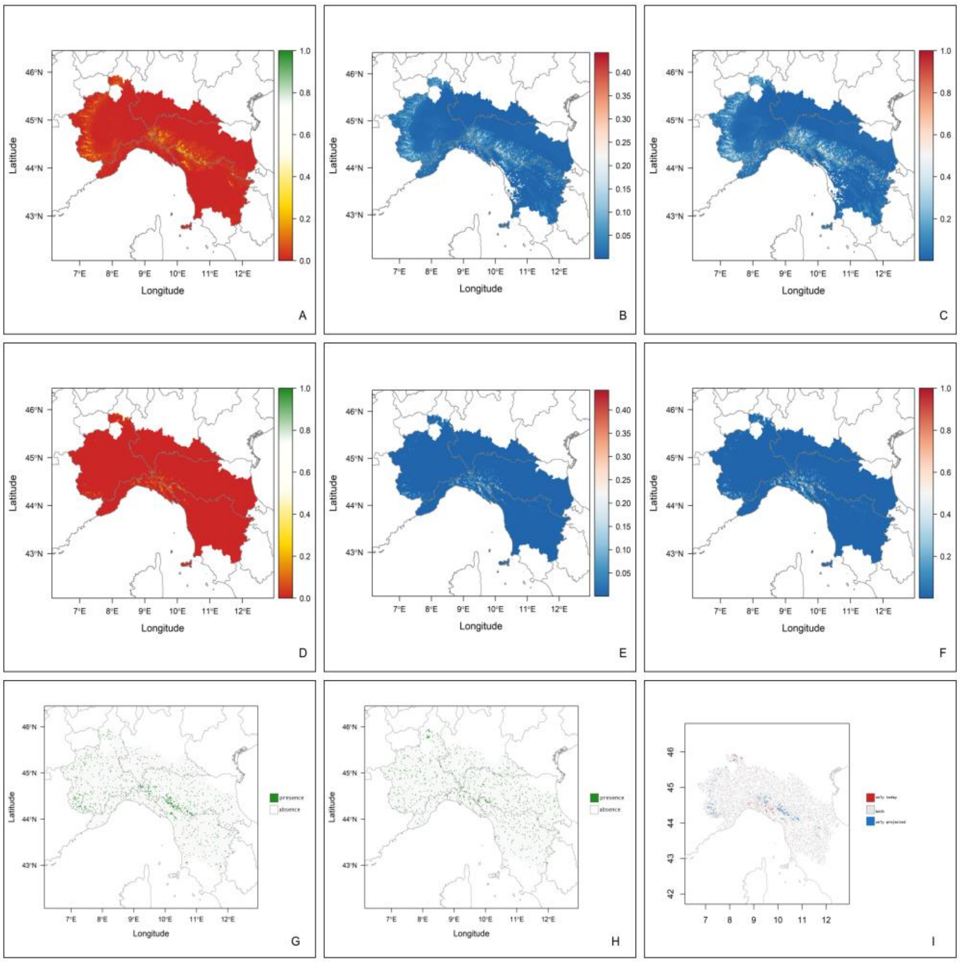

Fig. S.2.178-A) 'Projected' base output; B) 'Projected' uncertainty standard deviation; C) 'Projected' uncertainty range of values; D) 'Present' base output; E) 'Present' uncertainty standard deviation; F) 'Present' uncertainty range of values; G) 'Projected' binary distribution; H) 'Present' binary distribution; I) Range size variation.

Value	Present	Projected
AUC.train	0.787	0.924
AUC.test	0.733	0.861
AUC.diff	0.064	0.064
pROC	1.196	1.595
OR.p10	0.120	0.400
CBI	0.800	0.785
perc.stable	2.728	2.728
range.size	0.345	0.345

S.2.179 *Leontodon anomalus* RF

Species: *Leontodon anomalus* Ball

Procedure: RF

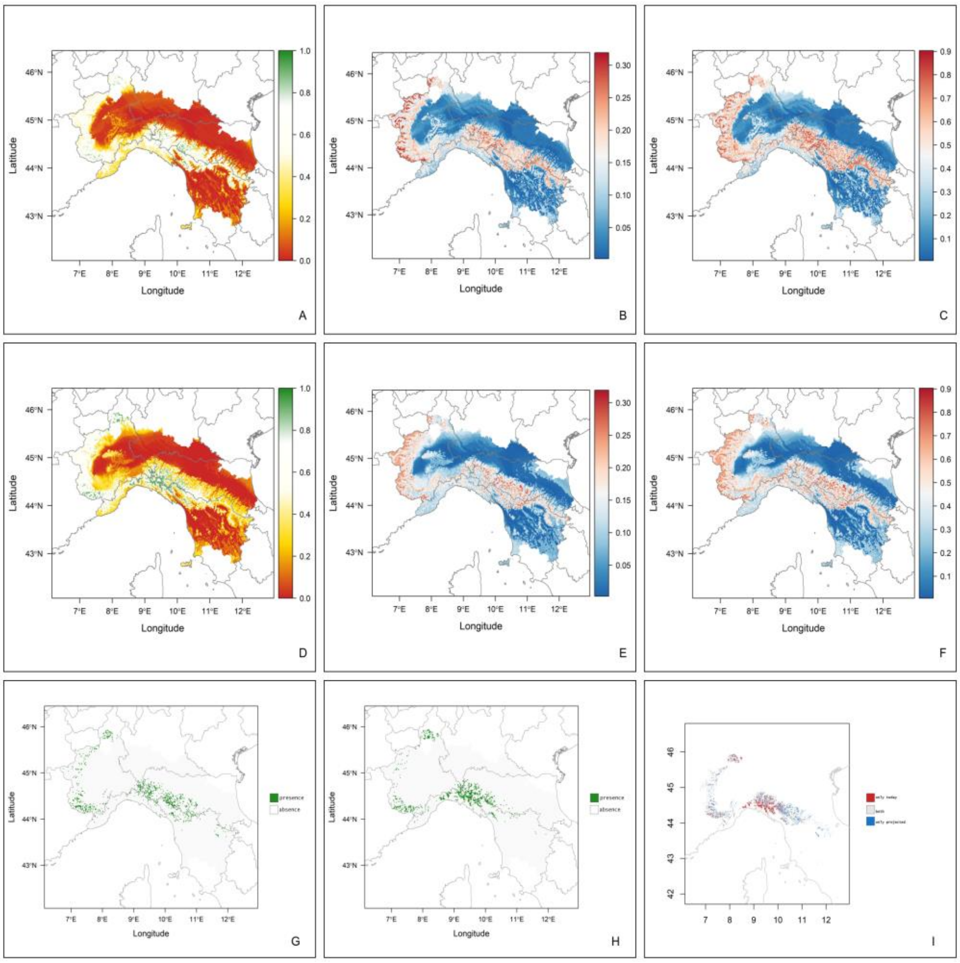

Fig. S.2.179-A) 'Projected' base output; B) 'Projected' uncertainty standard deviation; C) 'Projected' uncertainty range of values; D) 'Present' base output; E) 'Present' uncertainty standard deviation; F) 'Present' uncertainty range of values; G) 'Projected' binary distribution; H) 'Present' binary distribution; I) Range size variation.

Value	Present	Projected
AUC.train	1.000	1.000
AUC.test	0.980	0.924
AUC.diff	0.076	0.076
pROC	1.681	1.522
OR.p10	0.580	0.620
CBI	0.950	0.939
perc.stable	16.487	16.487
range.size	3.672	3.672

S.2.180 *Leontodon anomalus* SVM

Species: *Leontodon anomalus* Ball

Procedure: SVM

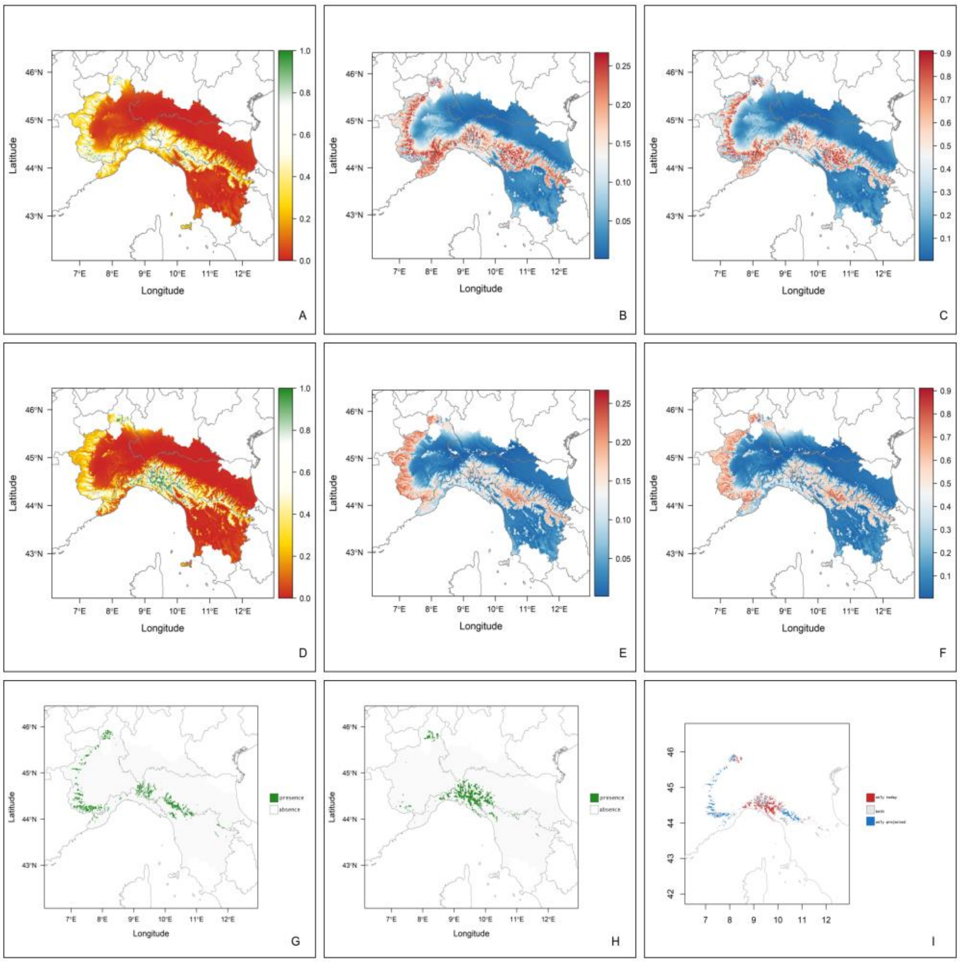

Fig. S.2.180-A) 'Projected' base output; B) 'Projected' uncertainty standard deviation; C) 'Projected' uncertainty range of values; D) 'Present' base output; E) 'Present' uncertainty standard deviation; F) 'Present' uncertainty range of values; G) 'Projected' binary distribution; H) 'Present' binary distribution; I) Range size variation.

Value	Present	Projected
AUC.train	0.920	0.919
AUC.test	0.866	0.864
AUC.diff	0.069	0.069
pROC	1.549	1.539
OR.p10	0.360	0.280
CBI	0.961	0.784
perc.stable	19.519	19.519
range.size	3.962	3.962

S.2.181 *Linaria purpurea* Biomod2

Species: *Linaria purpurea* (L.) Mill.

Procedure: Biomod2

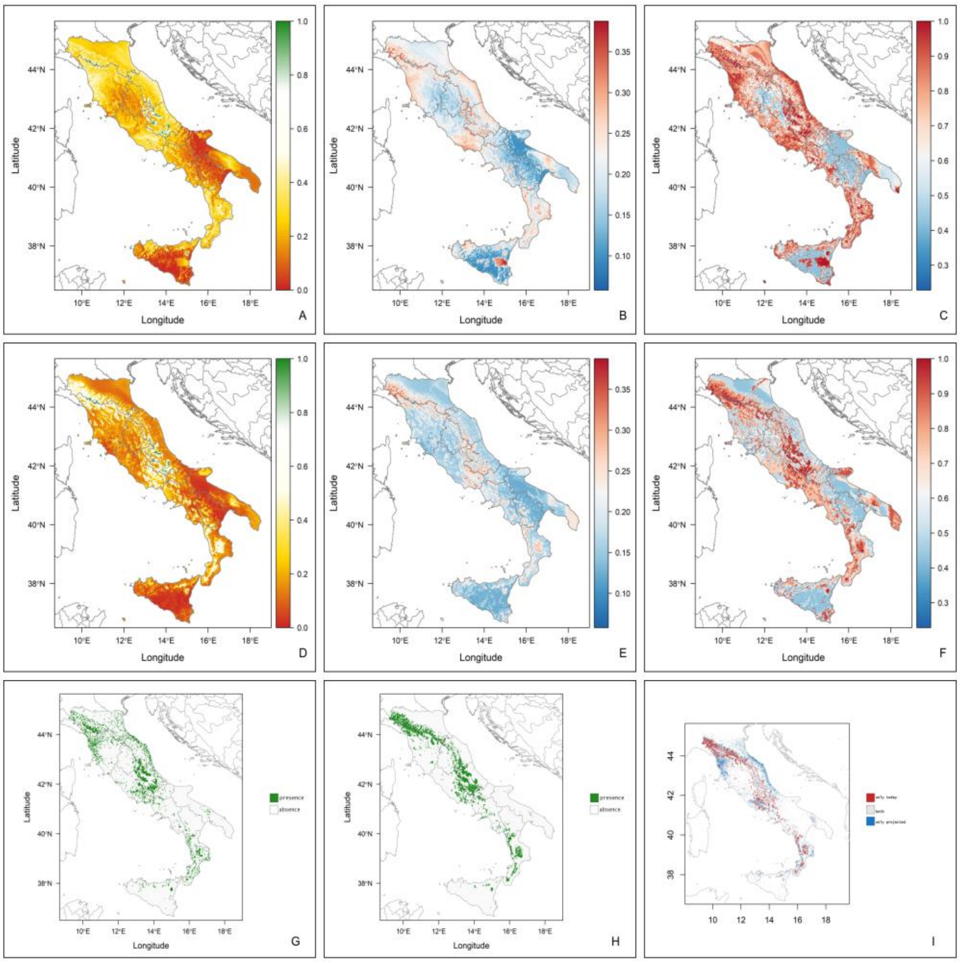

Fig. S.2.181-A) 'Projected' base output; B) 'Projected' uncertainty standard deviation; C) 'Projected' uncertainty range of values; D) 'Present' base output; E) 'Present' uncertainty standard deviation; F) 'Present' uncertainty range of values; G) 'Projected' binary distribution; H) 'Present' binary distribution; I) Range size variation.

Value	Present	Projected
AUC.train	0.941	0.968
AUC.test	0.736	0.722
AUC.diff	0.246	0.246
pROC	1.120	1.142
OR.p10	0.522	0.646
CBI	0.982	0.995
perc.stable	28.380	28.380
range.size	-0.371	-0.371

S.2.182 *Linaria purpurea* Bioclim

Species: *Linaria purpurea* (L.) Mill.

Procedure: Bioclim

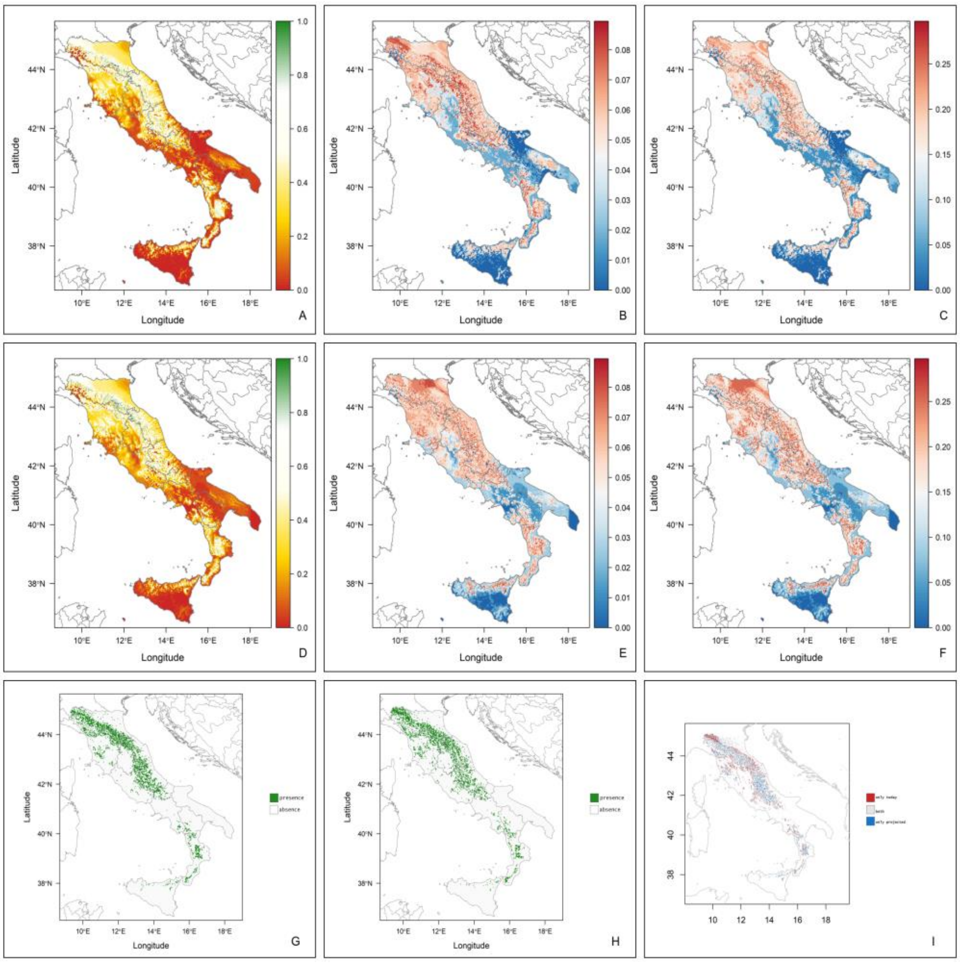

Fig. S.2.182-A) 'Projected' base output; B) 'Projected' uncertainty standard deviation; C) 'Projected' uncertainty range of values; D) 'Present' base output; E) 'Present' uncertainty standard deviation; F) 'Present' uncertainty range of values; G) 'Projected' binary distribution; H) 'Present' binary distribution; I) Range size variation.

Value	Present	Projected
AUC.train	0.546	0.581
AUC.test	0.506	0.538
AUC.diff	0.059	0.059
pROC	1.036	1.058
OR.p10	0.152	0.177
CBI	-0.120	0.170
perc.stable	52.522	52.522
range.size	-0.835	-0.835

S.2.183 *Linaria purpurea* Domain

Species: *Linaria purpurea* (L.) Mill.

Procedure: Domain

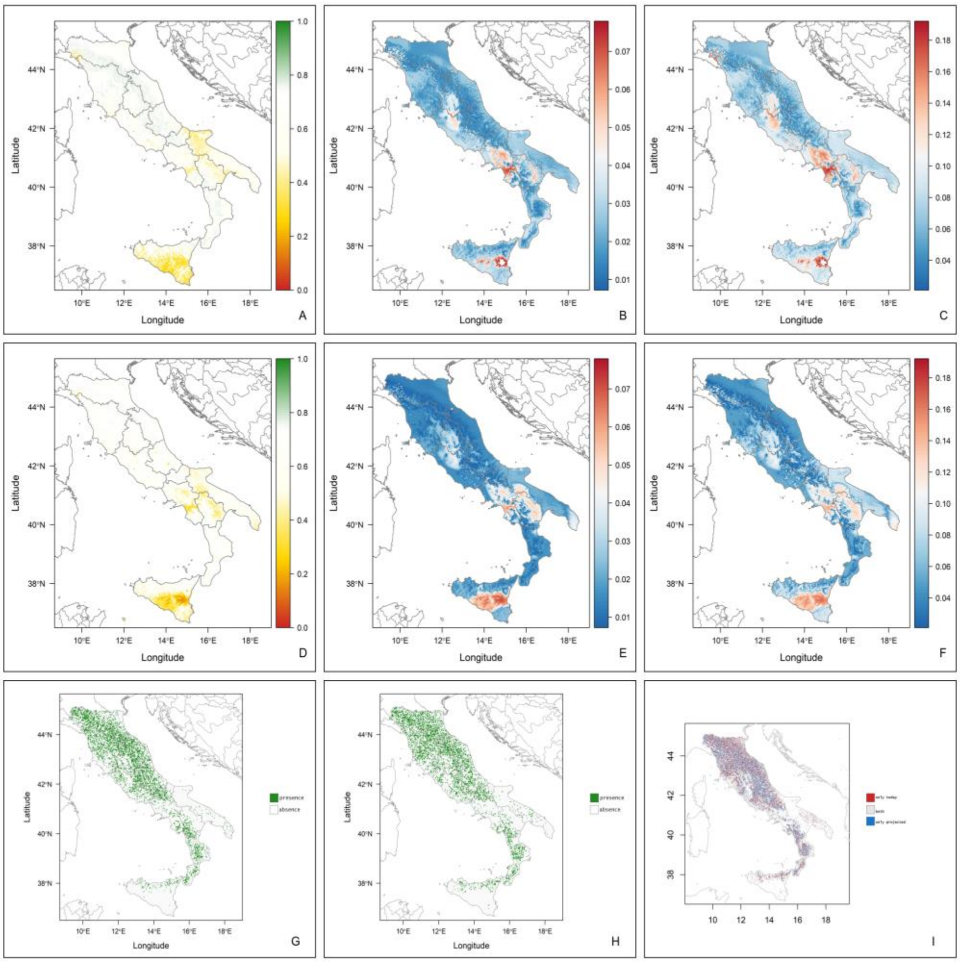

Fig. S.2.183-A) 'Projected' base output; B) 'Projected' uncertainty standard deviation; C) 'Projected' uncertainty range of values; D) 'Present' base output; E) 'Present' uncertainty standard deviation; F) 'Present' uncertainty range of values; G) 'Projected' binary distribution; H) 'Present' binary distribution; I) Range size variation.

Value	Present	Projected
AUC.train	0.508	0.558
AUC.test	0.502	0.531
AUC.diff	0.058	0.058
pROC	1.069	1.095
OR.p10	0.143	0.139
CBI	0.887	0.886
perc.stable	22.789	22.789
range.size	-0.174	-0.174

S.2.184 *Linaria purpurea* Maxent

Species: *Linaria purpurea* (L.) Mill.

Procedure: Maxent

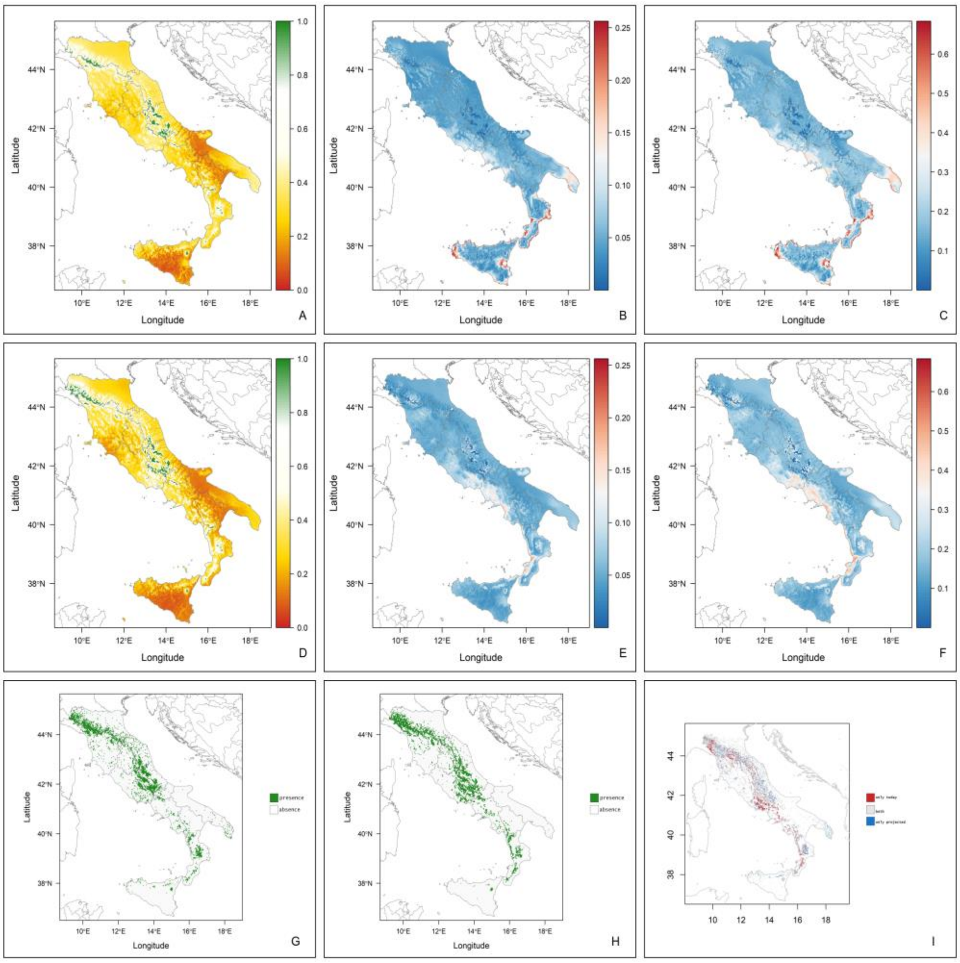

Fig. S.2.184-A) 'Projected' base output; B) 'Projected' uncertainty standard deviation; C) 'Projected' uncertainty range of values; D) 'Present' base output; E) 'Present' uncertainty standard deviation; F) 'Present' uncertainty range of values; G) 'Projected' binary distribution; H) 'Present' binary distribution; I) Range size variation.

Value	Present	Projected
AUC.train	0.772	0.742
AUC.test	0.727	0.699
AUC.diff	0.056	0.056
pROC	1.127	1.127
OR.p10	0.165	0.155
CBI	0.974	0.982
perc.stable	45.825	45.825
range.size	-0.064	-0.064

S.2.185 *Linaria purpurea* KDE

Species: *Linaria purpurea* (L.) Mill.

Procedure: KDE

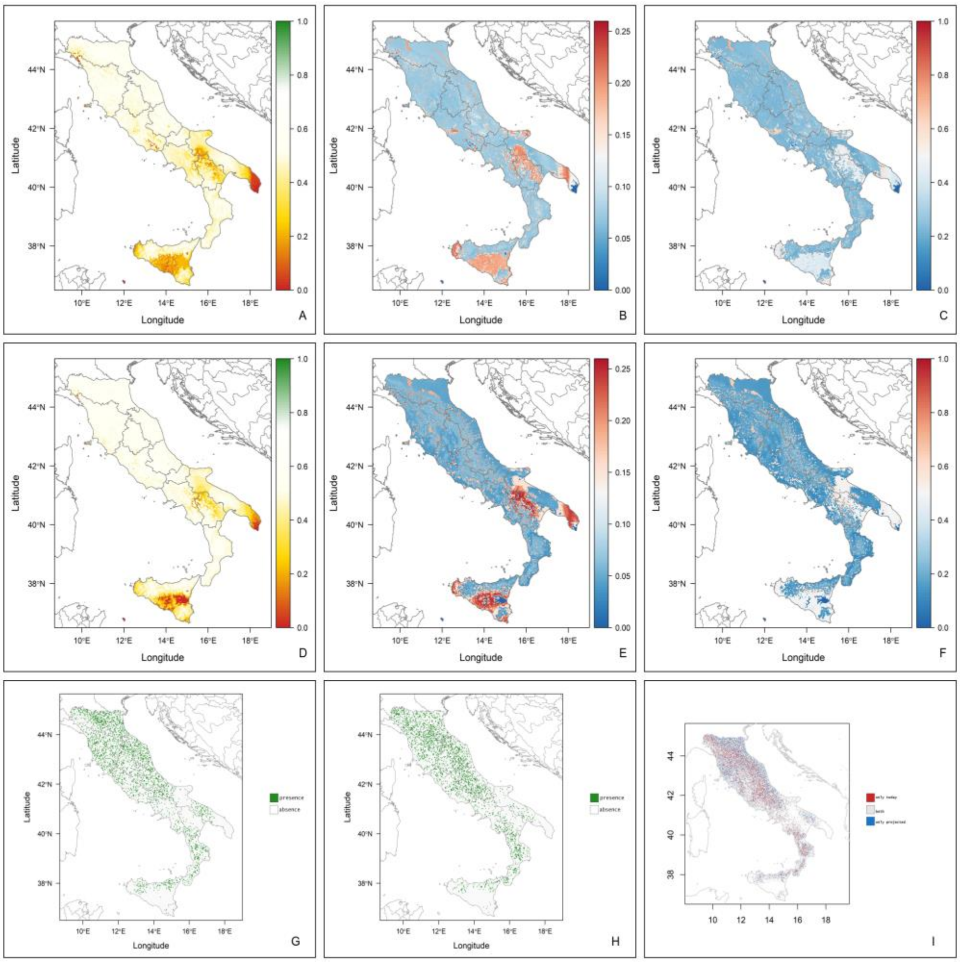

Fig. S.2.185-A) ‘Projected’ base output; B) ‘Projected’ uncertainty standard deviation; C) ‘Projected’ uncertainty range of values; D) ’Present’ base output; E) ’Present’ uncertainty standard deviation; F) ’Present’ uncertainty range of values; G) ‘Projected’ binary distribution; H) ‘Present’ binary distribution; I) Range size variation.

Value	Present	Projected
AUC.train	0.643	0.553
AUC.test	0.593	0.498
AUC.diff	0.056	0.060
pROC	1.123	1.088
OR.p10	0.130	0.143
CBI	0.867	0.526
perc.stable	9.716	9.716
range.size	-2.163	-2.163

S.2.186 *Linaria purpurea* CTA

Species: *Linaria purpurea* (L.) Mill.

Procedure: CTA

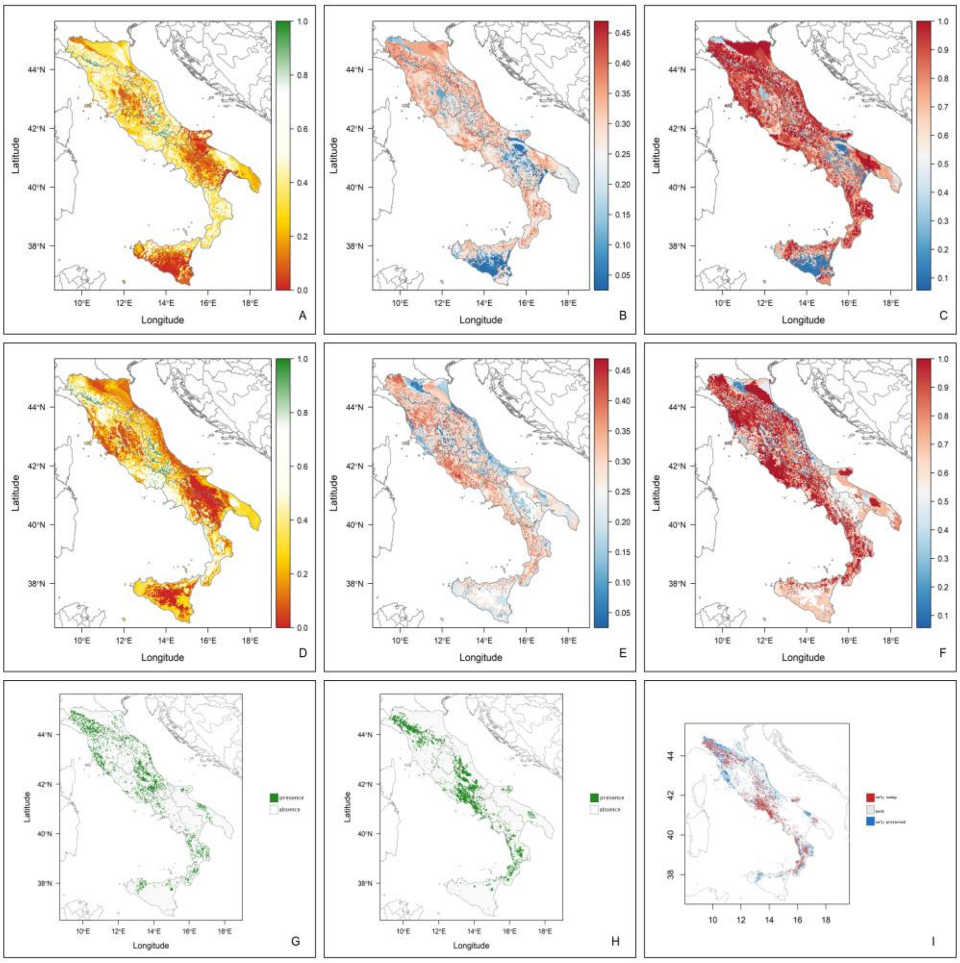

Fig. S.2.186-A) ‘Projected’ base output; B) ‘Projected’ uncertainty standard deviation; C) ‘Projected’ uncertainty range of values; D) ’Present’ base output; E) ’Present’ uncertainty standard deviation; F) ’Present’ uncertainty range of values; G) ‘Projected’ binary distribution; H) ‘Present’ binary distribution; I) Range size variation.

Value	Present	Projected
AUC.train	0.921	0.917
AUC.test	0.799	0.768
AUC.diff	0.149	0.149
pROC	1.081	1.075
OR.p10	0.278	0.344
CBI	0.995	0.983
perc.stable	19.211	19.211
range.size	-0.657	-0.657

S.2.187 *Linaria purpurea* FDA

Species: *Linaria purpurea* (L.) Mill.

Procedure: FDA

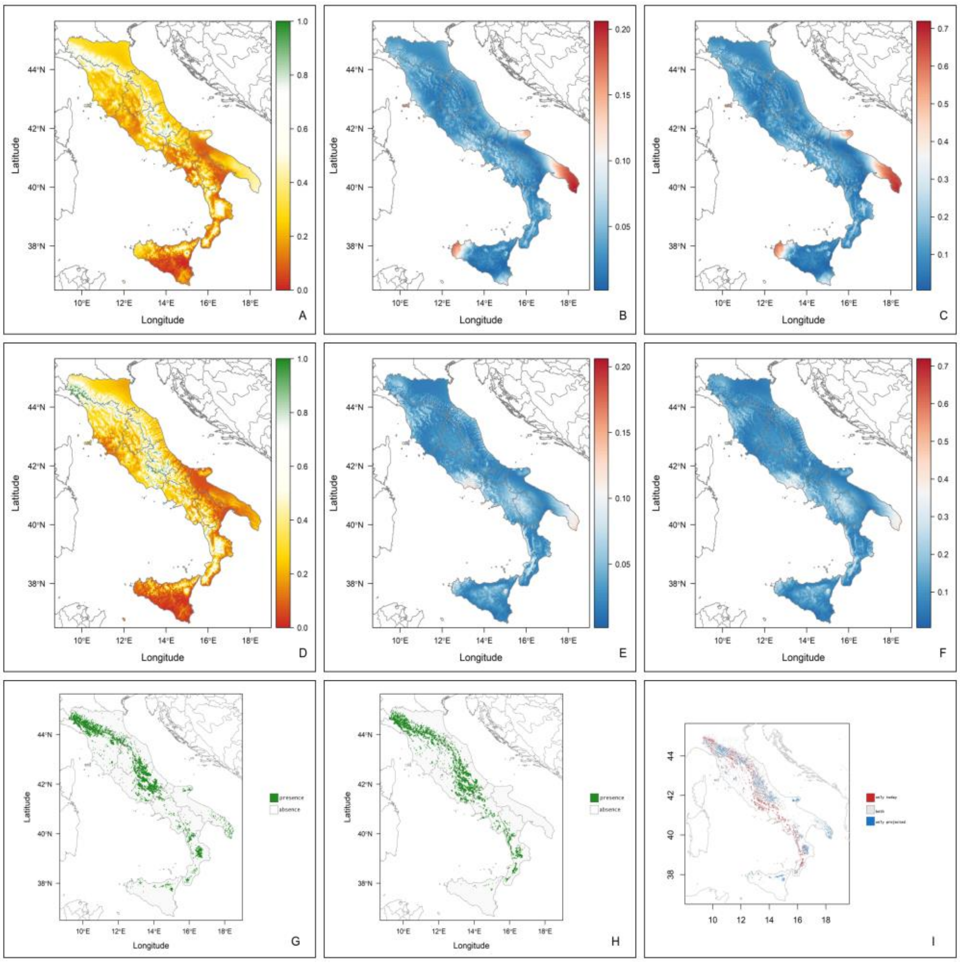

Fig. S.2.187-A) 'Projected' base output; B) 'Projected' uncertainty standard deviation; C) 'Projected' uncertainty range of values; D) 'Present' base output; E) 'Present' uncertainty standard deviation; F) 'Present' uncertainty range of values; G) 'Projected' binary distribution; H) 'Present' binary distribution; I) Range size variation.

Value	Present	Projected
AUC.train	0.767	0.713
AUC.test	0.768	0.696
AUC.diff	0.039	0.039
pROC	1.155	1.116
OR.p10	0.122	0.145
CBI	0.977	0.991
perc.stable	49.962	49.962
range.size	0.865	0.865

S.2.188 *Linaria purpurea* GAM

Species: *Linaria purpurea* (L.) Mill.

Procedure: GAM

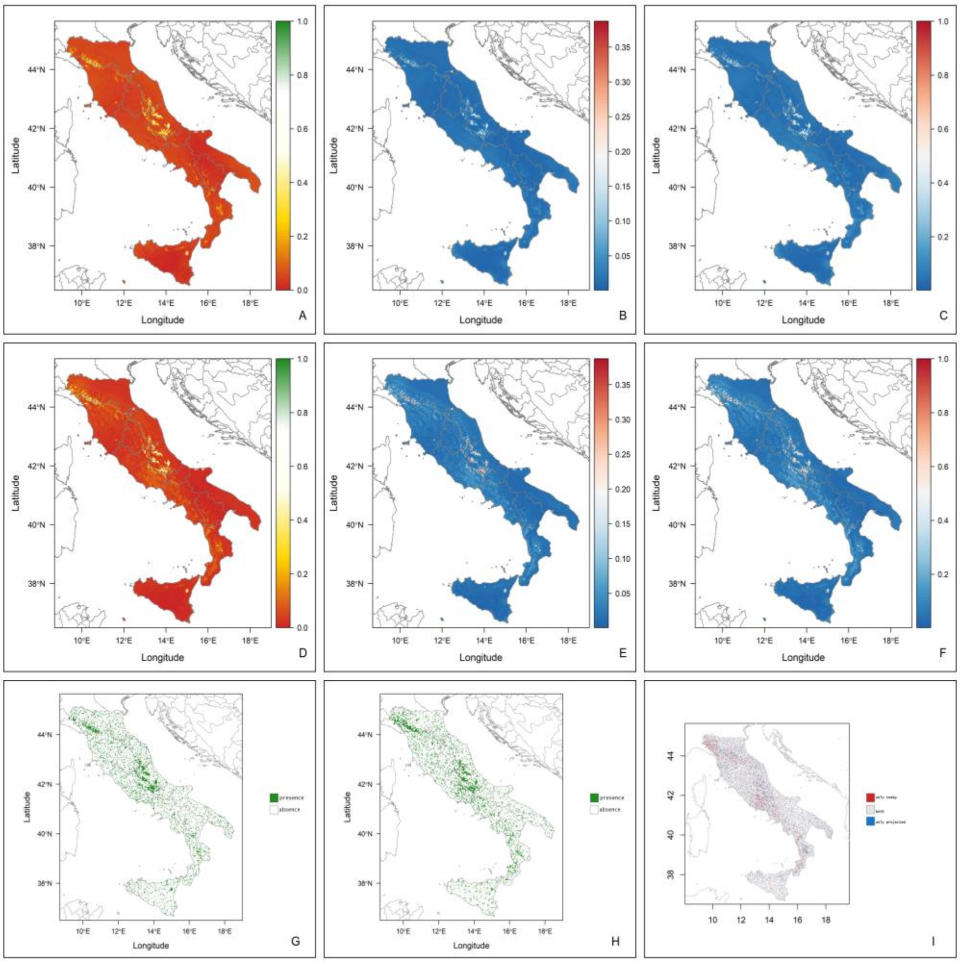

Fig. S.2.188-A) 'Projected' base output; B) 'Projected' uncertainty standard deviation; C) 'Projected' uncertainty range of values; D) 'Present' base output; E) 'Present' uncertainty standard deviation; F) 'Present' uncertainty range of values; G) 'Projected' binary distribution; H) 'Present' binary distribution; I) Range size variation.

Value	Present	Projected
AUC.train	0.776	0.748
AUC.test	0.741	0.683
AUC.diff	0.068	0.068
pROC	1.117	1.103
OR.p10	0.152	0.186
CBI	0.915	0.802
perc.stable	14.464	14.464
range.size	-1.654	-1.654

S.2.189 *Linaria purpurea* GLM

Species: *Linaria purpurea* (L.) Mill.

Procedure: GLM

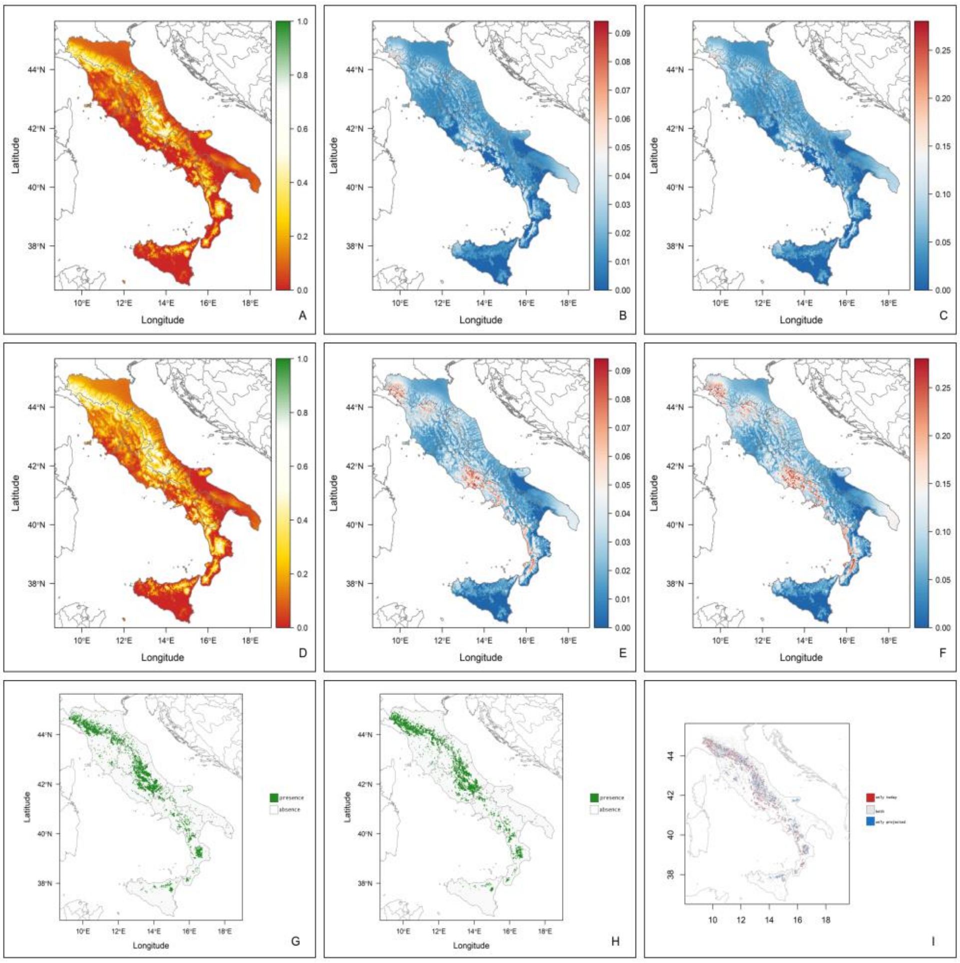

Fig. S.2.189-A) 'Projected' base output; B) 'Projected' uncertainty standard deviation; C) 'Projected' uncertainty range of values; D) 'Present' base output; E) 'Present' uncertainty standard deviation; F) 'Present' uncertainty range of values; G) 'Projected' binary distribution; H) 'Present' binary distribution; I) Range size variation.

Value	Present	Projected
AUC.train	0.751	0.696
AUC.test	0.706	0.671
AUC.diff	0.046	0.046
pROC	1.099	1.071
OR.p10	0.157	0.075
CBI	0.989	0.887
perc.stable	48.066	48.066
range.size	0.940	0.940

S.2.190 *Linaria purpurea* MARS

Species: *Linaria purpurea* (L.) Mill.

Procedure: MARS

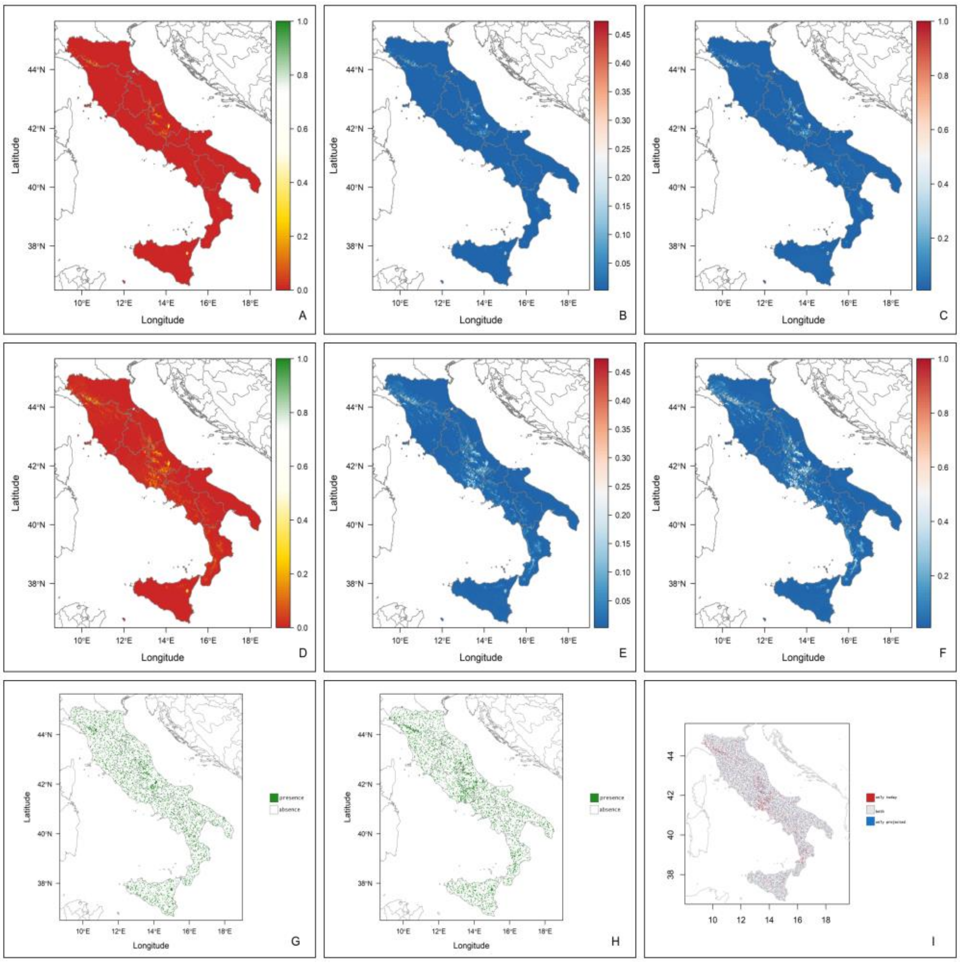

Fig. S.2.190-A) ‘Projected’ base output; B) ‘Projected’ uncertainty standard deviation; C) ‘Projected’ uncertainty range of values; D) ’Present’ base output; E) ’Present’ uncertainty standard deviation; F) ’Present’ uncertainty range of values; G) ‘Projected’ binary distribution; H) ‘Present’ binary distribution; I) Range size variation.

Value	Present	Projected
AUC.train	0.778	0.686
AUC.test	0.714	0.602
AUC.diff	0.084	0.084
pROC	1.058	1.017
OR.p10	0.157	0.127
CBI	0.598	0.644
perc.stable	8.737	8.737
range.size	0.181	0.181

S.2.191 *Linaria purpurea* RF

Species: *Linaria purpurea* (L.) Mill.

Procedure: RF

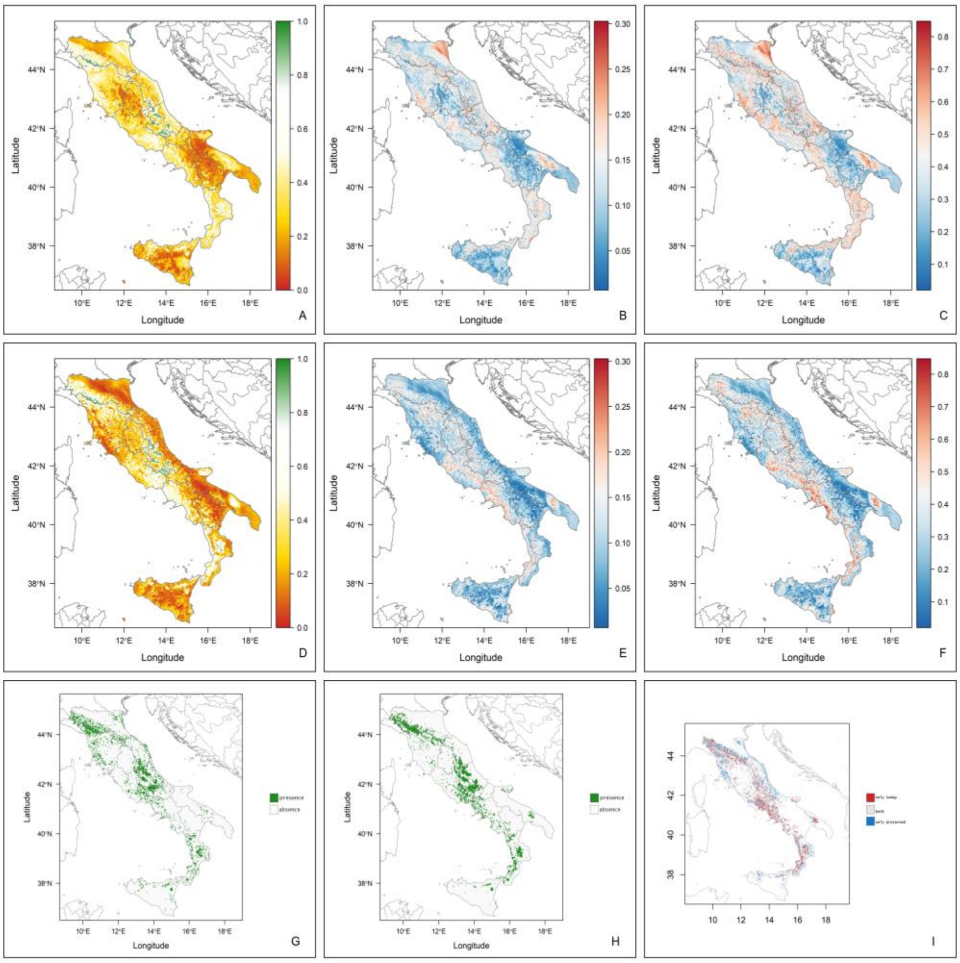

Fig. S.2.191-A) 'Projected' base output; B) 'Projected' uncertainty standard deviation; C) 'Projected' uncertainty range of values; D) 'Present' base output; E) 'Present' uncertainty standard deviation; F) 'Present' uncertainty range of values; G) 'Projected' binary distribution; H) 'Present' binary distribution; I) Range size variation.

Value	Present	Projected
AUC.train	1.000	1.000
AUC.test	0.938	0.918
AUC.diff	0.082	0.082
pROC	1.213	1.133
OR.p10	0.617	0.645
CBI	0.999	0.971
perc.stable	31.298	31.298
range.size	-0.419	-0.419

S.2.192 *Linaria purpurea* SVM

Species: *Linaria purpurea* (L.) Mill.

Procedure: SVM

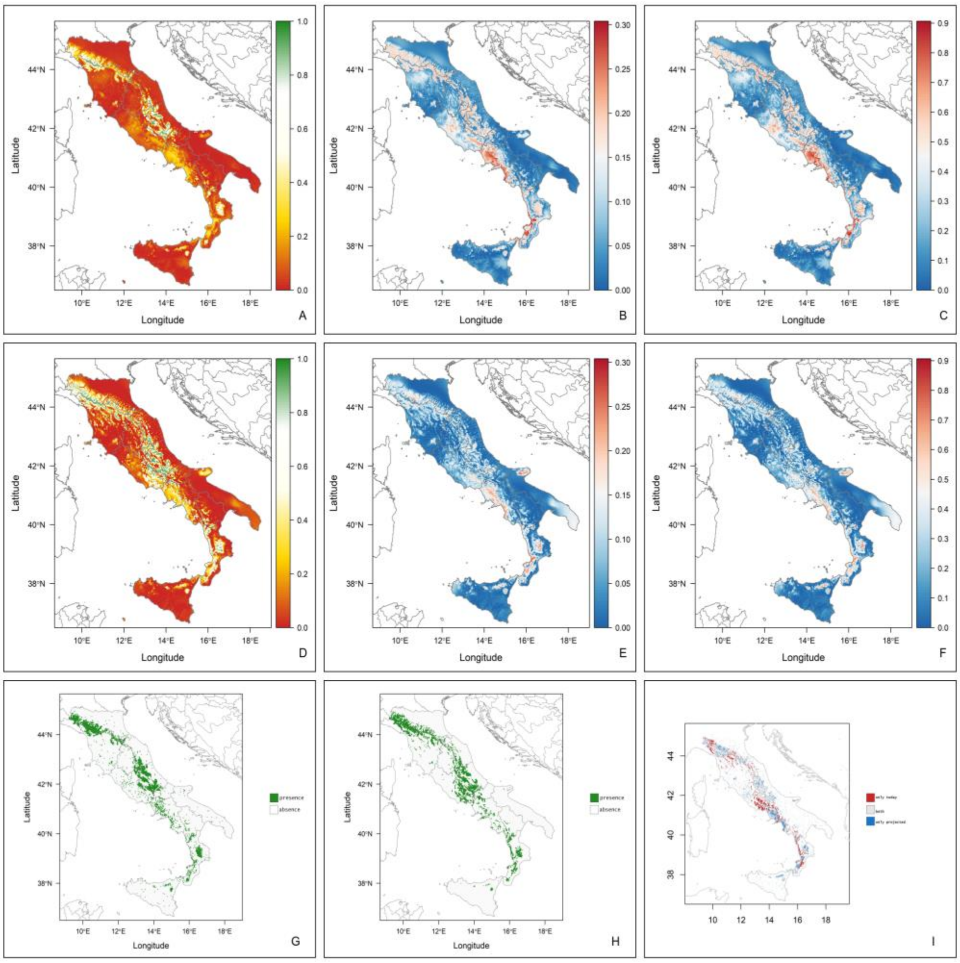

Fig. S.2.192-A) 'Projected' base output; B) 'Projected' uncertainty standard deviation; C) 'Projected' uncertainty range of values; D) 'Present' base output; E) 'Present' uncertainty standard deviation; F) 'Present' uncertainty range of values; G) 'Projected' binary distribution; H) 'Present' binary distribution; I) Range size variation.

Value	Present	Projected
AUC.train	0.836	0.780
AUC.test	0.796	0.732
AUC.diff	0.055	0.055
pROC	1.115	1.079
OR.p10	0.152	0.148
CBI	0.865	0.887
perc.stable	46.575	46.575
range.size	0.008	0.008

S.2.193 *Ornithogalum etruscum* Biomod2

Species: *Ornithogalum etruscum* Parl.

Procedure: Biomod2

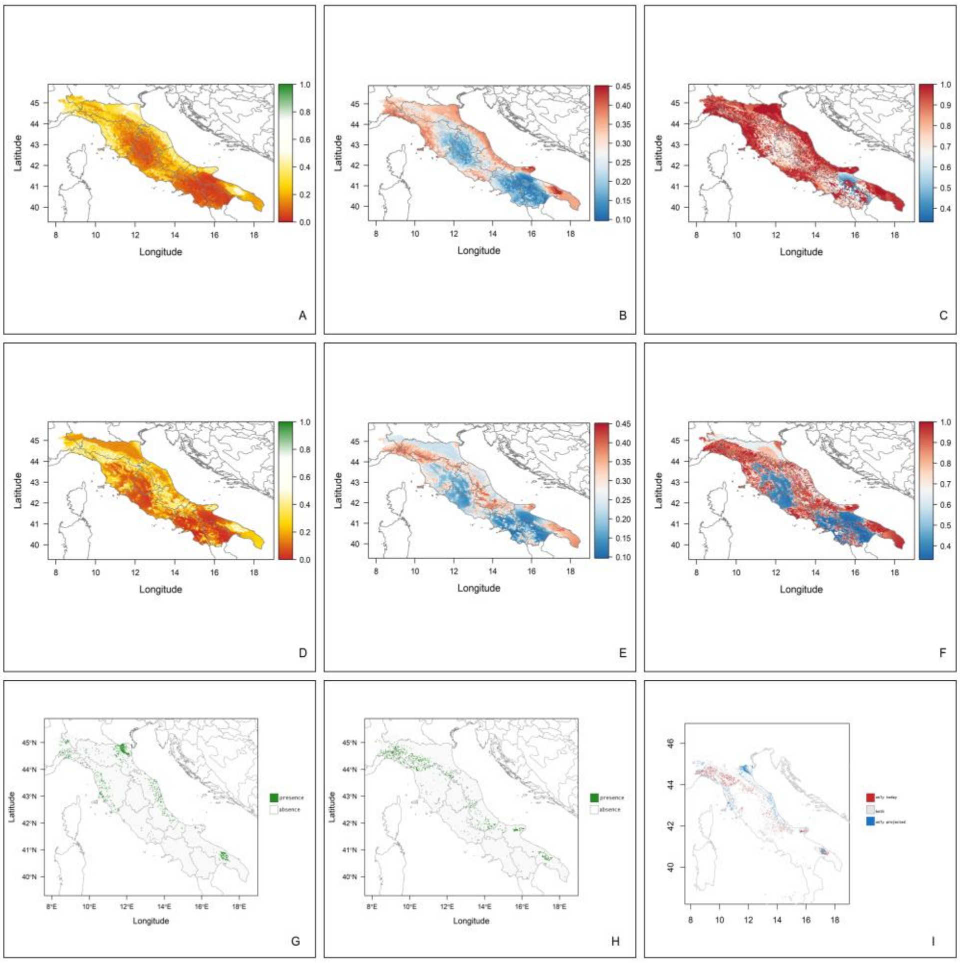

Fig. S.2.193-A) 'Projected' base output; B) 'Projected' uncertainty standard deviation; C) 'Projected' uncertainty range of values; D) 'Present' base output; E) 'Present' uncertainty standard deviation; F) 'Present' uncertainty range of values; G) 'Projected' binary distribution; H) 'Present' binary distribution; I) Range size variation.

Value	Present	Projected
AUC.train	1.000	1.000
AUC.test	0.639	0.587
AUC.diff	0.413	0.413
pROC	1.278	1.241
OR.p10	1.000	1.000
CBI	0.911	0.329
perc.stable	2.367	2.367
range.size	0.894	0.894

S.2.194 *Ornithogalum etruscum* Bioclim

Species: *Ornithogalum etruscum* Parl.

Procedure: Bioclim

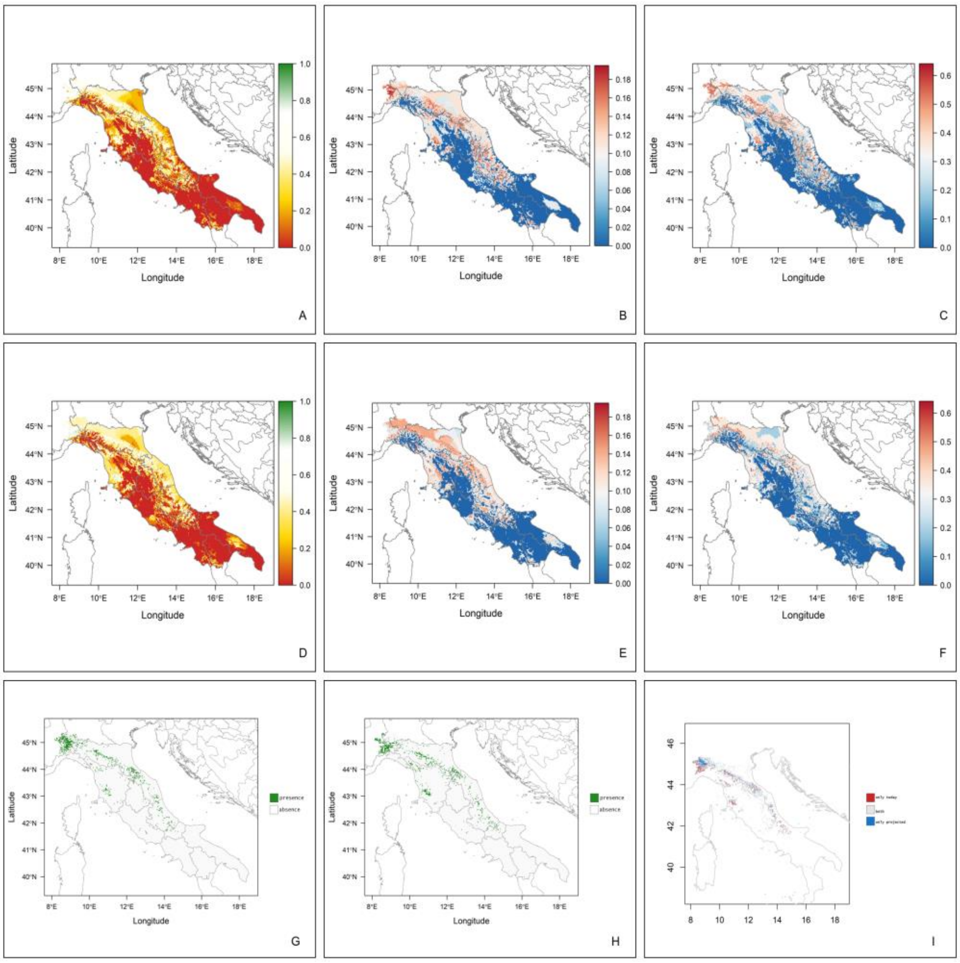

Fig. S.2.194-A) ‘Projected’ base output; B) ‘Projected’ uncertainty standard deviation; C) ‘Projected’ uncertainty range of values; D) ’Present’ base output; E) ’Present’ uncertainty standard deviation; F) ’Present’ uncertainty range of values; G) ‘Projected’ binary distribution; H) ‘Present’ binary distribution; I) Range size variation.

Value	Present	Projected
AUC.train	0.647	0.666
AUC.test	0.578	0.564
AUC.diff	0.102	0.102
pROC	1.148	1.151
OR.p10	0.310	0.310
CBI	-0.311	-0.401
perc.stable	22.271	22.271
range.size	-1.225	-1.225

S.2.195 *Ornithogalum etruscum* Domain

Species: *Ornithogalum etruscum* Parl.

Procedure: Domain

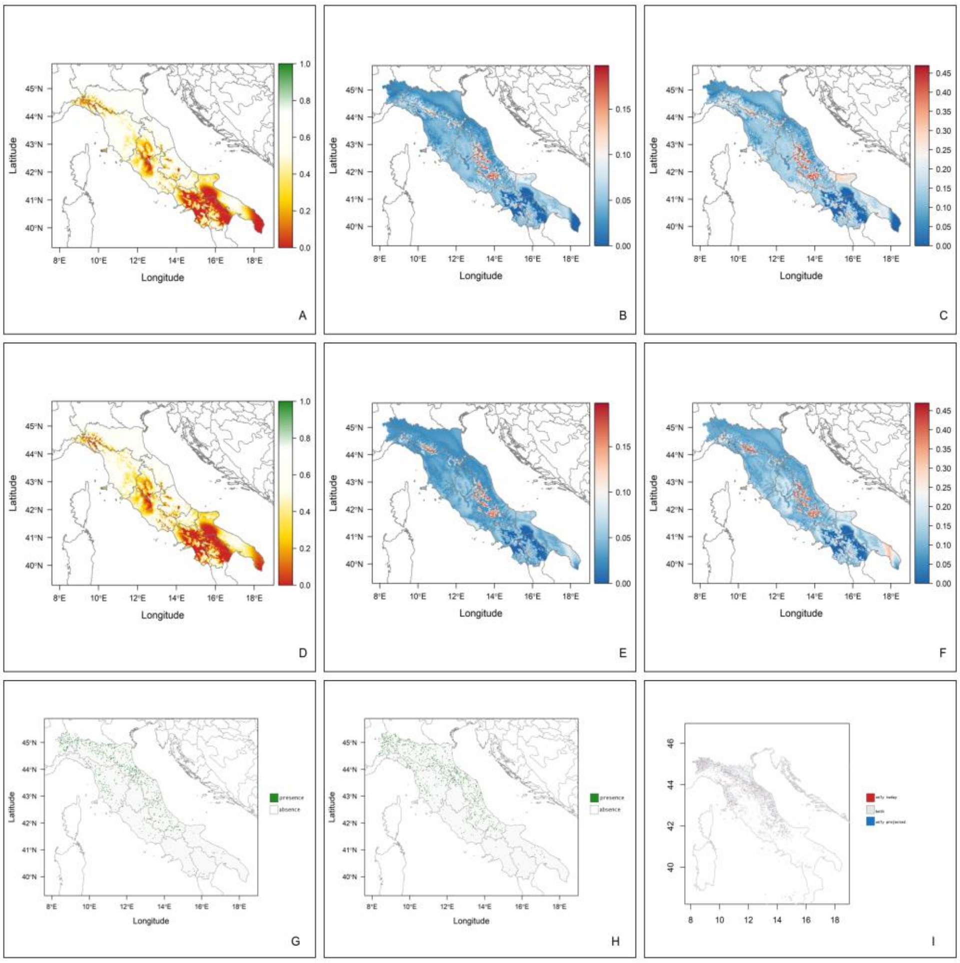

Fig. S.2.195-A) ‘Projected’ base output; B) ‘Projected’ uncertainty standard deviation; C) ‘Projected’ uncertainty range of values; D) ’Present’ base output; E) ’Present’ uncertainty standard deviation; F) ’Present’ uncertainty range of values; G) ‘Projected’ binary distribution; H) ‘Present’ binary distribution; I) Range size variation.

Value	Present	Projected
AUC.train	0.609	0.629
AUC.test	0.536	0.561
AUC.diff	0.076	0.076
pROC	1.248	1.248
OR.p10	0.390	0.303
CBI	0.642	0.329
perc.stable	5.018	5.018
range.size	-0.694	-0.694

S.2.196 *Ornithogalum etruscum* Maxent

Species: *Ornithogalum etruscum* Parl.

Procedure: Maxent

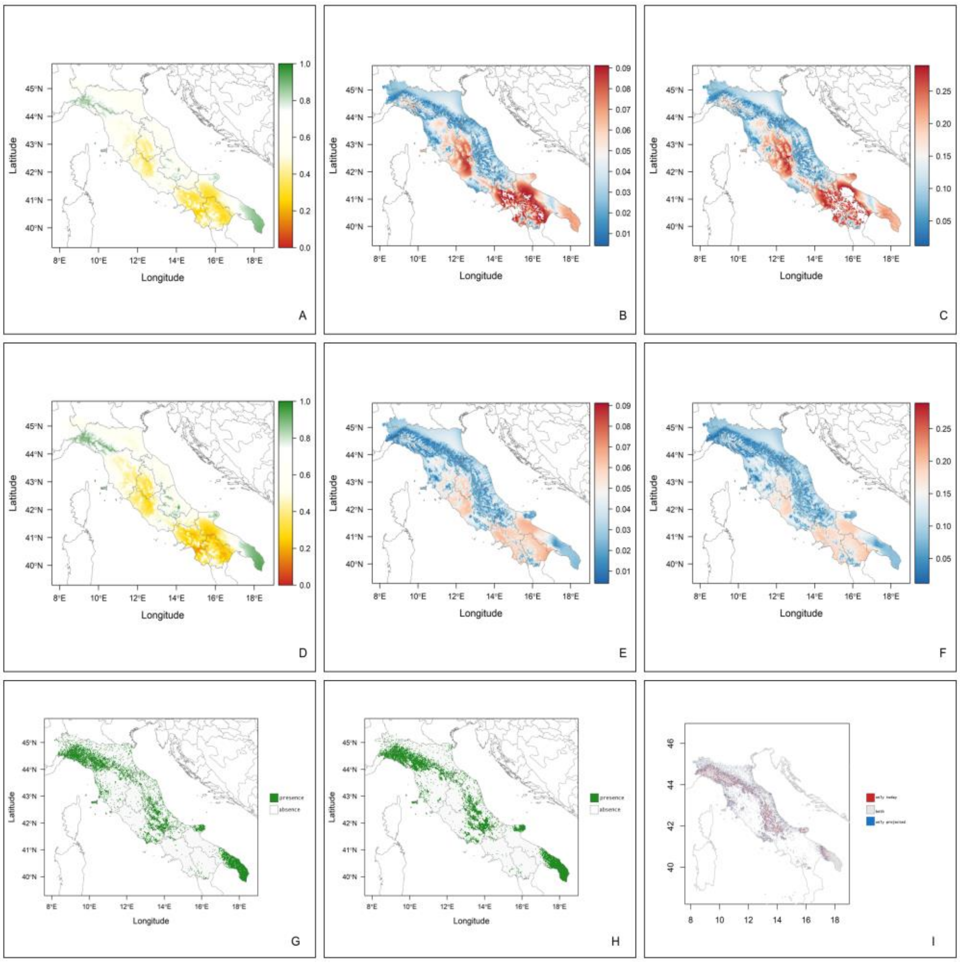

Fig. S.2.196-A) 'Projected' base output; B) 'Projected' uncertainty standard deviation; C) 'Projected' uncertainty range of values; D) 'Present' base output; E) 'Present' uncertainty standard deviation; F) 'Present' uncertainty range of values; G) 'Projected' binary distribution; H) 'Present' binary distribution; I) Range size variation.

Value	Present	Projected
AUC.train	0.704	0.666
AUC.test	0.705	0.615
AUC.diff	0.076	0.076
pROC	1.401	1.307
OR.p10	0.240	0.270
CBI	0.729	0.706
perc.stable	47.294	47.294
range.size	0.280	0.280

S.2.197 *Ornithogalum etruscum* KDE

Species: *Ornithogalum etruscum* Parl.

Procedure: KDE

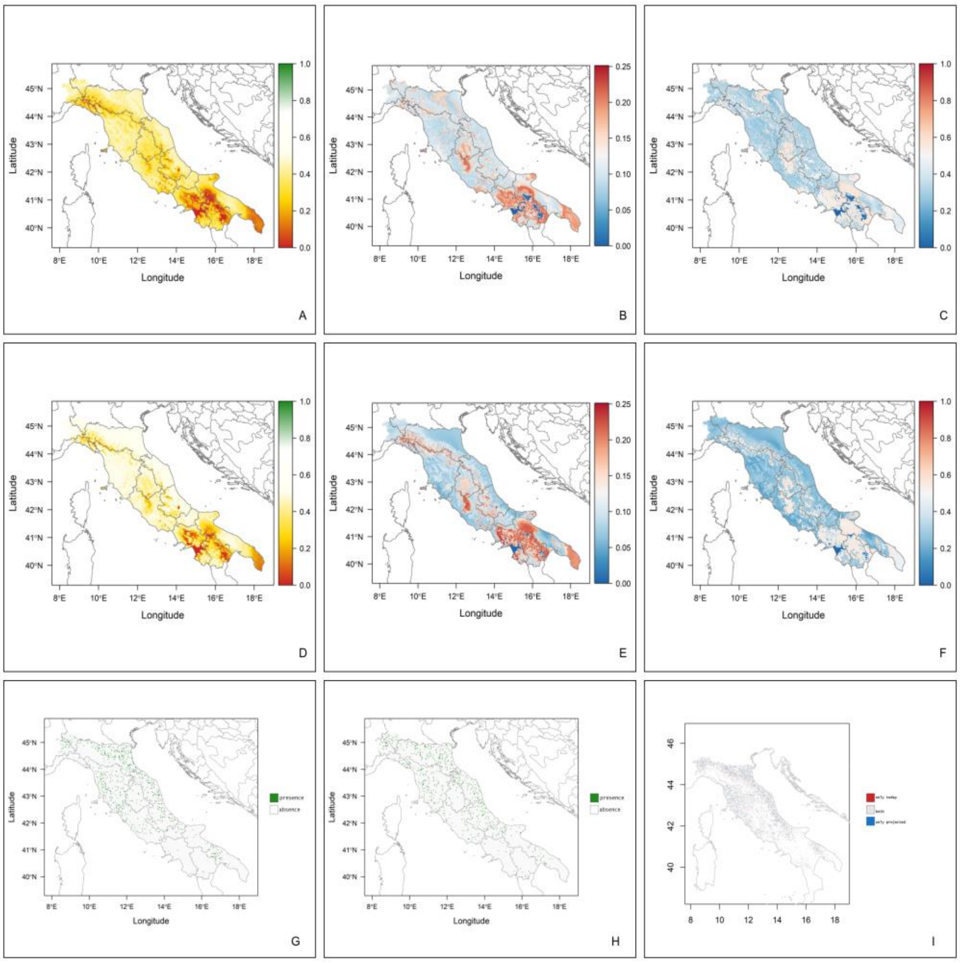

Fig. S.2.197-A) 'Projected' base output; B) 'Projected' uncertainty standard deviation; C) 'Projected' uncertainty range of values; D) 'Present' base output; E) 'Present' uncertainty standard deviation; F) 'Present' uncertainty range of values; G) 'Projected' binary distribution; H) 'Present' binary distribution; I) Range size variation.

Value	Present	Projected
AUC.train	0.631	0.639
AUC.test	0.414	0.539
AUC.diff	0.218	0.127
pROC	1.161	1.201
OR.p10	0.500	0.333
CBI	0.503	0.124
perc.stable	2.686	2.686
range.size	5.372	5.372

S.2.198 *Ornithogalum etruscum* CTA

Species: *Ornithogalum etruscum* Parl.

Procedure: CTA

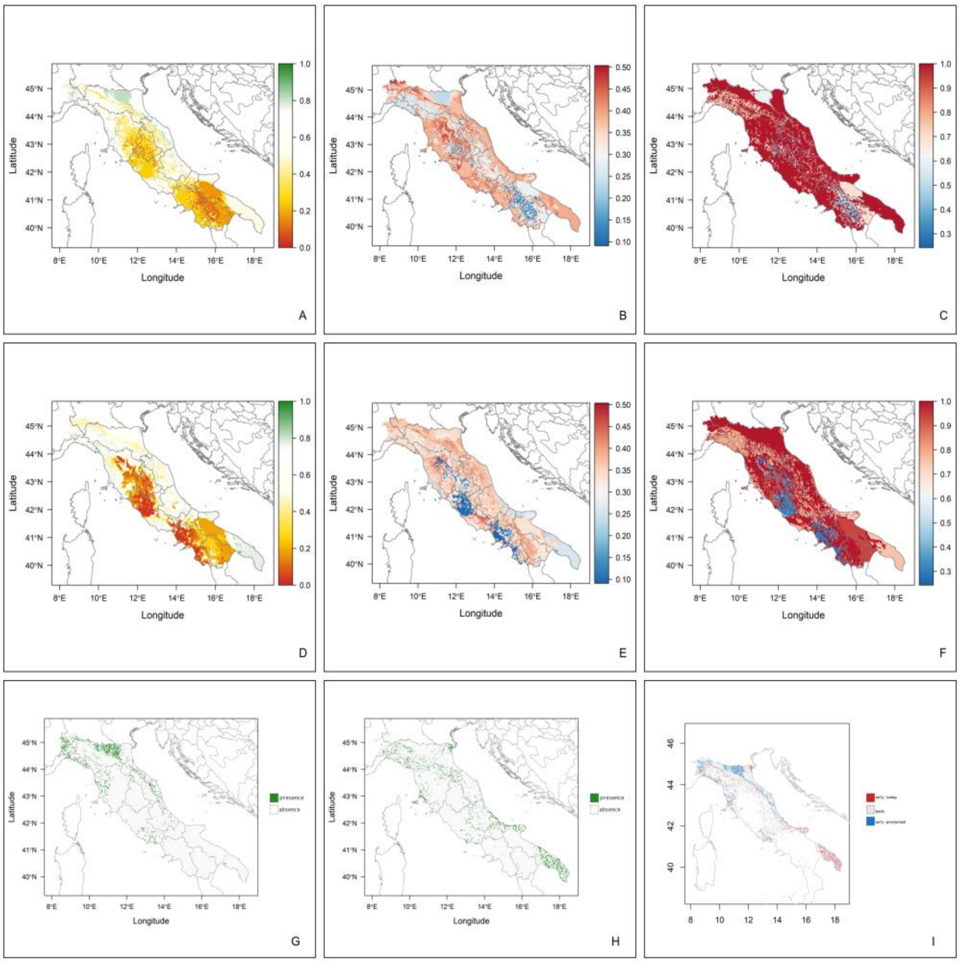

Fig. S.2.198-A) ‘Projected’ base output; B) ‘Projected’ uncertainty standard deviation; C) ‘Projected’ uncertainty range of values; D) ’Present’ base output; E) ’Present’ uncertainty standard deviation; F) ’Present’ uncertainty range of values; G) ‘Projected’ binary distribution; H) ‘Present’ binary distribution; I) Range size variation.

Value	Present	Projected
AUC.train	0.875	0.865
AUC.test	0.701	0.724
AUC.diff	0.141	0.141
pROC	1.133	1.127
OR.p10	0.380	0.307
CBI	0.894	0.070
perc.stable	2.118	2.118
range.size	-4.557	-4.557

S.2.199 *Ornithogalum etruscum* FDA

Species: *Ornithogalum etruscum* Parl.

Procedure: FDA

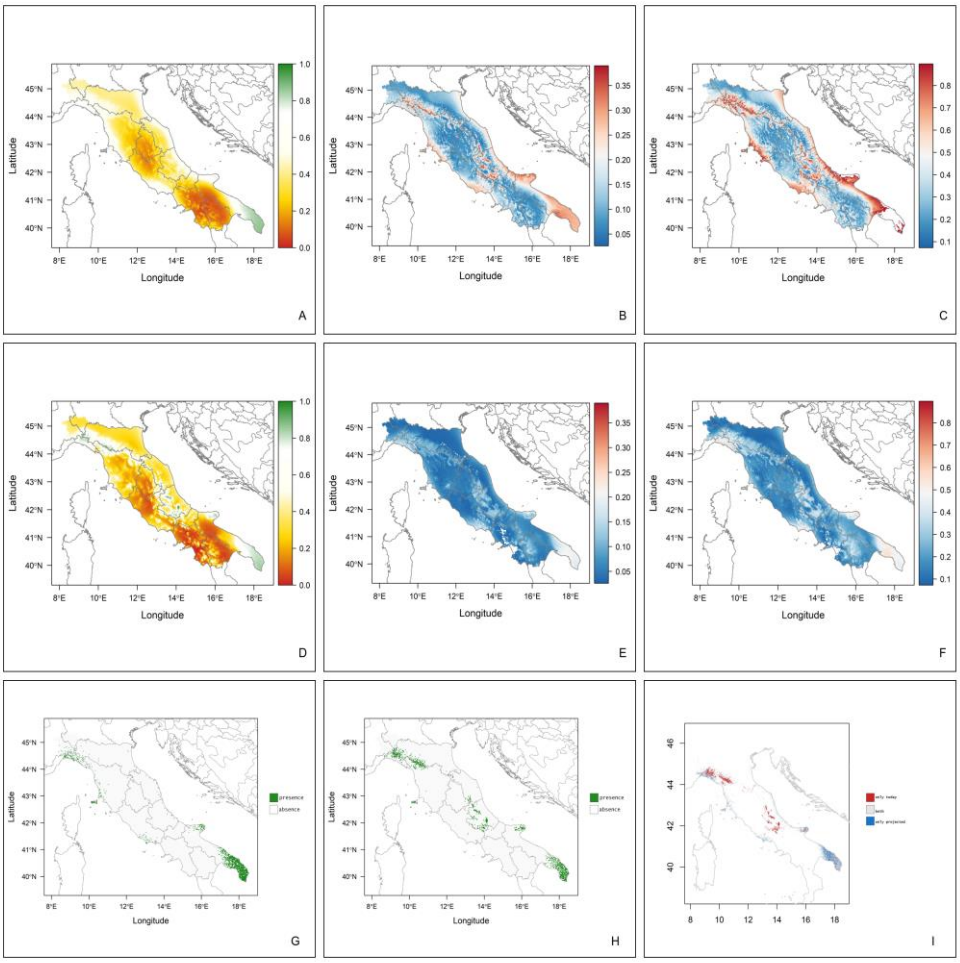

Fig. S.2.199-A) ‘Projected’ base output; B) ‘Projected’ uncertainty standard deviation; C) ‘Projected’ uncertainty range of values; D) ’Present’ base output; E) ’Present’ uncertainty standard deviation; F) ’Present’ uncertainty range of values; G) ‘Projected’ binary distribution; H) ‘Present’ binary distribution; I) Range size variation.

Value	Present	Projected
AUC.train	0.739	0.725
AUC.test	0.719	0.620
AUC.diff	0.127	0.127
pROC	1.347	1.247
OR.p10	0.240	0.350
CBI	0.660	0.407
perc.stable	22.533	22.533
range.size	-2.204	-2.204

S.2.200 *Ornithogalum etruscum* GAM

Species: *Ornithogalum etruscum* Parl.

Procedure: GAM

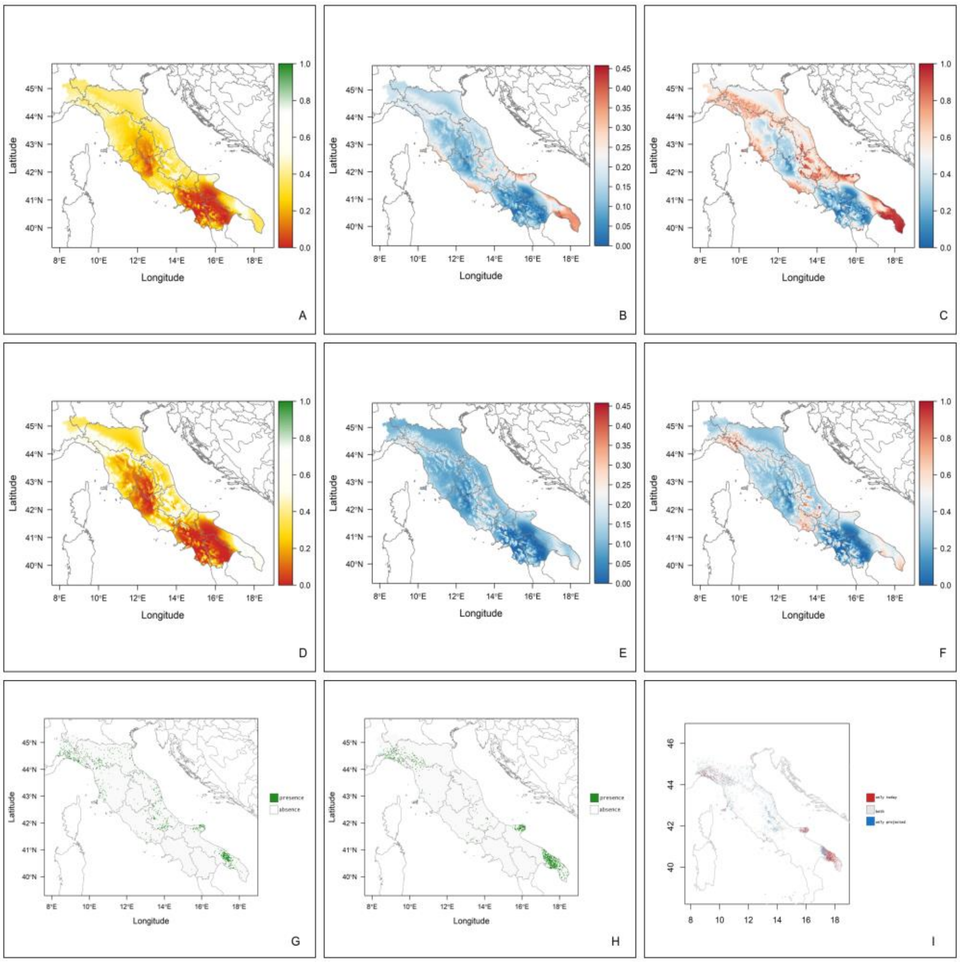

Fig. S.2.200-A) 'Projected' base output; B) 'Projected' uncertainty standard deviation; C) 'Projected' uncertainty range of values; D) 'Present' base output; E) 'Present' uncertainty standard deviation; F) 'Present' uncertainty range of values; G) 'Projected' binary distribution; H) 'Present' binary distribution; I) Range size variation.

Value	Present	Projected
AUC.train	0.755	0.752
AUC.test	0.606	0.584
AUC.diff	0.169	0.169
pROC	1.293	1.265
OR.p10	0.380	0.457
CBI	0.703	0.788
perc.stable	12.000	12.000
range.size	2.169	2.169

S.2.201 *Ornithogalum etruscum* GLM

Species: *Ornithogalum etruscum* Parl.

Procedure: GLM

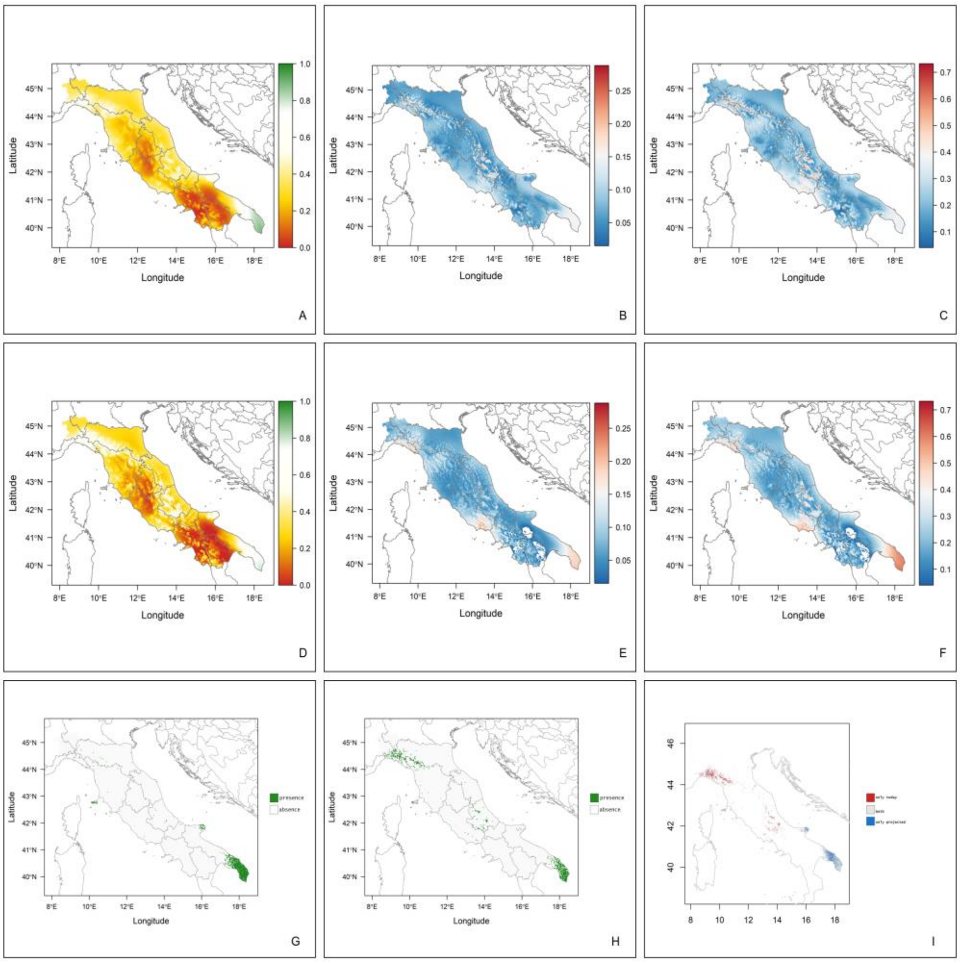

Fig. S.2.201-A) 'Projected' base output; B) 'Projected' uncertainty standard deviation; C) 'Projected' uncertainty range of values; D) 'Present' base output; E) 'Present' uncertainty standard deviation; F) 'Present' uncertainty range of values; G) 'Projected' binary distribution; H) 'Present' binary distribution; I) Range size variation.

Value	Present	Projected
AUC.train	0.709	0.694
AUC.test	0.642	0.585
AUC.diff	0.121	0.121
pROC	1.317	1.287
OR.p10	0.330	0.363
CBI	0.563	0.611
perc.stable	33.211	33.211
range.size	0.354	0.354

S.2.202 *Ornithogalum etruscum* MARS

Species: *Ornithogalum etruscum* Parl.

Procedure: MARS

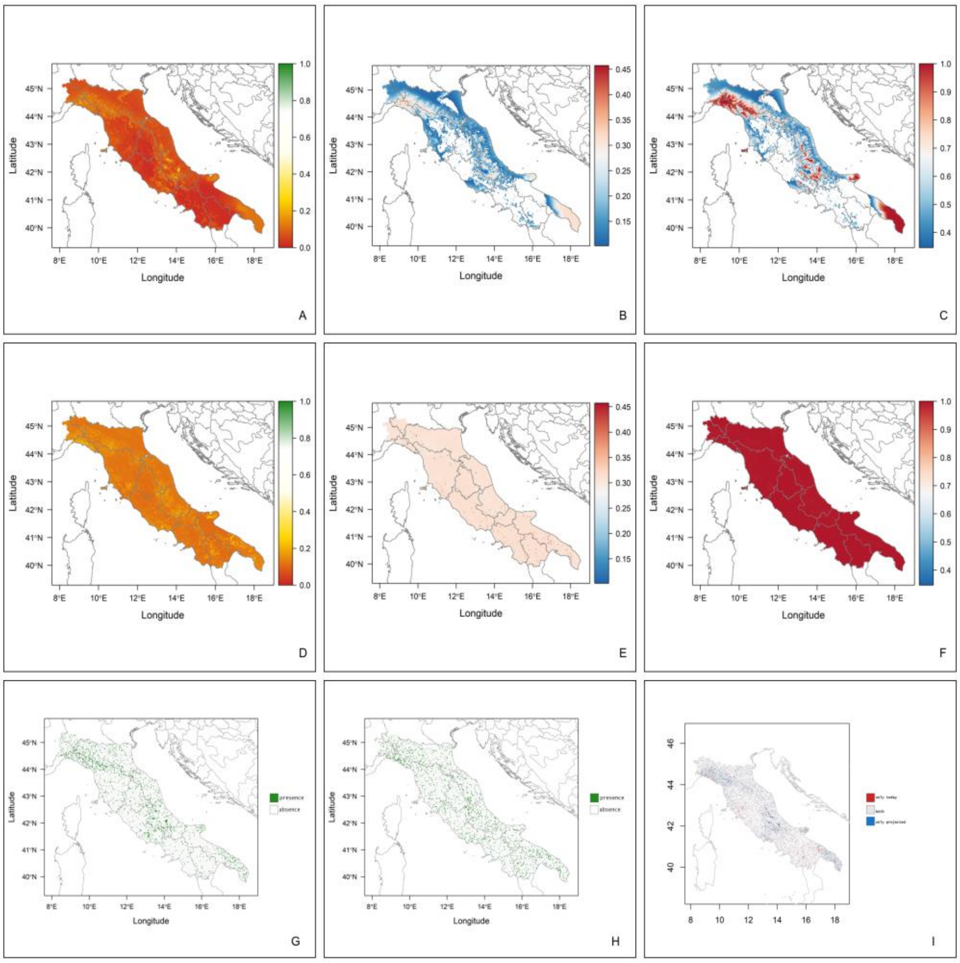

Fig. S.2.202-A) 'Projected' base output; B) 'Projected' uncertainty standard deviation; C) 'Projected' uncertainty range of values; D) 'Present' base output; E) 'Present' uncertainty standard deviation; F) 'Present' uncertainty range of values; G) 'Projected' binary distribution; H) 'Present' binary distribution; I) Range size variation.

Value	Present	Projected
AUC.train	0.728	0.690
AUC.test	0.518	0.514
AUC.diff	0.177	0.177
pROC	1.196	1.096
OR.p10	0.289	0.323
CBI	0.769	0.725
perc.stable	4.011	4.011
range.size	-0.591	-0.591

S.2.203 *Ornithogalum etruscum* RF

Species: *Ornithogalum etruscum* Parl.

Procedure: RF

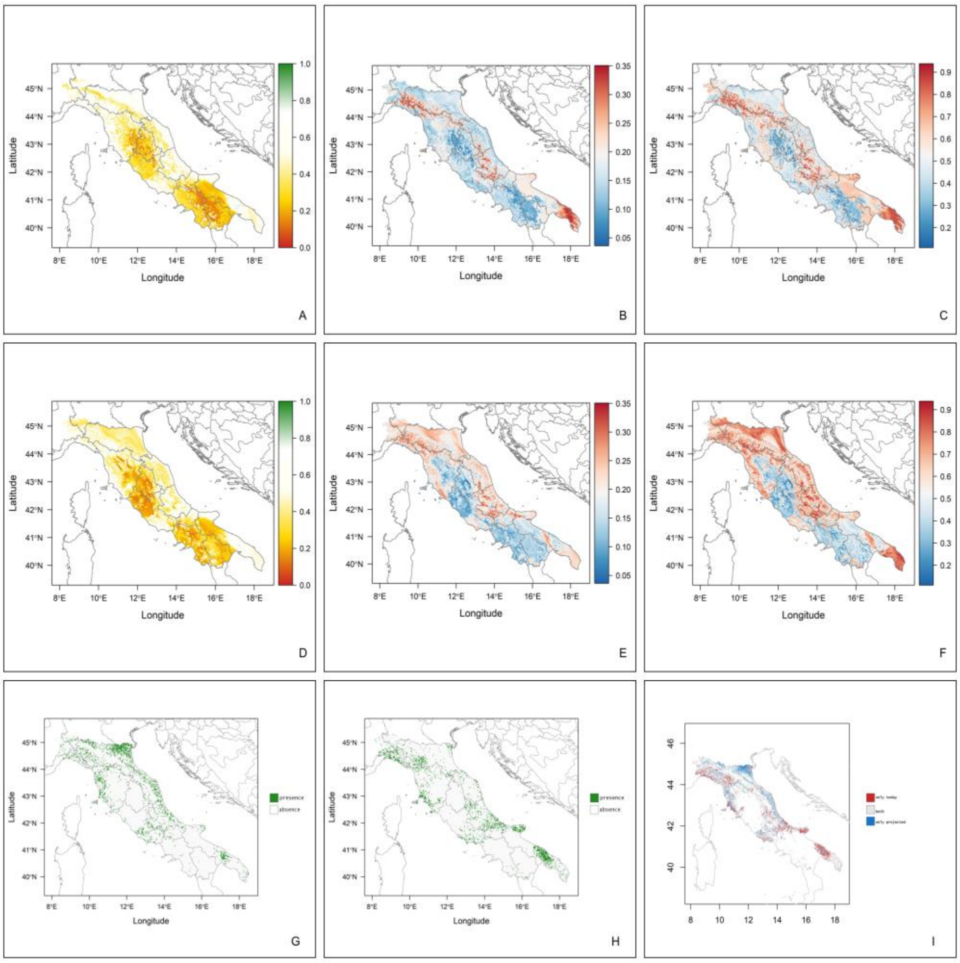

Fig. S.2.203-A) 'Projected' base output; B) 'Projected' uncertainty standard deviation; C) 'Projected' uncertainty range of values; D) 'Present' base output; E) 'Present' uncertainty standard deviation; F) 'Present' uncertainty range of values; G) 'Projected' binary distribution; H) 'Present' binary distribution; I) Range size variation.

Value	Present	Projected
AUC.train	1.000	1.000
AUC.test	0.875	0.935
AUC.diff	0.065	0.065
pROC	1.246	1.278
OR.p10	0.963	0.913
CBI	0.959	0.408
perc.stable	7.024	7.024
range.size	0.005	0.005

S.2.204 *Ornithogalum etruscum* SVM

Species: *Ornithogalum etruscum* Parl.

Procedure: SVM

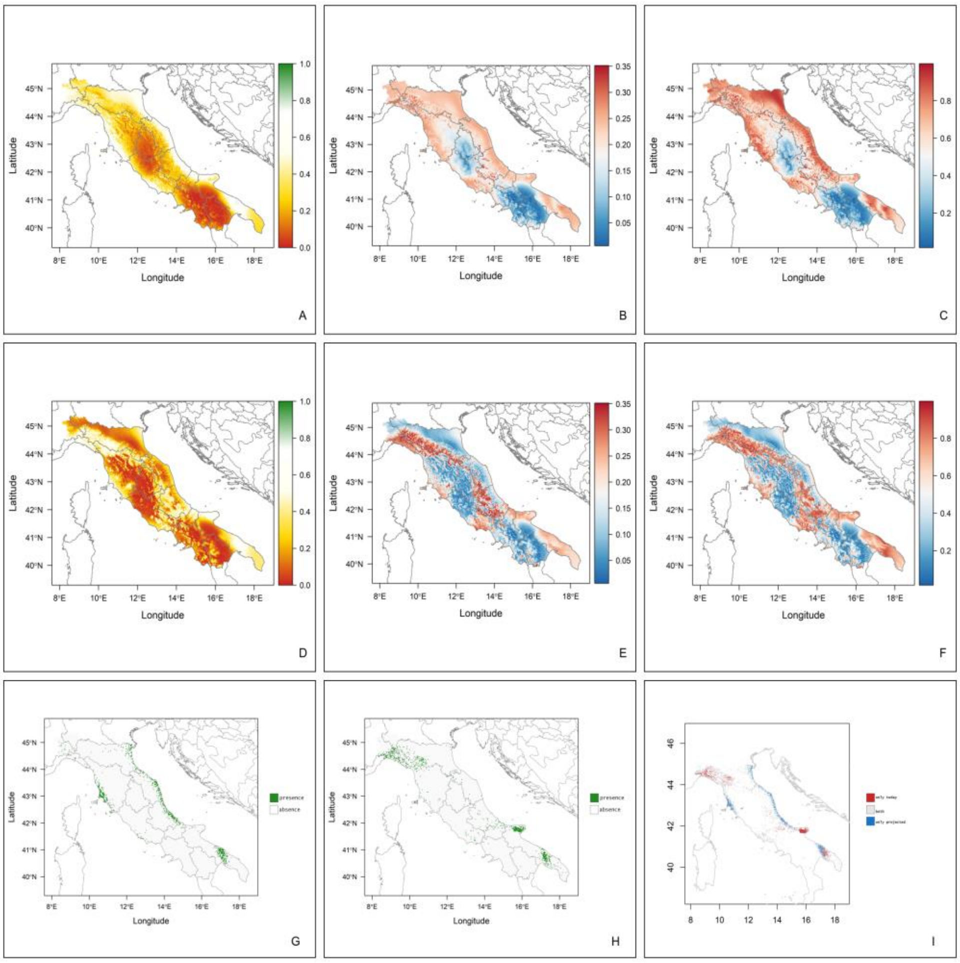

Fig. S.2.204-A) 'Projected' base output; B) 'Projected' uncertainty standard deviation; C) 'Projected' uncertainty range of values; D) 'Present' base output; E) 'Present' uncertainty standard deviation; F) 'Present' uncertainty range of values; G) 'Projected' binary distribution; H) 'Present' binary distribution; I) Range size variation.

Value	Present	Projected
AUC.train	0.882	0.835
AUC.test	0.733	0.785
AUC.diff	0.072	0.072
pROC	1.255	1.294
OR.p10	0.390	0.207
CBI	0.908	0.588
perc.stable	5.394	5.394
range.size	-1.422	-1.422

S.2.205 *Phyteuma ovatum* Biomod2

Species: *Phyteuma ovatum* Honck. subsp. *pseudospicatum* Pignatti

Procedure: Biomod2

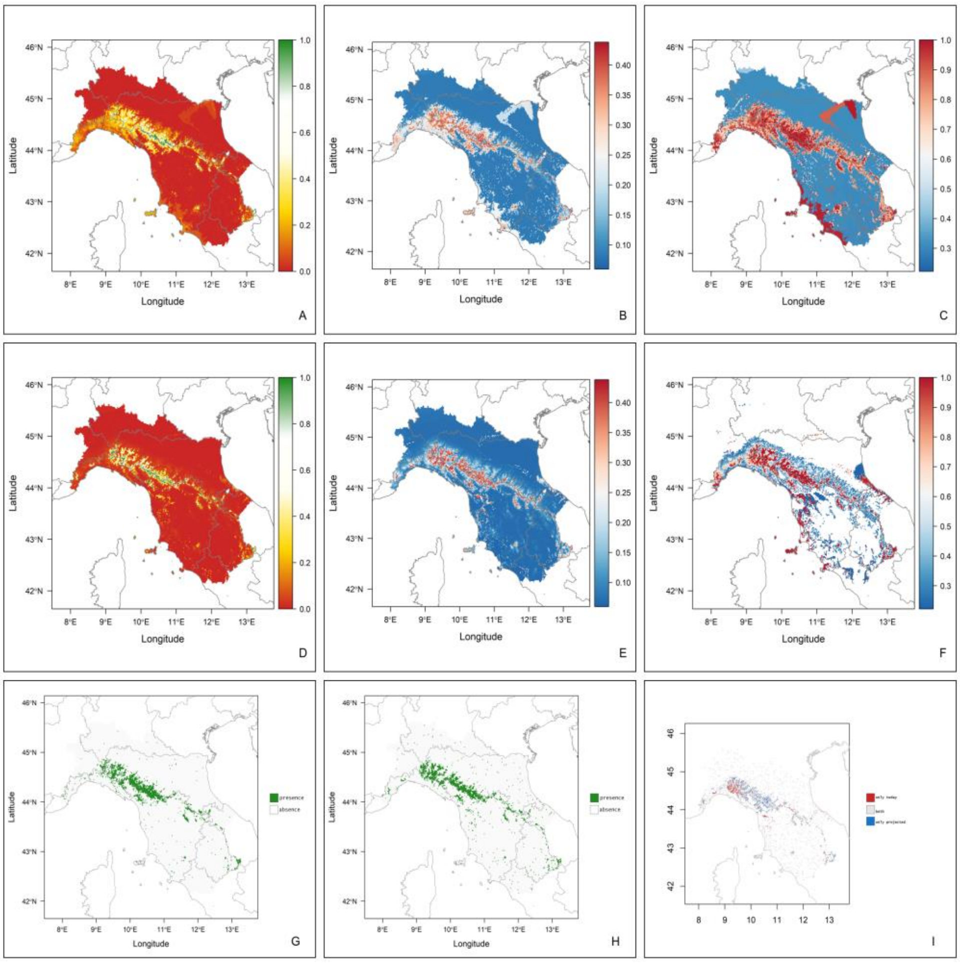

Fig. S.2.205-A) ‘Projected’ base output; B) ‘Projected’ uncertainty standard deviation; C) ‘Projected’ uncertainty range of values; D) ’Present’ base output; E) ’Present’ uncertainty standard deviation; F) ’Present’ uncertainty range of values; G) ‘Projected’ binary distribution; H) ‘Present’ binary distribution; I) Range size variation.

Value	Present	Projected
AUC.train	0.996	0.995
AUC.test	0.975	0.804
AUC.diff	0.191	0.191
pROC	1.870	1.348
OR.p10	0.511	0.711
CBI	0.614	0.871
perc.stable	47.203	47.203
range.size	-1.666	-1.666

S.2.206 *Phyteuma ovatum* Bioclim

Species: *Phyteuma ovatum* Honck. subsp. *pseudospicatum* Pignatti

Procedure: Bioclim

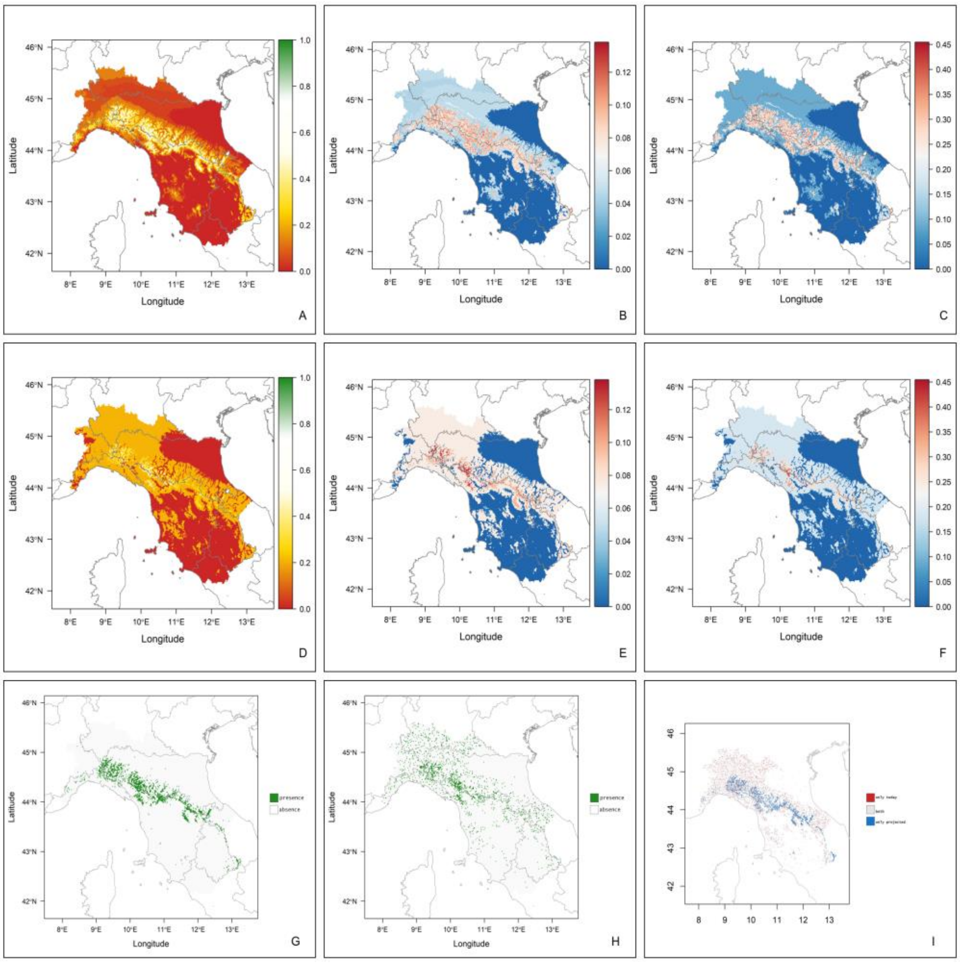

Fig. S.2.206-A) 'Projected' base output; B) 'Projected' uncertainty standard deviation; C) 'Projected' uncertainty range of values; D) 'Present' base output; E) 'Present' uncertainty standard deviation; F) 'Present' uncertainty range of values; G) 'Projected' binary distribution; H) 'Present' binary distribution; I) Range size variation.

Value	Present	Projected
AUC.train	0.828	0.799
AUC.test	0.732	0.747
AUC.diff	0.065	0.065
pROC	1.200	1.170
OR.p10	0.200	0.156
CBI	0.769	0.679
perc.stable	18.442	18.442
range.size	2.110	2.110

S.2.207 *Phyteuma ovatum* Domain

Species: *Phyteuma ovatum* Honck. subsp. *pseudospicatum* Pignatti

Procedure: Domain

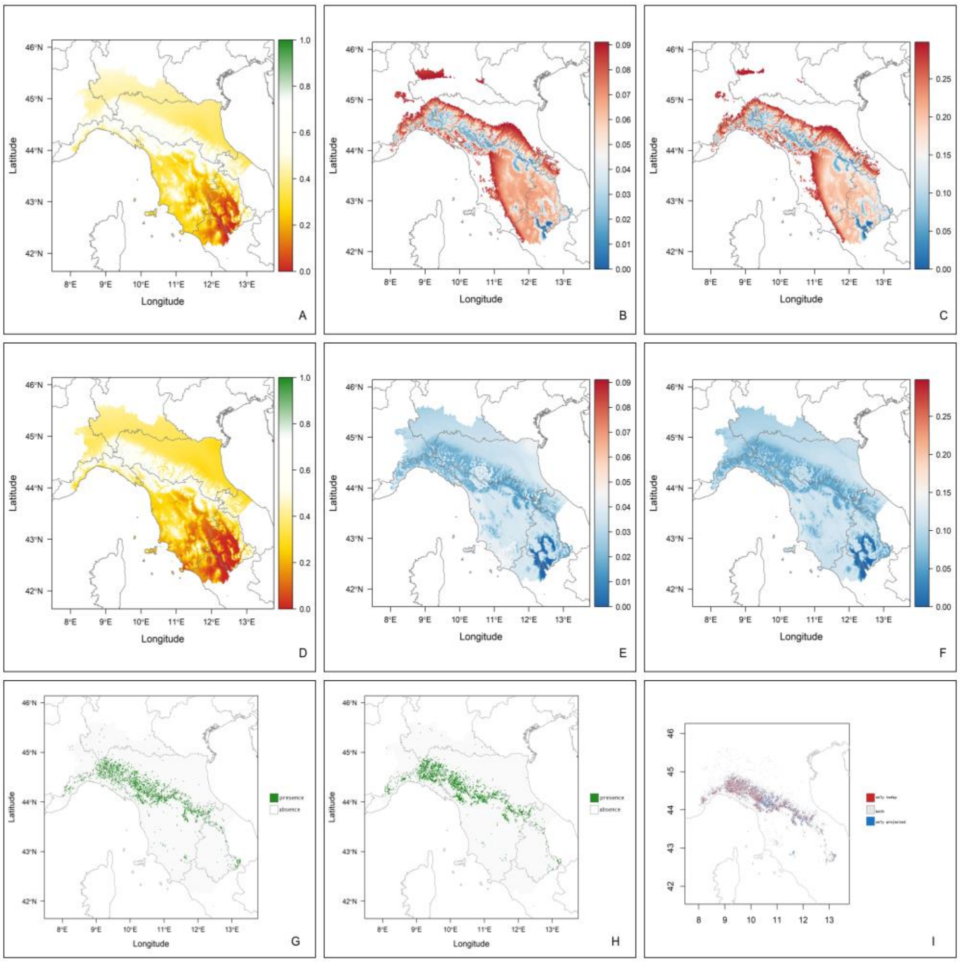

Fig. S.2.207-A) 'Projected' base output; B) 'Projected' uncertainty standard deviation; C) 'Projected' uncertainty range of values; D) 'Present' base output; E) 'Present' uncertainty standard deviation; F) 'Present' uncertainty range of values; G) 'Projected' binary distribution; H) 'Present' binary distribution; I) Range size variation.

Value	Present	Projected
AUC.train	0.897	0.809
AUC.test	0.899	0.795
AUC.diff	0.046	0.046
pROC	1.617	1.407
OR.p10	0.156	0.189
CBI	0.818	0.808
perc.stable	25.545	25.545
range.size	-0.028	-0.028

S.2.208 *Phyteuma ovatum* Maxent

Species: *Phyteuma ovatum* Honck. subsp. *pseudospicatum* Pignatti

Procedure: Maxent

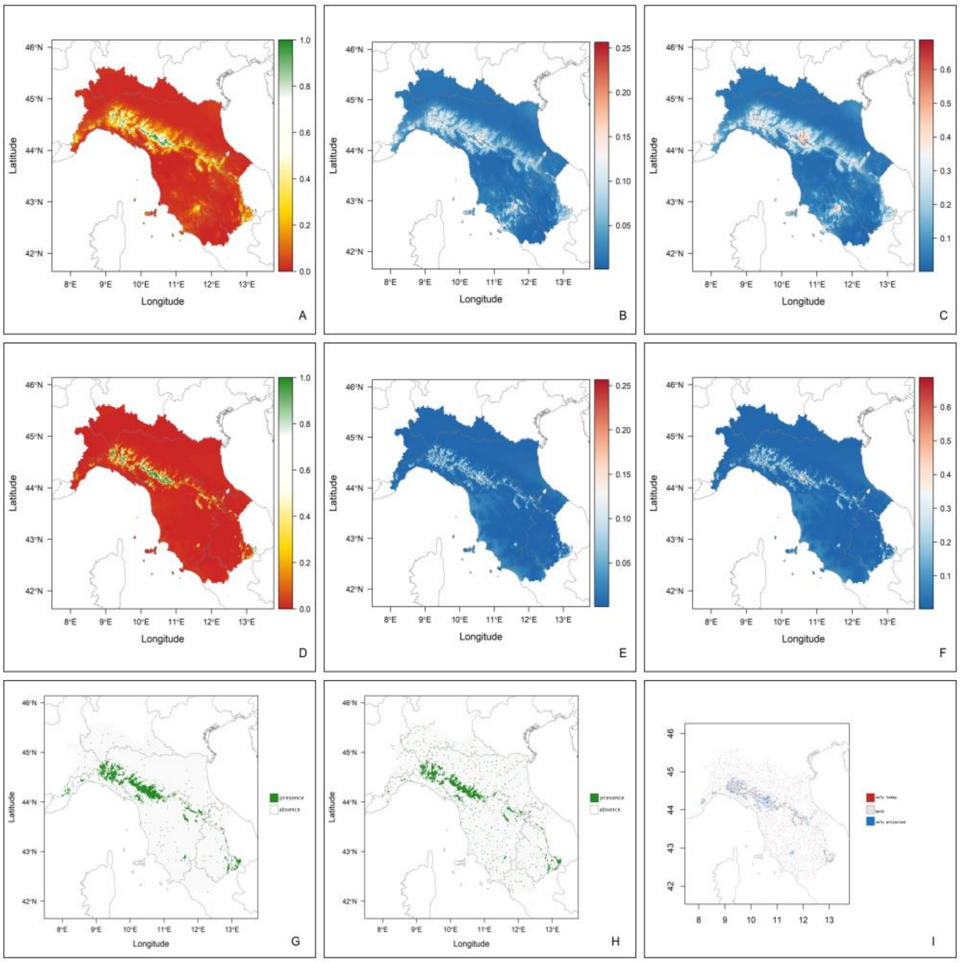

Fig. S.2.208-A) 'Projected' base output; B) 'Projected' uncertainty standard deviation; C) 'Projected' uncertainty range of values; D) 'Present' base output; E) 'Present' uncertainty standard deviation; F) 'Present' uncertainty range of values; G) 'Projected' binary distribution; H) 'Present' binary distribution; I) Range size variation.

Value	Present	Projected
AUC.train	0.964	0.914
AUC.test	0.911	0.865
AUC.diff	0.067	0.067
pROC	1.630	1.505
OR.p10	0.244	0.256
CBI	0.909	0.951
perc.stable	46.654	46.654
range.size	-3.005	-3.005

S.2.209 *Phyteuma ovatum* KDE

Species: *Phyteuma ovatum* Honck. subsp. *pseudospicatum* Pignatti

Procedure: KDE

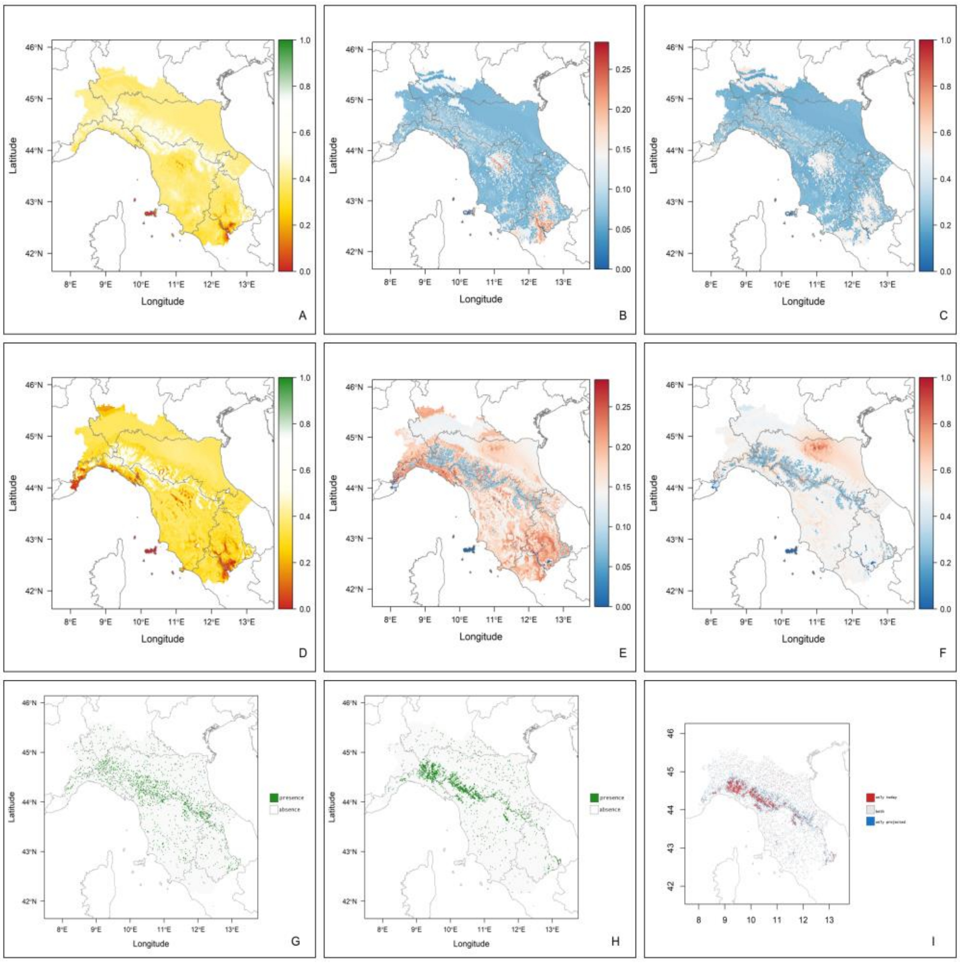

Fig. S.2.209-A) 'Projected' base output; B) 'Projected' uncertainty standard deviation; C) 'Projected' uncertainty range of values; D) 'Present' base output; E) 'Present' uncertainty standard deviation; F) 'Present' uncertainty range of values; G) 'Projected' binary distribution; H) 'Present' binary distribution; I) Range size variation.

Value	Present	Projected
AUC.train	0.941	0.812
AUC.test	0.896	0.771
AUC.diff	0.054	0.055
pROC	1.591	1.362
OR.p10	0.222	0.189
CBI	0.988	0.989
perc.stable	7.549	7.549
range.size	2.594	2.594

S.2.210 *Phyteuma ovatum* CTA

Species: *Phyteuma ovatum* Honck. subsp. *pseudospicatum* Pignatti

Procedure: CTA

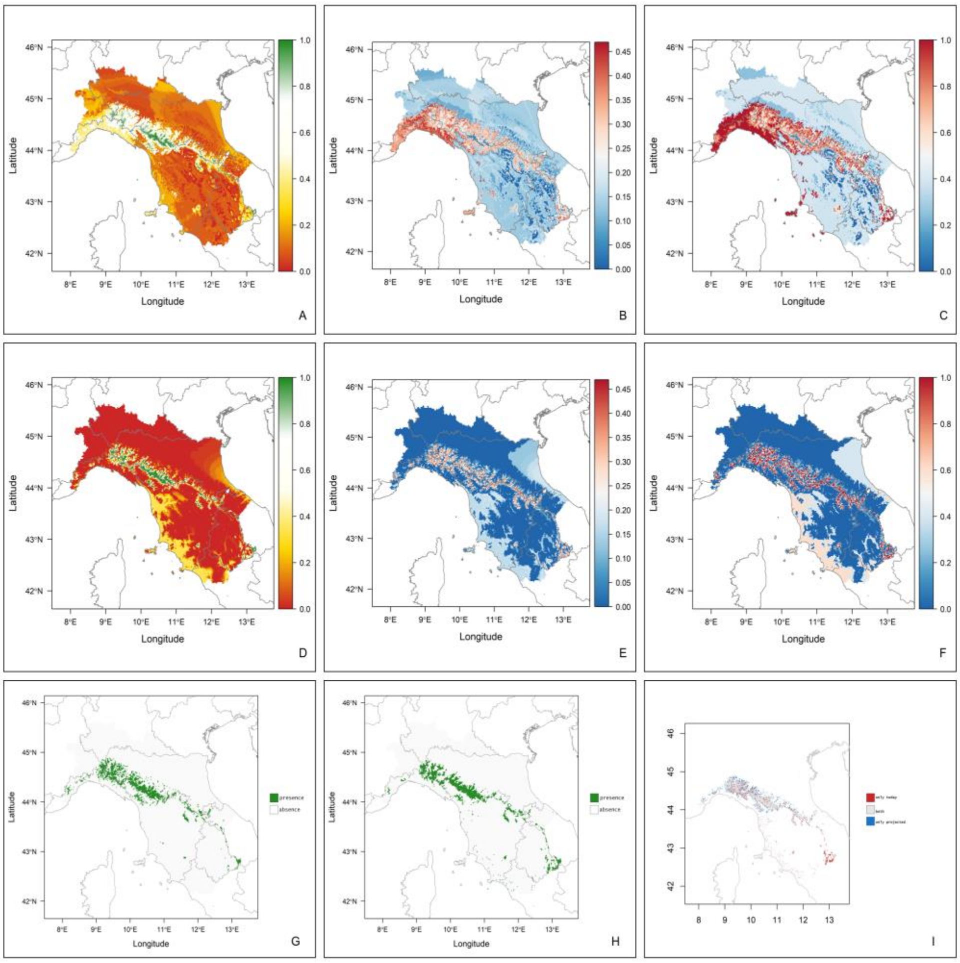

Fig. S.2.210-A) 'Projected' base output; B) 'Projected' uncertainty standard deviation; C) 'Projected' uncertainty range of values; D) 'Present' base output; E) 'Present' uncertainty standard deviation; F) 'Present' uncertainty range of values; G) 'Projected' binary distribution; H) 'Present' binary distribution; I) Range size variation.

Value	Present	Projected
AUC.train	0.983	0.952
AUC.test	0.928	0.881
AUC.diff	0.072	0.072
pROC	1.298	1.249
OR.p10	0.211	0.233
CBI	0.639	0.940
perc.stable	55.336	55.336
range.size	-0.693	-0.693

S.2.211 *Phyteuma ovatum* FDA

Species: *Phyteuma ovatum* Honck. subsp. *pseudospicatum* Pignatti

Procedure: FDA

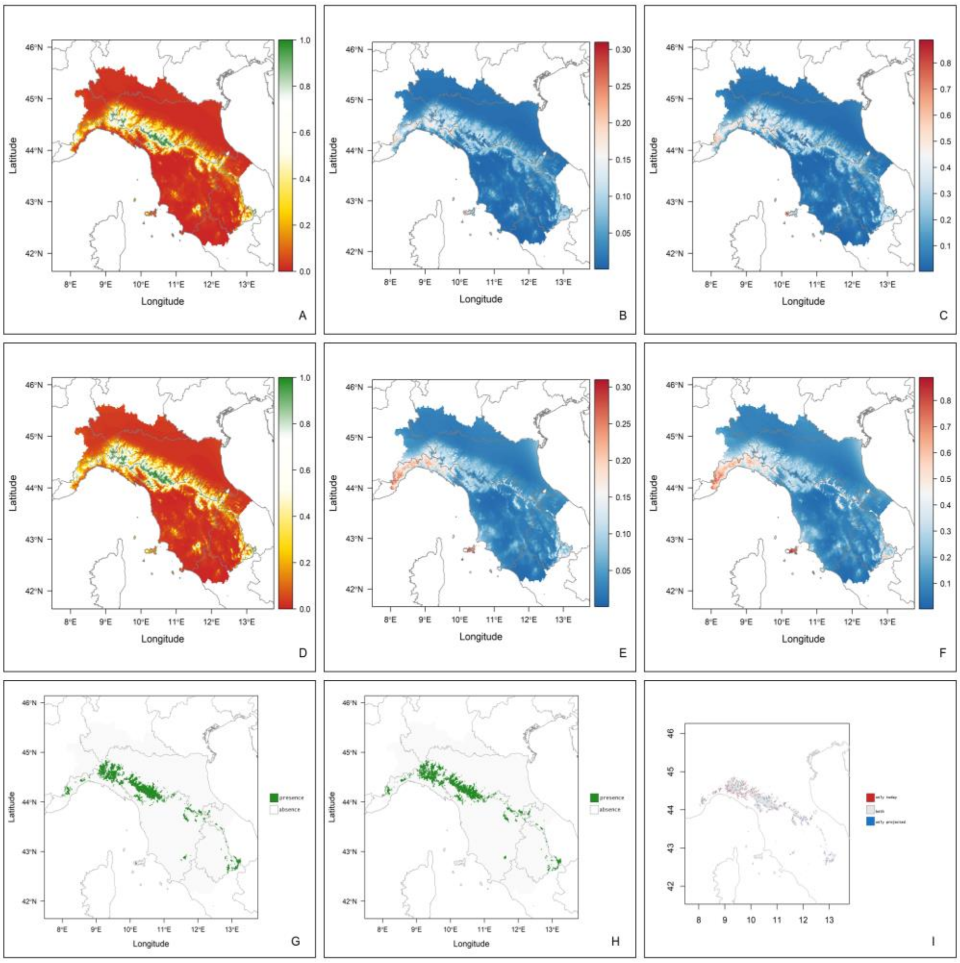

Fig. S.2.211-A) 'Projected' base output; B) 'Projected' uncertainty standard deviation; C) 'Projected' uncertainty range of values; D) 'Present' base output; E) 'Present' uncertainty standard deviation; F) 'Present' uncertainty range of values; G) 'Projected' binary distribution; H) 'Present' binary distribution; I) Range size variation.

Value	Present	Projected
AUC.train	0.891	0.918
AUC.test	0.921	0.865
AUC.diff	0.066	0.066
pROC	1.689	1.507
OR.p10	0.133	0.200
CBI	0.826	0.874
perc.stable	70.471	70.471
range.size	1.007	1.007

S.2.212 *Phyteuma ovatum* GAM

Species: *Phyteuma ovatum* Honck. subsp. *pseudospicatum* Pignatti

Procedure: GAM

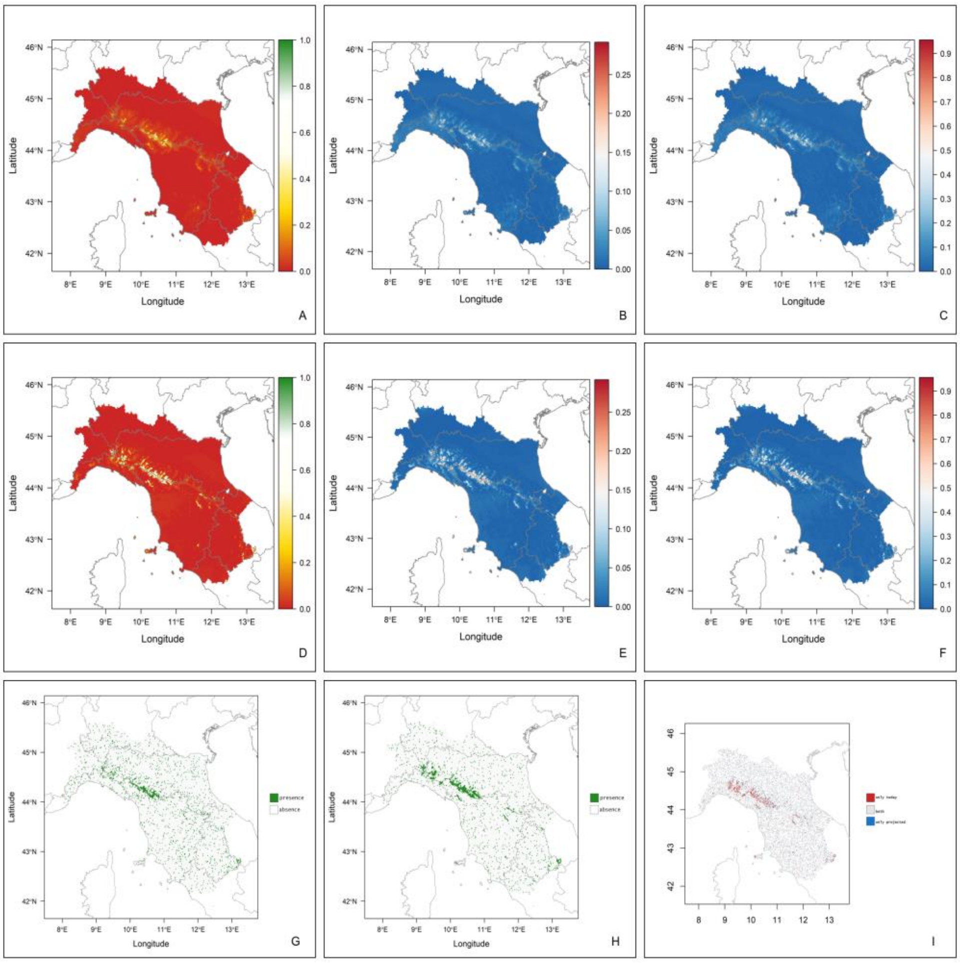

Fig. S.2.212-A) ‘Projected’ base output; B) ‘Projected’ uncertainty standard deviation; C) ‘Projected’ uncertainty range of values; D) ’Present’ base output; E) ’Present’ uncertainty standard deviation; F) ’Present’ uncertainty range of values; G) ‘Projected’ binary distribution; H) ‘Present’ binary distribution; I) Range size variation.

Value	Present	Projected
AUC.train	0.959	0.908
AUC.test	0.938	0.857
AUC.diff	0.083	0.083
pROC	1.712	1.512
OR.p10	0.156	0.233
CBI	0.852	0.572
perc.stable	12.295	12.295
range.size	2.069	2.069

S.2.213 *Phyteuma ovatum* GLM

Species: *Phyteuma ovatum* Honck. subsp. *pseudospicatum* Pignatti

Procedure: GLM

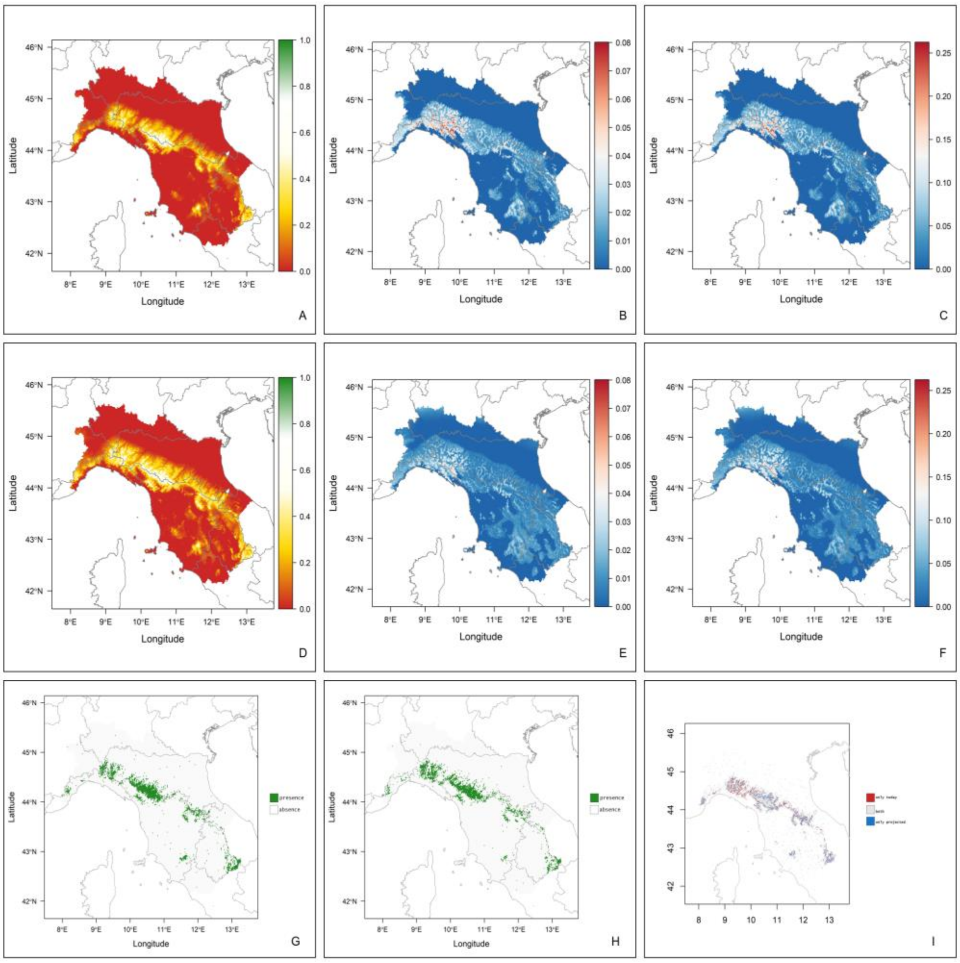

Fig. S.2.213-A) 'Projected' base output; B) 'Projected' uncertainty standard deviation; C) 'Projected' uncertainty range of values; D) 'Present' base output; E) 'Present' uncertainty standard deviation; F) 'Present' uncertainty range of values; G) 'Projected' binary distribution; H) 'Present' binary distribution; I) Range size variation.

Value	Present	Projected
AUC.train	0.913	0.901
AUC.test	0.914	0.867
AUC.diff	0.074	0.074
pROC	1.590	1.503
OR.p10	0.167	0.256
CBI	0.838	0.837
perc.stable	43.330	43.330
range.size	-0.392	-0.392

S.2.214 *Phyteuma ovatum* MARS

Species: *Phyteuma ovatum* Honck. subsp. *pseudospicatum* Pignatti

Procedure: MARS

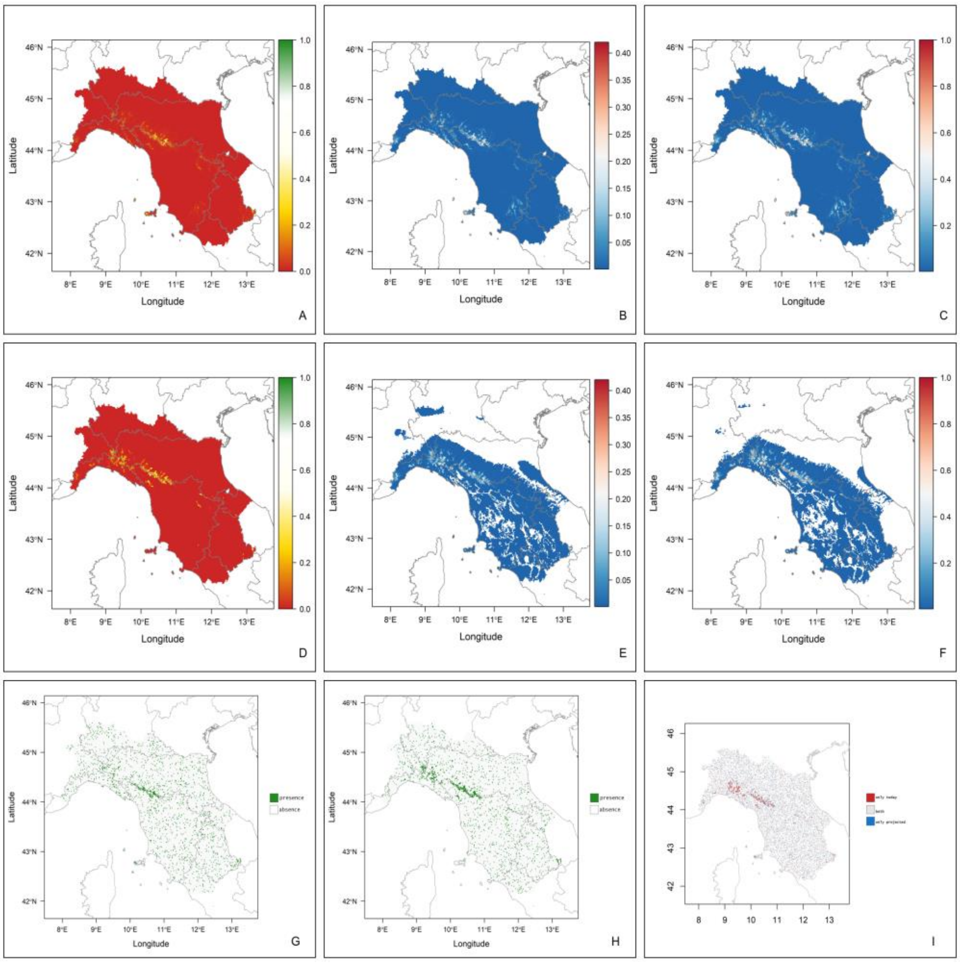

Fig. S.2.214-A) 'Projected' base output; B) 'Projected' uncertainty standard deviation; C) 'Projected' uncertainty range of values; D) 'Present' base output; E) 'Present' uncertainty standard deviation; F) 'Present' uncertainty range of values; G) 'Projected' binary distribution; H) 'Present' binary distribution; I) Range size variation.

Value	Present	Projected
AUC.train	0.897	0.904
AUC.test	0.883	0.758
AUC.diff	0.153	0.153
pROC	1.524	1.238
OR.p10	0.156	0.333
CBI	0.800	0.552
perc.stable	6.767	6.767
range.size	0.148	0.148

S.2.215 *Phyteuma ovatum* RF

Species: *Phyteuma ovatum* Honck. subsp. *pseudospicatum* Pignatti

Procedure: RF

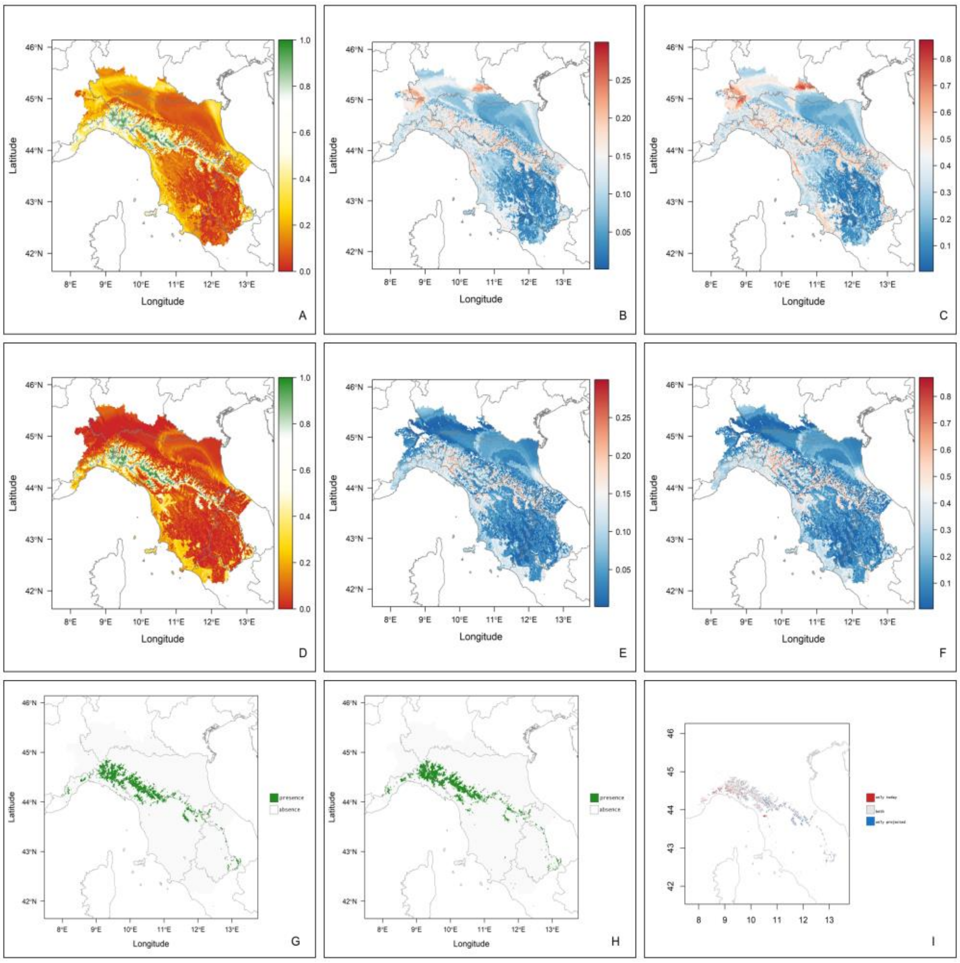

Fig. S.2.215-A) ‘Projected’ base output; B) ‘Projected’ uncertainty standard deviation; C) ‘Projected’ uncertainty range of values; D) ’Present’ base output; E) ’Present’ uncertainty standard deviation; F) ’Present’ uncertainty range of values; G) ‘Projected’ binary distribution; H) ‘Present’ binary distribution; I) Range size variation.

Value	Present	Projected
AUC.train	1.000	1.000
AUC.test	0.994	0.960
AUC.diff	0.040	0.040
pROC	1.845	1.591
OR.p10	0.267	0.322
CBI	0.971	0.897
perc.stable	67.746	67.746
range.size	0.233	0.233

S.2.216 *Phyteuma ovatum* SVM

Species: *Phyteuma ovatum* Honck. subsp. *pseudospicatum* Pignatti

Procedure: SVM

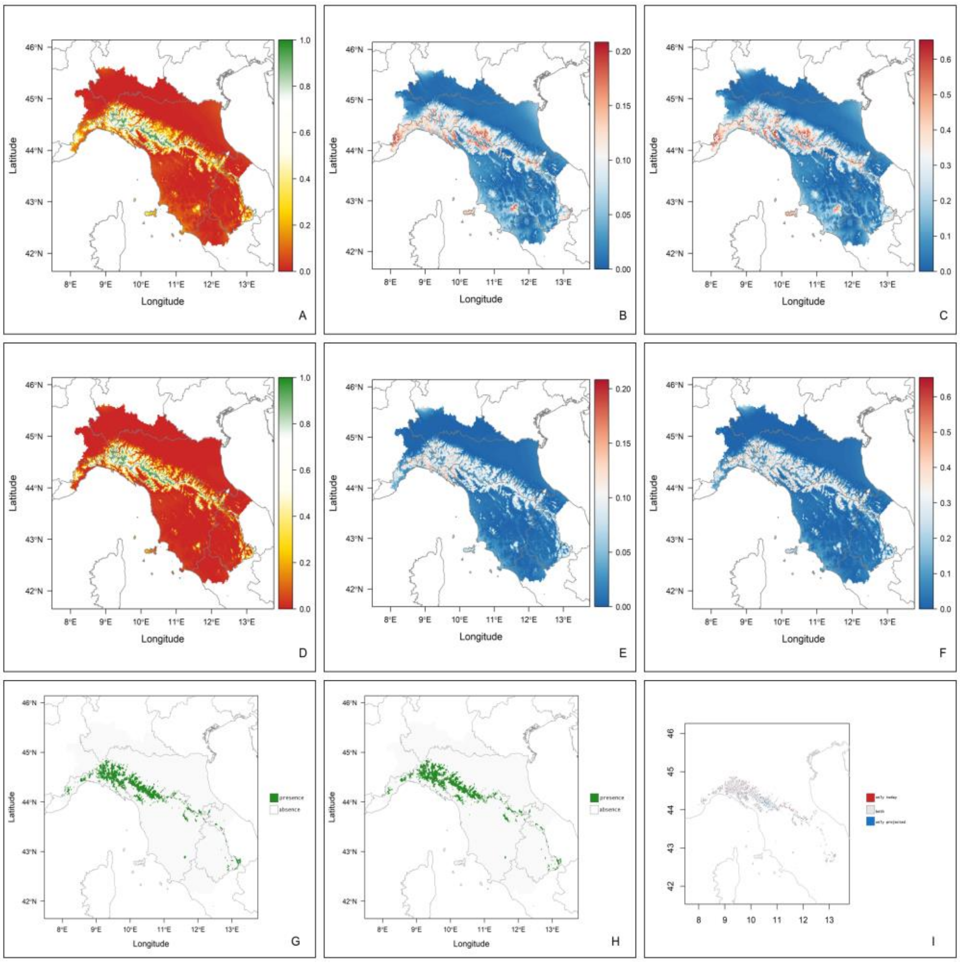

Fig. S.2.216-A) ‘Projected’ base output; B) ‘Projected’ uncertainty standard deviation; C) ‘Projected’ uncertainty range of values; D) ’Present’ base output; E) ’Present’ uncertainty standard deviation; F) ’Present’ uncertainty range of values; G) ‘Projected’ binary distribution; H) ‘Present’ binary distribution; I) Range size variation.

Value	Present	Projected
AUC.train	0.971	0.921
AUC.test	0.975	0.894
AUC.diff	0.045	0.045
pROC	1.791	1.541
OR.p10	0.156	0.256
CBI	0.739	0.839
perc.stable	74.177	74.177
range.size	2.117	2.117

S.2.217 *Polygala flavescens* Biomod2

Species: *Polygala flavescens* DC. subsp. *flavescens*

Procedure: Biomod2

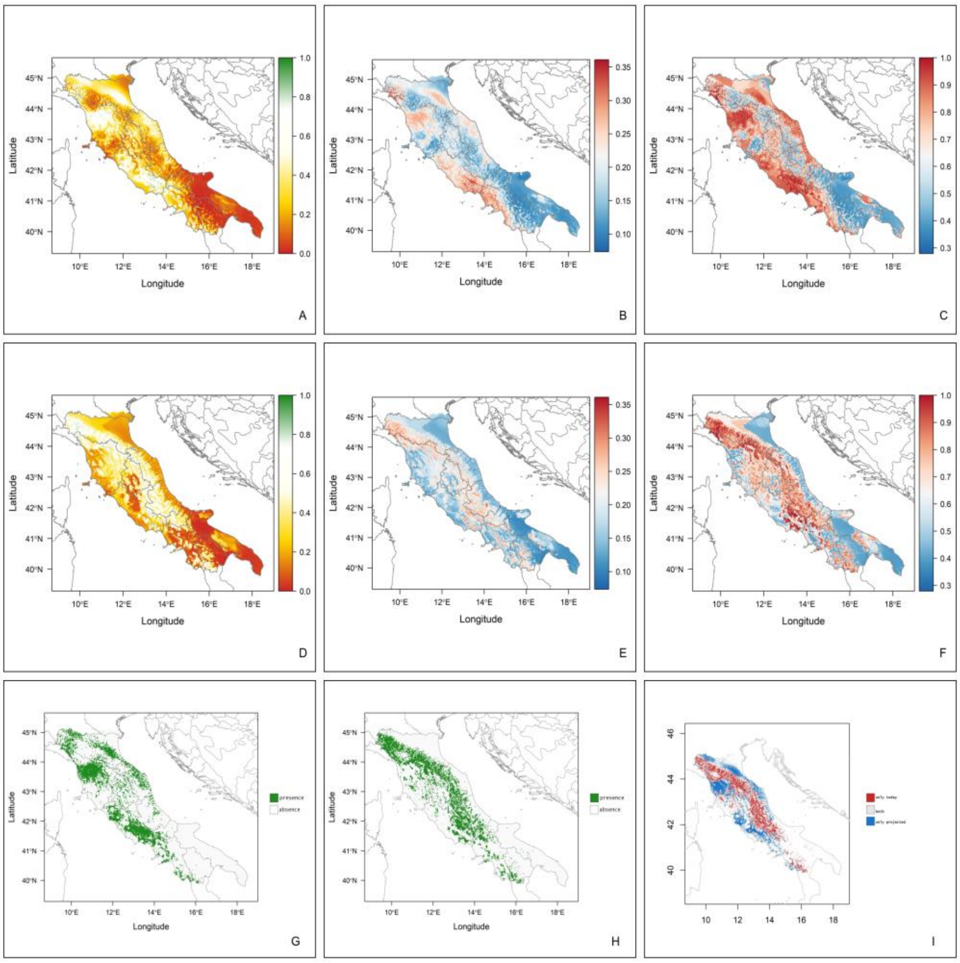

Fig. S.2.217-A) 'Projected' base output; B) 'Projected' uncertainty standard deviation; C) 'Projected' uncertainty range of values; D) 'Present' base output; E) 'Present' uncertainty standard deviation; F) 'Present' uncertainty range of values; G) 'Projected' binary distribution; H) 'Present' binary distribution; I) Range size variation.

Value	Present	Projected
AUC.train	0.921	0.934
AUC.test	0.734	0.772
AUC.diff	0.163	0.163
pROC	1.170	1.208
OR.p10	0.434	0.391
CBI	0.995	0.786
perc.stable	19.567	19.567
range.size	-0.997	-0.997

S.2.218 *Polygala flavescens* Bioclim

Species: *Polygala flavescens* DC. subsp. *flavescens*

Procedure: Bioclim

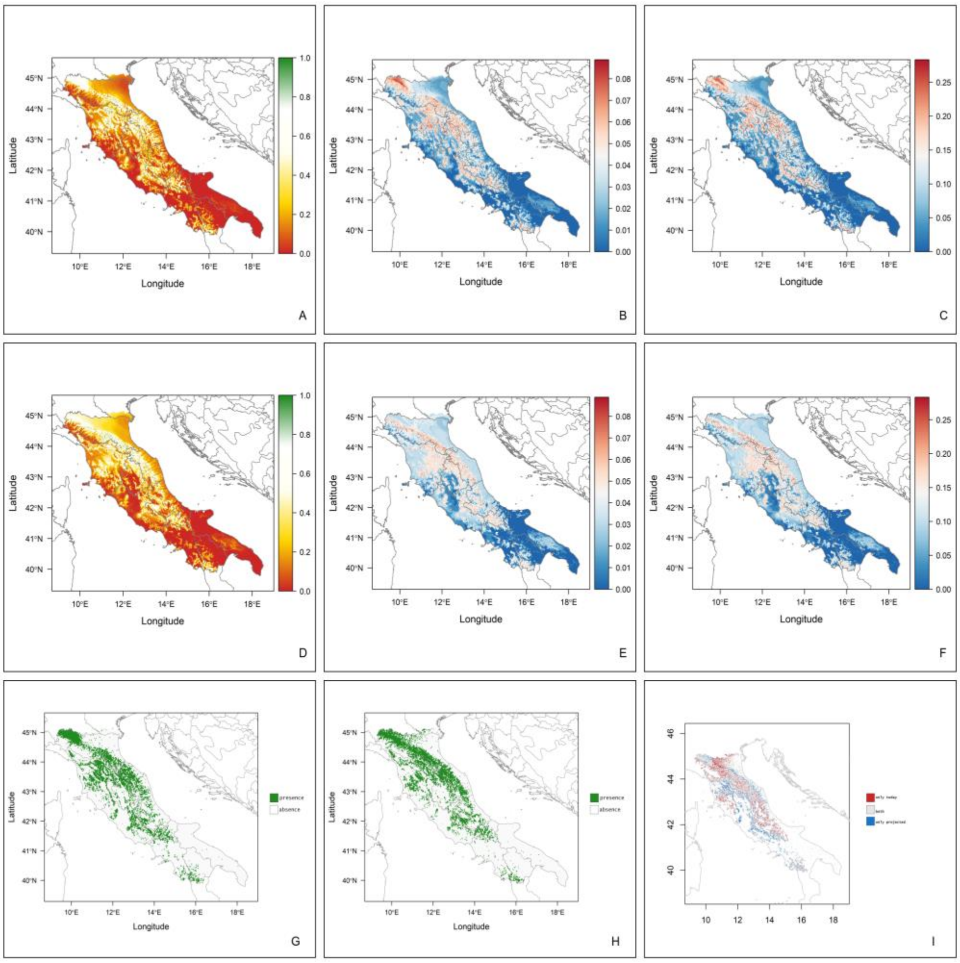

Fig. S.2.218-A) 'Projected' base output; B) 'Projected' uncertainty standard deviation; C) 'Projected' uncertainty range of values; D) 'Present' base output; E) 'Present' uncertainty standard deviation; F) 'Present' uncertainty range of values; G) 'Projected' binary distribution; H) 'Present' binary distribution; I) Range size variation.

Value	Present	Projected
AUC.train	0.659	0.647
AUC.test	0.617	0.618
AUC.diff	0.035	0.035
pROC	1.098	1.103
OR.p10	0.168	0.126
CBI	0.275	0.532
perc.stable	57.027	57.027
range.size	-0.316	-0.316

S.2.219 *Polygala flavescens* Domain

Species: *Polygala flavescens* DC. subsp. *flavescens*

Procedure: Domain

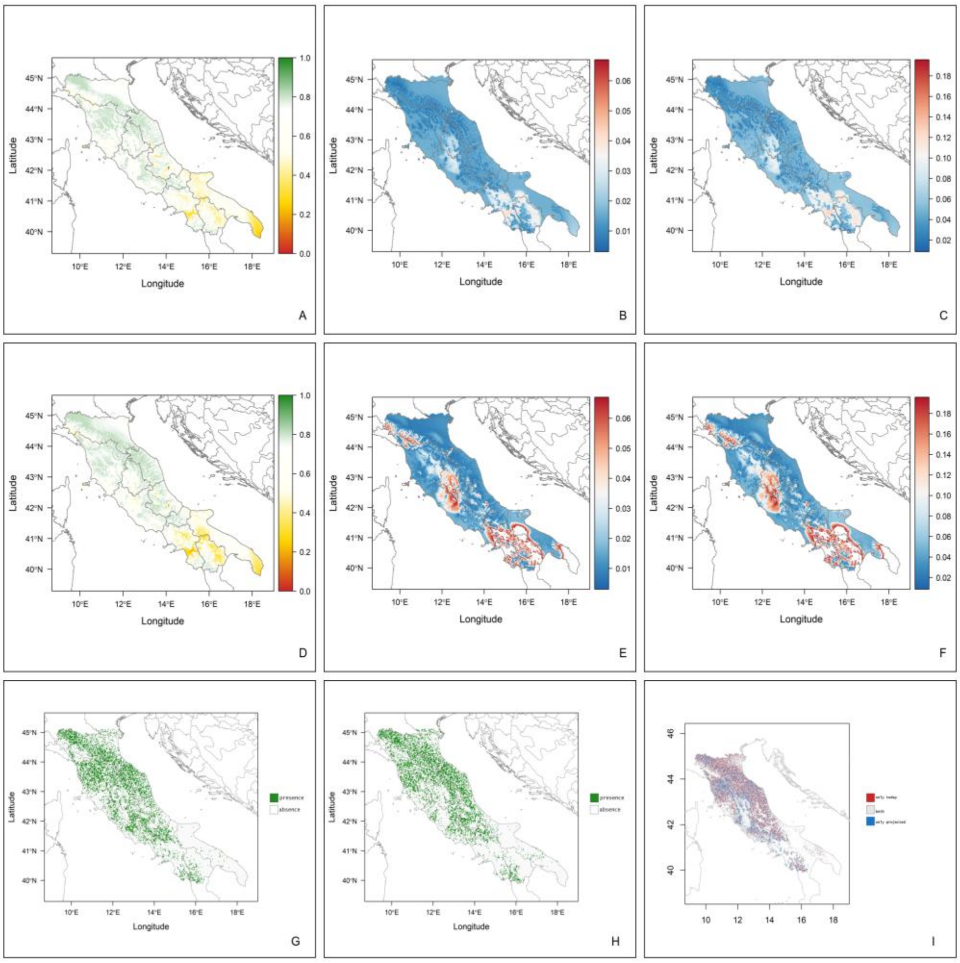

Fig. S.2.219-A) 'Projected' base output; B) 'Projected' uncertainty standard deviation; C) 'Projected' uncertainty range of values; D) 'Present' base output; E) 'Present' uncertainty standard deviation; F) 'Present' uncertainty range of values; G) 'Projected' binary distribution; H) 'Present' binary distribution; I) Range size variation.

Value	Present	Projected
AUC.train	0.622	0.621
AUC.test	0.602	0.616
AUC.diff	0.019	0.019
pROC	1.091	1.086
OR.p10	0.101	0.112
CBI	0.697	-0.075
perc.stable	26.744	26.744
range.size	-1.816	-1.816

S.2.220 *Polygala flavescens* Maxent

Species: *Polygala flavescens* DC. subsp. *flavescens*

Procedure: Maxent

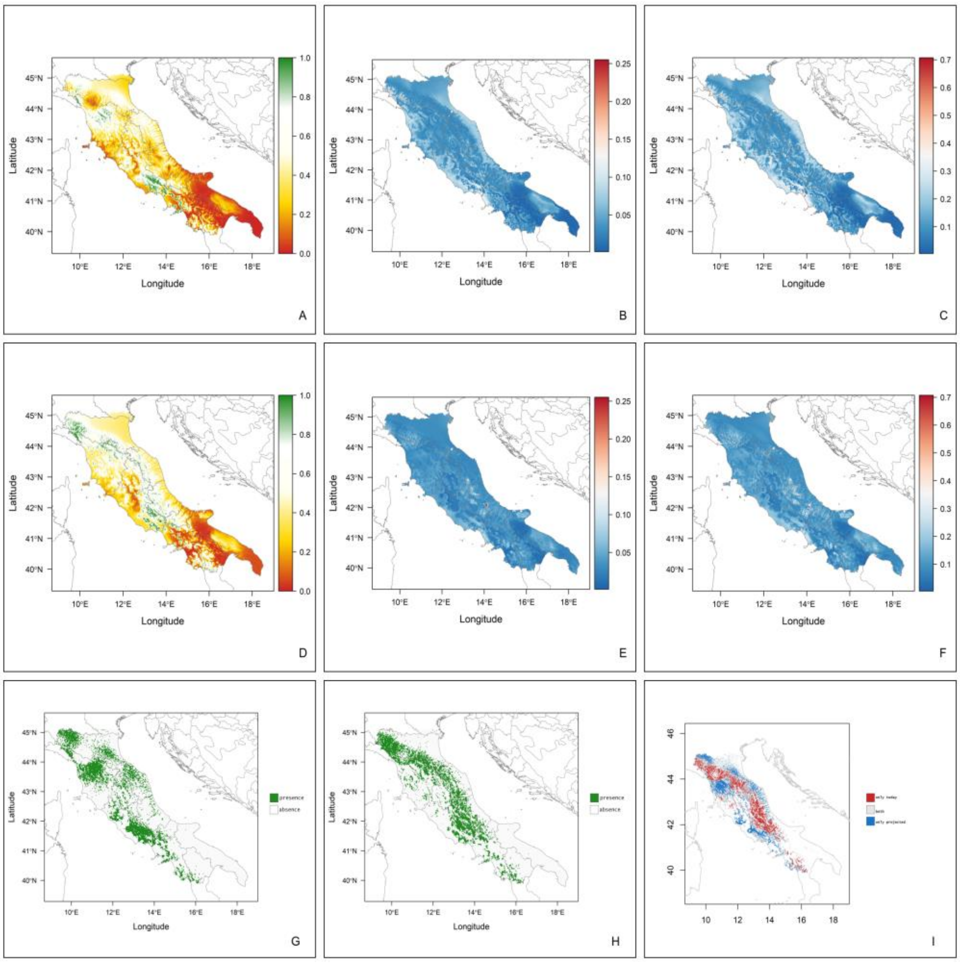

Fig. S.2.220-A) 'Projected' base output; B) 'Projected' uncertainty standard deviation; C) 'Projected' uncertainty range of values; D) 'Present' base output; E) 'Present' uncertainty standard deviation; F) 'Present' uncertainty range of values; G) 'Projected' binary distribution; H) 'Present' binary distribution; I) Range size variation.

Value	Present	Projected
AUC.train	0.738	0.775
AUC.test	0.722	0.767
AUC.diff	0.016	0.016
pROC	1.171	1.199
OR.p10	0.117	0.121
CBI	0.978	0.818
perc.stable	27.875	27.875
range.size	-0.746	-0.746

S.2.221 *Polygala flavescens* KDE

Species: *Polygala flavescens* DC. subsp. *flavescens*

Procedure: KDE

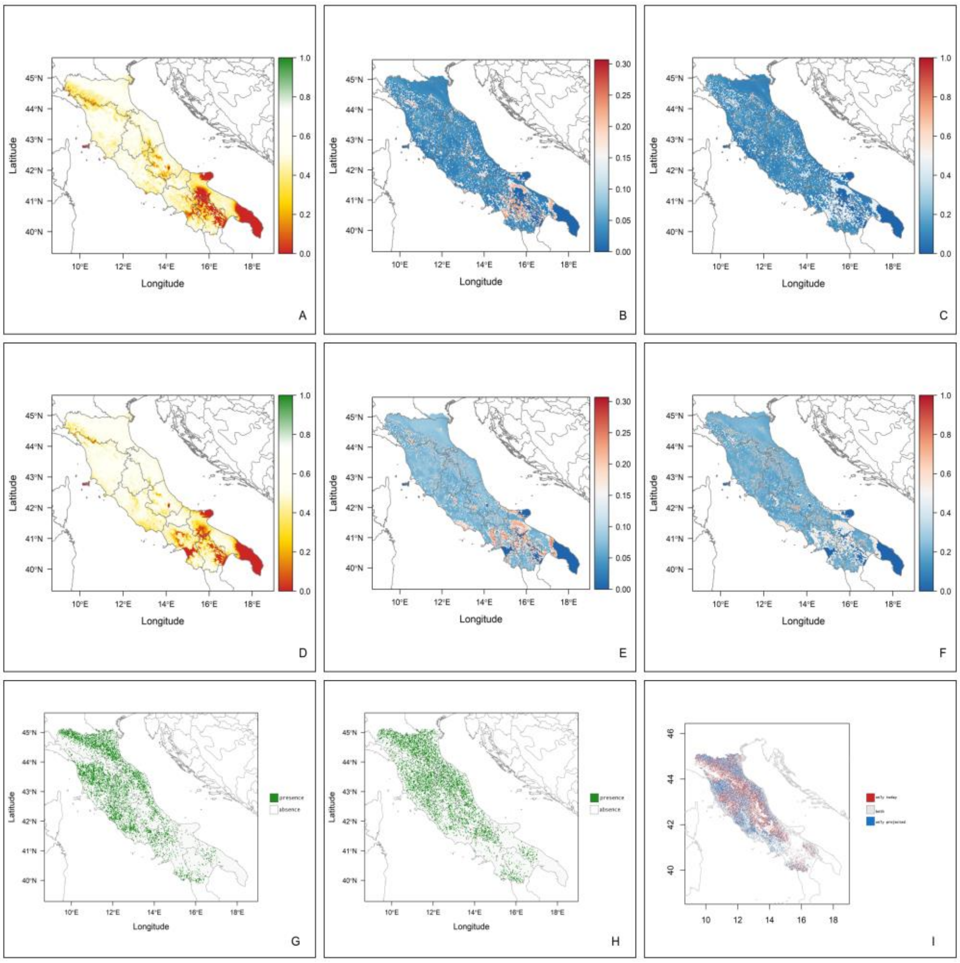

Fig. S.2.221-A) 'Projected' base output; B) 'Projected' uncertainty standard deviation; C) 'Projected' uncertainty range of values; D) 'Present' base output; E) 'Present' uncertainty standard deviation; F) 'Present' uncertainty range of values; G) 'Projected' binary distribution; H) 'Present' binary distribution; I) Range size variation.

Value	Present	Projected
AUC.train	0.584	0.613
AUC.test	0.553	0.562
AUC.diff	0.035	0.058
pROC	1.070	1.054
OR.p10	0.095	0.170
CBI	0.835	-0.127
perc.stable	19.619	19.619
range.size	0.104	0.104

S.2.222 *Polygala flavescens* CTA

Species: *Polygala flavescens* DC. subsp. *flavescens*

Procedure: CTA

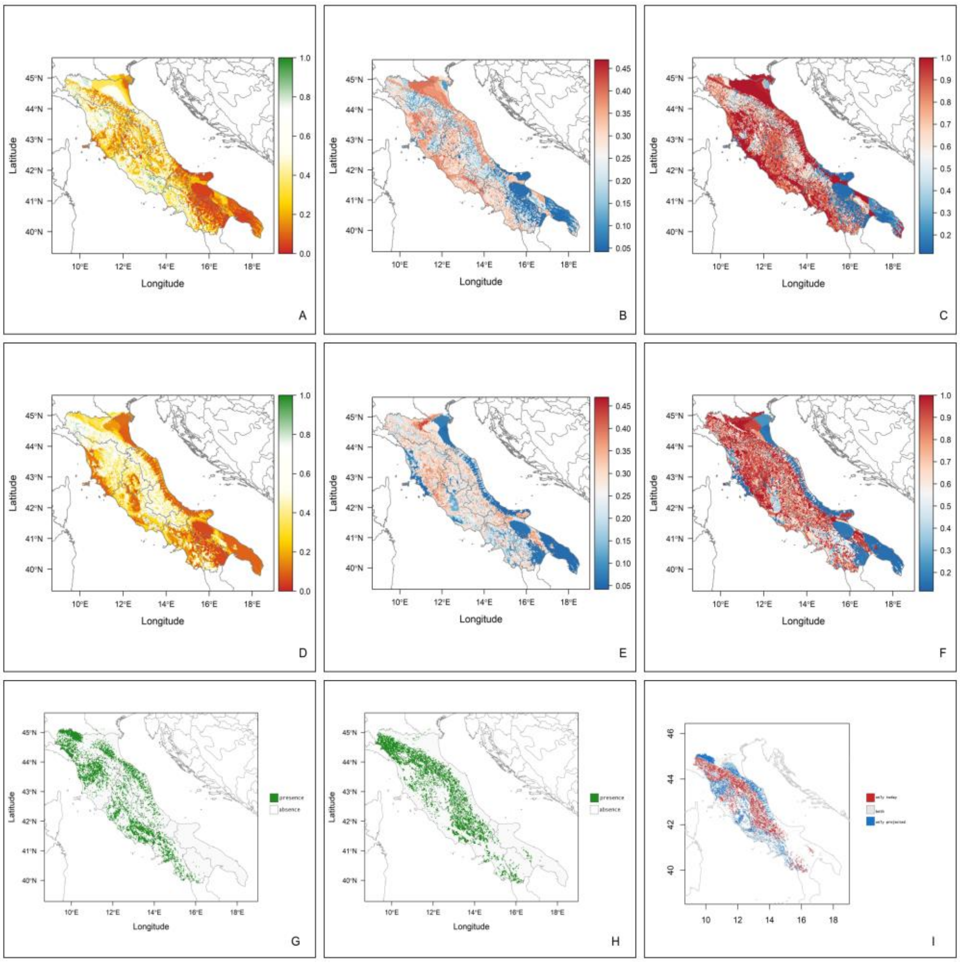

Fig. S.2.222-A) 'Projected' base output; B) 'Projected' uncertainty standard deviation; C) 'Projected' uncertainty range of values; D) 'Present' base output; E) 'Present' uncertainty standard deviation; F) 'Present' uncertainty range of values; G) 'Projected' binary distribution; H) 'Present' binary distribution; I) Range size variation.

Value	Present	Projected
AUC.train	0.856	0.883
AUC.test	0.749	0.763
AUC.diff	0.120	0.120
pROC	1.072	1.078
OR.p10	0.215	0.207
CBI	0.994	0.798
perc.stable	20.693	20.693
range.size	-0.765	-0.765

S.2.223 *Polygala flavescens* FDA

Species: *Polygala flavescens* DC. subsp. *flavescens*

Procedure: FDA

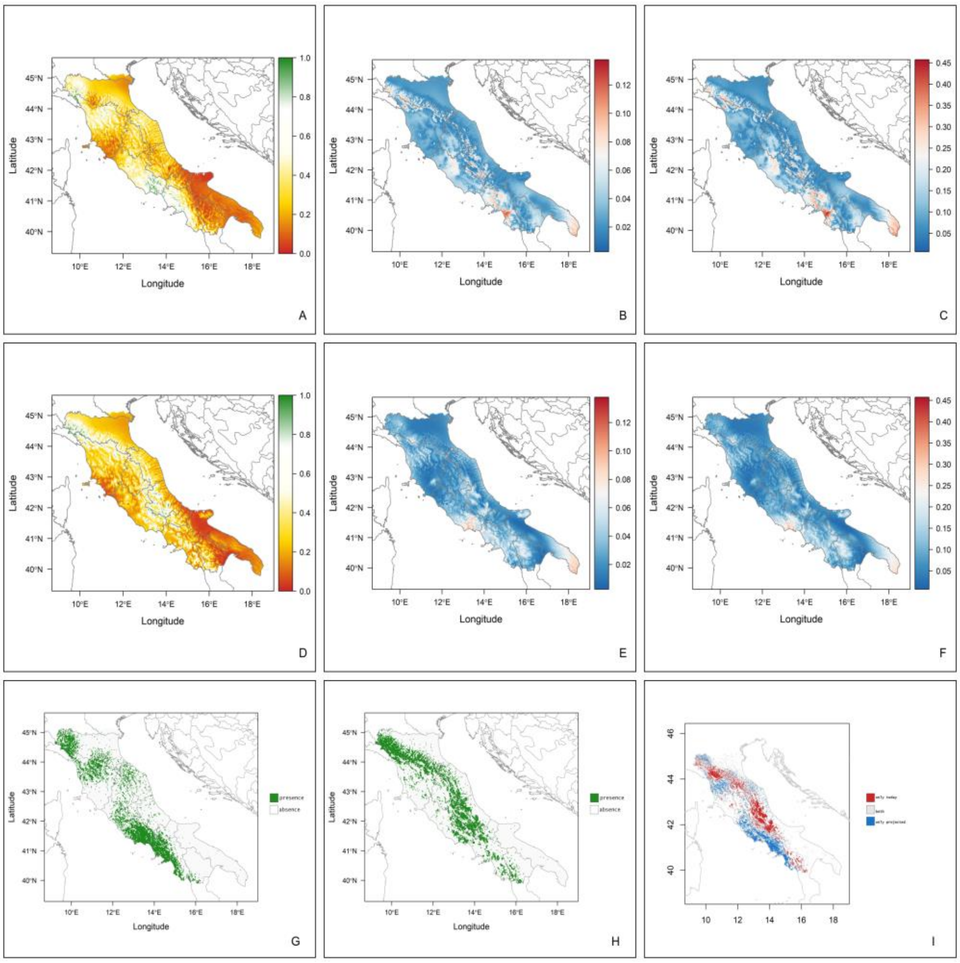

Fig. S.2.223-A) ‘Projected’ base output; B) ‘Projected’ uncertainty standard deviation; C) ‘Projected’ uncertainty range of values; D) ’Present’ base output; E) ’Present’ uncertainty standard deviation; F) ’Present’ uncertainty range of values; G) ‘Projected’ binary distribution; H) ‘Present’ binary distribution; I) Range size variation.

Value	Present	Projected
AUC.train	0.718	0.749
AUC.test	0.718	0.737
AUC.diff	0.031	0.031
pROC	1.133	1.179
OR.p10	0.098	0.123
CBI	0.911	0.885
perc.stable	30.746	30.746
range.size	-0.161	-0.161

S.2.224 *Polygala flavescens* GAM

Species: *Polygala flavescens* DC. subsp. *flavescens*

Procedure: GAM

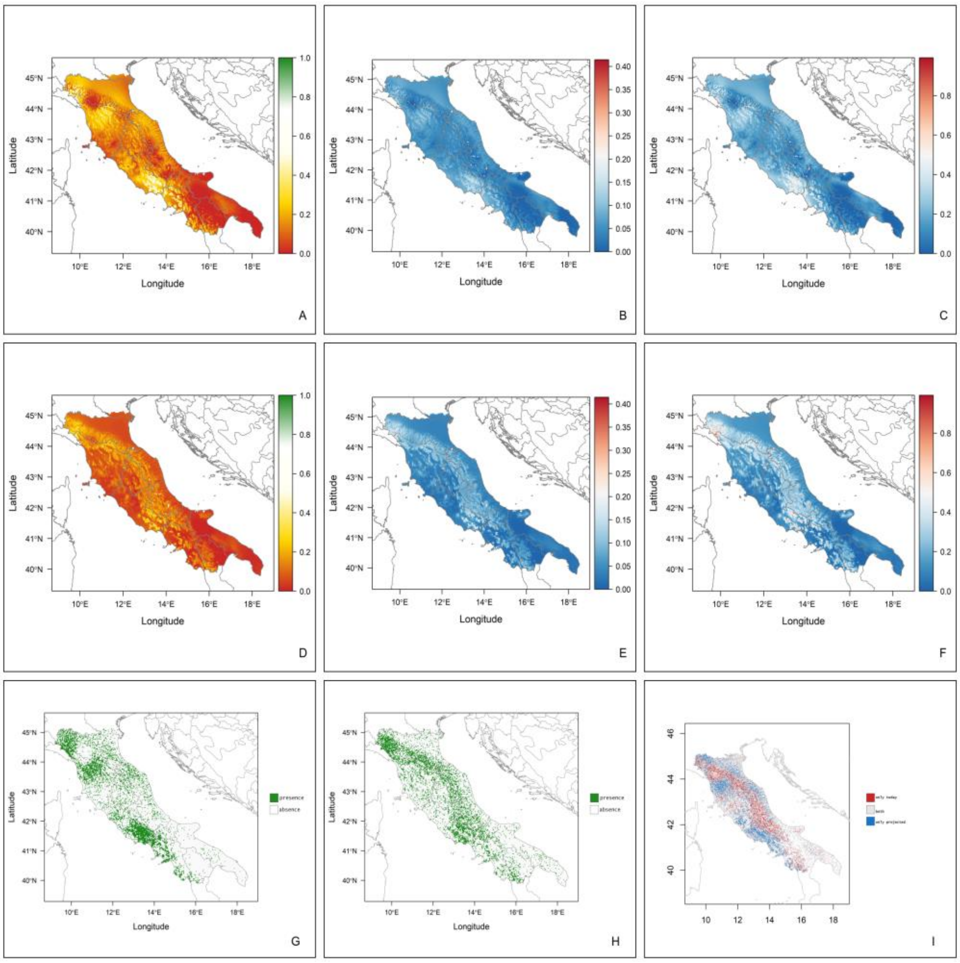

Fig. S.2.224-A) 'Projected' base output; B) 'Projected' uncertainty standard deviation; C) 'Projected' uncertainty range of values; D) 'Present' base output; E) 'Present' uncertainty standard deviation; F) 'Present' uncertainty range of values; G) 'Projected' binary distribution; H) 'Present' binary distribution; I) Range size variation.

Value	Present	Projected
AUC.train	0.727	0.784
AUC.test	0.713	0.733
AUC.diff	0.051	0.051
pROC	1.143	1.150
OR.p10	0.098	0.151
CBI	0.904	0.815
perc.stable	22.296	22.296
range.size	-1.050	-1.050

S.2.225 *Polygala flavescens* GLM

Species: *Polygala flavescens* DC. subsp. *flavescens*

Procedure: GLM

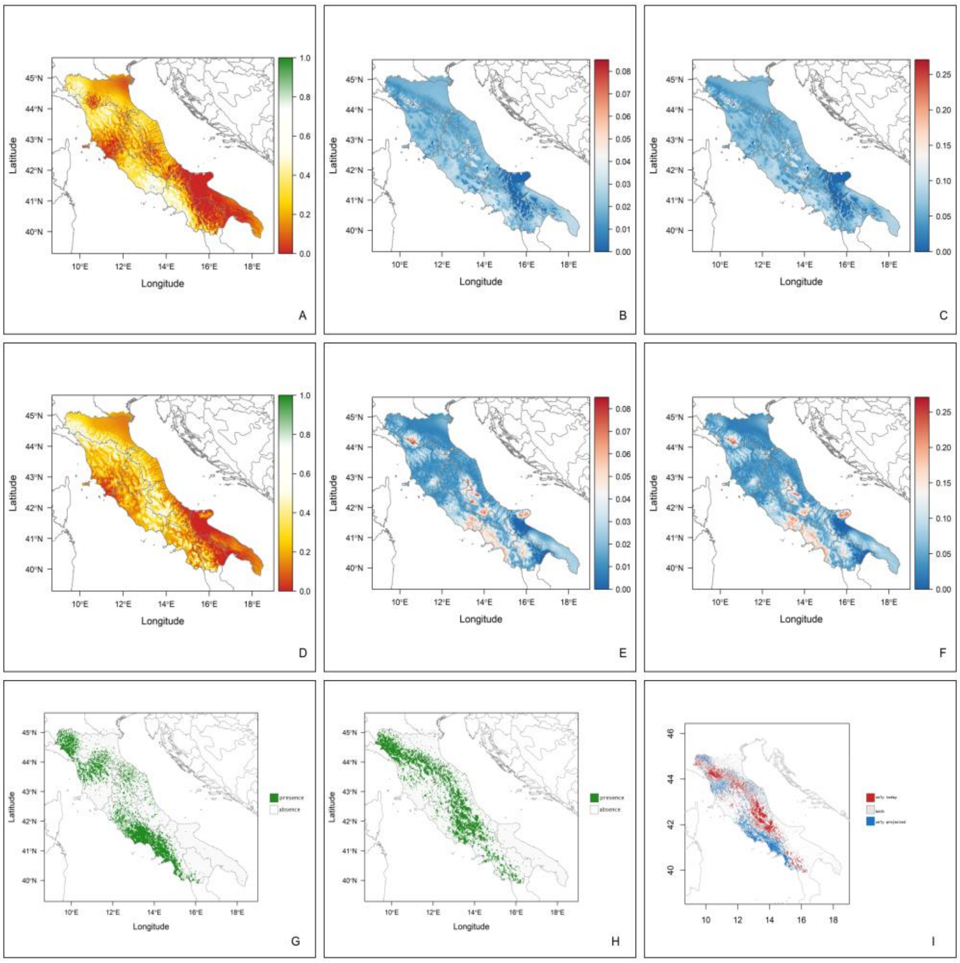

Fig. S.2.225-A) ‘Projected’ base output; B) ‘Projected’ uncertainty standard deviation; C) ‘Projected’ uncertainty range of values; D) ’Present’ base output; E) ’Present’ uncertainty standard deviation; F) ’Present’ uncertainty range of values; G) ‘Projected’ binary distribution; H) ‘Present’ binary distribution; I) Range size variation.

Value	Present	Projected
AUC.train	0.704	0.749
AUC.test	0.702	0.736
AUC.diff	0.028	0.028
pROC	1.127	1.196
OR.p10	0.110	0.119
CBI	0.835	0.907
perc.stable	30.025	30.025
range.size	0.112	0.112

S.2.226 *Polygala flavescens* MARS

Species: *Polygala flavescens* DC. subsp. *flavescens*

Procedure: MARS

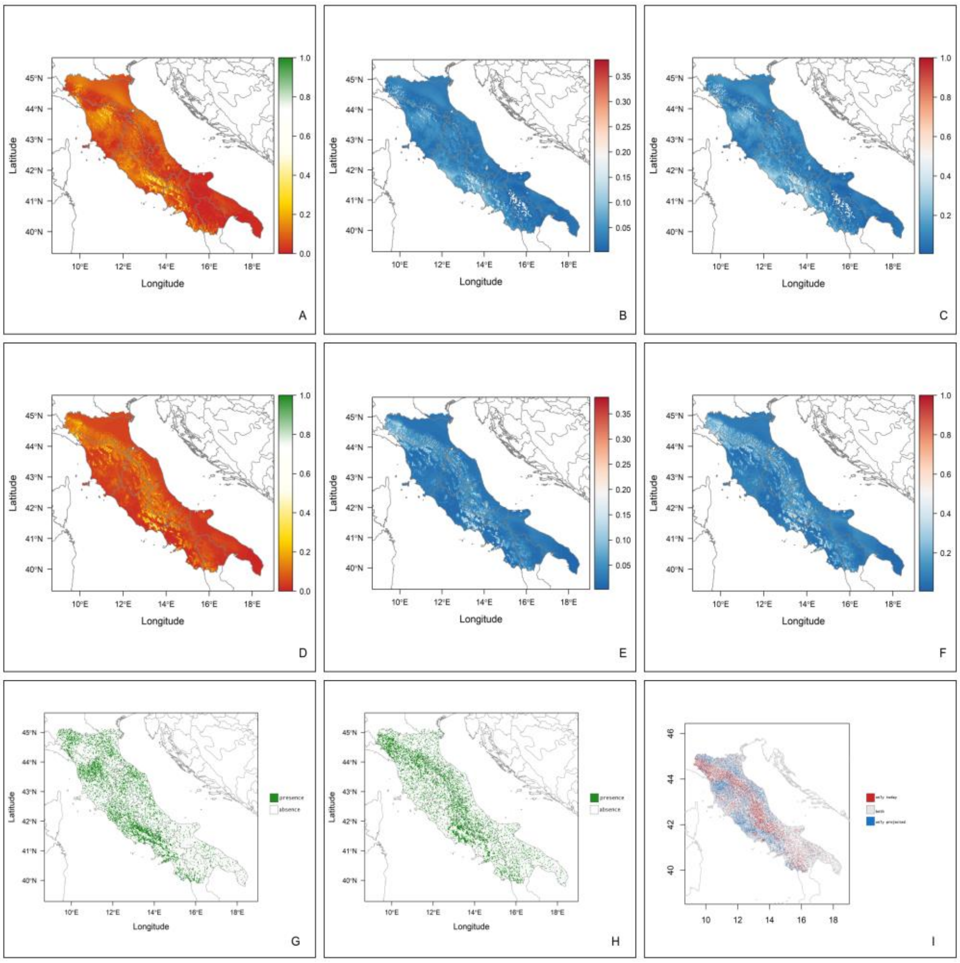

Fig. S.2.226-A) 'Projected' base output; B) 'Projected' uncertainty standard deviation; C) 'Projected' uncertainty range of values; D) 'Present' base output; E) 'Present' uncertainty standard deviation; F) 'Present' uncertainty range of values; G) 'Projected' binary distribution; H) 'Present' binary distribution; I) Range size variation.

Value	Present	Projected
AUC.train	0.731	0.803
AUC.test	0.681	0.750
AUC.diff	0.054	0.054
pROC	1.104	1.143
OR.p10	0.142	0.174
CBI	0.901	0.743
perc.stable	17.025	17.025
range.size	0.867	0.867

S.2.227 *Polygala flavescens* RF

Species: *Polygala flavescens* DC. subsp. *flavescens*

Procedure: RF

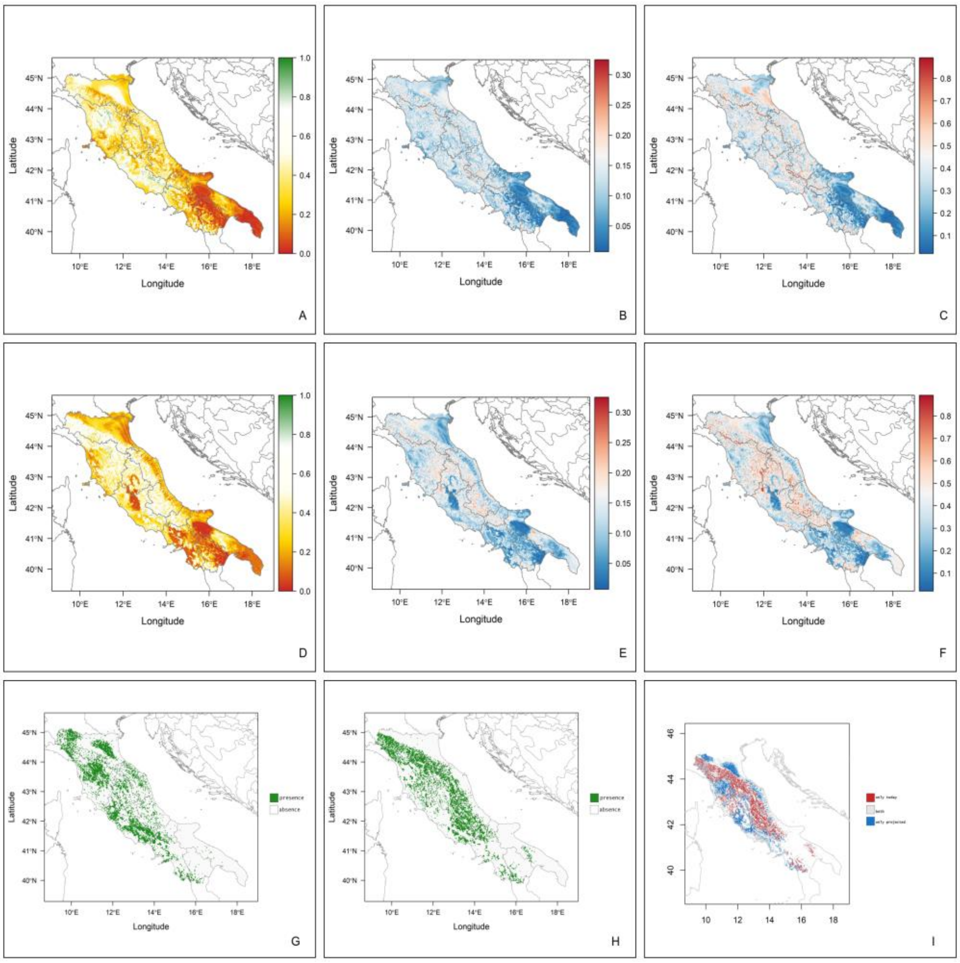

Fig. S.2.227-A) ‘Projected’ base output; B) ‘Projected’ uncertainty standard deviation; C) ‘Projected’ uncertainty range of values; D) ’Present’ base output; E) ’Present’ uncertainty standard deviation; F) ’Present’ uncertainty range of values; G) ‘Projected’ binary distribution; H) ‘Present’ binary distribution; I) Range size variation.

Value	Present	Projected
AUC.train	1.000	1.000
AUC.test	0.941	0.934
AUC.diff	0.066	0.066
pROC	1.168	1.167
OR.p10	0.734	0.667
CBI	0.991	0.928
perc.stable	22.861	22.861
range.size	-0.213	-0.213

S.2.228 *Polygala flavescens* SVM

Species: *Polygala flavescens* DC. subsp. *flavescens*

Procedure: SVM

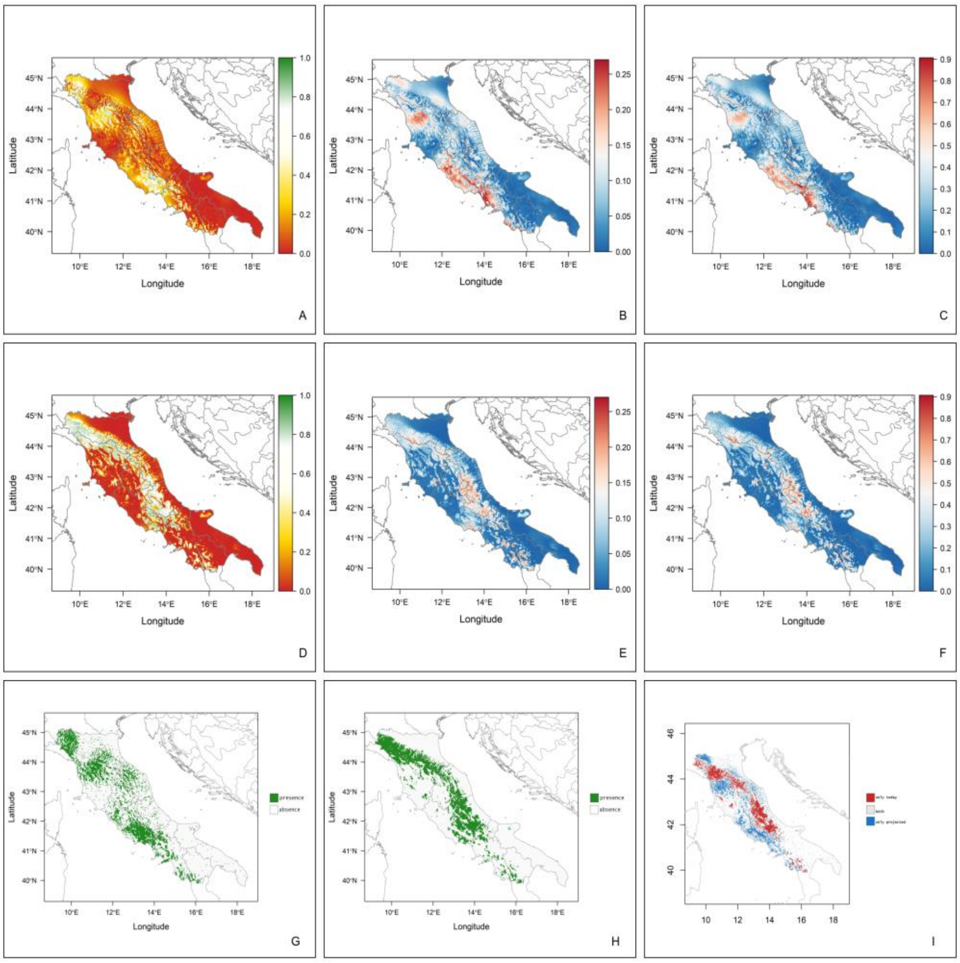

Fig. S.2.228-A) 'Projected' base output; B) 'Projected' uncertainty standard deviation; C) 'Projected' uncertainty range of values; D) 'Present' base output; E) 'Present' uncertainty standard deviation; F) 'Present' uncertainty range of values; G) 'Projected' binary distribution; H) 'Present' binary distribution; I) Range size variation.

Value	Present	Projected
AUC.train	0.756	0.782
AUC.test	0.734	0.753
AUC.diff	0.032	0.032
pROC	1.097	1.170
OR.p10	0.131	0.137
CBI	0.890	0.911
perc.stable	31.021	31.021
range.size	-0.602	-0.602

S.2.229 *Ranunculus pollinensis* Biomod2

Species: *Ranunculus pollinensis* (N.Terracc.) Chiov.

Procedure: Biomod2

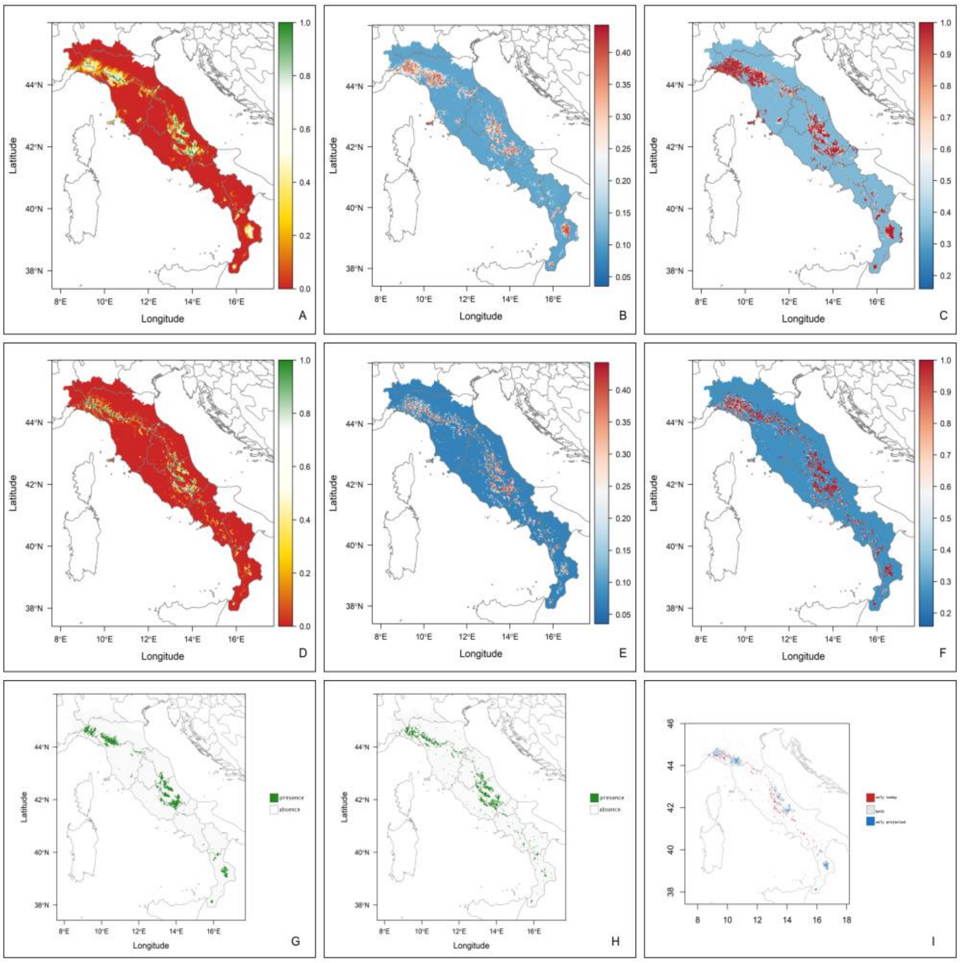

Fig. S.2.229-A) ‘Projected’ base output; B) ‘Projected’ uncertainty standard deviation; C) ‘Projected’ uncertainty range of values; D) ’Present’ base output; E) ’Present’ uncertainty standard deviation; F) ’Present’ uncertainty range of values; G) ‘Projected’ binary distribution; H) ‘Present’ binary distribution; I) Range size variation.

Value	Present	Projected
AUC.train	0.999	0.992
AUC.test	0.981	0.975
AUC.diff	0.017	0.017
pROC	1.860	1.853
OR.p10	0.422	0.278
CBI	0.907	0.971
perc.stable	49.342	49.342
range.size	1.898	1.898

S.2.230 *Ranunculus pollinensis* Bioclim

Species: *Ranunculus pollinensis* (N.Terracc.) Chiov.

Procedure: Bioclim

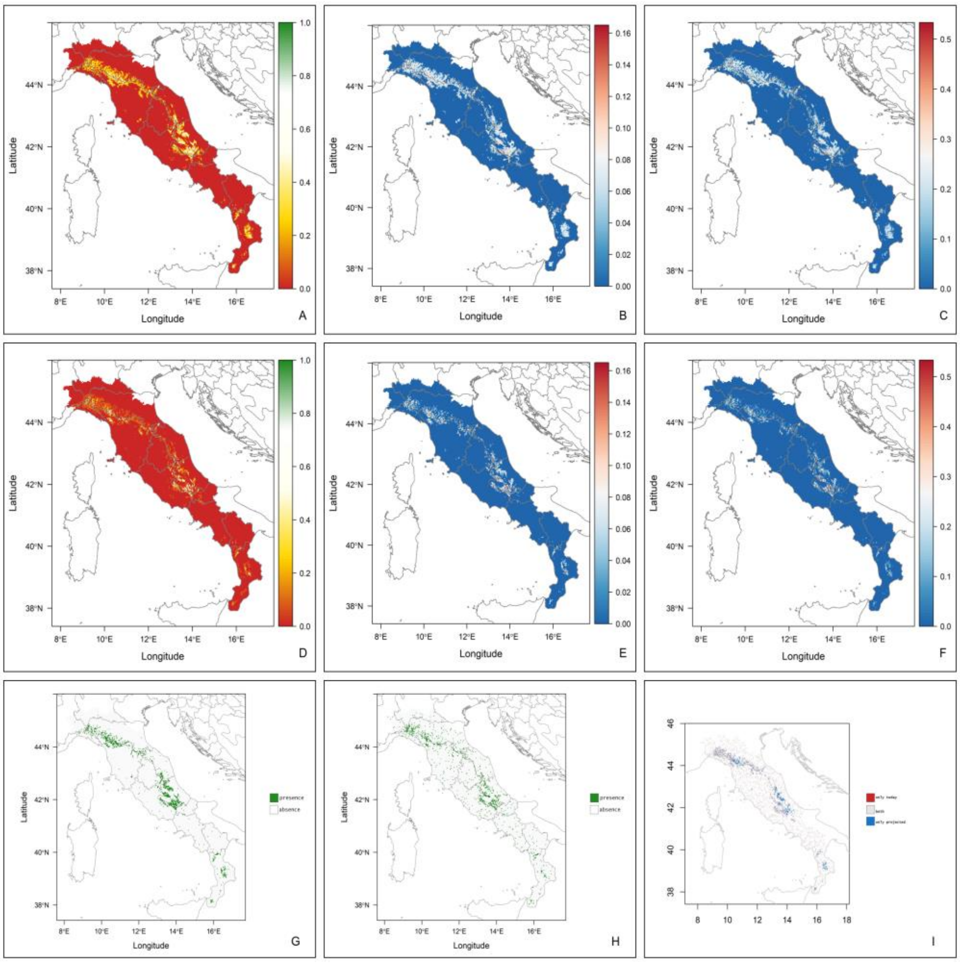

Fig. S.2.230-A) 'Projected' base output; B) 'Projected' uncertainty standard deviation; C) 'Projected' uncertainty range of values; D) 'Present' base output; E) 'Present' uncertainty standard deviation; F) 'Present' uncertainty range of values; G) 'Projected' binary distribution; H) 'Present' binary distribution; I) Range size variation.

Value	Present	Projected
AUC.train	0.964	0.937
AUC.test	0.884	0.804
AUC.diff	0.133	0.133
pROC	1.418	1.229
OR.p10	0.167	0.256
CBI	0.496	0.921
perc.stable	25.737	25.737
range.size	0.495	0.495

S.2.231 *Ranunculus pollinensis* Domain

Species: *Ranunculus pollinensis* (N.Terracc.) Chiov.

Procedure: Domain

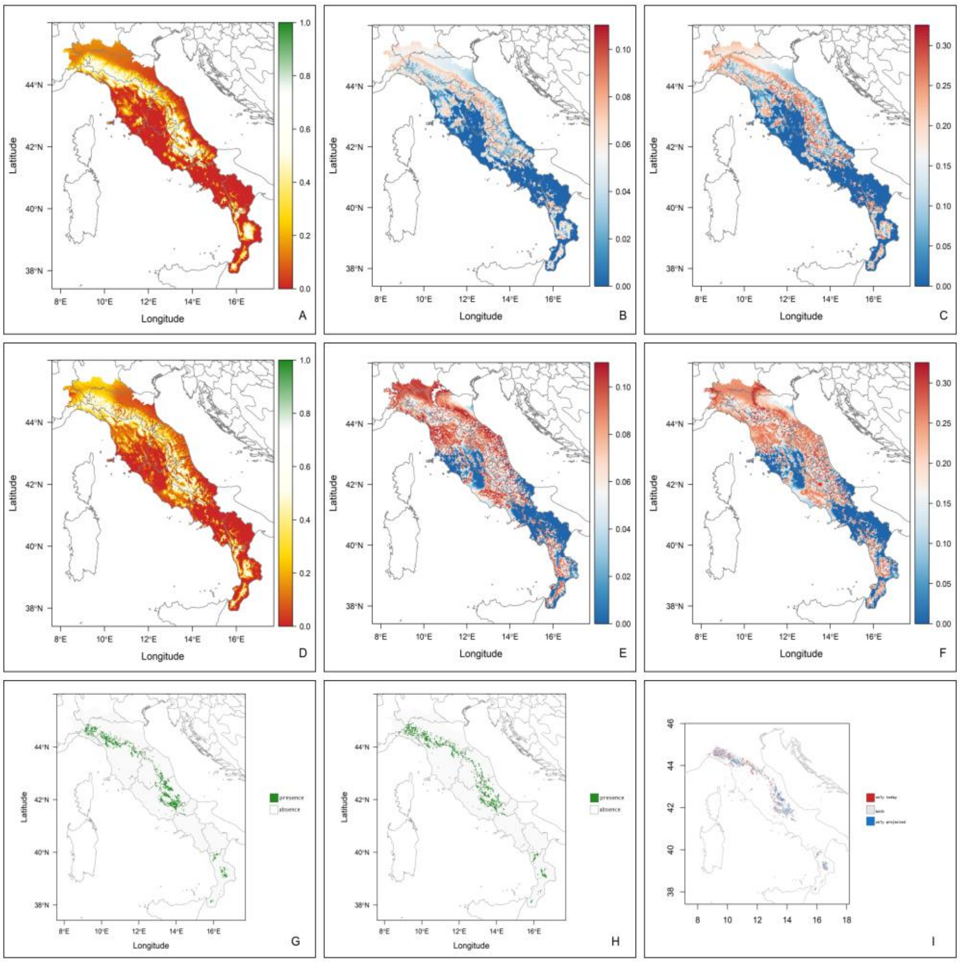

Fig. S.2.231-A) 'Projected' base output; B) 'Projected' uncertainty standard deviation; C) 'Projected' uncertainty range of values; D) 'Present' base output; E) 'Present' uncertainty standard deviation; F) 'Present' uncertainty range of values; G) 'Projected' binary distribution; H) 'Present' binary distribution; I) Range size variation.

Value	Present	Projected
AUC.train	0.955	0.944
AUC.test	0.943	0.937
AUC.diff	0.010	0.010
pROC	1.775	1.776
OR.p10	0.167	0.167
CBI	0.984	0.963
perc.stable	38.579	38.579
range.size	0.519	0.519

S.2.232 *Ranunculus pollinensis* Maxent

Species: *Ranunculus pollinensis* (N.Terracc.) Chiov.

Procedure: Maxent

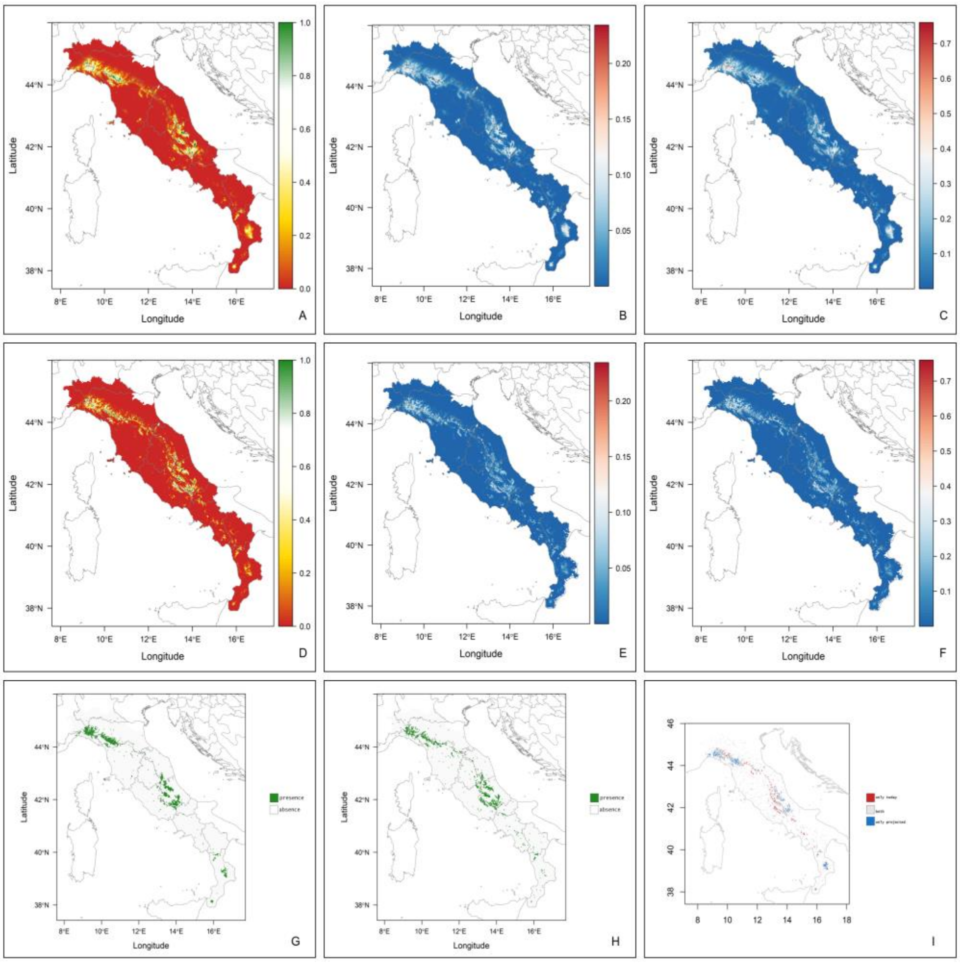

Fig. S.2.232-A) 'Projected' base output; B) 'Projected' uncertainty standard deviation; C) 'Projected' uncertainty range of values; D) 'Present' base output; E) 'Present' uncertainty standard deviation; F) 'Present' uncertainty range of values; G) 'Projected' binary distribution; H) 'Present' binary distribution; I) Range size variation.

Value	Present	Projected
AUC.train	0.977	0.966
AUC.test	0.981	0.951
AUC.diff	0.018	0.018
pROC	1.904	1.789
OR.p10	0.122	0.189
CBI	0.949	0.946
perc.stable	46.995	46.995
range.size	1.258	1.258

S.2.233 *Ranunculus pollinensis* KDE

Species: *Ranunculus pollinensis* (N.Terracc.) Chiov.

Procedure: KDE

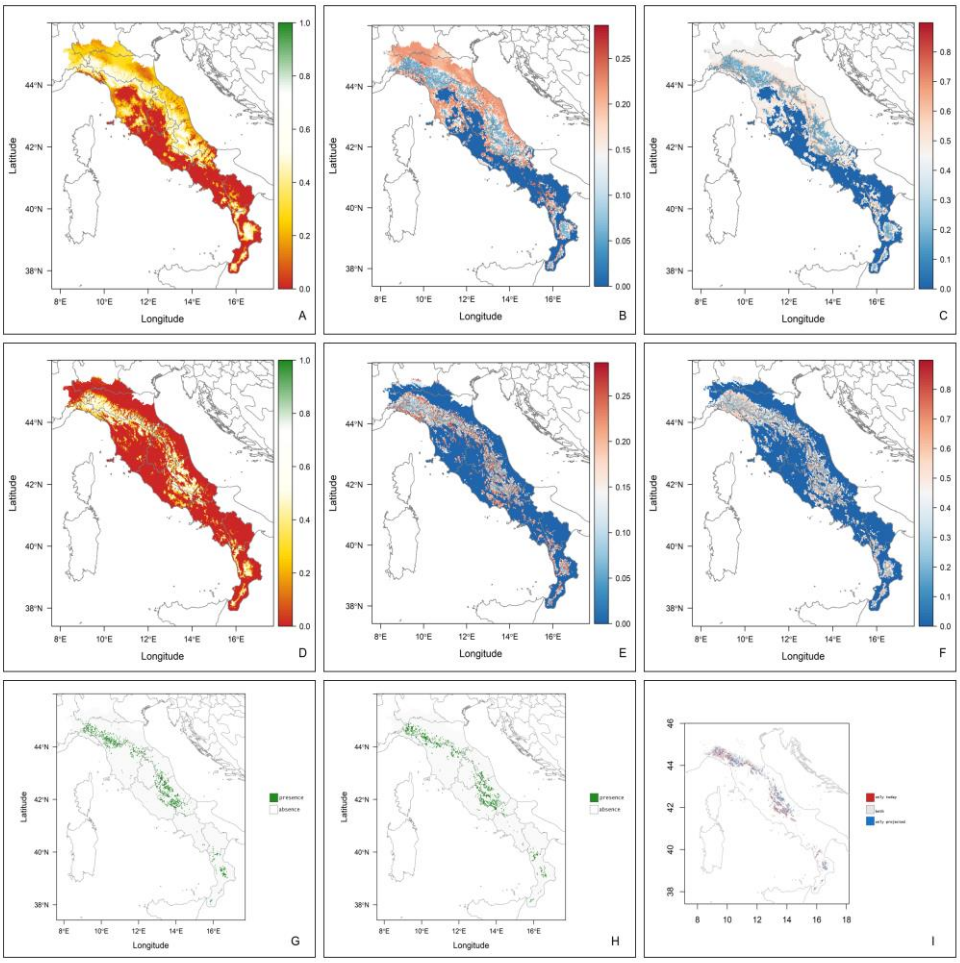

Fig. S.2.233-A) 'Projected' base output; B) 'Projected' uncertainty standard deviation; C) 'Projected' uncertainty range of values; D) 'Present' base output; E) 'Present' uncertainty standard deviation; F) 'Present' uncertainty range of values; G) 'Projected' binary distribution; H) 'Present' binary distribution; I) Range size variation.

Value	Present	Projected
AUC.train	0.958	0.948
AUC.test	0.927	0.908
AUC.diff	0.038	0.047
pROC	1.664	1.663
OR.p10	0.144	0.233
CBI	0.911	0.733
perc.stable	25.477	25.477
range.size	-1.083	-1.083

S.2.234 *Ranunculus pollinensis* CTA

Species: *Ranunculus pollinensis* (N.Terracc.) Chiov.

Procedure: CTA

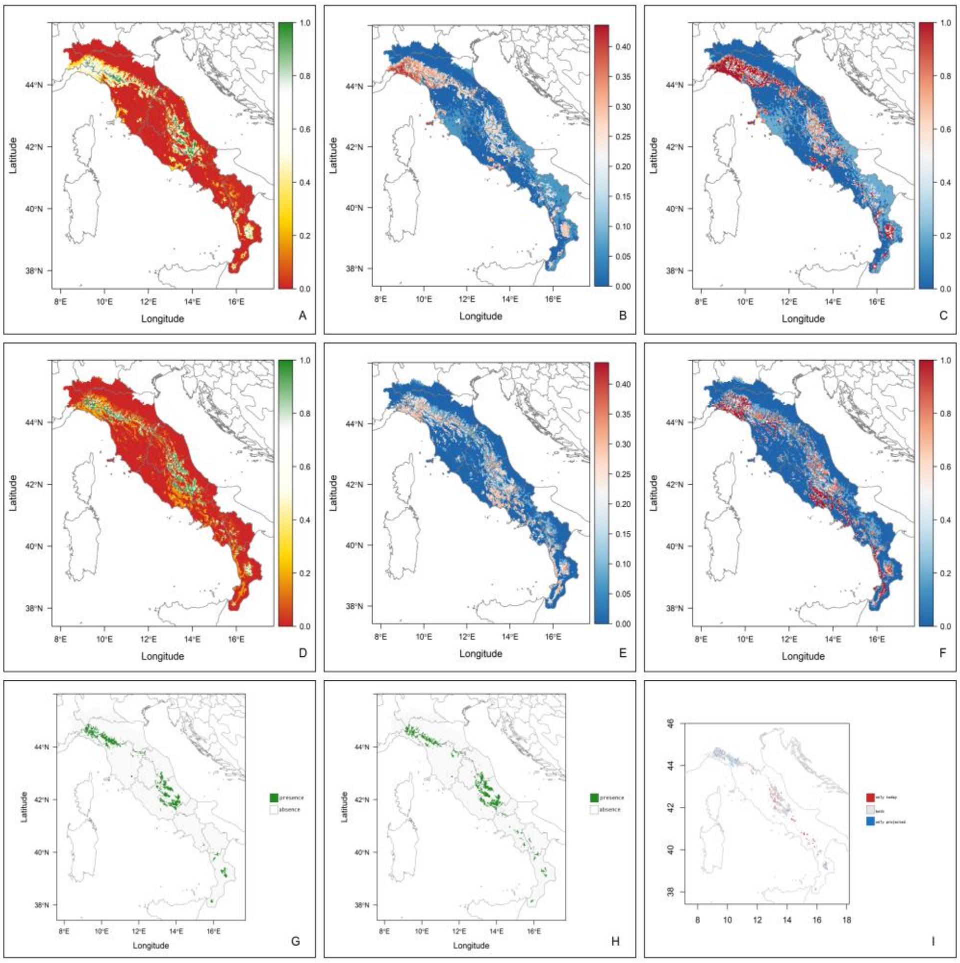

Fig. S.2.234-A) 'Projected' base output; B) 'Projected' uncertainty standard deviation; C) 'Projected' uncertainty range of values; D) 'Present' base output; E) 'Present' uncertainty standard deviation; F) 'Present' uncertainty range of values; G) 'Projected' binary distribution; H) 'Present' binary distribution; I) Range size variation.

Value	Present	Projected
AUC.train	0.987	0.980
AUC.test	0.970	0.922
AUC.diff	0.058	0.058
pROC	1.364	1.375
OR.p10	0.044	0.133
CBI	0.940	0.957
perc.stable	60.028	60.028
range.size	-0.619	-0.619

S.2.235 *Ranunculus pollinensis* FDA

Species: *Ranunculus pollinensis* (N.Terracc.) Chiov.

Procedure: FDA

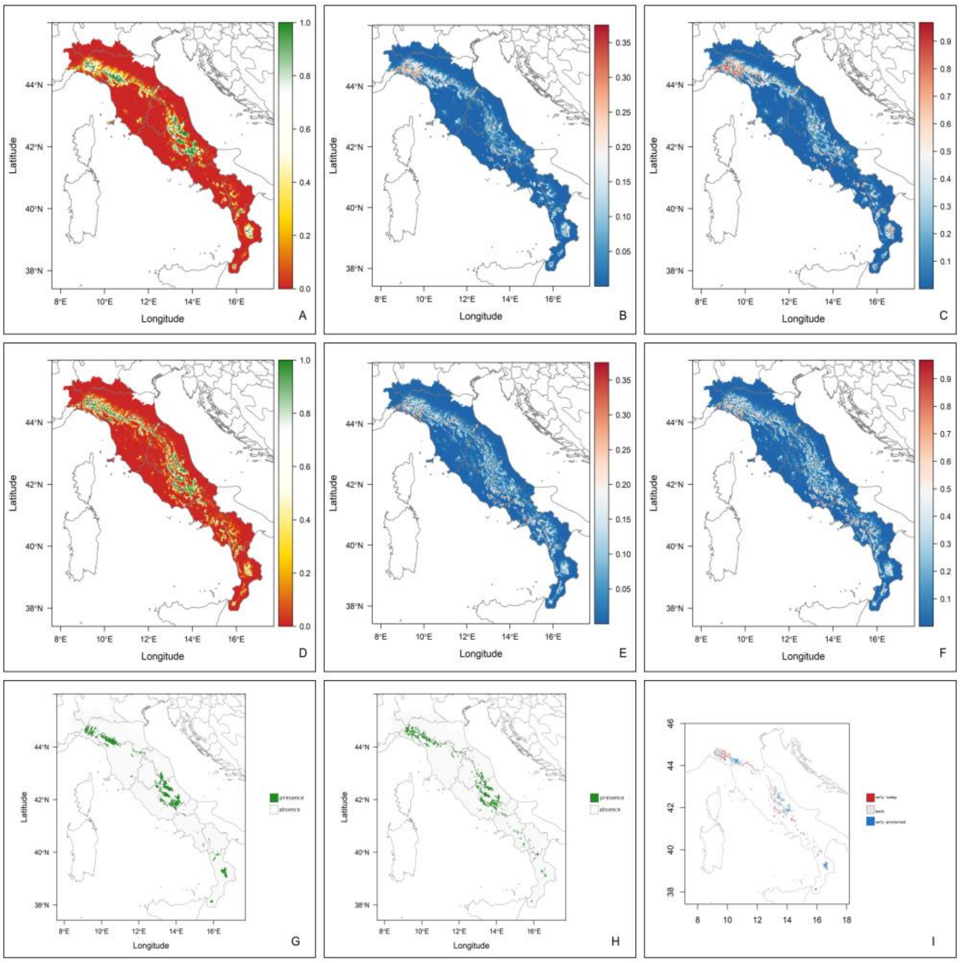

Fig. S.2.235-A) ‘Projected’ base output; B) ‘Projected’ uncertainty standard deviation; C) ‘Projected’ uncertainty range of values; D) ’Present’ base output; E) ’Present’ uncertainty standard deviation; F) ’Present’ uncertainty range of values; G) ‘Projected’ binary distribution; H) ‘Present’ binary distribution; I) Range size variation.

Value	Present	Projected
AUC.train	0.974	0.980
AUC.test	0.982	0.969
AUC.diff	0.011	0.011
pROC	1.889	1.778
OR.p10	0.144	0.211
CBI	0.907	0.971
perc.stable	55.376	55.376
range.size	1.859	1.859

S.2.236 *Ranunculus pollinensis* GAM

Species: *Ranunculus pollinensis* (N.Terracc.) Chiov.

Procedure: GAM

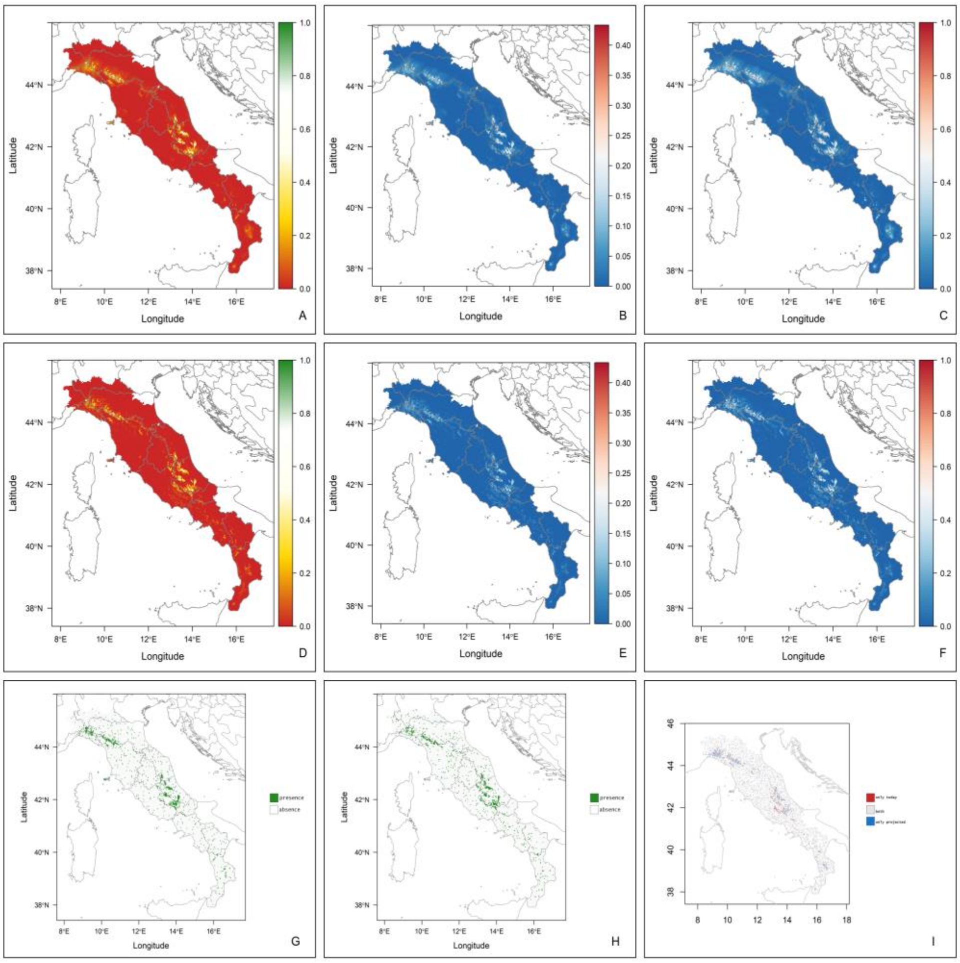

Fig. S.2.236-A) 'Projected' base output; B) 'Projected' uncertainty standard deviation; C) 'Projected' uncertainty range of values; D) 'Present' base output; E) 'Present' uncertainty standard deviation; F) 'Present' uncertainty range of values; G) 'Projected' binary distribution; H) 'Present' binary distribution; I) Range size variation.

Value	Present	Projected
AUC.train	0.980	0.965
AUC.test	0.964	0.941
AUC.diff	0.030	0.030
pROC	1.842	1.739
OR.p10	0.256	0.144
CBI	0.960	0.965
perc.stable	20.990	20.990
range.size	1.745	1.745

S.2.237 *Ranunculus pollinensis* GLM

Species: *Ranunculus pollinensis* (N.Terracc.) Chiov.

Procedure: GLM

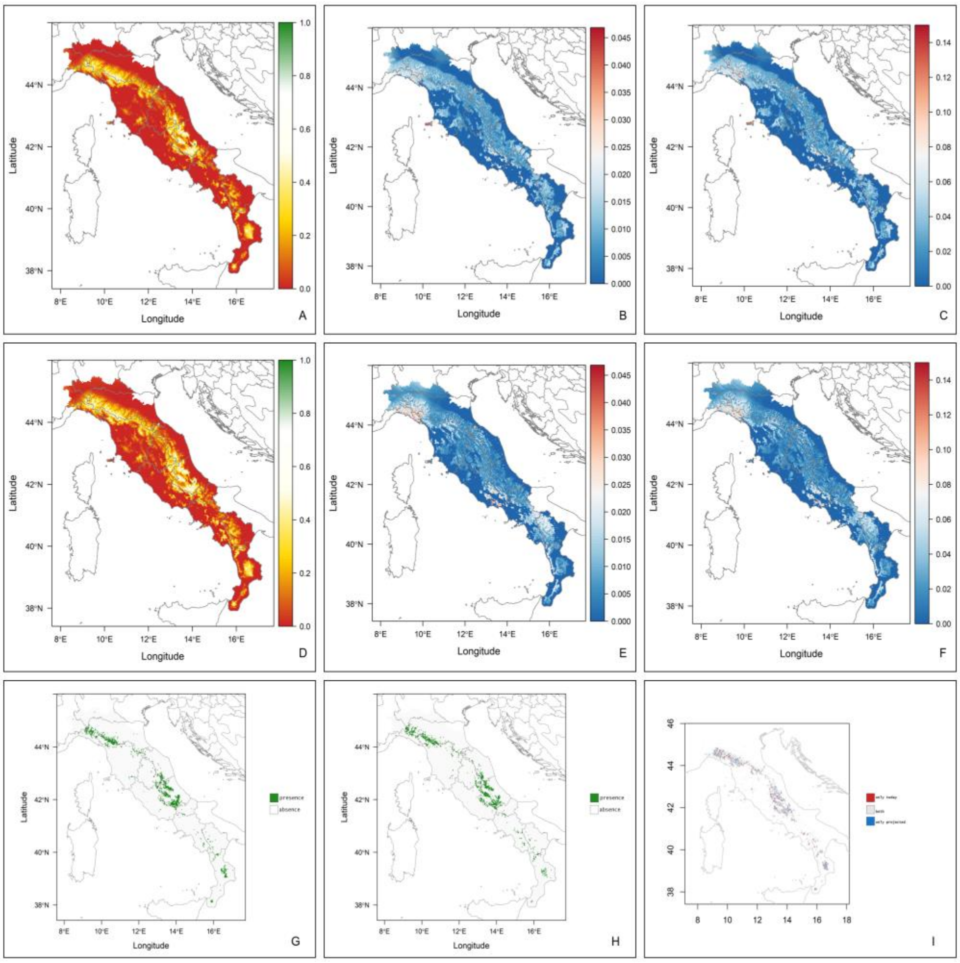

Fig. S.2.237-A) 'Projected' base output; B) 'Projected' uncertainty standard deviation; C) 'Projected' uncertainty range of values; D) 'Present' base output; E) 'Present' uncertainty standard deviation; F) 'Present' uncertainty range of values; G) 'Projected' binary distribution; H) 'Present' binary distribution; I) Range size variation.

Value	Present	Projected
AUC.train	0.975	0.962
AUC.test	0.974	0.957
AUC.diff	0.016	0.016
pROC	1.876	1.814
OR.p10	0.178	0.178
CBI	0.972	0.967
perc.stable	43.286	43.286
range.size	0.621	0.621

S.2.238 *Ranunculus pollinensis* MARS

Species: *Ranunculus pollinensis* (N.Terracc.) Chiov.

Procedure: MARS

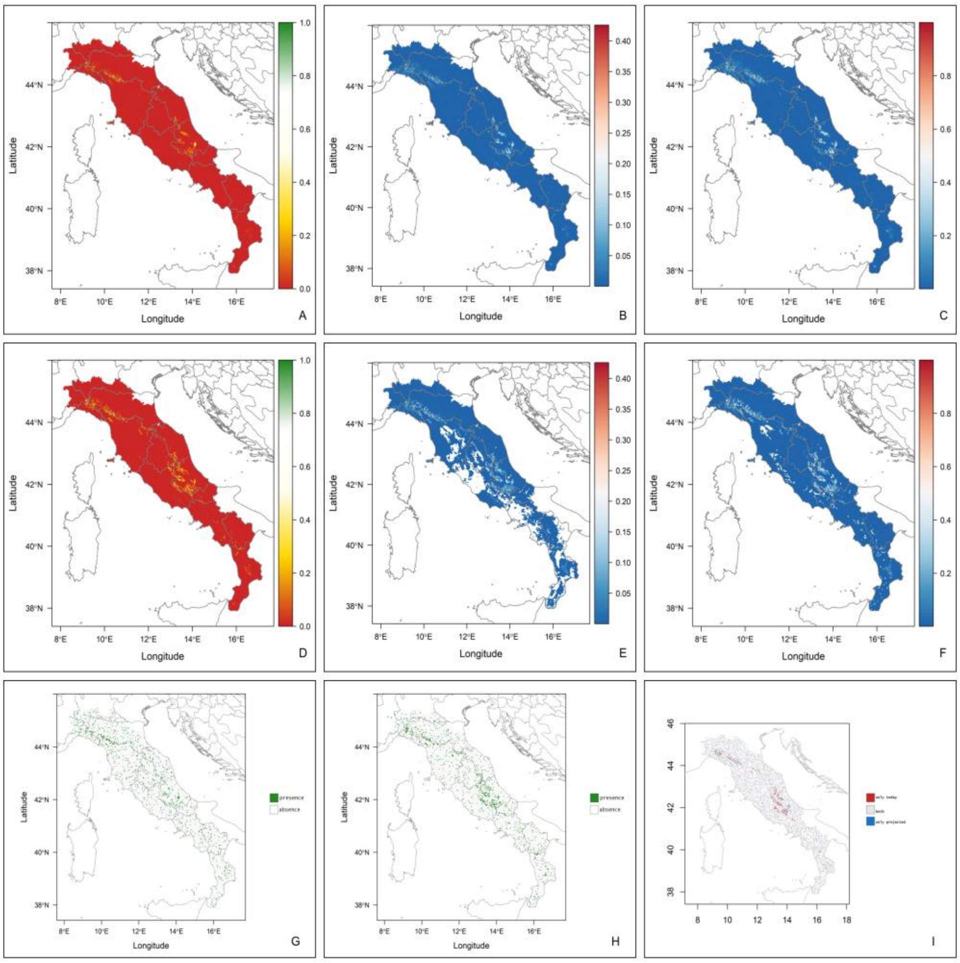

Fig. S.2.238-A) 'Projected' base output; B) 'Projected' uncertainty standard deviation; C) 'Projected' uncertainty range of values; D) 'Present' base output; E) 'Present' uncertainty standard deviation; F) 'Present' uncertainty range of values; G) 'Projected' binary distribution; H) 'Present' binary distribution; I) Range size variation.

Value	Present	Projected
AUC.train	0.965	0.955
AUC.test	0.959	0.851
AUC.diff	0.106	0.106
pROC	1.812	1.416
OR.p10	0.267	0.278
CBI	0.899	0.794
perc.stable	6.268	6.268
range.size	-0.843	-0.843

S.2.239 *Ranunculus pollinensis* RF

Species: *Ranunculus pollinensis* (N.Terracc.) Chiov.

Procedure: RF

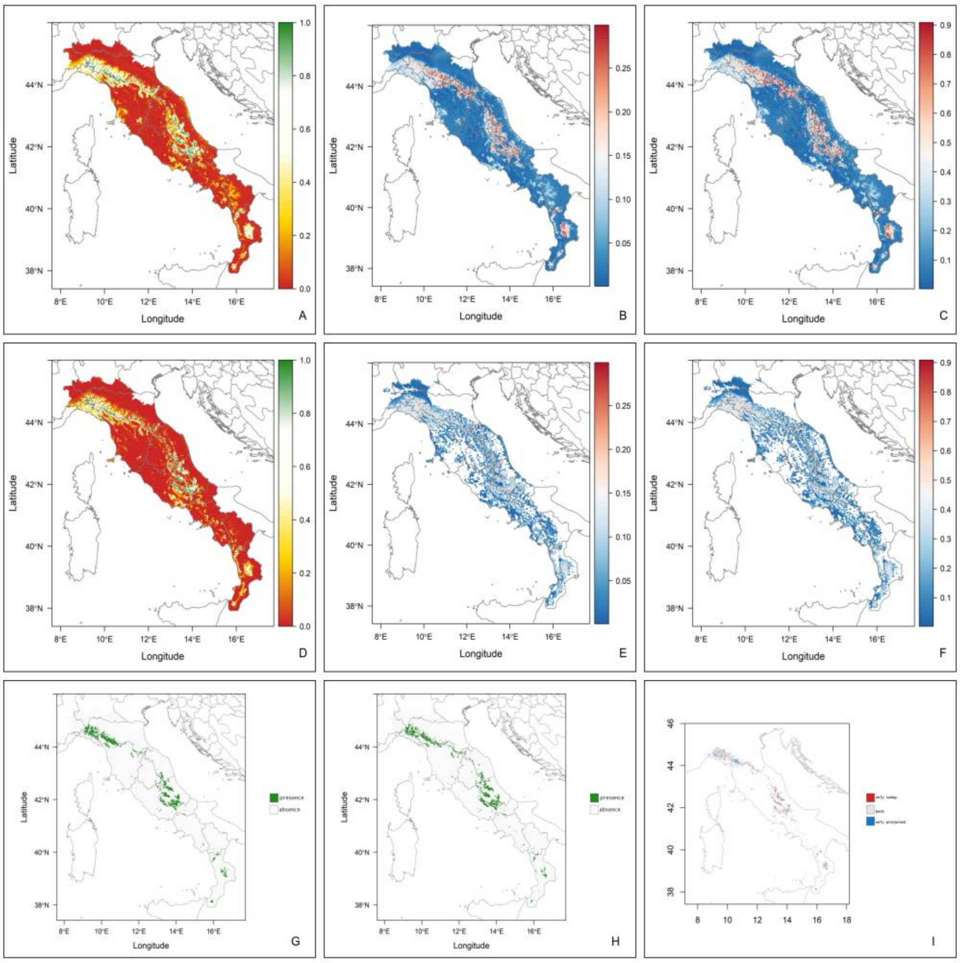

Fig. S.2.239-A) ‘Projected’ base output; B) ‘Projected’ uncertainty standard deviation; C) ‘Projected’ uncertainty range of values; D) ’Present’ base output; E) ’Present’ uncertainty standard deviation; F) ’Present’ uncertainty range of values; G) ‘Projected’ binary distribution; H) ‘Present’ binary distribution; I) Range size variation.

Value	Present	Projected
AUC.train	1.000	1.000
AUC.test	0.994	0.990
AUC.diff	0.010	0.010
pROC	1.877	1.775
OR.p10	0.356	0.278
CBI	0.984	0.936
perc.stable	61.983	61.983
range.size	-0.770	-0.770

S.2.240 *Ranunculus pollinensis* SVM

Species: *Ranunculus pollinensis* (N.Terracc.) Chiov.

Procedure: SVM

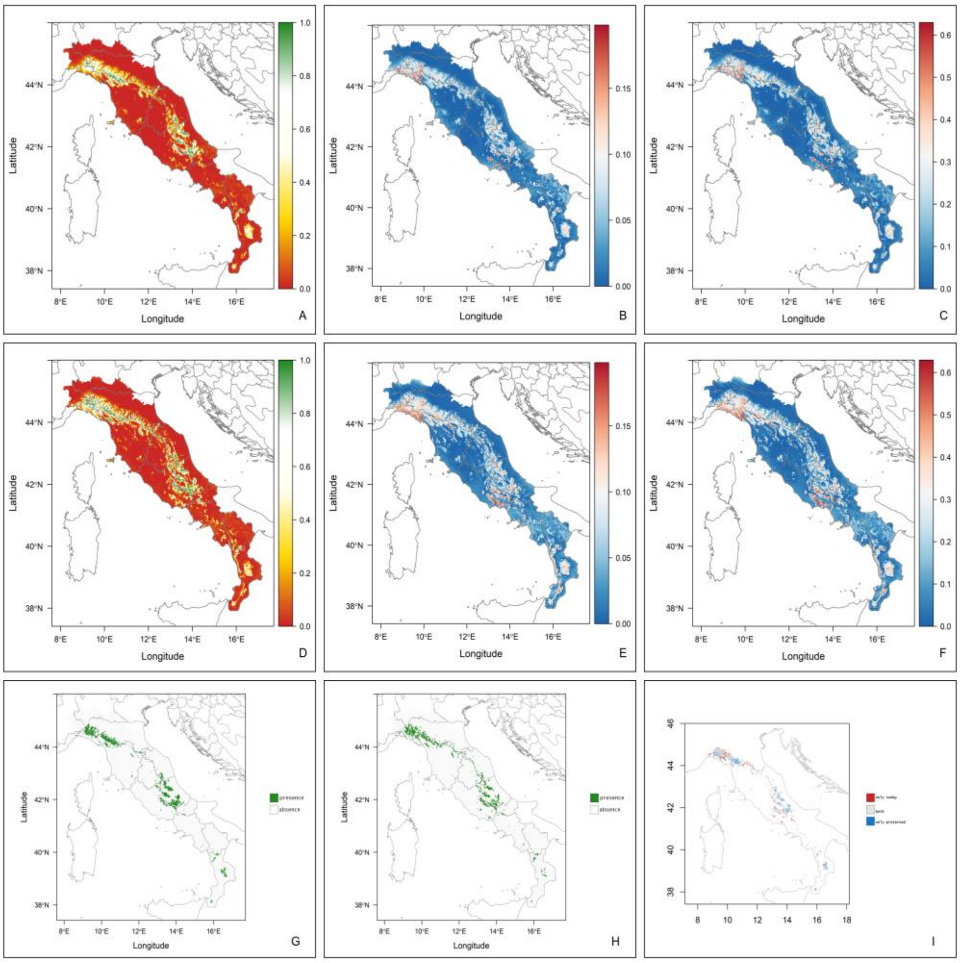

Fig. S.2.240-A) 'Projected' base output; B) 'Projected' uncertainty standard deviation; C) 'Projected' uncertainty range of values; D) 'Present' base output; E) 'Present' uncertainty standard deviation; F) 'Present' uncertainty range of values; G) 'Projected' binary distribution; H) 'Present' binary distribution; I) Range size variation.

Value	Present	Projected
AUC.train	0.981	0.972
AUC.test	0.976	0.967
AUC.diff	0.010	0.010
pROC	1.858	1.804
OR.p10	0.222	0.156
CBI	0.922	0.976
perc.stable	56.919	56.919
range.size	1.640	1.640

S.2.241 *Saxifraga porophylla* Biomod2

Species: *Saxifraga porophylla* Bertol. subsp. *porophylla*

Procedure: Biomod2

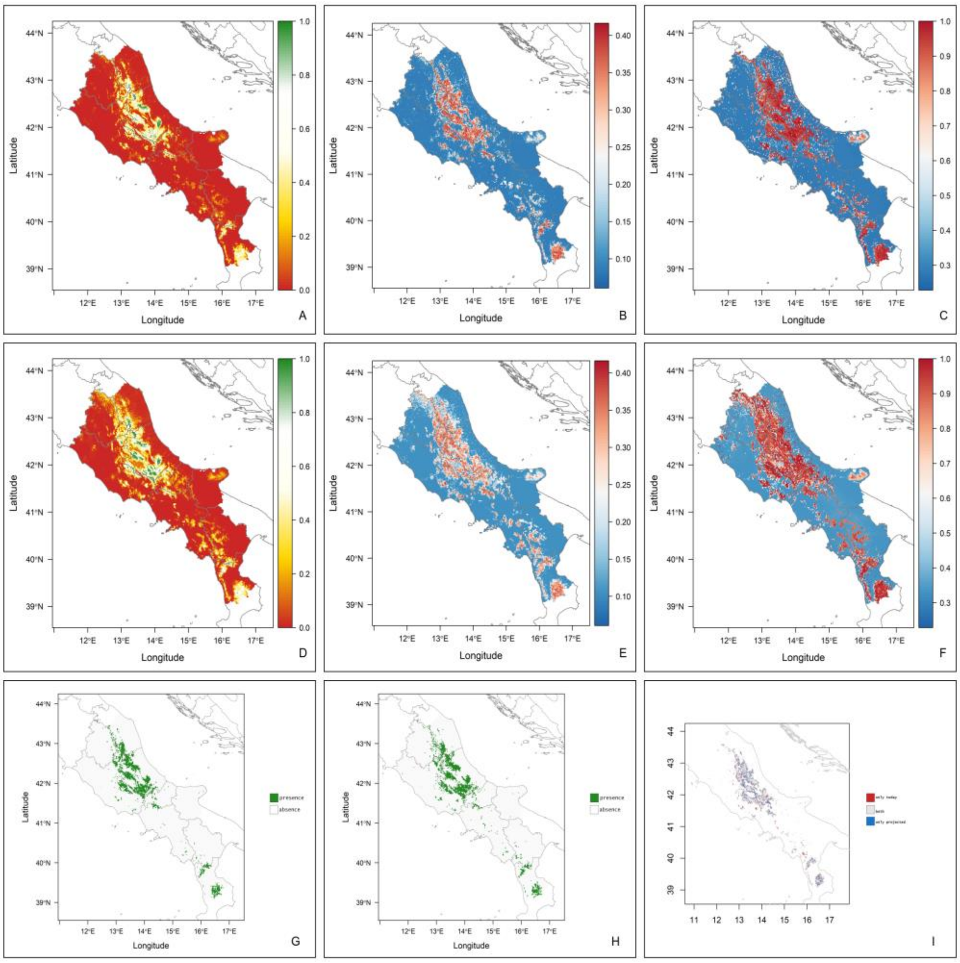

Fig. S.2.241-A) 'Projected' base output; B) 'Projected' uncertainty standard deviation; C) 'Projected' uncertainty range of values; D) 'Present' base output; E) 'Present' uncertainty standard deviation; F) 'Present' uncertainty range of values; G) 'Projected' binary distribution; H) 'Present' binary distribution; I) Range size variation.

Value	Present	Projected
AUC.train	0.987	0.991
AUC.test	0.936	0.952
AUC.diff	0.038	0.038
pROC	1.629	1.690
OR.p10	0.400	0.377
CBI	0.965	0.965
perc.stable	51.580	51.580
range.size	1.367	1.367

S.2.242 *Saxifraga porophylla* Bioclim

Species: *Saxifraga porophylla* Bertol. subsp. *porophylla*

Procedure: Bioclim

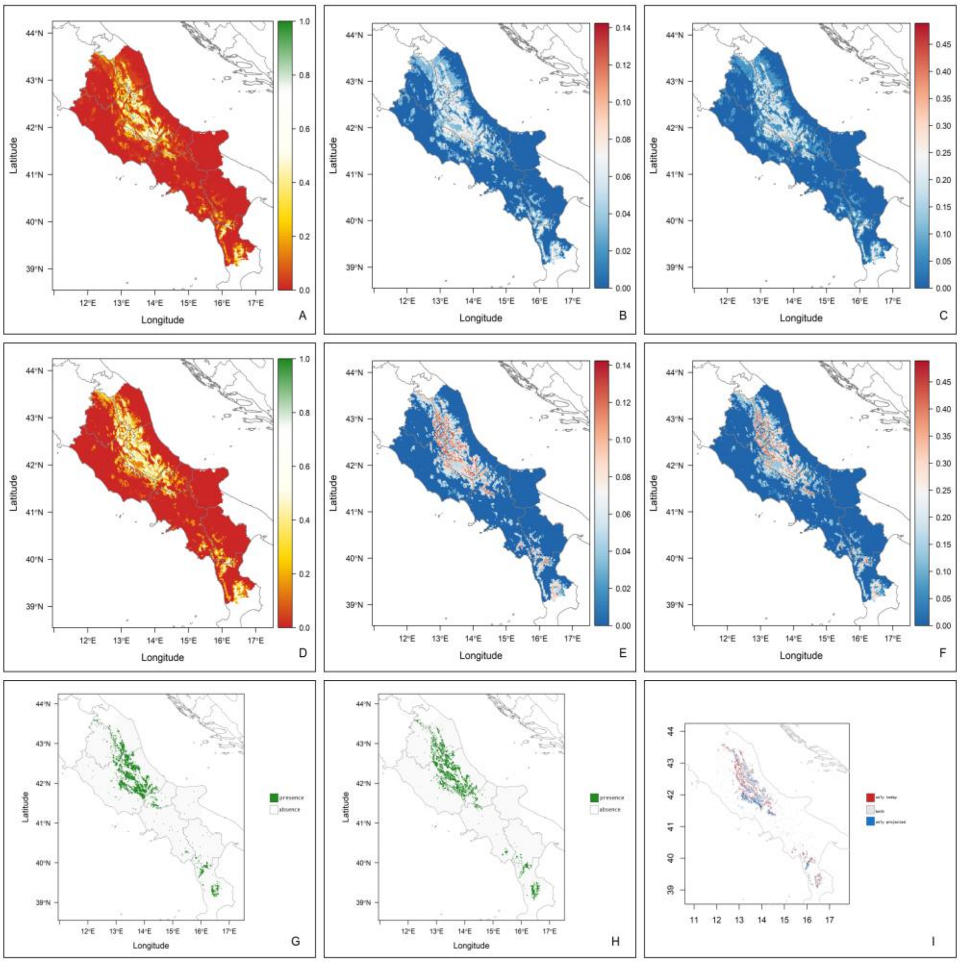

Fig. S.2.242-A) ‘Projected’ base output; B) ‘Projected’ uncertainty standard deviation; C) ‘Projected’ uncertainty range of values; D) ’Present’ base output; E) ’Present’ uncertainty standard deviation; F) ’Present’ uncertainty range of values; G) ‘Projected’ binary distribution; H) ‘Present’ binary distribution; I) Range size variation.

Value	Present	Projected
AUC.train	0.878	0.883
AUC.test	0.823	0.837
AUC.diff	0.048	0.048
pROC	1.326	1.306
OR.p10	0.131	0.238
CBI	0.249	0.922
perc.stable	47.868	47.868
range.size	-1.676	-1.676

S.2.243 *Saxifraga porophylla* Domain

Species: *Saxifraga porophylla* Bertol. subsp. *porophylla*

Procedure: Domain

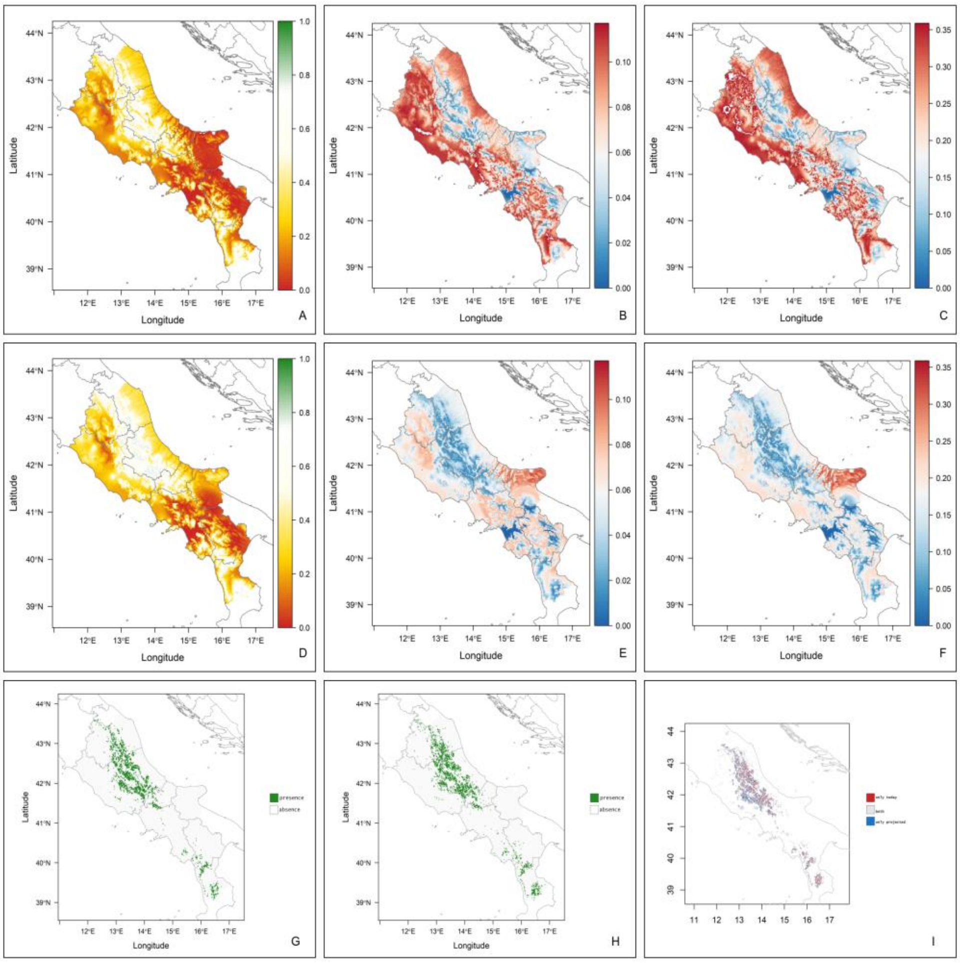

Fig. S.2.243-A) ‘Projected’ base output; B) ‘Projected’ uncertainty standard deviation; C) ‘Projected’ uncertainty range of values; D) ’Present’ base output; E) ’Present’ uncertainty standard deviation; F) ’Present’ uncertainty range of values; G) ‘Projected’ binary distribution; H) ‘Present’ binary distribution; I) Range size variation.

Value	Present	Projected
AUC.train	0.903	0.917
AUC.test	0.884	0.876
AUC.diff	0.042	0.042
pROC	1.544	1.538
OR.p10	0.185	0.231
CBI	0.979	0.975
perc.stable	36.821	36.821
range.size	-1.860	-1.860

S.2.244 *Saxifraga porophylla* Maxent

Species: *Saxifraga porophylla* Bertol. subsp. *porophylla*

Procedure: Maxent

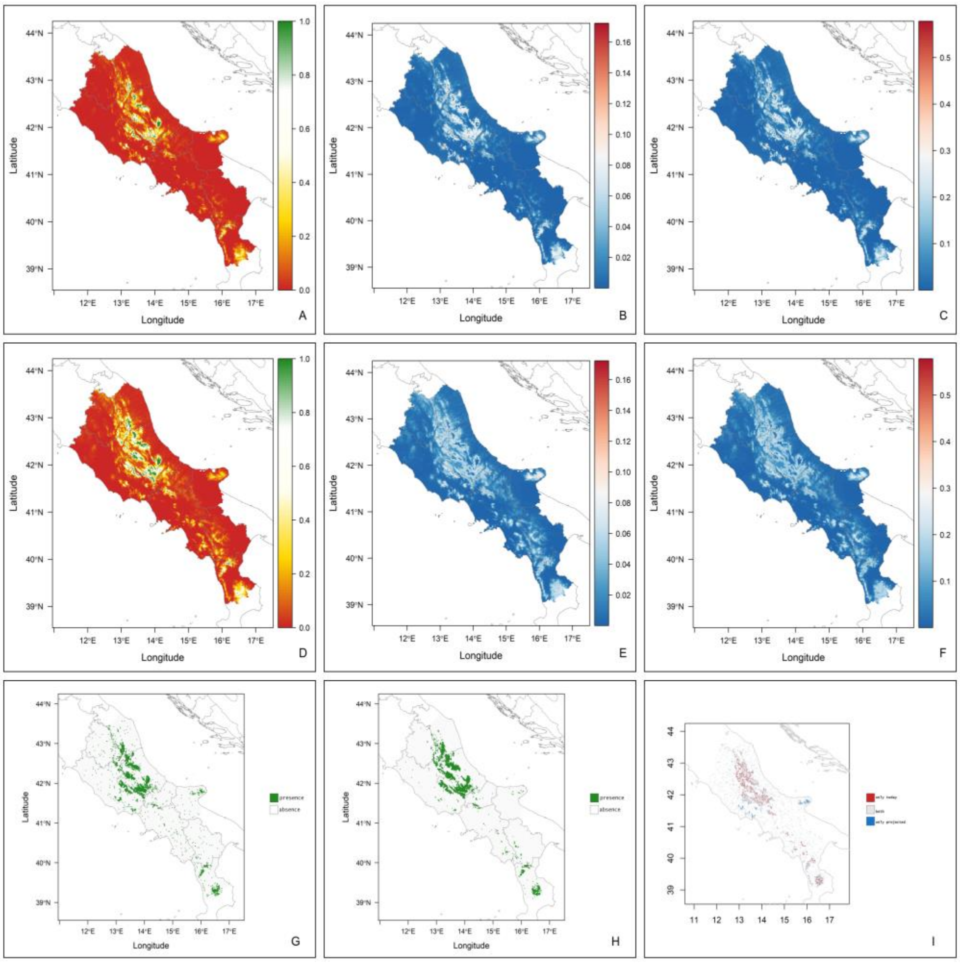

Fig. S.2.244-A) 'Projected' base output; B) 'Projected' uncertainty standard deviation; C) 'Projected' uncertainty range of values; D) 'Present' base output; E) 'Present' uncertainty standard deviation; F) 'Present' uncertainty range of values; G) 'Projected' binary distribution; H) 'Present' binary distribution; I) Range size variation.

Value	Present	Projected
AUC.train	0.930	0.929
AUC.test	0.938	0.962
AUC.diff	0.000	0.000
pROC	1.687	1.784
OR.p10	0.123	0.054
CBI	0.910	0.918
perc.stable	52.999	52.999
range.size	0.068	0.068

S.2.245 *Saxifraga porophylla* KDE

Species: *Saxifraga porophylla* Bertol. subsp. *porophylla*

Procedure: KDE

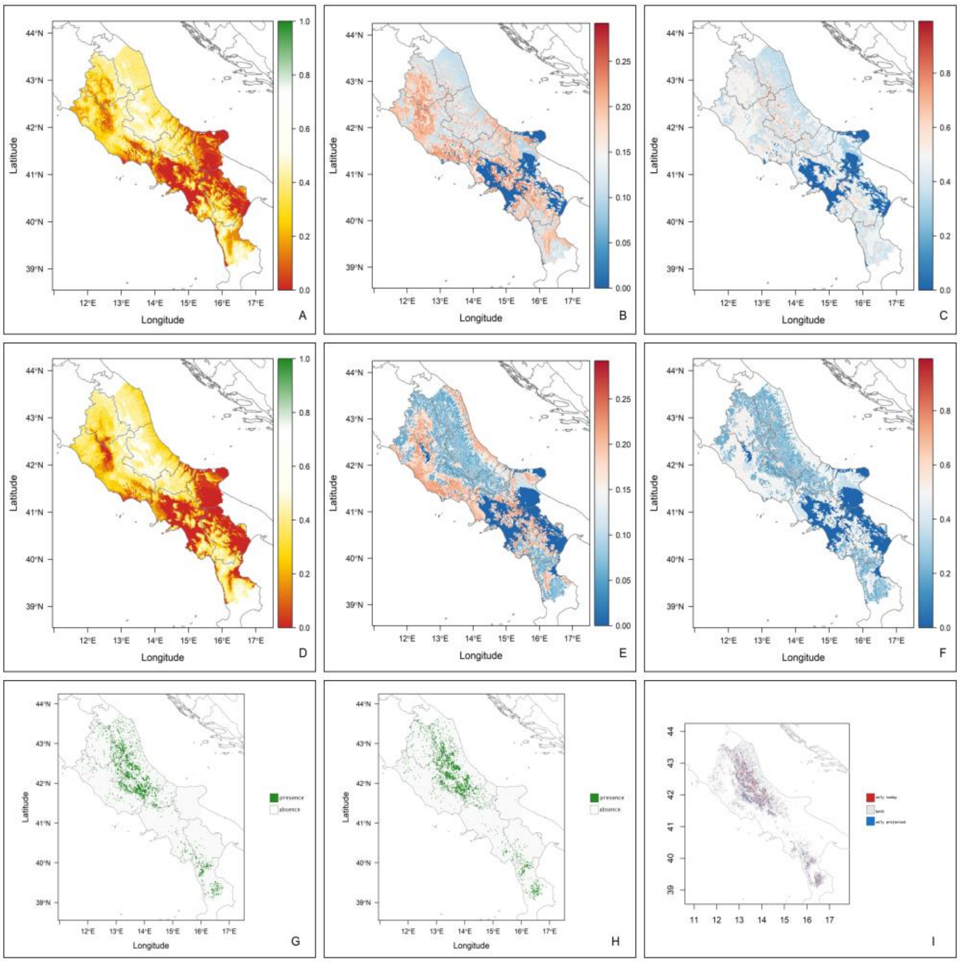

Fig. S.2.245-A) 'Projected' base output; B) 'Projected' uncertainty standard deviation; C) 'Projected' uncertainty range of values; D) 'Present' base output; E) 'Present' uncertainty standard deviation; F) 'Present' uncertainty range of values; G) 'Projected' binary distribution; H) 'Present' binary distribution; I) Range size variation.

Value	Present	Projected
AUC.train	0.914	0.929
AUC.test	0.892	0.881
AUC.diff	0.030	0.055
pROC	1.508	1.482
OR.p10	0.162	0.238
CBI	0.991	0.995
perc.stable	21.653	21.653
range.size	-0.784	-0.784

S.2.246 *Saxifraga porophylla* CTA

Species: *Saxifraga porophylla* Bertol. subsp. *porophylla*

Procedure: CTA

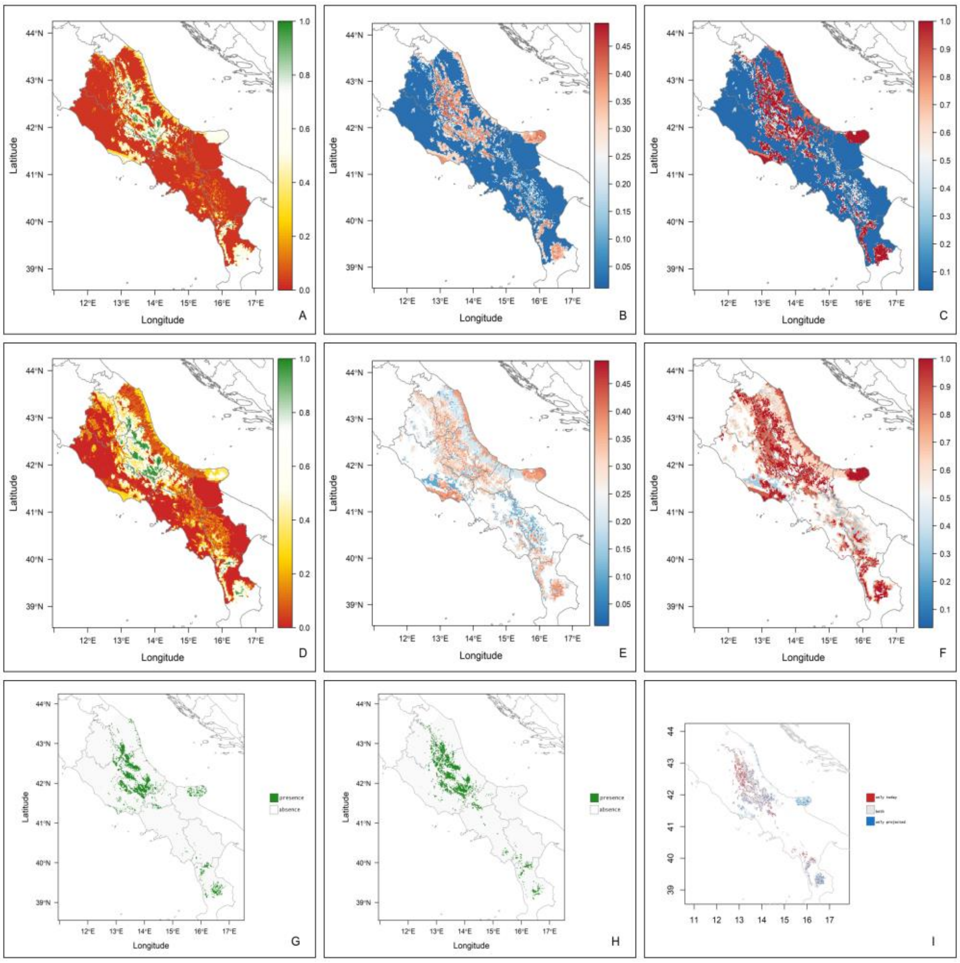

Fig. S.2.246-A) ‘Projected’ base output; B) ‘Projected’ uncertainty standard deviation; C) ‘Projected’ uncertainty range of values; D) ’Present’ base output; E) ’Present’ uncertainty standard deviation; F) ’Present’ uncertainty range of values; G) ‘Projected’ binary distribution; H) ‘Present’ binary distribution; I) Range size variation.

Value	Present	Projected
AUC.train	0.967	0.951
AUC.test	0.924	0.902
AUC.diff	0.051	0.051
pROC	1.316	1.316
OR.p10	0.177	0.177
CBI	0.879	0.892
perc.stable	41.445	41.445
range.size	-1.544	-1.544

S.2.247 *Saxifraga porophylla* FDA

Species: *Saxifraga porophylla* Bertol. subsp. *porophylla*

Procedure: FDA

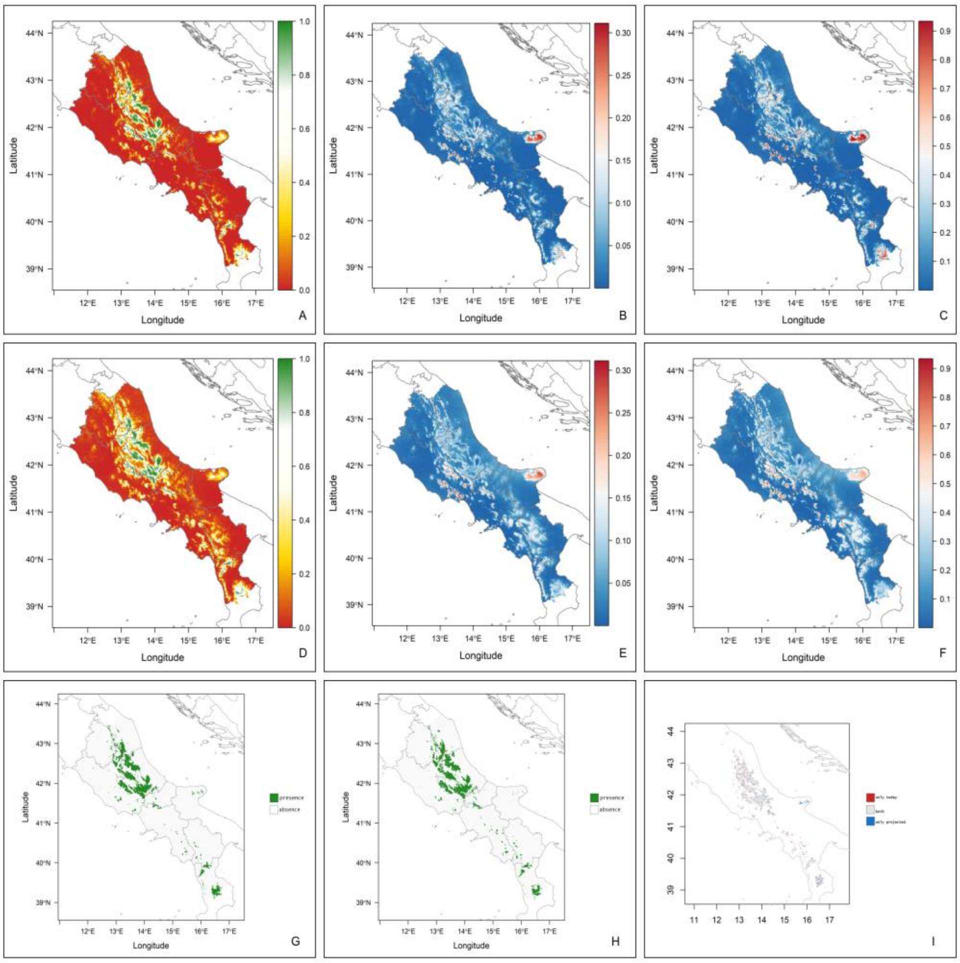

Fig. S.2.247-A) 'Projected' base output; B) 'Projected' uncertainty standard deviation; C) 'Projected' uncertainty range of values; D) 'Present' base output; E) 'Present' uncertainty standard deviation; F) 'Present' uncertainty range of values; G) 'Projected' binary distribution; H) 'Present' binary distribution; I) Range size variation.

Value	Present	Projected
AUC.train	0.931	0.951
AUC.test	0.935	0.942
AUC.diff	0.019	0.019
pROC	1.655	1.623
OR.p10	0.138	0.177
CBI	0.873	0.788
perc.stable	75.978	75.978
range.size	-0.172	-0.172

S.2.248 *Saxifraga porophylla* GAM

Species: *Saxifraga porophylla* Bertol. subsp. *porophylla*

Procedure: GAM

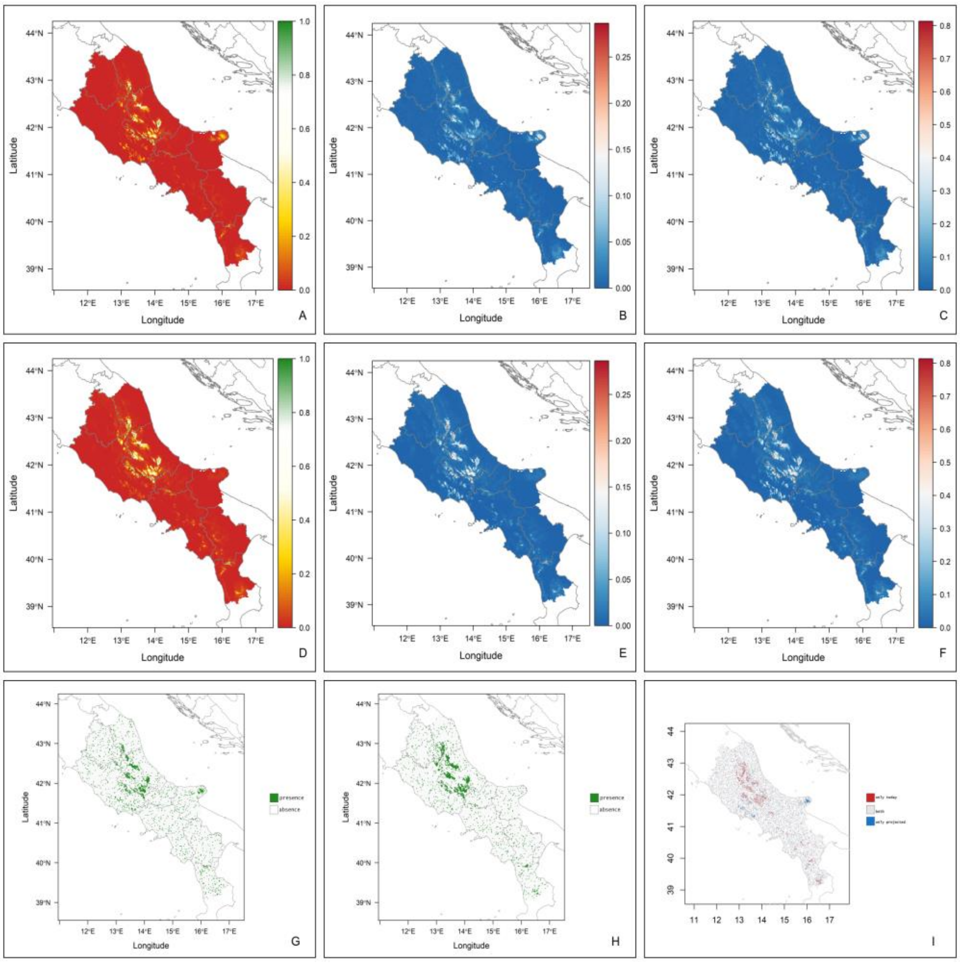

Fig. S.2.248-A) 'Projected' base output; B) 'Projected' uncertainty standard deviation; C) 'Projected' uncertainty range of values; D) 'Present' base output; E) 'Present' uncertainty standard deviation; F) 'Present' uncertainty range of values; G) 'Projected' binary distribution; H) 'Present' binary distribution; I) Range size variation.

Value	Present	Projected
AUC.train	0.942	0.926
AUC.test	0.883	0.841
AUC.diff	0.085	0.085
pROC	1.489	1.278
OR.p10	0.238	0.231
CBI	0.937	0.834
perc.stable	17.130	17.130
range.size	-0.738	-0.738

S.2.249 *Saxifraga porophylla* GLM

Species: *Saxifraga porophylla* Bertol. subsp. *porophylla*

Procedure: GLM

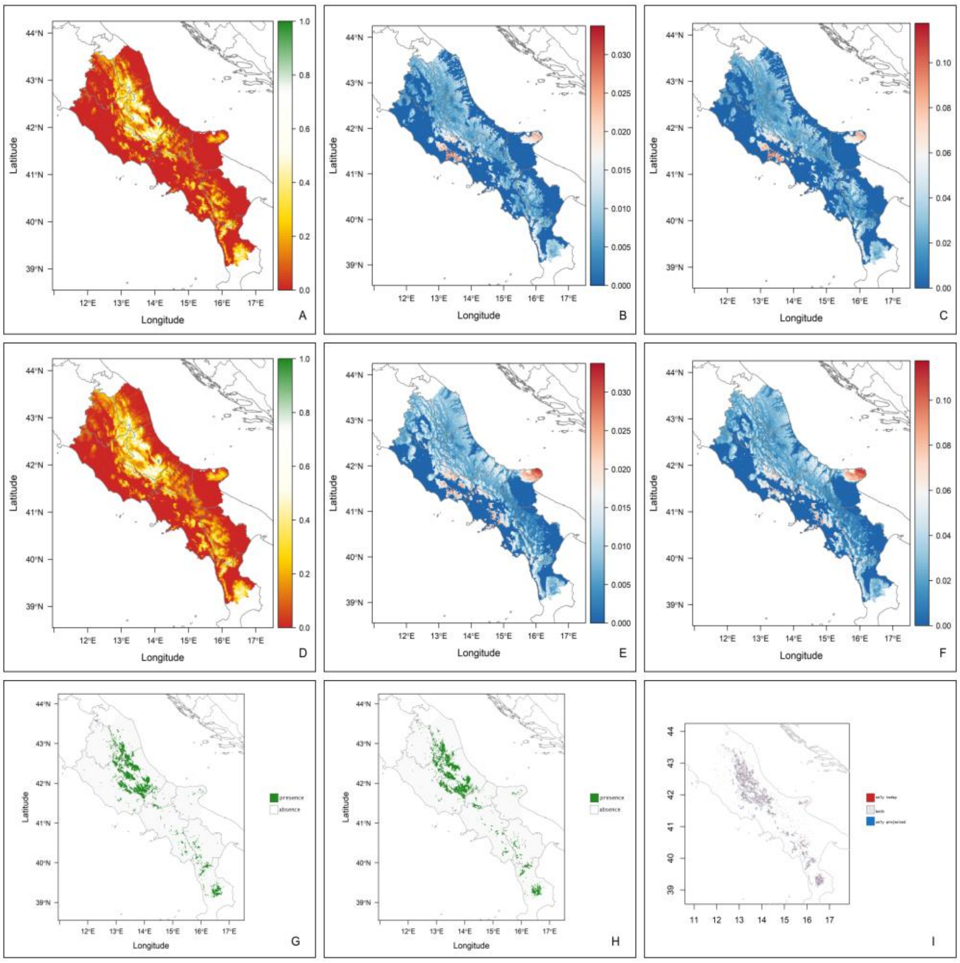

Fig. S.2.249-A) ‘Projected’ base output; B) ‘Projected’ uncertainty standard deviation; C) ‘Projected’ uncertainty range of values; D) ’Present’ base output; E) ’Present’ uncertainty standard deviation; F) ’Present’ uncertainty range of values; G) ‘Projected’ binary distribution; H) ‘Present’ binary distribution; I) Range size variation.

Value	Present	Projected
AUC.train	0.929	0.933
AUC.test	0.931	0.930
AUC.diff	0.023	0.023
pROC	1.674	1.618
OR.p10	0.123	0.154
CBI	0.918	0.924
perc.stable	49.170	49.170
range.size	1.057	1.057

S.2.250 *Saxifraga porophylla* MARS

Species: *Saxifraga porophylla* Bertol. subsp. *porophylla*

Procedure: MARS

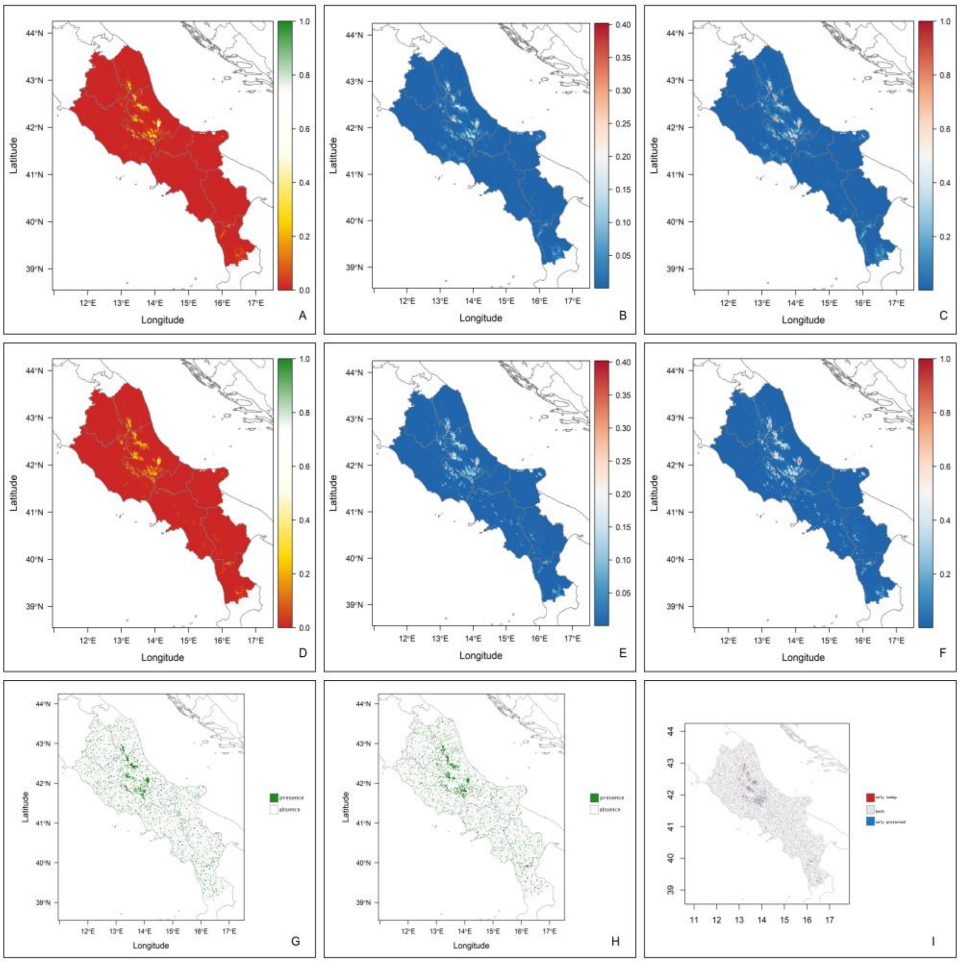

Fig. S.2.250-A) 'Projected' base output; B) 'Projected' uncertainty standard deviation; C) 'Projected' uncertainty range of values; D) 'Present' base output; E) 'Present' uncertainty standard deviation; F) 'Present' uncertainty range of values; G) 'Projected' binary distribution; H) 'Present' binary distribution; I) Range size variation.

Value	Present	Projected
AUC.train	0.925	0.946
AUC.test	0.862	0.871
AUC.diff	0.075	0.075
pROC	1.375	1.390
OR.p10	0.177	0.223
CBI	0.841	0.831
perc.stable	10.023	10.023
range.size	-2.744	-2.744

S.2.251 *Saxifraga porophylla* RF

Species: *Saxifraga porophylla* Bertol. subsp. *porophylla*

Procedure: RF

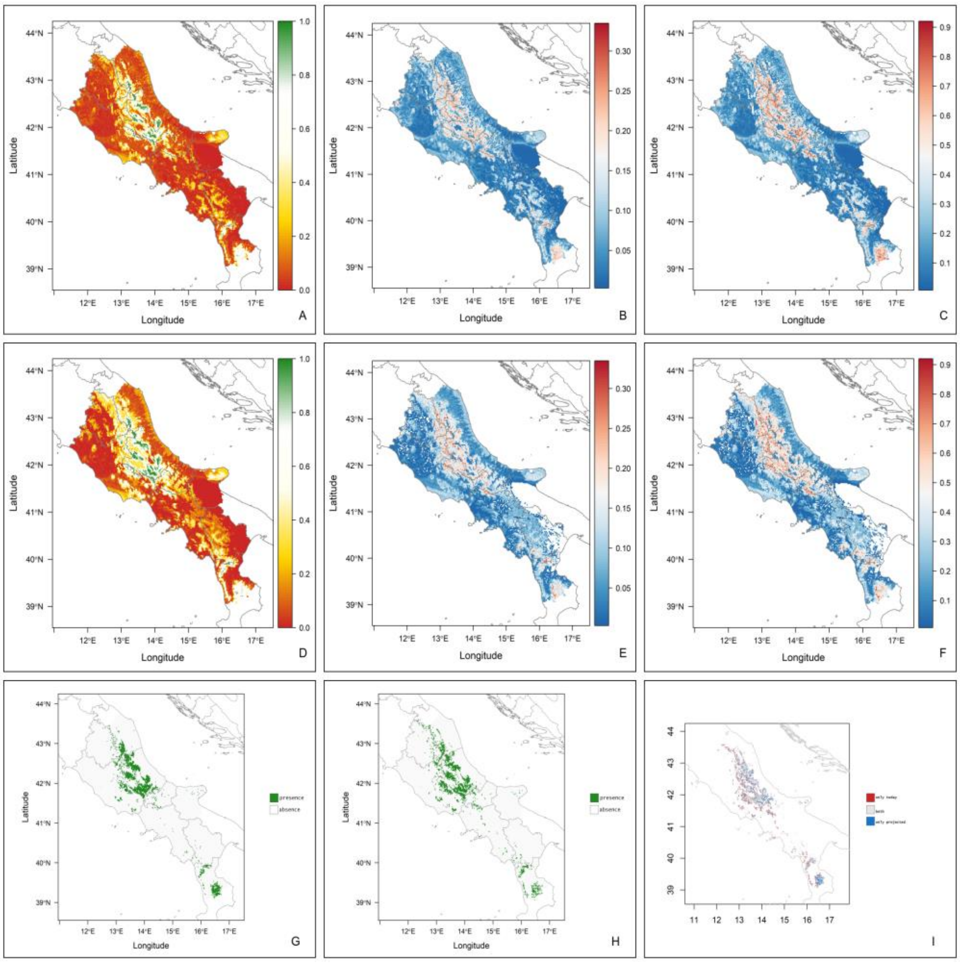

Fig. S.2.251-A) 'Projected' base output; B) 'Projected' uncertainty standard deviation; C) 'Projected' uncertainty range of values; D) 'Present' base output; E) 'Present' uncertainty standard deviation; F) 'Present' uncertainty range of values; G) 'Projected' binary distribution; H) 'Present' binary distribution; I) Range size variation.

Value	Present	Projected
AUC.train	1.000	1.000
AUC.test	0.987	0.923
AUC.diff	0.077	0.077
pROC	1.615	1.415
OR.p10	0.408	0.431
CBI	0.979	0.974
perc.stable	45.766	45.766
range.size	-1.823	-1.823

S.2.252 *Saxifraga porophylla* SVM

Species: *Saxifraga porophylla* Bertol. subsp. *porophylla*

Procedure: SVM

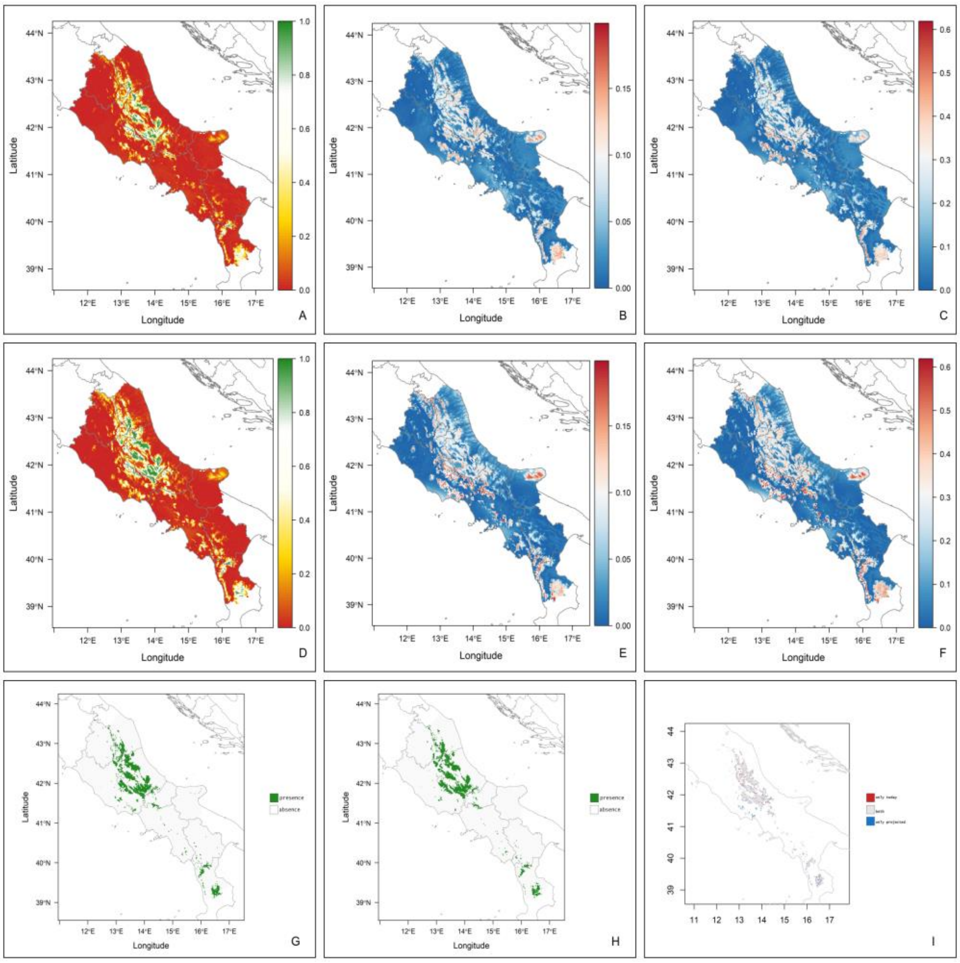

Fig. S.2.252-A) 'Projected' base output; B) 'Projected' uncertainty standard deviation; C) 'Projected' uncertainty range of values; D) 'Present' base output; E) 'Present' uncertainty standard deviation; F) 'Present' uncertainty range of values; G) 'Projected' binary distribution; H) 'Present' binary distribution; I) Range size variation.

Value	Present	Projected
AUC.train	0.933	0.943
AUC.test	0.932	0.932
AUC.diff	0.022	0.022
pROC	1.621	1.564
OR.p10	0.092	0.146
CBI	0.857	0.926
perc.stable	73.458	73.458
range.size	1.748	1.748

S.2.253 *Sedum magellense* Biomod2

Species: *Sedum magellense* Ten. subsp. *magellense*

Procedure: Biomod2

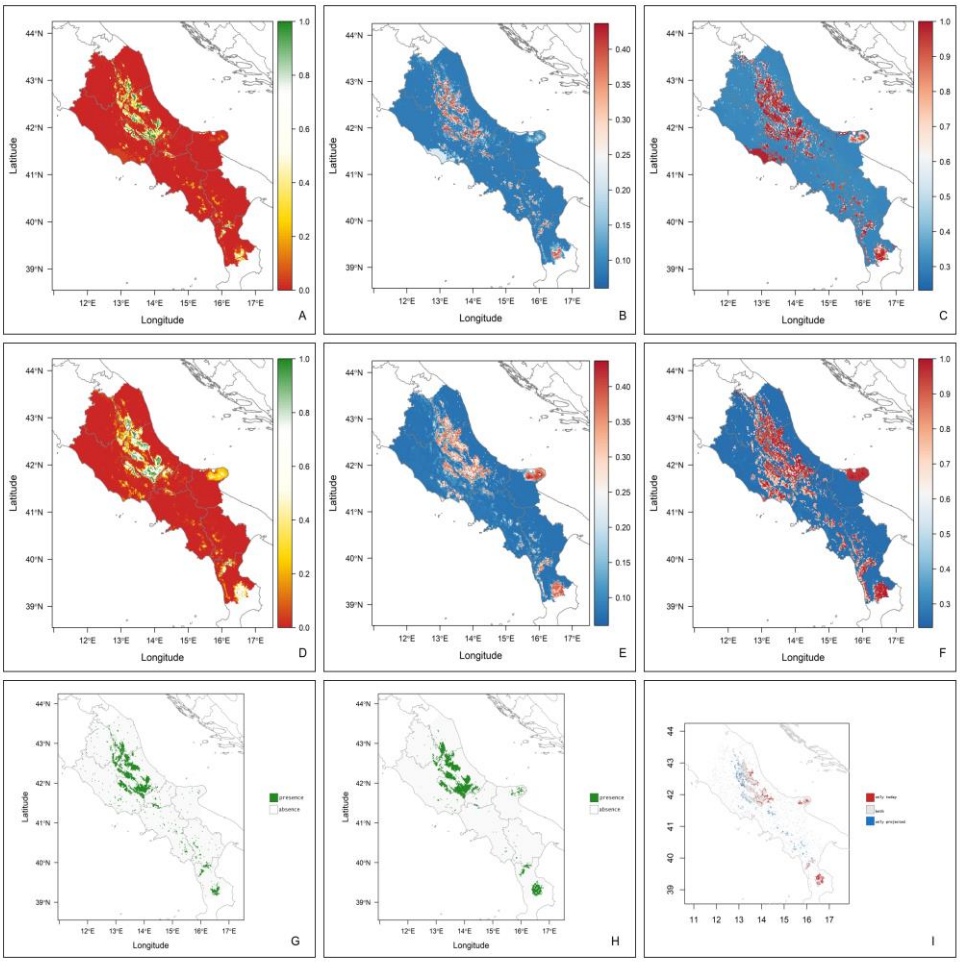

Fig. S.2.253-A) 'Projected' base output; B) 'Projected' uncertainty standard deviation; C) 'Projected' uncertainty range of values; D) 'Present' base output; E) 'Present' uncertainty standard deviation; F) 'Present' uncertainty range of values; G) 'Projected' binary distribution; H) 'Present' binary distribution; I) Range size variation.

Value	Present	Projected
AUC.train	0.993	0.995
AUC.test	0.939	0.909
AUC.diff	0.086	0.086
pROC	1.666	1.606
OR.p10	0.492	0.625
CBI	0.938	0.805
perc.stable	55.351	55.351
range.size	1.935	1.935

S.2.254 *Sedum magellense* Bioclim

Species: *Sedum magellense* Ten. subsp. *magellense*

Procedure: Bioclim

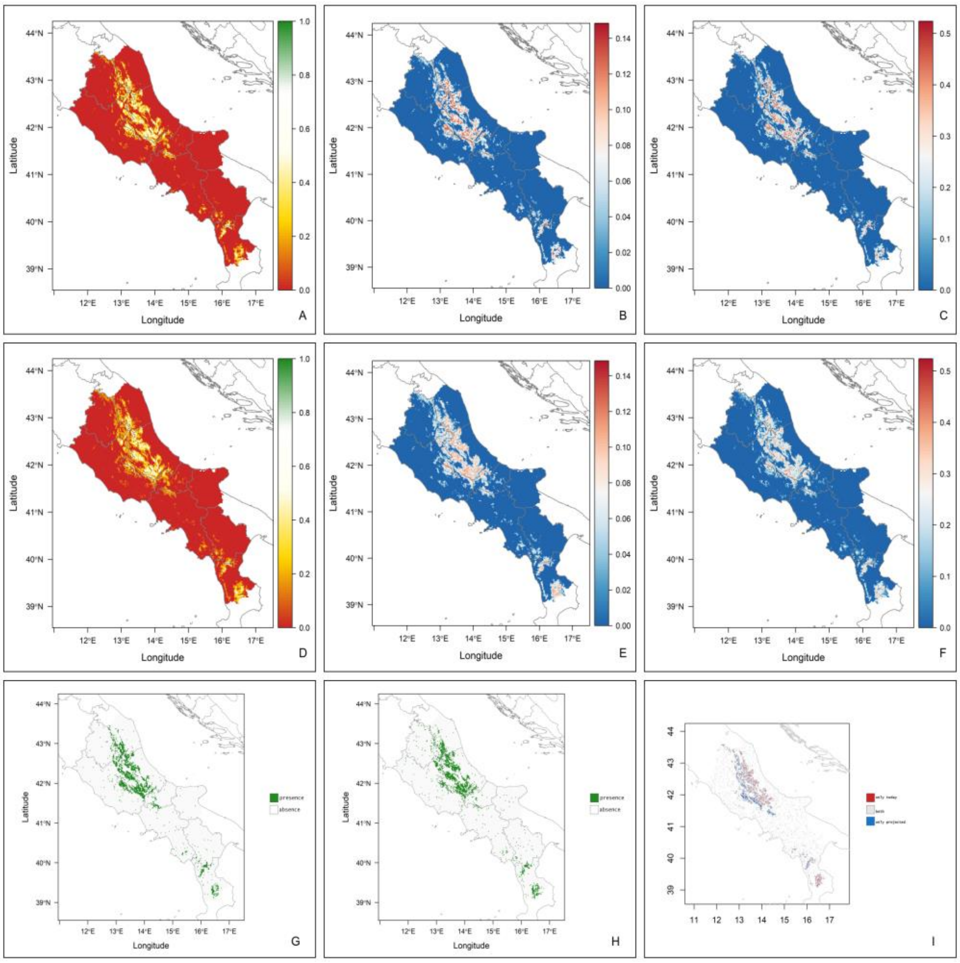

Fig. S.2.254-A) 'Projected' base output; B) 'Projected' uncertainty standard deviation; C) 'Projected' uncertainty range of values; D) 'Present' base output; E) 'Present' uncertainty standard deviation; F) 'Present' uncertainty range of values; G) 'Projected' binary distribution; H) 'Present' binary distribution; I) Range size variation.

Value	Present	Projected
AUC.train	0.931	0.923
AUC.test	0.835	0.870
AUC.diff	0.055	0.055
pROC	1.342	1.336
OR.p10	0.242	0.133
CBI	0.795	0.733
perc.stable	43.448	43.448
range.size	0.513	0.513

S.2.255 *Sedum magellense* Domain

Species: *Sedum magellense* Ten. subsp. *magellense*

Procedure: Domain

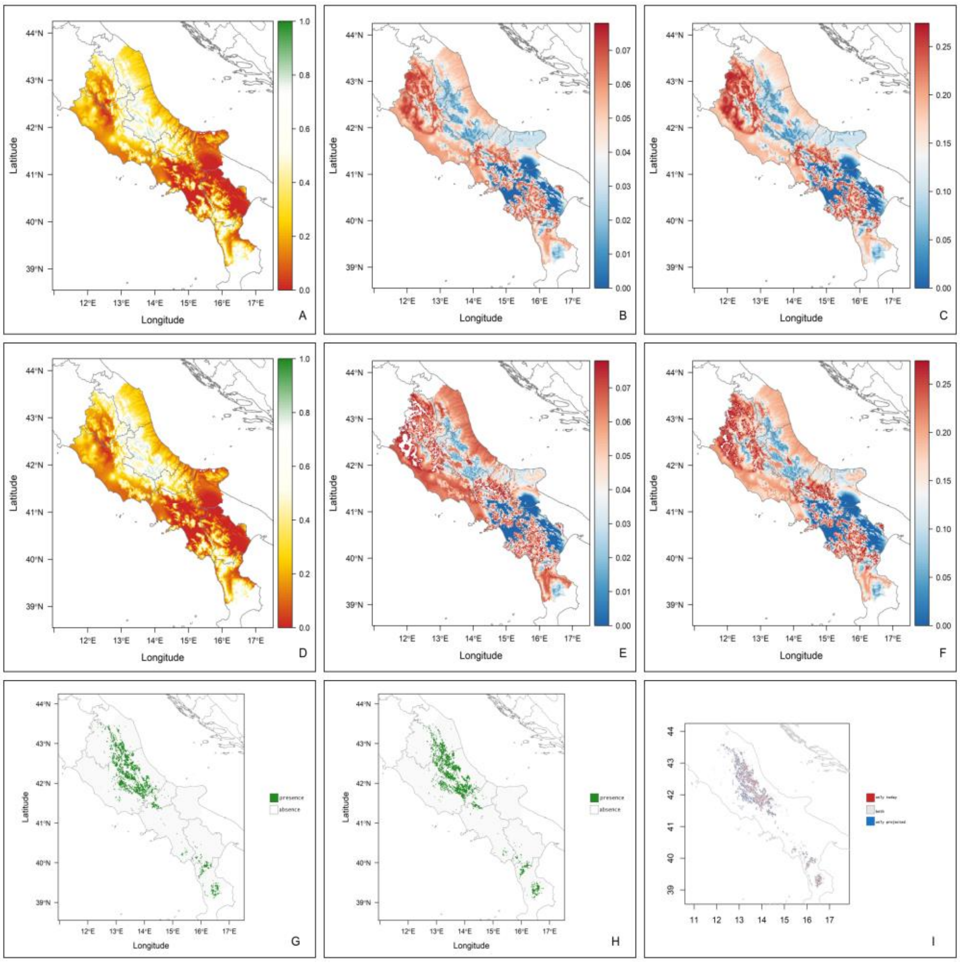

Fig. S.2.255-A) ‘Projected’ base output; B) ‘Projected’ uncertainty standard deviation; C) ‘Projected’ uncertainty range of values; D) ’Present’ base output; E) ’Present’ uncertainty standard deviation; F) ’Present’ uncertainty range of values; G) ‘Projected’ binary distribution; H) ‘Present’ binary distribution; I) Range size variation.

Value	Present	Projected
AUC.train	0.931	0.916
AUC.test	0.914	0.905
AUC.diff	0.019	0.019
pROC	1.671	1.656
OR.p10	0.200	0.150
CBI	0.910	0.961
perc.stable	45.750	45.750
range.size	1.103	1.103

S.2.256 *Sedum magellense* Maxent

Species: *Sedum magellense* Ten. subsp. *magellense*

Procedure: Maxent

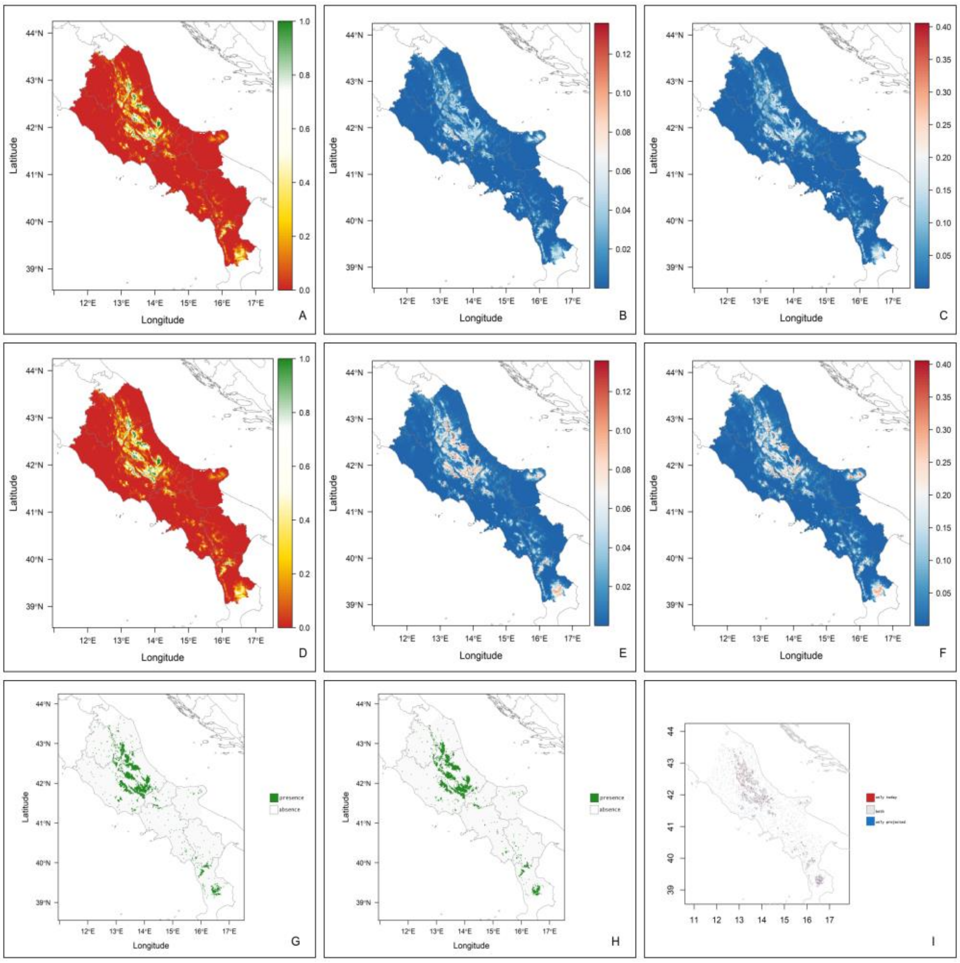

Fig. S.2.256-A) 'Projected' base output; B) 'Projected' uncertainty standard deviation; C) 'Projected' uncertainty range of values; D) 'Present' base output; E) 'Present' uncertainty standard deviation; F) 'Present' uncertainty range of values; G) 'Projected' binary distribution; H) 'Present' binary distribution; I) Range size variation.

Value	Present	Projected
AUC.train	0.958	0.958
AUC.test	0.962	0.942
AUC.diff	0.017	0.017
pROC	1.799	1.721
OR.p10	0.125	0.208
CBI	0.890	0.891
perc.stable	57.887	57.887
range.size	2.364	2.364

S.2.257 *Sedum magellense* KDE

Species: *Sedum magellense* Ten. subsp. *magellense*

Procedure: KDE

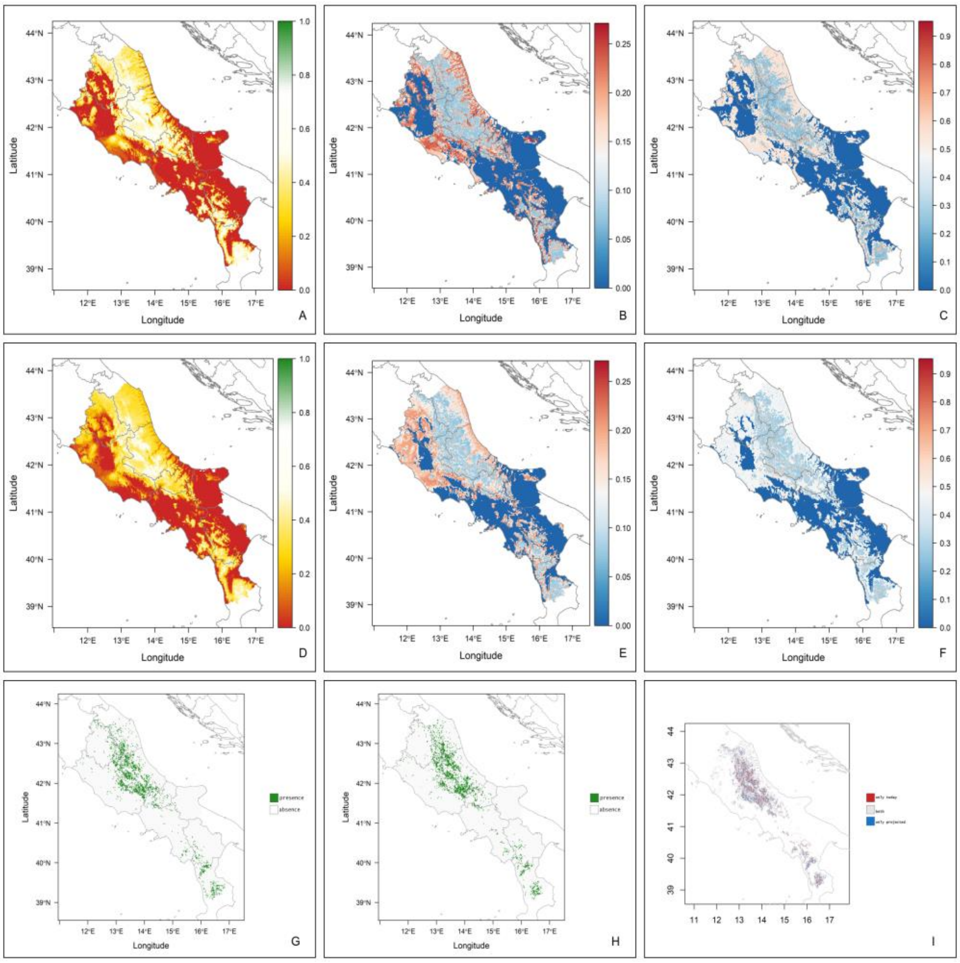

Fig. S.2.257-A) 'Projected' base output; B) 'Projected' uncertainty standard deviation; C) 'Projected' uncertainty range of values; D) 'Present' base output; E) 'Present' uncertainty standard deviation; F) 'Present' uncertainty range of values; G) 'Projected' binary distribution; H) 'Present' binary distribution; I) Range size variation.

Value	Present	Projected
AUC.train	0.936	0.913
AUC.test	0.908	0.896
AUC.diff	0.031	0.028
pROC	1.565	1.563
OR.p10	0.150	0.225
CBI	0.998	0.978
perc.stable	24.632	24.632
range.size	0.636	0.636

S.2.258 *Sedum magellense* CTA

Species: *Sedum magellense* Ten. subsp. *magellense*

Procedure: CTA

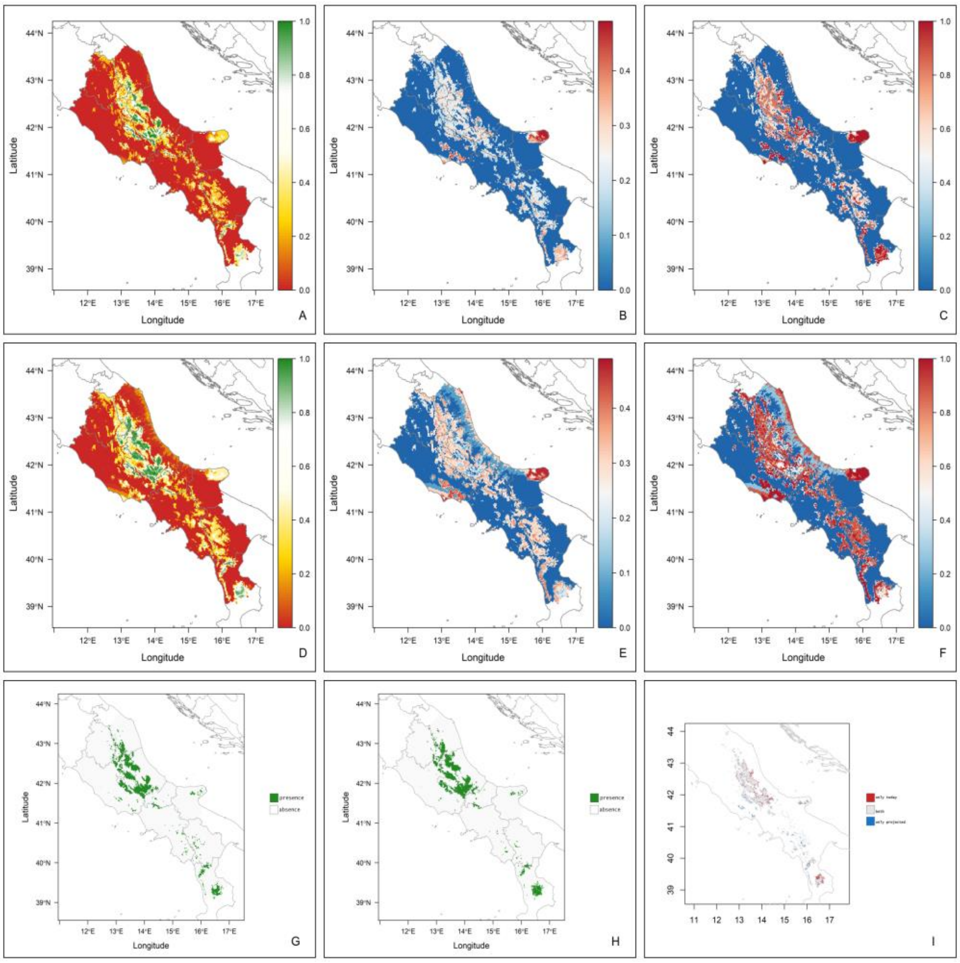

Fig. S.2.258-A) 'Projected' base output; B) 'Projected' uncertainty standard deviation; C) 'Projected' uncertainty range of values; D) 'Present' base output; E) 'Present' uncertainty standard deviation; F) 'Present' uncertainty range of values; G) 'Projected' binary distribution; H) 'Present' binary distribution; I) Range size variation.

Value	Present	Projected
AUC.train	0.978	0.970
AUC.test	0.947	0.900
AUC.diff	0.072	0.072
pROC	1.249	1.286
OR.p10	0.083	0.200
CBI	0.876	0.919
perc.stable	67.102	67.102
range.size	-0.447	-0.447

S.2.259 *Sedum magellense* FDA

Species: *Sedum magellense* Ten. subsp. *magellense*

Procedure: FDA

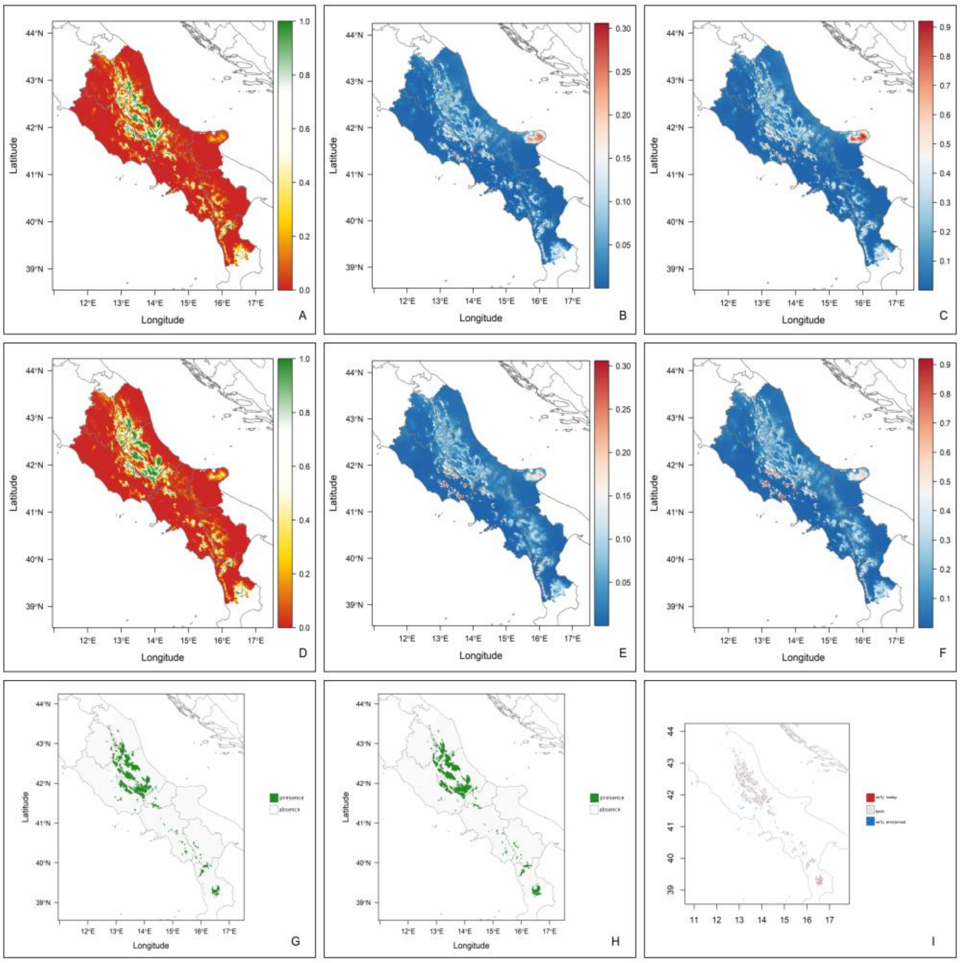

Fig. S.2.259-A) 'Projected' base output; B) 'Projected' uncertainty standard deviation; C) 'Projected' uncertainty range of values; D) 'Present' base output; E) 'Present' uncertainty standard deviation; F) 'Present' uncertainty range of values; G) 'Projected' binary distribution; H) 'Present' binary distribution; I) Range size variation.

Value	Present	Projected
AUC.train	0.968	0.959
AUC.test	0.957	0.948
AUC.diff	0.013	0.013
pROC	1.743	1.704
OR.p10	0.192	0.208
CBI	0.814	0.923
perc.stable	78.413	78.413
range.size	-1.444	-1.444

S.2.260 *Sedum magellense* GAM

Species: *Sedum magellense* Ten. subsp. *magellense*

Procedure: GAM

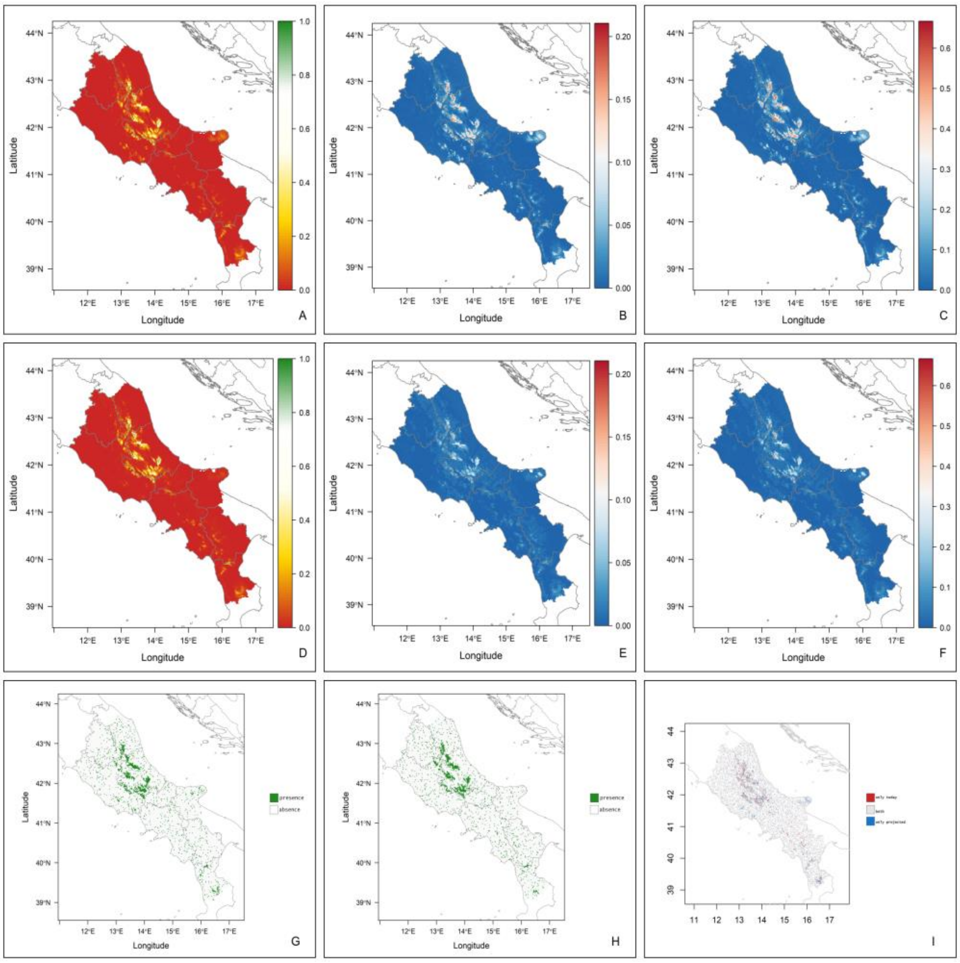

Fig. S.2.260-A) 'Projected' base output; B) 'Projected' uncertainty standard deviation; C) 'Projected' uncertainty range of values; D) 'Present' base output; E) 'Present' uncertainty standard deviation; F) 'Present' uncertainty range of values; G) 'Projected' binary distribution; H) 'Present' binary distribution; I) Range size variation.

Value	Present	Projected
AUC.train	0.960	0.943
AUC.test	0.952	0.936
AUC.diff	0.017	0.017
pROC	1.734	1.684
OR.p10	0.133	0.133
CBI	0.896	0.930
perc.stable	20.287	20.287
range.size	-0.102	-0.102

S.2.261 *Sedum magellense* GLM

Species: *Sedum magellense* Ten. subsp. *magellense*

Procedure: GLM

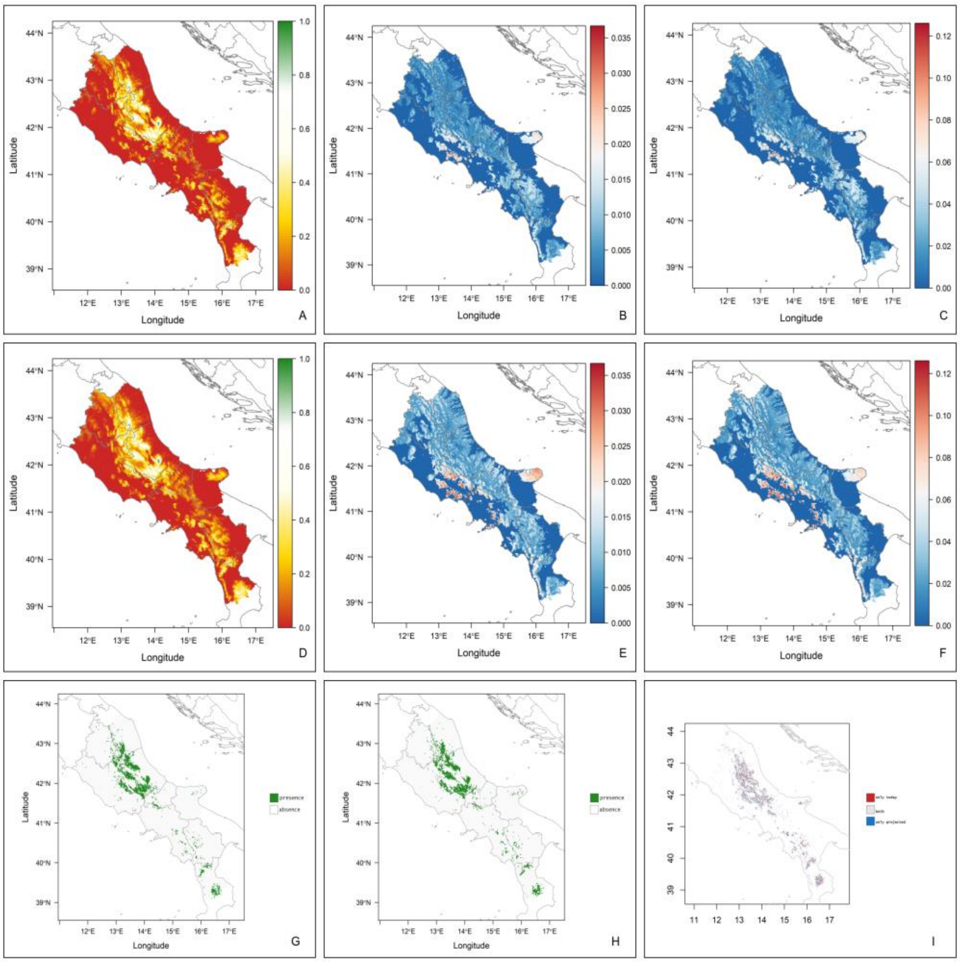

Fig. S.2.261-A) 'Projected' base output; B) 'Projected' uncertainty standard deviation; C) 'Projected' uncertainty range of values; D) 'Present' base output; E) 'Present' uncertainty standard deviation; F) 'Present' uncertainty range of values; G) 'Projected' binary distribution; H) 'Present' binary distribution; I) Range size variation.

Value	Present	Projected
AUC.train	0.959	0.949
AUC.test	0.960	0.960
AUC.diff	0.003	0.003
pROC	1.793	1.793
OR.p10	0.100	0.100
CBI	0.933	0.937
perc.stable	49.252	49.252
range.size	0.350	0.350

S.2.262 *Sedum magellense* MARS

Species: *Sedum magellense* Ten. subsp. *magellense*

Procedure: MARS

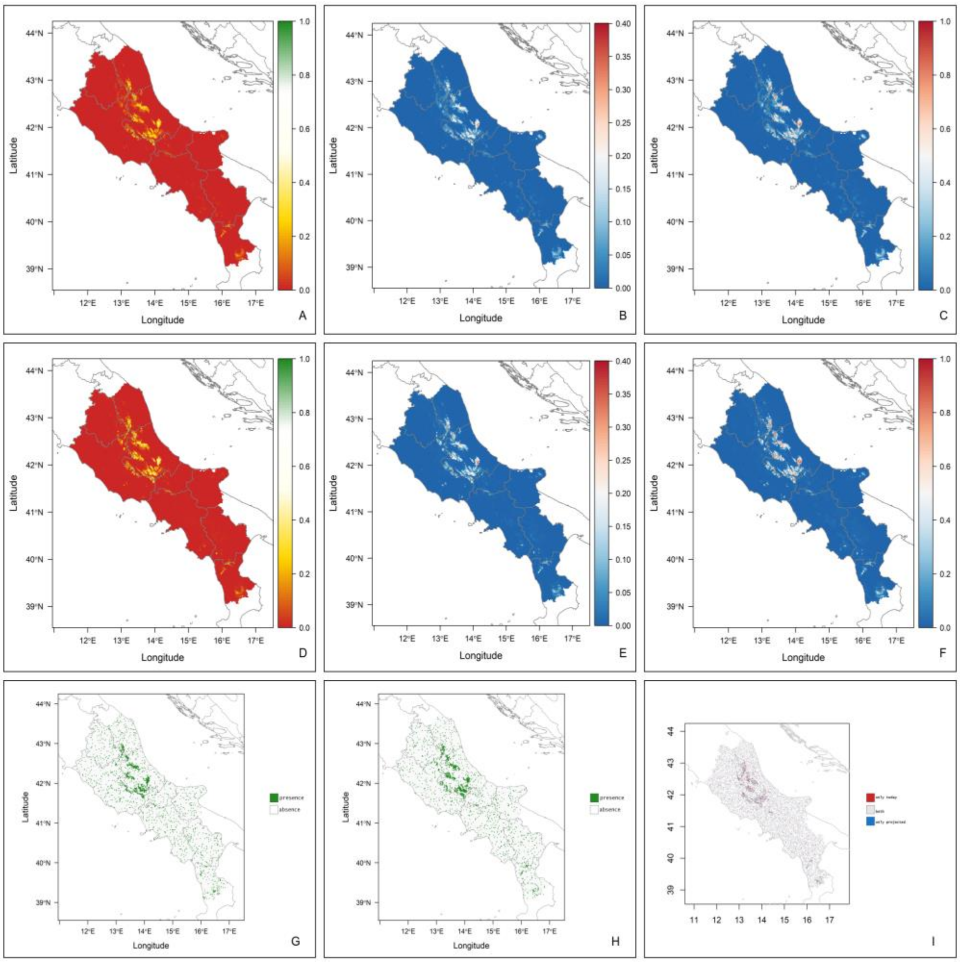

Fig. S.2.262-A) ‘Projected’ base output; B) ‘Projected’ uncertainty standard deviation; C) ‘Projected’ uncertainty range of values; D) ’Present’ base output; E) ’Present’ uncertainty standard deviation; F) ’Present’ uncertainty range of values; G) ‘Projected’ binary distribution; H) ‘Present’ binary distribution; I) Range size variation.

Value	Present	Projected
AUC.train	0.942	0.959
AUC.test	0.902	0.874
AUC.diff	0.087	0.087
pROC	1.525	1.458
OR.p10	0.167	0.283
CBI	0.959	0.863
perc.stable	14.506	14.506
range.size	-2.283	-2.283

S.2.263 *Sedum magellense* RF

Species: *Sedum magellense* Ten. subsp. *magellense*

Procedure: RF

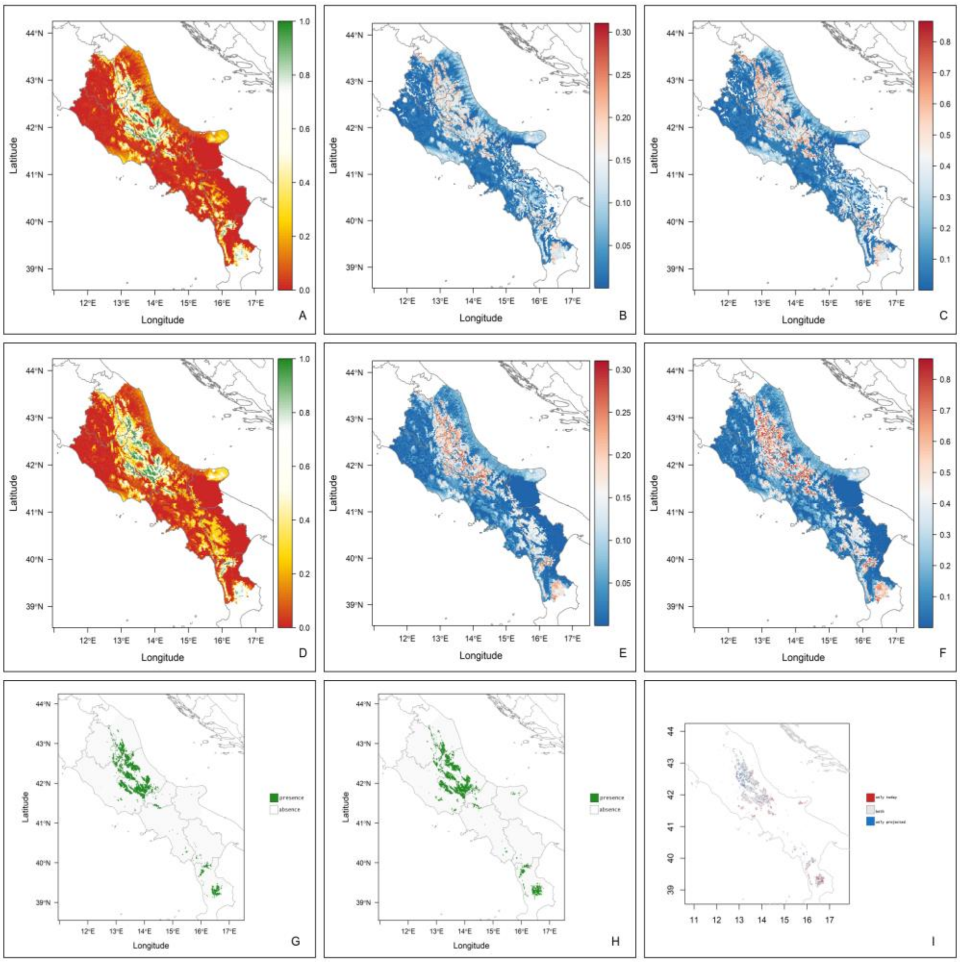

Fig. S.2.263-A) 'Projected' base output; B) 'Projected' uncertainty standard deviation; C) 'Projected' uncertainty range of values; D) 'Present' base output; E) 'Present' uncertainty standard deviation; F) 'Present' uncertainty range of values; G) 'Projected' binary distribution; H) 'Present' binary distribution; I) Range size variation.

Value	Present	Projected
AUC.train	1.000	1.000
AUC.test	0.976	0.979
AUC.diff	0.021	0.021
pROC	1.652	1.678
OR.p10	0.308	0.358
CBI	0.778	0.913
perc.stable	64.582	64.582
range.size	-0.743	-0.743

S.2.264 *Sedum magellense* SVM

Species: *Sedum magellense* Ten. subsp. *magellense*

Procedure: SVM

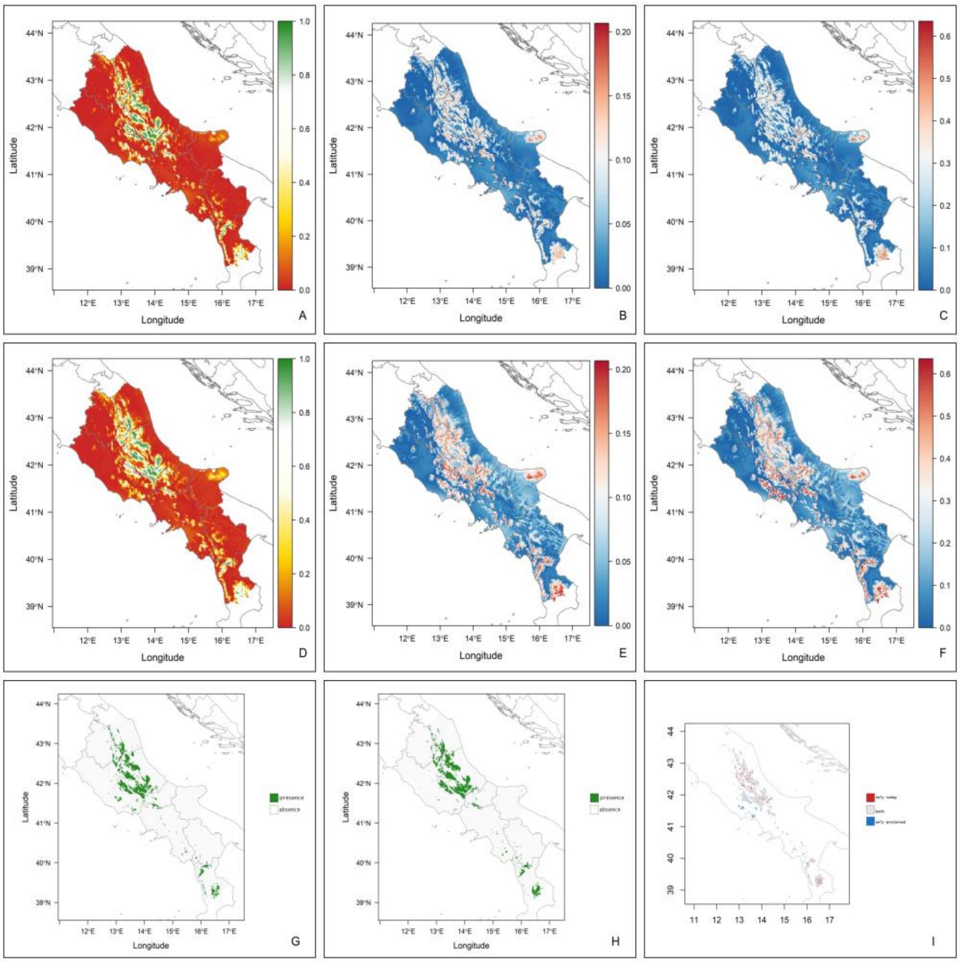

Fig. S.2.264-A) 'Projected' base output; B) 'Projected' uncertainty standard deviation; C) 'Projected' uncertainty range of values; D) 'Present' base output; E) 'Present' uncertainty standard deviation; F) 'Present' uncertainty range of values; G) 'Projected' binary distribution; H) 'Present' binary distribution; I) Range size variation.

Value	Present	Projected
AUC.train	0.963	0.968
AUC.test	0.965	0.955
AUC.diff	0.014	0.014
pROC	1.763	1.704
OR.p10	0.075	0.192
CBI	0.926	0.921
perc.stable	71.390	71.390
range.size	-1.869	-1.869

S.2.265 *Siler montanum* Biomod2

Species: *Siler montanum* Crantz subsp. *siculum* (Spreng.) Iamonico, Bartolucci & F.Conti

Procedure: Biomod2

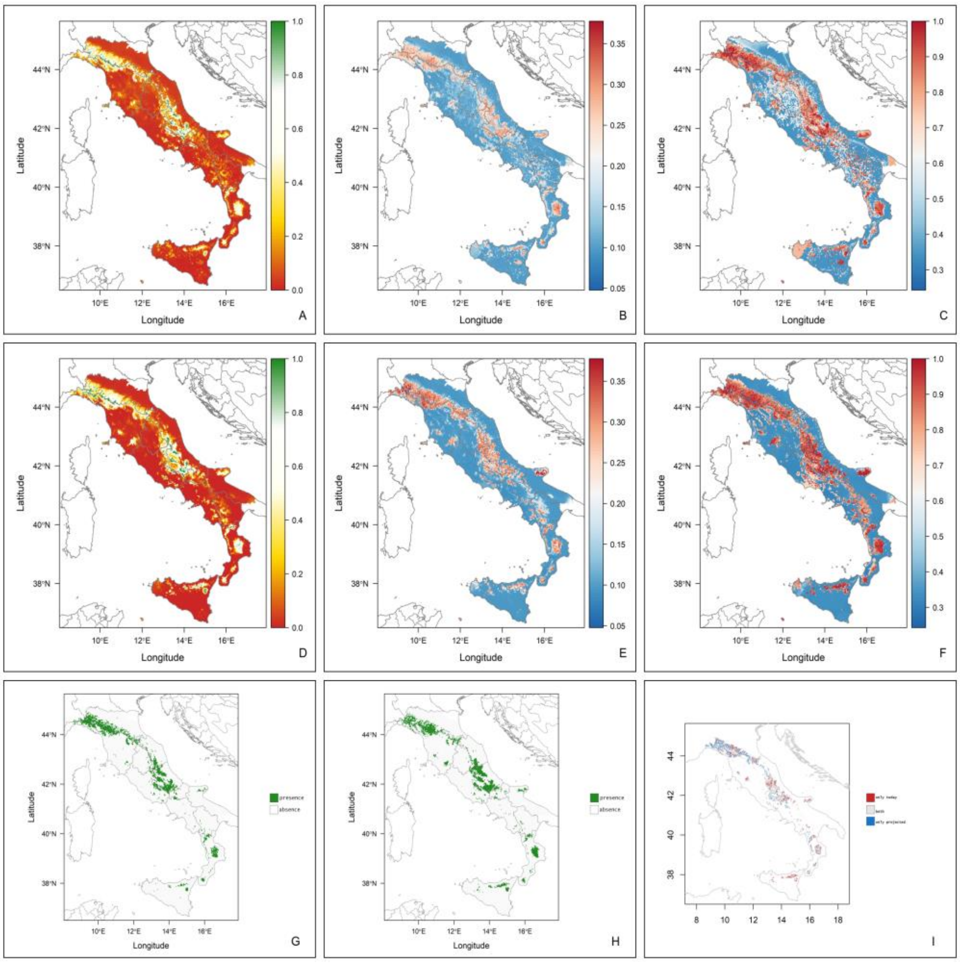

Fig. S.2.265-A) 'Projected' base output; B) 'Projected' uncertainty standard deviation; C) 'Projected' uncertainty range of values; D) 'Present' base output; E) 'Present' uncertainty standard deviation; F) 'Present' uncertainty range of values; G) 'Projected' binary distribution; H) 'Present' binary distribution; I) Range size variation.

Value	Present	Projected
AUC.train	0.981	0.964
AUC.test	0.936	0.846
AUC.diff	0.119	0.119
pROC	1.632	1.441
OR.p10	0.328	0.500
CBI	0.981	0.944
perc.stable	57.070	57.070
range.size	0.186	0.186

S.2.266 *Siler montanum* Bioclim

Species: *Siler montanum* Crantz subsp. *siculum* (Spreng.) Iamonico, Bartolucci & F.Conti

Procedure: Bioclim

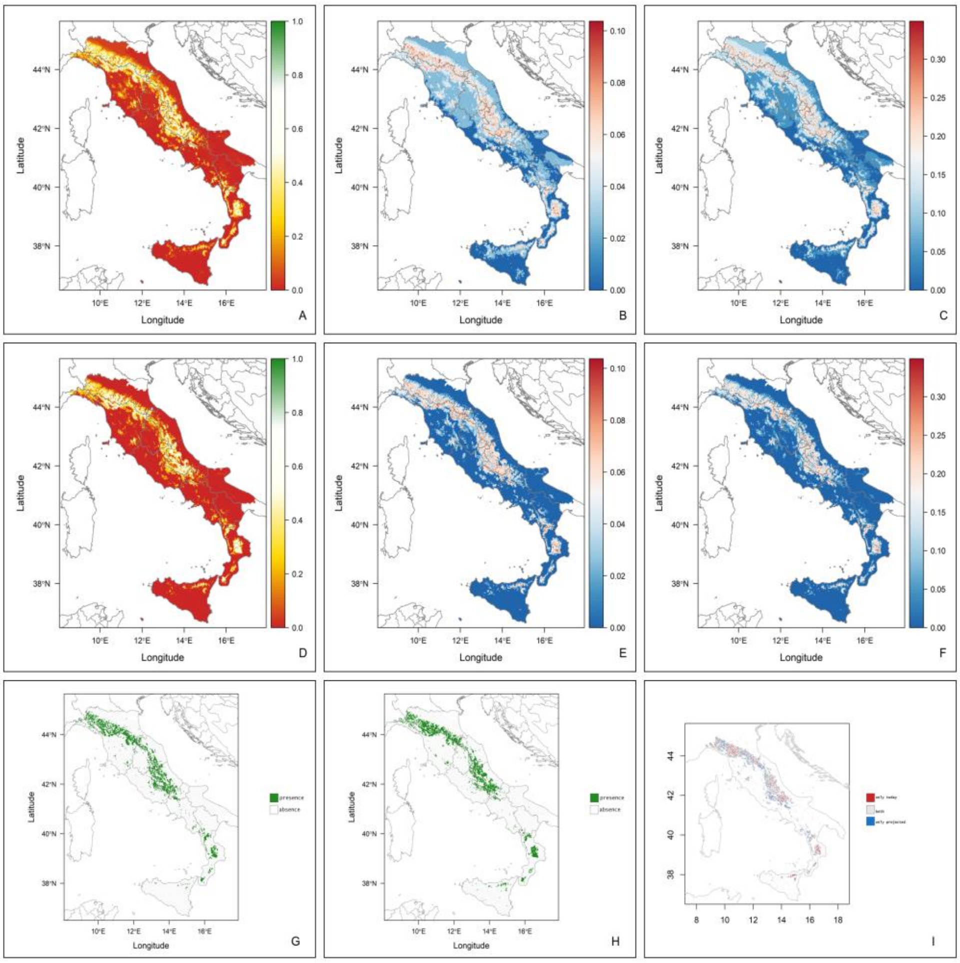

Fig. S.2.266-A) 'Projected' base output; B) 'Projected' uncertainty standard deviation; C) 'Projected' uncertainty range of values; D) 'Present' base output; E) 'Present' uncertainty standard deviation; F) 'Present' uncertainty range of values; G) 'Projected' binary distribution; H) 'Present' binary distribution; I) Range size variation.

Value	Present	Projected
AUC.train	0.870	0.787
AUC.test	0.827	0.724
AUC.diff	0.063	0.063
pROC	1.285	1.143
OR.p10	0.122	0.206
CBI	0.605	0.449
perc.stable	57.584	57.584
range.size	0.648	0.648

S.2.267 *Siler montanum* Domain

Species: *Siler montanum* Crantz subsp. *siculum* (Spreng.) Iamonico, Bartolucci & F.Conti

Procedure: Domain

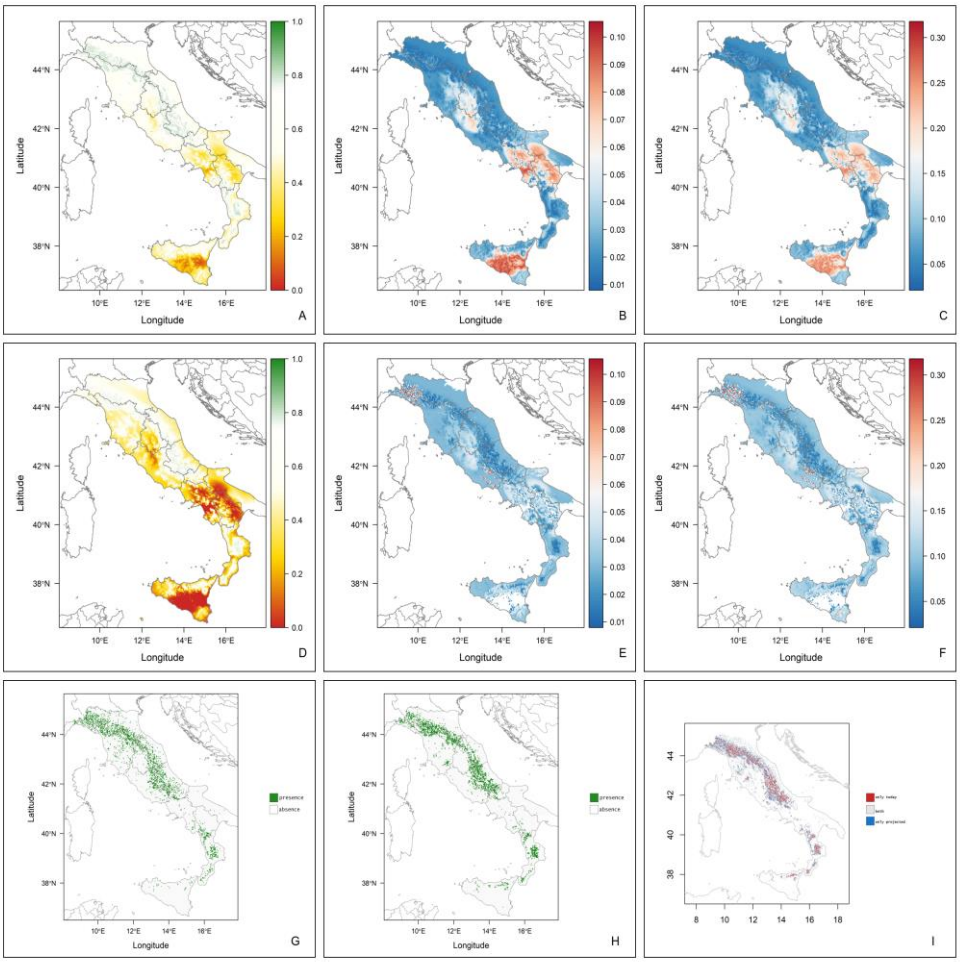

Fig. S.2.267-A) 'Projected' base output; B) 'Projected' uncertainty standard deviation; C) 'Projected' uncertainty range of values; D) 'Present' base output; E) 'Present' uncertainty standard deviation; F) 'Present' uncertainty range of values; G) 'Projected' binary distribution; H) 'Present' binary distribution; I) Range size variation.

Value	Present	Projected
AUC.train	0.822	0.744
AUC.test	0.792	0.730
AUC.diff	0.029	0.029
pROC	1.379	1.220
OR.p10	0.156	0.156
CBI	0.988	0.979
perc.stable	26.709	26.709
range.size	0.060	0.060

S.2.268 *Siler montanum* Maxent

Species: *Siler montanum* Crantz subsp. *siculum* (Spreng.) Iamonico, Bartolucci & F.Conti

Procedure: Maxent

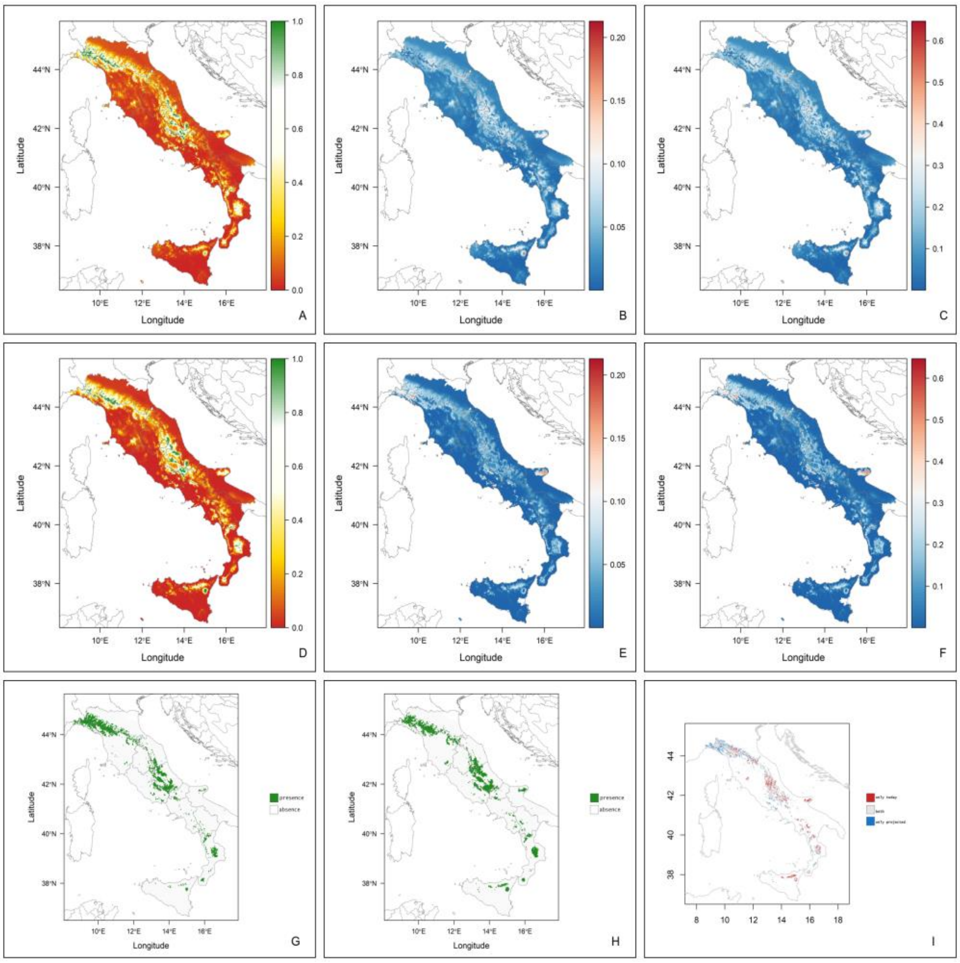

Fig. S.2.268-A) 'Projected' base output; B) 'Projected' uncertainty standard deviation; C) 'Projected' uncertainty range of values; D) 'Present' base output; E) 'Present' uncertainty standard deviation; F) 'Present' uncertainty range of values; G) 'Projected' binary distribution; H) 'Present' binary distribution; I) Range size variation.

Value	Present	Projected
AUC.train	0.924	0.876
AUC.test	0.925	0.852
AUC.diff	0.040	0.040
pROC	1.668	1.369
OR.p10	0.106	0.206
CBI	0.980	0.944
perc.stable	61.406	61.406
range.size	-2.141	-2.141

S.2.269 *Siler montanum* KDE

Species: *Siler montanum* Crantz subsp. *siculum* (Spreng.) Iamonico, Bartolucci & F.Conti

Procedure: KDE

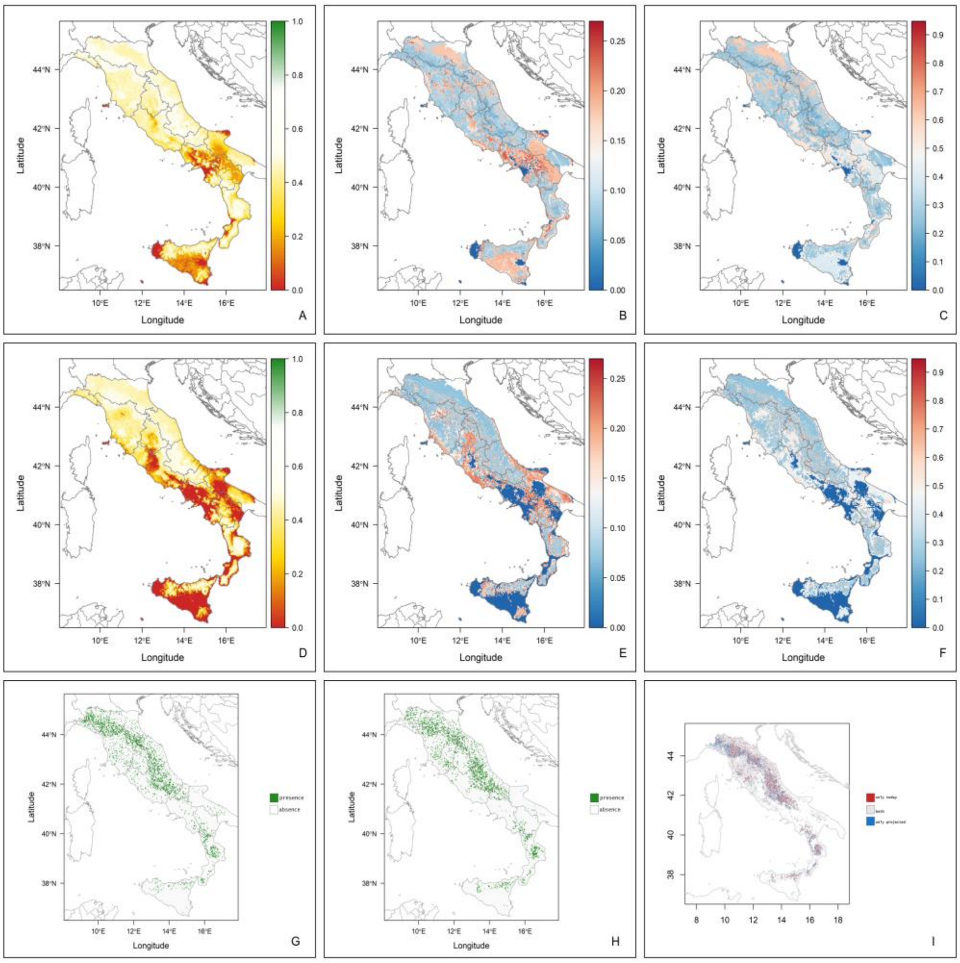

Fig. S.2.269-A) 'Projected' base output; B) 'Projected' uncertainty standard deviation; C) 'Projected' uncertainty range of values; D) 'Present' base output; E) 'Present' uncertainty standard deviation; F) 'Present' uncertainty range of values; G) 'Projected' binary distribution; H) 'Present' binary distribution; I) Range size variation.

Value	Present	Projected
AUC.train	0.832	0.789
AUC.test	0.799	0.769
AUC.diff	0.038	0.037
pROC	1.308	1.215
OR.p10	0.167	0.161
CBI	0.984	0.978
perc.stable	14.436	14.436
range.size	0.639	0.639

S.2.270 *Siler montanum* CTA

Species: *Siler montanum* Crantz subsp. *siculum* (Spreng.) Iamonico, Bartolucci & F.Conti

Procedure: CTA

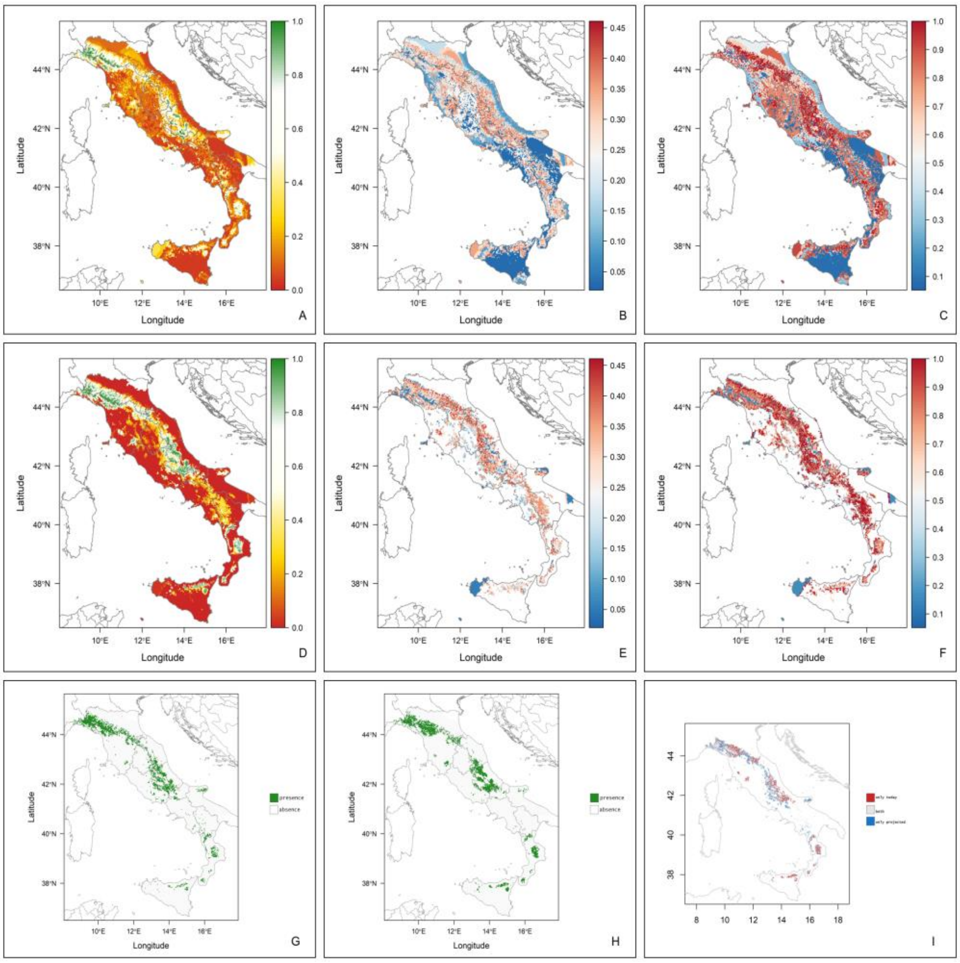

Fig. S.2.270-A) 'Projected' base output; B) 'Projected' uncertainty standard deviation; C) 'Projected' uncertainty range of values; D) 'Present' base output; E) 'Present' uncertainty standard deviation; F) 'Present' uncertainty range of values; G) 'Projected' binary distribution; H) 'Present' binary distribution; I) Range size variation.

Value	Present	Projected
AUC.train	0.964	0.934
AUC.test	0.915	0.805
AUC.diff	0.129	0.129
pROC	1.325	1.097
OR.p10	0.228	0.289
CBI	0.962	0.889
perc.stable	46.357	46.357
range.size	-1.263	-1.263

S.2.271 *Siler montanum* FDA

Species: *Siler montanum* Crantz subsp. *siculum* (Spreng.) Iamonico, Bartolucci & F.Conti

Procedure: FDA

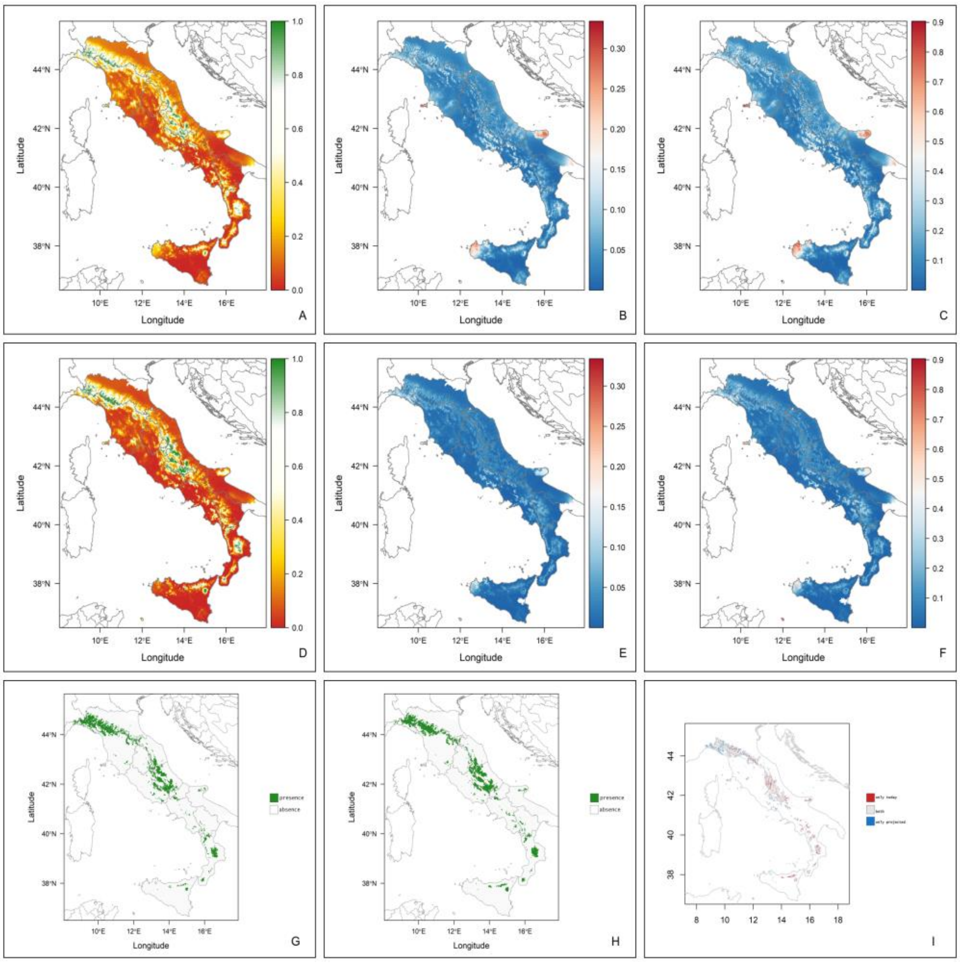

Fig. S.2.271-A) 'Projected' base output; B) 'Projected' uncertainty standard deviation; C) 'Projected' uncertainty range of values; D) 'Present' base output; E) 'Present' uncertainty standard deviation; F) 'Present' uncertainty range of values; G) 'Projected' binary distribution; H) 'Present' binary distribution; I) Range size variation.

Value	Present	Projected
AUC.train	0.925	0.881
AUC.test	0.930	0.879
AUC.diff	0.020	0.020
pROC	1.632	1.382
OR.p10	0.167	0.139
CBI	0.920	0.966
perc.stable	69.447	69.447
range.size	0.206	0.206

S.2.272 *Siler montanum* GAM

Species: *Siler montanum* Crantz subsp. *siculum* (Spreng.) Iamonico, Bartolucci & F.Conti

Procedure: GAM

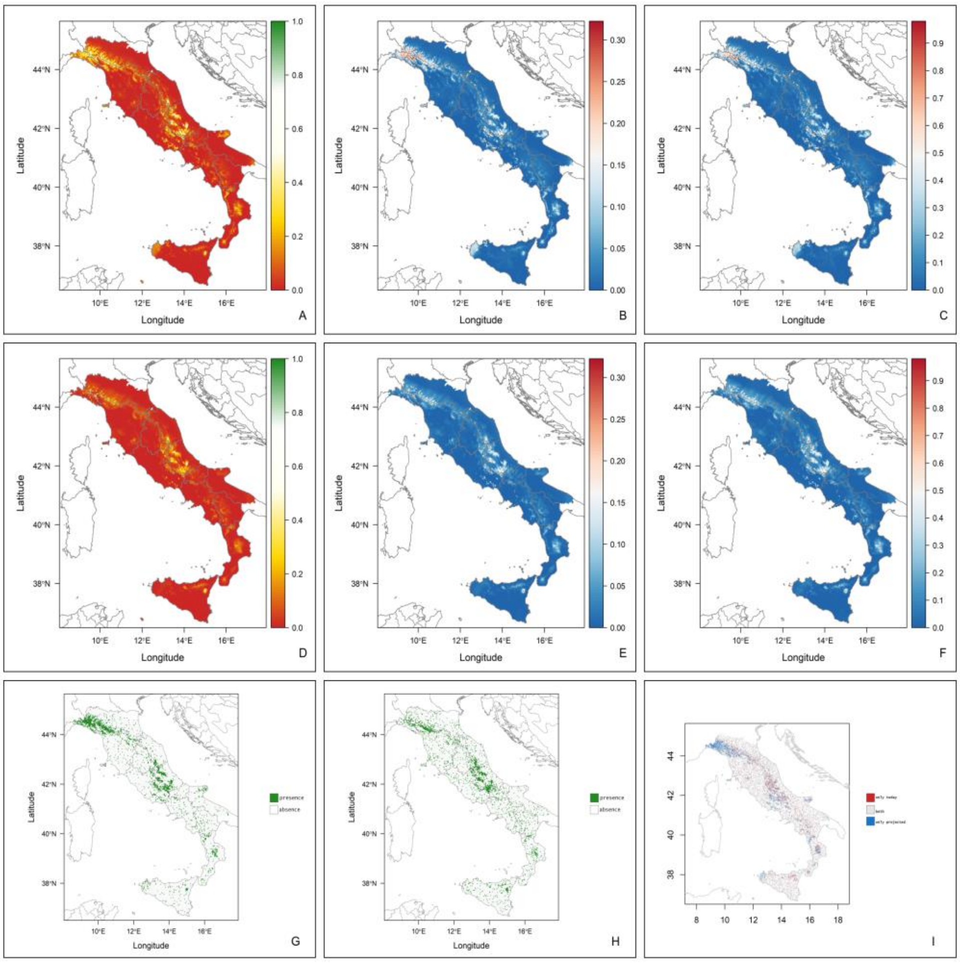

Fig. S.2.272-A) 'Projected' base output; B) 'Projected' uncertainty standard deviation; C) 'Projected' uncertainty range of values; D) 'Present' base output; E) 'Present' uncertainty standard deviation; F) 'Present' uncertainty range of values; G) 'Projected' binary distribution; H) 'Present' binary distribution; I) Range size variation.

Value	Present	Projected
AUC.train	0.925	0.851
AUC.test	0.925	0.861
AUC.diff	0.018	0.018
pROC	1.623	1.364
OR.p10	0.172	0.117
CBI	0.642	0.915
perc.stable	19.904	19.904
range.size	-0.222	-0.222

S.2.273 *Siler montanum* GLM

Species: *Siler montanum* Crantz subsp. *siculum* (Spreng.) Iamonico, Bartolucci & F.Conti

Procedure: GLM

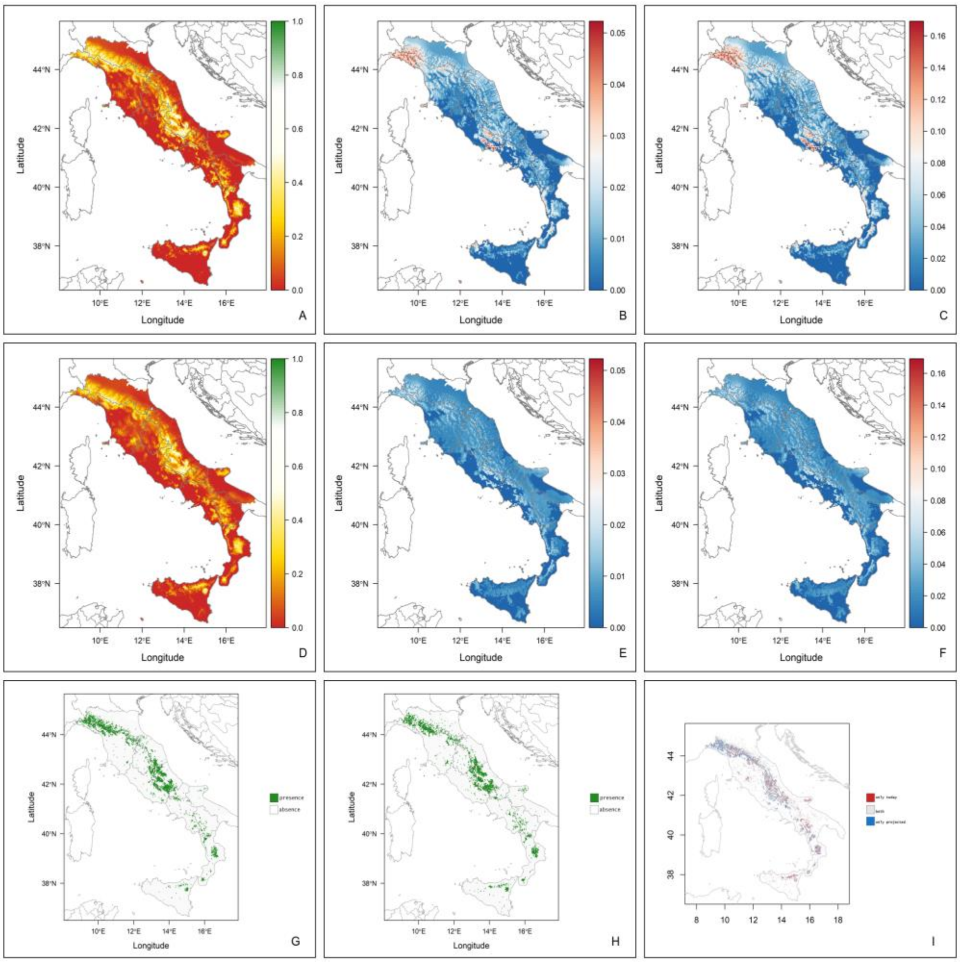

Fig. S.2.273-A) 'Projected' base output; B) 'Projected' uncertainty standard deviation; C) 'Projected' uncertainty range of values; D) 'Present' base output; E) 'Present' uncertainty standard deviation; F) 'Present' uncertainty range of values; G) 'Projected' binary distribution; H) 'Present' binary distribution; I) Range size variation.

Value	Present	Projected
AUC.train	0.914	0.866
AUC.test	0.915	0.867
AUC.diff	0.016	0.016
pROC	1.621	1.381
OR.p10	0.139	0.111
CBI	0.865	0.911
perc.stable	42.086	42.086
range.size	1.812	1.812

S.2.274 *Siler montanum* MARS

Species: *Siler montanum* Crantz subsp. *siculum* (Spreng.) Iamonico, Bartolucci & F.Conti

Procedure: MARS

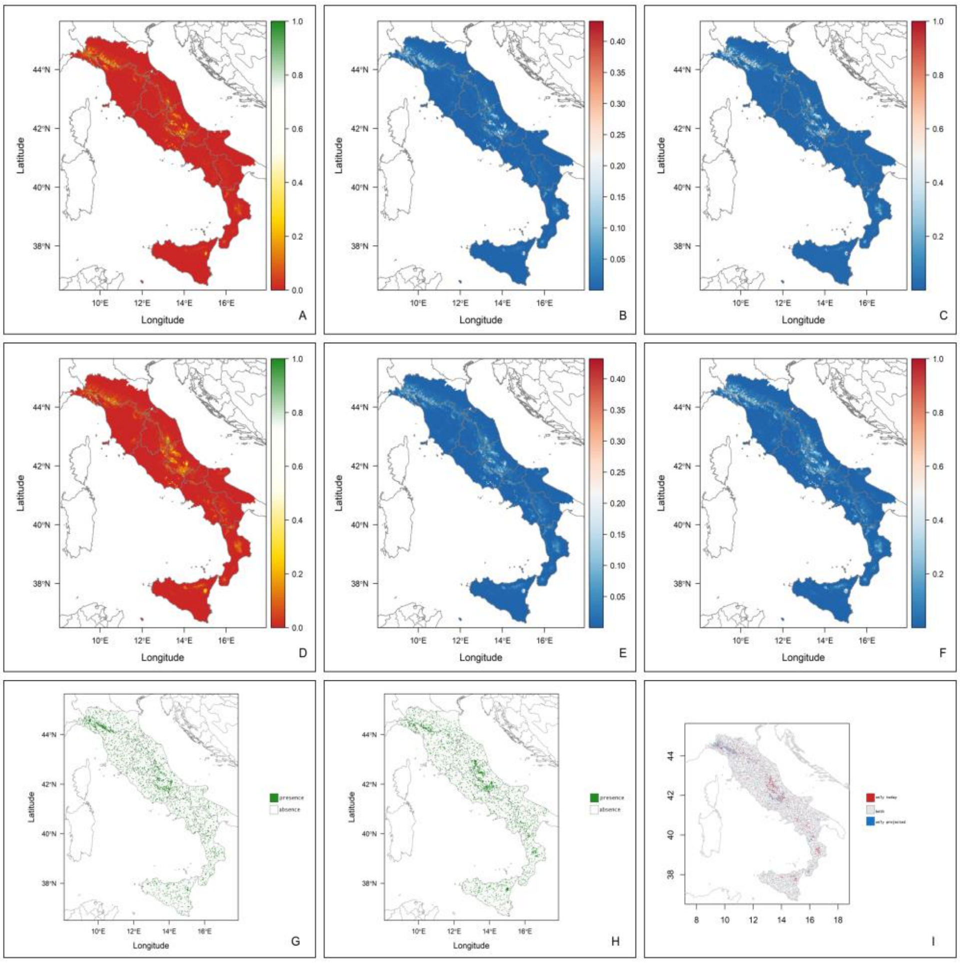

Fig. S.2.274-A) 'Projected' base output; B) 'Projected' uncertainty standard deviation; C) 'Projected' uncertainty range of values; D) 'Present' base output; E) 'Present' uncertainty standard deviation; F) 'Present' uncertainty range of values; G) 'Projected' binary distribution; H) 'Present' binary distribution; I) Range size variation.

Value	Present	Projected
AUC.train	0.931	0.863
AUC.test	0.858	0.803
AUC.diff	0.060	0.060
pROC	1.328	1.189
OR.p10	0.233	0.133
CBI	0.817	0.793
perc.stable	10.105	10.105
range.size	-1.445	-1.445

S.2.275 *Siler montanum* RF

Species: *Siler montanum* Crantz subsp. *siculum* (Spreng.) Iamonico, Bartolucci & F.Conti

Procedure: RF

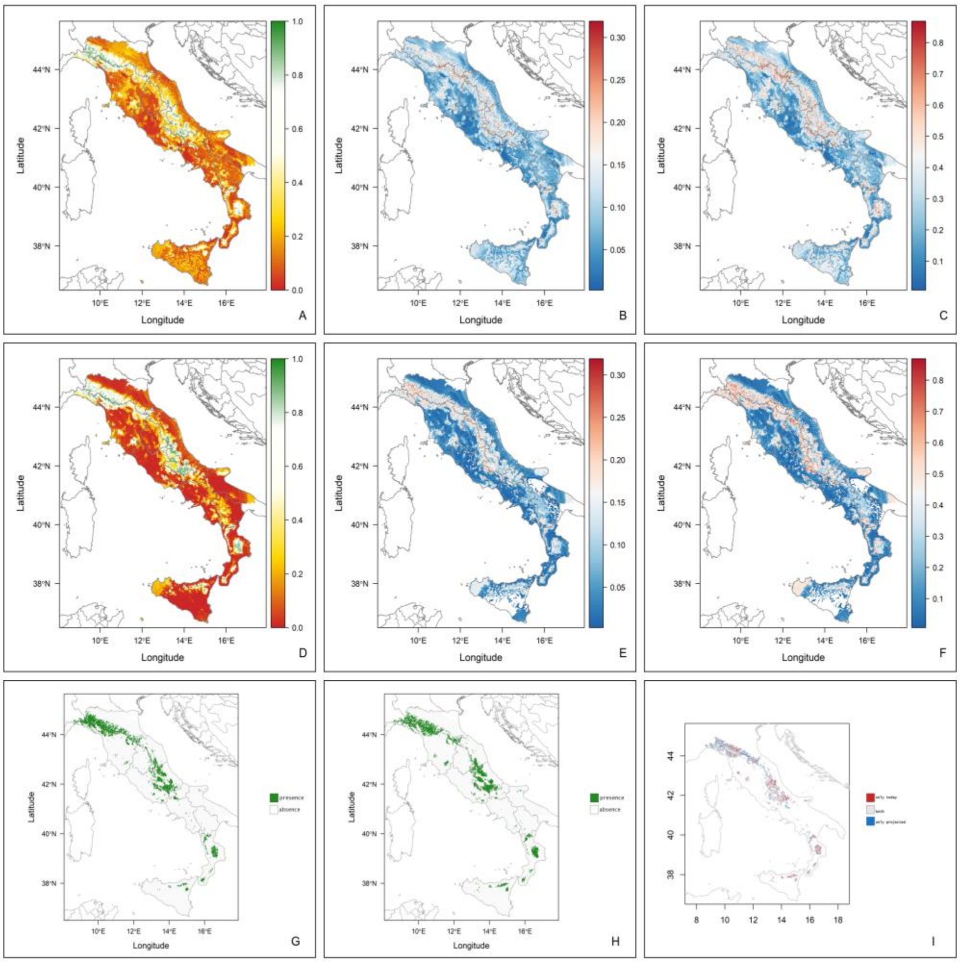

Fig. S.2.275-A) 'Projected' base output; B) 'Projected' uncertainty standard deviation; C) 'Projected' uncertainty range of values; D) 'Present' base output; E) 'Present' uncertainty standard deviation; F) 'Present' uncertainty range of values; G) 'Projected' binary distribution; H) 'Present' binary distribution; I) Range size variation.

Value	Present	Projected
AUC.train	1.000	1.000
AUC.test	0.966	0.949
AUC.diff	0.051	0.051
pROC	1.513	1.345
OR.p10	0.522	0.417
CBI	0.972	0.958
perc.stable	57.352	57.352
range.size	1.360	1.360

S.2.276 *Siler montanum* SVM

Species: *Siler montanum* Crantz subsp. *siculum* (Spreng.) Iamonico, Bartolucci & F.Conti

Procedure: SVM

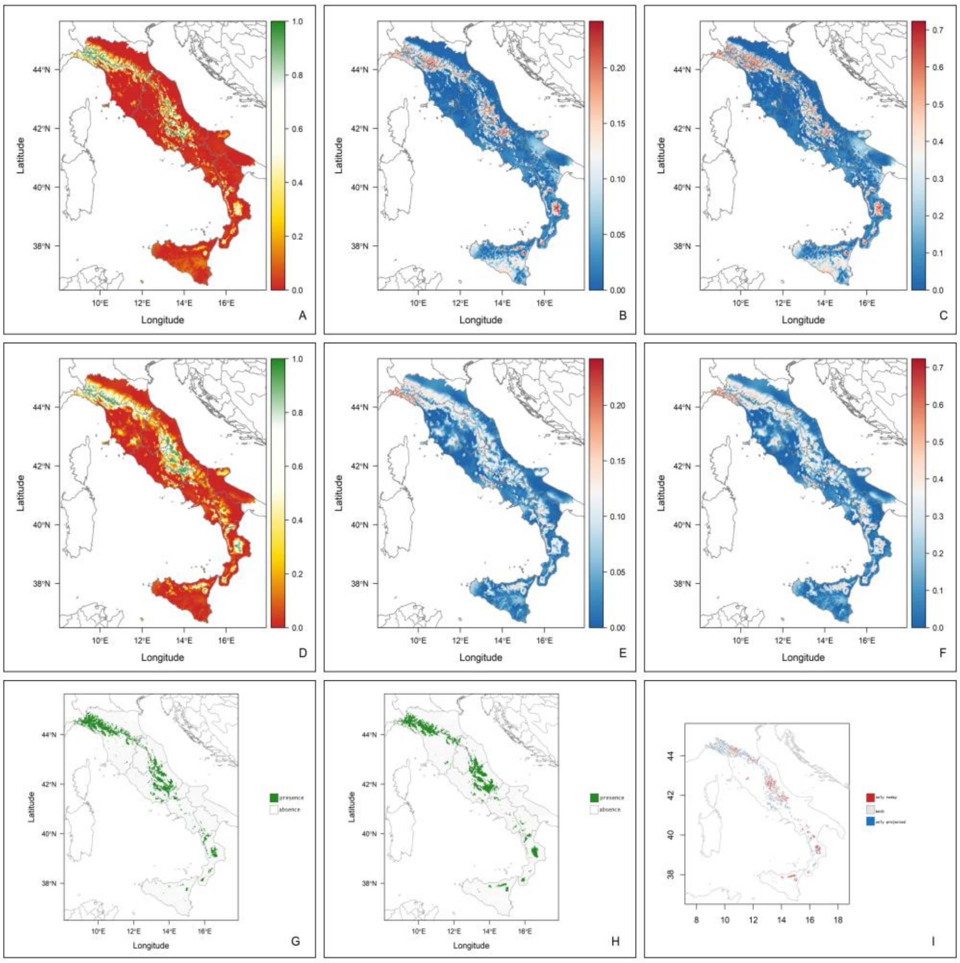

Fig. S.2.276-A) 'Projected' base output; B) 'Projected' uncertainty standard deviation; C) 'Projected' uncertainty range of values; D) 'Present' base output; E) 'Present' uncertainty standard deviation; F) 'Present' uncertainty range of values; G) 'Projected' binary distribution; H) 'Present' binary distribution; I) Range size variation.

Value	Present	Projected
AUC.train	0.934	0.874
AUC.test	0.918	0.849
AUC.diff	0.036	0.036
pROC	1.614	1.217
OR.p10	0.167	0.161
CBI	0.891	0.923
perc.stable	65.215	65.215
range.size	-0.539	-0.539

S.2.277 *Viola eugeniae* Biomod2

Species: *Viola eugeniae* Parl. subsp. *eugeniae*

Procedure: Biomod2

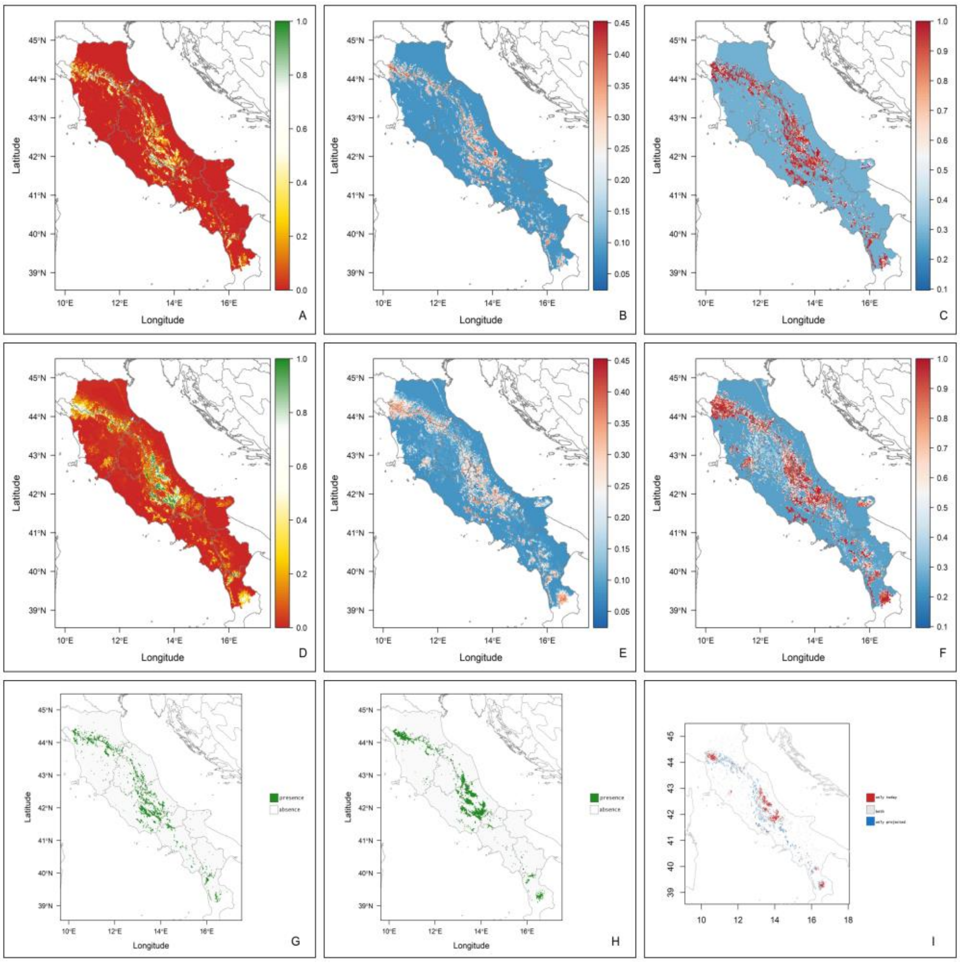

Fig. S.2.277-A) 'Projected' base output; B) 'Projected' uncertainty standard deviation; C) 'Projected' uncertainty range of values; D) 'Present' base output; E) 'Present' uncertainty standard deviation; F) 'Present' uncertainty range of values; G) 'Projected' binary distribution; H) 'Present' binary distribution; I) Range size variation.

Value	Present	Projected
AUC.train	0.990	0.991
AUC.test	0.987	0.972
AUC.diff	0.020	0.020
pROC	1.912	1.855
OR.p10	0.109	0.445
CBI	0.928	0.981
perc.stable	43.298	43.298
range.size	-0.186	-0.186

S.2.278 *Viola eugeniae* Bioclim

Species: *Viola eugeniae* Parl. subsp. *eugeniae*

Procedure: Bioclim

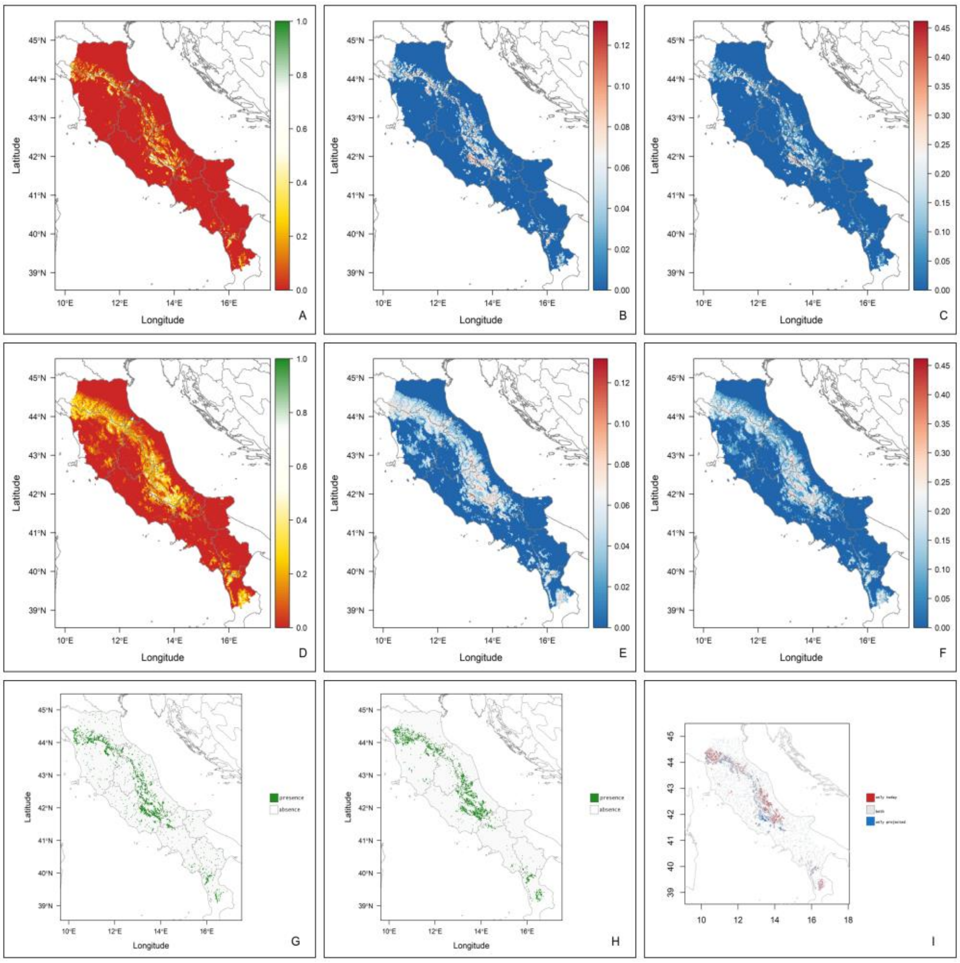

Fig. S.2.278-A) 'Projected' base output; B) 'Projected' uncertainty standard deviation; C) 'Projected' uncertainty range of values; D) 'Present' base output; E) 'Present' uncertainty standard deviation; F) 'Present' uncertainty range of values; G) 'Projected' binary distribution; H) 'Present' binary distribution; I) Range size variation.

Value	Present	Projected
AUC.train	0.899	0.937
AUC.test	0.859	0.879
AUC.diff	0.062	0.062
pROC	1.333	1.425
OR.p10	0.209	0.191
CBI	0.734	0.973
perc.stable	31.347	31.347
range.size	2.658	2.658

S.2.279 *Viola eugeniae* Domain

Species: *Viola eugeniae* Parl. subsp. *eugeniae*

Procedure: Domain

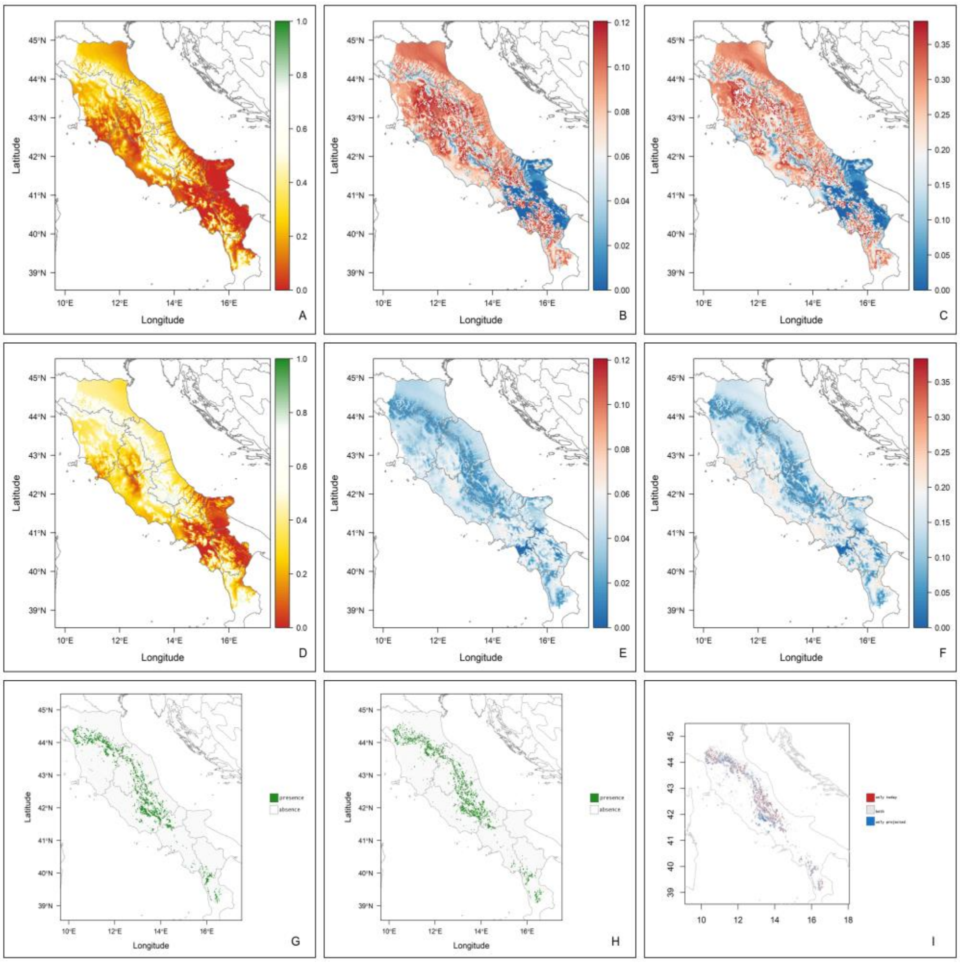

Fig. S.2.279-A) 'Projected' base output; B) 'Projected' uncertainty standard deviation; C) 'Projected' uncertainty range of values; D) 'Present' base output; E) 'Present' uncertainty standard deviation; F) 'Present' uncertainty range of values; G) 'Projected' binary distribution; H) 'Present' binary distribution; I) Range size variation.

Value	Present	Projected
AUC.train	0.908	0.931
AUC.test	0.881	0.900
AUC.diff	0.038	0.038
pROC	1.522	1.610
OR.p10	0.200	0.255
CBI	0.989	0.976
perc.stable	37.330	37.330
range.size	0.172	0.172

S.2.280 *Viola eugeniae* Maxent

Species: *Viola eugeniae* Parl. subsp. *eugeniae*

Procedure: Maxent

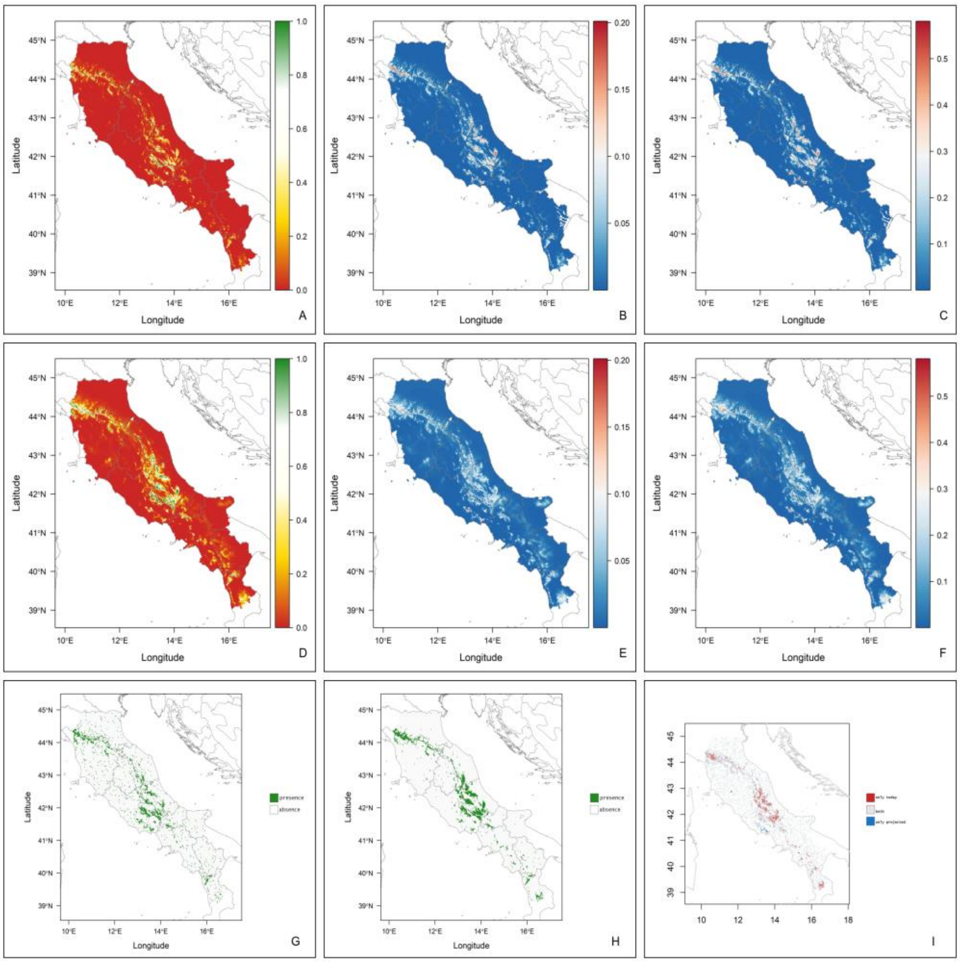

Fig. S.2.280-A) 'Projected' base output; B) 'Projected' uncertainty standard deviation; C) 'Projected' uncertainty range of values; D) 'Present' base output; E) 'Present' uncertainty standard deviation; F) 'Present' uncertainty range of values; G) 'Projected' binary distribution; H) 'Present' binary distribution; I) Range size variation.

Value	Present	Projected
AUC.train	0.950	0.968
AUC.test	0.956	0.955
AUC.diff	0.021	0.021
pROC	1.764	1.766
OR.p10	0.145	0.191
CBI	0.951	0.973
perc.stable	40.623	40.623
range.size	0.140	0.140

S.2.281 *Viola eugeniae* KDE

Species: *Viola eugeniae* Parl. subsp. *eugeniae*

Procedure: KDE

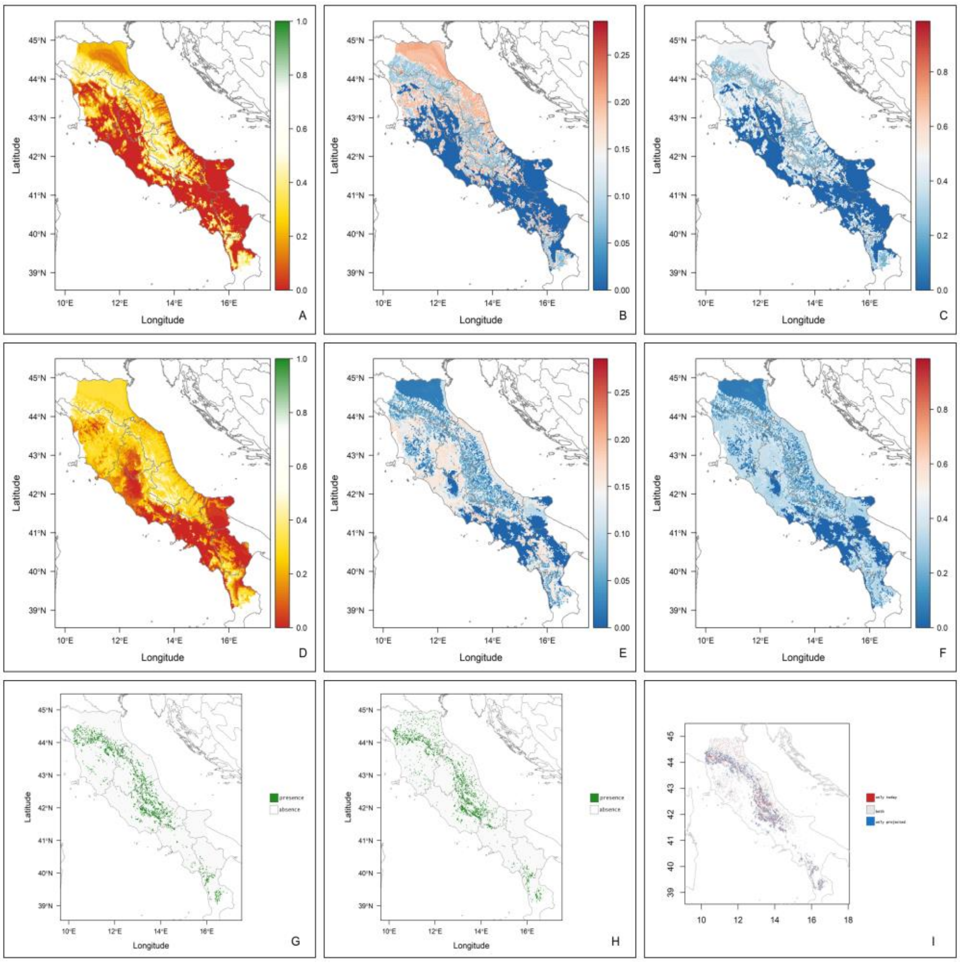

Fig. S.2.281-A) ‘Projected’ base output; B) ‘Projected’ uncertainty standard deviation; C) ‘Projected’ uncertainty range of values; D) ’Present’ base output; E) ’Present’ uncertainty standard deviation; F) ’Present’ uncertainty range of values; G) ‘Projected’ binary distribution; H) ‘Present’ binary distribution; I) Range size variation.

Value	Present	Projected
AUC.train	0.909	0.937
AUC.test	0.878	0.924
AUC.diff	0.051	0.026
pROC	1.501	1.652
OR.p10	0.191	0.118
CBI	0.983	0.955
perc.stable	23.606	23.606
range.size	0.710	0.710

S.2.282 *Viola eugeniae* CTA

Species: *Viola eugeniae* Parl. subsp. *eugeniae*

Procedure: CTA

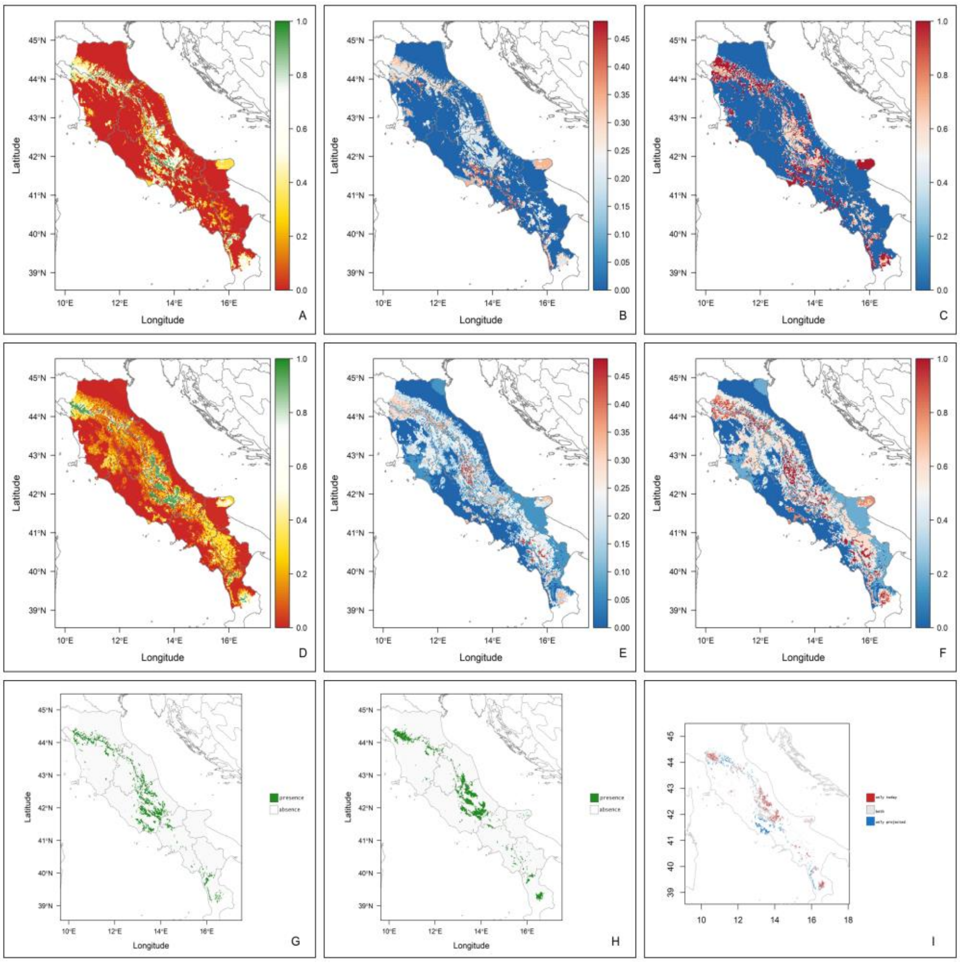

Fig. S.2.282-A) ‘Projected’ base output; B) ‘Projected’ uncertainty standard deviation; C) ‘Projected’ uncertainty range of values; D) ’Present’ base output; E) ’Present’ uncertainty standard deviation; F) ’Present’ uncertainty range of values; G) ‘Projected’ binary distribution; H) ‘Present’ binary distribution; I) Range size variation.

Value	Present	Projected
AUC.train	0.972	0.981
AUC.test	0.940	0.956
AUC.diff	0.027	0.027
pROC	1.333	1.397
OR.p10	0.145	0.127
CBI	0.828	0.827
perc.stable	49.205	49.205
range.size	-0.738	-0.738

S.2.283 *Viola eugeniae* FDA

Species: *Viola eugeniae* Parl. subsp. *eugeniae*

Procedure: FDA

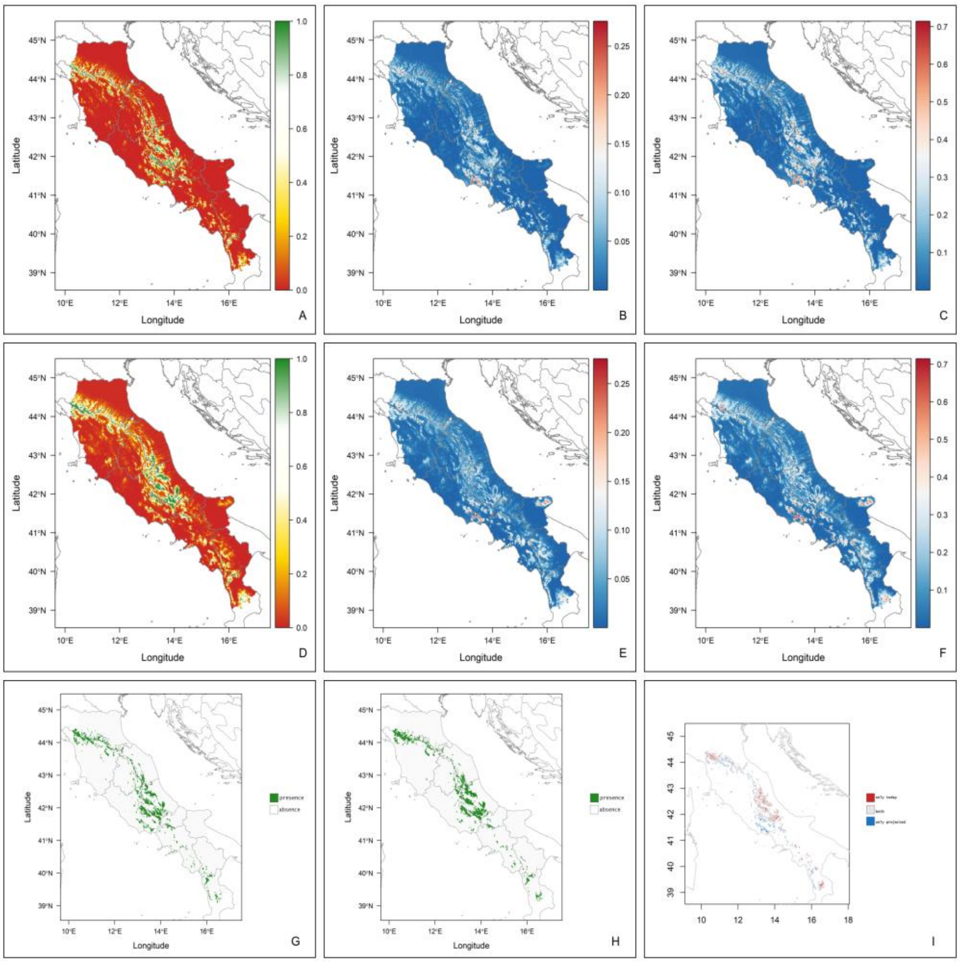

Fig. S.2.283-A) 'Projected' base output; B) 'Projected' uncertainty standard deviation; C) 'Projected' uncertainty range of values; D) 'Present' base output; E) 'Present' uncertainty standard deviation; F) 'Present' uncertainty range of values; G) 'Projected' binary distribution; H) 'Present' binary distribution; I) Range size variation.

Value	Present	Projected
AUC.train	0.935	0.959
AUC.test	0.946	0.957
AUC.diff	0.013	0.013
pROC	1.735	1.790
OR.p10	0.100	0.155
CBI	0.892	0.946
perc.stable	66.006	66.006
range.size	-1.793	-1.793

S.2.284 *Viola eugeniae* GAM

Species: *Viola eugeniae* Parl. subsp. *eugeniae*

Procedure: GAM

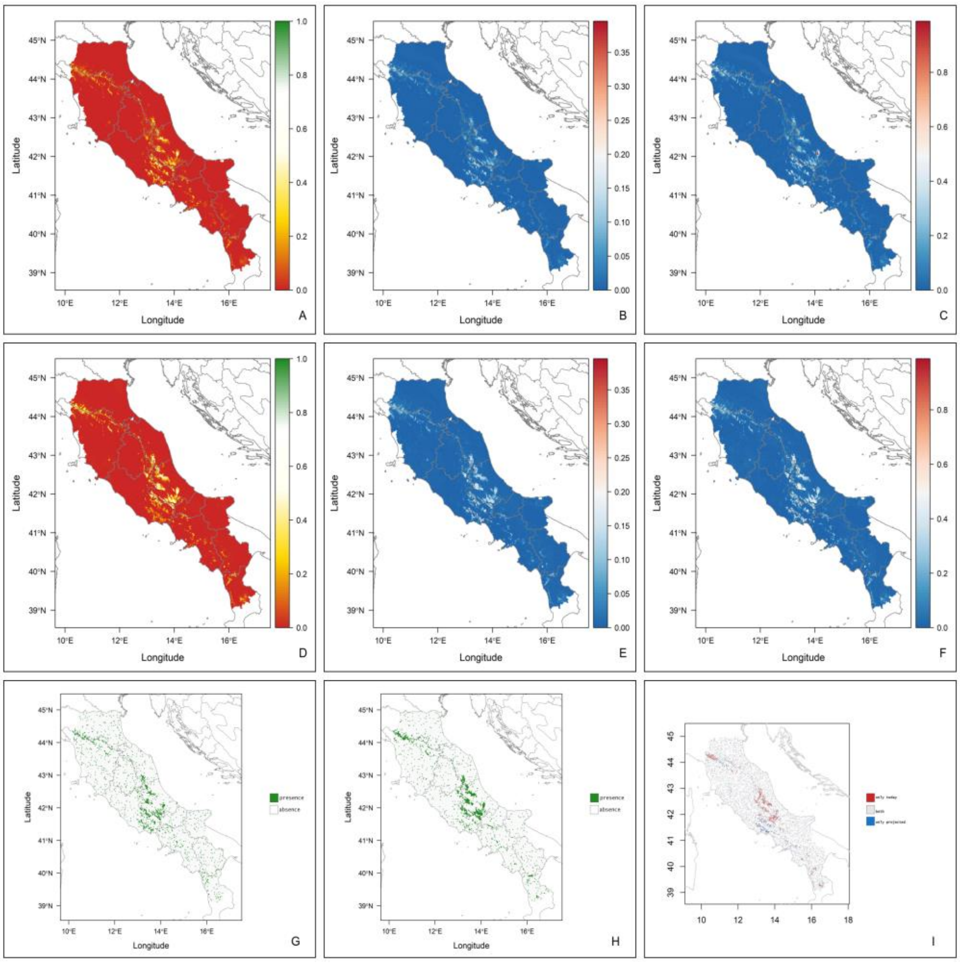

Fig. S.2.284-A) 'Projected' base output; B) 'Projected' uncertainty standard deviation; C) 'Projected' uncertainty range of values; D) 'Present' base output; E) 'Present' uncertainty standard deviation; F) 'Present' uncertainty range of values; G) 'Projected' binary distribution; H) 'Present' binary distribution; I) Range size variation.

Value	Present	Projected
AUC.train	0.963	0.950
AUC.test	0.913	0.915
AUC.diff	0.043	0.043
pROC	1.560	1.611
OR.p10	0.191	0.200
CBI	0.975	0.875
perc.stable	22.421	22.421
range.size	-0.795	-0.795

S.2.285 *Viola eugeniae* GLM

Species: *Viola eugeniae* Parl. subsp. *eugeniae*

Procedure: GLM

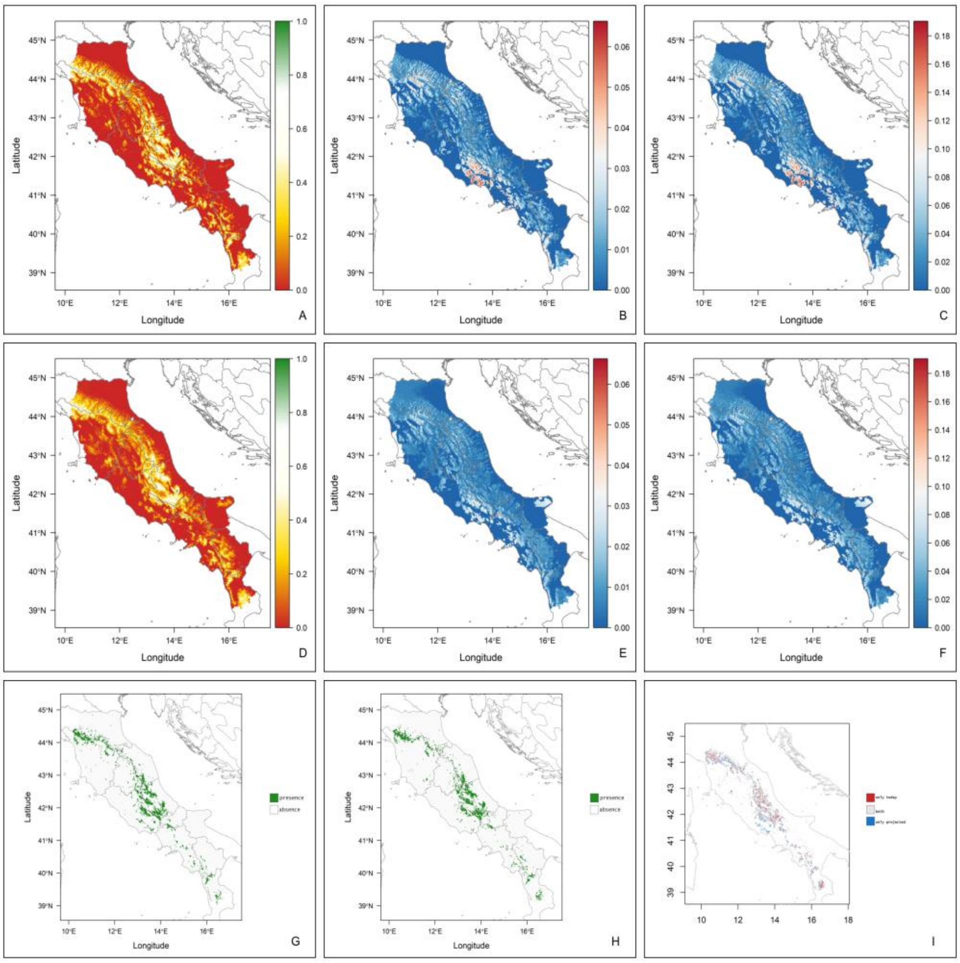

Fig. S.2.285-A) 'Projected' base output; B) 'Projected' uncertainty standard deviation; C) 'Projected' uncertainty range of values; D) 'Present' base output; E) 'Present' uncertainty standard deviation; F) 'Present' uncertainty range of values; G) 'Projected' binary distribution; H) 'Present' binary distribution; I) Range size variation.

Value	Present	Projected
AUC.train	0.942	0.961
AUC.test	0.952	0.966
AUC.diff	0.005	0.005
pROC	1.752	1.823
OR.p10	0.109	0.164
CBI	0.998	0.994
perc.stable	47.775	47.775
range.size	2.255	2.255

S.2.286 *Viola eugeniae* MARS

Species: *Viola eugeniae* Parl. subsp. *eugeniae*

Procedure: MARS

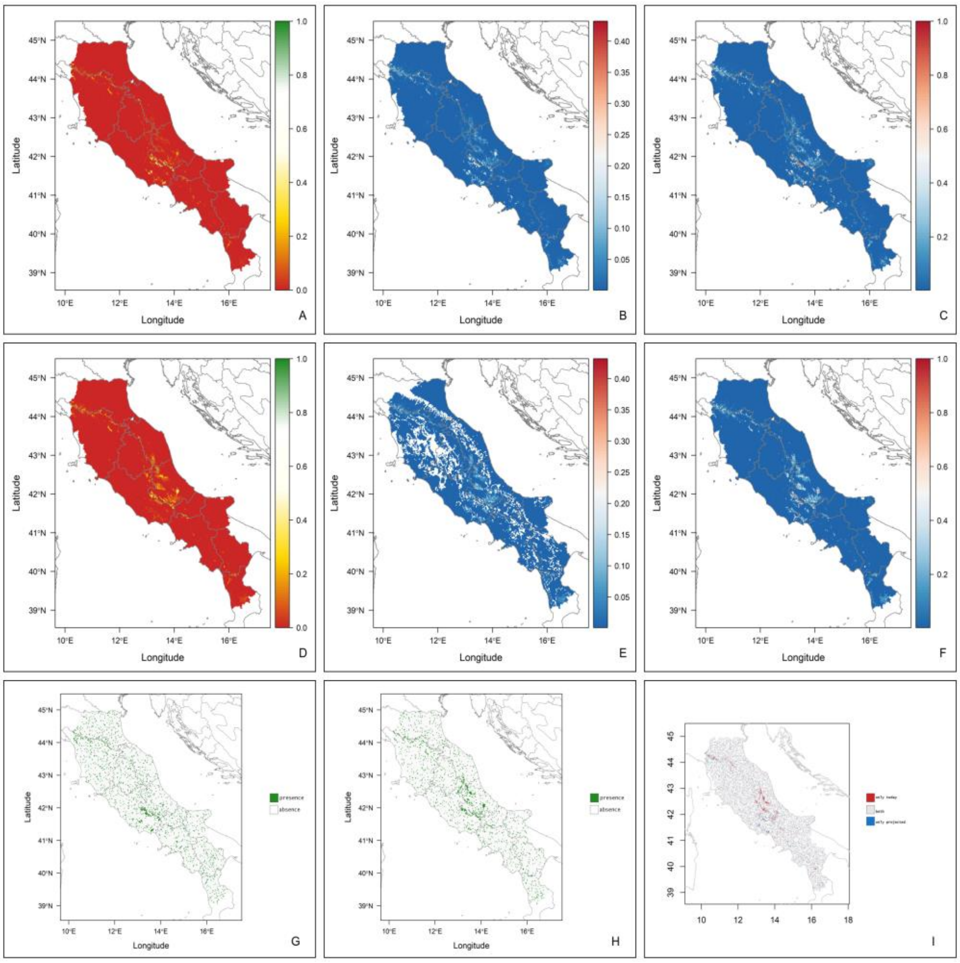

Fig. S.2.286-A) 'Projected' base output; B) 'Projected' uncertainty standard deviation; C) 'Projected' uncertainty range of values; D) 'Present' base output; E) 'Present' uncertainty standard deviation; F) 'Present' uncertainty range of values; G) 'Projected' binary distribution; H) 'Present' binary distribution; I) Range size variation.

Value	Present	Projected
AUC.train	0.932	0.941
AUC.test	0.901	0.818
AUC.diff	0.126	0.126
pROC	1.479	1.288
OR.p10	0.191	0.255
CBI	0.987	0.651
perc.stable	7.561	7.561
range.size	3.195	3.195

S.2.287 *Viola eugeniae* RF

Species: *Viola eugeniae* Parl. subsp. *eugeniae*

Procedure: RF

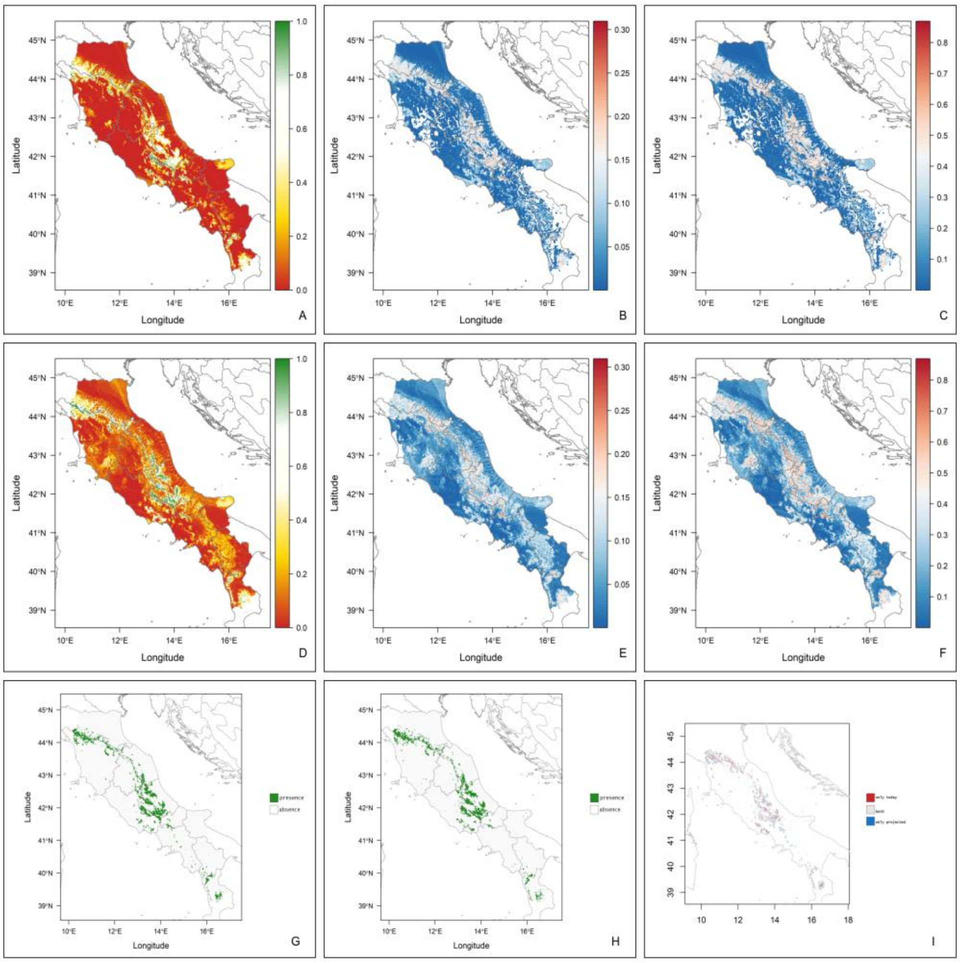

Fig. S.2.287-A) 'Projected' base output; B) 'Projected' uncertainty standard deviation; C) 'Projected' uncertainty range of values; D) 'Present' base output; E) 'Present' uncertainty standard deviation; F) 'Present' uncertainty range of values; G) 'Projected' binary distribution; H) 'Present' binary distribution; I) Range size variation.

Value	Present	Projected
AUC.train	1.000	1.000
AUC.test	0.988	0.975
AUC.diff	0.025	0.025
pROC	1.770	1.706
OR.p10	0.336	0.318
CBI	0.952	0.945
perc.stable	67.665	67.665
range.size	-1.580	-1.580

S.2.288 *Viola eugeniae* SVM

Species: *Viola eugeniae* Parl. subsp. *eugeniae*

Procedure: SVM

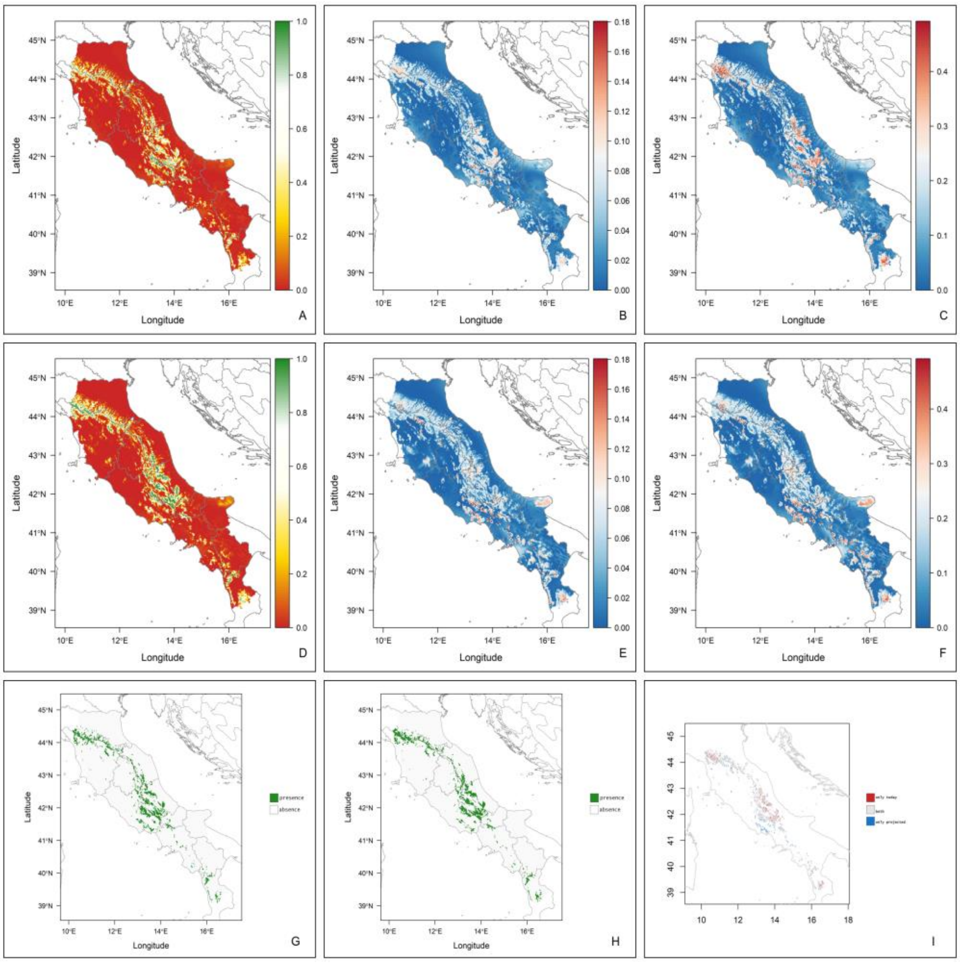

Fig. S.2.288-A) 'Projected' base output; B) 'Projected' uncertainty standard deviation; C) 'Projected' uncertainty range of values; D) 'Present' base output; E) 'Present' uncertainty standard deviation; F) 'Present' uncertainty range of values; G) 'Projected' binary distribution; H) 'Present' binary distribution; I) Range size variation.

Value	Present	Projected
AUC.train	0.942	0.971
AUC.test	0.943	0.943
AUC.diff	0.031	0.031
pROC	1.666	1.691
OR.p10	0.136	0.173
CBI	0.946	0.952
perc.stable	70.233	70.233
range.size	-0.103	-0.103

S.2.289 *Viola ferrarinii* Biomod2

Species: *Viola ferrarinii* Moraldo & Ricceri

Procedure: Biomod2

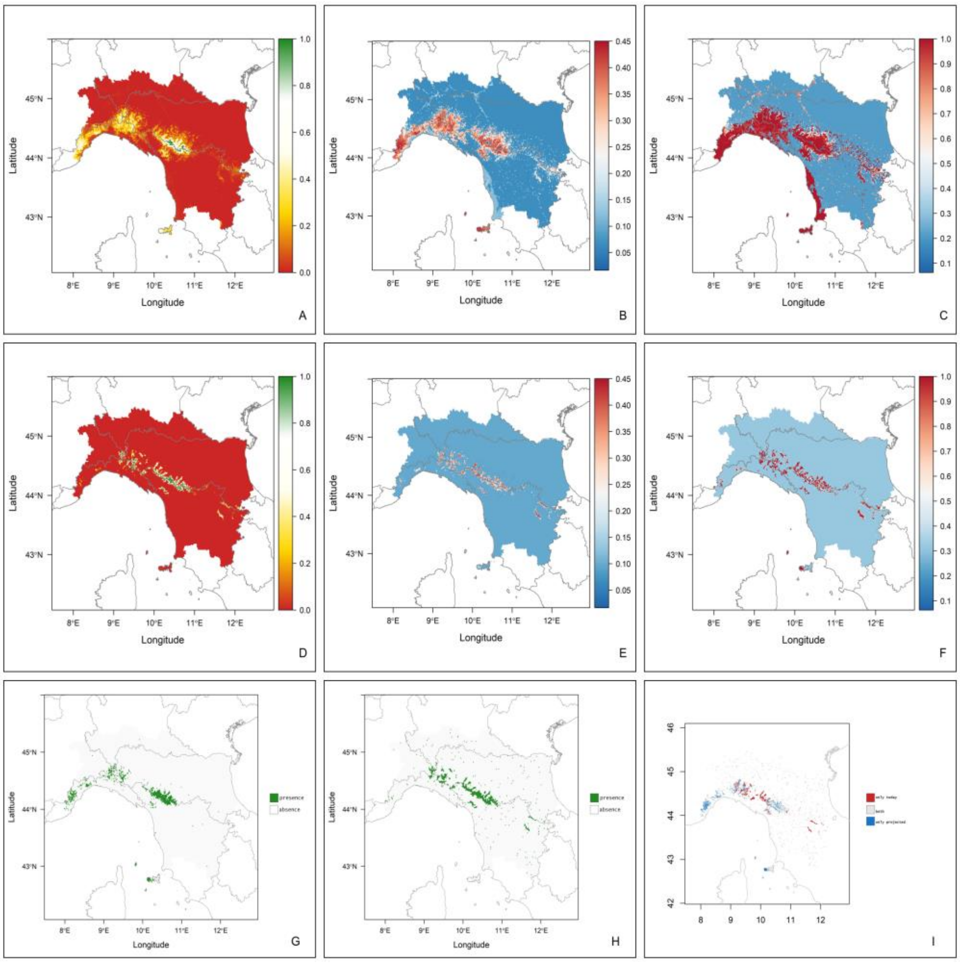

Fig. S.2.289-A) 'Projected' base output; B) 'Projected' uncertainty standard deviation; C) 'Projected' uncertainty range of values; D) 'Present' base output; E) 'Present' uncertainty standard deviation; F) 'Present' uncertainty range of values; G) 'Projected' binary distribution; H) 'Present' binary distribution; I) Range size variation.

Value	Present	Projected
AUC.train	0.999	0.999
AUC.test	1.000	0.988
AUC.diff	0.011	0.011
pROC	1.989	1.894
OR.p10	0.080	0.560
CBI	0.869	0.893
perc.stable	28.955	28.955
range.size	-3.162	-3.162

S.2.290 *Viola ferrarinii* Bioclim

Species: *Viola ferrarinii* Moraldo & Ricceri

Procedure: Bioclim

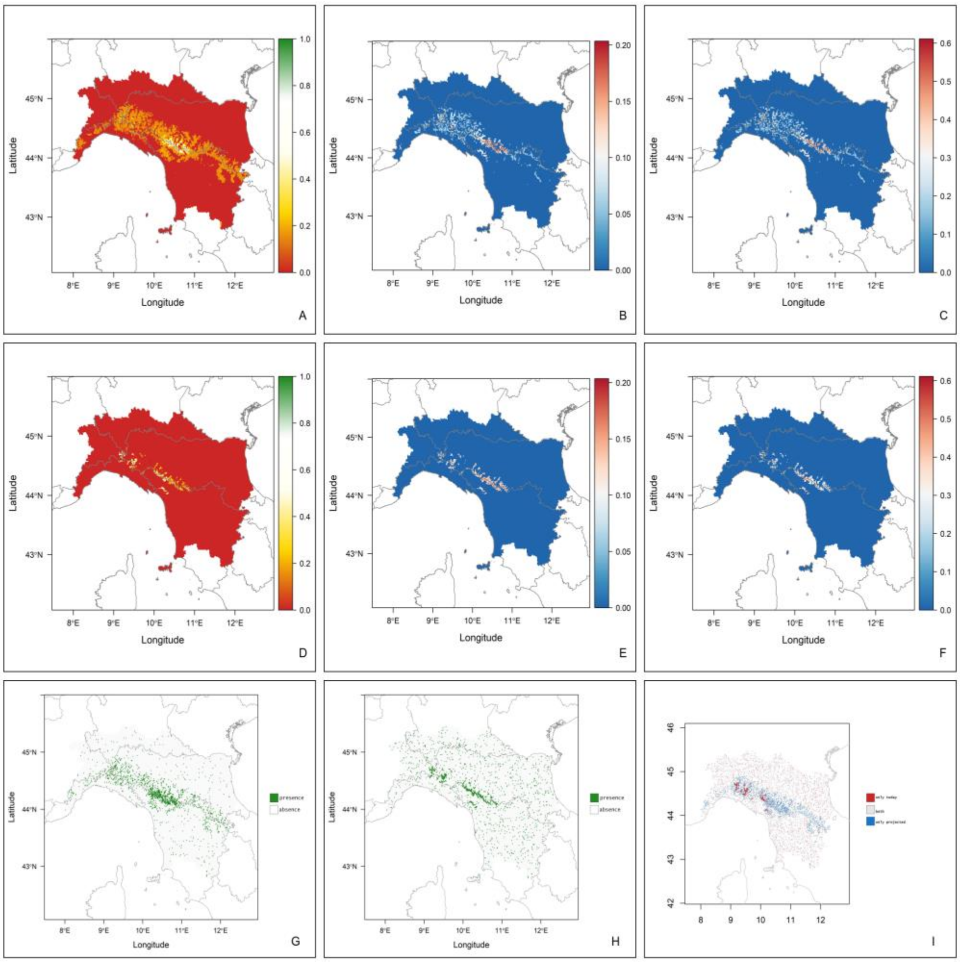

Fig. S.2.290-A) ‘Projected’ base output; B) ‘Projected’ uncertainty standard deviation; C) ‘Projected’ uncertainty range of values; D) ’Present’ base output; E) ’Present’ uncertainty standard deviation; F) ’Present’ uncertainty range of values; G) ‘Projected’ binary distribution; H) ‘Present’ binary distribution; I) Range size variation.

Value	Present	Projected
AUC.train	0.991	0.947
AUC.test	0.752	0.823
AUC.diff	0.125	0.125
pROC	1.216	1.466
OR.p10	0.480	0.200
CBI	0.054	0.459
perc.stable	8.405	8.405
range.size	0.805	0.805

S.2.291 *Viola ferrarinii* Domain

Species: *Viola ferrarinii* Moraldo & Ricceri

Procedure: Domain

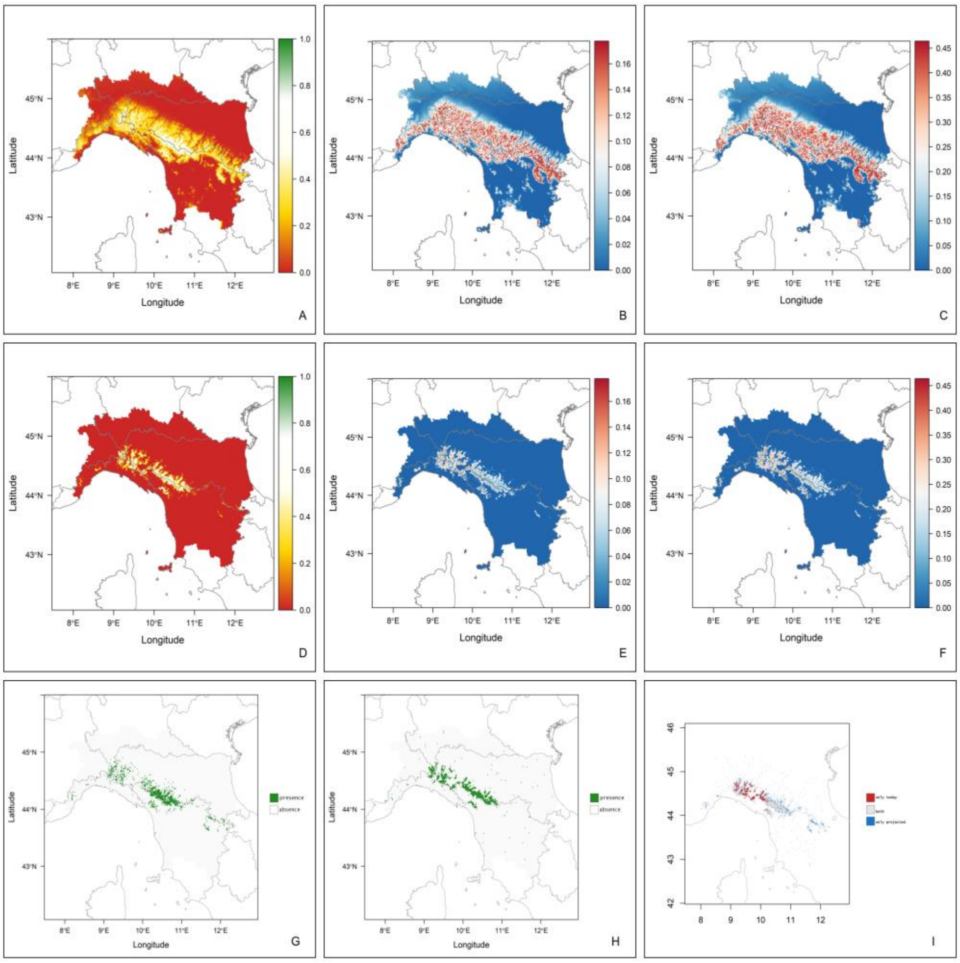

Fig. S.2.291-A) ‘Projected’ base output; B) ‘Projected’ uncertainty standard deviation; C) ‘Projected’ uncertainty range of values; D) ’Present’ base output; E) ’Present’ uncertainty standard deviation; F) ’Present’ uncertainty range of values; G) ‘Projected’ binary distribution; H) ‘Present’ binary distribution; I) Range size variation.

Value	Present	Projected
AUC.train	0.987	0.973
AUC.test	0.984	0.935
AUC.diff	0.047	0.047
pROC	1.958	1.774
OR.p10	0.240	0.360
CBI	0.979	0.778
perc.stable	33.632	33.632
range.size	0.846	0.846

S.2.292 *Viola ferrarinii* Maxent

Species: *Viola ferrarinii* Moraldo & Ricceri

Procedure: Maxent

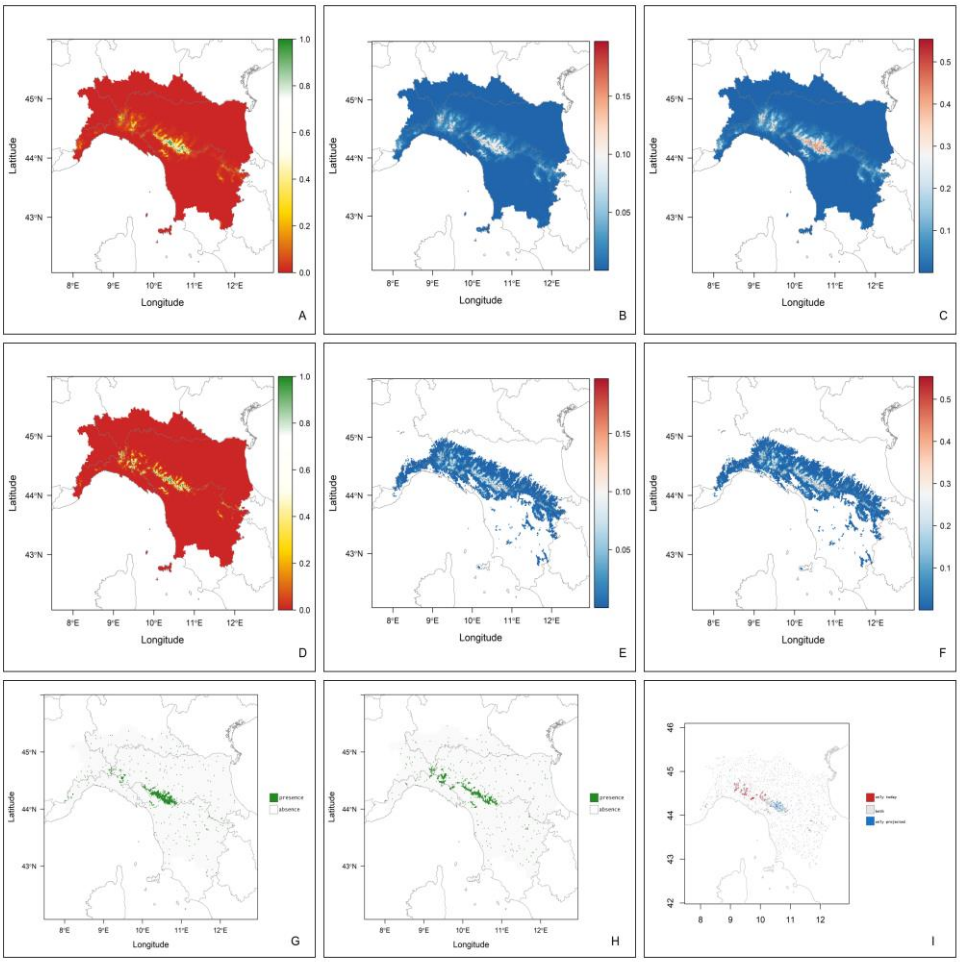

Fig. S.2.292-A) 'Projected' base output; B) 'Projected' uncertainty standard deviation; C) 'Projected' uncertainty range of values; D) 'Present' base output; E) 'Present' uncertainty standard deviation; F) 'Present' uncertainty range of values; G) 'Projected' binary distribution; H) 'Present' binary distribution; I) Range size variation.

Value	Present	Projected
AUC.train	0.993	0.979
AUC.test	0.993	0.982
AUC.diff	0.009	0.009
pROC	1.969	1.864
OR.p10	0.260	0.260
CBI	0.765	0.613
perc.stable	28.083	28.083
range.size	-0.825	-0.825

S.2.293 *Viola ferrarinii* KDE

Species: *Viola ferrarinii* Moraldo & Ricceri

Procedure: KDE

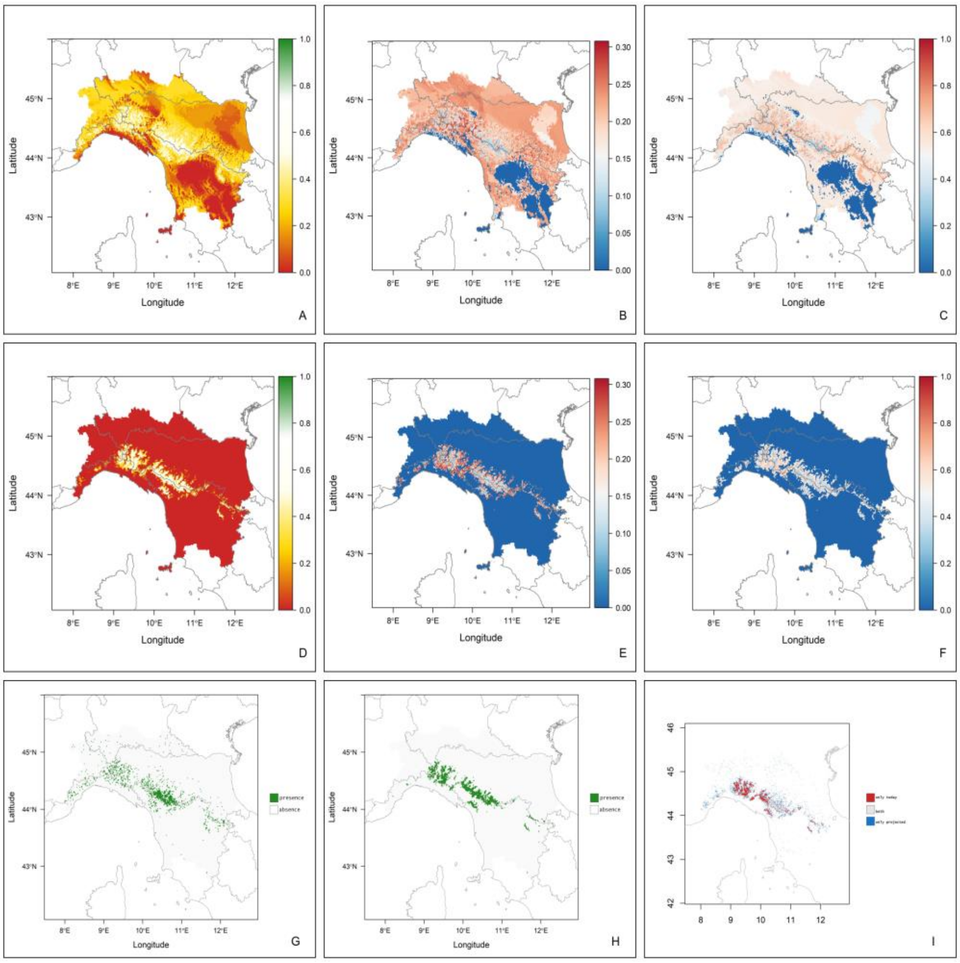

Fig. S.2.293-A) 'Projected' base output; B) 'Projected' uncertainty standard deviation; C) 'Projected' uncertainty range of values; D) 'Present' base output; E) 'Present' uncertainty standard deviation; F) 'Present' uncertainty range of values; G) 'Projected' binary distribution; H) 'Present' binary distribution; I) Range size variation.

Value	Present	Projected
AUC.train	0.985	0.967
AUC.test	0.949	0.900
AUC.diff	0.036	0.083
pROC	1.820	1.690
OR.p10	0.520	0.380
CBI	0.994	0.886
perc.stable	25.621	25.621
range.size	-1.359	-1.359

S.2.294 *Viola ferrarinii* CTA

Species: *Viola ferrarinii* Moraldo & Ricceri

Procedure: CTA

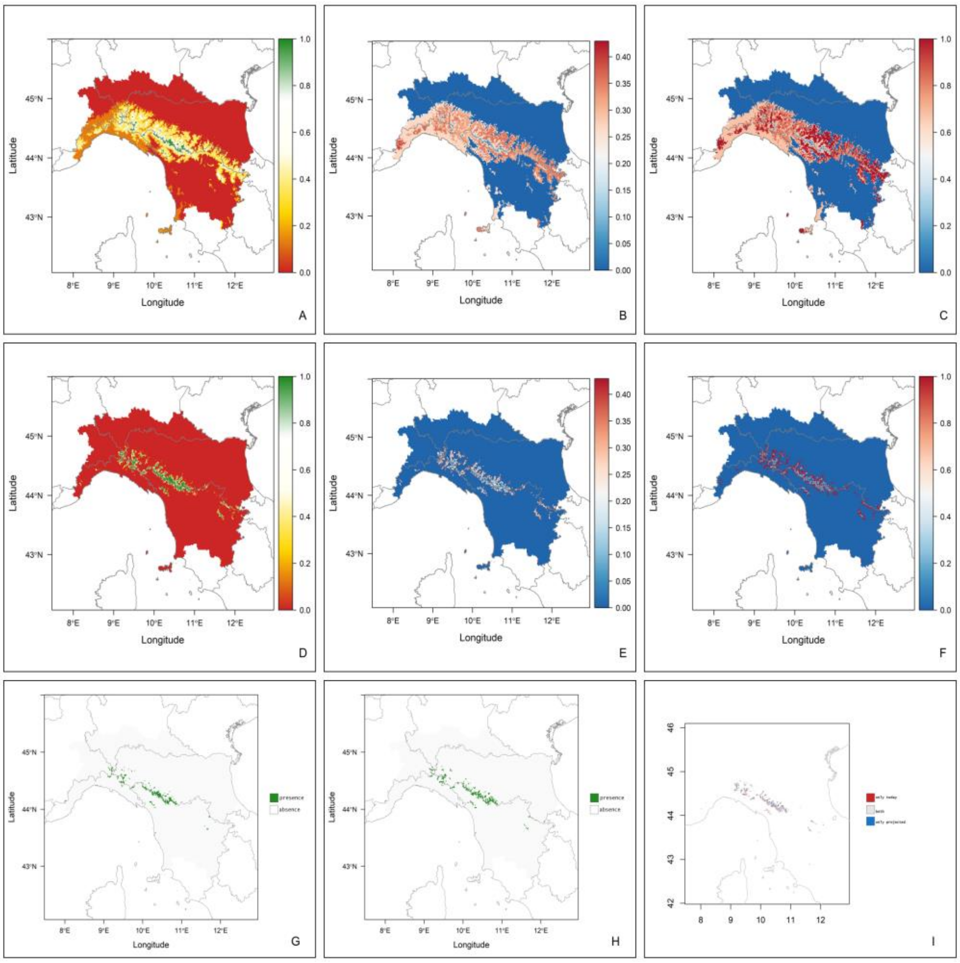

Fig. S.2.294-A) 'Projected' base output; B) 'Projected' uncertainty standard deviation; C) 'Projected' uncertainty range of values; D) 'Present' base output; E) 'Present' uncertainty standard deviation; F) 'Present' uncertainty range of values; G) 'Projected' binary distribution; H) 'Present' binary distribution; I) Range size variation.

Value	Present	Projected
AUC.train	0.996	0.983
AUC.test	0.966	0.952
AUC.diff	0.036	0.036
pROC	1.133	1.269
OR.p10	0.060	0.100
CBI	0.760	0.815
perc.stable	43.492	43.492
range.size	-9.076	-9.076

S.2.295 *Viola ferrarinii* FDA

Species: *Viola ferrarinii* Moraldo & Ricceri

Procedure: FDA

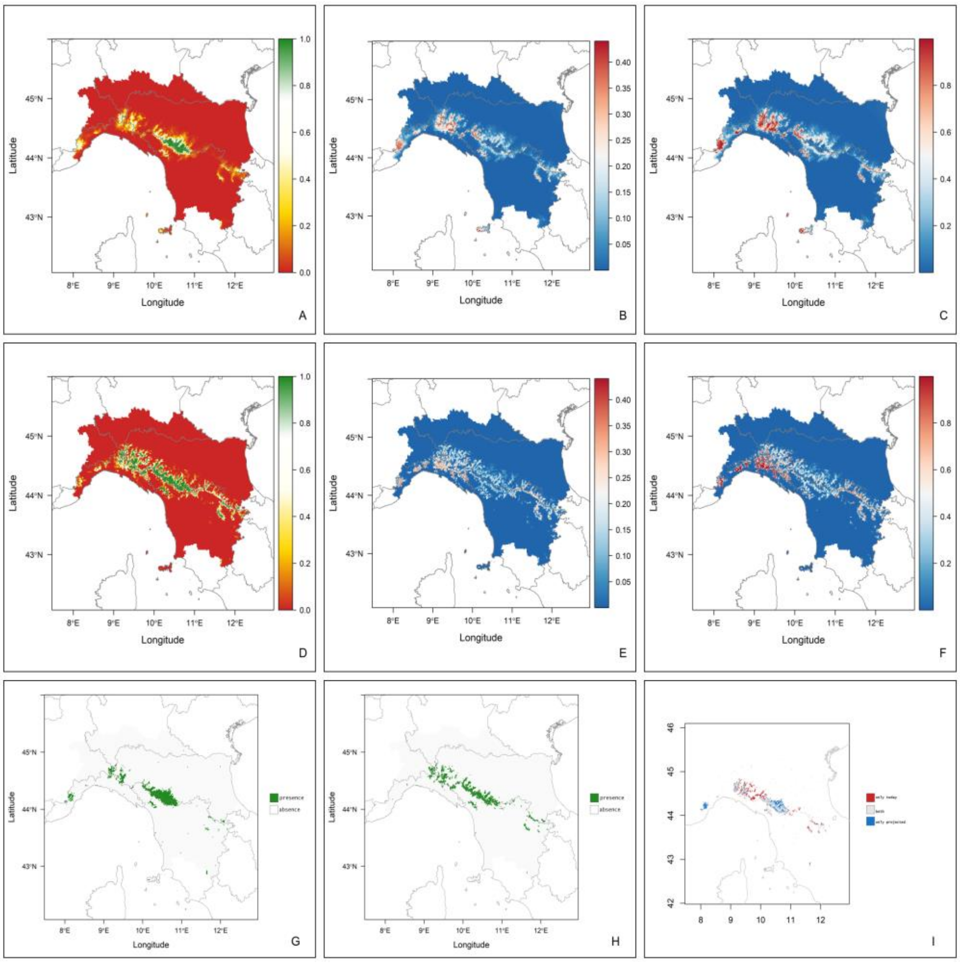

Fig. S.2.295-A) 'Projected' base output; B) 'Projected' uncertainty standard deviation; C) 'Projected' uncertainty range of values; D) 'Present' base output; E) 'Present' uncertainty standard deviation; F) 'Present' uncertainty range of values; G) 'Projected' binary distribution; H) 'Present' binary distribution; I) Range size variation.

Value	Present	Projected
AUC.train	0.990	0.989
AUC.test	0.993	0.988
AUC.diff	0.008	0.008
pROC	1.441	1.634
OR.p10	0.120	0.200
CBI	0.798	0.752
perc.stable	43.247	43.247
range.size	0.911	0.911

S.2.296 *Viola ferrarinii* GAM

Species: *Viola ferrarinii* Moraldo & Ricceri

Procedure: GAM

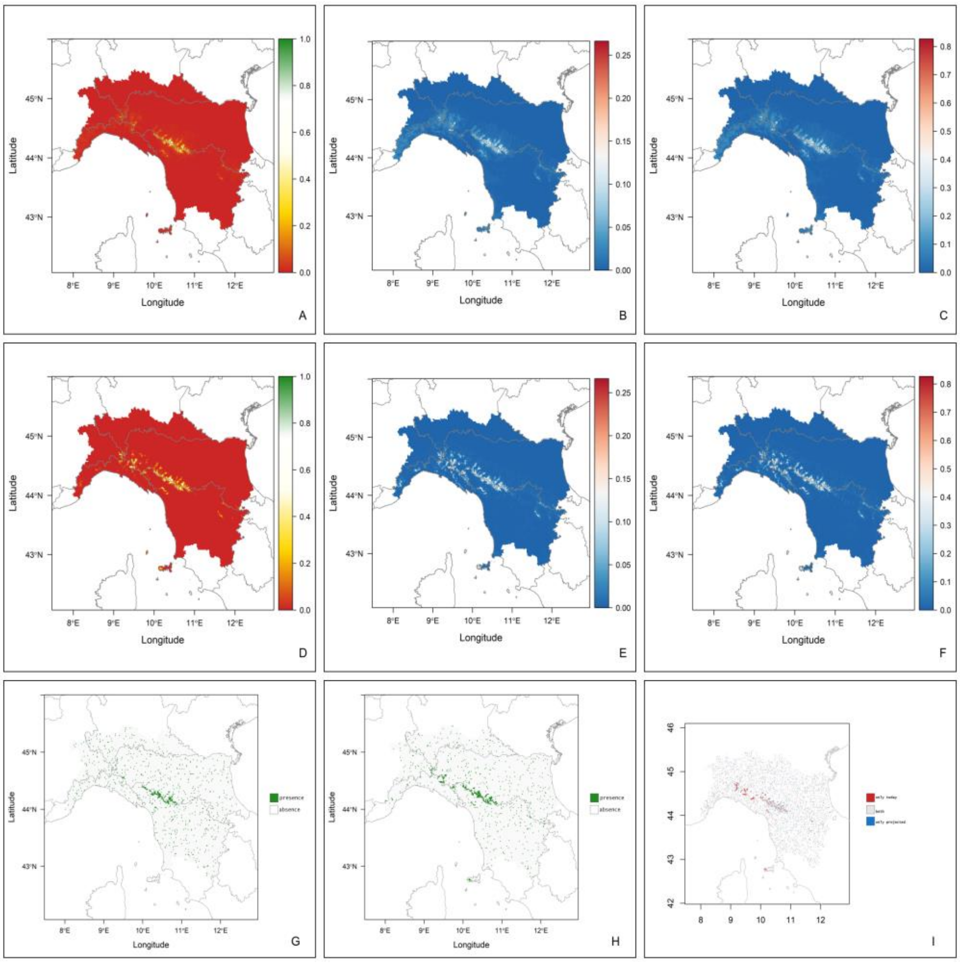

Fig. S.2.296-A) 'Projected' base output; B) 'Projected' uncertainty standard deviation; C) 'Projected' uncertainty range of values; D) 'Present' base output; E) 'Present' uncertainty standard deviation; F) 'Present' uncertainty range of values; G) 'Projected' binary distribution; H) 'Present' binary distribution; I) Range size variation.

Value	Present	Projected
AUC.train	0.993	0.983
AUC.test	0.968	0.958
AUC.diff	0.029	0.029
pROC	1.900	1.854
OR.p10	0.280	0.320
CBI	0.909	0.848
perc.stable	10.999	10.999
range.size	3.660	3.660

S.2.297 *Viola ferrarinii* GLM

Species: *Viola ferrarinii* Moraldo & Ricceri

Procedure: GLM

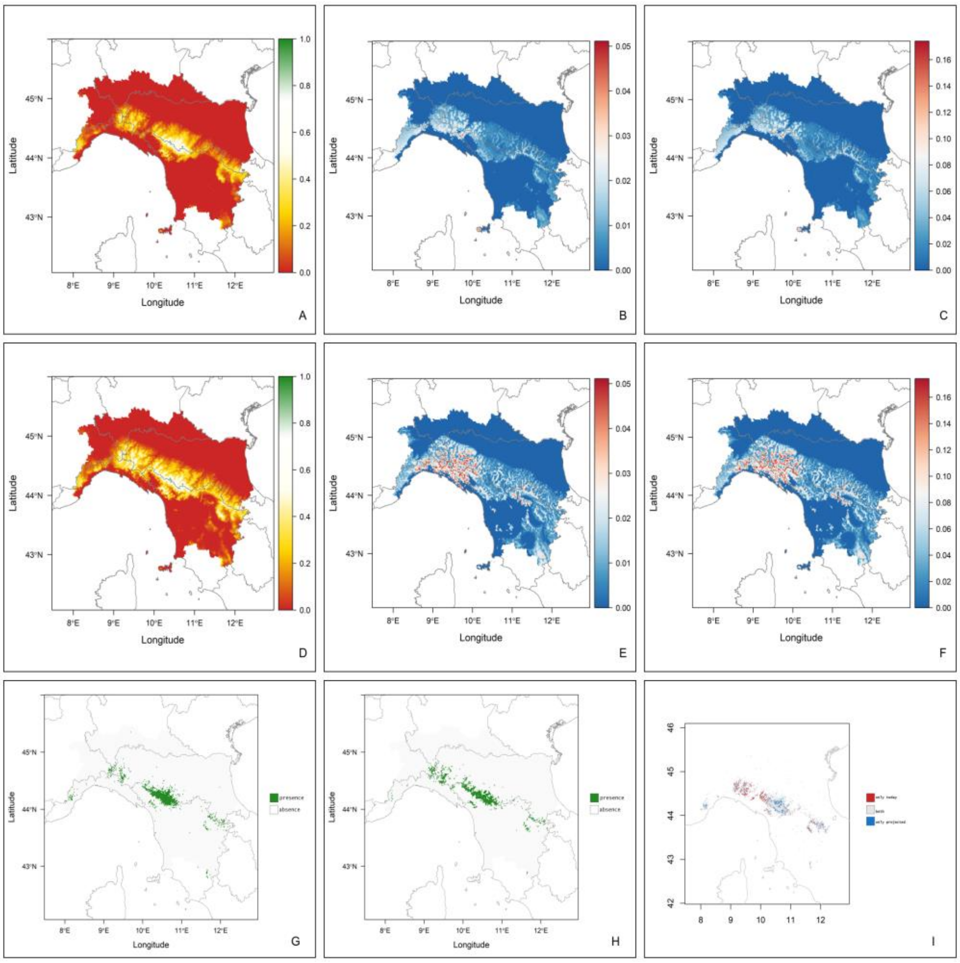

Fig. S.2.297-A) 'Projected' base output; B) 'Projected' uncertainty standard deviation; C) 'Projected' uncertainty range of values; D) 'Present' base output; E) 'Present' uncertainty standard deviation; F) 'Present' uncertainty range of values; G) 'Projected' binary distribution; H) 'Present' binary distribution; I) Range size variation.

Value	Present	Projected
AUC.train	0.992	0.982
AUC.test	0.990	0.985
AUC.diff	0.008	0.008
pROC	1.970	1.947
OR.p10	0.240	0.200
CBI	0.984	0.931
perc.stable	40.479	40.479
range.size	1.088	1.088

S.2.298 *Viola ferrarinii* MARS

Species: *Viola ferrarinii* Moraldo & Ricceri

Procedure: MARS

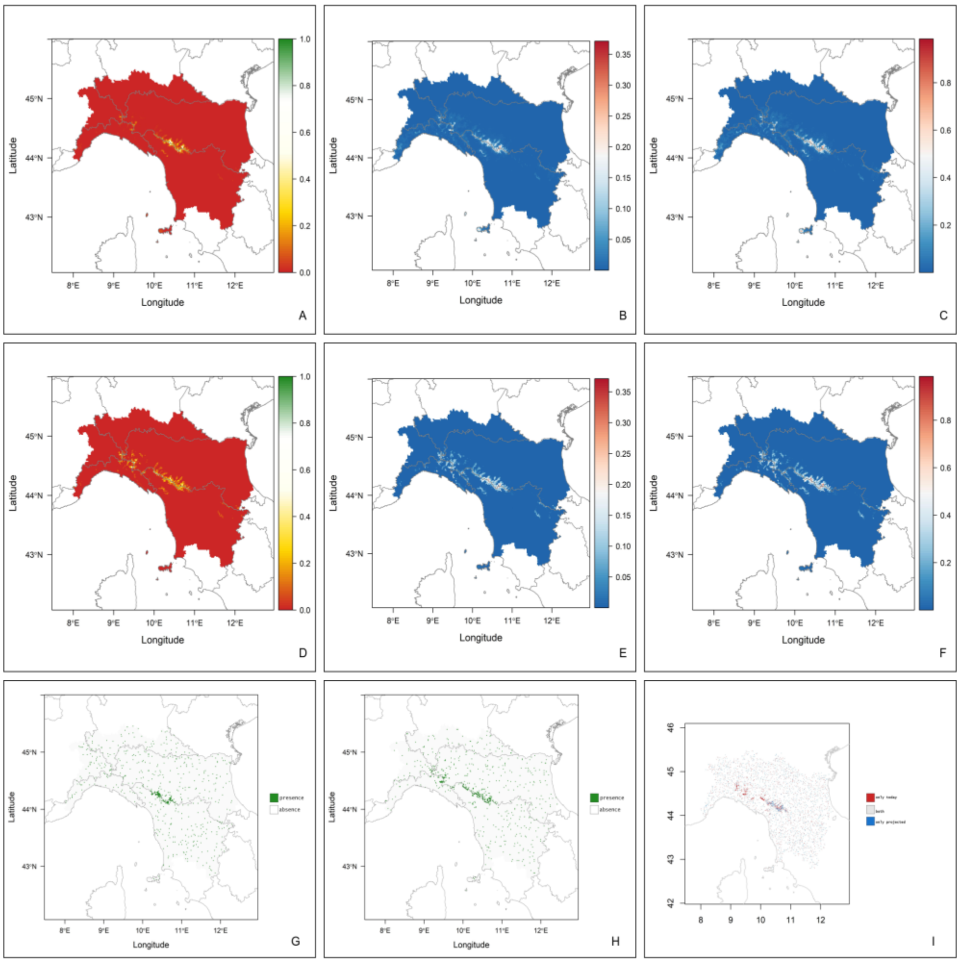

Fig. S.2.298-A) 'Projected' base output; B) 'Projected' uncertainty standard deviation; C) 'Projected' uncertainty range of values; D) 'Present' base output; E) 'Present' uncertainty standard deviation; F) 'Present' uncertainty range of values; G) 'Projected' binary distribution; H) 'Present' binary distribution; I) Range size variation.

Value	Present	Projected
AUC.train	0.996	0.973
AUC.test	0.920	0.901
AUC.diff	0.082	0.082
pROC	1.759	1.693
OR.p10	0.340	0.440
CBI	0.981	0.728
perc.stable	7.778	7.778
range.size	-5.755	-5.755

S.2.299 *Viola ferrarinii* RF

Species: *Viola ferrarinii* Moraldo & Ricceri

Procedure: RF

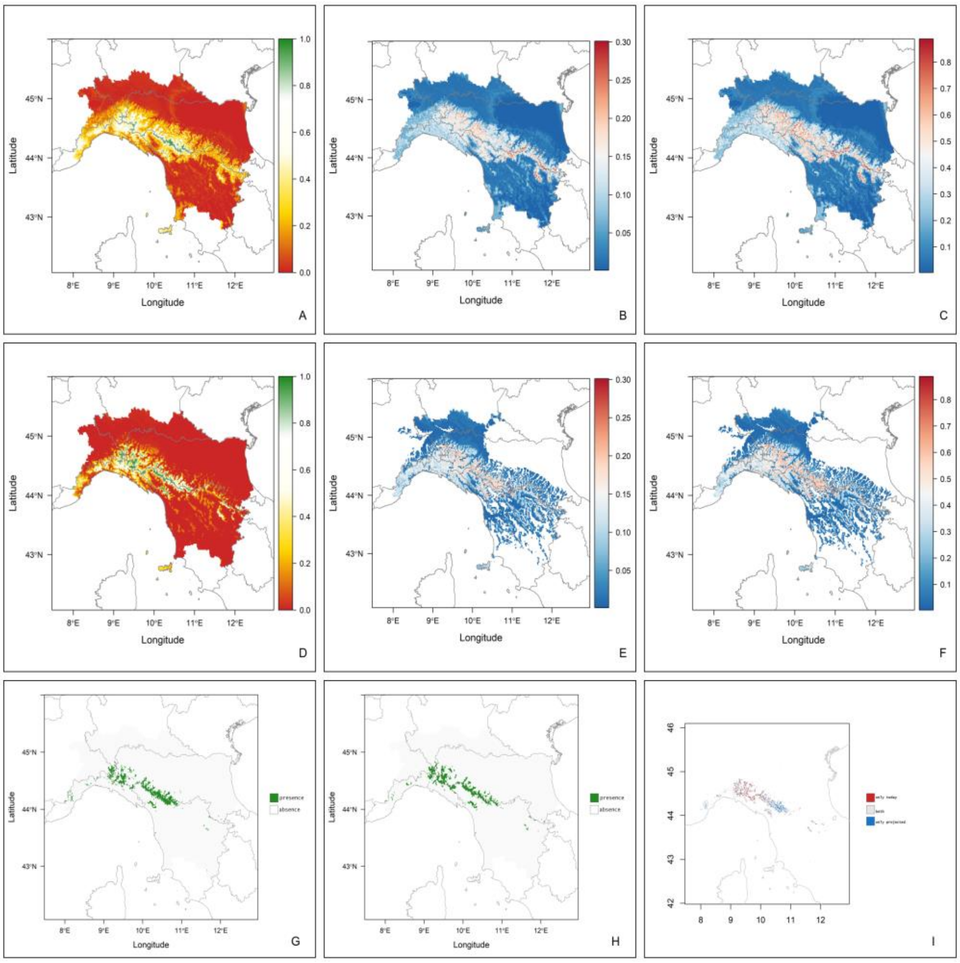

Fig. S.2.299-A) 'Projected' base output; B) 'Projected' uncertainty standard deviation; C) 'Projected' uncertainty range of values; D) 'Present' base output; E) 'Present' uncertainty standard deviation; F) 'Present' uncertainty range of values; G) 'Projected' binary distribution; H) 'Present' binary distribution; I) Range size variation.

Value	Present	Projected
AUC.train	1.000	1.000
AUC.test	1.000	1.000
AUC.diff	0.000	0.000
pROC	1.883	1.925
OR.p10	0.400	0.420
CBI	0.969	0.943
perc.stable	58.767	58.767
range.size	3.473	3.473

S.2.300 *Viola ferrarinii* SVM

Species: *Viola ferrarinii* Moraldo & Ricceri

Procedure: SVM

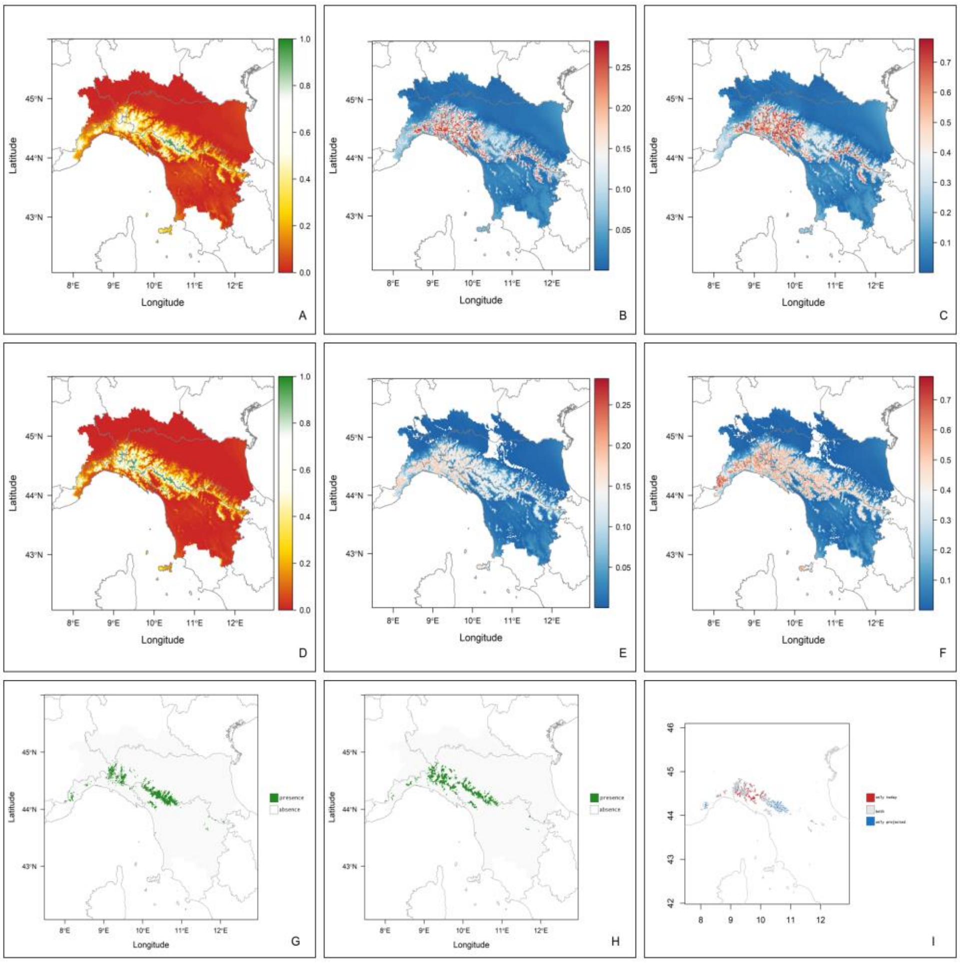

Fig. S.2.300-A) 'Projected' base output; B) 'Projected' uncertainty standard deviation; C) 'Projected' uncertainty range of values; D) 'Present' base output; E) 'Present' uncertainty standard deviation; F) 'Present' uncertainty range of values; G) 'Projected' binary distribution; H) 'Present' binary distribution; I) Range size variation.

Value	Present	Projected
AUC.train	0.995	0.988
AUC.test	0.992	0.973
AUC.diff	0.024	0.024
pROC	1.951	1.848
OR.p10	0.420	0.300
CBI	0.930	0.780
perc.stable	47.471	47.471
range.size	0.930	0.930
