# Supplementary material for: Assessing the Effectiveness of Correlative Ecological Niche Model Temporal Projection through Floristic Data
Source: Biology (Basel). 2022 Aug 14;11(8):1219. doi: 10.3390/biology11081219 (PMC9405103; doi:10.3390/biology11081219)
Supplement: Supplementary file 1 [file biology-11-01219-s001.zip › Dolci & Peruzzi Supplementary Material S.1-9.zip!/Supplementary material S.3.pdf]

Supplementary material S.3

Results of all modeling procedures obtained by using the first three axes of a principal component analysis (PCA) on the 35 bioclimatic variables

S.3.1 *Adenostyles australis* Biomod2

Species: *Adenostyles australis* (Ten.) Iamonico & Pignatti

Procedure: Biomod2

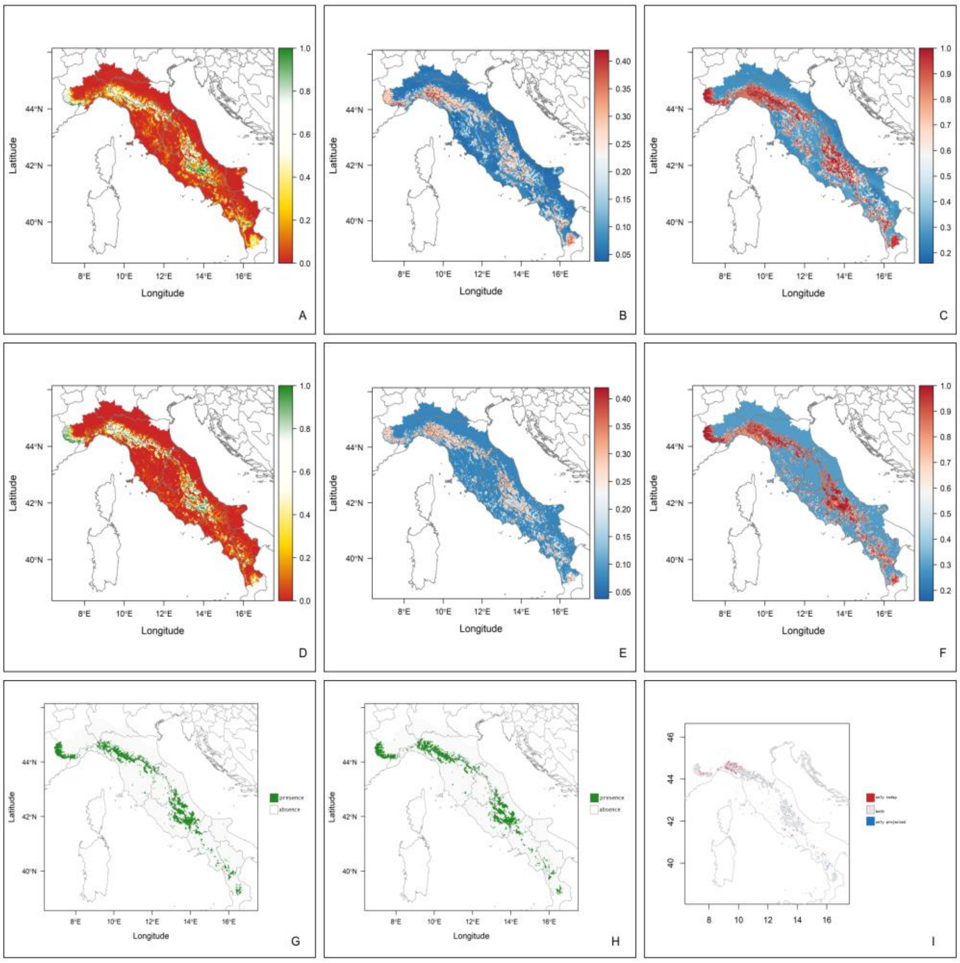

Fig. S.3.1-A) 'Projected' base output; B) 'Projected' uncertainty standard deviation; C) 'Projected' uncertainty range of values; D) 'Present' base output; E) 'Present' uncertainty standard deviation; F) 'Present' uncertainty range of values; G) 'Projected' binary distribution; H) 'Present' binary distribution; I) Range size variation.

Value	Present	Projected
AUC.train	0.942	0.981
AUC.test	0.942	0.901
AUC.diff	0.080	0.080
pROC	1.664	1.502
OR.p10	0.361	0.317
CBI	0.988	0.973
perc.stable	73.613	73.613
range.size	-0.925	-0.925

S.3.2 *Adenostyles australis* Bioclim

Species: *Adenostyles australis* (Ten.) Iamonico & Pignatti

Procedure: Bioclim

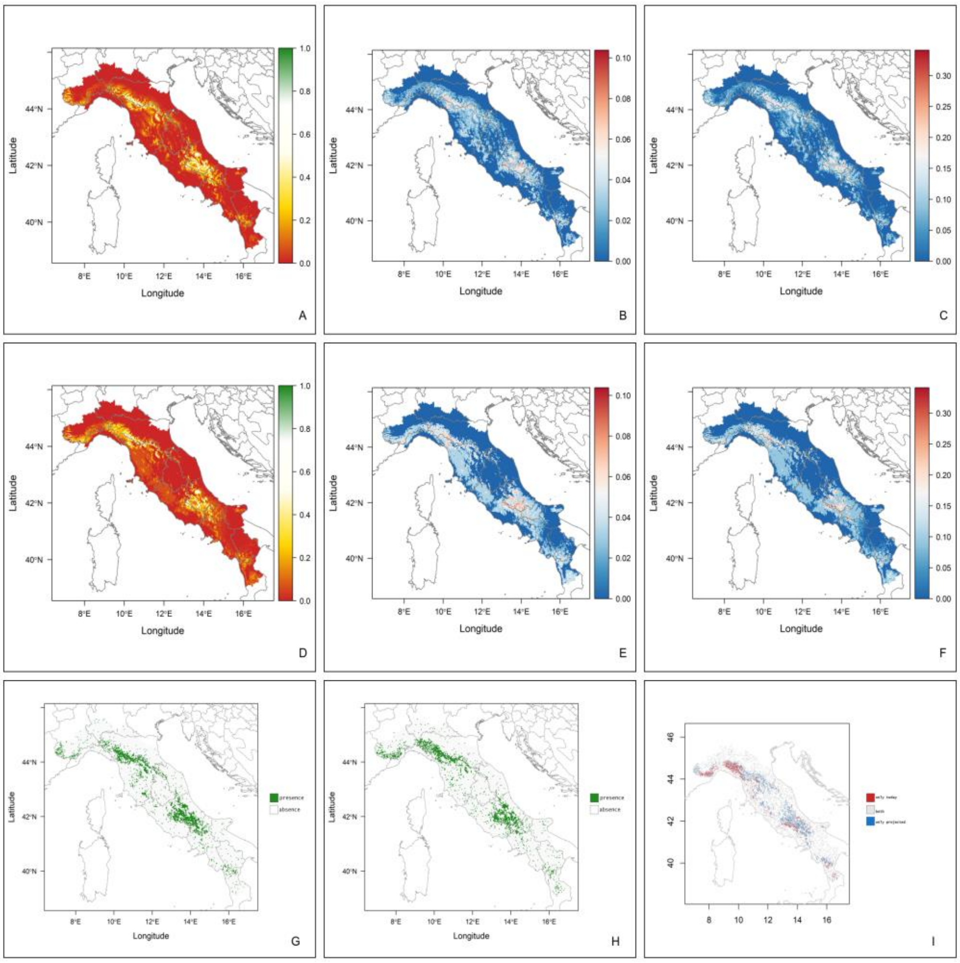

Fig. S.3.2-A) 'Projected' base output; B) 'Projected' uncertainty standard deviation; C) 'Projected' uncertainty range of values; D) 'Present' base output; E) 'Present' uncertainty standard deviation; F) 'Present' uncertainty range of values; G) 'Projected' binary distribution; H) 'Present' binary distribution; I) Range size variation.

Value	Present	Projected
AUC.train	0.799	0.883
AUC.test	0.799	0.847
AUC.diff	0.040	0.040
pROC	1.157	1.226
OR.p10	0.172	0.172
CBI	0.302	0.438
perc.stable	32.243	32.243
range.size	1.519	1.519

S.3.3 *Adenostyles australis* Domain

Species: *Adenostyles australis* (Ten.) Iamonico & Pignatti

Procedure: Domain

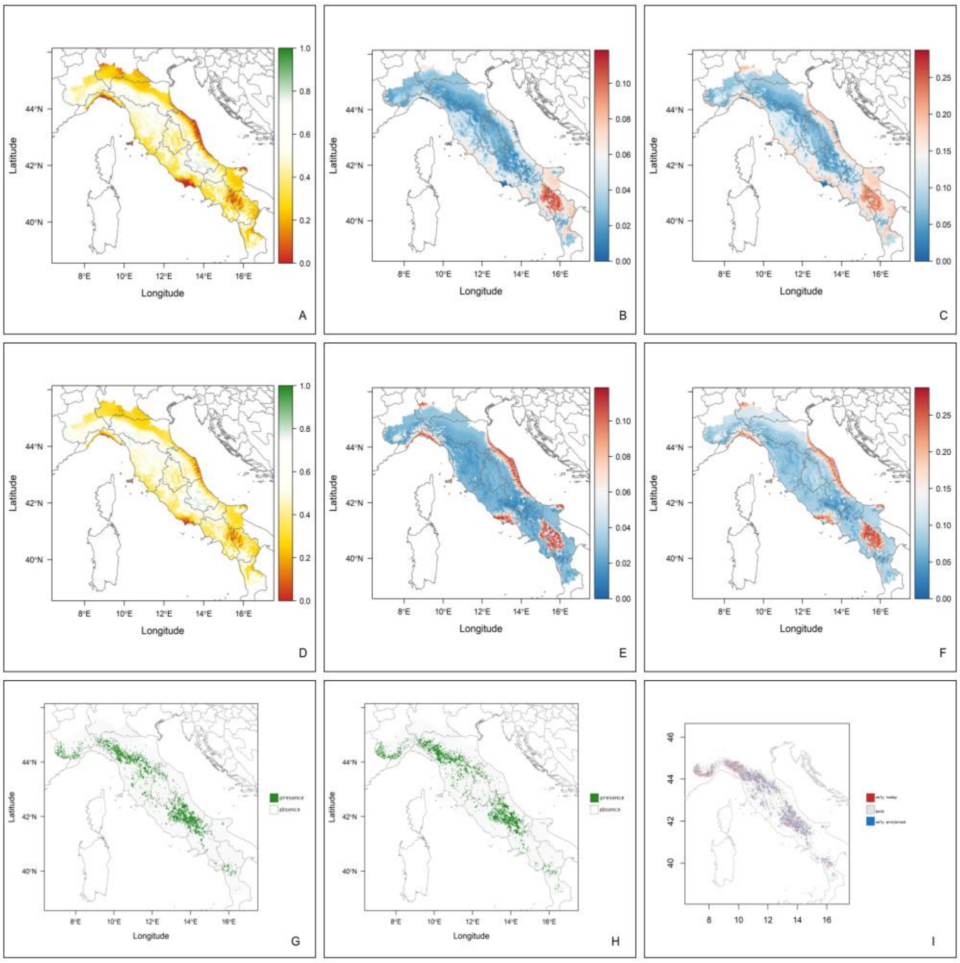

Fig. S.3.3-A) 'Projected' base output; B) 'Projected' uncertainty standard deviation; C) 'Projected' uncertainty range of values; D) 'Present' base output; E) 'Present' uncertainty standard deviation; F) 'Present' uncertainty range of values; G) 'Projected' binary distribution; H) 'Present' binary distribution; I) Range size variation.

Value	Present	Projected
AUC.train	0.822	0.886
AUC.test	0.822	0.834
AUC.diff	0.052	0.052
pROC	1.342	1.348
OR.p10	0.150	0.189
CBI	0.946	0.928
perc.stable	34.761	34.761
range.size	-0.573	-0.573

S.3.4 *Adenostyles australis* Maxent

Species: *Adenostyles australis* (Ten.) Iamonico & Pignatti

Procedure: Maxent

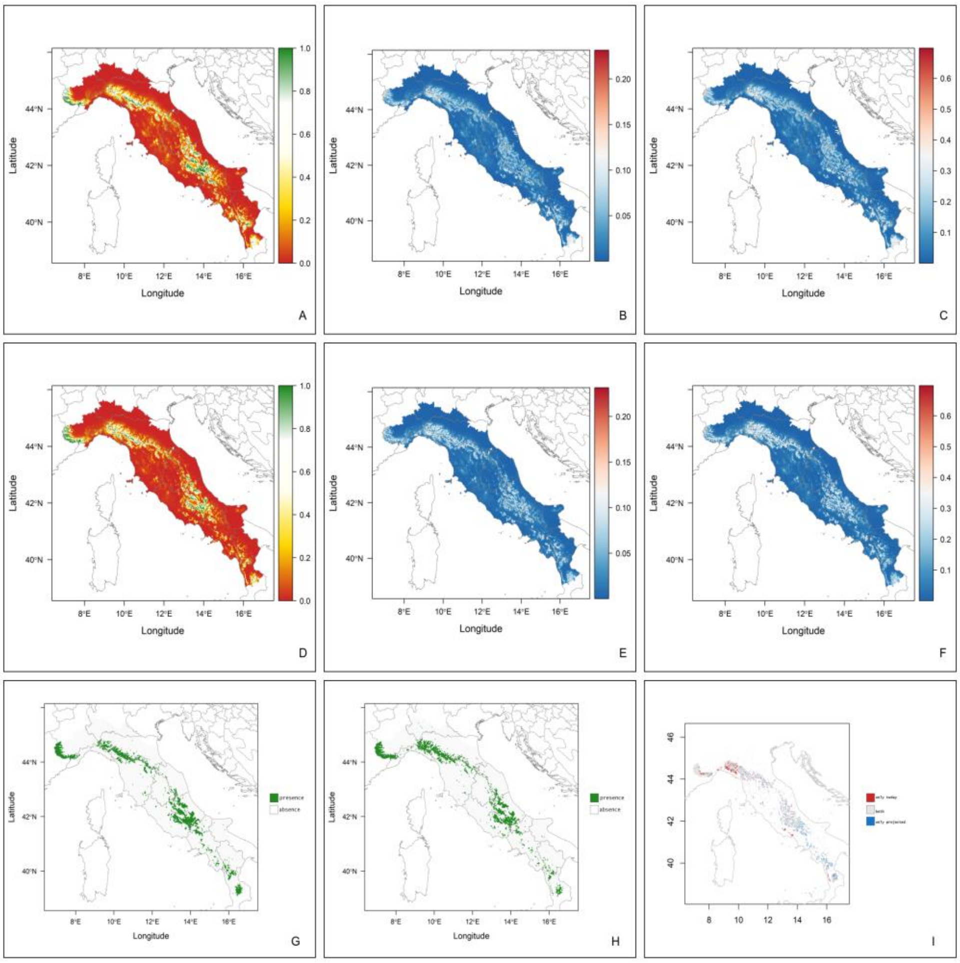

Fig. S.3.4-A) 'Projected' base output; B) 'Projected' uncertainty standard deviation; C) 'Projected' uncertainty range of values; D) 'Present' base output; E) 'Present' uncertainty standard deviation; F) 'Present' uncertainty range of values; G) 'Projected' binary distribution; H) 'Present' binary distribution; I) Range size variation.

Value	Present	Projected
AUC.train	0.923	0.919
AUC.test	0.923	0.919
AUC.diff	0.012	0.012
pROC	1.593	1.566
OR.p10	0.128	0.111
CBI	0.967	0.986
perc.stable	67.134	67.134
range.size	-0.938	-0.938

S.3.5 *Adenostyles australis* KDE

Species: *Adenostyles australis* (Ten.) Iamonico & Pignatti

Procedure: KDE

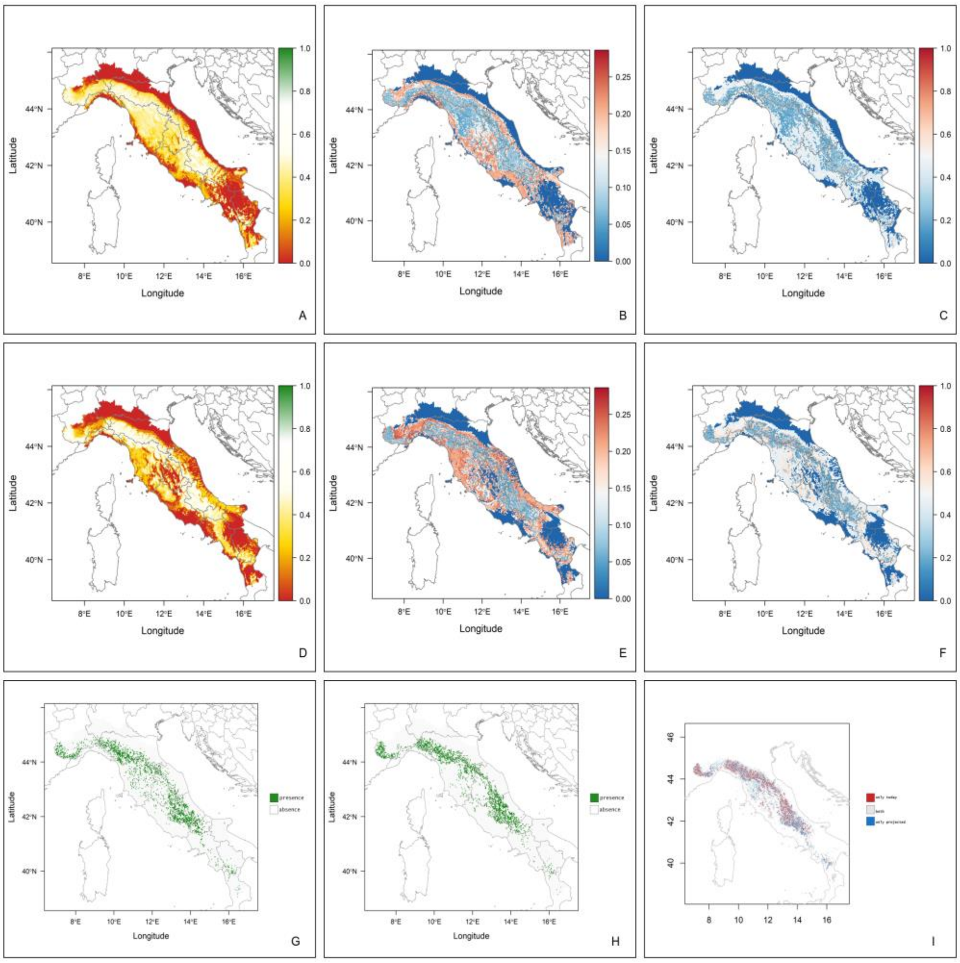

Fig. S.3.5-A) 'Projected' base output; B) 'Projected' uncertainty standard deviation; C) 'Projected' uncertainty range of values; D) 'Present' base output; E) 'Present' uncertainty standard deviation; F) 'Present' uncertainty range of values; G) 'Projected' binary distribution; H) 'Present' binary distribution; I) Range size variation.

Value	Present	Projected
AUC.train	0.862	0.908
AUC.test	0.862	0.872
AUC.diff	0.034	0.037
pROC	1.397	1.369
OR.p10	0.150	0.178
CBI	0.931	0.778
perc.stable	25.031	25.031
range.size	-1.532	-1.532

S.3.6 *Adenostyles australis* CTA

Species: *Adenostyles australis* (Ten.) Iamonico & Pignatti

Procedure: CTA

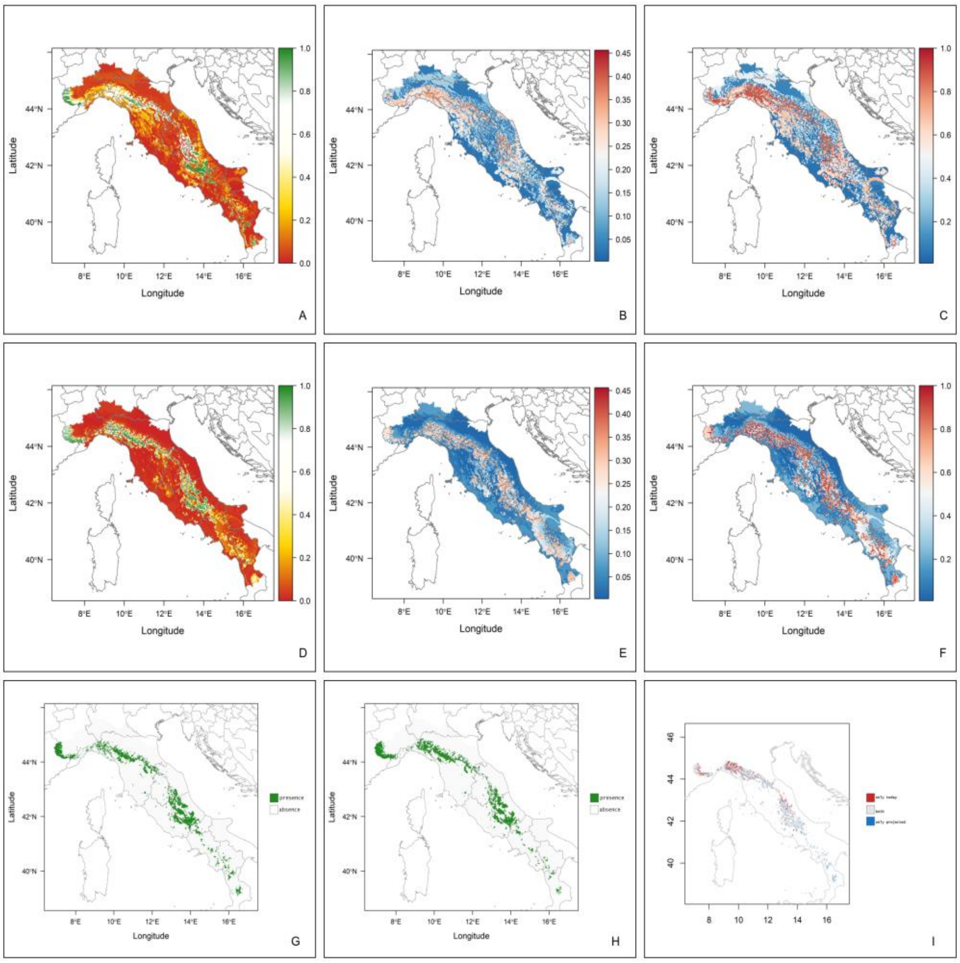

Fig. S.3.6-A) 'Projected' base output; B) 'Projected' uncertainty standard deviation; C) 'Projected' uncertainty range of values; D) 'Present' base output; E) 'Present' uncertainty standard deviation; F) 'Present' uncertainty range of values; G) 'Projected' binary distribution; H) 'Present' binary distribution; I) Range size variation.

Value	Present	Projected
AUC.train	0.908	0.941
AUC.test	0.908	0.909
AUC.diff	0.035	0.035
pROC	1.309	1.329
OR.p10	0.206	0.172
CBI	0.945	0.946
perc.stable	63.908	63.908
range.size	0.445	0.445

S.3.7 *Adenostyles australis* FDA

Species: *Adenostyles australis* (Ten.) Iamonico & Pignatti

Procedure: FDA

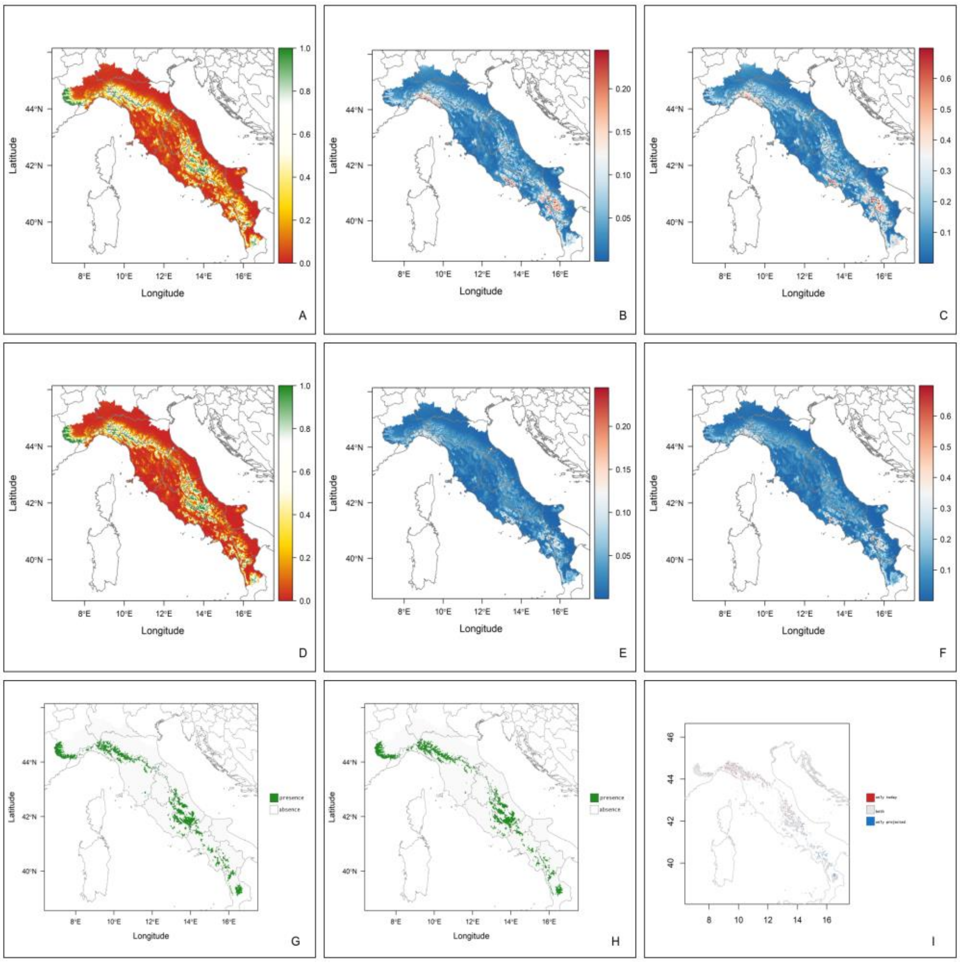

Fig. S.3.7-A) 'Projected' base output; B) 'Projected' uncertainty standard deviation; C) 'Projected' uncertainty range of values; D) 'Present' base output; E) 'Present' uncertainty standard deviation; F) 'Present' uncertainty range of values; G) 'Projected' binary distribution; H) 'Present' binary distribution; I) Range size variation.

Value	Present	Projected
AUC.train	0.916	0.912
AUC.test	0.916	0.898
AUC.diff	0.023	0.023
pROC	1.565	1.503
OR.p10	0.156	0.150
CBI	0.916	0.939
perc.stable	77.281	77.281
range.size	-0.072	-0.072

S.3.8 *Adenostyles australis* GAM

Species: *Adenostyles australis* (Ten.) Iamonico & Pignatti

Procedure: GAM

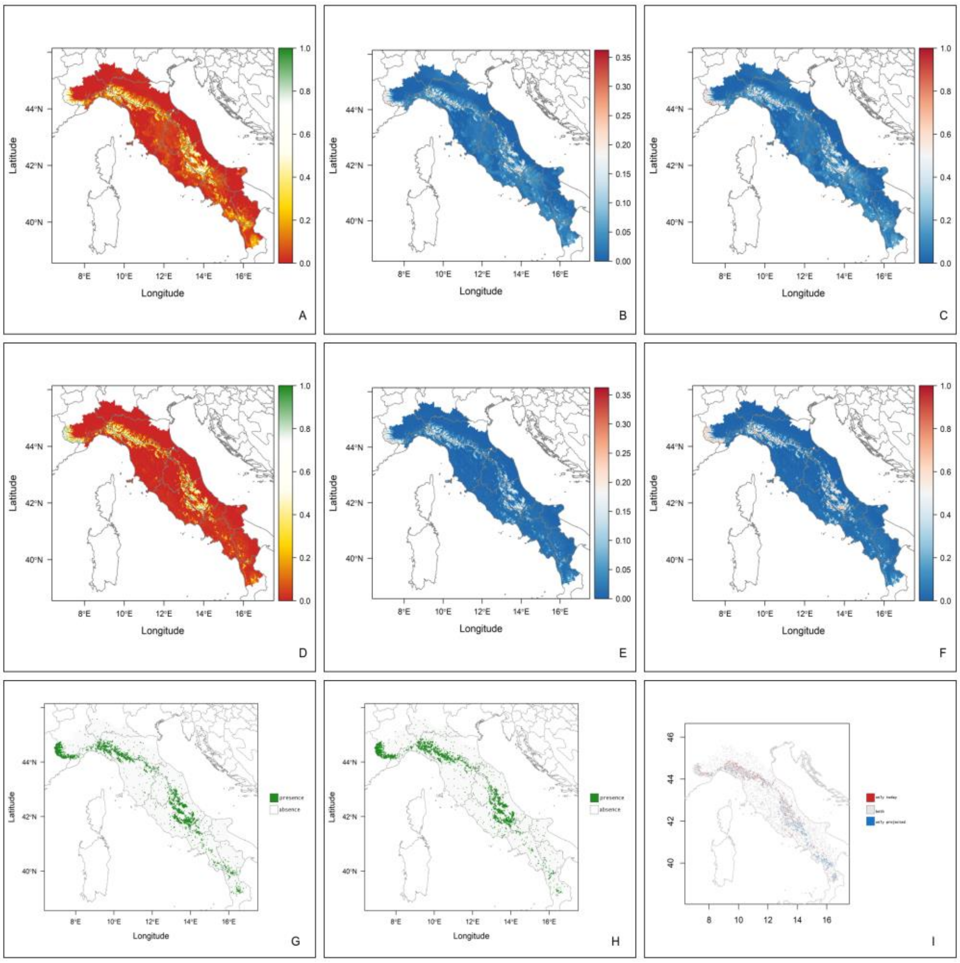

Fig. S.3.8-A) 'Projected' base output; B) 'Projected' uncertainty standard deviation; C) 'Projected' uncertainty range of values; D) 'Present' base output; E) 'Present' uncertainty standard deviation; F) 'Present' uncertainty range of values; G) 'Projected' binary distribution; H) 'Present' binary distribution; I) Range size variation.

Value	Present	Projected
AUC.train	0.928	0.906
AUC.test	0.928	0.863
AUC.diff	0.046	0.046
pROC	1.608	1.328
OR.p10	0.150	0.178
CBI	0.966	0.987
perc.stable	53.476	53.476
range.size	-1.356	-1.356

S.3.9 *Adenostyles australis* GLM

Species: *Adenostyles australis* (Ten.) Iamonico & Pignatti

Procedure: GLM

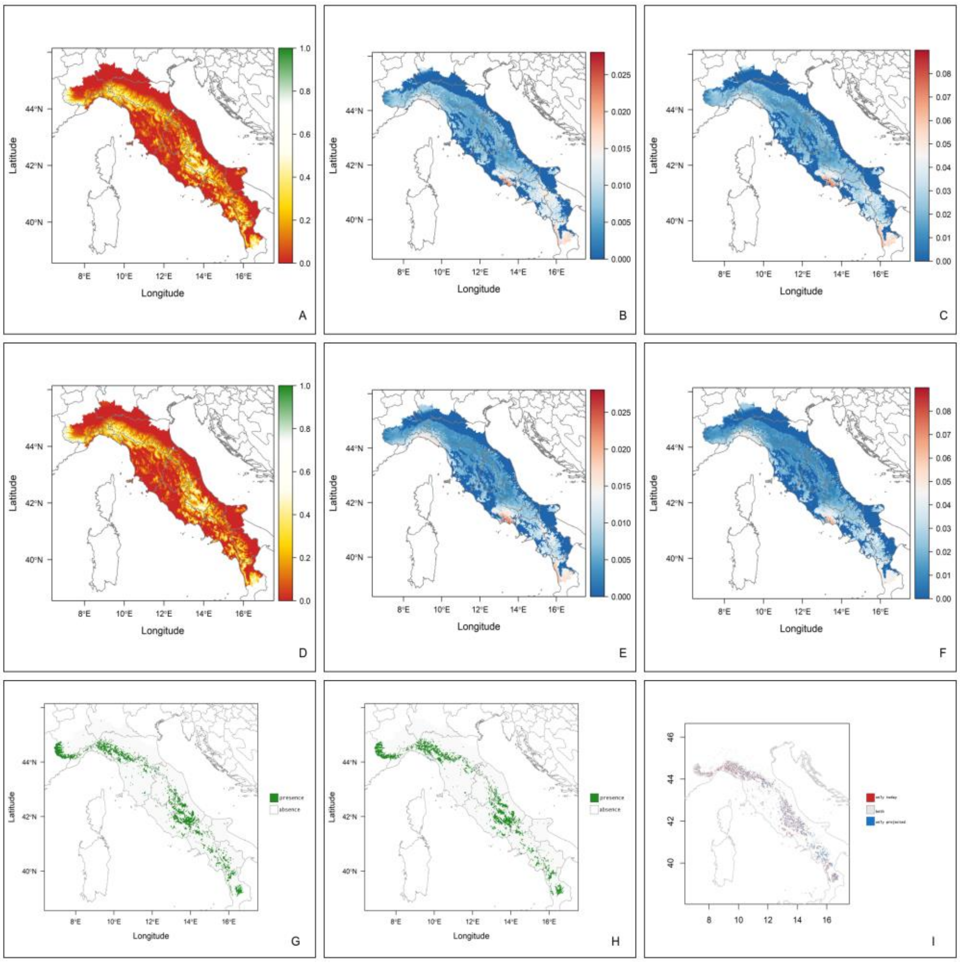

Fig. S.3.9-A) 'Projected' base output; B) 'Projected' uncertainty standard deviation; C) 'Projected' uncertainty range of values; D) 'Present' base output; E) 'Present' uncertainty standard deviation; F) 'Present' uncertainty range of values; G) 'Projected' binary distribution; H) 'Present' binary distribution; I) Range size variation.

Value	Present	Projected
AUC.train	0.920	0.919
AUC.test	0.920	0.876
AUC.diff	0.045	0.045
pROC	1.557	1.440
OR.p10	0.133	0.250
CBI	0.778	0.755
perc.stable	50.421	50.421
range.size	-0.154	-0.154

S.3.10 *Adenostyles australis* MARS

Species: *Adenostyles australis* (Ten.) Iamonico & Pignatti

Procedure: MARS

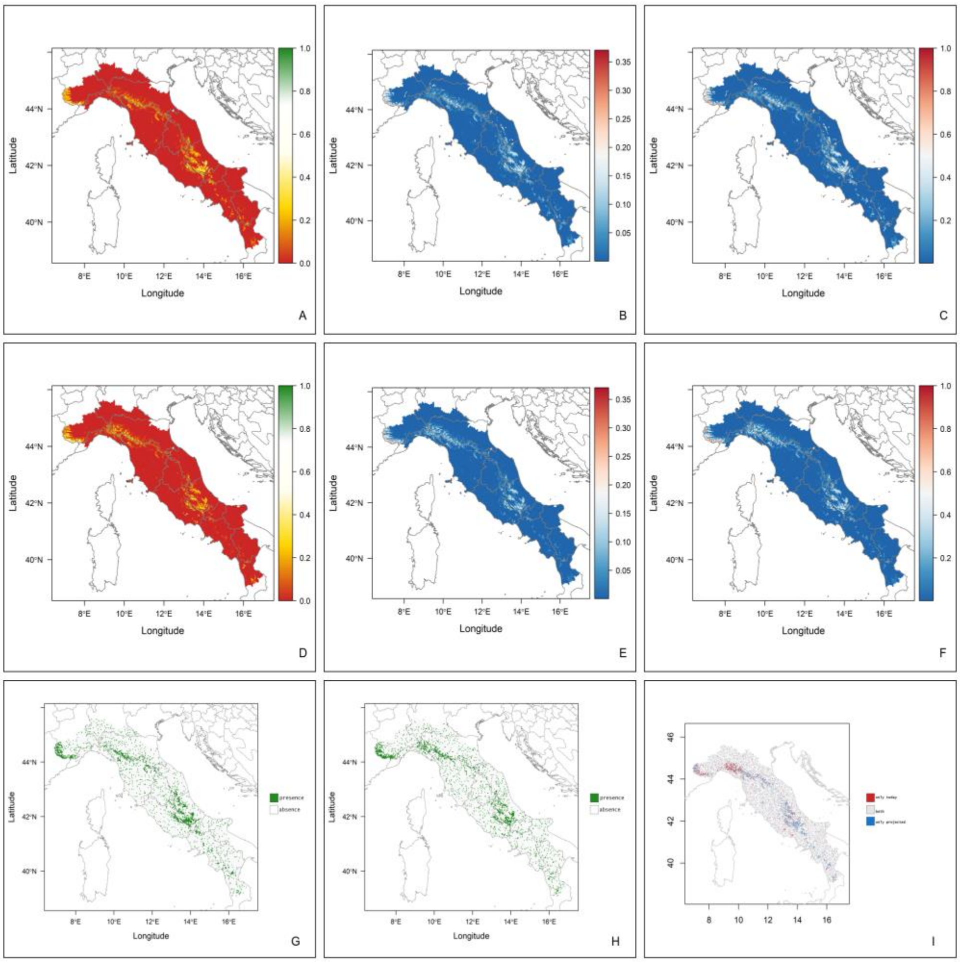

Fig. S.3.10-A) 'Projected' base output; B) 'Projected' uncertainty standard deviation; C) 'Projected' uncertainty range of values; D) 'Present' base output; E) 'Present' uncertainty standard deviation; F) 'Present' uncertainty range of values; G) 'Projected' binary distribution; H) 'Present' binary distribution; I) Range size variation.

Value	Present	Projected
AUC.train	0.865	0.911
AUC.test	0.865	0.869
AUC.diff	0.053	0.053
pROC	1.304	1.333
OR.p10	0.178	0.183
CBI	0.802	0.772
perc.stable	17.553	17.553
range.size	-0.771	-0.771

S.3.11 *Adenostyles australis* RF

Species: *Adenostyles australis* (Ten.) Iamonico & Pignatti

Procedure: RF

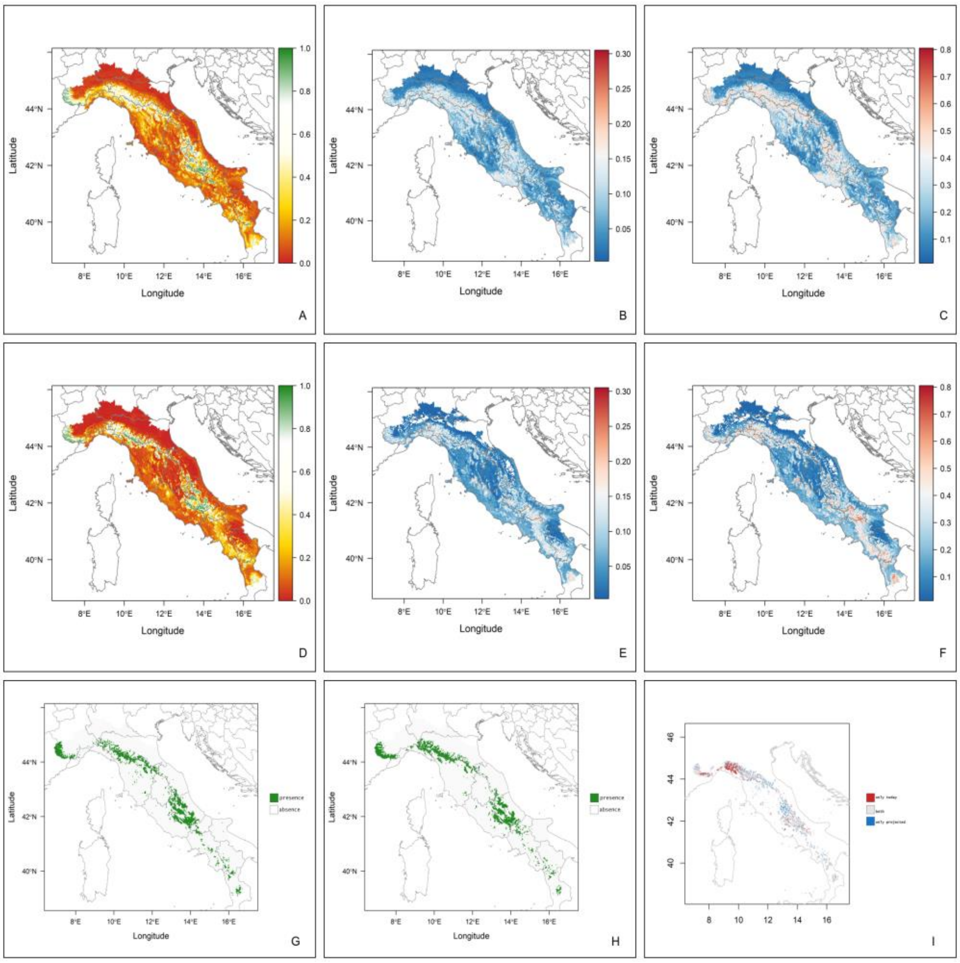

Fig. S.3.11-A) 'Projected' base output; B) 'Projected' uncertainty standard deviation; C) 'Projected' uncertainty range of values; D) 'Present' base output; E) 'Present' uncertainty standard deviation; F) 'Present' uncertainty range of values; G) 'Projected' binary distribution; H) 'Present' binary distribution; I) Range size variation.

Value	Present	Projected
AUC.train	0.982	1.000
AUC.test	0.982	0.959
AUC.diff	0.041	0.041
pROC	1.626	1.458
OR.p10	0.394	0.372
CBI	0.974	0.978
perc.stable	59.793	59.793
range.size	0.126	0.126

S.3.12 *Adenostyles australis* SVM

Species: *Adenostyles australis* (Ten.) Iamonico & Pignatti

Procedure: SVM

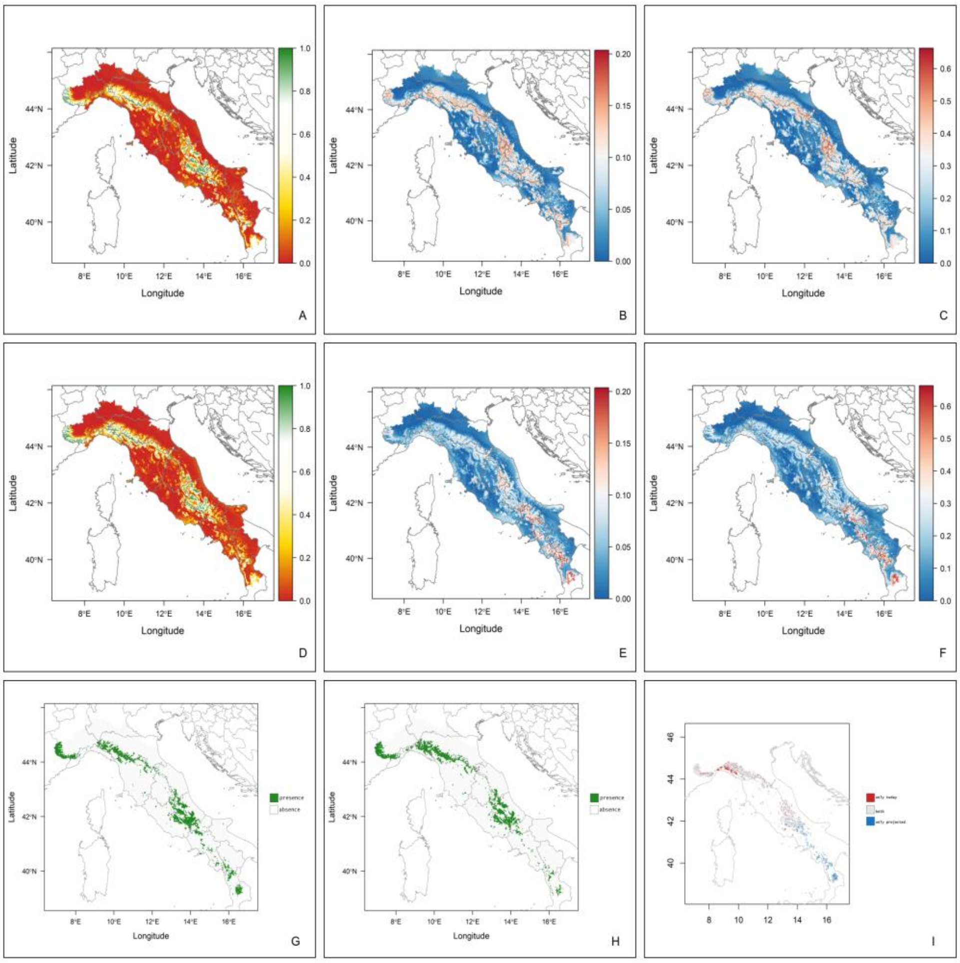

Fig. S.3.12-A) 'Projected' base output; B) 'Projected' uncertainty standard deviation; C) 'Projected' uncertainty range of values; D) 'Present' base output; E) 'Present' uncertainty standard deviation; F) 'Present' uncertainty range of values; G) 'Projected' binary distribution; H) 'Present' binary distribution; I) Range size variation.

Value	Present	Projected
AUC.train	0.942	0.919
AUC.test	0.942	0.915
AUC.diff	0.014	0.014
pROC	1.615	1.471
OR.p10	0.206	0.117
CBI	0.961	0.993
perc.stable	67.314	67.314
range.size	0.230	0.230

S.3.13 *Aubrieta columnae* Biomod2

Species: *Aubrieta columnae* Guss. subsp. *columnae*

Procedure: Biomod2

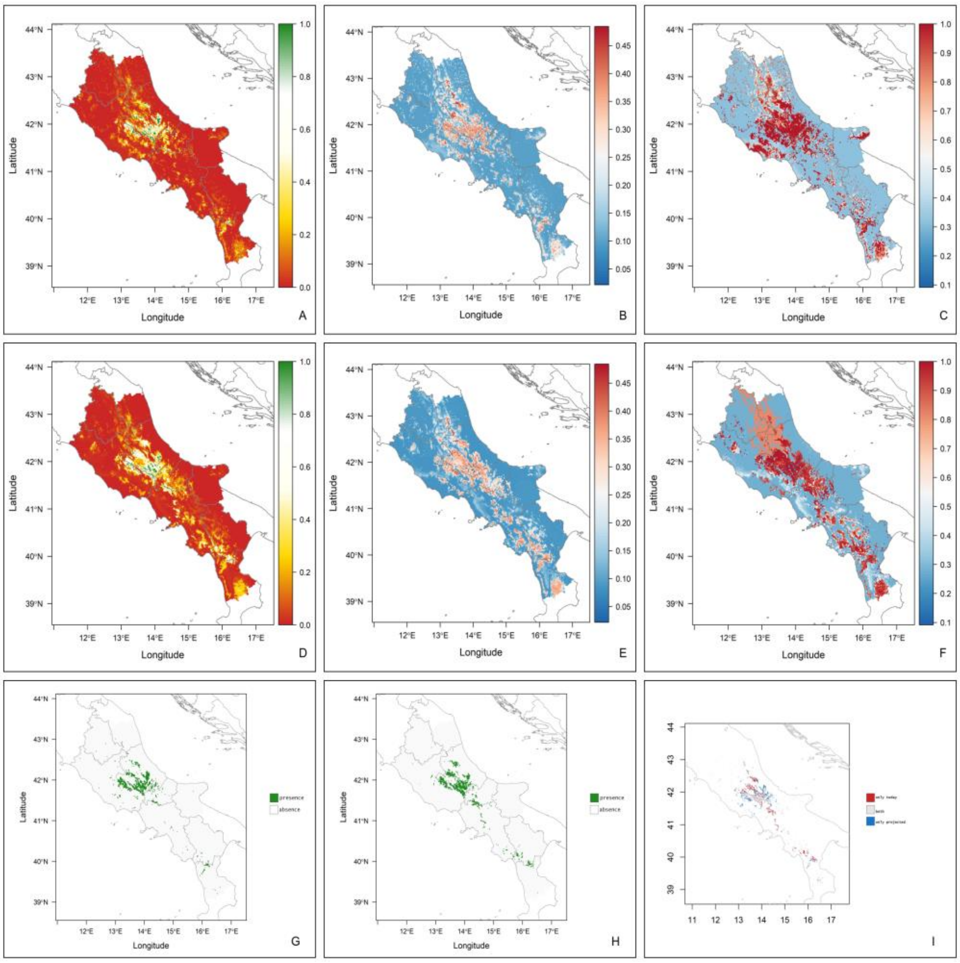

Fig. S.3.13-A) 'Projected' base output; B) 'Projected' uncertainty standard deviation; C) 'Projected' uncertainty range of values; D) 'Present' base output; E) 'Present' uncertainty standard deviation; F) 'Present' uncertainty range of values; G) 'Projected' binary distribution; H) 'Present' binary distribution; I) Range size variation.

Value	Present	Projected
AUC.train	0.969	0.998
AUC.test	0.969	0.958
AUC.diff	0.041	0.041
pROC	1.868	1.817
OR.p10	0.683	0.500
CBI	0.934	0.882
perc.stable	41.889	41.889
range.size	0.085	0.085

S.3.14 *Aubrieta columnae* Bioclim

Species: *Aubrieta columnae* Guss. subsp. *columnae*

Procedure: Bioclim

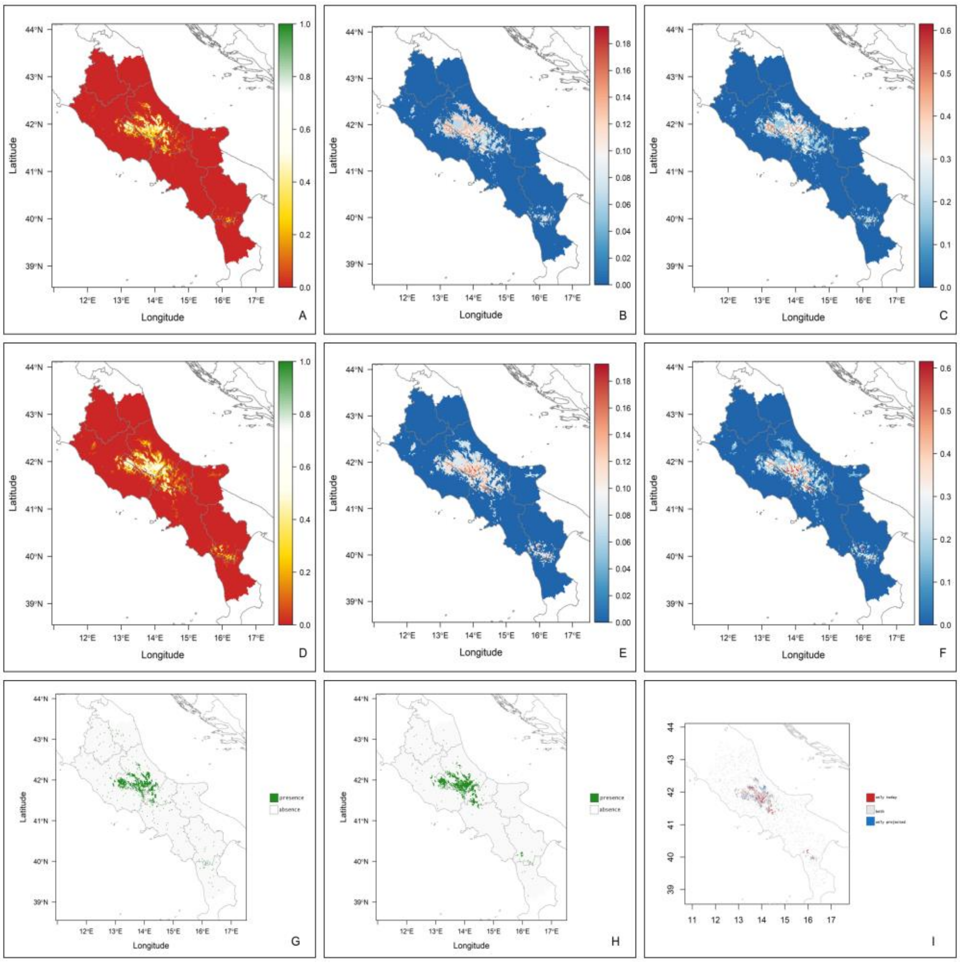

Fig. S.3.14-A) 'Projected' base output; B) 'Projected' uncertainty standard deviation; C) 'Projected' uncertainty range of values; D) 'Present' base output; E) 'Present' uncertainty standard deviation; F) 'Present' uncertainty range of values; G) 'Projected' binary distribution; H) 'Present' binary distribution; I) Range size variation.

Value	Present	Projected
AUC.train	0.775	0.962
AUC.test	0.775	0.693
AUC.diff	0.269	0.269
pROC	1.302	1.130
OR.p10	0.367	0.550
CBI	0.586	0.621
perc.stable	45.362	45.362
range.size	-0.335	-0.335

S.3.15 *Aubrieta columnae* Domain

Species: *Aubrieta columnae* Guss. subsp. *columnae*

Procedure: Domain

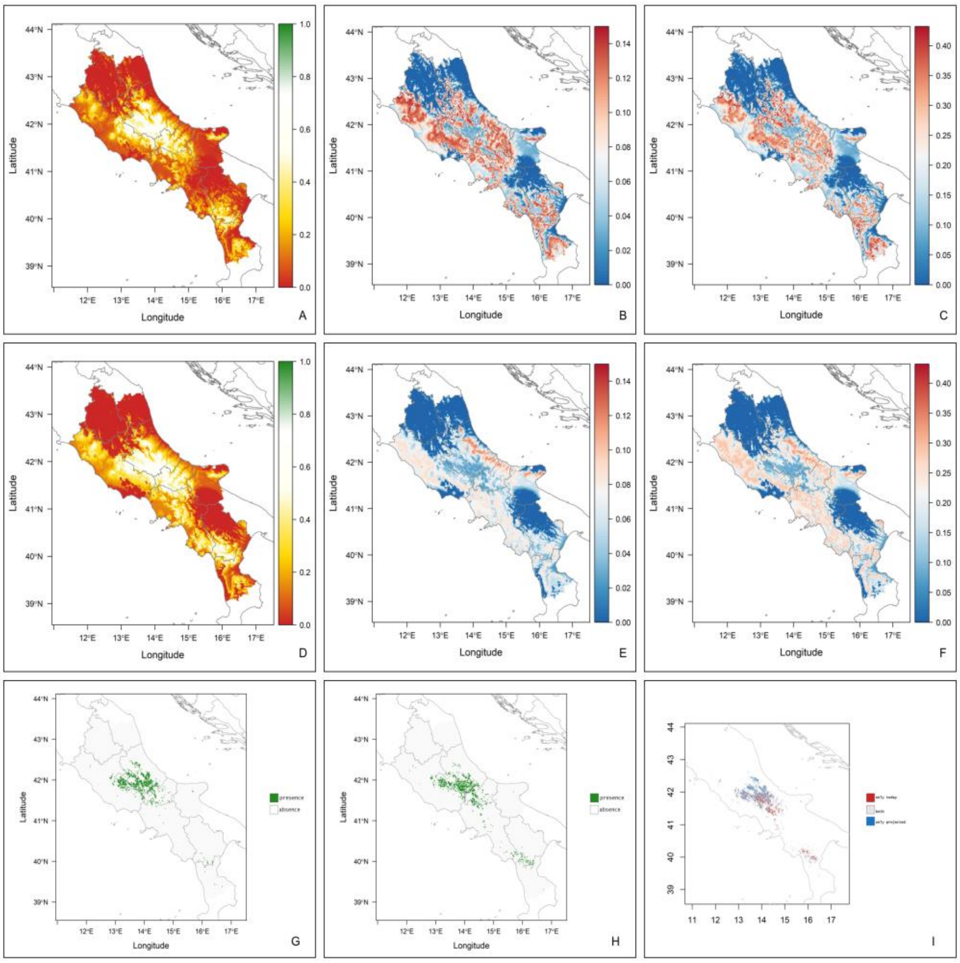

Fig. S.3.15-A) 'Projected' base output; B) 'Projected' uncertainty standard deviation; C) 'Projected' uncertainty range of values; D) 'Present' base output; E) 'Present' uncertainty standard deviation; F) 'Present' uncertainty range of values; G) 'Projected' binary distribution; H) 'Present' binary distribution; I) Range size variation.

Value	Present	Projected
AUC.train	0.934	0.952
AUC.test	0.934	0.930
AUC.diff	0.023	0.023
pROC	1.792	1.799
OR.p10	0.150	0.317
CBI	0.948	0.945
perc.stable	29.006	29.006
range.size	1.463	1.463

S.3.16 *Aubrieta columnae* Maxent

Species: *Aubrieta columnae* Guss. subsp. *columnae*

Procedure: Maxent

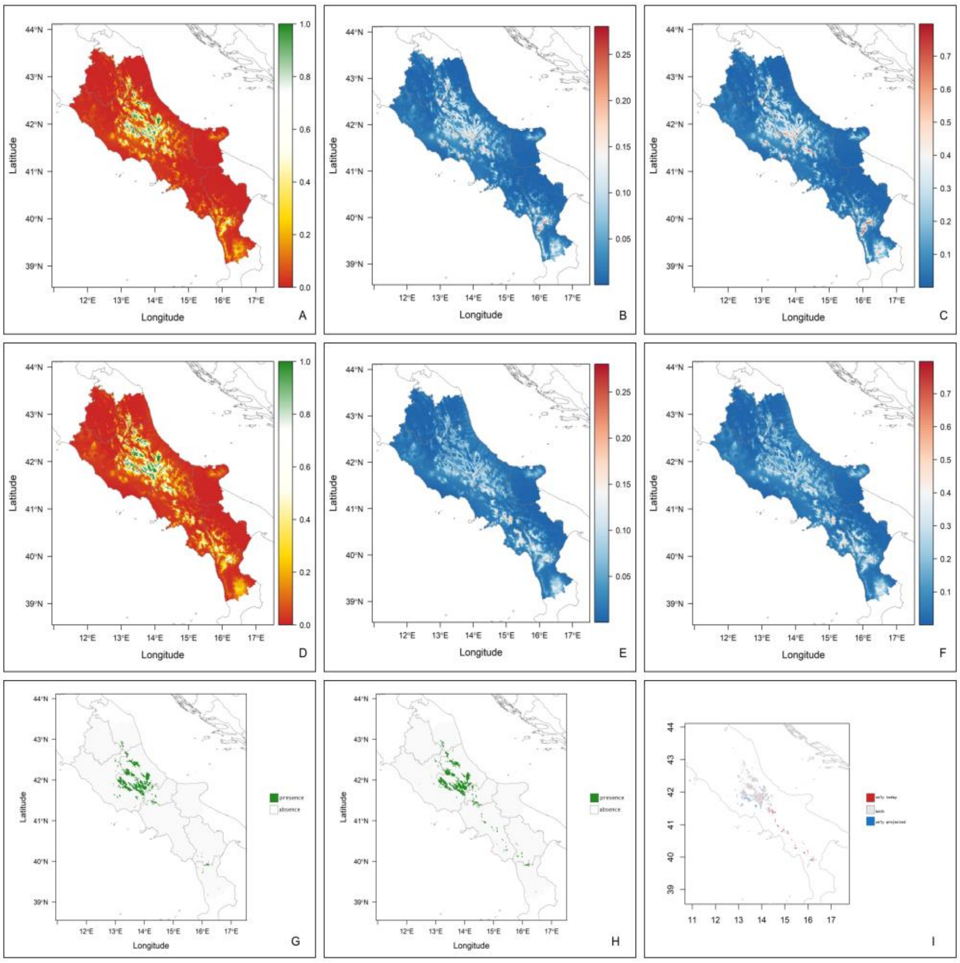

Fig. S.3.16-A) 'Projected' base output; B) 'Projected' uncertainty standard deviation; C) 'Projected' uncertainty range of values; D) 'Present' base output; E) 'Present' uncertainty standard deviation; F) 'Present' uncertainty range of values; G) 'Projected' binary distribution; H) 'Present' binary distribution; I) Range size variation.

Value	Present	Projected
AUC.train	0.941	0.958
AUC.test	0.941	0.932
AUC.diff	0.036	0.036
pROC	1.810	1.756
OR.p10	0.300	0.300
CBI	0.897	0.876
perc.stable	67.541	67.541
range.size	-1.660	-1.660

S.3.17 *Aubrieta columnae* KDE

Species: *Aubrieta columnae* Guss. subsp. *columnae*

Procedure: KDE

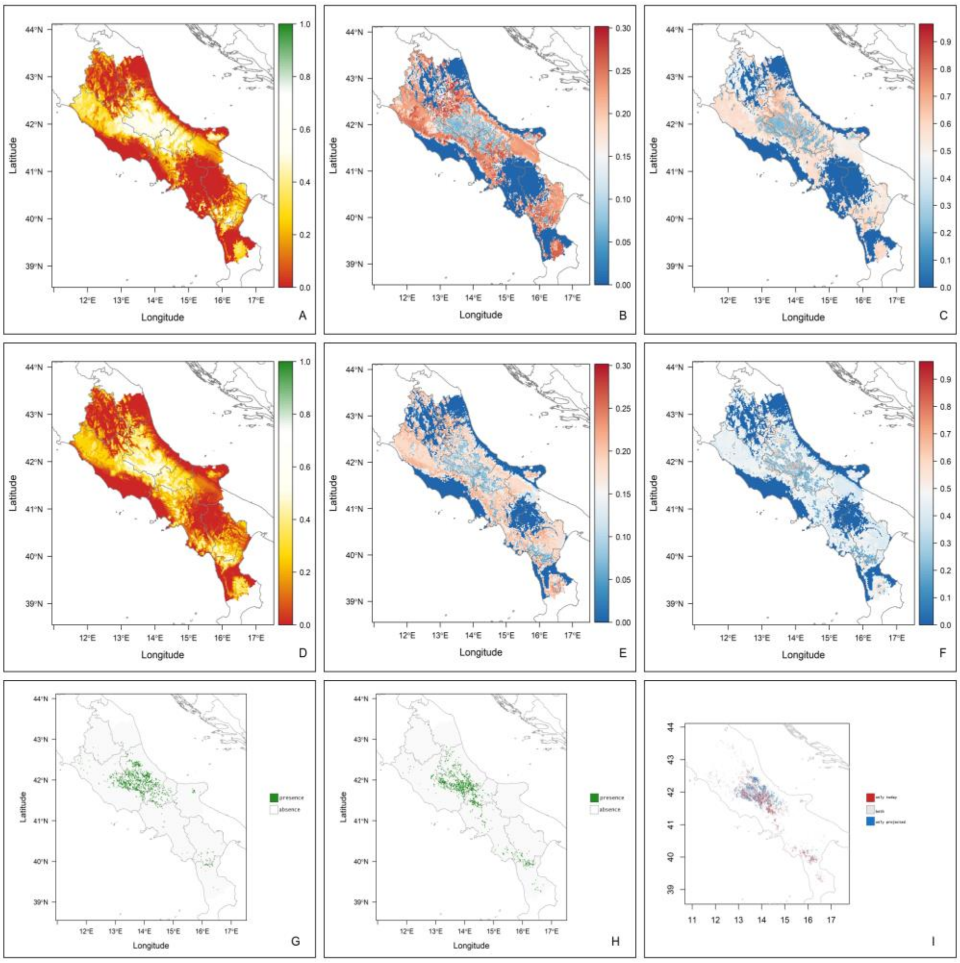

Fig. S.3.17-A) 'Projected' base output; B) 'Projected' uncertainty standard deviation; C) 'Projected' uncertainty range of values; D) 'Present' base output; E) 'Present' uncertainty standard deviation; F) 'Present' uncertainty range of values; G) 'Projected' binary distribution; H) 'Present' binary distribution; I) Range size variation.

Value	Present	Projected
AUC.train	0.884	0.953
AUC.test	0.884	0.932
AUC.diff	0.058	0.033
pROC	1.655	1.783
OR.p10	0.500	0.383
CBI	0.953	0.923
perc.stable	17.056	17.056
range.size	-0.322	-0.322

S.3.18 *Aubrieta columnae* CTA

Species: *Aubrieta columnae* Guss. subsp. *columnae*

Procedure: CTA

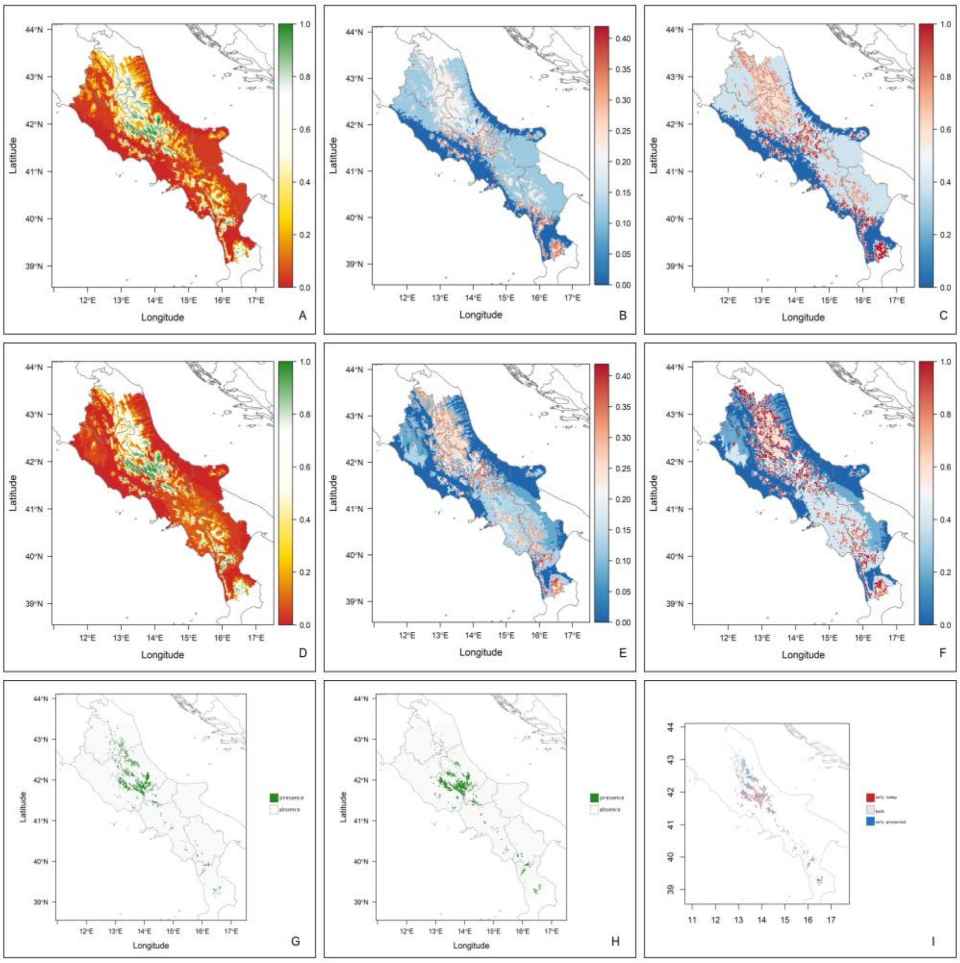

Fig. S.3.18-A) 'Projected' base output; B) 'Projected' uncertainty standard deviation; C) 'Projected' uncertainty range of values; D) 'Present' base output; E) 'Present' uncertainty standard deviation; F) 'Present' uncertainty range of values; G) 'Projected' binary distribution; H) 'Present' binary distribution; I) Range size variation.

Value	Present	Projected
AUC.train	0.913	0.958
AUC.test	0.913	0.940
AUC.diff	0.029	0.029
pROC	1.313	1.322
OR.p10	0.133	0.067
CBI	0.861	0.844
perc.stable	36.414	36.414
range.size	-1.754	-1.754

S.3.19 *Aubrieta columnae* FDA

Species: *Aubrieta columnae* Guss. subsp. *columnae*

Procedure: FDA

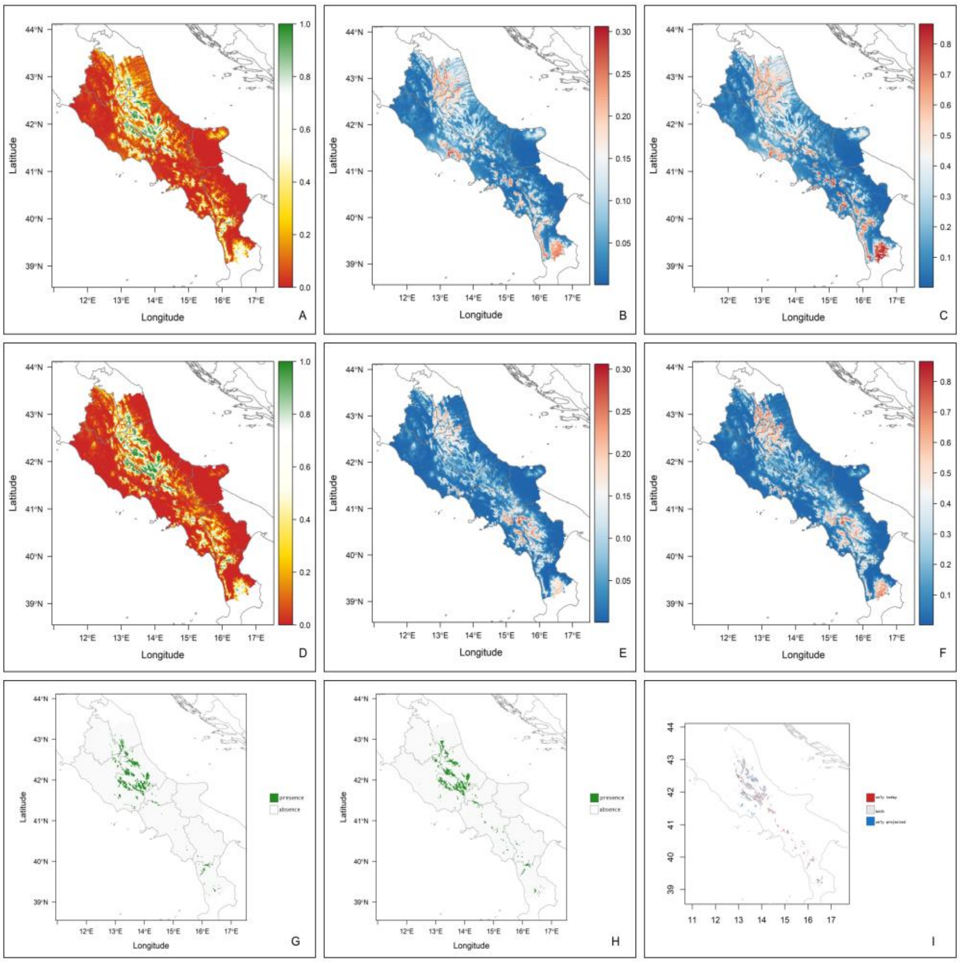

Fig. S.3.19-A) 'Projected' base output; B) 'Projected' uncertainty standard deviation; C) 'Projected' uncertainty range of values; D) 'Present' base output; E) 'Present' uncertainty standard deviation; F) 'Present' uncertainty range of values; G) 'Projected' binary distribution; H) 'Present' binary distribution; I) Range size variation.

Value	Present	Projected
AUC.train	0.900	0.931
AUC.test	0.900	0.935
AUC.diff	0.027	0.027
pROC	1.713	1.767
OR.p10	0.350	0.217
CBI	0.794	0.762
perc.stable	51.959	51.959
range.size	-0.194	-0.194

S.3.20 *Aubrieta columnae* GAM

Species: *Aubrieta columnae* Guss. subsp. *columnae*

Procedure: GAM

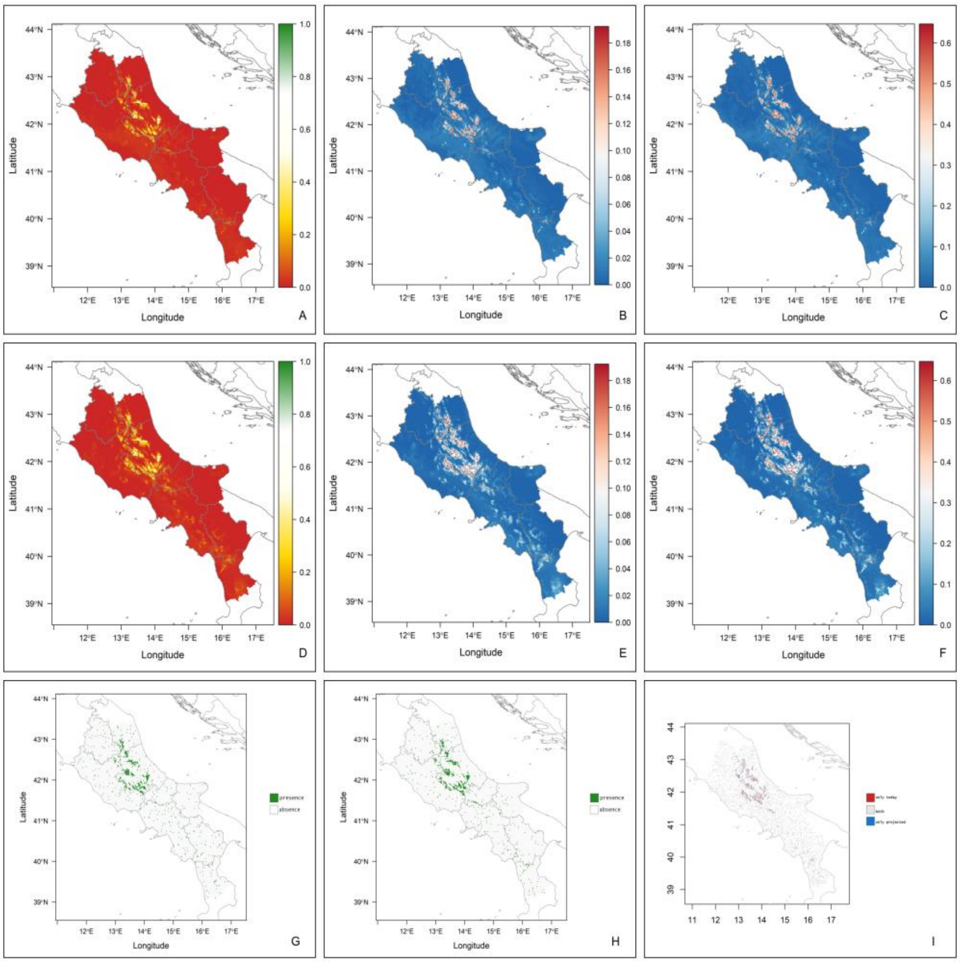

Fig. S.3.20-A) 'Projected' base output; B) 'Projected' uncertainty standard deviation; C) 'Projected' uncertainty range of values; D) 'Present' base output; E) 'Present' uncertainty standard deviation; F) 'Present' uncertainty range of values; G) 'Projected' binary distribution; H) 'Present' binary distribution; I) Range size variation.

Value	Present	Projected
AUC.train	0.910	0.934
AUC.test	0.910	0.821
AUC.diff	0.118	0.118
pROC	1.721	1.511
OR.p10	0.217	0.400
CBI	0.858	0.834
perc.stable	25.642	25.642
range.size	0.323	0.323

S.3.21 *Aubrieta columnae* GLM

Species: *Aubrieta columnae* Guss. subsp. *columnae*

Procedure: GLM

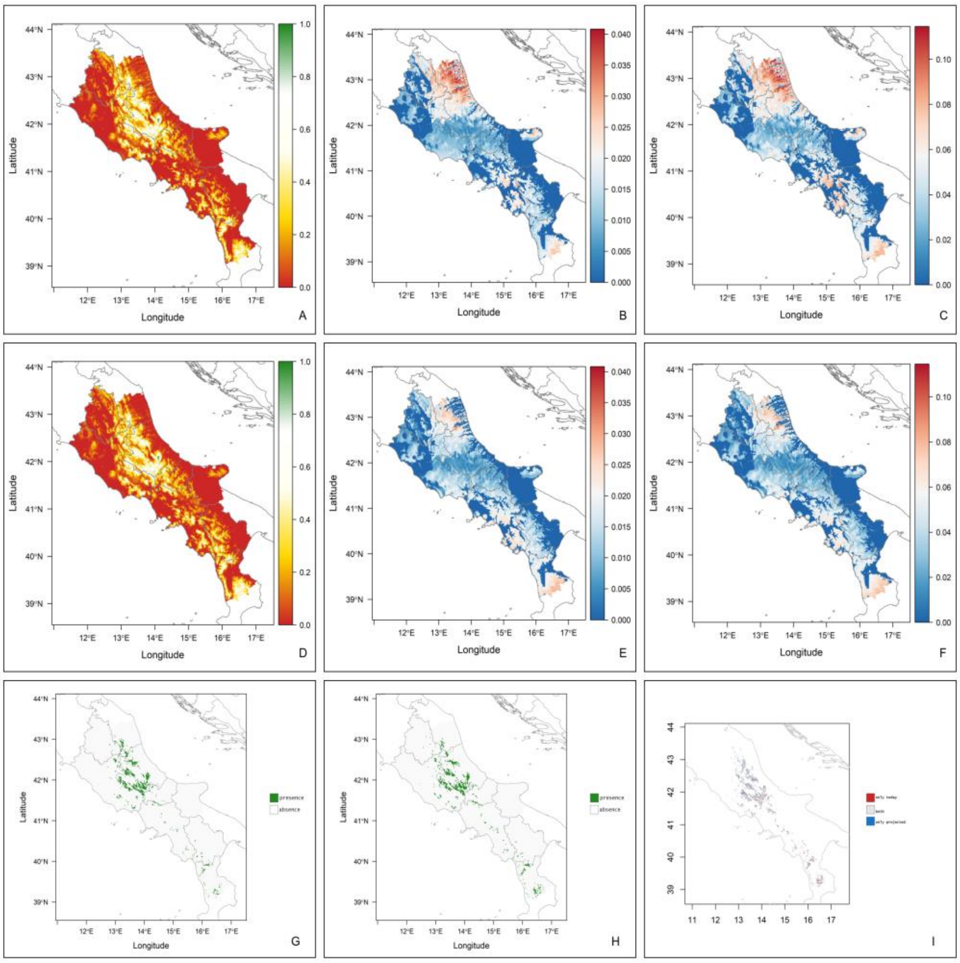

Fig. S.3.21-A) 'Projected' base output; B) 'Projected' uncertainty standard deviation; C) 'Projected' uncertainty range of values; D) 'Present' base output; E) 'Present' uncertainty standard deviation; F) 'Present' uncertainty range of values; G) 'Projected' binary distribution; H) 'Present' binary distribution; I) Range size variation.

Value	Present	Projected
AUC.train	0.945	0.936
AUC.test	0.945	0.933
AUC.diff	0.025	0.025
pROC	1.815	1.780
OR.p10	0.133	0.233
CBI	0.989	0.981
perc.stable	44.439	44.439
range.size	2.024	2.024

S.3.22 *Aubrieta columnae* MARS

Species: *Aubrieta columnae* Guss. subsp. *columnae*

Procedure: MARS

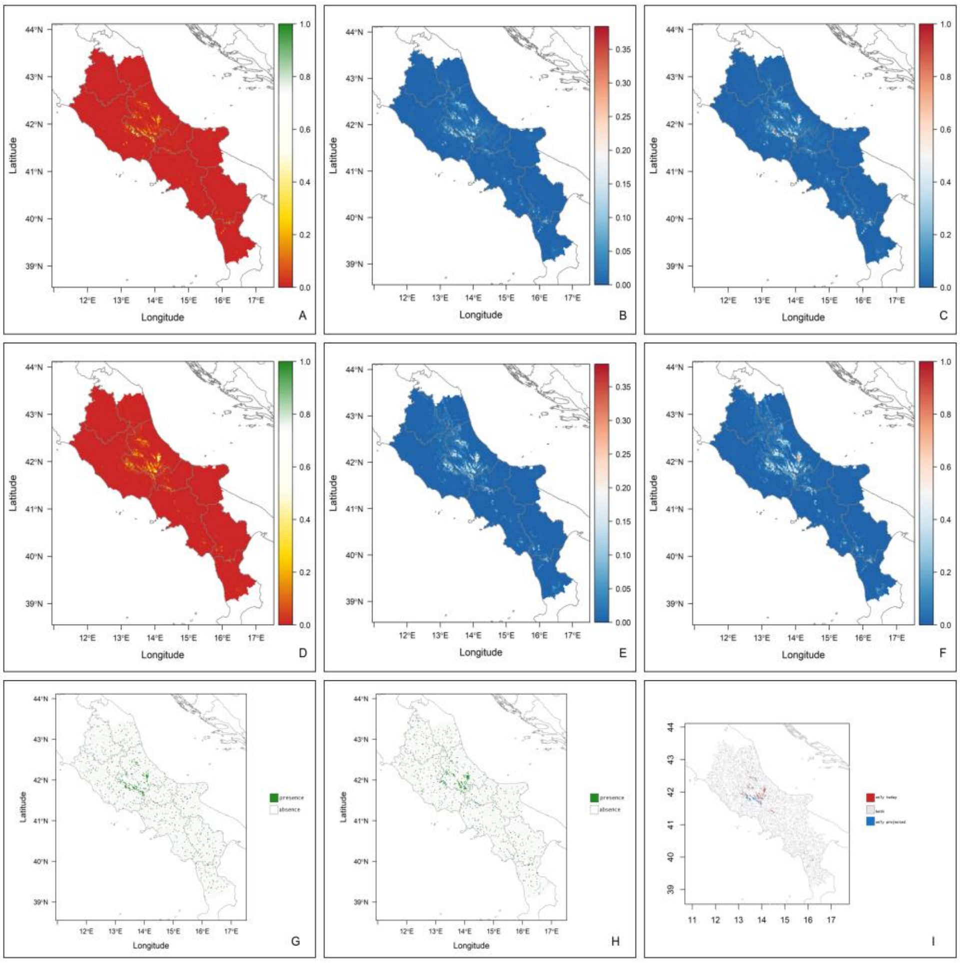

Fig. S.3.22-A) 'Projected' base output; B) 'Projected' uncertainty standard deviation; C) 'Projected' uncertainty range of values; D) 'Present' base output; E) 'Present' uncertainty standard deviation; F) 'Present' uncertainty range of values; G) 'Projected' binary distribution; H) 'Present' binary distribution; I) Range size variation.

Value	Present	Projected
AUC.train	0.821	0.944
AUC.test	0.821	0.845
AUC.diff	0.109	0.109
pROC	1.509	1.497
OR.p10	0.433	0.233
CBI	0.896	0.584
perc.stable	4.998	4.998
range.size	1.839	1.839

S.3.23 *Aubrieta columnae* RF

Species: *Aubrieta columnae* Guss. subsp. *columnae*

Procedure: RF

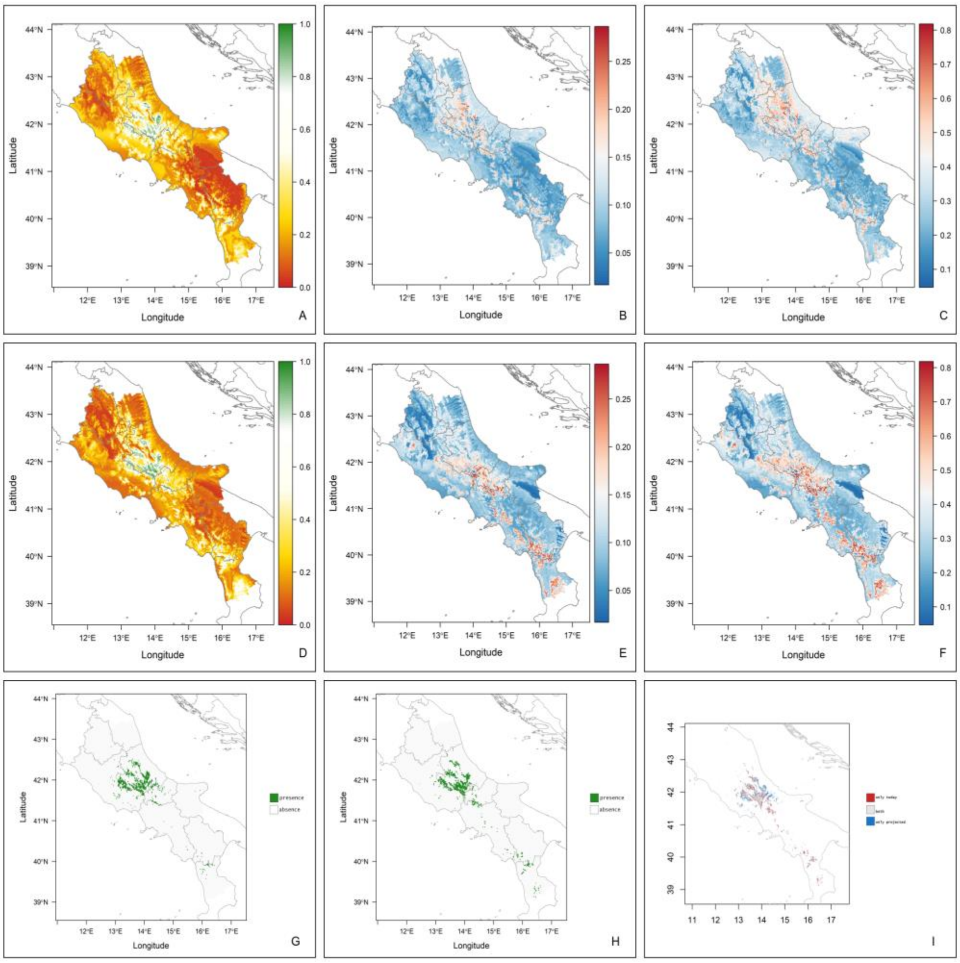

Fig. S.3.23-A) 'Projected' base output; B) 'Projected' uncertainty standard deviation; C) 'Projected' uncertainty range of values; D) 'Present' base output; E) 'Present' uncertainty standard deviation; F) 'Present' uncertainty range of values; G) 'Projected' binary distribution; H) 'Present' binary distribution; I) Range size variation.

Value	Present	Projected
AUC.train	0.981	1.000
AUC.test	0.981	0.988
AUC.diff	0.012	0.012
pROC	1.747	1.808
OR.p10	0.517	0.517
CBI	0.959	0.963
perc.stable	42.542	42.542
range.size	-0.915	-0.915

S.3.24 *Aubrieta columnae* SVM

Species: *Aubrieta columnae* Guss. subsp. *columnae*

Procedure: SVM

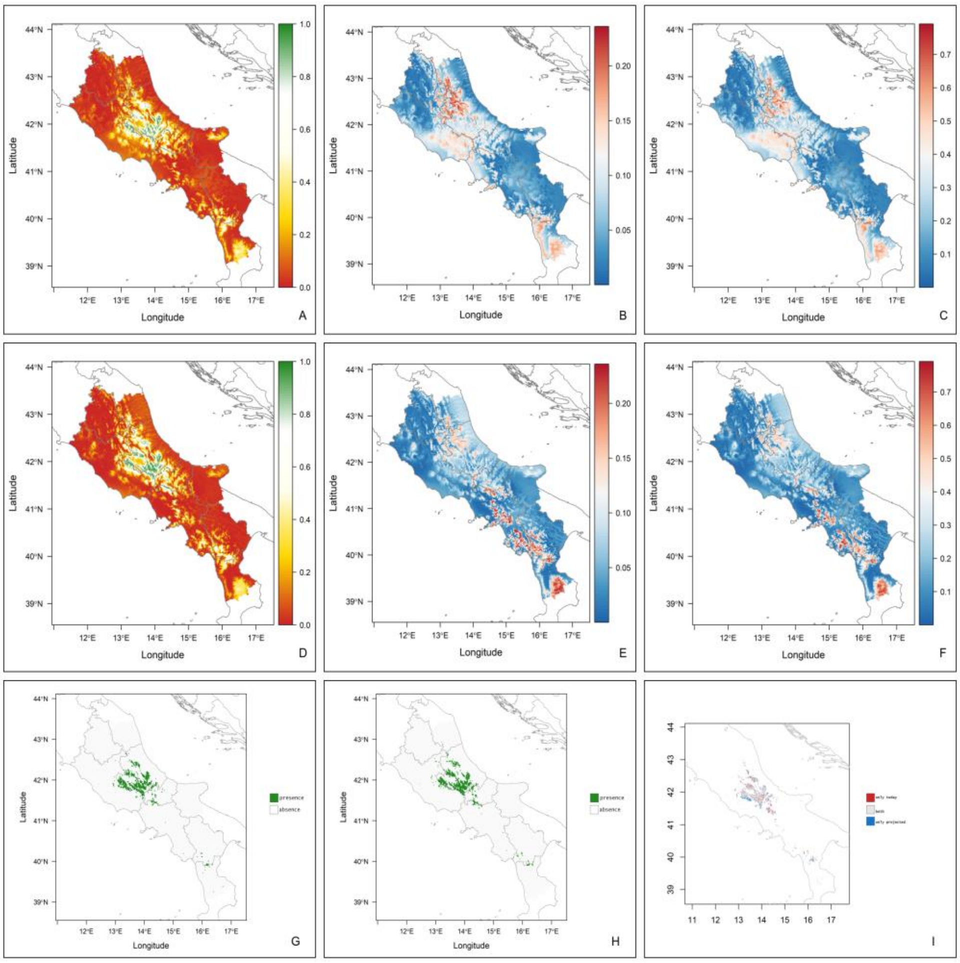

Fig. S.3.24-A) 'Projected' base output; B) 'Projected' uncertainty standard deviation; C) 'Projected' uncertainty range of values; D) 'Present' base output; E) 'Present' uncertainty standard deviation; F) 'Present' uncertainty range of values; G) 'Projected' binary distribution; H) 'Present' binary distribution; I) Range size variation.

Value	Present	Projected
AUC.train	0.949	0.974
AUC.test	0.949	0.980
AUC.diff	0.003	0.003
pROC	1.788	1.855
OR.p10	0.267	0.200
CBI	0.922	0.943
perc.stable	58.538	58.538
range.size	-2.097	-2.097

S.3.25 *Bellevalia webbiana* Biomod2

Species: *Bellevalia webbiana* Parl.

Procedure: Biomod2

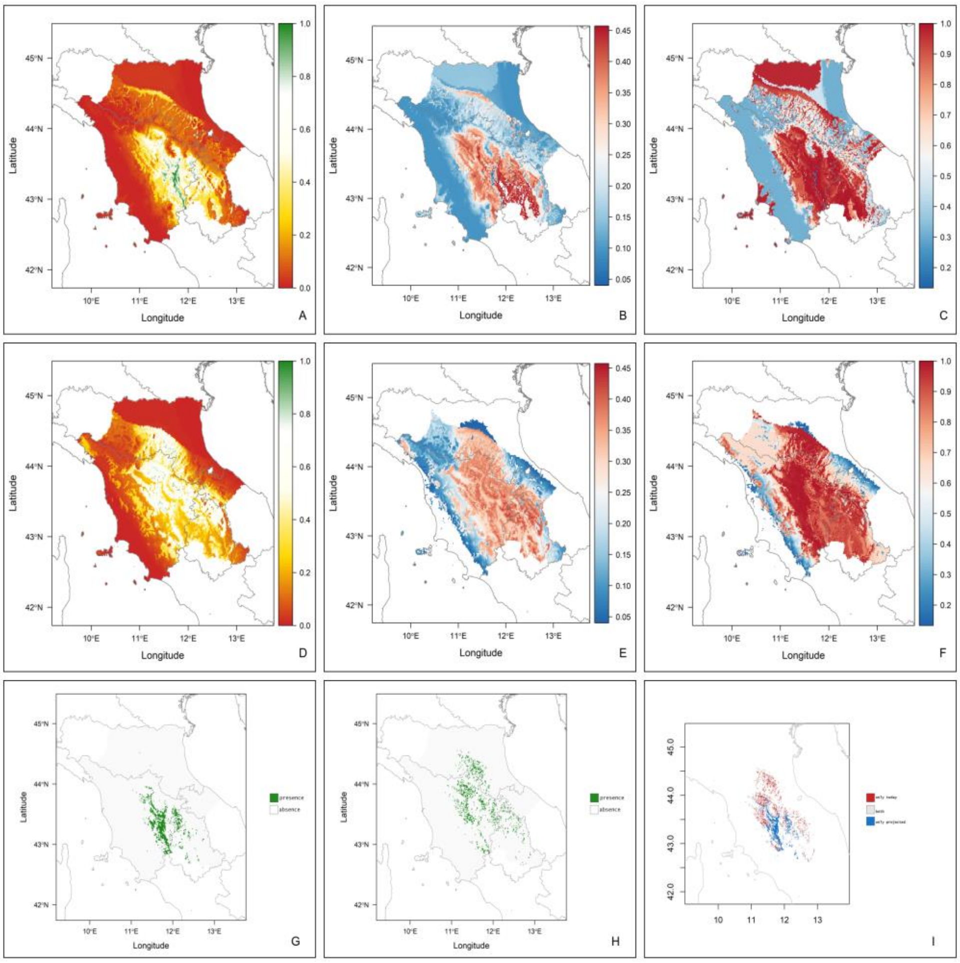

Fig. S.3.25-A) 'Projected' base output; B) 'Projected' uncertainty standard deviation; C) 'Projected' uncertainty range of values; D) 'Present' base output; E) 'Present' uncertainty standard deviation; F) 'Present' uncertainty range of values; G) 'Projected' binary distribution; H) 'Present' binary distribution; I) Range size variation.

Value	Present	Projected
AUC.train	0.921	0.998
AUC.test	0.921	0.777
AUC.diff	0.222	0.222
pROC	1.750	1.445
OR.p10	0.983	0.683
CBI	0.961	0.589
perc.stable	6.801	6.801
range.size	-1.517	-1.517

S.3.26 *Bellevalia webbiana* Bioclim

Species: *Bellevalia webbiana* Parl.

Procedure: Bioclim

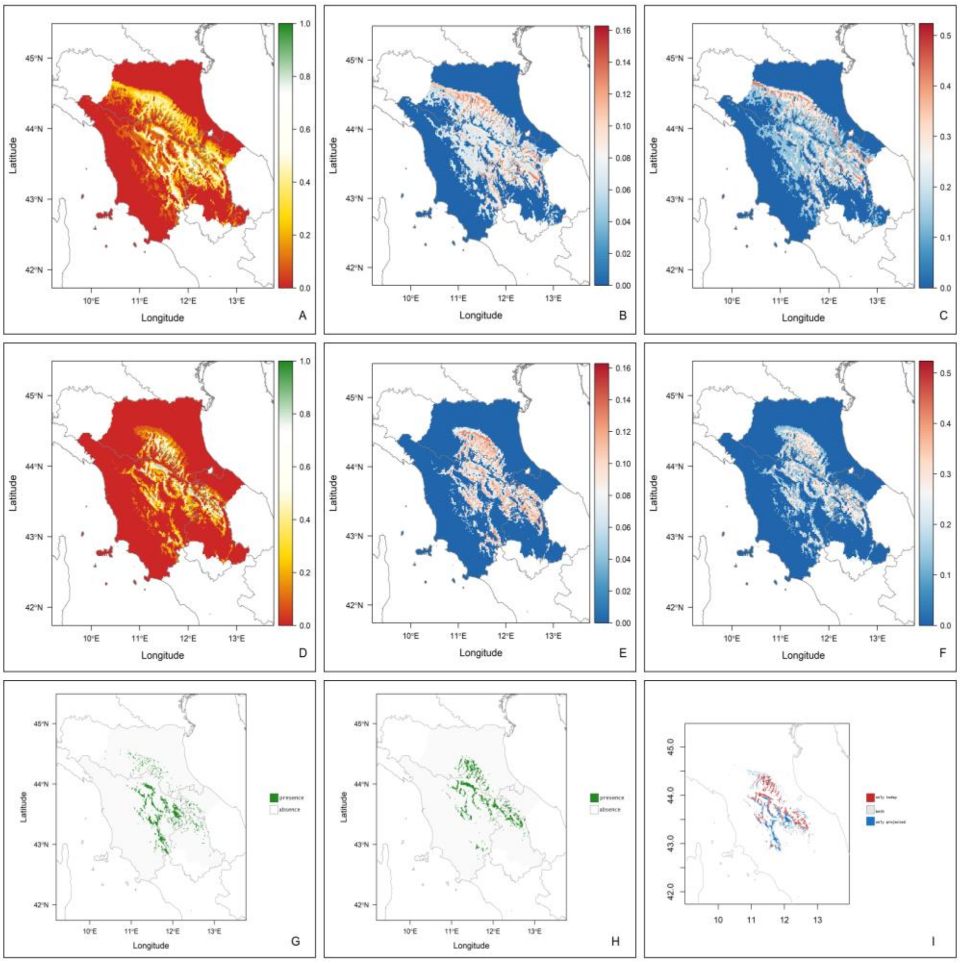

Fig. S.3.26-A) 'Projected' base output; B) 'Projected' uncertainty standard deviation; C) 'Projected' uncertainty range of values; D) 'Present' base output; E) 'Present' uncertainty standard deviation; F) 'Present' uncertainty range of values; G) 'Projected' binary distribution; H) 'Present' binary distribution; I) Range size variation.

Value	Present	Projected
AUC.train	0.761	0.839
AUC.test	0.761	0.775
AUC.diff	0.083	0.083
pROC	1.292	1.361
OR.p10	0.317	0.167
CBI	0.453	0.567
perc.stable	14.024	14.024
range.size	1.601	1.601

S.3.27 *Bellevalia webbiana* Domain

Species: *Bellevalia webbiana* Parl.

Procedure: Domain

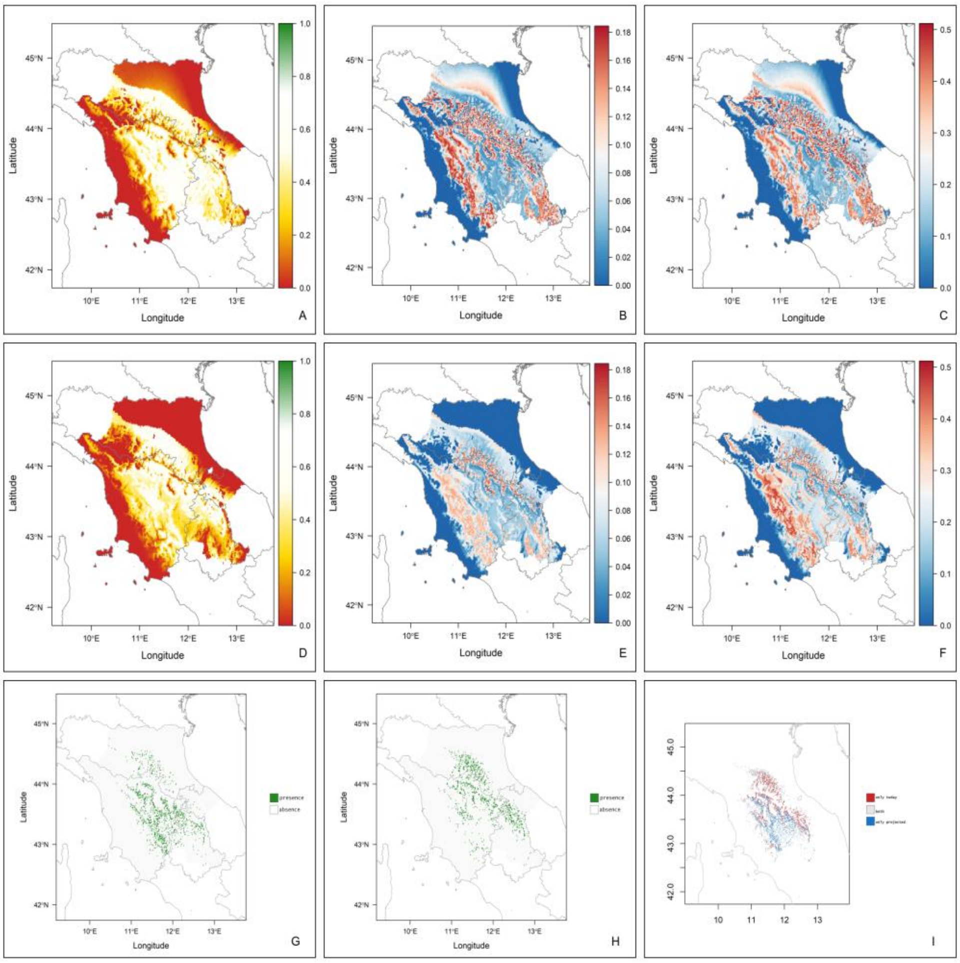

Fig. S.3.27-A) 'Projected' base output; B) 'Projected' uncertainty standard deviation; C) 'Projected' uncertainty range of values; D) 'Present' base output; E) 'Present' uncertainty standard deviation; F) 'Present' uncertainty range of values; G) 'Projected' binary distribution; H) 'Present' binary distribution; I) Range size variation.

Value	Present	Projected
AUC.train	0.822	0.816
AUC.test	0.822	0.771
AUC.diff	0.072	0.072
pROC	1.570	1.472
OR.p10	0.350	0.267
CBI	0.898	0.715
perc.stable	7.254	7.254
range.size	8.781	8.781

S.3.28 *Bellevalia webbiana* Maxent

Species: *Bellevalia webbiana* Parl.

Procedure: Maxent

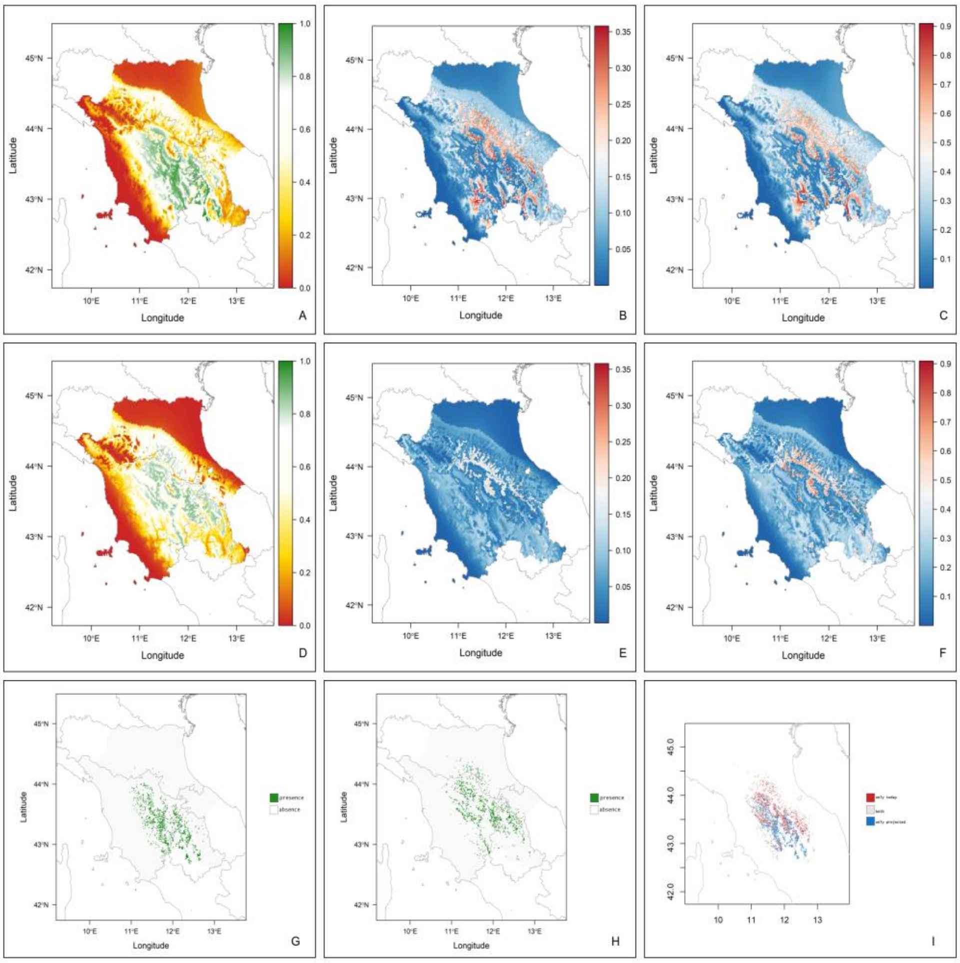

Fig. S.3.28-A) 'Projected' base output; B) 'Projected' uncertainty standard deviation; C) 'Projected' uncertainty range of values; D) 'Present' base output; E) 'Present' uncertainty standard deviation; F) 'Present' uncertainty range of values; G) 'Projected' binary distribution; H) 'Present' binary distribution; I) Range size variation.

Value	Present	Projected
AUC.train	0.818	0.812
AUC.test	0.818	0.791
AUC.diff	0.072	0.072
pROC	1.563	1.502
OR.p10	0.217	0.183
CBI	0.795	0.327
perc.stable	6.143	6.143
range.size	2.177	2.177

S.3.29 *Bellevalia webbiana* KDE

Species: *Bellevalia webbiana* Parl.

Procedure: KDE

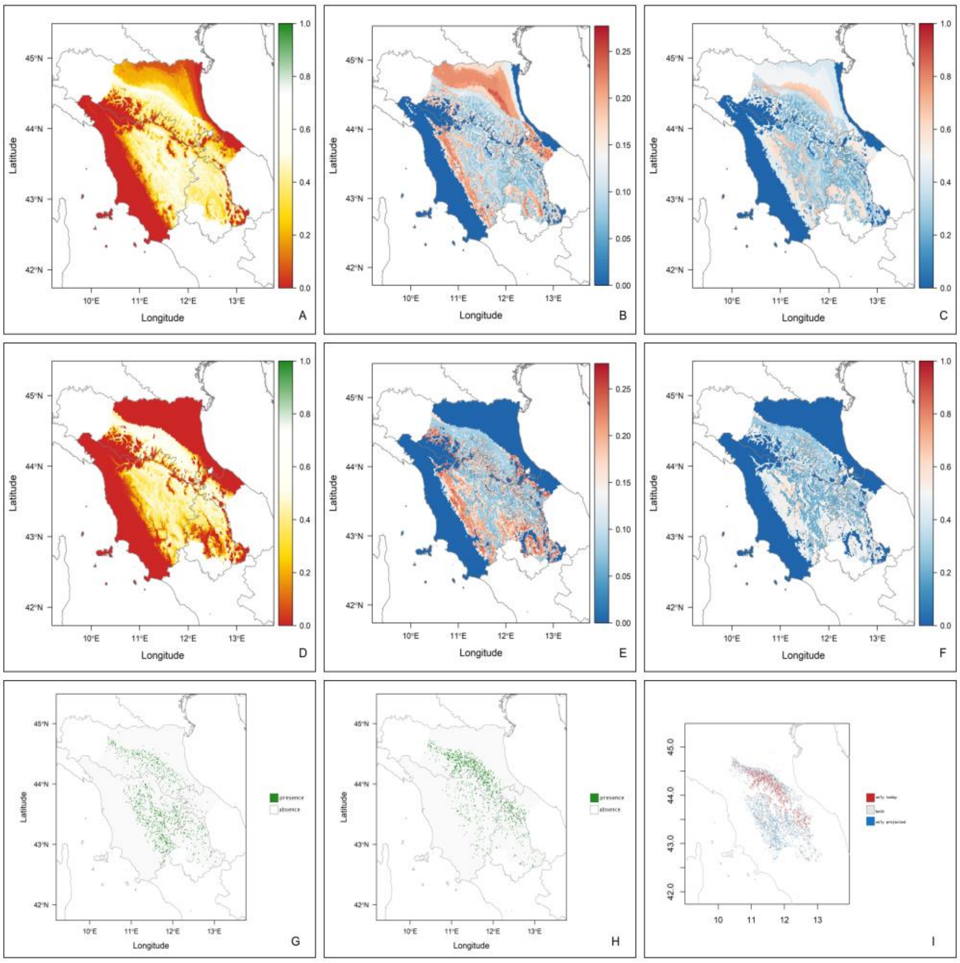

Fig. S.3.29-A) 'Projected' base output; B) 'Projected' uncertainty standard deviation; C) 'Projected' uncertainty range of values; D) 'Present' base output; E) 'Present' uncertainty standard deviation; F) 'Present' uncertainty range of values; G) 'Projected' binary distribution; H) 'Present' binary distribution; I) Range size variation.

Value	Present	Projected
AUC.train	0.780	0.822
AUC.test	0.780	0.789
AUC.diff	0.093	0.054
pROC	1.490	1.484
OR.p10	0.500	0.200
CBI	0.823	0.774
perc.stable	4.205	4.205
range.size	1.867	1.867

S.3.30 *Bellevalia webbiana* CTA

Species: *Bellevalia webbiana* Parl.

Procedure: CTA

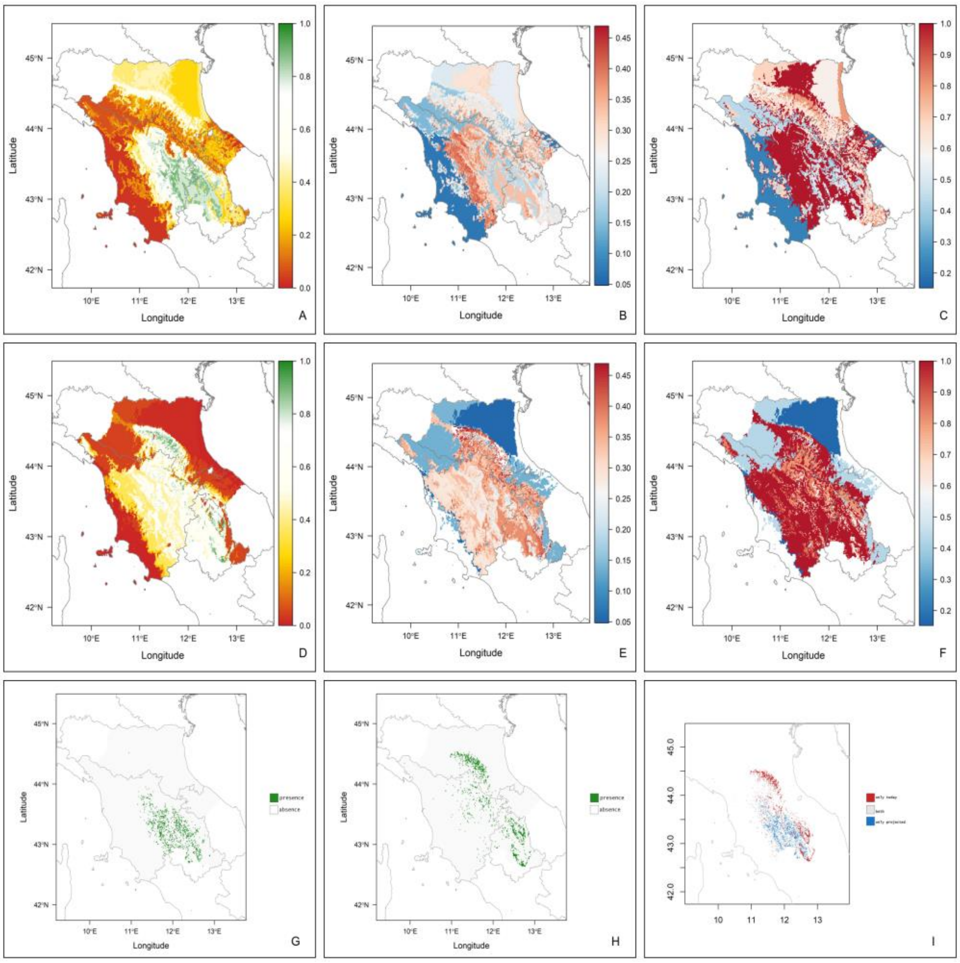

Fig. S.3.30-A) 'Projected' base output; B) 'Projected' uncertainty standard deviation; C) 'Projected' uncertainty range of values; D) 'Present' base output; E) 'Present' uncertainty standard deviation; F) 'Present' uncertainty range of values; G) 'Projected' binary distribution; H) 'Present' binary distribution; I) Range size variation.

Value	Present	Projected
AUC.train	0.793	0.908
AUC.test	0.793	0.788
AUC.diff	0.127	0.127
pROC	1.274	1.183
OR.p10	0.317	0.233
CBI	0.956	0.488
perc.stable	1.776	1.776
range.size	-4.356	-4.356

S.3.31 *Bellevalia webbiana* FDA

Species: *Bellevalia webbiana* Parl.

Procedure: FDA

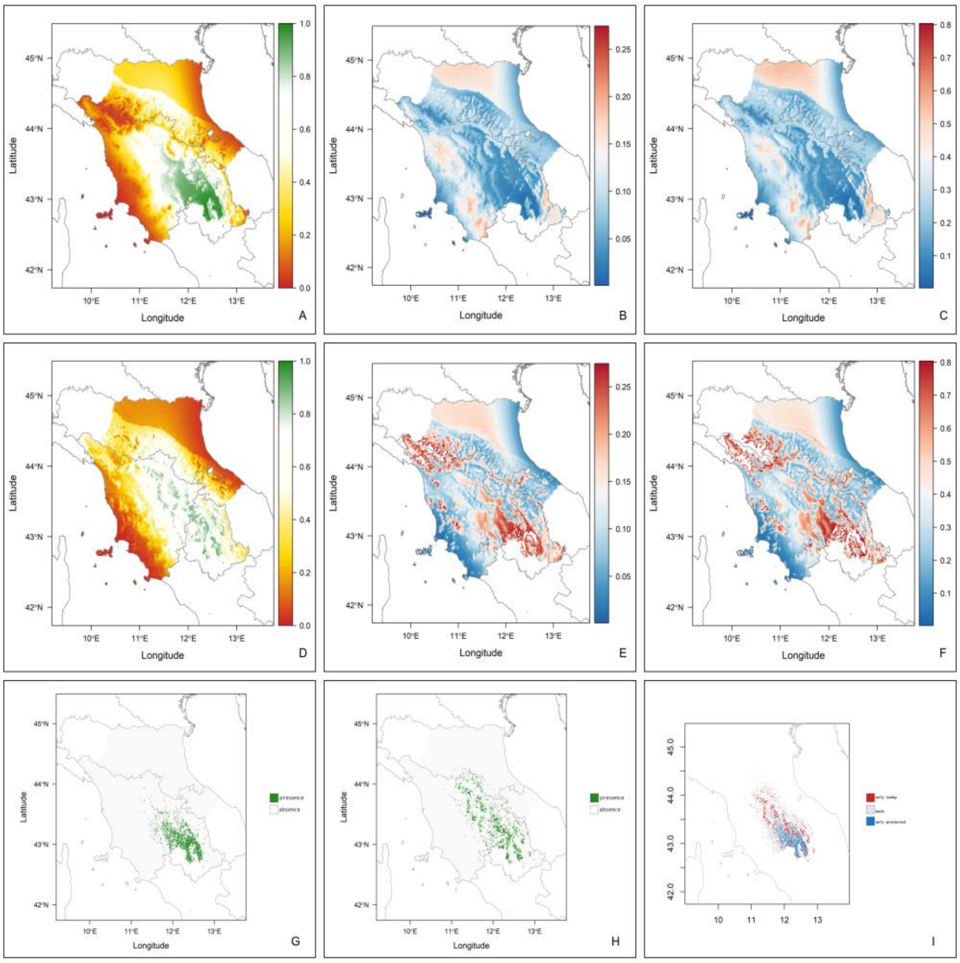

Fig. S.3.31-A) 'Projected' base output; B) 'Projected' uncertainty standard deviation; C) 'Projected' uncertainty range of values; D) 'Present' base output; E) 'Present' uncertainty standard deviation; F) 'Present' uncertainty range of values; G) 'Projected' binary distribution; H) 'Present' binary distribution; I) Range size variation.

Value	Present	Projected
AUC.train	0.747	0.808
AUC.test	0.747	0.785
AUC.diff	0.039	0.039
pROC	1.435	1.507
OR.p10	0.333	0.200
CBI	0.699	-0.366
perc.stable	10.813	10.813
range.size	0.606	0.606

S.3.32 *Bellevalia webbiana* GAM

Species: *Bellevalia webbiana* Parl.

Procedure: GAM

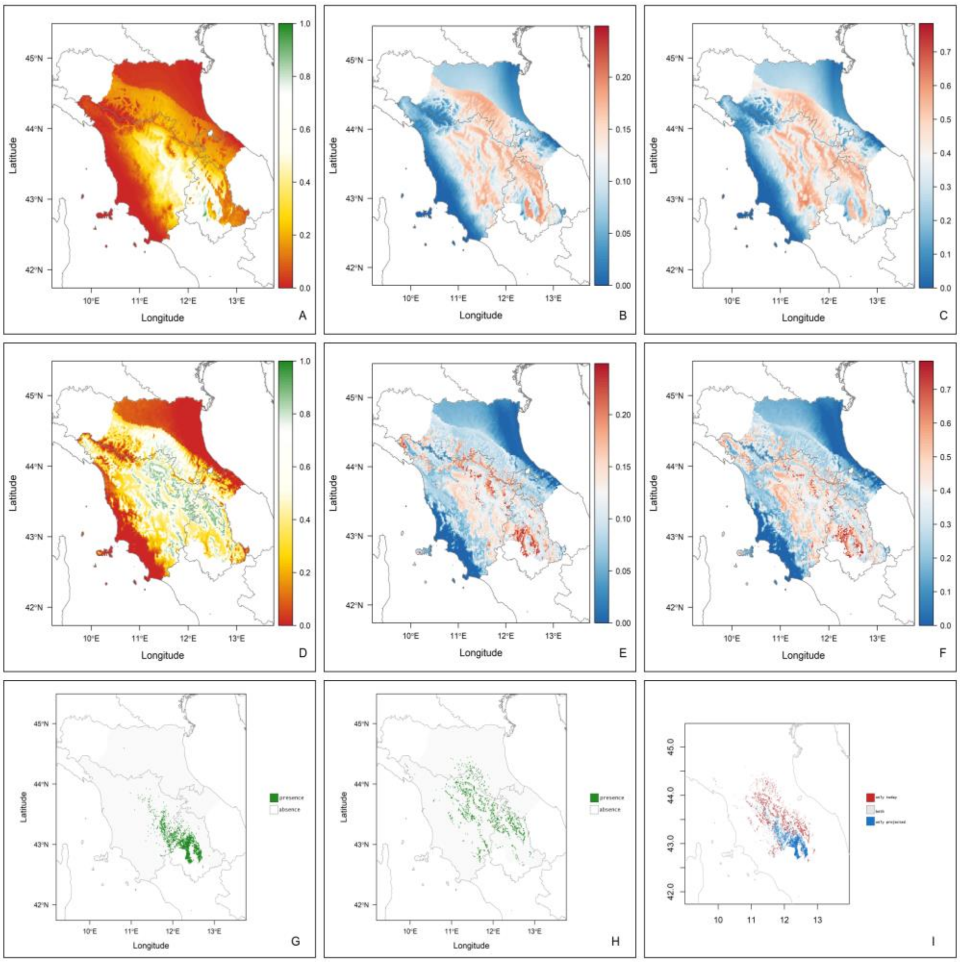

Fig. S.3.32-A) 'Projected' base output; B) 'Projected' uncertainty standard deviation; C) 'Projected' uncertainty range of values; D) 'Present' base output; E) 'Present' uncertainty standard deviation; F) 'Present' uncertainty range of values; G) 'Projected' binary distribution; H) 'Present' binary distribution; I) Range size variation.

Value	Present	Projected
AUC.train	0.806	0.801
AUC.test	0.806	0.796
AUC.diff	0.042	0.042
pROC	1.563	1.498
OR.p10	0.333	0.183
CBI	0.847	0.134
perc.stable	1.450	1.450
range.size	0.834	0.834

S.3.33 *Bellevalia webbiana* GLM

Species: *Bellevalia webbiana* Parl.

Procedure: GLM

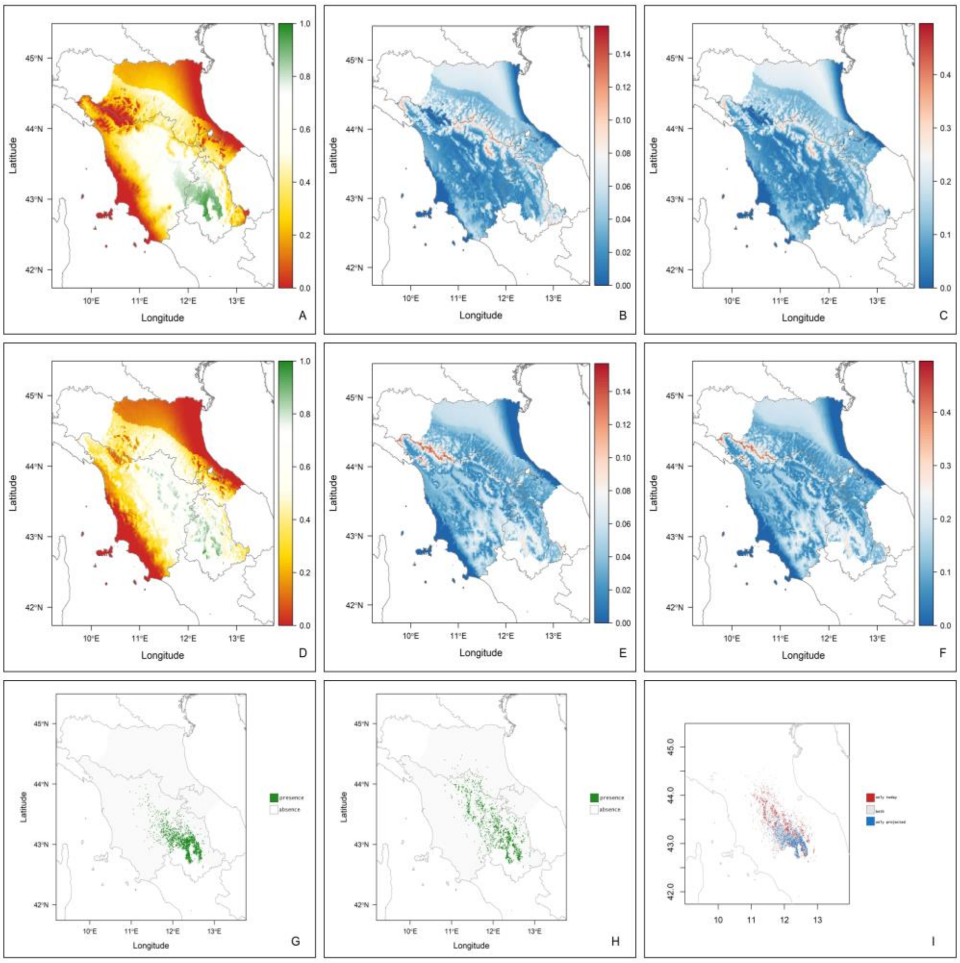

Fig. S.3.33-A) 'Projected' base output; B) 'Projected' uncertainty standard deviation; C) 'Projected' uncertainty range of values; D) 'Present' base output; E) 'Present' uncertainty standard deviation; F) 'Present' uncertainty range of values; G) 'Projected' binary distribution; H) 'Present' binary distribution; I) Range size variation.

Value	Present	Projected
AUC.train	0.761	0.764
AUC.test	0.761	0.789
AUC.diff	0.019	0.019
pROC	1.493	1.522
OR.p10	0.133	0.117
CBI	0.703	-0.268
perc.stable	15.089	15.089
range.size	-2.624	-2.624

S.3.34 *Bellevalia webbiana* MARS

Species: *Bellevalia webbiana* Parl.

Procedure: MARS

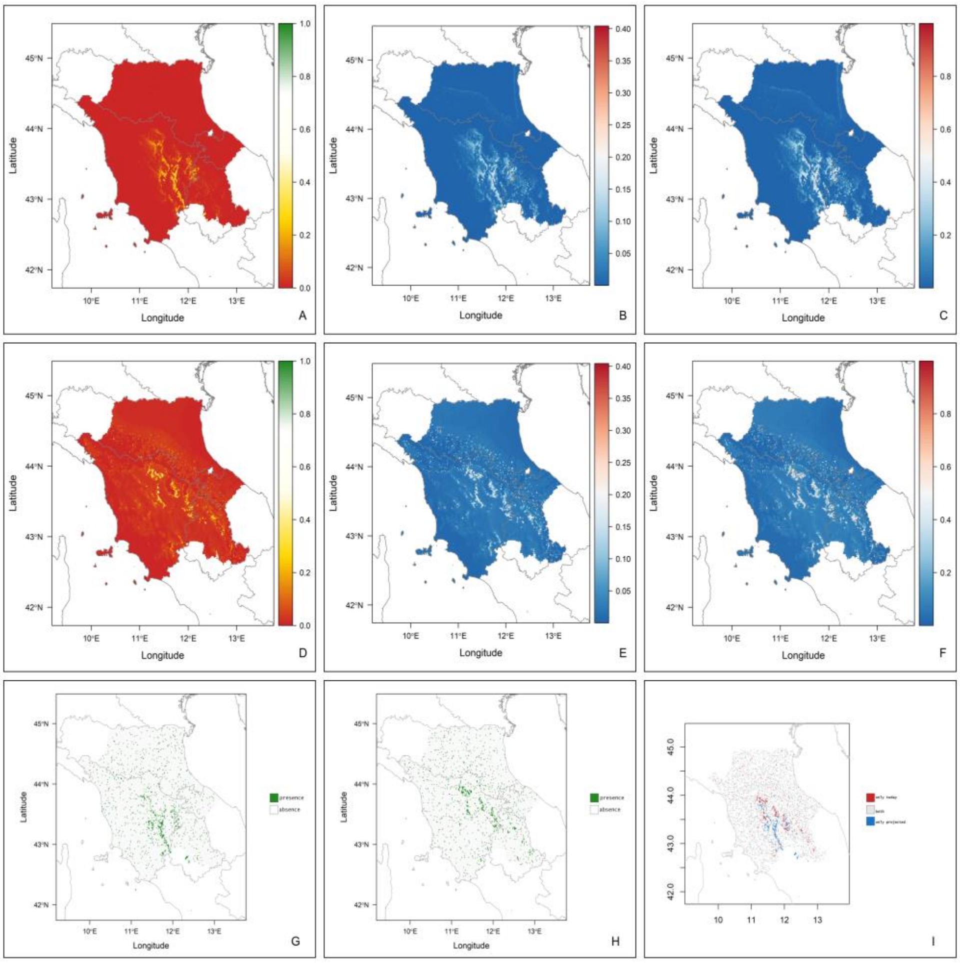

Fig. S.3.34-A) 'Projected' base output; B) 'Projected' uncertainty standard deviation; C) 'Projected' uncertainty range of values; D) 'Present' base output; E) 'Present' uncertainty standard deviation; F) 'Present' uncertainty range of values; G) 'Projected' binary distribution; H) 'Present' binary distribution; I) Range size variation.

Value	Present	Projected
AUC.train	0.771	0.809
AUC.test	0.771	0.625
AUC.diff	0.185	0.185
pROC	1.456	1.149
OR.p10	0.433	0.267
CBI	0.754	0.583
perc.stable	2.206	2.206
range.size	-5.859	-5.859

S.3.35 *Bellevalia webbiana* RF

Species: *Bellevalia webbiana* Parl.

Procedure: RF

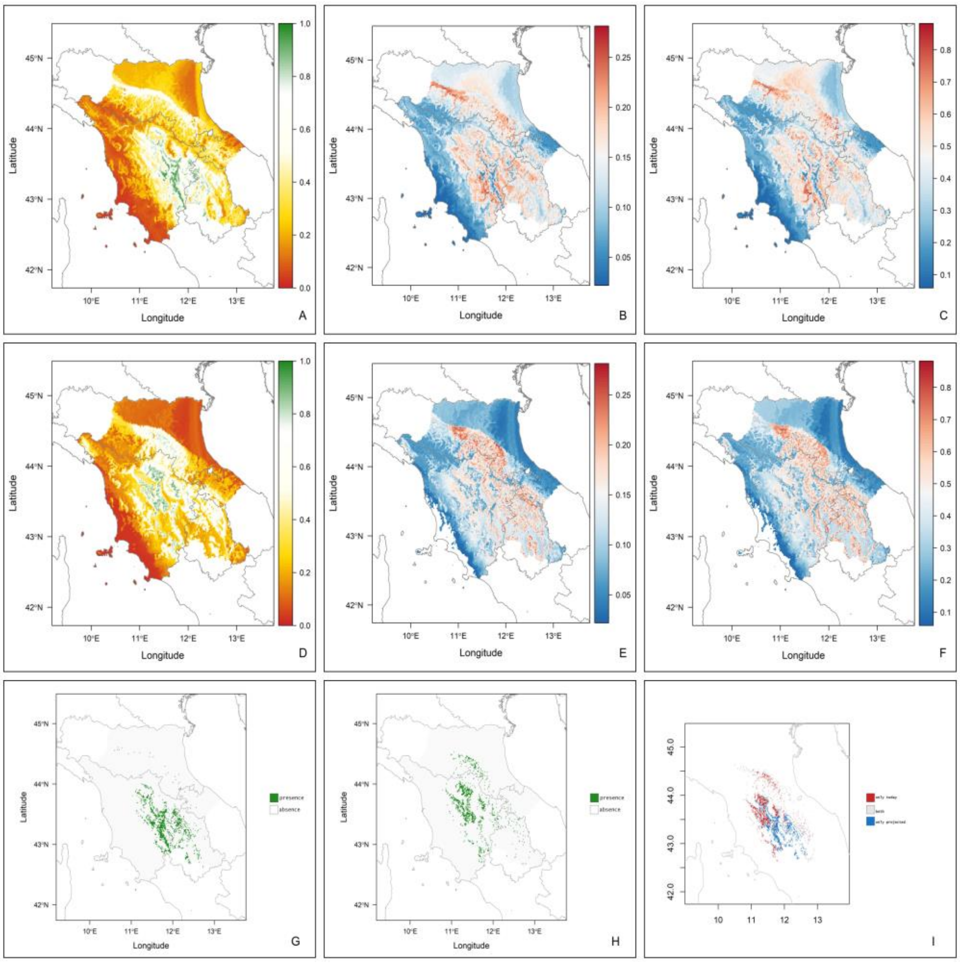

Fig. S.3.35-A) 'Projected' base output; B) 'Projected' uncertainty standard deviation; C) 'Projected' uncertainty range of values; D) 'Present' base output; E) 'Present' uncertainty standard deviation; F) 'Present' uncertainty range of values; G) 'Projected' binary distribution; H) 'Present' binary distribution; I) Range size variation.

Value	Present	Projected
AUC.train	0.973	1.000
AUC.test	0.973	0.975
AUC.diff	0.025	0.025
pROC	1.615	1.519
OR.p10	0.683	0.650
CBI	0.968	0.276
perc.stable	4.629	4.629
range.size	8.342	8.342

S.3.36 *Bellevalia webbiana* SVM

Species: *Bellevalia webbiana* Parl.

Procedure: SVM

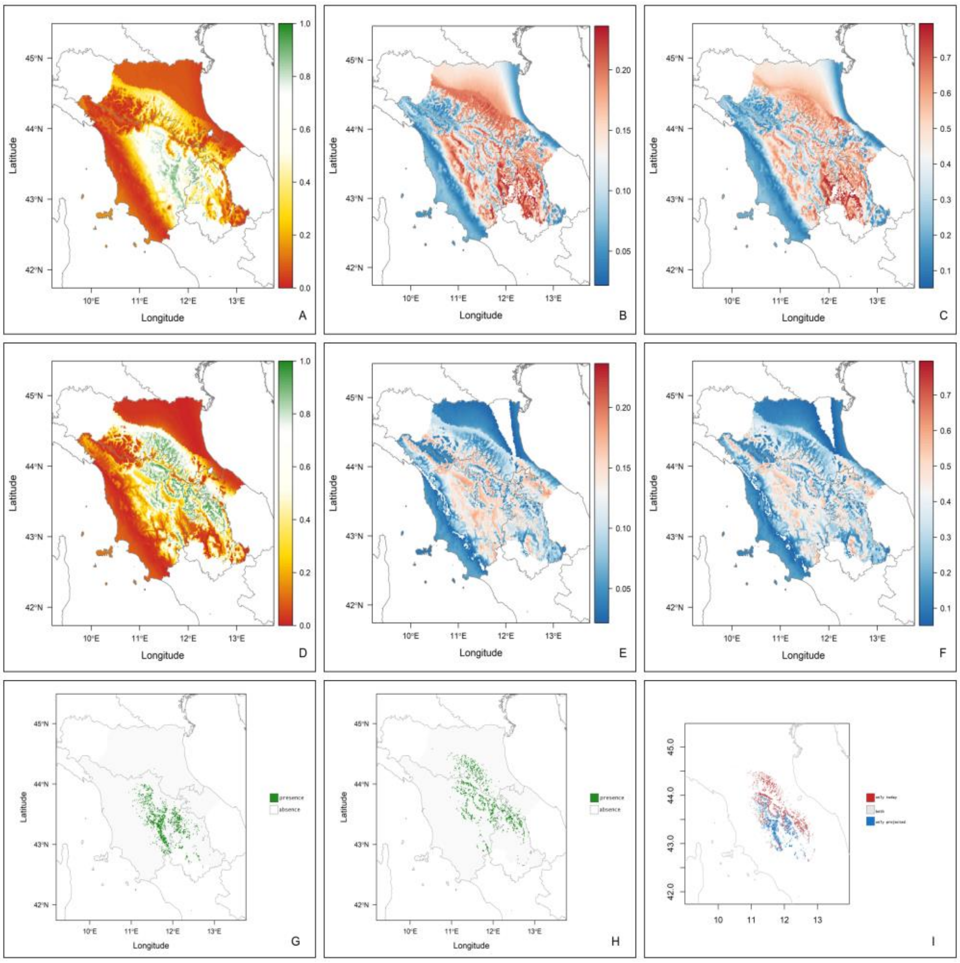

Fig. S.3.36-A) 'Projected' base output; B) 'Projected' uncertainty standard deviation; C) 'Projected' uncertainty range of values; D) 'Present' base output; E) 'Present' uncertainty standard deviation; F) 'Present' uncertainty range of values; G) 'Projected' binary distribution; H) 'Present' binary distribution; I) Range size variation.

Value	Present	Projected
AUC.train	0.865	0.895
AUC.test	0.865	0.825
AUC.diff	0.080	0.080
pROC	1.591	1.471
OR.p10	0.500	0.300
CBI	0.870	0.367
perc.stable	3.625	3.625
range.size	-3.736	-3.736

S.3.37 *Cardamine apennina* Biomod2

Species: *Cardamine apennina* Lihová & Marhold

Procedure: Biomod2

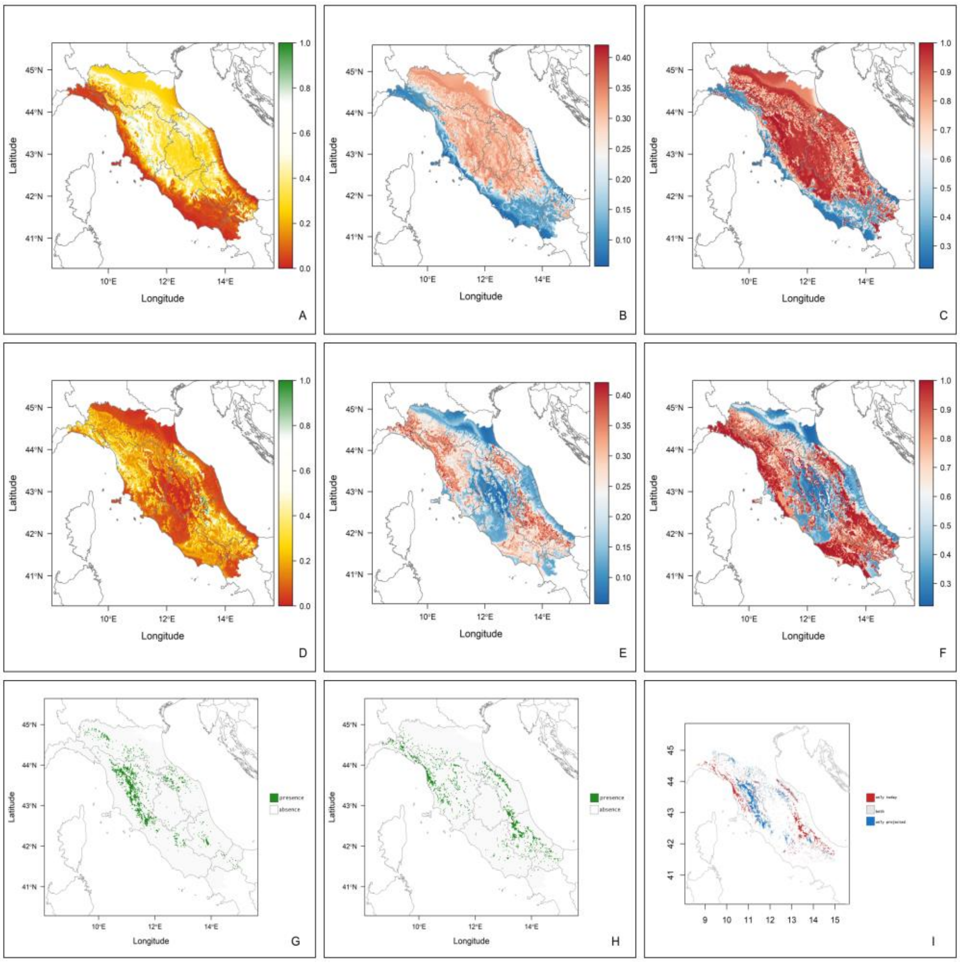

Fig. S.3.37-A) 'Projected' base output; B) 'Projected' uncertainty standard deviation; C) 'Projected' uncertainty range of values; D) 'Present' base output; E) 'Present' uncertainty standard deviation; F) 'Present' uncertainty range of values; G) 'Projected' binary distribution; H) 'Present' binary distribution; I) Range size variation.

Value	Present	Projected
AUC.train	0.918	0.985
AUC.test	0.918	0.917
AUC.diff	0.068	0.068
pROC	1.676	1.625
OR.p10	0.650	0.475
CBI	0.933	0.077
perc.stable	3.663	3.663
range.size	1.755	1.755

S.3.38 *Cardamine apennina* Bioclim

Species: *Cardamine apennina* Lihová & Marhold

Procedure: Bioclim

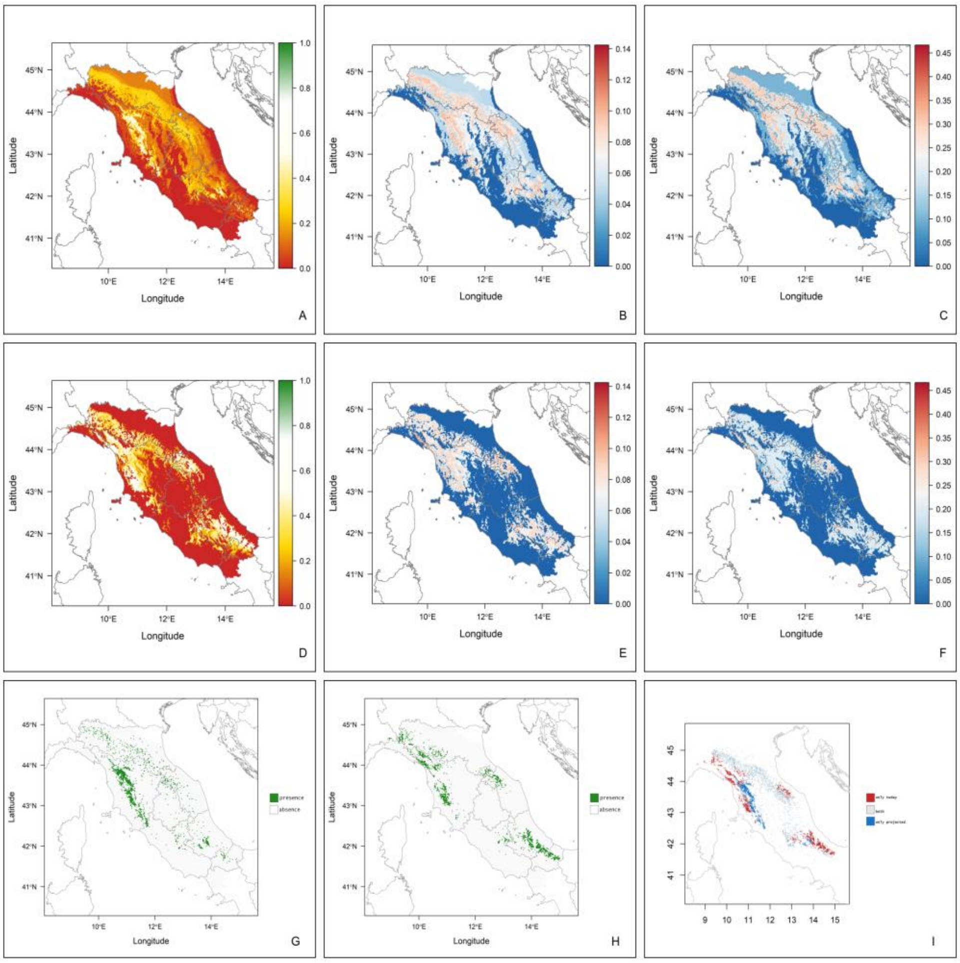

Fig. S.3.38-A) 'Projected' base output; B) 'Projected' uncertainty standard deviation; C) 'Projected' uncertainty range of values; D) 'Present' base output; E) 'Present' uncertainty standard deviation; F) 'Present' uncertainty range of values; G) 'Projected' binary distribution; H) 'Present' binary distribution; I) Range size variation.

Value	Present	Projected
AUC.train	0.727	0.804
AUC.test	0.727	0.713
AUC.diff	0.095	0.095
pROC	1.279	1.176
OR.p10	0.162	0.238
CBI	-0.506	0.471
perc.stable	4.352	4.352
range.size	1.143	1.143

S.3.39 *Cardamine apennina* Domain

Species: *Cardamine apennina* Lihová & Marhold

Procedure: Domain

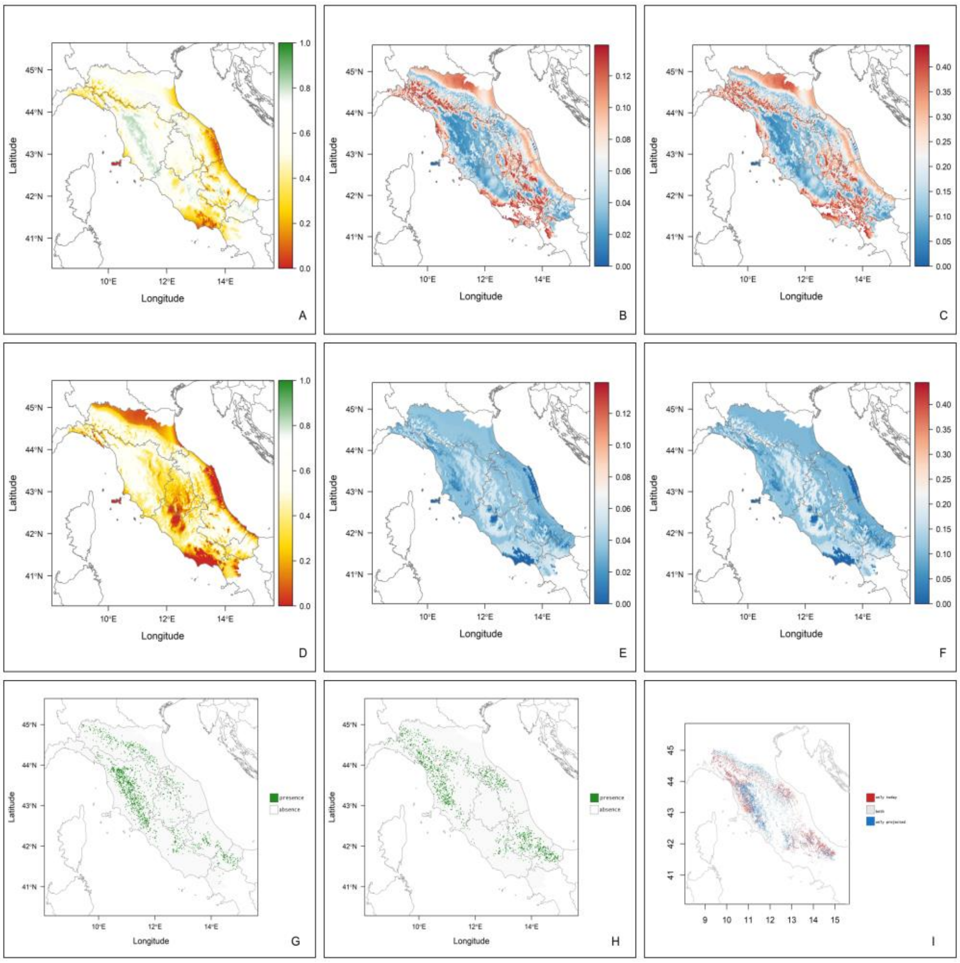

Fig. S.3.39-A) 'Projected' base output; B) 'Projected' uncertainty standard deviation; C) 'Projected' uncertainty range of values; D) 'Present' base output; E) 'Present' uncertainty standard deviation; F) 'Present' uncertainty range of values; G) 'Projected' binary distribution; H) 'Present' binary distribution; I) Range size variation.

Value	Present	Projected
AUC.train	0.713	0.753
AUC.test	0.713	0.759
AUC.diff	0.076	0.076
pROC	1.405	1.374
OR.p10	0.200	0.112
CBI	0.652	-0.442
perc.stable	5.829	5.829
range.size	0.011	0.011

S.3.40 *Cardamine apennina* Maxent

Species: *Cardamine apennina* Lihová & Marhold

Procedure: Maxent

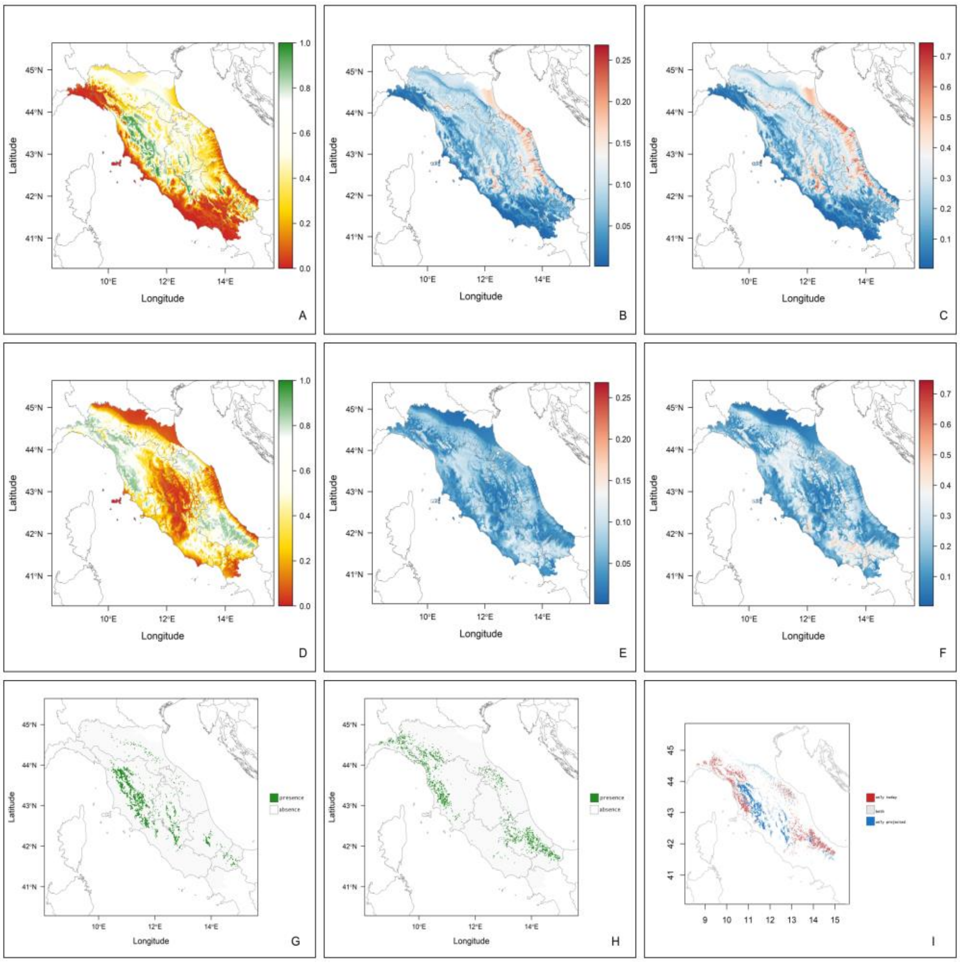

Fig. S.3.40-A) 'Projected' base output; B) 'Projected' uncertainty standard deviation; C) 'Projected' uncertainty range of values; D) 'Present' base output; E) 'Present' uncertainty standard deviation; F) 'Present' uncertainty range of values; G) 'Projected' binary distribution; H) 'Present' binary distribution; I) Range size variation.

Value	Present	Projected
AUC.train	0.780	0.842
AUC.test	0.780	0.763
AUC.diff	0.094	0.094
pROC	1.463	1.361
OR.p10	0.150	0.225
CBI	0.944	-0.312
perc.stable	2.437	2.437
range.size	-2.551	-2.551

S.3.41 *Cardamine apennina* KDE

Species: *Cardamine apennina* Lihová & Marhold

Procedure: KDE

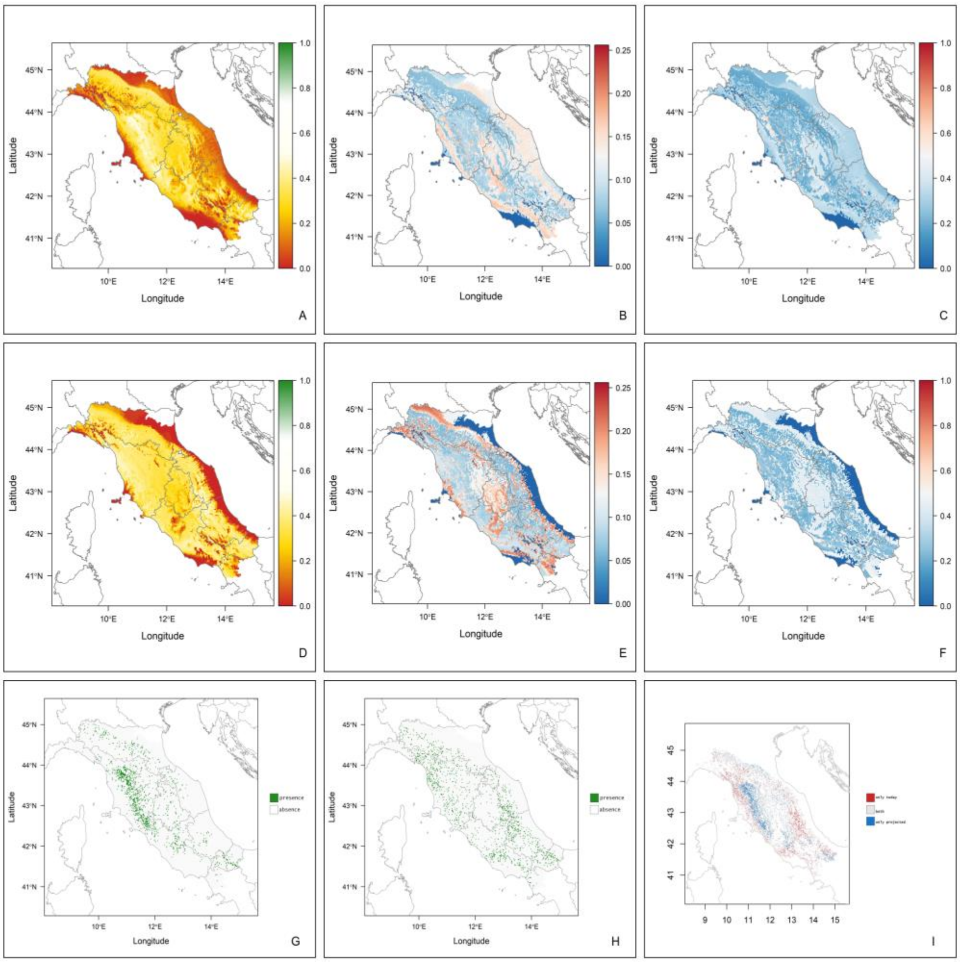

Fig. S.3.41-A) 'Projected' base output; B) 'Projected' uncertainty standard deviation; C) 'Projected' uncertainty range of values; D) 'Present' base output; E) 'Present' uncertainty standard deviation; F) 'Present' uncertainty range of values; G) 'Projected' binary distribution; H) 'Present' binary distribution; I) Range size variation.

Value	Present	Projected
AUC.train	0.808	0.780
AUC.test	0.808	0.739
AUC.diff	0.072	0.102
pROC	1.468	1.314
OR.p10	0.175	0.212
CBI	0.880	-0.243
perc.stable	3.120	3.120
range.size	-6.235	-6.235

S.3.42 *Cardamine apennina* CTA

Species: *Cardamine apennina* Lihová & Marhold

Procedure: CTA

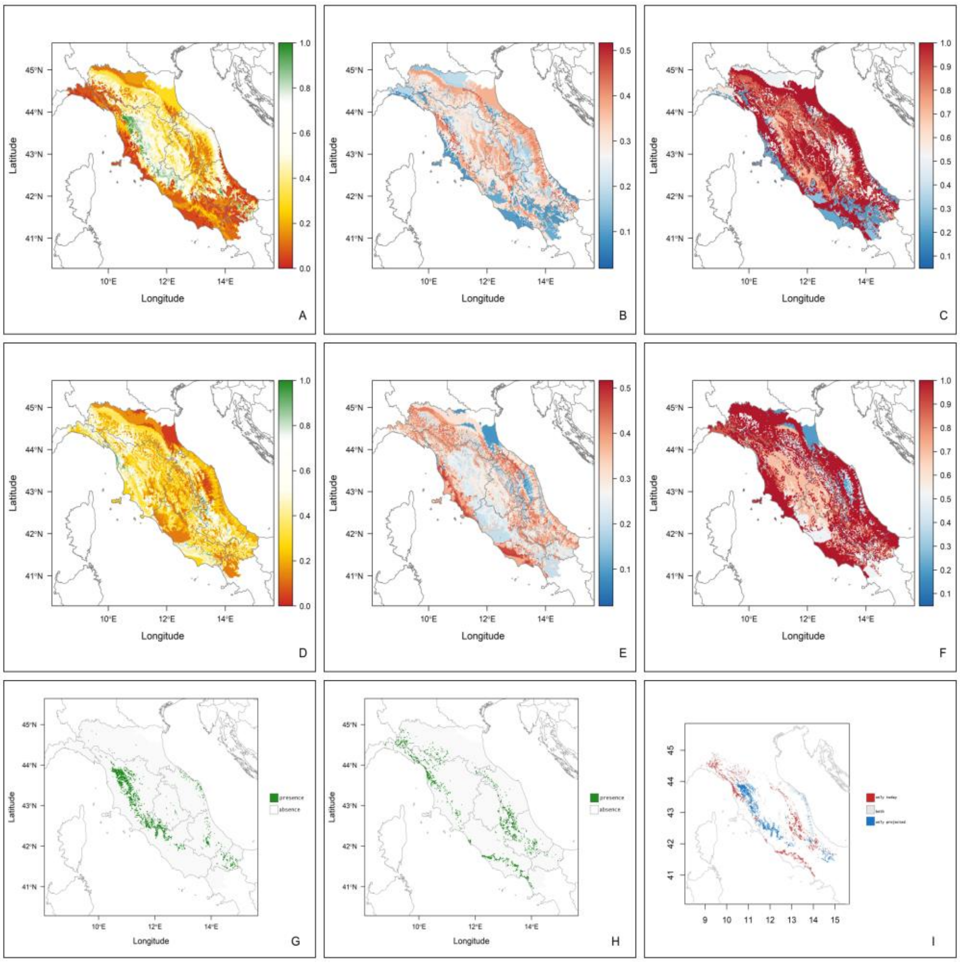

Fig. S.3.42-A) 'Projected' base output; B) 'Projected' uncertainty standard deviation; C) 'Projected' uncertainty range of values; D) 'Present' base output; E) 'Present' uncertainty standard deviation; F) 'Present' uncertainty range of values; G) 'Projected' binary distribution; H) 'Present' binary distribution; I) Range size variation.

Value	Present	Projected
AUC.train	0.853	0.925
AUC.test	0.853	0.780
AUC.diff	0.145	0.145
pROC	1.222	1.181
OR.p10	0.238	0.250
CBI	0.972	0.238
perc.stable	2.077	2.077
range.size	4.505	4.505

S.3.43 *Cardamine apennina* FDA

Species: *Cardamine apennina* Lihová & Marhold

Procedure: FDA

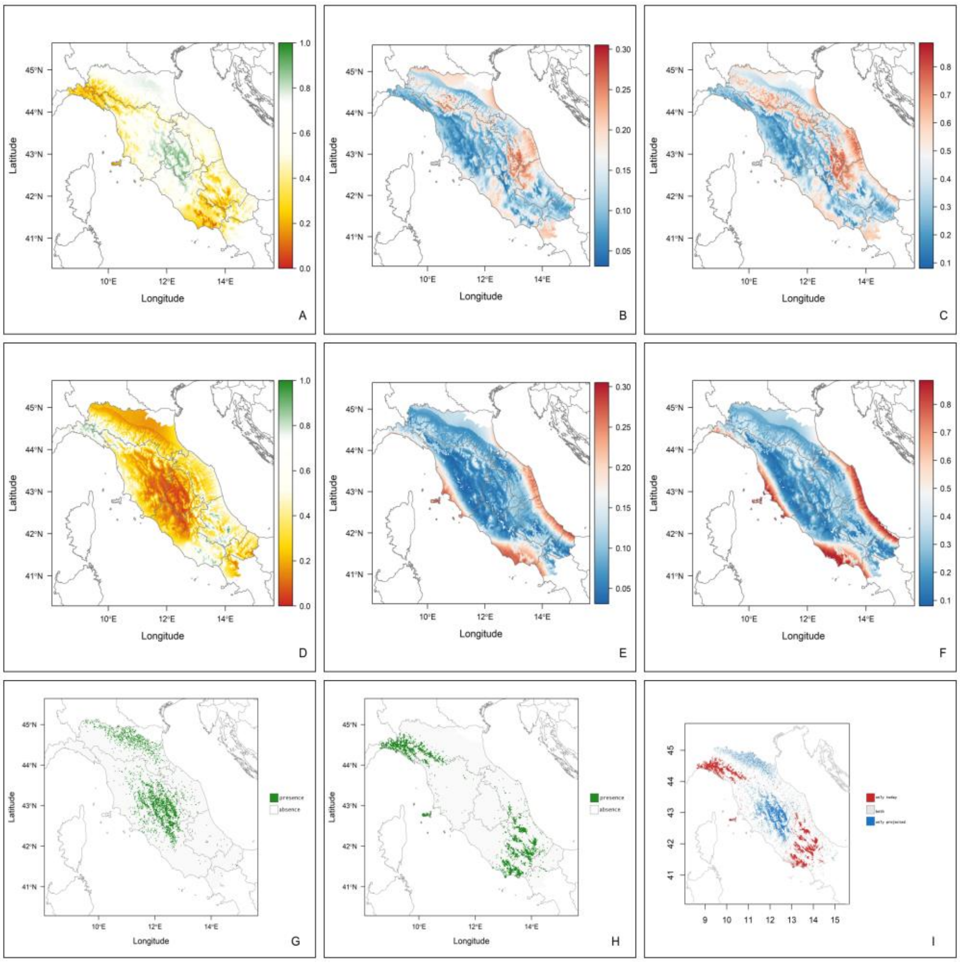

Fig. S.3.43-A) 'Projected' base output; B) 'Projected' uncertainty standard deviation; C) 'Projected' uncertainty range of values; D) 'Present' base output; E) 'Present' uncertainty standard deviation; F) 'Present' uncertainty range of values; G) 'Projected' binary distribution; H) 'Present' binary distribution; I) Range size variation.

Value	Present	Projected
AUC.train	0.692	0.691
AUC.test	0.692	0.628
AUC.diff	0.070	0.070
pROC	1.317	1.268
OR.p10	0.138	0.200
CBI	0.593	-0.538
perc.stable	0.011	0.011
range.size	1.851	1.851

S.3.44 *Cardamine apennina* GAM

Species: *Cardamine apennina* Lihová & Marhold

Procedure: GAM

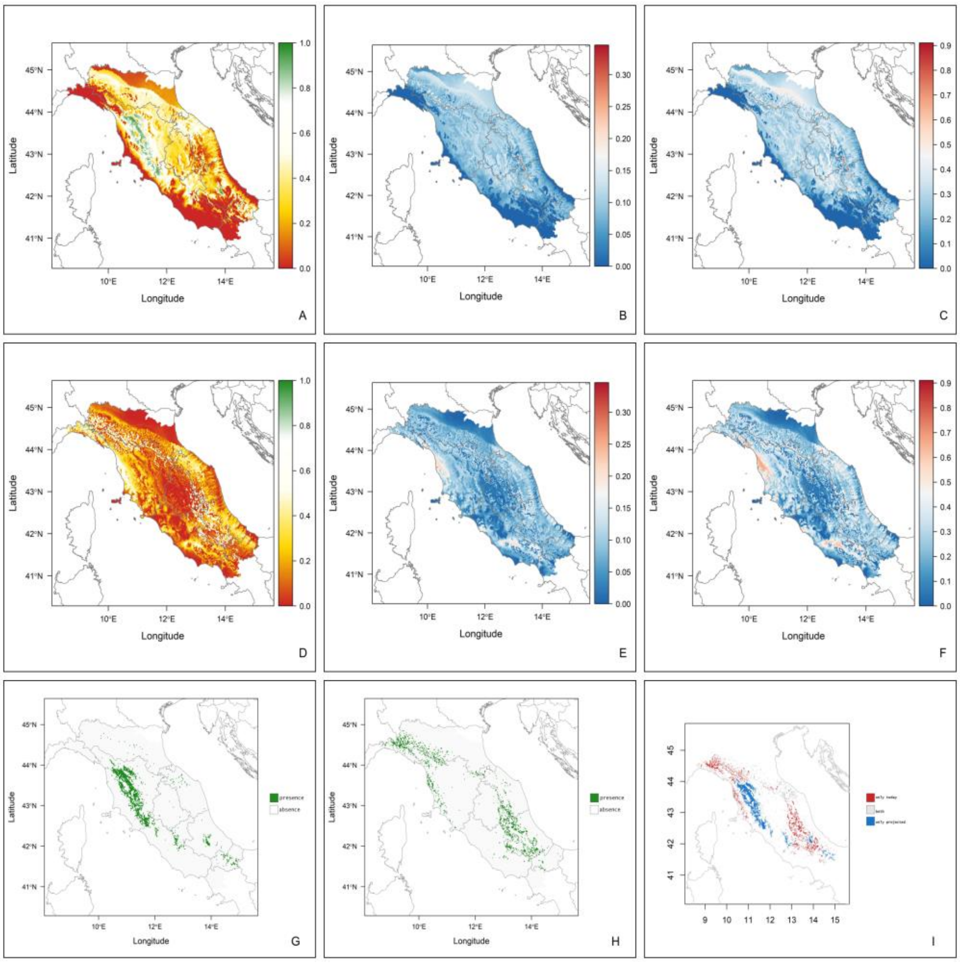

Fig. S.3.44-A) 'Projected' base output; B) 'Projected' uncertainty standard deviation; C) 'Projected' uncertainty range of values; D) 'Present' base output; E) 'Present' uncertainty standard deviation; F) 'Present' uncertainty range of values; G) 'Projected' binary distribution; H) 'Present' binary distribution; I) Range size variation.

Value	Present	Projected
AUC.train	0.754	0.852
AUC.test	0.754	0.762
AUC.diff	0.099	0.099
pROC	1.362	1.331
OR.p10	0.275	0.225
CBI	0.866	-0.086
perc.stable	0.277	0.277
range.size	2.998	2.998

S.3.45 *Cardamine apennina* GLM

Species: *Cardamine apennina* Lihová & Marhold

Procedure: GLM

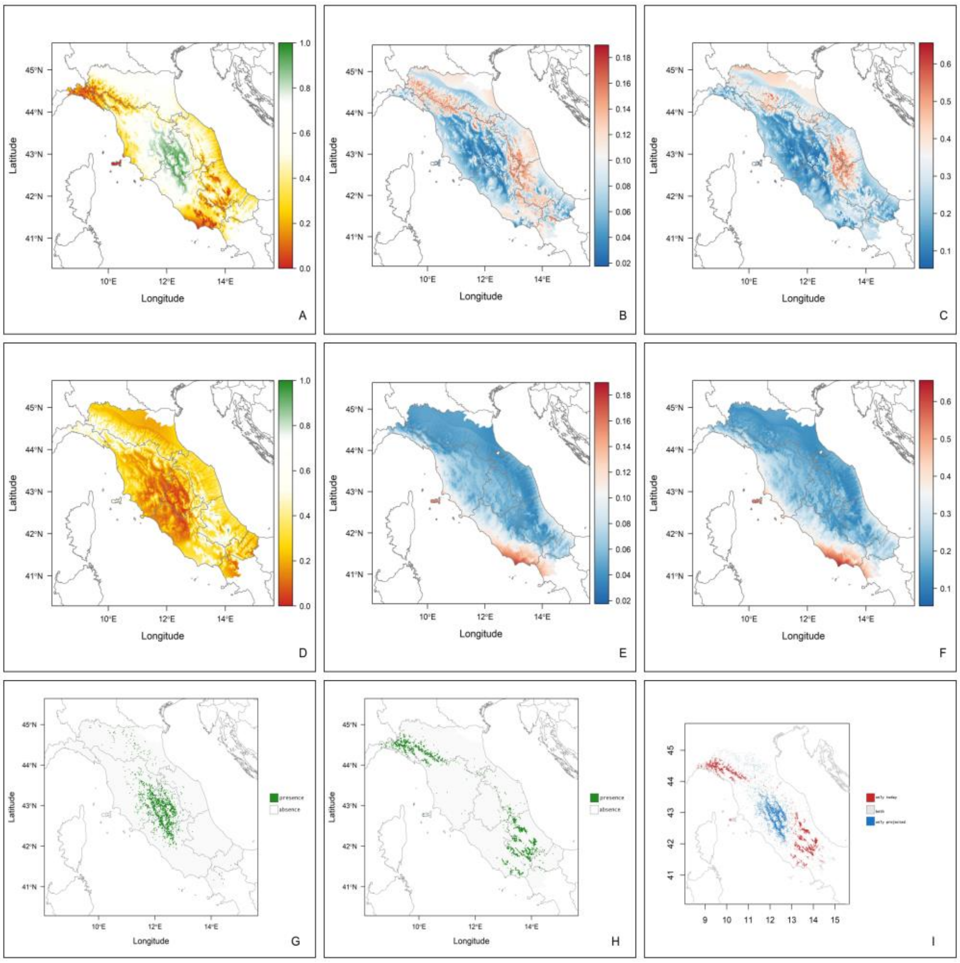

Fig. S.3.45-A) 'Projected' base output; B) 'Projected' uncertainty standard deviation; C) 'Projected' uncertainty range of values; D) 'Present' base output; E) 'Present' uncertainty standard deviation; F) 'Present' uncertainty range of values; G) 'Projected' binary distribution; H) 'Present' binary distribution; I) Range size variation.

Value	Present	Projected
AUC.train	0.621	0.673
AUC.test	0.621	0.666
AUC.diff	0.042	0.042
pROC	1.260	1.298
OR.p10	0.212	0.162
CBI	0.731	-0.583
perc.stable	0.013	0.013
range.size	0.440	0.440

S.3.46 *Cardamine apennina* MARS

Species: *Cardamine apennina* Lihová & Marhold

Procedure: MARS

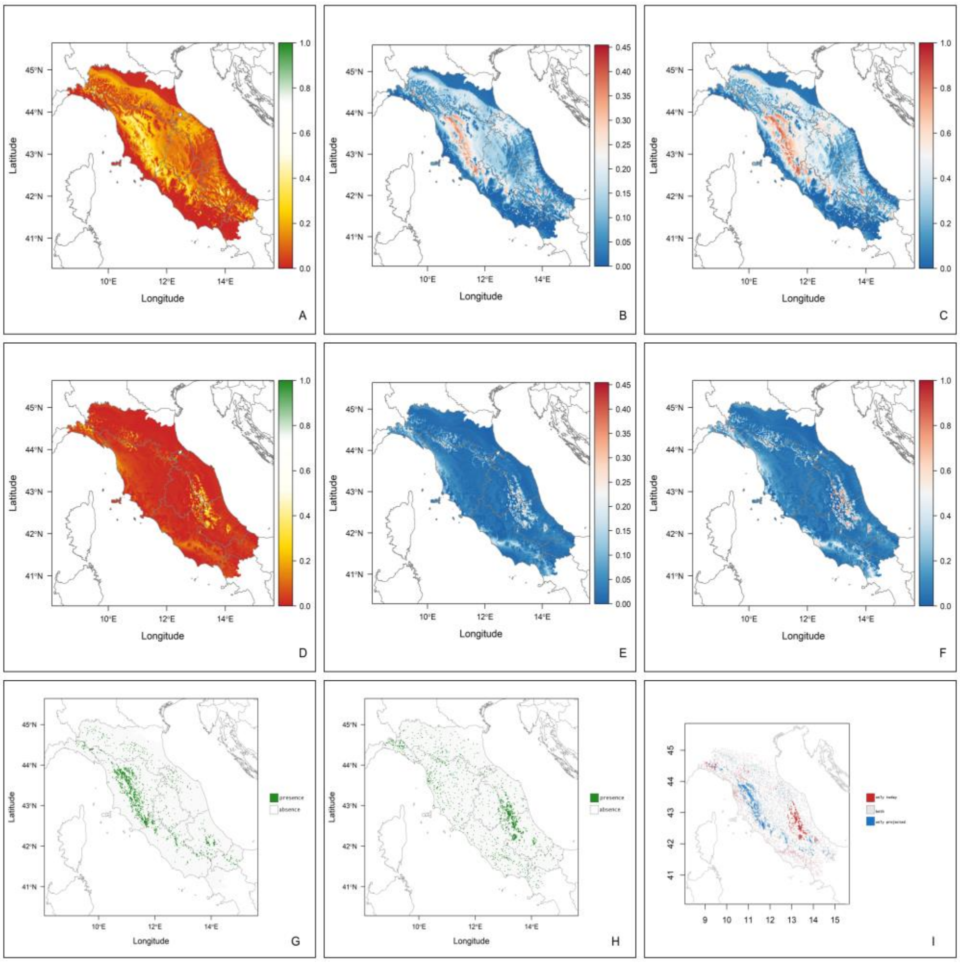

Fig. S.3.46-A) 'Projected' base output; B) 'Projected' uncertainty standard deviation; C) 'Projected' uncertainty range of values; D) 'Present' base output; E) 'Present' uncertainty standard deviation; F) 'Present' uncertainty range of values; G) 'Projected' binary distribution; H) 'Present' binary distribution; I) Range size variation.

Value	Present	Projected
AUC.train	0.797	0.904
AUC.test	0.797	0.757
AUC.diff	0.148	0.148
pROC	1.455	1.346
OR.p10	0.175	0.362
CBI	0.851	-0.365
perc.stable	1.914	1.914
range.size	3.123	3.123

S.3.47 *Cardamine apennina* RF

Species: *Cardamine apennina* Lihová & Marhold

Procedure: RF

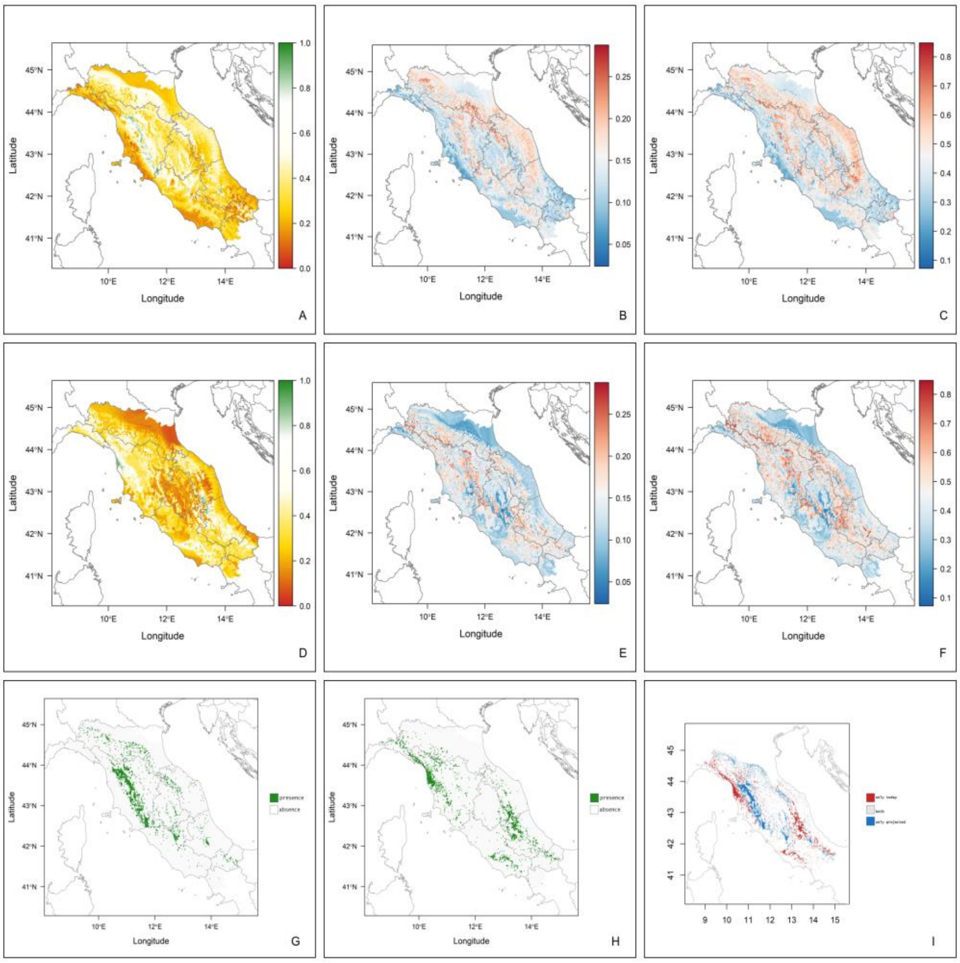

Fig. S.3.47-A) 'Projected' base output; B) 'Projected' uncertainty standard deviation; C) 'Projected' uncertainty range of values; D) 'Present' base output; E) 'Present' uncertainty standard deviation; F) 'Present' uncertainty range of values; G) 'Projected' binary distribution; H) 'Present' binary distribution; I) Range size variation.

Value	Present	Projected
AUC.train	0.929	1.000
AUC.test	0.929	0.910
AUC.diff	0.090	0.090
pROC	1.409	1.330
OR.p10	0.613	0.725
CBI	0.986	0.070
perc.stable	1.975	1.975
range.size	-1.056	-1.056

S.3.48 *Cardamine apennina* SVM

Species: *Cardamine apennina* Lihová & Marhold

Procedure: SVM

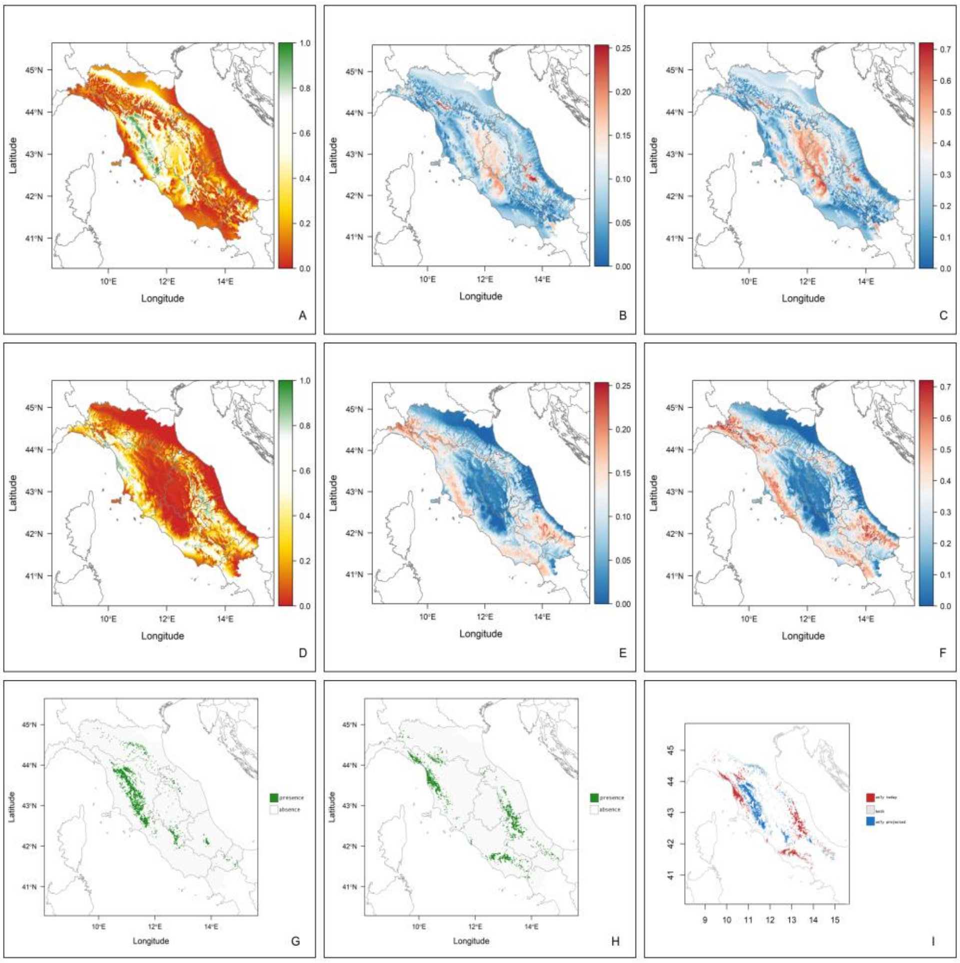

Fig. S.3.48-A) 'Projected' base output; B) 'Projected' uncertainty standard deviation; C) 'Projected' uncertainty range of values; D) 'Present' base output; E) 'Present' uncertainty standard deviation; F) 'Present' uncertainty range of values; G) 'Projected' binary distribution; H) 'Present' binary distribution; I) Range size variation.

Value	Present	Projected
AUC.train	0.862	0.877
AUC.test	0.862	0.806
AUC.diff	0.076	0.076
pROC	1.476	1.343
OR.p10	0.262	0.262
CBI	0.950	-0.178
perc.stable	0.012	0.012
range.size	2.255	2.255

S.3.49 *Carduus affinis* Biomod2

Species: *Carduus affinis* Guss. subsp. *affinis*

Procedure: Biomod2

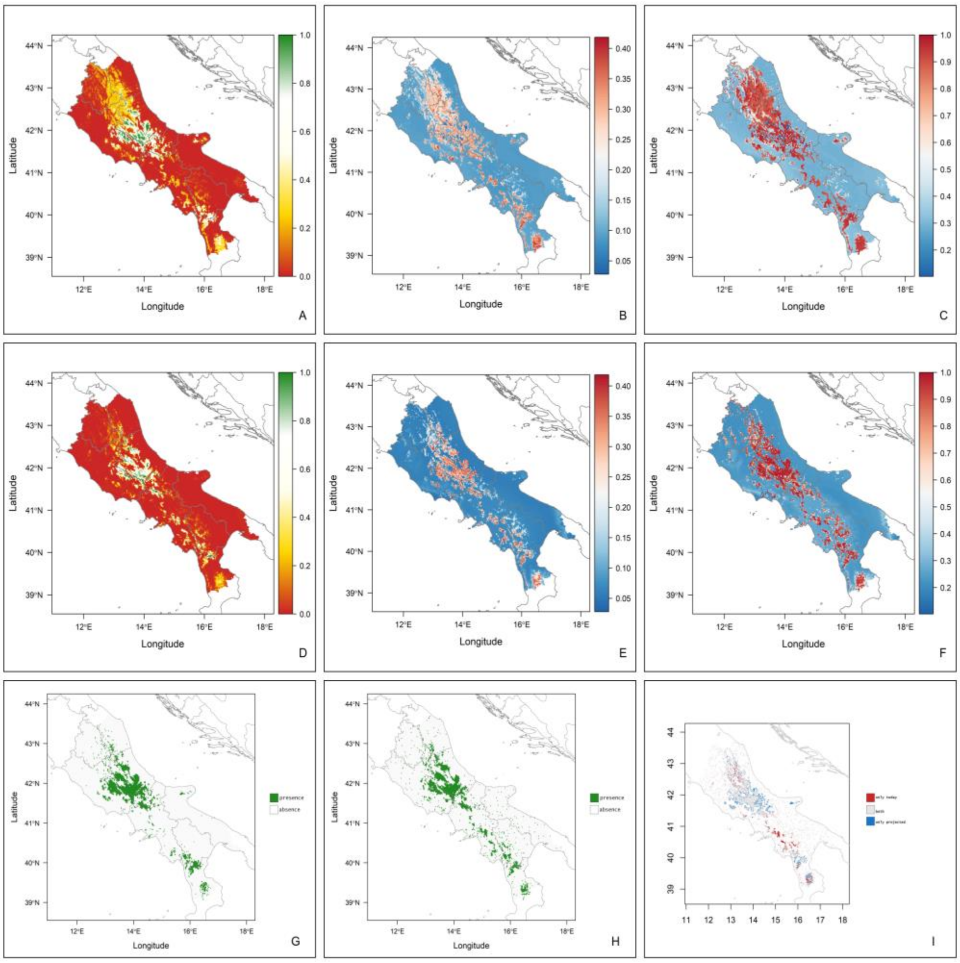

Fig. S.3.49-A) 'Projected' base output; B) 'Projected' uncertainty standard deviation; C) 'Projected' uncertainty range of values; D) 'Present' base output; E) 'Present' uncertainty standard deviation; F) 'Present' uncertainty range of values; G) 'Projected' binary distribution; H) 'Present' binary distribution; I) Range size variation.

Value	Present	Projected
AUC.train	0.946	0.987
AUC.test	0.946	0.909
AUC.diff	0.078	0.078
pROC	1.731	1.460
OR.p10	0.528	0.372
CBI	0.964	0.861
perc.stable	54.811	54.811
range.size	-0.149	-0.149

S.3.50 *Carduus affinis* Bioclim

Species: *Carduus affinis* Guss. subsp. *affinis*

Procedure: Bioclim

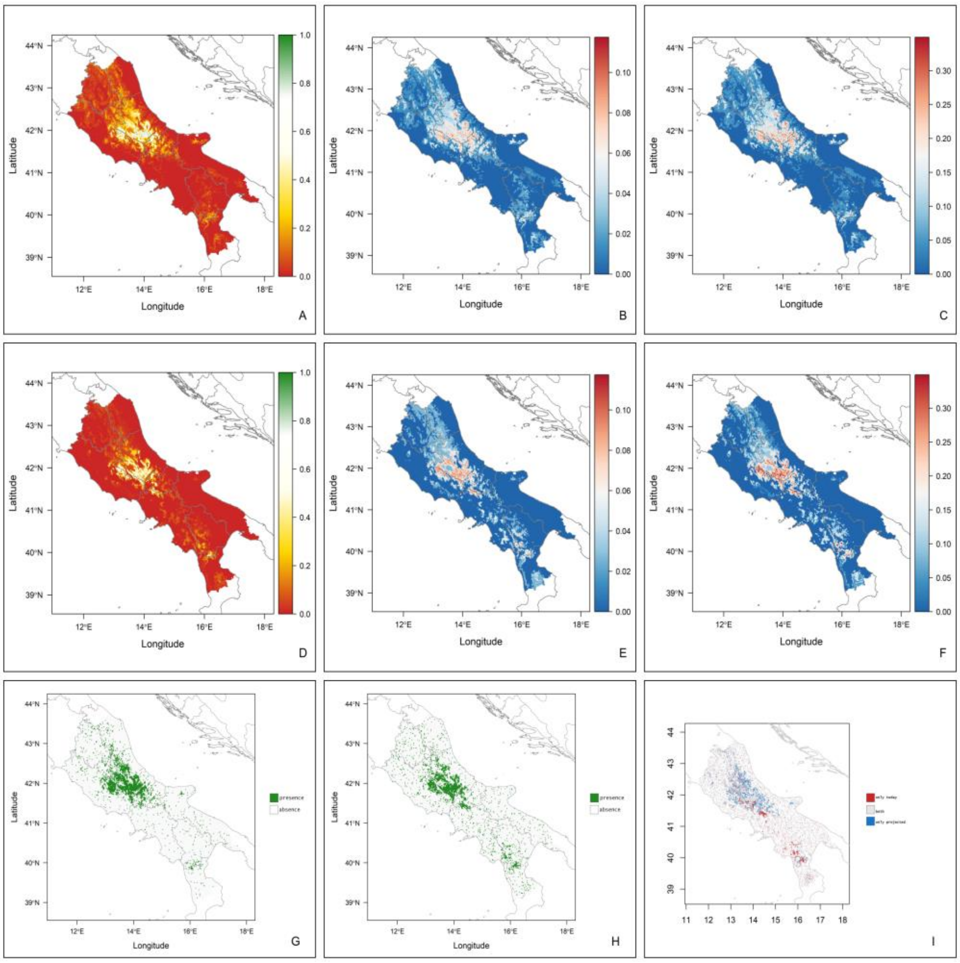

Fig. S.3.50-A) 'Projected' base output; B) 'Projected' uncertainty standard deviation; C) 'Projected' uncertainty range of values; D) 'Present' base output; E) 'Present' uncertainty standard deviation; F) 'Present' uncertainty range of values; G) 'Projected' binary distribution; H) 'Present' binary distribution; I) Range size variation.

Value	Present	Projected
AUC.train	0.828	0.870
AUC.test	0.828	0.819
AUC.diff	0.051	0.051
pROC	1.162	1.254
OR.p10	0.306	0.217
CBI	0.492	-0.027
perc.stable	29.572	29.572
range.size	-0.199	-0.199

S.3.51 *Carduus affinis* Domain

Species: *Carduus affinis* Guss. subsp. *affinis*

Procedure: Domain

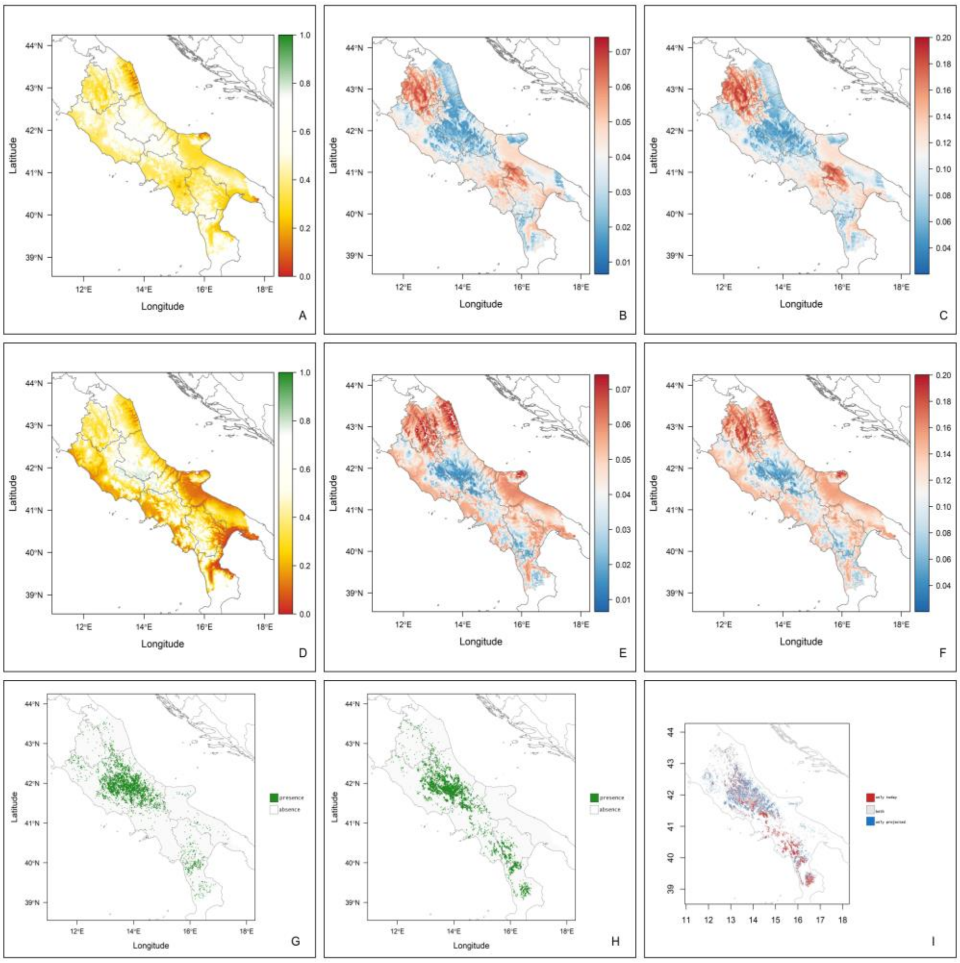

Fig. S.3.51-A) 'Projected' base output; B) 'Projected' uncertainty standard deviation; C) 'Projected' uncertainty range of values; D) 'Present' base output; E) 'Present' uncertainty standard deviation; F) 'Present' uncertainty range of values; G) 'Projected' binary distribution; H) 'Present' binary distribution; I) Range size variation.

Value	Present	Projected
AUC.train	0.907	0.863
AUC.test	0.907	0.834
AUC.diff	0.034	0.034
pROC	1.554	1.386
OR.p10	0.133	0.161
CBI	0.953	0.392
perc.stable	31.407	31.407
range.size	0.626	0.626

S.3.52 *Carduus affinis* Maxent

Species: *Carduus affinis* Guss. subsp. *affinis*

Procedure: Maxent

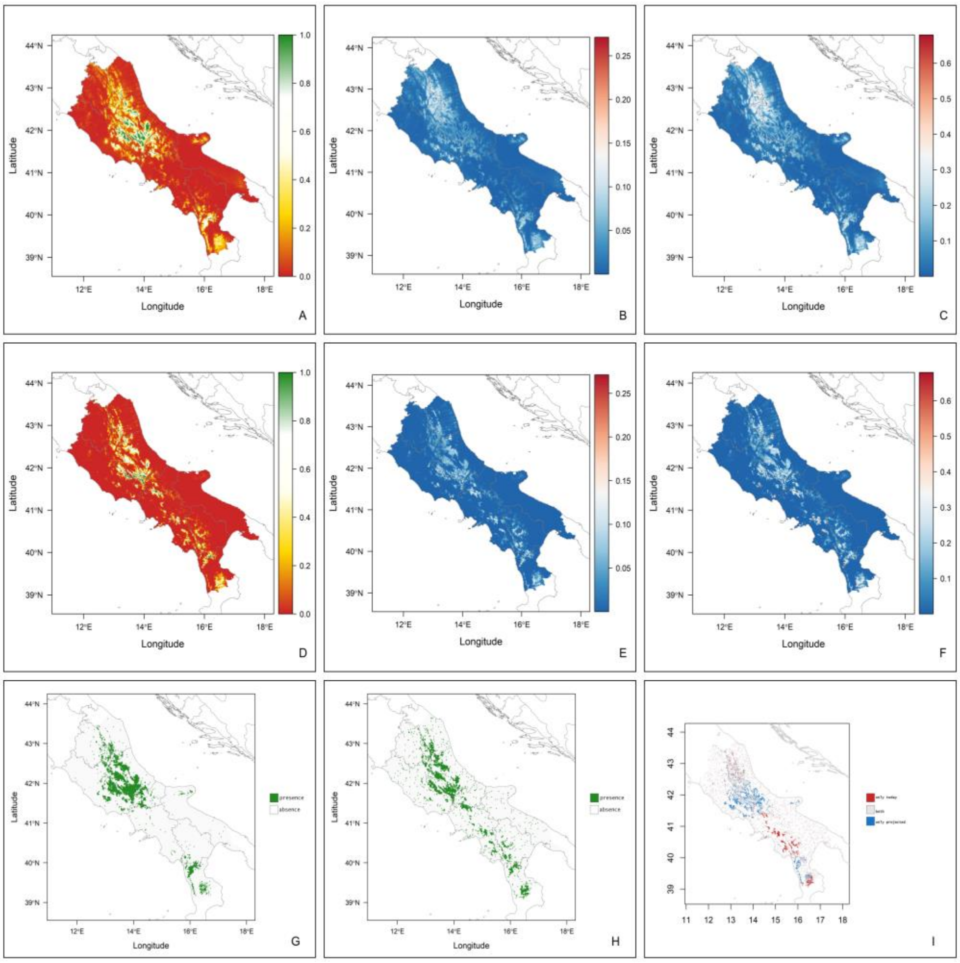

Fig. S.3.52-A) 'Projected' base output; B) 'Projected' uncertainty standard deviation; C) 'Projected' uncertainty range of values; D) 'Present' base output; E) 'Present' uncertainty standard deviation; F) 'Present' uncertainty range of values; G) 'Projected' binary distribution; H) 'Present' binary distribution; I) Range size variation.

Value	Present	Projected
AUC.train	0.956	0.926
AUC.test	0.956	0.925
AUC.diff	0.016	0.016
pROC	1.707	1.553
OR.p10	0.167	0.122
CBI	0.916	0.937
perc.stable	42.630	42.630
range.size	-0.633	-0.633

S.3.53 *Carduus affinis* KDE

Species: *Carduus affinis* Guss. subsp. *affinis*

Procedure: KDE

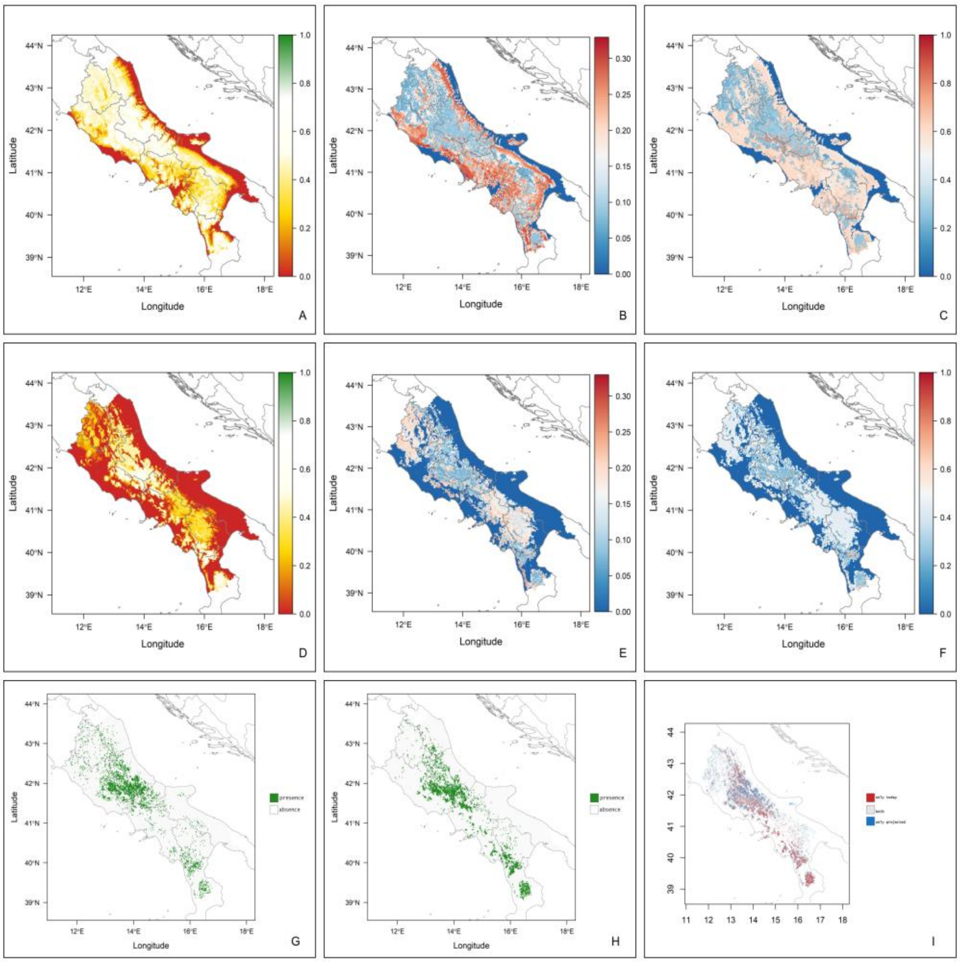

Fig. S.3.53-A) 'Projected' base output; B) 'Projected' uncertainty standard deviation; C) 'Projected' uncertainty range of values; D) 'Present' base output; E) 'Present' uncertainty standard deviation; F) 'Present' uncertainty range of values; G) 'Projected' binary distribution; H) 'Present' binary distribution; I) Range size variation.

Value	Present	Projected
AUC.train	0.913	0.895
AUC.test	0.913	0.890
AUC.diff	0.019	0.027
pROC	1.531	1.461
OR.p10	0.189	0.178
CBI	0.975	0.804
perc.stable	28.838	28.838
range.size	0.753	0.753

S.3.54 *Carduus affinis* CTA

Species: *Carduus affinis* Guss. subsp. *affinis*

Procedure: CTA

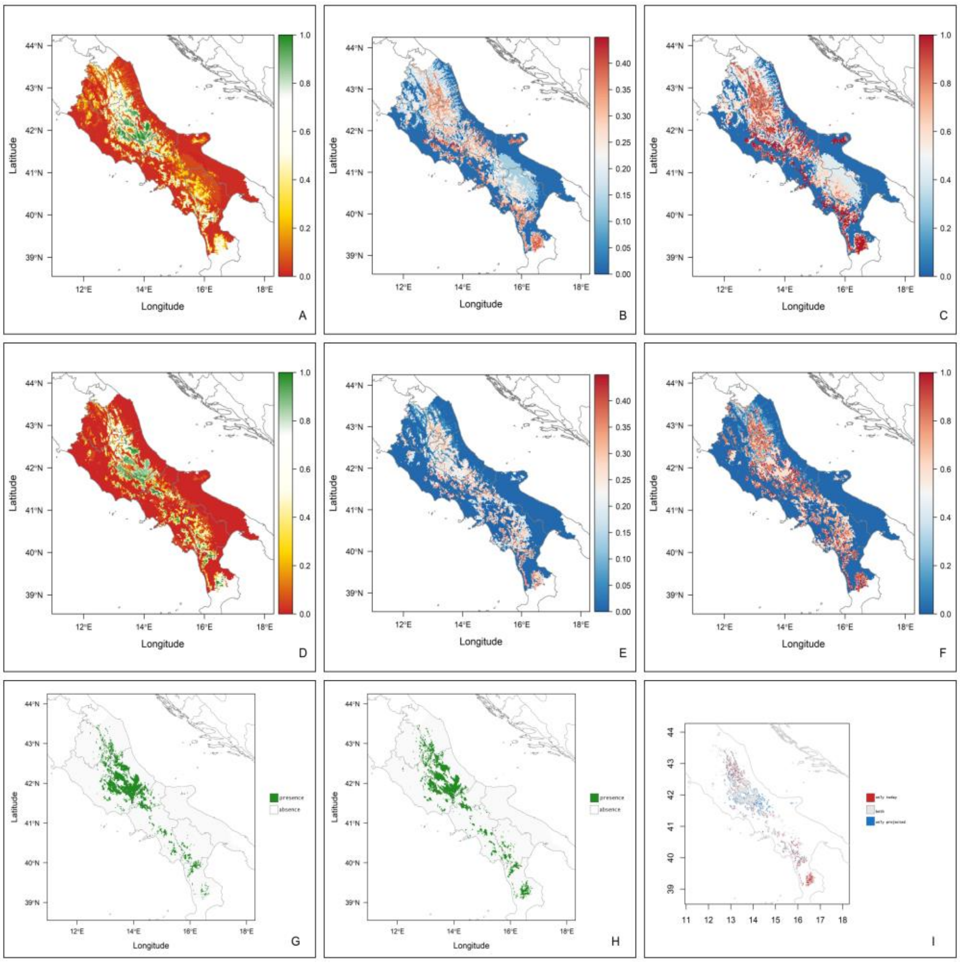

Fig. S.3.54-A) 'Projected' base output; B) 'Projected' uncertainty standard deviation; C) 'Projected' uncertainty range of values; D) 'Present' base output; E) 'Present' uncertainty standard deviation; F) 'Present' uncertainty range of values; G) 'Projected' binary distribution; H) 'Present' binary distribution; I) Range size variation.

Value	Present	Projected
AUC.train	0.956	0.960
AUC.test	0.956	0.928
AUC.diff	0.036	0.036
pROC	1.371	1.305
OR.p10	0.133	0.122
CBI	0.802	0.916
perc.stable	60.373	60.373
range.size	-0.364	-0.364

S.3.55 *Carduus affinis* FDA

Species: *Carduus affinis* Guss. subsp. *affinis*

Procedure: FDA

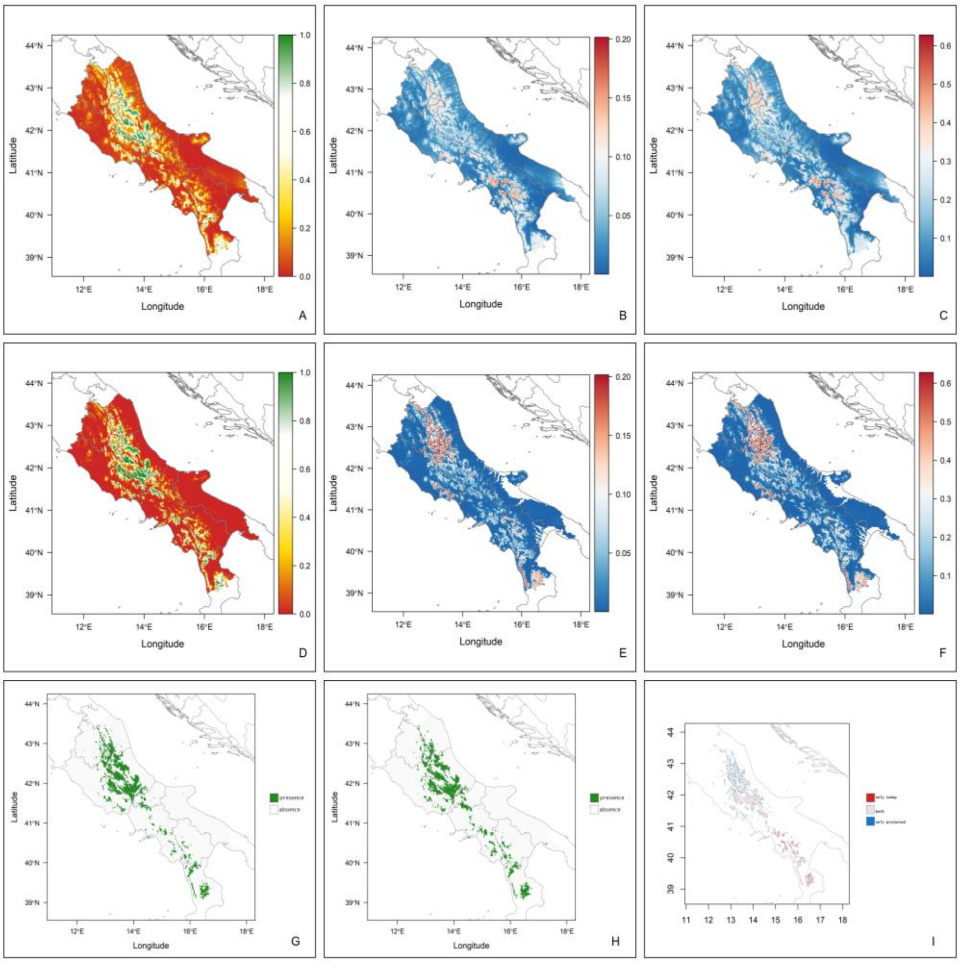

Fig. S.3.55-A) 'Projected' base output; B) 'Projected' uncertainty standard deviation; C) 'Projected' uncertainty range of values; D) 'Present' base output; E) 'Present' uncertainty standard deviation; F) 'Present' uncertainty range of values; G) 'Projected' binary distribution; H) 'Present' binary distribution; I) Range size variation.

Value	Present	Projected
AUC.train	0.954	0.909
AUC.test	0.954	0.909
AUC.diff	0.013	0.013
pROC	1.726	1.561
OR.p10	0.167	0.183
CBI	0.892	0.962
perc.stable	74.901	74.901
range.size	1.373	1.373

S.3.56 *Carduus affinis* GAM

Species: *Carduus affinis* Guss. subsp. *affinis*

Procedure: GAM

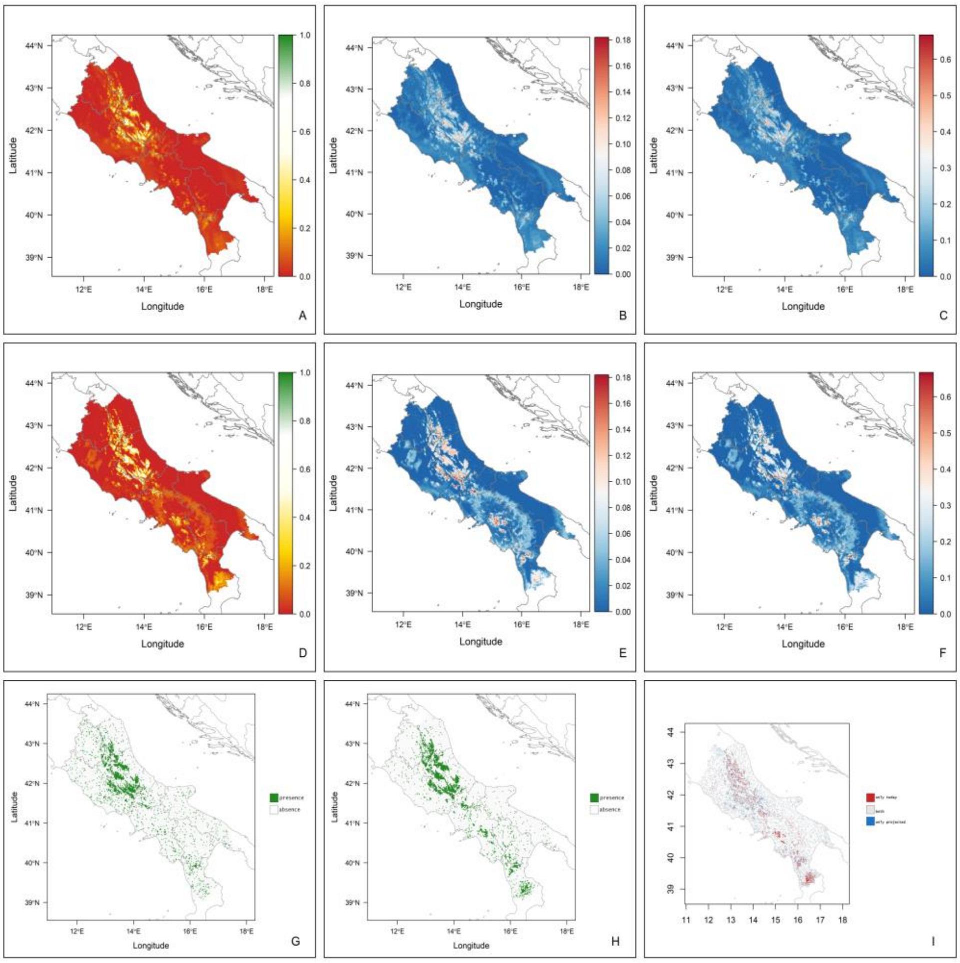

Fig. S.3.56-A) 'Projected' base output; B) 'Projected' uncertainty standard deviation; C) 'Projected' uncertainty range of values; D) 'Present' base output; E) 'Present' uncertainty standard deviation; F) 'Present' uncertainty range of values; G) 'Projected' binary distribution; H) 'Present' binary distribution; I) Range size variation.

Value	Present	Projected
AUC.train	0.947	0.929
AUC.test	0.947	0.875
AUC.diff	0.054	0.054
pROC	1.673	1.358
OR.p10	0.161	0.222
CBI	0.921	0.663
perc.stable	31.501	31.501
range.size	0.666	0.666

S.3.57 *Carduus affinis* GLM

Species: *Carduus affinis* Guss. subsp. *affinis*

Procedure: GLM

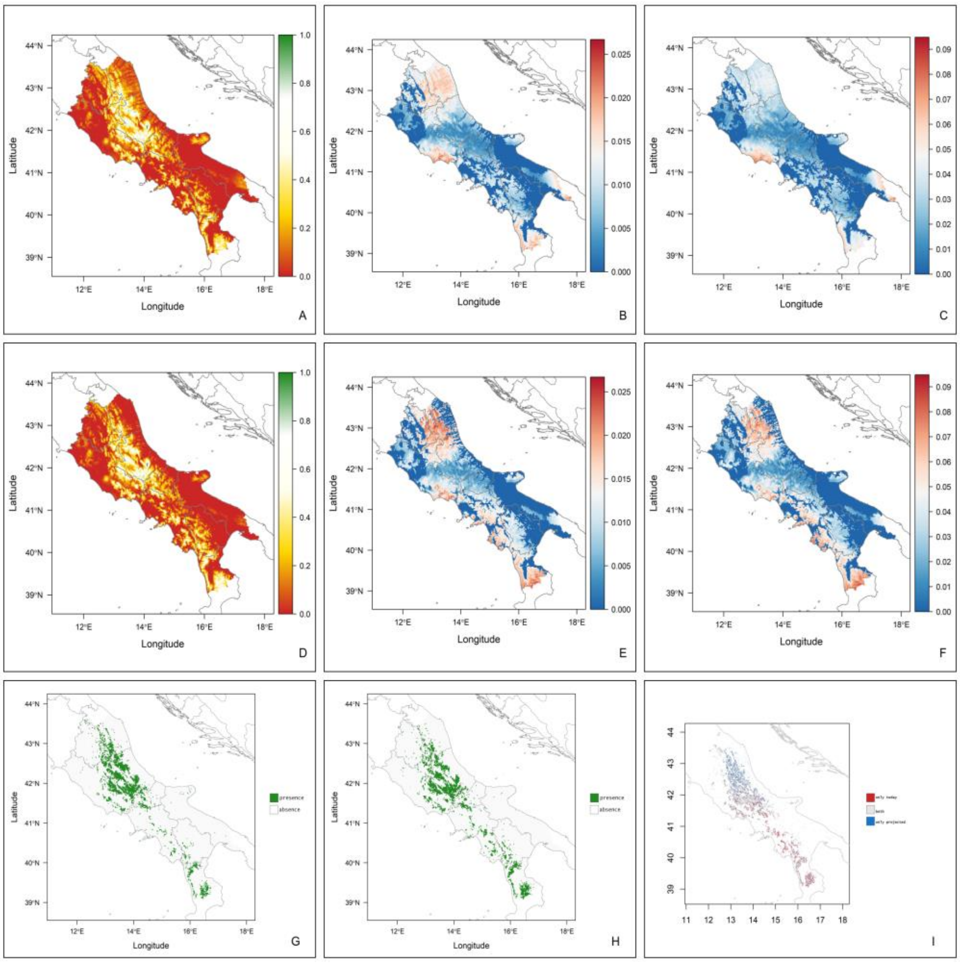

Fig. S.3.57-A) 'Projected' base output; B) 'Projected' uncertainty standard deviation; C) 'Projected' uncertainty range of values; D) 'Present' base output; E) 'Present' uncertainty standard deviation; F) 'Present' uncertainty range of values; G) 'Projected' binary distribution; H) 'Present' binary distribution; I) Range size variation.

Value	Present	Projected
AUC.train	0.941	0.910
AUC.test	0.941	0.926
AUC.diff	0.004	0.004
pROC	1.656	1.608
OR.p10	0.194	0.094
CBI	0.920	0.902
perc.stable	53.027	53.027
range.size	-0.032	-0.032

S.3.58 *Carduus affinis* MARS

Species: *Carduus affinis* Guss. subsp. *affinis*

Procedure: MARS

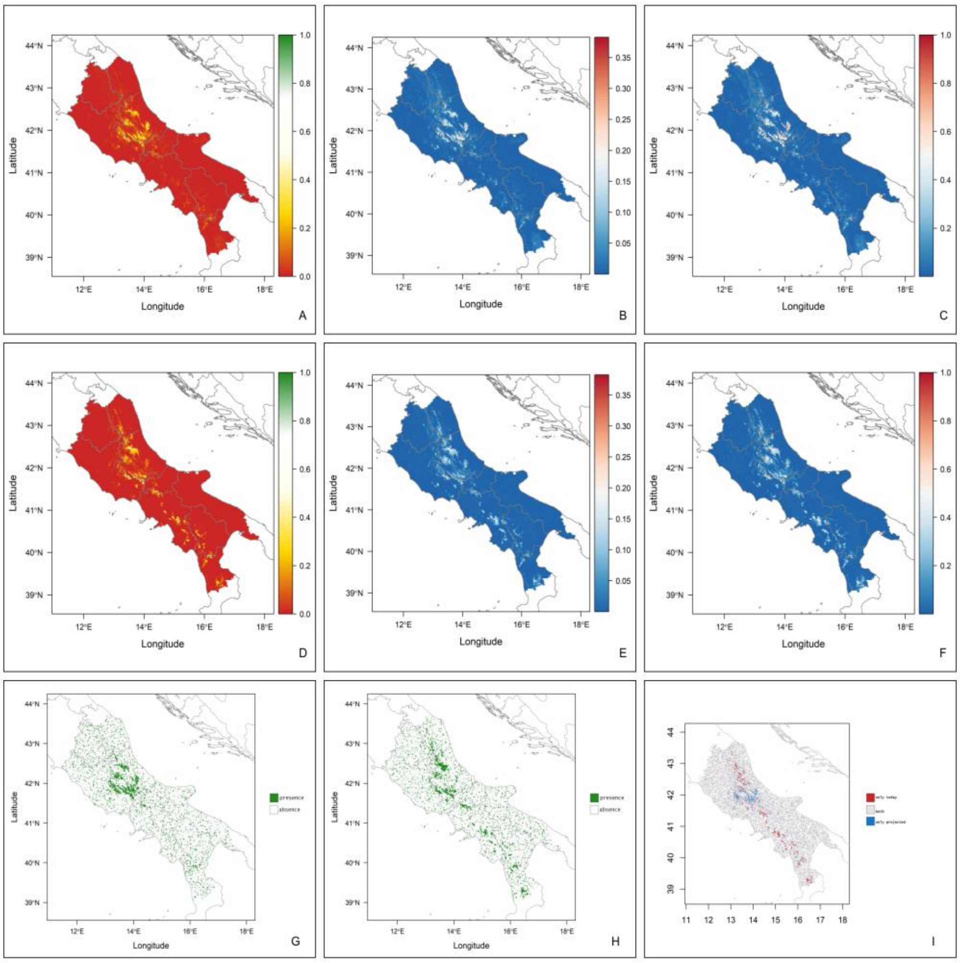

Fig. S.3.58-A) 'Projected' base output; B) 'Projected' uncertainty standard deviation; C) 'Projected' uncertainty range of values; D) 'Present' base output; E) 'Present' uncertainty standard deviation; F) 'Present' uncertainty range of values; G) 'Projected' binary distribution; H) 'Present' binary distribution; I) Range size variation.

Value	Present	Projected
AUC.train	0.909	0.928
AUC.test	0.909	0.865
AUC.diff	0.068	0.068
pROC	1.442	1.316
OR.p10	0.272	0.244
CBI	0.917	0.669
perc.stable	12.182	12.182
range.size	-1.587	-1.587

S.3.59 *Carduus affinis* RF

Species: *Carduus affinis* Guss. subsp. *affinis*

Procedure: RF

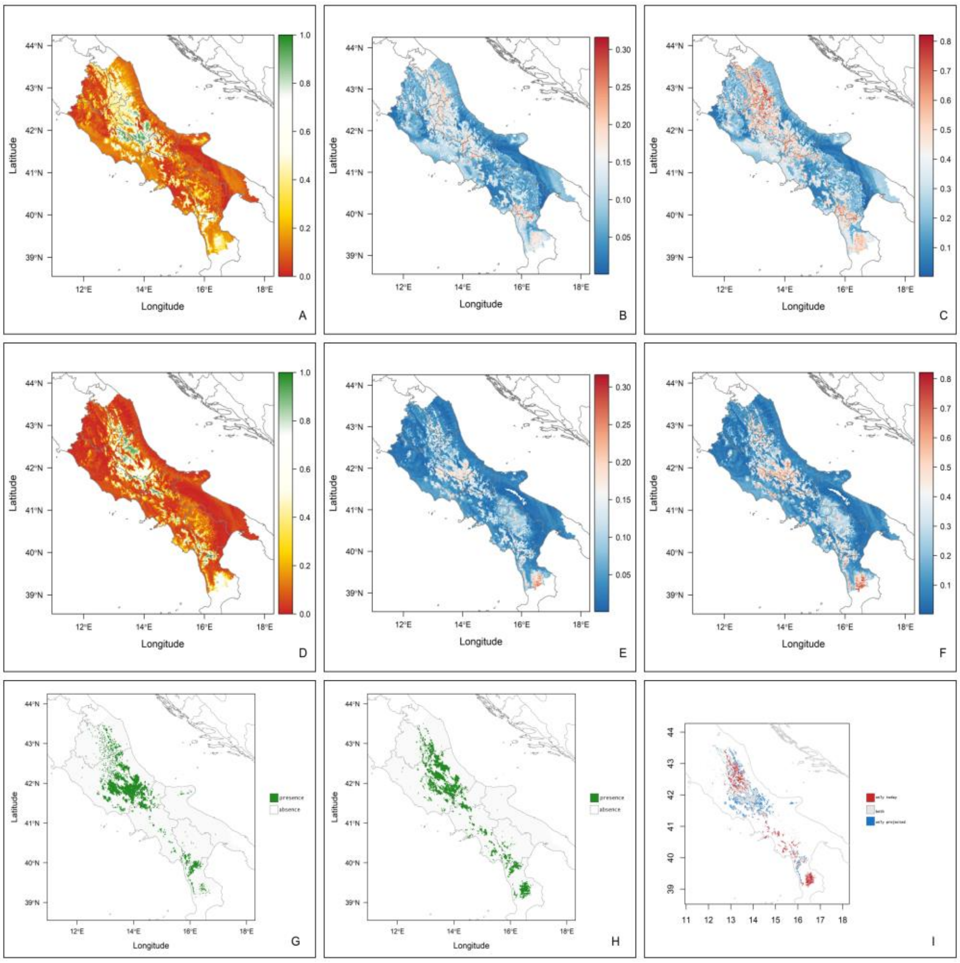

Fig. S.3.59-A) 'Projected' base output; B) 'Projected' uncertainty standard deviation; C) 'Projected' uncertainty range of values; D) 'Present' base output; E) 'Present' uncertainty standard deviation; F) 'Present' uncertainty range of values; G) 'Projected' binary distribution; H) 'Present' binary distribution; I) Range size variation.

Value	Present	Projected
AUC.train	0.985	1.000
AUC.test	0.985	0.939
AUC.diff	0.061	0.061
pROC	1.677	1.377
OR.p10	0.333	0.417
CBI	0.916	0.929
perc.stable	43.397	43.397
range.size	0.837	0.837

S.3.60 *Carduus affinis* SVM

Species: *Carduus affinis* Guss. subsp. *affinis*

Procedure: SVM

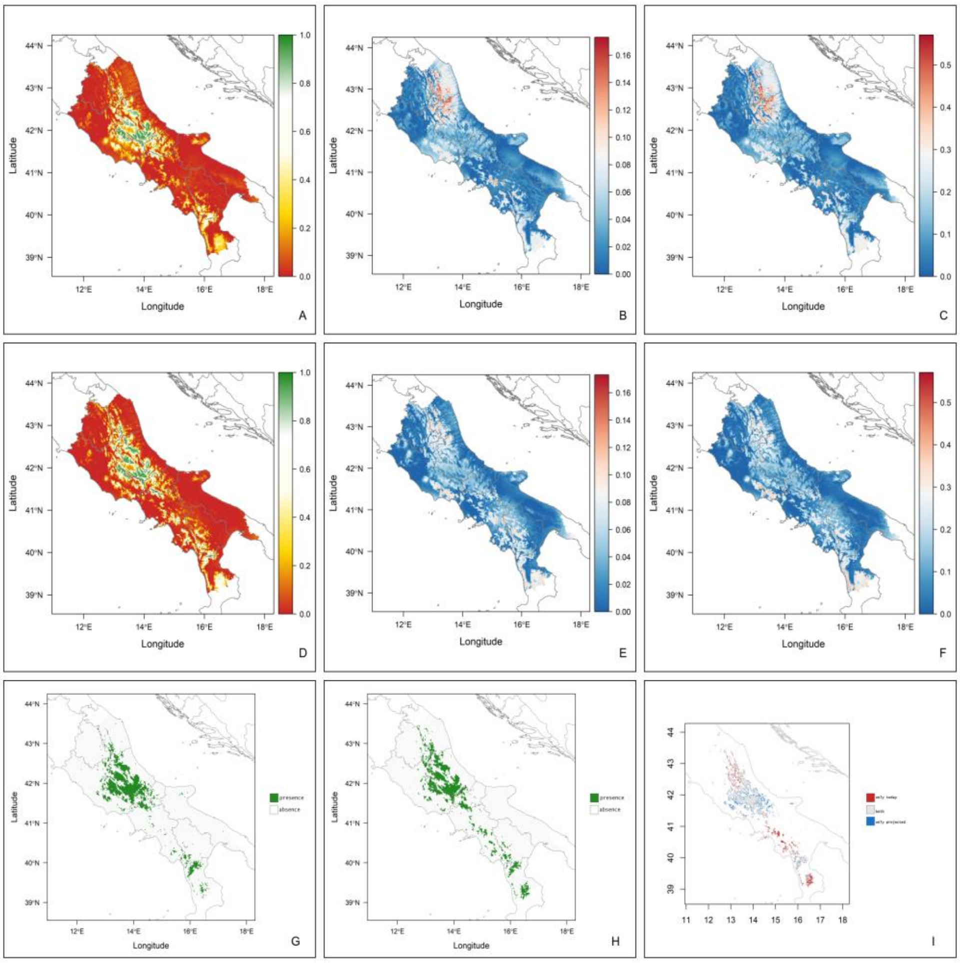

Fig. S.3.60-A) 'Projected' base output; B) 'Projected' uncertainty standard deviation; C) 'Projected' uncertainty range of values; D) 'Present' base output; E) 'Present' uncertainty standard deviation; F) 'Present' uncertainty range of values; G) 'Projected' binary distribution; H) 'Present' binary distribution; I) Range size variation.

Value	Present	Projected
AUC.train	0.958	0.935
AUC.test	0.958	0.925
AUC.diff	0.021	0.021
pROC	1.744	1.494
OR.p10	0.128	0.194
CBI	0.936	0.925
perc.stable	62.552	62.552
range.size	-0.707	-0.707

S.3.61 *Crocus biflorus* Biomod2

Species: *Crocus biflorus* Mill.

Procedure: Biomod2

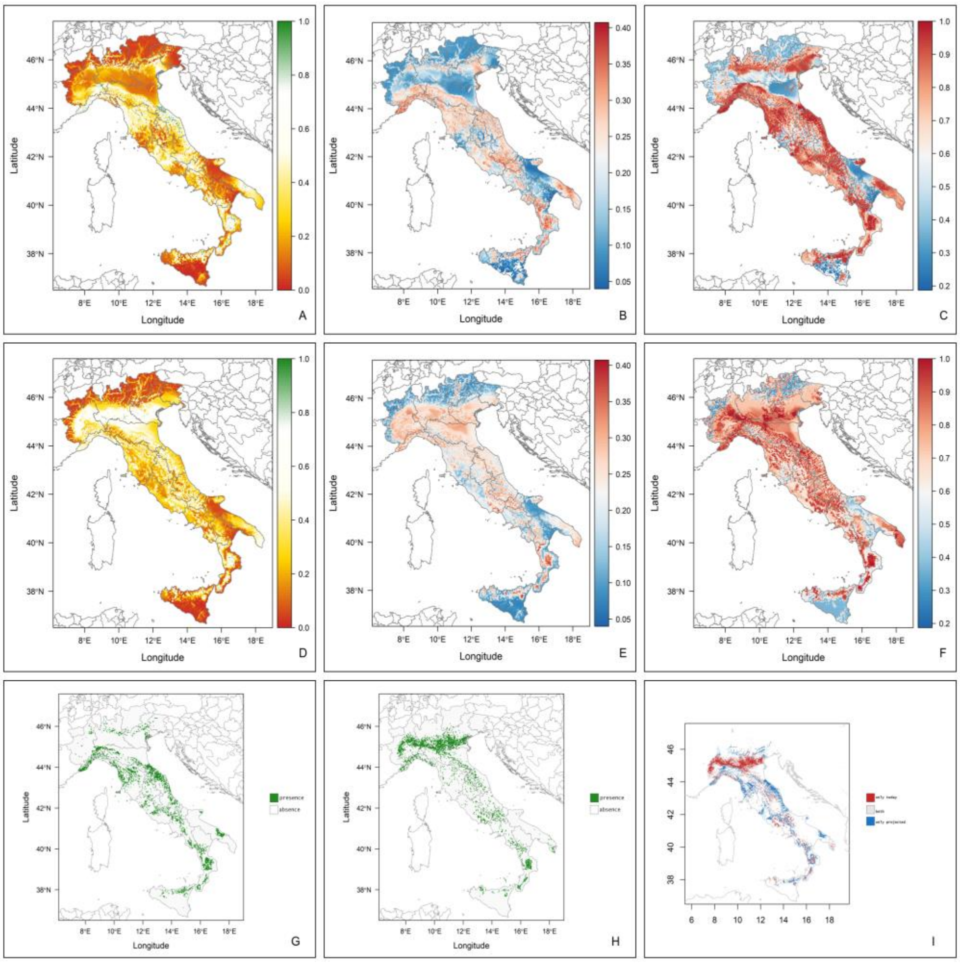

Fig. S.3.61-A) 'Projected' base output; B) 'Projected' uncertainty standard deviation; C) 'Projected' uncertainty range of values; D) 'Present' base output; E) 'Present' uncertainty standard deviation; F) 'Present' uncertainty range of values; G) 'Projected' binary distribution; H) 'Present' binary distribution; I) Range size variation.

Value	Present	Projected
AUC.train	0.671	0.959
AUC.test	0.671	0.778
AUC.diff	0.181	0.181
pROC	1.174	1.221
OR.p10	0.786	0.523
CBI	0.992	0.623
perc.stable	12.952	12.952
range.size	-0.726	-0.726

S.3.62 *Crocus biflorus* Bioclim

Species: *Crocus biflorus* Mill.

Procedure: Bioclim

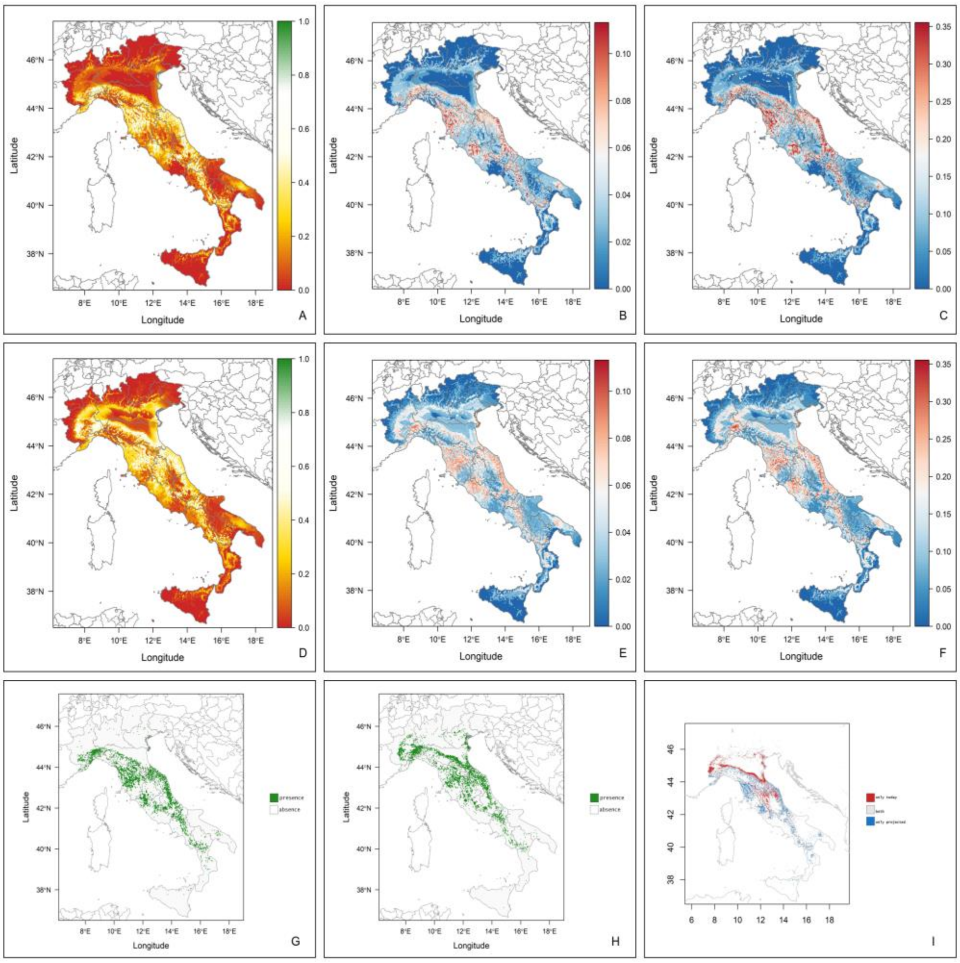

Fig. S.3.62-A) 'Projected' base output; B) 'Projected' uncertainty standard deviation; C) 'Projected' uncertainty range of values; D) 'Present' base output; E) 'Present' uncertainty standard deviation; F) 'Present' uncertainty range of values; G) 'Projected' binary distribution; H) 'Present' binary distribution; I) Range size variation.

Value	Present	Projected
AUC.train	0.617	0.696
AUC.test	0.617	0.681
AUC.diff	0.023	0.023
pROC	1.071	1.106
OR.p10	0.229	0.168
CBI	0.429	0.287
perc.stable	36.299	36.299
range.size	0.326	0.326

S.3.63 *Crocus biflorus* Domain

Species: *Crocus biflorus* Mill.

Procedure: Domain

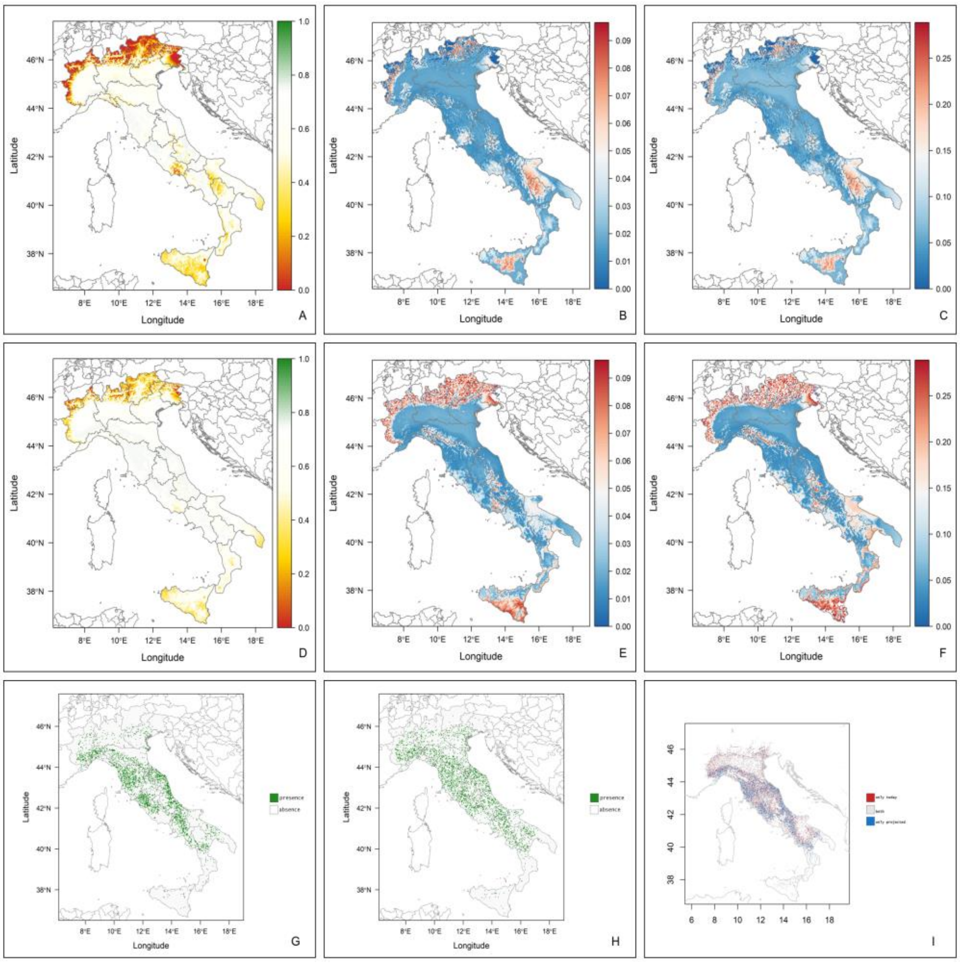

Fig. S.3.63-A) 'Projected' base output; B) 'Projected' uncertainty standard deviation; C) 'Projected' uncertainty range of values; D) 'Present' base output; E) 'Present' uncertainty standard deviation; F) 'Present' uncertainty range of values; G) 'Projected' binary distribution; H) 'Present' binary distribution; I) Range size variation.

Value	Present	Projected
AUC.train	0.544	0.652
AUC.test	0.544	0.637
AUC.diff	0.034	0.034
pROC	1.086	1.164
OR.p10	0.133	0.182
CBI	0.742	0.856
perc.stable	14.296	14.296
range.size	-0.702	-0.702

S.3.64 *Crocus biflorus* Maxent

Species: *Crocus biflorus* Mill.

Procedure: Maxent

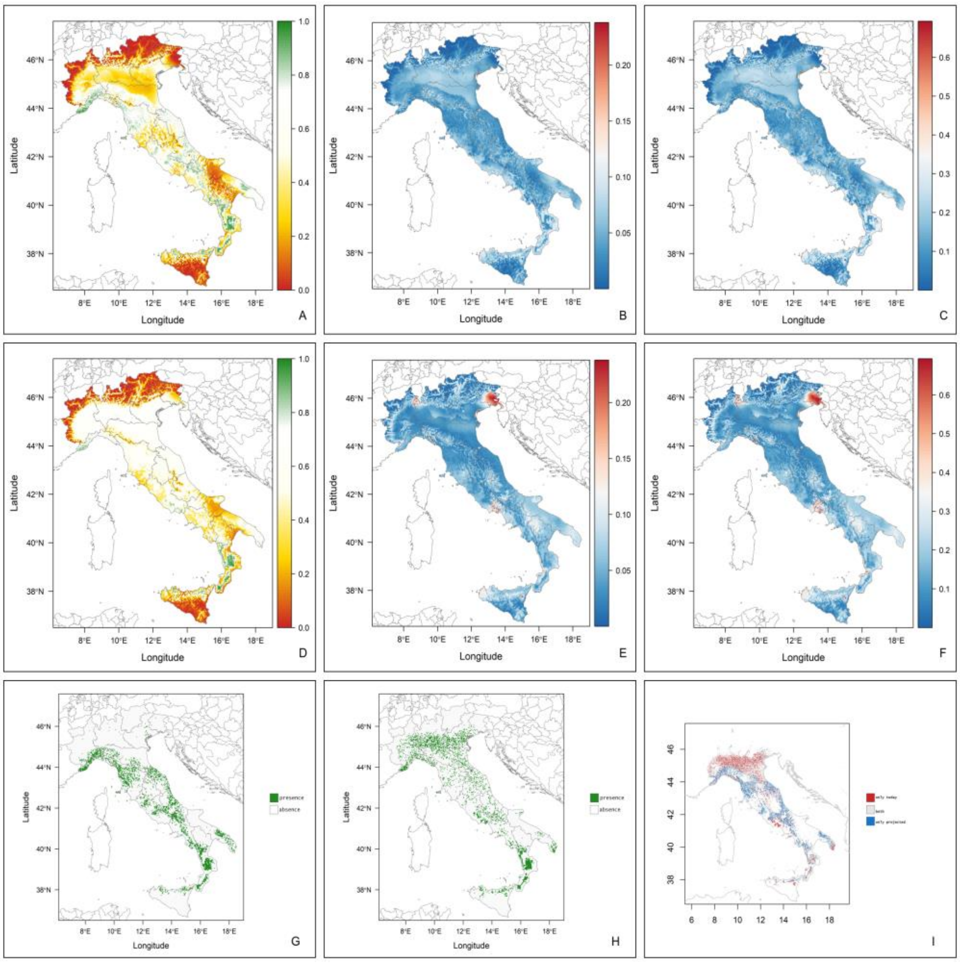

Fig. S.3.64-A) 'Projected' base output; B) 'Projected' uncertainty standard deviation; C) 'Projected' uncertainty range of values; D) 'Present' base output; E) 'Present' uncertainty standard deviation; F) 'Present' uncertainty range of values; G) 'Projected' binary distribution; H) 'Present' binary distribution; I) Range size variation.

Value	Present	Projected
AUC.train	0.686	0.762
AUC.test	0.686	0.730
AUC.diff	0.037	0.037
pROC	1.165	1.237
OR.p10	0.179	0.177
CBI	0.959	0.906
perc.stable	20.068	20.068
range.size	-0.927	-0.927

S.3.65 *Crocus biflorus* KDE

Species: *Crocus biflorus* Mill.

Procedure: KDE

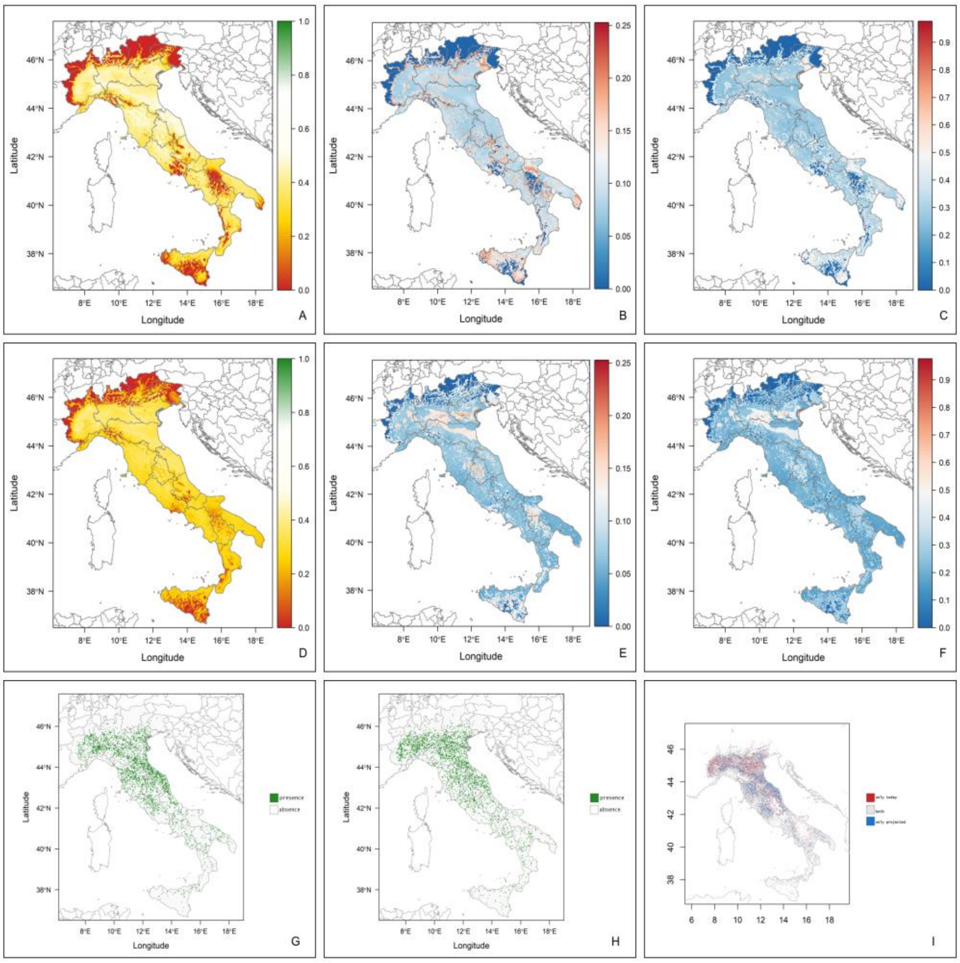

Fig. S.3.65-A) 'Projected' base output; B) 'Projected' uncertainty standard deviation; C) 'Projected' uncertainty range of values; D) 'Present' base output; E) 'Present' uncertainty standard deviation; F) 'Present' uncertainty range of values; G) 'Projected' binary distribution; H) 'Present' binary distribution; I) Range size variation.

Value	Present	Projected
AUC.train	0.620	0.671
AUC.test	0.620	0.643
AUC.diff	0.026	0.036
pROC	1.078	1.148
OR.p10	0.145	0.136
CBI	0.939	0.920
perc.stable	14.607	14.607
range.size	0.259	0.259

S.3.66 *Crocus biflorus* CTA

Species: *Crocus biflorus* Mill.

Procedure: CTA

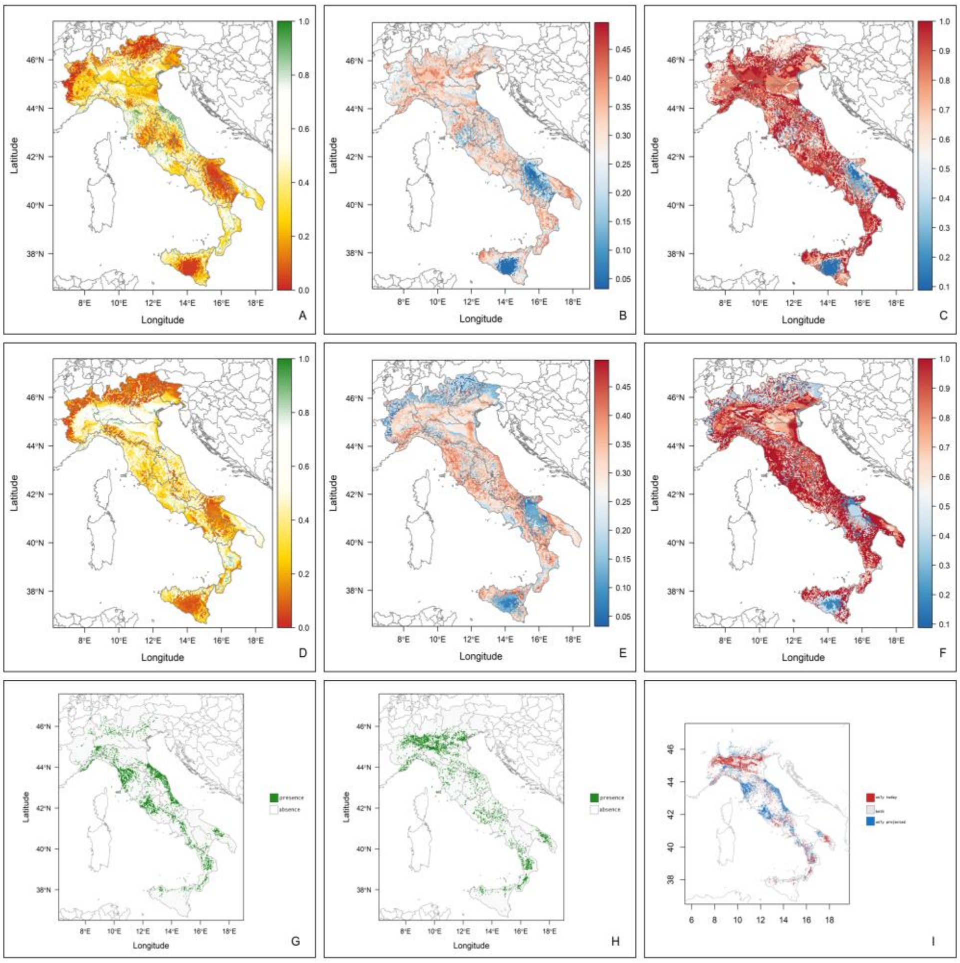

Fig. S.3.66-A) 'Projected' base output; B) 'Projected' uncertainty standard deviation; C) 'Projected' uncertainty range of values; D) 'Present' base output; E) 'Present' uncertainty standard deviation; F) 'Present' uncertainty range of values; G) 'Projected' binary distribution; H) 'Present' binary distribution; I) Range size variation.

Value	Present	Projected
AUC.train	0.771	0.921
AUC.test	0.771	0.769
AUC.diff	0.152	0.152
pROC	1.064	1.067
OR.p10	0.275	0.336
CBI	0.972	0.670
perc.stable	12.490	12.490
range.size	-0.365	-0.365

S.3.67 *Crocus biflorus* FDA

Species: *Crocus biflorus* Mill.

Procedure: FDA

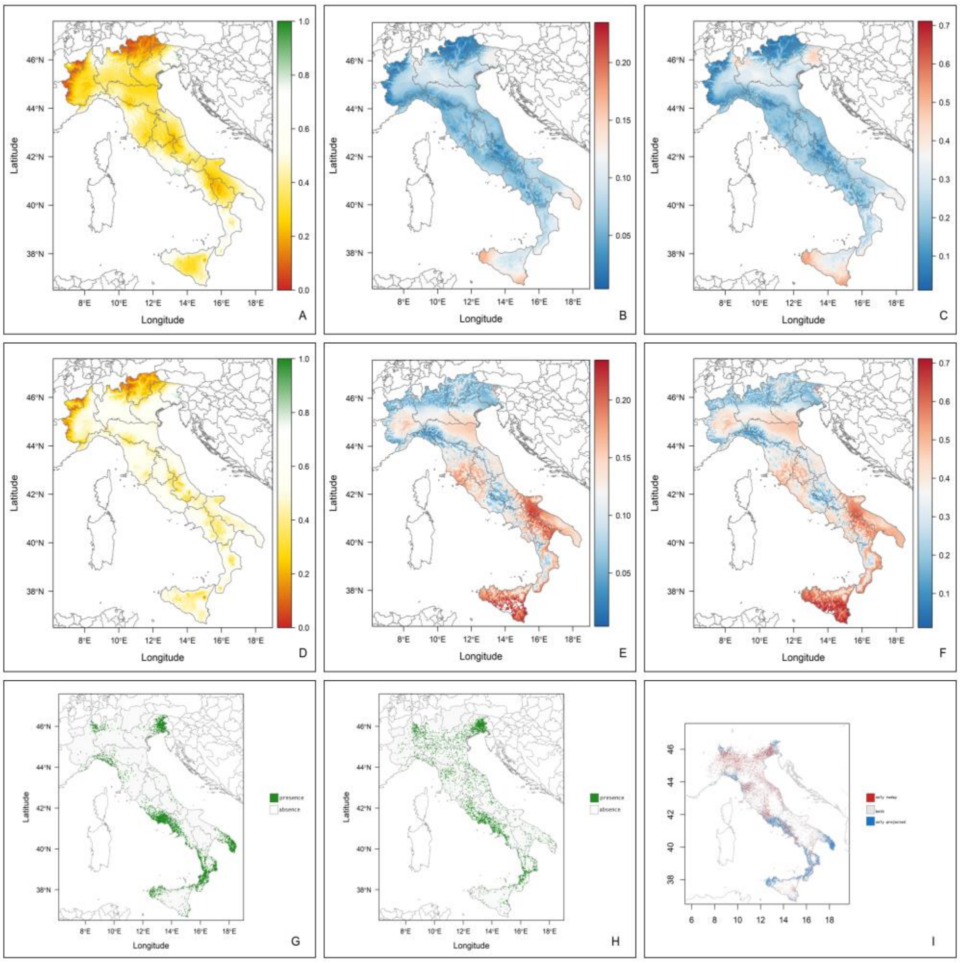

Fig. S.3.67-A) 'Projected' base output; B) 'Projected' uncertainty standard deviation; C) 'Projected' uncertainty range of values; D) 'Present' base output; E) 'Present' uncertainty standard deviation; F) 'Present' uncertainty range of values; G) 'Projected' binary distribution; H) 'Present' binary distribution; I) Range size variation.

Value	Present	Projected
AUC.train	0.593	0.642
AUC.test	0.593	0.613
AUC.diff	0.048	0.048
pROC	1.095	1.139
OR.p10	0.130	0.191
CBI	0.660	0.792
perc.stable	22.254	22.254
range.size	-1.290	-1.290

S.3.68 *Crocus biflorus* GAM

Species: *Crocus biflorus* Mill.

Procedure: GAM

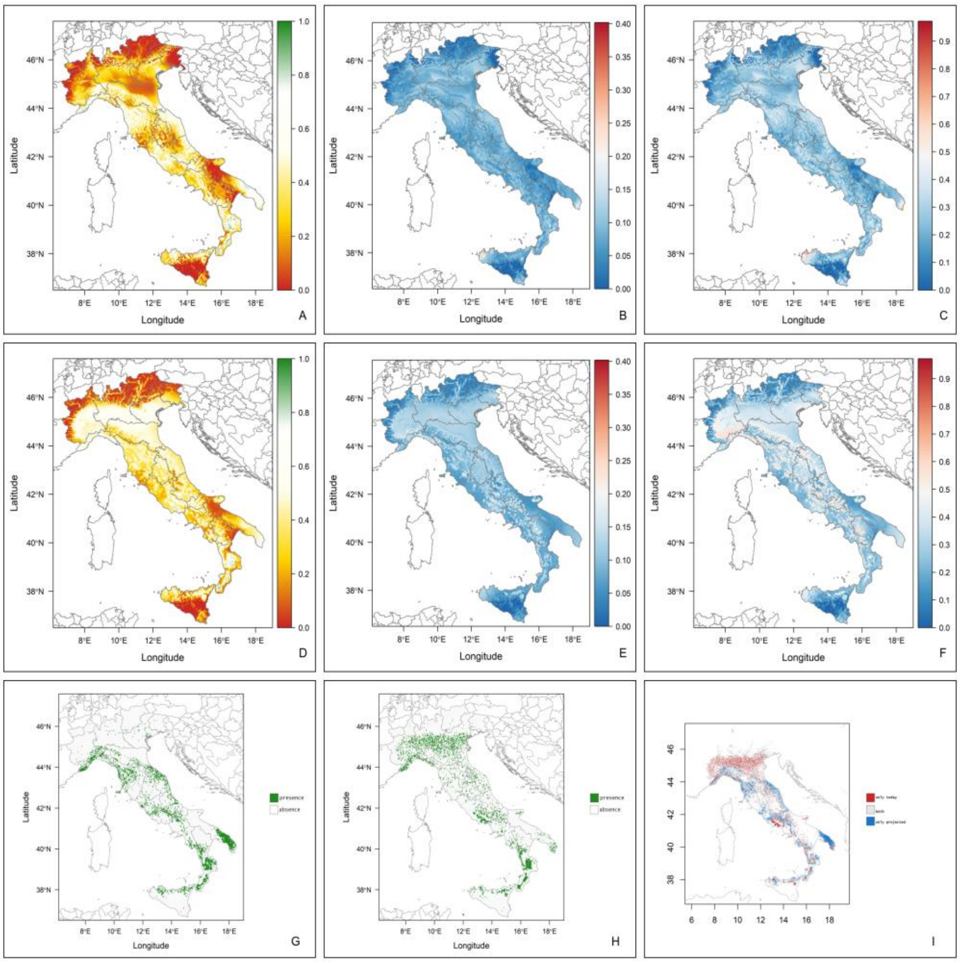

Fig. S.3.68-A) 'Projected' base output; B) 'Projected' uncertainty standard deviation; C) 'Projected' uncertainty range of values; D) 'Present' base output; E) 'Present' uncertainty standard deviation; F) 'Present' uncertainty range of values; G) 'Projected' binary distribution; H) 'Present' binary distribution; I) Range size variation.

Value	Present	Projected
AUC.train	0.636	0.775
AUC.test	0.636	0.722
AUC.diff	0.055	0.055
pROC	1.139	1.192
OR.p10	0.204	0.186
CBI	0.944	0.775
perc.stable	15.069	15.069
range.size	2.139	2.139

S.3.69 *Crocus biflorus* GLM

Species: *Crocus biflorus* Mill.

Procedure: GLM

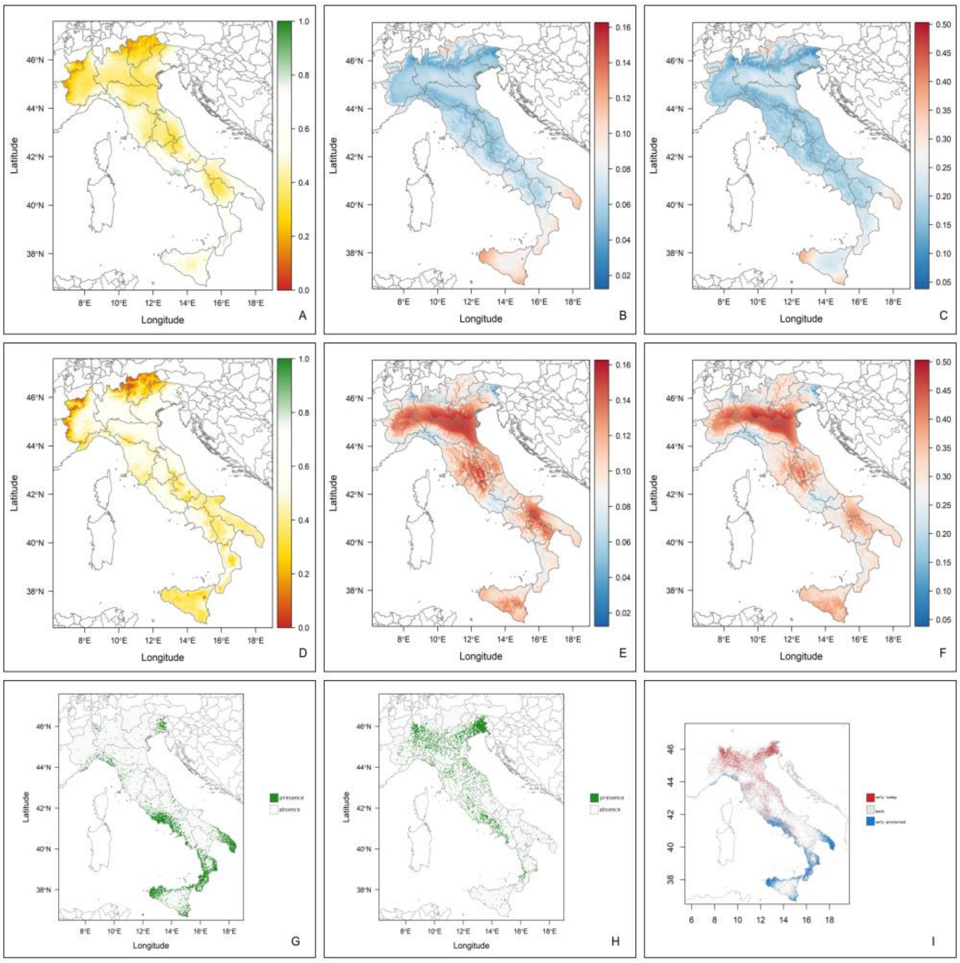

Fig. S.3.69-A) 'Projected' base output; B) 'Projected' uncertainty standard deviation; C) 'Projected' uncertainty range of values; D) 'Present' base output; E) 'Present' uncertainty standard deviation; F) 'Present' uncertainty range of values; G) 'Projected' binary distribution; H) 'Present' binary distribution; I) Range size variation.

Value	Present	Projected
AUC.train	0.554	0.614
AUC.test	0.554	0.600
AUC.diff	0.030	0.030
pROC	1.092	1.162
OR.p10	0.143	0.150
CBI	0.674	0.178
perc.stable	9.735	9.735
range.size	0.888	0.888

S.3.70 *Crocus biflorus* MARS

Species: *Crocus biflorus* Mill.

Procedure: MARS

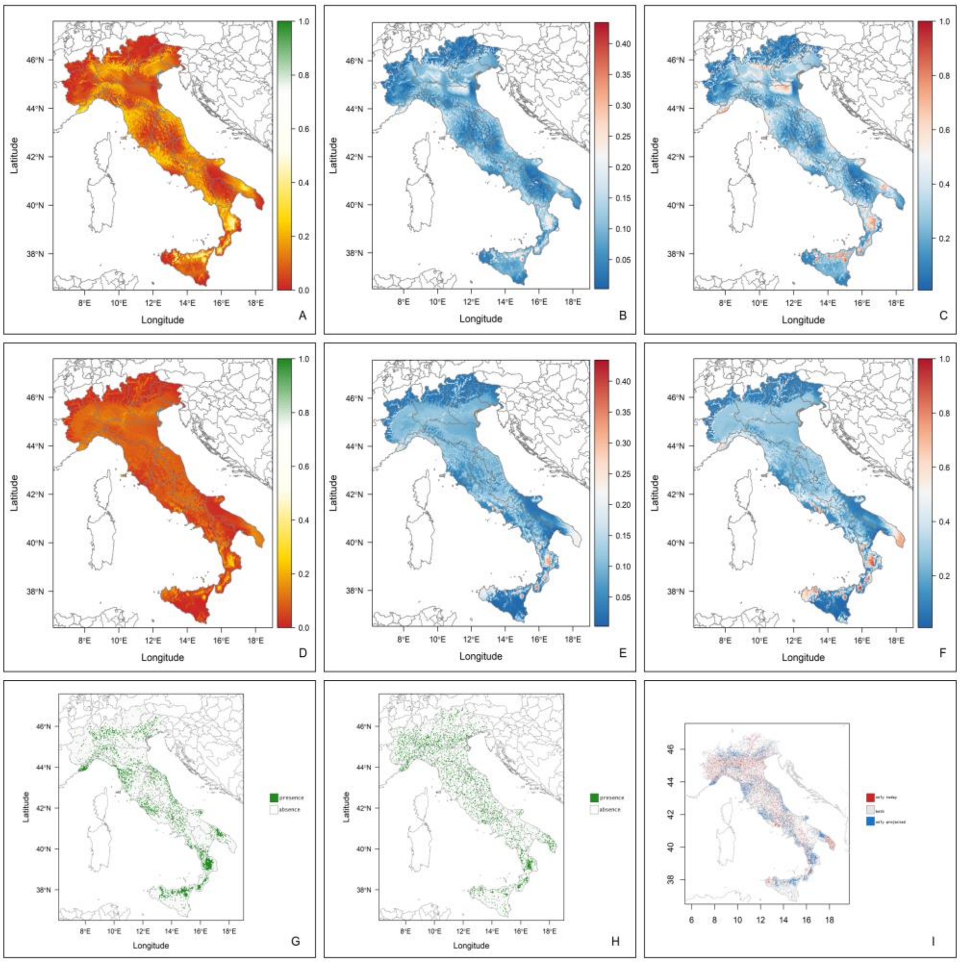

Fig. S.3.70-A) 'Projected' base output; B) 'Projected' uncertainty standard deviation; C) 'Projected' uncertainty range of values; D) 'Present' base output; E) 'Present' uncertainty standard deviation; F) 'Present' uncertainty range of values; G) 'Projected' binary distribution; H) 'Present' binary distribution; I) Range size variation.

Value	Present	Projected
AUC.train	0.605	0.800
AUC.test	0.605	0.716
AUC.diff	0.084	0.084
pROC	1.094	1.177
OR.p10	0.207	0.250
CBI	0.807	0.722
perc.stable	10.615	10.615
range.size	-0.360	-0.360

S.3.71 *Crocus biflorus* RF

Species: *Crocus biflorus* Mill.

Procedure: RF

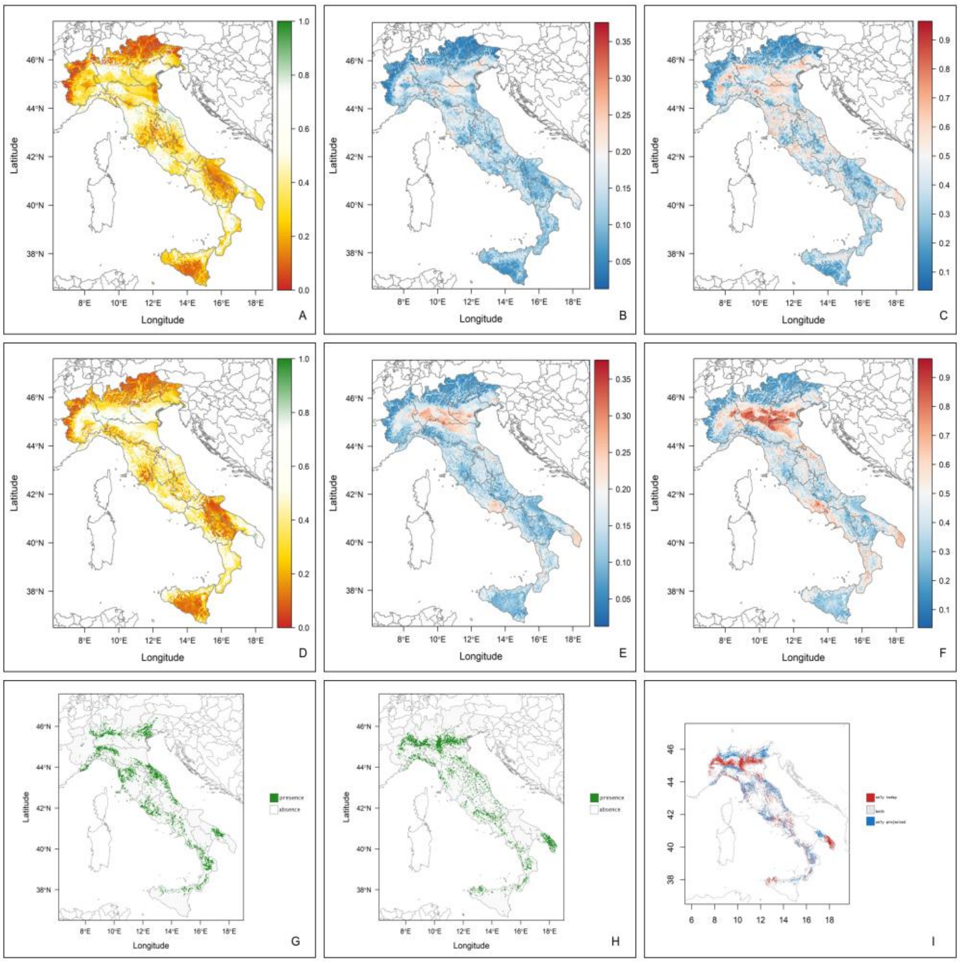

Fig. S.3.71-A) 'Projected' base output; B) 'Projected' uncertainty standard deviation; C) 'Projected' uncertainty range of values; D) 'Present' base output; E) 'Present' uncertainty standard deviation; F) 'Present' uncertainty range of values; G) 'Projected' binary distribution; H) 'Present' binary distribution; I) Range size variation.

Value	Present	Projected
AUC.train	0.925	1.000
AUC.test	0.925	0.908
AUC.diff	0.092	0.092
pROC	1.134	1.120
OR.p10	0.863	0.768
CBI	0.955	0.889
perc.stable	12.386	12.386
range.size	-0.560	-0.560

S.3.72 *Crocus biflorus* SVM

Species: *Crocus biflorus* Mill.

Procedure: SVM

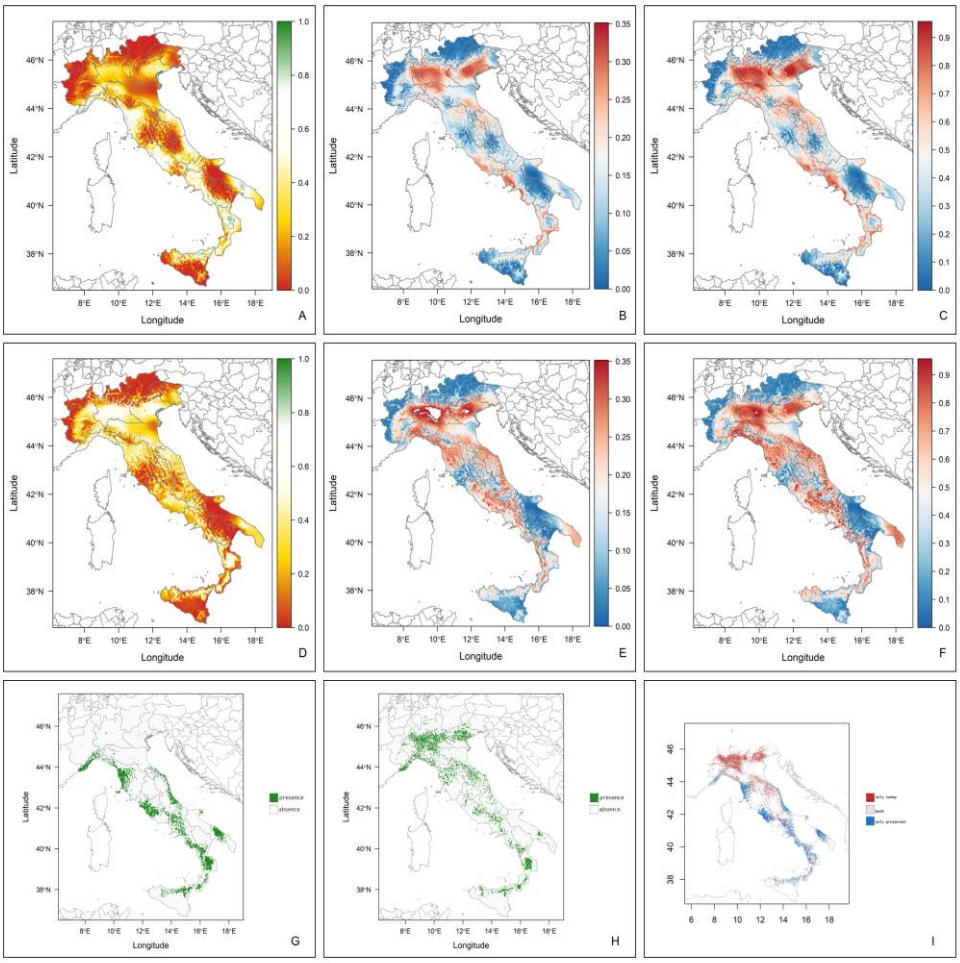

Fig. S.3.72-A) 'Projected' base output; B) 'Projected' uncertainty standard deviation; C) 'Projected' uncertainty range of values; D) 'Present' base output; E) 'Present' uncertainty standard deviation; F) 'Present' uncertainty range of values; G) 'Projected' binary distribution; H) 'Present' binary distribution; I) Range size variation.

Value	Present	Projected
AUC.train	0.720	0.817
AUC.test	0.720	0.763
AUC.diff	0.057	0.057
pROC	1.141	1.157
OR.p10	0.198	0.195
CBI	0.911	0.717
perc.stable	20.134	20.134
range.size	-0.230	-0.230

S.3.73 *Crocus etruscus* Biomod2

Species: *Crocus etruscus* Parl.

Procedure: Biomod2

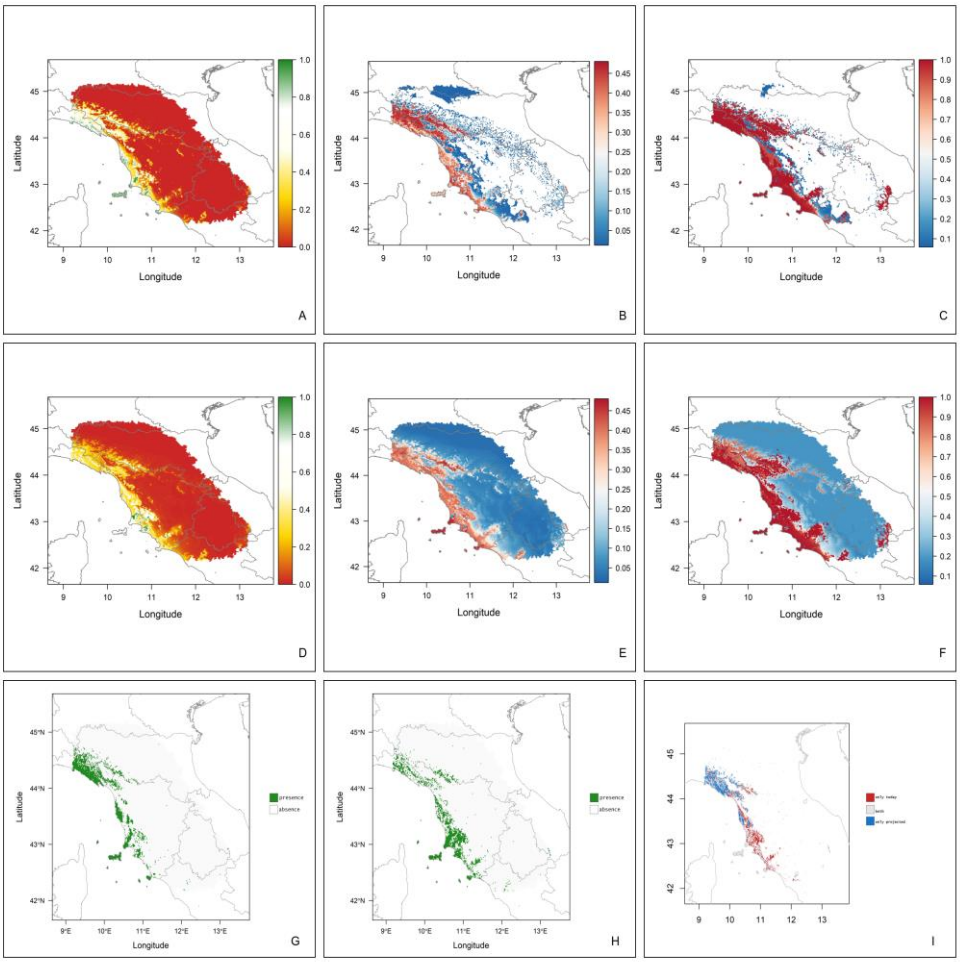

Fig. S.3.73-A) 'Projected' base output; B) 'Projected' uncertainty standard deviation; C) 'Projected' uncertainty range of values; D) 'Present' base output; E) 'Present' uncertainty standard deviation; F) 'Present' uncertainty range of values; G) 'Projected' binary distribution; H) 'Present' binary distribution; I) Range size variation.

Value	Present	Projected
AUC.train	0.994	1.000
AUC.test	0.994	0.929
AUC.diff	0.071	0.071
pROC	1.970	1.856
OR.p10	0.950	0.550
CBI	0.853	0.715
perc.stable	33.691	33.691
range.size	0.659	0.659

S.3.74 *Crocus etruscus* Bioclim

Species: *Crocus etruscus* Parl.

Procedure: Bioclim

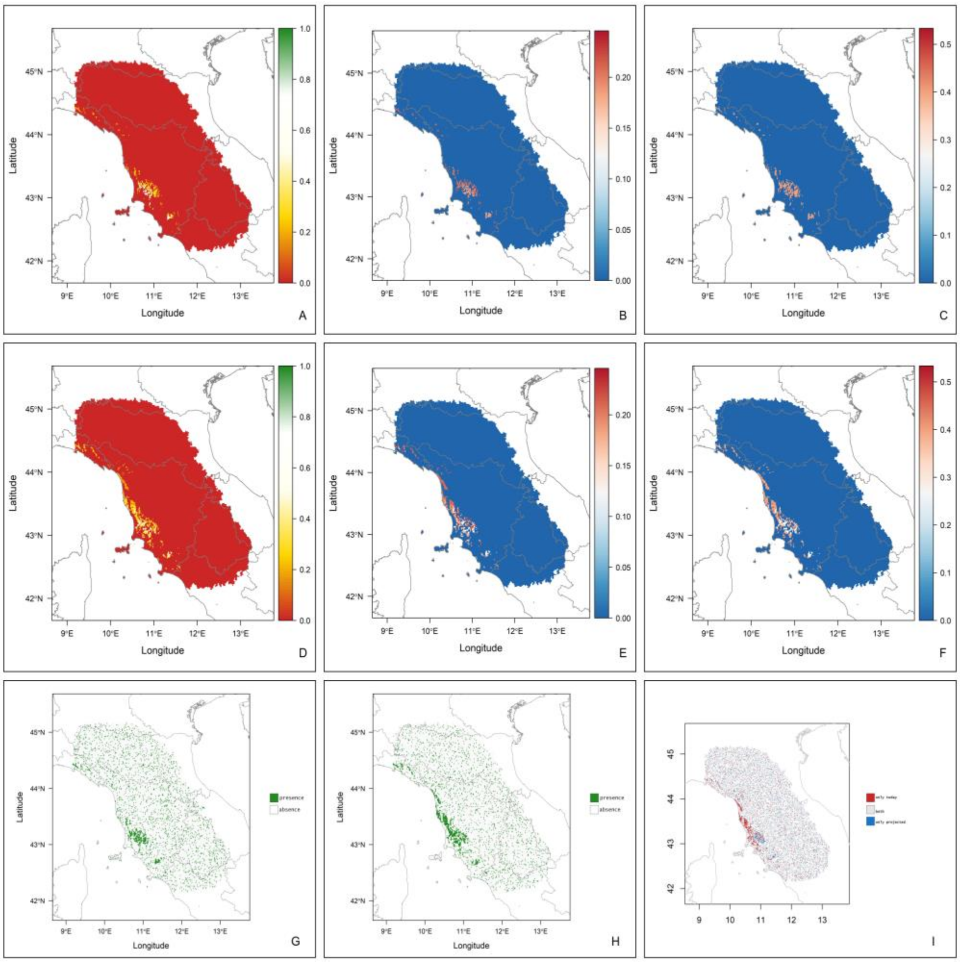

Fig. S.3.74-A) 'Projected' base output; B) 'Projected' uncertainty standard deviation; C) 'Projected' uncertainty range of values; D) 'Present' base output; E) 'Present' uncertainty standard deviation; F) 'Present' uncertainty range of values; G) 'Projected' binary distribution; H) 'Present' binary distribution; I) Range size variation.

Value	Present	Projected
AUC.train	0.567	0.994
AUC.test	0.567	0.621
AUC.diff	0.373	0.373
pROC	1.142	1.248
OR.p10	0.850	0.750
CBI	-0.256	0.199
perc.stable	9.074	9.074
range.size	2.096	2.096

S.3.75 *Crocus etruscus* Domain

Species: *Crocus etruscus* Parl.

Procedure: Domain

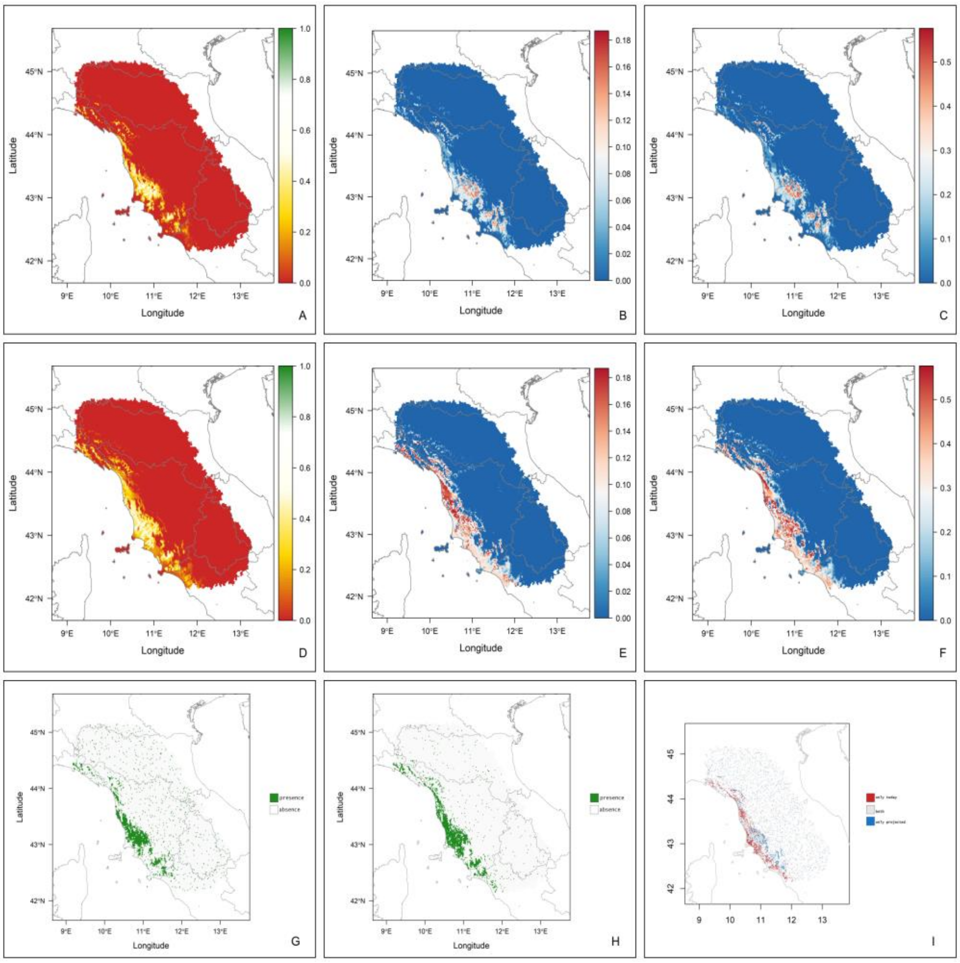

Fig. S.3.75-A) 'Projected' base output; B) 'Projected' uncertainty standard deviation; C) 'Projected' uncertainty range of values; D) 'Present' base output; E) 'Present' uncertainty standard deviation; F) 'Present' uncertainty range of values; G) 'Projected' binary distribution; H) 'Present' binary distribution; I) Range size variation.

Value	Present	Projected
AUC.train	0.788	0.994
AUC.test	0.788	0.961
AUC.diff	0.033	0.033
pROC	1.615	1.923
OR.p10	0.650	0.750
CBI	0.813	0.839
perc.stable	36.687	36.687
range.size	0.405	0.405

S.3.76 *Crocus etruscus* Maxent

Species: *Crocus etruscus* Parl.

Procedure: Maxent

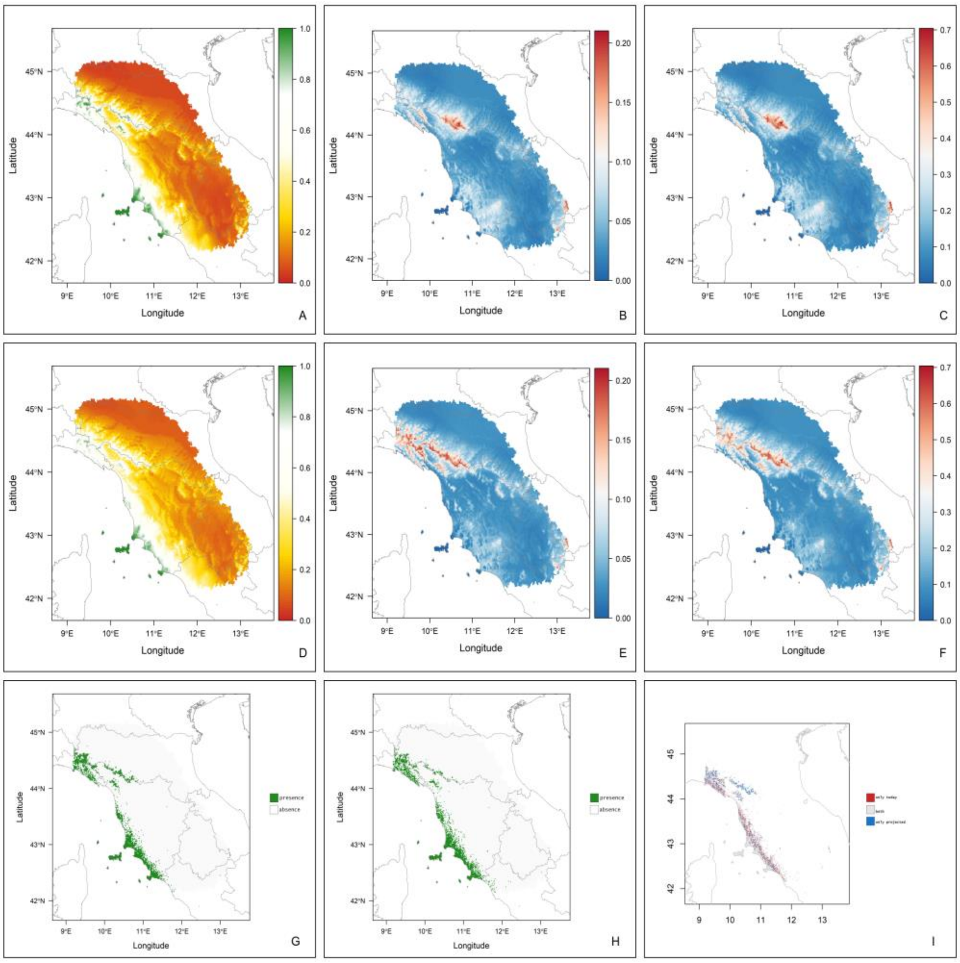

Fig. S.3.76-A) 'Projected' base output; B) 'Projected' uncertainty standard deviation; C) 'Projected' uncertainty range of values; D) 'Present' base output; E) 'Present' uncertainty standard deviation; F) 'Present' uncertainty range of values; G) 'Projected' binary distribution; H) 'Present' binary distribution; I) Range size variation.

Value	Present	Projected
AUC.train	0.909	0.954
AUC.test	0.909	0.949
AUC.diff	0.018	0.018
pROC	1.837	1.906
OR.p10	0.450	0.400
CBI	0.610	0.478
perc.stable	53.352	53.352
range.size	-1.781	-1.781

S.3.77 *Crocus etruscus* KDE

Species: *Crocus etruscus* Parl.

Procedure: KDE

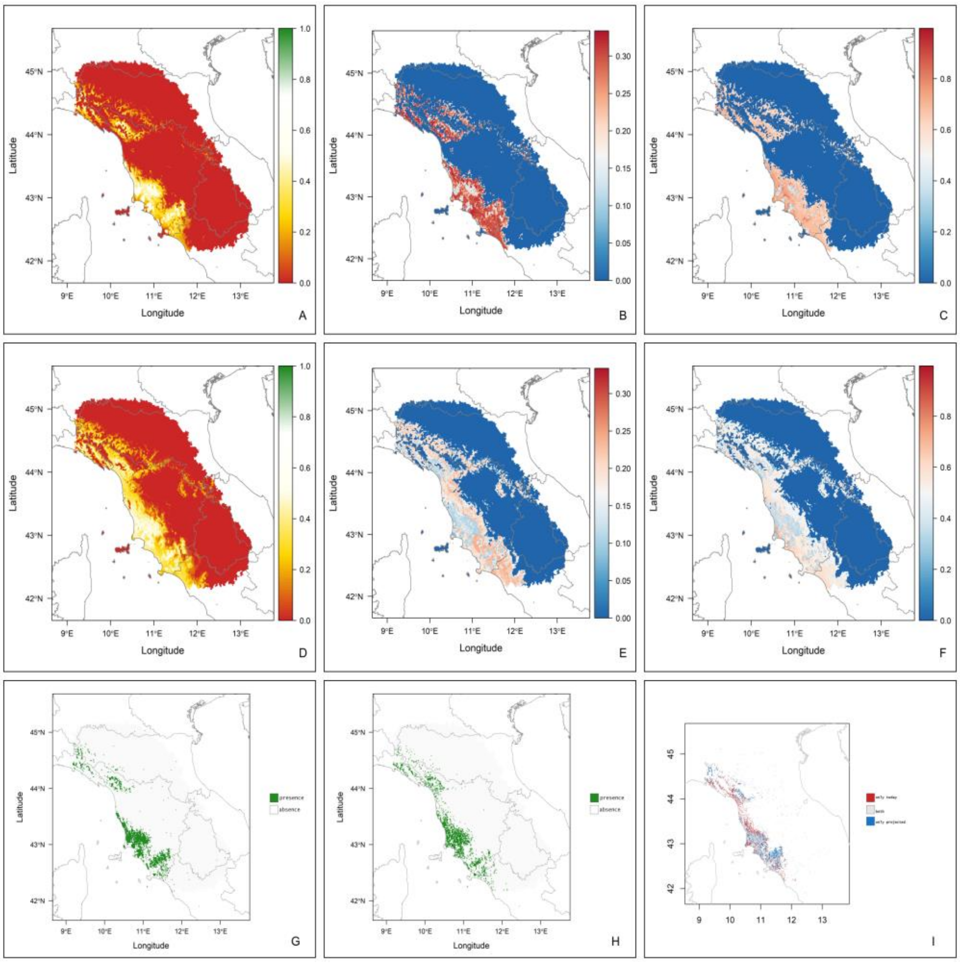

Fig. S.3.77-A) 'Projected' base output; B) 'Projected' uncertainty standard deviation; C) 'Projected' uncertainty range of values; D) 'Present' base output; E) 'Present' uncertainty standard deviation; F) 'Present' uncertainty range of values; G) 'Projected' binary distribution; H) 'Present' binary distribution; I) Range size variation.

Value	Present	Projected
AUC.train	0.960	0.979
AUC.test	0.960	0.956
AUC.diff	0.020	0.035
pROC	1.926	1.916
OR.p10	0.550	0.600
CBI	0.622	0.924
perc.stable	35.057	35.057
range.size	-0.977	-0.977

S.3.78 *Crocus etruscus* CTA

Species: *Crocus etruscus* Parl.

Procedure: CTA

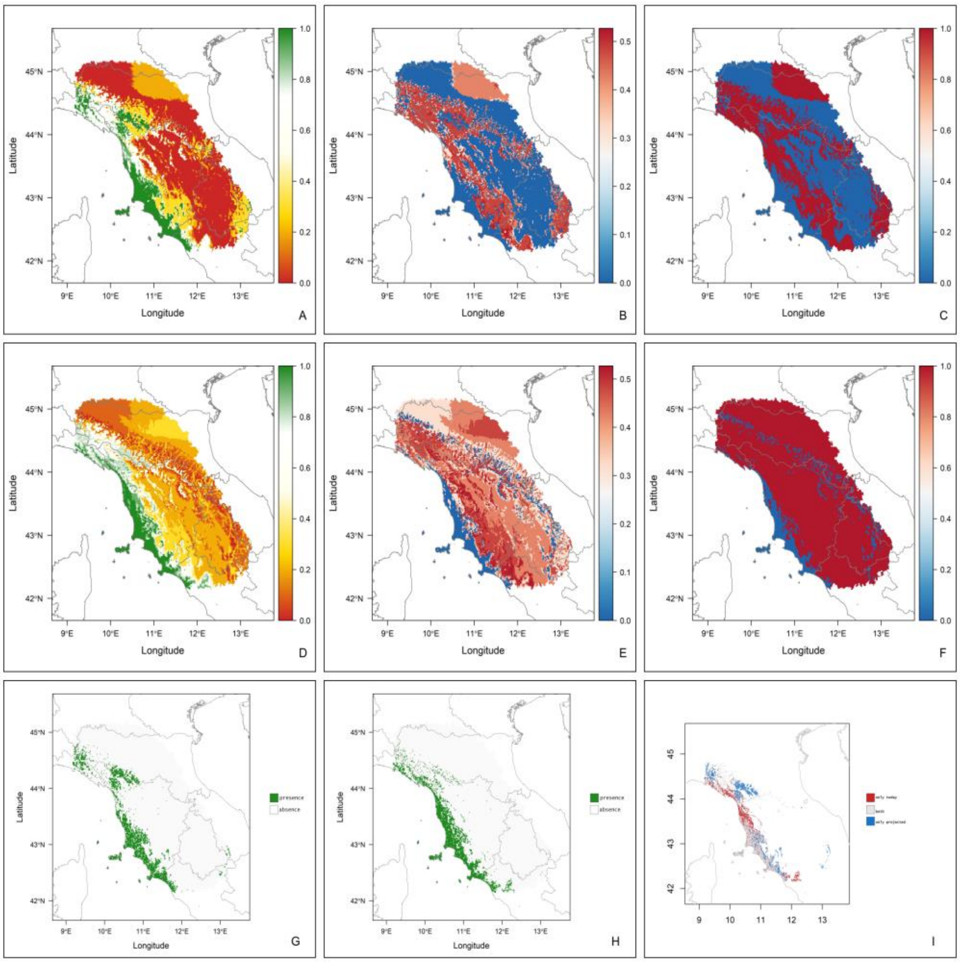

Fig. S.3.78-A) 'Projected' base output; B) 'Projected' uncertainty standard deviation; C) 'Projected' uncertainty range of values; D) 'Present' base output; E) 'Present' uncertainty standard deviation; F) 'Present' uncertainty range of values; G) 'Projected' binary distribution; H) 'Present' binary distribution; I) Range size variation.

Value	Present	Projected
AUC.train	0.889	0.964
AUC.test	0.889	0.964
AUC.diff	0.000	0.000
pROC	1.000	1.000
OR.p10	0.050	0.000
CBI	0.733	0.455
perc.stable	44.582	44.582
range.size	-0.750	-0.750

S.3.79 *Crocus etruscus* FDA

Species: *Crocus etruscus* Parl.

Procedure: FDA

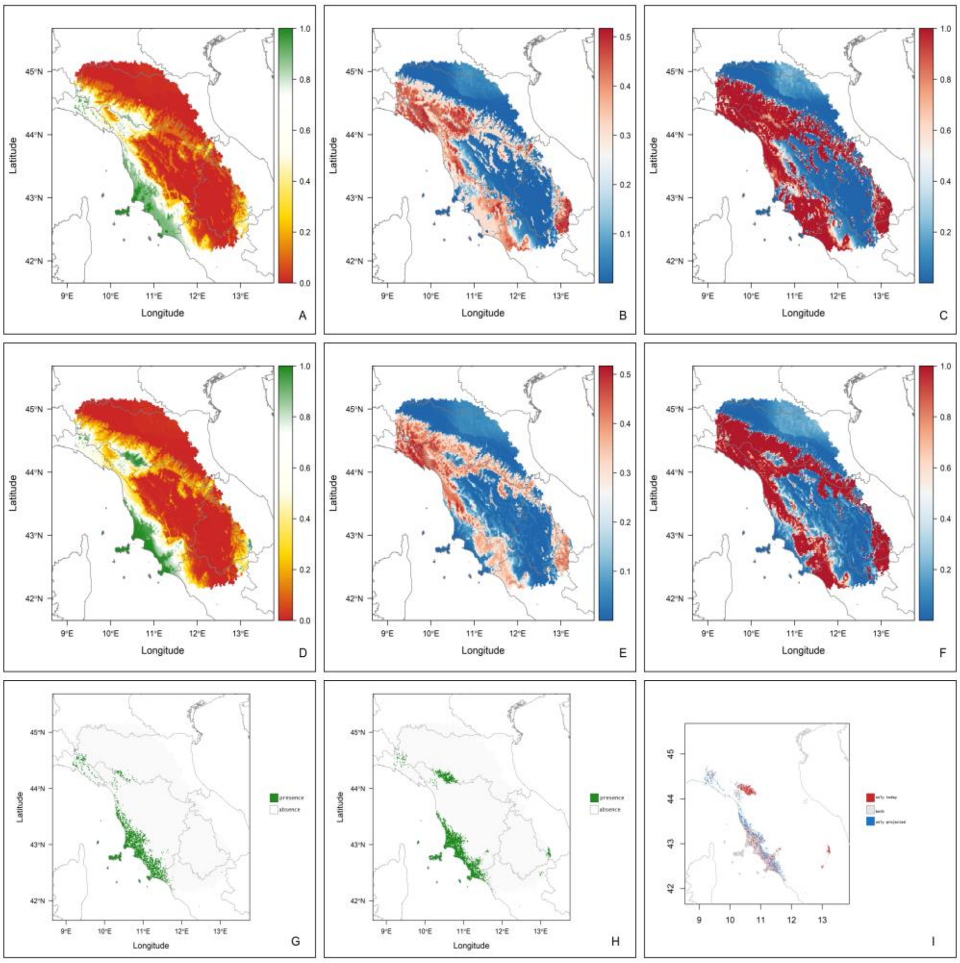

Fig. S.3.79-A) 'Projected' base output; B) 'Projected' uncertainty standard deviation; C) 'Projected' uncertainty range of values; D) 'Present' base output; E) 'Present' uncertainty standard deviation; F) 'Present' uncertainty range of values; G) 'Projected' binary distribution; H) 'Present' binary distribution; I) Range size variation.

Value	Present	Projected
AUC.train	0.936	0.989
AUC.test	0.936	0.986
AUC.diff	0.011	0.011
pROC	1.550	1.432
OR.p10	0.450	0.550
CBI	0.821	0.781
perc.stable	46.177	46.177
range.size	-1.526	-1.526

S.3.80 *Crocus etruscus* GAM

Species: *Crocus etruscus* Parl.

Procedure: GAM

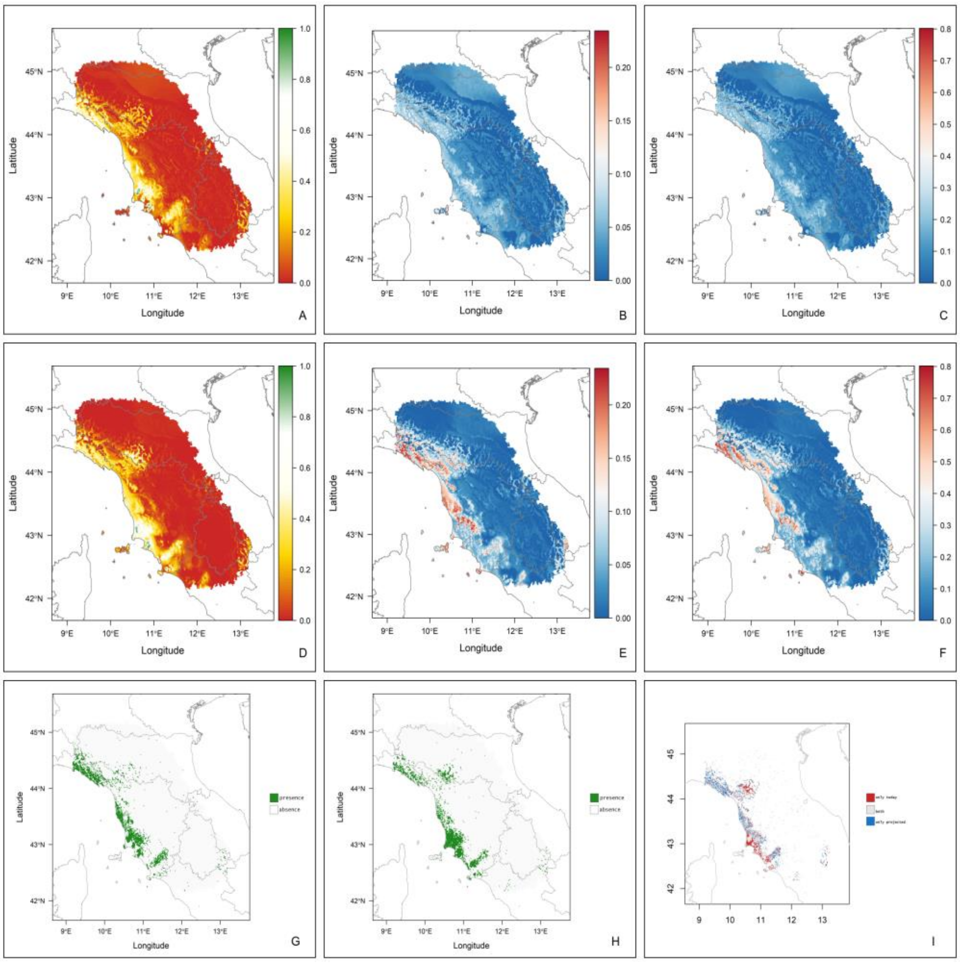

Fig. S.3.80-A) 'Projected' base output; B) 'Projected' uncertainty standard deviation; C) 'Projected' uncertainty range of values; D) 'Present' base output; E) 'Present' uncertainty standard deviation; F) 'Present' uncertainty range of values; G) 'Projected' binary distribution; H) 'Present' binary distribution; I) Range size variation.

Value	Present	Projected
AUC.train	0.951	0.989
AUC.test	0.951	0.968
AUC.diff	0.022	0.022
pROC	1.905	1.937
OR.p10	0.500	0.450
CBI	0.981	0.851
perc.stable	37.857	37.857
range.size	2.918	2.918

S.3.81 *Crocus etruscus* GLM

Species: *Crocus etruscus* Parl.

Procedure: GLM

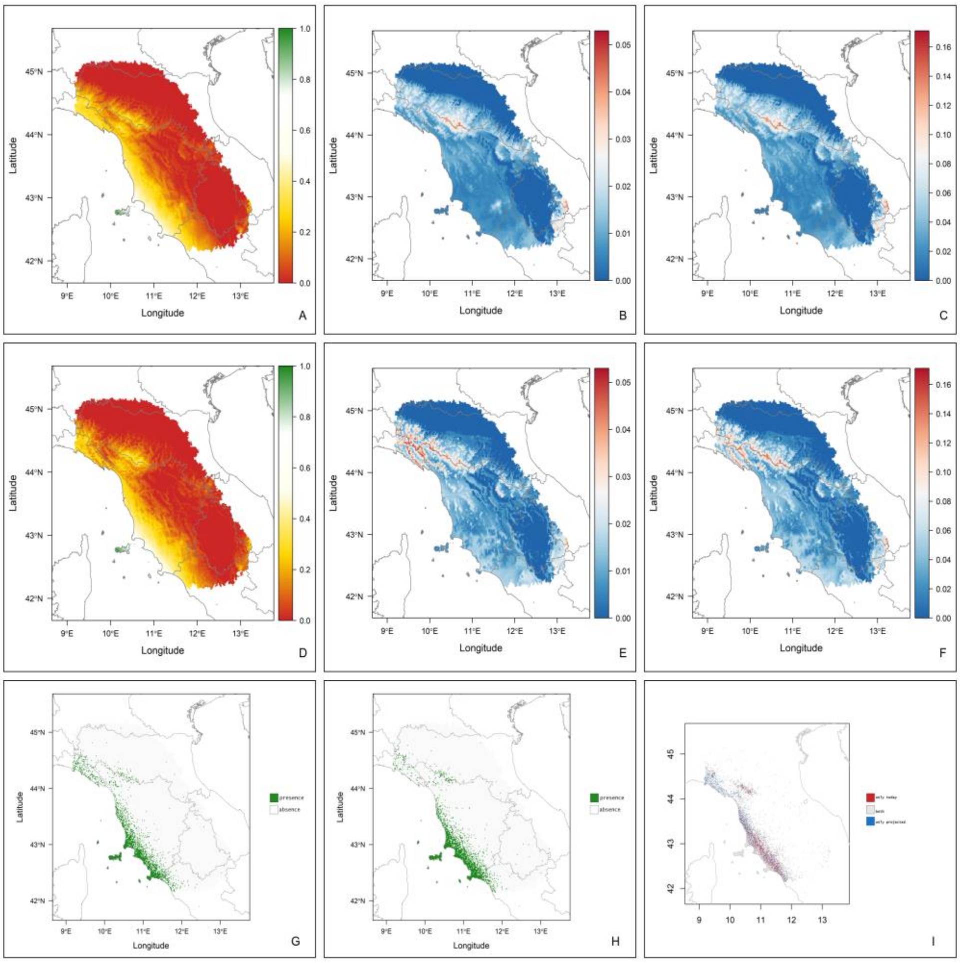

Fig. S.3.81-A) 'Projected' base output; B) 'Projected' uncertainty standard deviation; C) 'Projected' uncertainty range of values; D) 'Present' base output; E) 'Present' uncertainty standard deviation; F) 'Present' uncertainty range of values; G) 'Projected' binary distribution; H) 'Present' binary distribution; I) Range size variation.

Value	Present	Projected
AUC.train	0.931	0.959
AUC.test	0.931	0.946
AUC.diff	0.022	0.022
pROC	1.876	1.904
OR.p10	0.300	0.450
CBI	0.486	0.619
perc.stable	38.430	38.430
range.size	0.385	0.385

S.3.82 *Crocus etruscus* MARS

Species: *Crocus etruscus* Parl.

Procedure: MARS

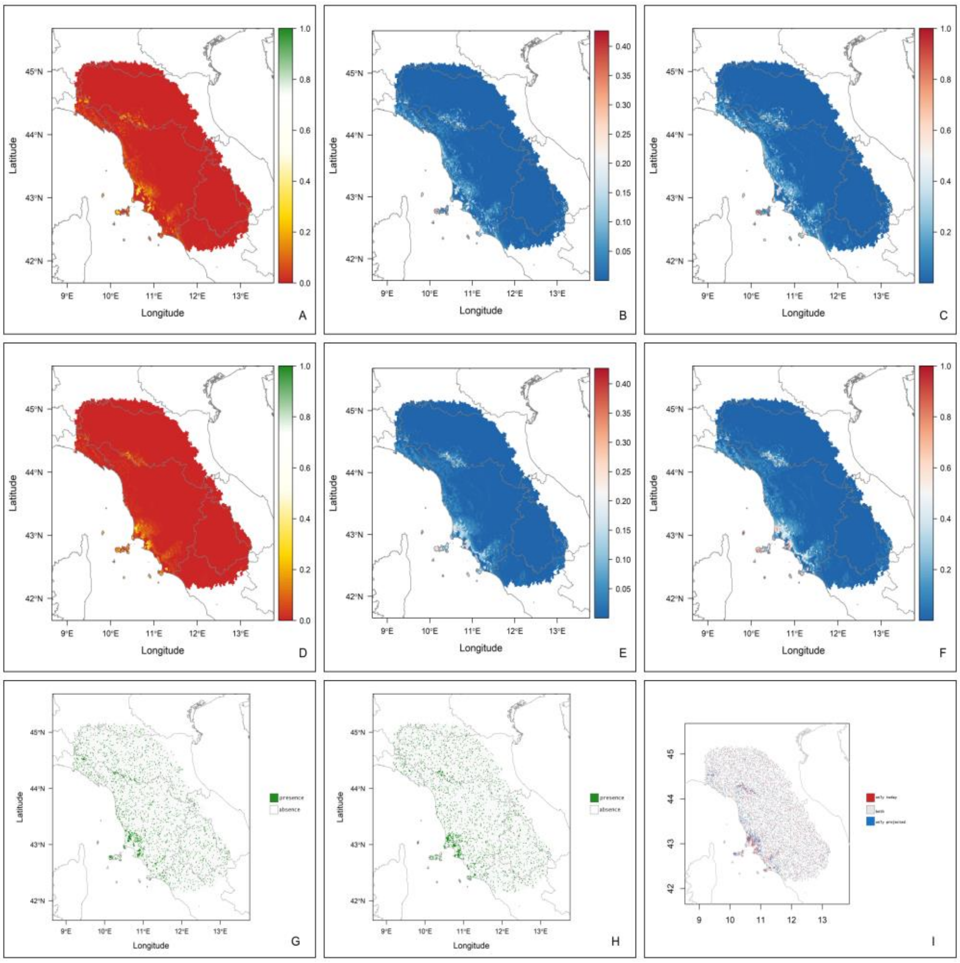

Fig. S.3.82-A) 'Projected' base output; B) 'Projected' uncertainty standard deviation; C) 'Projected' uncertainty range of values; D) 'Present' base output; E) 'Present' uncertainty standard deviation; F) 'Present' uncertainty range of values; G) 'Projected' binary distribution; H) 'Present' binary distribution; I) Range size variation.

Value	Present	Projected
AUC.train	0.711	0.983
AUC.test	0.711	0.905
AUC.diff	0.089	0.089
pROC	1.596	1.818
OR.p10	0.750	0.500
CBI	0.657	0.885
perc.stable	6.262	6.262
range.size	-2.477	-2.477

S.3.83 *Crocus etruscus* RF

Species: *Crocus etruscus* Parl.

Procedure: RF

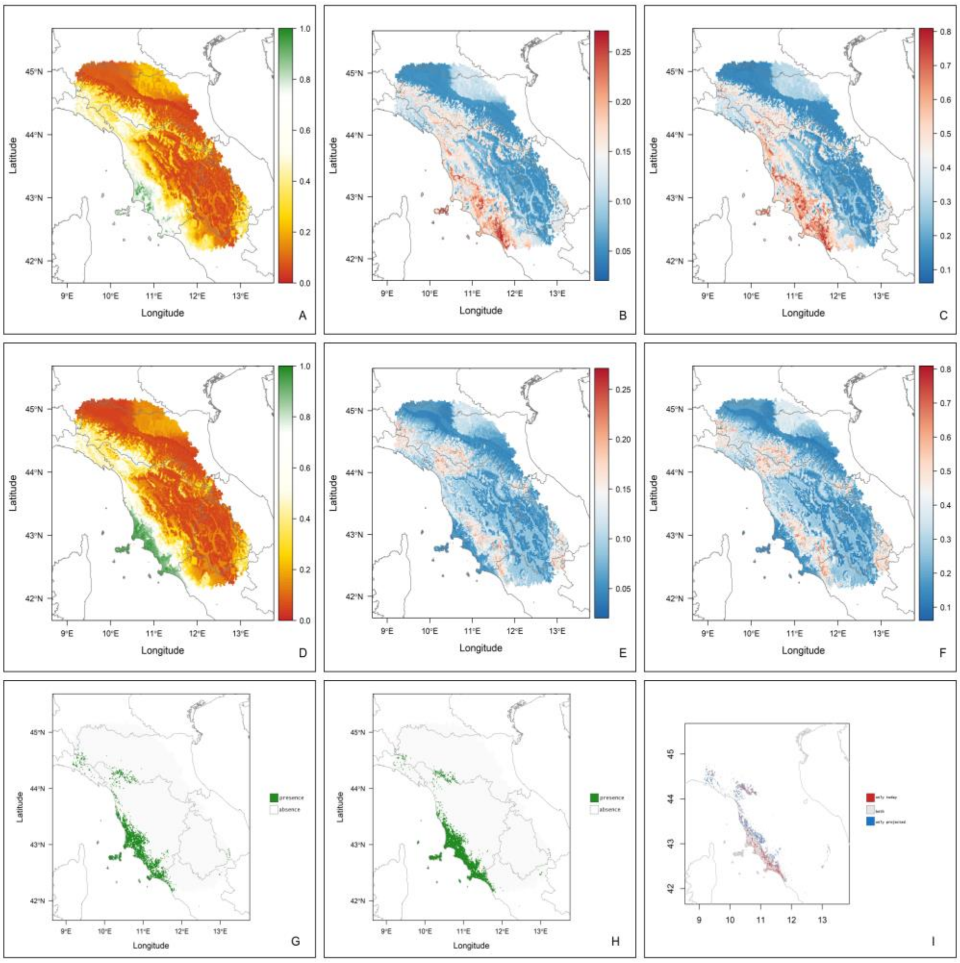

Fig. S.3.83-A) 'Projected' base output; B) 'Projected' uncertainty standard deviation; C) 'Projected' uncertainty range of values; D) 'Present' base output; E) 'Present' uncertainty standard deviation; F) 'Present' uncertainty range of values; G) 'Projected' binary distribution; H) 'Present' binary distribution; I) Range size variation.

Value	Present	Projected
AUC.train	1.000	1.000
AUC.test	1.000	0.971
AUC.diff	0.029	0.029
pROC	1.794	1.820
OR.p10	0.700	0.650
CBI	0.895	0.969
perc.stable	56.558	56.558
range.size	-1.682	-1.682

S.3.84 *Crocus etruscus* SVM

Species: *Crocus etruscus* Parl.

Procedure: SVM

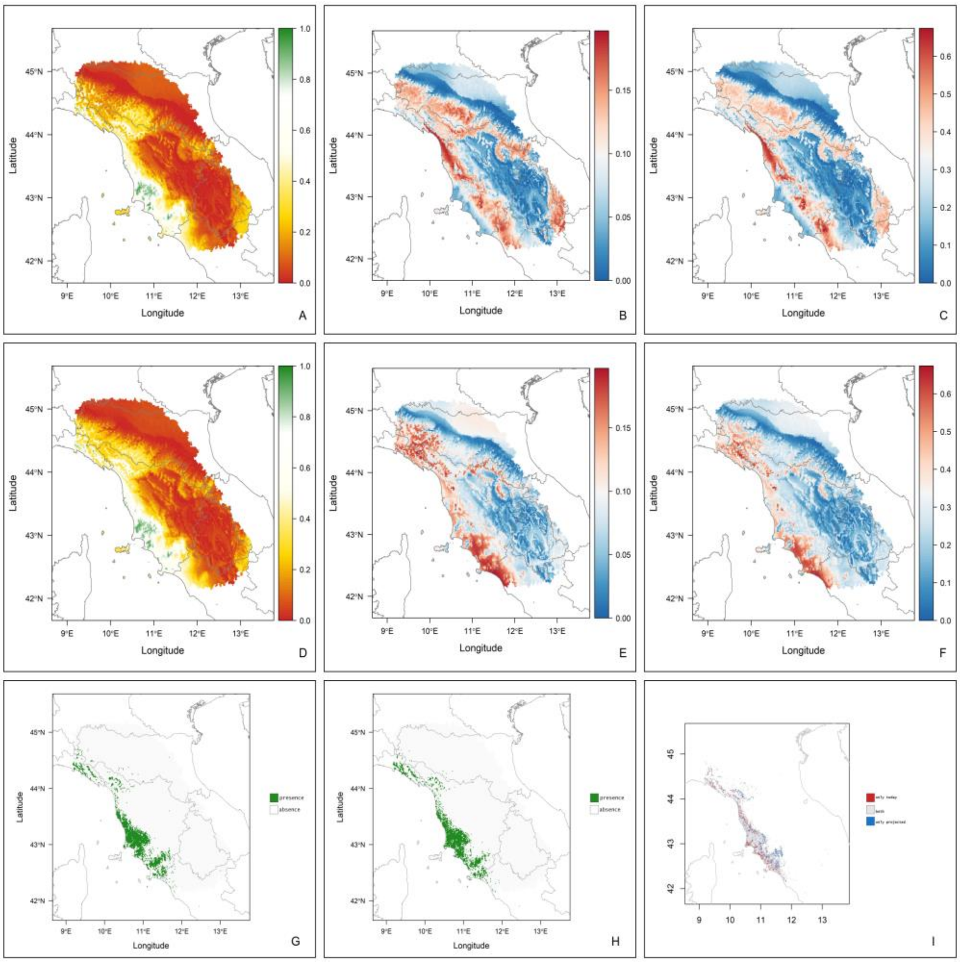

Fig. S.3.84-A) 'Projected' base output; B) 'Projected' uncertainty standard deviation; C) 'Projected' uncertainty range of values; D) 'Present' base output; E) 'Present' uncertainty standard deviation; F) 'Present' uncertainty range of values; G) 'Projected' binary distribution; H) 'Present' binary distribution; I) Range size variation.

Value	Present	Projected
AUC.train	0.986	1.000
AUC.test	0.986	1.000
AUC.diff	0.000	0.000
pROC	1.921	1.976
OR.p10	0.750	0.300
CBI	0.951	0.961
perc.stable	56.712	56.712
range.size	-0.406	-0.406

S.3.85 *Crocus imperati* Biomod2

Species: *Crocus imperati* Ten.

Procedure: Biomod2

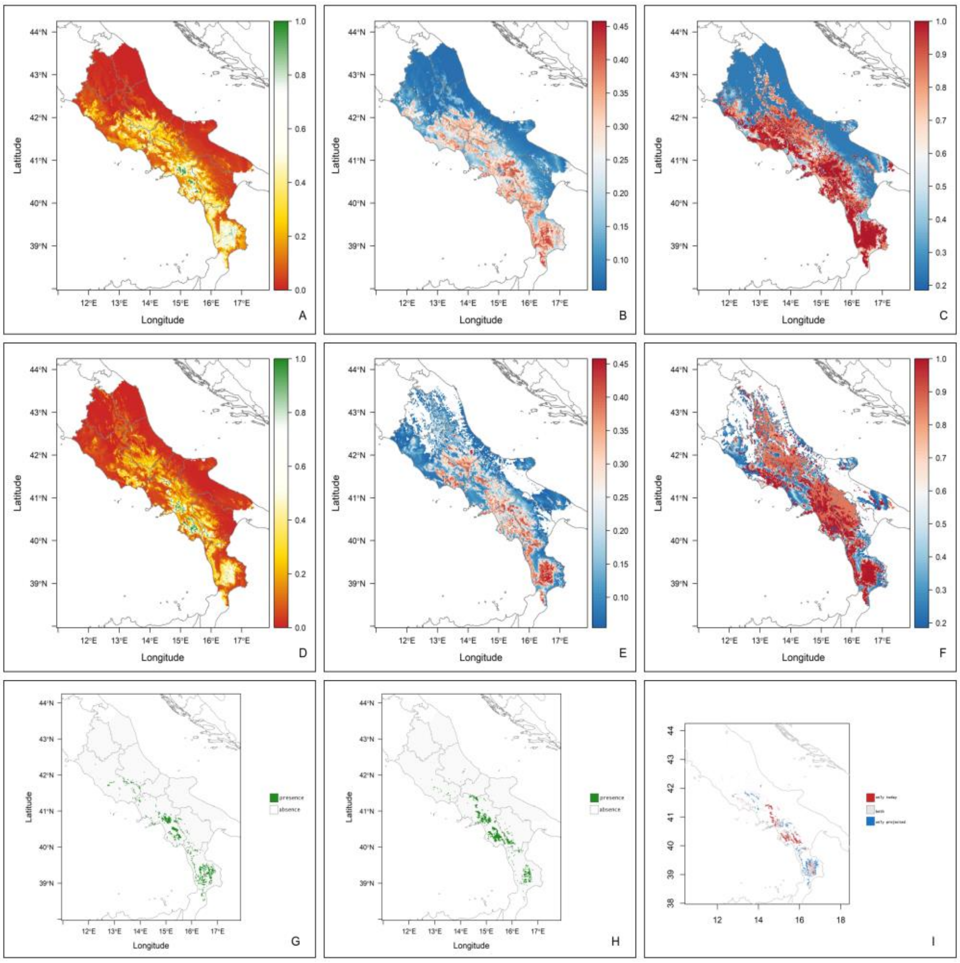

Fig. S.3.85-A) 'Projected' base output; B) 'Projected' uncertainty standard deviation; C) 'Projected' uncertainty range of values; D) 'Present' base output; E) 'Present' uncertainty standard deviation; F) 'Present' uncertainty range of values; G) 'Projected' binary distribution; H) 'Present' binary distribution; I) Range size variation.

Value	Present	Projected
AUC.train	0.915	0.995
AUC.test	0.915	0.875
AUC.diff	0.121	0.121
pROC	1.744	1.657
OR.p10	0.813	0.610
CBI	0.907	0.760
perc.stable	25.230	25.230
range.size	-1.681	-1.681

S.3.86 *Crocus imperati* Bioclim

Species: *Crocus imperati* Ten.

Procedure: Bioclim

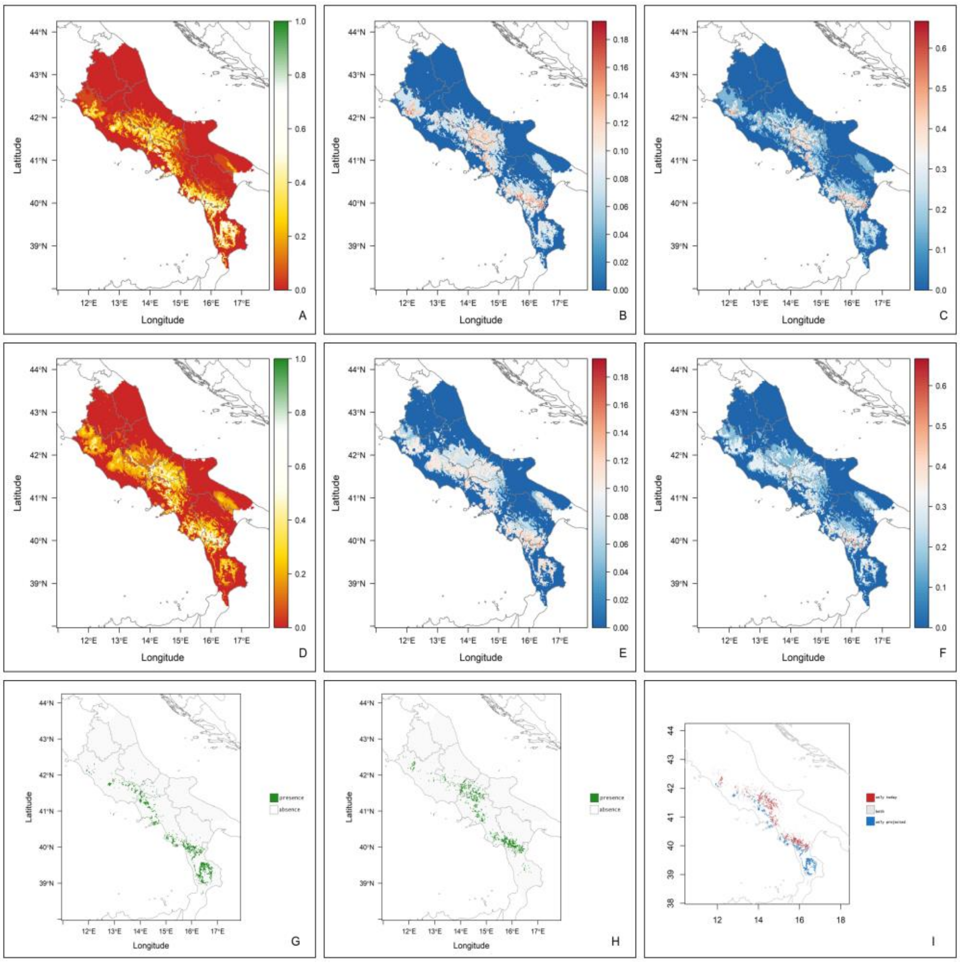

Fig. S.3.86-A) 'Projected' base output; B) 'Projected' uncertainty standard deviation; C) 'Projected' uncertainty range of values; D) 'Present' base output; E) 'Present' uncertainty standard deviation; F) 'Present' uncertainty range of values; G) 'Projected' binary distribution; H) 'Present' binary distribution; I) Range size variation.

Value	Present	Projected
AUC.train	0.797	0.848
AUC.test	0.797	0.698
AUC.diff	0.151	0.151
pROC	1.391	1.180
OR.p10	0.213	0.387
CBI	0.733	0.553
perc.stable	12.617	12.617
range.size	4.505	4.505

S.3.87 *Crocus imperati* Domain

Species: *Crocus imperati* Ten.

Procedure: Domain

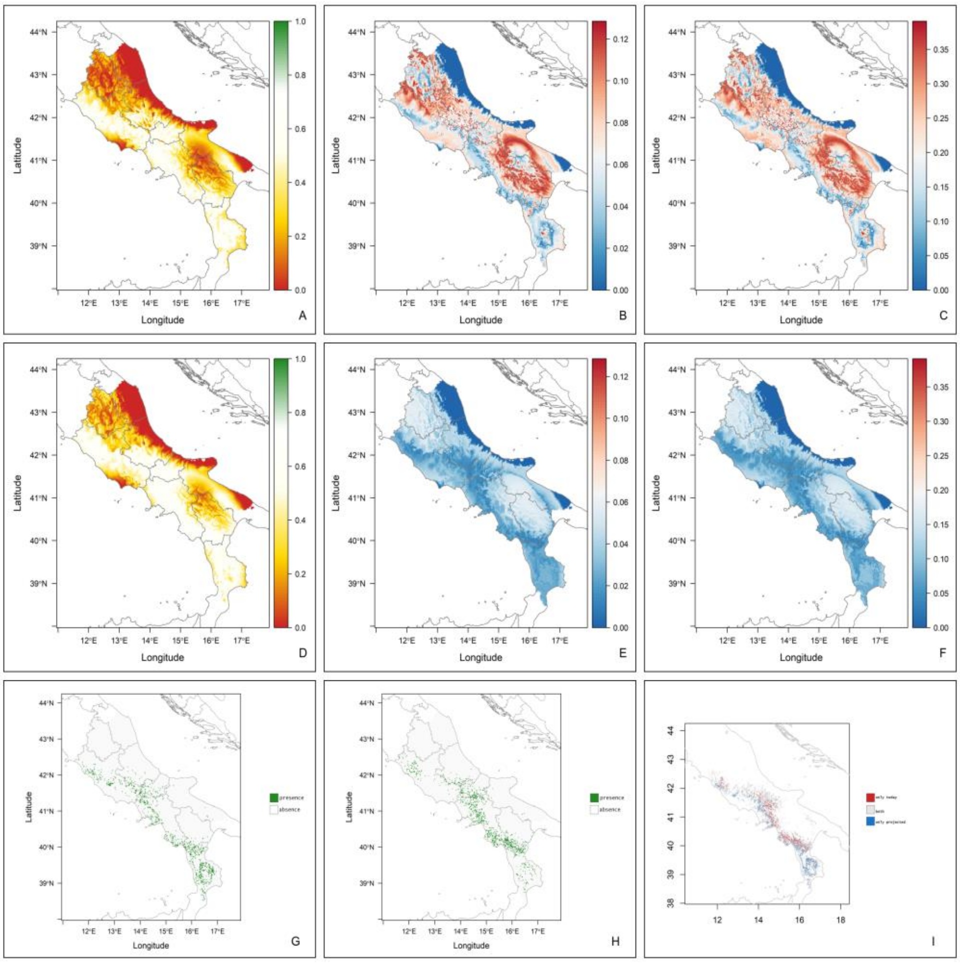

Fig. S.3.87-A) 'Projected' base output; B) 'Projected' uncertainty standard deviation; C) 'Projected' uncertainty range of values; D) 'Present' base output; E) 'Present' uncertainty standard deviation; F) 'Present' uncertainty range of values; G) 'Projected' binary distribution; H) 'Present' binary distribution; I) Range size variation.

Value	Present	Projected
AUC.train	0.885	0.820
AUC.test	0.885	0.749
AUC.diff	0.084	0.084
pROC	1.657	1.455
OR.p10	0.053	0.270
CBI	0.480	0.890
perc.stable	8.131	8.131
range.size	-0.939	-0.939

S.3.88 *Crocus imperati* Maxent

Species: *Crocus imperati* Ten.

Procedure: Maxent

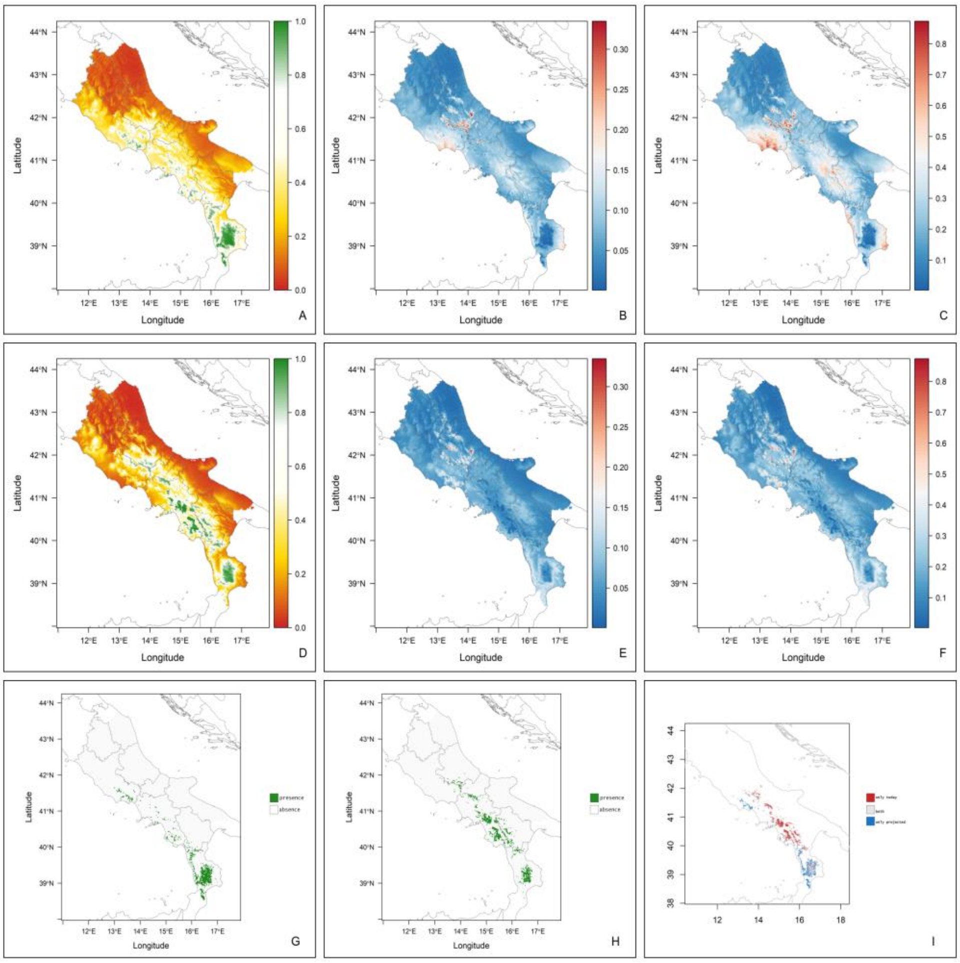

Fig. S.3.88-A) 'Projected' base output; B) 'Projected' uncertainty standard deviation; C) 'Projected' uncertainty range of values; D) 'Present' base output; E) 'Present' uncertainty standard deviation; F) 'Present' uncertainty range of values; G) 'Projected' binary distribution; H) 'Present' binary distribution; I) Range size variation.

Value	Present	Projected
AUC.train	0.873	0.873
AUC.test	0.873	0.834
AUC.diff	0.052	0.052
pROC	1.647	1.616
OR.p10	0.177	0.317
CBI	0.920	0.740
perc.stable	21.733	21.733
range.size	-5.235	-5.235

S.3.89 *Crocus imperati* KDE

Species: *Crocus imperati* Ten.

Procedure: KDE

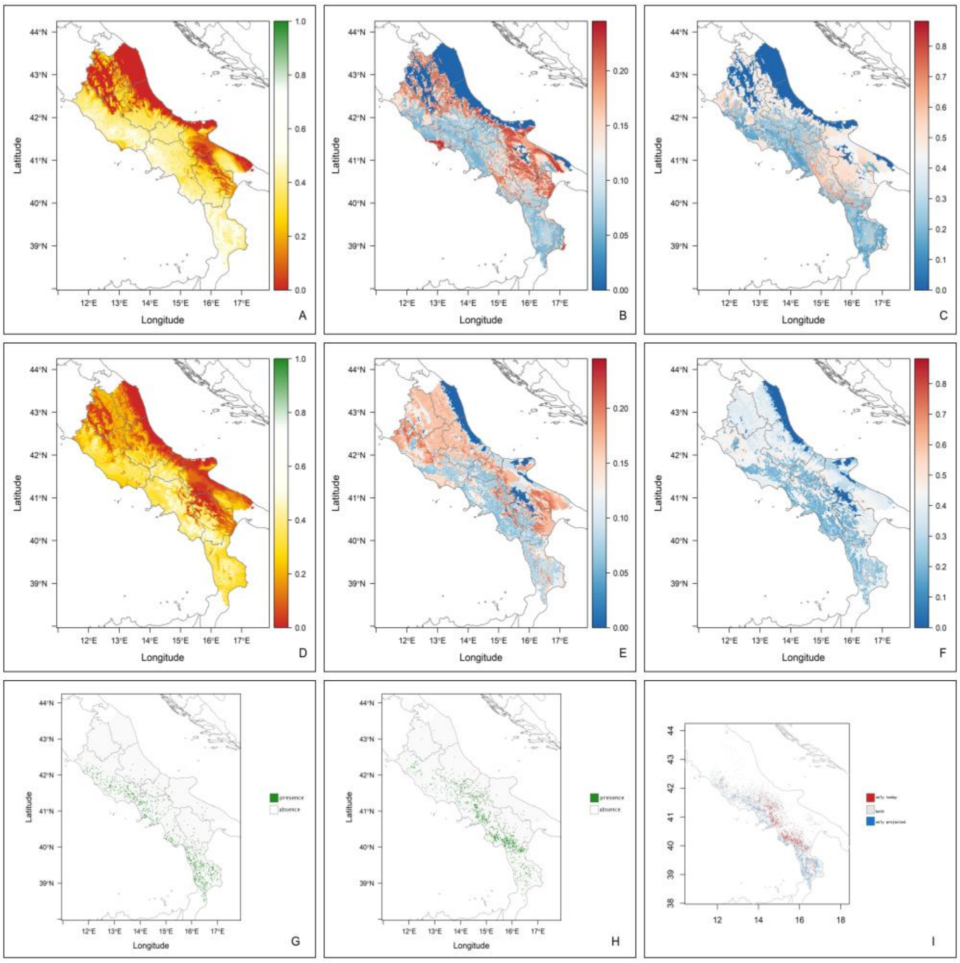

Fig. S.3.89-A) 'Projected' base output; B) 'Projected' uncertainty standard deviation; C) 'Projected' uncertainty range of values; D) 'Present' base output; E) 'Present' uncertainty standard deviation; F) 'Present' uncertainty range of values; G) 'Projected' binary distribution; H) 'Present' binary distribution; I) Range size variation.

Value	Present	Projected
AUC.train	0.826	0.809
AUC.test	0.826	0.811
AUC.diff	0.073	0.037
pROC	1.544	1.542
OR.p10	0.250	0.167
CBI	0.889	0.710
perc.stable	3.933	3.933
range.size	-1.609	-1.609

S.3.90 *Crocus imperati* CTA

Species: *Crocus imperati* Ten.

Procedure: CTA

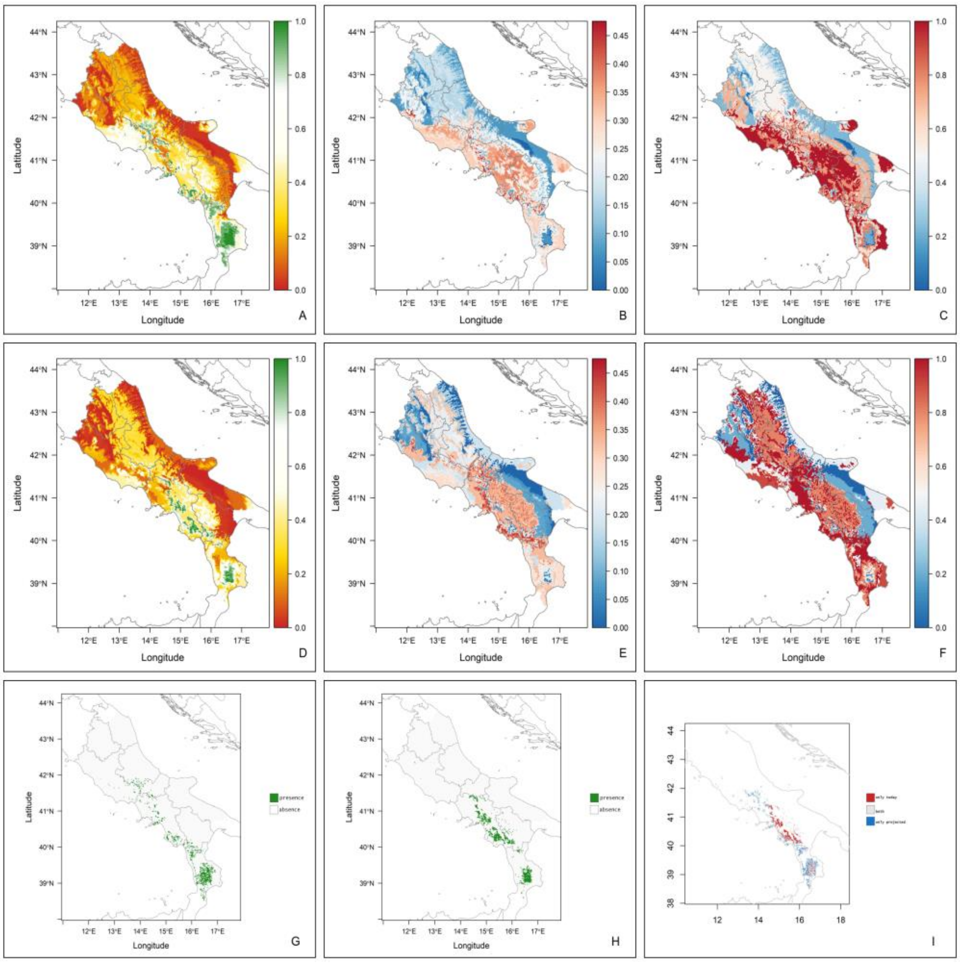

Fig. S.3.90-A) 'Projected' base output; B) 'Projected' uncertainty standard deviation; C) 'Projected' uncertainty range of values; D) 'Present' base output; E) 'Present' uncertainty standard deviation; F) 'Present' uncertainty range of values; G) 'Projected' binary distribution; H) 'Present' binary distribution; I) Range size variation.

Value	Present	Projected
AUC.train	0.852	0.912
AUC.test	0.852	0.830
AUC.diff	0.089	0.089
pROC	1.236	1.218
OR.p10	0.197	0.233
CBI	0.935	0.938
perc.stable	23.298	23.298
range.size	-2.800	-2.800

S.3.91 *Crocus imperati* FDA

Species: *Crocus imperati* Ten.

Procedure: FDA

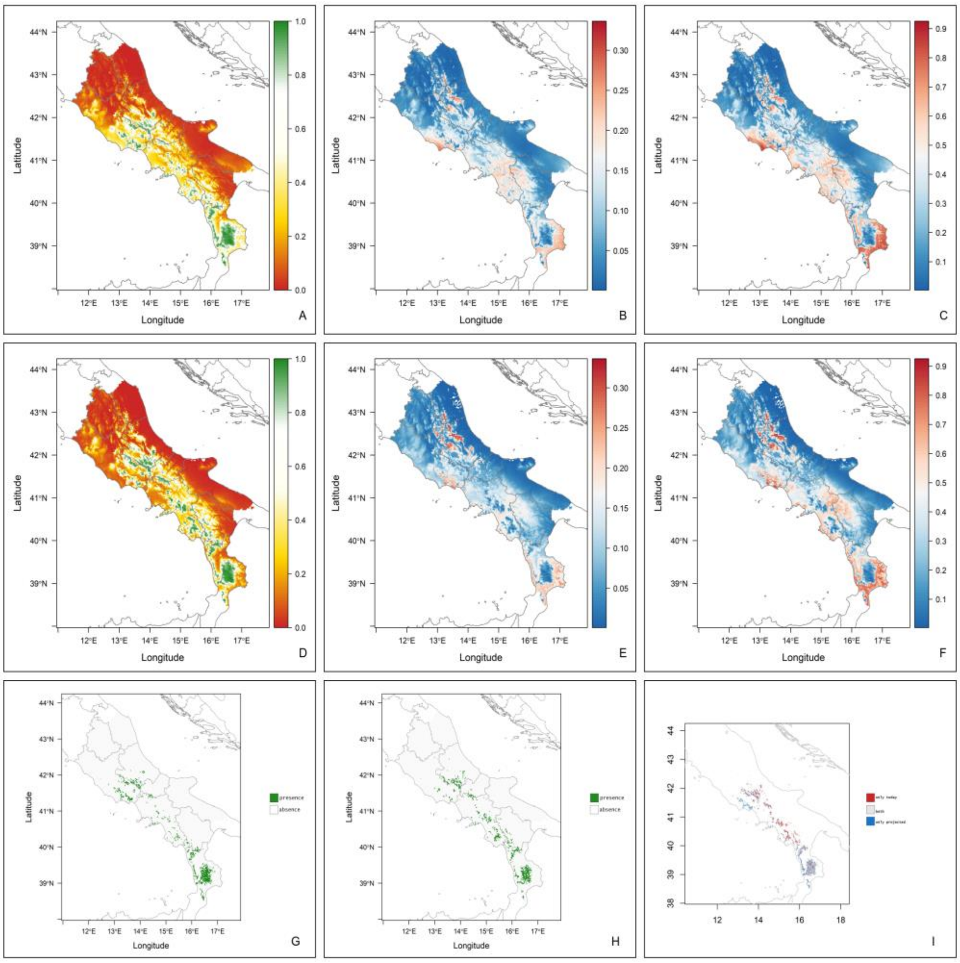

Fig. S.3.91-A) 'Projected' base output; B) 'Projected' uncertainty standard deviation; C) 'Projected' uncertainty range of values; D) 'Present' base output; E) 'Present' uncertainty standard deviation; F) 'Present' uncertainty range of values; G) 'Projected' binary distribution; H) 'Present' binary distribution; I) Range size variation.

Value	Present	Projected
AUC.train	0.884	0.874
AUC.test	0.884	0.882
AUC.diff	0.018	0.018
pROC	1.612	1.665
OR.p10	0.240	0.200
CBI	0.833	0.811
perc.stable	33.228	33.228
range.size	1.611	1.611

S.3.92 *Crocus imperati* GAM

Species: *Crocus imperati* Ten.

Procedure: GAM

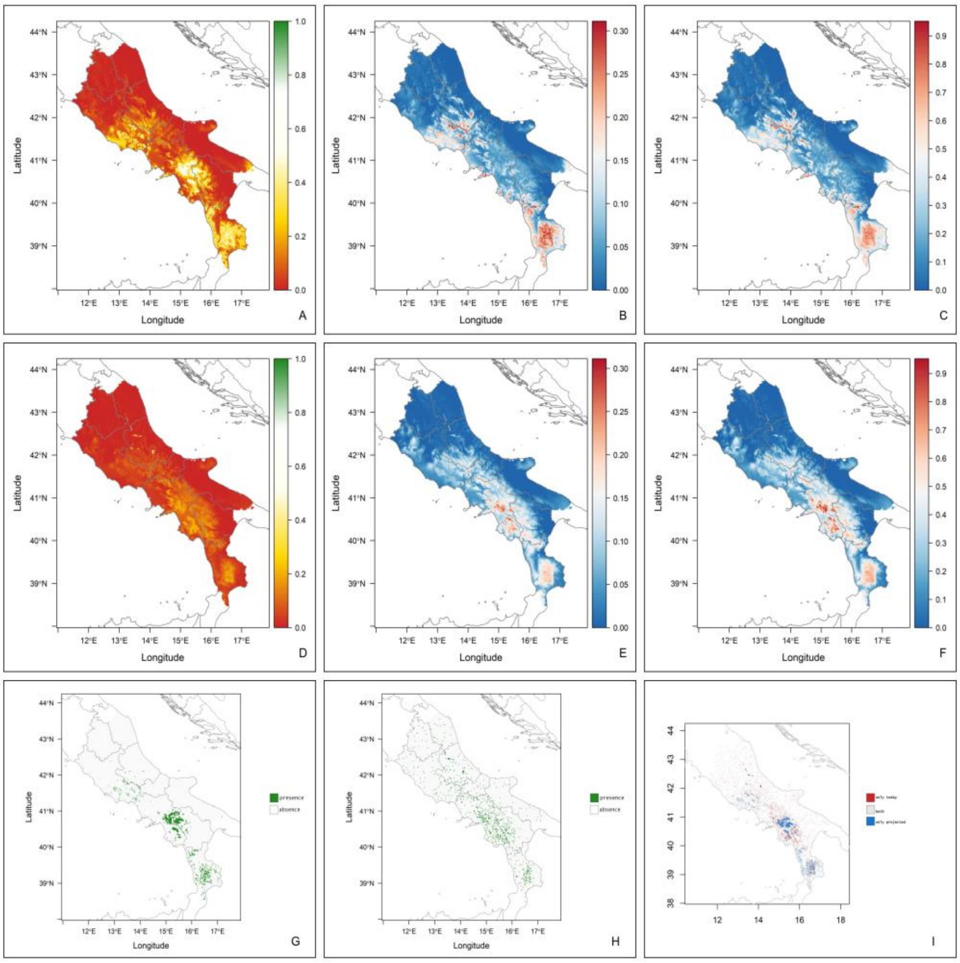

Fig. S.3.92-A) 'Projected' base output; B) 'Projected' uncertainty standard deviation; C) 'Projected' uncertainty range of values; D) 'Present' base output; E) 'Present' uncertainty standard deviation; F) 'Present' uncertainty range of values; G) 'Projected' binary distribution; H) 'Present' binary distribution; I) Range size variation.

Value	Present	Projected
AUC.train	0.796	0.907
AUC.test	0.796	0.827
AUC.diff	0.083	0.083
pROC	1.477	1.565
OR.p10	0.307	0.363
CBI	0.863	0.773
perc.stable	7.500	7.500
range.size	-3.793	-3.793

S.3.93 *Crocus imperati* GLM

Species: *Crocus imperati* Ten.

Procedure: GLM

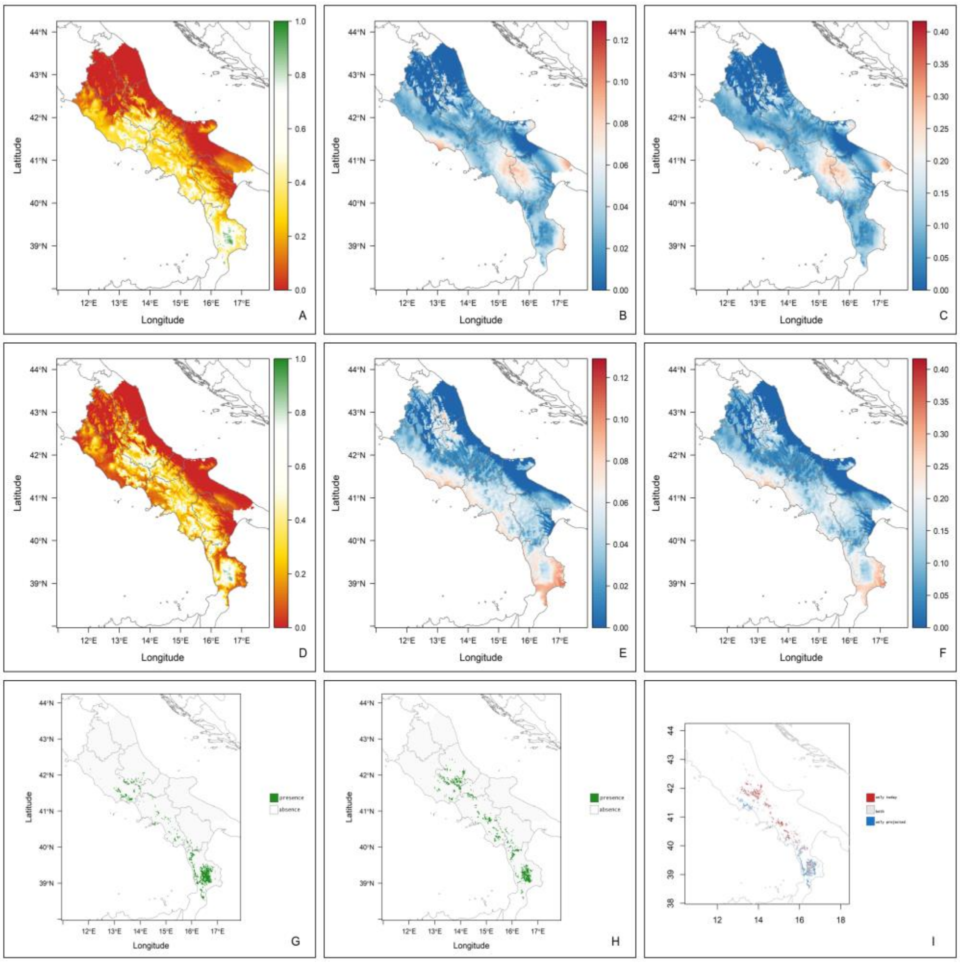

Fig. S.3.93-A) 'Projected' base output; B) 'Projected' uncertainty standard deviation; C) 'Projected' uncertainty range of values; D) 'Present' base output; E) 'Present' uncertainty standard deviation; F) 'Present' uncertainty range of values; G) 'Projected' binary distribution; H) 'Present' binary distribution; I) Range size variation.

Value	Present	Projected
AUC.train	0.844	0.858
AUC.test	0.844	0.879
AUC.diff	0.015	0.015
pROC	1.614	1.698
OR.p10	0.293	0.103
CBI	0.839	0.569
perc.stable	33.619	33.619
range.size	-3.056	-3.056

S.3.94 *Crocus imperati* MARS

Species: *Crocus imperati* Ten.

Procedure: MARS

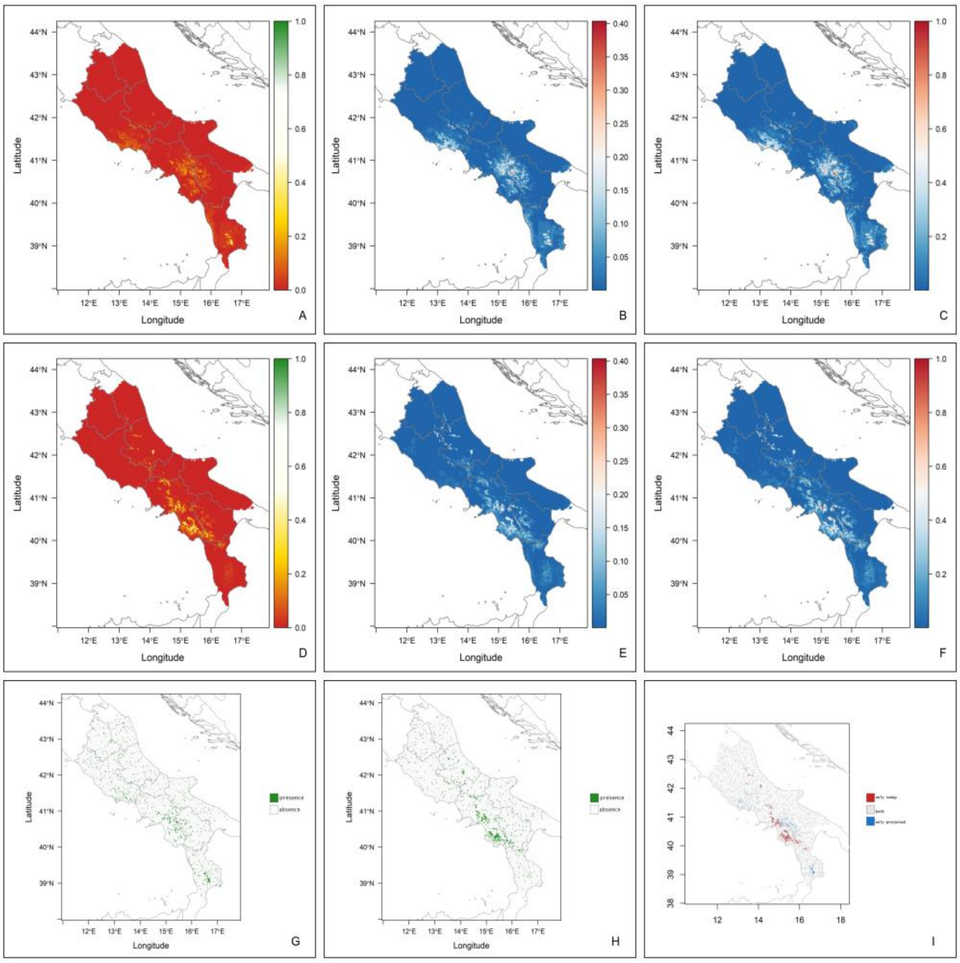

Fig. S.3.94-A) 'Projected' base output; B) 'Projected' uncertainty standard deviation; C) 'Projected' uncertainty range of values; D) 'Present' base output; E) 'Present' uncertainty standard deviation; F) 'Present' uncertainty range of values; G) 'Projected' binary distribution; H) 'Present' binary distribution; I) Range size variation.

Value	Present	Projected
AUC.train	0.697	0.873
AUC.test	0.697	0.708
AUC.diff	0.174	0.174
pROC	1.268	1.264
OR.p10	0.457	0.293
CBI	0.829	0.464
perc.stable	2.859	2.859
range.size	-4.689	-4.689

S.3.95 *Crocus imperati* RF

Species: *Crocus imperati* Ten.

Procedure: RF

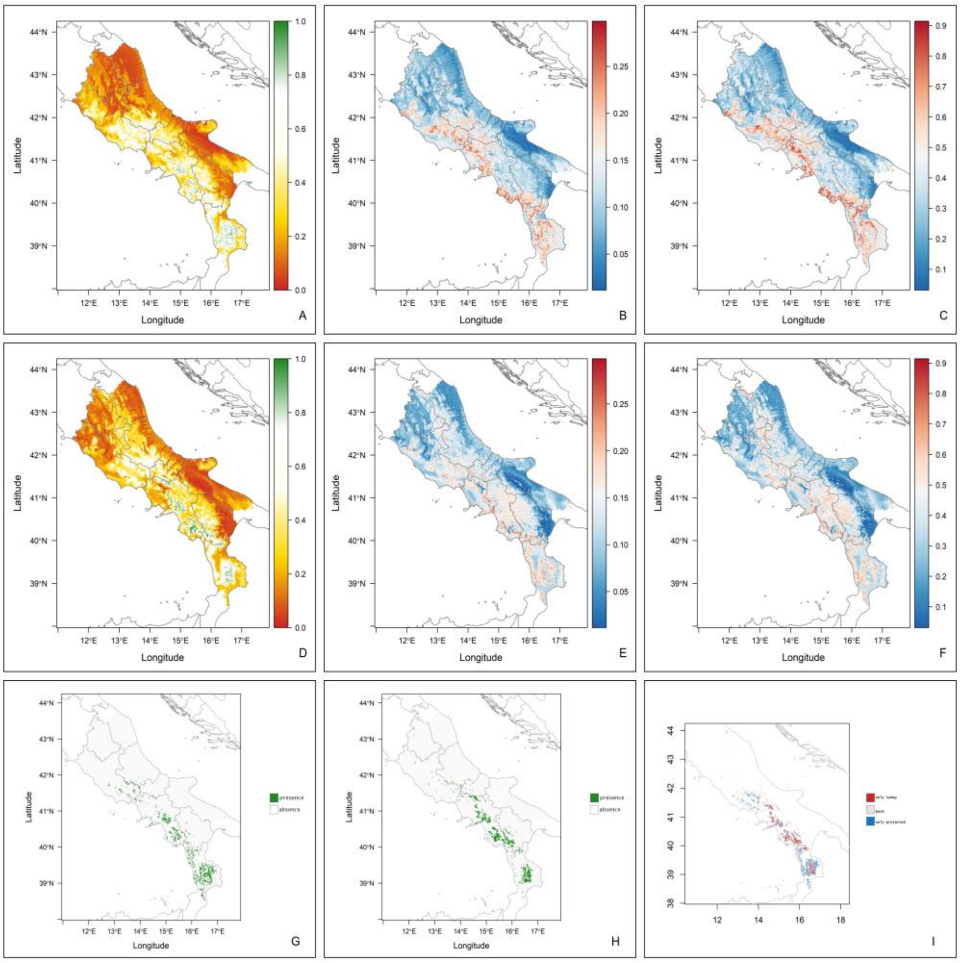

Fig. S.3.95-A) 'Projected' base output; B) 'Projected' uncertainty standard deviation; C) 'Projected' uncertainty range of values; D) 'Present' base output; E) 'Present' uncertainty standard deviation; F) 'Present' uncertainty range of values; G) 'Projected' binary distribution; H) 'Present' binary distribution; I) Range size variation.

Value	Present	Projected
AUC.train	0.967	1.000
AUC.test	0.967	0.939
AUC.diff	0.061	0.061
pROC	1.624	1.567
OR.p10	0.547	0.803
CBI	0.938	0.902
perc.stable	20.594	20.594
range.size	0.240	0.240

S.3.96 *Crocus imperati* SVM

Species: *Crocus imperati* Ten.

Procedure: SVM

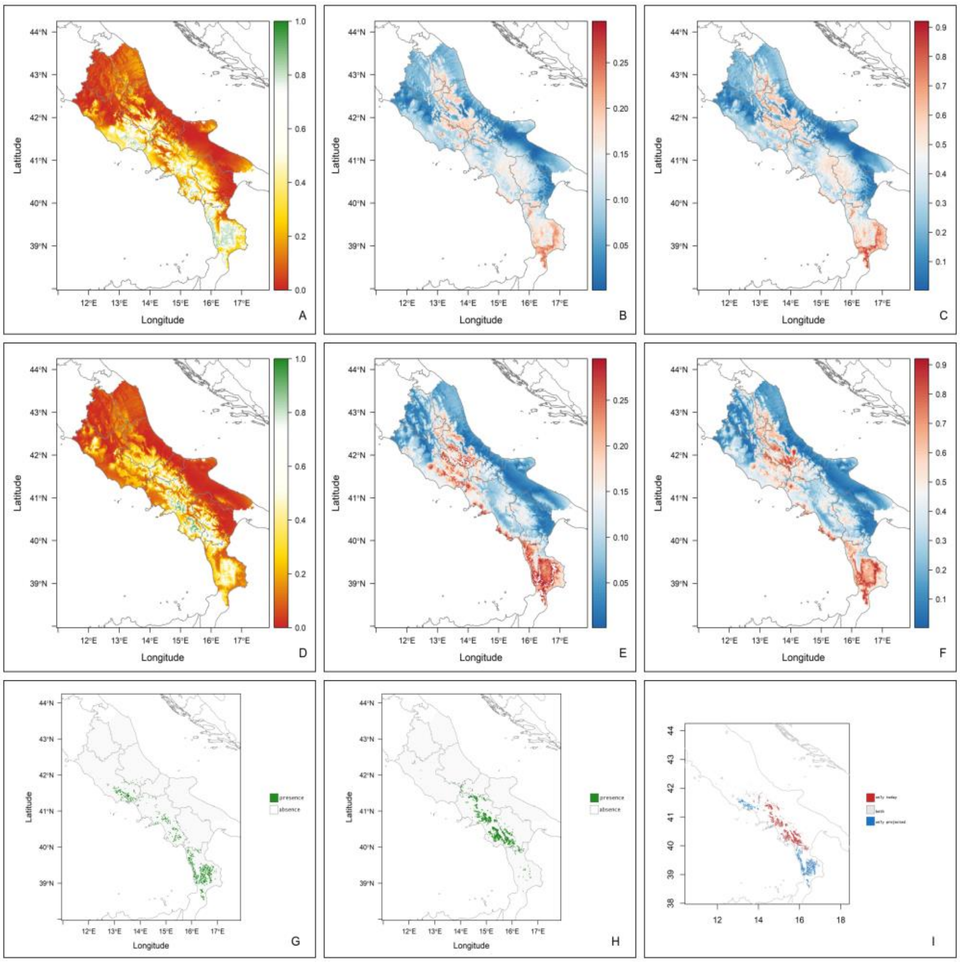

Fig. S.3.96-A) 'Projected' base output; B) 'Projected' uncertainty standard deviation; C) 'Projected' uncertainty range of values; D) 'Present' base output; E) 'Present' uncertainty standard deviation; F) 'Present' uncertainty range of values; G) 'Projected' binary distribution; H) 'Present' binary distribution; I) Range size variation.

Value	Present	Projected
AUC.train	0.866	0.919
AUC.test	0.866	0.880
AUC.diff	0.047	0.047
pROC	1.542	1.605
OR.p10	0.303	0.377
CBI	0.892	0.893
perc.stable	10.516	10.516
range.size	-2.619	-2.619

S.3.97 *Erysimum pseudorhaeticum* Biomod2

Species: *Erysimum pseudorhaeticum* Polatschek

Procedure: Biomod2

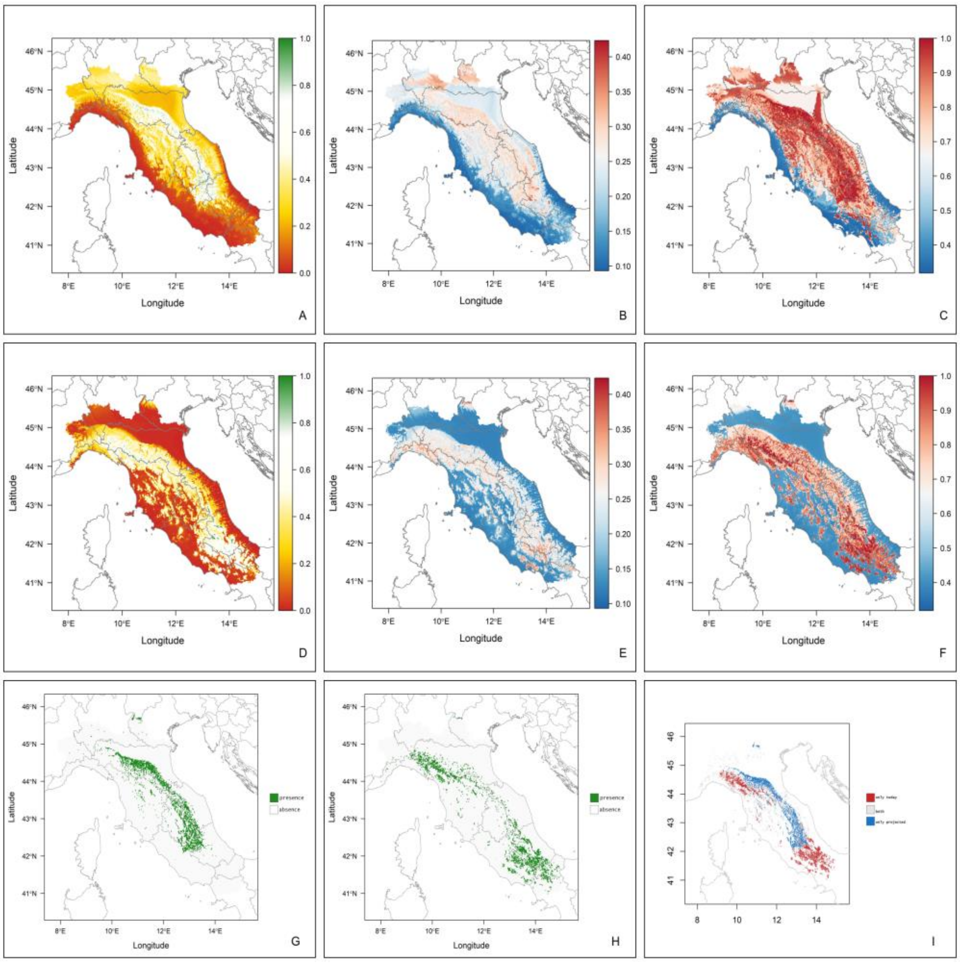

Fig. S.3.97-A) 'Projected' base output; B) 'Projected' uncertainty standard deviation; C) 'Projected' uncertainty range of values; D) 'Present' base output; E) 'Present' uncertainty standard deviation; F) 'Present' uncertainty range of values; G) 'Projected' binary distribution; H) 'Present' binary distribution; I) Range size variation.

Value	Present	Projected
AUC.train	0.853	0.977
AUC.test	0.853	0.805
AUC.diff	0.172	0.172
pROC	1.507	1.345
OR.p10	0.807	0.636
CBI	0.957	0.685
perc.stable	7.561	7.561
range.size	-0.606	-0.606

S.3.98 *Erysimum pseudorhaeticum* Bioclim

Species: *Erysimum pseudorhaeticum* Polatschek

Procedure: Bioclim

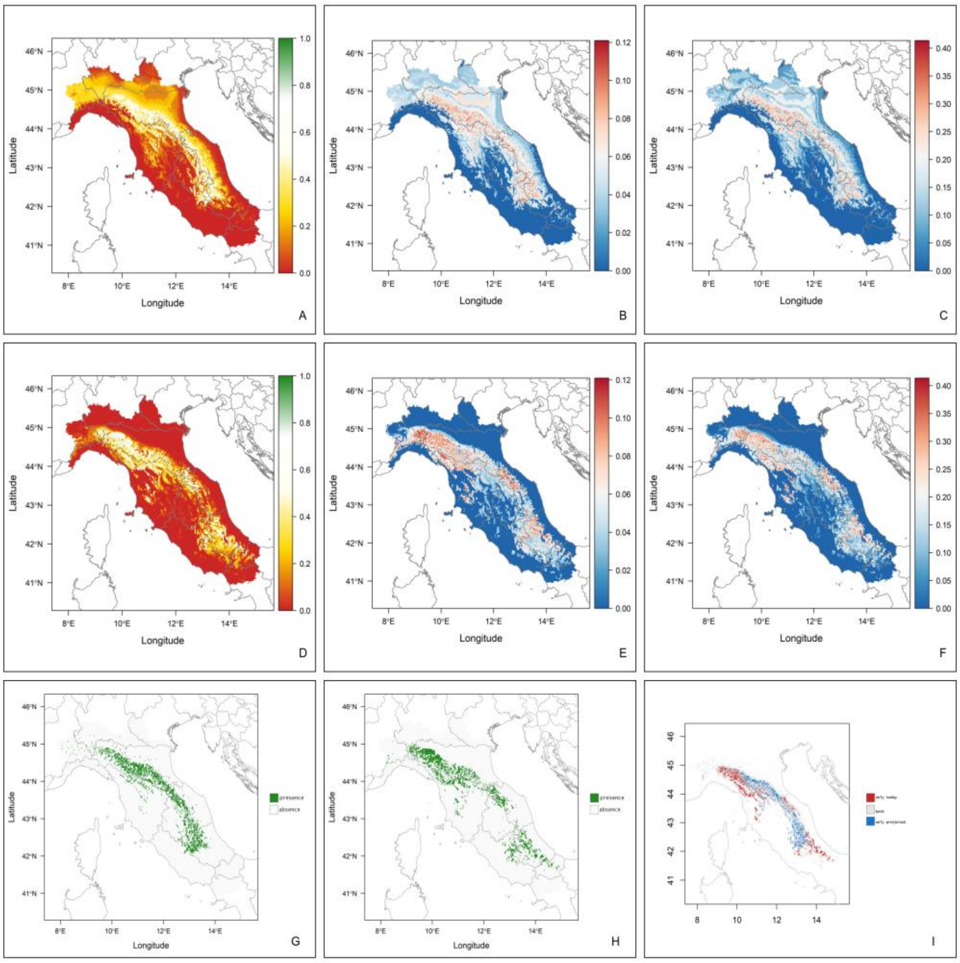

Fig. S.3.98-A) 'Projected' base output; B) 'Projected' uncertainty standard deviation; C) 'Projected' uncertainty range of values; D) 'Present' base output; E) 'Present' uncertainty standard deviation; F) 'Present' uncertainty range of values; G) 'Projected' binary distribution; H) 'Present' binary distribution; I) Range size variation.

Value	Present	Projected
AUC.train	0.731	0.779
AUC.test	0.731	0.723
AUC.diff	0.067	0.067
pROC	1.166	1.178
OR.p10	0.219	0.143
CBI	0.023	0.892
perc.stable	26.451	26.451
range.size	-0.204	-0.204

S.3.99 *Erysimum pseudorhaeticum* Domain

Species: *Erysimum pseudorhaeticum* Polatschek

Procedure: Domain

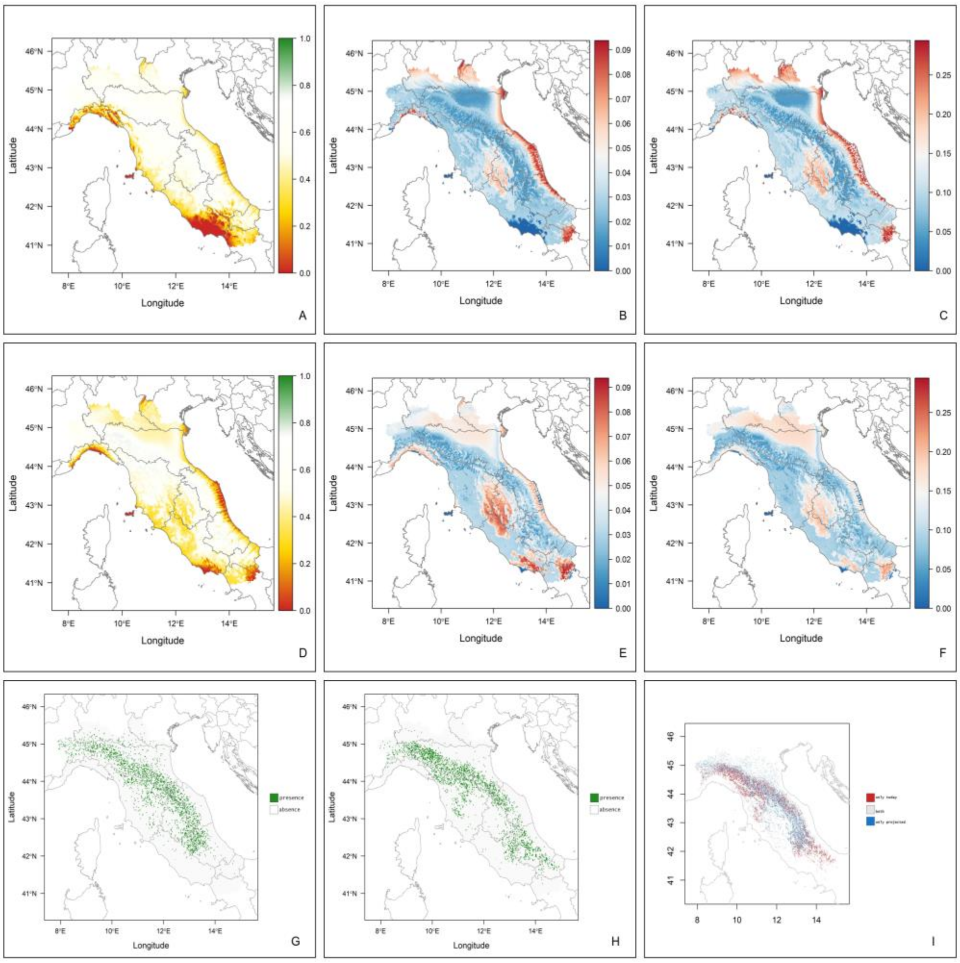

Fig. S.3.99-A) 'Projected' base output; B) 'Projected' uncertainty standard deviation; C) 'Projected' uncertainty range of values; D) 'Present' base output; E) 'Present' uncertainty standard deviation; F) 'Present' uncertainty range of values; G) 'Projected' binary distribution; H) 'Present' binary distribution; I) Range size variation.

Value	Present	Projected
AUC.train	0.676	0.706
AUC.test	0.676	0.692
AUC.diff	0.039	0.039
pROC	1.172	1.283
OR.p10	0.187	0.164
CBI	0.975	-0.512
perc.stable	13.595	13.595
range.size	0.443	0.443

S.3.100 *Erysimum pseudorhaeticum* Maxent

Species: *Erysimum pseudorhaeticum* Polatschek

Procedure: Maxent

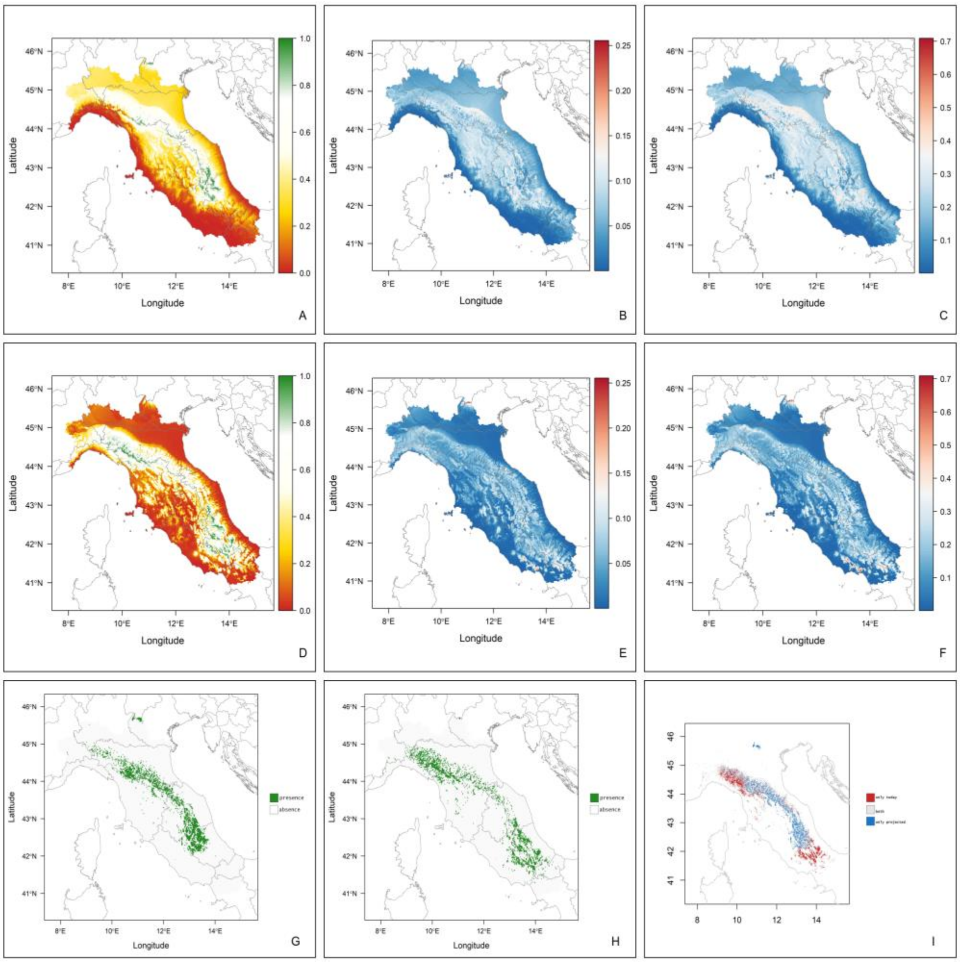

Fig. S.3.100-A) 'Projected' base output; B) 'Projected' uncertainty standard deviation; C) 'Projected' uncertainty range of values; D) 'Present' base output; E) 'Present' uncertainty standard deviation; F) 'Present' uncertainty range of values; G) 'Projected' binary distribution; H) 'Present' binary distribution; I) Range size variation.

Value	Present	Projected
AUC.train	0.820	0.839
AUC.test	0.820	0.831
AUC.diff	0.025	0.025
pROC	1.456	1.419
OR.p10	0.271	0.171
CBI	0.995	0.720
perc.stable	22.272	22.272
range.size	0.266	0.266

S.3.101 *Erysimum pseudorhaeticum* KDE

Species: *Erysimum pseudorhaeticum* Polatschek

Procedure: KDE

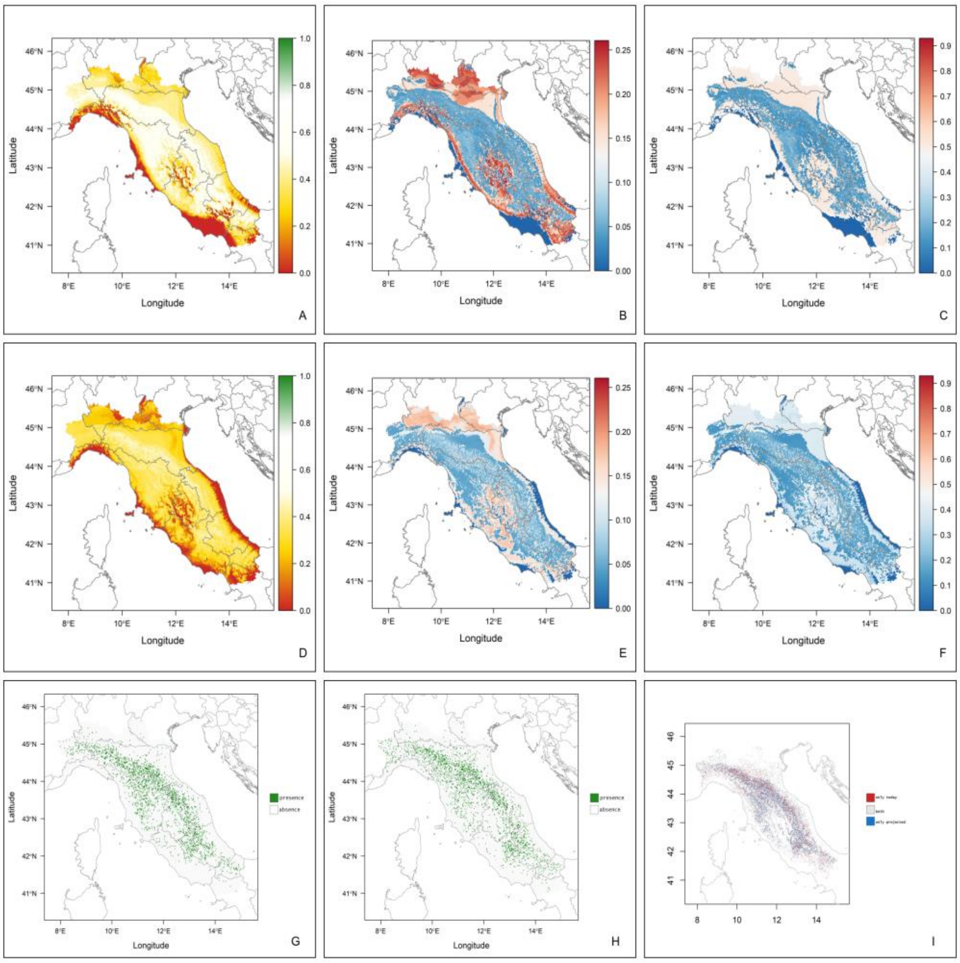

Fig. S.3.101-A) 'Projected' base output; B) 'Projected' uncertainty standard deviation; C) 'Projected' uncertainty range of values; D) 'Present' base output; E) 'Present' uncertainty standard deviation; F) 'Present' uncertainty range of values; G) 'Projected' binary distribution; H) 'Present' binary distribution; I) Range size variation.

Value	Present	Projected
AUC.train	0.654	0.724
AUC.test	0.654	0.645
AUC.diff	0.058	0.078
pROC	1.219	1.231
OR.p10	0.193	0.214
CBI	0.903	0.116
perc.stable	11.409	11.409
range.size	-0.769	-0.769

S.3.102 *Erysimum pseudorhaeticum* CTA

Species: *Erysimum pseudorhaeticum* Polatschek

Procedure: CTA

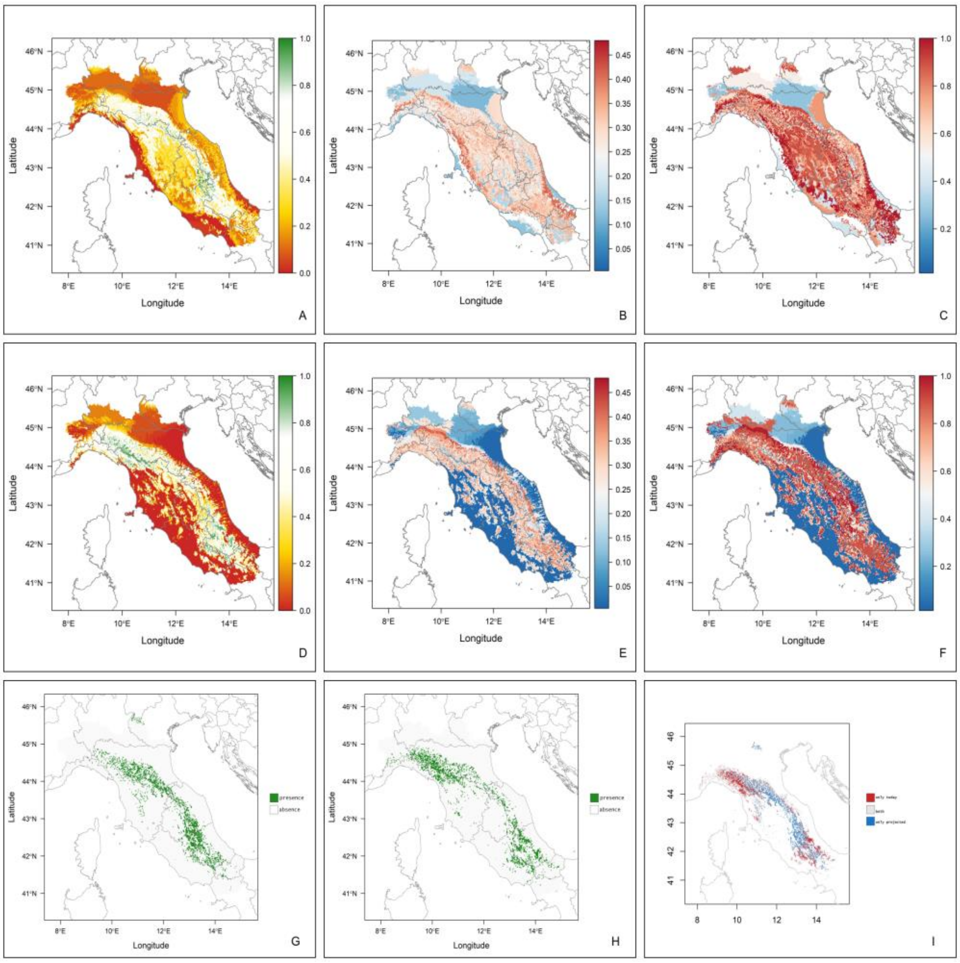

Fig. S.3.102-A) 'Projected' base output; B) 'Projected' uncertainty standard deviation; C) 'Projected' uncertainty range of values; D) 'Present' base output; E) 'Present' uncertainty standard deviation; F) 'Present' uncertainty range of values; G) 'Projected' binary distribution; H) 'Present' binary distribution; I) Range size variation.

Value	Present	Projected
AUC.train	0.823	0.935
AUC.test	0.823	0.776
AUC.diff	0.159	0.159
pROC	1.190	1.095
OR.p10	0.278	0.371
CBI	0.985	0.783
perc.stable	18.855	18.855
range.size	-0.530	-0.530

S.3.103 *Erysimum pseudorhaeticum* FDA

Species: *Erysimum pseudorhaeticum* Polatschek

Procedure: FDA

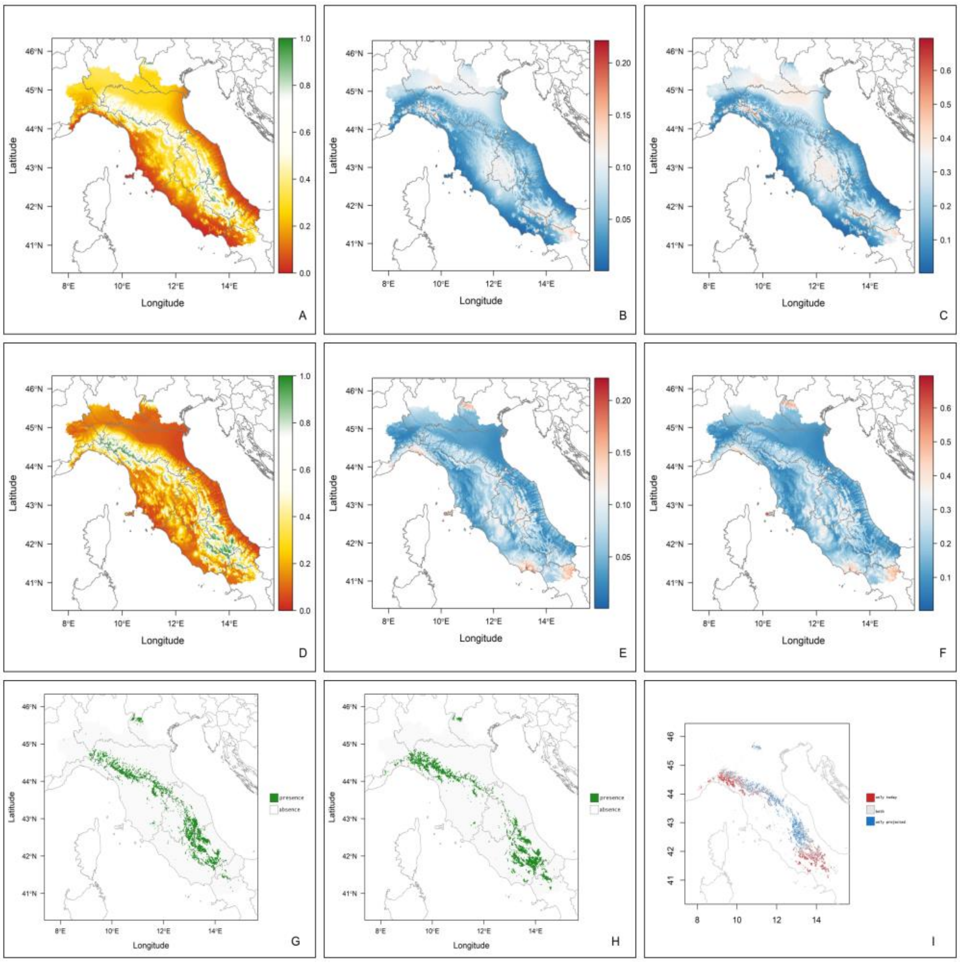

Fig. S.3.103-A) 'Projected' base output; B) 'Projected' uncertainty standard deviation; C) 'Projected' uncertainty range of values; D) 'Present' base output; E) 'Present' uncertainty standard deviation; F) 'Present' uncertainty range of values; G) 'Projected' binary distribution; H) 'Present' binary distribution; I) Range size variation.

Value	Present	Projected
AUC.train	0.826	0.801
AUC.test	0.826	0.813
AUC.diff	0.020	0.020
pROC	1.433	1.340
OR.p10	0.155	0.107
CBI	0.992	0.975
perc.stable	39.635	39.635
range.size	0.791	0.791

S.3.104 *Erysimum pseudorhaeticum* GAM

Species: *Erysimum pseudorhaeticum* Polatschek

Procedure: GAM

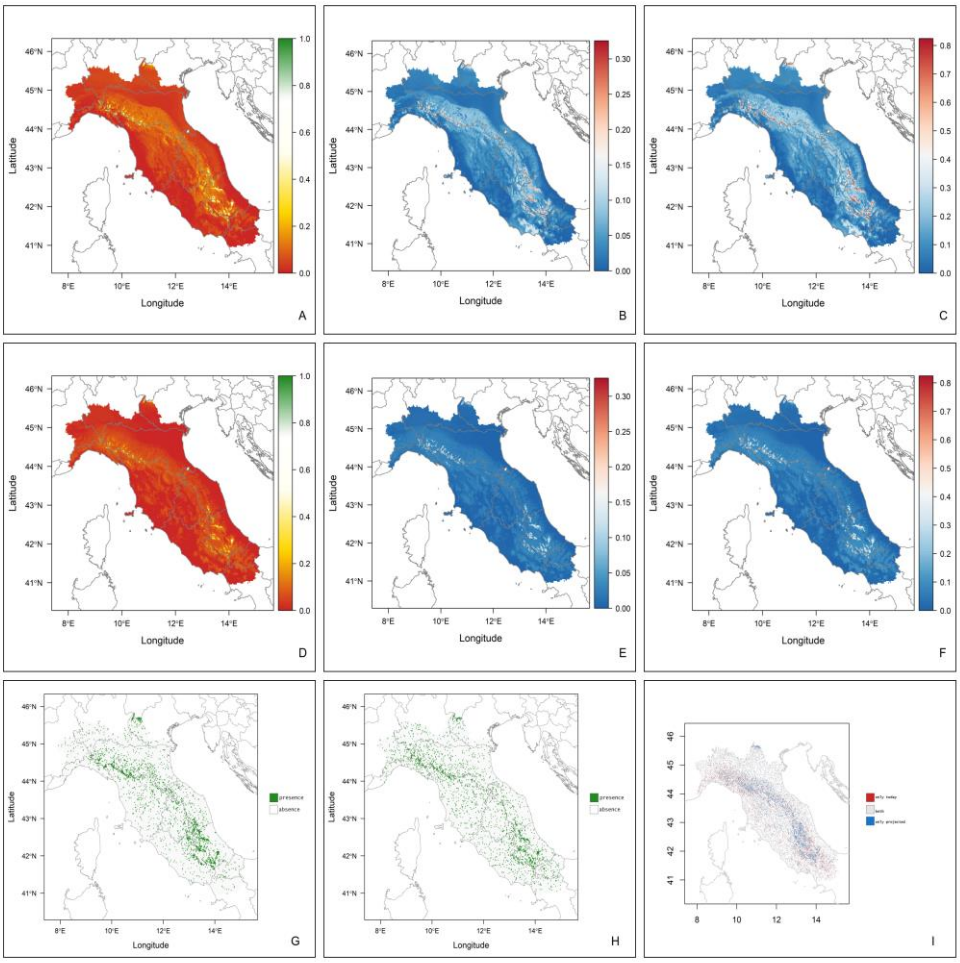

Fig. S.3.104-A) 'Projected' base output; B) 'Projected' uncertainty standard deviation; C) 'Projected' uncertainty range of values; D) 'Present' base output; E) 'Present' uncertainty standard deviation; F) 'Present' uncertainty range of values; G) 'Projected' binary distribution; H) 'Present' binary distribution; I) Range size variation.

Value	Present	Projected
AUC.train	0.787	0.847
AUC.test	0.787	0.773
AUC.diff	0.079	0.079
pROC	1.439	1.301
OR.p10	0.282	0.279
CBI	0.795	0.767
perc.stable	12.546	12.546
range.size	0.068	0.068

S.3.105 *Erysimum pseudorhaeticum* GLM

Species: *Erysimum pseudorhaeticum* Polatschek

Procedure: GLM

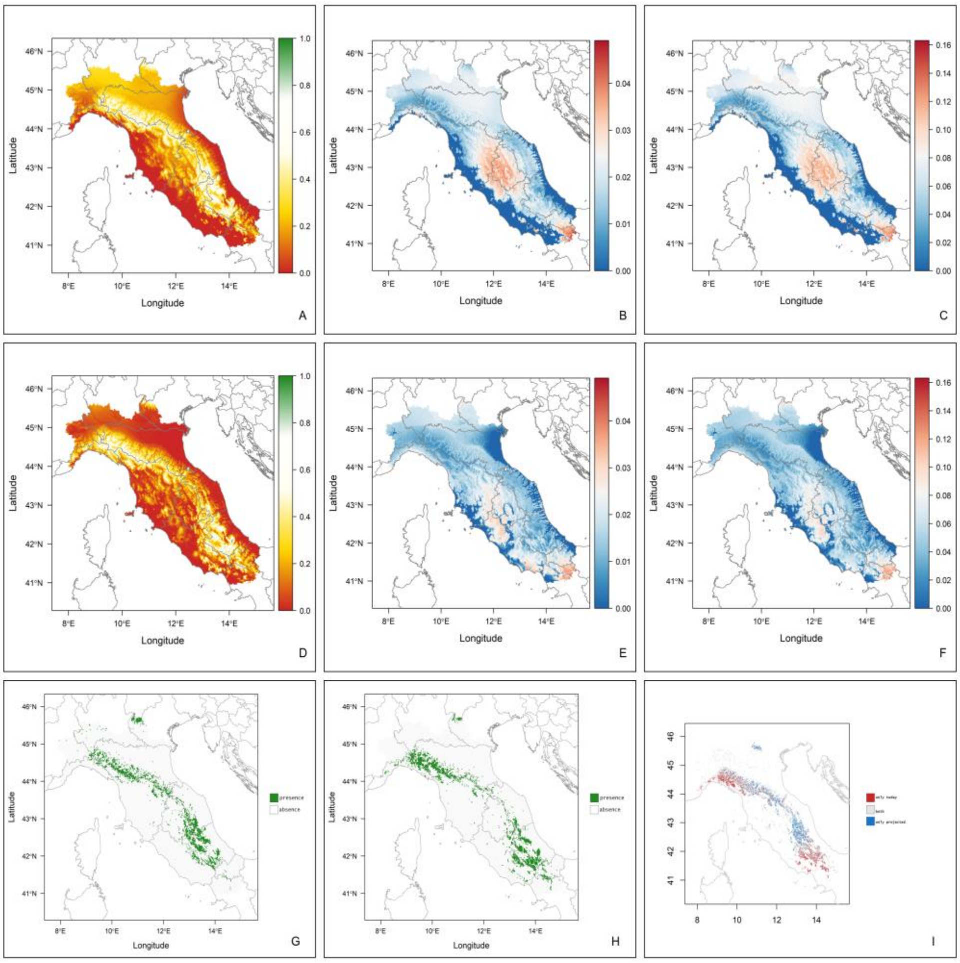

Fig. S.3.105-A) 'Projected' base output; B) 'Projected' uncertainty standard deviation; C) 'Projected' uncertainty range of values; D) 'Present' base output; E) 'Present' uncertainty standard deviation; F) 'Present' uncertainty range of values; G) 'Projected' binary distribution; H) 'Present' binary distribution; I) Range size variation.

Value	Present	Projected
AUC.train	0.819	0.788
AUC.test	0.819	0.801
AUC.diff	0.012	0.012
pROC	1.464	1.333
OR.p10	0.158	0.143
CBI	0.918	0.922
perc.stable	37.931	37.931
range.size	-3.024	-3.024

S.3.106 *Erysimum pseudorhaeticum* MARS

Species: *Erysimum pseudorhaeticum* Polatschek

Procedure: MARS

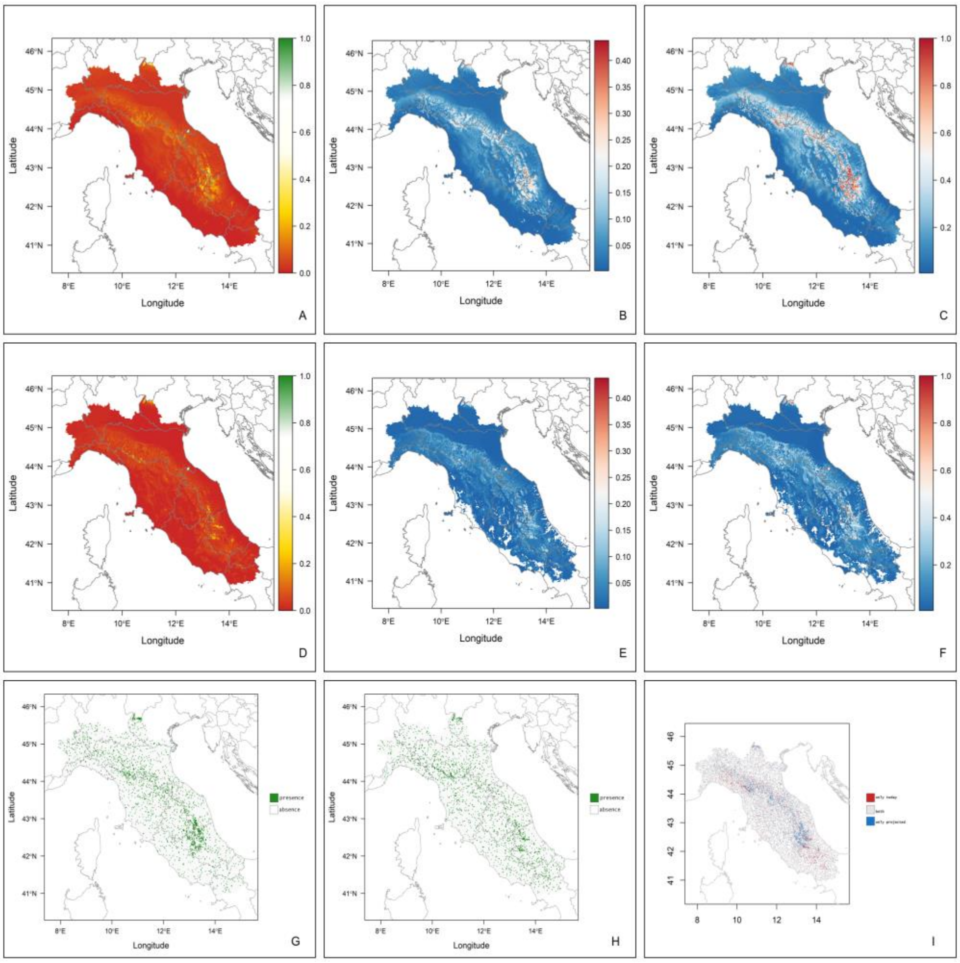

Fig. S.3.106-A) 'Projected' base output; B) 'Projected' uncertainty standard deviation; C) 'Projected' uncertainty range of values; D) 'Present' base output; E) 'Present' uncertainty standard deviation; F) 'Present' uncertainty range of values; G) 'Projected' binary distribution; H) 'Present' binary distribution; I) Range size variation.

Value	Present	Projected
AUC.train	0.721	0.805
AUC.test	0.721	0.755
AUC.diff	0.060	0.060
pROC	1.231	1.250
OR.p10	0.328	0.221
CBI	0.744	0.780
perc.stable	6.852	6.852
range.size	0.206	0.206

S.3.107 *Erysimum pseudorhaeticum* RF

Species: *Erysimum pseudorhaeticum* Polatschek

Procedure: RF

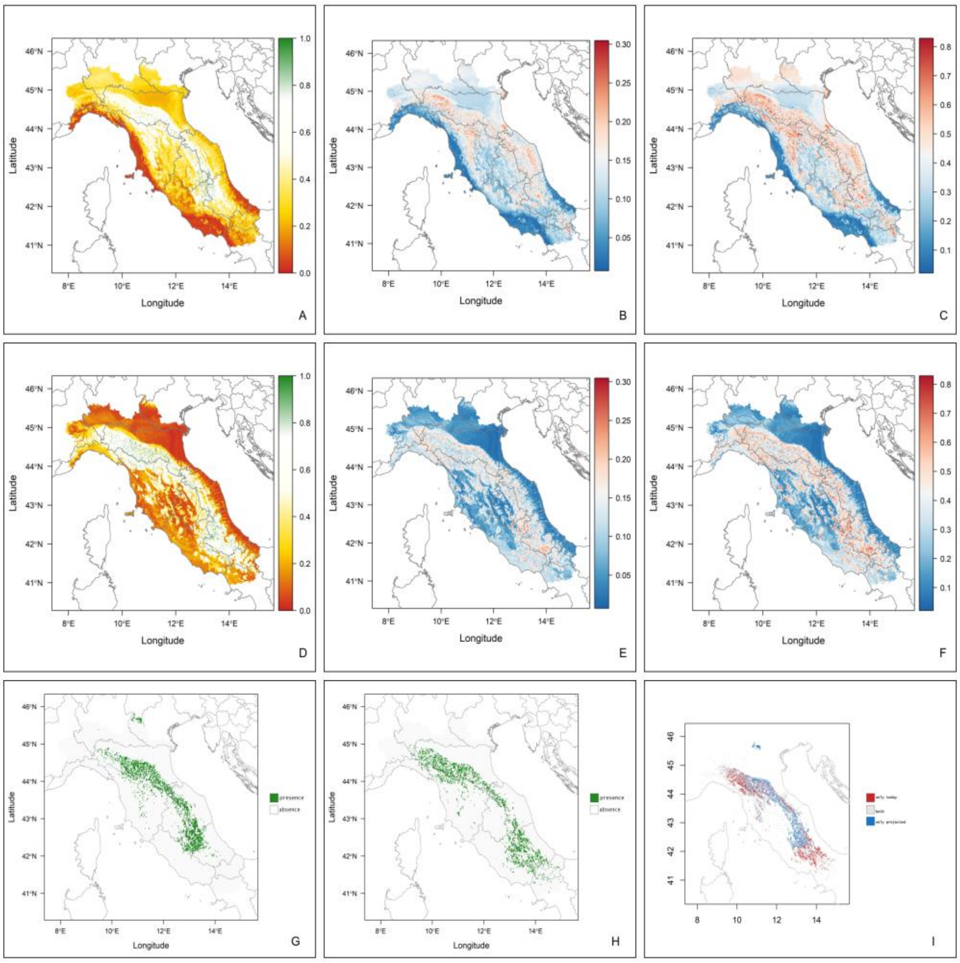

Fig. S.3.107-A) 'Projected' base output; B) 'Projected' uncertainty standard deviation; C) 'Projected' uncertainty range of values; D) 'Present' base output; E) 'Present' uncertainty standard deviation; F) 'Present' uncertainty range of values; G) 'Projected' binary distribution; H) 'Present' binary distribution; I) Range size variation.

Value	Present	Projected
AUC.train	0.980	1.000
AUC.test	0.980	0.940
AUC.diff	0.060	0.060
pROC	1.432	1.324
OR.p10	0.686	0.729
CBI	0.993	0.926
perc.stable	22.845	22.845
range.size	-0.986	-0.986

S.3.108 *Erysimum pseudorhaeticum* SVM

Species: *Erysimum pseudorhaeticum* Polatschek

Procedure: SVM

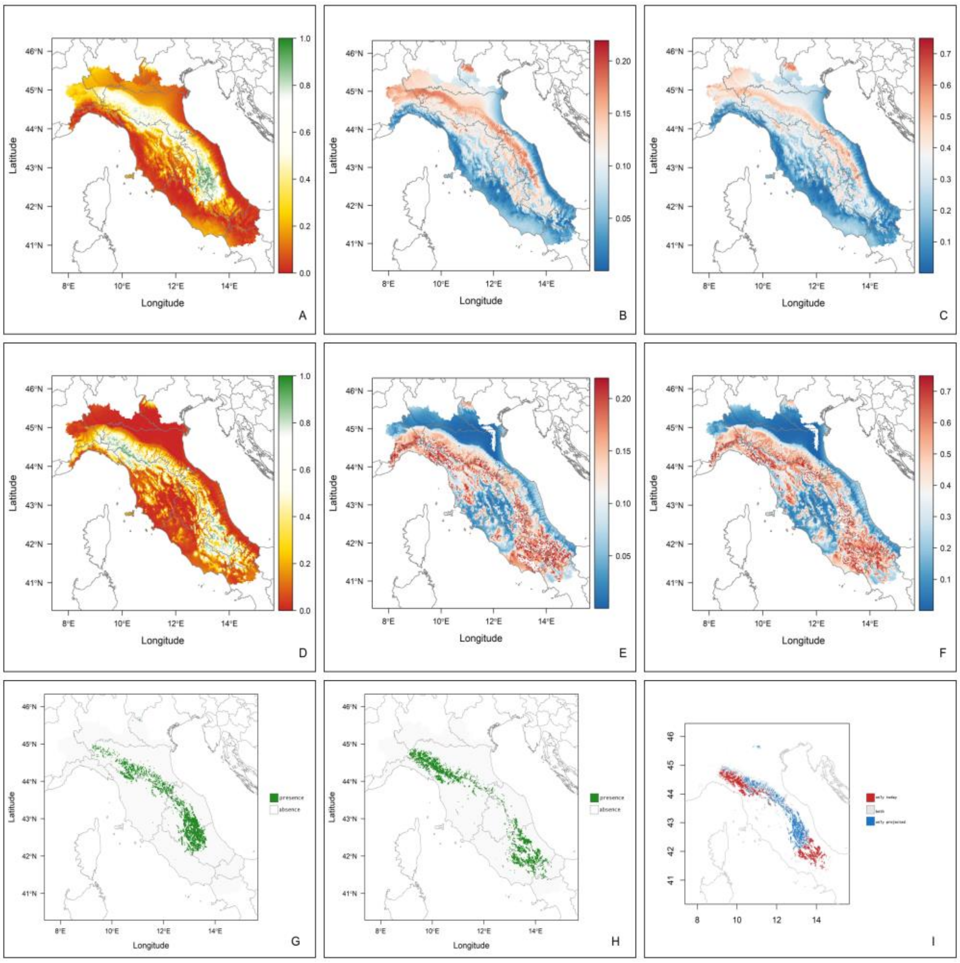

Fig. S.3.108-A) 'Projected' base output; B) 'Projected' uncertainty standard deviation; C) 'Projected' uncertainty range of values; D) 'Present' base output; E) 'Present' uncertainty standard deviation; F) 'Present' uncertainty range of values; G) 'Projected' binary distribution; H) 'Present' binary distribution; I) Range size variation.

Value	Present	Projected
AUC.train	0.826	0.869
AUC.test	0.826	0.818
AUC.diff	0.059	0.059
pROC	1.356	1.294
OR.p10	0.176	0.250
CBI	0.986	0.825
perc.stable	14.334	14.334
range.size	-1.565	-1.565

S.3.109 *Galium magellense* Biomod2

Species: *Galium magellense* Ten.

Procedure: Biomod2

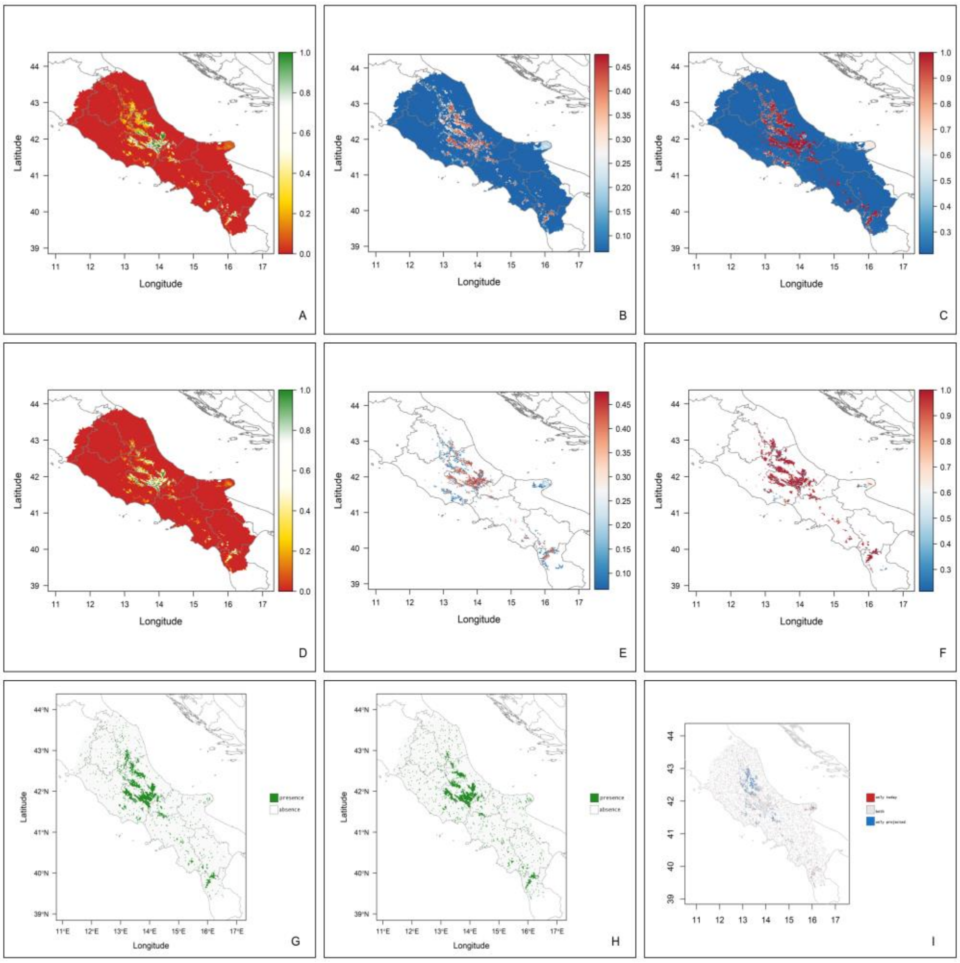

Fig. S.3.109-A) 'Projected' base output; B) 'Projected' uncertainty standard deviation; C) 'Projected' uncertainty range of values; D) 'Present' base output; E) 'Present' uncertainty standard deviation; F) 'Present' uncertainty range of values; G) 'Projected' binary distribution; H) 'Present' binary distribution; I) Range size variation.

Value	Present	Projected
AUC.train	0.994	1.000
AUC.test	0.994	0.994
AUC.diff	0.006	0.006
pROC	1.948	1.946
OR.p10	0.242	0.425
CBI	0.922	0.831
perc.stable	39.368	39.368
range.size	0.716	0.716

S.3.110 *Galium magellense* Bioclim

Species: *Galium magellense* Ten.

Procedure: Bioclim

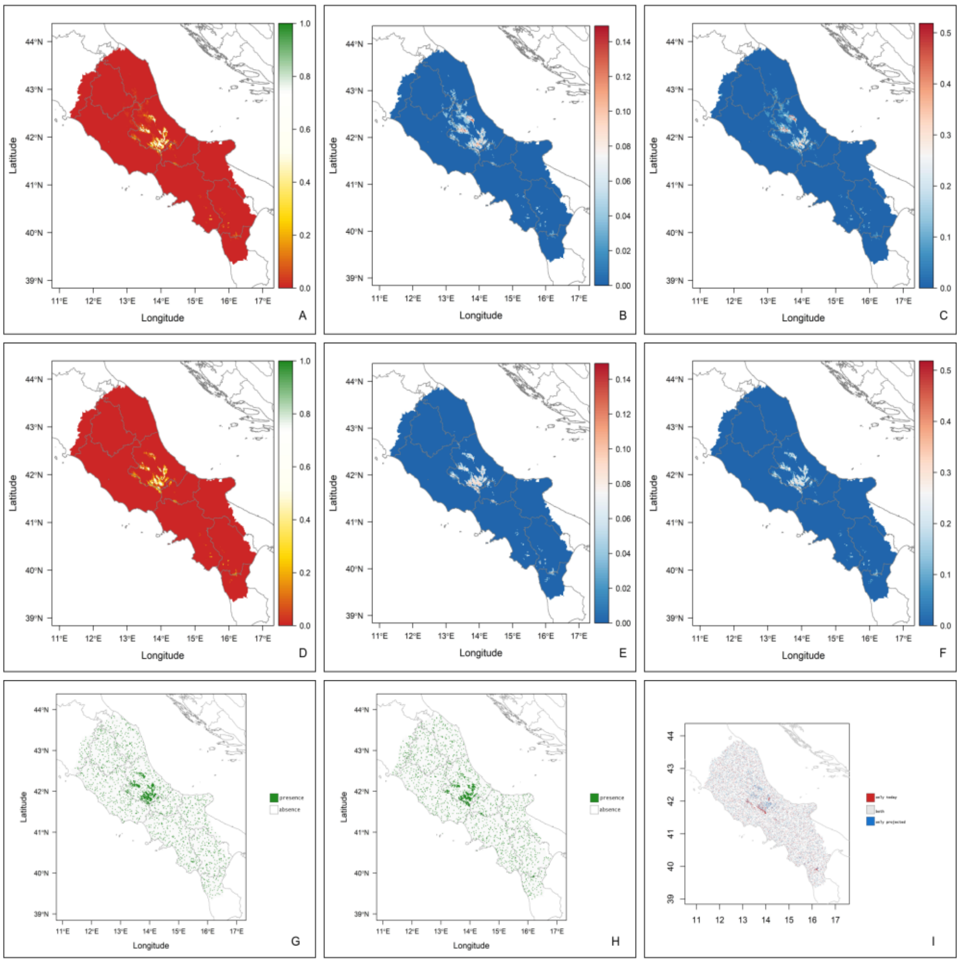

Fig. S.3.110-A) 'Projected' base output; B) 'Projected' uncertainty standard deviation; C) 'Projected' uncertainty range of values; D) 'Present' base output; E) 'Present' uncertainty standard deviation; F) 'Present' uncertainty range of values; G) 'Projected' binary distribution; H) 'Present' binary distribution; I) Range size variation.

Value	Present	Projected
AUC.train	0.907	0.985
AUC.test	0.907	0.884
AUC.diff	0.102	0.102
pROC	1.432	1.341
OR.p10	0.167	0.208
CBI	0.435	0.466
perc.stable	11.003	11.003
range.size	2.038	2.038

S.3.111 *Galium magellense* Domain

Species: *Galium magellense* Ten.

Procedure: Domain

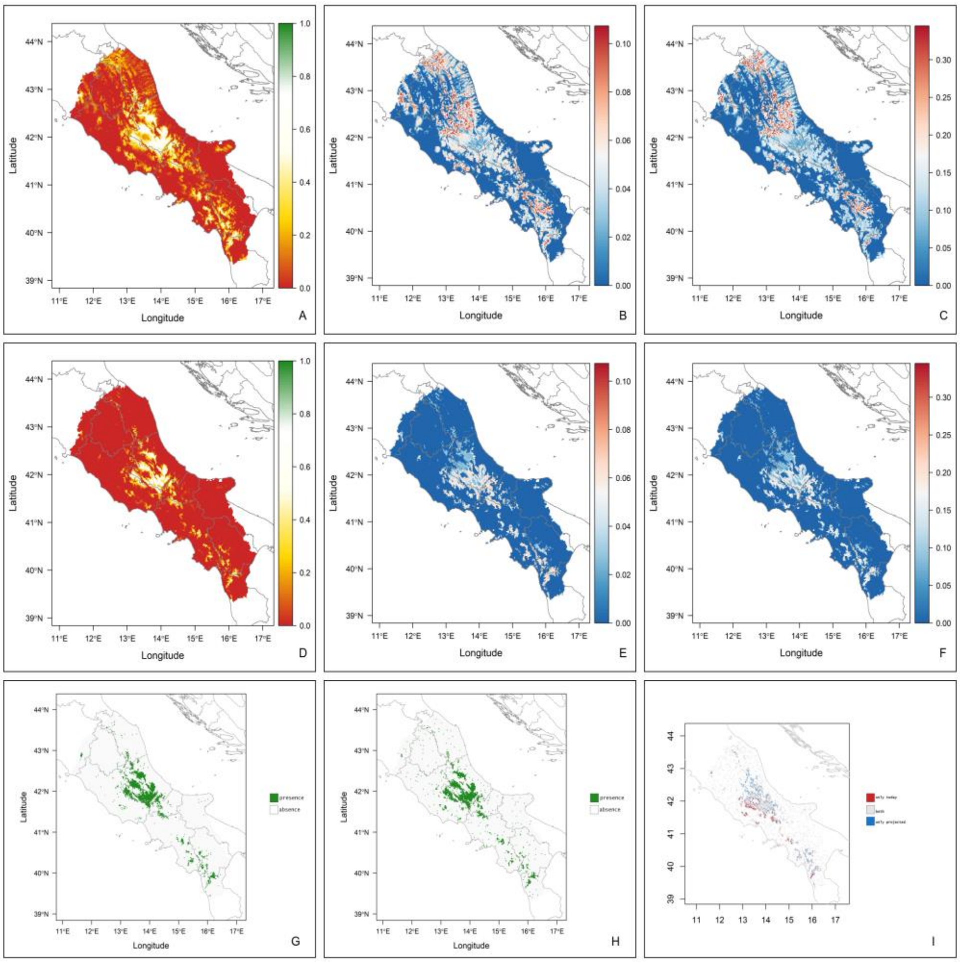

Fig. S.3.111-A) 'Projected' base output; B) 'Projected' uncertainty standard deviation; C) 'Projected' uncertainty range of values; D) 'Present' base output; E) 'Present' uncertainty standard deviation; F) 'Present' uncertainty range of values; G) 'Projected' binary distribution; H) 'Present' binary distribution; I) Range size variation.

Value	Present	Projected
AUC.train	0.985	0.980
AUC.test	0.985	0.976
AUC.diff	0.007	0.007
pROC	1.942	1.893
OR.p10	0.167	0.108
CBI	0.982	0.957
perc.stable	53.471	53.471
range.size	0.698	0.698

S.3.112 *Galium magellense* Maxent

Species: *Galium magellense* Ten.

Procedure: Maxent

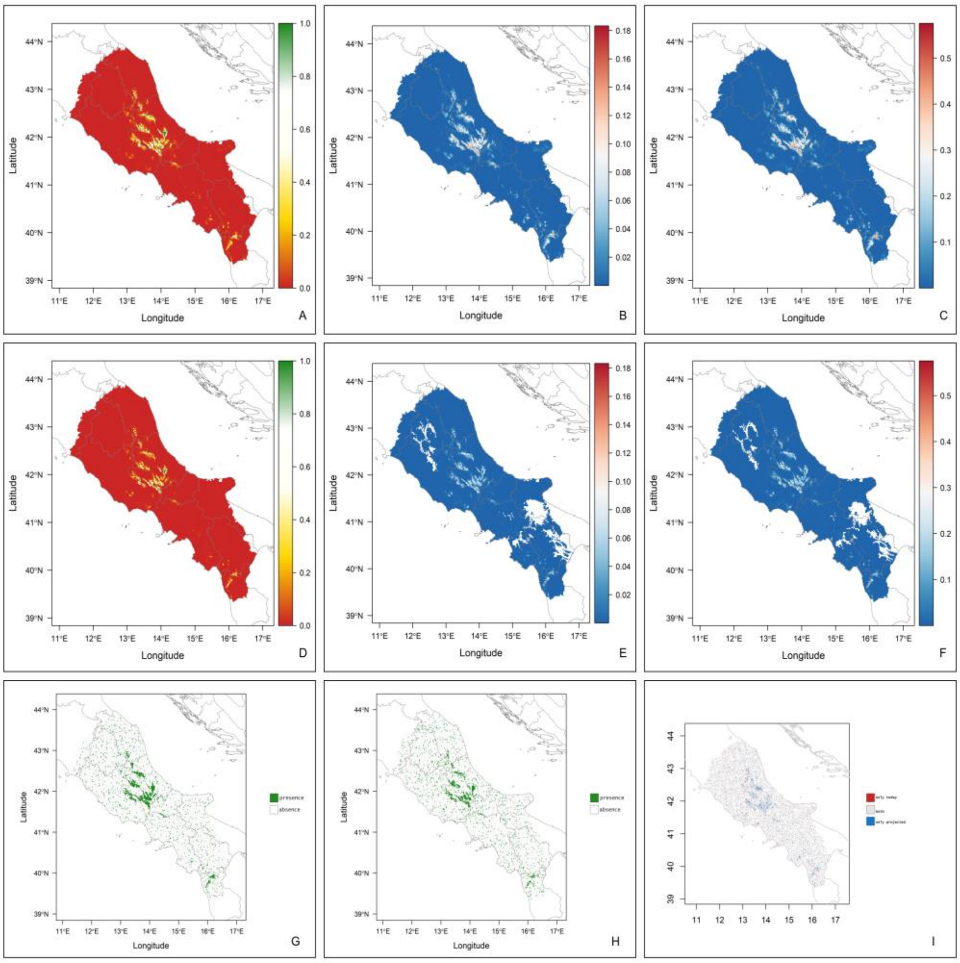

Fig. S.3.112-A) 'Projected' base output; B) 'Projected' uncertainty standard deviation; C) 'Projected' uncertainty range of values; D) 'Present' base output; E) 'Present' uncertainty standard deviation; F) 'Present' uncertainty range of values; G) 'Projected' binary distribution; H) 'Present' binary distribution; I) Range size variation.

Value	Present	Projected
AUC.train	0.991	0.991
AUC.test	0.991	0.986
AUC.diff	0.006	0.006
pROC	1.939	1.925
OR.p10	0.175	0.225
CBI	0.914	0.892
perc.stable	20.058	20.058
range.size	1.218	1.218

S.3.113 *Galium magellense* KDE

Species: *Galium magellense* Ten.

Procedure: KDE

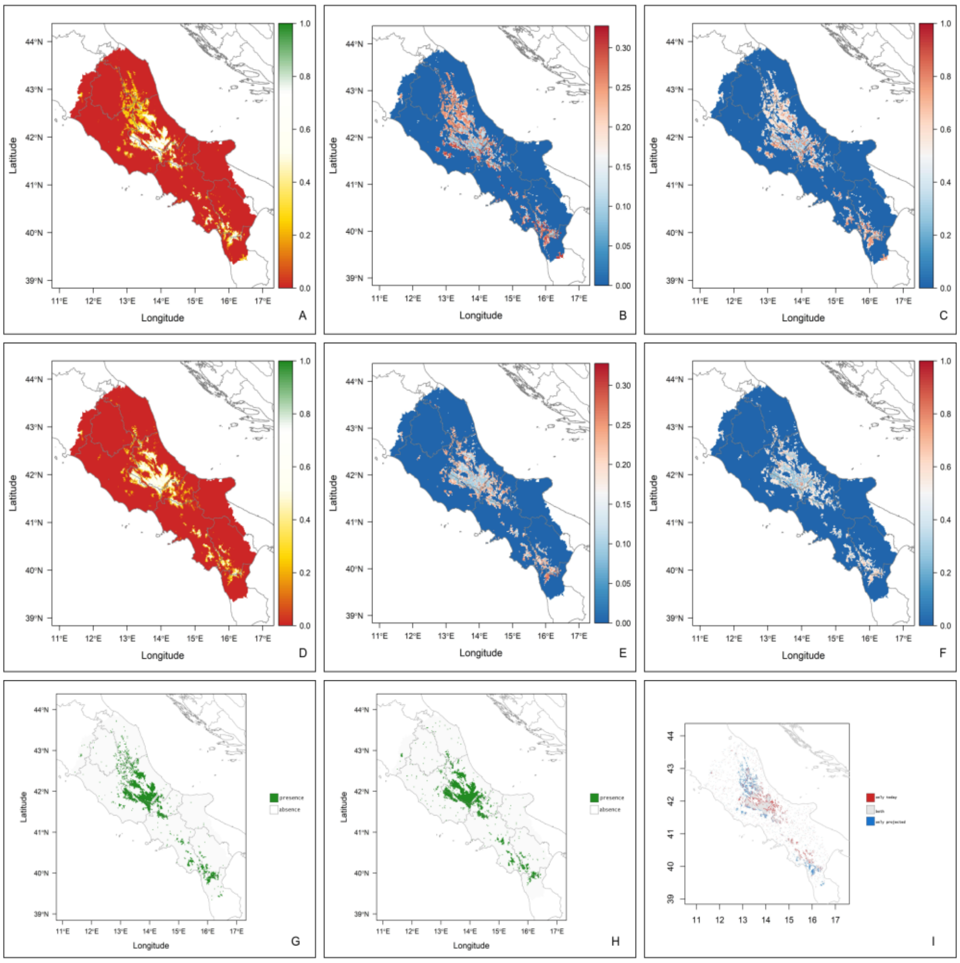

Fig. S.3.113-A) 'Projected' base output; B) 'Projected' uncertainty standard deviation; C) 'Projected' uncertainty range of values; D) 'Present' base output; E) 'Present' uncertainty standard deviation; F) 'Present' uncertainty range of values; G) 'Projected' binary distribution; H) 'Present' binary distribution; I) Range size variation.

Value	Present	Projected
AUC.train	0.966	0.974
AUC.test	0.966	0.943
AUC.diff	0.025	0.033
pROC	1.774	1.622
OR.p10	0.283	0.233
CBI	0.938	0.932
perc.stable	53.903	53.903
range.size	1.069	1.069

S.3.114 *Galium magellense* CTA

Species: *Galium magellense* Ten.

Procedure: CTA

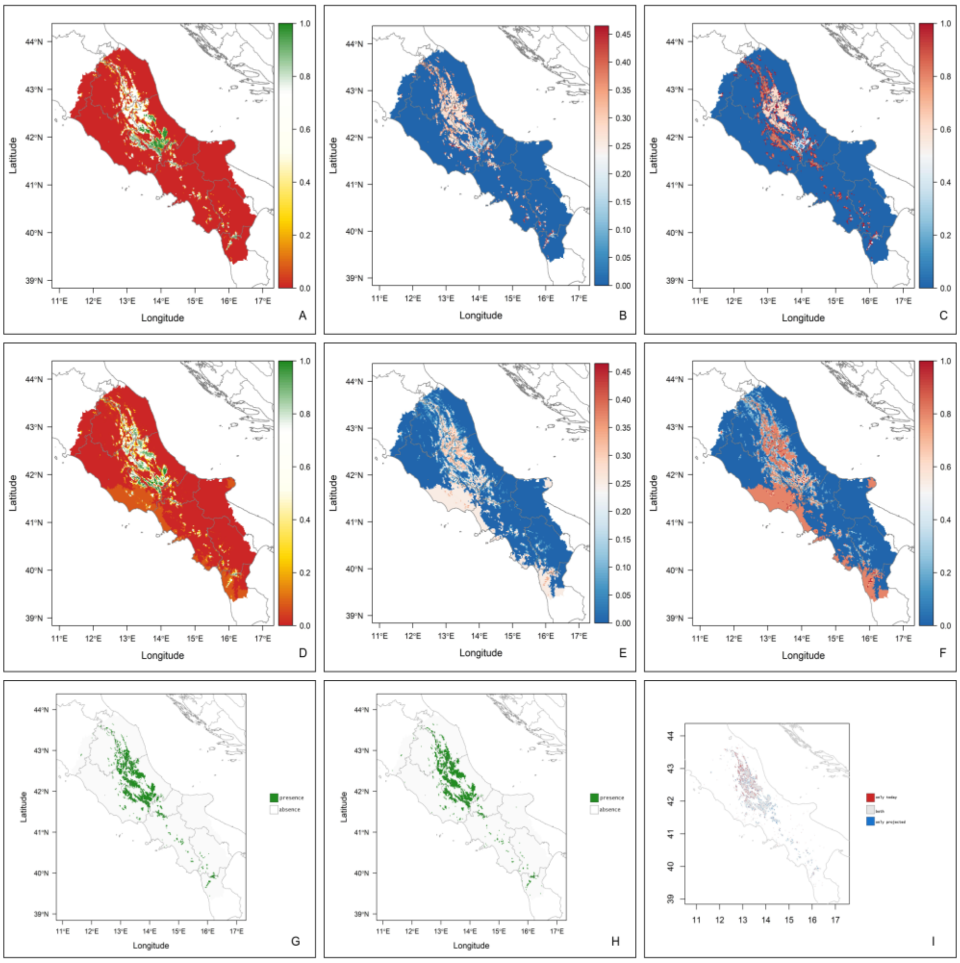

Fig. S.3.114-A) 'Projected' base output; B) 'Projected' uncertainty standard deviation; C) 'Projected' uncertainty range of values; D) 'Present' base output; E) 'Present' uncertainty standard deviation; F) 'Present' uncertainty range of values; G) 'Projected' binary distribution; H) 'Present' binary distribution; I) Range size variation.

Value	Present	Projected
AUC.train	0.975	0.995
AUC.test	0.975	0.974
AUC.diff	0.021	0.021
pROC	1.409	1.453
OR.p10	0.100	0.100
CBI	0.764	0.916
perc.stable	74.603	74.603
range.size	0.812	0.812

S.3.115 *Galium magellense* FDA

Species: *Galium magellense* Ten.

Procedure: FDA

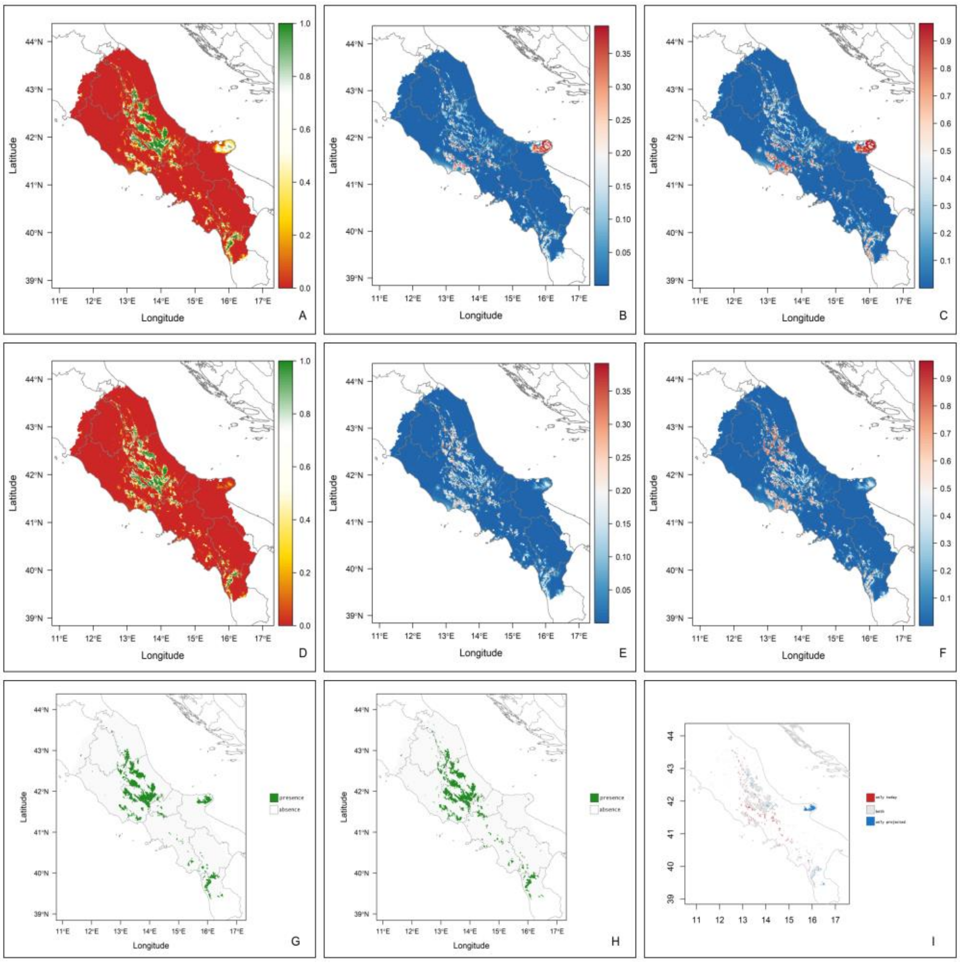

Fig. S.3.115-A) 'Projected' base output; B) 'Projected' uncertainty standard deviation; C) 'Projected' uncertainty range of values; D) 'Present' base output; E) 'Present' uncertainty standard deviation; F) 'Present' uncertainty range of values; G) 'Projected' binary distribution; H) 'Present' binary distribution; I) Range size variation.

Value	Present	Projected
AUC.train	0.992	0.994
AUC.test	0.992	0.994
AUC.diff	0.002	0.002
pROC	1.793	1.851
OR.p10	0.125	0.150
CBI	0.642	0.735
perc.stable	73.430	73.430
range.size	-0.326	-0.326

S.3.116 *Galium magellense* GAM

Species: *Galium magellense* Ten.

Procedure: GAM

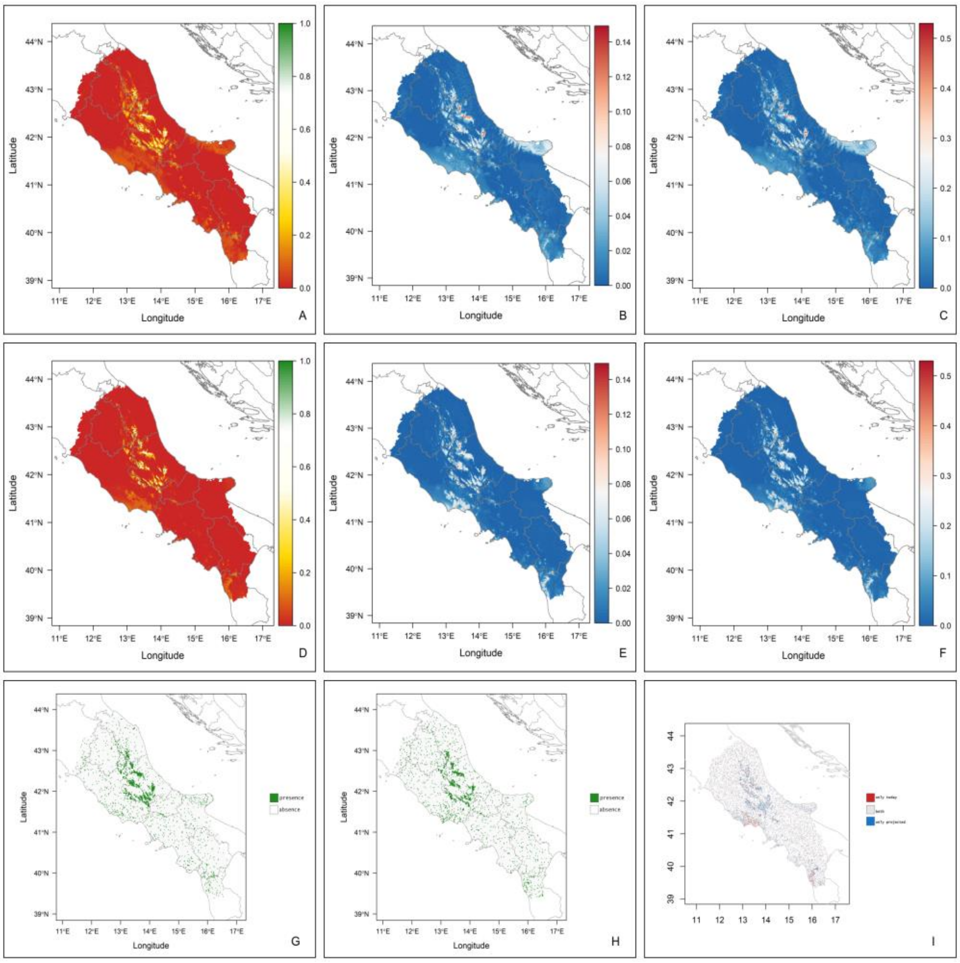

Fig. S.3.116-A) 'Projected' base output; B) 'Projected' uncertainty standard deviation; C) 'Projected' uncertainty range of values; D) 'Present' base output; E) 'Present' uncertainty standard deviation; F) 'Present' uncertainty range of values; G) 'Projected' binary distribution; H) 'Present' binary distribution; I) Range size variation.

Value	Present	Projected
AUC.train	0.985	0.984
AUC.test	0.985	0.987
AUC.diff	0.003	0.003
pROC	1.904	1.923
OR.p10	0.100	0.083
CBI	0.989	0.963
perc.stable	18.322	18.322
range.size	-0.529	-0.529

S.3.117 *Galium magellense* GLM

Species: *Galium magellense* Ten.

Procedure: GLM

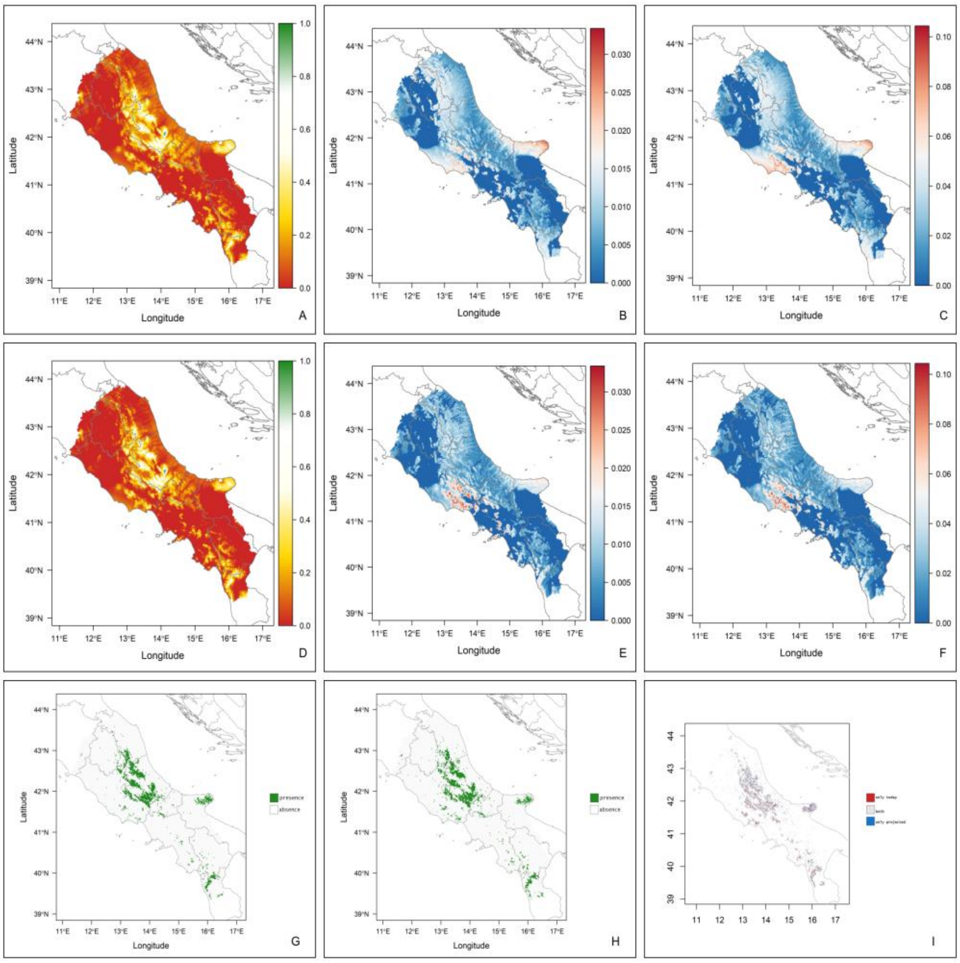

Fig. S.3.117-A) 'Projected' base output; B) 'Projected' uncertainty standard deviation; C) 'Projected' uncertainty range of values; D) 'Present' base output; E) 'Present' uncertainty standard deviation; F) 'Present' uncertainty range of values; G) 'Projected' binary distribution; H) 'Present' binary distribution; I) Range size variation.

Value	Present	Projected
AUC.train	0.991	0.986
AUC.test	0.991	0.988
AUC.diff	0.002	0.002
pROC	1.940	1.935
OR.p10	0.125	0.100
CBI	0.994	0.993
perc.stable	50.813	50.813
range.size	-4.077	-4.077

S.3.118 *Galium magellense* MARS

Species: *Galium magellense* Ten.

Procedure: MARS

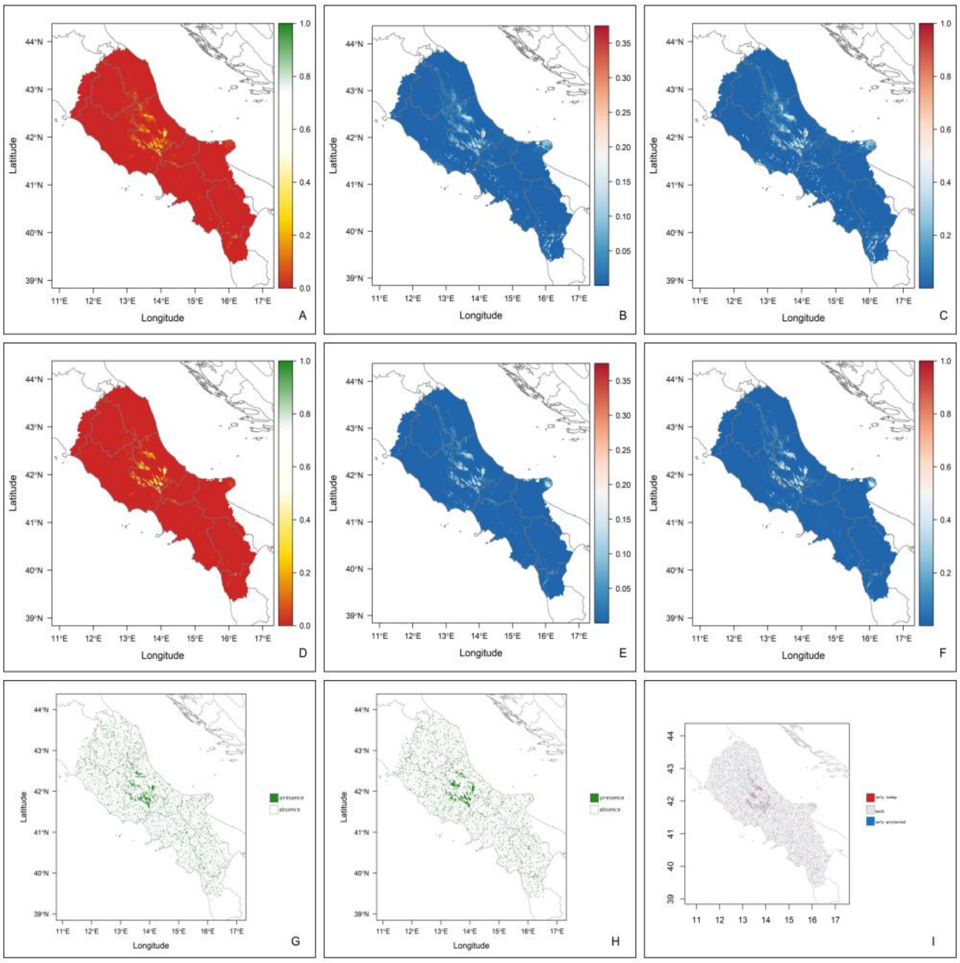

Fig. S.3.118-A) 'Projected' base output; B) 'Projected' uncertainty standard deviation; C) 'Projected' uncertainty range of values; D) 'Present' base output; E) 'Present' uncertainty standard deviation; F) 'Present' uncertainty range of values; G) 'Projected' binary distribution; H) 'Present' binary distribution; I) Range size variation.

Value	Present	Projected
AUC.train	0.971	0.990
AUC.test	0.971	0.946
AUC.diff	0.046	0.046
pROC	1.804	1.694
OR.p10	0.192	0.258
CBI	0.899	0.948
perc.stable	9.368	9.368
range.size	-0.409	-0.409

S.3.119 *Galium magellense* RF

Species: *Galium magellense* Ten.

Procedure: RF

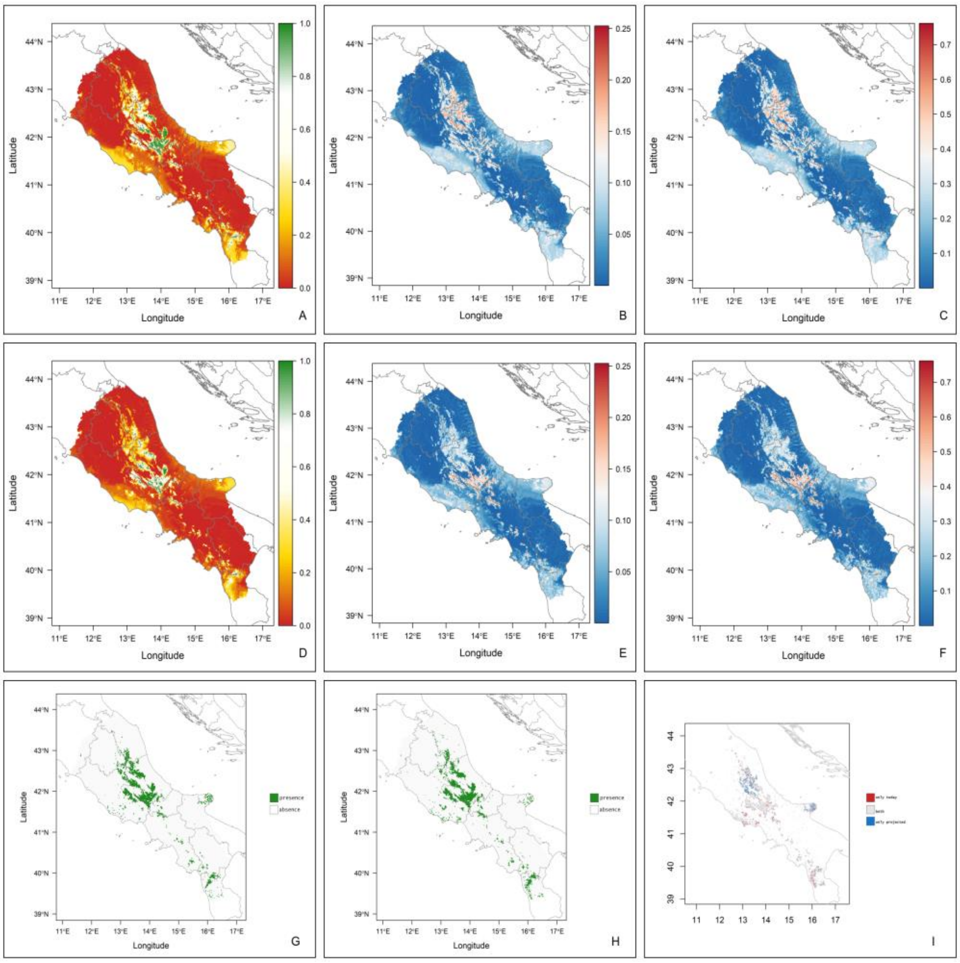

Fig. S.3.119-A) 'Projected' base output; B) 'Projected' uncertainty standard deviation; C) 'Projected' uncertainty range of values; D) 'Present' base output; E) 'Present' uncertainty standard deviation; F) 'Present' uncertainty range of values; G) 'Projected' binary distribution; H) 'Present' binary distribution; I) Range size variation.

Value	Present	Projected
AUC.train	0.998	1.000
AUC.test	0.998	0.999
AUC.diff	0.001	0.001
pROC	1.927	1.921
OR.p10	0.300	0.292
CBI	0.951	0.982
perc.stable	59.724	59.724
range.size	-0.212	-0.212

S.3.120 *Galium magellense* SVM

Species: *Galium magellense* Ten.

Procedure: SVM

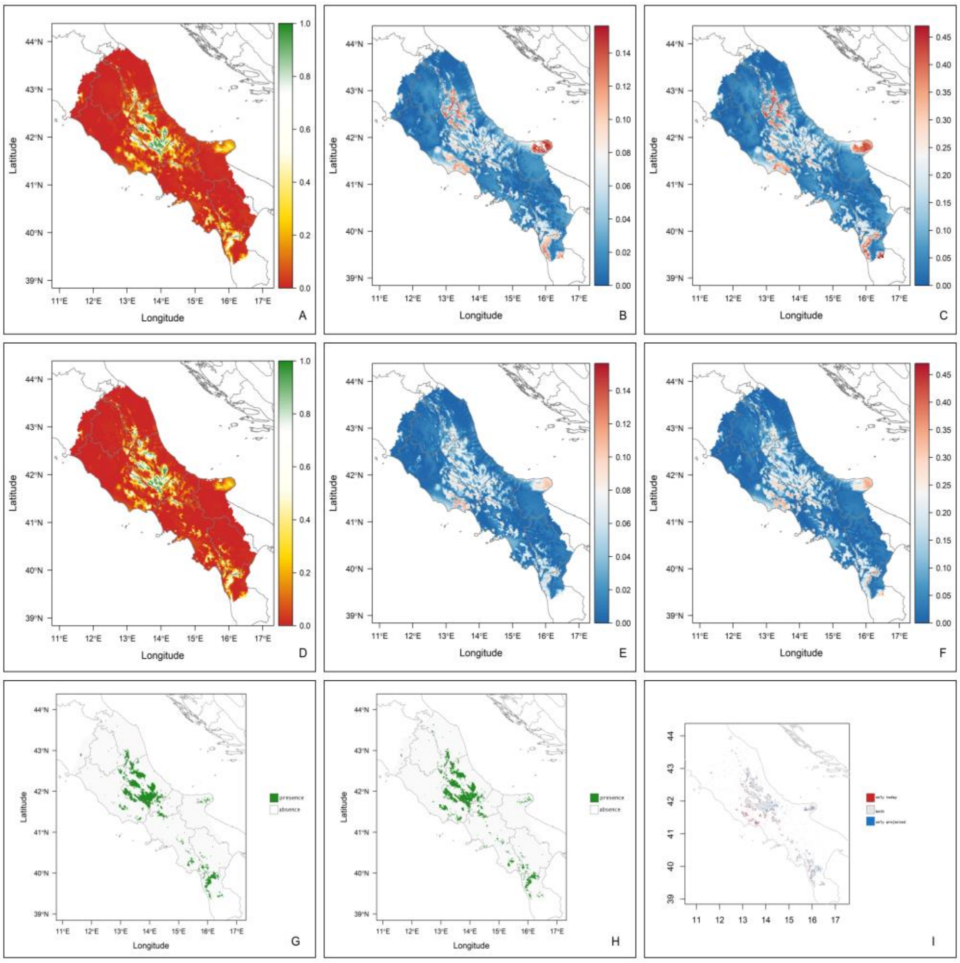

Fig. S.3.120-A) 'Projected' base output; B) 'Projected' uncertainty standard deviation; C) 'Projected' uncertainty range of values; D) 'Present' base output; E) 'Present' uncertainty standard deviation; F) 'Present' uncertainty range of values; G) 'Projected' binary distribution; H) 'Present' binary distribution; I) Range size variation.

Value	Present	Projected
AUC.train	0.989	0.990
AUC.test	0.989	0.991
AUC.diff	0.002	0.002
pROC	1.952	1.934
OR.p10	0.158	0.125
CBI	0.979	0.957
perc.stable	67.726	67.726
range.size	0.110	0.110

S.3.121 *Gypsophila arrostoi* Biomod2

Species: *Gypsophila arrostoi* Guss. subsp. *arrostoi*

Procedure: Biomod2

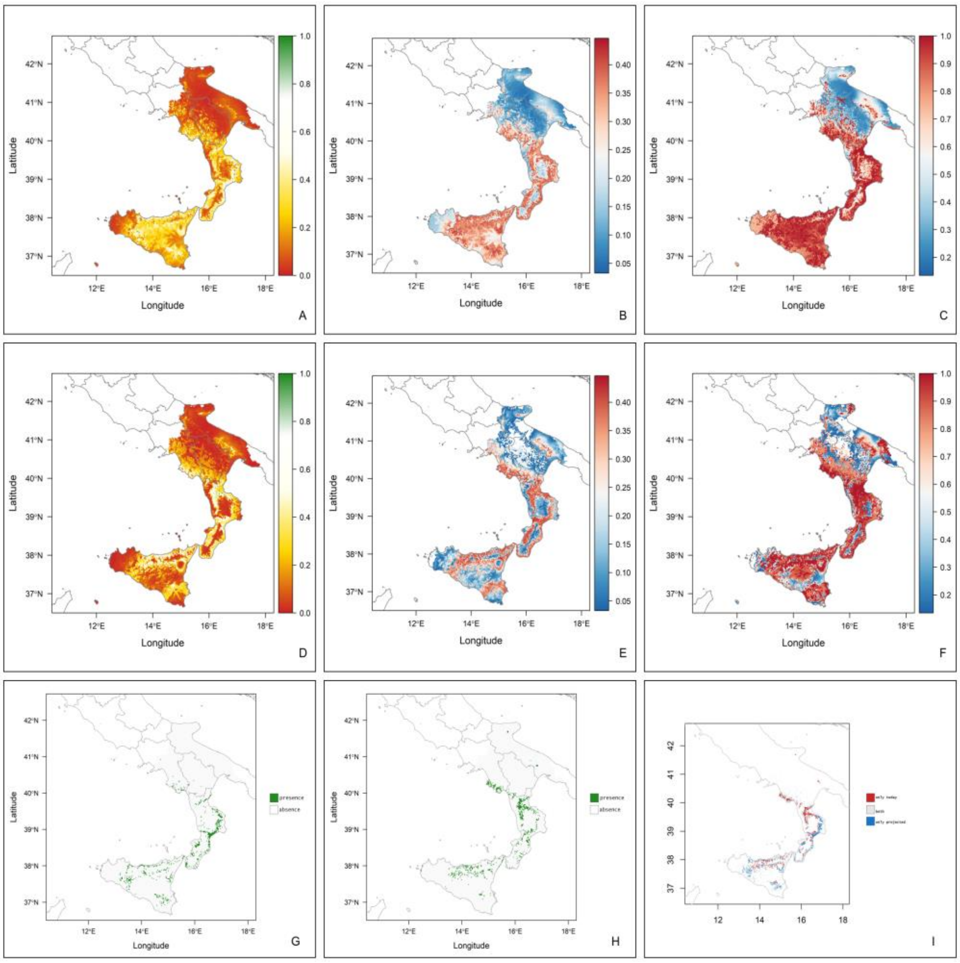

Fig. S.3.121-A) 'Projected' base output; B) 'Projected' uncertainty standard deviation; C) 'Projected' uncertainty range of values; D) 'Present' base output; E) 'Present' uncertainty standard deviation; F) 'Present' uncertainty range of values; G) 'Projected' binary distribution; H) 'Present' binary distribution; I) Range size variation.

Value	Present	Projected
AUC.train	0.886	0.999
AUC.test	0.886	0.800
AUC.diff	0.199	0.199
pROC	1.658	1.472
OR.p10	1.000	0.983
CBI	0.973	0.606
perc.stable	4.768	4.768
range.size	-3.133	-3.133

S.3.122 *Gypsophila arrostoi* Bioclim

Species: *Gypsophila arrostoi* Guss. subsp. *arrostoi*

Procedure: Bioclim

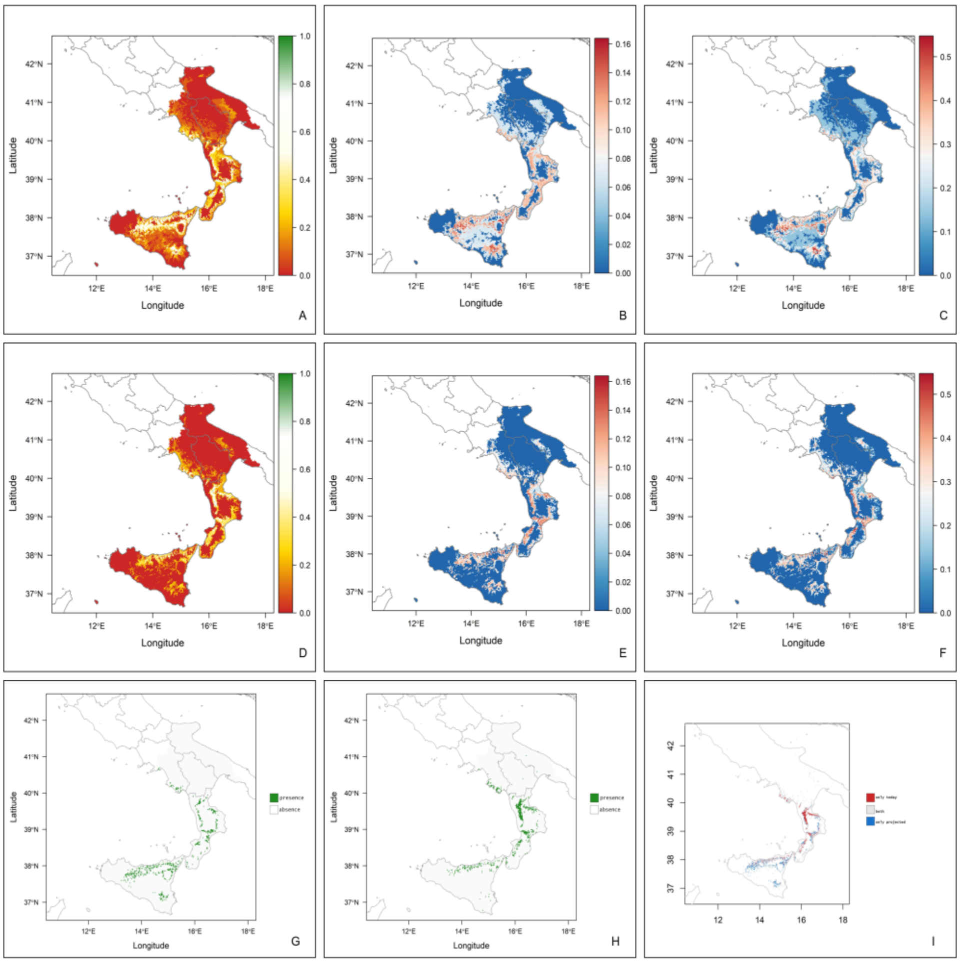

Fig. S.3.122-A) 'Projected' base output; B) 'Projected' uncertainty standard deviation; C) 'Projected' uncertainty range of values; D) 'Present' base output; E) 'Present' uncertainty standard deviation; F) 'Present' uncertainty range of values; G) 'Projected' binary distribution; H) 'Present' binary distribution; I) Range size variation.

Value	Present	Projected
AUC.train	0.739	0.839
AUC.test	0.739	0.740
AUC.diff	0.122	0.122
pROC	1.235	1.297
OR.p10	0.271	0.226
CBI	0.549	0.351
perc.stable	19.467	19.467
range.size	4.515	4.515

S.3.123 *Gypsophila arrostoi* Domain

Species: *Gypsophila arrostoi* Guss. subsp. *arrostoi*

Procedure: Domain

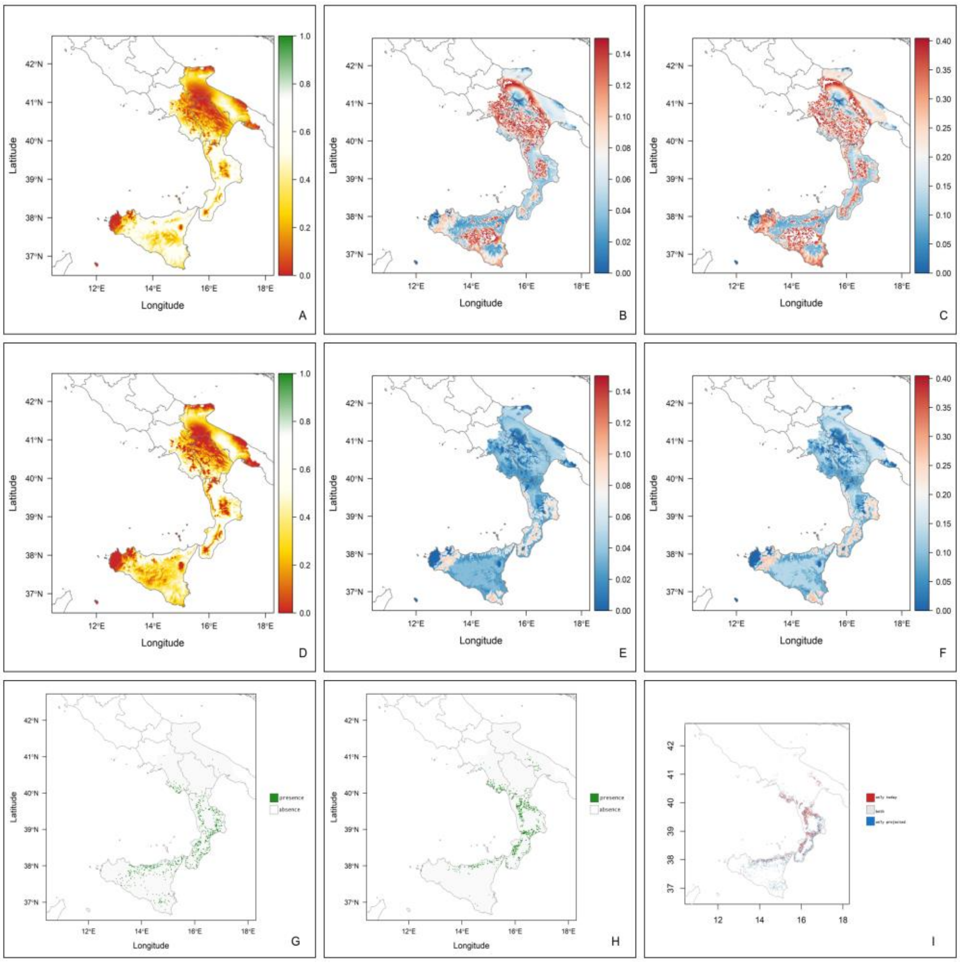

Fig. S.3.123-A) 'Projected' base output; B) 'Projected' uncertainty standard deviation; C) 'Projected' uncertainty range of values; D) 'Present' base output; E) 'Present' uncertainty standard deviation; F) 'Present' uncertainty range of values; G) 'Projected' binary distribution; H) 'Present' binary distribution; I) Range size variation.

Value	Present	Projected
AUC.train	0.818	0.816
AUC.test	0.818	0.710
AUC.diff	0.111	0.111
pROC	1.542	1.374
OR.p10	0.171	0.348
CBI	0.835	0.542
perc.stable	11.632	11.632
range.size	-4.746	-4.746

S.3.124 *Gypsophila arrostoi* Maxent

Species: *Gypsophila arrostoi* Guss. subsp. *arrostoi*

Procedure: Maxent

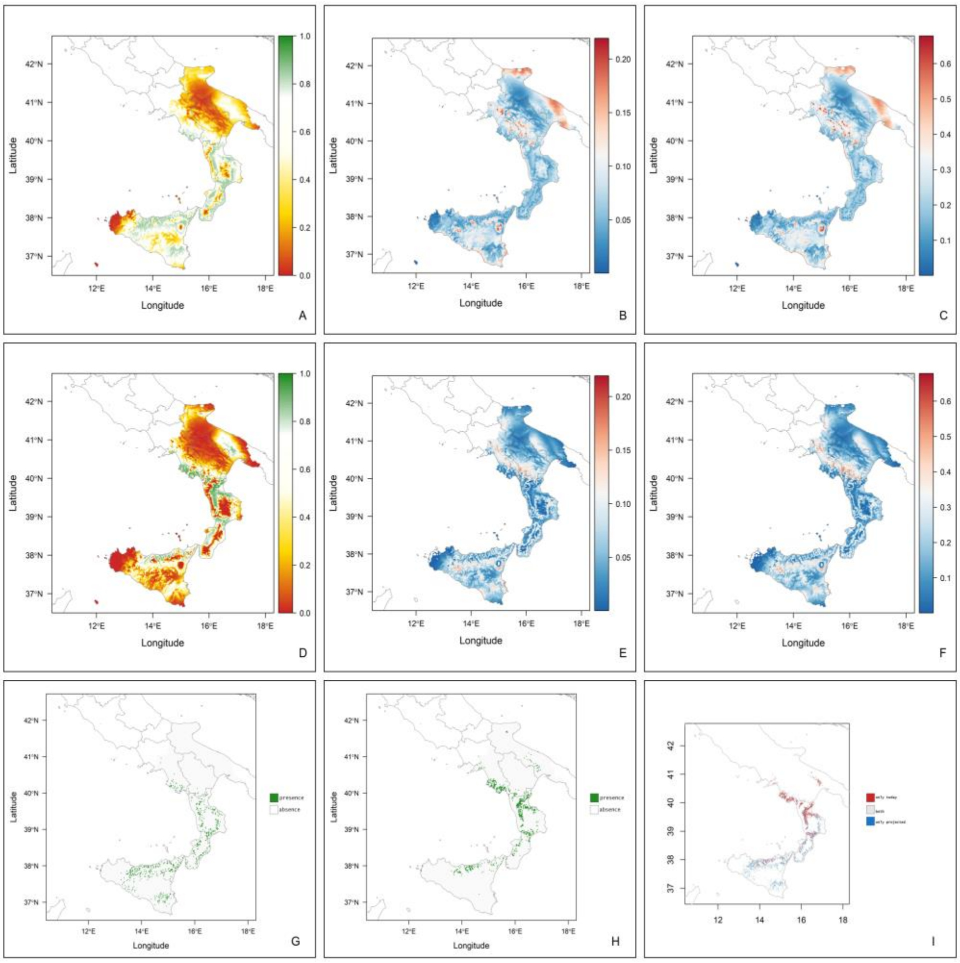

Fig. S.3.124-A) 'Projected' base output; B) 'Projected' uncertainty standard deviation; C) 'Projected' uncertainty range of values; D) 'Present' base output; E) 'Present' uncertainty standard deviation; F) 'Present' uncertainty range of values; G) 'Projected' binary distribution; H) 'Present' binary distribution; I) Range size variation.

Value	Present	Projected
AUC.train	0.799	0.791
AUC.test	0.799	0.731
AUC.diff	0.078	0.078
pROC	1.508	1.438
OR.p10	0.114	0.326
CBI	0.789	0.511
perc.stable	10.415	10.415
range.size	-4.361	-4.361

S.3.125 *Gypsophila arrostoi* KDE

Species: *Gypsophila arrostoi* Guss. subsp. *arrostoi*

Procedure: KDE

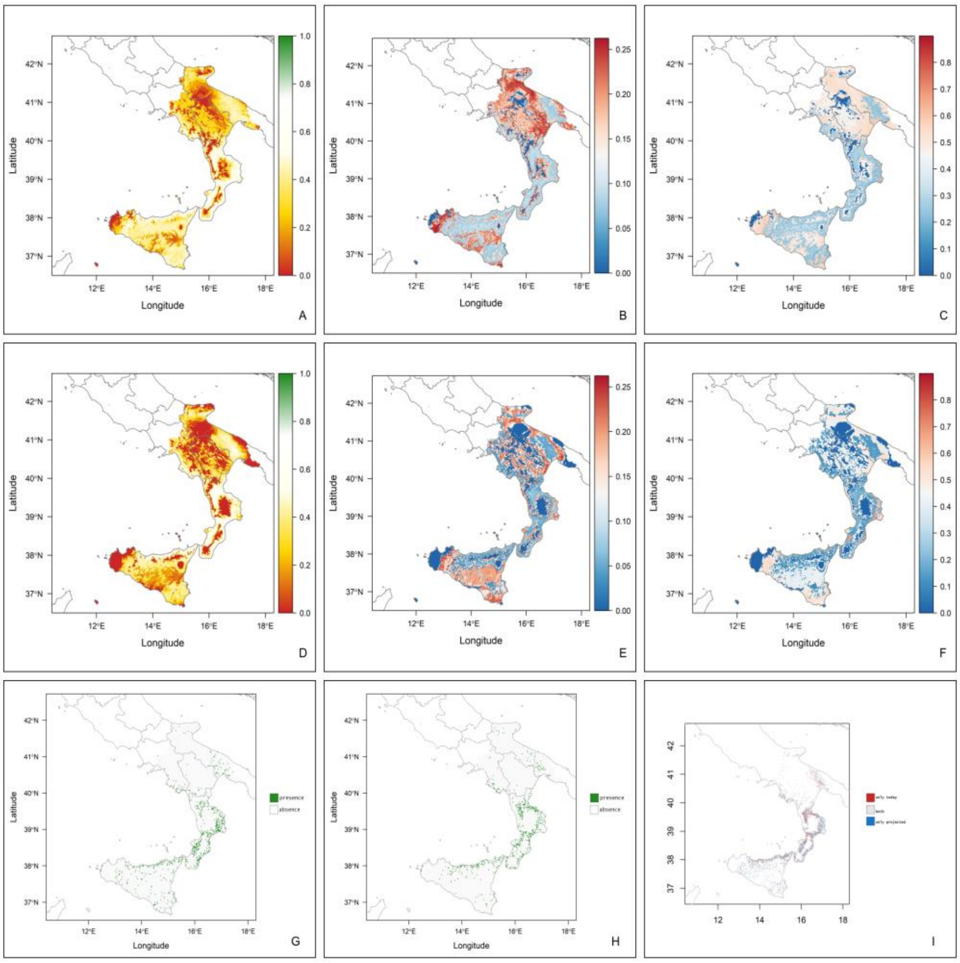

Fig. S.3.125-A) 'Projected' base output; B) 'Projected' uncertainty standard deviation; C) 'Projected' uncertainty range of values; D) 'Present' base output; E) 'Present' uncertainty standard deviation; F) 'Present' uncertainty range of values; G) 'Projected' binary distribution; H) 'Present' binary distribution; I) Range size variation.

Value	Present	Projected
AUC.train	0.791	0.814
AUC.test	0.791	0.719
AUC.diff	0.056	0.096
pROC	1.428	1.347
OR.p10	0.243	0.300
CBI	0.939	0.700
perc.stable	9.416	9.416
range.size	6.857	6.857

S.3.126 *Gypsophila arrostoi* CTA

Species: *Gypsophila arrostoi* Guss. subsp. *arrostoi*

Procedure: CTA

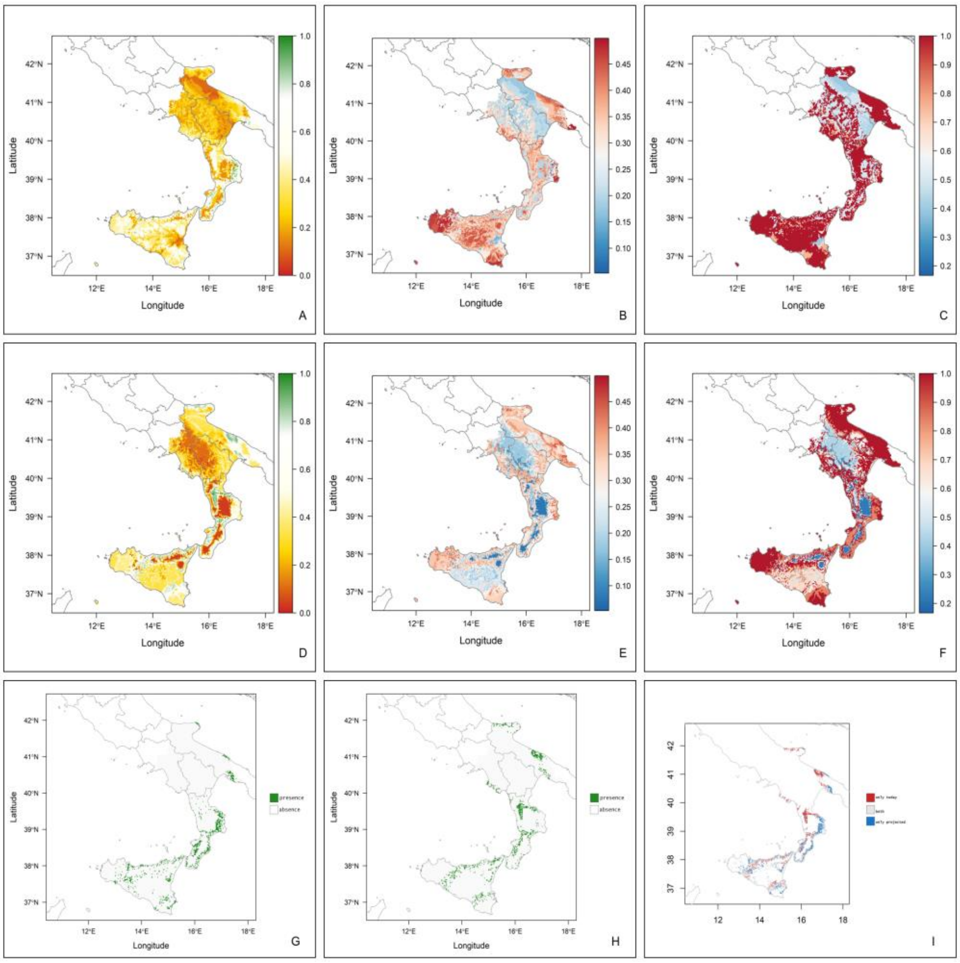

Fig. S.3.126-A) 'Projected' base output; B) 'Projected' uncertainty standard deviation; C) 'Projected' uncertainty range of values; D) 'Present' base output; E) 'Present' uncertainty standard deviation; F) 'Present' uncertainty range of values; G) 'Projected' binary distribution; H) 'Present' binary distribution; I) Range size variation.

Value	Present	Projected
AUC.train	0.804	0.925
AUC.test	0.804	0.721
AUC.diff	0.204	0.204
pROC	1.197	1.121
OR.p10	0.229	0.360
CBI	0.949	0.558
perc.stable	9.191	9.191
range.size	-1.841	-1.841

S.3.127 *Gypsophila arrostoi* FDA

Species: *Gypsophila arrostoi* Guss. subsp. *arrostoi*

Procedure: FDA

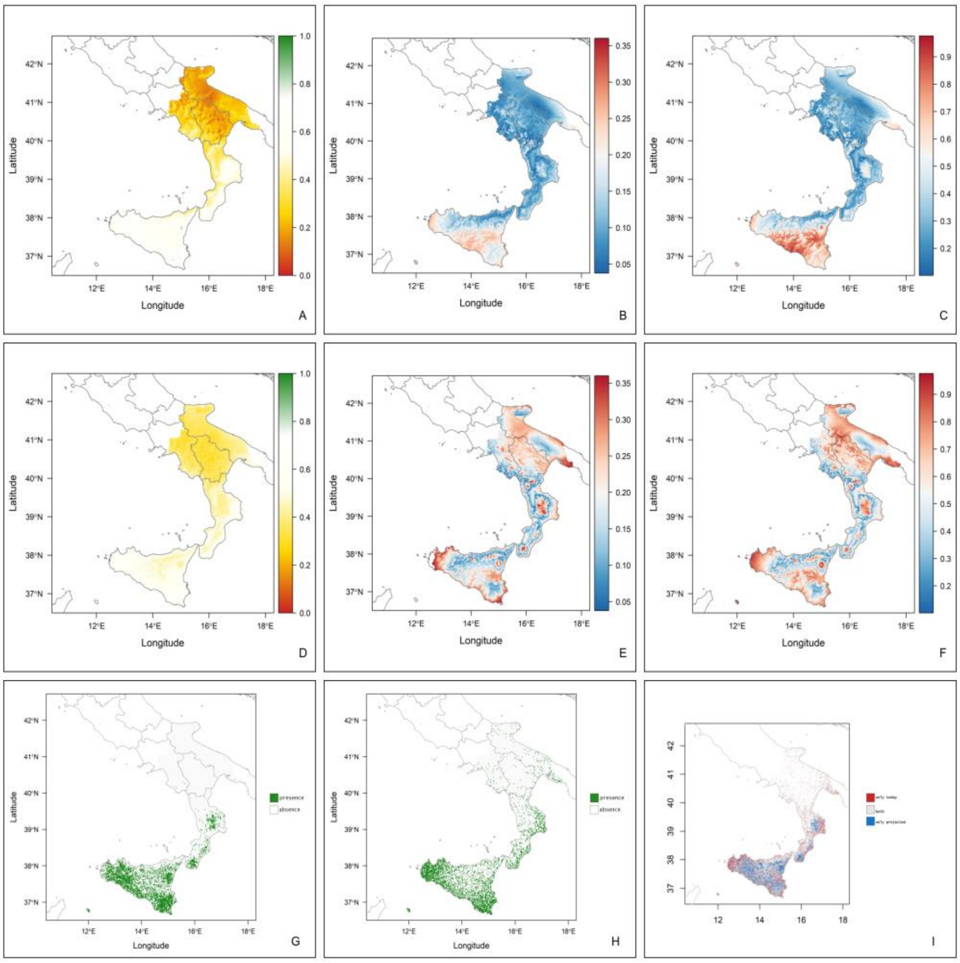

Fig. S.3.127-A) 'Projected' base output; B) 'Projected' uncertainty standard deviation; C) 'Projected' uncertainty range of values; D) 'Present' base output; E) 'Present' uncertainty standard deviation; F) 'Present' uncertainty range of values; G) 'Projected' binary distribution; H) 'Present' binary distribution; I) Range size variation.

Value	Present	Projected
AUC.train	0.598	0.631
AUC.test	0.598	0.646
AUC.diff	0.014	0.014
pROC	1.173	1.352
OR.p10	0.271	0.229
CBI	0.177	0.093
perc.stable	27.096	27.096
range.size	-1.081	-1.081

S.3.128 *Gypsophila arrostoi* GAM

Species: *Gypsophila arrostoi* Guss. subsp. *arrostoi*

Procedure: GAM

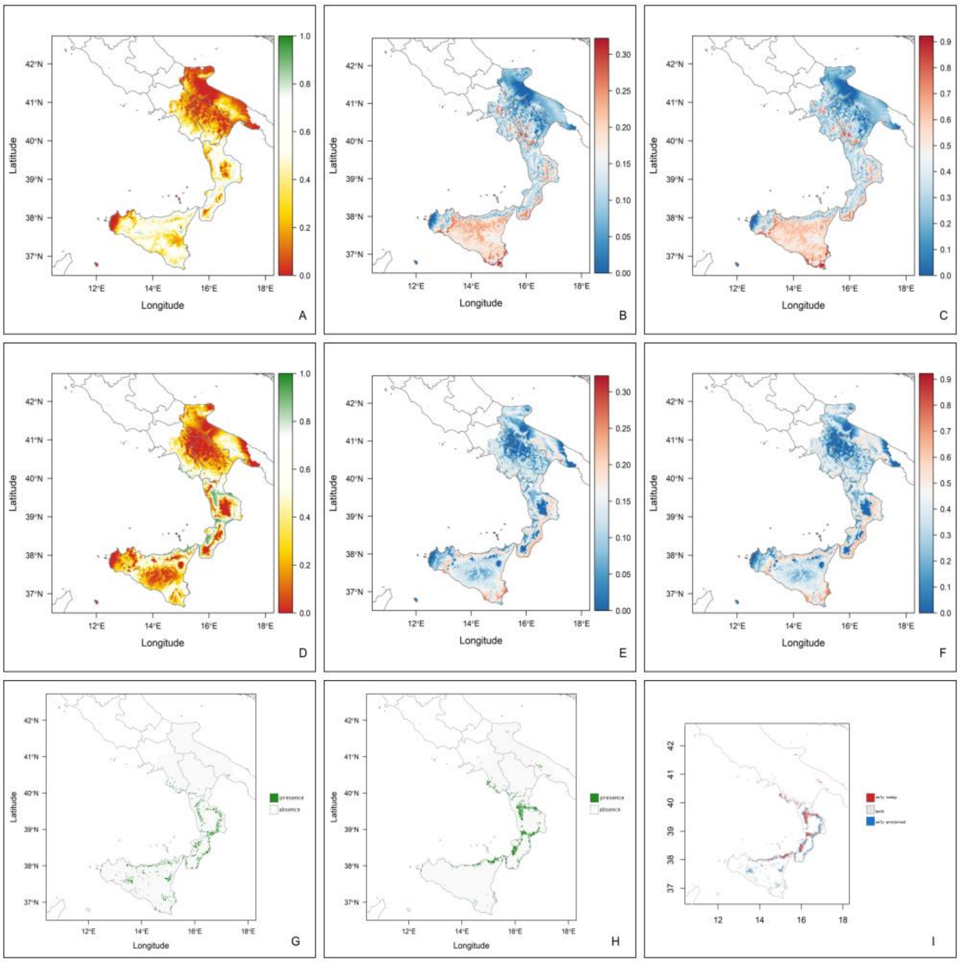

Fig. S.3.128-A) 'Projected' base output; B) 'Projected' uncertainty standard deviation; C) 'Projected' uncertainty range of values; D) 'Present' base output; E) 'Present' uncertainty standard deviation; F) 'Present' uncertainty range of values; G) 'Projected' binary distribution; H) 'Present' binary distribution; I) Range size variation.

Value	Present	Projected
AUC.train	0.758	0.820
AUC.test	0.758	0.638
AUC.diff	0.182	0.182
pROC	1.447	1.323
OR.p10	0.257	0.508
CBI	0.789	0.914
perc.stable	11.064	11.064
range.size	1.060	1.060

S.3.129 *Gypsophila arrostoi* GLM

Species: *Gypsophila arrostoi* Guss. subsp. *arrostoi*

Procedure: GLM

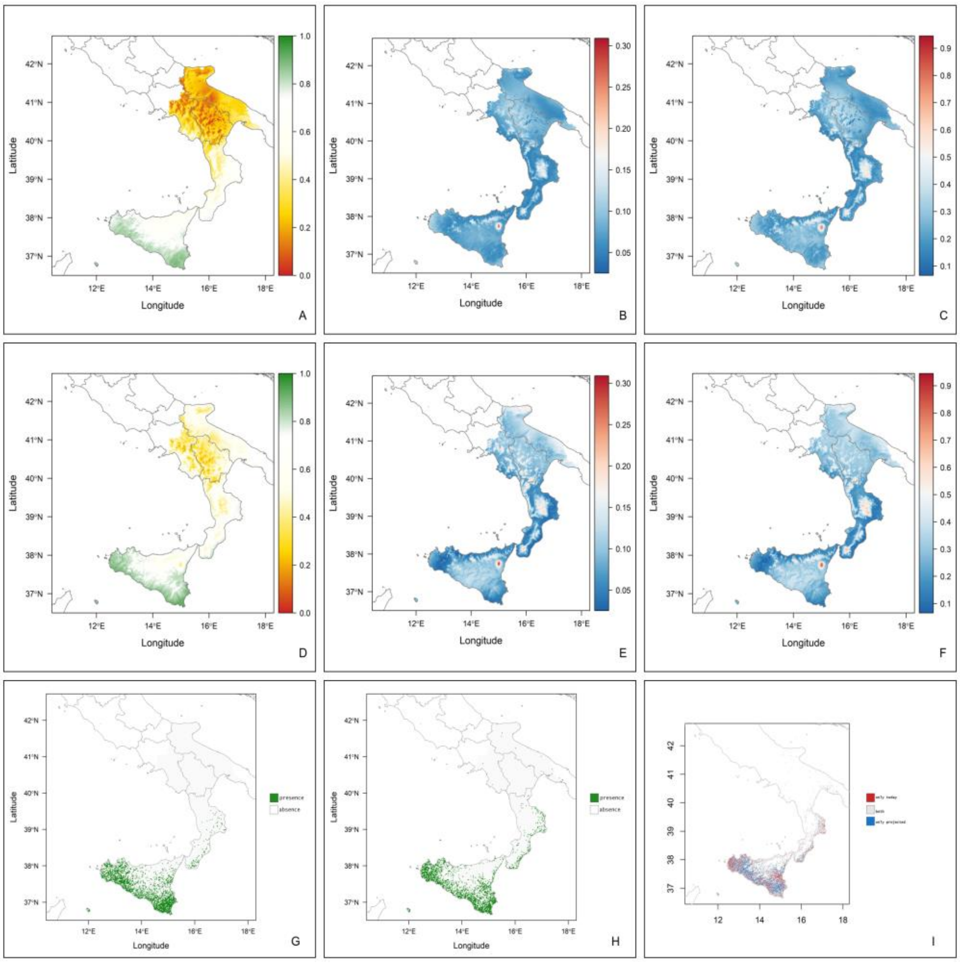

Fig. S.3.129-A) 'Projected' base output; B) 'Projected' uncertainty standard deviation; C) 'Projected' uncertainty range of values; D) 'Present' base output; E) 'Present' uncertainty standard deviation; F) 'Present' uncertainty range of values; G) 'Projected' binary distribution; H) 'Present' binary distribution; I) Range size variation.

Value	Present	Projected
AUC.train	0.518	0.657
AUC.test	0.518	0.607
AUC.diff	0.064	0.064
pROC	1.192	1.305
OR.p10	0.314	0.264
CBI	0.305	0.245
perc.stable	31.887	31.887
range.size	-0.150	-0.150

S.3.130 *Gypsophila arrostoi* MARS

Species: *Gypsophila arrostoi* Guss. subsp. *arrostoi*

Procedure: MARS

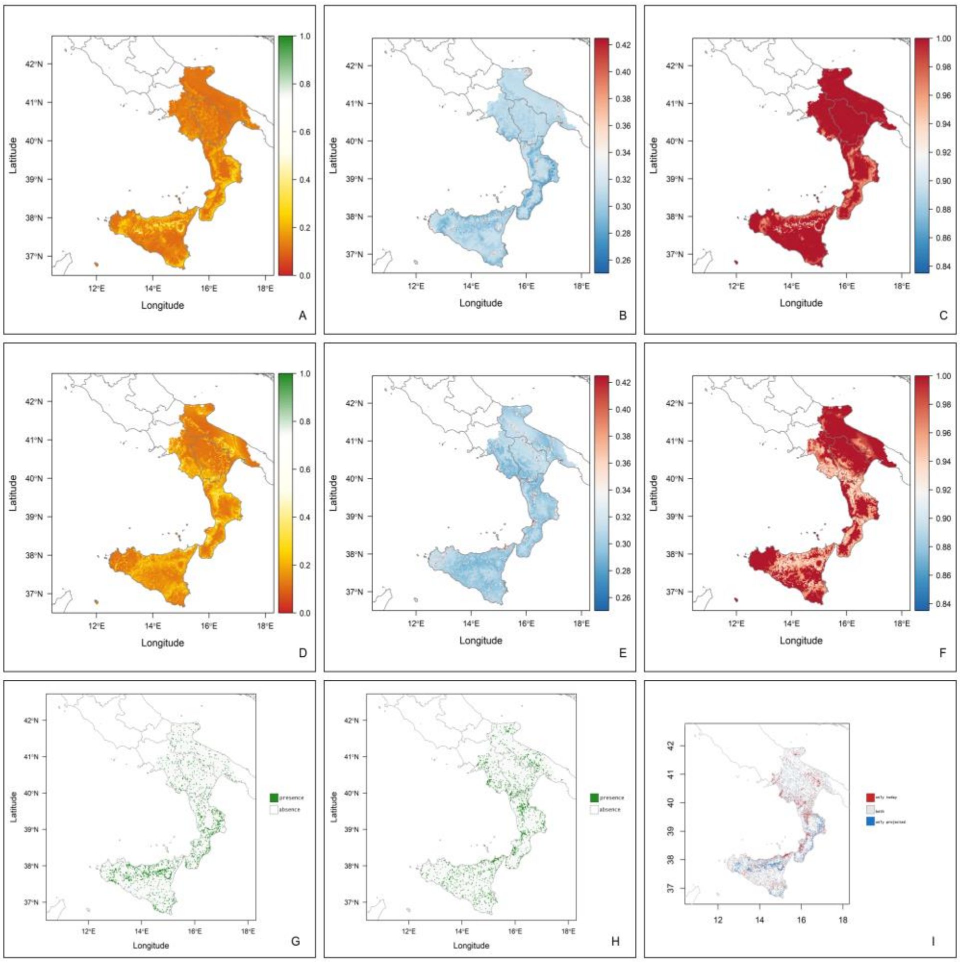

Fig. S.3.130-A) 'Projected' base output; B) 'Projected' uncertainty standard deviation; C) 'Projected' uncertainty range of values; D) 'Present' base output; E) 'Present' uncertainty standard deviation; F) 'Present' uncertainty range of values; G) 'Projected' binary distribution; H) 'Present' binary distribution; I) Range size variation.

Value	Present	Projected
AUC.train	0.702	0.824
AUC.test	0.702	0.666
AUC.diff	0.158	0.158
pROC	1.379	1.256
OR.p10	0.294	0.271
CBI	0.869	0.211
perc.stable	5.999	5.999
range.size	2.168	2.168

S.3.131 *Gypsophila arrostoi* RF

Species: *Gypsophila arrostoi* Guss. subsp. *arrostoi*

Procedure: RF

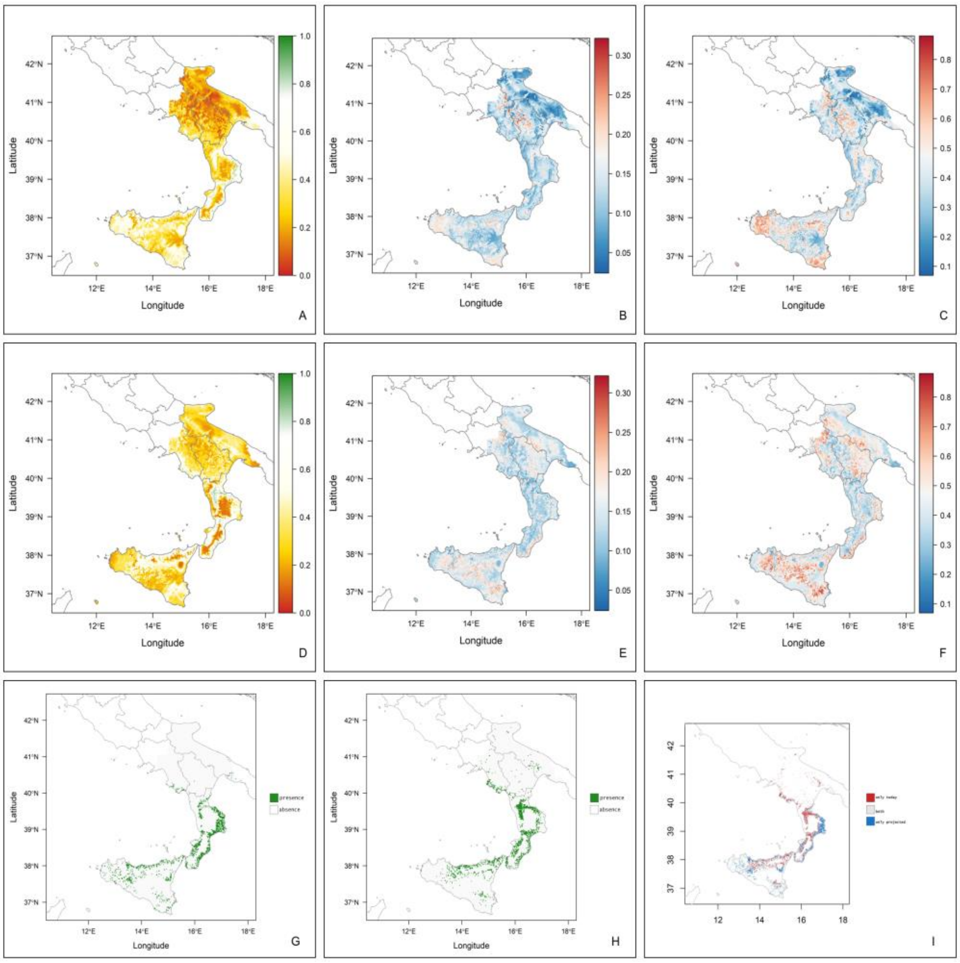

Fig. S.3.131-A) 'Projected' base output; B) 'Projected' uncertainty standard deviation; C) 'Projected' uncertainty range of values; D) 'Present' base output; E) 'Present' uncertainty standard deviation; F) 'Present' uncertainty range of values; G) 'Projected' binary distribution; H) 'Present' binary distribution; I) Range size variation.

Value	Present	Projected
AUC.train	0.973	1.000
AUC.test	0.973	0.938
AUC.diff	0.062	0.062
pROC	1.445	1.431
OR.p10	0.843	0.848
CBI	0.970	0.870
perc.stable	23.344	23.344
range.size	0.084	0.084

S.3.132 *Gypsophila arrostoi* SVM

Species: *Gypsophila arrostoi* Guss. subsp. *arrostoi*

Procedure: SVM

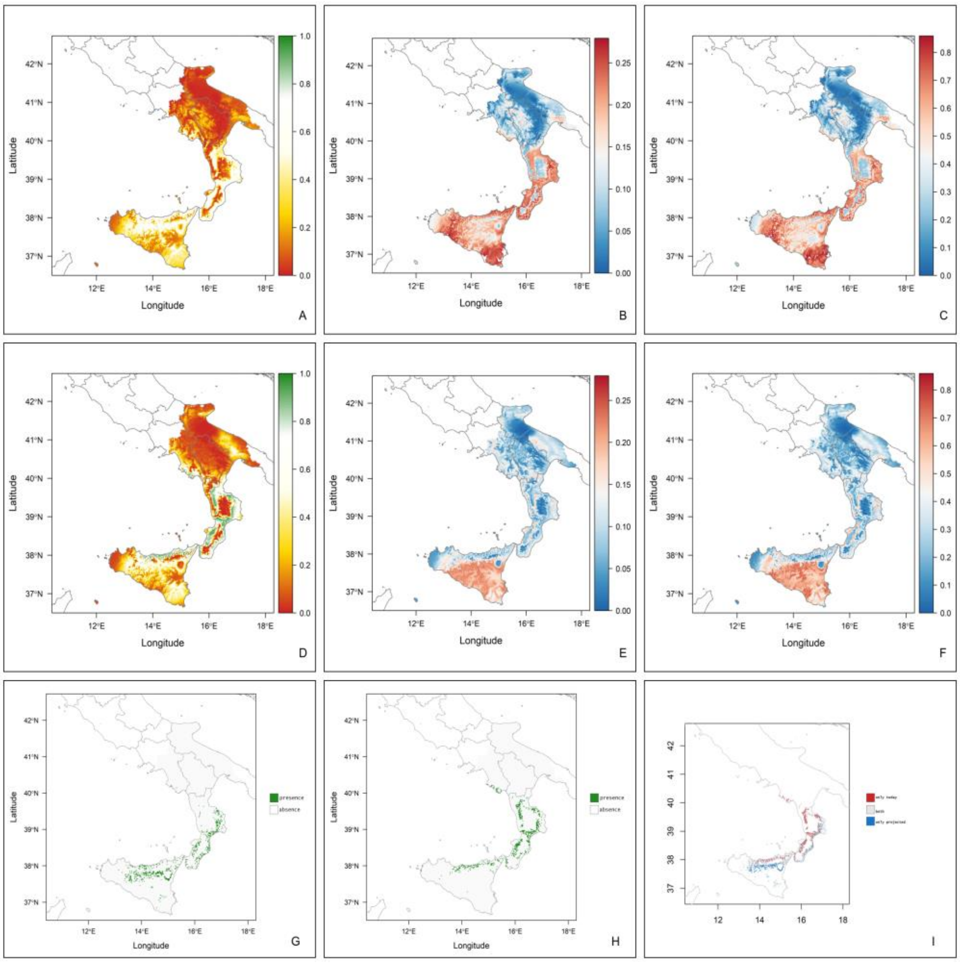

Fig. S.3.132-A) 'Projected' base output; B) 'Projected' uncertainty standard deviation; C) 'Projected' uncertainty range of values; D) 'Present' base output; E) 'Present' uncertainty standard deviation; F) 'Present' uncertainty range of values; G) 'Projected' binary distribution; H) 'Present' binary distribution; I) Range size variation.

Value	Present	Projected
AUC.train	0.796	0.916
AUC.test	0.796	0.873
AUC.diff	0.052	0.052
pROC	1.340	1.552
OR.p10	0.343	0.248
CBI	0.903	0.757
perc.stable	16.087	16.087
range.size	6.857	6.857

S.3.133 *Helleborus viridis* Biomod2

Species: *Helleborus viridis* L. subsp. bocconei (Ten.) Peruzzi

Procedure: Biomod2

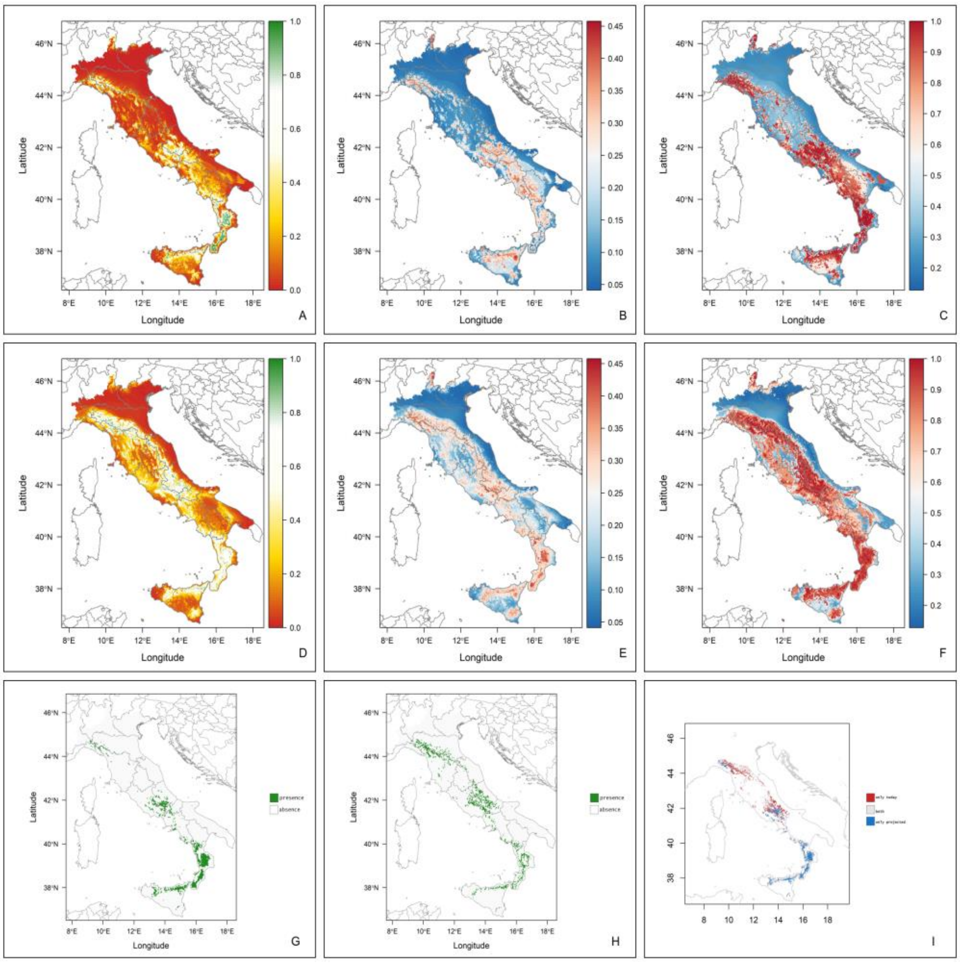

Fig. S.3.133-A) 'Projected' base output; B) 'Projected' uncertainty standard deviation; C) 'Projected' uncertainty range of values; D) 'Present' base output; E) 'Present' uncertainty standard deviation; F) 'Present' uncertainty range of values; G) 'Projected' binary distribution; H) 'Present' binary distribution; I) Range size variation.

Value	Present	Projected
AUC.train	0.763	0.983
AUC.test	0.763	0.881
AUC.diff	0.102	0.102
pROC	1.385	1.494
OR.p10	0.891	0.373
CBI	0.963	0.908
perc.stable	20.809	20.809
range.size	2.631	2.631

S.3.134 *Helleborus viridis* Bioclim

Species: *Helleborus viridis* L. subsp. bocconei (Ten.) Peruzzi

Procedure: Bioclim

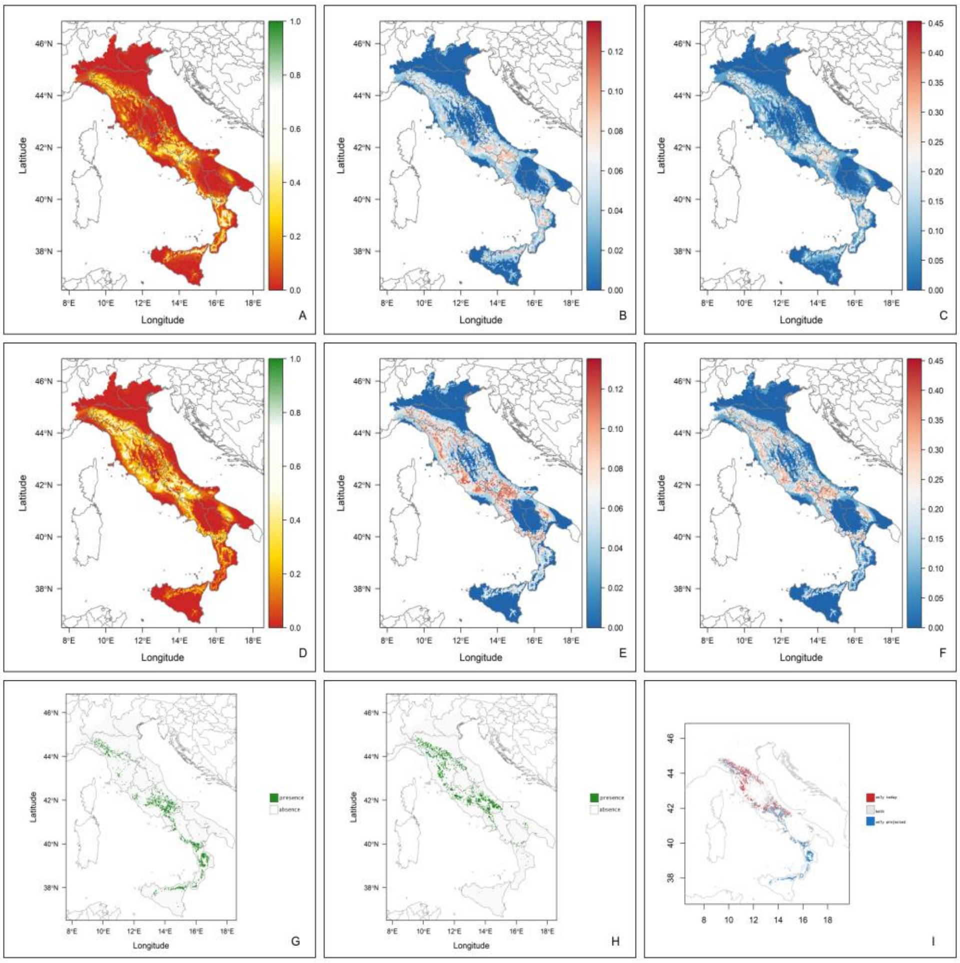

Fig. S.3.134-A) 'Projected' base output; B) 'Projected' uncertainty standard deviation; C) 'Projected' uncertainty range of values; D) 'Present' base output; E) 'Present' uncertainty standard deviation; F) 'Present' uncertainty range of values; G) 'Projected' binary distribution; H) 'Present' binary distribution; I) Range size variation.

Value	Present	Projected
AUC.train	0.694	0.852
AUC.test	0.694	0.785
AUC.diff	0.068	0.068
pROC	1.141	1.236
OR.p10	0.218	0.127
CBI	0.212	0.400
perc.stable	16.510	16.510
range.size	2.404	2.404

S.3.135 *Helleborus viridis* Domain

Species: *Helleborus viridis* L. subsp. bocconei (Ten.) Peruzzi

Procedure: Domain

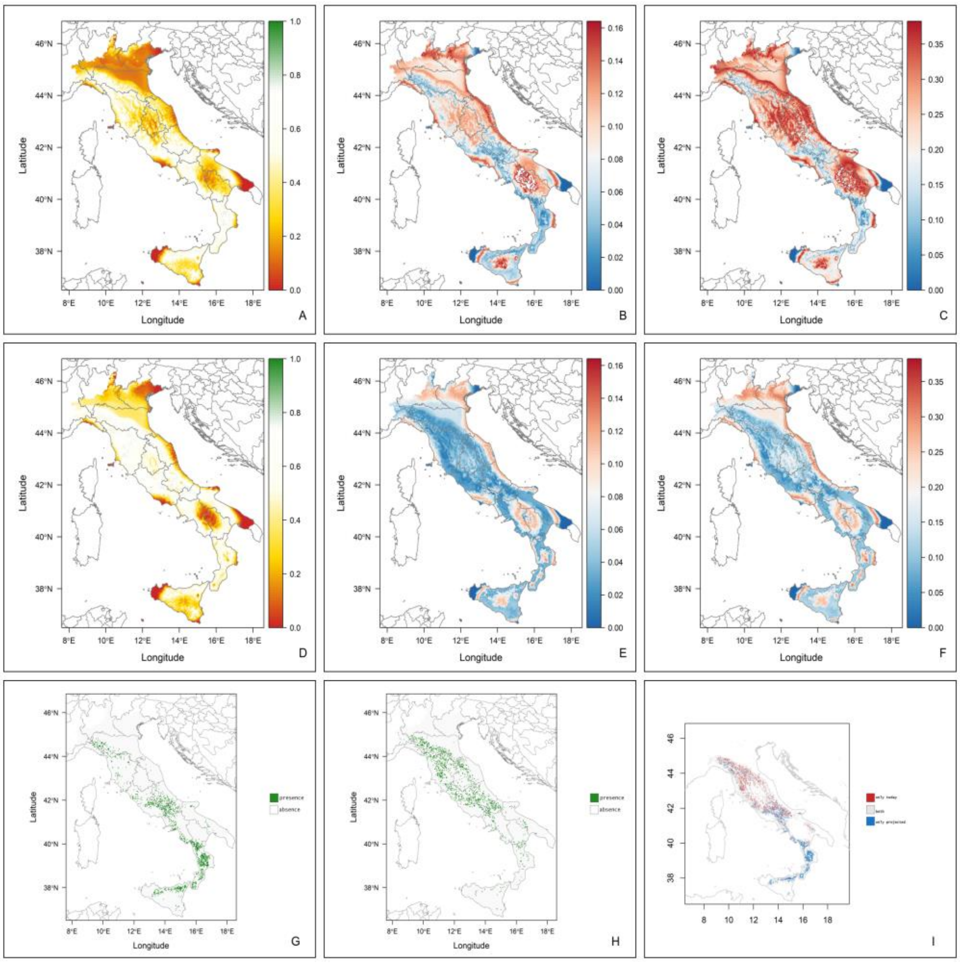

Fig. S.3.135-A) 'Projected' base output; B) 'Projected' uncertainty standard deviation; C) 'Projected' uncertainty range of values; D) 'Present' base output; E) 'Present' uncertainty standard deviation; F) 'Present' uncertainty range of values; G) 'Projected' binary distribution; H) 'Present' binary distribution; I) Range size variation.

Value	Present	Projected
AUC.train	0.702	0.861
AUC.test	0.702	0.819
AUC.diff	0.066	0.066
pROC	1.297	1.440
OR.p10	0.264	0.264
CBI	0.917	0.962
perc.stable	8.350	8.350
range.size	0.141	0.141

S.3.136 *Helleborus viridis* Maxent

Species: *Helleborus viridis* L. subsp. bocconei (Ten.) Peruzzi

Procedure: Maxent

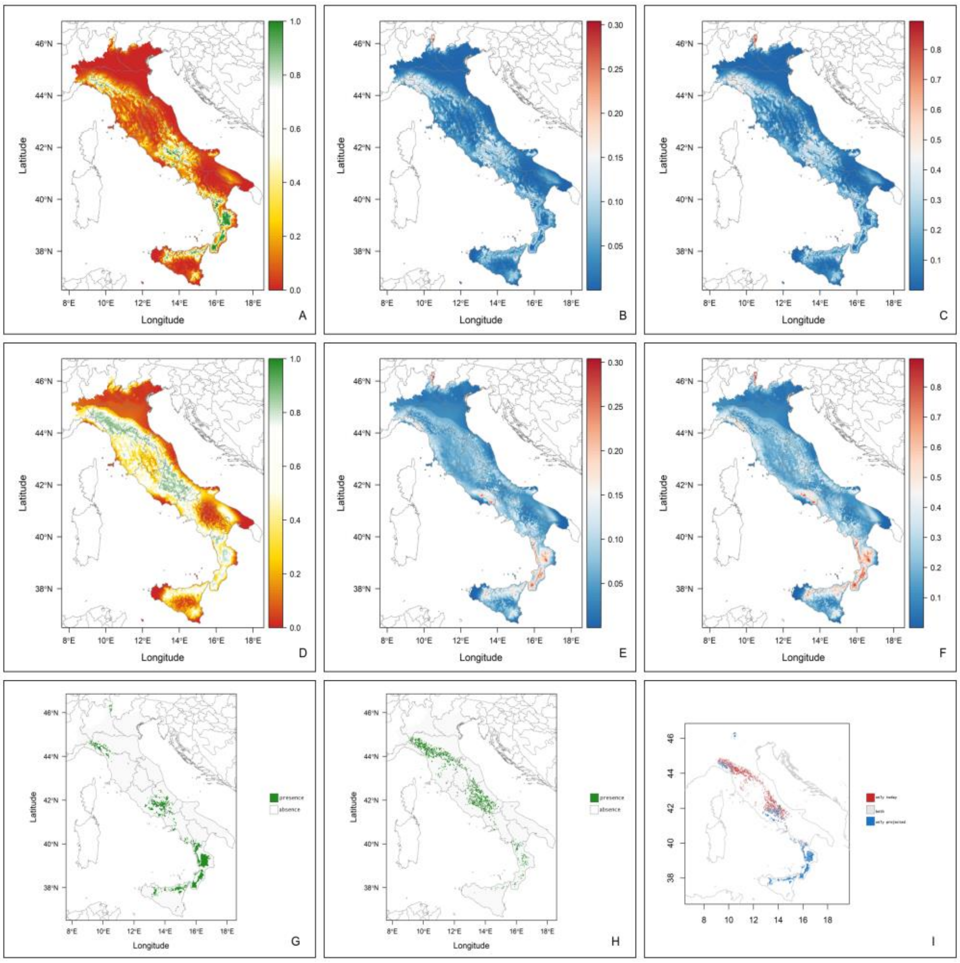

Fig. S.3.136-A) 'Projected' base output; B) 'Projected' uncertainty standard deviation; C) 'Projected' uncertainty range of values; D) 'Present' base output; E) 'Present' uncertainty standard deviation; F) 'Present' uncertainty range of values; G) 'Projected' binary distribution; H) 'Present' binary distribution; I) Range size variation.

Value	Present	Projected
AUC.train	0.737	0.905
AUC.test	0.737	0.872
AUC.diff	0.037	0.037
pROC	1.347	1.527
OR.p10	0.255	0.245
CBI	0.958	0.809
perc.stable	15.326	15.326
range.size	1.222	1.222

S.3.137 *Helleborus viridis* KDE

Species: *Helleborus viridis* L. subsp. bocconei (Ten.) Peruzzi

Procedure: KDE

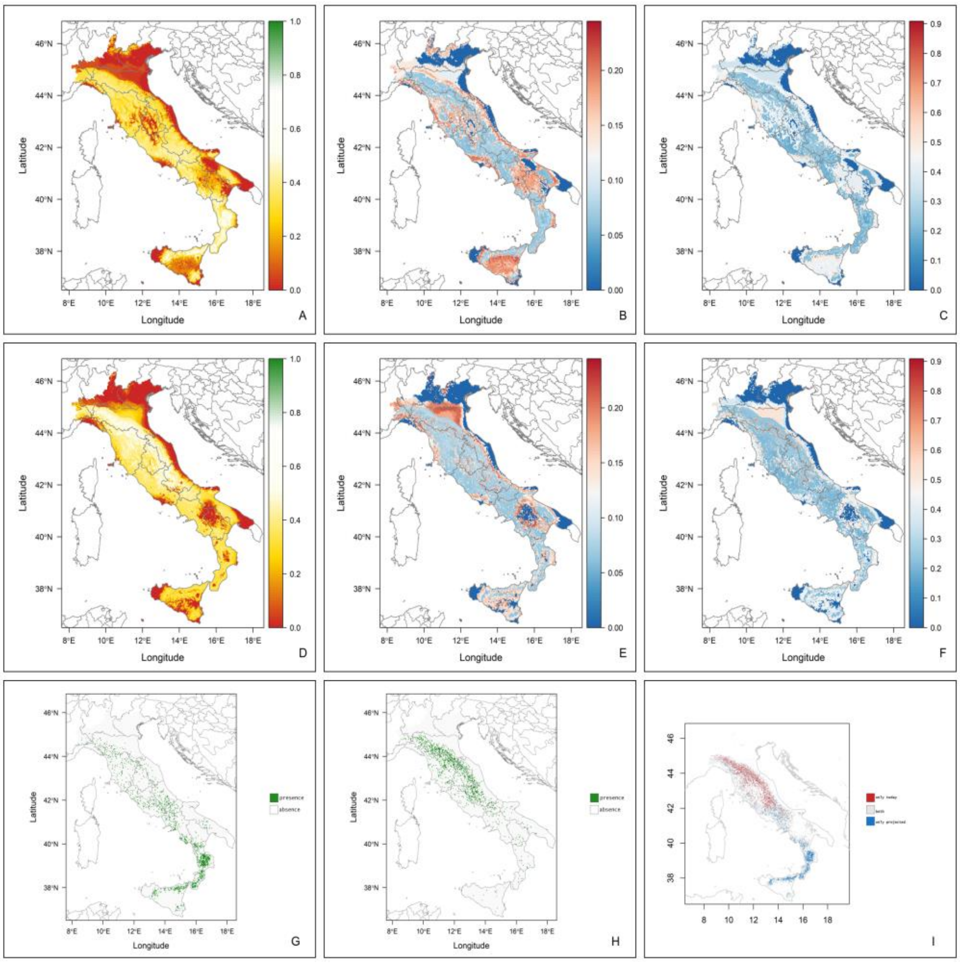

Fig. S.3.137-A) 'Projected' base output; B) 'Projected' uncertainty standard deviation; C) 'Projected' uncertainty range of values; D) 'Present' base output; E) 'Present' uncertainty standard deviation; F) 'Present' uncertainty range of values; G) 'Projected' binary distribution; H) 'Present' binary distribution; I) Range size variation.

Value	Present	Projected
AUC.train	0.733	0.881
AUC.test	0.733	0.795
AUC.diff	0.059	0.090
pROC	1.277	1.314
OR.p10	0.191	0.309
CBI	0.966	0.976
perc.stable	4.479	4.479
range.size	1.637	1.637

S.3.138 *Helleborus viridis* CTA

Species: *Helleborus viridis* L. subsp. bocconei (Ten.) Peruzzi

Procedure: CTA

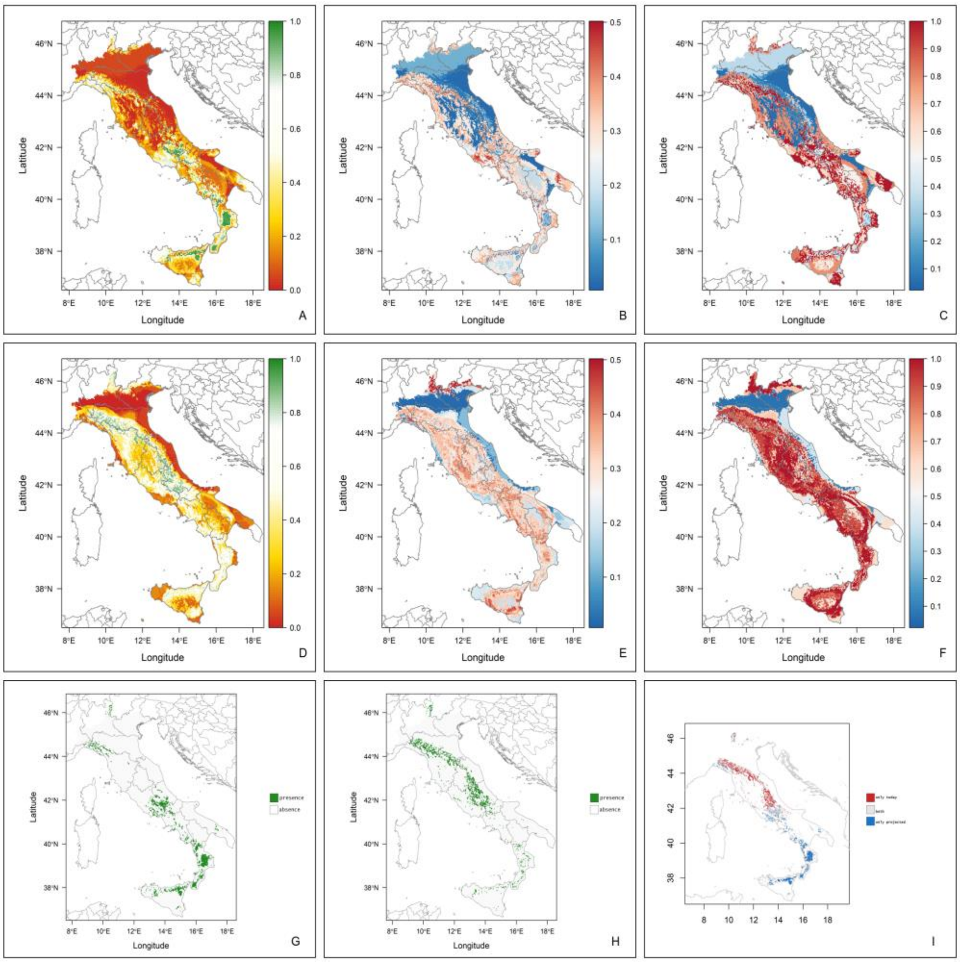

Fig. S.3.138-A) 'Projected' base output; B) 'Projected' uncertainty standard deviation; C) 'Projected' uncertainty range of values; D) 'Present' base output; E) 'Present' uncertainty standard deviation; F) 'Present' uncertainty range of values; G) 'Projected' binary distribution; H) 'Present' binary distribution; I) Range size variation.

Value	Present	Projected
AUC.train	0.774	0.949
AUC.test	0.774	0.826
AUC.diff	0.123	0.123
pROC	1.129	1.143
OR.p10	0.345	0.255
CBI	0.955	0.796
perc.stable	15.480	15.480
range.size	-2.395	-2.395

S.3.139 *Helleborus viridis* FDA

Species: *Helleborus viridis* L. subsp. bocconei (Ten.) Peruzzi

Procedure: FDA

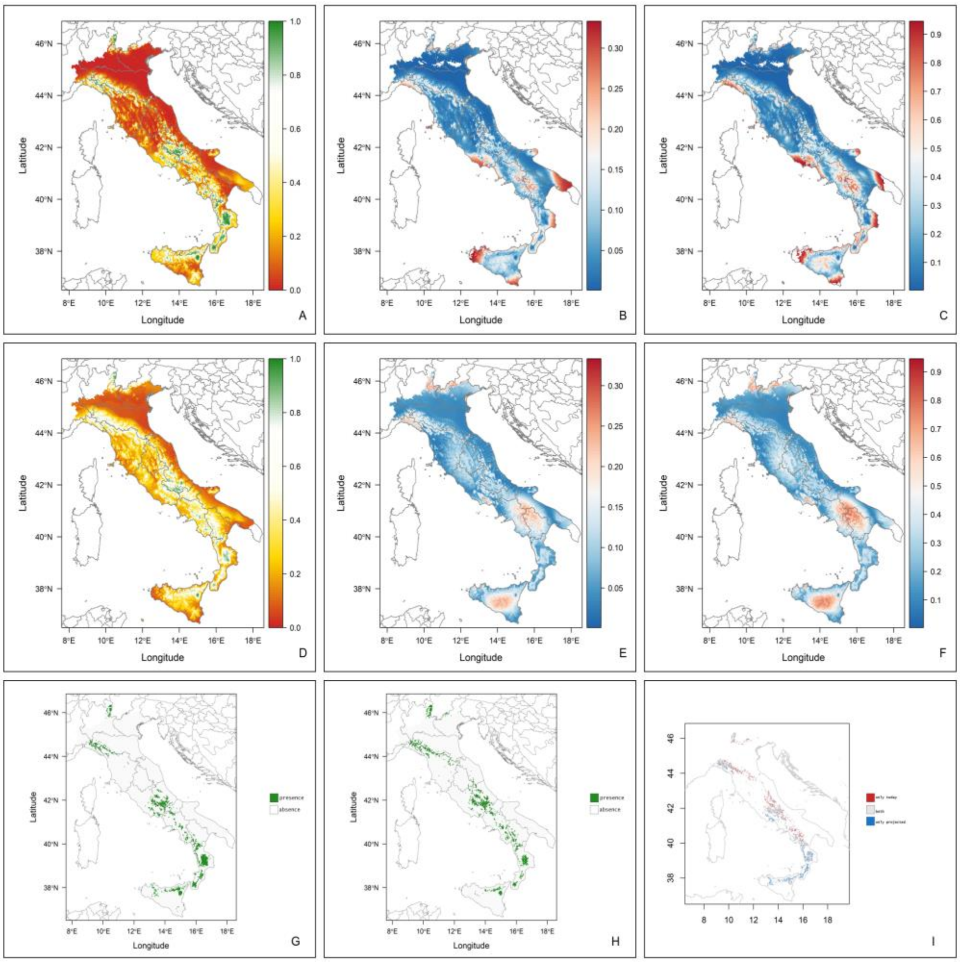

Fig. S.3.139-A) 'Projected' base output; B) 'Projected' uncertainty standard deviation; C) 'Projected' uncertainty range of values; D) 'Present' base output; E) 'Present' uncertainty standard deviation; F) 'Present' uncertainty range of values; G) 'Projected' binary distribution; H) 'Present' binary distribution; I) Range size variation.

Value	Present	Projected
AUC.train	0.753	0.908
AUC.test	0.753	0.874
AUC.diff	0.042	0.042
pROC	1.344	1.511
OR.p10	0.136	0.273
CBI	0.660	0.847
perc.stable	47.255	47.255
range.size	0.365	0.365

S.3.140 *Helleborus viridis* GAM

Species: *Helleborus viridis* L. subsp. bocconei (Ten.) Peruzzi

Procedure: GAM

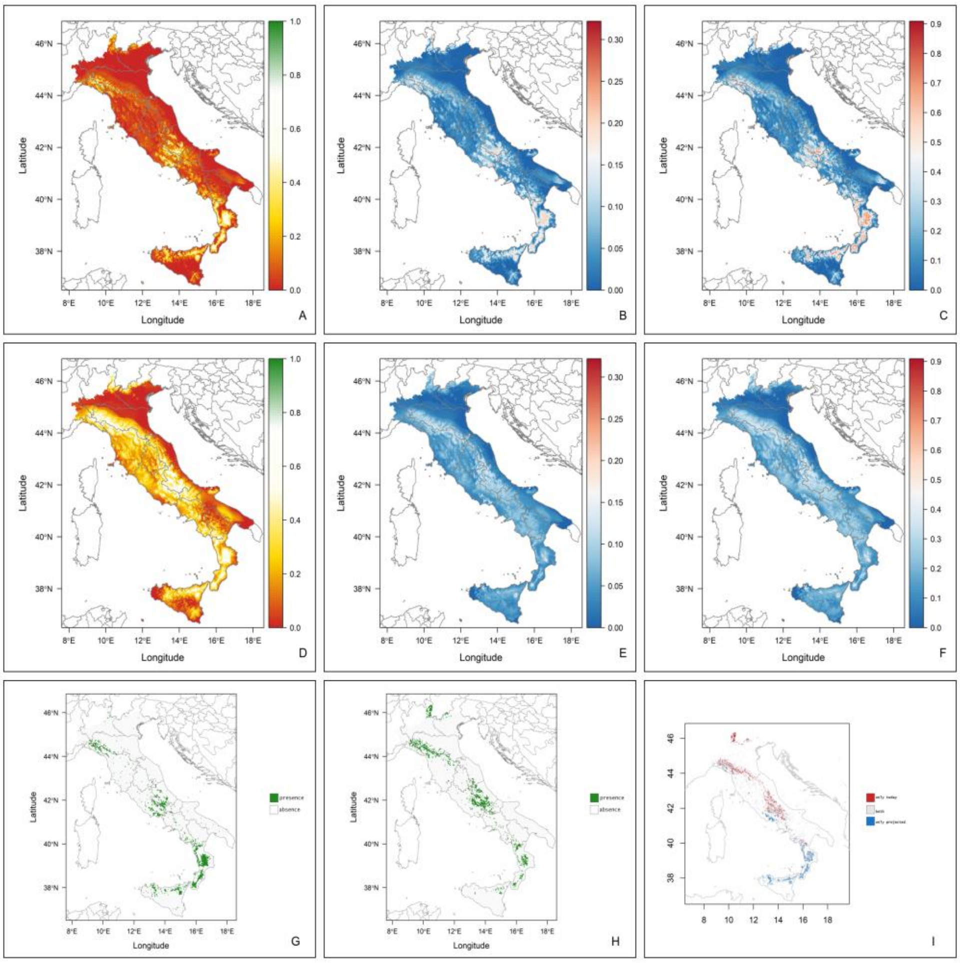

Fig. S.3.140-A) 'Projected' base output; B) 'Projected' uncertainty standard deviation; C) 'Projected' uncertainty range of values; D) 'Present' base output; E) 'Present' uncertainty standard deviation; F) 'Present' uncertainty range of values; G) 'Projected' binary distribution; H) 'Present' binary distribution; I) Range size variation.

Value	Present	Projected
AUC.train	0.778	0.894
AUC.test	0.778	0.855
AUC.diff	0.054	0.054
pROC	1.413	1.455
OR.p10	0.136	0.209
CBI	0.549	0.410
perc.stable	30.624	30.624
range.size	-1.138	-1.138

S.3.141 *Helleborus viridis* GLM

Species: *Helleborus viridis* L. subsp. bocconei (Ten.) Peruzzi

Procedure: GLM

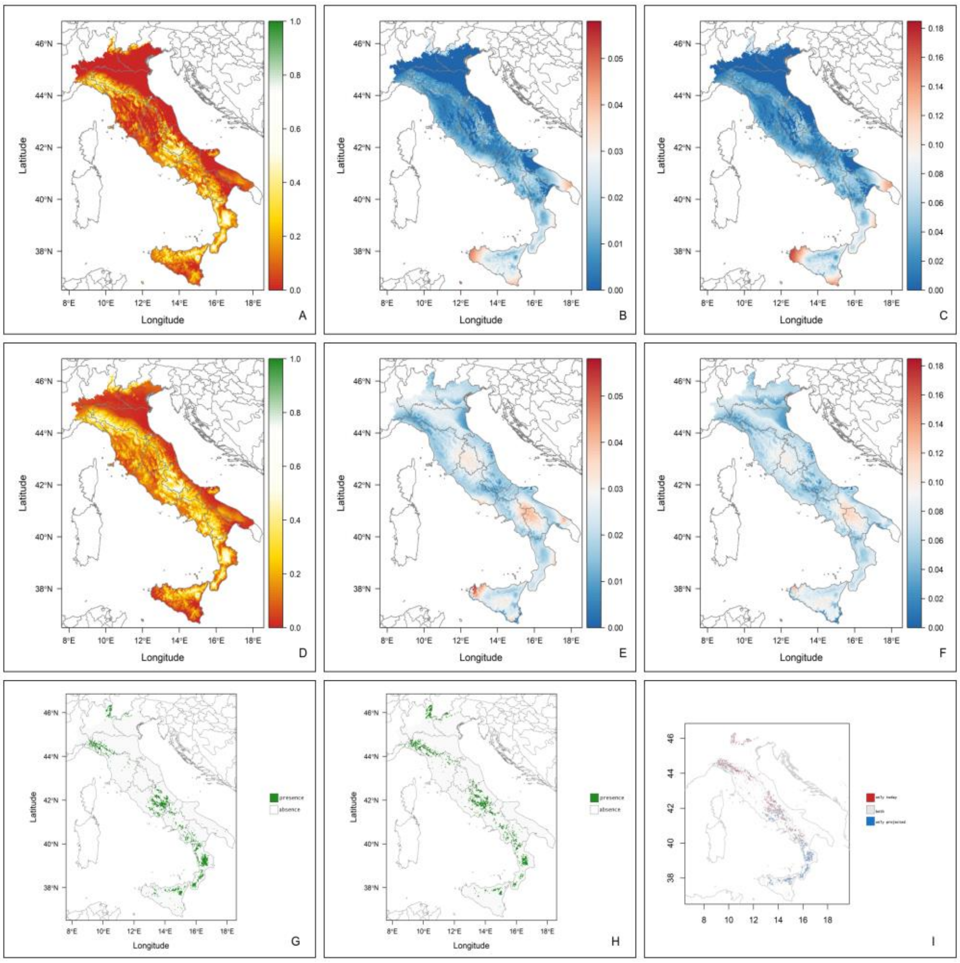

Fig. S.3.141-A) 'Projected' base output; B) 'Projected' uncertainty standard deviation; C) 'Projected' uncertainty range of values; D) 'Present' base output; E) 'Present' uncertainty standard deviation; F) 'Present' uncertainty range of values; G) 'Projected' binary distribution; H) 'Present' binary distribution; I) Range size variation.

Value	Present	Projected
AUC.train	0.750	0.894
AUC.test	0.750	0.885
AUC.diff	0.013	0.013
pROC	1.379	1.586
OR.p10	0.182	0.109
CBI	0.319	0.352
perc.stable	38.371	38.371
range.size	-0.265	-0.265

S.3.142 *Helleborus viridis* MARS

Species: *Helleborus viridis* L. subsp. bocconei (Ten.) Peruzzi

Procedure: MARS

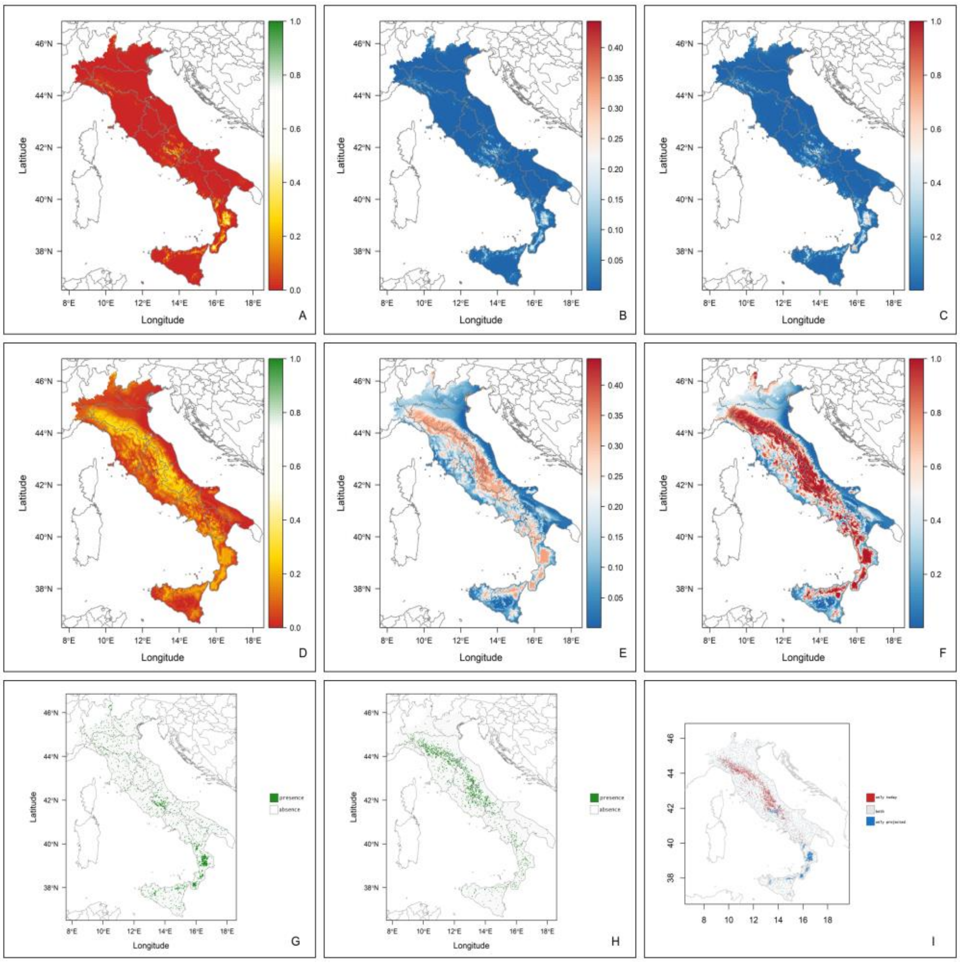

Fig. S.3.142-A) 'Projected' base output; B) 'Projected' uncertainty standard deviation; C) 'Projected' uncertainty range of values; D) 'Present' base output; E) 'Present' uncertainty standard deviation; F) 'Present' uncertainty range of values; G) 'Projected' binary distribution; H) 'Present' binary distribution; I) Range size variation.

Value	Present	Projected
AUC.train	0.757	0.861
AUC.test	0.757	0.814
AUC.diff	0.056	0.056
pROC	1.327	1.261
OR.p10	0.173	0.145
CBI	0.928	0.160
perc.stable	6.075	6.075
range.size	-0.121	-0.121

S.3.143 *Helleborus viridis* RF

Species: *Helleborus viridis* L. subsp. bocconei (Ten.) Peruzzi

Procedure: RF

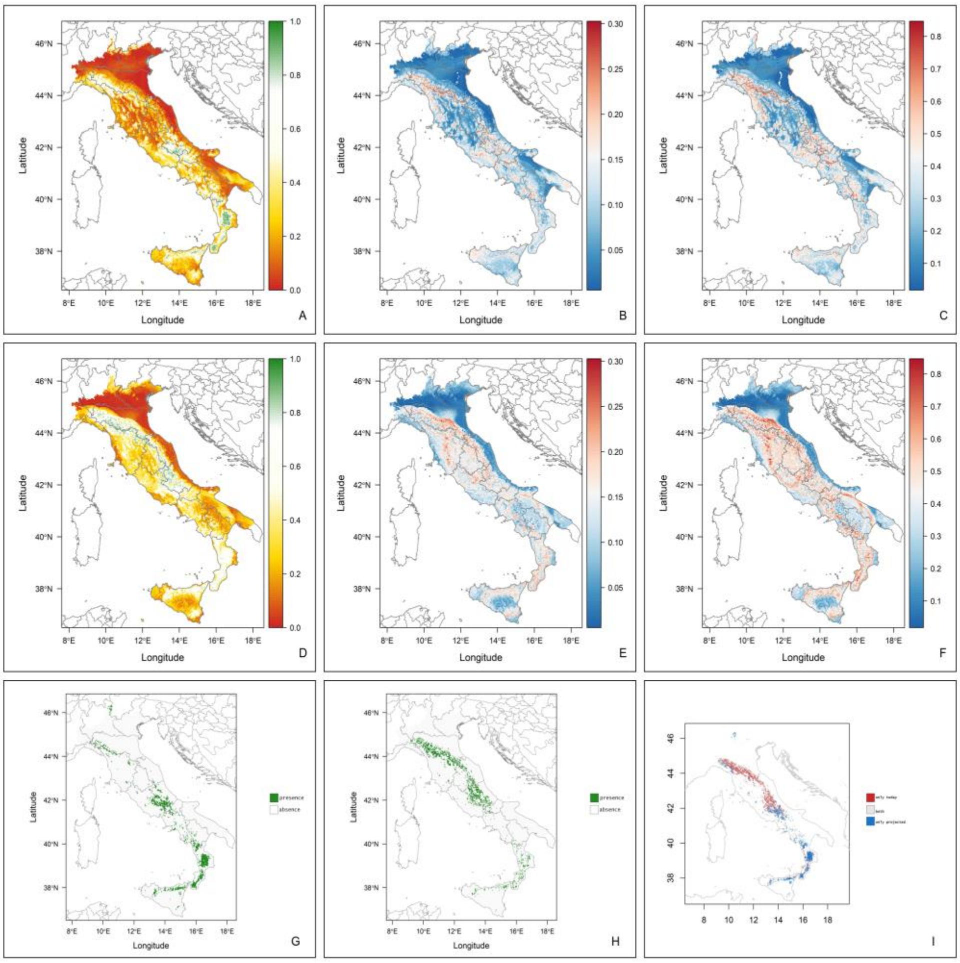

Fig. S.3.143-A) 'Projected' base output; B) 'Projected' uncertainty standard deviation; C) 'Projected' uncertainty range of values; D) 'Present' base output; E) 'Present' uncertainty standard deviation; F) 'Present' uncertainty range of values; G) 'Projected' binary distribution; H) 'Present' binary distribution; I) Range size variation.

Value	Present	Projected
AUC.train	0.938	1.000
AUC.test	0.938	0.973
AUC.diff	0.027	0.027
pROC	1.302	1.523
OR.p10	0.764	0.564
CBI	0.991	0.667
perc.stable	16.732	16.732
range.size	-1.617	-1.617

S.3.144 *Helleborus viridis* SVM

Species: *Helleborus viridis* L. subsp. bocconei (Ten.) Peruzzi

Procedure: SVM

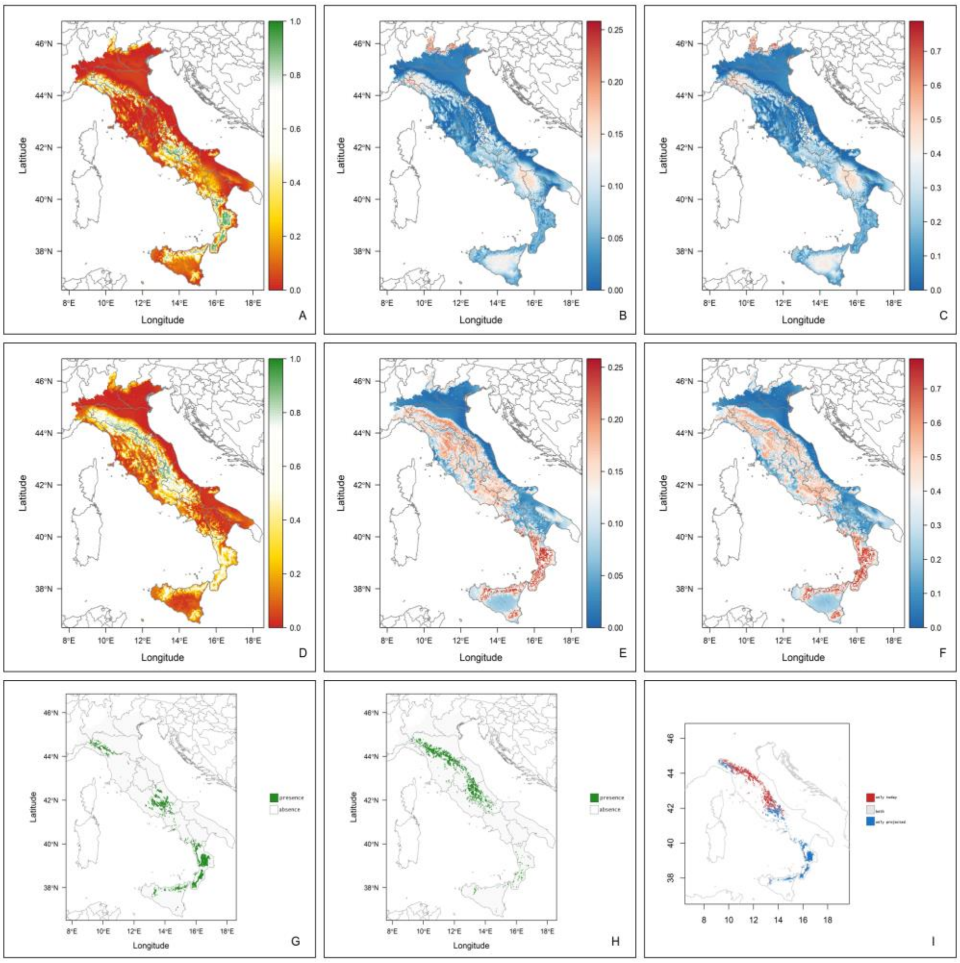

Fig. S.3.144-A) 'Projected' base output; B) 'Projected' uncertainty standard deviation; C) 'Projected' uncertainty range of values; D) 'Present' base output; E) 'Present' uncertainty standard deviation; F) 'Present' uncertainty range of values; G) 'Projected' binary distribution; H) 'Present' binary distribution; I) Range size variation.

Value	Present	Projected
AUC.train	0.833	0.935
AUC.test	0.833	0.893
AUC.diff	0.046	0.046
pROC	1.357	1.476
OR.p10	0.164	0.227
CBI	0.945	0.823
perc.stable	10.446	10.446
range.size	-1.588	-1.588

S.3.145 *Klasea flavescens* Biomod2

Species: *Klasea flavescens* (L.) Holub subsp. *cichoracea* (L.) Greuter & Wagenitz

Procedure: Biomod2

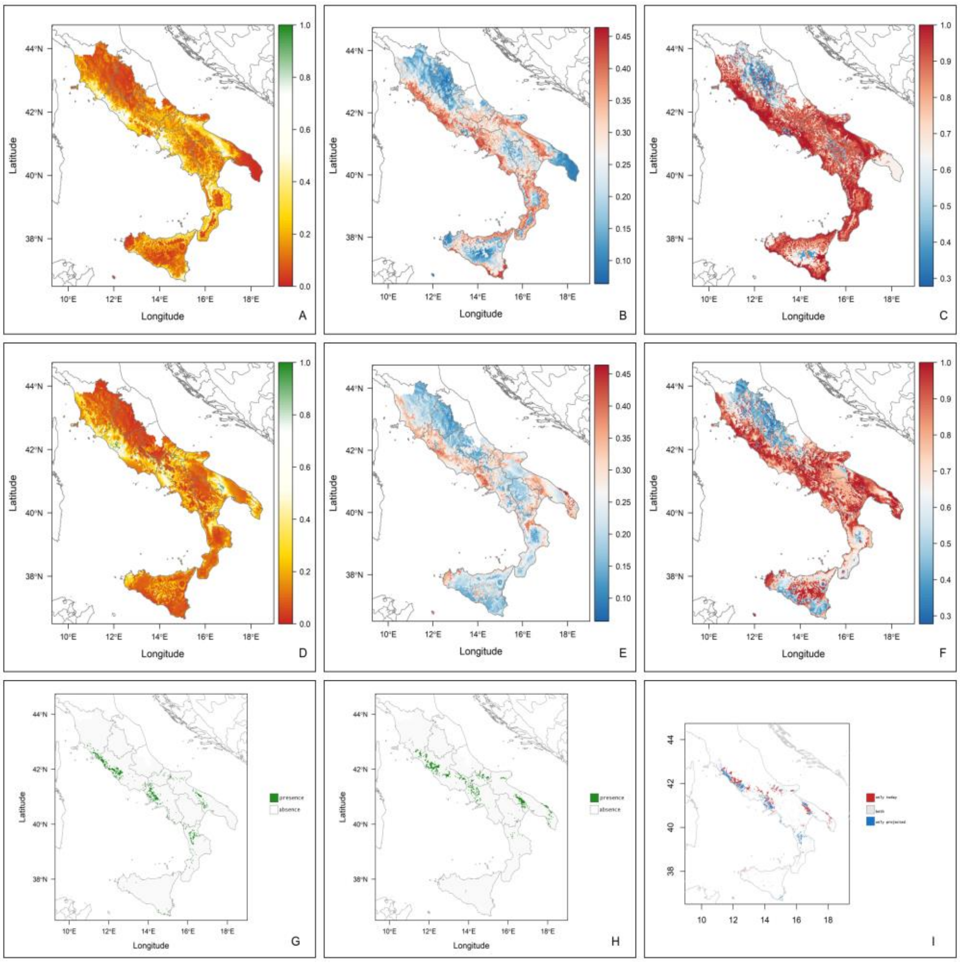

Fig. S.3.145-A) 'Projected' base output; B) 'Projected' uncertainty standard deviation; C) 'Projected' uncertainty range of values; D) 'Present' base output; E) 'Present' uncertainty standard deviation; F) 'Present' uncertainty range of values; G) 'Projected' binary distribution; H) 'Present' binary distribution; I) Range size variation.

Value	Present	Projected
AUC.train	0.601	0.999
AUC.test	0.601	0.584
AUC.diff	0.415	0.415
pROC	1.196	1.210
OR.p10	0.833	1.000
CBI	0.974	0.603
perc.stable	6.424	6.424
range.size	2.107	2.107

S.3.146 *Klasea flavescens* Bioclim

Species: *Klasea flavescens* (L.) Holub subsp. *cichoracea* (L.) Greuter & Wagenitz

Procedure: Bioclim

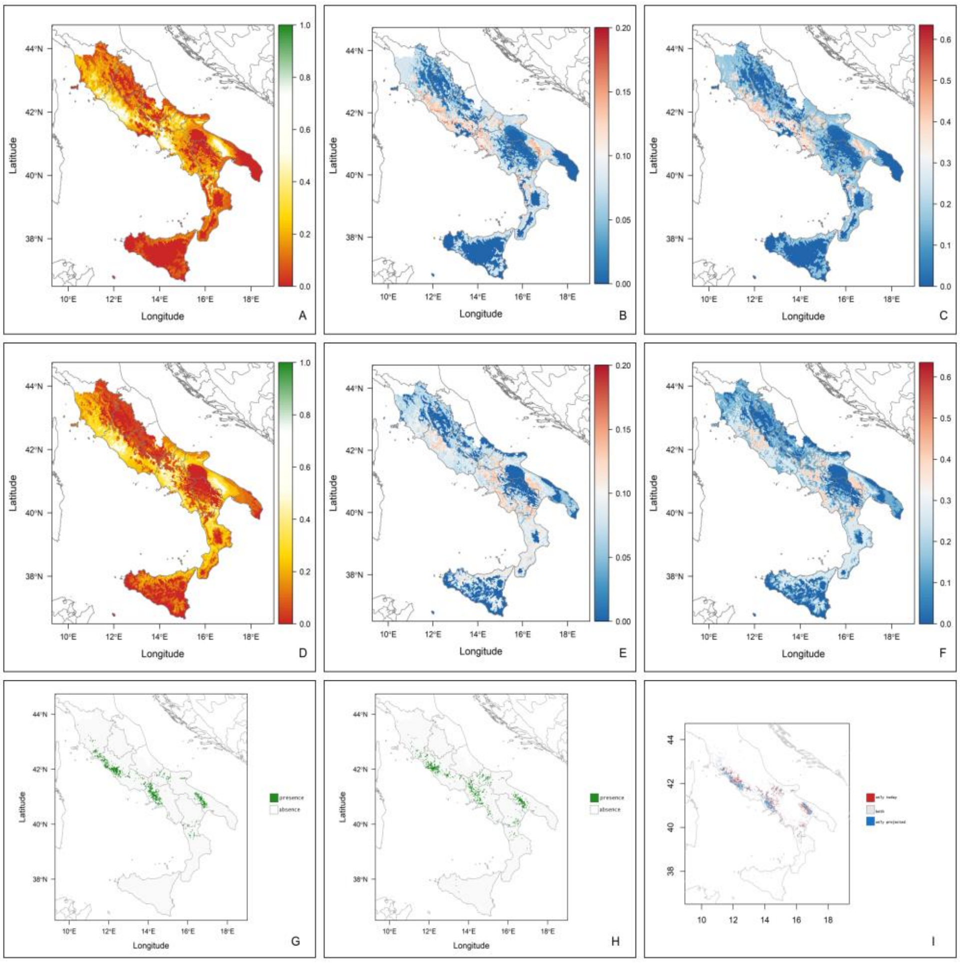

Fig. S.3.146-A) 'Projected' base output; B) 'Projected' uncertainty standard deviation; C) 'Projected' uncertainty range of values; D) 'Present' base output; E) 'Present' uncertainty standard deviation; F) 'Present' uncertainty range of values; G) 'Projected' binary distribution; H) 'Present' binary distribution; I) Range size variation.

Value	Present	Projected
AUC.train	0.644	0.749
AUC.test	0.644	0.663
AUC.diff	0.089	0.089
pROC	1.179	1.237
OR.p10	0.300	0.287
CBI	0.855	0.819
perc.stable	21.112	21.112
range.size	1.795	1.795

S.3.147 *Klasea flavescens* Domain

Species: *Klasea flavescens* (L.) Holub subsp. *cichoracea* (L.) Greuter & Wagenitz

Procedure: Domain

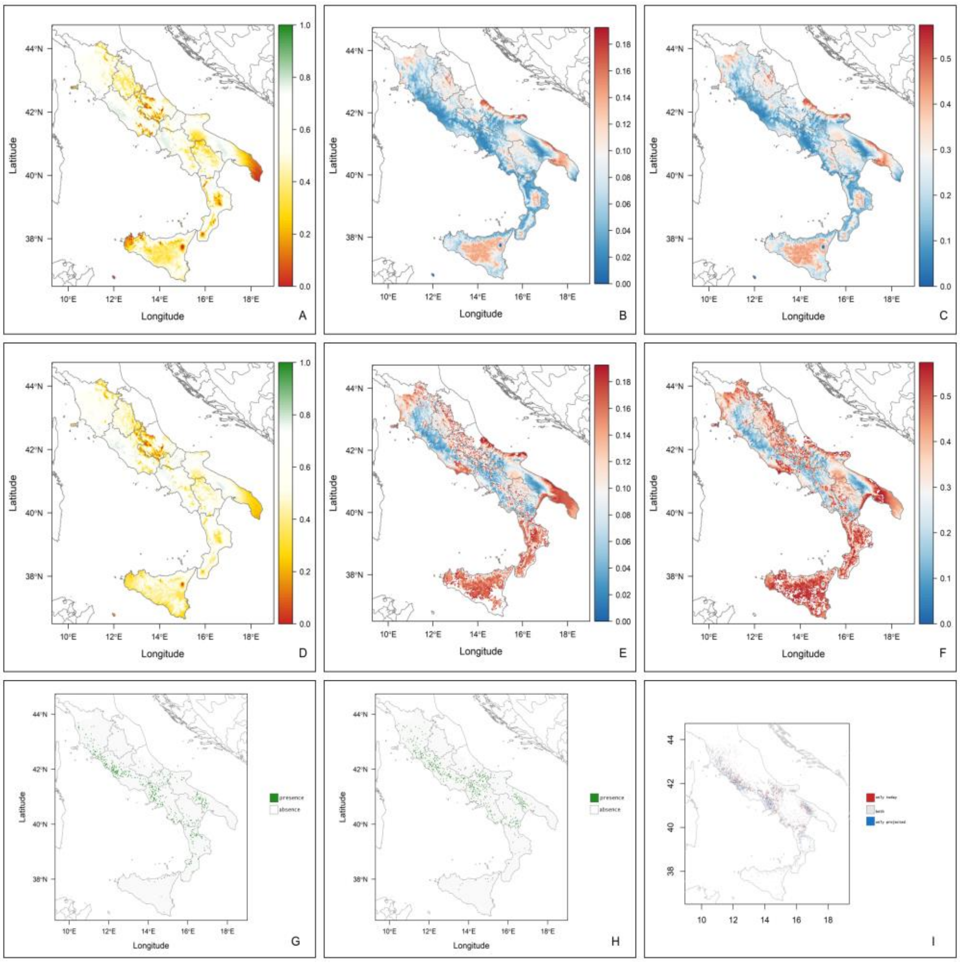

Fig. S.3.147-A) 'Projected' base output; B) 'Projected' uncertainty standard deviation; C) 'Projected' uncertainty range of values; D) 'Present' base output; E) 'Present' uncertainty standard deviation; F) 'Present' uncertainty range of values; G) 'Projected' binary distribution; H) 'Present' binary distribution; I) Range size variation.

Value	Present	Projected
AUC.train	0.664	0.734
AUC.test	0.664	0.690
AUC.diff	0.092	0.092
pROC	1.366	1.381
OR.p10	0.300	0.282
CBI	-0.164	-0.193
perc.stable	5.856	5.856
range.size	0.858	0.858

S.3.148 *Klasea flavescens* Maxent

Species: *Klasea flavescens* (L.) Holub subsp. *cichoracea* (L.) Greuter & Wagenitz

Procedure: Maxent

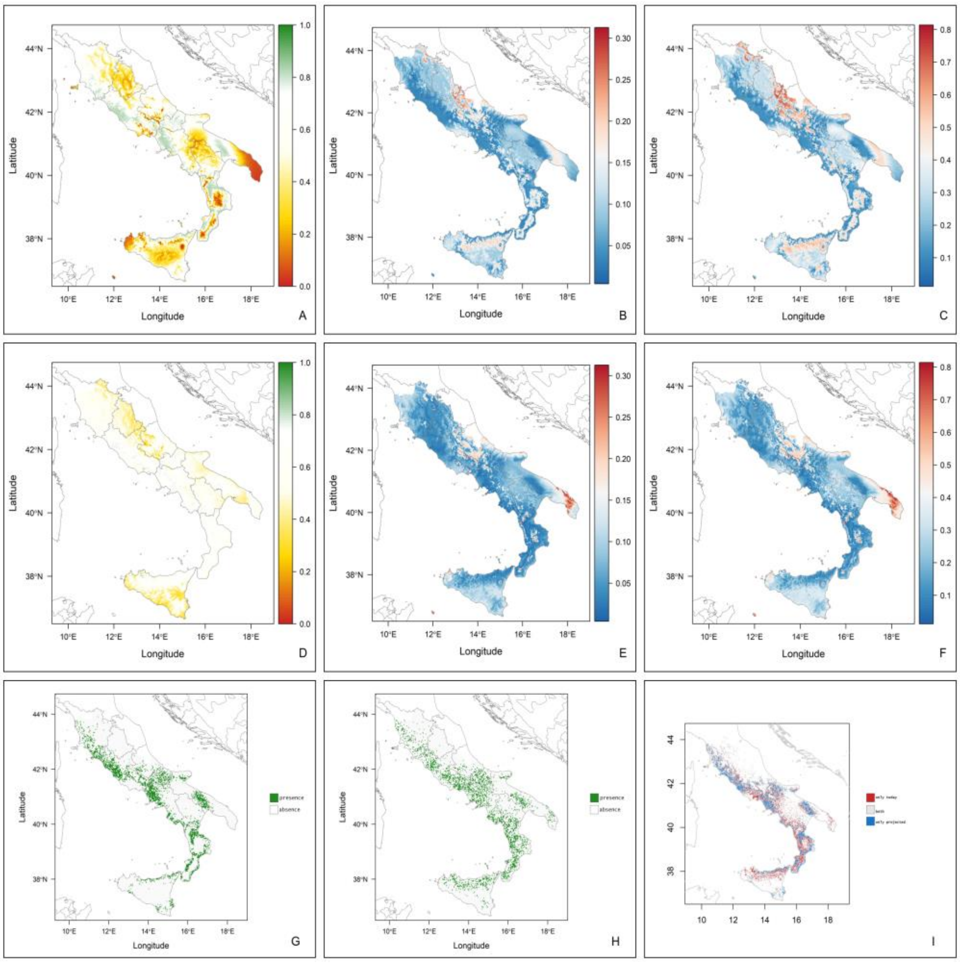

Fig. S.3.148-A) 'Projected' base output; B) 'Projected' uncertainty standard deviation; C) 'Projected' uncertainty range of values; D) 'Present' base output; E) 'Present' uncertainty standard deviation; F) 'Present' uncertainty range of values; G) 'Projected' binary distribution; H) 'Present' binary distribution; I) Range size variation.

Value	Present	Projected
AUC.train	0.625	0.747
AUC.test	0.625	0.603
AUC.diff	0.149	0.149
pROC	1.253	1.266
OR.p10	0.283	0.342
CBI	0.934	-0.333
perc.stable	15.820	15.820
range.size	1.090	1.090

S.3.149 *Klasea flavescens* KDE

Species: *Klasea flavescens* (L.) Holub subsp. *cichoracea* (L.) Greuter & Wagenitz

Procedure: KDE

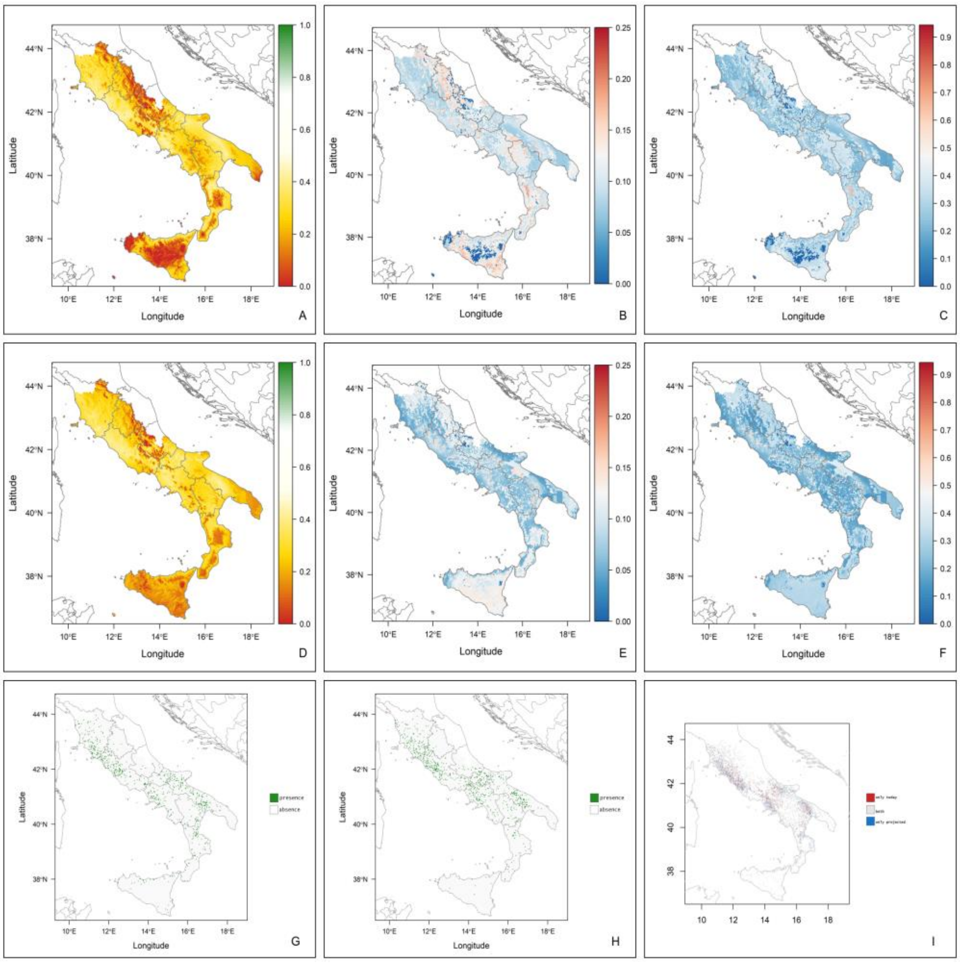

Fig. S.3.149-A) 'Projected' base output; B) 'Projected' uncertainty standard deviation; C) 'Projected' uncertainty range of values; D) 'Present' base output; E) 'Present' uncertainty standard deviation; F) 'Present' uncertainty range of values; G) 'Projected' binary distribution; H) 'Present' binary distribution; I) Range size variation.

Value	Present	Projected
AUC.train	0.660	0.772
AUC.test	0.660	0.705
AUC.diff	0.098	0.082
pROC	1.304	1.346
OR.p10	0.250	0.183
CBI	0.899	0.771
perc.stable	4.362	4.362
range.size	0.088	0.088

S.3.150 *Klasea flavescens* CTA

Species: *Klasea flavescens* (L.) Holub subsp. *cichoracea* (L.) Greuter & Wagenitz

Procedure: CTA

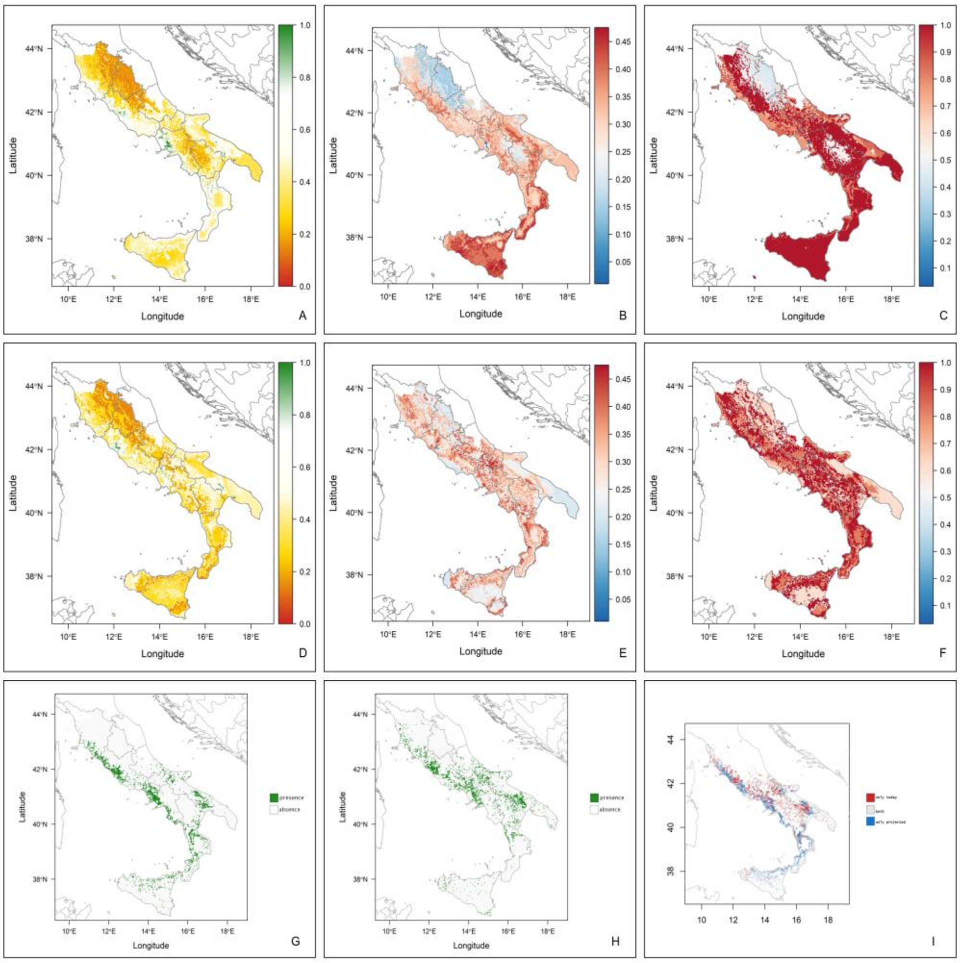

Fig. S.3.150-A) 'Projected' base output; B) 'Projected' uncertainty standard deviation; C) 'Projected' uncertainty range of values; D) 'Present' base output; E) 'Present' uncertainty standard deviation; F) 'Present' uncertainty range of values; G) 'Projected' binary distribution; H) 'Present' binary distribution; I) Range size variation.

Value	Present	Projected
AUC.train	0.721	0.854
AUC.test	0.721	0.718
AUC.diff	0.154	0.154
pROC	1.164	1.151
OR.p10	0.367	0.295
CBI	0.964	0.102
perc.stable	11.111	11.111
range.size	-0.289	-0.289

S.3.151 *Klasea flavescens* FDA

Species: *Klasea flavescens* (L.) Holub subsp. *cichoracea* (L.) Greuter & Wagenitz

Procedure: FDA

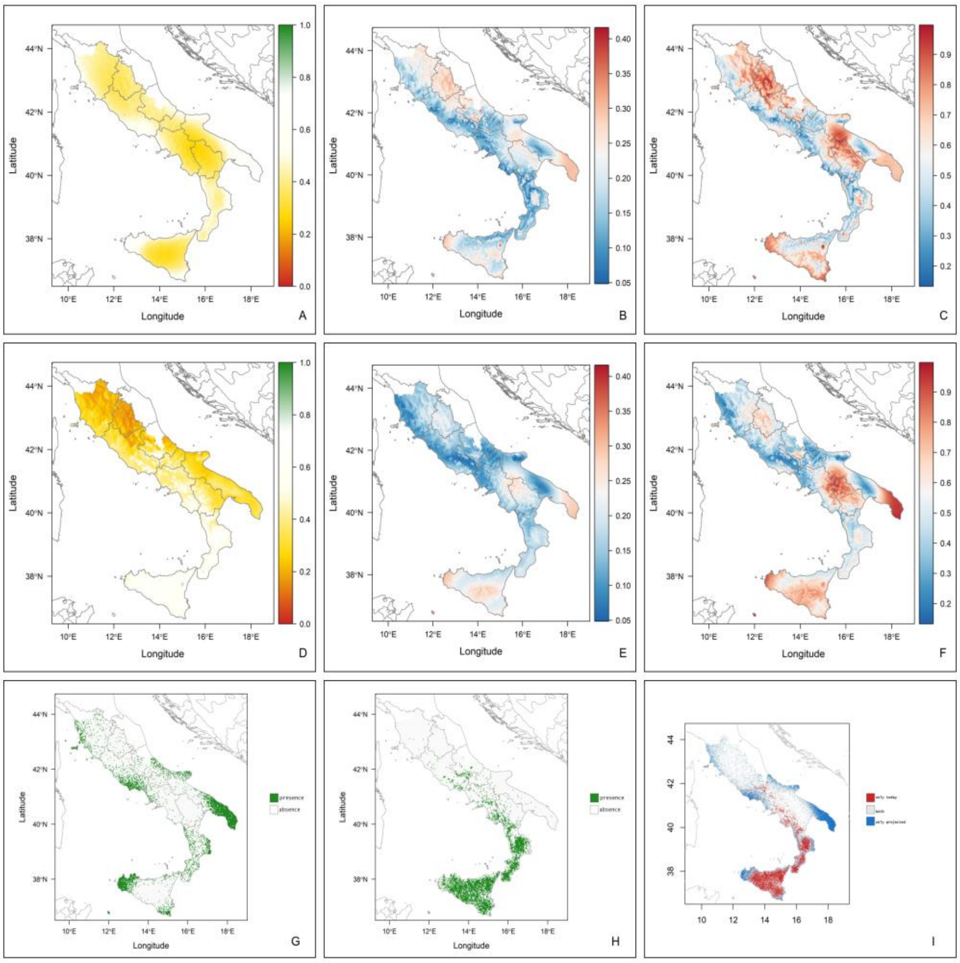

Fig. S.3.151-A) 'Projected' base output; B) 'Projected' uncertainty standard deviation; C) 'Projected' uncertainty range of values; D) 'Present' base output; E) 'Present' uncertainty standard deviation; F) 'Present' uncertainty range of values; G) 'Projected' binary distribution; H) 'Present' binary distribution; I) Range size variation.

Value	Present	Projected
AUC.train	0.562	0.646
AUC.test	0.562	0.609
AUC.diff	0.078	0.078
pROC	1.209	1.241
OR.p10	0.317	0.250
CBI	-0.099	0.220
perc.stable	8.875	8.875
range.size	-0.859	-0.859

S.3.152 *Klasea flavescens* GAM

Species: *Klasea flavescens* (L.) Holub subsp. *cichoracea* (L.) Greuter & Wagenitz

Procedure: GAM

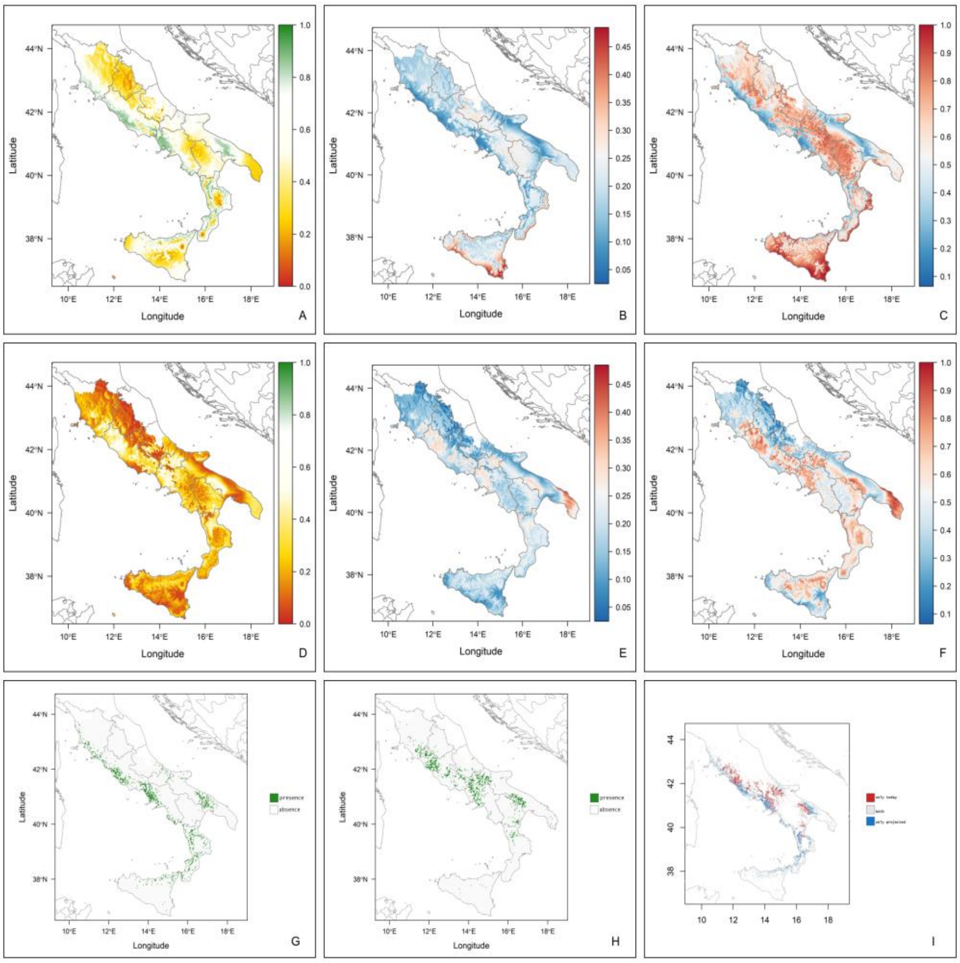

Fig. S.3.152-A) 'Projected' base output; B) 'Projected' uncertainty standard deviation; C) 'Projected' uncertainty range of values; D) 'Present' base output; E) 'Present' uncertainty standard deviation; F) 'Present' uncertainty range of values; G) 'Projected' binary distribution; H) 'Present' binary distribution; I) Range size variation.

Value	Present	Projected
AUC.train	0.550	0.748
AUC.test	0.550	0.529
AUC.diff	0.219	0.219
pROC	1.203	1.197
OR.p10	0.517	0.435
CBI	0.820	0.136
perc.stable	5.281	5.281
range.size	4.014	4.014

S.3.153 *Klasea flavescens* GLM

Species: *Klasea flavescens* (L.) Holub subsp. *cichoracea* (L.) Greuter & Wagenitz

Procedure: GLM

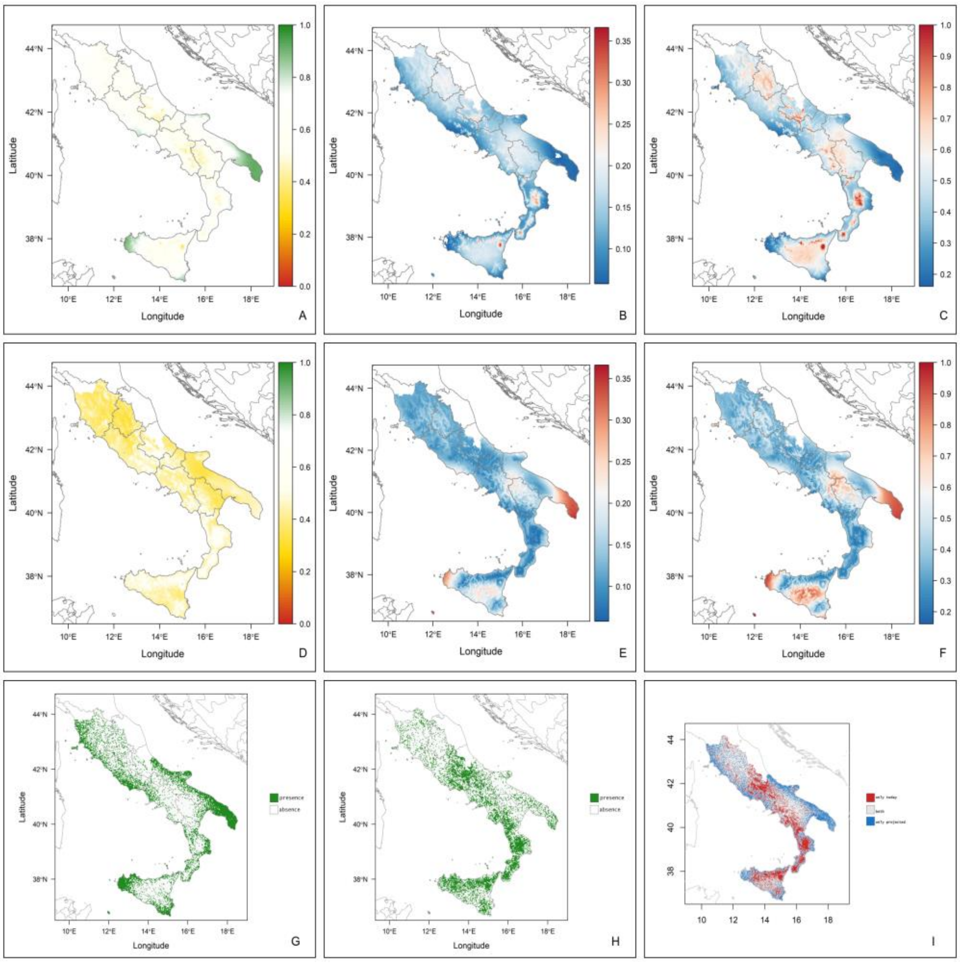

Fig. S.3.153-A) 'Projected' base output; B) 'Projected' uncertainty standard deviation; C) 'Projected' uncertainty range of values; D) 'Present' base output; E) 'Present' uncertainty standard deviation; F) 'Present' uncertainty range of values; G) 'Projected' binary distribution; H) 'Present' binary distribution; I) Range size variation.

Value	Present	Projected
AUC.train	0.382	0.586
AUC.test	0.382	0.494
AUC.diff	0.100	0.100
pROC	1.129	1.207
OR.p10	0.400	0.330
CBI	0.385	-0.142
perc.stable	17.150	17.150
range.size	0.504	0.504

S.3.154 *Klasea flavescens* MARS

Species: *Klasea flavescens* (L.) Holub subsp. *cichoracea* (L.) Greuter & Wagenitz

Procedure: MARS

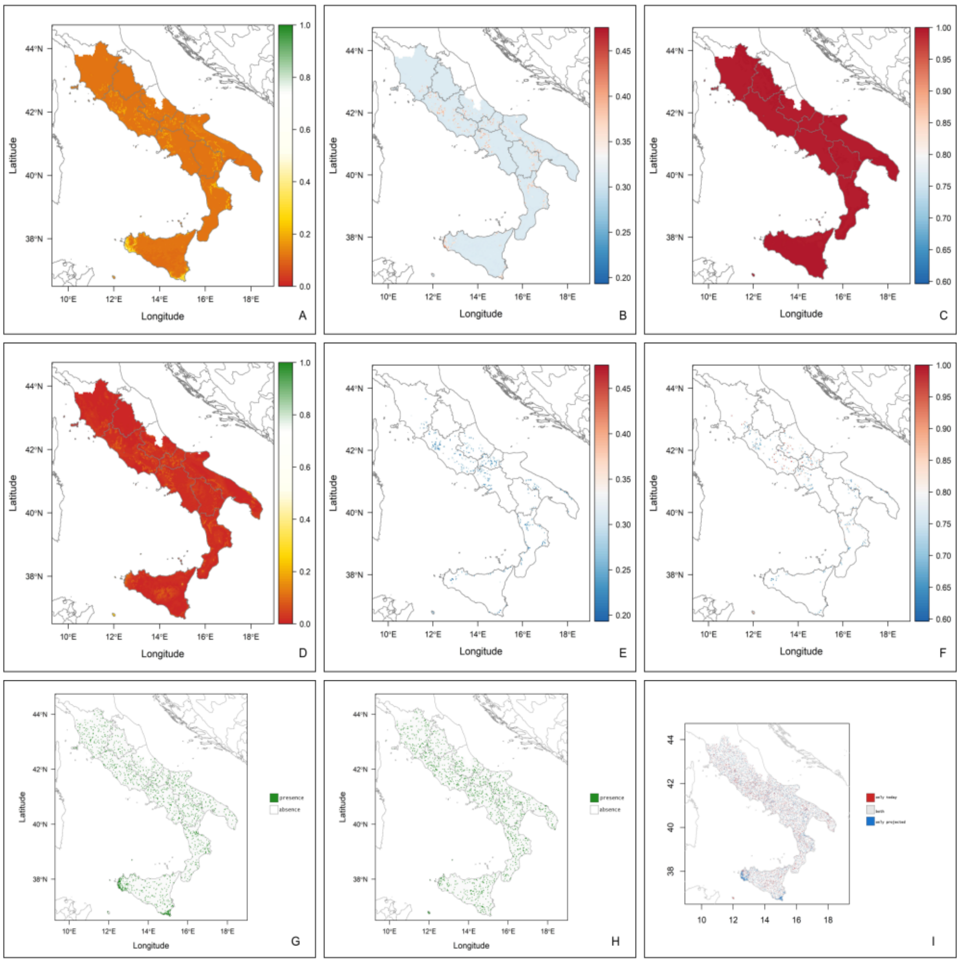

Fig. S.3.154-A) 'Projected' base output; B) 'Projected' uncertainty standard deviation; C) 'Projected' uncertainty range of values; D) 'Present' base output; E) 'Present' uncertainty standard deviation; F) 'Present' uncertainty range of values; G) 'Projected' binary distribution; H) 'Present' binary distribution; I) Range size variation.

Value	Present	Projected
AUC.train	0.577	0.638
AUC.test	0.577	0.539
AUC.diff	0.109	0.109
pROC	1.168	1.389
OR.p10	0.400	0.293
CBI	0.742	0.218
perc.stable	4.478	4.478
range.size	2.413	2.413

S.3.155 *Klasea flavescens* RF

Species: *Klasea flavescens* (L.) Holub subsp. *cichoracea* (L.) Greuter & Wagenitz

Procedure: RF

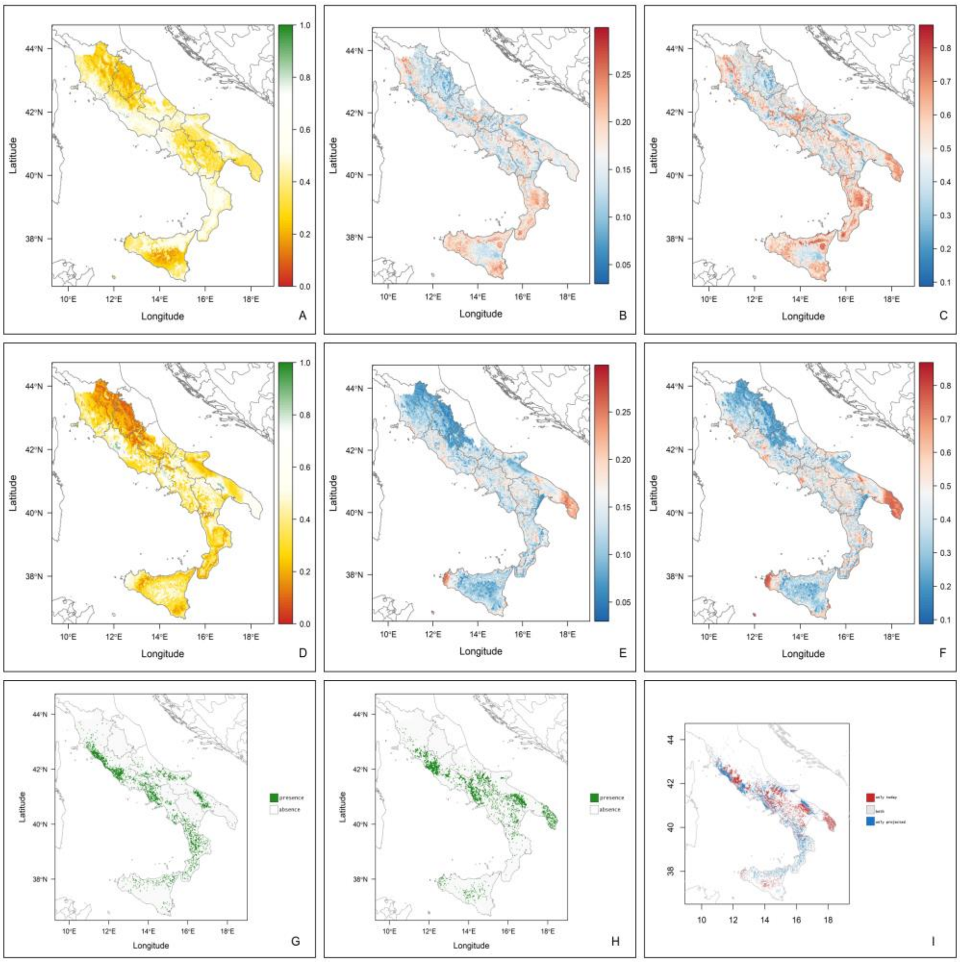

Fig. S.3.155-A) 'Projected' base output; B) 'Projected' uncertainty standard deviation; C) 'Projected' uncertainty range of values; D) 'Present' base output; E) 'Present' uncertainty standard deviation; F) 'Present' uncertainty range of values; G) 'Projected' binary distribution; H) 'Present' binary distribution; I) Range size variation.

Value	Present	Projected
AUC.train	0.933	1.000
AUC.test	0.933	0.899
AUC.diff	0.101	0.101
pROC	1.384	1.238
OR.p10	0.767	0.890
CBI	0.965	0.813
perc.stable	9.754	9.754
range.size	0.607	0.607

S.3.156 *Klasea flavescens* SVM

Species: *Klasea flavescens* (L.) Holub subsp. *cichoracea* (L.) Greuter & Wagenitz

Procedure: SVM

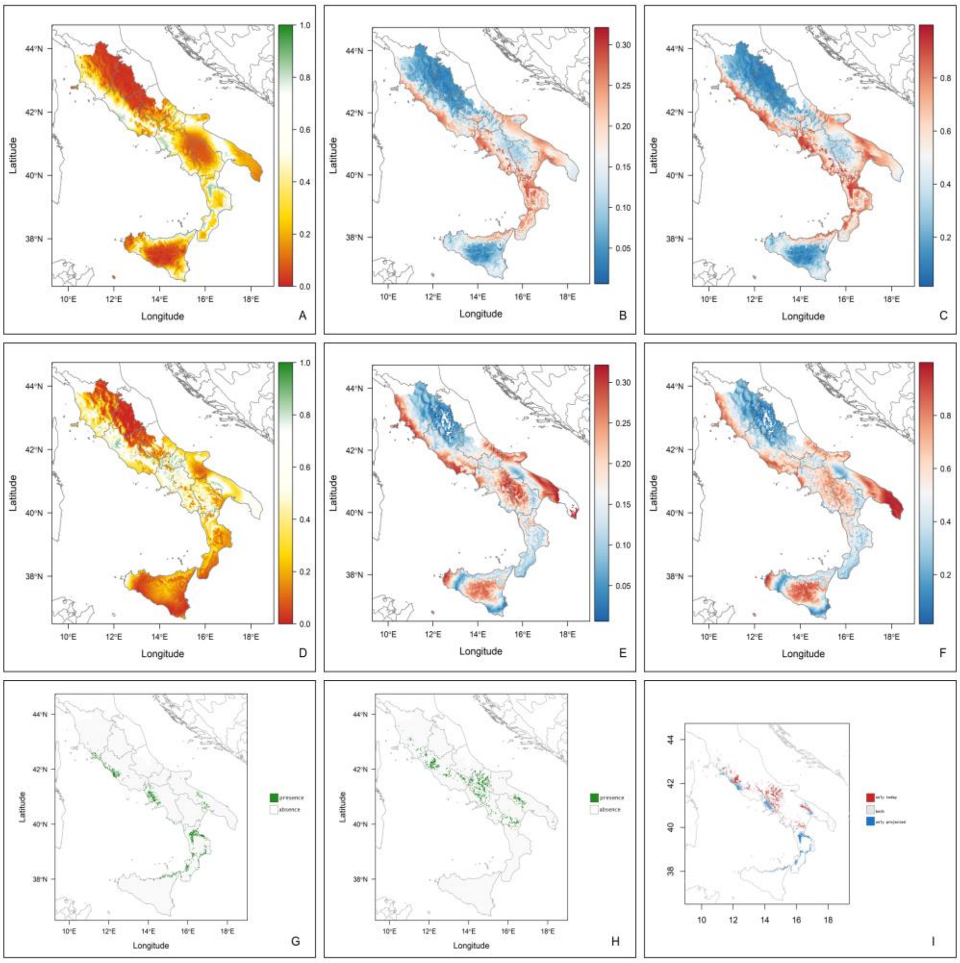

Fig. S.3.156-A) 'Projected' base output; B) 'Projected' uncertainty standard deviation; C) 'Projected' uncertainty range of values; D) 'Present' base output; E) 'Present' uncertainty standard deviation; F) 'Present' uncertainty range of values; G) 'Projected' binary distribution; H) 'Present' binary distribution; I) Range size variation.

Value	Present	Projected
AUC.train	0.817	0.877
AUC.test	0.817	0.776
AUC.diff	0.104	0.104
pROC	1.359	1.340
OR.p10	0.333	0.363
CBI	0.939	0.634
perc.stable	1.014	1.014
range.size	-2.584	-2.584

S.3.157 *Koeleria splendens* Biomod2

Species: *Koeleria splendens* C.Presl

Procedure: Biomod2

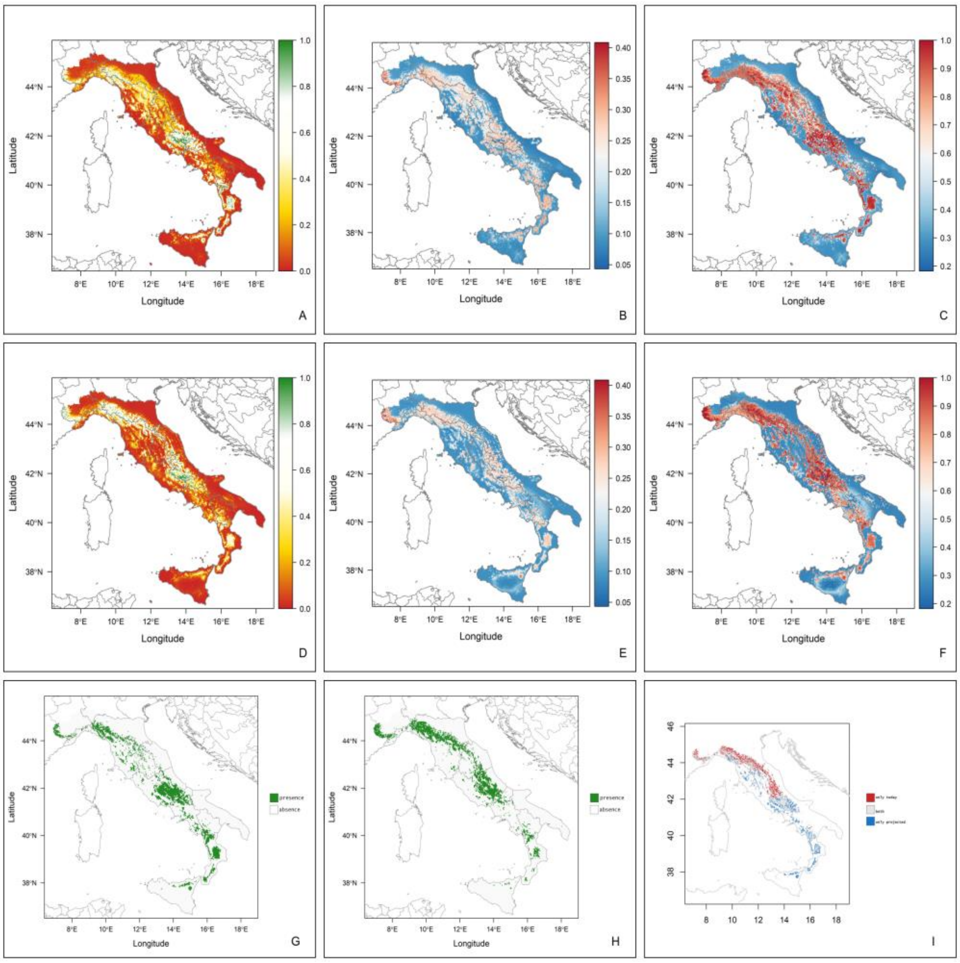

Fig. S.3.157-A) 'Projected' base output; B) 'Projected' uncertainty standard deviation; C) 'Projected' uncertainty range of values; D) 'Present' base output; E) 'Present' uncertainty standard deviation; F) 'Present' uncertainty range of values; G) 'Projected' binary distribution; H) 'Present' binary distribution; I) Range size variation.

Value	Present	Projected
AUC.train	0.877	0.963
AUC.test	0.877	0.880
AUC.diff	0.083	0.083
pROC	1.324	1.346
OR.p10	0.240	0.364
CBI	0.960	0.986
perc.stable	44.655	44.655
range.size	0.386	0.386

S.3.158 *Koeleria splendens* Bioclim

Species: *Koeleria splendens* C.Presl

Procedure: Bioclim

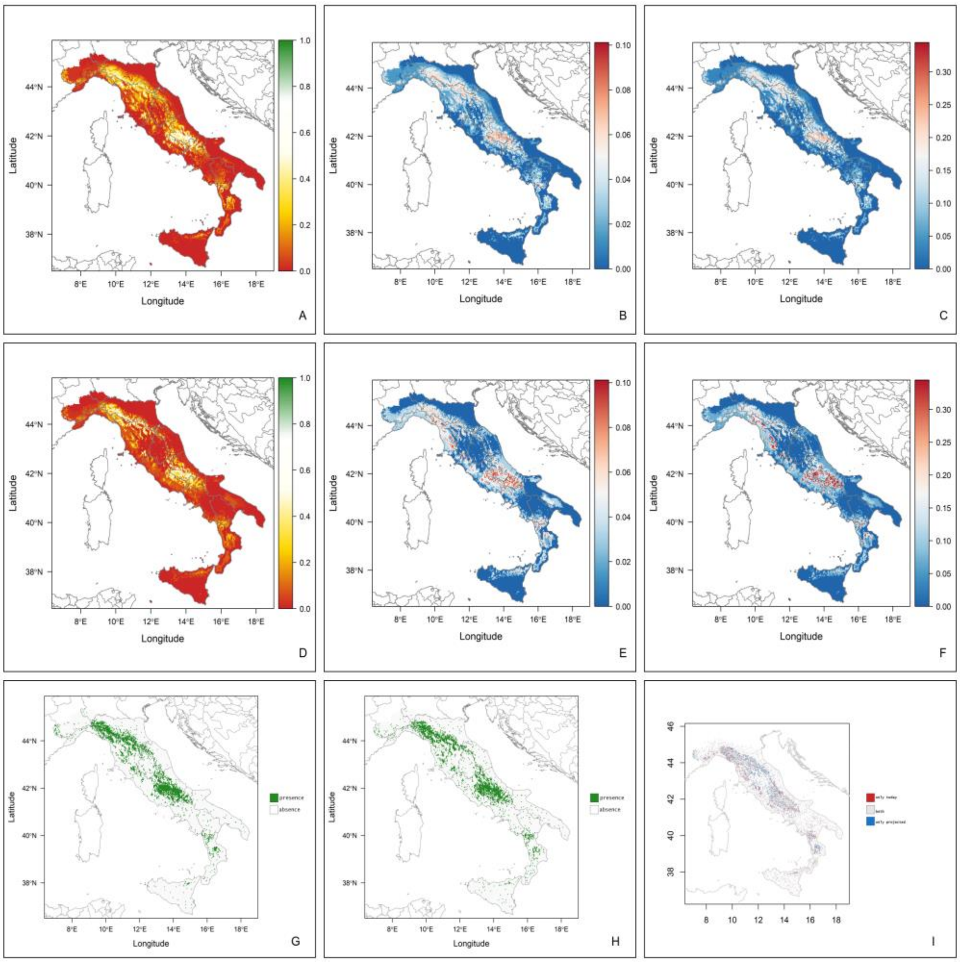

Fig. S.3.158-A) 'Projected' base output; B) 'Projected' uncertainty standard deviation; C) 'Projected' uncertainty range of values; D) 'Present' base output; E) 'Present' uncertainty standard deviation; F) 'Present' uncertainty range of values; G) 'Projected' binary distribution; H) 'Present' binary distribution; I) Range size variation.

Value	Present	Projected
AUC.train	0.824	0.836
AUC.test	0.824	0.796
AUC.diff	0.041	0.041
pROC	1.154	1.132
OR.p10	0.192	0.180
CBI	0.640	0.836
perc.stable	48.405	48.405
range.size	-0.668	-0.668

S.3.159 *Koeleria splendens* Domain

Species: *Koeleria splendens* C.Presl

Procedure: Domain

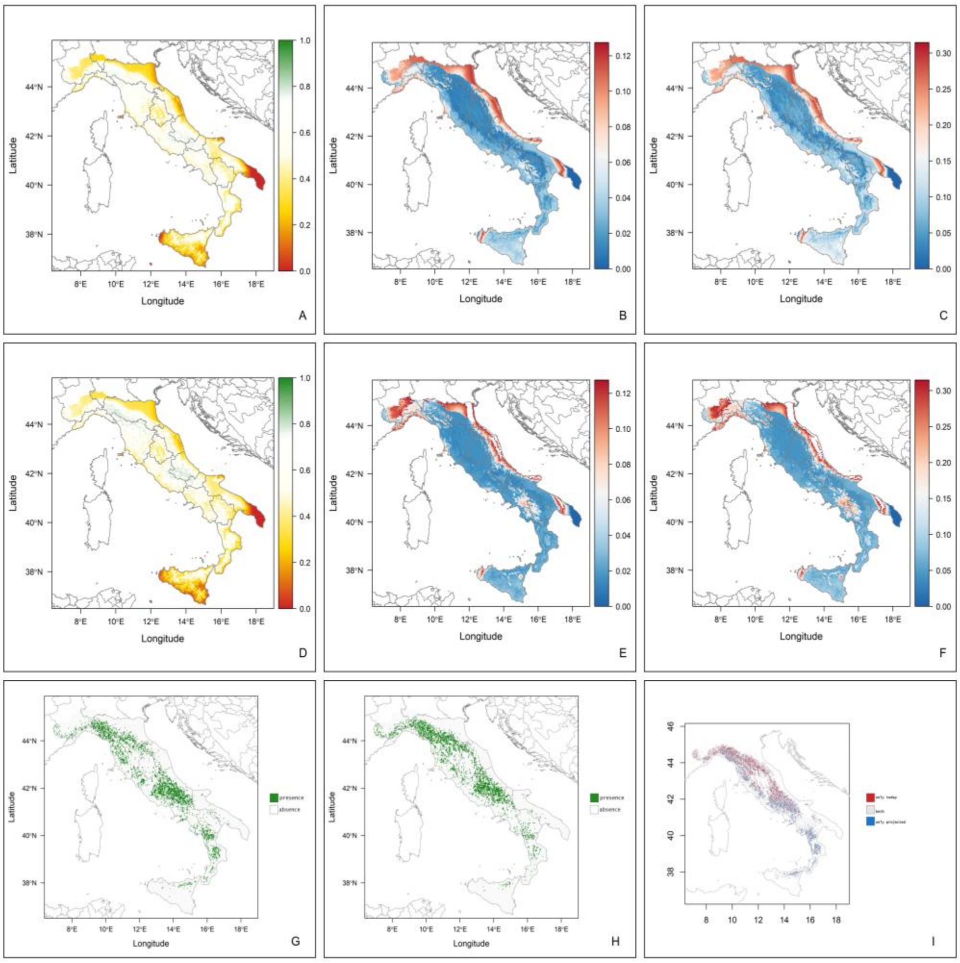

Fig. S.3.159-A) 'Projected' base output; B) 'Projected' uncertainty standard deviation; C) 'Projected' uncertainty range of values; D) 'Present' base output; E) 'Present' uncertainty standard deviation; F) 'Present' uncertainty range of values; G) 'Projected' binary distribution; H) 'Present' binary distribution; I) Range size variation.

Value	Present	Projected
AUC.train	0.837	0.804
AUC.test	0.837	0.807
AUC.diff	0.010	0.010
pROC	1.304	1.279
OR.p10	0.120	0.112
CBI	0.838	0.596
perc.stable	31.401	31.401
range.size	-0.845	-0.845

S.3.160 *Koeleria splendens* Maxent

Species: *Koeleria splendens* C.Presl

Procedure: Maxent

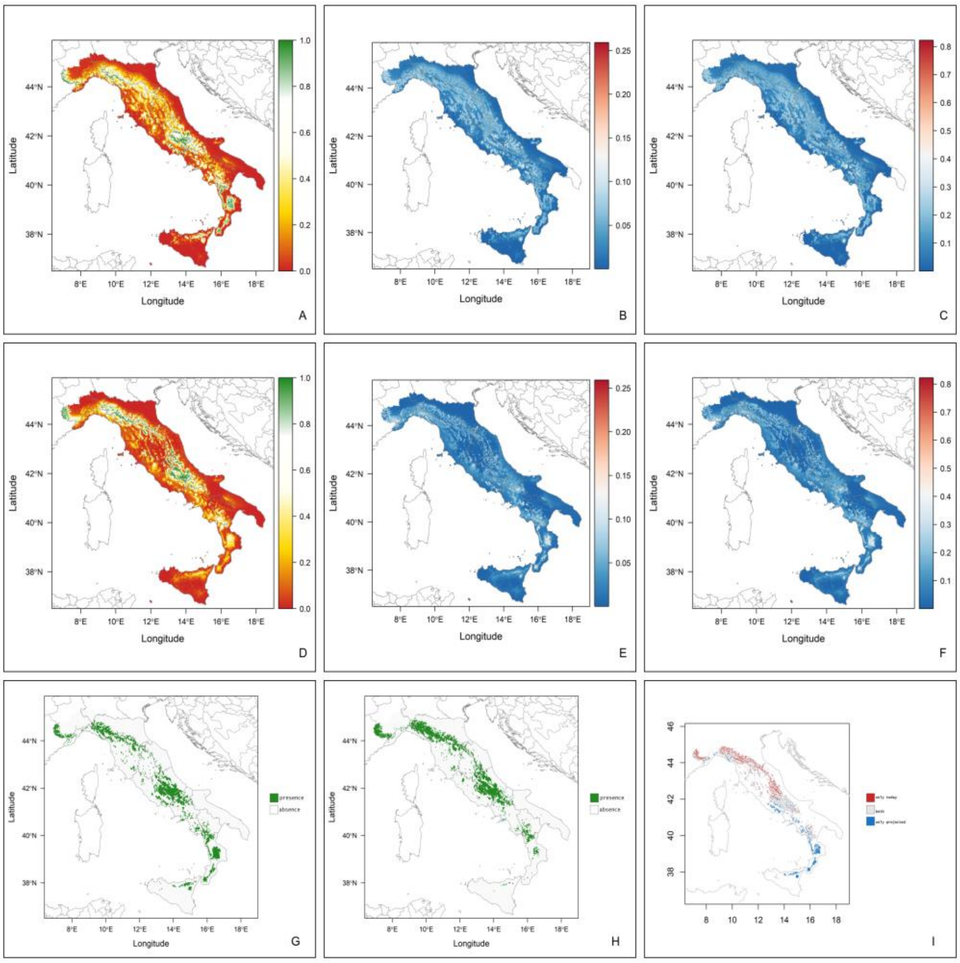

Fig. S.3.160-A) 'Projected' base output; B) 'Projected' uncertainty standard deviation; C) 'Projected' uncertainty range of values; D) 'Present' base output; E) 'Present' uncertainty standard deviation; F) 'Present' uncertainty range of values; G) 'Projected' binary distribution; H) 'Present' binary distribution; I) Range size variation.

Value	Present	Projected
AUC.train	0.879	0.883
AUC.test	0.879	0.874
AUC.diff	0.025	0.025
pROC	1.406	1.420
OR.p10	0.132	0.136
CBI	0.977	0.980
perc.stable	54.696	54.696
range.size	-0.129	-0.129

S.3.161 *Koeleria splendens* KDE

Species: *Koeleria splendens* C.Presl

Procedure: KDE

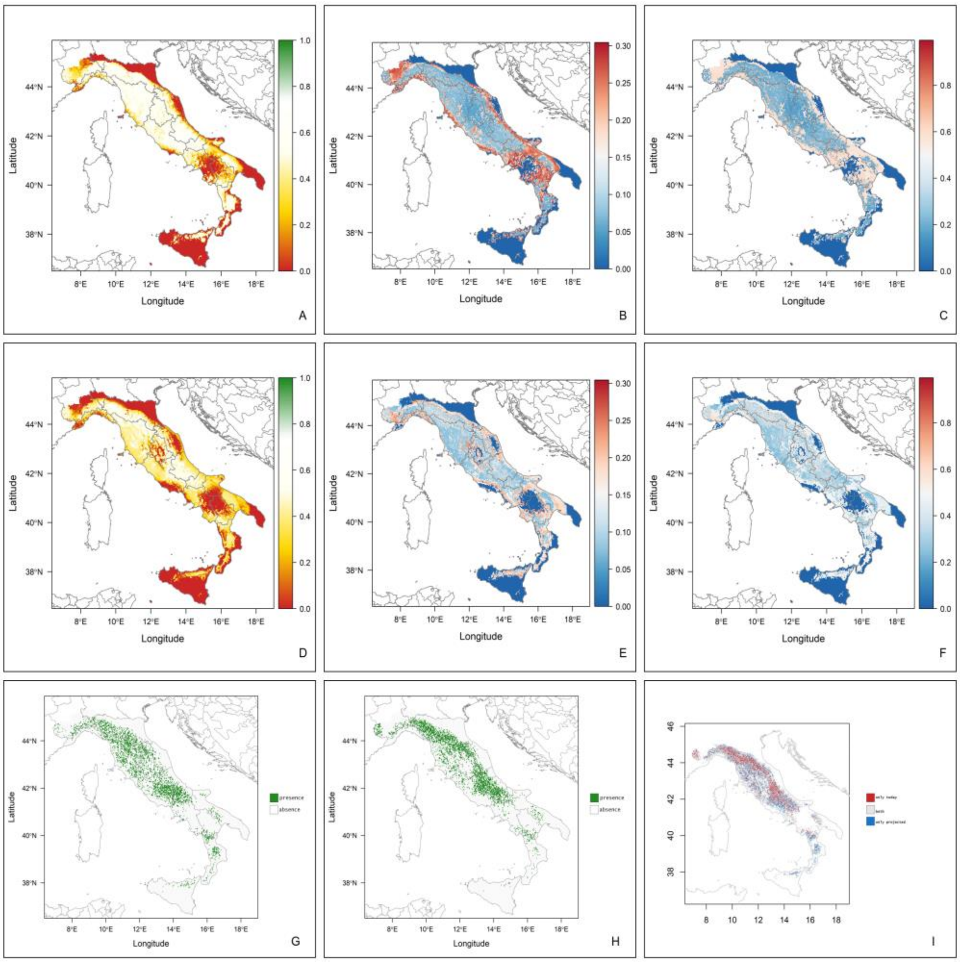

Fig. S.3.161-A) 'Projected' base output; B) 'Projected' uncertainty standard deviation; C) 'Projected' uncertainty range of values; D) 'Present' base output; E) 'Present' uncertainty standard deviation; F) 'Present' uncertainty range of values; G) 'Projected' binary distribution; H) 'Present' binary distribution; I) Range size variation.

Value	Present	Projected
AUC.train	0.841	0.833
AUC.test	0.841	0.783
AUC.diff	0.030	0.055
pROC	1.269	1.218
OR.p10	0.096	0.196
CBI	0.968	0.951
perc.stable	22.996	22.996
range.size	0.767	0.767

S.3.162 *Koeleria splendens* CTA

Species: *Koeleria splendens* C.Presl

Procedure: CTA

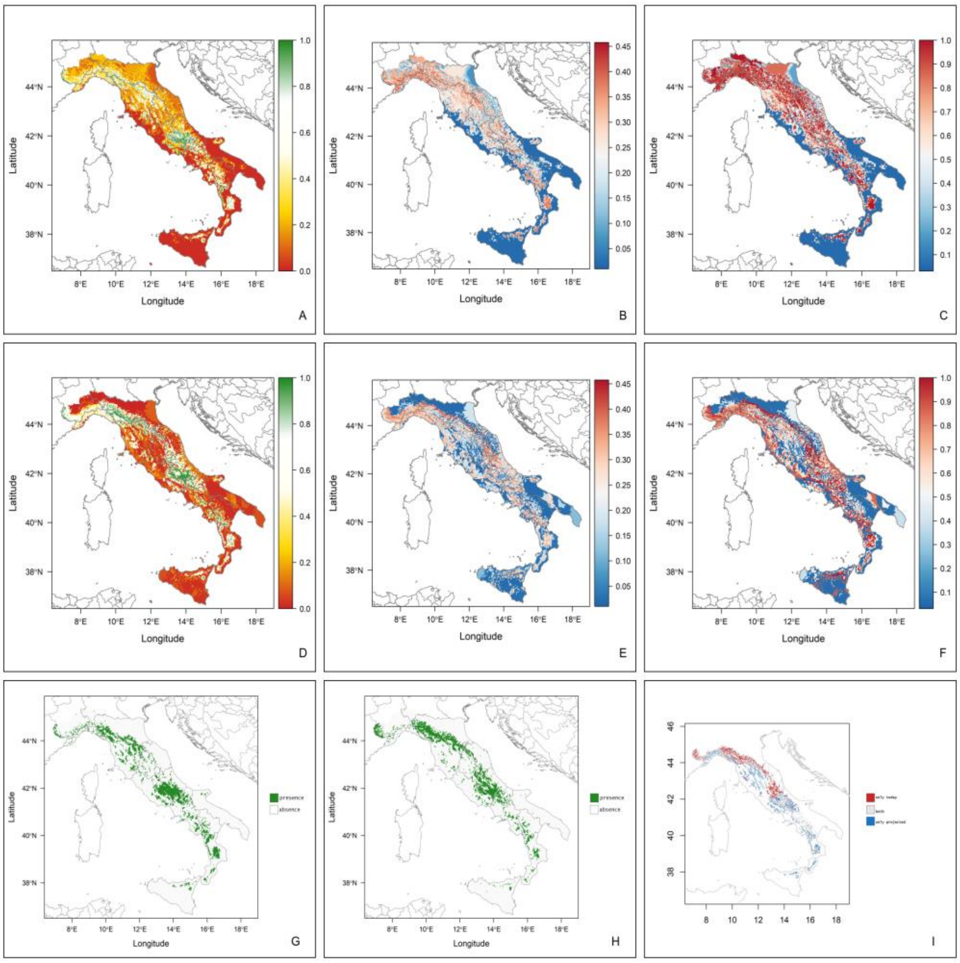

Fig. S.3.162-A) 'Projected' base output; B) 'Projected' uncertainty standard deviation; C) 'Projected' uncertainty range of values; D) 'Present' base output; E) 'Present' uncertainty standard deviation; F) 'Present' uncertainty range of values; G) 'Projected' binary distribution; H) 'Present' binary distribution; I) Range size variation.

Value	Present	Projected
AUC.train	0.876	0.955
AUC.test	0.876	0.883
AUC.diff	0.072	0.072
pROC	1.152	1.153
OR.p10	0.200	0.196
CBI	0.958	0.852
perc.stable	43.770	43.770
range.size	0.025	0.025

S.3.163 *Koeleria splendens* FDA

Species: *Koeleria splendens* C.Presl

Procedure: FDA

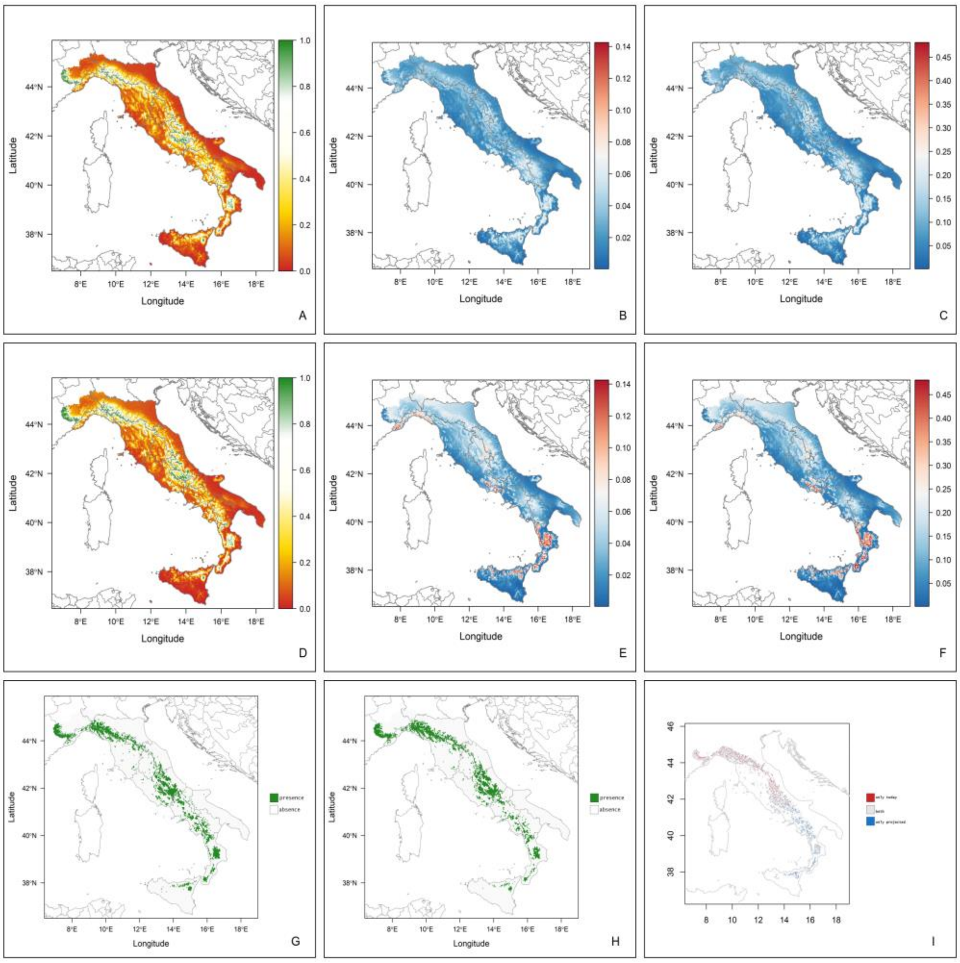

Fig. S.3.163-A) 'Projected' base output; B) 'Projected' uncertainty standard deviation; C) 'Projected' uncertainty range of values; D) 'Present' base output; E) 'Present' uncertainty standard deviation; F) 'Present' uncertainty range of values; G) 'Projected' binary distribution; H) 'Present' binary distribution; I) Range size variation.

Value	Present	Projected
AUC.train	0.855	0.863
AUC.test	0.855	0.841
AUC.diff	0.023	0.023
pROC	1.310	1.383
OR.p10	0.128	0.132
CBI	0.990	0.992
perc.stable	71.110	71.110
range.size	1.180	1.180

S.3.164 *Koeleria splendens* GAM

Species: *Koeleria splendens* C.Presl

Procedure: GAM

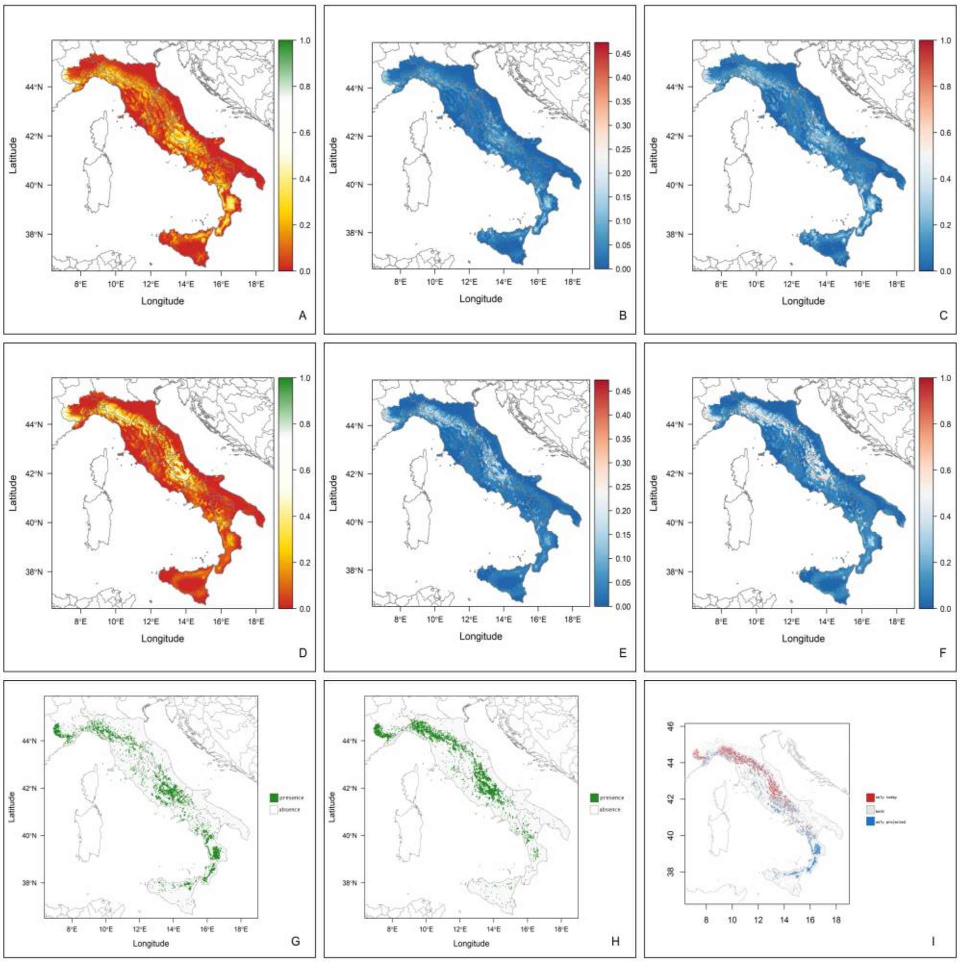

Fig. S.3.164-A) 'Projected' base output; B) 'Projected' uncertainty standard deviation; C) 'Projected' uncertainty range of values; D) 'Present' base output; E) 'Present' uncertainty standard deviation; F) 'Present' uncertainty range of values; G) 'Projected' binary distribution; H) 'Present' binary distribution; I) Range size variation.

Value	Present	Projected
AUC.train	0.830	0.874
AUC.test	0.830	0.849
AUC.diff	0.027	0.027
pROC	1.186	1.334
OR.p10	0.184	0.168
CBI	0.934	0.818
perc.stable	33.622	33.622
range.size	0.532	0.532

S.3.165 *Koeleria splendens* GLM

Species: *Koeleria splendens* C.Presl

Procedure: GLM

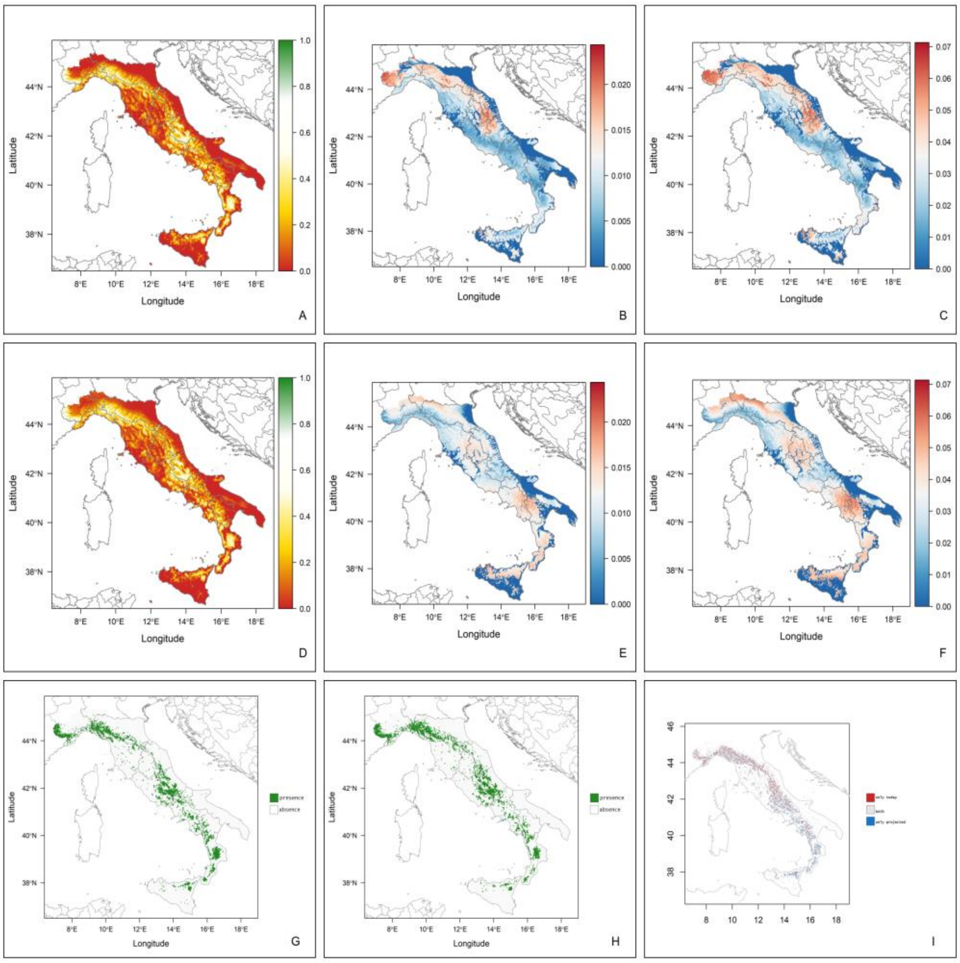

Fig. S.3.165-A) 'Projected' base output; B) 'Projected' uncertainty standard deviation; C) 'Projected' uncertainty range of values; D) 'Present' base output; E) 'Present' uncertainty standard deviation; F) 'Present' uncertainty range of values; G) 'Projected' binary distribution; H) 'Present' binary distribution; I) Range size variation.

Value	Present	Projected
AUC.train	0.847	0.853
AUC.test	0.847	0.849
AUC.diff	0.019	0.019
pROC	1.297	1.375
OR.p10	0.144	0.116
CBI	0.850	0.905
perc.stable	53.371	53.371
range.size	-0.411	-0.411

S.3.166 *Koeleria splendens* MARS

Species: *Koeleria splendens* C.Presl

Procedure: MARS

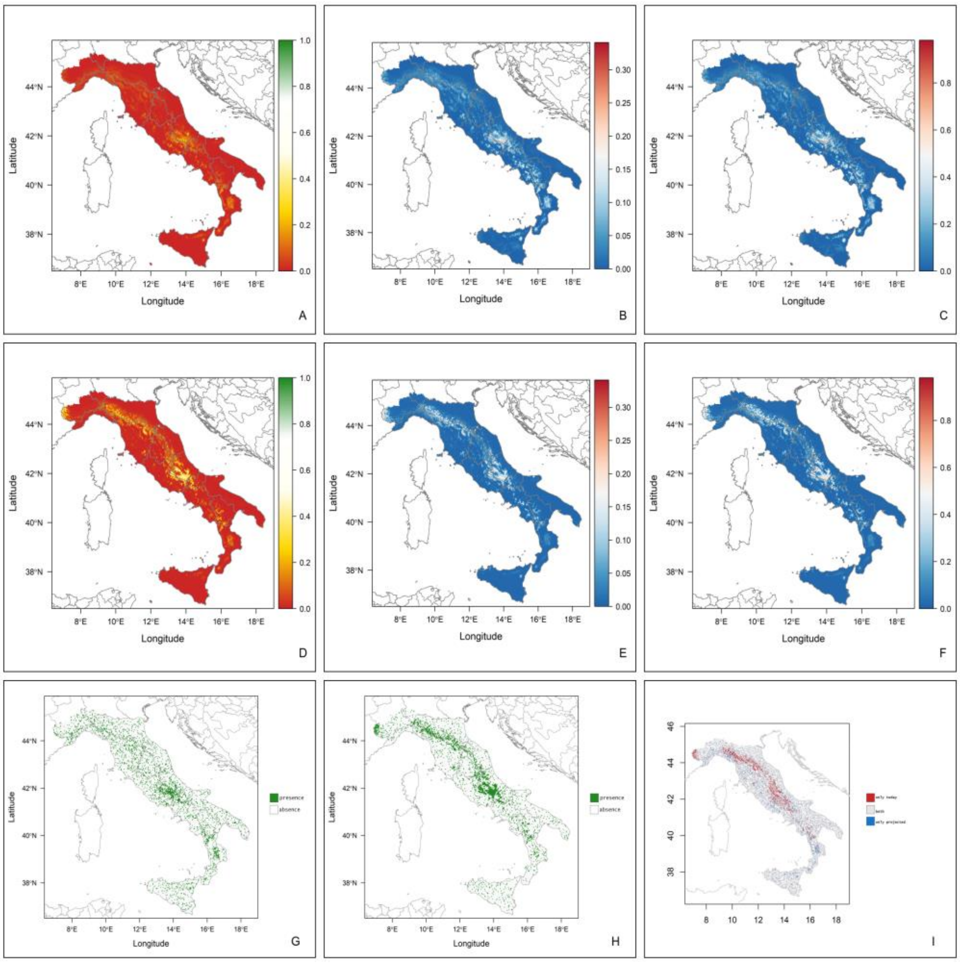

Fig. S.3.166-A) 'Projected' base output; B) 'Projected' uncertainty standard deviation; C) 'Projected' uncertainty range of values; D) 'Present' base output; E) 'Present' uncertainty standard deviation; F) 'Present' uncertainty range of values; G) 'Projected' binary distribution; H) 'Present' binary distribution; I) Range size variation.

Value	Present	Projected
AUC.train	0.834	0.895
AUC.test	0.834	0.864
AUC.diff	0.040	0.040
pROC	1.159	1.296
OR.p10	0.132	0.140
CBI	0.863	0.867
perc.stable	14.243	14.243
range.size	0.327	0.327

S.3.167 *Koeleria splendens* RF

Species: *Koeleria splendens* C.Presl

Procedure: RF

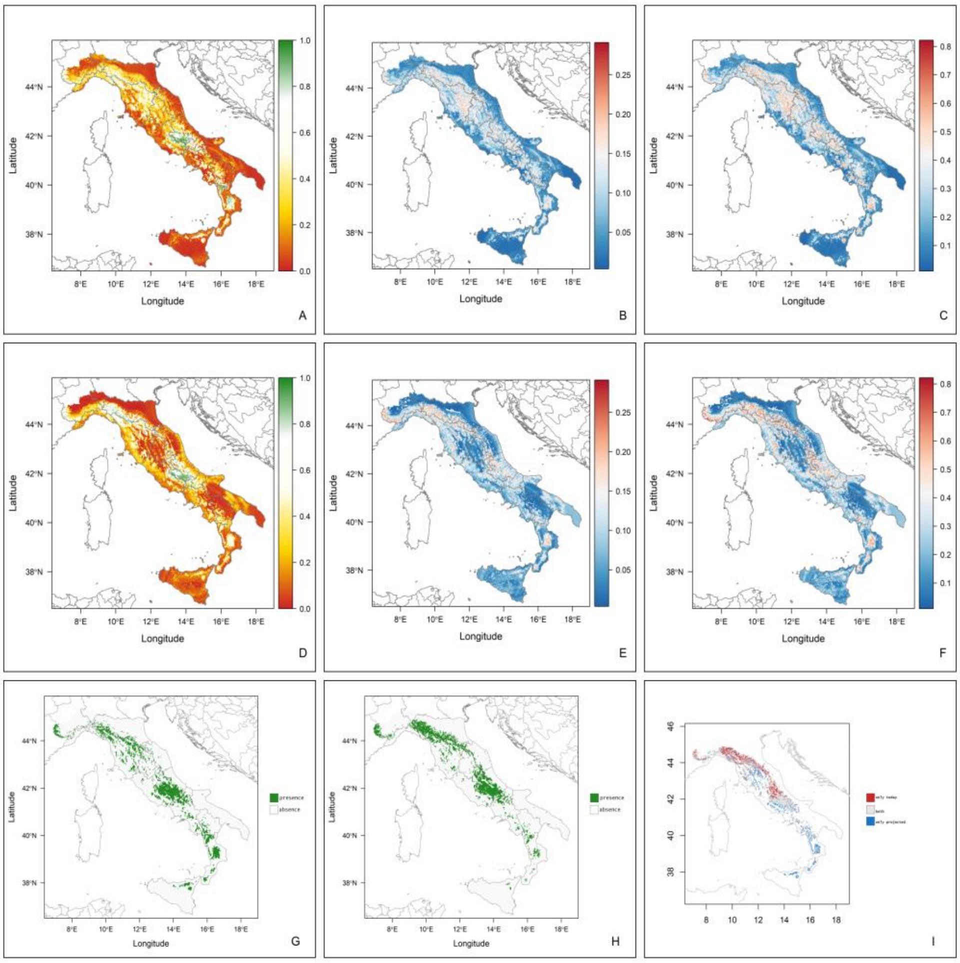

Fig. S.3.167-A) 'Projected' base output; B) 'Projected' uncertainty standard deviation; C) 'Projected' uncertainty range of values; D) 'Present' base output; E) 'Present' uncertainty standard deviation; F) 'Present' uncertainty range of values; G) 'Projected' binary distribution; H) 'Present' binary distribution; I) Range size variation.

Value	Present	Projected
AUC.train	0.973	1.000
AUC.test	0.973	0.973
AUC.diff	0.027	0.027
pROC	1.446	1.408
OR.p10	0.544	0.488
CBI	0.994	0.991
perc.stable	44.455	44.455
range.size	0.279	0.279

S.3.168 *Koeleria splendens* SVM

Species: *Koeleria splendens* C.Presl

Procedure: SVM

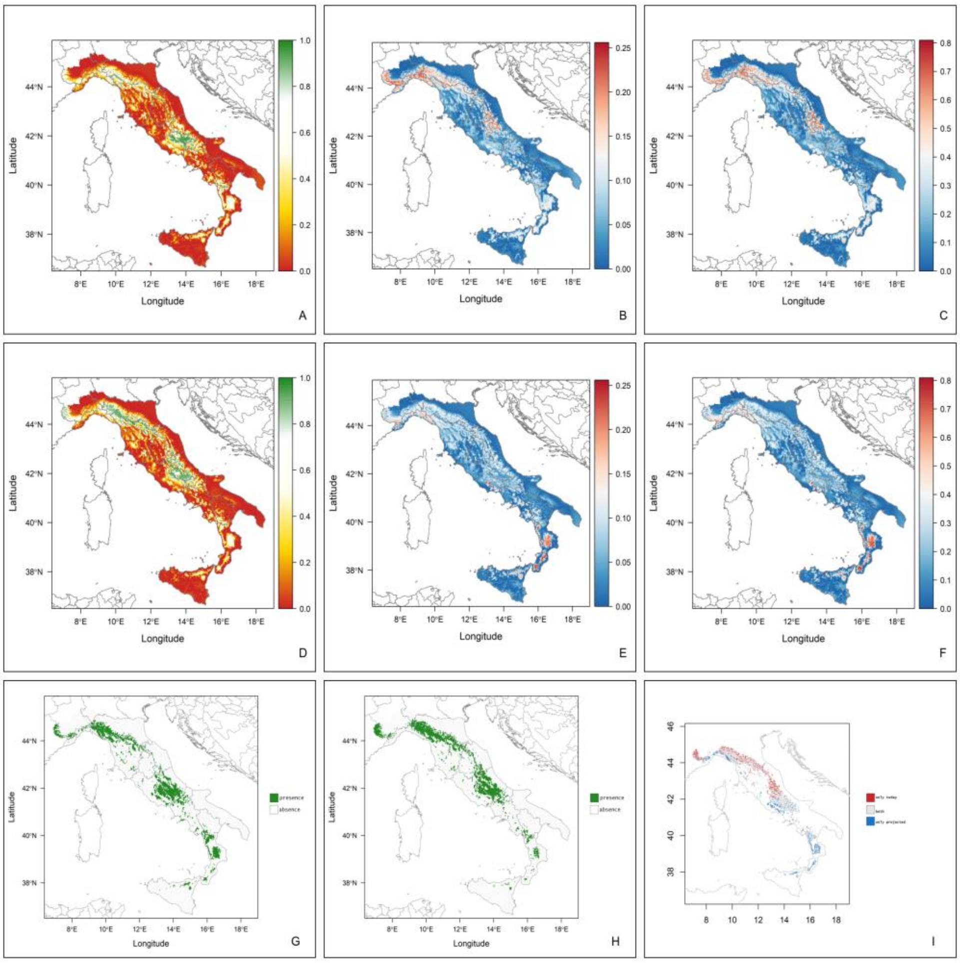

Fig. S.3.168-A) 'Projected' base output; B) 'Projected' uncertainty standard deviation; C) 'Projected' uncertainty range of values; D) 'Present' base output; E) 'Present' uncertainty standard deviation; F) 'Present' uncertainty range of values; G) 'Projected' binary distribution; H) 'Present' binary distribution; I) Range size variation.

Value	Present	Projected
AUC.train	0.885	0.894
AUC.test	0.885	0.886
AUC.diff	0.022	0.022
pROC	1.302	1.355
OR.p10	0.128	0.120
CBI	0.984	0.951
perc.stable	59.728	59.728
range.size	-0.393	-0.393

S.3.169 *Leontodon anomalus* Biomod2

Species: *Leontodon anomalus* Ball

Procedure: Biomod2

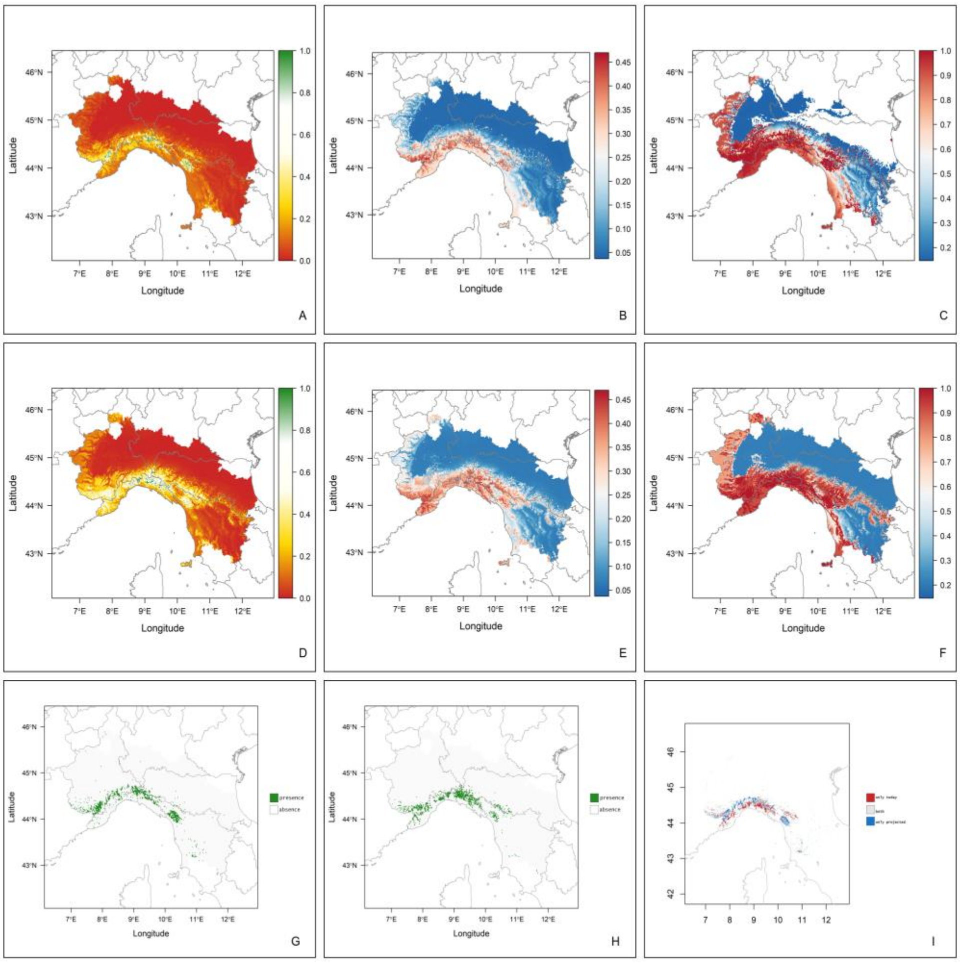

Fig. S.3.169-A) 'Projected' base output; B) 'Projected' uncertainty standard deviation; C) 'Projected' uncertainty range of values; D) 'Present' base output; E) 'Present' uncertainty standard deviation; F) 'Present' uncertainty range of values; G) 'Projected' binary distribution; H) 'Present' binary distribution; I) Range size variation.

Value	Present	Projected
AUC.train	0.923	1.000
AUC.test	0.923	0.870
AUC.diff	0.130	0.130
pROC	1.791	1.584
OR.p10	0.800	0.780
CBI	0.910	0.121
perc.stable	19.886	19.886
range.size	-0.853	-0.853

S.3.170 *Leontodon anomalus* Bioclim

Species: *Leontodon anomalus* Ball

Procedure: Bioclim

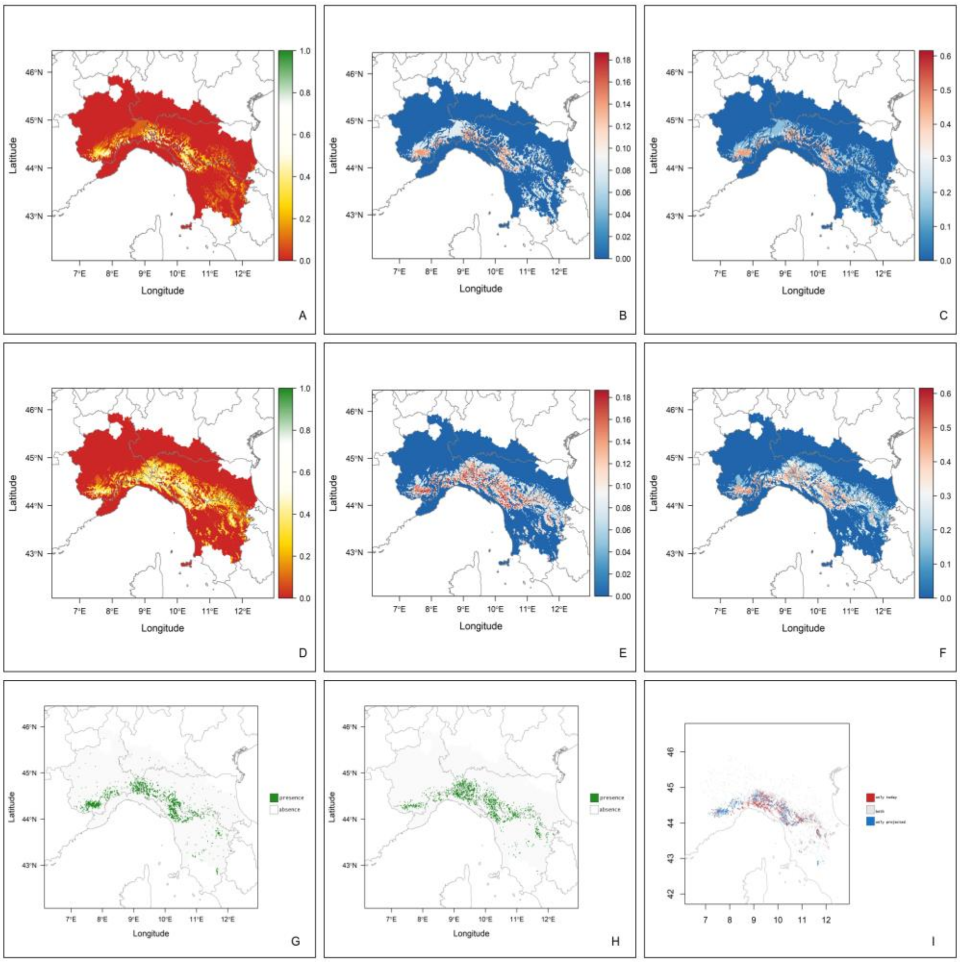

Fig. S.3.170-A) 'Projected' base output; B) 'Projected' uncertainty standard deviation; C) 'Projected' uncertainty range of values; D) 'Present' base output; E) 'Present' uncertainty standard deviation; F) 'Present' uncertainty range of values; G) 'Projected' binary distribution; H) 'Present' binary distribution; I) Range size variation.

Value	Present	Projected
AUC.train	0.742	0.930
AUC.test	0.742	0.770
AUC.diff	0.164	0.164
pROC	1.316	1.261
OR.p10	0.300	0.340
CBI	0.507	0.517
perc.stable	21.973	21.973
range.size	-1.727	-1.727

S.3.171 *Leontodon anomalus* Domain

Species: *Leontodon anomalus* Ball

Procedure: Domain

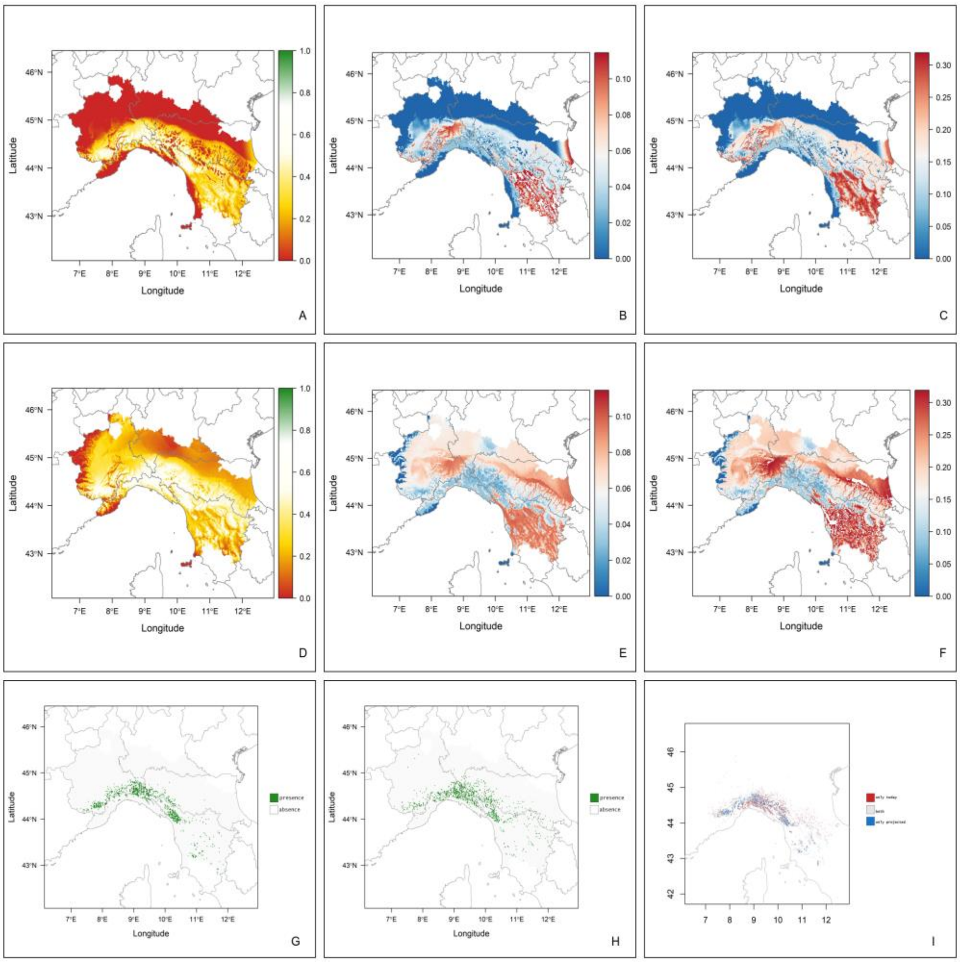

Fig. S.3.171-A) 'Projected' base output; B) 'Projected' uncertainty standard deviation; C) 'Projected' uncertainty range of values; D) 'Present' base output; E) 'Present' uncertainty standard deviation; F) 'Present' uncertainty range of values; G) 'Projected' binary distribution; H) 'Present' binary distribution; I) Range size variation.

Value	Present	Projected
AUC.train	0.842	0.933
AUC.test	0.842	0.907
AUC.diff	0.038	0.038
pROC	1.607	1.712
OR.p10	0.380	0.240
CBI	0.913	0.816
perc.stable	19.235	19.235
range.size	-5.312	-5.312

S.3.172 *Leontodon anomalus* Maxent

Species: *Leontodon anomalus* Ball

Procedure: Maxent

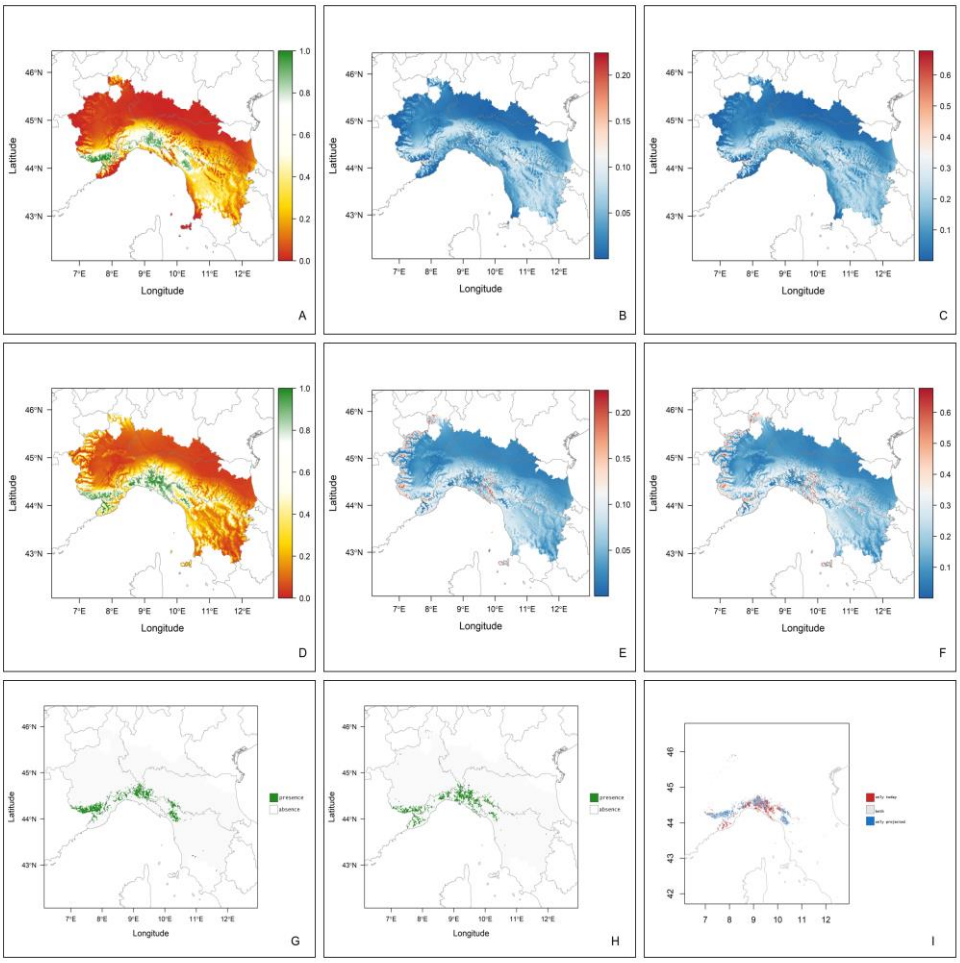

Fig. S.3.172-A) 'Projected' base output; B) 'Projected' uncertainty standard deviation; C) 'Projected' uncertainty range of values; D) 'Present' base output; E) 'Present' uncertainty standard deviation; F) 'Present' uncertainty range of values; G) 'Projected' binary distribution; H) 'Present' binary distribution; I) Range size variation.

Value	Present	Projected
AUC.train	0.880	0.914
AUC.test	0.880	0.932
AUC.diff	0.024	0.024
pROC	1.688	1.798
OR.p10	0.320	0.180
CBI	0.918	0.970
perc.stable	24.676	24.676
range.size	3.102	3.102

S.3.173 *Leontodon anomalus* KDE

Species: *Leontodon anomalus* Ball

Procedure: KDE

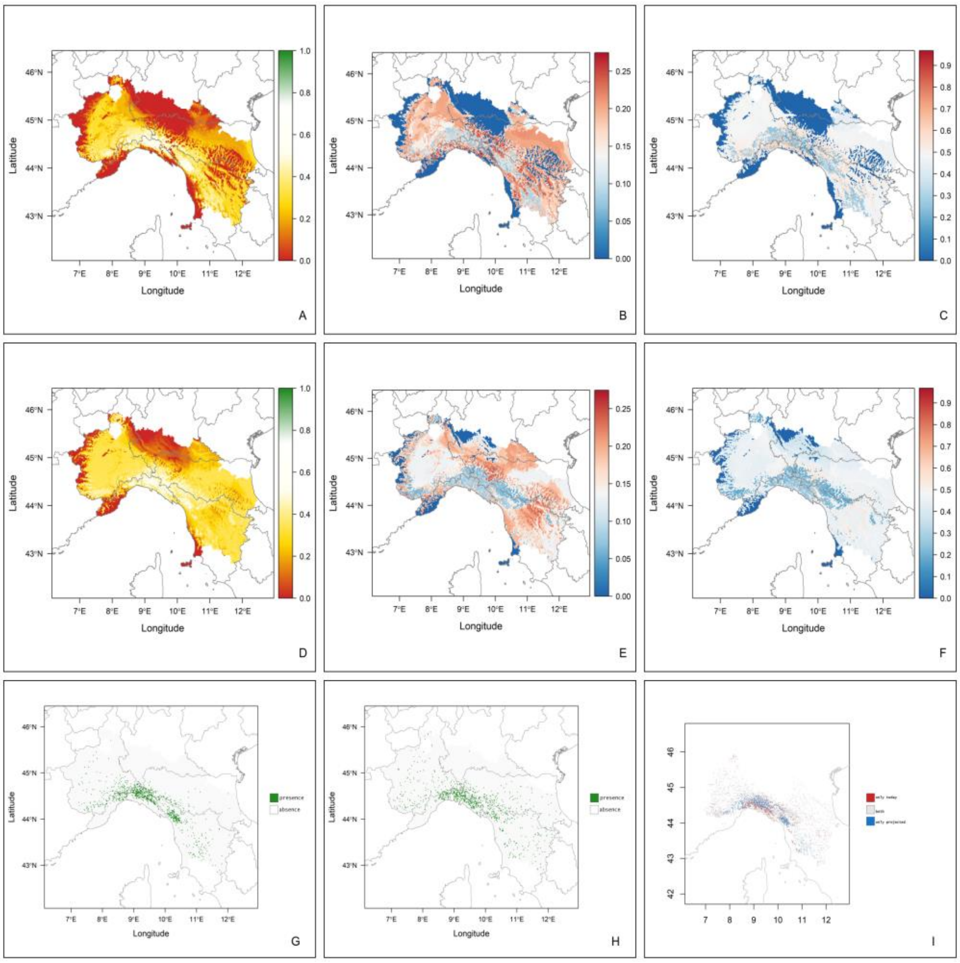

Fig. S.3.173-A) 'Projected' base output; B) 'Projected' uncertainty standard deviation; C) 'Projected' uncertainty range of values; D) 'Present' base output; E) 'Present' uncertainty standard deviation; F) 'Present' uncertainty range of values; G) 'Projected' binary distribution; H) 'Present' binary distribution; I) Range size variation.

Value	Present	Projected
AUC.train	0.853	0.928
AUC.test	0.853	0.851
AUC.diff	0.075	0.084
pROC	1.592	1.574
OR.p10	0.340	0.320
CBI	0.995	0.943
perc.stable	11.311	11.311
range.size	-0.051	-0.051

S.3.174 *Leontodon anomalus* CTA

Species: *Leontodon anomalus* Ball

Procedure: CTA

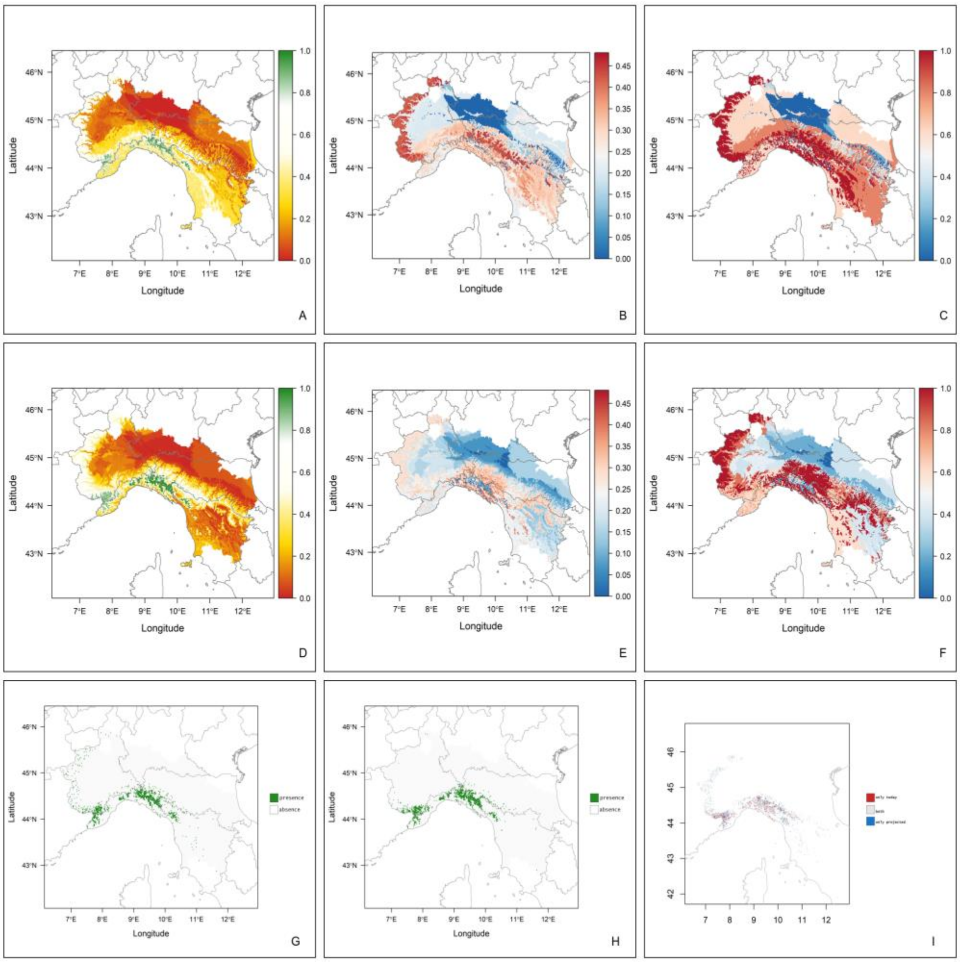

Fig. S.3.174-A) 'Projected' base output; B) 'Projected' uncertainty standard deviation; C) 'Projected' uncertainty range of values; D) 'Present' base output; E) 'Present' uncertainty standard deviation; F) 'Present' uncertainty range of values; G) 'Projected' binary distribution; H) 'Present' binary distribution; I) Range size variation.

Value	Present	Projected
AUC.train	0.844	0.934
AUC.test	0.844	0.780
AUC.diff	0.159	0.159
pROC	1.204	1.179
OR.p10	0.260	0.340
CBI	0.956	0.935
perc.stable	49.399	49.399
range.size	1.429	1.429

S.3.175 *Leontodon anomalus* FDA

Species: *Leontodon anomalus* Ball

Procedure: FDA

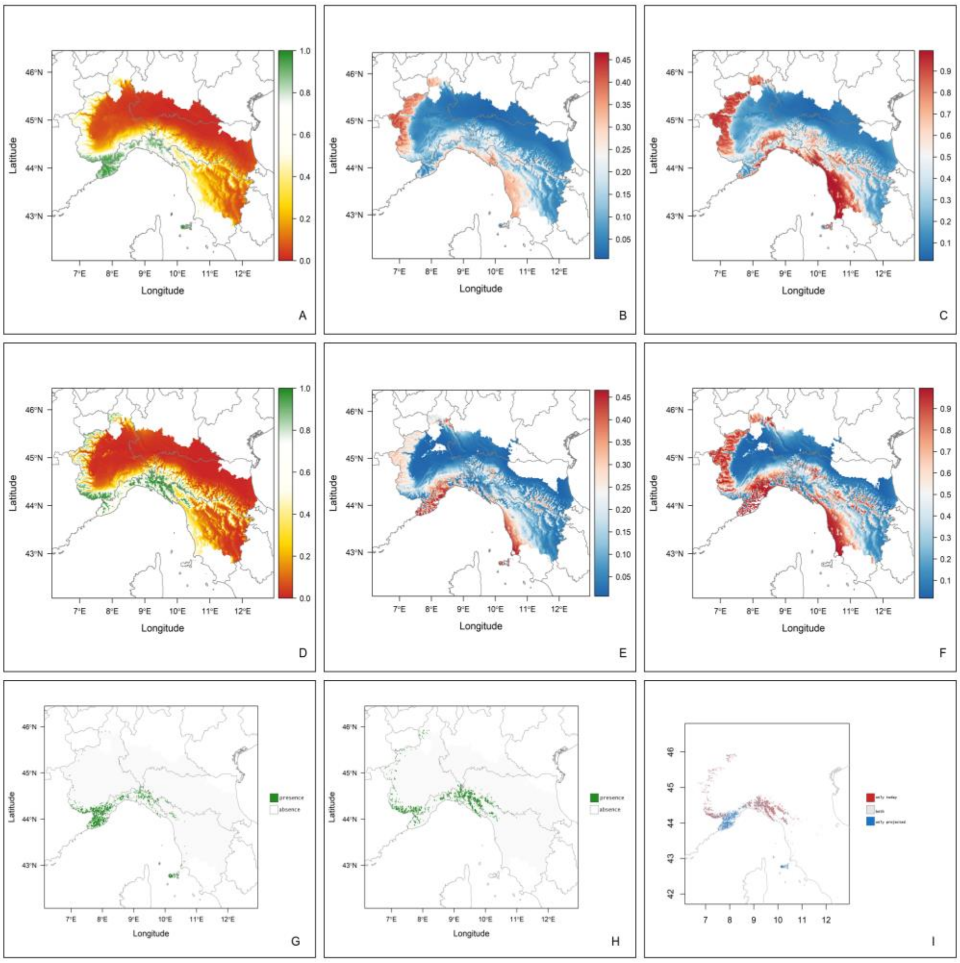

Fig. S.3.175-A) 'Projected' base output; B) 'Projected' uncertainty standard deviation; C) 'Projected' uncertainty range of values; D) 'Present' base output; E) 'Present' uncertainty standard deviation; F) 'Present' uncertainty range of values; G) 'Projected' binary distribution; H) 'Present' binary distribution; I) Range size variation.

Value	Present	Projected
AUC.train	0.862	0.864
AUC.test	0.862	0.899
AUC.diff	0.007	0.007
pROC	1.581	1.641
OR.p10	0.360	0.180
CBI	0.853	0.893
perc.stable	19.733	19.733
range.size	-1.130	-1.130

S.3.176 *Leontodon anomalus* GAM

Species: *Leontodon anomalus* Ball

Procedure: GAM

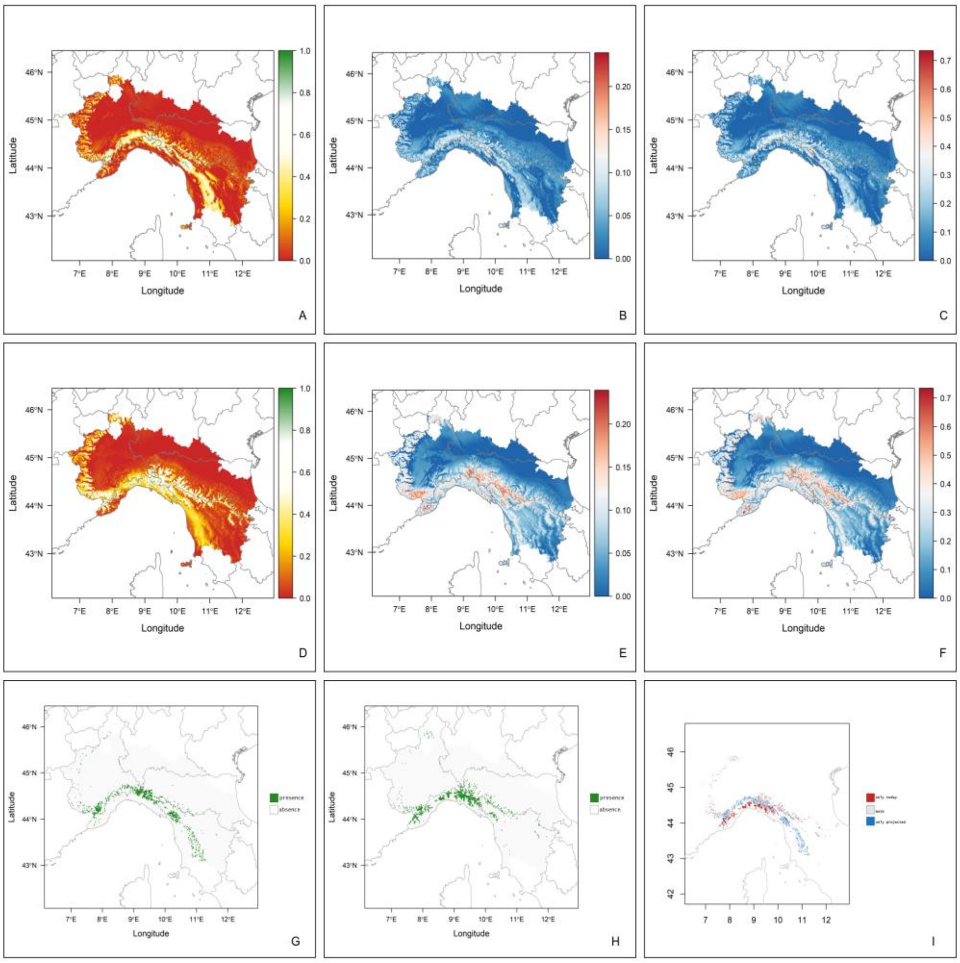

Fig. S.3.176-A) 'Projected' base output; B) 'Projected' uncertainty standard deviation; C) 'Projected' uncertainty range of values; D) 'Present' base output; E) 'Present' uncertainty standard deviation; F) 'Present' uncertainty range of values; G) 'Projected' binary distribution; H) 'Present' binary distribution; I) Range size variation.

Value	Present	Projected
AUC.train	0.878	0.922
AUC.test	0.878	0.931
AUC.diff	0.023	0.023
pROC	1.672	1.773
OR.p10	0.280	0.280
CBI	0.975	0.787
perc.stable	19.404	19.404
range.size	1.285	1.285

S.3.177 *Leontodon anomalus* GLM

Species: *Leontodon anomalus* Ball

Procedure: GLM

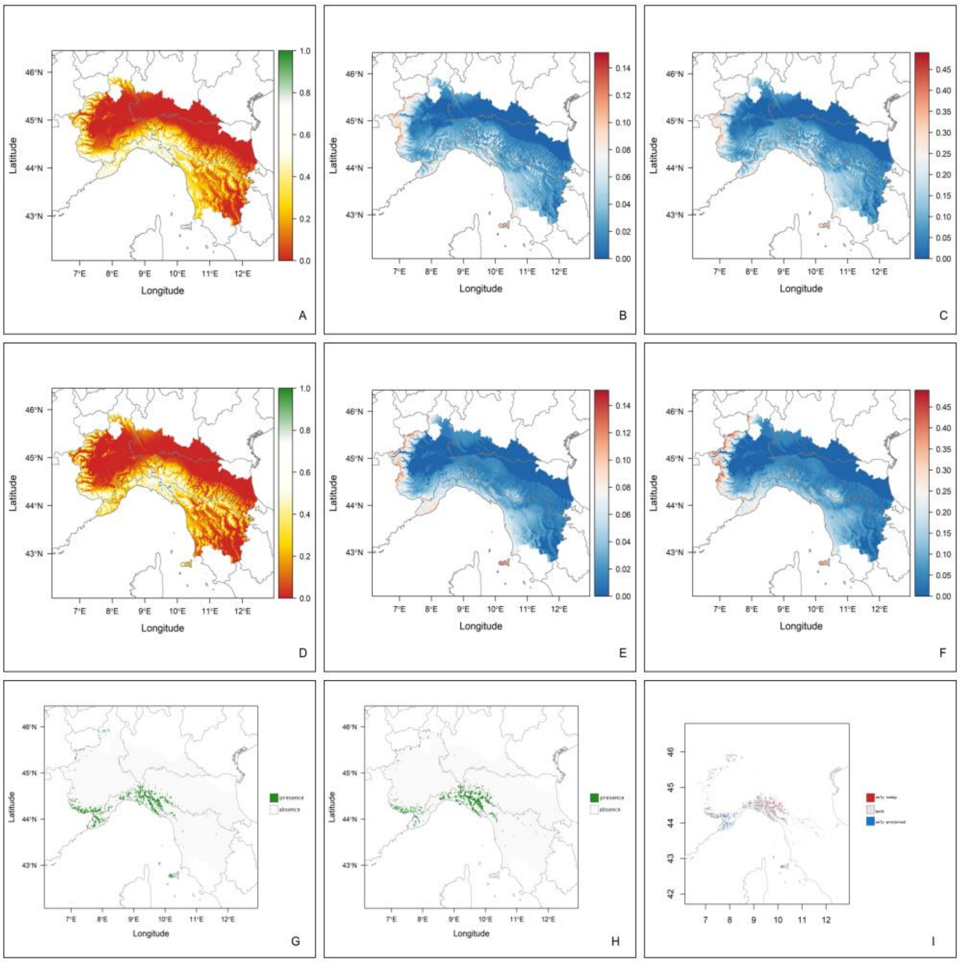

Fig. S.3.177-A) 'Projected' base output; B) 'Projected' uncertainty standard deviation; C) 'Projected' uncertainty range of values; D) 'Present' base output; E) 'Present' uncertainty standard deviation; F) 'Present' uncertainty range of values; G) 'Projected' binary distribution; H) 'Present' binary distribution; I) Range size variation.

Value	Present	Projected
AUC.train	0.867	0.830
AUC.test	0.867	0.838
AUC.diff	0.035	0.035
pROC	1.656	1.617
OR.p10	0.300	0.180
CBI	0.987	0.965
perc.stable	39.553	39.553
range.size	0.383	0.383

S.3.178 *Leontodon anomalus* MARS

Species: *Leontodon anomalus* Ball

Procedure: MARS

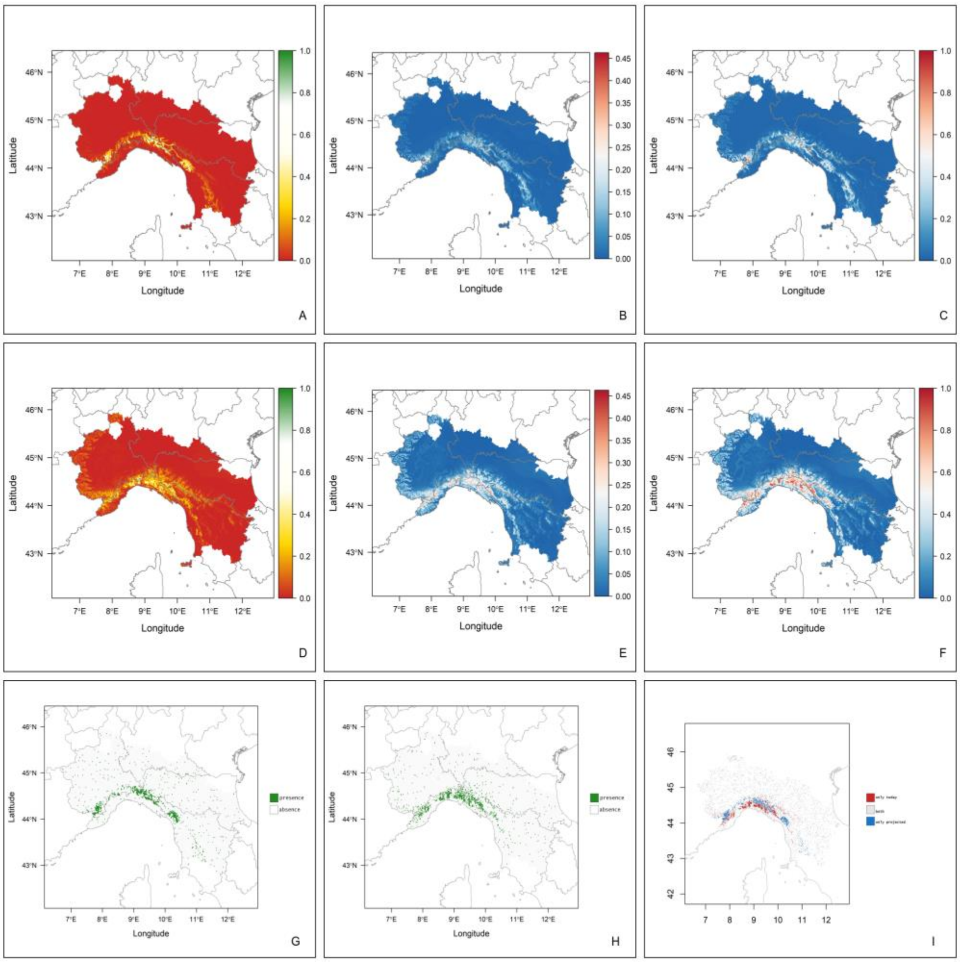

Fig. S.3.178-A) 'Projected' base output; B) 'Projected' uncertainty standard deviation; C) 'Projected' uncertainty range of values; D) 'Present' base output; E) 'Present' uncertainty standard deviation; F) 'Present' uncertainty range of values; G) 'Projected' binary distribution; H) 'Present' binary distribution; I) Range size variation.

Value	Present	Projected
AUC.train	0.790	0.897
AUC.test	0.790	0.876
AUC.diff	0.063	0.063
pROC	1.426	1.583
OR.p10	0.480	0.240
CBI	0.933	0.201
perc.stable	7.599	7.599
range.size	-3.631	-3.631

S.3.179 *Leontodon anomalus* RF

Species: *Leontodon anomalus* Ball

Procedure: RF

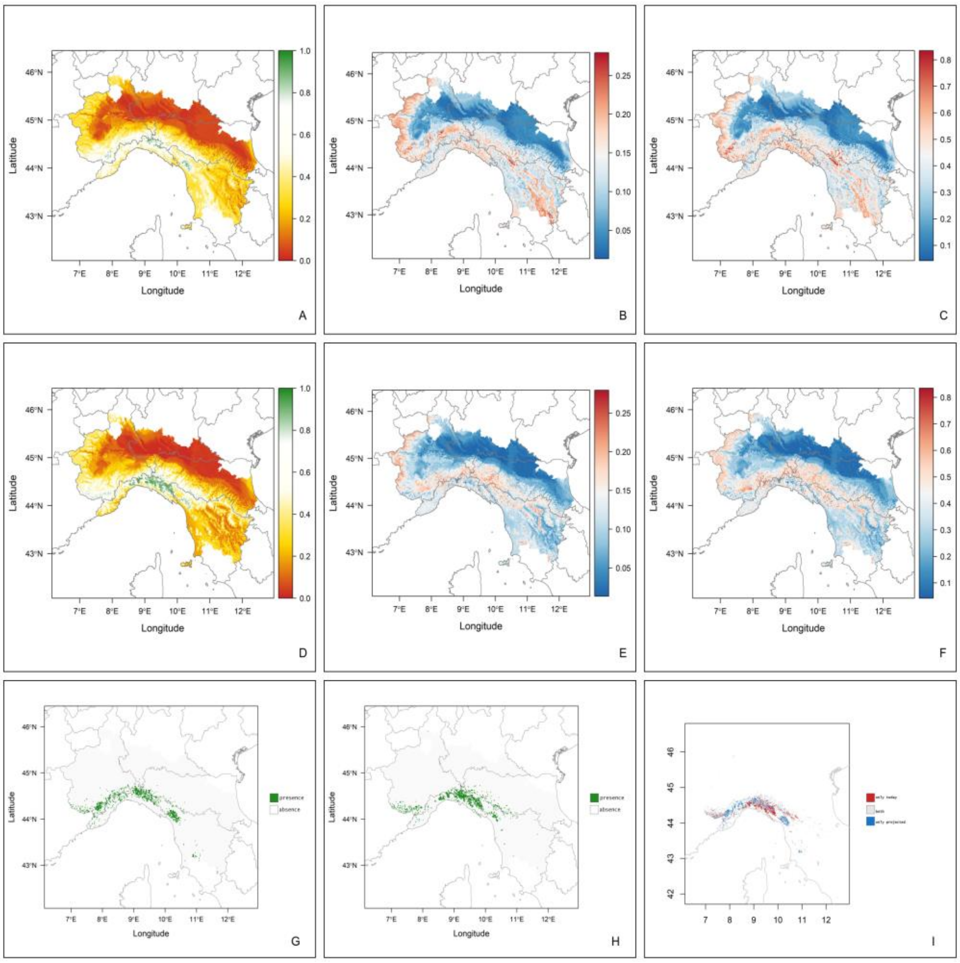

Fig. S.3.179-A) 'Projected' base output; B) 'Projected' uncertainty standard deviation; C) 'Projected' uncertainty range of values; D) 'Present' base output; E) 'Present' uncertainty standard deviation; F) 'Present' uncertainty range of values; G) 'Projected' binary distribution; H) 'Present' binary distribution; I) Range size variation.

Value	Present	Projected
AUC.train	0.981	1.000
AUC.test	0.981	0.958
AUC.diff	0.042	0.042
pROC	1.662	1.675
OR.p10	0.540	0.420
CBI	0.985	0.892
perc.stable	17.957	17.957
range.size	-0.314	-0.314

S.3.180 *Leontodon anomalus* SVM

Species: *Leontodon anomalus* Ball

Procedure: SVM

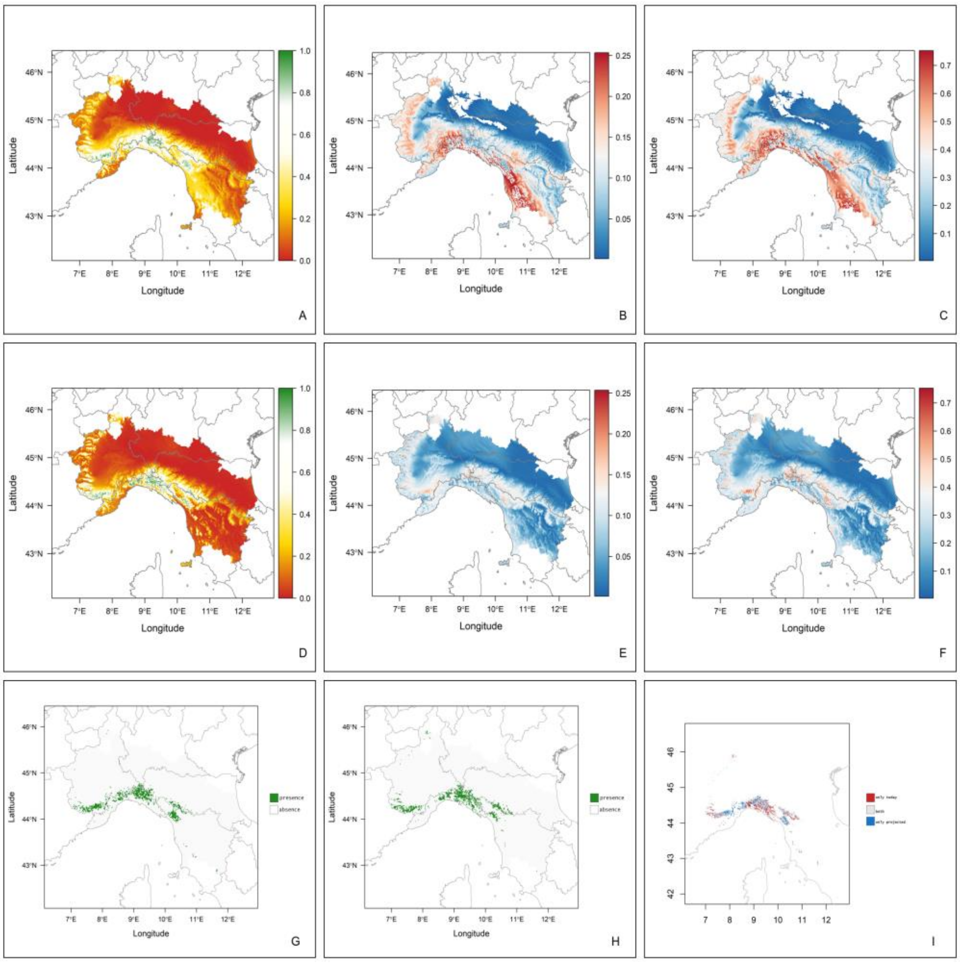

Fig. S.3.180-A) 'Projected' base output; B) 'Projected' uncertainty standard deviation; C) 'Projected' uncertainty range of values; D) 'Present' base output; E) 'Present' uncertainty standard deviation; F) 'Present' uncertainty range of values; G) 'Projected' binary distribution; H) 'Present' binary distribution; I) Range size variation.

Value	Present	Projected
AUC.train	0.932	0.977
AUC.test	0.932	0.949
AUC.diff	0.031	0.031
pROC	1.704	1.736
OR.p10	0.300	0.320
CBI	0.962	0.947
perc.stable	31.342	31.342
range.size	-1.187	-1.187

S.3.181 *Linaria purpurea* Biomod2

Species: *Linaria purpurea* (L.) Mill.

Procedure: Biomod2

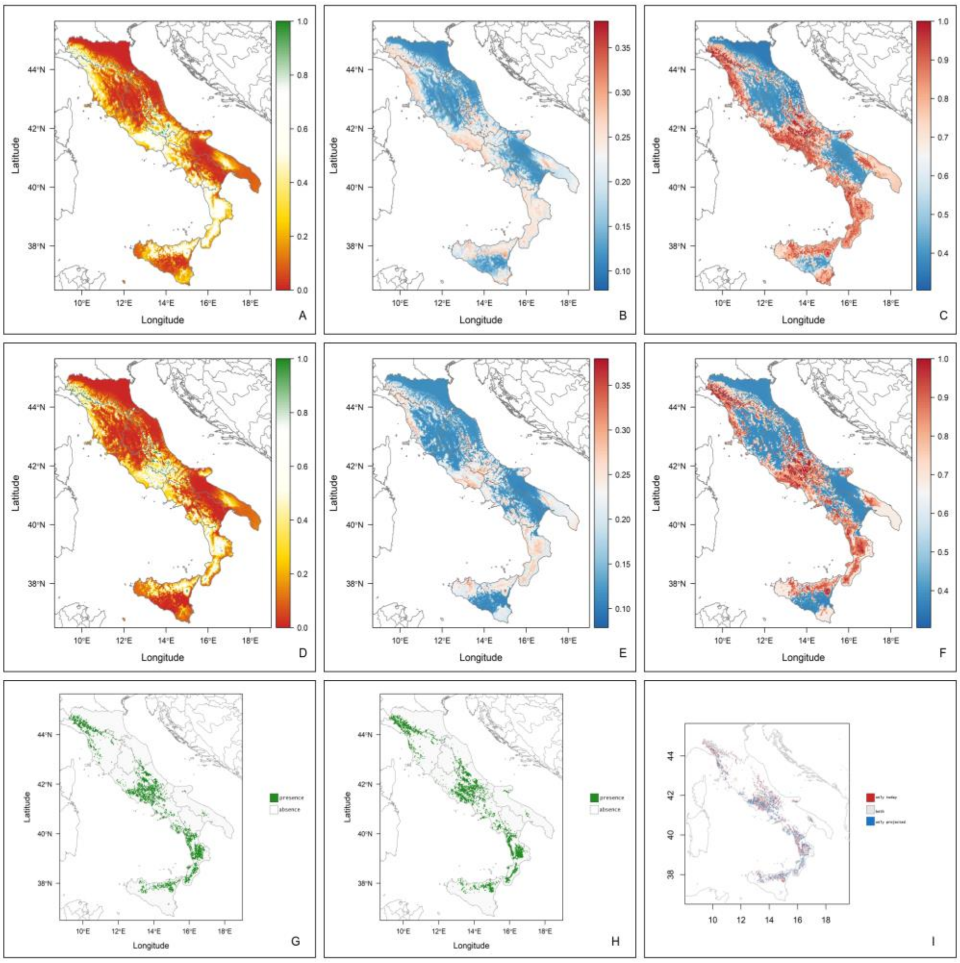

Fig. S.3.181-A) 'Projected' base output; B) 'Projected' uncertainty standard deviation; C) 'Projected' uncertainty range of values; D) 'Present' base output; E) 'Present' uncertainty standard deviation; F) 'Present' uncertainty range of values; G) 'Projected' binary distribution; H) 'Present' binary distribution; I) Range size variation.

Value	Present	Projected
AUC.train	0.788	0.948
AUC.test	0.788	0.783
AUC.diff	0.165	0.165
pROC	1.210	1.238
OR.p10	0.543	0.459
CBI	0.983	0.967
perc.stable	48.590	48.590
range.size	1.386	1.386

S.3.182 *Linaria purpurea* Bioclim

Species: *Linaria purpurea* (L.) Mill.

Procedure: Bioclim

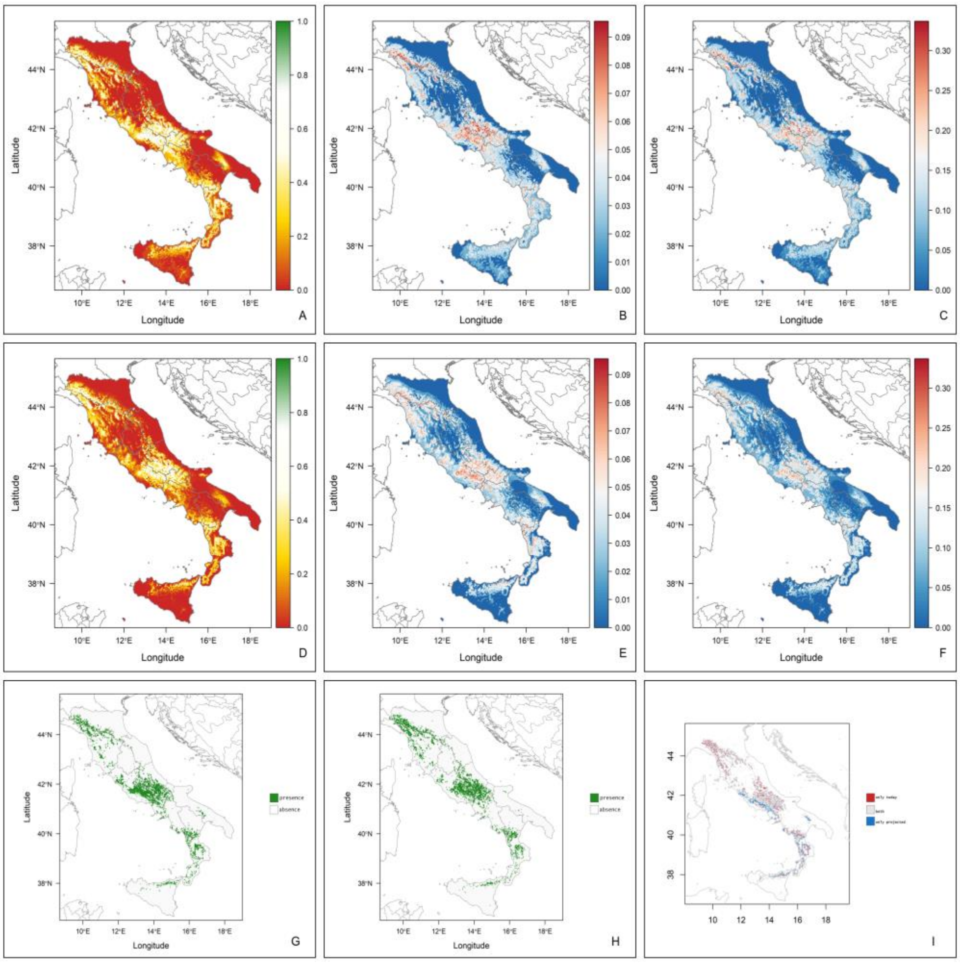

Fig. S.3.182-A) 'Projected' base output; B) 'Projected' uncertainty standard deviation; C) 'Projected' uncertainty range of values; D) 'Present' base output; E) 'Present' uncertainty standard deviation; F) 'Present' uncertainty range of values; G) 'Projected' binary distribution; H) 'Present' binary distribution; I) Range size variation.

Value	Present	Projected
AUC.train	0.674	0.737
AUC.test	0.674	0.696
AUC.diff	0.049	0.049
pROC	1.060	1.137
OR.p10	0.235	0.234
CBI	0.852	0.910
perc.stable	49.965	49.965
range.size	-0.335	-0.335

S.3.183 *Linaria purpurea* Domain

Species: *Linaria purpurea* (L.) Mill.

Procedure: Domain

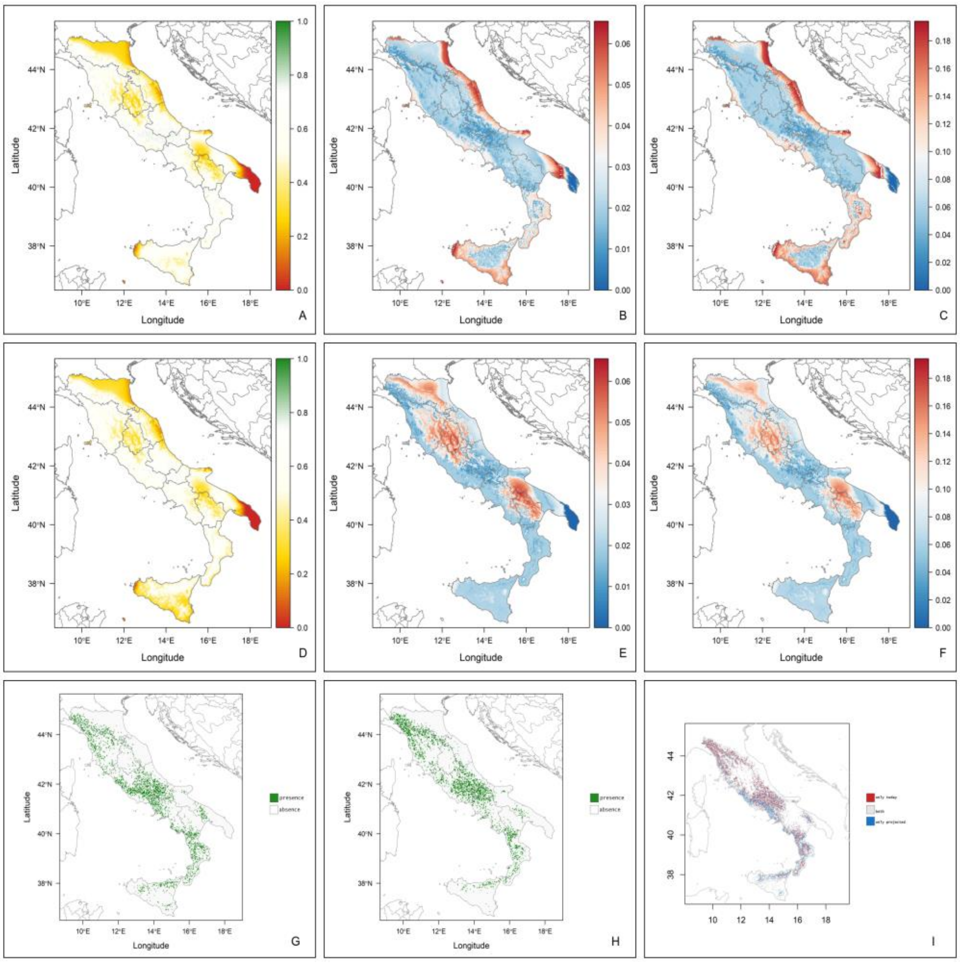

Fig. S.3.183-A) 'Projected' base output; B) 'Projected' uncertainty standard deviation; C) 'Projected' uncertainty range of values; D) 'Present' base output; E) 'Present' uncertainty standard deviation; F) 'Present' uncertainty range of values; G) 'Projected' binary distribution; H) 'Present' binary distribution; I) Range size variation.

Value	Present	Projected
AUC.train	0.738	0.717
AUC.test	0.738	0.687
AUC.diff	0.033	0.033
pROC	1.266	1.179
OR.p10	0.113	0.163
CBI	0.997	0.987
perc.stable	21.390	21.390
range.size	-0.085	-0.085

S.3.184 *Linaria purpurea* Maxent

Species: *Linaria purpurea* (L.) Mill.

Procedure: Maxent

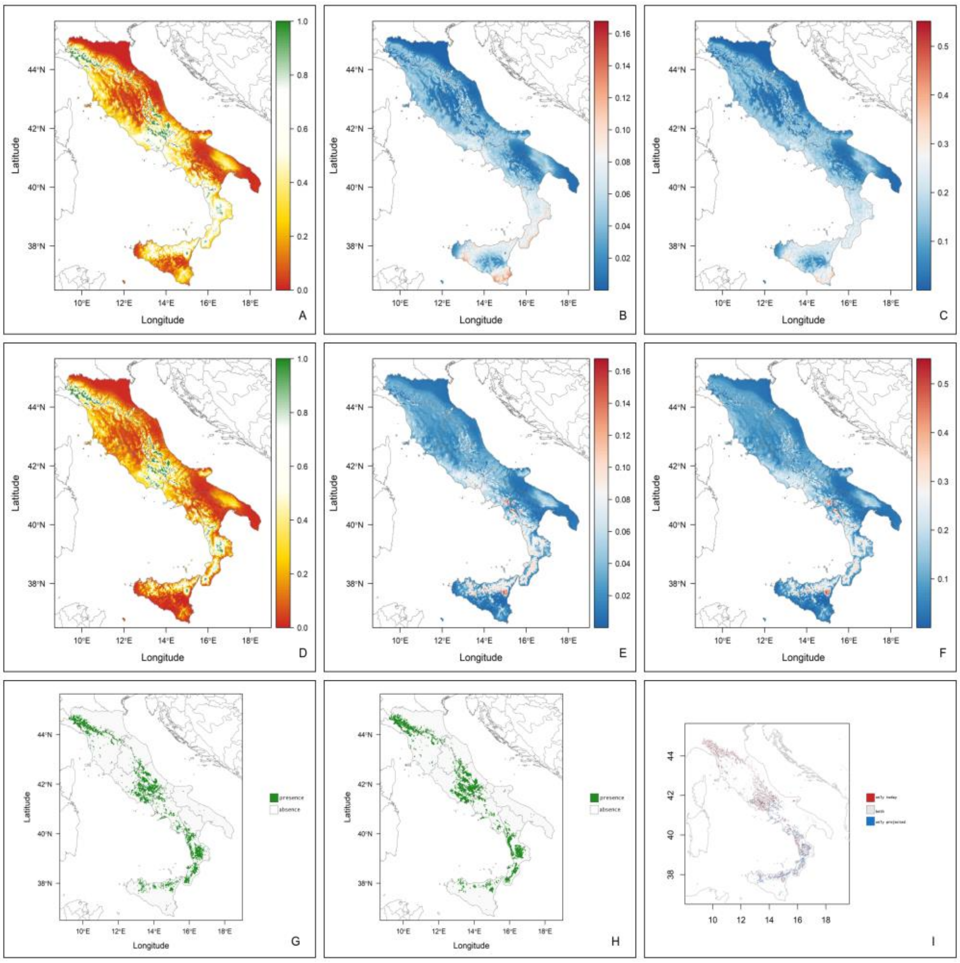

Fig. S.3.184-A) 'Projected' base output; B) 'Projected' uncertainty standard deviation; C) 'Projected' uncertainty range of values; D) 'Present' base output; E) 'Present' uncertainty standard deviation; F) 'Present' uncertainty range of values; G) 'Projected' binary distribution; H) 'Present' binary distribution; I) Range size variation.

Value	Present	Projected
AUC.train	0.840	0.818
AUC.test	0.840	0.797
AUC.diff	0.033	0.033
pROC	1.315	1.303
OR.p10	0.122	0.182
CBI	0.973	0.988
perc.stable	60.846	60.846
range.size	-0.521	-0.521

S.3.185 *Linaria purpurea* KDE

Species: *Linaria purpurea* (L.) Mill.

Procedure: KDE

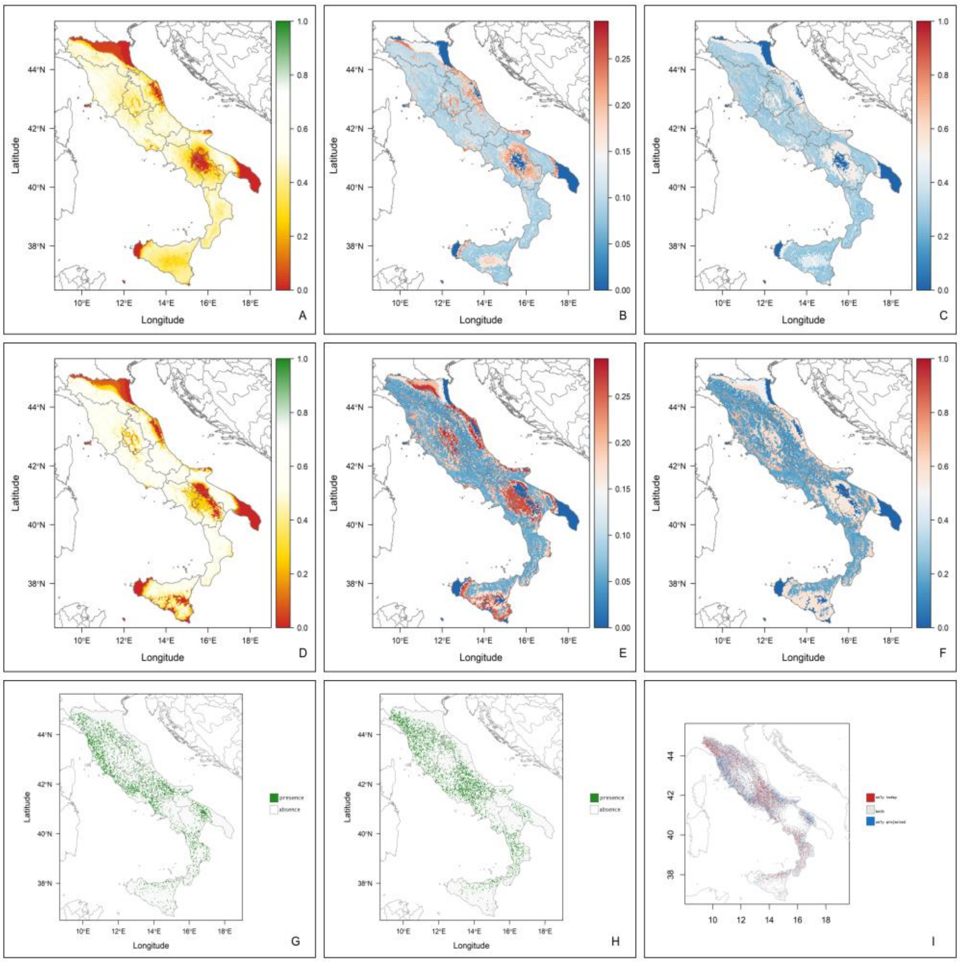

Fig. S.3.185-A) 'Projected' base output; B) 'Projected' uncertainty standard deviation; C) 'Projected' uncertainty range of values; D) 'Present' base output; E) 'Present' uncertainty standard deviation; F) 'Present' uncertainty range of values; G) 'Projected' binary distribution; H) 'Present' binary distribution; I) Range size variation.

Value	Present	Projected
AUC.train	0.762	0.646
AUC.test	0.762	0.561
AUC.diff	0.035	0.093
pROC	1.176	1.078
OR.p10	0.126	0.183
CBI	0.898	0.870
perc.stable	12.441	12.441
range.size	-0.447	-0.447

S.3.186 *Linaria purpurea* CTA

Species: *Linaria purpurea* (L.) Mill.

Procedure: CTA

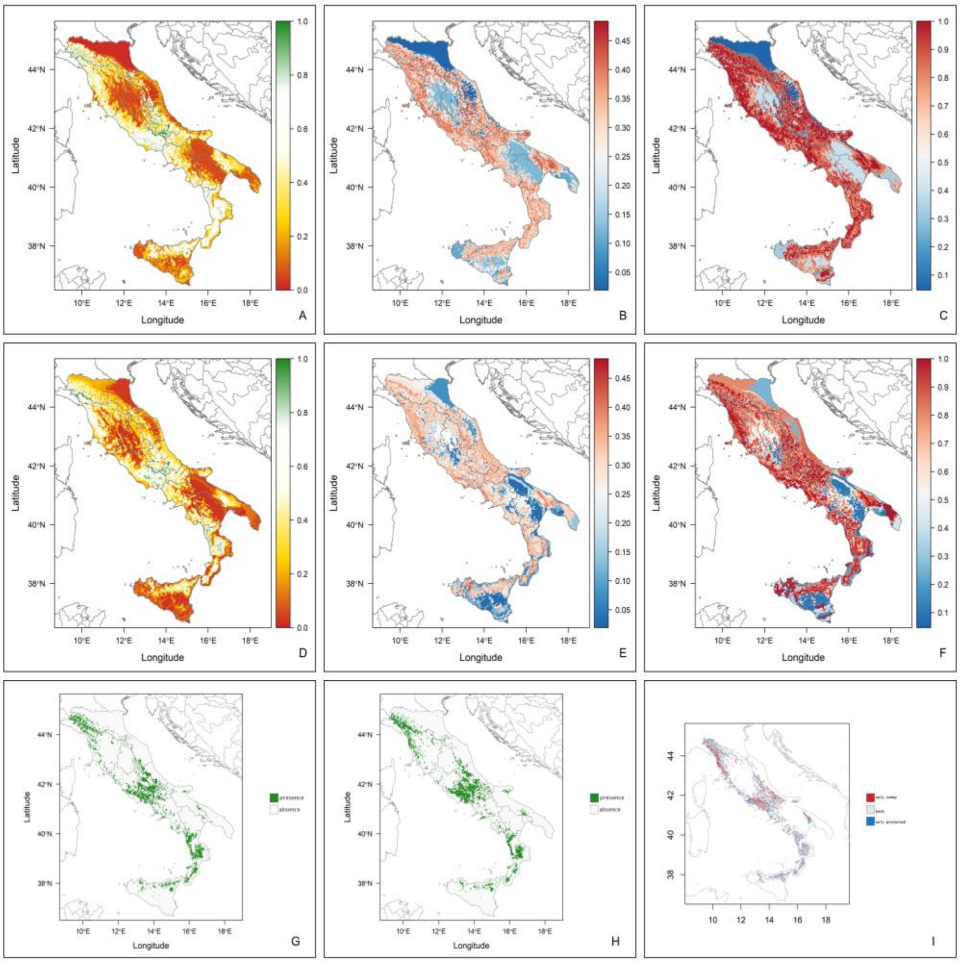

Fig. S.3.186-A) 'Projected' base output; B) 'Projected' uncertainty standard deviation; C) 'Projected' uncertainty range of values; D) 'Present' base output; E) 'Present' uncertainty standard deviation; F) 'Present' uncertainty range of values; G) 'Projected' binary distribution; H) 'Present' binary distribution; I) Range size variation.

Value	Present	Projected
AUC.train	0.801	0.945
AUC.test	0.801	0.811
AUC.diff	0.134	0.134
pROC	1.099	1.078
OR.p10	0.304	0.329
CBI	0.993	0.981
perc.stable	38.277	38.277
range.size	-0.252	-0.252

S.3.187 *Linaria purpurea* FDA

Species: *Linaria purpurea* (L.) Mill.

Procedure: FDA

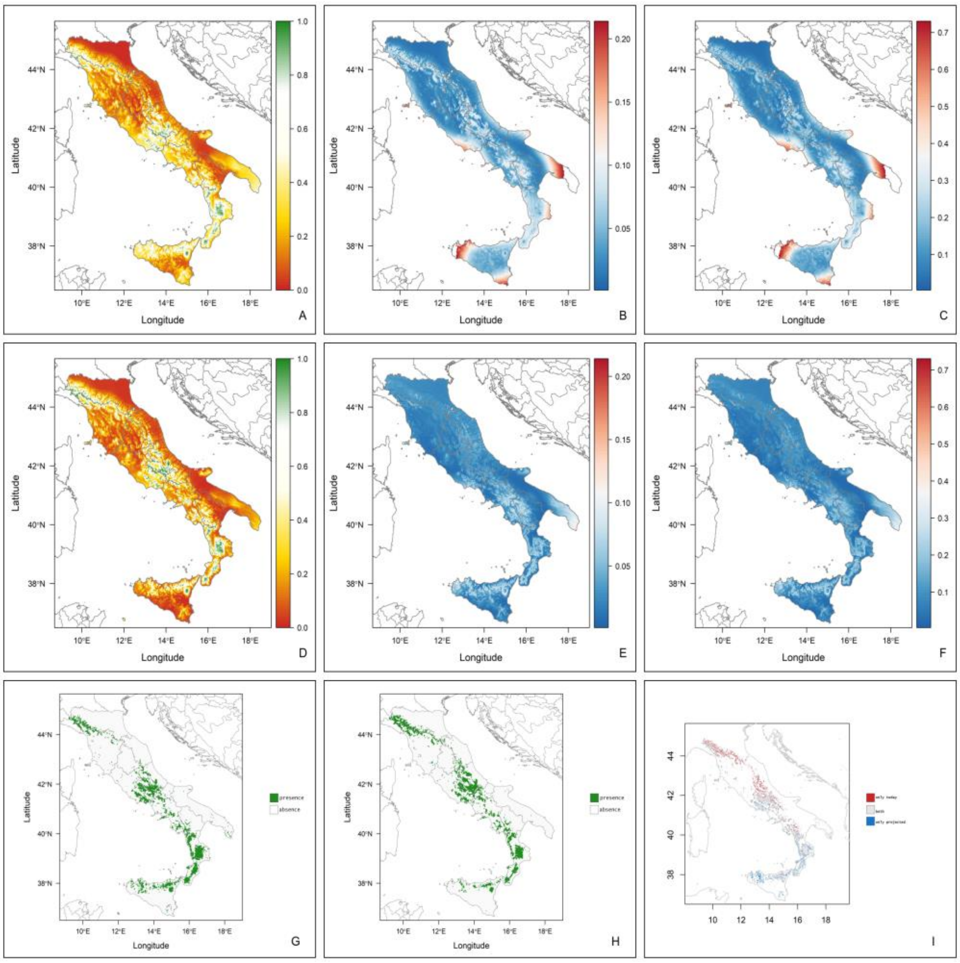

Fig. S.3.187-A) 'Projected' base output; B) 'Projected' uncertainty standard deviation; C) 'Projected' uncertainty range of values; D) 'Present' base output; E) 'Present' uncertainty standard deviation; F) 'Present' uncertainty range of values; G) 'Projected' binary distribution; H) 'Present' binary distribution; I) Range size variation.

Value	Present	Projected
AUC.train	0.855	0.781
AUC.test	0.855	0.776
AUC.diff	0.024	0.024
pROC	1.318	1.233
OR.p10	0.100	0.154
CBI	0.993	0.985
perc.stable	60.467	60.467
range.size	0.417	0.417

S.3.188 *Linaria purpurea* GAM

Species: *Linaria purpurea* (L.) Mill.

Procedure: GAM

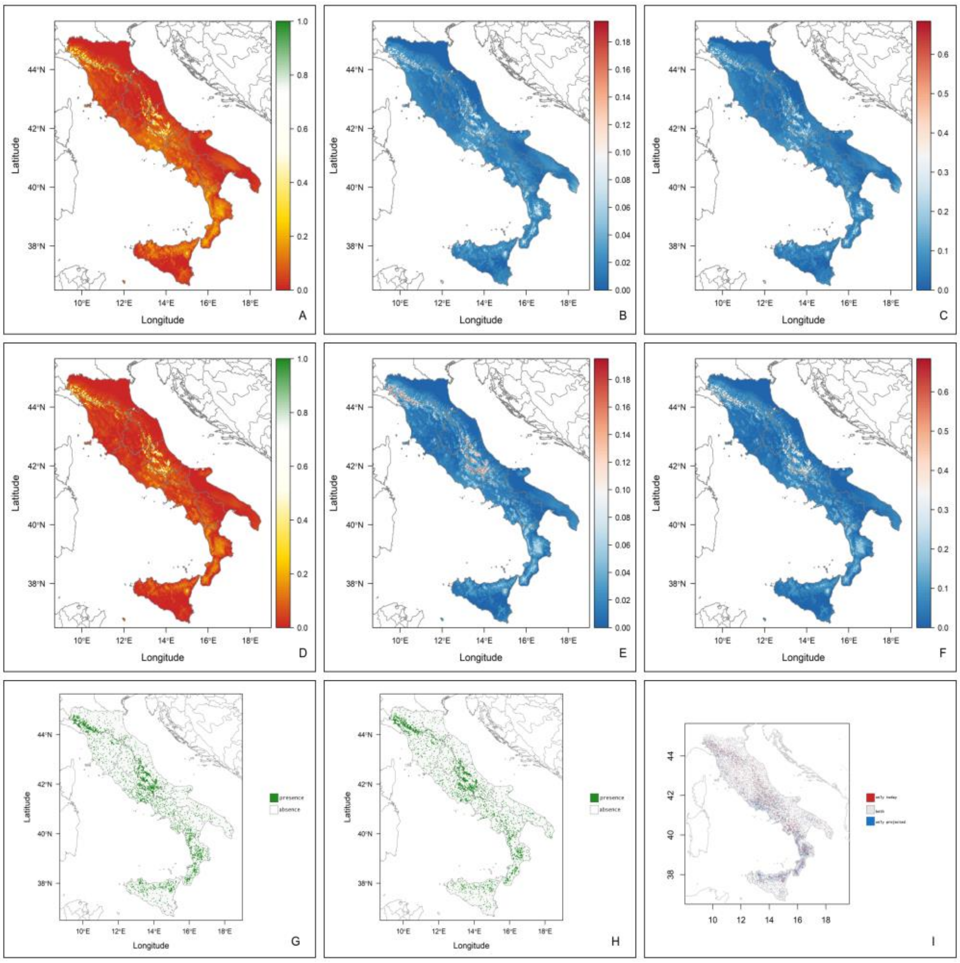

Fig. S.3.188-A) 'Projected' base output; B) 'Projected' uncertainty standard deviation; C) 'Projected' uncertainty range of values; D) 'Present' base output; E) 'Present' uncertainty standard deviation; F) 'Present' uncertainty range of values; G) 'Projected' binary distribution; H) 'Present' binary distribution; I) Range size variation.

Value	Present	Projected
AUC.train	0.809	0.810
AUC.test	0.809	0.760
AUC.diff	0.053	0.053
pROC	1.257	1.239
OR.p10	0.178	0.185
CBI	0.909	0.917
perc.stable	21.997	21.997
range.size	1.003	1.003

S.3.189 *Linaria purpurea* GLM

Species: *Linaria purpurea* (L.) Mill.

Procedure: GLM

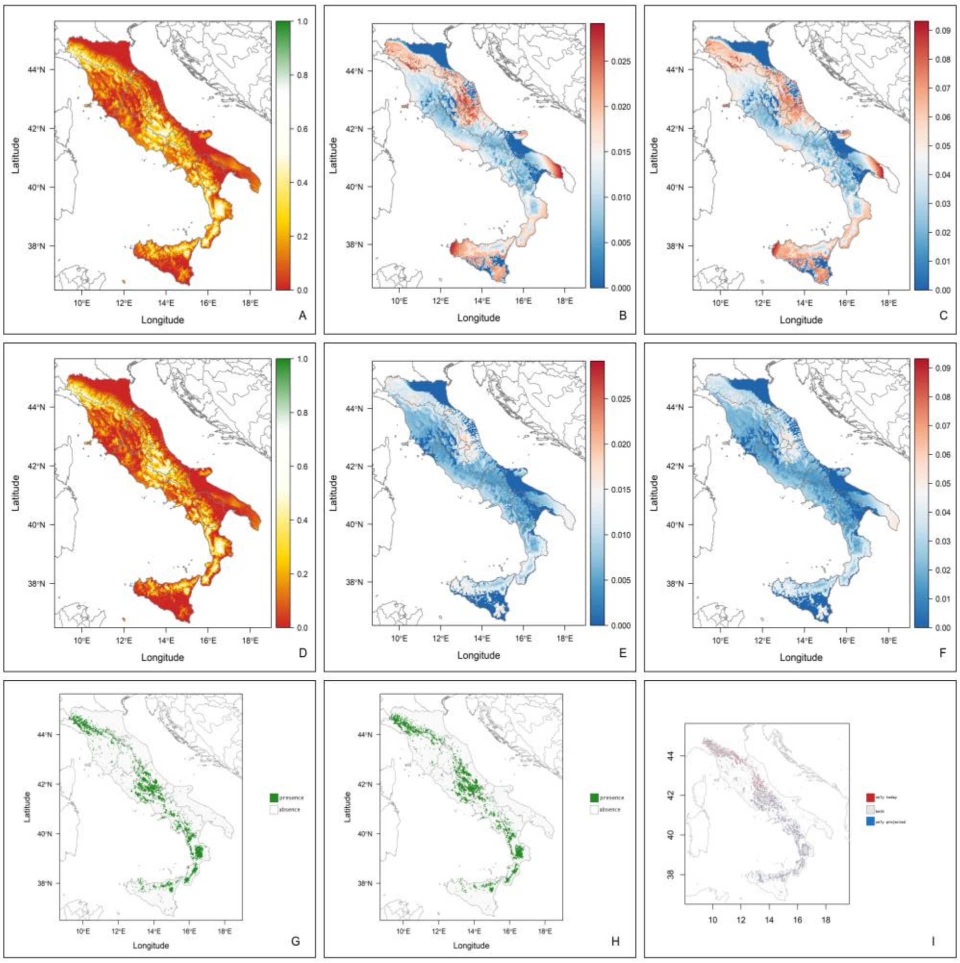

Fig. S.3.189-A) 'Projected' base output; B) 'Projected' uncertainty standard deviation; C) 'Projected' uncertainty range of values; D) 'Present' base output; E) 'Present' uncertainty standard deviation; F) 'Present' uncertainty range of values; G) 'Projected' binary distribution; H) 'Present' binary distribution; I) Range size variation.

Value	Present	Projected
AUC.train	0.819	0.753
AUC.test	0.819	0.758
AUC.diff	0.013	0.013
pROC	1.272	1.224
OR.p10	0.152	0.085
CBI	0.925	0.928
perc.stable	51.271	51.271
range.size	0.348	0.348

S.3.190 *Linaria purpurea* MARS

Species: *Linaria purpurea* (L.) Mill.

Procedure: MARS

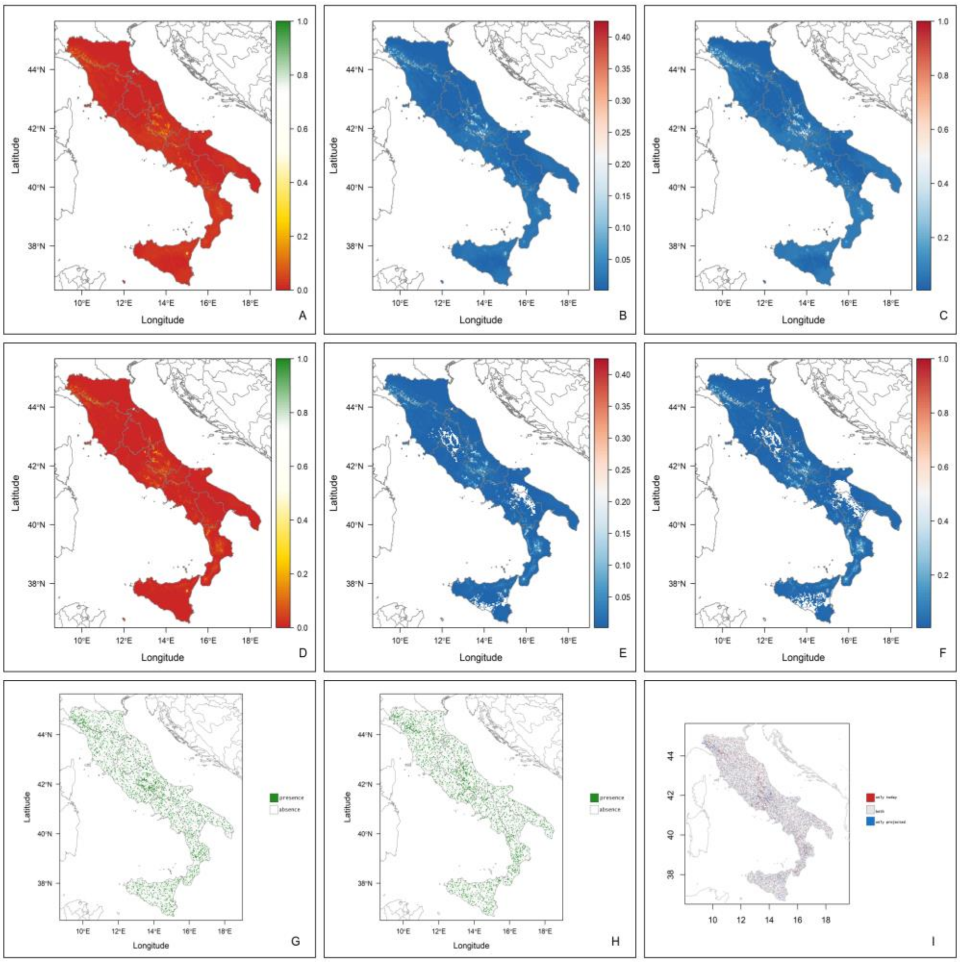

Fig. S.3.190-A) 'Projected' base output; B) 'Projected' uncertainty standard deviation; C) 'Projected' uncertainty range of values; D) 'Present' base output; E) 'Present' uncertainty standard deviation; F) 'Present' uncertainty range of values; G) 'Projected' binary distribution; H) 'Present' binary distribution; I) Range size variation.

Value	Present	Projected
AUC.train	0.820	0.811
AUC.test	0.820	0.728
AUC.diff	0.085	0.085
pROC	1.223	1.143
OR.p10	0.130	0.221
CBI	0.817	0.813
perc.stable	8.791	8.791
range.size	-1.847	-1.847

S.3.191 *Linaria purpurea* RF

Species: *Linaria purpurea* (L.) Mill.

Procedure: RF

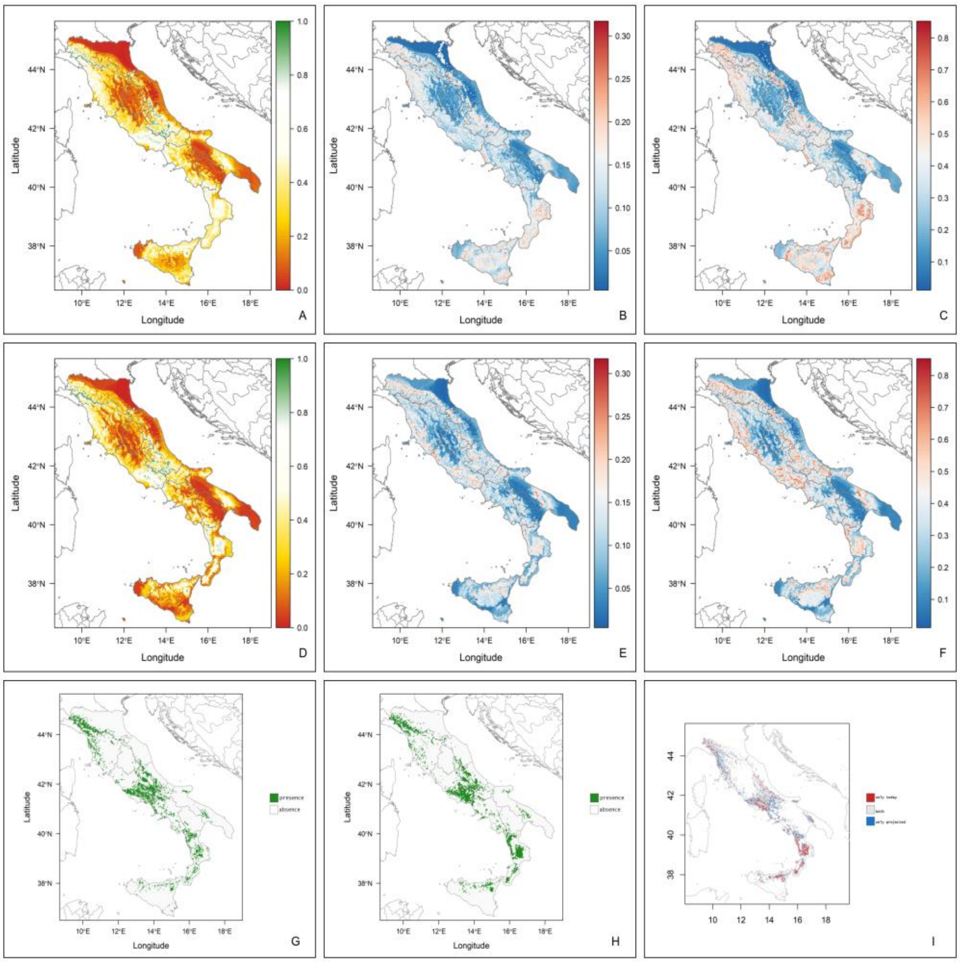

Fig. S.3.191-A) 'Projected' base output; B) 'Projected' uncertainty standard deviation; C) 'Projected' uncertainty range of values; D) 'Present' base output; E) 'Present' uncertainty standard deviation; F) 'Present' uncertainty range of values; G) 'Projected' binary distribution; H) 'Present' binary distribution; I) Range size variation.

Value	Present	Projected
AUC.train	0.940	1.000
AUC.test	0.940	0.962
AUC.diff	0.038	0.038
pROC	1.261	1.262
OR.p10	0.609	0.654
CBI	0.990	0.996
perc.stable	41.078	41.078
range.size	-0.962	-0.962

S.3.192 *Linaria purpurea* SVM

Species: *Linaria purpurea* (L.) Mill.

Procedure: SVM

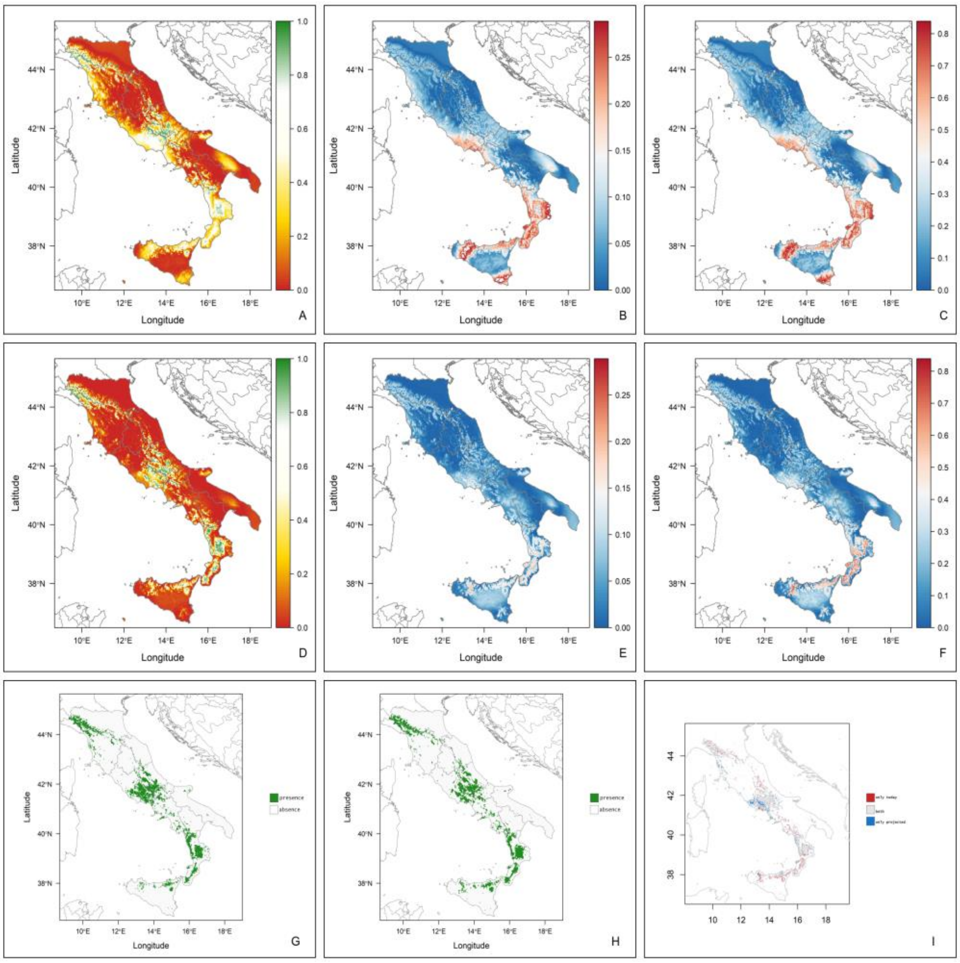

Fig. S.3.192-A) 'Projected' base output; B) 'Projected' uncertainty standard deviation; C) 'Projected' uncertainty range of values; D) 'Present' base output; E) 'Present' uncertainty standard deviation; F) 'Present' uncertainty range of values; G) 'Projected' binary distribution; H) 'Present' binary distribution; I) Range size variation.

Value	Present	Projected
AUC.train	0.842	0.851
AUC.test	0.842	0.812
AUC.diff	0.040	0.040
pROC	1.245	1.219
OR.p10	0.130	0.144
CBI	0.946	0.980
perc.stable	68.963	68.963
range.size	-0.564	-0.564

S.3.193 *Ornithogalum etruscum* Biomod2

Species: *Ornithogalum etruscum* Parl.

Procedure: Biomod2

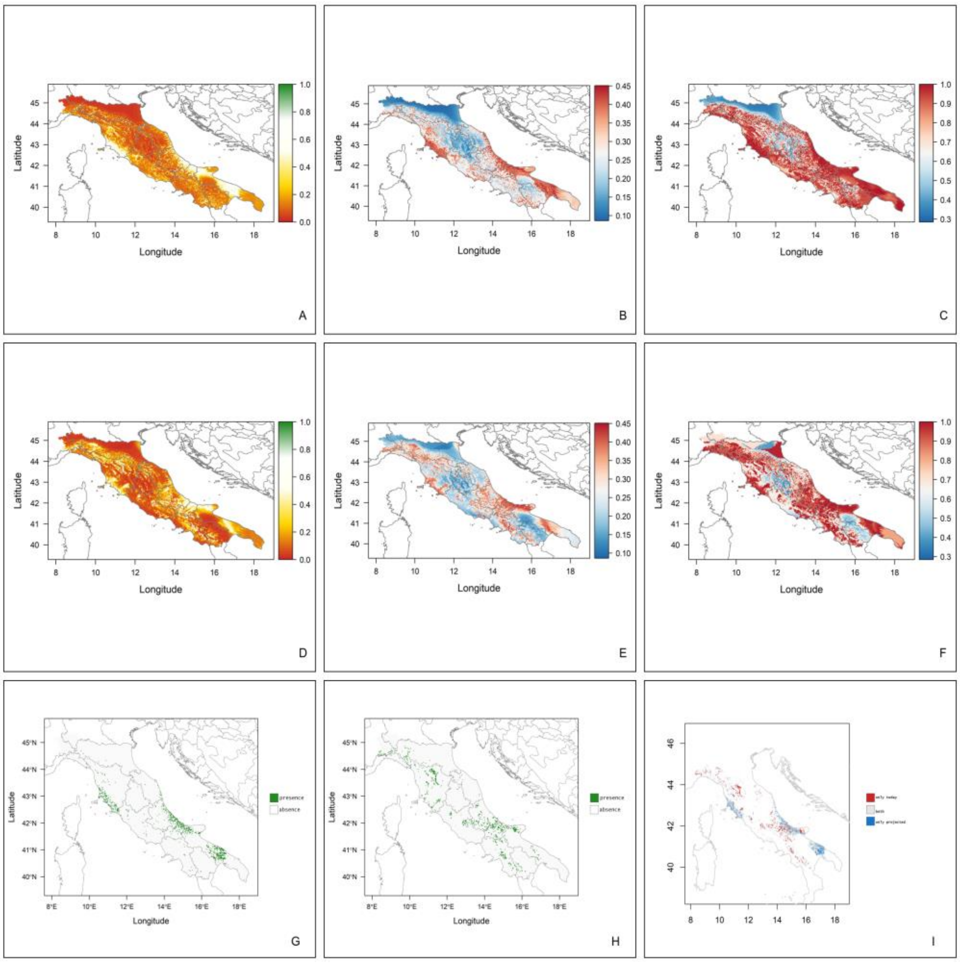

Fig. S.3.193-A) 'Projected' base output; B) 'Projected' uncertainty standard deviation; C) 'Projected' uncertainty range of values; D) 'Present' base output; E) 'Present' uncertainty standard deviation; F) 'Present' uncertainty range of values; G) 'Projected' binary distribution; H) 'Present' binary distribution; I) Range size variation.

Value	Present	Projected
AUC.train	0.749	1.000
AUC.test	0.749	0.640
AUC.diff	0.359	0.359
pROC	1.403	1.283
OR.p10	1.000	1.000
CBI	0.958	0.726
perc.stable	4.461	4.461
range.size	3.810	3.810

S.3.194 *Ornithogalum etruscum* Bioclim

Species: *Ornithogalum etruscum* Parl.

Procedure: Bioclim

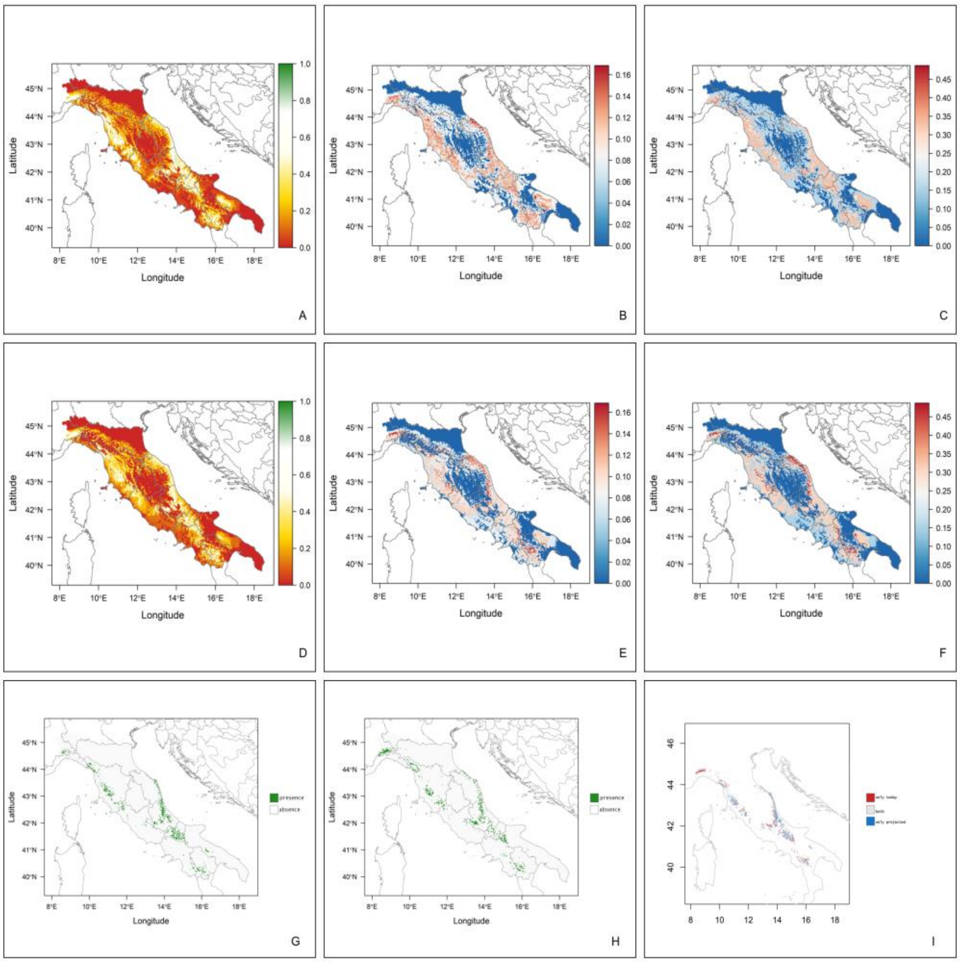

Fig. S.3.194-A) 'Projected' base output; B) 'Projected' uncertainty standard deviation; C) 'Projected' uncertainty range of values; D) 'Present' base output; E) 'Present' uncertainty standard deviation; F) 'Present' uncertainty range of values; G) 'Projected' binary distribution; H) 'Present' binary distribution; I) Range size variation.

Value	Present	Projected
AUC.train	0.615	0.707
AUC.test	0.615	0.582
AUC.diff	0.131	0.131
pROC	1.169	1.120
OR.p10	0.300	0.370
CBI	0.401	0.674
perc.stable	22.456	22.456
range.size	0.040	0.040

S.3.195 *Ornithogalum etruscum* Domain

Species: *Ornithogalum etruscum* Parl.

Procedure: Domain

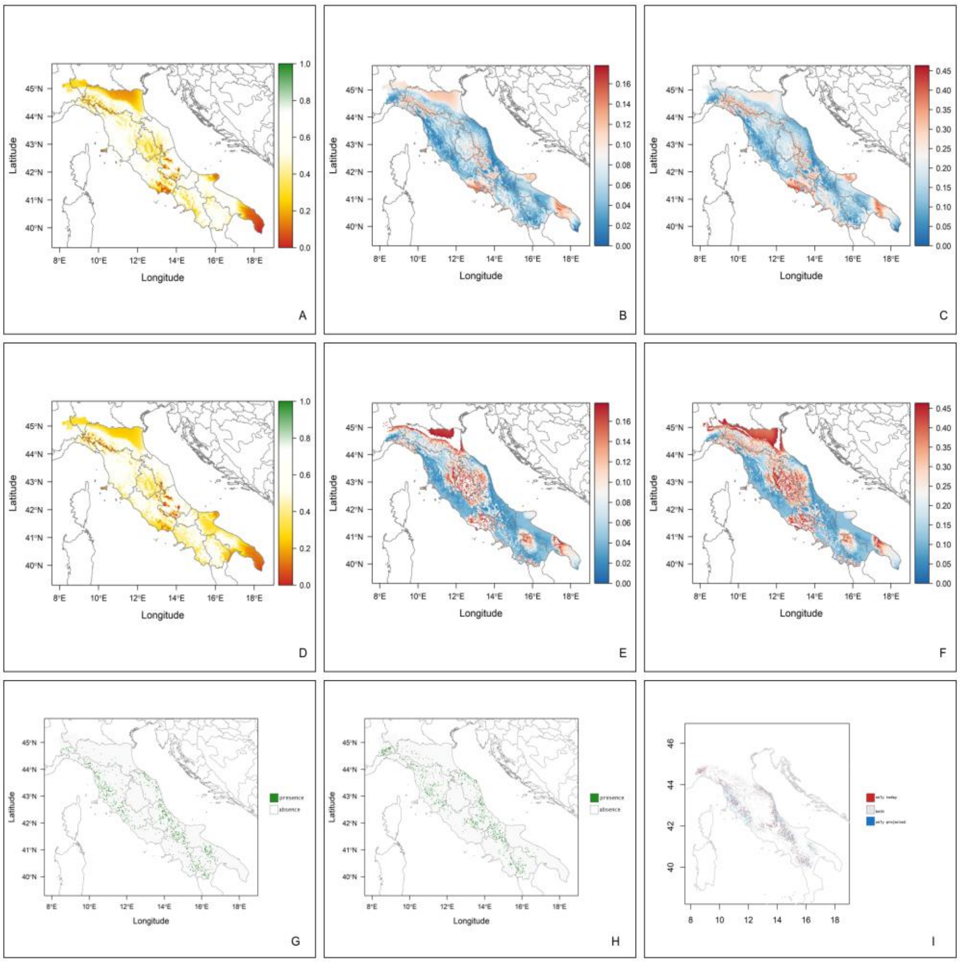

Fig. S.3.195-A) 'Projected' base output; B) 'Projected' uncertainty standard deviation; C) 'Projected' uncertainty range of values; D) 'Present' base output; E) 'Present' uncertainty standard deviation; F) 'Present' uncertainty range of values; G) 'Projected' binary distribution; H) 'Present' binary distribution; I) Range size variation.

Value	Present	Projected
AUC.train	0.648	0.671
AUC.test	0.648	0.547
AUC.diff	0.145	0.145
pROC	1.323	1.211
OR.p10	0.250	0.350
CBI	0.092	0.303
perc.stable	5.806	5.806
range.size	5.655	5.655

S.3.196 *Ornithogalum etruscum* Maxent

Species: *Ornithogalum etruscum* Parl.

Procedure: Maxent

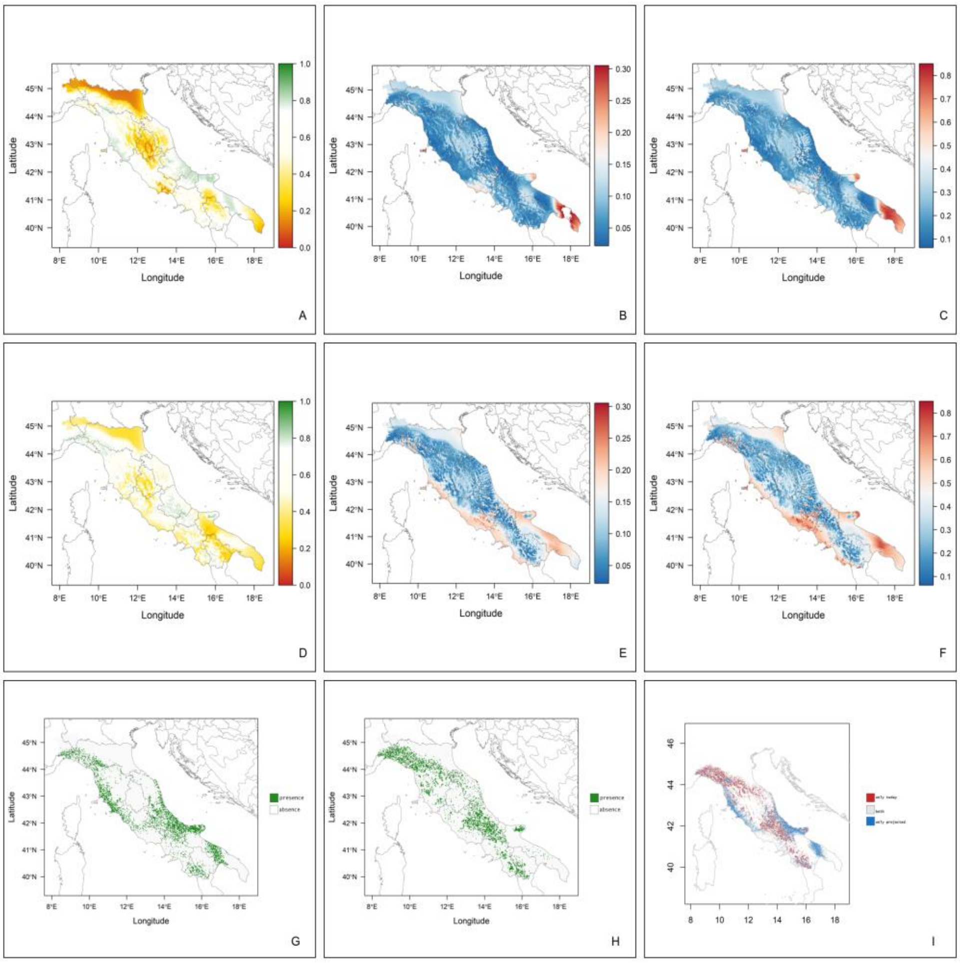

Fig. S.3.196-A) 'Projected' base output; B) 'Projected' uncertainty standard deviation; C) 'Projected' uncertainty range of values; D) 'Present' base output; E) 'Present' uncertainty standard deviation; F) 'Present' uncertainty range of values; G) 'Projected' binary distribution; H) 'Present' binary distribution; I) Range size variation.

Value	Present	Projected
AUC.train	0.626	0.739
AUC.test	0.626	0.657
AUC.diff	0.109	0.109
pROC	1.285	1.344
OR.p10	0.367	0.363
CBI	0.875	0.921
perc.stable	16.134	16.134
range.size	-0.328	-0.328

S.3.197 *Ornithogalum etruscum* KDE

Species: *Ornithogalum etruscum* Parl.

Procedure: KDE

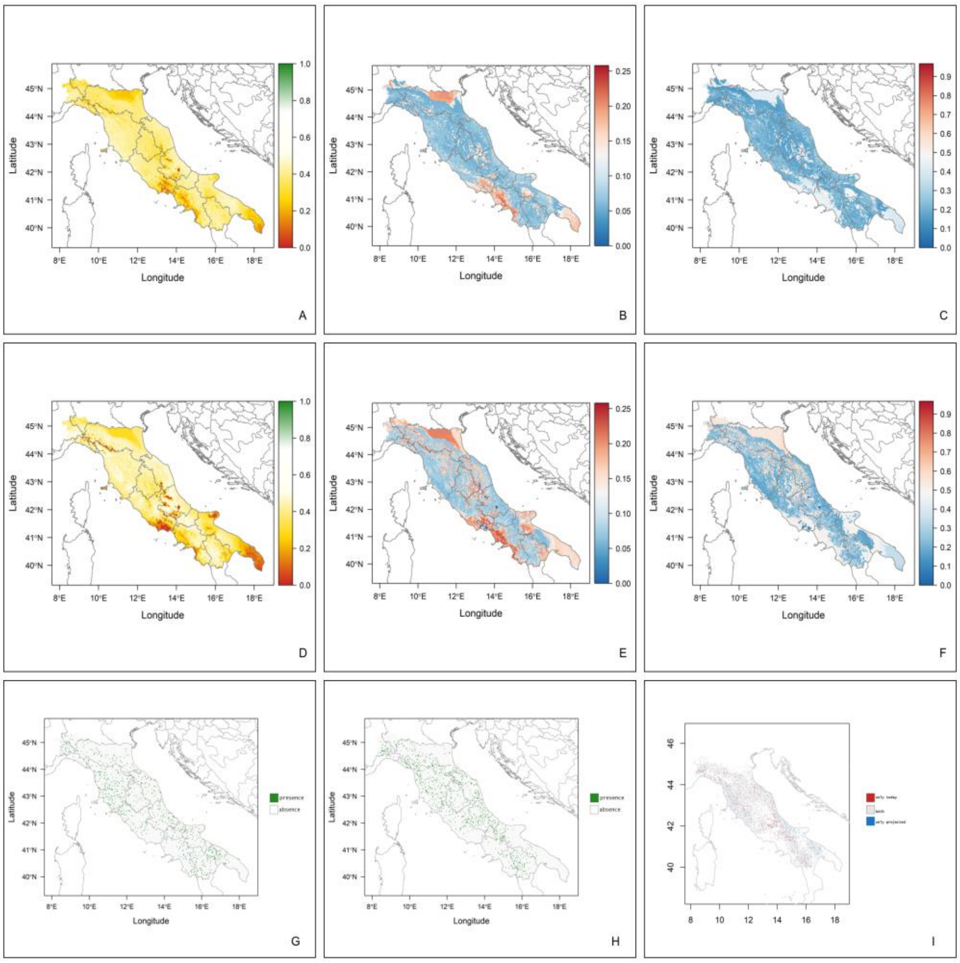

Fig. S.3.197-A) 'Projected' base output; B) 'Projected' uncertainty standard deviation; C) 'Projected' uncertainty range of values; D) 'Present' base output; E) 'Present' uncertainty standard deviation; F) 'Present' uncertainty range of values; G) 'Projected' binary distribution; H) 'Present' binary distribution; I) Range size variation.

Value	Present	Projected
AUC.train	0.570	0.643
AUC.test	0.570	0.567
AUC.diff	0.155	0.105
pROC	1.241	1.240
OR.p10	0.417	0.267
CBI	0.021	-0.026
perc.stable	2.875	2.875
range.size	0.338	0.338

S.3.198 *Ornithogalum etruscum* CTA

Species: *Ornithogalum etruscum* Parl.

Procedure: CTA

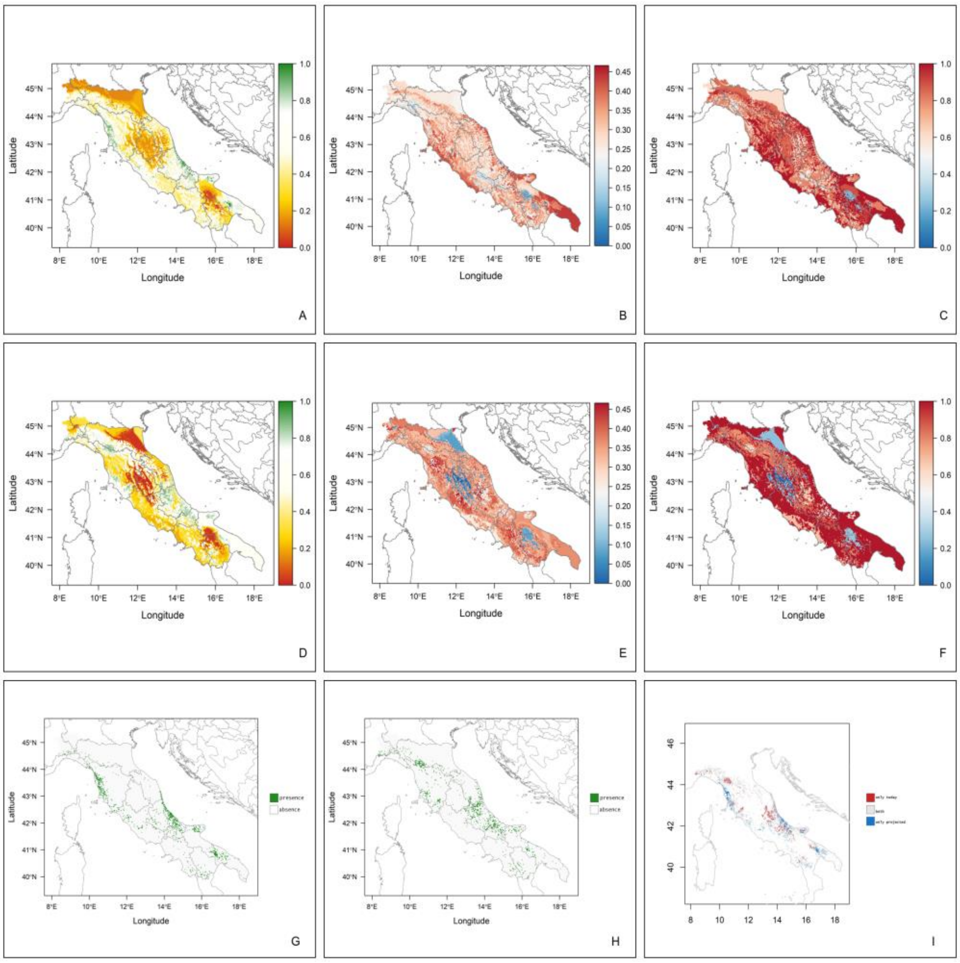

Fig. S.3.198-A) 'Projected' base output; B) 'Projected' uncertainty standard deviation; C) 'Projected' uncertainty range of values; D) 'Present' base output; E) 'Present' uncertainty standard deviation; F) 'Present' uncertainty range of values; G) 'Projected' binary distribution; H) 'Present' binary distribution; I) Range size variation.

Value	Present	Projected
AUC.train	0.718	0.885
AUC.test	0.718	0.715
AUC.diff	0.171	0.171
pROC	1.156	1.112
OR.p10	0.367	0.490
CBI	0.940	0.528
perc.stable	7.436	7.436
range.size	1.072	1.072

S.3.199 *Ornithogalum etruscum* FDA

Species: *Ornithogalum etruscum* Parl.

Procedure: FDA

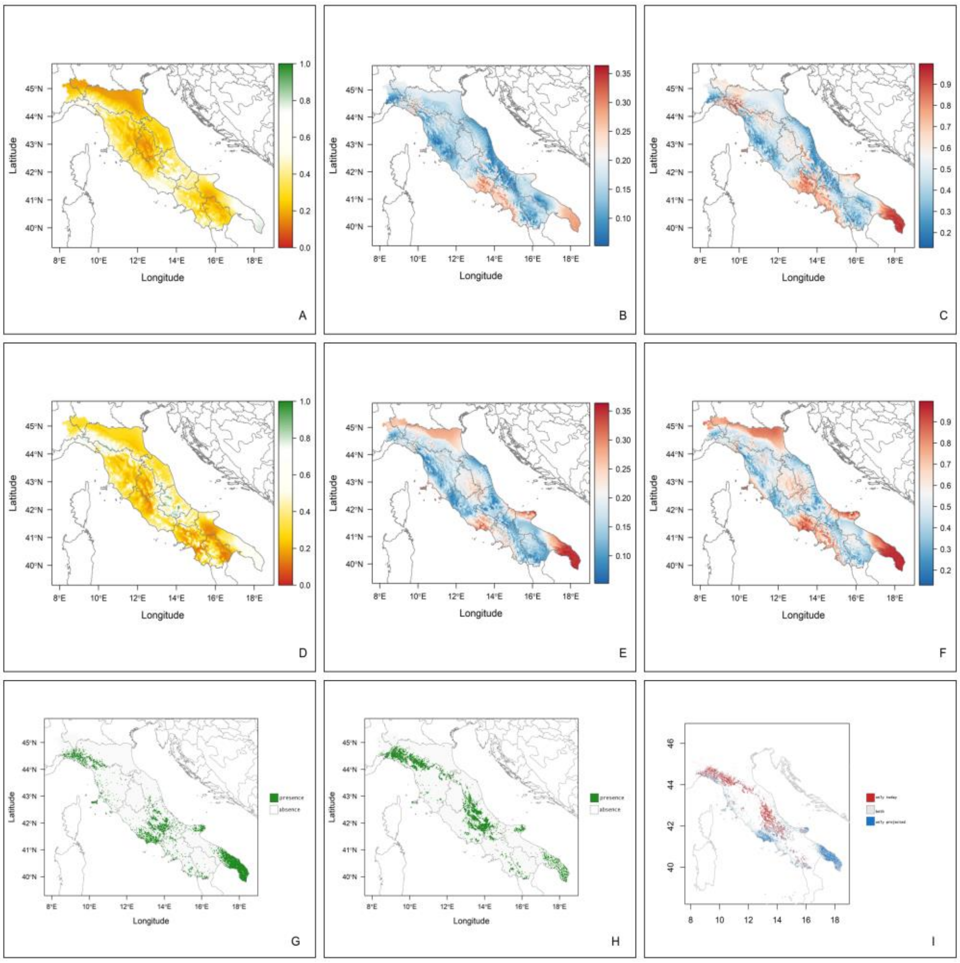

Fig. S.3.199-A) 'Projected' base output; B) 'Projected' uncertainty standard deviation; C) 'Projected' uncertainty range of values; D) 'Present' base output; E) 'Present' uncertainty standard deviation; F) 'Present' uncertainty range of values; G) 'Projected' binary distribution; H) 'Present' binary distribution; I) Range size variation.

Value	Present	Projected
AUC.train	0.596	0.674
AUC.test	0.596	0.585
AUC.diff	0.103	0.103
pROC	1.210	1.260
OR.p10	0.400	0.423
CBI	0.628	0.174
perc.stable	31.547	31.547
range.size	1.041	1.041

S.3.200 *Ornithogalum etruscum* GAM

Species: *Ornithogalum etruscum* Parl.

Procedure: GAM

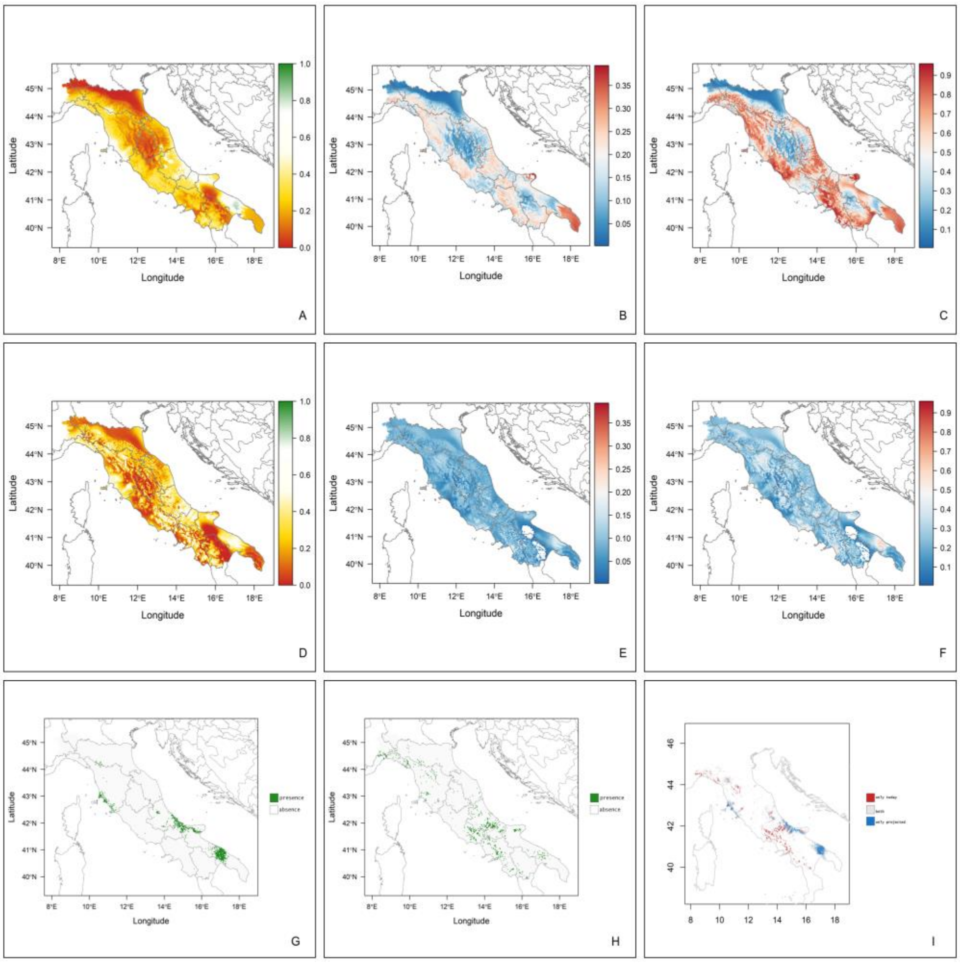

Fig. S.3.200-A) 'Projected' base output; B) 'Projected' uncertainty standard deviation; C) 'Projected' uncertainty range of values; D) 'Present' base output; E) 'Present' uncertainty standard deviation; F) 'Present' uncertainty range of values; G) 'Projected' binary distribution; H) 'Present' binary distribution; I) Range size variation.

Value	Present	Projected
AUC.train	0.773	0.777
AUC.test	0.773	0.659
AUC.diff	0.118	0.118
pROC	1.490	1.330
OR.p10	0.367	0.393
CBI	0.858	0.558
perc.stable	7.583	7.583
range.size	0.083	0.083

S.3.201 *Ornithogalum etruscum* GLM

Species: *Ornithogalum etruscum* Parl.

Procedure: GLM

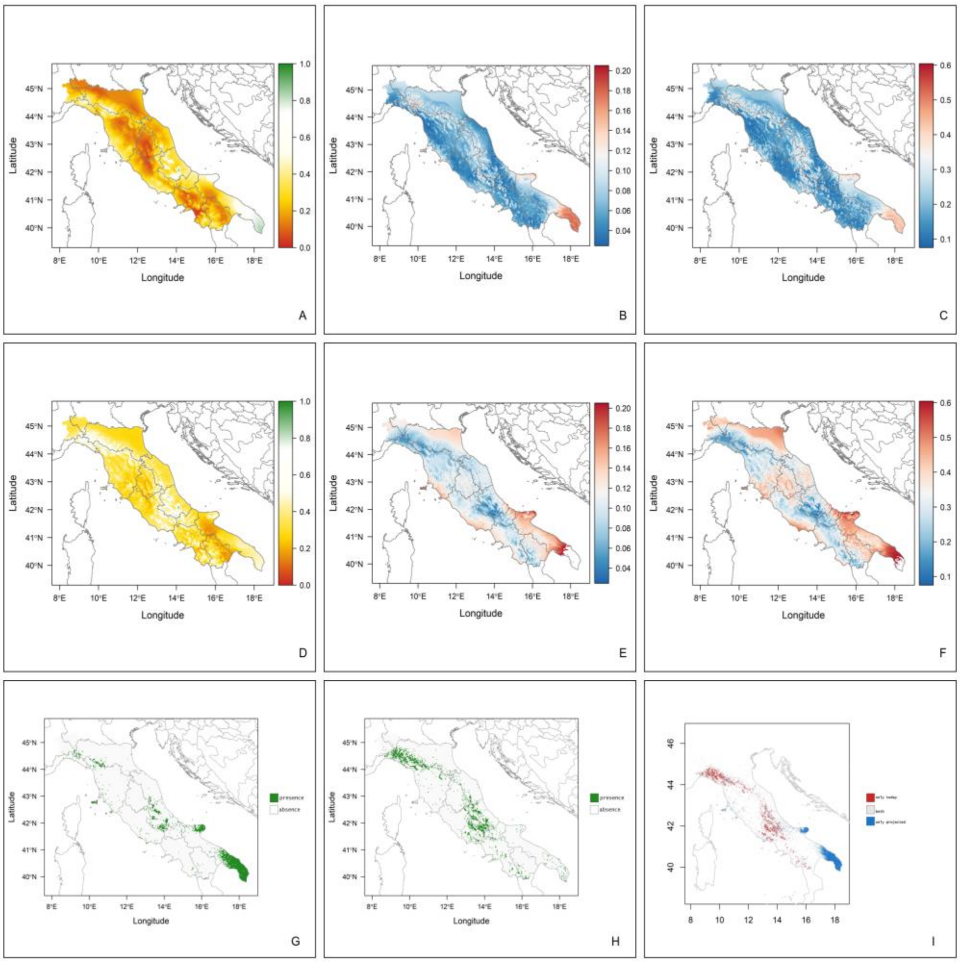

Fig. S.3.201-A) 'Projected' base output; B) 'Projected' uncertainty standard deviation; C) 'Projected' uncertainty range of values; D) 'Present' base output; E) 'Present' uncertainty standard deviation; F) 'Present' uncertainty range of values; G) 'Projected' binary distribution; H) 'Present' binary distribution; I) Range size variation.

Value	Present	Projected
AUC.train	0.582	0.686
AUC.test	0.582	0.631
AUC.diff	0.089	0.089
pROC	1.275	1.306
OR.p10	0.350	0.227
CBI	0.665	0.635
perc.stable	13.641	13.641
range.size	1.630	1.630

S.3.202 *Ornithogalum etruscum* MARS

Species: *Ornithogalum etruscum* Parl.

Procedure: MARS

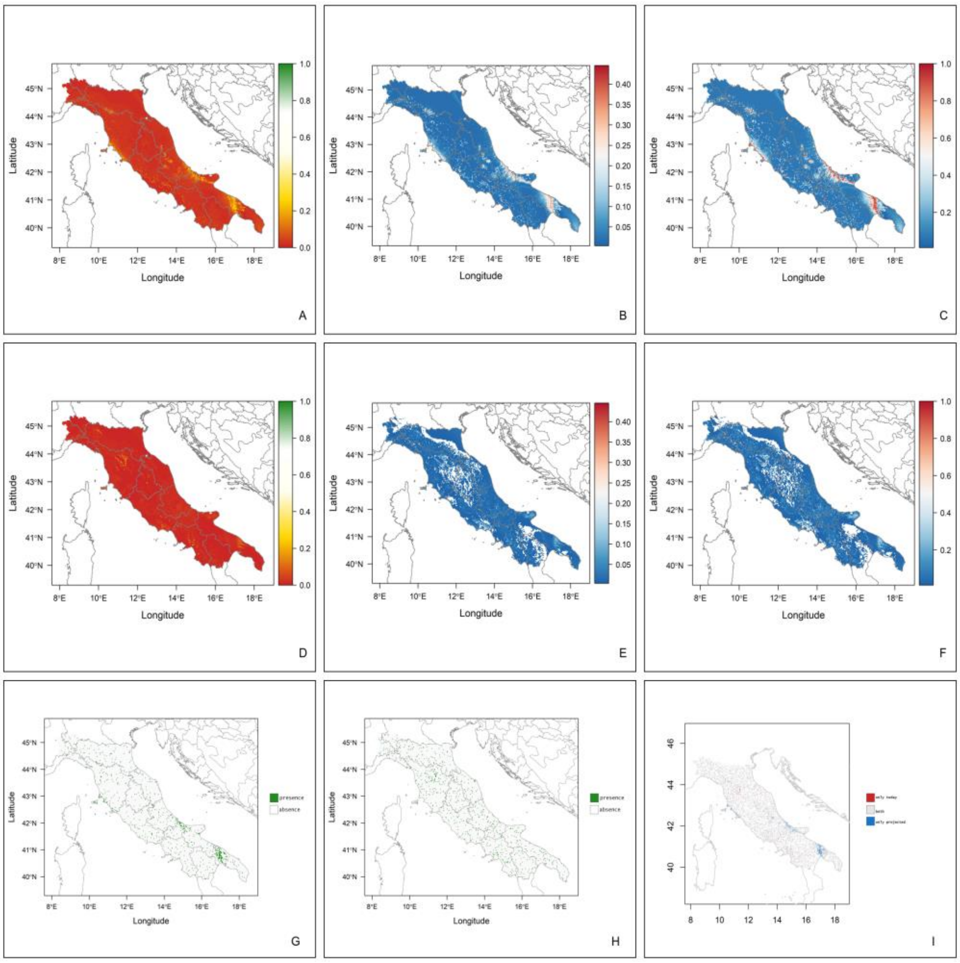

Fig. S.3.202-A) 'Projected' base output; B) 'Projected' uncertainty standard deviation; C) 'Projected' uncertainty range of values; D) 'Present' base output; E) 'Present' uncertainty standard deviation; F) 'Present' uncertainty range of values; G) 'Projected' binary distribution; H) 'Present' binary distribution; I) Range size variation.

Value	Present	Projected
AUC.train	0.534	0.679
AUC.test	0.534	0.529
AUC.diff	0.151	0.151
pROC	1.111	1.100
OR.p10	0.400	0.253
CBI	0.774	0.537
perc.stable	2.037	2.037
range.size	-3.558	-3.558

S.3.203 *Ornithogalum etruscum* RF

Species: *Ornithogalum etruscum* Parl.

Procedure: RF

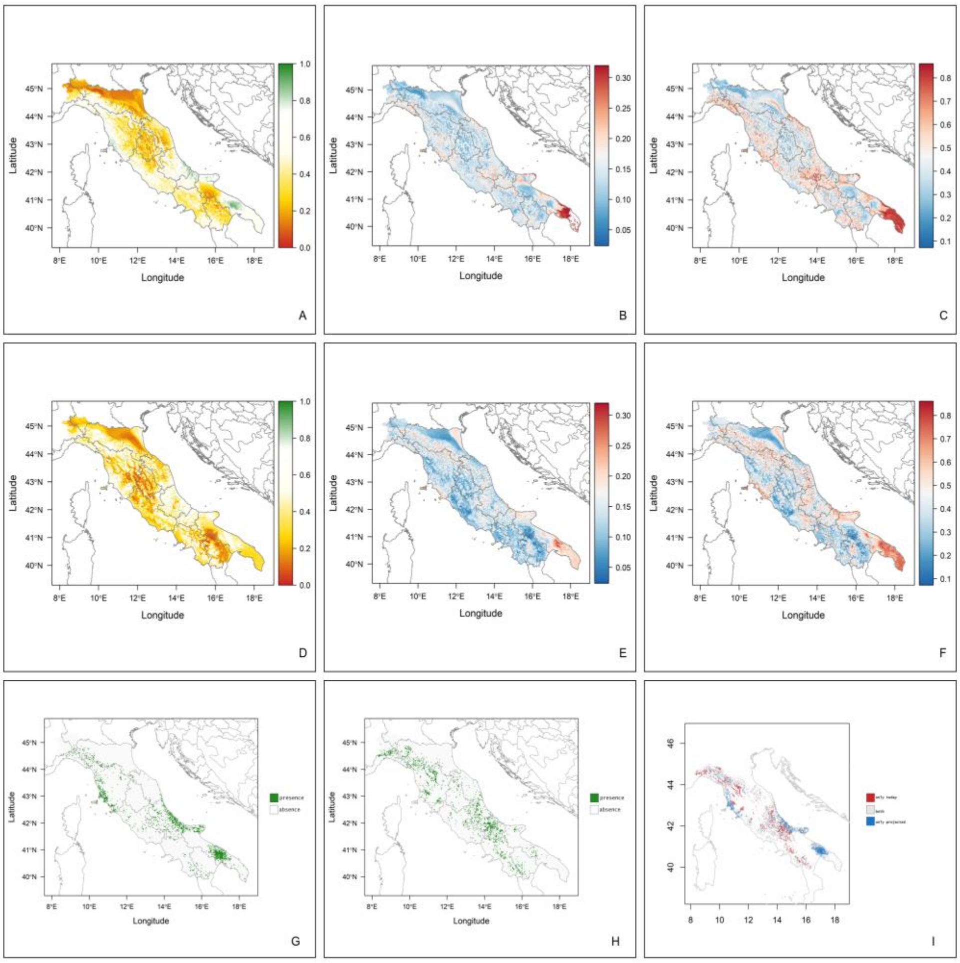

Fig. S.3.203-A) 'Projected' base output; B) 'Projected' uncertainty standard deviation; C) 'Projected' uncertainty range of values; D) 'Present' base output; E) 'Present' uncertainty standard deviation; F) 'Present' uncertainty range of values; G) 'Projected' binary distribution; H) 'Present' binary distribution; I) Range size variation.

Value	Present	Projected
AUC.train	0.960	1.000
AUC.test	0.960	0.963
AUC.diff	0.037	0.037
pROC	1.353	1.337
OR.p10	0.900	0.783
CBI	0.954	0.810
perc.stable	6.500	6.500
range.size	0.942	0.942

S.3.204 *Ornithogalum etruscum* SVM

Species: *Ornithogalum etruscum* Parl.

Procedure: SVM

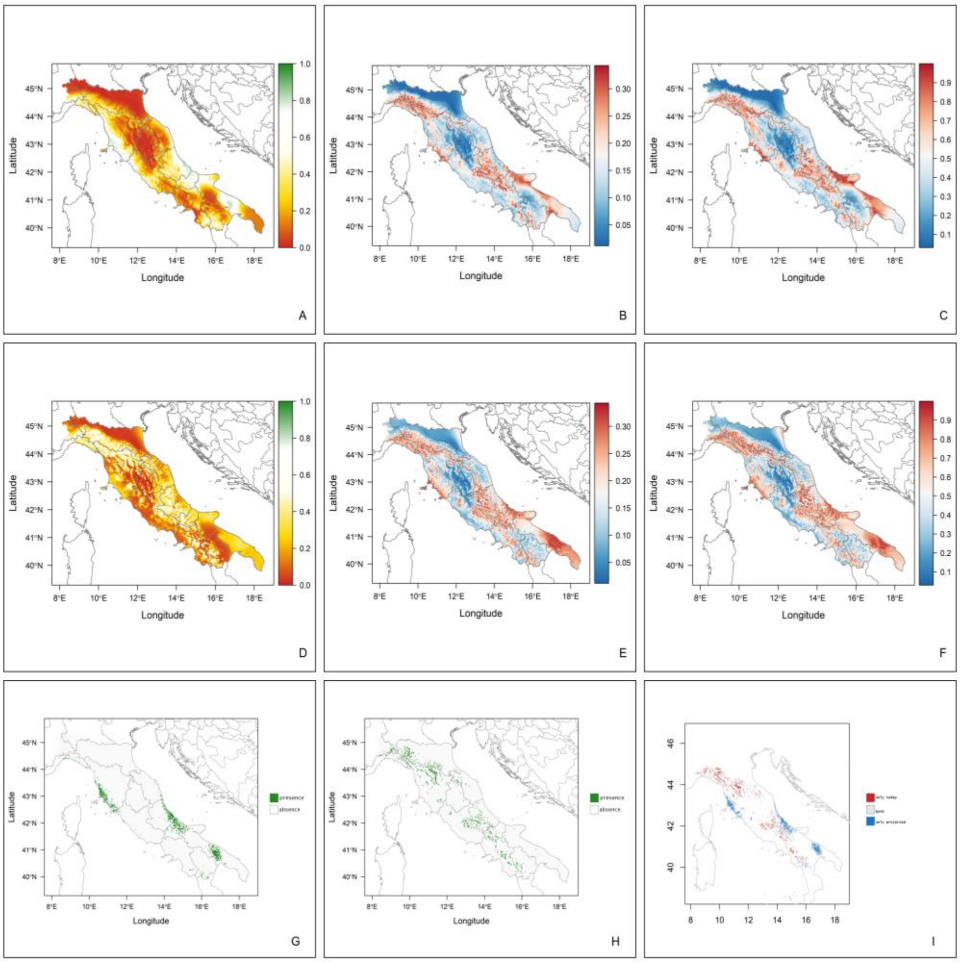

Fig. S.3.204-A) 'Projected' base output; B) 'Projected' uncertainty standard deviation; C) 'Projected' uncertainty range of values; D) 'Present' base output; E) 'Present' uncertainty standard deviation; F) 'Present' uncertainty range of values; G) 'Projected' binary distribution; H) 'Present' binary distribution; I) Range size variation.

Value	Present	Projected
AUC.train	0.774	0.876
AUC.test	0.774	0.708
AUC.diff	0.167	0.167
pROC	1.290	1.212
OR.p10	0.400	0.467
CBI	0.855	0.593
perc.stable	0.186	0.186
range.size	-1.306	-1.306

S.3.205 *Phyteuma ovatum* Biomod2

Species: *Phyteuma ovatum* Honck. subsp. *pseudospicatum* Pignatti

Procedure: Biomod2

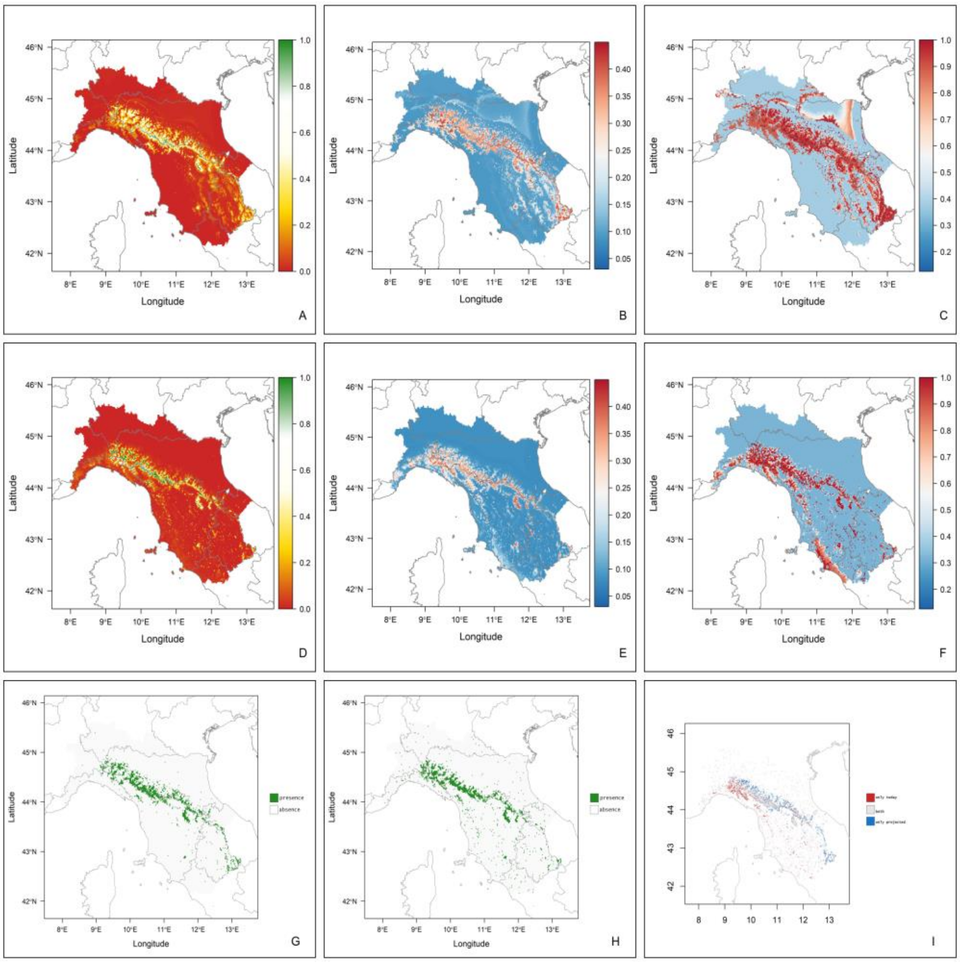

Fig. S.3.205-A) 'Projected' base output; B) 'Projected' uncertainty standard deviation; C) 'Projected' uncertainty range of values; D) 'Present' base output; E) 'Present' uncertainty standard deviation; F) 'Present' uncertainty range of values; G) 'Projected' binary distribution; H) 'Present' binary distribution; I) Range size variation.

Value	Present	Projected
AUC.train	0.984	0.994
AUC.test	0.984	0.750
AUC.diff	0.244	0.244
pROC	1.892	1.279
OR.p10	0.289	0.744
CBI	0.689	0.937
perc.stable	43.304	43.304
range.size	-1.591	-1.591

S.3.206 *Phyteuma ovatum* Bioclim

Species: *Phyteuma ovatum* Honck. subsp. *pseudospicatum* Pignatti

Procedure: Bioclim

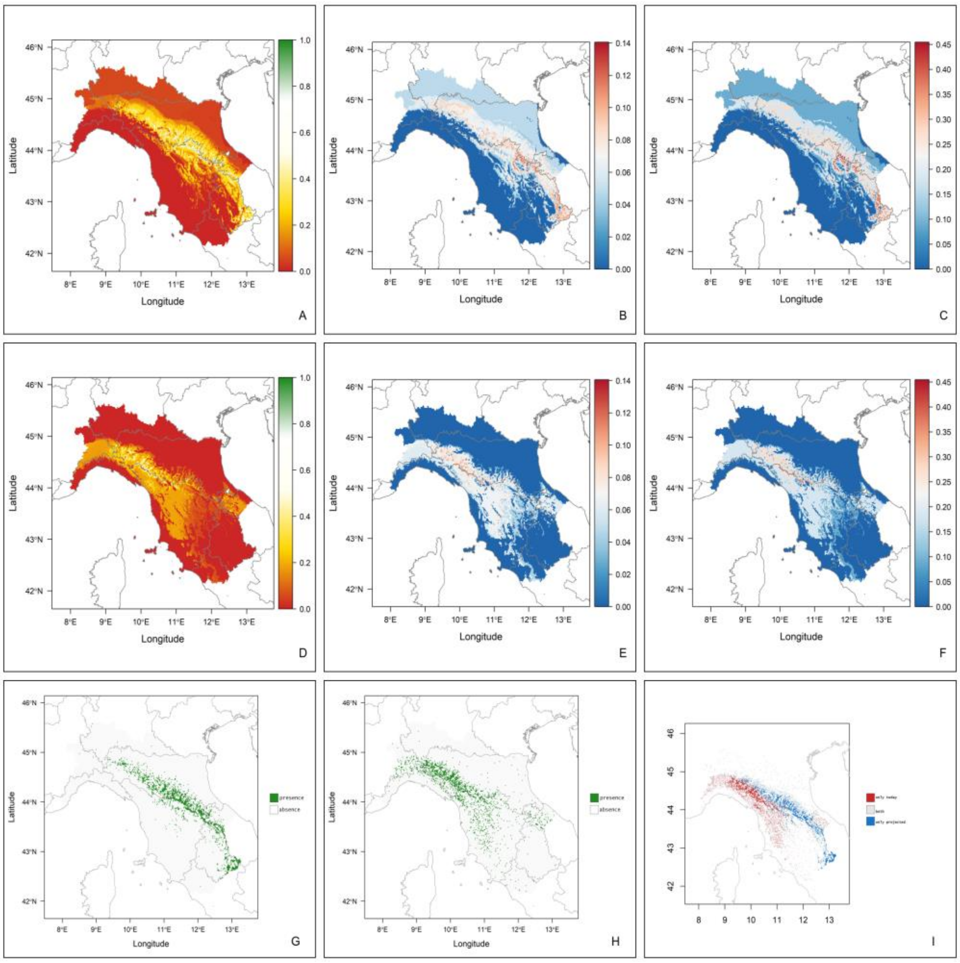

Fig. S.3.206-A) 'Projected' base output; B) 'Projected' uncertainty standard deviation; C) 'Projected' uncertainty range of values; D) 'Present' base output; E) 'Present' uncertainty standard deviation; F) 'Present' uncertainty range of values; G) 'Projected' binary distribution; H) 'Present' binary distribution; I) Range size variation.

Value	Present	Projected
AUC.train	0.787	0.849
AUC.test	0.787	0.772
AUC.diff	0.090	0.090
pROC	1.245	1.277
OR.p10	0.189	0.233
CBI	0.854	0.453
perc.stable	4.198	4.198
range.size	4.036	4.036

S.3.207 *Phyteuma ovatum* Domain

Species: *Phyteuma ovatum* Honck. subsp. *pseudospicatum* Pignatti

Procedure: Domain

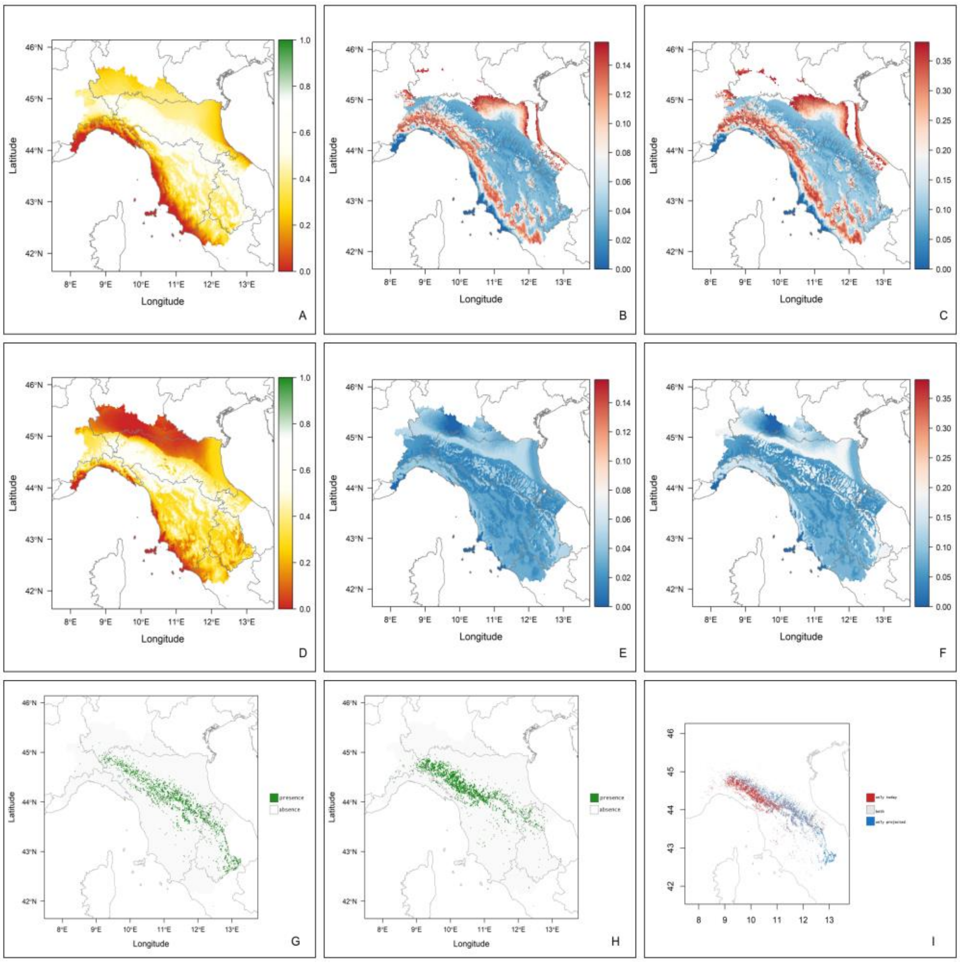

Fig. S.3.207-A) 'Projected' base output; B) 'Projected' uncertainty standard deviation; C) 'Projected' uncertainty range of values; D) 'Present' base output; E) 'Present' uncertainty standard deviation; F) 'Present' uncertainty range of values; G) 'Projected' binary distribution; H) 'Present' binary distribution; I) Range size variation.

Value	Present	Projected
AUC.train	0.856	0.852
AUC.test	0.856	0.830
AUC.diff	0.044	0.044
pROC	1.524	1.444
OR.p10	0.200	0.200
CBI	0.799	0.251
perc.stable	15.484	15.484
range.size	-6.898	-6.898

S.3.208 *Phyteuma ovatum* Maxent

Species: *Phyteuma ovatum* Honck. subsp. *pseudospicatum* Pignatti

Procedure: Maxent

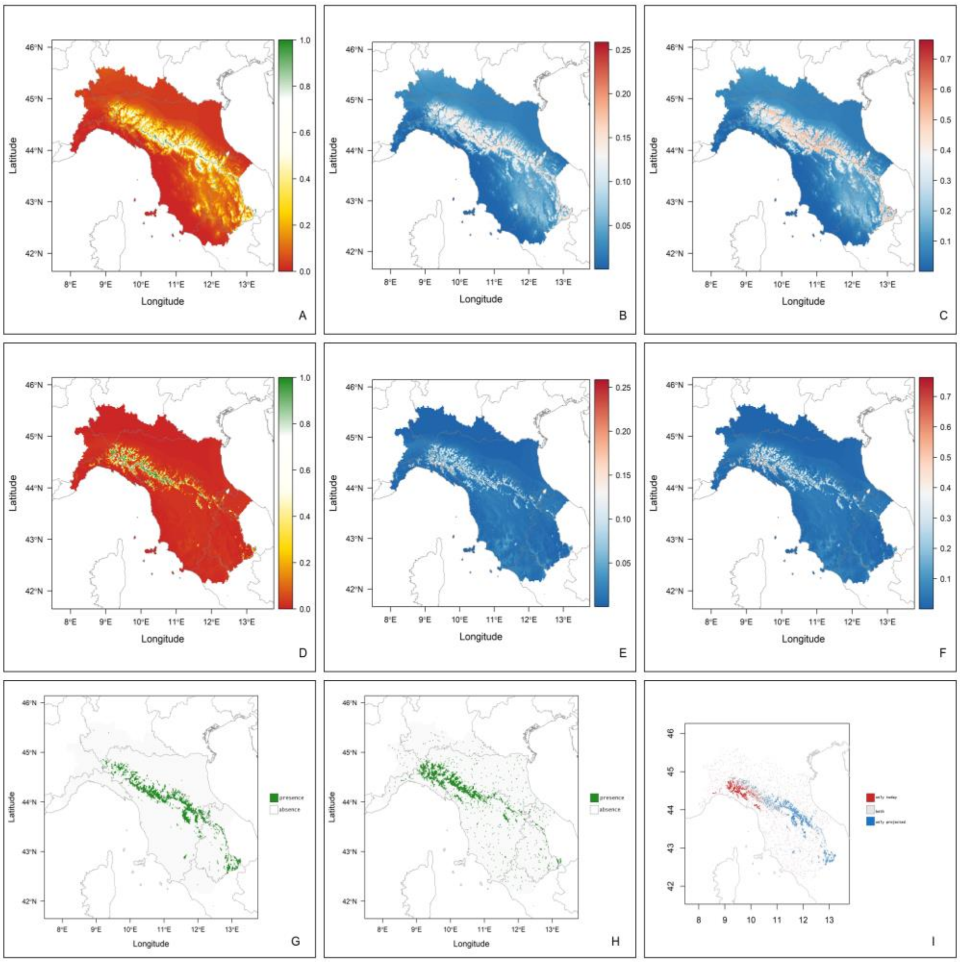

Fig. S.3.208-A) 'Projected' base output; B) 'Projected' uncertainty standard deviation; C) 'Projected' uncertainty range of values; D) 'Present' base output; E) 'Present' uncertainty standard deviation; F) 'Present' uncertainty range of values; G) 'Projected' binary distribution; H) 'Present' binary distribution; I) Range size variation.

Value	Present	Projected
AUC.train	0.919	0.909
AUC.test	0.919	0.900
AUC.diff	0.034	0.034
pROC	1.678	1.608
OR.p10	0.211	0.189
CBI	0.940	0.967
perc.stable	29.302	29.302
range.size	1.154	1.154

S.3.209 *Phyteuma ovatum* KDE

Species: *Phyteuma ovatum* Honck. subsp. *pseudospicatum* Pignatti

Procedure: KDE

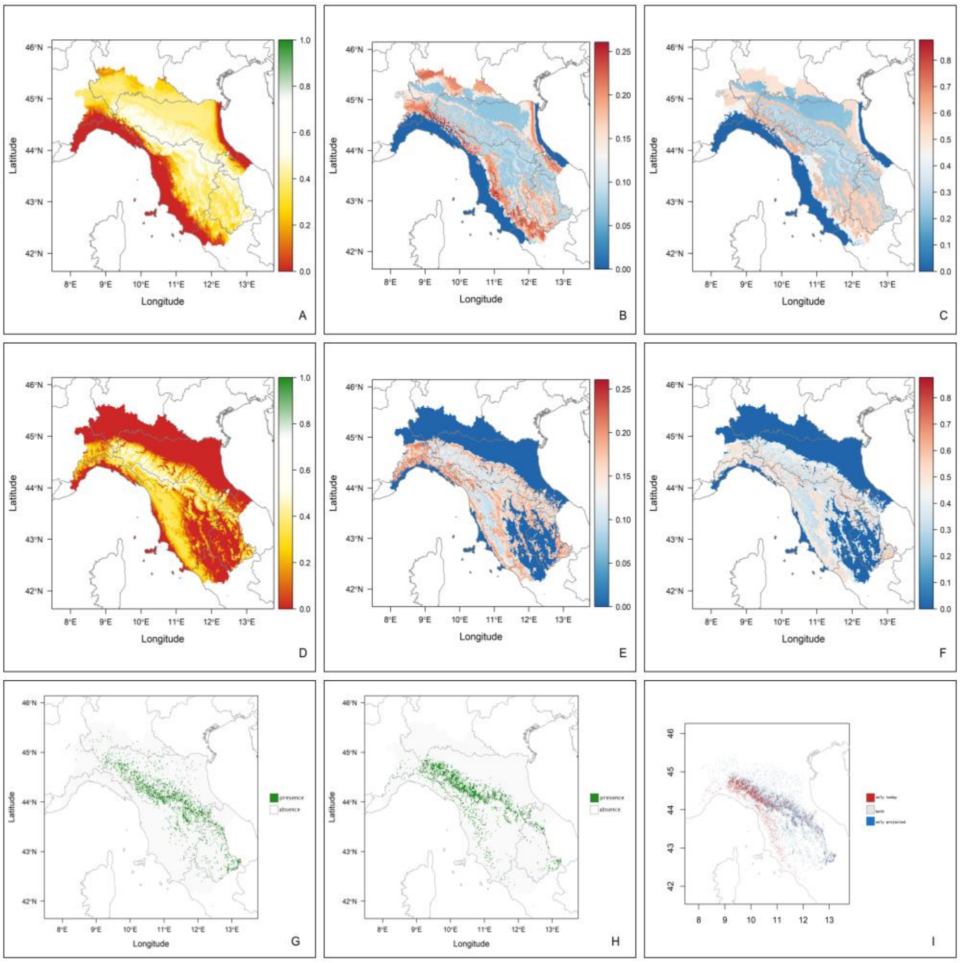

Fig. S.3.209-A) 'Projected' base output; B) 'Projected' uncertainty standard deviation; C) 'Projected' uncertainty range of values; D) 'Present' base output; E) 'Present' uncertainty standard deviation; F) 'Present' uncertainty range of values; G) 'Projected' binary distribution; H) 'Present' binary distribution; I) Range size variation.

Value	Present	Projected
AUC.train	0.938	0.869
AUC.test	0.938	0.867
AUC.diff	0.021	0.023
pROC	1.713	1.539
OR.p10	0.178	0.089
CBI	0.994	0.528
perc.stable	17.027	17.027
range.size	-0.238	-0.238

S.3.210 *Phyteuma ovatum* CTA

Species: *Phyteuma ovatum* Honck. subsp. *pseudospicatum* Pignatti

Procedure: CTA

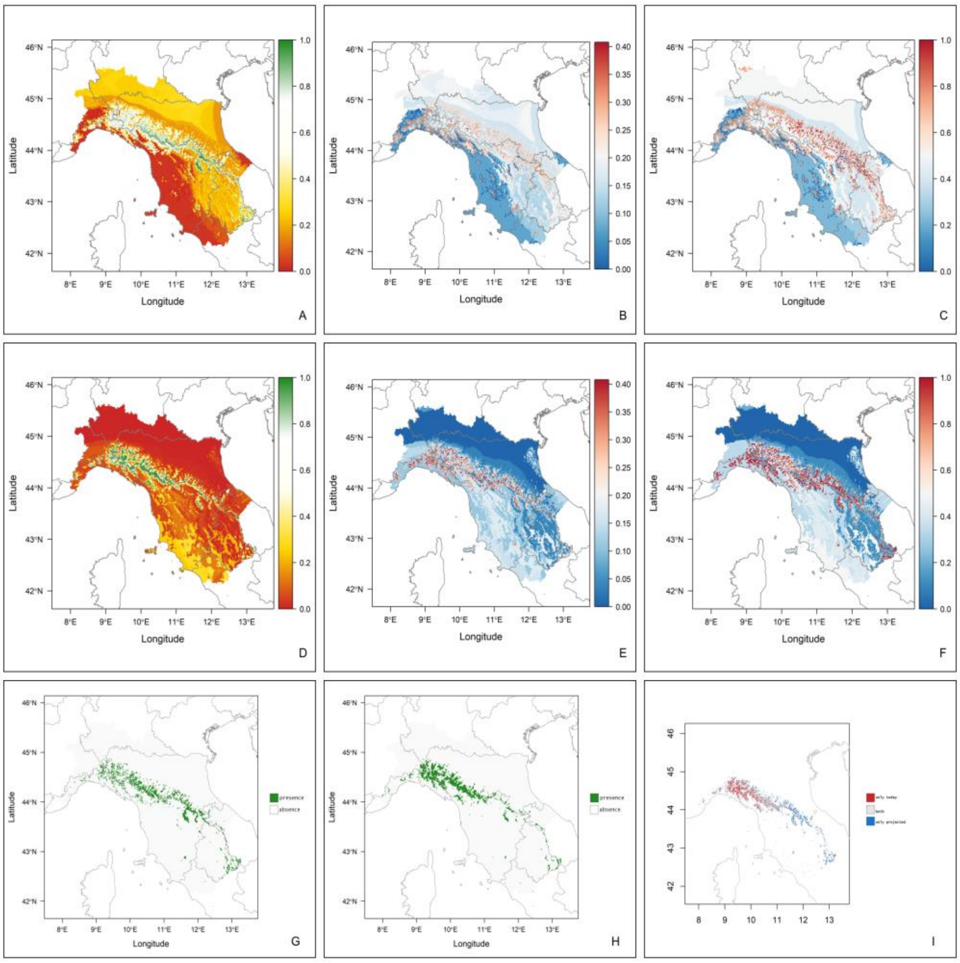

Fig. S.3.210-A) 'Projected' base output; B) 'Projected' uncertainty standard deviation; C) 'Projected' uncertainty range of values; D) 'Present' base output; E) 'Present' uncertainty standard deviation; F) 'Present' uncertainty range of values; G) 'Projected' binary distribution; H) 'Present' binary distribution; I) Range size variation.

Value	Present	Projected
AUC.train	0.960	0.958
AUC.test	0.960	0.894
AUC.diff	0.068	0.068
pROC	1.384	1.317
OR.p10	0.144	0.200
CBI	0.832	0.594
perc.stable	39.102	39.102
range.size	-0.226	-0.226

S.3.211 *Phyteuma ovatum* FDA

Species: *Phyteuma ovatum* Honck. subsp. *pseudospicatum* Pignatti

Procedure: FDA

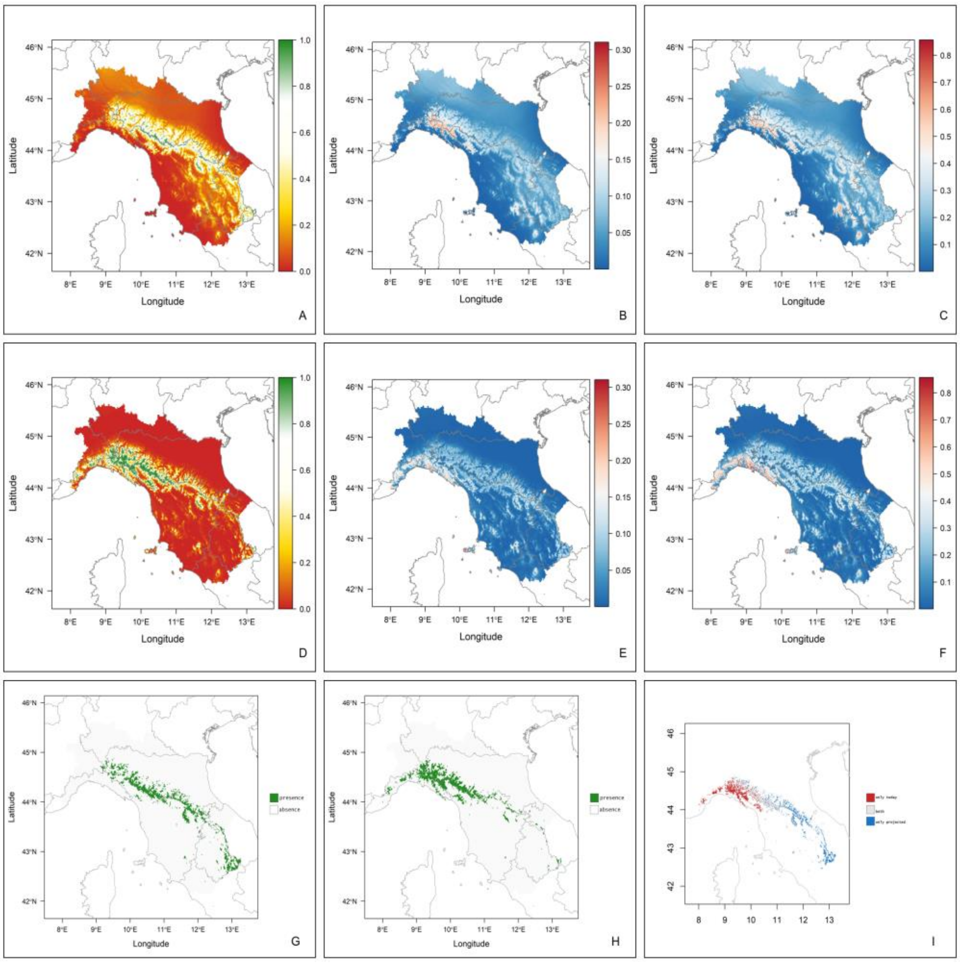

Fig. S.3.211-A) 'Projected' base output; B) 'Projected' uncertainty standard deviation; C) 'Projected' uncertainty range of values; D) 'Present' base output; E) 'Present' uncertainty standard deviation; F) 'Present' uncertainty range of values; G) 'Projected' binary distribution; H) 'Present' binary distribution; I) Range size variation.

Value	Present	Projected
AUC.train	0.884	0.910
AUC.test	0.884	0.897
AUC.diff	0.031	0.031
pROC	1.521	1.593
OR.p10	0.278	0.222
CBI	0.520	0.897
perc.stable	33.850	33.850
range.size	0.674	0.674

S.3.212 *Phyteuma ovatum* GAM

Species: *Phyteuma ovatum* Honck. subsp. *pseudospicatum* Pignatti

Procedure: GAM

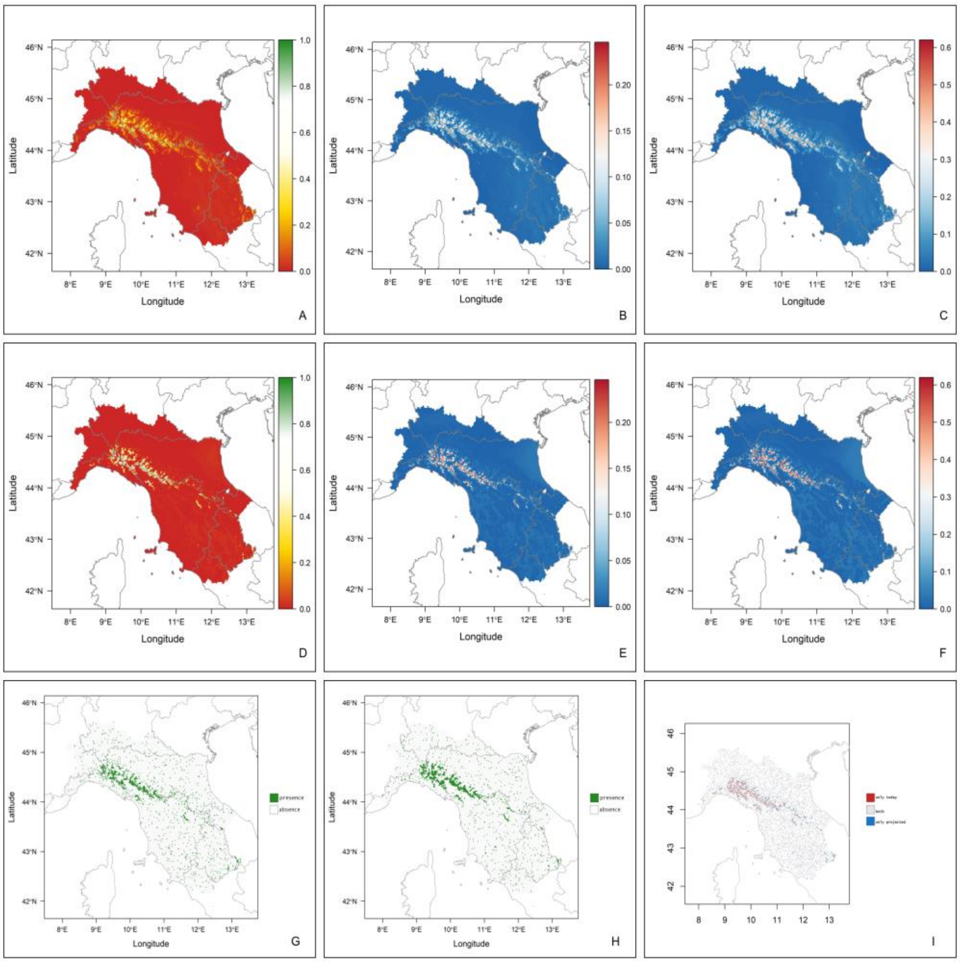

Fig. S.3.212-A) 'Projected' base output; B) 'Projected' uncertainty standard deviation; C) 'Projected' uncertainty range of values; D) 'Present' base output; E) 'Present' uncertainty standard deviation; F) 'Present' uncertainty range of values; G) 'Projected' binary distribution; H) 'Present' binary distribution; I) Range size variation.

Value	Present	Projected
AUC.train	0.932	0.902
AUC.test	0.932	0.877
AUC.diff	0.046	0.046
pROC	1.684	1.527
OR.p10	0.189	0.189
CBI	0.899	0.627
perc.stable	24.172	24.172
range.size	-0.073	-0.073

S.3.213 *Phyteuma ovatum* GLM

Species: *Phyteuma ovatum* Honck. subsp. *pseudospicatum* Pignatti

Procedure: GLM

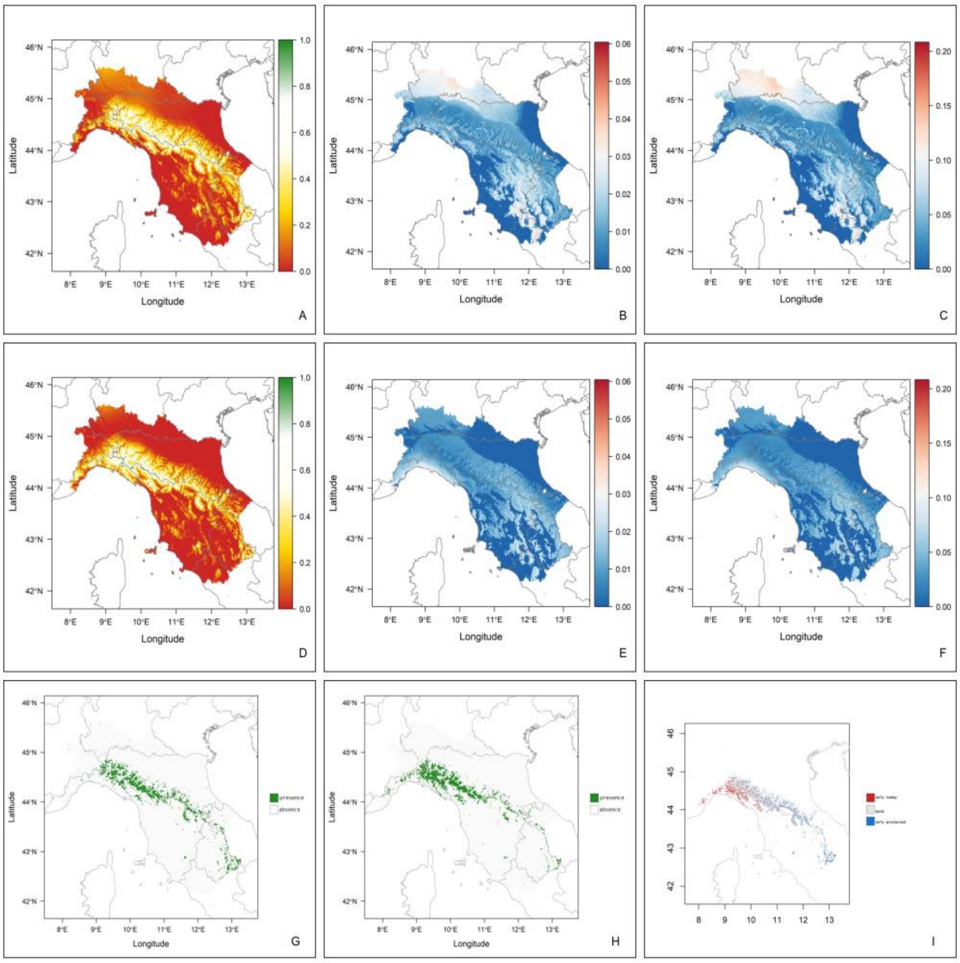

Fig. S.3.213-A) 'Projected' base output; B) 'Projected' uncertainty standard deviation; C) 'Projected' uncertainty range of values; D) 'Present' base output; E) 'Present' uncertainty standard deviation; F) 'Present' uncertainty range of values; G) 'Projected' binary distribution; H) 'Present' binary distribution; I) Range size variation.

Value	Present	Projected
AUC.train	0.908	0.887
AUC.test	0.908	0.921
AUC.diff	0.005	0.005
pROC	1.607	1.673
OR.p10	0.167	0.133
CBI	0.946	0.660
perc.stable	44.171	44.171
range.size	0.823	0.823

S.3.214 *Phyteuma ovatum* MARS

Species: *Phyteuma ovatum* Honck. subsp. *pseudospicatum* Pignatti

Procedure: MARS

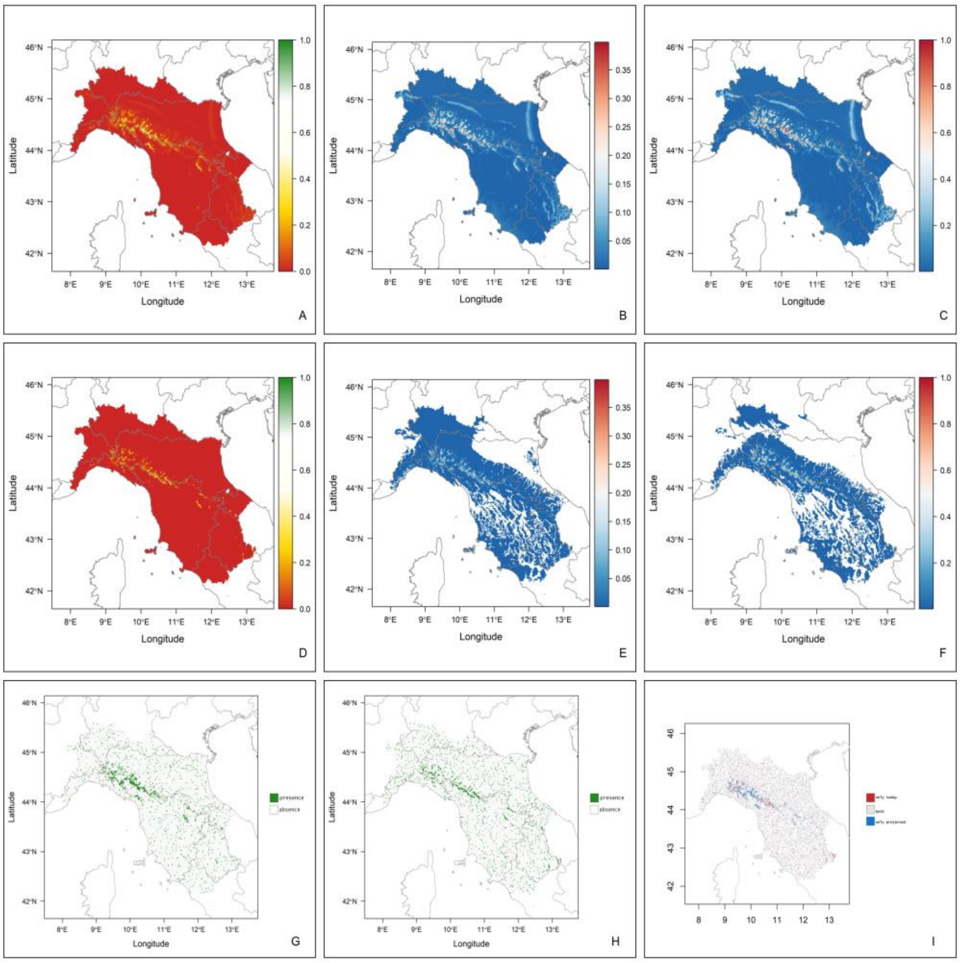

Fig. S.3.214-A) 'Projected' base output; B) 'Projected' uncertainty standard deviation; C) 'Projected' uncertainty range of values; D) 'Present' base output; E) 'Present' uncertainty standard deviation; F) 'Present' uncertainty range of values; G) 'Projected' binary distribution; H) 'Present' binary distribution; I) Range size variation.

Value	Present	Projected
AUC.train	0.766	0.910
AUC.test	0.766	0.839
AUC.diff	0.080	0.080
pROC	1.274	1.390
OR.p10	0.356	0.233
CBI	0.620	0.733
perc.stable	9.050	9.050
range.size	-2.639	-2.639

S.3.215 *Phyteuma ovatum* RF

Species: *Phyteuma ovatum* Honck. subsp. *pseudospicatum* Pignatti

Procedure: RF

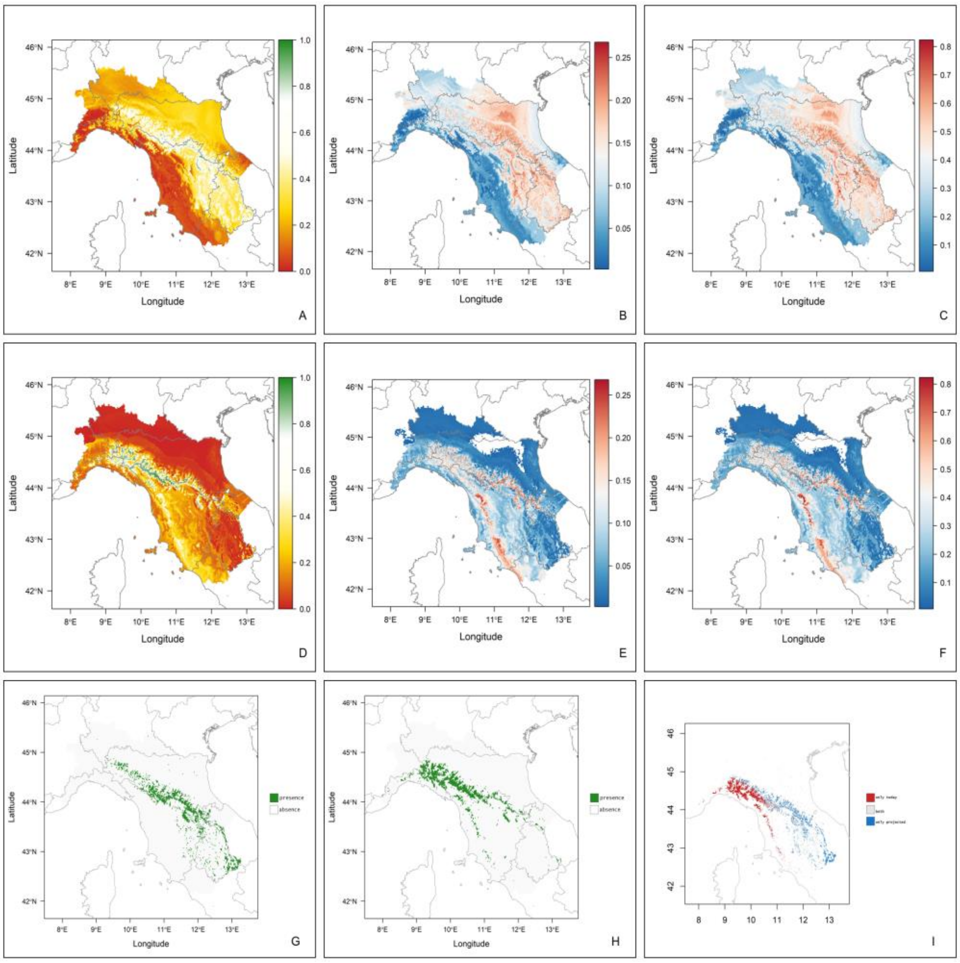

Fig. S.3.215-A) 'Projected' base output; B) 'Projected' uncertainty standard deviation; C) 'Projected' uncertainty range of values; D) 'Present' base output; E) 'Present' uncertainty standard deviation; F) 'Present' uncertainty range of values; G) 'Projected' binary distribution; H) 'Present' binary distribution; I) Range size variation.

Value	Present	Projected
AUC.train	0.993	1.000
AUC.test	0.993	0.954
AUC.diff	0.046	0.046
pROC	1.790	1.517
OR.p10	0.444	0.422
CBI	0.933	0.837
perc.stable	19.845	19.845
range.size	-1.123	-1.123

S.3.216 *Phyteuma ovatum* SVM

Species: *Phyteuma ovatum* Honck. subsp. *pseudospicatum* Pignatti

Procedure: SVM

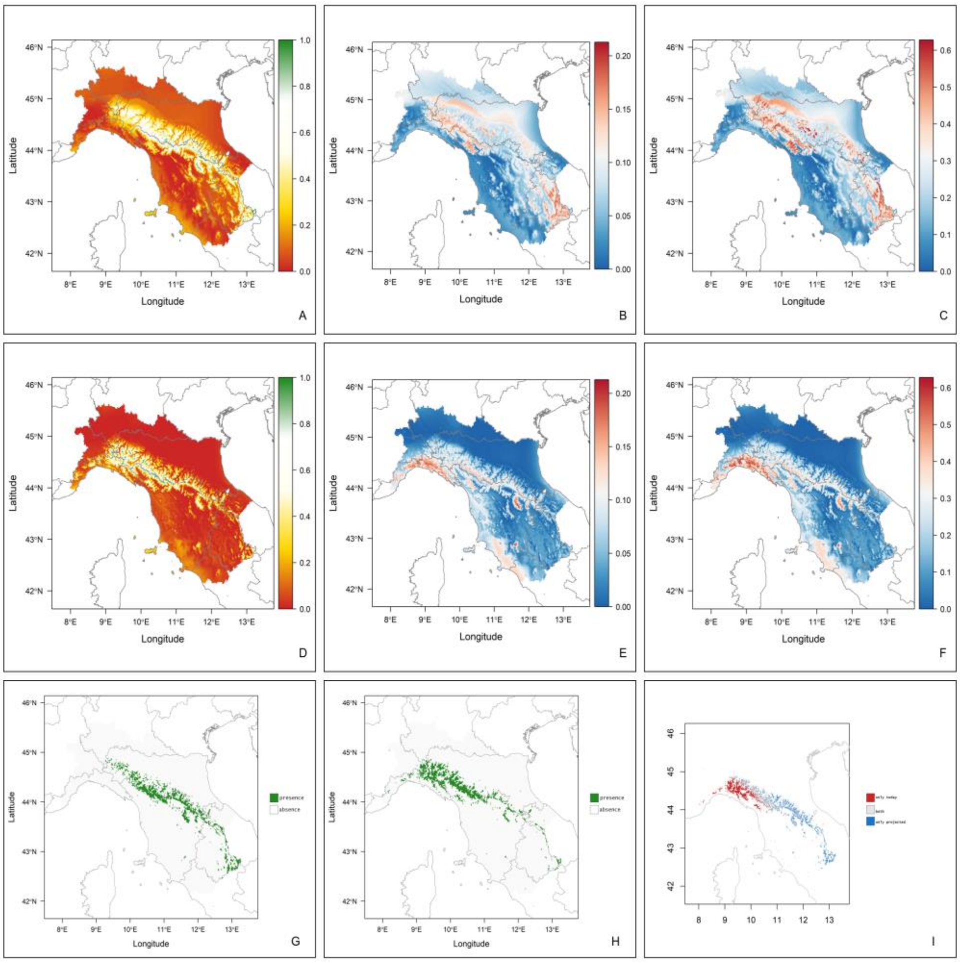

Fig. S.3.216-A) 'Projected' base output; B) 'Projected' uncertainty standard deviation; C) 'Projected' uncertainty range of values; D) 'Present' base output; E) 'Present' uncertainty standard deviation; F) 'Present' uncertainty range of values; G) 'Projected' binary distribution; H) 'Present' binary distribution; I) Range size variation.

Value	Present	Projected
AUC.train	0.947	0.936
AUC.test	0.947	0.909
AUC.diff	0.038	0.038
pROC	1.735	1.520
OR.p10	0.256	0.189
CBI	0.906	0.900
perc.stable	32.786	32.786
range.size	0.291	0.291

S.3.217 *Polygala flavescens* Biomod2

Species: *Polygala flavescens* DC. subsp. *flavescens*

Procedure: Biomod2

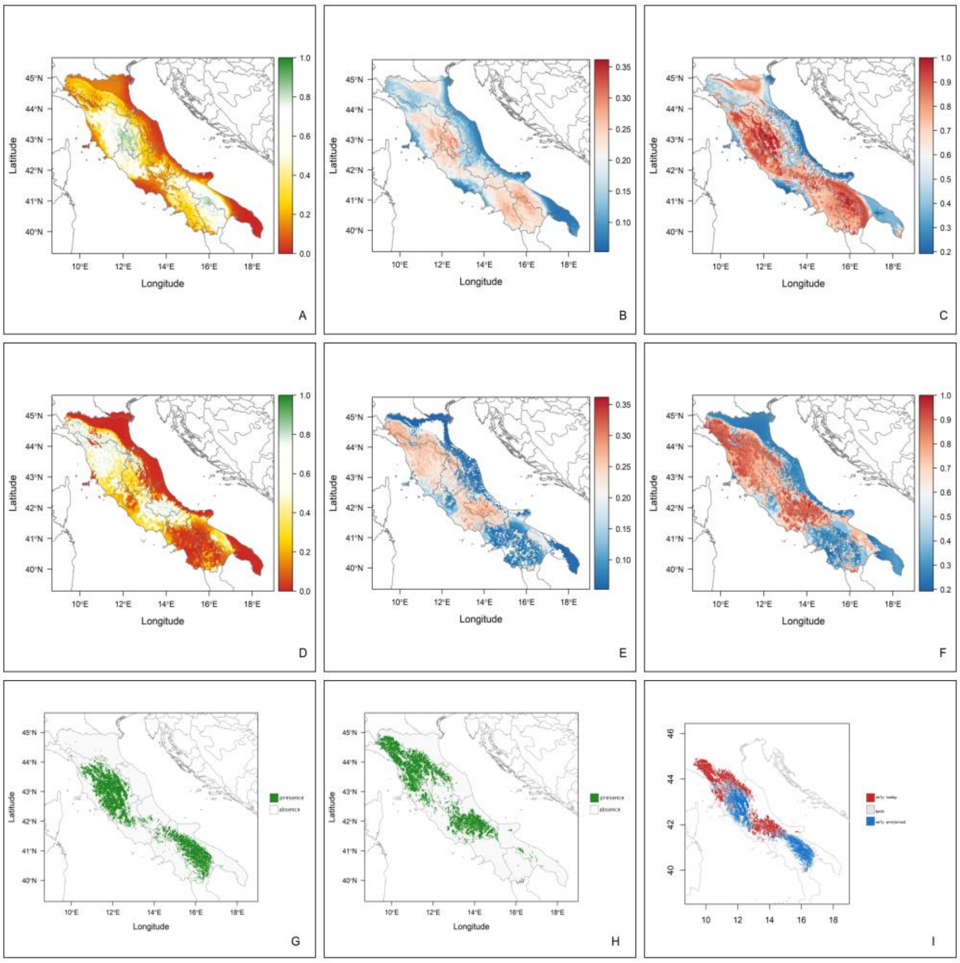

Fig. S.3.217-A) 'Projected' base output; B) 'Projected' uncertainty standard deviation; C) 'Projected' uncertainty range of values; D) 'Present' base output; E) 'Present' uncertainty standard deviation; F) 'Present' uncertainty range of values; G) 'Projected' binary distribution; H) 'Present' binary distribution; I) Range size variation.

Value	Present	Projected
AUC.train	0.780	0.923
AUC.test	0.780	0.801
AUC.diff	0.122	0.122
pROC	1.277	1.283
OR.p10	0.347	0.300
CBI	0.994	-0.280
perc.stable	12.217	12.217
range.size	0.265	0.265

S.3.218 *Polygala flavescens* Bioclim

Species: *Polygala flavescens* DC. subsp. *flavescens*

Procedure: Bioclim

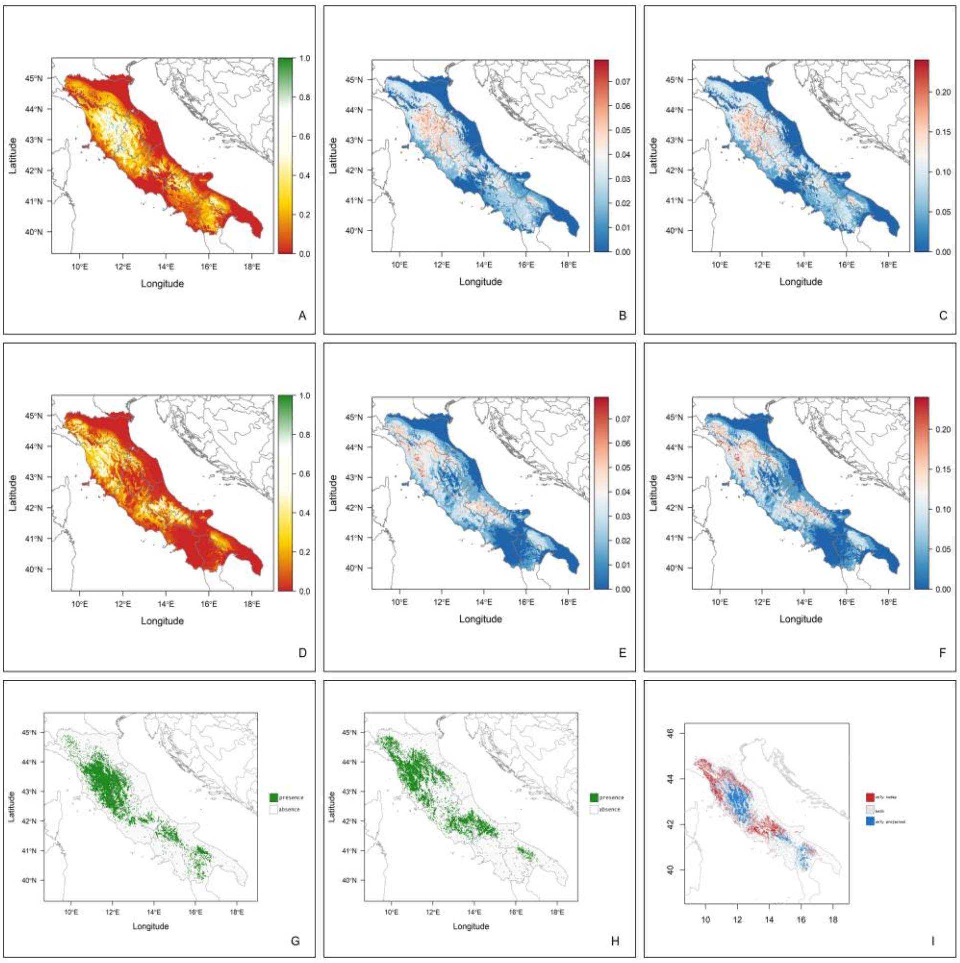

Fig. S.3.218-A) 'Projected' base output; B) 'Projected' uncertainty standard deviation; C) 'Projected' uncertainty range of values; D) 'Present' base output; E) 'Present' uncertainty standard deviation; F) 'Present' uncertainty range of values; G) 'Projected' binary distribution; H) 'Present' binary distribution; I) Range size variation.

Value	Present	Projected
AUC.train	0.727	0.729
AUC.test	0.727	0.699
AUC.diff	0.035	0.035
pROC	1.177	1.121
OR.p10	0.123	0.130
CBI	0.827	0.141
perc.stable	31.881	31.881
range.size	0.101	0.101

S.3.219 *Polygala flavescens* Domain

Species: *Polygala flavescens* DC. subsp. *flavescens*

Procedure: Domain

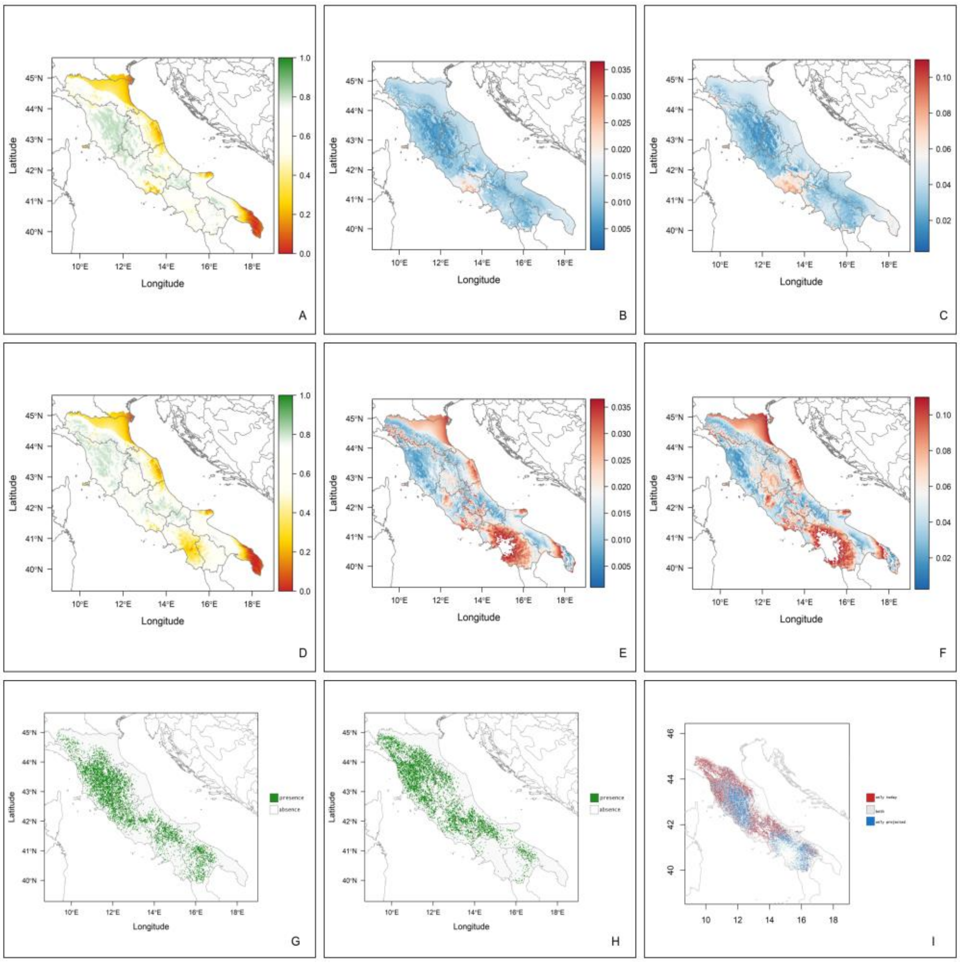

Fig. S.3.219-A) 'Projected' base output; B) 'Projected' uncertainty standard deviation; C) 'Projected' uncertainty range of values; D) 'Present' base output; E) 'Present' uncertainty standard deviation; F) 'Present' uncertainty range of values; G) 'Projected' binary distribution; H) 'Present' binary distribution; I) Range size variation.

Value	Present	Projected
AUC.train	0.712	0.701
AUC.test	0.712	0.680
AUC.diff	0.026	0.026
pROC	1.187	1.133
OR.p10	0.120	0.130
CBI	0.916	0.886
perc.stable	25.824	25.824
range.size	-0.549	-0.549

S.3.220 *Polygala flavescens* Maxent

Species: *Polygala flavescens* DC. subsp. *flavescens*

Procedure: Maxent

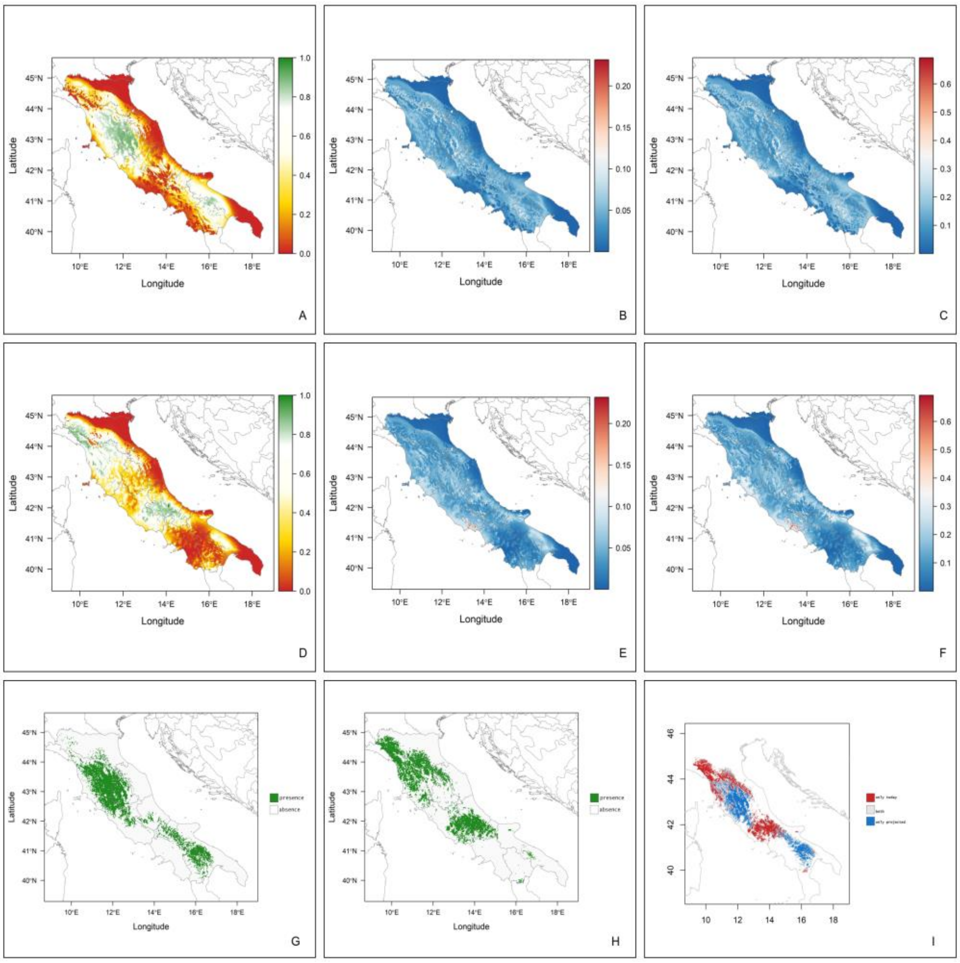

Fig. S.3.220-A) 'Projected' base output; B) 'Projected' uncertainty standard deviation; C) 'Projected' uncertainty range of values; D) 'Present' base output; E) 'Present' uncertainty standard deviation; F) 'Present' uncertainty range of values; G) 'Projected' binary distribution; H) 'Present' binary distribution; I) Range size variation.

Value	Present	Projected
AUC.train	0.760	0.801
AUC.test	0.760	0.772
AUC.diff	0.032	0.032
pROC	1.293	1.265
OR.p10	0.152	0.130
CBI	0.986	0.564
perc.stable	17.970	17.970
range.size	-0.002	-0.002

S.3.221 *Polygala flavescens* KDE

Species: *Polygala flavescens* DC. subsp. *flavescens*

Procedure: KDE

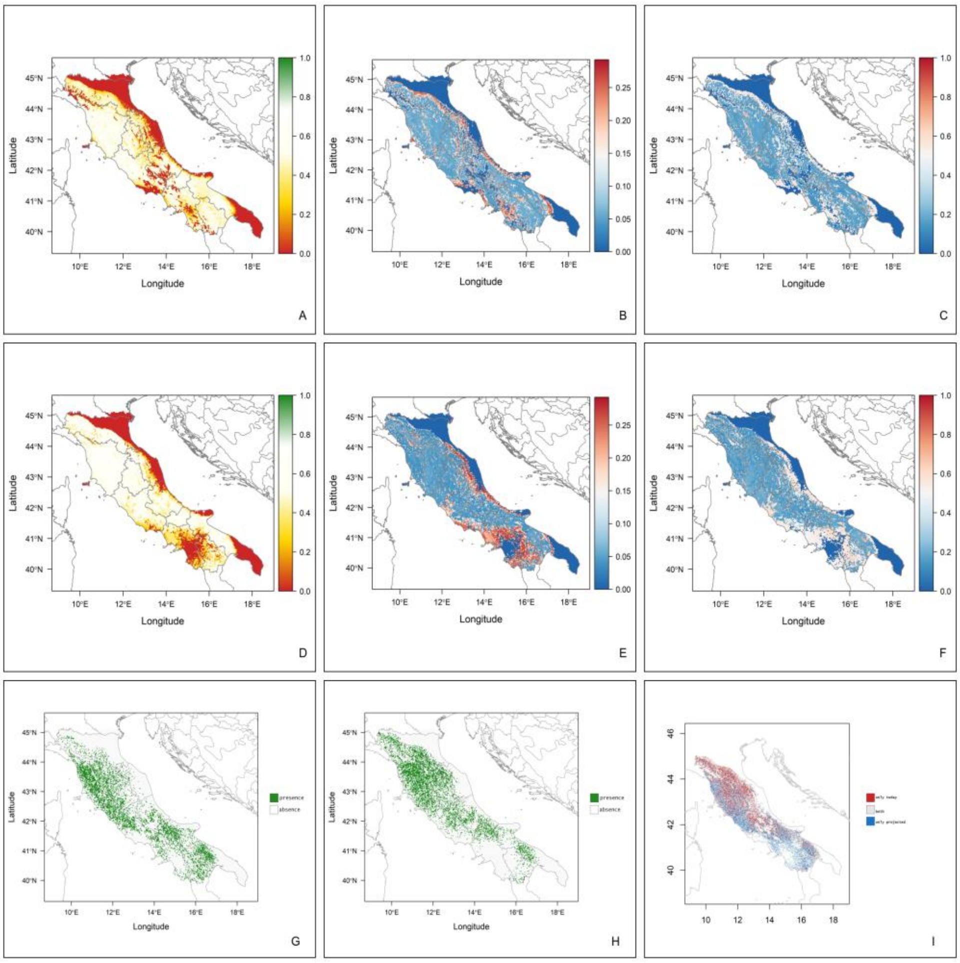

Fig. S.3.221-A) 'Projected' base output; B) 'Projected' uncertainty standard deviation; C) 'Projected' uncertainty range of values; D) 'Present' base output; E) 'Present' uncertainty standard deviation; F) 'Present' uncertainty range of values; G) 'Projected' binary distribution; H) 'Present' binary distribution; I) Range size variation.

Value	Present	Projected
AUC.train	0.657	0.650
AUC.test	0.657	0.614
AUC.diff	0.049	0.039
pROC	1.120	1.096
OR.p10	0.156	0.114
CBI	0.803	0.742
perc.stable	23.506	23.506
range.size	-0.385	-0.385

S.3.222 *Polygala flavescens* CTA

Species: *Polygala flavescens* DC. subsp. *flavescens*

Procedure: CTA

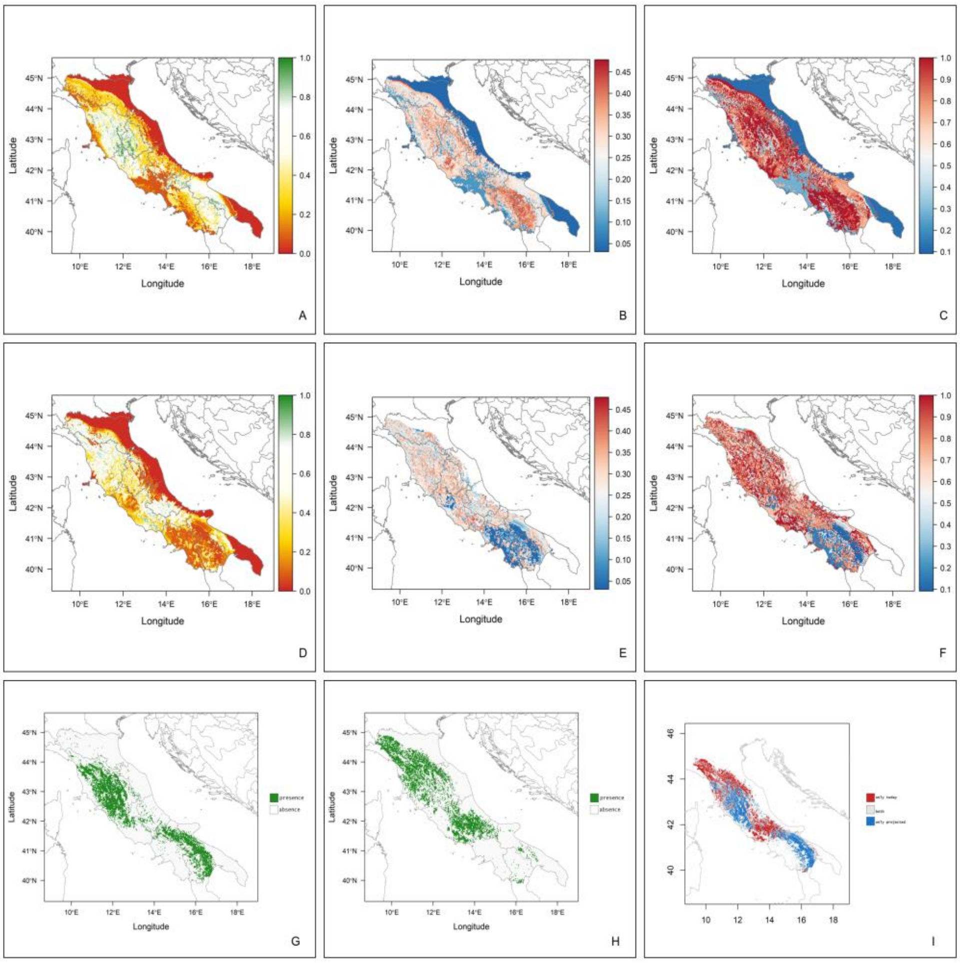

Fig. S.3.222-A) 'Projected' base output; B) 'Projected' uncertainty standard deviation; C) 'Projected' uncertainty range of values; D) 'Present' base output; E) 'Present' uncertainty standard deviation; F) 'Present' uncertainty range of values; G) 'Projected' binary distribution; H) 'Present' binary distribution; I) Range size variation.

Value	Present	Projected
AUC.train	0.786	0.903
AUC.test	0.786	0.828
AUC.diff	0.075	0.075
pROC	1.121	1.166
OR.p10	0.273	0.235
CBI	0.963	-0.136
perc.stable	13.508	13.508
range.size	0.538	0.538

S.3.223 *Polygala flavescens* FDA

Species: *Polygala flavescens* DC. subsp. *flavescens*

Procedure: FDA

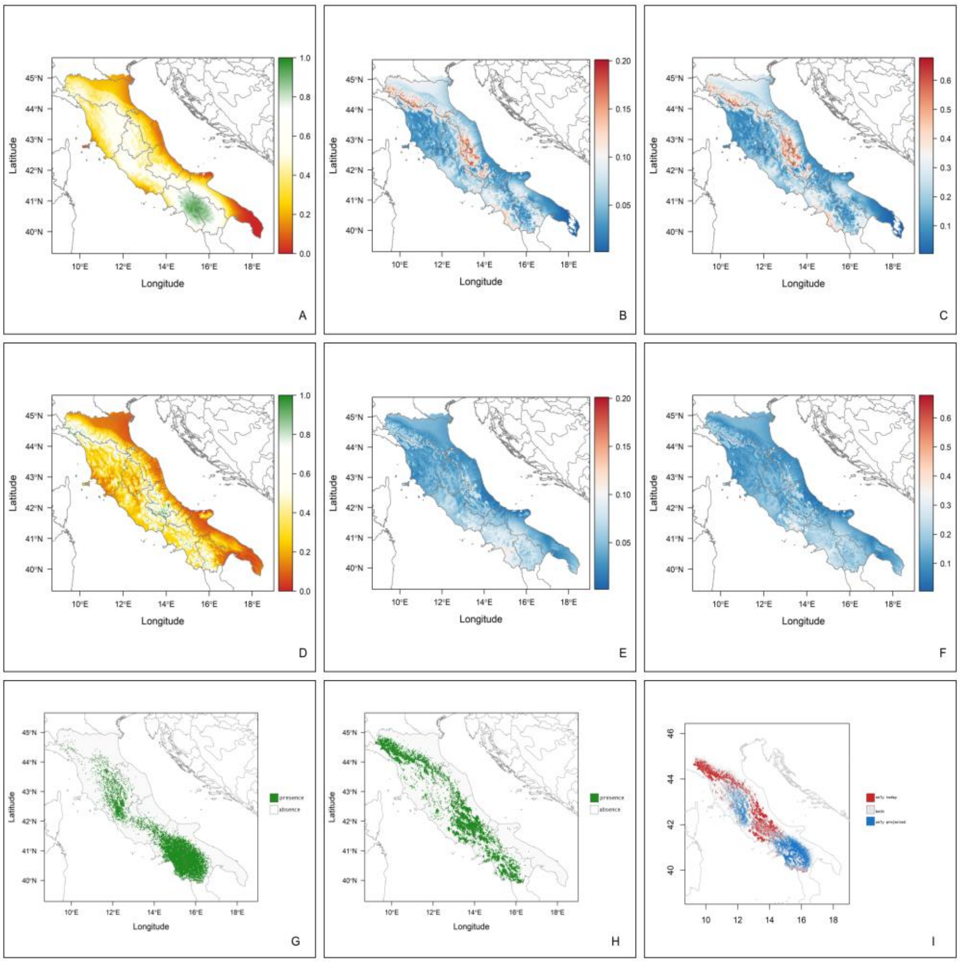

Fig. S.3.223-A) 'Projected' base output; B) 'Projected' uncertainty standard deviation; C) 'Projected' uncertainty range of values; D) 'Present' base output; E) 'Present' uncertainty standard deviation; F) 'Present' uncertainty range of values; G) 'Projected' binary distribution; H) 'Present' binary distribution; I) Range size variation.

Value	Present	Projected
AUC.train	0.702	0.736
AUC.test	0.702	0.733
AUC.diff	0.014	0.014
pROC	1.166	1.225
OR.p10	0.100	0.119
CBI	0.954	0.332
perc.stable	21.380	21.380
range.size	-0.158	-0.158

S.3.224 *Polygala flavescens* GAM

Species: *Polygala flavescens* DC. subsp. *flavescens*

Procedure: GAM

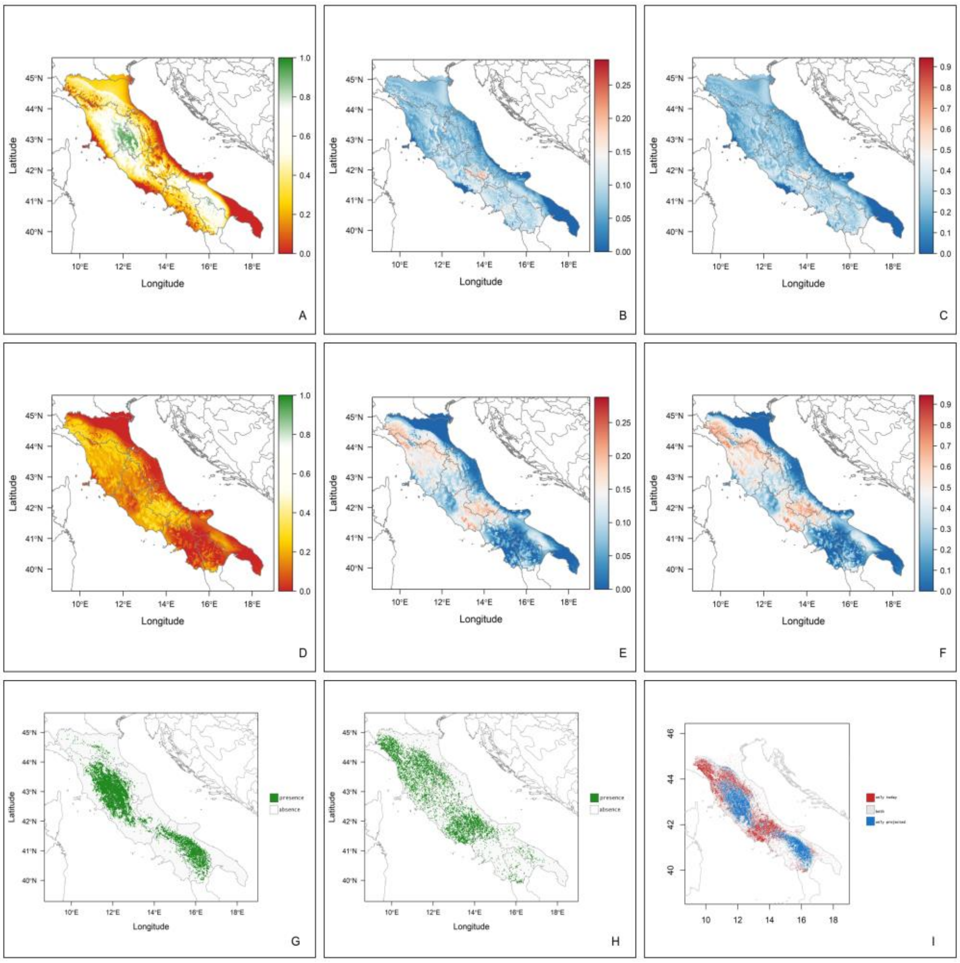

Fig. S.3.224-A) 'Projected' base output; B) 'Projected' uncertainty standard deviation; C) 'Projected' uncertainty range of values; D) 'Present' base output; E) 'Present' uncertainty standard deviation; F) 'Present' uncertainty range of values; G) 'Projected' binary distribution; H) 'Present' binary distribution; I) Range size variation.

Value	Present	Projected
AUC.train	0.791	0.780
AUC.test	0.791	0.756
AUC.diff	0.033	0.033
pROC	1.325	1.232
OR.p10	0.108	0.142
CBI	0.817	0.151
perc.stable	13.464	13.464
range.size	0.075	0.075

S.3.225 *Polygala flavescens* GLM

Species: *Polygala flavescens* DC. subsp. *flavescens*

Procedure: GLM

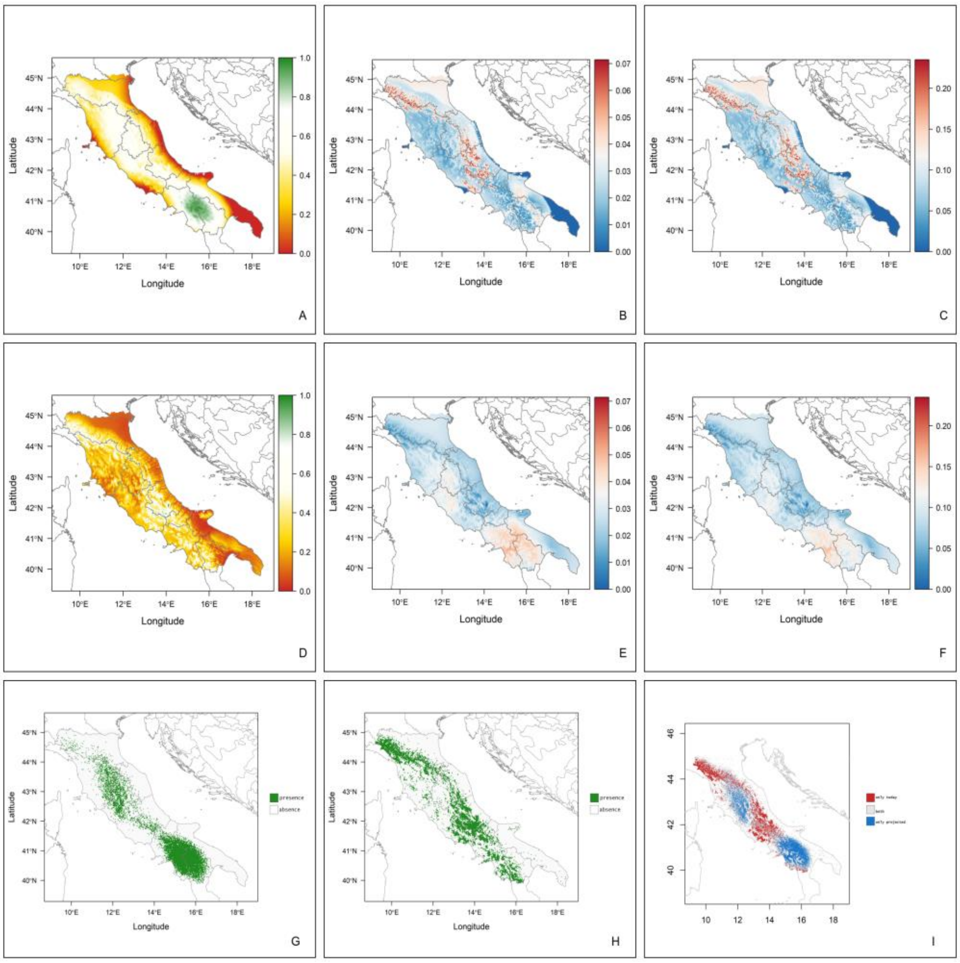

Fig. S.3.225-A) 'Projected' base output; B) 'Projected' uncertainty standard deviation; C) 'Projected' uncertainty range of values; D) 'Present' base output; E) 'Present' uncertainty standard deviation; F) 'Present' uncertainty range of values; G) 'Projected' binary distribution; H) 'Present' binary distribution; I) Range size variation.

Value	Present	Projected
AUC.train	0.692	0.717
AUC.test	0.692	0.720
AUC.diff	0.012	0.012
pROC	1.170	1.239
OR.p10	0.080	0.112
CBI	0.941	0.415
perc.stable	19.039	19.039
range.size	-0.211	-0.211

S.3.226 *Polygala flavescens* MARS

Species: *Polygala flavescens* DC. subsp. *flavescens*

Procedure: MARS

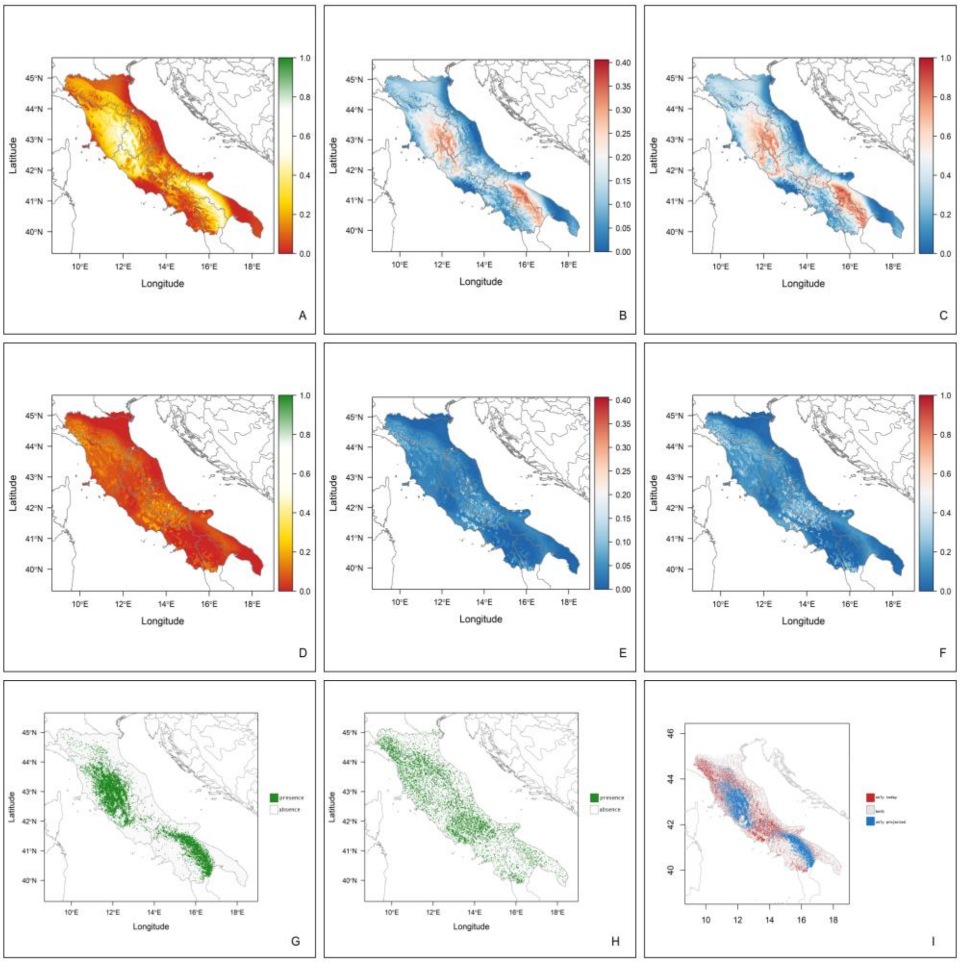

Fig. S.3.226-A) 'Projected' base output; B) 'Projected' uncertainty standard deviation; C) 'Projected' uncertainty range of values; D) 'Present' base output; E) 'Present' uncertainty standard deviation; F) 'Present' uncertainty range of values; G) 'Projected' binary distribution; H) 'Present' binary distribution; I) Range size variation.

Value	Present	Projected
AUC.train	0.742	0.804
AUC.test	0.742	0.772
AUC.diff	0.034	0.034
pROC	1.222	1.234
OR.p10	0.148	0.135
CBI	0.893	-0.293
perc.stable	11.326	11.326
range.size	-0.196	-0.196

S.3.227 *Polygala flavescens* RF

Species: *Polygala flavescens* DC. subsp. *flavescens*

Procedure: RF

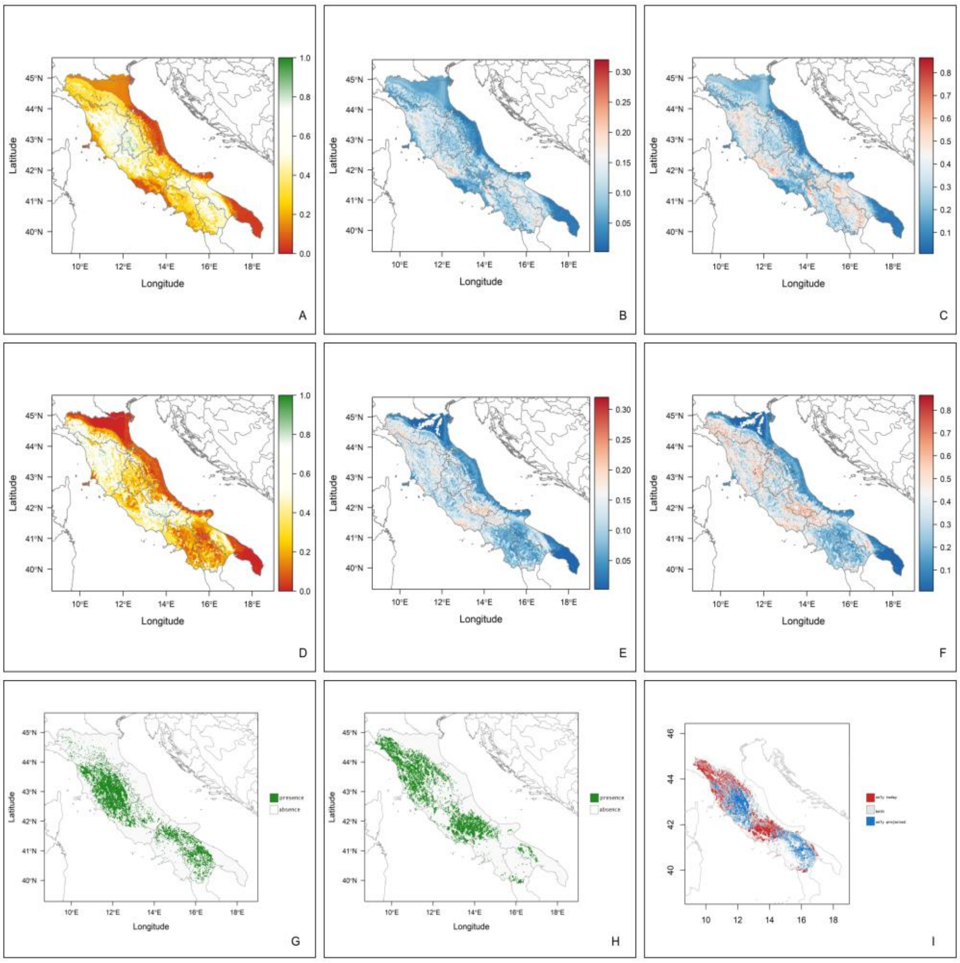

Fig. S.3.227-A) 'Projected' base output; B) 'Projected' uncertainty standard deviation; C) 'Projected' uncertainty range of values; D) 'Present' base output; E) 'Present' uncertainty standard deviation; F) 'Present' uncertainty range of values; G) 'Projected' binary distribution; H) 'Present' binary distribution; I) Range size variation.

Value	Present	Projected
AUC.train	0.962	1.000
AUC.test	0.962	0.946
AUC.diff	0.054	0.054
pROC	1.281	1.231
OR.p10	0.717	0.707
CBI	0.988	0.319
perc.stable	16.868	16.868
range.size	-0.864	-0.864

S.3.228 *Polygala flavescens* SVM

Species: *Polygala flavescens* DC. subsp. *flavescens*

Procedure: SVM

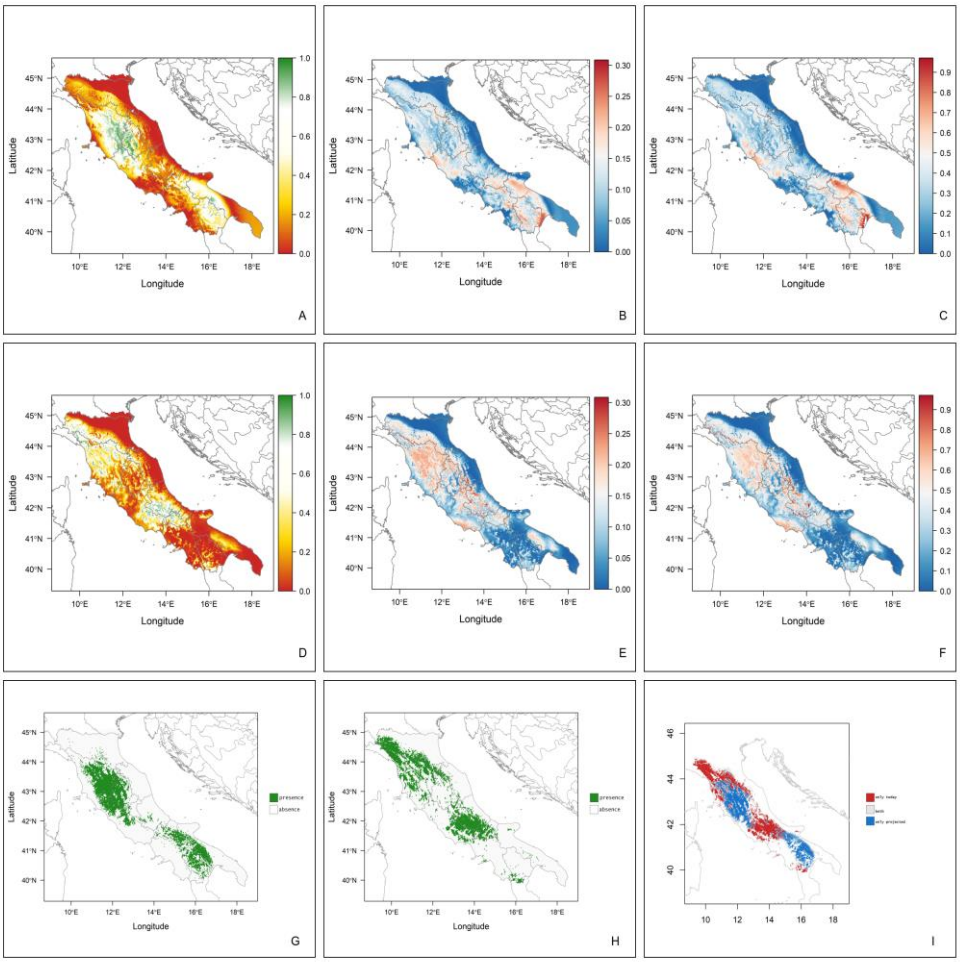

Fig. S.3.228-A) 'Projected' base output; B) 'Projected' uncertainty standard deviation; C) 'Projected' uncertainty range of values; D) 'Present' base output; E) 'Present' uncertainty standard deviation; F) 'Present' uncertainty range of values; G) 'Projected' binary distribution; H) 'Present' binary distribution; I) Range size variation.

Value	Present	Projected
AUC.train	0.766	0.810
AUC.test	0.766	0.788
AUC.diff	0.025	0.025
pROC	1.210	1.198
OR.p10	0.138	0.123
CBI	0.970	0.006
perc.stable	8.646	8.646
range.size	0.328	0.328

S.3.229 *Ranunculus pollinensis* Biomod2

Species: *Ranunculus pollinensis* (N.Terracc.) Chiov.

Procedure: Biomod2

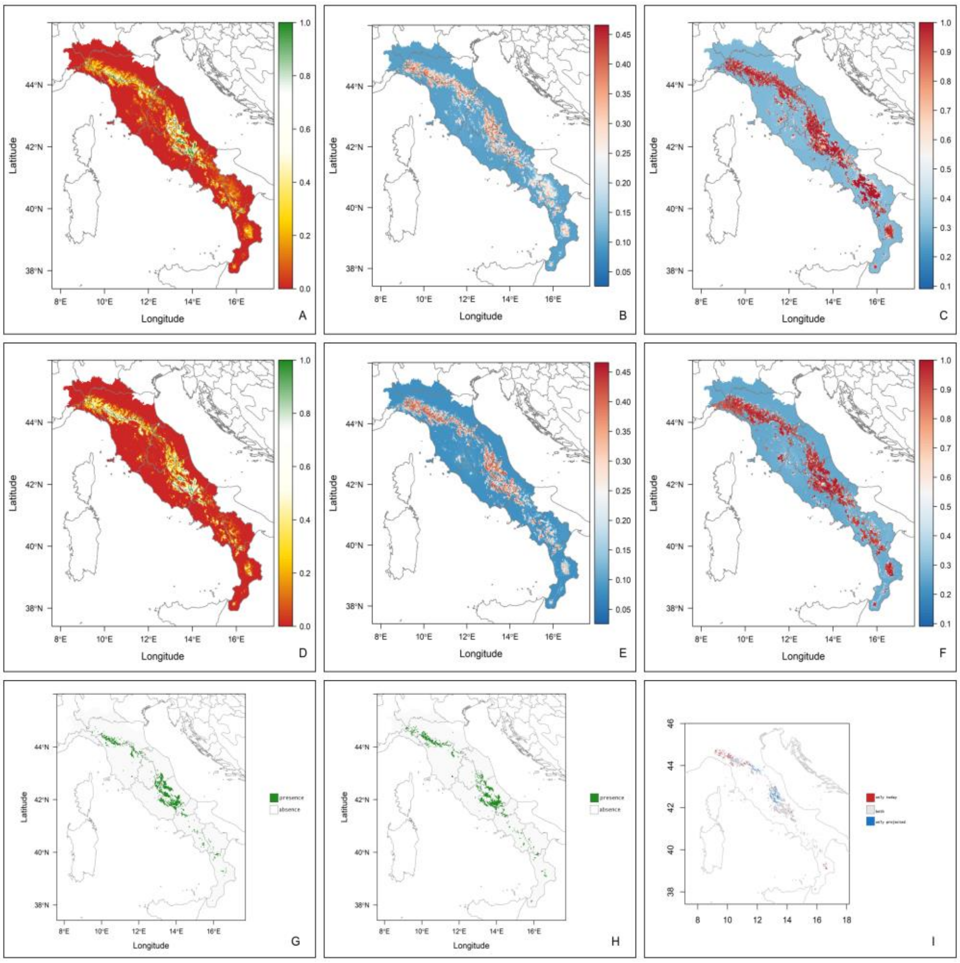

Fig. S.3.229-A) 'Projected' base output; B) 'Projected' uncertainty standard deviation; C) 'Projected' uncertainty range of values; D) 'Present' base output; E) 'Present' uncertainty standard deviation; F) 'Present' uncertainty range of values; G) 'Projected' binary distribution; H) 'Present' binary distribution; I) Range size variation.

Value	Present	Projected
AUC.train	0.980	0.992
AUC.test	0.980	0.979
AUC.diff	0.013	0.013
pROC	1.908	1.890
OR.p10	0.600	0.167
CBI	0.989	0.953
perc.stable	52.133	52.133
range.size	2.339	2.339

S.3.230 *Ranunculus pollinensis* Bioclim

Species: *Ranunculus pollinensis* (N.Terracc.) Chiov.

Procedure: Bioclim

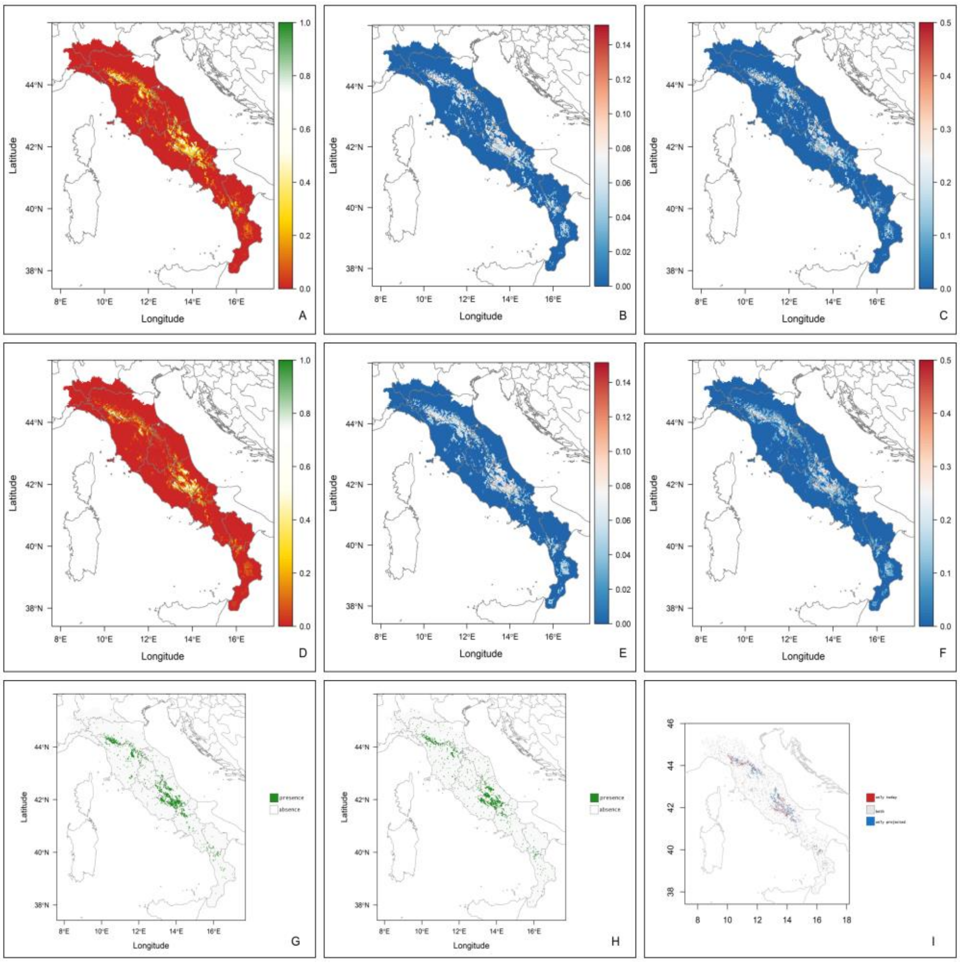

Fig. S.3.230-A) 'Projected' base output; B) 'Projected' uncertainty standard deviation; C) 'Projected' uncertainty range of values; D) 'Present' base output; E) 'Present' uncertainty standard deviation; F) 'Present' uncertainty range of values; G) 'Projected' binary distribution; H) 'Present' binary distribution; I) Range size variation.

Value	Present	Projected
AUC.train	0.790	0.936
AUC.test	0.790	0.834
AUC.diff	0.103	0.103
pROC	1.192	1.269
OR.p10	0.322	0.222
CBI	0.471	0.683
perc.stable	29.534	29.534
range.size	0.442	0.442

S.3.231 *Ranunculus pollinensis* Domain

Species: *Ranunculus pollinensis* (N.Terracc.) Chiov.

Procedure: Domain

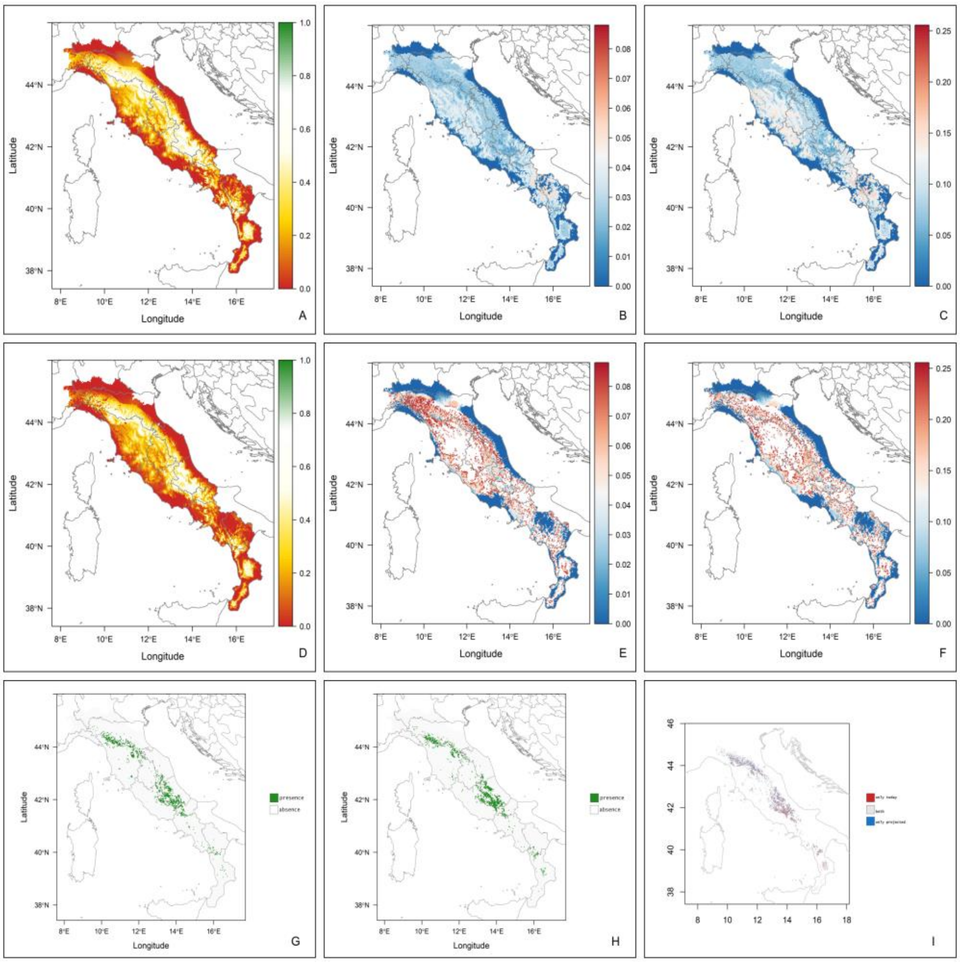

Fig. S.3.231-A) 'Projected' base output; B) 'Projected' uncertainty standard deviation; C) 'Projected' uncertainty range of values; D) 'Present' base output; E) 'Present' uncertainty standard deviation; F) 'Present' uncertainty range of values; G) 'Projected' binary distribution; H) 'Present' binary distribution; I) Range size variation.

Value	Present	Projected
AUC.train	0.923	0.933
AUC.test	0.923	0.937
AUC.diff	0.006	0.006
pROC	1.730	1.745
OR.p10	0.267	0.067
CBI	0.992	0.980
perc.stable	39.373	39.373
range.size	-0.032	-0.032

S.3.232 *Ranunculus pollinensis* Maxent

Species: *Ranunculus pollinensis* (N.Terracc.) Chiov.

Procedure: Maxent

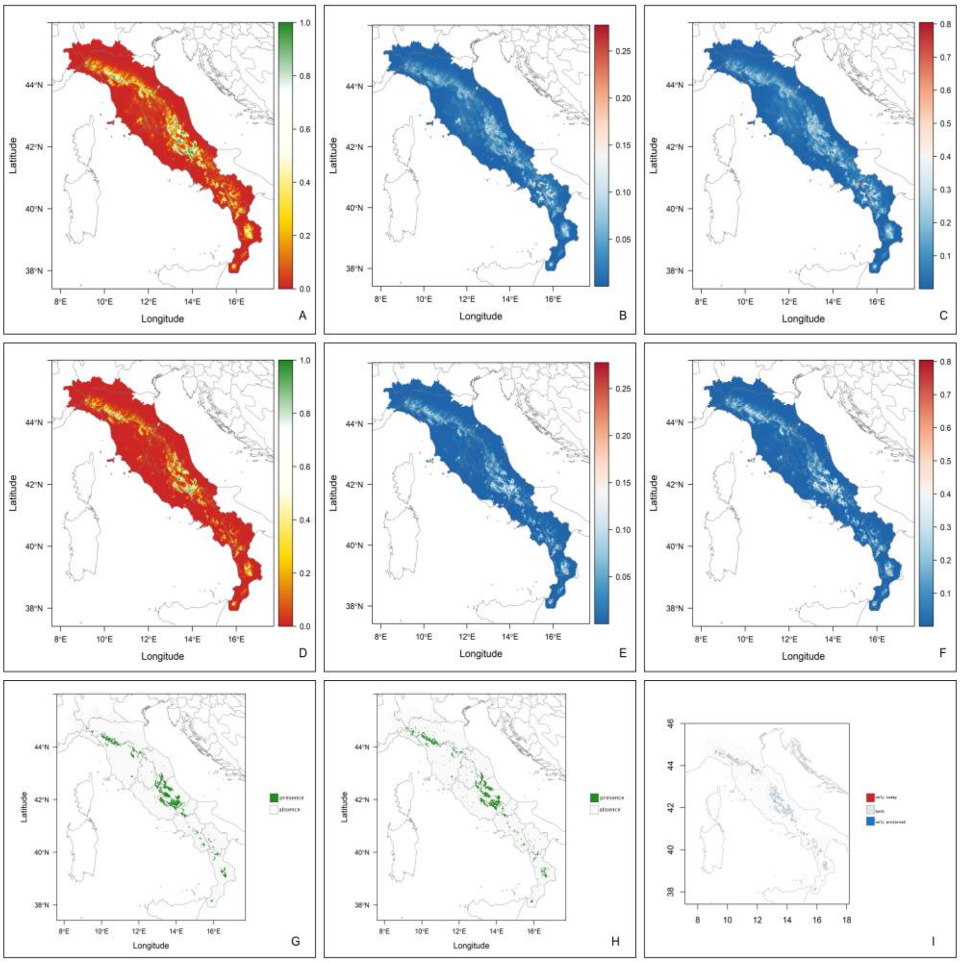

Fig. S.3.232-A) 'Projected' base output; B) 'Projected' uncertainty standard deviation; C) 'Projected' uncertainty range of values; D) 'Present' base output; E) 'Present' uncertainty standard deviation; F) 'Present' uncertainty range of values; G) 'Projected' binary distribution; H) 'Present' binary distribution; I) Range size variation.

Value	Present	Projected
AUC.train	0.962	0.958
AUC.test	0.962	0.954
AUC.diff	0.015	0.015
pROC	1.818	1.794
OR.p10	0.189	0.133
CBI	0.983	0.978
perc.stable	56.595	56.595
range.size	1.065	1.065

S.3.233 *Ranunculus pollinensis* KDE

Species: *Ranunculus pollinensis* (N.Terracc.) Chiov.

Procedure: KDE

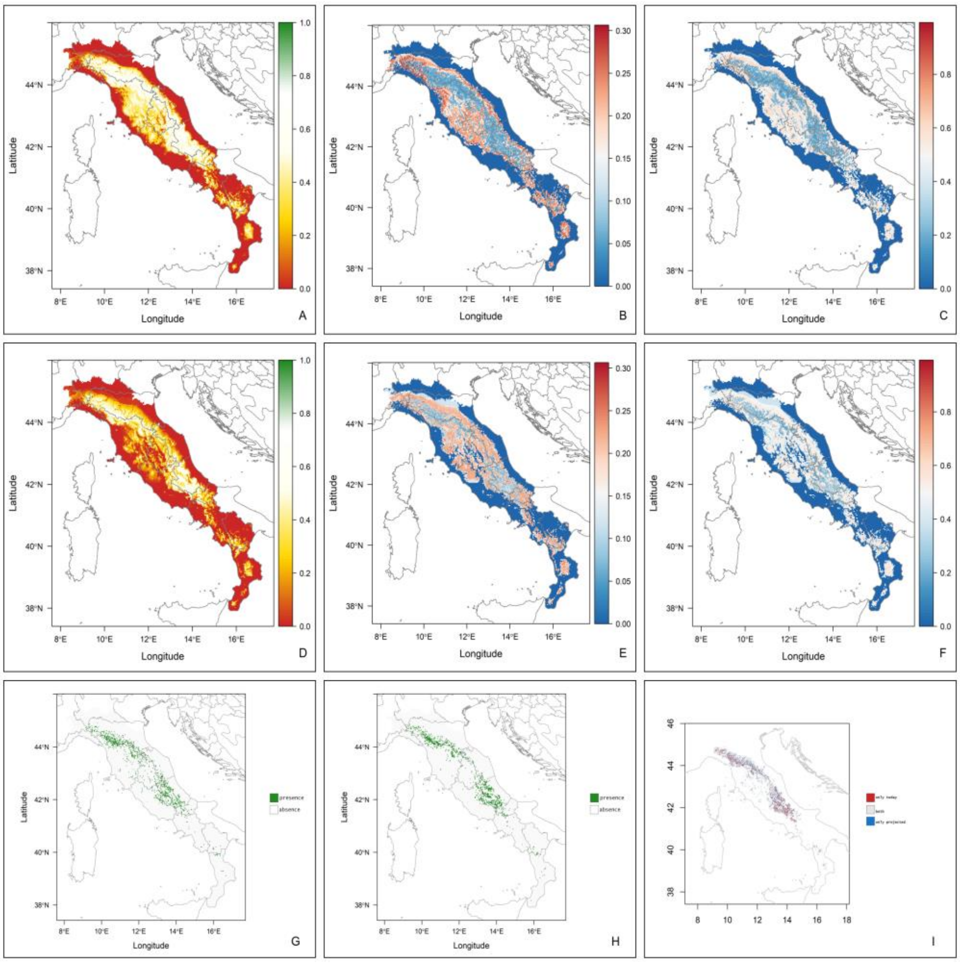

Fig. S.3.233-A) 'Projected' base output; B) 'Projected' uncertainty standard deviation; C) 'Projected' uncertainty range of values; D) 'Present' base output; E) 'Present' uncertainty standard deviation; F) 'Present' uncertainty range of values; G) 'Projected' binary distribution; H) 'Present' binary distribution; I) Range size variation.

Value	Present	Projected
AUC.train	0.875	0.929
AUC.test	0.875	0.884
AUC.diff	0.071	0.053
pROC	1.487	1.544
OR.p10	0.256	0.211
CBI	0.958	0.967
perc.stable	21.657	21.657
range.size	3.360	3.360

S.3.234 *Ranunculus pollinensis* CTA

Species: *Ranunculus pollinensis* (N.Terracc.) Chiov.

Procedure: CTA

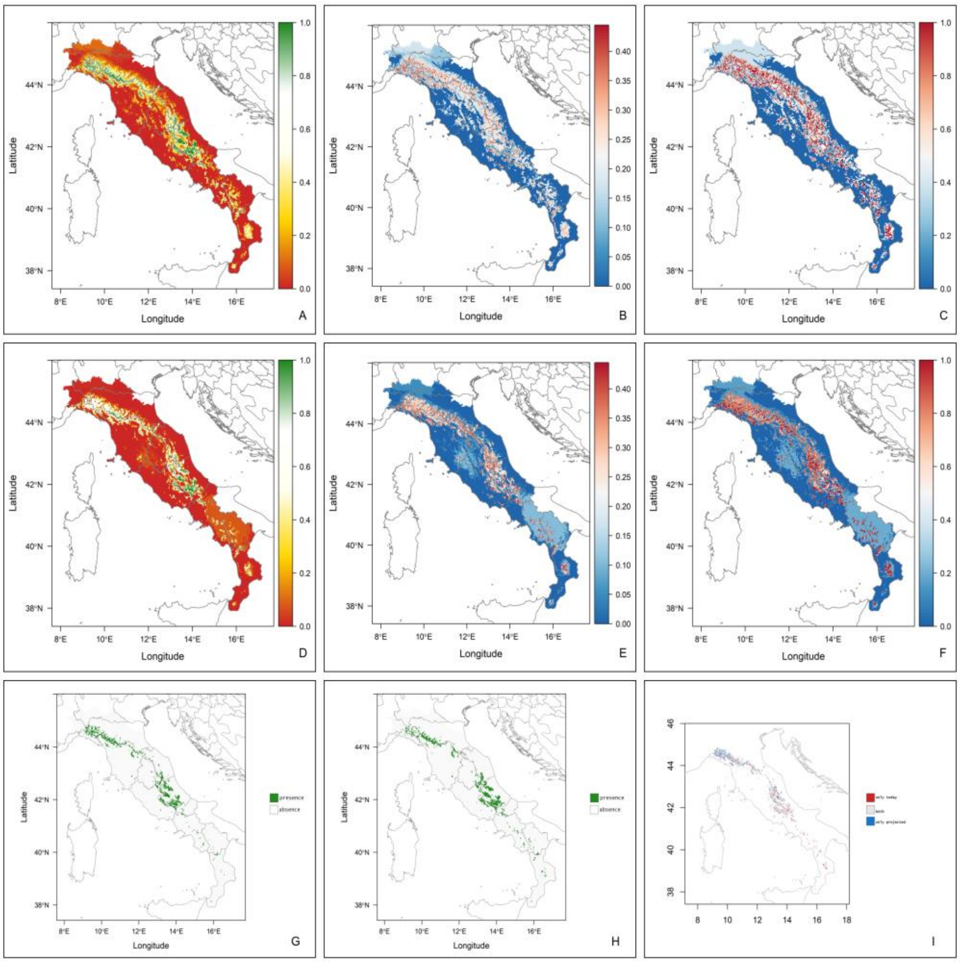

Fig. S.3.234-A) 'Projected' base output; B) 'Projected' uncertainty standard deviation; C) 'Projected' uncertainty range of values; D) 'Present' base output; E) 'Present' uncertainty standard deviation; F) 'Present' uncertainty range of values; G) 'Projected' binary distribution; H) 'Present' binary distribution; I) Range size variation.

Value	Present	Projected
AUC.train	0.898	0.981
AUC.test	0.898	0.919
AUC.diff	0.062	0.062
pROC	1.318	1.265
OR.p10	0.211	0.144
CBI	0.909	0.852
perc.stable	52.497	52.497
range.size	-1.276	-1.276

S.3.235 *Ranunculus pollinensis* FDA

Species: *Ranunculus pollinensis* (N.Terracc.) Chiov.

Procedure: FDA

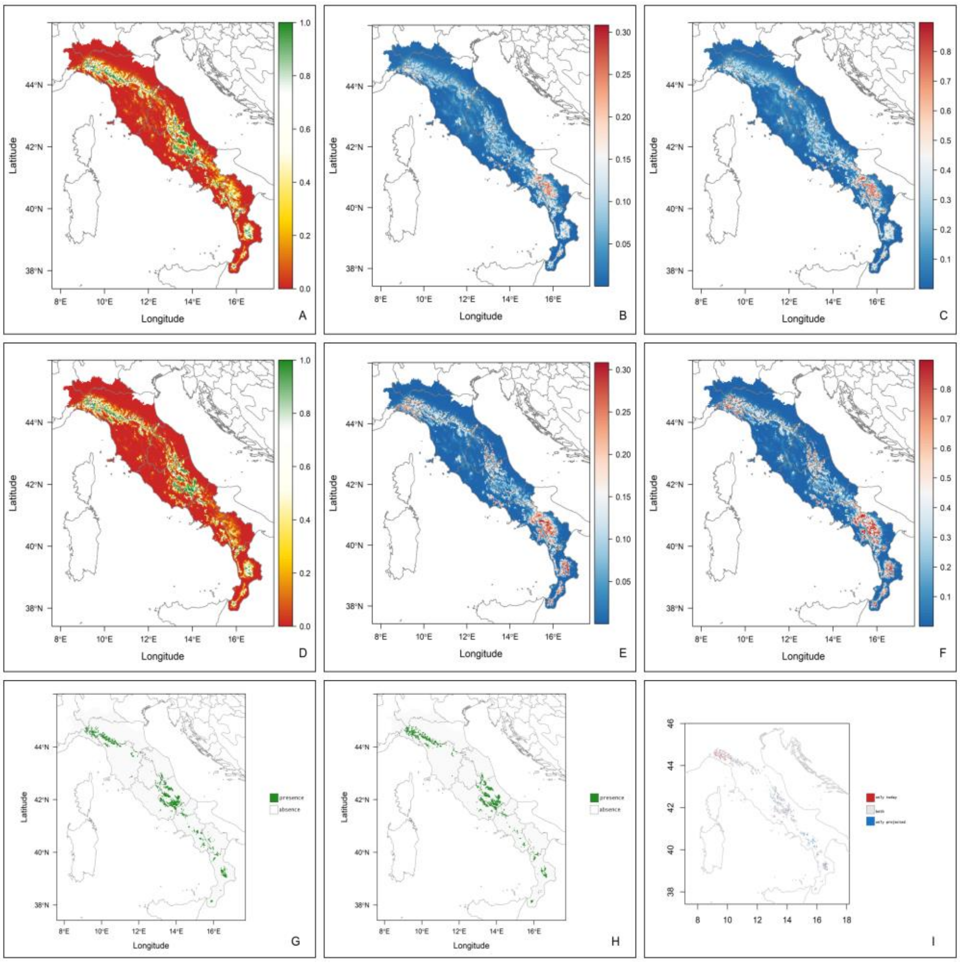

Fig. S.3.235-A) 'Projected' base output; B) 'Projected' uncertainty standard deviation; C) 'Projected' uncertainty range of values; D) 'Present' base output; E) 'Present' uncertainty standard deviation; F) 'Present' uncertainty range of values; G) 'Projected' binary distribution; H) 'Present' binary distribution; I) Range size variation.

Value	Present	Projected
AUC.train	0.953	0.958
AUC.test	0.953	0.948
AUC.diff	0.015	0.015
pROC	1.735	1.733
OR.p10	0.200	0.133
CBI	0.908	0.917
perc.stable	65.906	65.906
range.size	3.042	3.042

S.3.236 *Ranunculus pollinensis* GAM

Species: *Ranunculus pollinensis* (N.Terracc.) Chiov.

Procedure: GAM

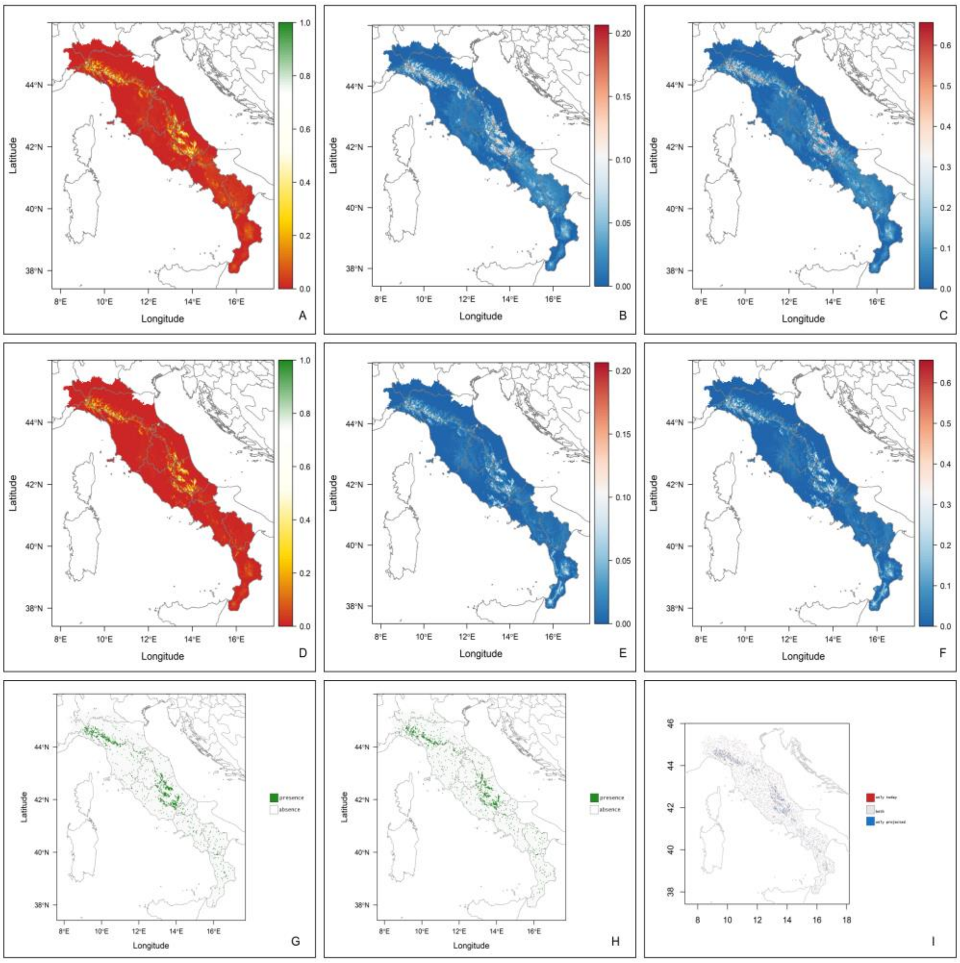

Fig. S.3.236-A) 'Projected' base output; B) 'Projected' uncertainty standard deviation; C) 'Projected' uncertainty range of values; D) 'Present' base output; E) 'Present' uncertainty standard deviation; F) 'Present' uncertainty range of values; G) 'Projected' binary distribution; H) 'Present' binary distribution; I) Range size variation.

Value	Present	Projected
AUC.train	0.949	0.943
AUC.test	0.949	0.912
AUC.diff	0.035	0.035
pROC	1.775	1.630
OR.p10	0.200	0.222
CBI	0.921	0.989
perc.stable	24.884	24.884
range.size	-1.581	-1.581

S.3.237 *Ranunculus pollinensis* GLM

Species: *Ranunculus pollinensis* (N.Terracc.) Chiov.

Procedure: GLM

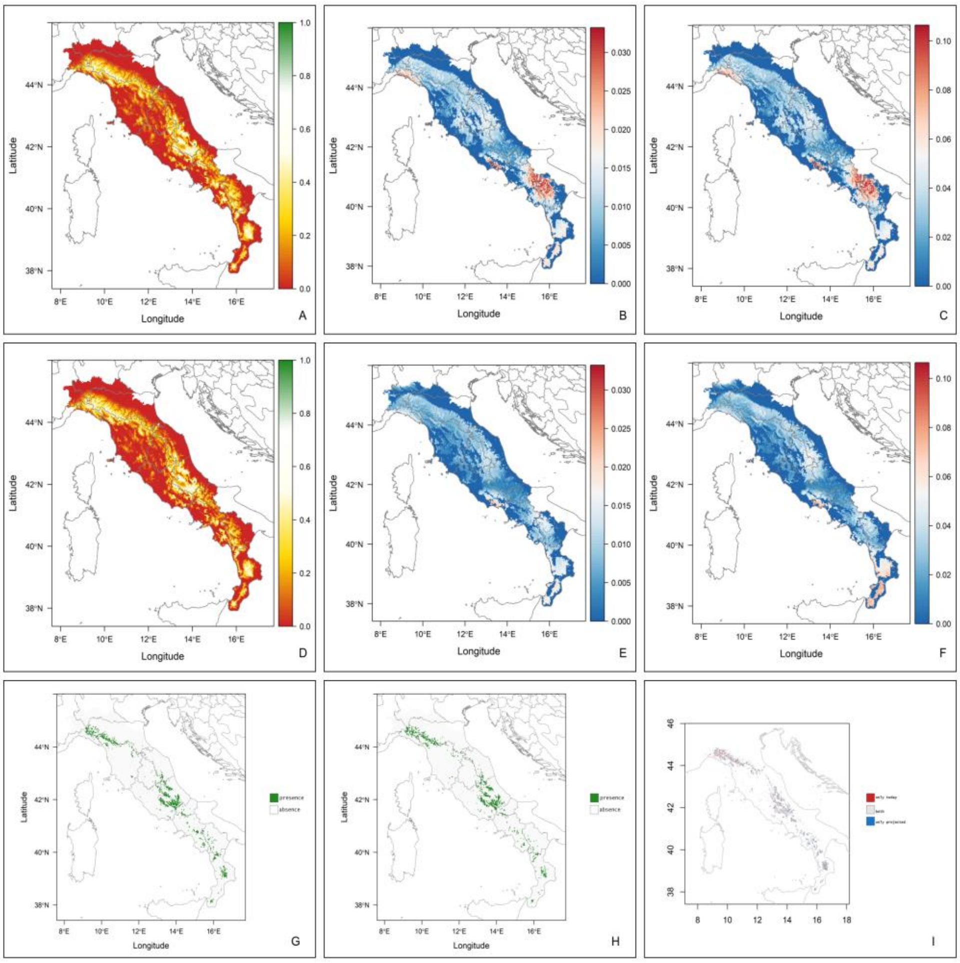

Fig. S.3.237-A) 'Projected' base output; B) 'Projected' uncertainty standard deviation; C) 'Projected' uncertainty range of values; D) 'Present' base output; E) 'Present' uncertainty standard deviation; F) 'Present' uncertainty range of values; G) 'Projected' binary distribution; H) 'Present' binary distribution; I) Range size variation.

Value	Present	Projected
AUC.train	0.970	0.946
AUC.test	0.970	0.939
AUC.diff	0.018	0.018
pROC	1.864	1.744
OR.p10	0.044	0.167
CBI	0.980	0.984
perc.stable	45.058	45.058
range.size	2.343	2.343

S.3.238 *Ranunculus pollinensis* MARS

Species: *Ranunculus pollinensis* (N.Terracc.) Chiov.

Procedure: MARS

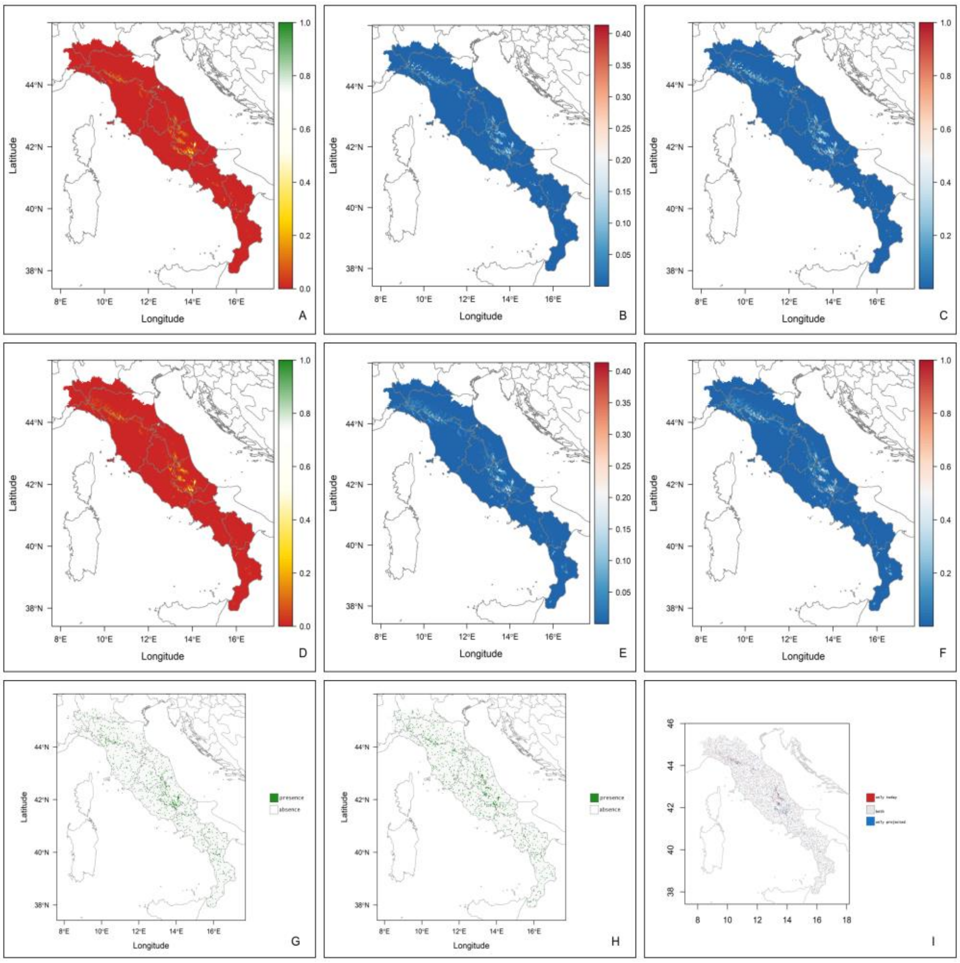

Fig. S.3.238-A) 'Projected' base output; B) 'Projected' uncertainty standard deviation; C) 'Projected' uncertainty range of values; D) 'Present' base output; E) 'Present' uncertainty standard deviation; F) 'Present' uncertainty range of values; G) 'Projected' binary distribution; H) 'Present' binary distribution; I) Range size variation.

Value	Present	Projected
AUC.train	0.874	0.943
AUC.test	0.874	0.908
AUC.diff	0.044	0.044
pROC	1.485	1.552
OR.p10	0.189	0.156
CBI	0.968	0.939
perc.stable	6.976	6.976
range.size	-2.855	-2.855

S.3.239 *Ranunculus pollinensis* RF

Species: *Ranunculus pollinensis* (N.Terracc.) Chiov.

Procedure: RF

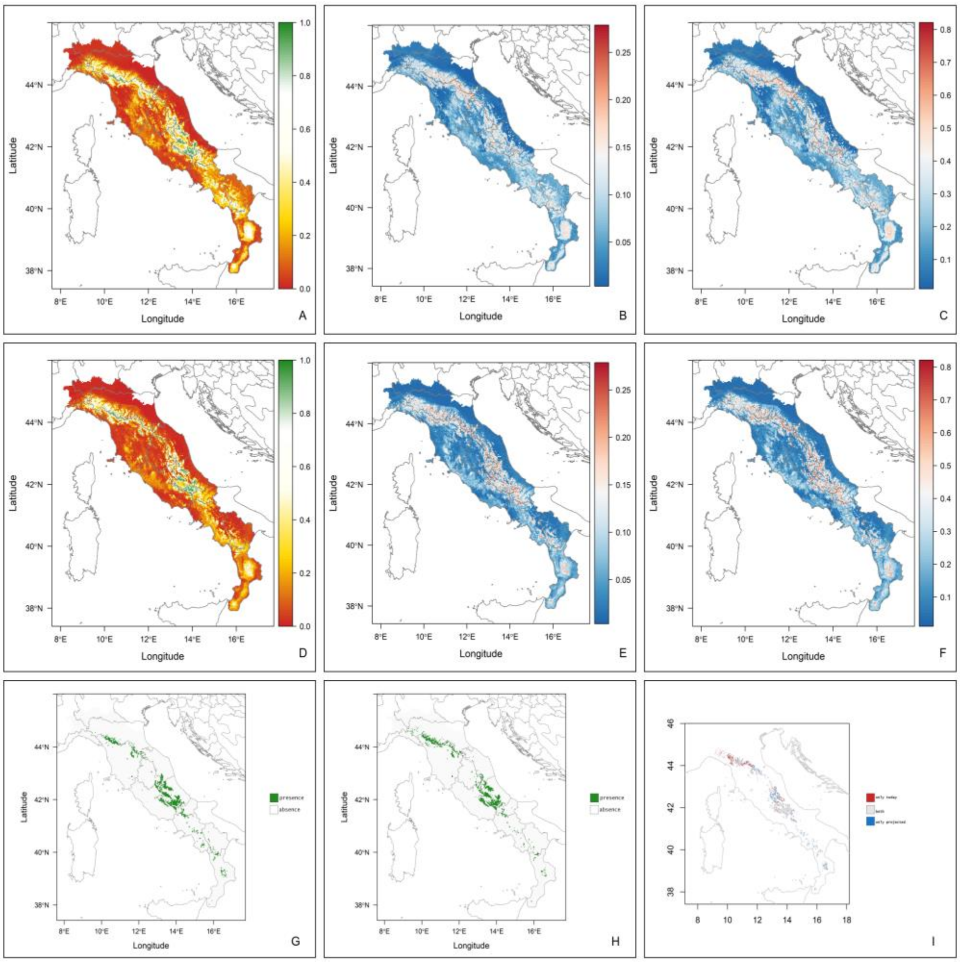

Fig. S.3.239-A) 'Projected' base output; B) 'Projected' uncertainty standard deviation; C) 'Projected' uncertainty range of values; D) 'Present' base output; E) 'Present' uncertainty standard deviation; F) 'Present' uncertainty range of values; G) 'Projected' binary distribution; H) 'Present' binary distribution; I) Range size variation.

Value	Present	Projected
AUC.train	0.977	1.000
AUC.test	0.977	0.988
AUC.diff	0.012	0.012
pROC	1.747	1.743
OR.p10	0.356	0.400
CBI	0.976	0.974
perc.stable	54.299	54.299
range.size	2.132	2.132

S.3.240 *Ranunculus pollinensis* SVM

Species: *Ranunculus pollinensis* (N.Terracc.) Chiov.

Procedure: SVM

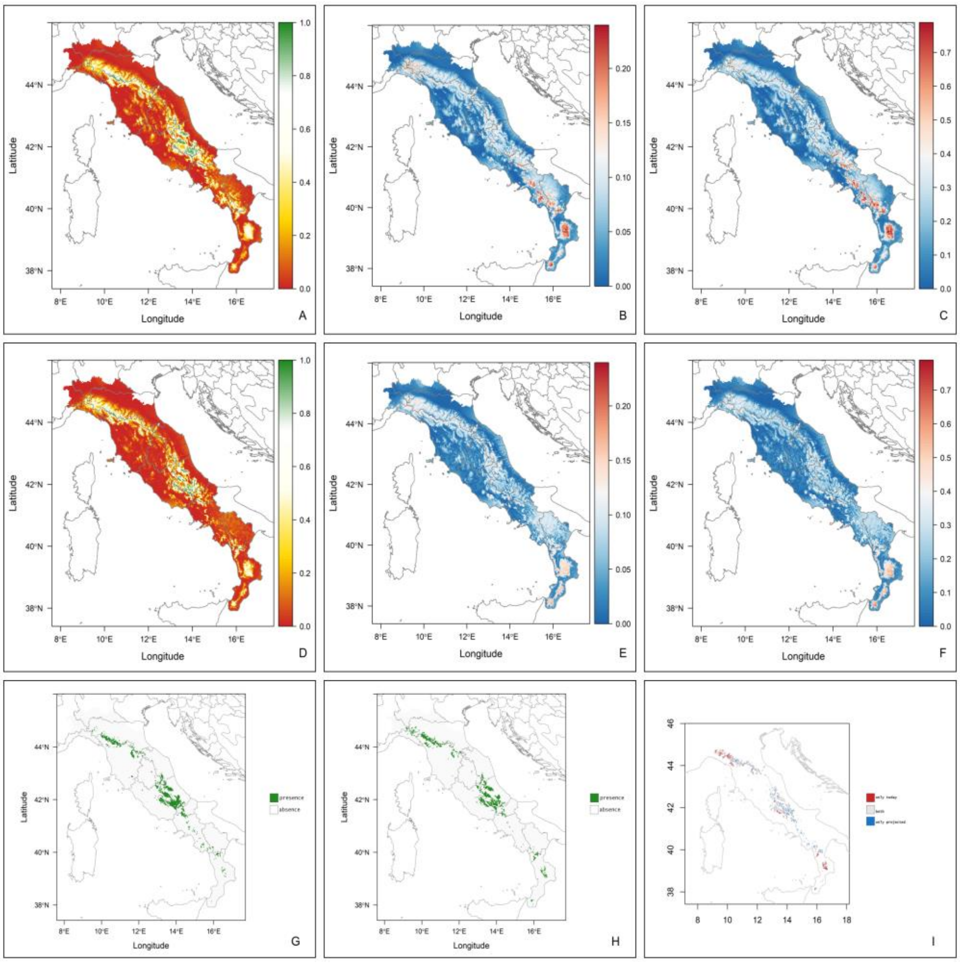

Fig. S.3.240-A) 'Projected' base output; B) 'Projected' uncertainty standard deviation; C) 'Projected' uncertainty range of values; D) 'Present' base output; E) 'Present' uncertainty standard deviation; F) 'Present' uncertainty range of values; G) 'Projected' binary distribution; H) 'Present' binary distribution; I) Range size variation.

Value	Present	Projected
AUC.train	0.948	0.967
AUC.test	0.948	0.951
AUC.diff	0.019	0.019
pROC	1.747	1.756
OR.p10	0.211	0.133
CBI	0.985	0.976
perc.stable	53.968	53.968
range.size	0.055	0.055

S.3.241 *Saxifraga porophylla* Biomod2

Species: *Saxifraga porophylla* Bertol. subsp. *porophylla*

Procedure: Biomod2

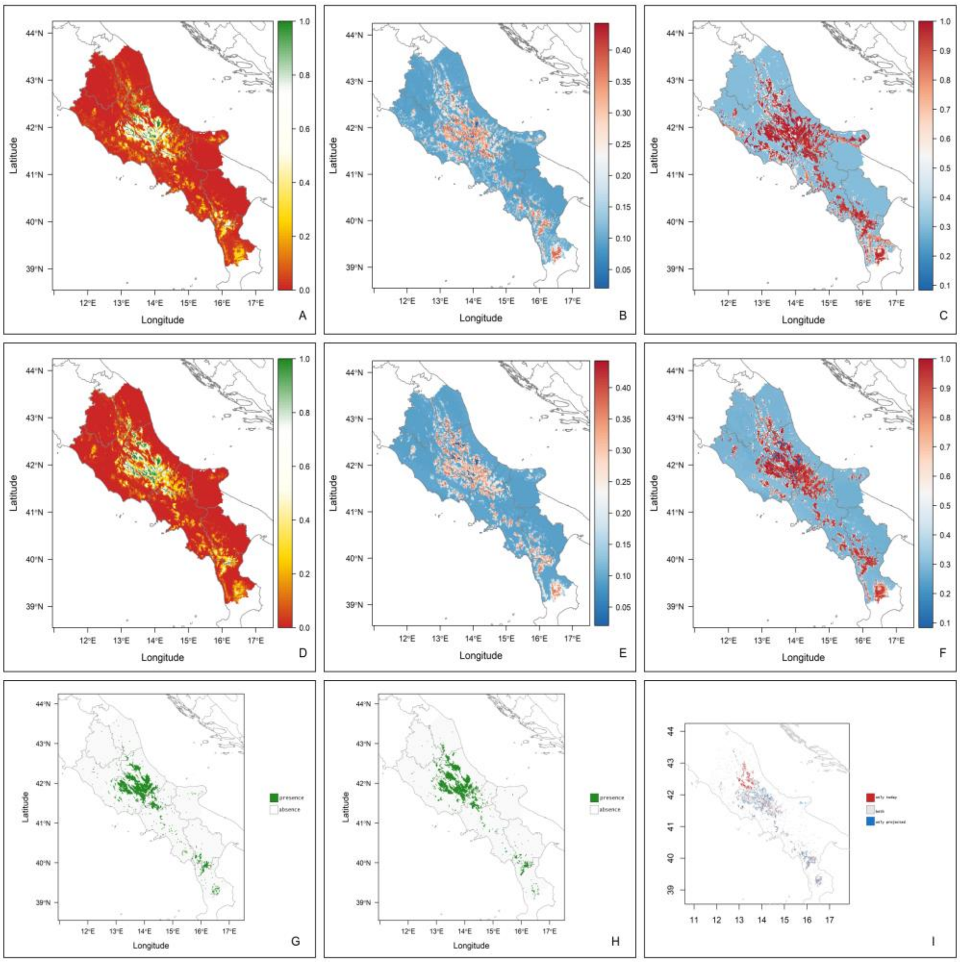

Fig. S.3.241-A) 'Projected' base output; B) 'Projected' uncertainty standard deviation; C) 'Projected' uncertainty range of values; D) 'Present' base output; E) 'Present' uncertainty standard deviation; F) 'Present' uncertainty range of values; G) 'Projected' binary distribution; H) 'Present' binary distribution; I) Range size variation.

Value	Present	Projected
AUC.train	0.958	0.991
AUC.test	0.958	0.973
AUC.diff	0.018	0.018
pROC	1.768	1.819
OR.p10	0.454	0.269
CBI	0.918	0.731
perc.stable	53.373	53.373
range.size	1.220	1.220

S.3.242 *Saxifraga porophylla* Bioclim

Species: *Saxifraga porophylla* Bertol. subsp. *porophylla*

Procedure: Bioclim

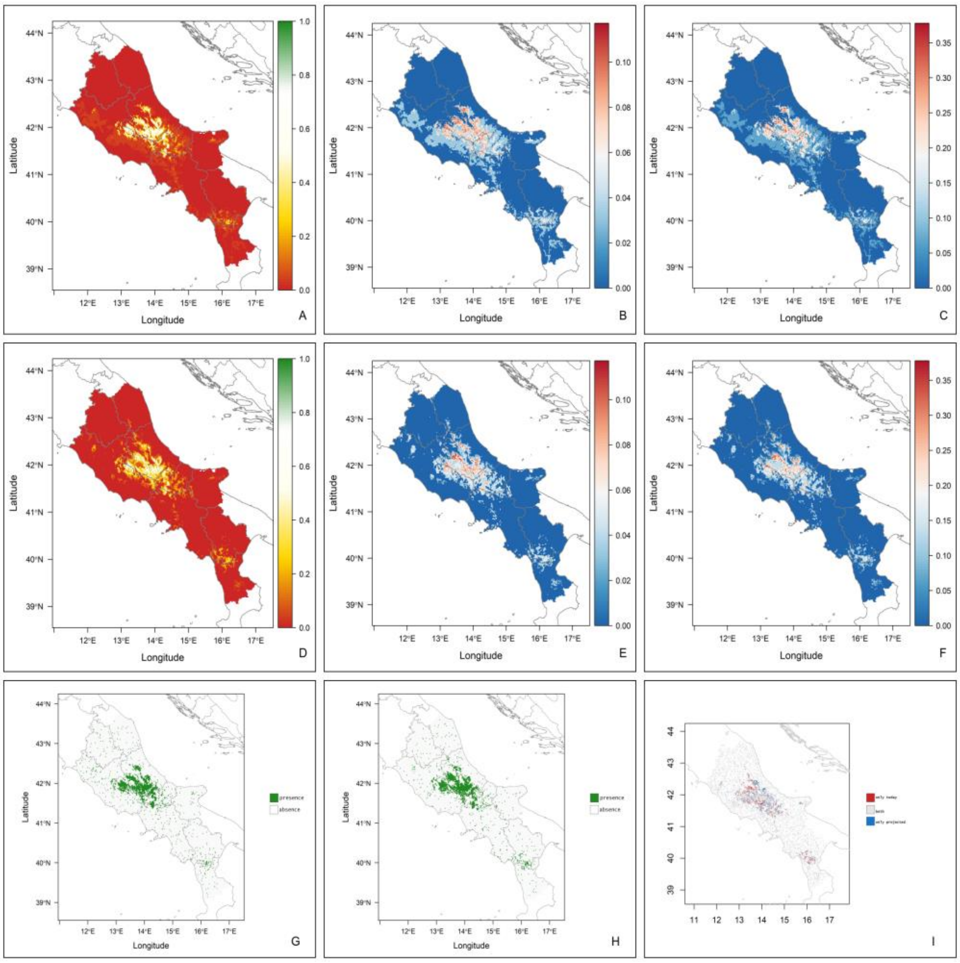

Fig. S.3.242-A) 'Projected' base output; B) 'Projected' uncertainty standard deviation; C) 'Projected' uncertainty range of values; D) 'Present' base output; E) 'Present' uncertainty standard deviation; F) 'Present' uncertainty range of values; G) 'Projected' binary distribution; H) 'Present' binary distribution; I) Range size variation.

Value	Present	Projected
AUC.train	0.813	0.932
AUC.test	0.813	0.856
AUC.diff	0.082	0.082
pROC	1.192	1.322
OR.p10	0.238	0.185
CBI	0.257	0.586
perc.stable	40.338	40.338
range.size	-2.498	-2.498

S.3.243 *Saxifraga porophylla* Domain

Species: *Saxifraga porophylla* Bertol. subsp. *porophylla*

Procedure: Domain

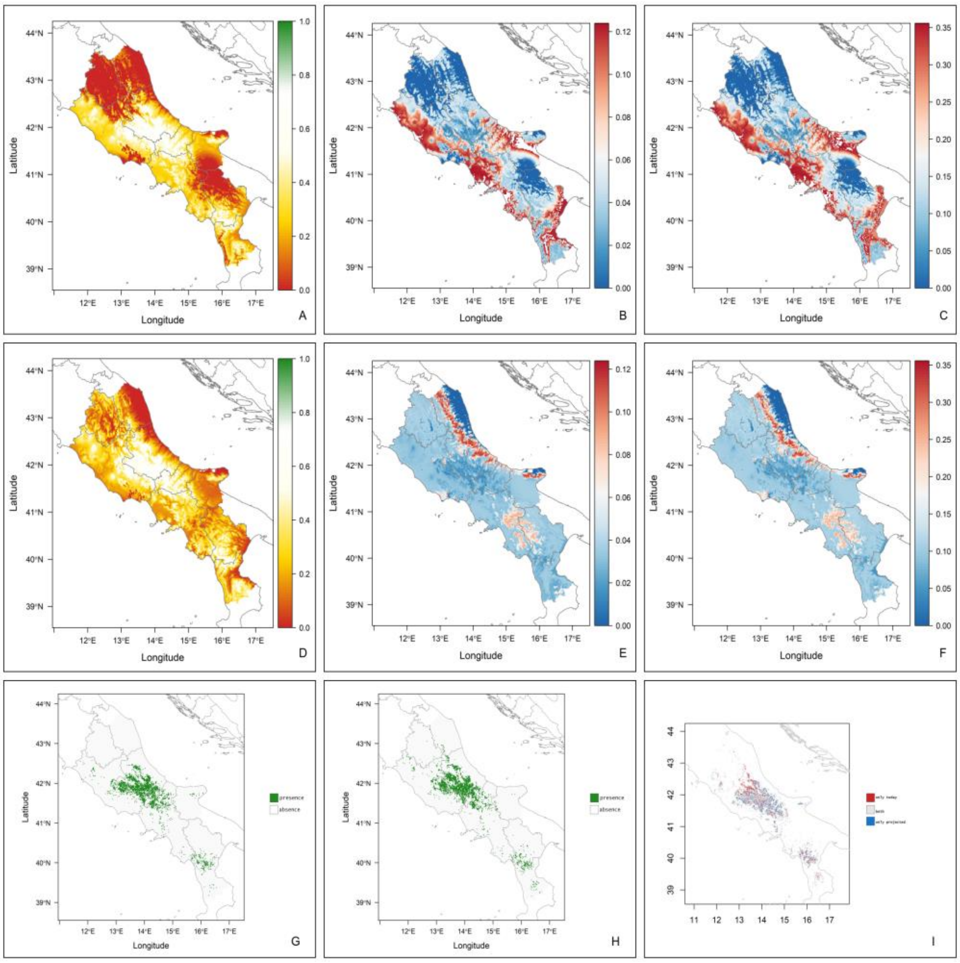

Fig. S.3.243-A) 'Projected' base output; B) 'Projected' uncertainty standard deviation; C) 'Projected' uncertainty range of values; D) 'Present' base output; E) 'Present' uncertainty standard deviation; F) 'Present' uncertainty range of values; G) 'Projected' binary distribution; H) 'Present' binary distribution; I) Range size variation.

Value	Present	Projected
AUC.train	0.907	0.936
AUC.test	0.907	0.928
AUC.diff	0.019	0.019
pROC	1.652	1.704
OR.p10	0.177	0.131
CBI	0.961	0.984
perc.stable	41.948	41.948
range.size	-1.347	-1.347

S.3.244 *Saxifraga porophylla* Maxent

Species: *Saxifraga porophylla* Bertol. subsp. *porophylla*

Procedure: Maxent

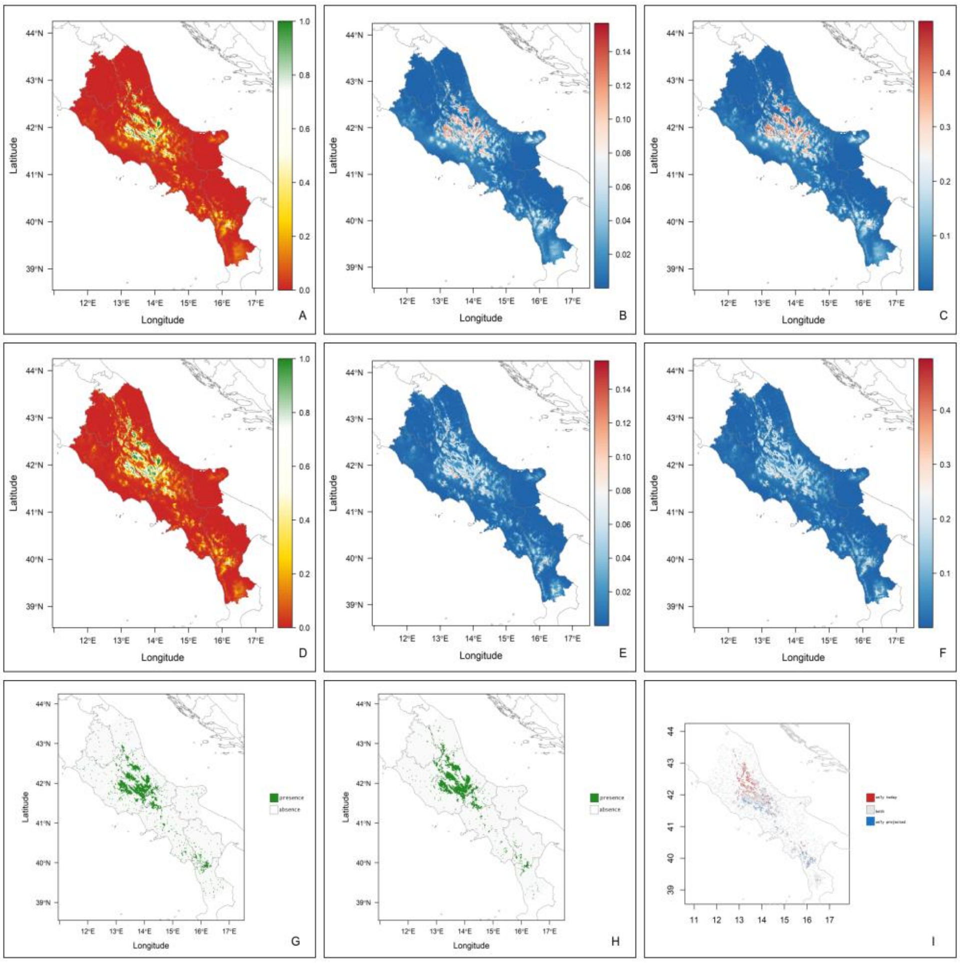

Fig. S.3.244-A) 'Projected' base output; B) 'Projected' uncertainty standard deviation; C) 'Projected' uncertainty range of values; D) 'Present' base output; E) 'Present' uncertainty standard deviation; F) 'Present' uncertainty range of values; G) 'Projected' binary distribution; H) 'Present' binary distribution; I) Range size variation.

Value	Present	Projected
AUC.train	0.953	0.956
AUC.test	0.953	0.953
AUC.diff	0.019	0.019
pROC	1.772	1.742
OR.p10	0.138	0.169
CBI	0.895	0.713
perc.stable	49.852	49.852
range.size	-0.019	-0.019

S.3.245 *Saxifraga porophylla* KDE

Species: *Saxifraga porophylla* Bertol. subsp. *porophylla*

Procedure: KDE

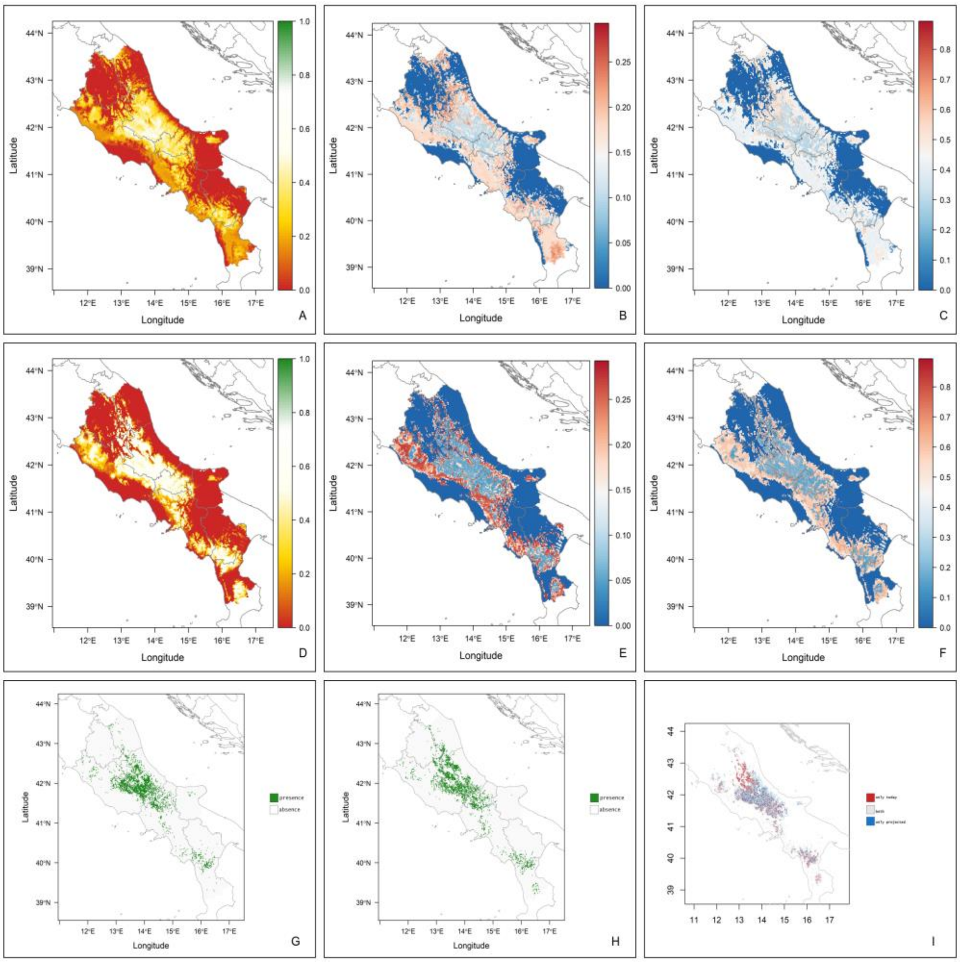

Fig. S.3.245-A) 'Projected' base output; B) 'Projected' uncertainty standard deviation; C) 'Projected' uncertainty range of values; D) 'Present' base output; E) 'Present' uncertainty standard deviation; F) 'Present' uncertainty range of values; G) 'Projected' binary distribution; H) 'Present' binary distribution; I) Range size variation.

Value	Present	Projected
AUC.train	0.923	0.937
AUC.test	0.923	0.904
AUC.diff	0.033	0.043
pROC	1.632	1.480
OR.p10	0.223	0.146
CBI	0.966	0.952
perc.stable	29.277	29.277
range.size	-0.240	-0.240

S.3.246 *Saxifraga porophylla* CTA

Species: *Saxifraga porophylla* Bertol. subsp. *porophylla*

Procedure: CTA

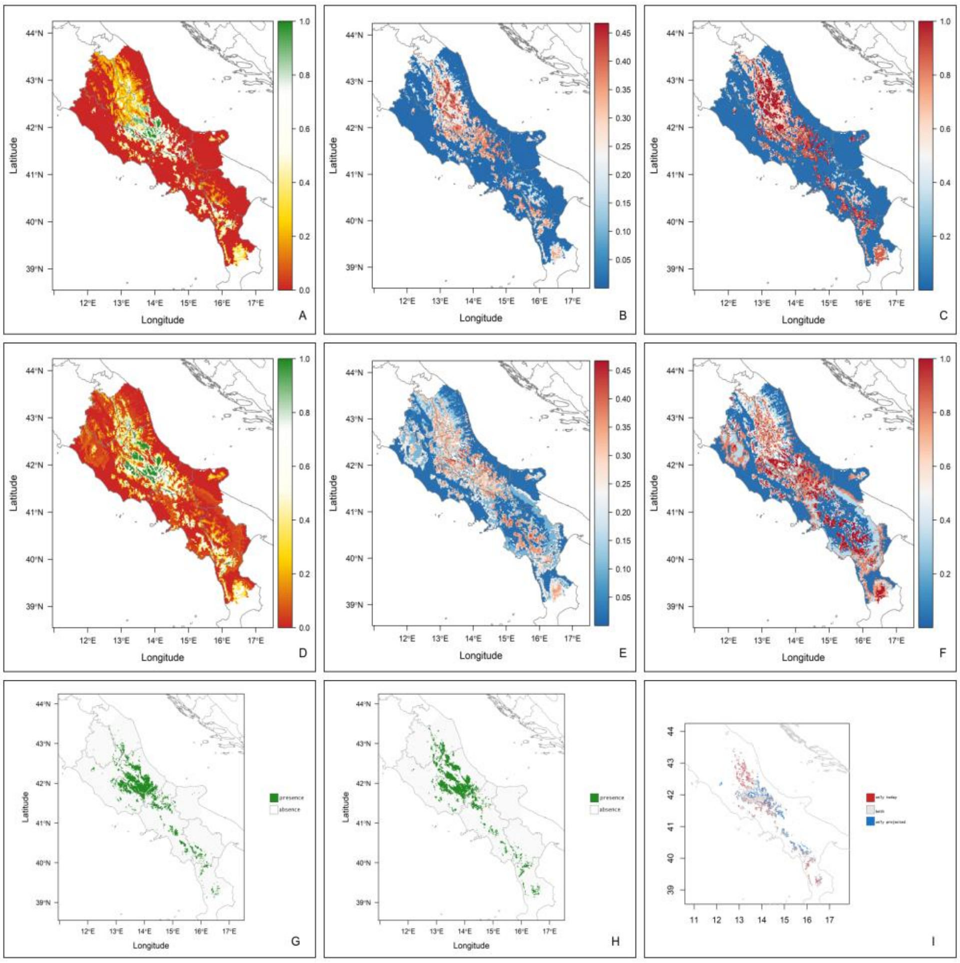

Fig. S.3.246-A) 'Projected' base output; B) 'Projected' uncertainty standard deviation; C) 'Projected' uncertainty range of values; D) 'Present' base output; E) 'Present' uncertainty standard deviation; F) 'Present' uncertainty range of values; G) 'Projected' binary distribution; H) 'Present' binary distribution; I) Range size variation.

Value	Present	Projected
AUC.train	0.930	0.969
AUC.test	0.930	0.931
AUC.diff	0.045	0.045
pROC	1.337	1.331
OR.p10	0.177	0.169
CBI	0.952	0.839
perc.stable	52.140	52.140
range.size	1.259	1.259

S.3.247 *Saxifraga porophylla* FDA

Species: *Saxifraga porophylla* Bertol. subsp. *porophylla*

Procedure: FDA

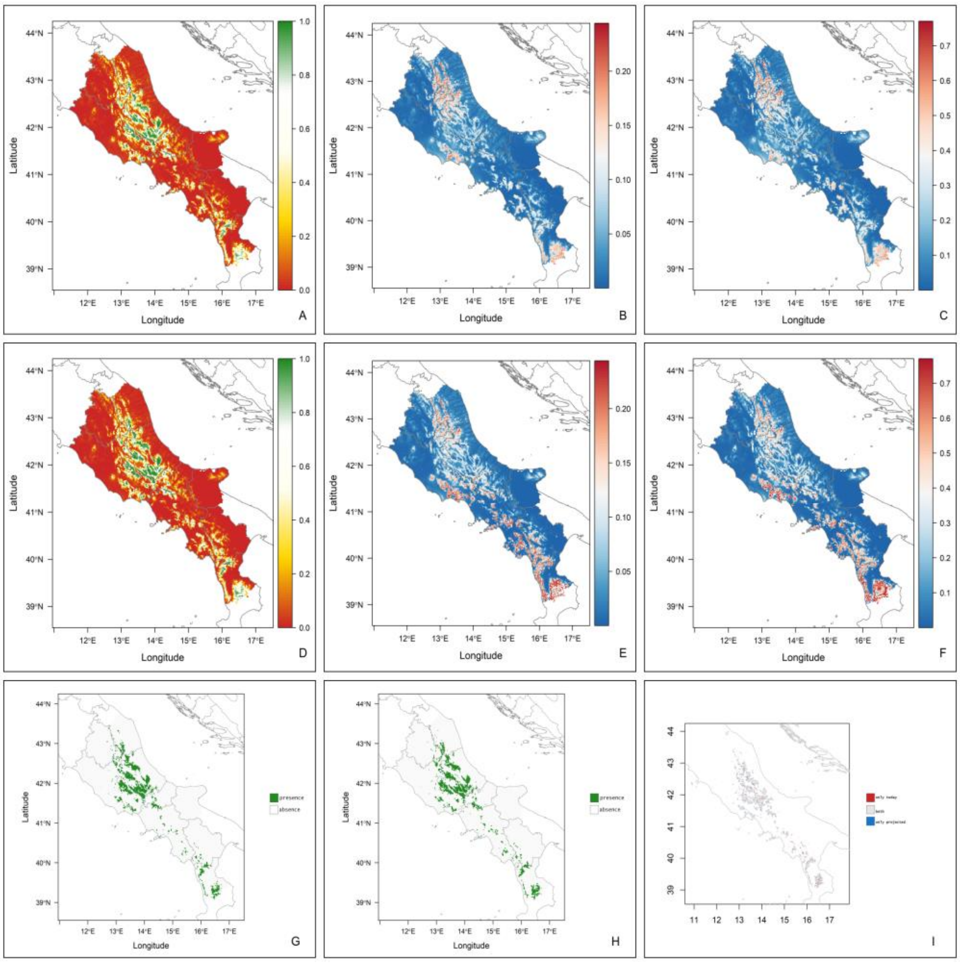

Fig. S.3.247-A) 'Projected' base output; B) 'Projected' uncertainty standard deviation; C) 'Projected' uncertainty range of values; D) 'Present' base output; E) 'Present' uncertainty standard deviation; F) 'Present' uncertainty range of values; G) 'Projected' binary distribution; H) 'Present' binary distribution; I) Range size variation.

Value	Present	Projected
AUC.train	0.948	0.942
AUC.test	0.948	0.931
AUC.diff	0.018	0.018
pROC	1.684	1.608
OR.p10	0.123	0.169
CBI	0.897	0.879
perc.stable	75.196	75.196
range.size	0.193	0.193

S.3.248 *Saxifraga porophylla* GAM

Species: *Saxifraga porophylla* Bertol. subsp. *porophylla*

Procedure: GAM

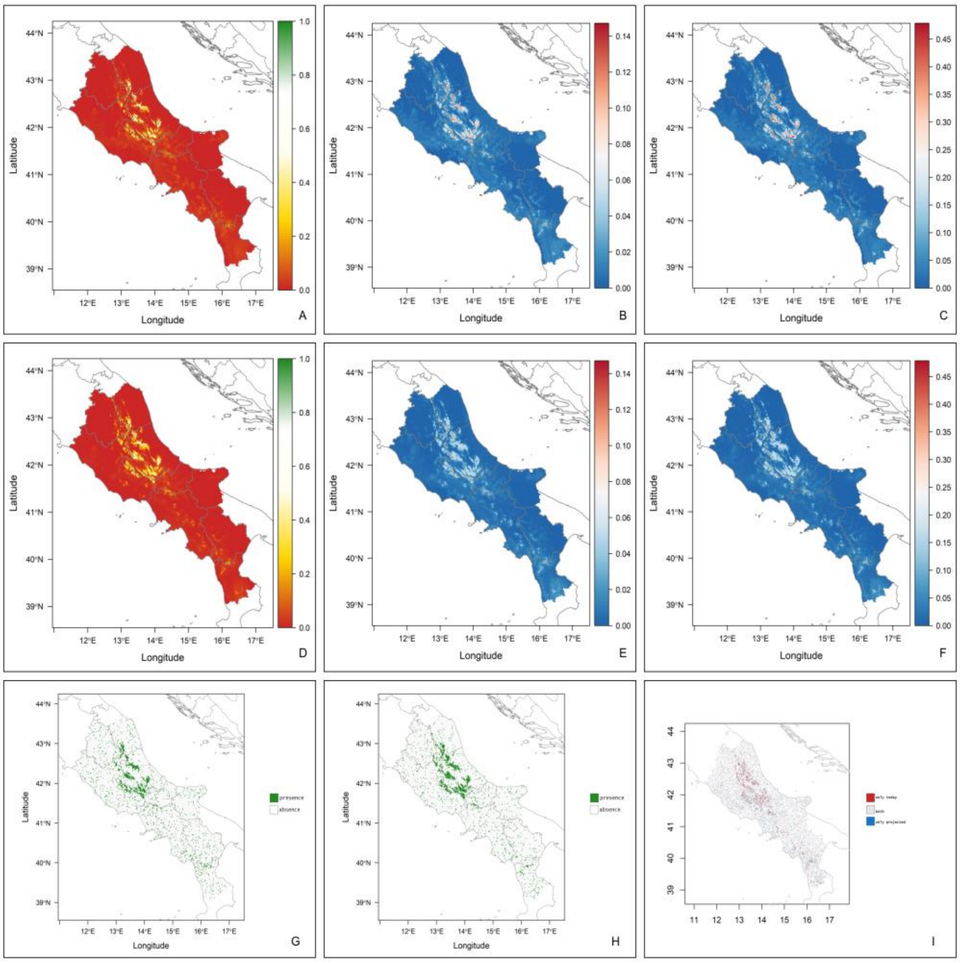

Fig. S.3.248-A) 'Projected' base output; B) 'Projected' uncertainty standard deviation; C) 'Projected' uncertainty range of values; D) 'Present' base output; E) 'Present' uncertainty standard deviation; F) 'Present' uncertainty range of values; G) 'Projected' binary distribution; H) 'Present' binary distribution; I) Range size variation.

Value	Present	Projected
AUC.train	0.937	0.946
AUC.test	0.937	0.899
AUC.diff	0.052	0.052
pROC	1.681	1.520
OR.p10	0.192	0.238
CBI	0.901	0.866
perc.stable	21.573	21.573
range.size	-0.436	-0.436

S.3.249 *Saxifraga porophylla* GLM

Species: *Saxifraga porophylla* Bertol. subsp. *porophylla*

Procedure: GLM

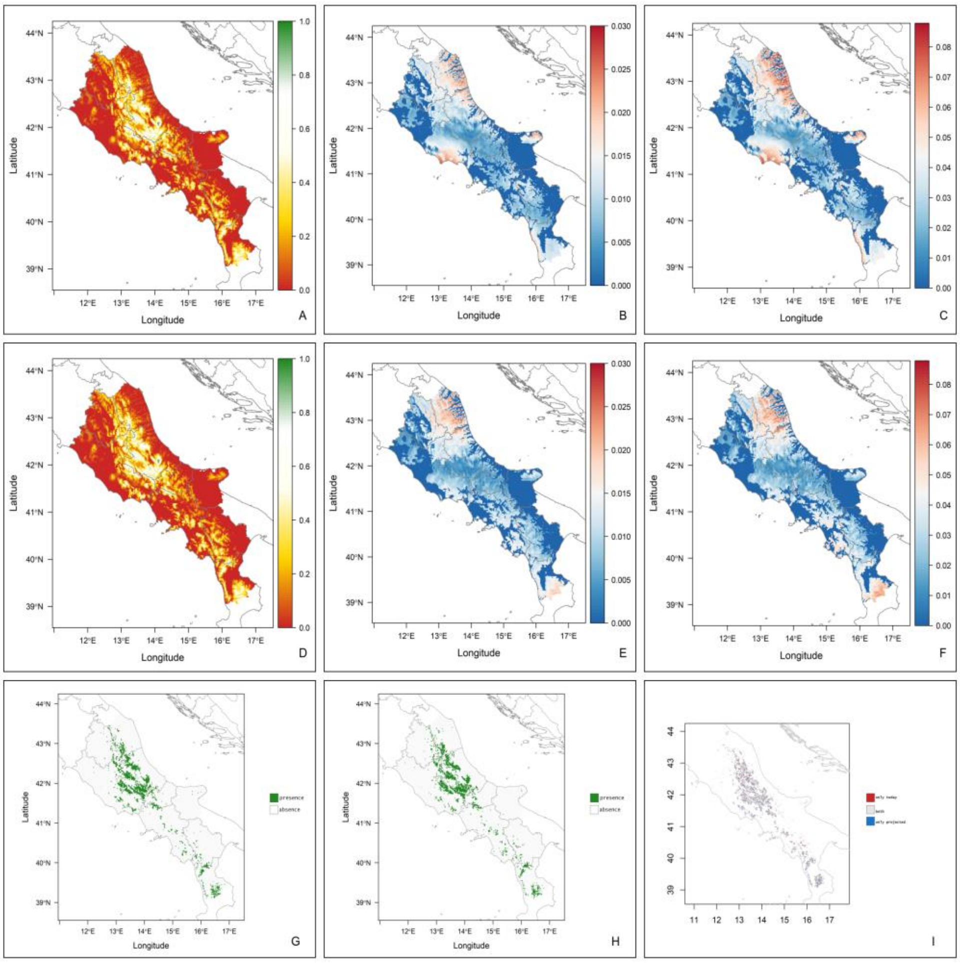

Fig. S.3.249-A) 'Projected' base output; B) 'Projected' uncertainty standard deviation; C) 'Projected' uncertainty range of values; D) 'Present' base output; E) 'Present' uncertainty standard deviation; F) 'Present' uncertainty range of values; G) 'Projected' binary distribution; H) 'Present' binary distribution; I) Range size variation.

Value	Present	Projected
AUC.train	0.938	0.932
AUC.test	0.938	0.932
AUC.diff	0.015	0.015
pROC	1.662	1.649
OR.p10	0.146	0.177
CBI	0.984	0.980
perc.stable	52.295	52.295
range.size	0.477	0.477

S.3.250 *Saxifraga porophylla* MARS

Species: *Saxifraga porophylla* Bertol. subsp. *porophylla*

Procedure: MARS

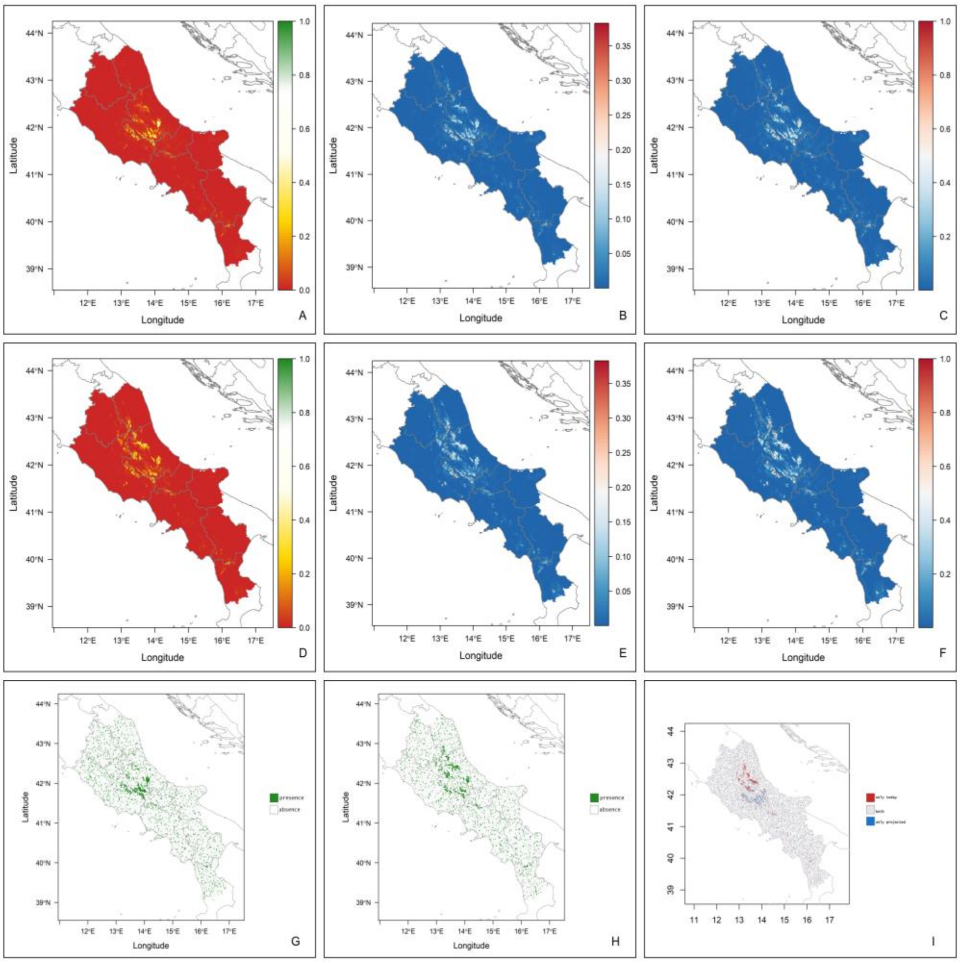

Fig. S.3.250-A) 'Projected' base output; B) 'Projected' uncertainty standard deviation; C) 'Projected' uncertainty range of values; D) 'Present' base output; E) 'Present' uncertainty standard deviation; F) 'Present' uncertainty range of values; G) 'Projected' binary distribution; H) 'Present' binary distribution; I) Range size variation.

Value	Present	Projected
AUC.train	0.865	0.945
AUC.test	0.865	0.903
AUC.diff	0.049	0.049
pROC	1.414	1.488
OR.p10	0.115	0.154
CBI	0.938	0.847
perc.stable	8.943	8.943
range.size	1.452	1.452

S.3.251 *Saxifraga porophylla* RF

Species: *Saxifraga porophylla* Bertol. subsp. *porophylla*

Procedure: RF

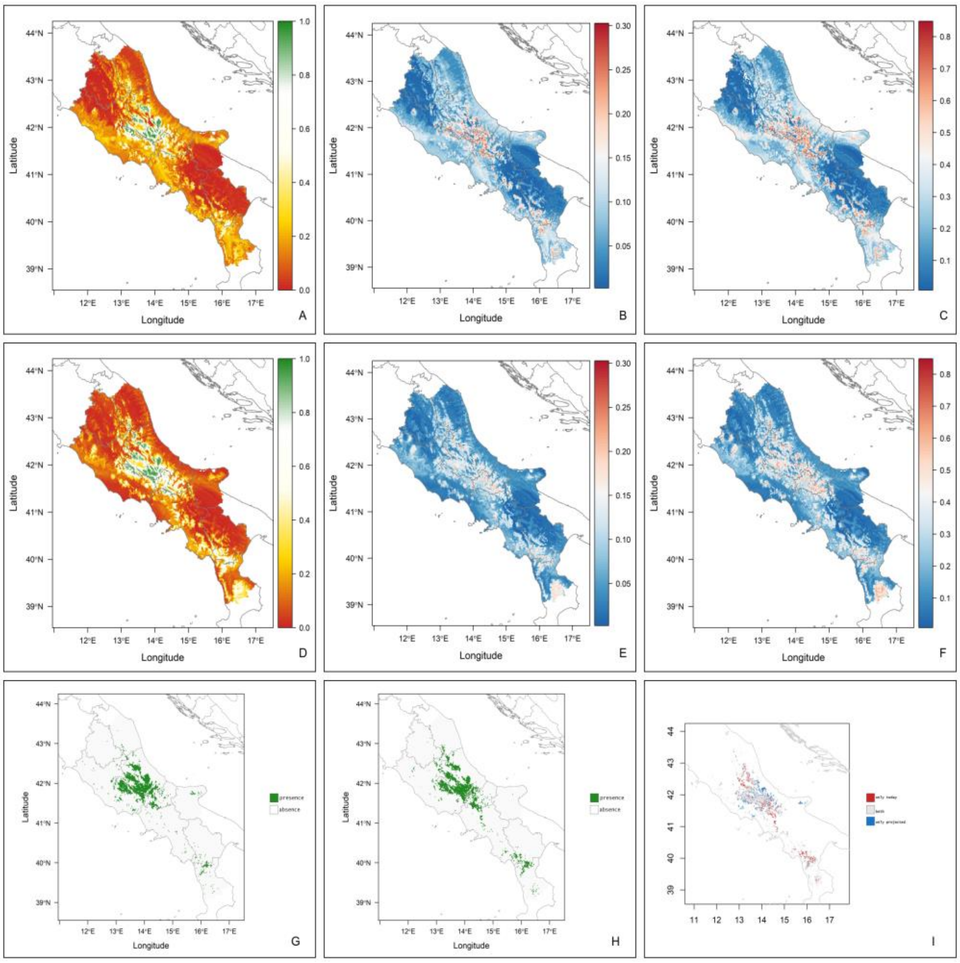

Fig. S.3.251-A) 'Projected' base output; B) 'Projected' uncertainty standard deviation; C) 'Projected' uncertainty range of values; D) 'Present' base output; E) 'Present' uncertainty standard deviation; F) 'Present' uncertainty range of values; G) 'Projected' binary distribution; H) 'Present' binary distribution; I) Range size variation.

Value	Present	Projected
AUC.train	0.991	1.000
AUC.test	0.991	0.981
AUC.diff	0.019	0.019
pROC	1.736	1.633
OR.p10	0.377	0.346
CBI	0.966	0.949
perc.stable	54.143	54.143
range.size	-1.334	-1.334

S.3.252 *Saxifraga porophylla* SVM

Species: *Saxifraga porophylla* Bertol. subsp. *porophylla*

Procedure: SVM

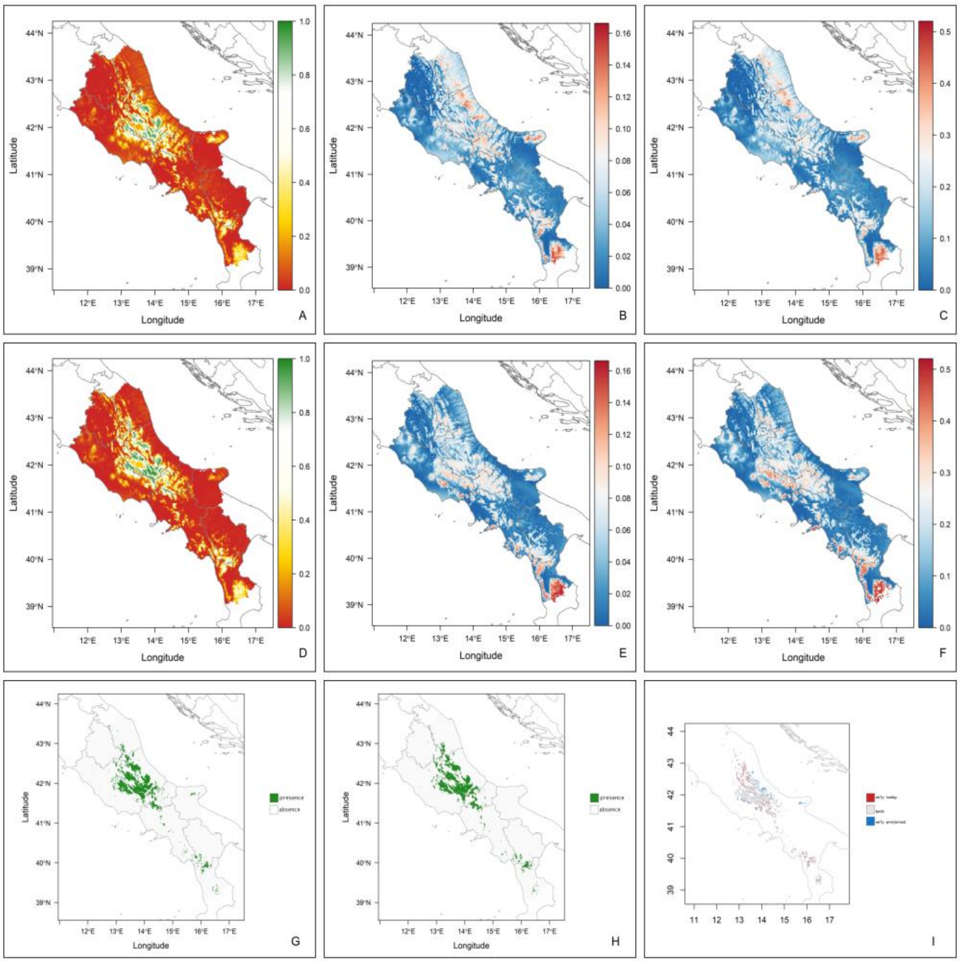

Fig. S.3.252-A) 'Projected' base output; B) 'Projected' uncertainty standard deviation; C) 'Projected' uncertainty range of values; D) 'Present' base output; E) 'Present' uncertainty standard deviation; F) 'Present' uncertainty range of values; G) 'Projected' binary distribution; H) 'Present' binary distribution; I) Range size variation.

Value	Present	Projected
AUC.train	0.950	0.969
AUC.test	0.950	0.937
AUC.diff	0.036	0.036
pROC	1.727	1.573
OR.p10	0.169	0.138
CBI	0.920	0.968
perc.stable	68.835	68.835
range.size	0.576	0.576

S.3.253 *Sedum magellense* Biomod2

Species: *Sedum magellense* Ten. subsp. *magellense*

Procedure: Biomod2

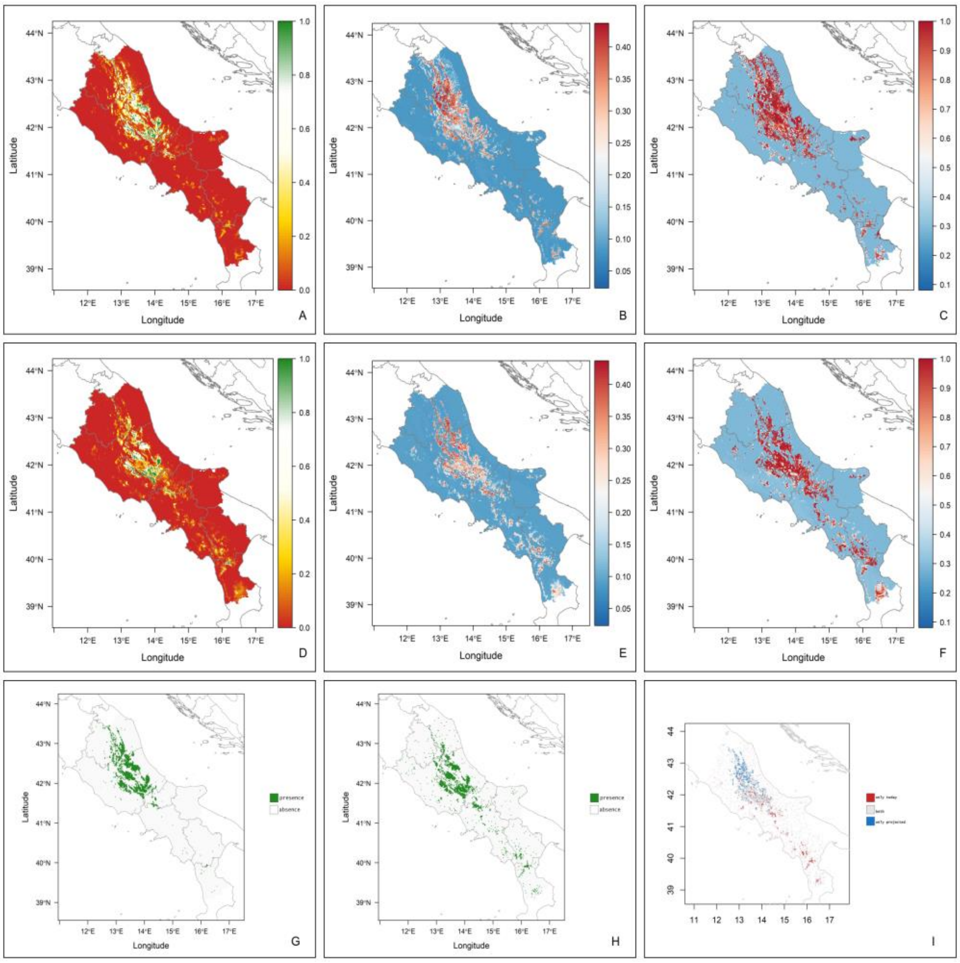

Fig. S.3.253-A) 'Projected' base output; B) 'Projected' uncertainty standard deviation; C) 'Projected' uncertainty range of values; D) 'Present' base output; E) 'Present' uncertainty standard deviation; F) 'Present' uncertainty range of values; G) 'Projected' binary distribution; H) 'Present' binary distribution; I) Range size variation.

Value	Present	Projected
AUC.train	0.971	0.993
AUC.test	0.971	0.940
AUC.diff	0.053	0.053
pROC	1.814	1.709
OR.p10	0.392	0.567
CBI	0.936	0.863
perc.stable	47.207	47.207
range.size	-1.574	-1.574

S.3.254 *Sedum magellense* Bioclim

Species: *Sedum magellense* Ten. subsp. *magellense*

Procedure: Bioclim

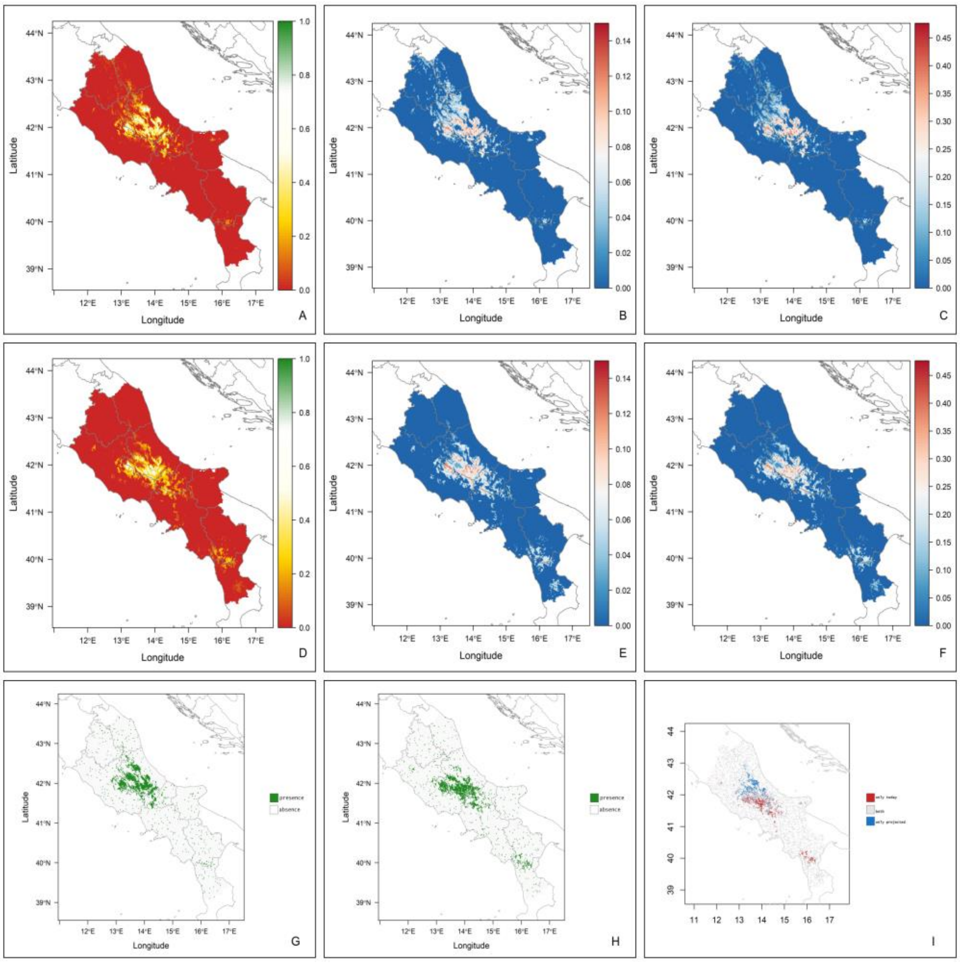

Fig. S.3.254-A) 'Projected' base output; B) 'Projected' uncertainty standard deviation; C) 'Projected' uncertainty range of values; D) 'Present' base output; E) 'Present' uncertainty standard deviation; F) 'Present' uncertainty range of values; G) 'Projected' binary distribution; H) 'Present' binary distribution; I) Range size variation.

Value	Present	Projected
AUC.train	0.873	0.940
AUC.test	0.873	0.880
AUC.diff	0.061	0.061
pROC	1.359	1.397
OR.p10	0.150	0.133
CBI	0.460	0.724
perc.stable	28.318	28.318
range.size	-0.696	-0.696

S.3.255 *Sedum magellense* Domain

Species: *Sedum magellense* Ten. subsp. *magellense*

Procedure: Domain

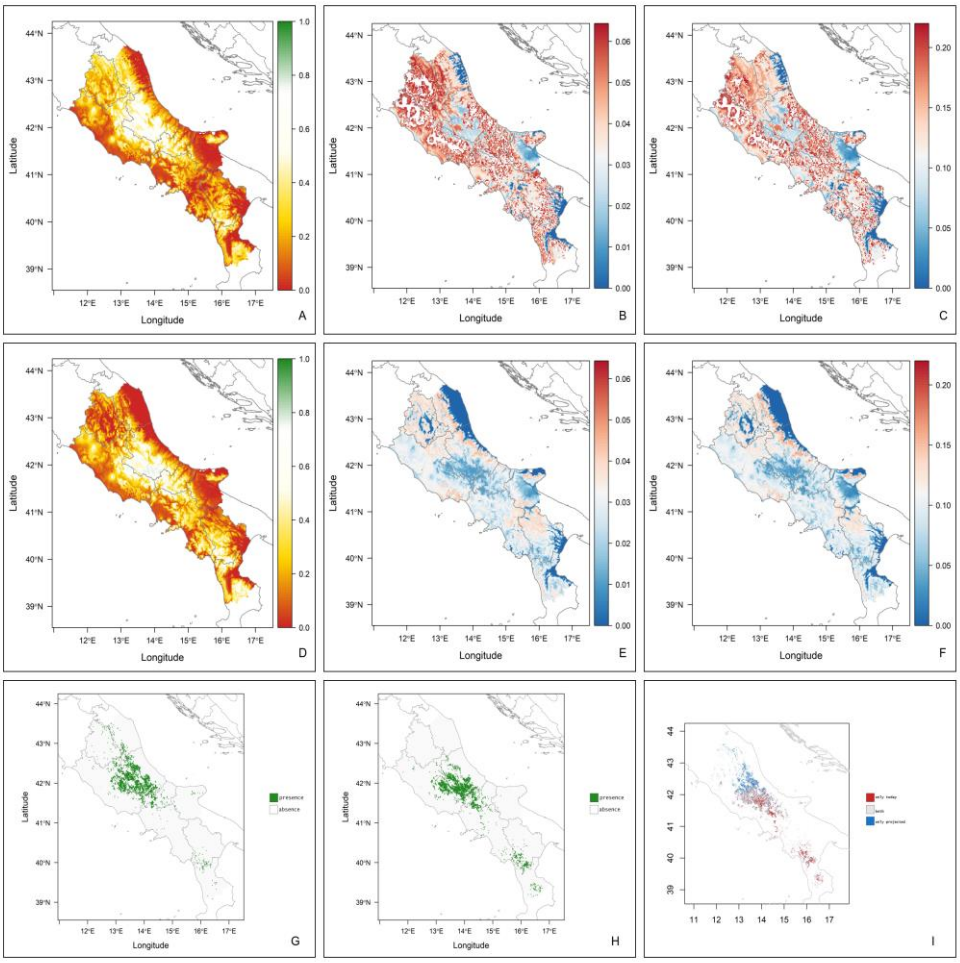

Fig. S.3.255-A) 'Projected' base output; B) 'Projected' uncertainty standard deviation; C) 'Projected' uncertainty range of values; D) 'Present' base output; E) 'Present' uncertainty standard deviation; F) 'Present' uncertainty range of values; G) 'Projected' binary distribution; H) 'Present' binary distribution; I) Range size variation.

Value	Present	Projected
AUC.train	0.951	0.934
AUC.test	0.951	0.919
AUC.diff	0.017	0.017
pROC	1.816	1.718
OR.p10	0.108	0.242
CBI	0.993	0.975
perc.stable	35.568	35.568
range.size	1.247	1.247

S.3.256 *Sedum magellense* Maxent

Species: *Sedum magellense* Ten. subsp. *magellense*

Procedure: Maxent

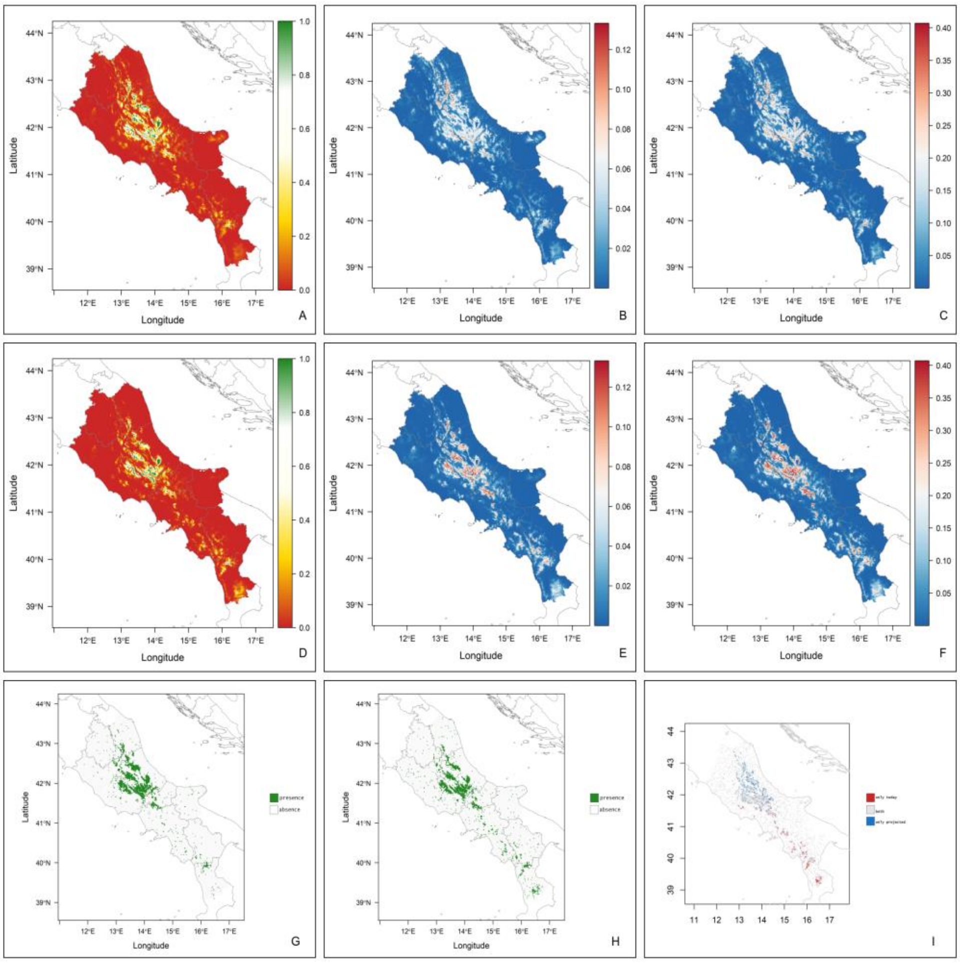

Fig. S.3.256-A) 'Projected' base output; B) 'Projected' uncertainty standard deviation; C) 'Projected' uncertainty range of values; D) 'Present' base output; E) 'Present' uncertainty standard deviation; F) 'Present' uncertainty range of values; G) 'Projected' binary distribution; H) 'Present' binary distribution; I) Range size variation.

Value	Present	Projected
AUC.train	0.961	0.963
AUC.test	0.961	0.947
AUC.diff	0.023	0.023
pROC	1.801	1.719
OR.p10	0.175	0.167
CBI	0.863	0.748
perc.stable	48.013	48.013
range.size	1.328	1.328

S.3.257 *Sedum magellense* KDE

Species: *Sedum magellense* Ten. subsp. *magellense*

Procedure: KDE

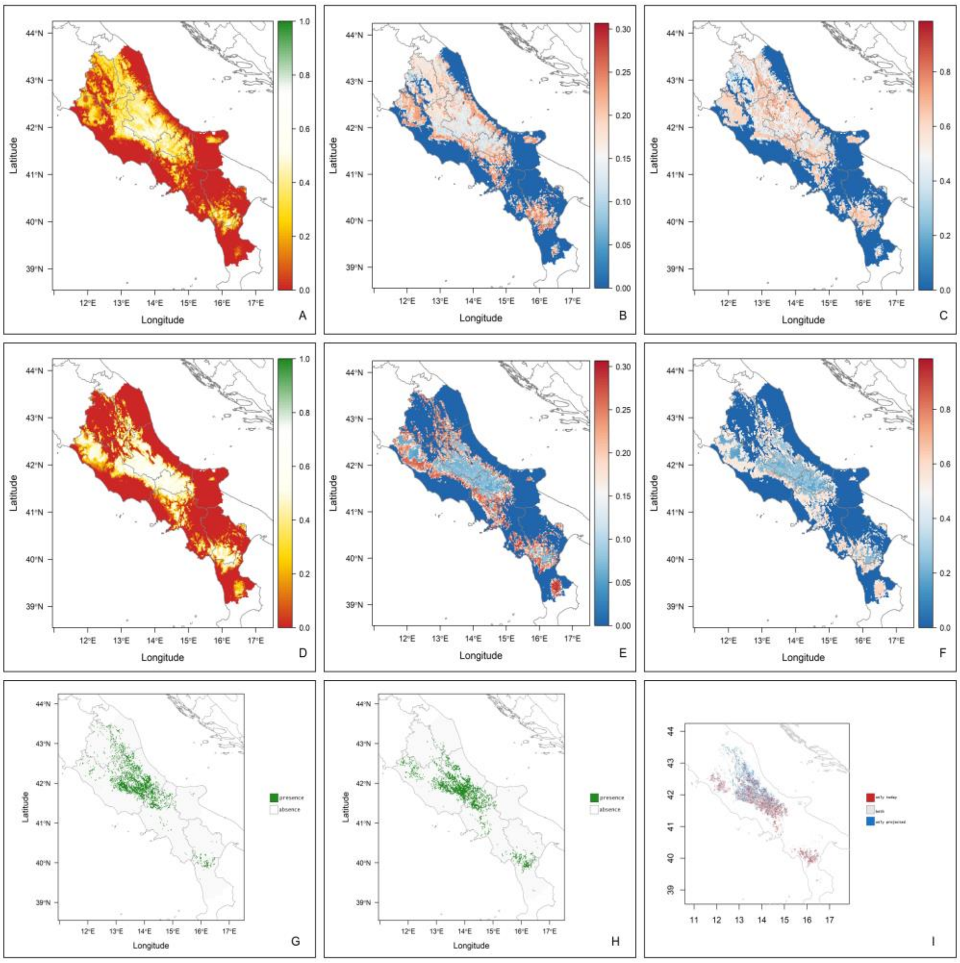

Fig. S.3.257-A) 'Projected' base output; B) 'Projected' uncertainty standard deviation; C) 'Projected' uncertainty range of values; D) 'Present' base output; E) 'Present' uncertainty standard deviation; F) 'Present' uncertainty range of values; G) 'Projected' binary distribution; H) 'Present' binary distribution; I) Range size variation.

Value	Present	Projected
AUC.train	0.948	0.945
AUC.test	0.948	0.925
AUC.diff	0.016	0.024
pROC	1.751	1.645
OR.p10	0.167	0.158
CBI	0.833	0.883
perc.stable	28.726	28.726
range.size	-0.762	-0.762

S.3.258 *Sedum magellense* CTA

Species: *Sedum magellense* Ten. subsp. *magellense*

Procedure: CTA

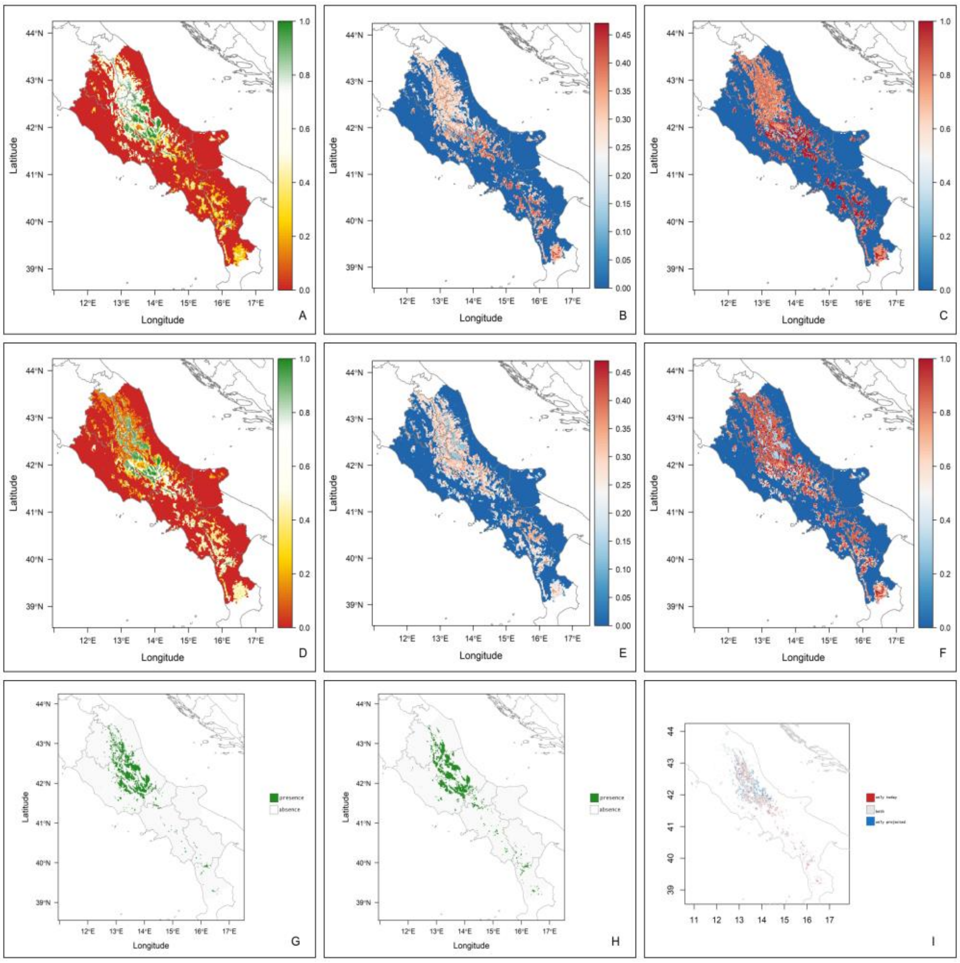

Fig. S.3.258-A) 'Projected' base output; B) 'Projected' uncertainty standard deviation; C) 'Projected' uncertainty range of values; D) 'Present' base output; E) 'Present' uncertainty standard deviation; F) 'Present' uncertainty range of values; G) 'Projected' binary distribution; H) 'Present' binary distribution; I) Range size variation.

Value	Present	Projected
AUC.train	0.932	0.979
AUC.test	0.932	0.946
AUC.diff	0.034	0.034
pROC	1.320	1.349
OR.p10	0.200	0.125
CBI	0.861	0.727
perc.stable	64.328	64.328
range.size	-0.711	-0.711

S.3.259 *Sedum magellense* FDA

Species: *Sedum magellense* Ten. subsp. *magellense*

Procedure: FDA

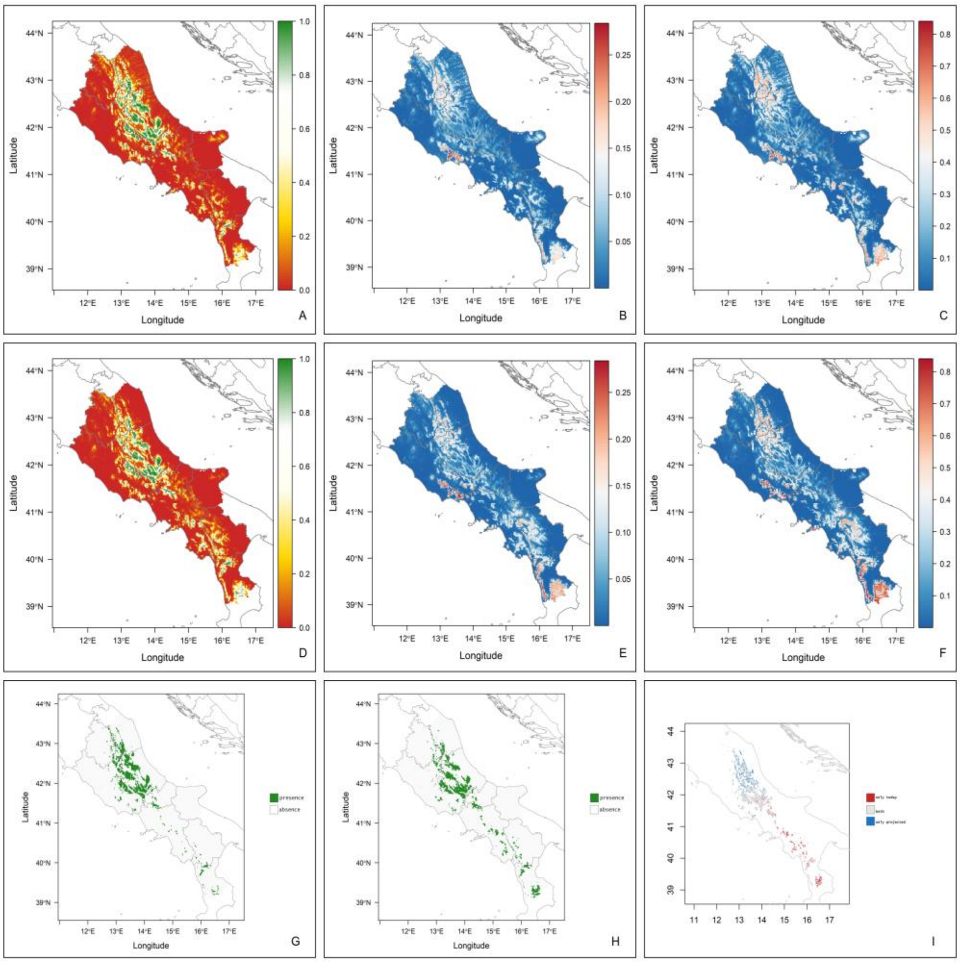

Fig. S.3.259-A) 'Projected' base output; B) 'Projected' uncertainty standard deviation; C) 'Projected' uncertainty range of values; D) 'Present' base output; E) 'Present' uncertainty standard deviation; F) 'Present' uncertainty range of values; G) 'Projected' binary distribution; H) 'Present' binary distribution; I) Range size variation.

Value	Present	Projected
AUC.train	0.967	0.961
AUC.test	0.967	0.955
AUC.diff	0.009	0.009
pROC	1.798	1.730
OR.p10	0.100	0.150
CBI	0.897	0.891
perc.stable	61.850	61.850
range.size	1.623	1.623

S.3.260 *Sedum magellense* GAM

Species: *Sedum magellense* Ten. subsp. *magellense*

Procedure: GAM

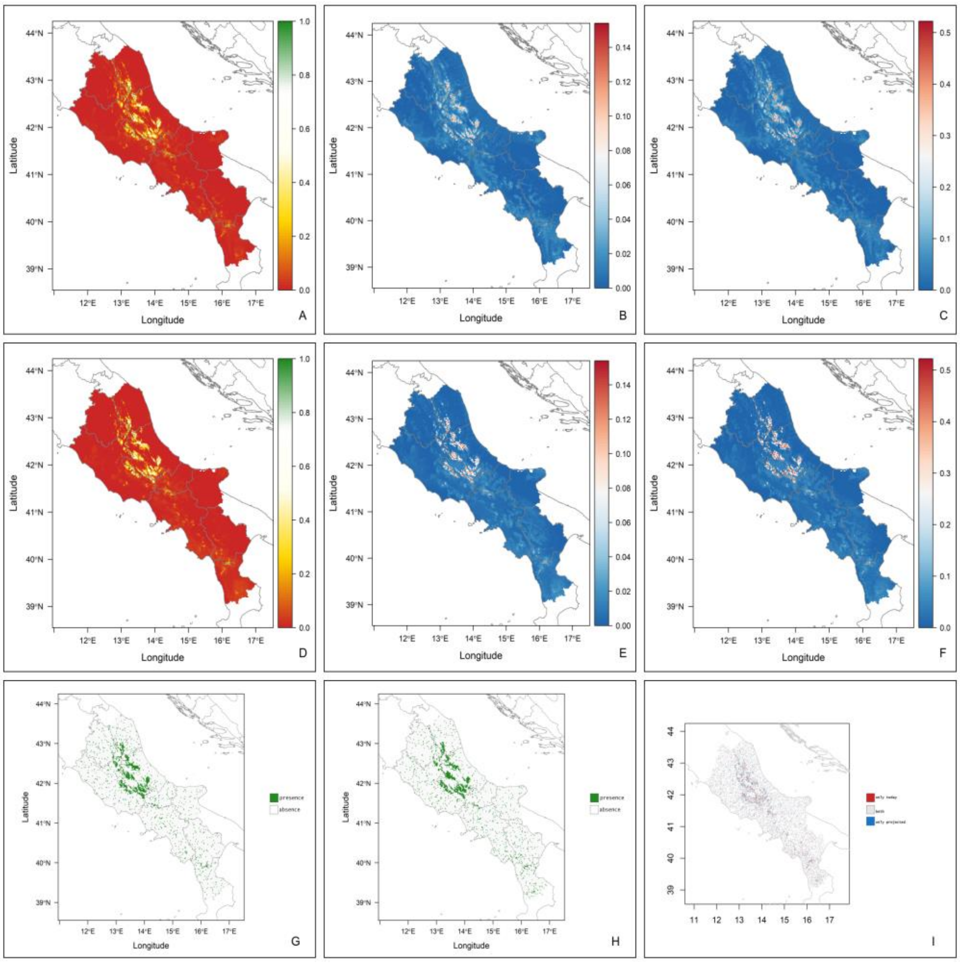

Fig. S.3.260-A) 'Projected' base output; B) 'Projected' uncertainty standard deviation; C) 'Projected' uncertainty range of values; D) 'Present' base output; E) 'Present' uncertainty standard deviation; F) 'Present' uncertainty range of values; G) 'Projected' binary distribution; H) 'Present' binary distribution; I) Range size variation.

Value	Present	Projected
AUC.train	0.950	0.948
AUC.test	0.950	0.936
AUC.diff	0.024	0.024
pROC	1.740	1.707
OR.p10	0.142	0.125
CBI	0.972	0.970
perc.stable	27.118	27.118
range.size	2.874	2.874

S.3.261 *Sedum magellense* GLM

Species: *Sedum magellense* Ten. subsp. *magellense*

Procedure: GLM

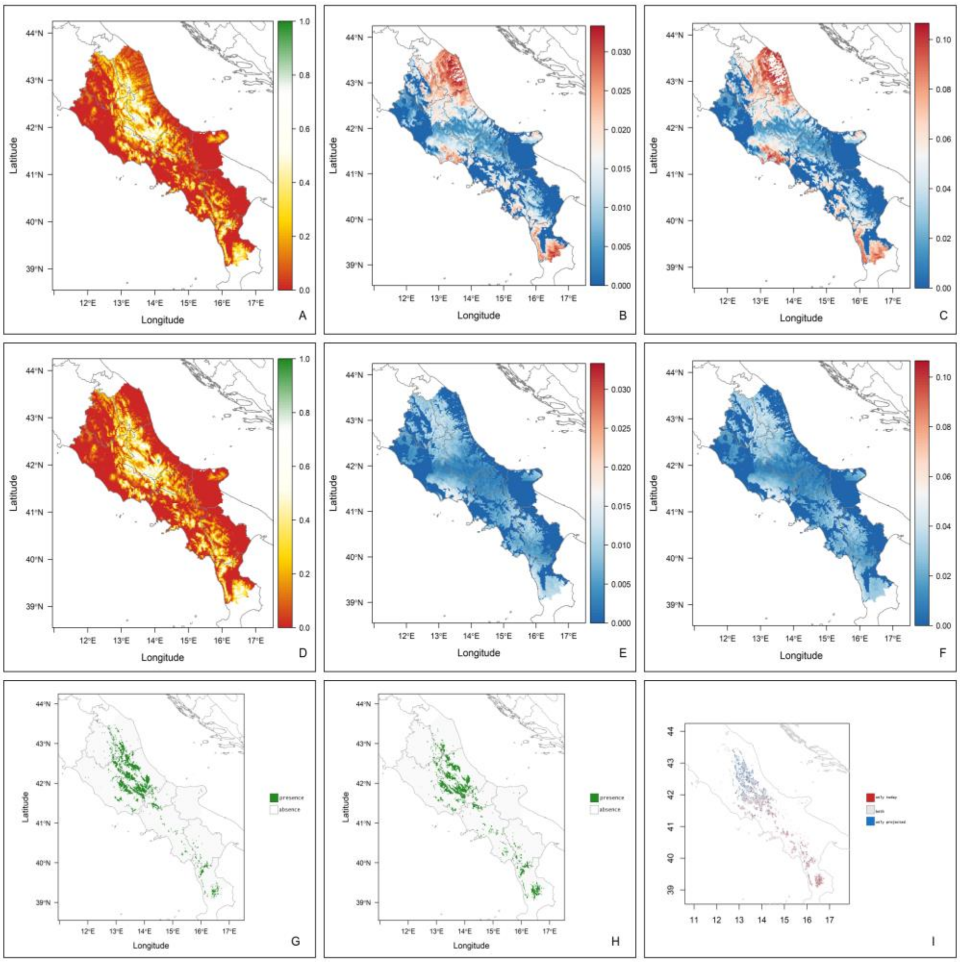

Fig. S.3.261-A) 'Projected' base output; B) 'Projected' uncertainty standard deviation; C) 'Projected' uncertainty range of values; D) 'Present' base output; E) 'Present' uncertainty standard deviation; F) 'Present' uncertainty range of values; G) 'Projected' binary distribution; H) 'Present' binary distribution; I) Range size variation.

Value	Present	Projected
AUC.train	0.960	0.956
AUC.test	0.960	0.947
AUC.diff	0.015	0.015
pROC	1.777	1.735
OR.p10	0.125	0.167
CBI	0.973	0.973
perc.stable	48.680	48.680
range.size	2.368	2.368

S.3.262 *Sedum magellense* MARS

Species: *Sedum magellense* Ten. subsp. *magellense*

Procedure: MARS

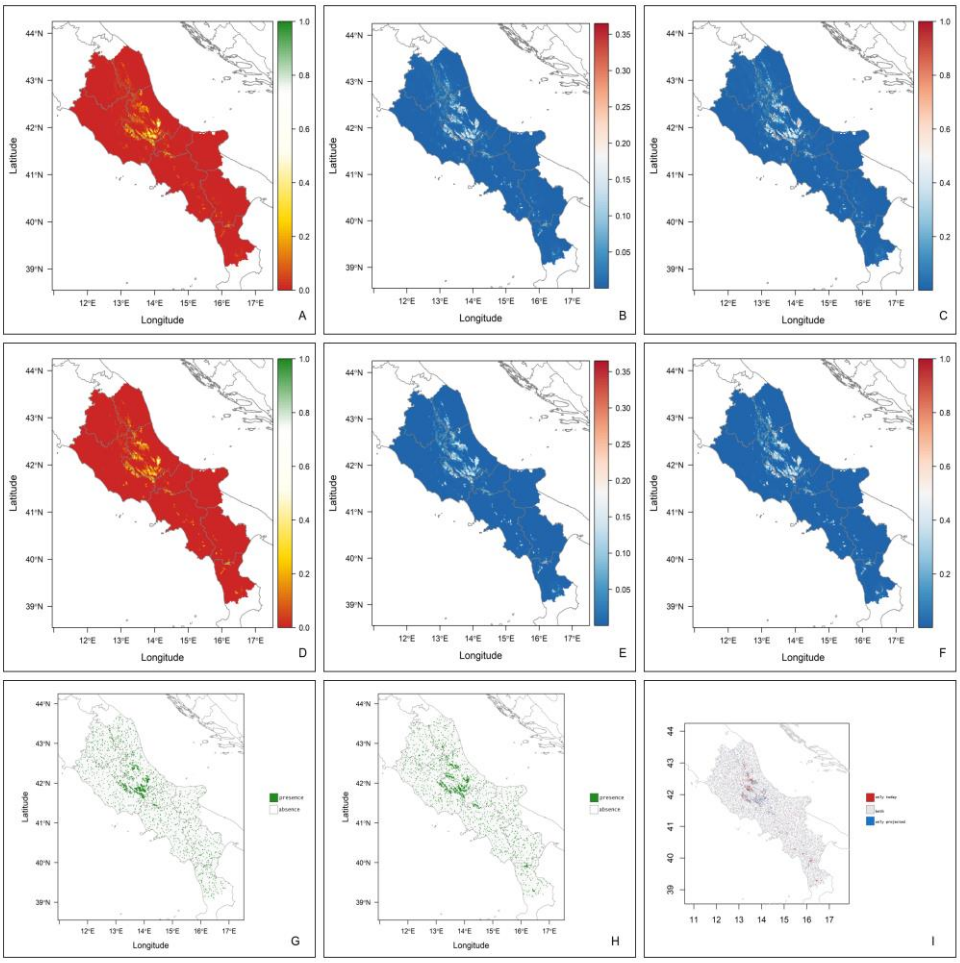

Fig. S.3.262-A) 'Projected' base output; B) 'Projected' uncertainty standard deviation; C) 'Projected' uncertainty range of values; D) 'Present' base output; E) 'Present' uncertainty standard deviation; F) 'Present' uncertainty range of values; G) 'Projected' binary distribution; H) 'Present' binary distribution; I) Range size variation.

Value	Present	Projected
AUC.train	0.917	0.961
AUC.test	0.917	0.923
AUC.diff	0.041	0.041
pROC	1.463	1.626
OR.p10	0.225	0.158
CBI	0.922	0.783
perc.stable	11.345	11.345
range.size	1.480	1.480

S.3.263 *Sedum magellense* RF

Species: *Sedum magellense* Ten. subsp. *magellense*

Procedure: RF

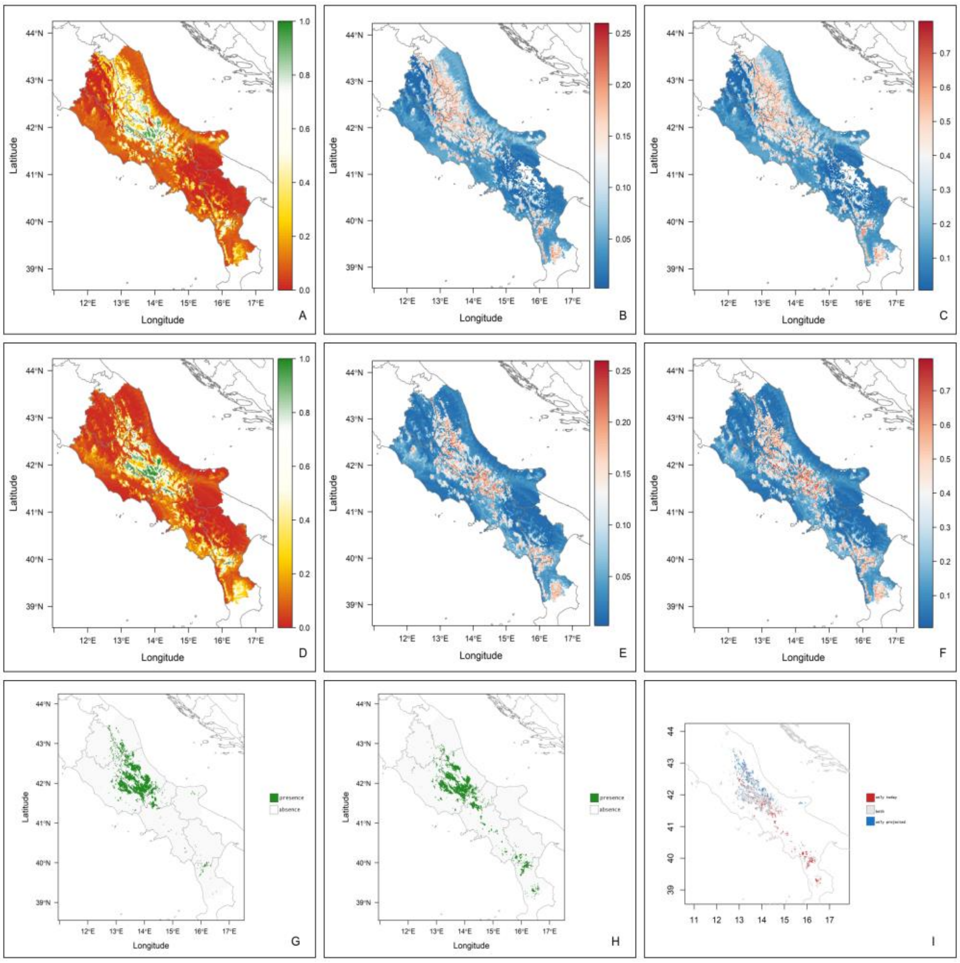

Fig. S.3.263-A) 'Projected' base output; B) 'Projected' uncertainty standard deviation; C) 'Projected' uncertainty range of values; D) 'Present' base output; E) 'Present' uncertainty standard deviation; F) 'Present' uncertainty range of values; G) 'Projected' binary distribution; H) 'Present' binary distribution; I) Range size variation.

Value	Present	Projected
AUC.train	0.990	1.000
AUC.test	0.990	0.991
AUC.diff	0.009	0.009
pROC	1.763	1.759
OR.p10	0.392	0.350
CBI	0.903	0.958
perc.stable	50.359	50.359
range.size	1.310	1.310

S.3.264 *Sedum magellense* SVM

Species: *Sedum magellense* Ten. subsp. *magellense*

Procedure: SVM

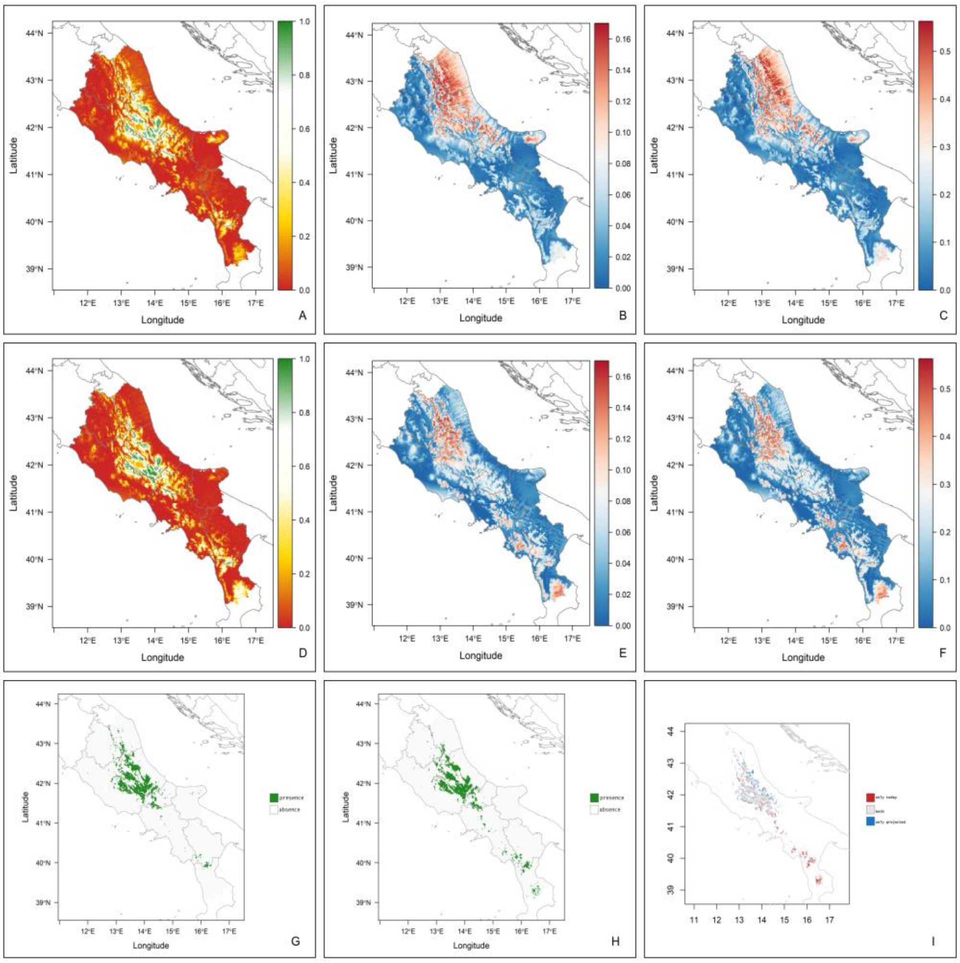

Fig. S.3.264-A) 'Projected' base output; B) 'Projected' uncertainty standard deviation; C) 'Projected' uncertainty range of values; D) 'Present' base output; E) 'Present' uncertainty standard deviation; F) 'Present' uncertainty range of values; G) 'Projected' binary distribution; H) 'Present' binary distribution; I) Range size variation.

Value	Present	Projected
AUC.train	0.969	0.967
AUC.test	0.969	0.958
AUC.diff	0.013	0.013
pROC	1.823	1.742
OR.p10	0.125	0.175
CBI	0.869	0.916
perc.stable	62.302	62.302
range.size	-1.645	-1.645

S.3.265 *Siler montanum* Biomod2

Species: *Siler montanum* Crantz subsp. *siculum* (Spreng.) Iamonico, Bartolucci & F.Conti

Procedure: Biomod2

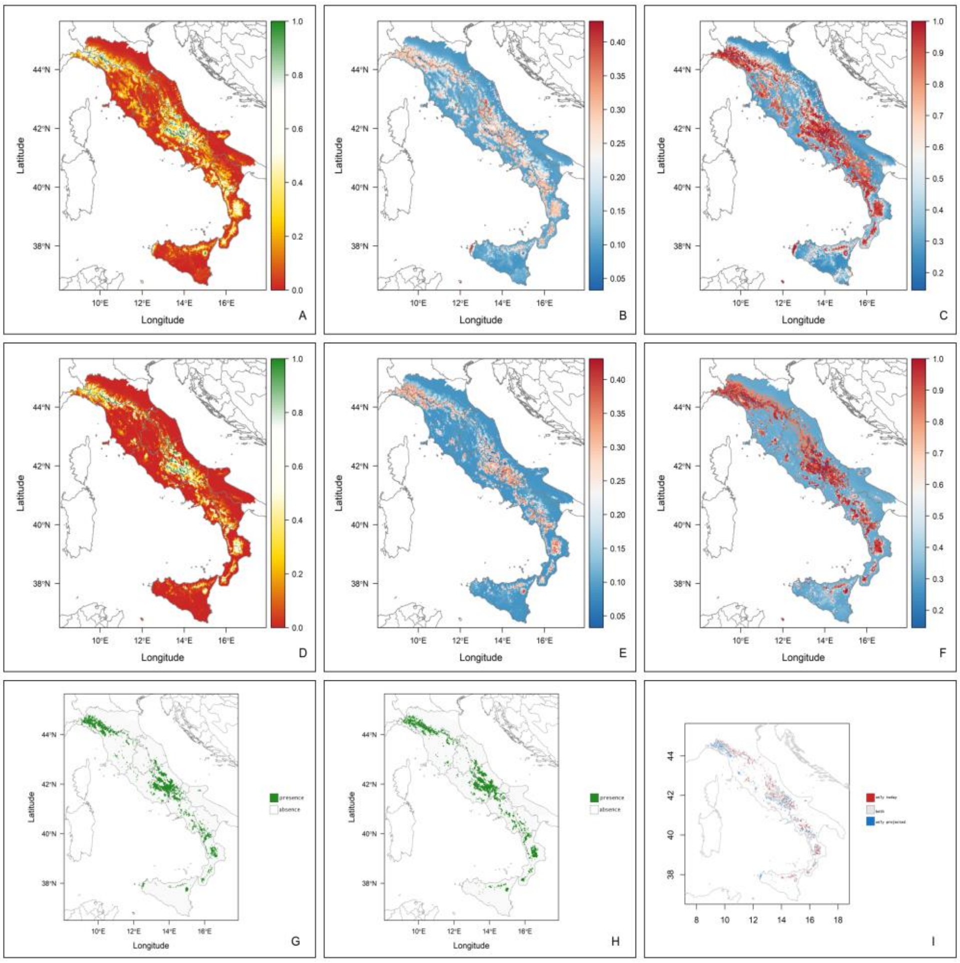

Fig. S.3.265-A) 'Projected' base output; B) 'Projected' uncertainty standard deviation; C) 'Projected' uncertainty range of values; D) 'Present' base output; E) 'Present' uncertainty standard deviation; F) 'Present' uncertainty range of values; G) 'Projected' binary distribution; H) 'Present' binary distribution; I) Range size variation.

Value	Present	Projected
AUC.train	0.890	0.975
AUC.test	0.890	0.863
AUC.diff	0.112	0.112
pROC	1.477	1.503
OR.p10	0.678	0.517
CBI	0.991	0.995
perc.stable	57.824	57.824
range.size	-0.690	-0.690

S.3.266 *Siler montanum* Bioclim

Species: *Siler montanum* Crantz subsp. *siculum* (Spreng.) Iamonico, Bartolucci & F.Conti

Procedure: Bioclim

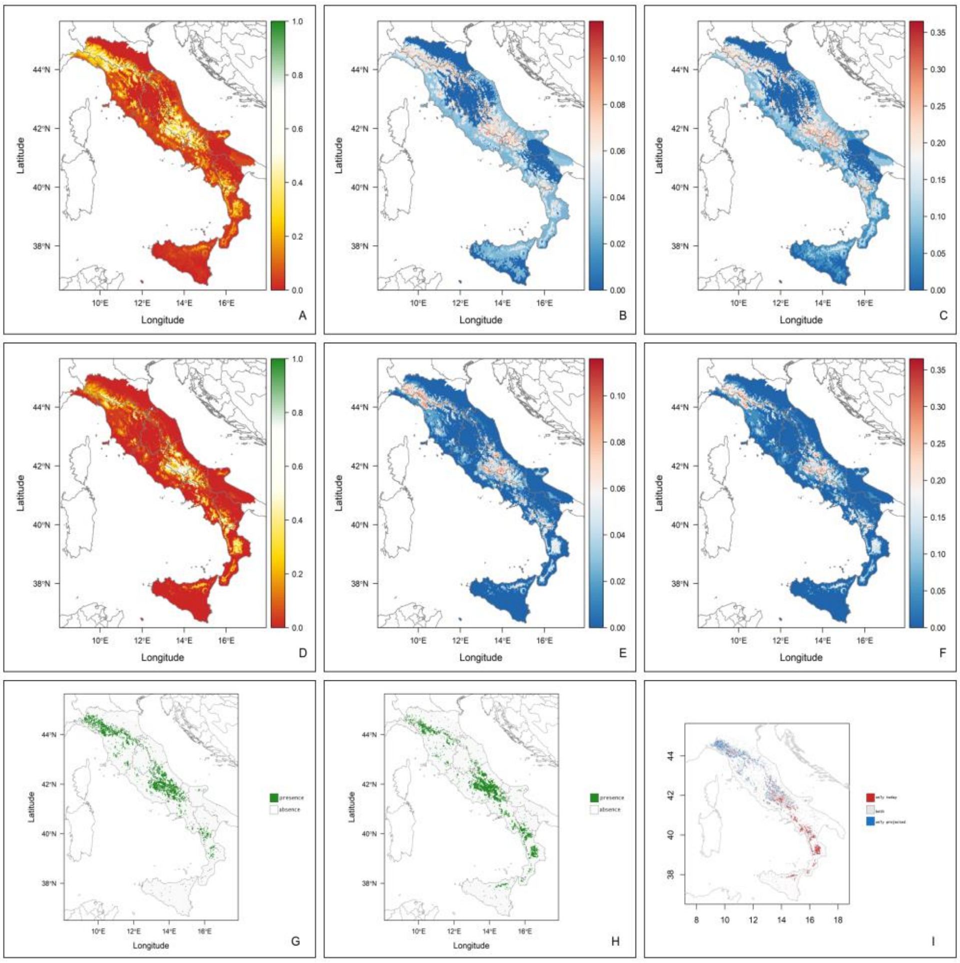

Fig. S.3.266-A) 'Projected' base output; B) 'Projected' uncertainty standard deviation; C) 'Projected' uncertainty range of values; D) 'Present' base output; E) 'Present' uncertainty standard deviation; F) 'Present' uncertainty range of values; G) 'Projected' binary distribution; H) 'Present' binary distribution; I) Range size variation.

Value	Present	Projected
AUC.train	0.830	0.817
AUC.test	0.830	0.754
AUC.diff	0.064	0.064
pROC	1.277	1.110
OR.p10	0.128	0.206
CBI	0.856	0.789
perc.stable	40.742	40.742
range.size	-0.003	-0.003

S.3.267 *Siler montanum* Domain

Species: *Siler montanum* Crantz subsp. *siculum* (Spreng.) Iamonico, Bartolucci & F.Conti

Procedure: Domain

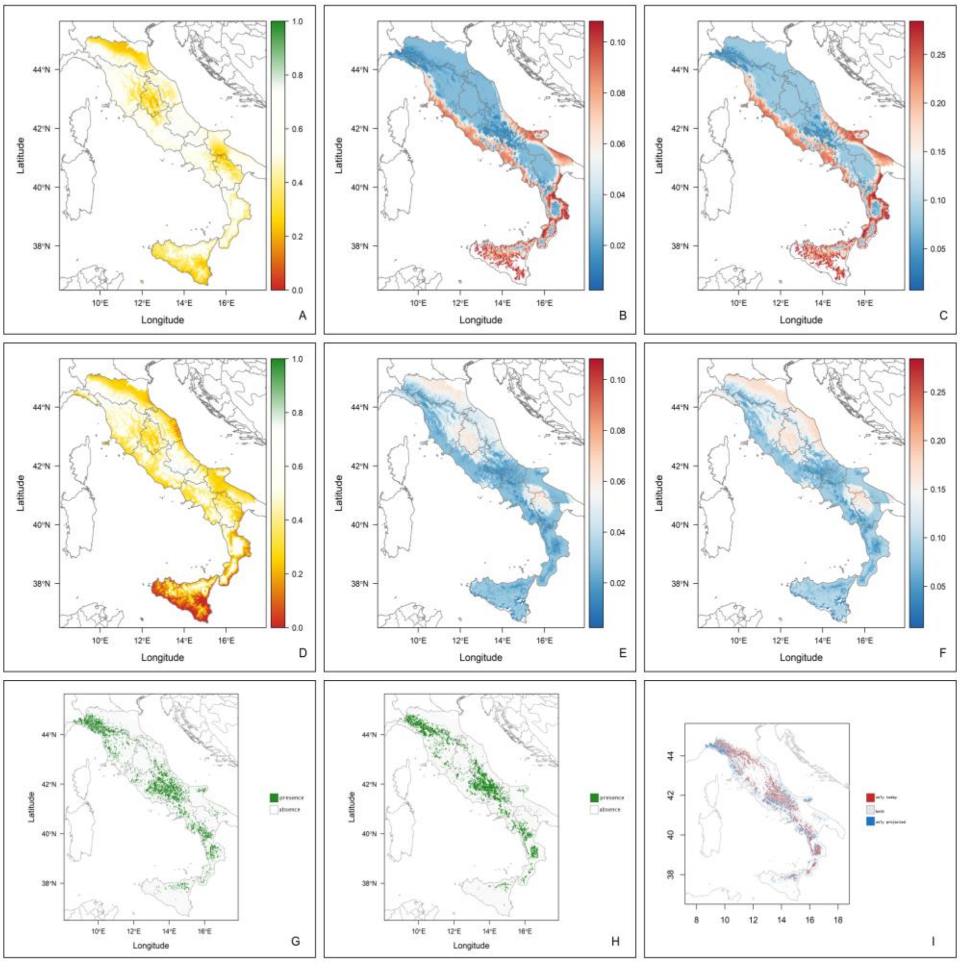

Fig. S.3.267-A) 'Projected' base output; B) 'Projected' uncertainty standard deviation; C) 'Projected' uncertainty range of values; D) 'Present' base output; E) 'Present' uncertainty standard deviation; F) 'Present' uncertainty range of values; G) 'Projected' binary distribution; H) 'Present' binary distribution; I) Range size variation.

Value	Present	Projected
AUC.train	0.856	0.797
AUC.test	0.856	0.784
AUC.diff	0.023	0.023
pROC	1.502	1.234
OR.p10	0.128	0.189
CBI	0.996	0.848
perc.stable	31.933	31.933
range.size	0.578	0.578

S.3.268 *Siler montanum* Maxent

Species: *Siler montanum* Crantz subsp. *siculum* (Spreng.) Iamonico, Bartolucci & F.Conti

Procedure: Maxent

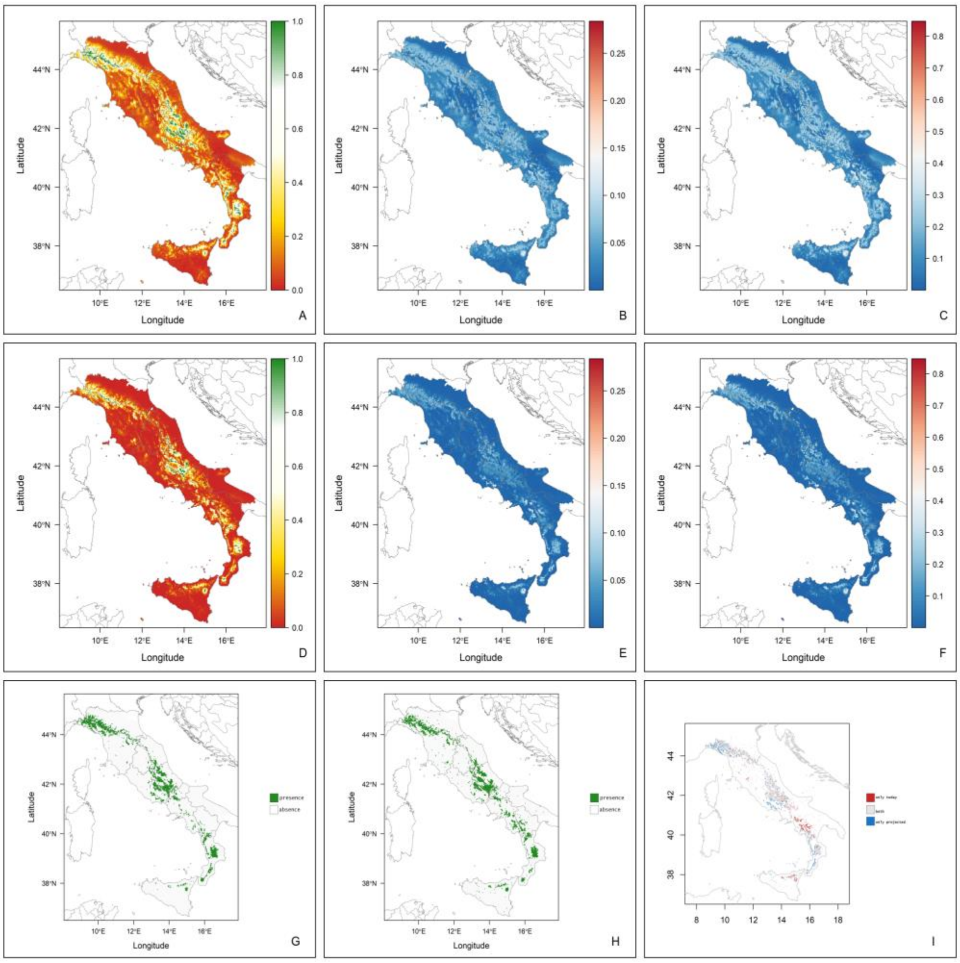

Fig. S.3.268-A) 'Projected' base output; B) 'Projected' uncertainty standard deviation; C) 'Projected' uncertainty range of values; D) 'Present' base output; E) 'Present' uncertainty standard deviation; F) 'Present' uncertainty range of values; G) 'Projected' binary distribution; H) 'Present' binary distribution; I) Range size variation.

Value	Present	Projected
AUC.train	0.929	0.869
AUC.test	0.929	0.888
AUC.diff	0.016	0.016
pROC	1.603	1.481
OR.p10	0.128	0.117
CBI	0.954	0.969
perc.stable	62.285	62.285
range.size	0.712	0.712

S.3.269 *Siler montanum* KDE

Species: *Siler montanum* Crantz subsp. *siculum* (Spreng.) Iamonico, Bartolucci & F.Conti

Procedure: KDE

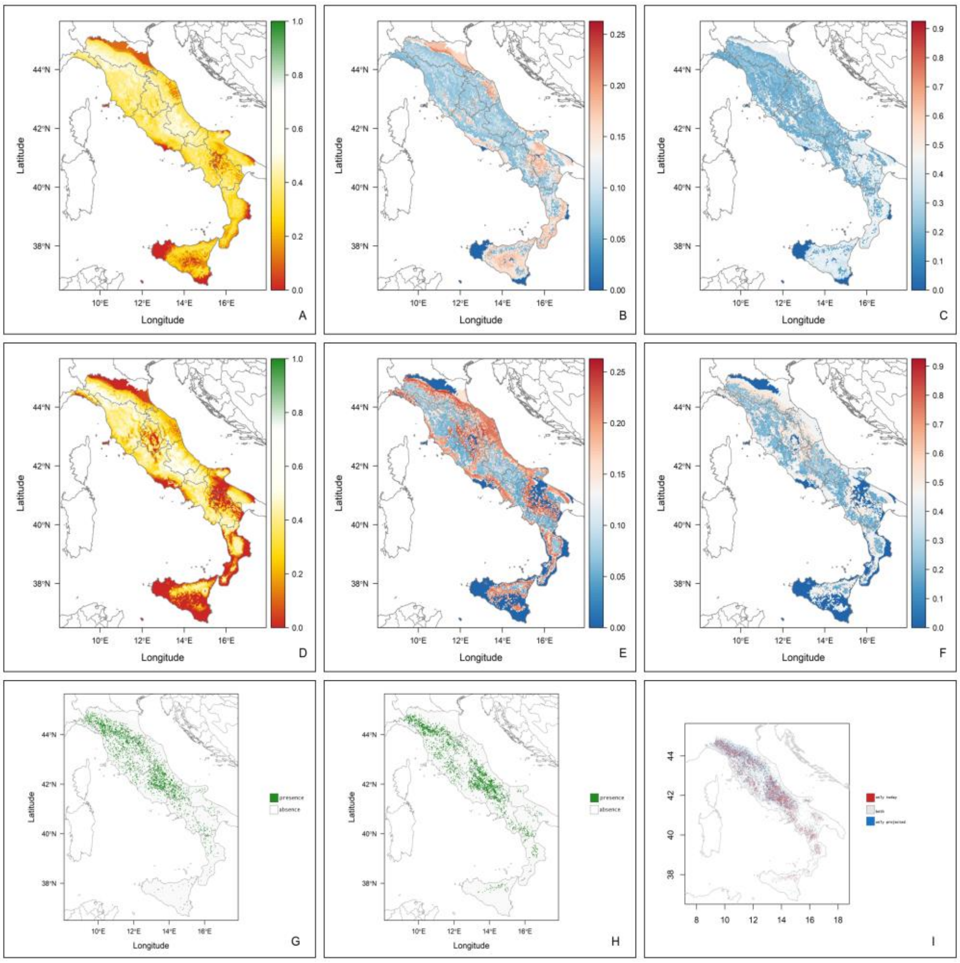

Fig. S.3.269-A) 'Projected' base output; B) 'Projected' uncertainty standard deviation; C) 'Projected' uncertainty range of values; D) 'Present' base output; E) 'Present' uncertainty standard deviation; F) 'Present' uncertainty range of values; G) 'Projected' binary distribution; H) 'Present' binary distribution; I) Range size variation.

Value	Present	Projected
AUC.train	0.838	0.760
AUC.test	0.838	0.755
AUC.diff	0.046	0.027
pROC	1.344	1.176
OR.p10	0.222	0.156
CBI	0.979	0.943
perc.stable	17.736	17.736
range.size	1.012	1.012

S.3.270 *Siler montanum* CTA

Species: *Siler montanum* Crantz subsp. *siculum* (Spreng.) Iamonico, Bartolucci & F.Conti

Procedure: CTA

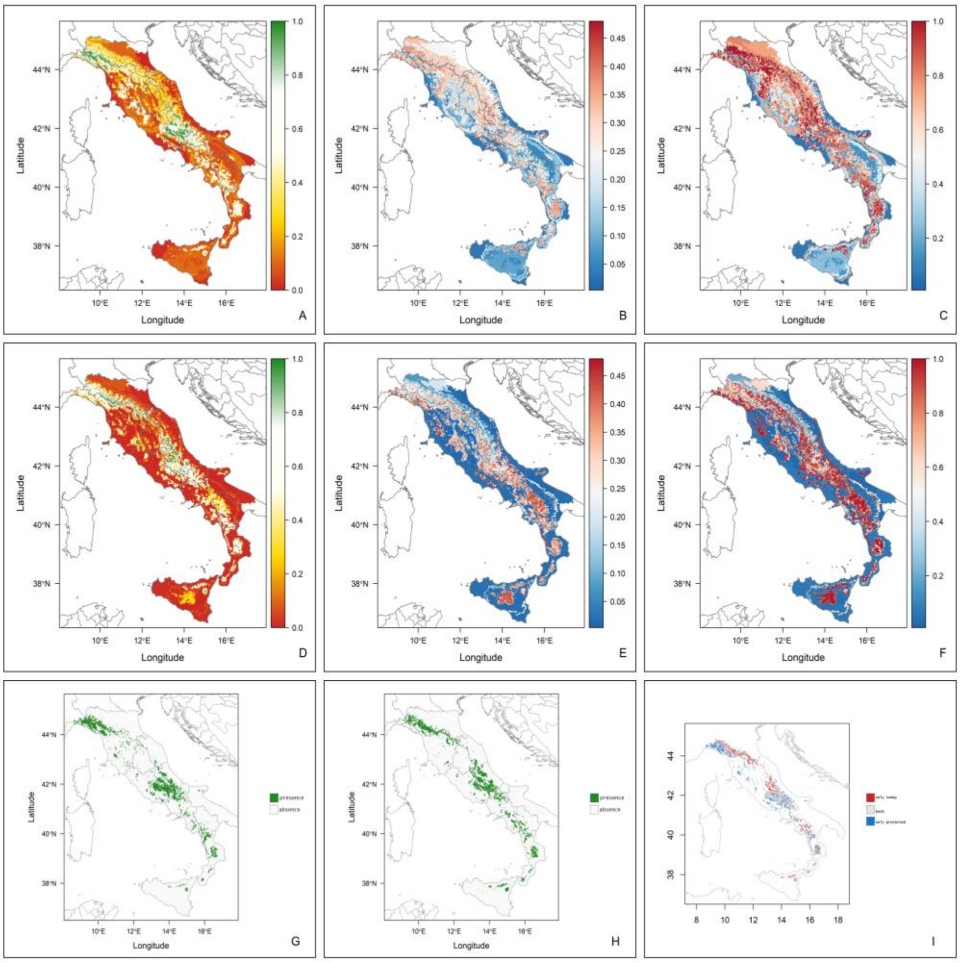

Fig. S.3.270-A) 'Projected' base output; B) 'Projected' uncertainty standard deviation; C) 'Projected' uncertainty range of values; D) 'Present' base output; E) 'Present' uncertainty standard deviation; F) 'Present' uncertainty range of values; G) 'Projected' binary distribution; H) 'Present' binary distribution; I) Range size variation.

Value	Present	Projected
AUC.train	0.888	0.946
AUC.test	0.888	0.881
AUC.diff	0.069	0.069
pROC	1.220	1.248
OR.p10	0.233	0.172
CBI	0.975	0.981
perc.stable	41.361	41.361
range.size	0.448	0.448

S.3.271 *Siler montanum* FDA

Species: *Siler montanum* Crantz subsp. *siculum* (Spreng.) Iamonico, Bartolucci & F.Conti

Procedure: FDA

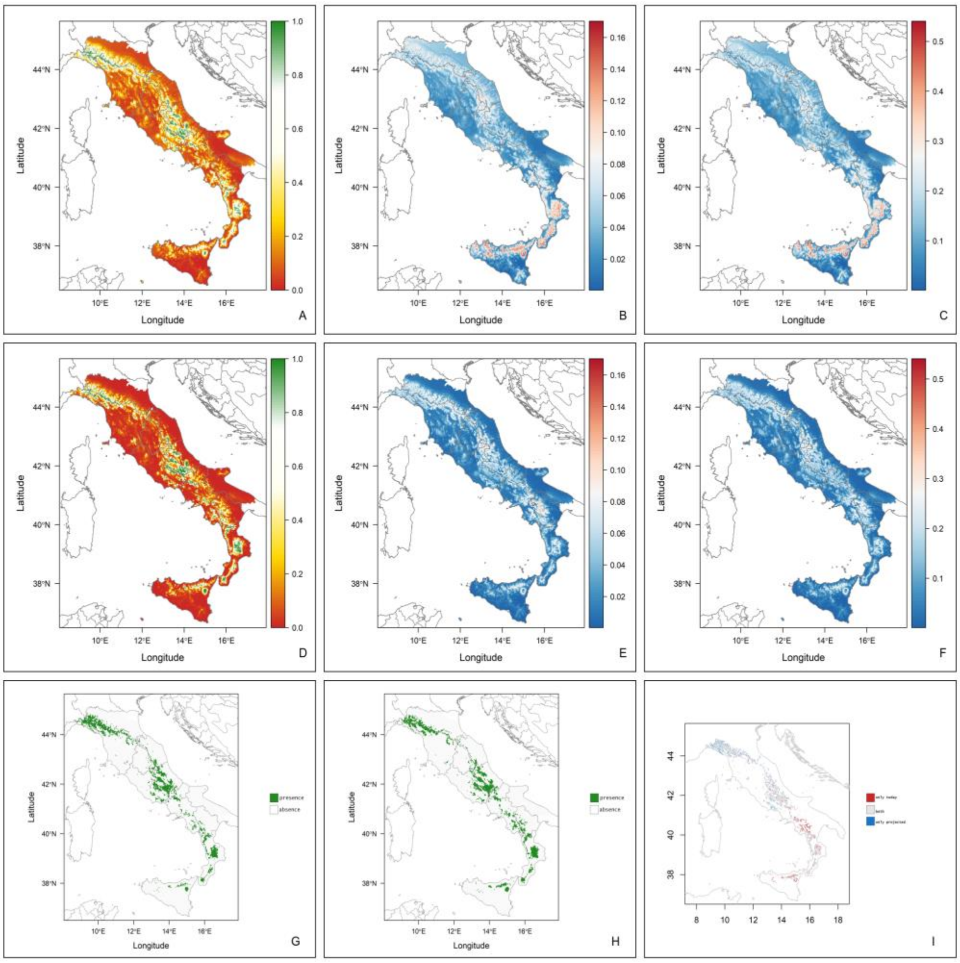

Fig. S.3.271-A) 'Projected' base output; B) 'Projected' uncertainty standard deviation; C) 'Projected' uncertainty range of values; D) 'Present' base output; E) 'Present' uncertainty standard deviation; F) 'Present' uncertainty range of values; G) 'Projected' binary distribution; H) 'Present' binary distribution; I) Range size variation.

Value	Present	Projected
AUC.train	0.910	0.878
AUC.test	0.910	0.869
AUC.diff	0.024	0.024
pROC	1.534	1.382
OR.p10	0.167	0.156
CBI	0.954	0.984
perc.stable	69.418	69.418
range.size	-0.218	-0.218

S.3.272 *Siler montanum* GAM

Species: *Siler montanum* Crantz subsp. *siculum* (Spreng.) Iamonico, Bartolucci & F.Conti

Procedure: GAM

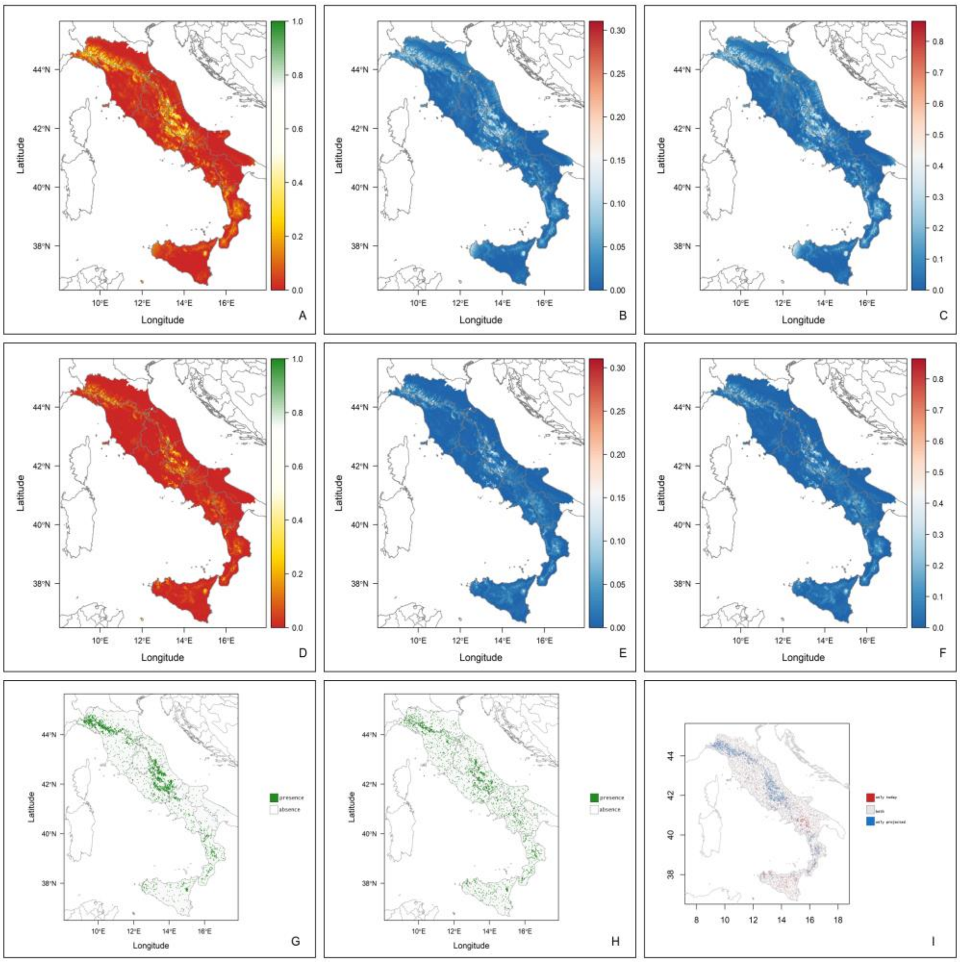

Fig. S.3.272-A) 'Projected' base output; B) 'Projected' uncertainty standard deviation; C) 'Projected' uncertainty range of values; D) 'Present' base output; E) 'Present' uncertainty standard deviation; F) 'Present' uncertainty range of values; G) 'Projected' binary distribution; H) 'Present' binary distribution; I) Range size variation.

Value	Present	Projected
AUC.train	0.919	0.862
AUC.test	0.919	0.843
AUC.diff	0.027	0.027
pROC	1.553	1.331
OR.p10	0.167	0.139
CBI	0.867	0.916
perc.stable	18.255	18.255
range.size	-0.573	-0.573

S.3.273 *Siler montanum* GLM

Species: *Siler montanum* Crantz subsp. *siculum* (Spreng.) Iamonico, Bartolucci & F.Conti

Procedure: GLM

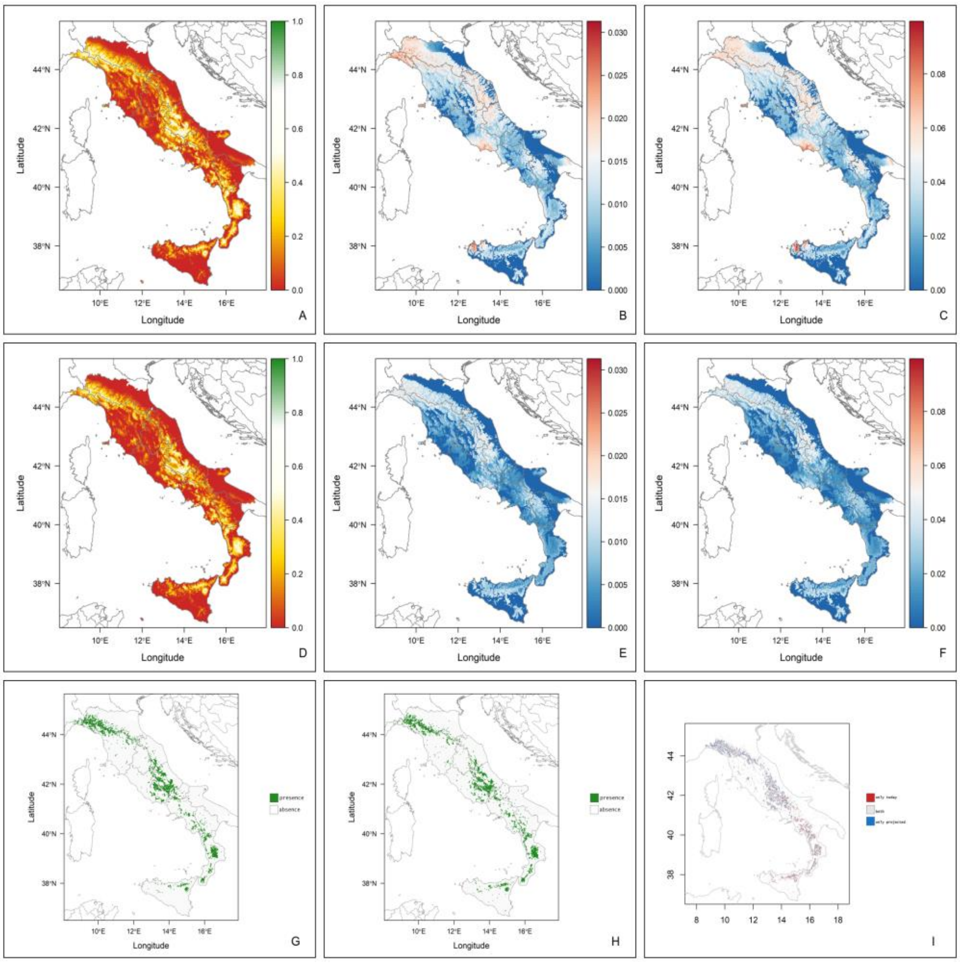

Fig. S.3.273-A) 'Projected' base output; B) 'Projected' uncertainty standard deviation; C) 'Projected' uncertainty range of values; D) 'Present' base output; E) 'Present' uncertainty standard deviation; F) 'Present' uncertainty range of values; G) 'Projected' binary distribution; H) 'Present' binary distribution; I) Range size variation.

Value	Present	Projected
AUC.train	0.917	0.866
AUC.test	0.917	0.872
AUC.diff	0.017	0.017
pROC	1.568	1.387
OR.p10	0.156	0.156
CBI	0.866	0.928
perc.stable	48.684	48.684
range.size	0.864	0.864

S.3.274 *Siler montanum* MARS

Species: *Siler montanum* Crantz subsp. *siculum* (Spreng.) Iamonico, Bartolucci & F.Conti

Procedure: MARS

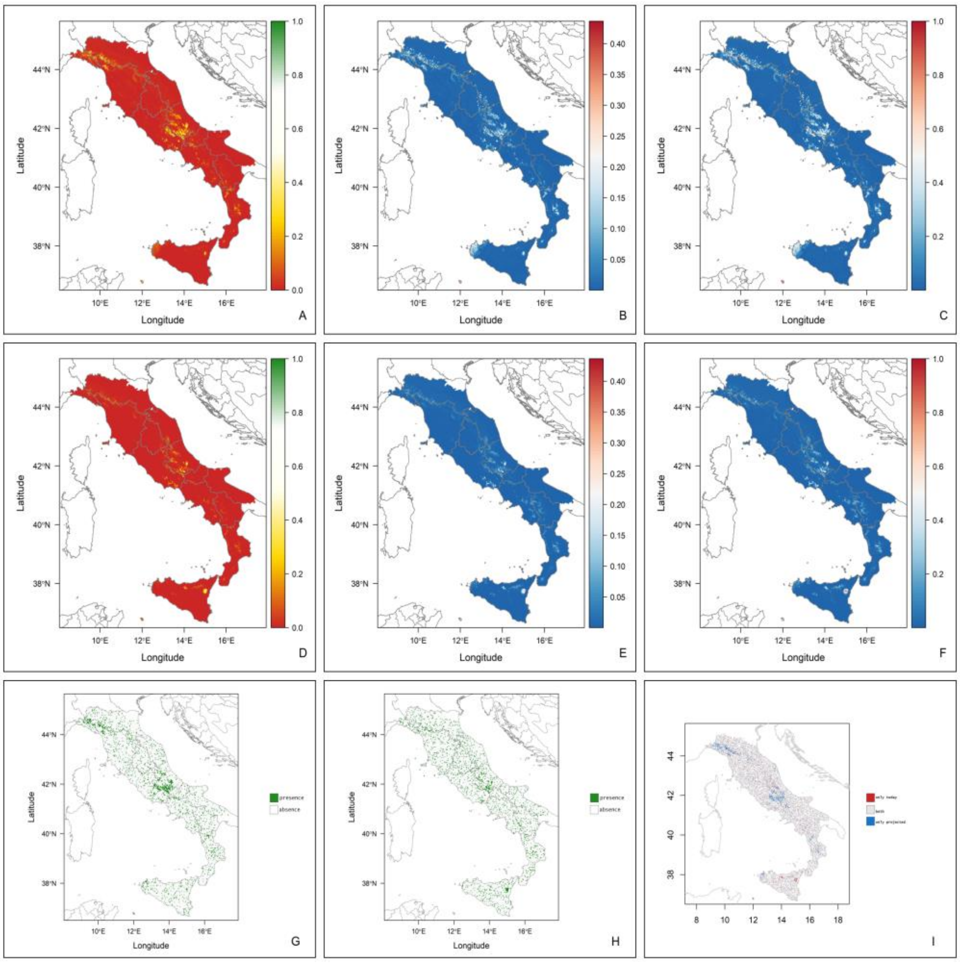

Fig. S.3.274-A) 'Projected' base output; B) 'Projected' uncertainty standard deviation; C) 'Projected' uncertainty range of values; D) 'Present' base output; E) 'Present' uncertainty standard deviation; F) 'Present' uncertainty range of values; G) 'Projected' binary distribution; H) 'Present' binary distribution; I) Range size variation.

Value	Present	Projected
AUC.train	0.911	0.890
AUC.test	0.911	0.842
AUC.diff	0.052	0.052
pROC	1.434	1.274
OR.p10	0.117	0.178
CBI	0.666	0.810
perc.stable	9.035	9.035
range.size	-0.675	-0.675

S.3.275 *Siler montanum* RF

Species: *Siler montanum* Crantz subsp. *siculum* (Spreng.) Iamonico, Bartolucci & F.Conti

Procedure: RF

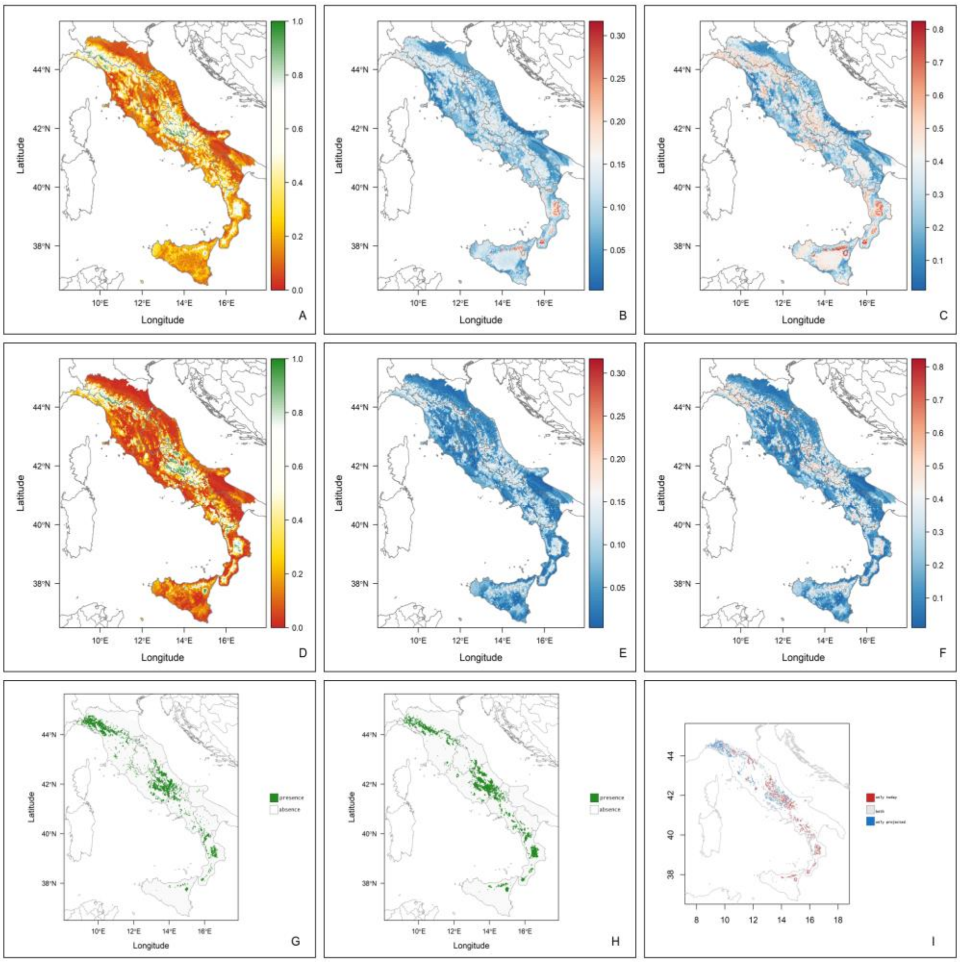

Fig. S.3.275-A) 'Projected' base output; B) 'Projected' uncertainty standard deviation; C) 'Projected' uncertainty range of values; D) 'Present' base output; E) 'Present' uncertainty standard deviation; F) 'Present' uncertainty range of values; G) 'Projected' binary distribution; H) 'Present' binary distribution; I) Range size variation.

Value	Present	Projected
AUC.train	0.965	1.000
AUC.test	0.965	0.969
AUC.diff	0.031	0.031
pROC	1.458	1.393
OR.p10	0.406	0.494
CBI	0.963	0.967
perc.stable	49.953	49.953
range.size	-0.315	-0.315

S.3.276 *Siler montanum* SVM

Species: *Siler montanum* Crantz subsp. *siculum* (Spreng.) Iamonico, Bartolucci & F.Conti

Procedure: SVM

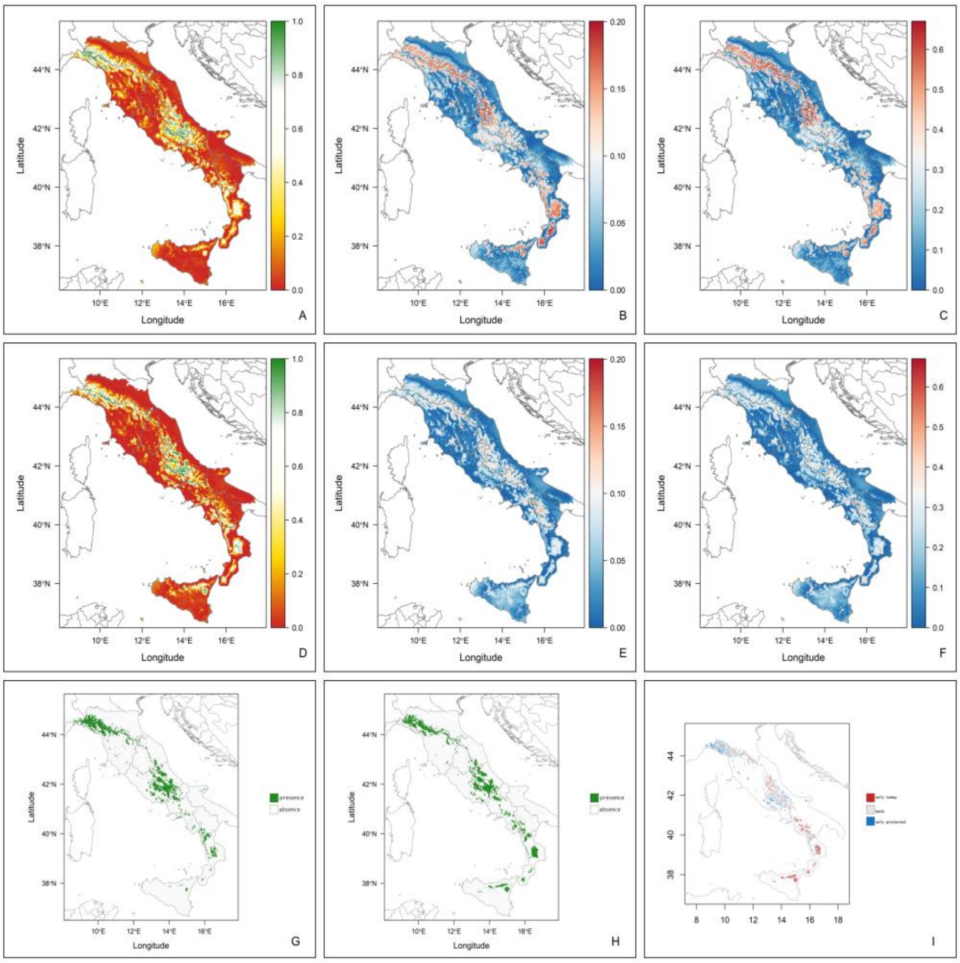

Fig. S.3.276-A) 'Projected' base output; B) 'Projected' uncertainty standard deviation; C) 'Projected' uncertainty range of values; D) 'Present' base output; E) 'Present' uncertainty standard deviation; F) 'Present' uncertainty range of values; G) 'Projected' binary distribution; H) 'Present' binary distribution; I) Range size variation.

Value	Present	Projected
AUC.train	0.936	0.921
AUC.test	0.936	0.889
AUC.diff	0.033	0.033
pROC	1.549	1.372
OR.p10	0.150	0.200
CBI	0.977	0.986
perc.stable	61.464	61.464
range.size	-0.355	-0.355

S.3.277 *Viola eugeniae* Biomod2

Species: *Viola eugeniae* Parl. subsp. *eugeniae*

Procedure: Biomod2

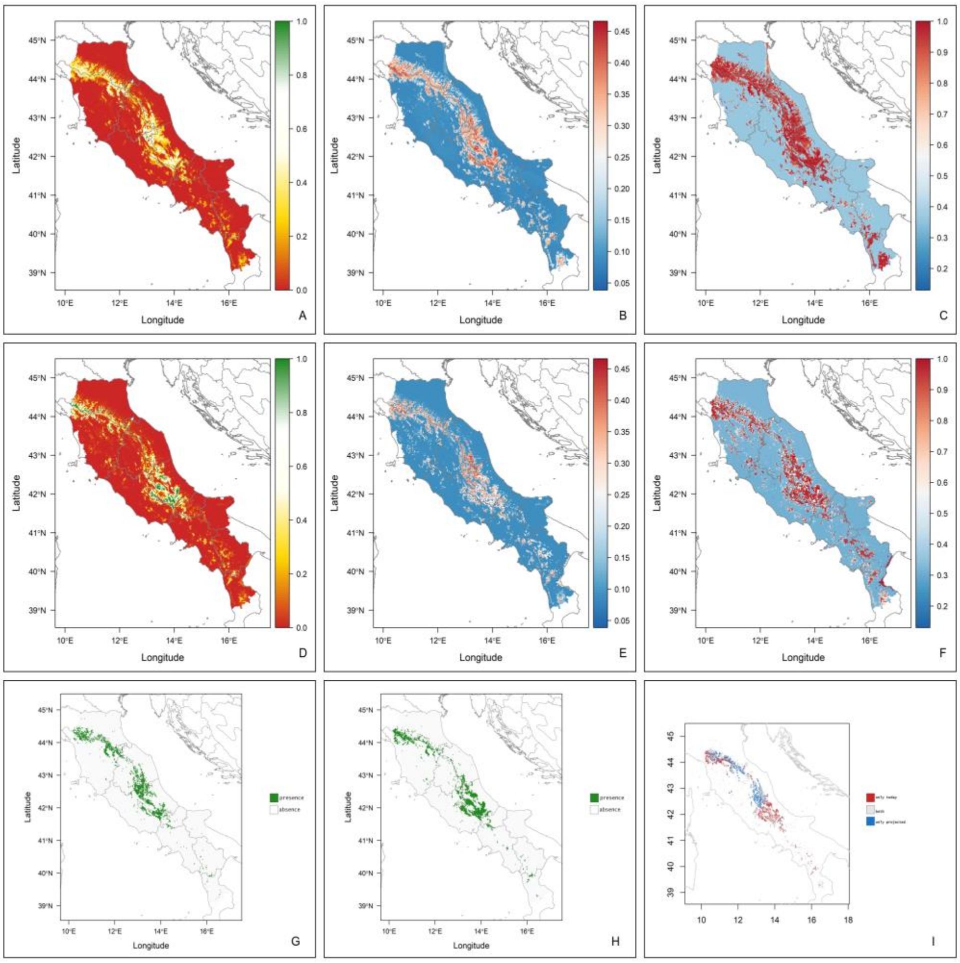

Fig. S.3.277-A) 'Projected' base output; B) 'Projected' uncertainty standard deviation; C) 'Projected' uncertainty range of values; D) 'Present' base output; E) 'Present' uncertainty standard deviation; F) 'Present' uncertainty range of values; G) 'Projected' binary distribution; H) 'Present' binary distribution; I) Range size variation.

Value	Present	Projected
AUC.train	0.960	0.993
AUC.test	0.960	0.974
AUC.diff	0.019	0.019
pROC	1.762	1.860
OR.p10	0.382	0.400
CBI	0.963	0.805
perc.stable	35.587	35.587
range.size	-1.882	-1.882

S.3.278 *Viola eugeniae* Bioclim

Species: *Viola eugeniae* Parl. subsp. *eugeniae*

Procedure: Bioclim

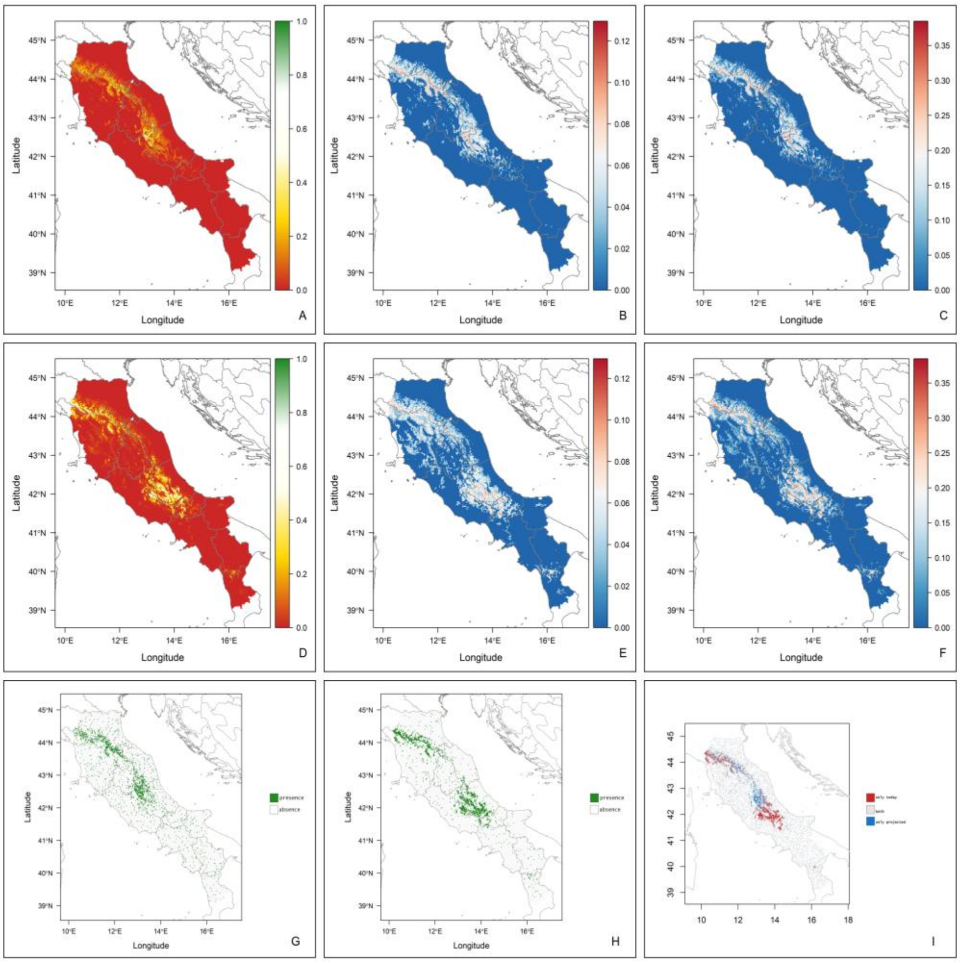

Fig. S.3.278-A) 'Projected' base output; B) 'Projected' uncertainty standard deviation; C) 'Projected' uncertainty range of values; D) 'Present' base output; E) 'Present' uncertainty standard deviation; F) 'Present' uncertainty range of values; G) 'Projected' binary distribution; H) 'Present' binary distribution; I) Range size variation.

Value	Present	Projected
AUC.train	0.823	0.942
AUC.test	0.823	0.838
AUC.diff	0.103	0.103
pROC	1.205	1.200
OR.p10	0.236	0.218
CBI	0.771	0.301
perc.stable	13.336	13.336
range.size	0.335	0.335

S.3.279 *Viola eugeniae* Domain

Species: *Viola eugeniae* Parl. subsp. *eugeniae*

Procedure: Domain

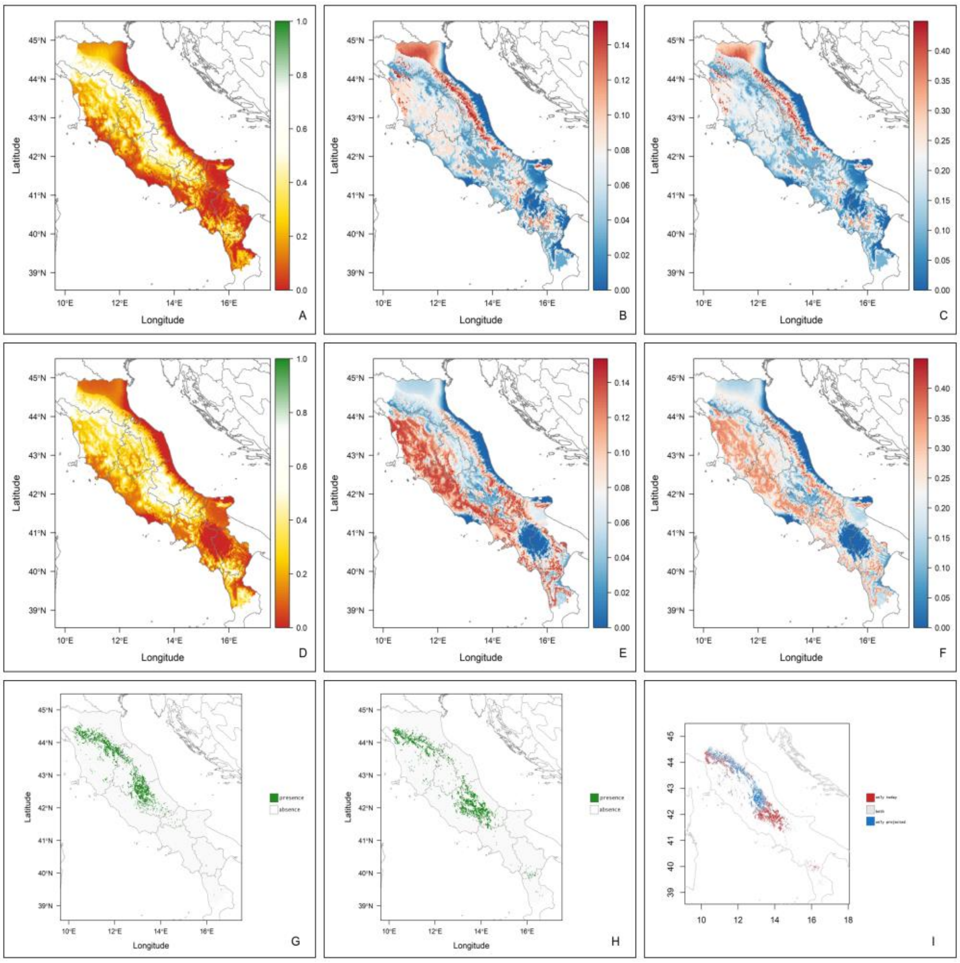

Fig. S.3.279-A) 'Projected' base output; B) 'Projected' uncertainty standard deviation; C) 'Projected' uncertainty range of values; D) 'Present' base output; E) 'Present' uncertainty standard deviation; F) 'Present' uncertainty range of values; G) 'Projected' binary distribution; H) 'Present' binary distribution; I) Range size variation.

Value	Present	Projected
AUC.train	0.911	0.916
AUC.test	0.911	0.906
AUC.diff	0.025	0.025
pROC	1.681	1.613
OR.p10	0.182	0.182
CBI	0.968	0.772
perc.stable	21.462	21.462
range.size	-0.822	-0.822

S.3.280 *Viola eugeniae* Maxent

Species: *Viola eugeniae* Parl. subsp. *eugeniae*

Procedure: Maxent

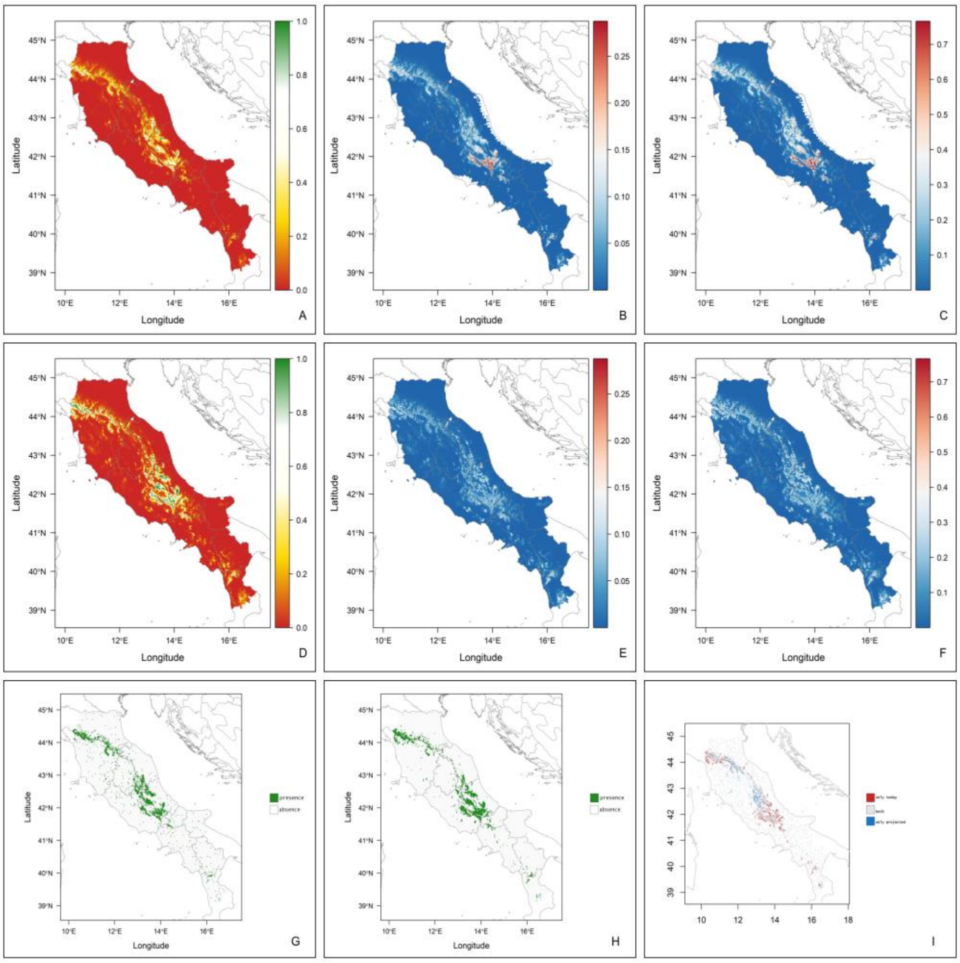

Fig. S.3.280-A) 'Projected' base output; B) 'Projected' uncertainty standard deviation; C) 'Projected' uncertainty range of values; D) 'Present' base output; E) 'Present' uncertainty standard deviation; F) 'Present' uncertainty range of values; G) 'Projected' binary distribution; H) 'Present' binary distribution; I) Range size variation.

Value	Present	Projected
AUC.train	0.937	0.970
AUC.test	0.937	0.953
AUC.diff	0.023	0.023
pROC	1.688	1.759
OR.p10	0.209	0.191
CBI	0.979	0.968
perc.stable	46.433	46.433
range.size	1.990	1.990

S.3.281 *Viola eugeniae* KDE

Species: *Viola eugeniae* Parl. subsp. *eugeniae*

Procedure: KDE

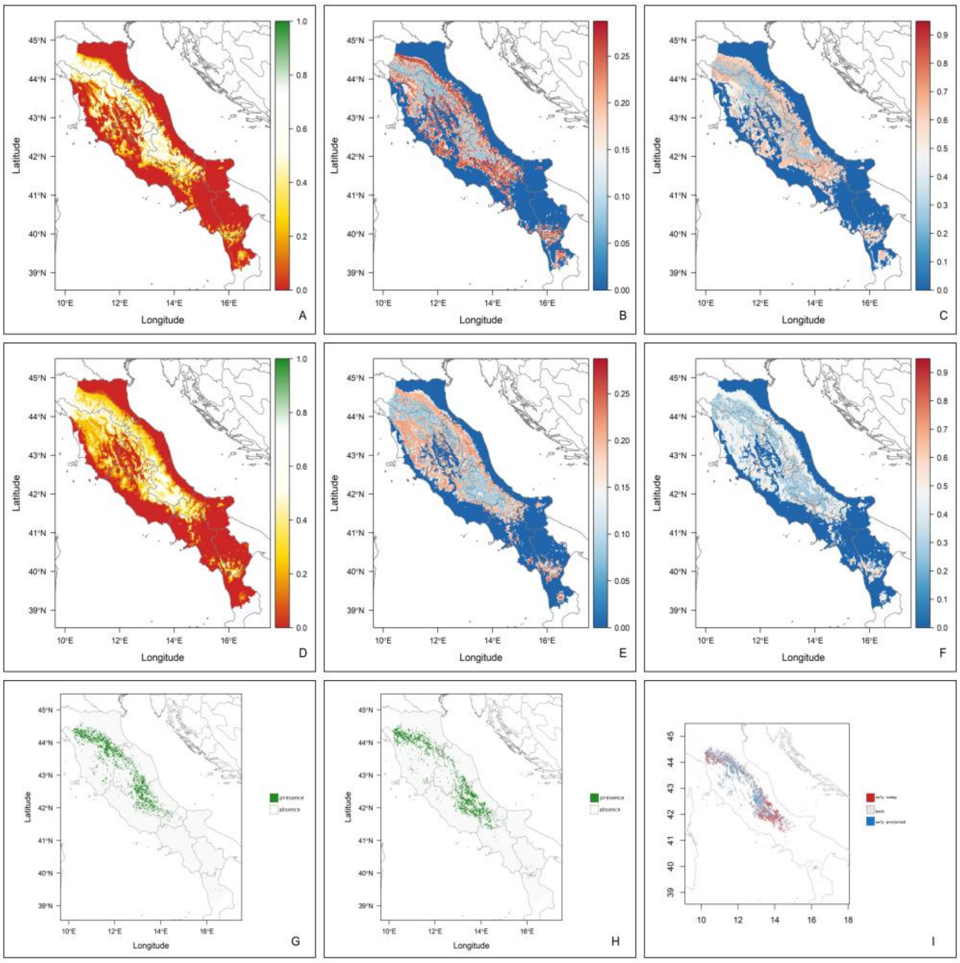

Fig. S.3.281-A) 'Projected' base output; B) 'Projected' uncertainty standard deviation; C) 'Projected' uncertainty range of values; D) 'Present' base output; E) 'Present' uncertainty standard deviation; F) 'Present' uncertainty range of values; G) 'Projected' binary distribution; H) 'Present' binary distribution; I) Range size variation.

Value	Present	Projected
AUC.train	0.893	0.921
AUC.test	0.893	0.872
AUC.diff	0.048	0.049
pROC	1.541	1.522
OR.p10	0.209	0.273
CBI	0.980	0.832
perc.stable	23.086	23.086
range.size	-1.060	-1.060

S.3.282 *Viola eugeniae* CTA

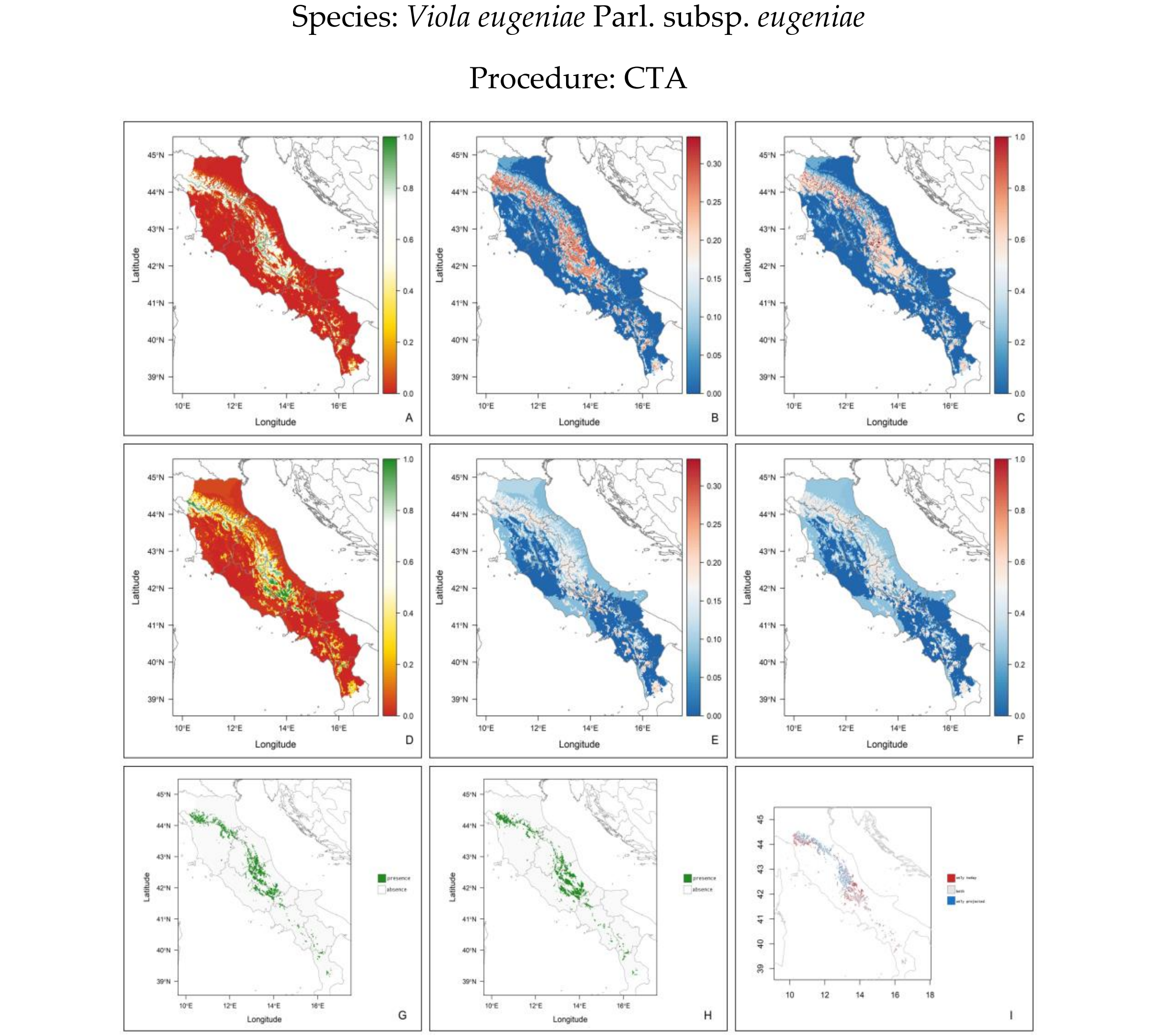

Fig. S.3.282-A) 'Projected' base output; B) 'Projected' uncertainty standard deviation; C) 'Projected' uncertainty range of values; D) 'Present' base output; E) 'Present' uncertainty standard deviation; F) 'Present' uncertainty range of values; G) 'Projected' binary distribution; H) 'Present' binary distribution; I) Range size variation.

Value	Present	Projected
AUC.train	0.931	0.972
AUC.test	0.931	0.947
AUC.diff	0.032	0.032
pROC	1.410	1.464
OR.p10	0.118	0.100
CBI	0.860	0.826
perc.stable	53.087	53.087
range.size	0.135	0.135

S.3.283 *Viola eugeniae* FDA

Species: *Viola eugeniae* Parl. subsp. *eugeniae*

Procedure: FDA

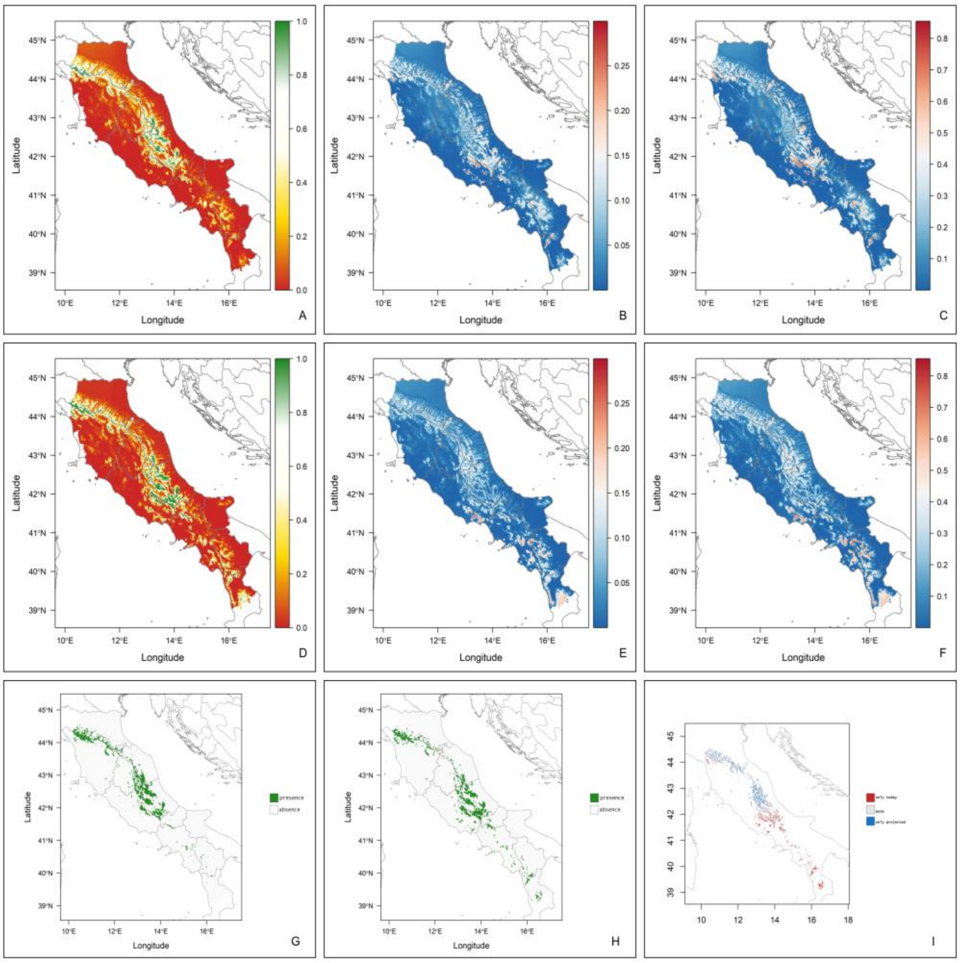

Fig. S.3.283-A) 'Projected' base output; B) 'Projected' uncertainty standard deviation; C) 'Projected' uncertainty range of values; D) 'Present' base output; E) 'Present' uncertainty standard deviation; F) 'Present' uncertainty range of values; G) 'Projected' binary distribution; H) 'Present' binary distribution; I) Range size variation.

Value	Present	Projected
AUC.train	0.945	0.958
AUC.test	0.945	0.967
AUC.diff	0.004	0.004
pROC	1.711	1.831
OR.p10	0.182	0.118
CBI	0.897	0.963
perc.stable	55.551	55.551
range.size	-0.560	-0.560

S.3.284 *Viola eugeniae* GAM

Species: *Viola eugeniae* Parl. subsp. *eugeniae*

Procedure: GAM

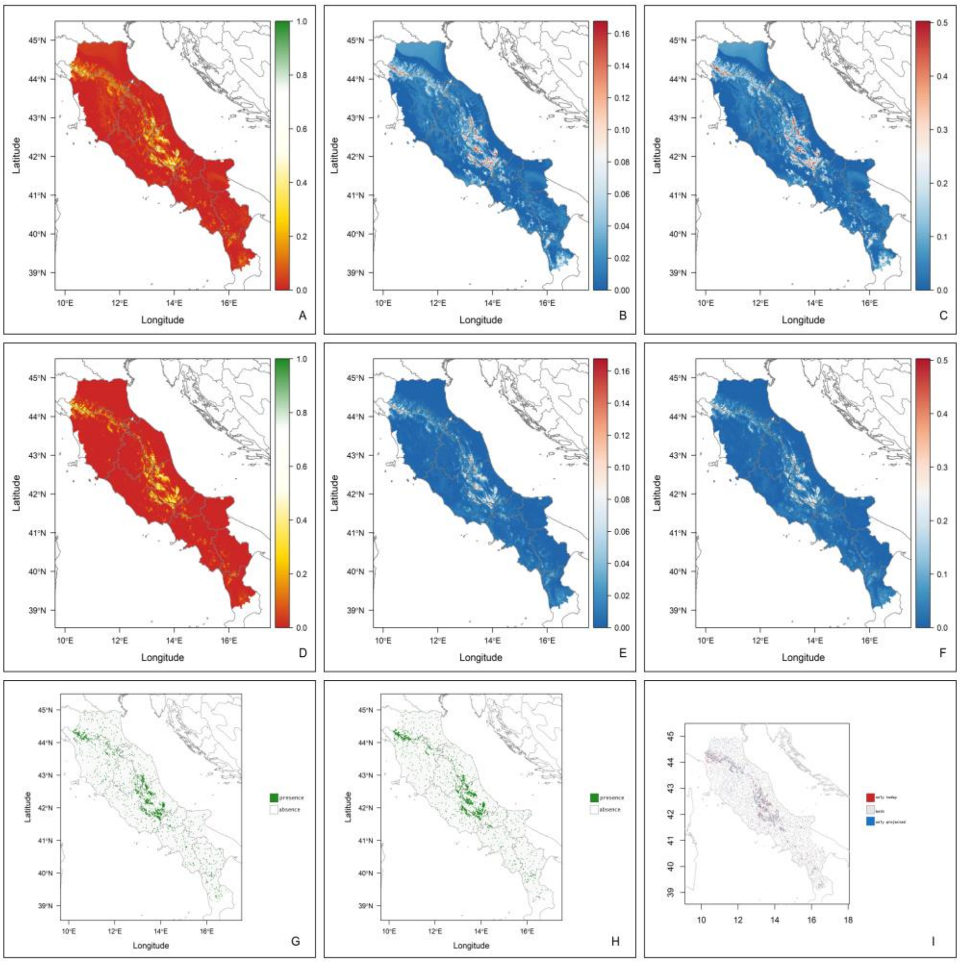

Fig. S.3.284-A) 'Projected' base output; B) 'Projected' uncertainty standard deviation; C) 'Projected' uncertainty range of values; D) 'Present' base output; E) 'Present' uncertainty standard deviation; F) 'Present' uncertainty range of values; G) 'Projected' binary distribution; H) 'Present' binary distribution; I) Range size variation.

Value	Present	Projected
AUC.train	0.915	0.963
AUC.test	0.915	0.961
AUC.diff	0.010	0.010
pROC	1.606	1.796
OR.p10	0.155	0.127
CBI	0.979	0.978
perc.stable	23.156	23.156
range.size	0.329	0.329

S.3.285 *Viola eugeniae* GLM

Species: *Viola eugeniae* Parl. subsp. *eugeniae*

Procedure: GLM

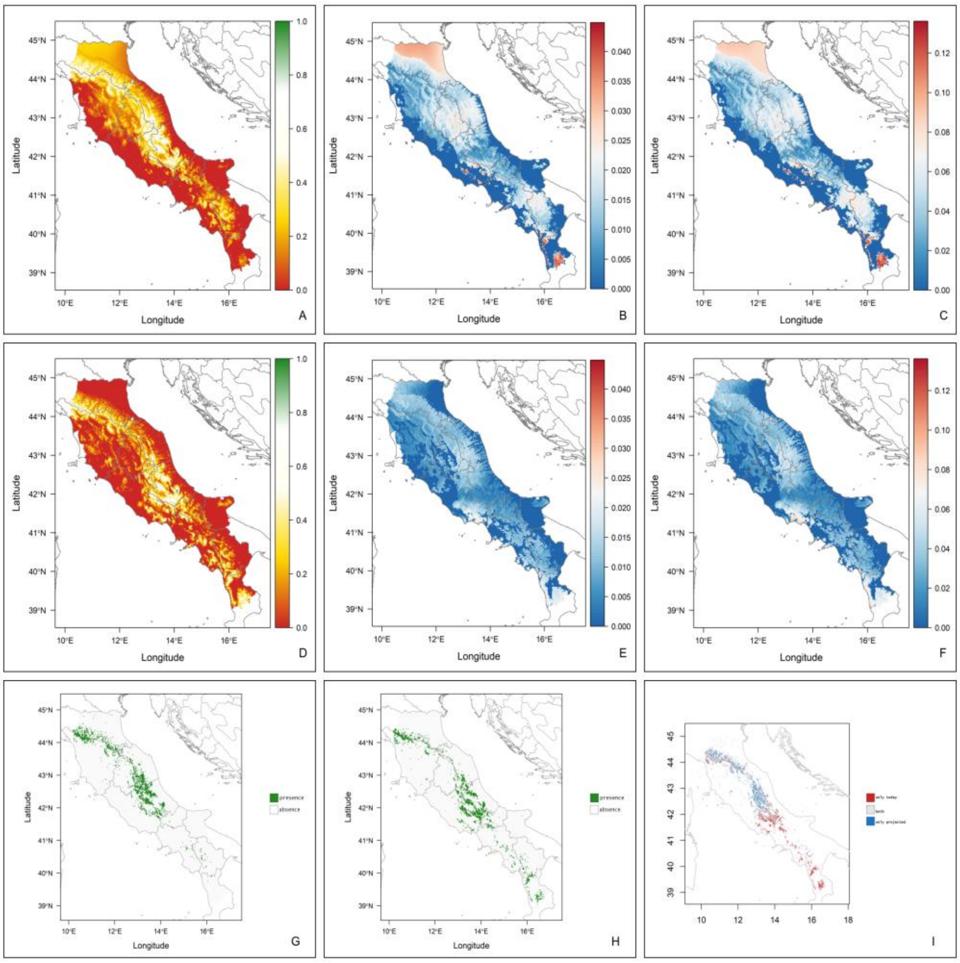

Fig. S.3.285-A) 'Projected' base output; B) 'Projected' uncertainty standard deviation; C) 'Projected' uncertainty range of values; D) 'Present' base output; E) 'Present' uncertainty standard deviation; F) 'Present' uncertainty range of values; G) 'Projected' binary distribution; H) 'Present' binary distribution; I) Range size variation.

Value	Present	Projected
AUC.train	0.947	0.957
AUC.test	0.947	0.956
AUC.diff	0.009	0.009
pROC	1.751	1.782
OR.p10	0.118	0.118
CBI	0.995	0.980
perc.stable	33.357	33.357
range.size	-2.881	-2.881

S.3.286 *Viola eugeniae* MARS

Species: *Viola eugeniae* Parl. subsp. *eugeniae*

Procedure: MARS

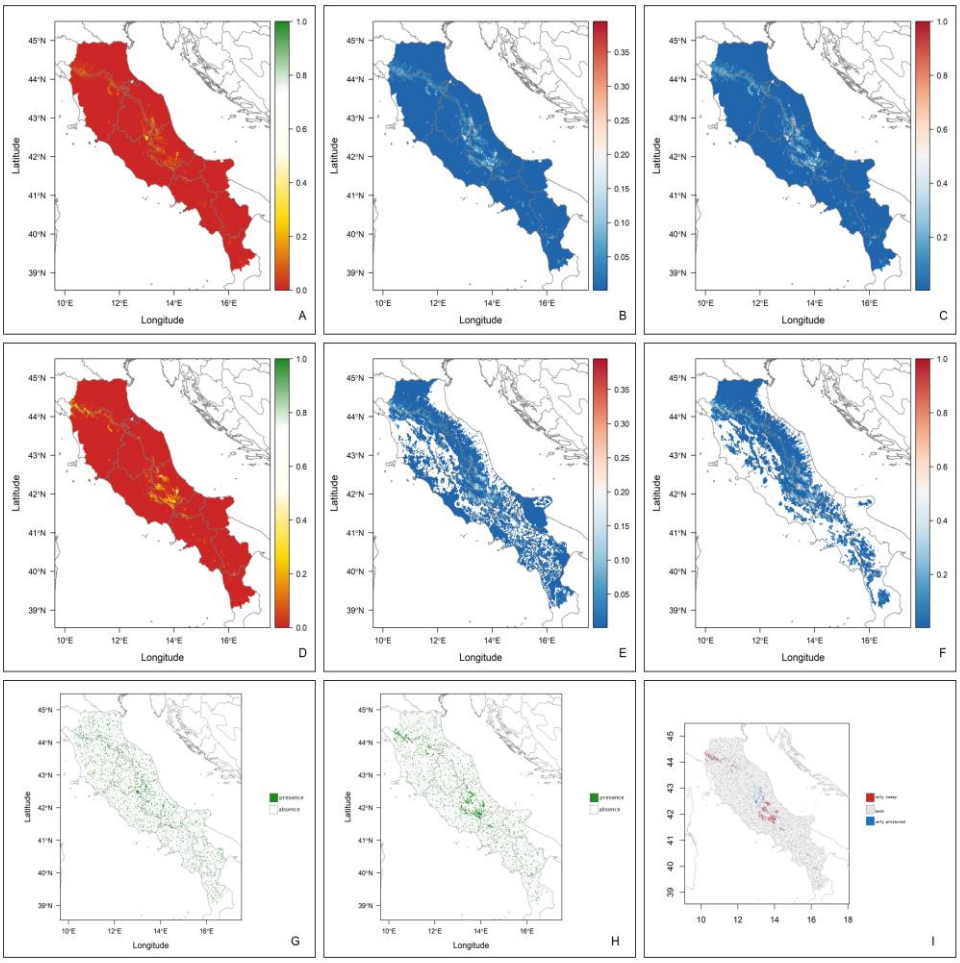

Fig. S.3.286-A) 'Projected' base output; B) 'Projected' uncertainty standard deviation; C) 'Projected' uncertainty range of values; D) 'Present' base output; E) 'Present' uncertainty standard deviation; F) 'Present' uncertainty range of values; G) 'Projected' binary distribution; H) 'Present' binary distribution; I) Range size variation.

Value	Present	Projected
AUC.train	0.825	0.951
AUC.test	0.825	0.875
AUC.diff	0.087	0.087
pROC	1.289	1.500
OR.p10	0.300	0.273
CBI	0.967	0.767
perc.stable	5.550	5.550
range.size	-0.171	-0.171

S.3.287 *Viola eugeniae* RF

Species: *Viola eugeniae* Parl. subsp. *eugeniae*

Procedure: RF

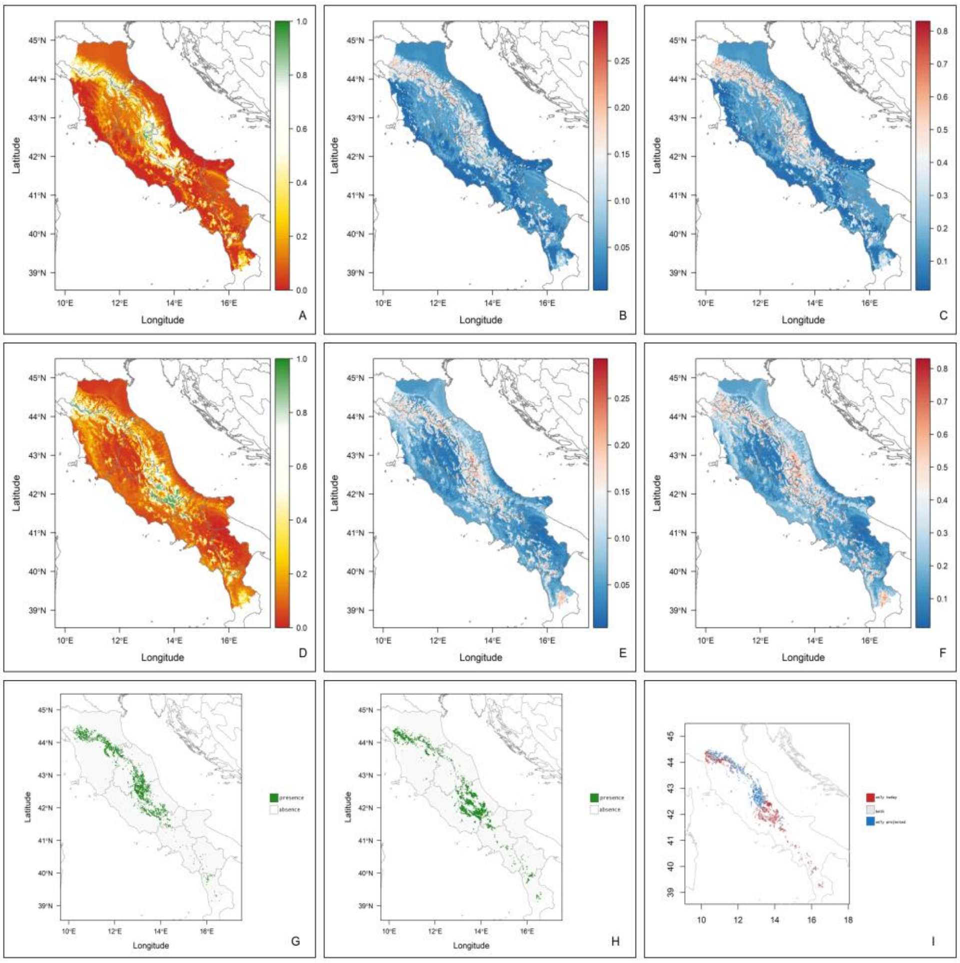

Fig. S.3.287-A) 'Projected' base output; B) 'Projected' uncertainty standard deviation; C) 'Projected' uncertainty range of values; D) 'Present' base output; E) 'Present' uncertainty standard deviation; F) 'Present' uncertainty range of values; G) 'Projected' binary distribution; H) 'Present' binary distribution; I) Range size variation.

Value	Present	Projected
AUC.train	0.960	1.000
AUC.test	0.960	0.997
AUC.diff	0.003	0.003
pROC	1.568	1.816
OR.p10	0.455	0.400
CBI	0.946	0.543
perc.stable	31.216	31.216
range.size	-0.178	-0.178

S.3.288 *Viola eugeniae* SVM

Species: *Viola eugeniae* Parl. subsp. *eugeniae*

Procedure: SVM

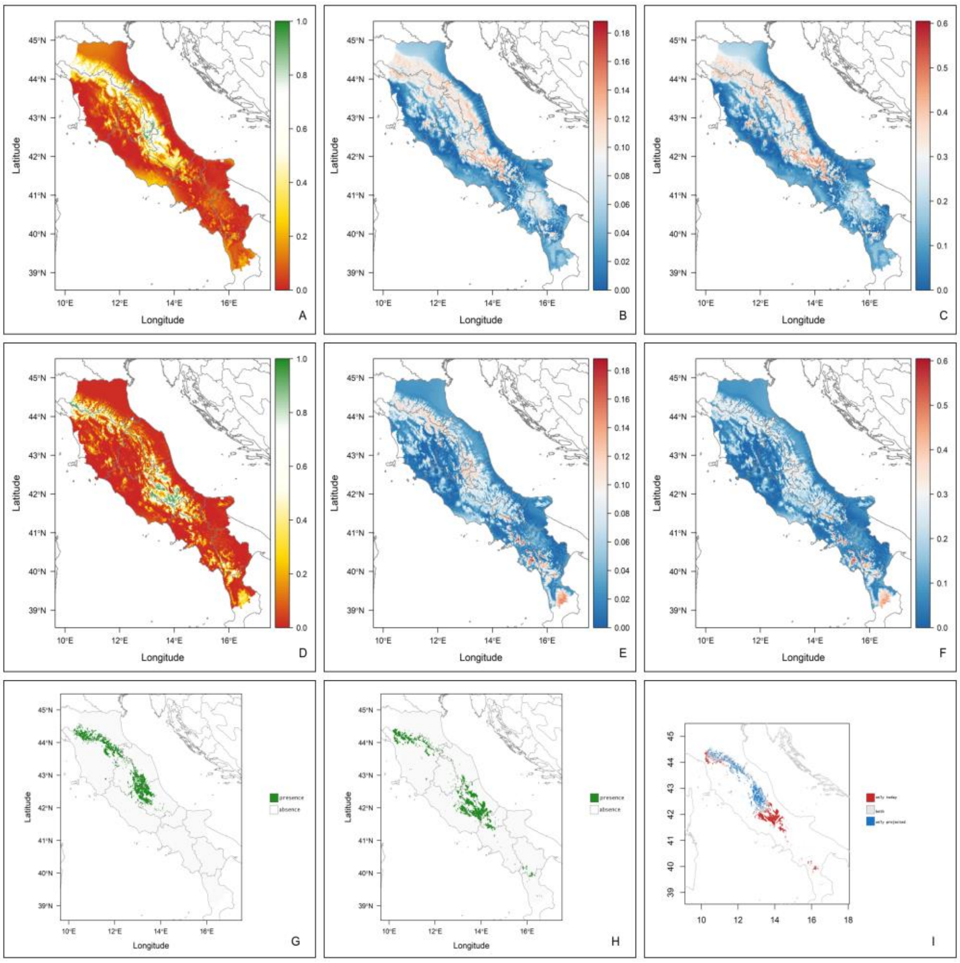

Fig. S.3.288-A) 'Projected' base output; B) 'Projected' uncertainty standard deviation; C) 'Projected' uncertainty range of values; D) 'Present' base output; E) 'Present' uncertainty standard deviation; F) 'Present' uncertainty range of values; G) 'Projected' binary distribution; H) 'Present' binary distribution; I) Range size variation.

Value	Present	Projected
AUC.train	0.943	0.976
AUC.test	0.943	0.980
AUC.diff	0.002	0.002
pROC	1.785	1.843
OR.p10	0.136	0.164
CBI	0.942	0.483
perc.stable	24.350	24.350
range.size	1.755	1.755

S.3.289 *Viola ferrarinii* Biomod2

Species: *Viola ferrarinii* Moraldo & Ricceri

Procedure: Biomod2

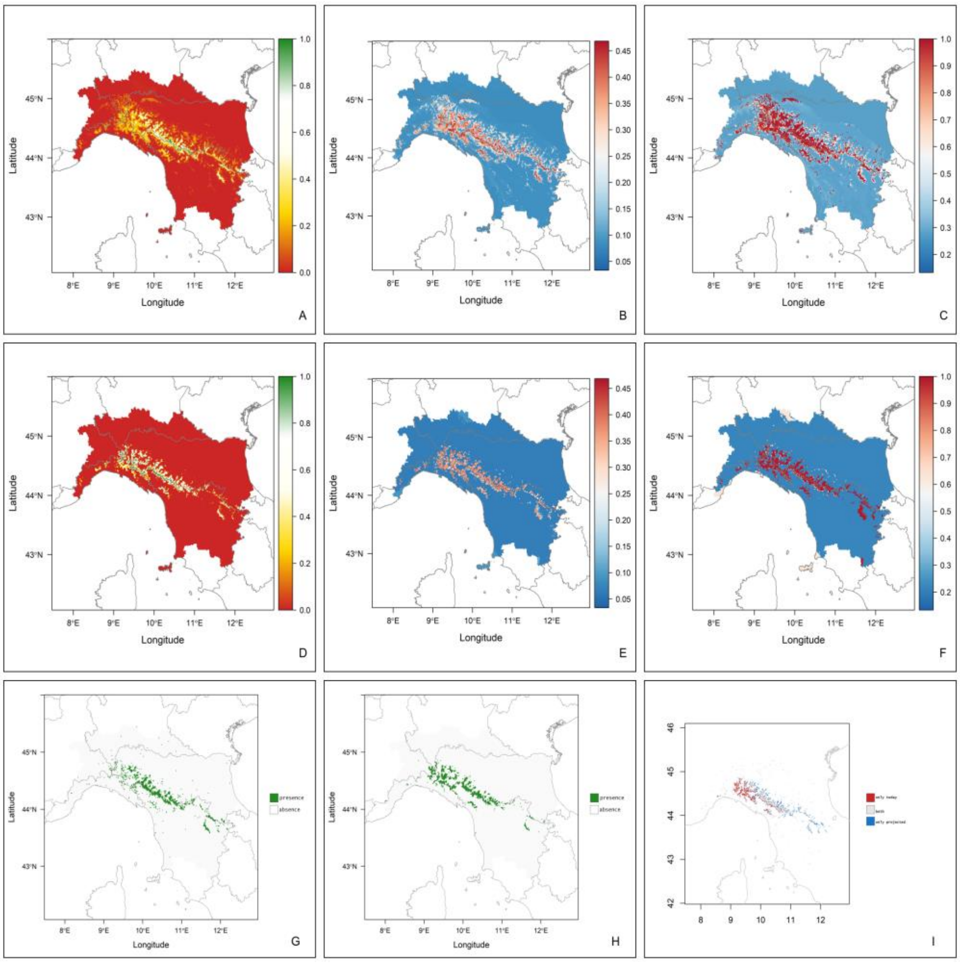

Fig. S.3.289-A) 'Projected' base output; B) 'Projected' uncertainty standard deviation; C) 'Projected' uncertainty range of values; D) 'Present' base output; E) 'Present' uncertainty standard deviation; F) 'Present' uncertainty range of values; G) 'Projected' binary distribution; H) 'Present' binary distribution; I) Range size variation.

Value	Present	Projected
AUC.train	0.996	1.000
AUC.test	0.996	0.986
AUC.diff	0.014	0.014
pROC	1.966	1.933
OR.p10	0.600	0.280
CBI	0.917	0.783
perc.stable	39.757	39.757
range.size	3.380	3.380

S.3.290 *Viola ferrarinii* Bioclim

Species: *Viola ferrarinii* Moraldo & Ricceri

Procedure: Bioclim

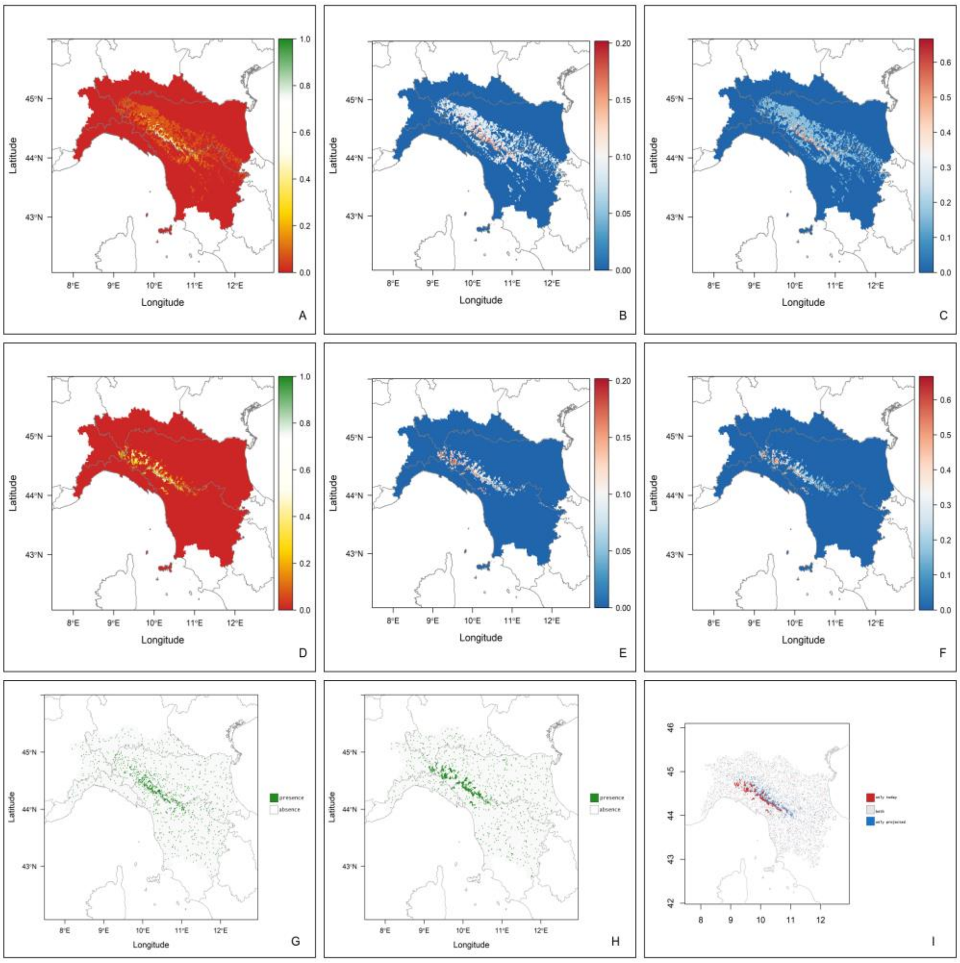

Fig. S.3.290-A) 'Projected' base output; B) 'Projected' uncertainty standard deviation; C) 'Projected' uncertainty range of values; D) 'Present' base output; E) 'Present' uncertainty standard deviation; F) 'Present' uncertainty range of values; G) 'Projected' binary distribution; H) 'Present' binary distribution; I) Range size variation.

Value	Present	Projected
AUC.train	0.785	0.956
AUC.test	0.785	0.685
AUC.diff	0.271	0.271
pROC	1.324	1.128
OR.p10	0.400	0.560
CBI	0.478	0.663
perc.stable	4.156	4.156
range.size	-3.666	-3.666

S.3.291 *Viola ferrarinii* Domain

Species: *Viola ferrarinii* Moraldo & Ricceri

Procedure: Domain

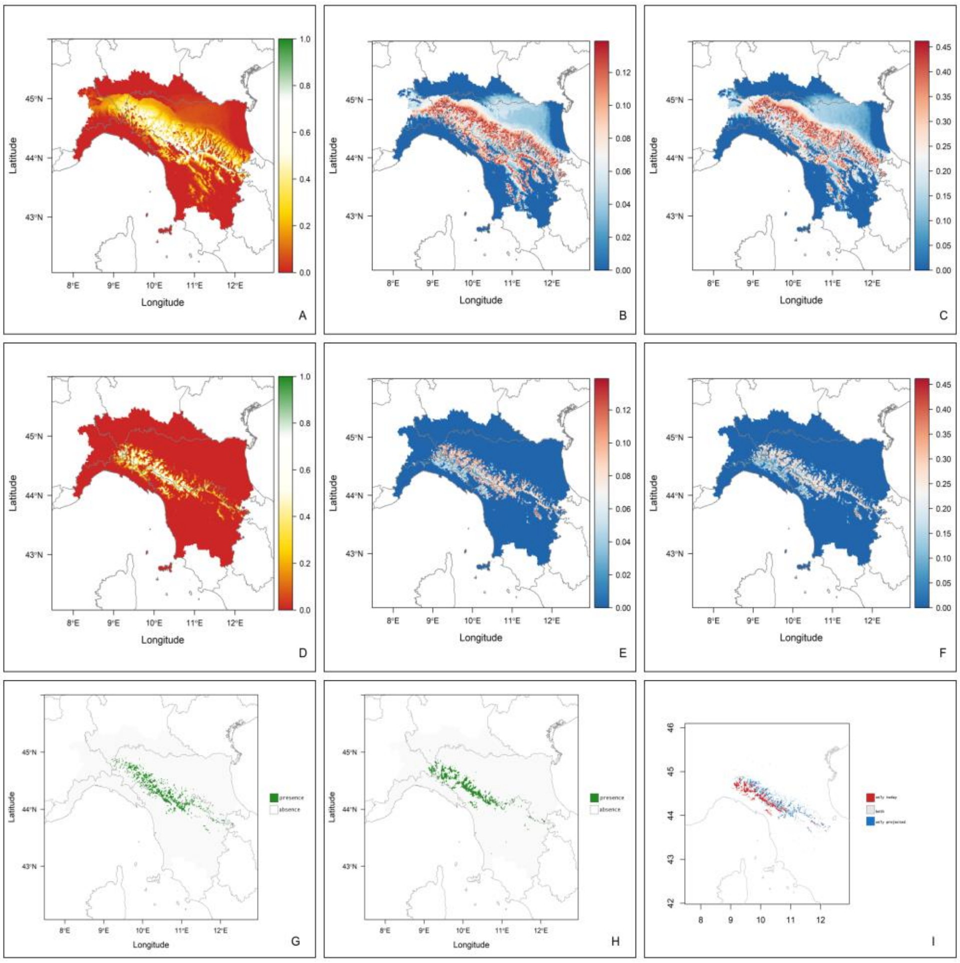

Fig. S.3.291-A) 'Projected' base output; B) 'Projected' uncertainty standard deviation; C) 'Projected' uncertainty range of values; D) 'Present' base output; E) 'Present' uncertainty standard deviation; F) 'Present' uncertainty range of values; G) 'Projected' binary distribution; H) 'Present' binary distribution; I) Range size variation.

Value	Present	Projected
AUC.train	0.980	0.945
AUC.test	0.980	0.884
AUC.diff	0.068	0.068
pROC	1.935	1.644
OR.p10	0.360	0.260
CBI	0.918	0.651
perc.stable	15.949	15.949
range.size	1.849	1.849

S.3.292 *Viola ferrarinii* Maxent

Species: *Viola ferrarinii* Moraldo & Ricceri

Procedure: Maxent

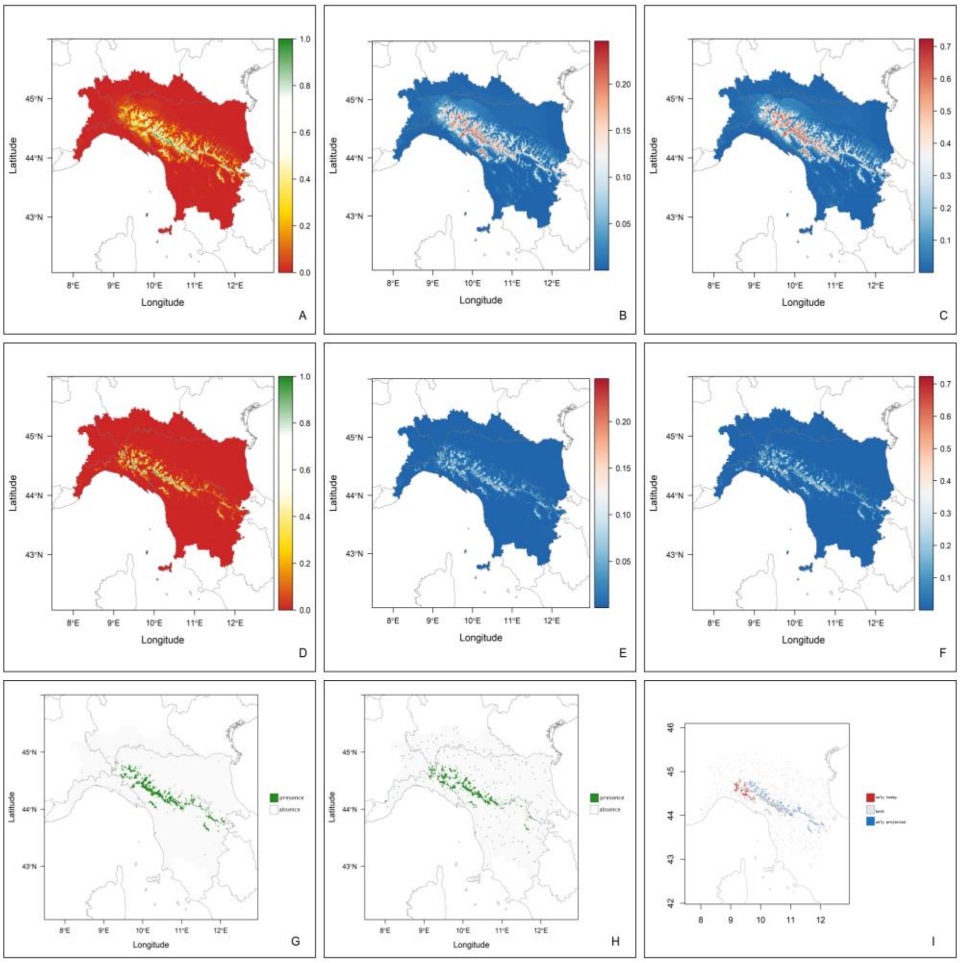

Fig. S.3.292-A) 'Projected' base output; B) 'Projected' uncertainty standard deviation; C) 'Projected' uncertainty range of values; D) 'Present' base output; E) 'Present' uncertainty standard deviation; F) 'Present' uncertainty range of values; G) 'Projected' binary distribution; H) 'Present' binary distribution; I) Range size variation.

Value	Present	Projected
AUC.train	0.988	0.976
AUC.test	0.988	0.949
AUC.diff	0.036	0.036
pROC	1.931	1.812
OR.p10	0.260	0.280
CBI	0.600	0.845
perc.stable	35.962	35.962
range.size	-0.347	-0.347

S.3.293 *Viola ferrarinii* KDE

Species: *Viola ferrarinii* Moraldo & Ricceri

Procedure: KDE

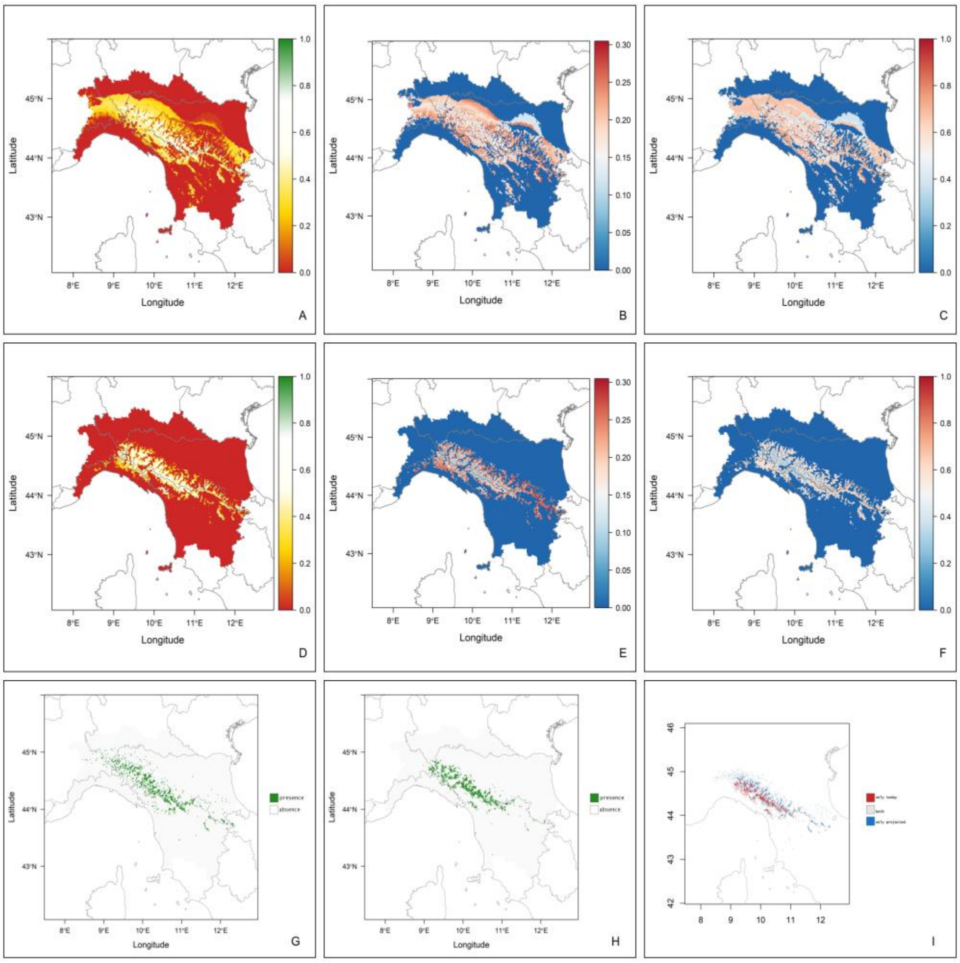

Fig. S.3.293-A) 'Projected' base output; B) 'Projected' uncertainty standard deviation; C) 'Projected' uncertainty range of values; D) 'Present' base output; E) 'Present' uncertainty standard deviation; F) 'Present' uncertainty range of values; G) 'Projected' binary distribution; H) 'Present' binary distribution; I) Range size variation.

Value	Present	Projected
AUC.train	0.897	0.943
AUC.test	0.897	0.916
AUC.diff	0.083	0.038
pROC	1.659	1.721
OR.p10	0.280	0.200
CBI	0.974	0.724
perc.stable	23.018	23.018
range.size	6.201	6.201

S.3.294 *Viola ferrarinii* CTA

Species: *Viola ferrarinii* Moraldo & Ricceri

Procedure: CTA

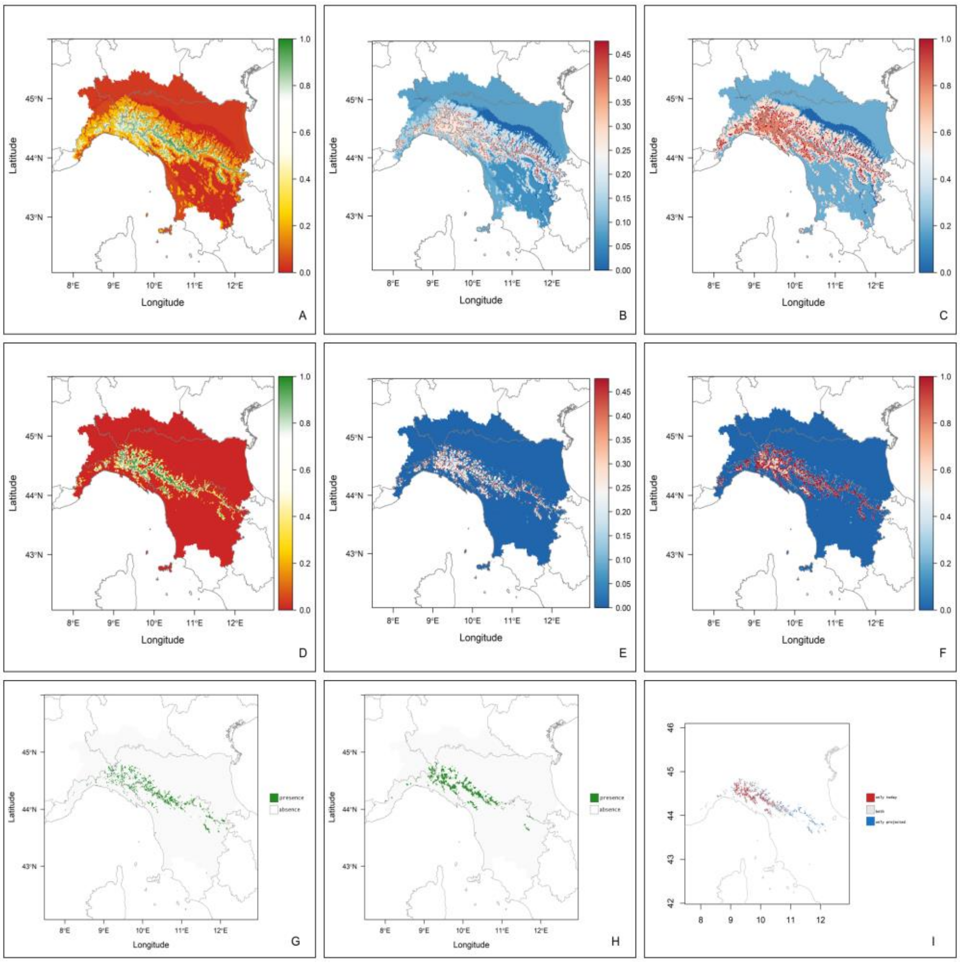

Fig. S.3.294-A) 'Projected' base output; B) 'Projected' uncertainty standard deviation; C) 'Projected' uncertainty range of values; D) 'Present' base output; E) 'Present' uncertainty standard deviation; F) 'Present' uncertainty range of values; G) 'Projected' binary distribution; H) 'Present' binary distribution; I) Range size variation.

Value	Present	Projected
AUC.train	0.934	0.971
AUC.test	0.934	0.971
AUC.diff	0.013	0.013
pROC	1.176	1.381
OR.p10	0.200	0.020
CBI	0.942	0.969
perc.stable	31.253	31.253
range.size	-5.665	-5.665

S.3.295 *Viola ferrarinii* FDA

Species: *Viola ferrarinii* Moraldo & Ricceri

Procedure: FDA

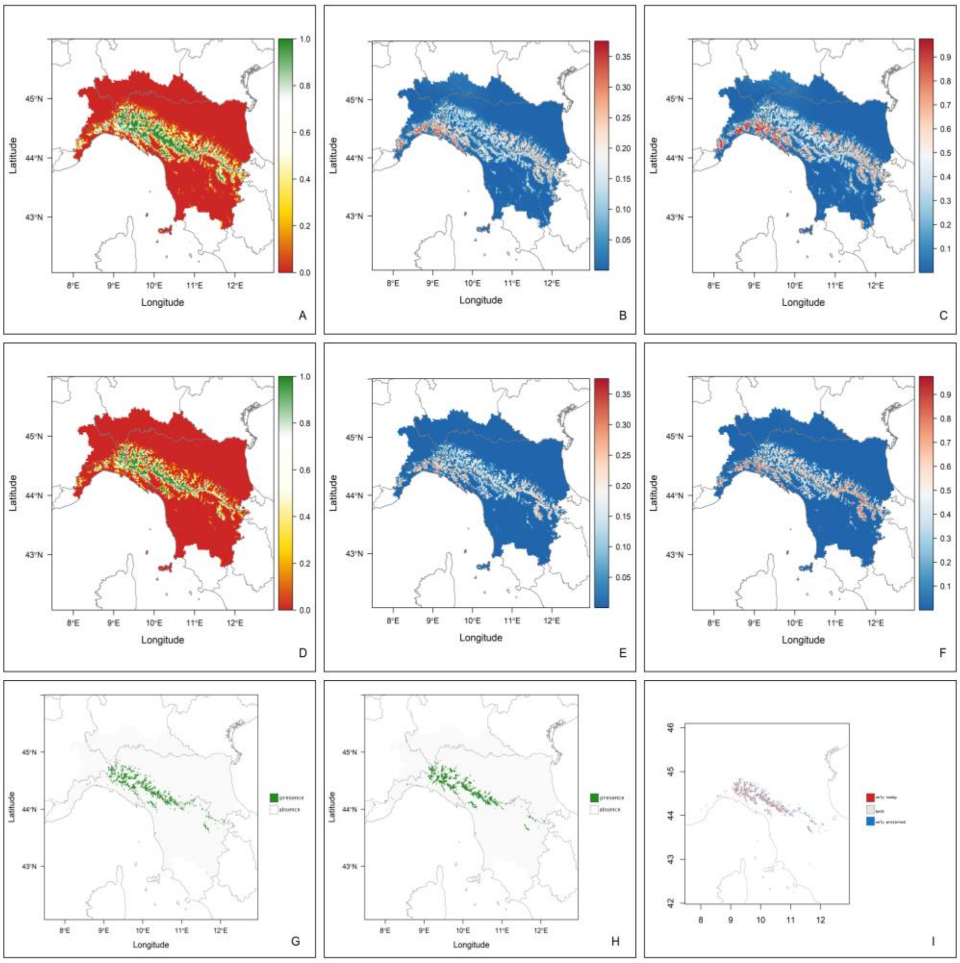

Fig. S.3.295-A) 'Projected' base output; B) 'Projected' uncertainty standard deviation; C) 'Projected' uncertainty range of values; D) 'Present' base output; E) 'Present' uncertainty standard deviation; F) 'Present' uncertainty range of values; G) 'Projected' binary distribution; H) 'Present' binary distribution; I) Range size variation.

Value	Present	Projected
AUC.train	0.976	0.981
AUC.test	0.976	0.969
AUC.diff	0.025	0.025
pROC	1.770	1.721
OR.p10	0.280	0.220
CBI	0.897	0.236
perc.stable	46.247	46.247
range.size	-1.669	-1.669

S.3.296 *Viola ferrarinii* GAM

Species: *Viola ferrarinii* Moraldo & Ricceri

Procedure: GAM

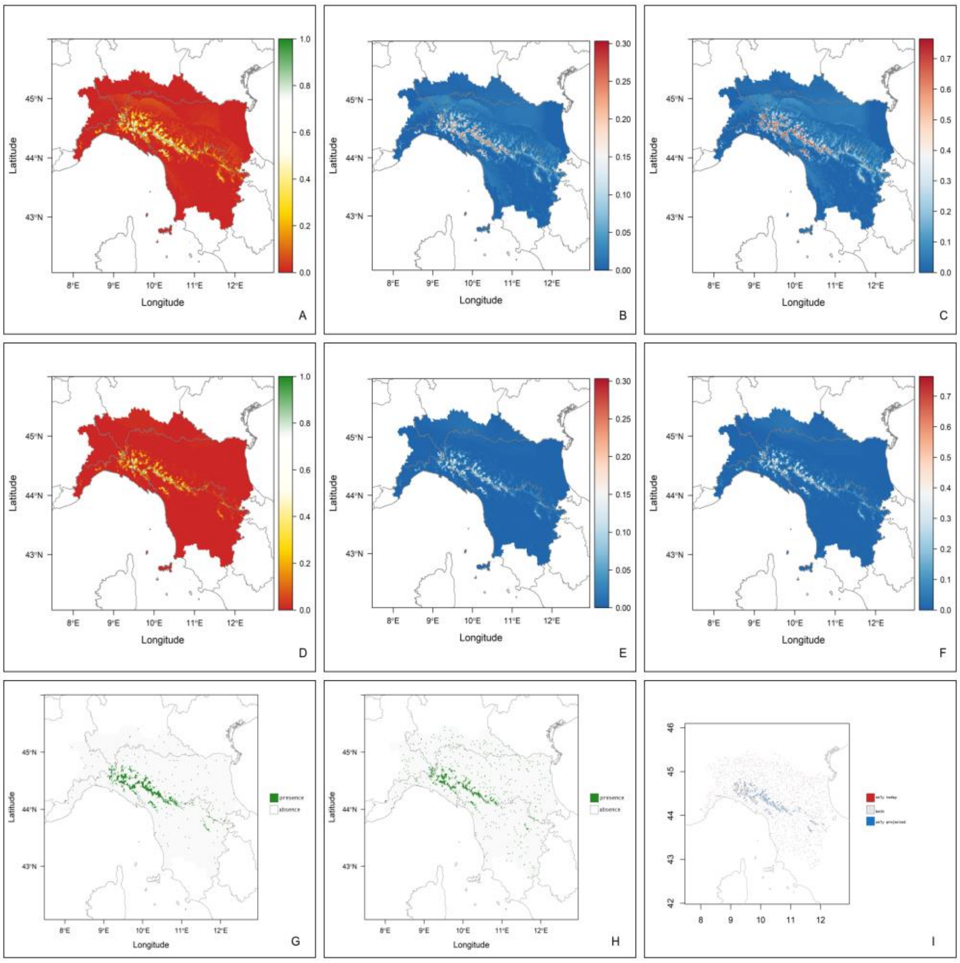

Fig. S.3.296-A) 'Projected' base output; B) 'Projected' uncertainty standard deviation; C) 'Projected' uncertainty range of values; D) 'Present' base output; E) 'Present' uncertainty standard deviation; F) 'Present' uncertainty range of values; G) 'Projected' binary distribution; H) 'Present' binary distribution; I) Range size variation.

Value	Present	Projected
AUC.train	0.987	0.967
AUC.test	0.987	0.930
AUC.diff	0.047	0.047
pROC	1.955	1.788
OR.p10	0.240	0.280
CBI	0.966	0.949
perc.stable	33.418	33.418
range.size	-6.206	-6.206

S.3.297 *Viola ferrarinii* GLM

Species: *Viola ferrarinii* Moraldo & Ricceri

Procedure: GLM

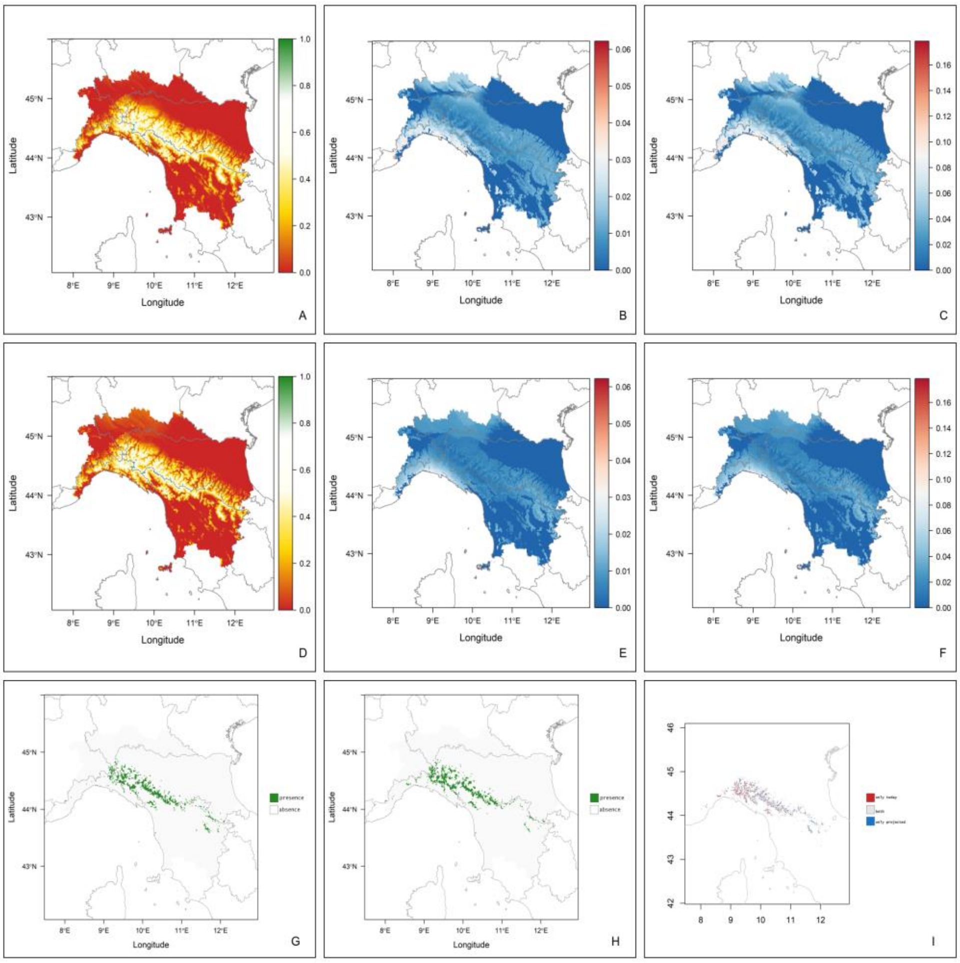

Fig. S.3.297-A) 'Projected' base output; B) 'Projected' uncertainty standard deviation; C) 'Projected' uncertainty range of values; D) 'Present' base output; E) 'Present' uncertainty standard deviation; F) 'Present' uncertainty range of values; G) 'Projected' binary distribution; H) 'Present' binary distribution; I) Range size variation.

Value	Present	Projected
AUC.train	0.985	0.959
AUC.test	0.985	0.964
AUC.diff	0.017	0.017
pROC	1.946	1.874
OR.p10	0.320	0.140
CBI	0.989	0.980
perc.stable	48.425	48.425
range.size	-2.132	-2.132

S.3.298 *Viola ferrarinii* MARS

Species: *Viola ferrarinii* Moraldo & Ricceri

Procedure: MARS

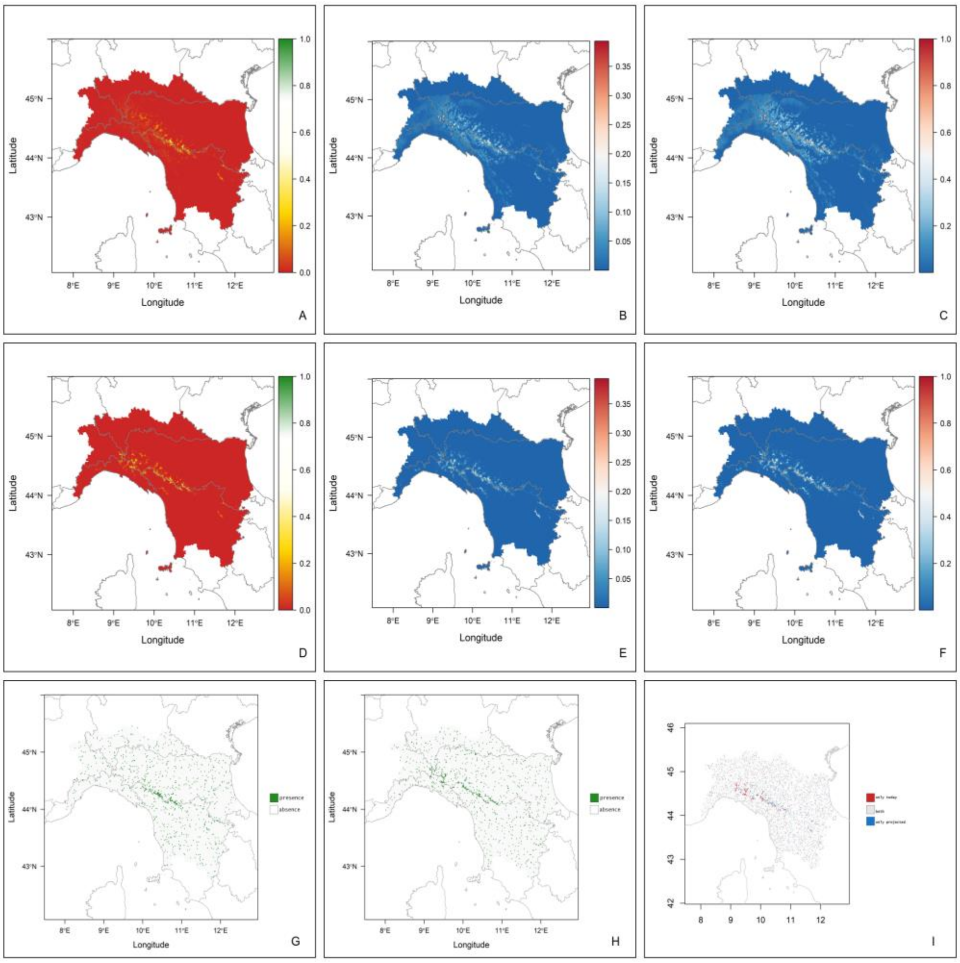

Fig. S.3.298-A) 'Projected' base output; B) 'Projected' uncertainty standard deviation; C) 'Projected' uncertainty range of values; D) 'Present' base output; E) 'Present' uncertainty standard deviation; F) 'Present' uncertainty range of values; G) 'Projected' binary distribution; H) 'Present' binary distribution; I) Range size variation.

Value	Present	Projected
AUC.train	0.899	0.952
AUC.test	0.899	0.907
AUC.diff	0.063	0.063
pROC	1.582	1.671
OR.p10	0.400	0.300
CBI	0.738	0.819
perc.stable	4.633	4.633
range.size	-2.987	-2.987

S.3.299 *Viola ferrarinii* RF

Species: *Viola ferrarinii* Moraldo & Ricceri

Procedure: RF

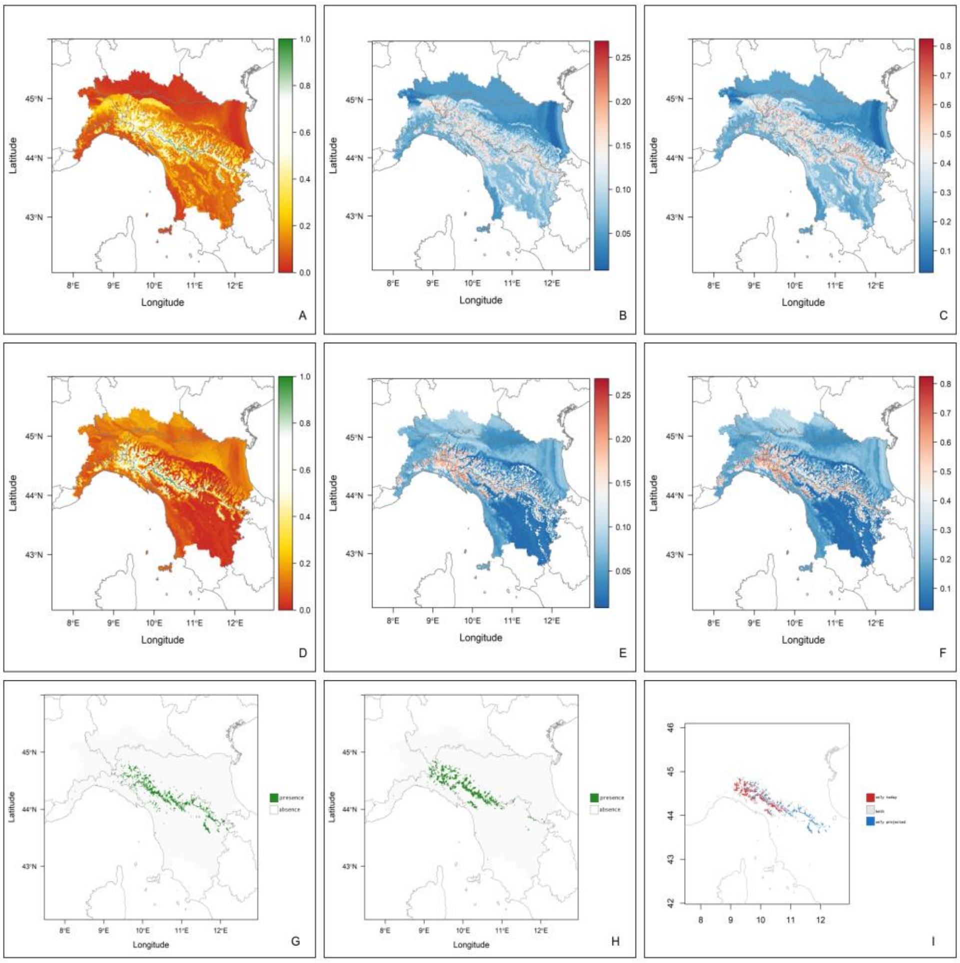

Fig. S.3.299-A) 'Projected' base output; B) 'Projected' uncertainty standard deviation; C) 'Projected' uncertainty range of values; D) 'Present' base output; E) 'Present' uncertainty standard deviation; F) 'Present' uncertainty range of values; G) 'Projected' binary distribution; H) 'Present' binary distribution; I) Range size variation.

Value	Present	Projected
AUC.train	1.000	1.000
AUC.test	1.000	0.989
AUC.diff	0.011	0.011
pROC	1.933	1.866
OR.p10	0.540	0.420
CBI	0.989	0.097
perc.stable	24.156	24.156
range.size	-0.273	-0.273

S.3.300 *Viola ferrarinii* SVM

Species: *Viola ferrarinii* Moraldo & Ricceri

Procedure: SVM

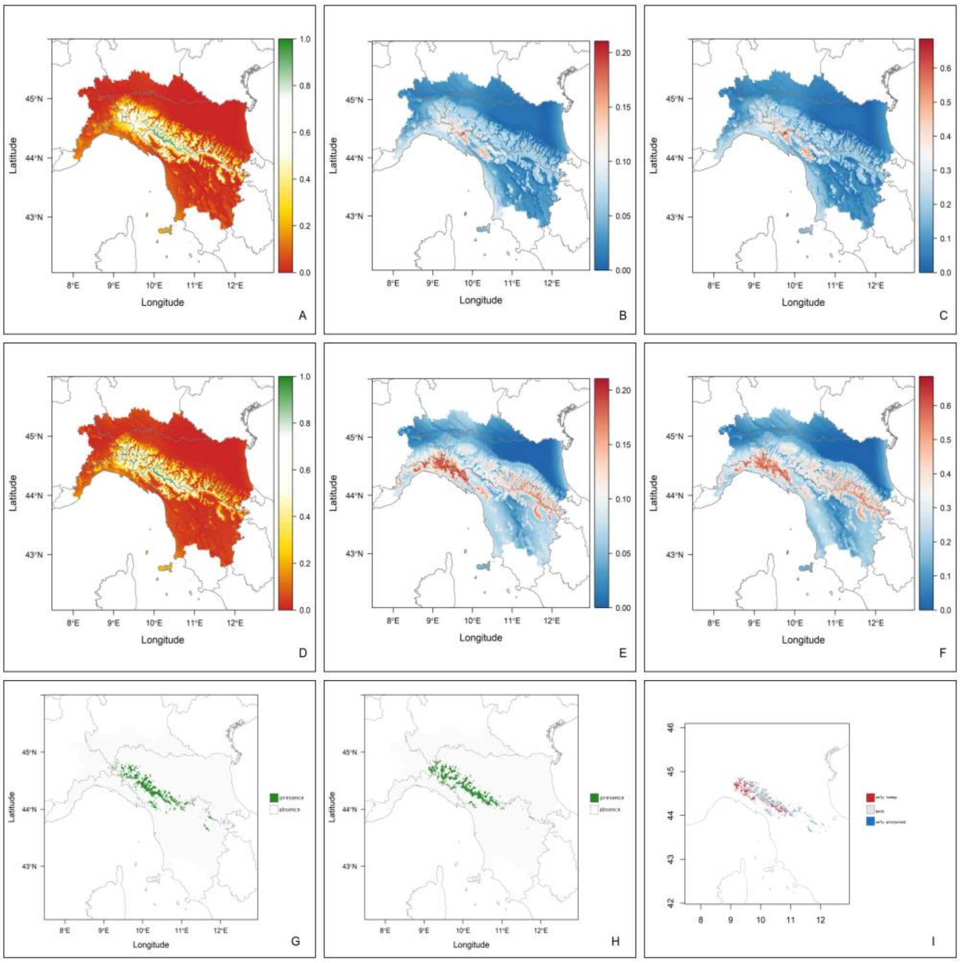

Fig. S.3.300-A) 'Projected' base output; B) 'Projected' uncertainty standard deviation; C) 'Projected' uncertainty range of values; D) 'Present' base output; E) 'Present' uncertainty standard deviation; F) 'Present' uncertainty range of values; G) 'Projected' binary distribution; H) 'Present' binary distribution; I) Range size variation.

Value	Present	Projected
AUC.train	0.984	0.988
AUC.test	0.984	0.960
AUC.diff	0.032	0.032
pROC	1.922	1.768
OR.p10	0.320	0.380
CBI	0.995	0.769
perc.stable	39.640	39.640
range.size	-3.678	-3.678
