# Supplementary material for: Assessing the Effectiveness of Correlative Ecological Niche Model Temporal Projection through Floristic Data
Source: Biology (Basel). 2022 Aug 14;11(8):1219. doi: 10.3390/biology11081219 (PMC9405103; doi:10.3390/biology11081219)
Supplement: Supplementary file 1 [file biology-11-01219-s001.zip › Dolci & Peruzzi Supplementary Material S.1-9.zip!/Supplementary material S.4.pdf]

Supplementary material S.4

Results of all modeling procedures obtained by using 3 selected bioclimatic variables. The table reports all calculations related to the comparison between distributions calculated with historical and modern data (par. 2.4) and the final summary values of distance (par. 2.6).

Table S.4.A. Results of pairwise Mann-Whitney tests for the values of Schoener's D-index (3 variables). A total of 21 cases of statistically significant differences were highlighted. X = statistically significant difference.

	**Bioclim**	**Biomod2**	**CTA**	**Domain**	**FDA**	**GAM**	**GLM**	**KDE**	**MARS**	**Maxent**	**RF**	**SVM**
**Bioclim**		-	-	X	-	-	X	-	-	-	-	-
**Biomod2**	-		-	X	X	-	X	X	-	X	-	-
**CTA**	-	-		X	X	-	X	X	-	X	-	-
**Domain**	X	X	X		-	X	-	-	X	-	X	-
**FDA**	-	X	X	-		X	-	-	X	-	-	-
**GAM**	-	-	-	X	X		X	-	-	-	-	-
**GLM**	X	X	X	-	-	X		-	X	-	-	-
**KDE**	-	X	X	-	-	-	-		X	-	-	-
**MARS**	-	-	-	X	X	-	X	X		X	-	-
**Maxent**	-	X	X	-	-	-	-	-	X		-	-
**RF**	-	-	-	X	-	-	-	-	-	-		-
**SVM**	-	-	-	-	-	-	-	-	-	-	-	

Table S.4.B. Results of pairwise Mann-Whitney tests for the values of stability (3 variables). A total of 17 cases of statistically significant differences were highlighted. X = statistically significant difference.

	**Bioclim**	**Biomod2**	**CTA**	**Domain**	**FDA**	**GAM**	**GLM**	**KDE**	**MARS**	**Maxent**	**RF**	**SVM**
**Bioclim**		-	-	-	-	-	-	-	X	-	-	-
**Biomod2**	-		-	-	-	-	-	-	X	-	-	-
**CTA**	-	-		-	-	-	-	-	X	-	-	-
**Domain**	-	-	-		-	-	-	-	X	-	-	-
**FDA**	-	-	-	-		X	-	X	X	-	-	-
**GAM**	-	-	-	-	X		X	-	X	X	-	-
**GLM**	-	-	-	-	-	X		X	X	-	-	-
**KDE**	-	-	-	-	X	-	X		-	X	-	X
**MARS**	X	X	X	X	X	X	X	-		X	X	X
**Maxent**	-	-	-	-	-	X	-	X	X		-	-
**RF**	-	-	-	-	-	-	-	-	X	-		-
**SVM**	-	-	-	-	-	-	-	X	X	-	-	

Table S.4.C. Summary values of 'Range Size variation'. The Kruskal-Wallis test showed the absence of statistically significant differences among groups (p = 0.089). Values near to 0 are to be considered optimal.

Method	Mean	Median	sd
Bioclim	− 0.211	− 0.201	1.555
Biomod2	− 0.390	− 0.186	1.740
CTA	− 0.810	− 0.657	2.230
Domain	0.585	0.172	1.869
FDA	− 0.324	− 0.172	1.371
GAM	0.842	0.427	2.041
GLM	0.409	0.354	1.301
KDE	0.271	− 0.079	2.243
MARS	− 0.543	-0.427	1.654
Maxent	0.162	0.068	1.395
RF	0.130	− 0.033	1.577
SVM	0.168	− 0.215	1.436

Table S.4.D. Summary values of 'stability'. The Kruskal-Wallis test showed statistically significant differences among groups (p = 1.026 × 10-13). Values closer to 100 are to be considered optimal.

Method	Mean	Median	sd
Bioclim	31.147	25.737	16.857
Biomod2	30.640	28.955	19.936
CTA	32.841	34.480	22.408
Domain	24.363	22.922	14.736
FDA	46.116	49.546	24.832
GAM	17.287	17.196	7.084
GLM	35.660	42.086	14.148
KDE	15.829	14.436	12.306
MARS	8.154	6.972	4.114
Maxent	39.209	40.623	17.462
RF	35.914	36.074	23.349
SVM	43.775	47.471	25.131

Table S.4.E. Summary values of 'D-index'. The Kruskal-Wallis test showed statistically significant differences among groups (p = 1.983 × 10-19). Values closer to 1 are to be considered optimal.

Method	Mean	Median	sd
Bioclim	0.705	0.756	0.175
Biomod2	0.703	0.719	0.097
CTA	0.712	0.726	0.110
Domain	0.884	0.925	0.131
FDA	0.851	0.855	0.081
GAM	0.751	0.764	0.079
GLM	0.861	0.867	0.068
KDE	0.829	0.887	0.167
MARS	0.691	0.693	0.110
Maxent	0.841	0.845	0.076
RF	0.790	0.790	0.050
SVM	0.790	0.809	0.101

Table S.4.F. Summary values of 'distance'. The Kruskal-Wallis test showed statistically significant differences among groups (p = 9.937×10-14). Values near to 0 are to be considered optimal.

Method	Mean	Median	sd
Bioclim	68.870	74.264	16.860
Biomod2	69.382	71.115	19.937
CTA	67.201	65.539	22.403
Domain	75.659	77.093	14.750
FDA	53.904	50.456	24.825
GAM	82.741	82.856	7.096
GLM	64.353	57.943	14.149
KDE	84.197	85.567	12.323
MARS	91.861	93.028	4.114
Maxent	60.808	59.377	17.459
RF	64.107	63.930	23.342
SVM	56.245	52.537	25.126

S.4.G. Summary results of CBI for present and projected potential distributions. All present distribution show better results. A total of 3 cases of statistically significant differences were highlighted. X = statistically significant difference.

Procedure	CBI 'present' (%)	CBI 'projected' (%)	Difference (%)	Significant
Bioclim	0.502	0.550	-0.048	-
Biomod2	0.928	0.804	0.124	-
CTA	0.898	0.740	0.158	-
Domain	0.831	0.743	0.088	-
FDA	0.806	0.786	0.020	-
GAM	0.867	0.796	0.071	-
GLM	0.823	0.773	0.050	-
KDE	0.883	0.682	0.201	X
MARS	0.844	0.634	0.210	X
Maxent	0.902	0.827	0.075	-
RF	0.954	0.853	0.101	X
SVM	0.897	0.834	0.063	-

S.4.H. Results of pairwise Mann-Whitney tests for the values of CBI (projected). A total of 3 cases of statistically significant differences were highlighted. X = statistically significant comparison.

	**Bioclimm**	**Biomod2**	**CTA**	**Domain**	**FDA**	**GAM**	**GLM**	**KDE**	**MARS**	**Maxent**	**RF**	**SVM**
**Bioclim**		-	-	-	-	-	-	-	-	X	X	X
**Biomod2**	-		-	-	-	-	-	-	-	-	-	-
**CTA**	-	-		-	-	-	-	-	-	-	-	-
**Domain**	-	-	-		-	-	-	-	-	-	-	-
**FDA**	-	-	-	-		-	-	-	-	-	-	-
**GAM**	-	-	-	-	-		-	-	-	-	-	-
**GLM**	-	-	-	-	-	-		-	-	-	-	-
**KDE**	-	-	-	-	-	-	-		-	-	-	-
**MARS**	-	-	-	-	-	-	-	-		-	-	-
**Maxent**	X	-	-	-	-	-	-	-	-		-	-
**RF**	X	-	-	-	-	-	-	-	-	-		-
**SVM**	X	-	-	-	-	-	-	-	-	-	-	

Table S.4.I. Complete list of results calculated for each species and algorithm by using 3 selected climatic variables.

Species	Method	Range Size Variation	Uncertainty	D-Index	Distance
Adenostyles australis	Bioclim	-2.421	39.528	0.782	60.520
Aubrieta columnae	Bioclim	-0.637	44.840	0.850	55.164
Bellevalia webbiana	Bioclim	0.569	22.762	0.672	77.241
Cardamine apennina	Bioclim	1.396	6.991	0.623	93.021
Carduus affinis	Bioclim	1.442	44.188	0.824	55.830
Crocus biflorus	Bioclim	-0.201	51.618	0.868	48.382
Crocus etruscus	Bioclim	-2.735	14.910	0.315	85.137
Crocus imperati	Bioclim	1.594	15.375	0.605	84.641
Erysimum pseudorhaeticum	Bioclim	-1.071	41.870	0.873	58.140
Galium magellense	Bioclim	-2.289	20.238	0.672	79.796
Gypsophila arrostoi	Bioclim	-1.404	8.604	0.469	91.408
Helleborus viridis	Bioclim	0.923	10.288	0.759	89.718
Klasea flavescens	Bioclim	-2.813	20.509	0.682	79.542
Koeleria splendens	Bioclim	0.786	50.687	0.737	49.320
Leontodon anomalus	Bioclim	-1.581	21.608	0.756	78.408
Linaria purpurea	Bioclim	-0.835	52.522	0.913	47.485
Ornithogalum etruscum	Bioclim	-1.225	22.271	0.842	77.739
Phyteuma ovatum	Bioclim	2.110	18.442	0.673	81.586
Polygala flavescens	Bioclim	-0.316	57.027	0.835	42.974
Ranunculus pollinensis	Bioclim	0.495	25.737	0.680	74.265
Sedum magellense	Bioclim	0.513	43.448	0.838	56.554
Siler montanum	Bioclim	0.648	57.584	0.801	42.422
Saxifraga porophylla	Bioclim	-1.676	47.868	0.791	52.159
Viola eugeniae	Bioclim	2.658	31.347	0.575	68.706
Viola ferrarinii	Bioclim	0.805	8.405	0.179	91.602
Adenostyles australis	Biomod2	-0.008	55.589	0.736	44.411
Aubrieta columnae	Biomod2	1.952	49.154	0.778	50.884
Bellevalia webbiana	Biomod2	-2.568	14.460	0.680	85.579
Cardamine apennina	Biomod2	1.328	7.459	0.616	92.551
Carduus affinis	Biomod2	0.532	60.249	0.786	39.755
Crocus biflorus	Biomod2	-0.946	16.364	0.777	83.642
Crocus etruscus	Biomod2	-0.643	14.634	0.597	85.370
Crocus imperati	Biomod2	-1.786	13.881	0.719	86.138
Erysimum pseudorhaeticum	Biomod2	-2.033	35.891	0.789	64.142
Galium magellense	Biomod2	-2.431	41.705	0.739	58.346
Gypsophila arrostoi	Biomod2	-4.699	7.729	0.681	92.391
Helleborus viridis	Biomod2	1.794	12.584	0.655	87.435
Klasea flavescens	Biomod2	0.288	1.660	0.620	98.341
Koeleria splendens	Biomod2	-0.103	45.844	0.827	54.157
Leontodon anomalus	Biomod2	-0.317	5.683	0.709	94.318
Linaria purpurea	Biomod2	-0.371	28.380	0.776	71.622
Ornithogalum etruscum	Biomod2	0.894	2.367	0.720	97.638
Phyteuma ovatum	Biomod2	-1.666	47.203	0.698	52.824
Polygala flavescens	Biomod2	-0.997	19.567	0.707	80.440
Ranunculus pollinensis	Biomod2	1.898	49.342	0.598	50.695
Sedum magellense	Biomod2	1.935	55.351	0.727	44.692

Siler montanum	Biomod2	0.186	57.070	0.821	42.931
Saxifraga porophylla	Biomod2	1.367	51.580	0.798	48.440
Viola eugeniae	Biomod2	-0.186	43.298	0.659	56.704
Viola ferrarinii	Biomod2	-3.162	28.955	0.367	71.118
Adenostyles australis	CTA	-0.258	76.247	0.722	23.756
Aubrieta columnae	CTA	-0.568	50.043	0.792	49.961
Bellevalia webbiana	CTA	-0.687	13.596	0.781	86.407
Cardamine apennina	CTA	0.791	0.030	0.605	99.974
Carduus affinis	CTA	-0.490	57.191	0.782	42.813
Crocus biflorus	CTA	-1.454	6.887	0.756	93.125
Crocus etruscus	CTA	-0.137	18.983	0.815	81.017
Crocus imperati	CTA	2.173	13.614	0.680	86.414
Erysimum pseudorhaeticum	CTA	1.606	34.480	0.721	65.540
Galium magellense	CTA	-1.184	52.725	0.670	47.291
Gypsophila arrostoi	CTA	2.634	5.191	0.734	94.846
Helleborus viridis	CTA	-0.744	7.256	0.758	92.747
Klasea flavescens	CTA	0.818	13.251	0.732	86.753
Koeleria splendens	CTA	-0.096	38.783	0.762	61.217
Leontodon anomalus	CTA	-2.302	27.761	0.836	72.276
Linaria purpurea	CTA	-0.657	19.211	0.726	80.792
Ornithogalum etruscum	CTA	-4.557	2.118	0.834	97.988
Phyteuma ovatum	CTA	-0.693	55.336	0.472	44.672
Polygala flavescens	CTA	-0.765	20.693	0.718	79.311
Ranunculus pollinensis	CTA	-0.619	60.028	0.680	39.979
Sedum magellense	CTA	-0.447	67.102	0.833	32.902
Siler montanum	CTA	-1.263	46.357	0.703	53.659
Saxifraga porophylla	CTA	-1.544	41.445	0.722	58.576
Viola eugeniae	CTA	-0.738	49.205	0.606	50.802
Viola ferrarinii	CTA	-9.076	43.492	0.359	57.236
Adenostyles australis	Domain	-0.306	33.285	0.975	66.716
Aubrieta columnae	Domain	0.000	21.087	0.935	78.913
Bellevalia webbiana	Domain	-0.353	9.665	0.908	90.335
Cardamine apennina	Domain	0.128	9.423	0.777	90.577
Carduus affinis	Domain	0.792	49.312	0.941	50.694
Crocus biflorus	Domain	-2.098	18.396	0.958	81.631
Crocus etruscus	Domain	1.511	22.922	0.697	77.094
Crocus imperati	Domain	5.938	9.549	0.830	90.646
Erysimum pseudorhaeticum	Domain	1.418	16.644	0.982	83.368
Galium magellense	Domain	0.495	59.439	0.885	40.564
Gypsophila arrostoi	Domain	0.728	7.314	0.830	92.689
Helleborus viridis	Domain	1.740	5.977	0.925	94.039
Klasea flavescens	Domain	2.003	7.348	0.848	92.674
Koeleria splendens	Domain	-0.925	26.845	0.934	73.161
Leontodon anomalus	Domain	5.424	12.946	0.906	87.223
Linaria purpurea	Domain	-0.174	22.789	0.972	77.212
Ornithogalum etruscum	Domain	-0.694	5.018	0.974	94.984
Phyteuma ovatum	Domain	-0.028	25.545	0.966	74.455
Polygala flavescens	Domain	-1.816	26.744	0.974	73.278

Ranunculus pollinensis	Domain	0.519	38.579	0.841	61.423
Sedum magellense	Domain	1.103	45.750	0.980	54.261
Siler montanum	Domain	0.060	26.709	0.892	73.291
Saxifraga porophylla	Domain	-1.860	36.821	0.932	63.207
Viola eugeniae	Domain	0.172	37.330	0.879	62.671
Viola ferrarinii	Domain	0.846	33.632	0.357	66.376
Adenostyles australis	FDA	1.134	73.982	0.910	26.043
Aubrieta columnae	FDA	-0.544	57.554	0.857	42.450
Bellevalia webbiana	FDA	-4.940	30.891	0.783	69.286
Cardamine apennina	FDA	0.707	14.278	0.832	85.725
Carduus affinis	FDA	-0.441	76.328	0.907	23.677
Crocus biflorus	FDA	0.136	26.420	0.975	73.580
Crocus etruscus	FDA	0.254	12.192	0.698	87.809
Crocus imperati	FDA	0.150	30.959	0.855	69.041
Erysimum pseudorhaeticum	FDA	-0.436	49.546	0.843	50.456
Galium magellense	FDA	-1.544	86.124	0.955	13.962
Gypsophila arrostoi	FDA	0.640	15.049	0.827	84.953
Helleborus viridis	FDA	-0.853	38.288	0.942	61.718
Klasea flavescens	FDA	-0.311	9.358	0.857	90.643
Koeleria splendens	FDA	-0.897	60.899	0.893	39.111
Leontodon anomalus	FDA	-0.237	8.863	0.836	91.137
Linaria purpurea	FDA	0.865	49.962	0.881	50.045
Ornithogalum etruscum	FDA	-2.204	22.533	0.855	77.498
Phyteuma ovatum	FDA	1.007	70.471	0.936	29.546
Polygala flavescens	FDA	-0.161	30.746	0.812	69.254
Ranunculus pollinensis	FDA	1.859	55.376	0.762	44.663
Sedum magellense	FDA	-1.444	78.413	0.942	21.635
Siler montanum	FDA	0.206	69.447	0.869	30.554
Saxifraga porophylla	FDA	-0.172	75.978	0.837	24.023
Viola eugeniae	FDA	-1.793	66.006	0.798	34.042
Viola ferrarinii	FDA	0.911	43.247	0.614	56.762
Adenostyles australis	GAM	0.004	30.990	0.754	69.010
Aubrieta columnae	GAM	2.957	17.196	0.776	82.857
Bellevalia webbiana	GAM	3.213	18.685	0.731	81.379
Cardamine apennina	GAM	-2.810	3.418	0.601	96.624
Carduus affinis	GAM	0.073	23.141	0.811	76.859
Crocus biflorus	GAM	-1.327	14.093	0.906	85.917
Crocus etruscus	GAM	2.750	26.866	0.698	73.187
Crocus imperati	GAM	6.644	12.454	0.779	87.798
Erysimum pseudorhaeticum	GAM	0.073	23.052	0.740	76.949
Galium magellense	GAM	-0.687	16.518	0.729	83.485
Gypsophila arrostoi	GAM	1.307	8.978	0.764	91.032
Helleborus viridis	GAM	1.792	19.002	0.783	81.018
Klasea flavescens	GAM	0.785	1.209	0.768	98.794
Koeleria splendens	GAM	0.778	28.000	0.759	72.005
Leontodon anomalus	GAM	0.427	15.778	0.670	84.224
Linaria purpurea	GAM	-1.654	14.464	0.824	85.552
Ornithogalum etruscum	GAM	2.169	12.000	0.881	88.027

Phyteuma ovatum	GAM	2.069	12.295	0.583	87.730
Polygala flavescens	GAM	-1.050	22.296	0.742	77.712
Ranunculus pollinensis	GAM	1.745	20.990	0.694	79.029
Sedum magellense	GAM	-0.102	20.287	0.862	79.713
Siler montanum	GAM	-0.222	19.904	0.766	80.097
Saxifraga porophylla	GAM	-0.738	17.130	0.766	82.873
Viola eugeniae	GAM	-0.795	22.421	0.769	77.583
Viola ferrarinii	GAM	3.660	10.999	0.614	89.077
Adenostyles australis	GLM	-1.452	46.015	0.907	54.005
Aubrieta columnae	GLM	0.380	43.315	0.925	56.687
Bellevalia webbiana	GLM	3.254	35.832	0.867	64.251
Cardamine apennina	GLM	-3.235	3.518	0.757	96.537
Carduus affinis	GLM	1.669	50.755	0.921	49.273
Crocus biflorus	GLM	-0.851	29.377	0.972	70.628
Crocus etruscus	GLM	1.216	16.513	0.716	83.496
Crocus imperati	GLM	0.802	26.266	0.812	73.738
Erysimum pseudorhaeticum	GLM	0.349	47.159	0.850	52.842
Galium magellense	GLM	0.286	49.266	0.988	50.735
Gypsophila arrostoi	GLM	-0.413	21.744	0.799	78.257
Helleborus viridis	GLM	0.243	31.524	0.813	68.477
Klasea flavescens	GLM	0.202	1.862	0.795	98.139
Koeleria splendens	GLM	1.008	42.650	0.869	57.359
Leontodon anomalus	GLM	-1.430	19.031	0.789	80.982
Linaria purpurea	GLM	0.940	48.066	0.903	51.943
Ornithogalum etruscum	GLM	0.354	33.211	0.914	66.790
Phyteuma ovatum	GLM	-0.392	43.330	0.864	56.671
Polygala flavescens	GLM	0.112	30.025	0.792	69.975
Ranunculus pollinensis	GLM	0.621	43.286	0.897	56.718
Sedum magellense	GLM	0.350	49.252	0.926	50.749
Siler montanum	GLM	1.812	42.086	0.886	57.943
Saxifraga porophylla	GLM	1.057	49.170	0.909	50.841
Viola eugeniae	GLM	2.255	47.775	0.863	52.274
Viola ferrarinii	GLM	1.088	40.479	0.801	59.531
Adenostyles australis	KDE	1.342	20.442	0.919	79.569
Aubrieta columnae	KDE	4.530	13.102	0.884	87.016
Bellevalia webbiana	KDE	-2.411	4.374	0.875	95.656
Cardamine apennina	KDE	-2.240	5.287	0.825	94.740
Carduus affinis	KDE	-0.893	37.876	0.589	62.132
Crocus biflorus	KDE	0.195	15.701	0.916	84.299
Crocus etruscus	KDE	-0.719	21.106	0.805	78.898
Crocus imperati	KDE	5.395	6.086	0.887	94.069
Erysimum pseudorhaeticum	KDE	0.924	4.967	0.934	95.038
Galium magellense	KDE	-0.447	54.786	0.694	45.217
Gypsophila arrostoi	KDE	-3.064	4.396	0.851	95.654
Helleborus viridis	KDE	-0.079	5.829	0.923	94.171
Klasea flavescens	KDE	1.332	3.369	0.895	96.641
Koeleria splendens	KDE	-0.960	16.352	0.873	83.654
Leontodon anomalus	KDE	-0.788	7.065	0.945	92.938

Linaria purpurea	KDE	-2.163	9.716	0.956	90.310
Ornithogalum etruscum	KDE	5.372	2.686	0.940	97.462
Phyteuma ovatum	KDE	2.594	7.549	0.925	92.487
Polygala flavescens	KDE	0.104	19.619	0.934	80.382
Ranunculus pollinensis	KDE	-1.083	25.477	0.524	74.532
Sedum magellense	KDE	0.636	24.632	0.891	75.370
Siler montanum	KDE	0.639	14.436	0.852	85.567
Saxifraga porophylla	KDE	-0.784	21.653	0.920	78.351
Viola eugeniae	KDE	0.710	23.606	0.735	76.398
Viola ferrarinii	KDE	-1.359	25.621	0.223	74.395
Adenostyles australis	MARS	-0.206	16.903	0.766	83.098
Aubrieta columnae	MARS	0.381	6.433	0.713	93.568
Bellevalia webbiana	MARS	1.692	5.830	0.600	94.186
Cardamine apennina	MARS	-1.361	1.587	0.444	98.424
Carduus affinis	MARS	-0.427	12.480	0.705	87.521
Crocus biflorus	MARS	-0.109	6.972	0.847	93.028
Crocus etruscus	MARS	-0.114	6.463	0.565	93.538
Crocus imperati	MARS	-1.620	3.607	0.648	96.407
Erysimum pseudorhaeticum	MARS	0.905	6.514	0.677	93.491
Galium magellense	MARS	-0.561	8.876	0.693	91.126
Gypsophila arrostoi	MARS	-1.294	5.605	0.631	94.405
Helleborus viridis	MARS	0.209	10.610	0.850	89.391
Klasea flavescens	MARS	-1.150	3.787	0.864	96.220
Koeleria splendens	MARS	-0.999	12.685	0.702	87.321
Leontodon anomalus	MARS	0.345	2.728	0.480	97.274
Linaria purpurea	MARS	0.181	8.737	0.767	91.264
Ornithogalum etruscum	MARS	-0.591	4.011	0.794	95.991
Phyteuma ovatum	MARS	0.148	6.767	0.624	93.234
Polygala flavescens	MARS	0.867	17.025	0.686	82.980
Ranunculus pollinensis	MARS	-0.843	6.268	0.564	93.737
Sedum magellense	MARS	-2.283	14.506	0.810	85.525
Siler montanum	MARS	-1.445	10.105	0.755	89.907
Saxifraga porophylla	MARS	-2.744	10.023	0.779	90.019
Viola eugeniae	MARS	3.195	7.561	0.682	92.495
Viola ferrarinii	MARS	-5.755	7.778	0.620	92.402
Adenostyles australis	Maxent	0.285	65.162	0.891	34.839
Aubrieta columnae	Maxent	1.293	68.083	0.854	31.944
Bellevalia webbiana	Maxent	0.637	40.202	0.823	59.802
Cardamine apennina	Maxent	2.954	6.812	0.694	93.235
Carduus affinis	Maxent	-0.321	53.662	0.854	46.340
Crocus biflorus	Maxent	0.063	14.610	0.917	85.390
Crocus etruscus	Maxent	2.077	23.171	0.856	76.857
Crocus imperati	Maxent	-1.059	36.960	0.828	63.049
Erysimum pseudorhaeticum	Maxent	-1.063	53.992	0.861	46.021
Galium magellense	Maxent	2.114	23.262	0.960	76.767
Gypsophila arrostoi	Maxent	-0.351	27.618	0.841	72.383
Helleborus viridis	Maxent	-1.302	29.192	0.887	70.820
Klasea flavescens	Maxent	-0.078	4.579	0.818	95.421

Koeleria splendens	Maxent	0.353	50.160	0.824	49.842
Leontodon anomalus	Maxent	1.128	27.109	0.824	72.900
Linaria purpurea	Maxent	-0.064	45.825	0.925	54.175
Ornithogalum etruscum	Maxent	0.280	47.294	0.970	52.707
Phyteuma ovatum	Maxent	-3.005	46.654	0.718	53.431
Polygala flavescens	Maxent	-0.746	27.875	0.824	72.129
Ranunculus pollinensis	Maxent	1.258	46.995	0.750	53.020
Sedum magellense	Maxent	2.364	57.887	0.951	42.179
Siler montanum	Maxent	-2.141	61.406	0.845	38.654
Saxifraga porophylla	Maxent	0.068	52.999	0.863	47.001
Viola eugeniae	Maxent	0.140	40.623	0.772	59.378
Viola ferrarinii	Maxent	-0.825	28.083	0.679	71.923
Adenostyles australis	RF	-0.329	65.780	0.775	34.222
Aubrieta columnae	RF	1.691	41.818	0.830	58.206
Bellevalia webbiana	RF	-2.207	5.908	0.810	94.118
Cardamine apennina	RF	-0.187	4.568	0.765	95.433
Carduus affinis	RF	1.566	64.296	0.796	35.739
Crocus biflorus	RF	0.277	14.035	0.841	85.966
Crocus etruscus	RF	-0.135	15.043	0.839	84.957
Crocus imperati	RF	-3.191	9.452	0.742	90.605
Erysimum pseudorhaeticum	RF	-0.757	36.074	0.768	63.931
Galium magellense	RF	1.755	56.895	0.713	43.142
Gypsophila arrostoi	RF	-0.033	11.513	0.782	88.488
Helleborus viridis	RF	0.797	16.630	0.831	83.374
Klasea flavescens	RF	0.778	15.156	0.848	84.848
Koeleria splendens	RF	0.029	39.141	0.806	60.860
Leontodon anomalus	RF	3.672	16.487	0.838	83.594
Linaria purpurea	RF	-0.419	31.298	0.790	68.703
Ornithogalum etruscum	RF	0.005	7.024	0.874	92.976
Phyteuma ovatum	RF	0.233	67.746	0.714	32.256
Polygala flavescens	RF	-0.213	22.861	0.778	77.140
Ranunculus pollinensis	RF	-0.770	61.983	0.765	38.025
Sedum magellense	RF	-0.743	64.582	0.862	35.427
Siler montanum	RF	1.360	57.352	0.769	42.670
Saxifraga porophylla	RF	-1.823	45.766	0.814	54.265
Viola eugeniae	RF	-1.580	67.665	0.706	32.375
Viola ferrarinii	RF	3.473	58.767	0.697	41.380
Adenostyles australis	SVM	-0.253	70.976	0.833	29.026
Aubrieta columnae	SVM	-0.694	61.412	0.926	38.595
Bellevalia webbiana	SVM	1.732	19.617	0.724	80.402
Cardamine apennina	SVM	0.780	10.568	0.586	89.436
Carduus affinis	SVM	-1.159	80.764	0.952	19.271
Crocus biflorus	SVM	-0.799	24.247	0.817	75.757
Crocus etruscus	SVM	-0.222	24.572	0.818	75.428
Crocus imperati	SVM	-0.393	26.675	0.789	73.326
Erysimum pseudorhaeticum	SVM	0.043	49.546	0.787	50.454
Galium magellense	SVM	-0.215	72.955	0.910	27.046
Gypsophila arrostoi	SVM	2.549	14.682	0.701	85.357

Helleborus viridis	SVM	-0.170	27.394	0.670	72.607
Klasea flavescens	SVM	-1.733	0.882	0.711	99.134
Koeleria splendens	SVM	-1.132	48.711	0.709	51.303
Leontodon anomalus	SVM	3.962	19.519	0.816	80.579
Linaria purpurea	SVM	0.008	46.575	0.747	53.426
Ornithogalum etruscum	SVM	-1.422	5.394	0.720	94.617
Phyteuma ovatum	SVM	2.117	74.177	0.875	25.910
Polygala flavescens	SVM	-0.602	31.021	0.558	68.983
Ranunculus pollinensis	SVM	1.640	56.919	0.809	43.112
Sedum magellense	SVM	-1.869	71.390	0.894	28.671
Siler montanum	SVM	-0.539	65.215	0.756	34.790
Saxifraga porophylla	SVM	1.748	73.458	0.873	26.600
Viola eugeniae	SVM	-0.103	70.233	0.878	29.767
Viola ferrarinii	SVM	0.930	47.471	0.876	52.537
