# Supplementary material for: Assessing the Effectiveness of Correlative Ecological Niche Model Temporal Projection through Floristic Data
Source: Biology (Basel). 2022 Aug 14;11(8):1219. doi: 10.3390/biology11081219 (PMC9405103; doi:10.3390/biology11081219)
Supplement: Supplementary file 1 [file biology-11-01219-s001.zip › Dolci & Peruzzi Supplementary Material S.1-9.zip!/Supplementary material S.5.pdf]

Supplementary material S.5

Results of all modeling procedures obtained by using the first three axes of a principal component analysis (PCA) of the 35 bioclimatic variables. The table reports all calculations related to the comparison between distributions calculated with historical and modern data (par. 2.4) and the final summary values of distance (par. 2.6).

Table S.5.A. Results of pairwise Mann-Whitney tests for the values of Schoener's D-index (first 3 axes of PCA [35 variables]). A total of 19 cases of statistically significant differences were highlighted. X = statistically significant difference.

	Bioclim	Biomod2	CTA	Domain	FDA	GAM	GLM	KDE	MARS	Maxent	RF	SVM
**Bioclim**		-	-	X	X	-	X	X	-	X	X	-
**Biomod2**	-		-	X	X	-	X	-	-	-	-	-
**CTA**	-	-		X	X	-	X	-	-	-	-	-
**Domain**	X	X	X		-	-	-	-	X	-	-	-
**FDA**	X	X	X	-		X	-	-	X	-	-	-
**GAM**	-	-	-	-	X		X	-	-	-	-	-
**GLM**	X	X	X	-	-	X		-	X	-	-	-
**KDE**	X	-	-	-	-	-	-		X	-	-	-
**MARS**	-	-	-	X	X	-	X	X		-	X	-
**Maxent**	X	-	-	-	-	-	-	-	-		-	-
**RF**	X	-	-	-	-	-	-	-	X	-		-
**SVM**	-	-	-	-	-	-	-	-	-	-	-	

Table S.5.B. Results of pairwise Mann-Whitney tests for the values of stability (first 3 axes of PCA [35 variables]). A total of 10 cases of statistically significant differences were highlighted. X = statistically significant difference.

	Bioclim	Biomod2	CTA	Domain	FDA	GAM	GLM	KDE	MARS	Maxent	RF	SVM
**Bioclim**		-	-	-	-	-	-	-	X	-	-	-
**Biomod2**	-		-	-	-	-	-	-	X	-	-	-
**CTA**	-	-		-	-	-	-	-	X	-	-	-
**Domain**	-	-	-		-	-	-	-	X	-	-	-
**FDA**	-	-	-	-		-	-	X	X	-	-	-
**GAM**	-	-	-	-	-		-	-	X	-	-	-
**GLM**	-	-	-	-	-	-		-	X	-	-	-
**KDE**	-	-	-	-	X	-	-		-	-	-	-
**MARS**	X	X	X	X	X	X	X	-		X	X	-
**Maxent**	-	-	-	-	-	-	-	-	X		-	-
**RF**	-	-	-	-	-	-	-	-	X	-		-
**SVM**	-	-	-	-	-	-	-	-	-	-	-	

Table S.5.C. Summary values of 'Range Size variation' . The Kruskal-Wallis test showed the absence of statistically significant differences among groups (p = 0.258). Values near to 0 are to be considered optimal.

Method	Mean	Median	sd
Bioclim	0.663	0.326	1.980
Biomod2	0.216	0.085	1.776
CTA	− 0.516	− 0.289	1.982
Domain	− 0.004	0.011	3.042
FDA	0.227	0.193	1.177
GAM	0.221	0.323	2.141
GLM	− 0.222	0.348	1.769
KDE	0.383	− 0.051	2.503
MARS	− 0.873	− 0.409	2.316
Maxent	− 0.164	− 0.019	1.916
RF	0.100	− 0.273	1.952
SVM	− 0.488	− 0.406	2.115

Table S.5.D. Summary values of 'stability'. The Kruskal-Wallis test showed statistically significant differences among groups (p = 1.74 × 10-10). Values closer to 100 are to be considered optimal.

Method	Mean	Median	sd
Bioclim	24.536	22.456	13.892
Biomod2	31.623	35.587	20.475
CTA	34.413	38.277	21.620
Domain	22.626	21.390	13.366
FDA	45.007	46.247	23.333
GAM	20.770	21.573	12.323
GLM	36.739	39.553	15.576
KDE	17.963	17.056	12.114
MARS	7.636	6.976	3.893
Maxent	34.708	29.302	20.509
RF	31.404	24.156	18.929
SVM	36.049	32.786	25.996

Table S.5.E. Summary values of 'D-index'. The Kruskal-Wallis test showed statistically significant differences among groups (p = 2.47 × 10-17). Values closer to 1 are to be considered optimal.

Method	Mean	Median	sd
Biomod2	0.714	0.712	0.077
Bioclim	0.619	0.664	0.163
Domain	0.835	0.875	0.142
Maxent	0.778	0.813	0.097
KDE	0.786	0.817	0.123
CTA	0.734	0.747	0.091
FDA	0.833	0.839	0.081
GAM	0.741	0.755	0.086
GLM	0.863	0.883	0.080
MARS	0.649	0.662	0.121
RF	0.783	0.797	0.078
SVM	0.754	0.788	0.135

Table S.5.F. Summary values of 'distance'. The Kruskal-Wallis test showed statistically significant differences among groups (p = 1.73×10-10). Values near to 0 are to be considered optimal.

Method	Mean	Median	sd
Bioclim	75.489	77.544	13.908
Biomod2	68.399	64.441	20.478
CTA	65.614	61.723	21.634
Domain	77.425	78.611	13.406
FDA	55.008	53.779	23.327
GAM	79.259	78.428	12.315
GLM	63.287	60.448	15.569
KDE	82.073	82.944	12.126
MARS	92.395	93.068	3.917
Maxent	65.317	70.708	20.517
RF	68.618	75.845	18.946
SVM	63.981	67.215	26.011

Table S.5.G. Summary results of CBI for present and projected potential distributions calculated for all procedures (first 3 axes of PCA [35 variables]). All present distribution show better results. A total of 6 cases of statistically significant differences were highlighted. X = statistically significant difference.

Procedure	CBI 'present'	CBI 'projected'	Difference	Significant
Bioclim	0.467	0.533	-0.066	-
Biomod2	0.941	0.708	0.233	X
CTA	0.917	0.704	0.213	X
Domain	0.814	0.637	0.177	-
FDA	0.759	0.646	0.113	-
GAM	0.887	0.706	0.181	-
GLM	0.806	0.634	0.172	-
KDE	0.881	0.736	0.145	X
MARS	0.832	0.578	0.254	X
Maxent	0.902	0.726	0.176	-
RF	0.962	0.783	0.179	X
SVM	0.938	0.764	0.174	X

Table S.5.H. Complete list of results calculated for each species and algorithm by using first 3 axes of PCA [35 variables]

Species	Method	Range Size Variation	Uncertainty	D-Index	Distance
Adenostyles australis	Bioclim	1.519	32.243	0.676	67.775
Aubrieta columnae	Bioclim	-0.335	45.362	0.753	54.640
Bellevalia webbiana	Bioclim	1.601	14.024	0.552	85.992
Cardamine apennina	Bioclim	1.143	4.352	0.501	95.657
Carduus affinis	Bioclim	-0.199	29.572	0.664	70.429
Crocus biflorus	Bioclim	0.326	36.299	0.762	63.702
Crocus etruscus	Bioclim	2.096	9.074	0.427	90.952
Crocus imperati	Bioclim	4.505	12.617	0.672	87.500
Erysimum pseudorhaeticum	Bioclim	-0.204	26.451	0.527	73.551
Galium magellense	Bioclim	2.038	11.003	0.668	89.021

Gypsophila arrostoi	Bioclim	4.515	19.467	0.614	80.661
Helleborus viridis	Bioclim	2.404	16.510	0.636	83.525
Klasea flavescens	Bioclim	1.795	21.112	0.740	78.908
Koeleria splendens	Bioclim	-0.668	48.405	0.778	51.600
Leontodon anomalus	Bioclim	-1.727	21.973	0.441	78.048
Linaria purpurea	Bioclim	-0.335	49.965	0.849	50.036
Ornithogalum etruscum	Bioclim	0.040	22.456	0.796	77.544
Phyteuma ovatum	Bioclim	4.036	4.198	0.329	95.890
Polygala flavescens	Bioclim	0.101	31.881	0.613	68.120
Ranunculus pollinensis	Bioclim	0.442	29.534	0.724	70.468
Sedum magellense	Bioclim	-0.696	28.318	0.622	71.686
Siler montanum	Bioclim	-0.003	40.742	0.716	59.259
Saxifraga porophylla	Bioclim	-2.498	40.338	0.754	59.714
Viola eugeniae	Bioclim	0.335	13.336	0.530	86.666
Viola ferrarinii	Bioclim	-3.666	4.156	0.131	95.918
Adenostyles australis	Biomod2	-0.925	73.613	0.837	26.403
Aubrieta columnae	Biomod2	0.085	41.889	0.712	58.112
Bellevalia webbiana	Biomod2	-1.517	6.801	0.670	93.212
Cardamine apennina	Biomod2	1.755	3.663	0.612	96.354
Carduus affinis	Biomod2	-0.149	54.811	0.723	45.190
Crocus biflorus	Biomod2	-0.726	12.952	0.720	87.051
Crocus etruscus	Biomod2	0.659	33.691	0.718	66.313
Crocus imperati	Biomod2	-1.681	25.230	0.772	74.790
Erysimum pseudorhaeticum	Biomod2	-0.606	7.561	0.596	92.442
Galium magellense	Biomod2	0.716	39.368	0.793	60.636
Gypsophila arrostoi	Biomod2	-3.133	4.768	0.710	95.284
Helleborus viridis	Biomod2	2.631	20.809	0.681	79.236
Klasea flavescens	Biomod2	2.107	6.424	0.694	93.600
Koeleria splendens	Biomod2	0.386	44.655	0.773	55.346
Leontodon anomalus	Biomod2	-0.853	19.886	0.752	80.119
Linaria purpurea	Biomod2	1.386	48.590	0.877	51.428
Ornithogalum etruscum	Biomod2	3.810	4.461	0.672	95.615
Phyteuma ovatum	Biomod2	-1.591	43.304	0.640	56.719
Polygala flavescens	Biomod2	0.265	12.217	0.599	87.784
Ranunculus pollinensis	Biomod2	2.339	52.133	0.802	47.925
Sedum magellense	Biomod2	-1.574	47.207	0.707	52.818
Siler montanum	Biomod2	-0.690	57.824	0.758	42.182
Saxifraga porophylla	Biomod2	1.220	53.373	0.797	46.644
Viola eugeniae	Biomod2	-1.882	35.587	0.678	64.441
Viola ferrarinii	Biomod2	3.380	39.757	0.567	60.339
Adenostyles australis	CTA	0.445	63.908	0.739	36.096
Aubrieta columnae	CTA	-1.754	36.414	0.880	63.610
Bellevalia webbiana	CTA	-4.356	1.776	0.649	98.321
Cardamine apennina	CTA	4.505	2.077	0.639	98.027
Carduus affinis	CTA	-0.364	60.373	0.747	39.629
Crocus biflorus	CTA	-0.365	12.490	0.732	87.512
Crocus etruscus	CTA	-0.750	44.582	0.771	55.423
Crocus imperati	CTA	-2.800	23.298	0.793	76.753

Erysimum pseudorhaeticum	CTA	-0.530	18.855	0.699	81.147
Galium magellense	CTA	0.812	74.603	0.807	25.411
Gypsophila arrostoi	CTA	-1.841	9.191	0.794	90.828
Helleborus viridis	CTA	-2.395	15.480	0.649	84.555
Klasea flavescens	CTA	-0.289	11.111	0.820	88.890
Koeleria splendens	CTA	0.025	43.770	0.717	56.231
Leontodon anomalus	CTA	1.429	49.399	0.783	50.622
Linaria purpurea	CTA	-0.252	38.277	0.792	61.724
Ornithogalum etruscum	CTA	1.072	7.436	0.802	92.571
Phyteuma ovatum	CTA	-0.226	39.102	0.503	60.901
Polygala flavescens	CTA	0.538	13.508	0.624	86.494
Ranunculus pollinensis	CTA	-1.276	52.497	0.792	47.521
Sedum magellense	CTA	-0.711	64.328	0.729	35.681
Siler montanum	CTA	0.448	41.361	0.721	58.641
Saxifraga porophylla	CTA	1.259	52.140	0.807	47.877
Viola eugeniae	CTA	0.135	53.087	0.824	46.913
Viola ferrarinii	CTA	-5.665	31.253	0.533	68.981
Adenostyles australis	Domain	-0.573	34.761	0.961	65.242
Aubrieta columnae	Domain	1.463	29.006	0.831	71.009
Bellevalia webbiana	Domain	8.781	7.254	0.805	93.161
Cardamine apennina	Domain	0.011	5.829	0.841	94.171
Carduus affinis	Domain	0.626	31.407	0.874	68.596
Crocus biflorus	Domain	-0.702	14.296	0.930	85.707
Crocus etruscus	Domain	0.405	36.687	0.647	63.315
Crocus imperati	Domain	-0.939	8.131	0.908	91.874
Erysimum pseudorhaeticum	Domain	0.443	13.595	0.901	86.406
Galium magellense	Domain	0.698	53.471	0.583	46.537
Gypsophila arrostoi	Domain	-4.746	11.632	0.875	88.496
Helleborus viridis	Domain	0.141	8.350	0.860	91.651
Klasea flavescens	Domain	0.858	5.856	0.946	94.148
Koeleria splendens	Domain	-0.845	31.401	0.960	68.604
Leontodon anomalus	Domain	-5.312	19.235	0.660	80.940
Linaria purpurea	Domain	-0.085	21.390	0.955	78.611
Ornithogalum etruscum	Domain	5.655	5.806	0.932	94.364
Phyteuma ovatum	Domain	-6.898	15.484	0.825	84.797
Polygala flavescens	Domain	-0.549	25.824	0.940	74.178
Ranunculus pollinensis	Domain	-0.032	39.373	0.919	60.627
Sedum magellense	Domain	1.247	35.568	0.825	64.444
Siler montanum	Domain	0.578	31.933	0.884	68.070
Saxifraga porophylla	Domain	-1.347	41.948	0.791	58.068
Viola eugeniae	Domain	-0.822	21.462	0.884	78.542
Viola ferrarinii	Domain	1.849	15.949	0.349	84.074
Adenostyles australis	FDA	-0.072	77.281	0.914	22.719
Aubrieta columnae	FDA	-0.194	51.959	0.839	48.042
Bellevalia webbiana	FDA	0.606	10.813	0.820	89.189
Cardamine apennina	FDA	1.851	0.011	0.655	100.007
Carduus affinis	FDA	1.373	74.901	0.809	25.137
Crocus biflorus	FDA	-1.290	22.254	0.905	77.756

Crocus etruscus	FDA	-1.526	46.177	0.900	53.845
Crocus imperati	FDA	1.611	33.228	0.856	66.791
Erysimum pseudorhaeticum	FDA	0.791	39.635	0.818	60.370
Galium magellense	FDA	-0.326	73.430	0.851	26.573
Gypsophila arrostoi	FDA	-1.081	27.096	0.873	72.912
Helleborus viridis	FDA	0.365	47.255	0.758	52.746
Klasea flavescens	FDA	-0.859	8.875	0.813	91.130
Koeleria splendens	FDA	1.180	71.110	0.925	28.914
Leontodon anomalus	FDA	-1.130	19.733	0.859	80.275
Linaria purpurea	FDA	0.417	60.467	0.868	39.535
Ornithogalum etruscum	FDA	1.041	31.547	0.880	68.461
Phyteuma ovatum	FDA	0.674	33.850	0.581	66.155
Polygala flavescens	FDA	-0.158	21.380	0.795	78.621
Ranunculus pollinensis	FDA	3.042	65.906	0.883	34.230
Sedum magellense	FDA	1.623	61.850	0.837	38.185
Siler montanum	FDA	-0.218	69.418	0.794	30.584
Saxifraga porophylla	FDA	0.193	75.196	0.961	24.804
Viola eugeniae	FDA	-0.560	55.551	0.813	44.453
Viola ferrarinii	FDA	-1.669	46.247	0.810	53.780
Adenostyles australis	GAM	-1.356	53.476	0.785	46.544
Aubrieta columnae	GAM	0.323	25.642	0.825	74.359
Bellevalia webbiana	GAM	0.834	1.450	0.731	98.554
Cardamine apennina	GAM	2.998	0.277	0.517	99.769
Carduus affinis	GAM	0.666	31.501	0.697	68.503
Crocus biflorus	GAM	2.139	15.069	0.777	84.958
Crocus etruscus	GAM	2.918	37.857	0.776	62.212
Crocus imperati	GAM	-3.793	7.500	0.698	92.578
Erysimum pseudorhaeticum	GAM	0.068	12.546	0.755	87.454
Galium magellense	GAM	-0.529	18.322	0.701	81.681
Gypsophila arrostoi	GAM	1.060	11.064	0.776	88.942
Helleborus viridis	GAM	-1.138	30.624	0.730	69.386
Klasea flavescens	GAM	4.014	5.281	0.791	94.805
Koeleria splendens	GAM	0.532	33.622	0.775	66.380
Leontodon anomalus	GAM	1.285	19.404	0.587	80.607
Linaria purpurea	GAM	1.003	21.997	0.868	78.009
Ornithogalum etruscum	GAM	0.083	7.583	0.773	92.418
Phyteuma ovatum	GAM	-0.073	24.172	0.590	75.829
Polygala flavescens	GAM	0.075	13.464	0.652	86.537
Ranunculus pollinensis	GAM	-1.581	24.884	0.857	75.133
Sedum magellense	GAM	2.874	27.118	0.849	72.938
Siler montanum	GAM	-0.573	18.255	0.748	81.748
Saxifraga porophylla	GAM	-0.436	21.573	0.821	78.428
Viola eugeniae	GAM	0.329	23.156	0.717	76.845
Viola ferrarinii	GAM	-6.206	33.418	0.731	66.871
Adenostyles australis	GLM	-0.154	50.421	0.944	49.580
Aubrieta columnae	GLM	2.024	44.439	0.931	55.598
Bellevalia webbiana	GLM	-2.624	15.089	0.856	84.952
Cardamine apennina	GLM	0.440	0.013	0.654	99.989

Carduus affinis	GLM	-0.032	53.027	0.876	46.974
Crocus biflorus	GLM	0.888	9.735	0.883	90.269
Crocus etruscus	GLM	0.385	38.430	0.911	61.571
Crocus imperati	GLM	-3.056	33.619	0.807	66.452
Erysimum pseudorhaeticum	GLM	-3.024	37.931	0.761	62.143
Galium magellense	GLM	-4.077	50.813	0.929	49.355
Gypsophila arrostoi	GLM	-0.150	31.887	0.874	68.113
Helleborus viridis	GLM	-0.265	38.371	0.804	61.630
Klasea flavescens	GLM	0.504	17.150	0.901	82.852
Koeleria splendens	GLM	-0.411	53.371	0.922	46.631
Leontodon anomalus	GLM	0.383	39.553	0.901	60.449
Linaria purpurea	GLM	0.348	51.271	0.916	48.730
Ornithogalum etruscum	GLM	1.630	13.641	0.838	86.375
Phyteuma ovatum	GLM	0.823	44.171	0.797	55.835
Polygala flavescens	GLM	-0.211	19.039	0.757	80.961
Ranunculus pollinensis	GLM	2.343	45.058	0.945	54.992
Sedum magellense	GLM	2.368	48.680	0.878	51.375
Siler montanum	GLM	0.864	48.684	0.913	51.324
Saxifraga porophylla	GLM	0.477	52.295	0.969	47.707
Viola eugeniae	GLM	-2.881	33.357	0.701	66.706
Viola ferrarinii	GLM	-2.132	48.425	0.899	51.620
Adenostyles australis	KDE	-1.532	25.031	0.793	74.985
Aubrieta columnae	KDE	-0.322	17.056	0.806	82.944
Bellevalia webbiana	KDE	1.867	4.205	0.780	95.813
Cardamine apennina	KDE	-6.235	3.120	0.822	97.081
Carduus affinis	KDE	0.753	28.838	0.611	71.167
Crocus biflorus	KDE	0.259	14.607	0.903	85.394
Crocus etruscus	KDE	-0.977	35.057	0.640	64.951
Crocus imperati	KDE	-1.609	3.933	0.853	96.081
Erysimum pseudorhaeticum	KDE	-0.769	11.409	0.896	88.594
Galium magellense	KDE	1.069	53.903	0.767	46.110
Gypsophila arrostoi	KDE	6.857	9.416	0.830	90.843
Helleborus viridis	KDE	1.637	4.479	0.817	95.535
Klasea flavescens	KDE	0.088	4.362	0.889	95.638
Koeleria splendens	KDE	0.767	22.996	0.858	77.008
Leontodon anomalus	KDE	-0.051	11.311	0.787	88.690
Linaria purpurea	KDE	-0.447	12.441	0.929	87.560
Ornithogalum etruscum	KDE	0.338	2.875	0.928	97.125
Phyteuma ovatum	KDE	-0.238	17.027	0.470	82.975
Polygala flavescens	KDE	-0.385	23.506	0.856	76.495
Ranunculus pollinensis	KDE	3.360	21.657	0.824	78.415
Sedum magellense	KDE	-0.762	28.726	0.724	71.278
Siler montanum	KDE	1.012	17.736	0.839	82.271
Saxifraga porophylla	KDE	-0.240	29.277	0.762	70.724
Viola eugeniae	KDE	-1.060	23.086	0.794	76.922
Viola ferrarinii	KDE	6.201	23.018	0.464	77.233
Adenostyles australis	MARS	-0.771	17.553	0.741	82.451
Aubrieta columnae	MARS	1.839	4.998	0.704	95.020

Bellevalia webbiana	MARS	-5.859	2.206	0.502	97.971
Cardamine apennina	MARS	3.123	1.914	0.427	98.137
Carduus affinis	MARS	-1.587	12.182	0.661	87.833
Crocus biflorus	MARS	-0.360	10.615	0.718	89.386
Crocus etruscus	MARS	-2.477	6.262	0.603	93.771
Crocus imperati	MARS	-4.689	2.859	0.395	97.256
Erysimum pseudorhaeticum	MARS	0.206	6.852	0.734	93.148
Galium magellense	MARS	-0.409	9.368	0.738	90.633
Gypsophila arrostoi	MARS	2.168	5.999	0.855	94.026
Helleborus viridis	MARS	-0.121	6.075	0.522	93.927
Klasea flavescens	MARS	2.413	4.478	0.716	95.553
Koeleria splendens	MARS	0.327	14.243	0.662	85.758
Leontodon anomalus	MARS	-3.631	7.599	0.468	92.474
Linaria purpurea	MARS	-1.847	8.791	0.794	91.228
Ornithogalum etruscum	MARS	-3.558	2.037	0.659	98.028
Phyteuma ovatum	MARS	-2.639	9.050	0.538	90.989
Polygala flavescens	MARS	-0.196	11.326	0.606	88.675
Ranunculus pollinensis	MARS	-2.855	6.976	0.750	93.068
Sedum magellense	MARS	1.480	11.345	0.758	88.668
Siler montanum	MARS	-0.675	9.035	0.761	90.967
Saxifraga porophylla	MARS	1.452	8.943	0.732	91.069
Viola eugeniae	MARS	-0.171	5.550	0.625	94.451
Viola ferrarinii	MARS	-2.987	4.633	0.565	95.415
Adenostyles australis	Maxent	-0.938	67.134	0.853	32.880
Aubrieta columnae	Maxent	-1.660	67.541	0.840	32.501
Bellevalia webbiana	Maxent	2.177	6.143	0.826	93.883
Cardamine apennina	Maxent	-2.551	2.437	0.632	97.597
Carduus affinis	Maxent	-0.633	42.630	0.659	57.375
Crocus biflorus	Maxent	-0.927	20.068	0.839	79.938
Crocus etruscus	Maxent	-1.781	53.352	0.925	46.682
Crocus imperati	Maxent	-5.235	21.733	0.833	78.442
Erysimum pseudorhaeticum	Maxent	0.266	22.272	0.689	77.729
Galium magellense	Maxent	1.218	20.058	0.854	79.951
Gypsophila arrostoi	Maxent	-4.361	10.415	0.792	89.692
Helleborus viridis	Maxent	1.222	15.326	0.681	84.684
Klasea flavescens	Maxent	1.090	15.820	0.852	84.187
Koeleria splendens	Maxent	-0.129	54.696	0.804	45.304
Leontodon anomalus	Maxent	3.102	24.676	0.720	75.388
Linaria purpurea	Maxent	-0.521	60.846	0.878	39.158
Ornithogalum etruscum	Maxent	-0.328	16.134	0.874	83.867
Phyteuma ovatum	Maxent	1.154	29.302	0.541	70.709
Polygala flavescens	Maxent	-0.002	17.970	0.675	82.030
Ranunculus pollinensis	Maxent	1.065	56.595	0.860	43.418
Sedum magellense	Maxent	1.328	48.013	0.813	52.005
Siler montanum	Maxent	0.712	62.285	0.772	37.723
Saxifraga porophylla	Maxent	-0.019	49.852	0.829	50.148
Viola eugeniae	Maxent	1.990	46.433	0.787	53.604
Viola ferrarinii	Maxent	-0.347	35.962	0.626	64.040

Adenostyles australis	RF	0.126	59.793	0.797	40.207
Aubrieta columnae	RF	-0.915	42.542	0.829	57.466
Bellevalia webbiana	RF	8.342	4.629	0.759	95.735
Cardamine apennina	RF	-1.056	1.975	0.756	98.031
Carduus affinis	RF	0.837	43.397	0.725	56.610
Crocus biflorus	RF	-0.560	12.386	0.813	87.616
Crocus etruscus	RF	-1.682	56.558	0.920	43.475
Crocus imperati	RF	0.240	20.594	0.835	79.407
Erysimum pseudorhaeticum	RF	-0.986	22.845	0.744	77.161
Galium magellense	RF	-0.212	59.724	0.869	40.277
Gypsophila arrostoi	RF	0.084	23.344	0.831	76.657
Helleborus viridis	RF	-1.617	16.732	0.799	83.284
Klasea flavescens	RF	0.607	9.754	0.807	90.248
Koeleria splendens	RF	0.279	44.455	0.765	55.547
Leontodon anomalus	RF	-0.314	17.957	0.860	82.044
Linaria purpurea	RF	-0.962	41.078	0.856	58.930
Ornithogalum etruscum	RF	0.942	6.500	0.818	93.505
Phyteuma ovatum	RF	-1.123	19.845	0.522	80.164
Polygala flavescens	RF	-0.864	16.868	0.703	83.137
Ranunculus pollinensis	RF	2.132	54.299	0.822	45.751
Sedum magellense	RF	1.310	50.359	0.762	49.659
Siler montanum	RF	-0.315	49.953	0.779	50.049
Saxifraga porophylla	RF	-1.334	54.143	0.790	45.877
Viola eugeniae	RF	-0.178	31.216	0.729	68.785
Viola ferrarinii	RF	-0.273	24.156	0.675	75.846
Adenostyles australis	SVM	0.230	67.314	0.885	32.687
Aubrieta columnae	SVM	-2.097	58.538	0.843	41.516
Bellevalia webbiana	SVM	-3.736	3.625	0.712	96.447
Cardamine apennina	SVM	2.255	0.012	0.360	100.015
Carduus affinis	SVM	-0.707	62.552	0.821	37.455
Crocus biflorus	SVM	-0.230	20.134	0.729	79.866
Crocus etruscus	SVM	-0.406	56.712	0.920	43.290
Crocus imperati	SVM	-2.619	10.516	0.766	89.523
Erysimum pseudorhaeticum	SVM	-1.565	14.334	0.625	85.681
Galium magellense	SVM	0.110	67.726	0.915	32.274
Gypsophila arrostoi	SVM	6.857	16.087	0.839	84.192
Helleborus viridis	SVM	-1.588	10.446	0.671	89.569
Klasea flavescens	SVM	-2.584	1.014	0.682	99.020
Koeleria splendens	SVM	-0.393	59.728	0.848	40.274
Leontodon anomalus	SVM	-1.187	31.342	0.788	68.668
Linaria purpurea	SVM	-0.564	68.963	0.765	31.043
Ornithogalum etruscum	SVM	-1.306	0.186	0.673	99.824
Phyteuma ovatum	SVM	0.291	32.786	0.639	67.216
Polygala flavescens	SVM	0.328	8.646	0.497	91.356
Ranunculus pollinensis	SVM	0.055	53.968	0.853	46.033
Sedum magellense	SVM	-1.645	62.302	0.829	37.734
Siler montanum	SVM	-0.355	61.464	0.825	38.538
Saxifraga porophylla	SVM	0.576	68.835	0.864	31.171

Viola eugeniae	SVM	1.755	24.350	0.627	75.671
Viola ferrarinii	SVM	-3.678	39.640	0.870	60.472
