# Supplementary material for: Assessing the Effectiveness of Correlative Ecological Niche Model Temporal Projection through Floristic Data
Source: Biology (Basel). 2022 Aug 14;11(8):1219. doi: 10.3390/biology11081219 (PMC9405103; doi:10.3390/biology11081219)
Supplement: Supplementary file 1 [file biology-11-01219-s001.zip › Dolci & Peruzzi Supplementary Material S.1-9.zip!/Supplementary material S.6.pdf]

Supplementary material S.6

Complete list of evaluations metrics acquired in all modeling procedures based on the use of 3 environmental variables. Table S.6.A reports all results for procedures based on historical data. Table S.6.B reports all results for procedures based on modern data.

Table S.6.A. Complete list of evaluation metrics calculated for all procedures based on 3 environmental variables and historical data.

Species	Method	AUC_train (past)	AUC_test (past)	AUC_diff (past)	pROC (past)	OR_p10 (past)	CBI (projected)
Adenostyles australis	Bioclim	0.847943	0.795122	0.063054	1.153581	0.205556	0.787
Aubrieta columnae	Bioclim	0.908588	0.817908	0.098256	1.394919	0.2	0.842
Bellevalia webbiana	Bioclim	0.865932	0.735867	0.136382	1.307329	0.25	-0.033
Cardamine apennina	Bioclim	0.773338	0.642363	0.141579	1.116892	0.2375	0.2
Carduus affinis	Bioclim	0.876989	0.841864	0.041534	1.303178	0.161111	0.908
Crocus biflorus	Bioclim	0.6603	0.628066	0.036017	1.091979	0.172727	0.674
Crocus etruscus	Bioclim	0.97291	0.60695	0.36822	1.224506	0.75	0.387
Crocus imperati	Bioclim	0.722371	0.563892	0.160306	1.155784	0.283333	0.606
Erysimum pseudorhaeticum	Bioclim	0.643823	0.628757	0.03267	1.084454	0.185714	0.831
Galium magellense	Bioclim	0.974285	0.913746	0.061421	1.486335	0.125	0.675
Gypsophila arrostoi	Bioclim	0.794434	0.680527	0.12832	1.225736	0.310952	0.623
Helleborus viridis	Bioclim	0.632308	0.595427	0.067286	1.11686	0.181818	0.807
Klasea flavescens	Bioclim	0.746393	0.702258	0.084397	1.274727	0.2	0.568
Koeleria splendens	Bioclim	0.774741	0.751748	0.030072	1.154758	0.128	0.379
Leontodon anomalus	Bioclim	0.841877	0.80291	0.073438	1.390384	0.14	0.061
Linaria purpurea	Bioclim	0.580873	0.538411	0.058653	1.058376	0.17747	0.17
Ornithogalum etruscum	Bioclim	0.665817	0.563867	0.10195	1.151128	0.31	-0.401
Phyteuma ovatum	Bioclim	0.798789	0.747039	0.064628	1.169661	0.155556	0.679
Polygala flavescens	Bioclim	0.64674	0.618209	0.034644	1.102835	0.125581	0.532
Ranunculus pollinensis	Bioclim	0.937155	0.803806	0.133349	1.229256	0.255556	0.921
Saxifraga porophylla	Bioclim	0.882575	0.837346	0.048278	1.30568	0.238462	0.922
Sedum magellense	Bioclim	0.923132	0.870242	0.055439	1.335794	0.133333	0.733
Siler montanum	Bioclim	0.786931	0.723517	0.063414	1.142704	0.205556	0.449
Viola eugeniae	Bioclim	0.936548	0.878509	0.062342	1.424738	0.190909	0.973
Viola ferrarinii	Bioclim	0.947246	0.82274	0.124506	1.465618	0.2	0.459
Adenostyles australis	Biomod2	0.981765	0.873054	0.108711	1.374859	0.416667	0.976
Aubrieta columnae	Biomod2	0.992817	0.945462	0.047355	1.782578	0.383333	0.876
Bellevalia webbiana	Biomod2	0.999041	0.936143	0.062898	1.78221	0.7	0.809
Cardamine apennina	Biomod2	0.99467	0.900563	0.094108	1.597682	0.5875	0.467
Carduus affinis	Biomod2	0.979706	0.967545	0.012161	1.768366	0.166667	0.901
Crocus biflorus	Biomod2	0.984558	0.714061	0.270498	1.194884	0.840909	0.624
Crocus etruscus	Biomod2	1	0.9052	0.0948	1.860766	0.5	0.661
Crocus imperati	Biomod2	0.996541	0.74681	0.249731	1.430185	1	0.82
Erysimum pseudorhaeticum	Biomod2	0.977942	0.83759	0.140352	1.366703	0.621429	0.919
Galium magellense	Biomod2	0.997561	0.994019	0.003543	1.947504	0.266667	0.958
Gypsophila arrostoi	Biomod2	0.999831	0.742892	0.256939	1.399159	1	0.544
Helleborus viridis	Biomod2	0.994982	0.753425	0.241557	1.315845	0.936364	0.828
Klasea flavescens	Biomod2	0.999449	0.7274	0.272049	1.351563	0.85	0.356

Koeleria splendens	Biomod2	0.955466	0.890105	0.065361	1.391254	0.28	0.984
Leontodon anomalus	Biomod2	0.99903	0.859938	0.139091	1.556804	0.78	0.843
Linaria purpurea	Biomod2	0.96804	0.722197	0.245843	1.141902	0.64585	0.995
Ornithogalum etruscum	Biomod2	0.99982	0.587247	0.412573	1.241164	1	0.329
Phyteuma ovatum	Biomod2	0.994599	0.803697	0.190902	1.347531	0.711111	0.871
Polygala flavescens	Biomod2	0.934099	0.771537	0.162562	1.208432	0.390698	0.786
Ranunculus pollinensis	Biomod2	0.99232	0.975422	0.016898	1.852697	0.277778	0.971
Saxifraga porophylla	Biomod2	0.99066	0.952436	0.038224	1.689713	0.376923	0.965
Sedum magellense	Biomod2	0.994862	0.909333	0.085529	1.605722	0.625	0.805
Siler montanum	Biomod2	0.964338	0.845757	0.118581	1.441177	0.5	0.944
Viola eugeniae	Biomod2	0.991473	0.971706	0.019767	1.855072	0.445455	0.981
Viola ferrarinii	Biomod2	0.999194	0.987967	0.011228	1.893571	0.56	0.893
Adenostyles australis	CTA	0.957339	0.866348	0.090991	1.144764	0.222222	0.987
Aubrieta columnae	CTA	0.972672	0.936404	0.041464	1.254782	0.116667	0.914
Bellevalia webbiana	CTA	0.948214	0.87375	0.074464	1.384558	0.25	0.615
Cardamine apennina	CTA	0.906964	0.783929	0.124821	1.163675	0.2	0.419
Carduus affinis	CTA	0.964901	0.917639	0.047262	1.343613	0.166667	0.786
Crocus biflorus	CTA	0.923221	0.714134	0.209086	1.03197	0.4	0.328
Crocus etruscus	CTA	0.921429	0.871429	0.05	1	0.1	0.22
Crocus imperati	CTA	0.91125	0.8225	0.092857	1.28329	0.233333	0.698
Erysimum pseudorhaeticum	CTA	0.923082	0.781508	0.141575	1.114417	0.357143	0.916
Galium magellense	CTA	0.9915	0.977991	0.013865	1.201566	0.033333	0.411
Gypsophila arrostoi	CTA	0.916602	0.763634	0.154925	1.179739	0.290952	0.86
Helleborus viridis	CTA	0.885556	0.749747	0.13597	1.132674	0.272727	0.794
Klasea flavescens	CTA	0.859683	0.692857	0.179921	1.110317	0.316667	0.604
Koeleria splendens	CTA	0.939344	0.838265	0.101427	1.12828	0.284	0.965
Leontodon anomalus	CTA	0.924359	0.811667	0.122179	1.271068	0.34	0.901
Linaria purpurea	CTA	0.916709	0.767722	0.148987	1.074592	0.343676	0.983
Ornithogalum etruscum	CTA	0.86525	0.724035	0.141215	1.126593	0.306667	0.07
Phyteuma ovatum	CTA	0.951906	0.881183	0.072499	1.249114	0.233333	0.94
Polygala flavescens	CTA	0.883227	0.762839	0.120388	1.077862	0.206977	0.798
Ranunculus pollinensis	CTA	0.979828	0.921839	0.058391	1.374904	0.133333	0.957
Saxifraga porophylla	CTA	0.951163	0.901521	0.051354	1.315781	0.176923	0.892
Sedum magellense	CTA	0.970179	0.899896	0.072009	1.285851	0.2	0.919
Siler montanum	CTA	0.934187	0.805139	0.129048	1.09729	0.288889	0.889
Viola eugeniae	CTA	0.981029	0.955774	0.026692	1.397262	0.127273	0.827
Viola ferrarinii	CTA	0.983088	0.952353	0.036373	1.268654	0.1	0.815
Adenostyles australis	Domain	0.830006	0.825431	0.029136	1.35161	0.127778	0.845
Aubrieta columnae	Domain	0.917746	0.902883	0.022431	1.747259	0.316667	0.78
Bellevalia webbiana	Domain	0.825811	0.747508	0.087631	1.416363	0.233333	0.609
Cardamine apennina	Domain	0.73056	0.803313	0.010423	1.468175	0.0875	-0.074
Carduus affinis	Domain	0.874176	0.833158	0.05229	1.395052	0.222222	0.91
Crocus biflorus	Domain	0.639202	0.640552	0.02136	1.174294	0.145455	0.758
Crocus etruscus	Domain	0.96169	0.656425	0.305265	1.451484	0.65	0.941
Crocus imperati	Domain	0.720264	0.607892	0.11328	1.267414	0.433333	0.788
Erysimum pseudorhaeticum	Domain	0.581708	0.564129	0.043157	1.152376	0.214286	0.982
Galium magellense	Domain	0.968372	0.96755	0.004367	1.902696	0.133333	0.922

Gypsophila arrostoi	Domain	0.760613	0.708331	0.053538	1.360126	0.328095	0.243
Helleborus viridis	Domain	0.626782	0.546359	0.089552	1.143122	0.218182	0.895
Klasea flavescens	Domain	0.722973	0.6725	0.072443	1.338434	0.3	0.538
Koeleria splendens	Domain	0.731366	0.739748	0.014035	1.231142	0.104	0.998
Leontodon anomalus	Domain	0.876254	0.80156	0.087828	1.532466	0.34	0.868
Linaria purpurea	Domain	0.558346	0.531407	0.058109	1.095175	0.13913	0.886
Ornithogalum etruscum	Domain	0.628691	0.56102	0.075893	1.248114	0.303333	0.329
Phyteuma ovatum	Domain	0.809439	0.794978	0.046055	1.407376	0.188889	0.808
Polygala flavescens	Domain	0.62123	0.615738	0.019362	1.085672	0.111628	-0.075
Ranunculus pollinensis	Domain	0.943798	0.937156	0.009957	1.775942	0.166667	0.963
Saxifraga porophylla	Domain	0.91691	0.875615	0.042157	1.538118	0.230769	0.975
Sedum magellense	Domain	0.915896	0.904725	0.018756	1.656102	0.15	0.961
Siler montanum	Domain	0.743592	0.729775	0.028818	1.219593	0.155556	0.979
Viola eugeniae	Domain	0.931256	0.899973	0.037991	1.609718	0.254545	0.976
Viola ferrarinii	Domain	0.973392	0.93505	0.047116	1.773841	0.36	0.778
Adenostyles australis	FDA	0.901658	0.919945	0.006121	1.535009	0.077778	0.979
Aubrieta columnae	FDA	0.945344	0.929825	0.032389	1.775606	0.233333	0.897
Bellevalia webbiana	FDA	0.901786	0.897083	0.029464	1.70446	0.233333	0.785
Cardamine apennina	FDA	0.729286	0.654018	0.085714	1.217948	0.2375	0.212
Carduus affinis	FDA	0.931746	0.931759	0.009828	1.608532	0.122222	0.971
Crocus biflorus	FDA	0.59033	0.568742	0.041007	1.098677	0.195455	0.74
Crocus etruscus	FDA	0.937143	0.914286	0.067143	1.699235	0.4	0.479
Crocus imperati	FDA	0.844464	0.78	0.082321	1.438195	0.25	0.75
Erysimum pseudorhaeticum	FDA	0.791613	0.76381	0.051695	1.242402	0.221429	0.95
Galium magellense	FDA	0.986325	0.986111	0.003704	1.914232	0.116667	0.568
Gypsophila arrostoi	FDA	0.580735	0.578302	0.046729	1.225583	0.145238	0.62
Helleborus viridis	FDA	0.698778	0.672475	0.048859	1.205457	0.236364	0.475
Klasea flavescens	FDA	0.608254	0.538889	0.077778	1.199062	0.316667	0.84
Koeleria splendens	FDA	0.839863	0.854361	0.018374	1.398618	0.116	0.988
Leontodon anomalus	FDA	0.853419	0.876667	0.022479	1.62211	0.1	0.892
Linaria purpurea	FDA	0.712928	0.695949	0.038751	1.115809	0.145455	0.991
Ornithogalum etruscum	FDA	0.725439	0.620175	0.126754	1.247013	0.35	0.407
Phyteuma ovatum	FDA	0.918035	0.865233	0.066471	1.506617	0.2	0.874
Polygala flavescens	FDA	0.749157	0.73712	0.030665	1.179457	0.123256	0.885
Ranunculus pollinensis	FDA	0.98	0.968966	0.011437	1.77829	0.211111	0.971
Saxifraga porophylla	FDA	0.951085	0.942397	0.019153	1.622947	0.176923	0.788
Sedum magellense	FDA	0.959196	0.947917	0.013006	1.703985	0.208333	0.923
Siler montanum	FDA	0.880595	0.879398	0.020476	1.381807	0.138889	0.966
Viola eugeniae	FDA	0.959459	0.957248	0.013013	1.790071	0.154545	0.946
Viola ferrarinii	FDA	0.988725	0.988235	0.008431	1.633655	0.2	0.752
Adenostyles australis	GAM	0.915534	0.886433	0.036588	1.40175	0.155556	0.895
Aubrieta columnae	GAM	0.95495	0.883742	0.075939	1.621058	0.283333	0.785
Bellevalia webbiana	GAM	0.932589	0.884017	0.05234	1.700769	0.333333	0.897
Cardamine apennina	GAM	0.84944	0.765419	0.095451	1.382652	0.2875	0.528
Carduus affinis	GAM	0.925037	0.931156	0.011177	1.634603	0.15	0.758
Crocus biflorus	GAM	0.662369	0.616445	0.048452	1.168236	0.181818	0.883
Crocus etruscus	GAM	0.97716	0.93975	0.04378	1.886968	0.65	0.899

Crocus imperati	GAM	0.799914	0.780792	0.05319	1.52881	0.133333	0.775
Erysimum pseudorhaeticum	GAM	0.809287	0.771236	0.067238	1.250479	0.221429	0.848
Galium magellense	GAM	0.983013	0.9827	0.003185	1.903459	0.116667	0.987
Gypsophila arrostoi	GAM	0.80057	0.710762	0.095898	1.378995	0.381905	0.913
Helleborus viridis	GAM	0.799916	0.641323	0.158593	1.186355	0.354545	0.451
Klasea flavescens	GAM	0.776603	0.661883	0.119617	1.324305	0.333333	0.252
Koeleria splendens	GAM	0.874048	0.836874	0.045486	1.294423	0.188	0.933
Leontodon anomalus	GAM	0.891988	0.77615	0.116014	1.443051	0.4	0.748
Linaria purpurea	GAM	0.747528	0.683479	0.068464	1.102698	0.185771	0.802
Ornithogalum etruscum	GAM	0.752114	0.58353	0.168584	1.265239	0.456667	0.788
Phyteuma ovatum	GAM	0.907509	0.85715	0.082821	1.511928	0.233333	0.572
Polygala flavescens	GAM	0.783746	0.733092	0.051288	1.149925	0.151163	0.815
Ranunculus pollinensis	GAM	0.96478	0.940689	0.030334	1.738952	0.144444	0.965
Saxifraga porophylla	GAM	0.92635	0.841146	0.085204	1.278275	0.230769	0.834
Sedum magellense	GAM	0.942825	0.936371	0.016864	1.683734	0.133333	0.93
Siler montanum	GAM	0.850854	0.860722	0.017697	1.363895	0.116667	0.915
Viola eugeniae	GAM	0.95044	0.914882	0.04252	1.6106	0.2	0.875
Viola ferrarinii	GAM	0.982829	0.95771	0.029493	1.854447	0.32	0.848
Adenostyles australis	GLM	0.89528	0.901475	0.012357	1.509366	0.127778	0.538
Aubrieta columnae	GLM	0.940915	0.953708	0.008488	1.837205	0.166667	0.988
Bellevalia webbiana	GLM	0.870004	0.868867	0.017661	1.647571	0.183333	0.894
Cardamine apennina	GLM	0.690093	0.615763	0.08572	1.214296	0.2	0.16
Carduus affinis	GLM	0.931807	0.942439	0.008737	1.674542	0.116667	0.862
Crocus biflorus	GLM	0.600042	0.572595	0.032301	1.100739	0.154545	0.681
Crocus etruscus	GLM	0.92402	0.91665	0.04604	1.867295	0.25	0.8
Crocus imperati	GLM	0.787782	0.780367	0.043483	1.527234	0.183333	0.824
Erysimum pseudorhaeticum	GLM	0.792073	0.771925	0.03504	1.247208	0.15	0.919
Galium magellense	GLM	0.986928	0.985008	0.003843	1.915881	0.166667	0.993
Gypsophila arrostoi	GLM	0.583866	0.436039	0.152726	1.155637	0.359524	0.706
Helleborus viridis	GLM	0.728392	0.6727	0.071813	1.211455	0.2	0.12
Klasea flavescens	GLM	0.620857	0.597275	0.071858	1.287691	0.183333	0.315
Koeleria splendens	GLM	0.859129	0.832274	0.032033	1.354862	0.148	0.74
Leontodon anomalus	GLM	0.797381	0.77314	0.074348	1.483942	0.24	0.873
Linaria purpurea	GLM	0.695739	0.670688	0.046254	1.071415	0.074704	0.887
Ornithogalum etruscum	GLM	0.693659	0.58452	0.120597	1.286506	0.363333	0.611
Phyteuma ovatum	GLM	0.900875	0.867267	0.07402	1.502983	0.255556	0.837
Polygala flavescens	GLM	0.74897	0.735777	0.027727	1.195542	0.118605	0.907
Ranunculus pollinensis	GLM	0.962358	0.956811	0.016023	1.814233	0.177778	0.967
Saxifraga porophylla	GLM	0.933437	0.930315	0.022967	1.617634	0.153846	0.924
Sedum magellense	GLM	0.948905	0.960304	0.002769	1.79277	0.1	0.937
Siler montanum	GLM	0.866076	0.867203	0.016254	1.380647	0.111111	0.911
Viola eugeniae	GLM	0.961423	0.965823	0.004916	1.823009	0.163636	0.994
Viola ferrarinii	GLM	0.981667	0.9845	0.007823	1.946675	0.2	0.931
Adenostyles australis	KDE	0.868876	0.845322	0.036215	1.343496	0.161111	0.92
Aubrieta columnae	KDE	0.932862	0.849625	0.08373	1.586466	0.383333	0.948
Bellevalia webbiana	KDE	0.774532	0.757075	0.059012	1.427381	0.25	0.39
Cardamine apennina	KDE	0.747875	0.739313	0.039933	1.316177	0.0875	0.131

Carduus affinis	KDE	0.901686	0.860117	0.047135	1.387084	0.205556	0.907
Crocus biflorus	KDE	0.651803	0.601984	0.057139	1.101457	0.15	0.779
Crocus etruscus	KDE	0.95115	0.749775	0.201375	1.591889	0.65	0.644
Crocus imperati	KDE	0.77615	0.598692	0.177458	1.208979	0.4	0.671
Erysimum pseudorhaeticum	KDE	0.533116	0.467625	0.066017	1.099645	0.135714	0.223
Galium magellense	KDE	0.969237	0.9651	0.01235	1.821953	0.158333	0.943
Gypsophila arrostoi	KDE	0.742291	0.69415	0.049701	1.290816	0.185714	0.729
Helleborus viridis	KDE	0.598152	0.496427	0.101725	1.11806	0.209091	0.854
Klasea flavescens	KDE	0.735127	0.719642	0.037848	1.370973	0.183333	0.459
Koeleria splendens	KDE	0.702854	0.667116	0.042751	1.123887	0.168	0.762
Leontodon anomalus	KDE	0.811881	0.79876	0.059322	1.547248	0.28	0.662
Linaria purpurea	KDE	0.552859	0.497978	0.060346	1.087577	0.143478	0.526
Ornithogalum etruscum	KDE	0.638523	0.538683	0.126844	1.200643	0.333333	0.124
Phyteuma ovatum	KDE	0.811775	0.771489	0.05483	1.362494	0.188889	0.989
Polygala flavescens	KDE	0.613087	0.56197	0.05829	1.05442	0.169767	-0.127
Ranunculus pollinensis	KDE	0.948168	0.908372	0.046643	1.663348	0.233333	0.733
Saxifraga porophylla	KDE	0.929062	0.881258	0.054845	1.481642	0.238462	0.995
Sedum magellense	KDE	0.913436	0.895554	0.028218	1.562894	0.225	0.978
Siler montanum	KDE	0.78946	0.769083	0.03725	1.214963	0.161111	0.978
Viola eugeniae	KDE	0.936794	0.923518	0.026345	1.651948	0.118182	0.955
Viola ferrarinii	KDE	0.966808	0.89965	0.083486	1.689579	0.38	0.886
Adenostyles australis	MARS	0.906292	0.842878	0.063414	1.211299	0.211111	0.849
Aubrieta columnae	MARS	0.951735	0.811417	0.144356	1.460746	0.333333	0.734
Bellevalia webbiana	MARS	0.921582	0.832533	0.089049	1.575347	0.483333	0.848
Cardamine apennina	MARS	0.839595	0.71485	0.125606	1.248866	0.2	-0.238
Carduus affinis	MARS	0.934101	0.904108	0.038684	1.452769	0.227778	0.69
Crocus biflorus	MARS	0.689068	0.578659	0.112693	1.120666	0.245455	0.916
Crocus etruscus	MARS	0.94343	0.8217	0.1271	1.707302	0.35	0.697
Crocus imperati	MARS	0.818121	0.6725	0.155668	1.290199	0.25	0.739
Erysimum pseudorhaeticum	MARS	0.800503	0.721339	0.079564	1.115447	0.178571	0.714
Galium magellense	MARS	0.987469	0.936708	0.051119	1.626952	0.266667	0.953
Gypsophila arrostoi	MARS	0.74204	0.584467	0.166544	1.157876	0.289048	0.48
Helleborus viridis	MARS	0.727868	0.628036	0.101744	1.10568	0.2	0.415
Klasea flavescens	MARS	0.716013	0.510967	0.205047	1.335205	0.264936	-0.921
Koeleria splendens	MARS	0.88015	0.853388	0.034841	1.319421	0.14	0.867
Leontodon anomalus	MARS	0.923758	0.86098	0.063967	1.59547	0.4	0.785
Linaria purpurea	MARS	0.6863	0.602109	0.08432	1.017079	0.12668	0.644
Ornithogalum etruscum	MARS	0.690308	0.513585	0.17749	1.095845	0.323333	0.725
Phyteuma ovatum	MARS	0.904048	0.75775	0.153192	1.237976	0.333333	0.552
Polygala flavescens	MARS	0.802718	0.749938	0.054226	1.143342	0.174419	0.743
Ranunculus pollinensis	MARS	0.954943	0.850567	0.105799	1.415565	0.277778	0.794
Saxifraga porophylla	MARS	0.945892	0.871208	0.074684	1.389719	0.223077	0.831
Sedum magellense	MARS	0.959259	0.874492	0.087037	1.457533	0.283333	0.863
Siler montanum	MARS	0.863471	0.803269	0.060202	1.189279	0.133333	0.793
Viola eugeniae	MARS	0.941335	0.818118	0.125808	1.287614	0.254545	0.651
Viola ferrarinii	MARS	0.972667	0.90095	0.08173	1.692554	0.44	0.728
Adenostyles australis	Maxent	0.902913	0.906389	0.019908	1.549909	0.122222	0.975

Aubrieta columnae	Maxent	0.947255	0.946228	0.020351	1.803455	0.2	0.869
Bellevalia webbiana	Maxent	0.880621	0.863073	0.02928	1.657836	0.15	0.865
Cardamine apennina	Maxent	0.832442	0.788163	0.068748	1.403508	0.1875	0.265
Carduus affinis	Maxent	0.935452	0.936223	0.016059	1.661314	0.138889	0.955
Crocus biflorus	Maxent	0.705142	0.660786	0.049477	1.192779	0.2	0.936
Crocus etruscus	Maxent	0.93247	0.90291	0.062626	1.842454	0.35	0.464
Crocus imperati	Maxent	0.798736	0.788086	0.066781	1.534308	0.266667	0.792
Erysimum pseudorhaeticum	Maxent	0.789122	0.790849	0.034452	1.336755	0.142857	0.891
Galium magellense	Maxent	0.987561	0.98512	0.003453	1.917068	0.15	0.958
Gypsophila arrostoi	Maxent	0.719757	0.52556	0.194197	1.260558	0.37619	0.957
Helleborus viridis	Maxent	0.7406	0.679667	0.072288	1.221507	0.236364	0.949
Klasea flavescens	Maxent	0.796294	0.602548	0.199229	1.274371	0.4	0.426
Koeleria splendens	Maxent	0.87979	0.852328	0.030906	1.384463	0.2	0.944
Leontodon anomalus	Maxent	0.826552	0.803323	0.04392	1.540187	0.22	0.689
Linaria purpurea	Maxent	0.741813	0.698955	0.055735	1.127366	0.155138	0.982
Ornithogalum etruscum	Maxent	0.665802	0.615357	0.07569	1.307141	0.27	0.706
Phyteuma ovatum	Maxent	0.91374	0.865129	0.067315	1.505158	0.255556	0.951
Polygala flavescens	Maxent	0.775163	0.766821	0.016402	1.199348	0.12093	0.818
Ranunculus pollinensis	Maxent	0.966027	0.95072	0.01798	1.788653	0.188889	0.946
Saxifraga porophylla	Maxent	0.929112	0.961898	0	1.784119	0.053846	0.918
Sedum magellense	Maxent	0.957517	0.94244	0.01735	1.72117	0.208333	0.891
Siler montanum	Maxent	0.876473	0.851993	0.039563	1.368812	0.205556	0.944
Viola eugeniae	Maxent	0.967807	0.954503	0.02053	1.765716	0.190909	0.973
Viola ferrarinii	Maxent	0.979337	0.982332	0.009278	1.864372	0.26	0.613
Adenostyles australis	RF	1	0.952823	0.047177	1.406571	0.411111	0.971
Aubrieta columnae	RF	1	0.932018	0.067982	1.661193	0.3	0.823
Bellevalia webbiana	RF	1	0.983333	0.016667	1.633757	0.616667	0.784
Cardamine apennina	RF	1	0.947321	0.052679	1.399825	0.475	0.643
Carduus affinis	RF	1	0.968472	0.031528	1.56125	0.438889	0.945
Crocus biflorus	RF	1	0.932036	0.067964	1.156475	0.8	0.716
Crocus etruscus	RF	1	0.942857	0.057143	1.729213	0.75	0.642
Crocus imperati	RF	1	0.868333	0.131667	1.344279	0.766667	0.777
Erysimum pseudorhaeticum	RF	1	0.942698	0.057302	1.305673	0.557143	0.984
Galium magellense	RF	1	0.998718	0.001282	1.90042	0.291667	0.859
Gypsophila arrostoi	RF	1	0.952899	0.047101	1.364218	0.87381	0.771
Helleborus viridis	RF	1	0.943182	0.056818	1.244761	0.763636	0.93
Klasea flavescens	RF	1	0.898413	0.101587	1.322862	0.65	0.687
Koeleria splendens	RF	1	0.962482	0.037518	1.334662	0.54	0.987
Leontodon anomalus	RF	1	0.923889	0.076111	1.521836	0.62	0.939
Linaria purpurea	RF	1	0.917985	0.082015	1.132954	0.644862	0.971
Ornithogalum etruscum	RF	1	0.934912	0.065088	1.278226	0.913333	0.408
Phyteuma ovatum	RF	1	0.959677	0.040323	1.59088	0.322222	0.897
Polygala flavescens	RF	1	0.934485	0.065515	1.166529	0.667442	0.928
Ranunculus pollinensis	RF	1	0.99023	0.00977	1.775499	0.277778	0.936
Saxifraga porophylla	RF	1	0.923345	0.076655	1.414877	0.430769	0.974
Sedum magellense	RF	1	0.979375	0.020625	1.677632	0.358333	0.913
Siler montanum	RF	1	0.949074	0.050926	1.345179	0.416667	0.958

Viola eugeniae	RF	1	0.974939	0.025061	1.706045	0.318182	0.945
Viola ferrarinii	RF	1	1	0	1.925235	0.42	0.943
Adenostyles australis	SVM	0.908292	0.891985	0.030558	1.416891	0.144444	0.964
Aubrieta columnae	SVM	0.929352	0.938596	0.016194	1.79025	0.133333	0.957
Bellevalia webbiana	SVM	0.935714	0.925833	0.018333	1.746801	0.2	0.901
Cardamine apennina	SVM	0.870536	0.825	0.061518	1.381483	0.2375	0.435
Carduus affinis	SVM	0.937619	0.922176	0.02334	1.546519	0.177778	0.921
Crocus biflorus	SVM	0.751961	0.675436	0.076638	1.122168	0.190909	0.831
Crocus etruscus	SVM	0.991429	0.95	0.047143	1.779557	0.5	0.933
Crocus imperati	SVM	0.888393	0.8625	0.044583	1.493486	0.216667	0.789
Erysimum pseudorhaeticum	SVM	0.835806	0.800952	0.048088	1.216548	0.185714	0.784
Galium magellense	SVM	0.987179	0.986111	0.002754	1.924542	0.15	0.895
Gypsophila arrostoi	SVM	0.869526	0.76971	0.100127	1.262333	0.351905	0.9
Helleborus viridis	SVM	0.802444	0.762121	0.052939	1.227507	0.172727	0.809
Klasea flavescens	SVM	0.831587	0.789286	0.061984	1.401737	0.266667	0.319
Koeleria splendens	SVM	0.86558	0.872819	0.01273	1.266171	0.116	0.936
Leontodon anomalus	SVM	0.91859	0.864444	0.069444	1.53934	0.28	0.784
Linaria purpurea	SVM	0.780148	0.731697	0.0552	1.078545	0.148221	0.887
Ornithogalum etruscum	SVM	0.835442	0.785175	0.071808	1.294183	0.206667	0.588
Phyteuma ovatum	SVM	0.921334	0.894086	0.0453	1.541423	0.255556	0.839
Polygala flavescens	SVM	0.781664	0.753253	0.032155	1.170311	0.137209	0.911
Ranunculus pollinensis	SVM	0.971897	0.967433	0.01046	1.804221	0.155556	0.976
Saxifraga porophylla	SVM	0.943101	0.931843	0.02249	1.564334	0.146154	0.926
Sedum magellense	SVM	0.9675	0.954792	0.014226	1.703576	0.191667	0.921
Siler montanum	SVM	0.87375	0.848843	0.036495	1.216959	0.161111	0.923
Viola eugeniae	SVM	0.97079	0.942875	0.0311	1.691211	0.172727	0.952
Viola ferrarinii	SVM	0.987745	0.972941	0.023529	1.847704	0.3	0.78

Table S.6.B. Complete list of evaluation metrics calculated for all procedures based on 3 environmental variables and modern data.

Species	Method	AUC_train (present)	AUC_test (present)	AUC_diff (present)	pROC (present)	OR_p10 (present)	CBI (present)
Adenostyles australis	Bioclim	0.868322	0.822278	0.063054	1.21155	0.155556	0.725
Aubrieta columnae	Bioclim	0.905669	0.784725	0.098256	1.337619	0.233333	0.473
Bellevalia webbiana	Bioclim	0.921982	0.810108	0.136382	1.397976	0.35	0.476
Cardamine apennina	Bioclim	0.81119	0.754956	0.141579	1.308865	0.1875	0.541
Carduus affinis	Bioclim	0.934189	0.891872	0.041534	1.383057	0.211111	0.724
Crocus biflorus	Bioclim	0.70526	0.649226	0.036017	1.087787	0.180303	0.549
Crocus etruscus	Bioclim	0.95446	0.69325	0.36822	1.29789	0.55	0.061
Crocus imperati	Bioclim	0.843512	0.698032	0.160306	1.243262	0.303333	0.367
Erysimum pseudorhaeticum	Bioclim	0.784577	0.725046	0.03267	1.176923	0.25	0.448
Galium magellense	Bioclim	0.975031	0.919238	0.061421	1.484897	0.15	0.662
Gypsophila arrostoi	Bioclim	0.772911	0.627643	0.12832	1.139539	0.32381	0.914
Helleborus viridis	Bioclim	0.628886	0.613818	0.067286	1.134255	0.118182	0.654

Klasea flavescens	Bioclim	0.665607	0.589175	0.084397	1.165128	0.166667	0.762
Koeleria splendens	Bioclim	0.818224	0.788204	0.030072	1.179198	0.12	0.958
Leontodon anomalus	Bioclim	0.866908	0.74475	0.073438	1.255638	0.3	0.682
Linaria purpurea	Bioclim	0.545913	0.50558	0.058653	1.035909	0.152174	-0.12
Ornithogalum etruscum	Bioclim	0.64673	0.57831	0.10195	1.148089	0.31	-0.311
Phyteuma ovatum	Bioclim	0.827673	0.732233	0.064628	1.199511	0.2	0.769
Polygala flavescens	Bioclim	0.659201	0.617282	0.034644	1.097991	0.16794	0.275
Ranunculus pollinensis	Bioclim	0.96393	0.884406	0.133349	1.417638	0.166667	0.496
Saxifraga porophylla	Bioclim	0.877627	0.822719	0.048278	1.326448	0.130769	0.249
Sedum magellense	Bioclim	0.931455	0.834733	0.055439	1.341712	0.241667	0.795
Siler montanum	Bioclim	0.86993	0.827372	0.063414	1.285498	0.122222	0.605
Viola eugeniae	Bioclim	0.898885	0.859159	0.062342	1.332983	0.209091	0.734
Viola ferrarinii	Bioclim	0.990742	0.75215	0.124506	1.215665	0.48	0.054
Adenostyles australis	Biomod2	0.98478	0.932339	0.108711	1.681636	0.4	0.865
Aubrieta columnae	Biomod2	0.994331	0.881103	0.047355	1.627501	0.666667	0.922
Bellevalia webbiana	Biomod2	0.994122	0.907857	0.062898	1.731009	0.8	0.927
Cardamine apennina	Biomod2	0.99764	0.82015	0.094108	1.434929	0.7125	0.92
Carduus affinis	Biomod2	0.991256	0.962177	0.012161	1.778342	0.416667	0.923
Crocus biflorus	Biomod2	0.985403	0.714289	0.270498	1.151355	0.92381	0.965
Crocus etruscus	Biomod2	1	0.988	0.0948	1.942947	1	0.957
Crocus imperati	Biomod2	0.998226	0.857569	0.249731	1.614679	0.816667	0.914
Erysimum pseudorhaeticum	Biomod2	0.959515	0.852604	0.140352	1.47605	0.414286	0.989
Galium magellense	Biomod2	0.996963	0.986599	0.003543	1.898238	0.333333	0.922
Gypsophila arrostoi	Biomod2	0.992707	0.822252	0.256939	1.469755	0.871429	0.952
Helleborus viridis	Biomod2	0.99559	0.586945	0.241557	1.100253	0.945455	0.983
Klasea flavescens	Biomod2	0.996311	0.598867	0.272049	1.250037	1	0.984
Koeleria splendens	Biomod2	0.964116	0.841592	0.065361	1.229065	0.316	0.953
Leontodon anomalus	Biomod2	0.99716	0.9536	0.139091	1.861247	0.98	0.931
Linaria purpurea	Biomod2	0.941449	0.736008	0.245843	1.119883	0.521739	0.982
Ornithogalum etruscum	Biomod2	0.999929	0.638646	0.412573	1.278329	1	0.911
Phyteuma ovatum	Biomod2	0.995909	0.974939	0.190902	1.870163	0.511111	0.614
Polygala flavescens	Biomod2	0.920962	0.733691	0.162562	1.170196	0.43433	0.995
Ranunculus pollinensis	Biomod2	0.99855	0.980956	0.016898	1.859616	0.422222	0.907
Saxifraga porophylla	Biomod2	0.986524	0.935933	0.038224	1.628746	0.4	0.965
Sedum magellense	Biomod2	0.99273	0.938738	0.085529	1.665771	0.491667	0.938
Siler montanum	Biomod2	0.980961	0.935804	0.118581	1.631824	0.327778	0.981
Viola eugeniae	Biomod2	0.989959	0.986923	0.019767	1.911742	0.109091	0.928
Viola ferrarinii	Biomod2	0.999	0.999533	0.011228	1.989476	0.08	0.869
Adenostyles australis	CTA	0.955986	0.900091	0.090991	1.25459	0.211111	0.883
Aubrieta columnae	CTA	0.959109	0.902193	0.041464	1.289143	0.2	0.863
Bellevalia webbiana	CTA	0.935179	0.879583	0.074464	1.293898	0.166667	0.929
Cardamine apennina	CTA	0.93875	0.845089	0.124821	1.257684	0.2375	0.898
Carduus affinis	CTA	0.969782	0.941713	0.047262	1.576173	0.177778	0.928
Crocus biflorus	CTA	0.899235	0.710852	0.209086	1.03897	0.37013	0.994
Crocus etruscus	CTA	0.787143	0.657143	0.05	1	0.4	0.933
Crocus imperati	CTA	0.958819	0.864167	0.092857	1.221067	0.246667	0.678
Erysimum pseudorhaeticum	CTA	0.933333	0.827063	0.141575	1.18627	0.264286	0.986
Galium magellense	CTA	0.984093	0.927671	0.013865	1.168701	0.133333	0.929

Gypsophila arrostoi	CTA	0.911639	0.79648	0.154925	1.162547	0.3	0.891
Helleborus viridis	CTA	0.890389	0.700631	0.13597	1.071492	0.372727	0.958
Klasea flavescens	CTA	0.875556	0.70119	0.179921	1.118723	0.316667	0.877
Koeleria splendens	CTA	0.940642	0.879663	0.101427	1.192607	0.224	0.973
Leontodon anomalus	CTA	0.952564	0.895	0.122179	1.332752	0.22	0.965
Linaria purpurea	CTA	0.920559	0.798885	0.148987	1.081266	0.278261	0.995
Ornithogalum etruscum	CTA	0.875337	0.700702	0.141215	1.132524	0.38	0.894
Phyteuma ovatum	CTA	0.982991	0.928495	0.072499	1.298099	0.211111	0.639
Polygala flavescens	CTA	0.855702	0.749123	0.120388	1.072472	0.214784	0.994
Ranunculus pollinensis	CTA	0.987155	0.970307	0.058391	1.364045	0.044444	0.94
Saxifraga porophylla	CTA	0.967209	0.924329	0.051354	1.315775	0.176923	0.879
Sedum magellense	CTA	0.978304	0.947083	0.072009	1.249258	0.083333	0.876
Siler montanum	CTA	0.964246	0.914769	0.129048	1.324545	0.227778	0.962
Viola eugeniae	CTA	0.971622	0.939803	0.026692	1.333	0.145455	0.828
Viola ferrarinii	CTA	0.996078	0.965882	0.036373	1.133209	0.06	0.76
Adenostyles australis	Domain	0.850034	0.813331	0.029136	1.322445	0.177778	0.75
Aubrieta columnae	Domain	0.895331	0.868667	0.022431	1.668007	0.45	0.916
Bellevalia webbiana	Domain	0.89325	0.849533	0.087631	1.60996	0.266667	0.865
Cardamine apennina	Domain	0.752168	0.704188	0.010423	1.408259	0.1875	0.581
Carduus affinis	Domain	0.931221	0.923558	0.05229	1.623523	0.172222	0.917
Crocus biflorus	Domain	0.674701	0.686491	0.02136	1.182252	0.09632	0.678
Crocus etruscus	Domain	0.916	0.8646	0.305265	1.779387	0.3	0.776
Crocus imperati	Domain	0.817821	0.747125	0.11328	1.420795	0.273333	0.749
Erysimum pseudorhaeticum	Domain	0.698773	0.687889	0.043157	1.192018	0.185714	0.933
Galium magellense	Domain	0.97703	0.969717	0.004367	1.826859	0.108333	0.995
Gypsophila arrostoi	Domain	0.759646	0.657594	0.053538	1.305922	0.333333	0.908
Helleborus viridis	Domain	0.612118	0.558918	0.089552	1.137134	0.218182	0.833
Klasea flavescens	Domain	0.61796	0.585225	0.072443	1.25014	0.283333	0.046
Koeleria splendens	Domain	0.812053	0.799062	0.014035	1.280256	0.132	0.978
Leontodon anomalus	Domain	0.828665	0.85237	0.087828	1.624528	0.18	0.985
Linaria purpurea	Domain	0.507835	0.501896	0.058109	1.069085	0.143478	0.887
Ornithogalum etruscum	Domain	0.608566	0.535873	0.075893	1.248407	0.39	0.642
Phyteuma ovatum	Domain	0.8971	0.899444	0.046055	1.616754	0.155556	0.818
Polygala flavescens	Domain	0.621862	0.602339	0.019362	1.091154	0.100831	0.697
Ranunculus pollinensis	Domain	0.955378	0.942889	0.009957	1.774872	0.166667	0.984
Saxifraga porophylla	Domain	0.902783	0.884077	0.042157	1.543955	0.184615	0.979
Sedum magellense	Domain	0.930527	0.913863	0.018756	1.670887	0.2	0.91
Siler montanum	Domain	0.822392	0.792375	0.028818	1.378926	0.155556	0.988
Viola eugeniae	Domain	0.90811	0.881277	0.037991	1.522303	0.2	0.989
Viola ferrarinii	Domain	0.987179	0.9844	0.047116	1.958309	0.24	0.979
Adenostyles australis	FDA	0.921121	0.89827	0.006121	1.475696	0.194444	0.936
Aubrieta columnae	FDA	0.950607	0.930702	0.032389	1.771411	0.183333	0.65
Bellevalia webbiana	FDA	0.903571	0.9075	0.029464	1.722781	0.266667	0.947
Cardamine apennina	FDA	0.698393	0.640179	0.085714	1.258165	0.2375	0.487
Carduus affinis	FDA	0.948929	0.958426	0.009828	1.823853	0.072222	0.913
Crocus biflorus	FDA	0.608644	0.55921	0.041007	1.097636	0.145238	0.734
Crocus etruscus	FDA	0.965714	0.842857	0.067143	1.599392	0.5	0.895
Crocus imperati	FDA	0.873791	0.818667	0.082321	1.507778	0.313333	0.604

Erysimum pseudorhaeticum	FDA	0.822724	0.822222	0.051695	1.393007	0.128571	0.946
Galium magellense	FDA	0.982146	0.982051	0.003704	1.812019	0.15	0.595
Gypsophila arrostoi	FDA	0.752645	0.69648	0.046729	1.321832	0.27381	0.745
Helleborus viridis	FDA	0.633556	0.659091	0.048859	1.195781	0.127273	0.59
Klasea flavescens	FDA	0.638095	0.623016	0.077778	1.229367	0.233333	0.59
Koeleria splendens	FDA	0.86398	0.859181	0.018374	1.381784	0.104	0.988
Leontodon anomalus	FDA	0.865385	0.876667	0.022479	1.576524	0.26	0.942
Linaria purpurea	FDA	0.7669	0.767726	0.038751	1.154635	0.121739	0.977
Ornithogalum etruscum	FDA	0.738698	0.718596	0.126754	1.347142	0.24	0.66
Phyteuma ovatum	FDA	0.891056	0.920789	0.066471	1.688748	0.133333	0.826
Polygala flavescens	FDA	0.717747	0.717767	0.030665	1.133271	0.09845	0.911
Ranunculus pollinensis	FDA	0.974483	0.981609	0.011437	1.8895	0.144444	0.907
Saxifraga porophylla	FDA	0.93062	0.935242	0.019153	1.655334	0.138462	0.873
Sedum magellense	FDA	0.967768	0.956667	0.013006	1.742916	0.191667	0.814
Siler montanum	FDA	0.92504	0.930463	0.020476	1.631588	0.166667	0.92
Viola eugeniae	FDA	0.935135	0.946192	0.013013	1.734828	0.1	0.892
Viola ferrarinii	FDA	0.989706	0.992941	0.008431	1.441228	0.12	0.798
Adenostyles australis	GAM	0.940242	0.925825	0.036588	1.551241	0.138889	0.99
Aubrieta columnae	GAM	0.956158	0.82955	0.075939	1.506667	0.383333	0.873
Bellevalia webbiana	GAM	0.91545	0.897467	0.05234	1.713777	0.3	0.862
Cardamine apennina	GAM	0.9089	0.881338	0.095451	1.605157	0.1625	0.824
Carduus affinis	GAM	0.961288	0.961339	0.011177	1.763792	0.116667	0.863
Crocus biflorus	GAM	0.703451	0.654709	0.048452	1.176299	0.165801	0.955
Crocus etruscus	GAM	0.95694	0.8022	0.04378	1.708773	0.75	0.847
Crocus imperati	GAM	0.91246	0.787618	0.05319	1.465604	0.386667	0.835
Erysimum pseudorhaeticum	GAM	0.858211	0.824196	0.067238	1.399576	0.178571	0.758
Galium magellense	GAM	0.978326	0.961079	0.003185	1.759854	0.166667	0.955
Gypsophila arrostoi	GAM	0.799053	0.724369	0.095898	1.36492	0.3	0.823
Helleborus viridis	GAM	0.68407	0.672836	0.158593	1.178268	0.163636	0.668
Klasea flavescens	GAM	0.669853	0.553892	0.119617	1.232748	0.333333	0.935
Koeleria splendens	GAM	0.866755	0.857346	0.045486	1.317188	0.108	0.986
Leontodon anomalus	GAM	0.896869	0.84888	0.116014	1.582192	0.34	0.797
Linaria purpurea	GAM	0.77612	0.74092	0.068464	1.116574	0.152174	0.915
Ornithogalum etruscum	GAM	0.755256	0.605867	0.168584	1.292968	0.38	0.703
Phyteuma ovatum	GAM	0.959482	0.937956	0.082821	1.71228	0.155556	0.852
Polygala flavescens	GAM	0.726548	0.712916	0.051288	1.142549	0.097951	0.904
Ranunculus pollinensis	GAM	0.98025	0.964194	0.030334	1.842439	0.255556	0.96
Saxifraga porophylla	GAM	0.941892	0.883327	0.085204	1.489249	0.238462	0.937
Sedum magellense	GAM	0.960213	0.951829	0.016864	1.734415	0.133333	0.896
Siler montanum	GAM	0.924789	0.924672	0.017697	1.623258	0.172222	0.642
Viola eugeniae	GAM	0.962921	0.912691	0.04252	1.559789	0.190909	0.975
Viola ferrarinii	GAM	0.993479	0.96836	0.029493	1.900309	0.28	0.909
Adenostyles australis	GLM	0.915394	0.915106	0.012357	1.518996	0.127778	0.603
Aubrieta columnae	GLM	0.943623	0.920058	0.008488	1.742096	0.35	0.989
Bellevalia webbiana	GLM	0.908861	0.89305	0.017661	1.712018	0.25	0.897
Cardamine apennina	GLM	0.652278	0.5724	0.08572	1.252716	0.1625	0.718
Carduus affinis	GLM	0.954681	0.964186	0.008737	1.797543	0.088889	0.894
Crocus biflorus	GLM	0.596605	0.584617	0.032301	1.119657	0.114069	0.691

Crocus etruscus	GLM	0.88378	0.88085	0.04604	1.800111	0.25	0.712
Crocus imperati	GLM	0.871841	0.800888	0.043483	1.523947	0.376667	0.834
Erysimum pseudorhaeticum	GLM	0.841842	0.806793	0.03504	1.383755	0.192857	0.909
Galium magellense	GLM	0.982343	0.979292	0.003843	1.878009	0.2	0.99
Gypsophila arrostoi	GLM	0.723371	0.721864	0.152726	1.381028	0.185714	0.727
Helleborus viridis	GLM	0.669738	0.675223	0.071813	1.183946	0.118182	0.533
Klasea flavescens	GLM	0.61642	0.491458	0.071858	1.191107	0.283333	0.664
Koeleria splendens	GLM	0.864791	0.868016	0.032033	1.363029	0.108	0.616
Leontodon anomalus	GLM	0.810696	0.85383	0.074348	1.590866	0.16	0.914
Linaria purpurea	GLM	0.751328	0.705548	0.046254	1.099367	0.156522	0.989
Ornithogalum etruscum	GLM	0.709213	0.64247	0.120597	1.317056	0.33	0.563
Phyteuma ovatum	GLM	0.912916	0.914111	0.07402	1.589742	0.166667	0.838
Polygala flavescens	GLM	0.704249	0.702023	0.027727	1.127195	0.109745	0.835
Ranunculus pollinensis	GLM	0.97476	0.974078	0.016023	1.876351	0.177778	0.972
Saxifraga porophylla	GLM	0.929358	0.930538	0.022967	1.674062	0.123077	0.918
Sedum magellense	GLM	0.958745	0.959629	0.002769	1.793087	0.1	0.933
Siler montanum	GLM	0.914105	0.914694	0.016254	1.621309	0.138889	0.865
Viola eugeniae	GLM	0.941642	0.952386	0.004916	1.752234	0.109091	0.998
Viola ferrarinii	GLM	0.991938	0.99012	0.007823	1.969896	0.24	0.984
Adenostyles australis	KDE	0.878331	0.892622	0.019484	1.52175	0.133333	0.954
Aubrieta columnae	KDE	0.918162	0.895458	0.041828	1.703172	0.266667	0.96
Bellevalia webbiana	KDE	0.858111	0.817992	0.049477	1.567325	0.266667	0.902
Cardamine apennina	KDE	0.792108	0.720244	0.087538	1.367991	0.25	0.835
Carduus affinis	KDE	0.947581	0.892944	0.055244	1.380022	0.25	0.973
Crocus biflorus	KDE	0.675219	0.66042	0.040446	1.100461	0.140909	0.794
Crocus etruscus	KDE	0.94543	0.712925	0.234025	1.536671	0.75	0.714
Crocus imperati	KDE	0.818579	0.740317	0.096193	1.407625	0.25	0.836
Erysimum pseudorhaeticum	KDE	0.686569	0.64115	0.061953	1.139341	0.207143	0.91
Galium magellense	KDE	0.979165	0.912625	0.066584	1.423873	0.266667	0.979
Gypsophila arrostoi	KDE	0.829866	0.727921	0.113605	1.321752	0.314286	0.878
Helleborus viridis	KDE	0.637444	0.501077	0.14882	1.075671	0.309091	0.932
Klasea flavescens	KDE	0.6451	0.6038	0.054187	1.259088	0.25	0.476
Koeleria splendens	KDE	0.840096	0.81697	0.031204	1.259652	0.14	0.895
Leontodon anomalus	KDE	0.833508	0.78461	0.074448	1.551892	0.22	0.994
Linaria purpurea	KDE	0.642819	0.592972	0.056095	1.123161	0.130435	0.867
Ornithogalum etruscum	KDE	0.630612	0.414258	0.217524	1.160781	0.5	0.503
Phyteuma ovatum	KDE	0.940891	0.896133	0.05446	1.590618	0.222222	0.988
Polygala flavescens	KDE	0.583906	0.553134	0.035371	1.070097	0.095349	0.835
Ranunculus pollinensis	KDE	0.958433	0.926639	0.038495	1.664221	0.144444	0.911
Saxifraga porophylla	KDE	0.913685	0.892435	0.029544	1.508455	0.161538	0.991
Sedum magellense	KDE	0.93595	0.908425	0.030703	1.564657	0.15	0.998
Siler montanum	KDE	0.831512	0.798886	0.038303	1.308136	0.166667	0.984
Viola eugeniae	KDE	0.908656	0.87775	0.051144	1.501096	0.190909	0.983
Viola ferrarinii	KDE	0.985175	0.94905	0.036125	1.819535	0.52	0.994
Adenostyles australis	MARS	0.924043	0.930858	0.063414	1.549597	0.127778	0.894
Aubrieta columnae	MARS	0.919677	0.746292	0.144356	1.34835	0.35	0.839
Bellevalia webbiana	MARS	0.837257	0.743458	0.089049	1.385957	0.133333	0.786
Cardamine apennina	MARS	0.868113	0.805213	0.125606	1.431413	0.1875	0.823

Carduus affinis	MARS	0.935501	0.898561	0.038684	1.413274	0.205556	0.836
Crocus biflorus	MARS	0.699384	0.623312	0.112693	1.124954	0.220996	0.937
Crocus etruscus	MARS	0.91451	0.825275	0.1271	1.694092	0.6	0.799
Crocus imperati	MARS	0.81171	0.690495	0.155668	1.18418	0.206667	0.798
Erysimum pseudorhaeticum	MARS	0.879658	0.726196	0.079564	1.159556	0.385714	0.735
Galium magellense	MARS	0.975926	0.953071	0.051119	1.675916	0.191667	0.979
Gypsophila arrostoi	MARS	0.812493	0.64675	0.166544	1.209003	0.283333	0.914
Helleborus viridis	MARS	0.745342	0.628309	0.101744	1.148048	0.254545	0.735
Klasea flavescens	MARS	0.618483	0.486583	0.205047	1.050473	0.25	0.748
Koeleria splendens	MARS	0.899516	0.880524	0.034841	1.325354	0.104	0.924
Leontodon anomalus	MARS	0.786781	0.733	0.063967	1.195895	0.12	0.8
Linaria purpurea	MARS	0.777584	0.713835	0.08432	1.058402	0.156522	0.598
Ornithogalum etruscum	MARS	0.727985	0.517968	0.17749	1.195845	0.28857	0.769
Phyteuma ovatum	MARS	0.897402	0.882789	0.153192	1.523671	0.155556	0.8
Polygala flavescens	MARS	0.731078	0.681489	0.054226	1.104243	0.142303	0.901
Ranunculus pollinensis	MARS	0.964875	0.958744	0.105799	1.811641	0.266667	0.899
Saxifraga porophylla	MARS	0.92501	0.861565	0.074684	1.374879	0.176923	0.841
Sedum magellense	MARS	0.942452	0.901908	0.087037	1.524822	0.166667	0.959
Siler montanum	MARS	0.93068	0.858472	0.060202	1.327953	0.233333	0.817
Viola eugeniae	MARS	0.931923	0.901068	0.125808	1.479425	0.190909	0.987
Viola ferrarinii	MARS	0.996308	0.92015	0.08173	1.75889	0.34	0.981
Adenostyles australis	Maxent	0.928594	0.922157	0.019908	1.5555	0.144444	0.969
Aubrieta columnae	Maxent	0.933884	0.937676	0.020351	1.796393	0.183333	0.827
Bellevalia webbiana	Maxent	0.909849	0.910543	0.02928	1.728002	0.183333	0.882
Cardamine apennina	Maxent	0.887476	0.873474	0.068748	1.58913	0.1625	0.915
Carduus affinis	Maxent	0.96155	0.958416	0.016059	1.763787	0.138889	0.966
Crocus biflorus	Maxent	0.720076	0.67496	0.049477	1.152458	0.182684	0.958
Crocus etruscus	Maxent	0.902755	0.841523	0.062626	1.744244	0.5	0.782
Crocus imperati	Maxent	0.864756	0.786884	0.066781	1.489684	0.303333	0.724
Erysimum pseudorhaeticum	Maxent	0.847159	0.8156	0.034452	1.428698	0.192857	0.965
Galium magellense	Maxent	0.984412	0.984394	0.003453	1.896132	0.15	0.955
Gypsophila arrostoi	Maxent	0.80502	0.75357	0.194197	1.412403	0.166667	0.951
Helleborus viridis	Maxent	0.699741	0.66673	0.072288	1.195139	0.145455	0.945
Klasea flavescens	Maxent	0.683525	0.63858	0.199229	1.308703	0.266667	0.732
Koeleria splendens	Maxent	0.89316	0.876025	0.030906	1.37901	0.152	0.988
Leontodon anomalus	Maxent	0.863763	0.788145	0.04392	1.485843	0.3	0.949
Linaria purpurea	Maxent	0.772418	0.726511	0.055735	1.127029	0.165217	0.974
Ornithogalum etruscum	Maxent	0.704364	0.705274	0.07569	1.400695	0.24	0.729
Phyteuma ovatum	Maxent	0.963631	0.910857	0.067315	1.629537	0.244444	0.909
Polygala flavescens	Maxent	0.738423	0.721978	0.016402	1.170542	0.116777	0.978
Ranunculus pollinensis	Maxent	0.976902	0.980892	0.01798	1.904232	0.122222	0.949
Saxifraga porophylla	Maxent	0.930294	0.937861	0	1.687323	0.123077	0.91
Sedum magellense	Maxent	0.9584	0.962032	0.01735	1.799154	0.125	0.89
Siler montanum	Maxent	0.924467	0.925438	0.039563	1.6679	0.105556	0.98
Viola eugeniae	Maxent	0.949999	0.955634	0.02053	1.764241	0.145455	0.951
Viola ferrarinii	Maxent	0.993356	0.992662	0.009278	1.969058	0.26	0.765
Adenostyles australis	RF	1	0.966029	0.047177	1.549124	0.366667	0.955
Aubrieta columnae	RF	1	0.972807	0.067982	1.722377	0.283333	0.897

Bellevalia webbiana	RF	1	0.978333	0.016667	1.678339	0.566667	0.966
Cardamine apennina	RF	1	0.9625	0.052679	1.487993	0.475	0.943
Carduus affinis	RF	1	0.97125	0.031528	1.630605	0.333333	0.958
Crocus biflorus	RF	1	0.931618	0.067964	1.141987	0.8329	0.998
Crocus etruscus	RF	1	1	0.057143	1.810257	0.75	0.921
Crocus imperati	RF	1	0.973	0.131667	1.569846	0.796667	0.952
Erysimum pseudorhaeticum	RF	1	0.938254	0.057302	1.336857	0.685714	0.98
Galium magellense	RF	1	0.99765	0.001282	1.88809	0.233333	0.932
Gypsophila arrostoi	RF	1	0.931159	0.047101	1.337575	0.742857	0.974
Helleborus viridis	RF	1	0.926389	0.056818	1.188107	0.790909	0.991
Klasea flavescens	RF	1	0.875397	0.101587	1.18826	0.9	0.909
Koeleria splendens	RF	0.99999	0.966554	0.037518	1.398999	0.424	0.982
Leontodon anomalus	RF	1	0.98	0.076111	1.680623	0.58	0.95
Linaria purpurea	RF	1	0.937876	0.082015	1.213004	0.617391	0.999
Ornithogalum etruscum	RF	1	0.875351	0.065088	1.245521	0.963333	0.959
Phyteuma ovatum	RF	1	0.994265	0.040323	1.845052	0.266667	0.971
Polygala flavescens	RF	0.999989	0.941006	0.065515	1.167787	0.734441	0.991
Ranunculus pollinensis	RF	1	0.99387	0.00977	1.876618	0.355556	0.984
Saxifraga porophylla	RF	1	0.987299	0.076655	1.614675	0.407692	0.979
Sedum magellense	RF	1	0.975521	0.020625	1.65215	0.308333	0.778
Siler montanum	RF	1	0.966065	0.050926	1.512667	0.522222	0.972
Viola eugeniae	RF	0.999948	0.987715	0.025061	1.770264	0.336364	0.952
Viola ferrarinii	RF	1	1	0	1.883214	0.4	0.969
Adenostyles australis	SVM	0.93862	0.923862	0.030558	1.552988	0.127778	0.898
Aubrieta columnae	SVM	0.95	0.935088	0.016194	1.711268	0.3	0.903
Bellevalia webbiana	SVM	0.961786	0.929167	0.018333	1.690825	0.283333	0.931
Cardamine apennina	SVM	0.910179	0.862054	0.061518	1.495564	0.1875	0.892
Carduus affinis	SVM	0.952778	0.951204	0.02334	1.715664	0.177778	0.89
Crocus biflorus	SVM	0.741749	0.705174	0.076638	1.101175	0.155628	0.944
Crocus etruscus	SVM	0.988571	0.971429	0.047143	1.753142	0.75	0.915
Crocus imperati	SVM	0.947802	0.89	0.044583	1.571816	0.396667	0.821
Erysimum pseudorhaeticum	SVM	0.881577	0.845873	0.048088	1.390029	0.171429	0.914
Galium magellense	SVM	0.984425	0.983974	0.002754	1.900498	0.158333	0.885
Gypsophila arrostoi	SVM	0.877391	0.754244	0.100127	1.261456	0.347619	0.946
Helleborus viridis	SVM	0.797889	0.738258	0.052939	1.194671	0.209091	0.866
Klasea flavescens	SVM	0.803175	0.618651	0.061984	1.181938	0.383333	0.84
Koeleria splendens	SVM	0.902576	0.874602	0.01273	1.28772	0.156	0.95
Leontodon anomalus	SVM	0.920085	0.865556	0.069444	1.54879	0.36	0.961
Linaria purpurea	SVM	0.836364	0.796321	0.0552	1.115288	0.152174	0.865
Ornithogalum etruscum	SVM	0.882119	0.732807	0.071808	1.254546	0.39	0.908
Phyteuma ovatum	SVM	0.970674	0.97491	0.0453	1.791474	0.155556	0.739
Polygala flavescens	SVM	0.755683	0.734193	0.032155	1.096561	0.130731	0.89
Ranunculus pollinensis	SVM	0.981034	0.975862	0.01046	1.857797	0.222222	0.922
Saxifraga porophylla	SVM	0.933372	0.931843	0.02249	1.620984	0.092308	0.857
Sedum magellense	SVM	0.963482	0.964792	0.014226	1.762798	0.075	0.926
Siler montanum	SVM	0.933849	0.918194	0.036495	1.614161	0.166667	0.891
Viola eugeniae	SVM	0.942412	0.942752	0.0311	1.666113	0.136364	0.946
Viola ferrarinii	SVM	0.995098	0.991765	0.023529	1.951414	0.42	0.93
