# Supplementary material for: Assessing the Effectiveness of Correlative Ecological Niche Model Temporal Projection through Floristic Data
Source: Biology (Basel). 2022 Aug 14;11(8):1219. doi: 10.3390/biology11081219 (PMC9405103; doi:10.3390/biology11081219)
Supplement: Supplementary file 1 [file biology-11-01219-s001.zip › Dolci & Peruzzi Supplementary Material S.1-9.zip!/Supplementary material S.7.pdf]

Supplementary material S.7

Complete list of evaluations metrics acquired in all modeling procedures based on the use of 35 environmental variables than converted into 3 layers by applying dimensionality reduction technique (PCA). Table S.7.A reports all results for procedures based on historical data. Table S.7.B reports all results for procedures based on modern data.

Table S.7.A. Complete list of evaluation metrics calculated for all procedures based on 35 environmental variables (plus dimensionality reduction via PCA) and historical data.

Species	Method	AUC_train (past)	AUC_test (past)	AUC_diff (past)	pROC (past)	OR_p10 (past)	CBI (projected)
Adenostyles australis	Bioclim	0.883	0.847	0.040	1.226	0.172	0.973
Aubrieta columnae	Bioclim	0.962	0.693	0.269	1.130	0.550	0.882
Bellevalia webbiana	Bioclim	0.839	0.775	0.083	1.361	0.167	0.589
Cardamine apennina	Bioclim	0.804	0.713	0.095	1.176	0.238	0.077
Carduus affinis	Bioclim	0.870	0.819	0.051	1.254	0.217	0.861
Crocus biflorus	Bioclim	0.696	0.681	0.023	1.106	0.168	0.623
Crocus etruscus	Bioclim	0.994	0.621	0.373	1.248	0.750	0.715
Crocus imperati	Bioclim	0.848	0.698	0.151	1.180	0.387	0.760
Erysimum pseudorhaeticum	Bioclim	0.779	0.723	0.067	1.178	0.143	0.685
Galium magellense	Bioclim	0.985	0.884	0.102	1.341	0.208	0.831
Gypsophila arrostoi	Bioclim	0.839	0.740	0.122	1.297	0.226	0.606
Helleborus viridis	Bioclim	0.852	0.785	0.068	1.236	0.127	0.908
Klasea flavescens	Bioclim	0.749	0.663	0.089	1.237	0.287	0.603
Koeleria splendens	Bioclim	0.836	0.796	0.041	1.132	0.180	0.986
Leontodon anomalus	Bioclim	0.930	0.770	0.164	1.261	0.340	0.121
Linaria purpurea	Bioclim	0.737	0.696	0.049	1.137	0.234	0.967
Ornithogalum etruscum	Bioclim	0.707	0.582	0.131	1.120	0.370	0.726
Phyteuma ovatum	Bioclim	0.849	0.772	0.090	1.277	0.233	0.937
Polygala flavescens	Bioclim	0.729	0.699	0.035	1.121	0.130	-0.280
Ranunculus pollinensis	Bioclim	0.936	0.834	0.103	1.269	0.222	0.953
Sedum magellense	Bioclim	0.940	0.880	0.061	1.397	0.133	0.863
Siler montanum	Bioclim	0.817	0.754	0.064	1.110	0.206	0.995
Saxifraga porophylla	Bioclim	0.932	0.856	0.082	1.322	0.185	0.731
Viola eugeniae	Bioclim	0.942	0.838	0.103	1.200	0.218	0.805
Viola ferrarinii	Bioclim	0.956	0.685	0.271	1.128	0.560	0.783
Adenostyles australis	Biomod2	0.981	0.901	0.080	1.502	0.317	0.438
Aubrieta columnae	Biomod2	0.998	0.958	0.041	1.817	0.500	0.621
Bellevalia webbiana	Biomod2	0.998	0.777	0.222	1.445	0.683	0.567
Cardamine apennina	Biomod2	0.985	0.917	0.068	1.625	0.475	0.471
Carduus affinis	Biomod2	0.987	0.909	0.078	1.460	0.372	-0.027
Crocus biflorus	Biomod2	0.959	0.778	0.181	1.221	0.523	0.287
Crocus etruscus	Biomod2	1.000	0.929	0.071	1.856	0.550	0.199
Crocus imperati	Biomod2	0.995	0.875	0.121	1.657	0.610	0.553

Erysimum pseudorhaeticum	Biomod2	0.977	0.805	0.172	1.345	0.636	0.892
Galium magellense	Biomod2	1.000	0.994	0.006	1.946	0.425	0.466
Gypsophila arrostoi	Biomod2	0.999	0.800	0.199	1.472	0.983	0.351
Helleborus viridis	Biomod2	0.983	0.881	0.102	1.494	0.373	0.400
Klasea flavescens	Biomod2	0.999	0.584	0.415	1.210	1.000	0.819
Koeleria splendens	Biomod2	0.963	0.880	0.083	1.346	0.364	0.836
Leontodon anomalus	Biomod2	1.000	0.870	0.130	1.584	0.780	0.517
Linaria purpurea	Biomod2	0.948	0.783	0.165	1.238	0.459	0.910
Ornithogalum etruscum	Biomod2	1.000	0.640	0.359	1.283	1.000	0.674
Phyteuma ovatum	Biomod2	0.994	0.750	0.244	1.279	0.744	0.453
Polygala flavescens	Biomod2	0.923	0.801	0.122	1.283	0.300	0.141
Ranunculus pollinensis	Biomod2	0.992	0.979	0.013	1.890	0.167	0.683
Sedum magellense	Biomod2	0.993	0.940	0.053	1.709	0.567	0.724
Siler montanum	Biomod2	0.975	0.863	0.112	1.503	0.517	0.789
Saxifraga porophylla	Biomod2	0.991	0.973	0.018	1.819	0.269	0.586
Viola eugeniae	Biomod2	0.993	0.974	0.019	1.860	0.400	0.301
Viola ferrarinii	Biomod2	1.000	0.986	0.014	1.933	0.280	0.663
Adenostyles australis	CTA	0.941	0.909	0.035	1.329	0.172	0.987
Aubrieta columnae	CTA	0.958	0.940	0.029	1.322	0.067	0.834
Bellevalia webbiana	CTA	0.908	0.788	0.127	1.183	0.233	0.134
Cardamine apennina	CTA	0.925	0.780	0.145	1.181	0.250	-0.086
Carduus affinis	CTA	0.960	0.928	0.036	1.305	0.122	0.663
Crocus biflorus	CTA	0.921	0.769	0.152	1.067	0.336	0.775
Crocus etruscus	CTA	0.964	0.964	0.000	1.000	0.000	0.851
Crocus imperati	CTA	0.912	0.830	0.089	1.218	0.233	0.773
Erysimum pseudorhaeticum	CTA	0.935	0.776	0.159	1.095	0.371	0.767
Galium magellense	CTA	0.995	0.974	0.021	1.453	0.100	0.963
Gypsophila arrostoi	CTA	0.925	0.721	0.204	1.121	0.360	0.914
Helleborus viridis	CTA	0.949	0.826	0.123	1.143	0.255	0.410
Klasea flavescens	CTA	0.854	0.718	0.154	1.151	0.295	0.136
Koeleria splendens	CTA	0.955	0.883	0.072	1.153	0.196	0.818
Leontodon anomalus	CTA	0.934	0.780	0.159	1.179	0.340	0.787
Linaria purpurea	CTA	0.945	0.811	0.134	1.078	0.329	0.917
Ornithogalum etruscum	CTA	0.885	0.715	0.171	1.112	0.490	0.558
Phyteuma ovatum	CTA	0.958	0.894	0.068	1.317	0.200	0.627
Polygala flavescens	CTA	0.903	0.828	0.075	1.166	0.235	0.151
Ranunculus pollinensis	CTA	0.981	0.919	0.062	1.265	0.144	0.989
Sedum magellense	CTA	0.979	0.946	0.034	1.349	0.125	0.970
Siler montanum	CTA	0.946	0.881	0.069	1.248	0.172	0.916
Saxifraga porophylla	CTA	0.969	0.931	0.045	1.331	0.169	0.866
Viola eugeniae	CTA	0.972	0.947	0.032	1.464	0.100	0.978
Viola ferrarinii	CTA	0.971	0.971	0.013	1.381	0.020	0.949
Adenostyles australis	Domain	0.886	0.834	0.052	1.348	0.189	0.946
Aubrieta columnae	Domain	0.952	0.930	0.023	1.799	0.317	0.844
Bellevalia webbiana	Domain	0.816	0.771	0.072	1.472	0.267	0.488

Cardamine apennina	Domain	0.753	0.759	0.076	1.374	0.113	0.238
Carduus affinis	Domain	0.863	0.834	0.034	1.386	0.161	0.916
Crocus biflorus	Domain	0.652	0.637	0.034	1.164	0.182	0.670
Crocus etruscus	Domain	0.994	0.961	0.033	1.923	0.750	0.455
Crocus imperati	Domain	0.820	0.749	0.084	1.455	0.270	0.938
Erysimum pseudorhaeticum	Domain	0.706	0.692	0.039	1.283	0.164	0.783
Galium magellense	Domain	0.980	0.976	0.007	1.893	0.108	0.916
Gypsophila arrostoi	Domain	0.816	0.710	0.111	1.374	0.348	0.558
Helleborus viridis	Domain	0.861	0.819	0.066	1.440	0.264	0.796
Klasea flavescens	Domain	0.734	0.690	0.092	1.381	0.282	0.102
Koeleria splendens	Domain	0.804	0.807	0.010	1.279	0.112	0.852
Leontodon anomalus	Domain	0.933	0.907	0.038	1.712	0.240	0.935
Linaria purpurea	Domain	0.717	0.687	0.033	1.179	0.163	0.981
Ornithogalum etruscum	Domain	0.671	0.547	0.145	1.211	0.350	0.528
Phyteuma ovatum	Domain	0.852	0.830	0.044	1.444	0.200	0.594
Polygala flavescens	Domain	0.701	0.680	0.026	1.133	0.130	-0.136
Ranunculus pollinensis	Domain	0.933	0.937	0.006	1.745	0.067	0.852
Sedum magellense	Domain	0.934	0.919	0.017	1.718	0.242	0.727
Siler montanum	Domain	0.797	0.784	0.023	1.234	0.189	0.981
Saxifraga porophylla	Domain	0.936	0.928	0.019	1.704	0.131	0.839
Viola eugeniae	Domain	0.916	0.906	0.025	1.613	0.182	0.826
Viola ferrarinii	Domain	0.945	0.884	0.068	1.644	0.260	0.969
Adenostyles australis	FDA	0.912	0.898	0.023	1.503	0.150	0.755
Aubrieta columnae	FDA	0.931	0.935	0.027	1.767	0.217	0.981
Bellevalia webbiana	FDA	0.808	0.785	0.039	1.507	0.200	-0.268
Cardamine apennina	FDA	0.691	0.628	0.070	1.268	0.200	-0.583
Carduus affinis	FDA	0.909	0.909	0.013	1.561	0.183	0.902
Crocus biflorus	FDA	0.642	0.613	0.048	1.139	0.191	0.178
Crocus etruscus	FDA	0.989	0.986	0.011	1.432	0.550	0.619
Crocus imperati	FDA	0.874	0.882	0.018	1.665	0.200	0.569
Erysimum pseudorhaeticum	FDA	0.801	0.813	0.020	1.340	0.107	0.922
Galium magellense	FDA	0.994	0.994	0.002	1.851	0.150	0.993
Gypsophila arrostoi	FDA	0.631	0.646	0.014	1.352	0.229	0.245
Helleborus viridis	FDA	0.908	0.874	0.042	1.511	0.273	0.352
Klasea flavescens	FDA	0.646	0.609	0.078	1.241	0.250	-0.142
Koeleria splendens	FDA	0.863	0.841	0.023	1.383	0.132	0.905
Leontodon anomalus	FDA	0.864	0.899	0.007	1.641	0.180	0.965
Linaria purpurea	FDA	0.781	0.776	0.024	1.233	0.154	0.928
Ornithogalum etruscum	FDA	0.674	0.585	0.103	1.260	0.423	0.635
Phyteuma ovatum	FDA	0.910	0.897	0.031	1.593	0.222	0.660
Polygala flavescens	FDA	0.736	0.733	0.014	1.225	0.119	0.415
Ranunculus pollinensis	FDA	0.958	0.948	0.015	1.733	0.133	0.984
Sedum magellense	FDA	0.961	0.955	0.009	1.730	0.150	0.973
Siler montanum	FDA	0.878	0.869	0.024	1.382	0.156	0.928
Saxifraga porophylla	FDA	0.942	0.931	0.018	1.608	0.169	0.980

Viola eugeniae	FDA	0.958	0.967	0.004	1.831	0.118	0.980
Viola ferrarinii	FDA	0.981	0.969	0.025	1.721	0.220	0.980
Adenostyles australis	GAM	0.906	0.863	0.046	1.328	0.178	0.778
Aubrieta columnae	GAM	0.934	0.821	0.118	1.511	0.400	0.923
Bellevalia webbiana	GAM	0.801	0.796	0.042	1.498	0.183	0.774
Cardamine apennina	GAM	0.852	0.762	0.099	1.331	0.225	-0.243
Carduus affinis	GAM	0.929	0.875	0.054	1.358	0.222	0.804
Crocus biflorus	GAM	0.775	0.722	0.055	1.192	0.186	0.920
Crocus etruscus	GAM	0.989	0.968	0.022	1.937	0.450	0.924
Crocus imperati	GAM	0.907	0.827	0.083	1.565	0.363	0.710
Erysimum pseudorhaeticum	GAM	0.847	0.773	0.079	1.301	0.279	0.116
Galium magellense	GAM	0.984	0.987	0.003	1.923	0.083	0.932
Gypsophila arrostoi	GAM	0.820	0.638	0.182	1.323	0.508	0.700
Helleborus viridis	GAM	0.894	0.855	0.054	1.455	0.209	0.976
Klasea flavescens	GAM	0.748	0.529	0.219	1.197	0.435	0.771
Koeleria splendens	GAM	0.874	0.849	0.027	1.334	0.168	0.951
Leontodon anomalus	GAM	0.922	0.931	0.023	1.773	0.280	0.943
Linaria purpurea	GAM	0.810	0.760	0.053	1.239	0.185	0.870
Ornithogalum etruscum	GAM	0.777	0.659	0.118	1.330	0.393	-0.026
Phyteuma ovatum	GAM	0.902	0.877	0.046	1.527	0.189	0.528
Polygala flavescens	GAM	0.780	0.756	0.033	1.232	0.142	0.742
Ranunculus pollinensis	GAM	0.943	0.912	0.035	1.630	0.222	0.967
Sedum magellense	GAM	0.948	0.936	0.024	1.707	0.125	0.883
Siler montanum	GAM	0.862	0.843	0.027	1.331	0.139	0.943
Saxifraga porophylla	GAM	0.946	0.899	0.052	1.520	0.238	0.952
Viola eugeniae	GAM	0.963	0.961	0.010	1.796	0.127	0.832
Viola ferrarinii	GAM	0.967	0.930	0.047	1.788	0.280	0.724
Adenostyles australis	GLM	0.919	0.876	0.045	1.440	0.250	0.772
Aubrieta columnae	GLM	0.936	0.933	0.025	1.780	0.233	0.584
Bellevalia webbiana	GLM	0.764	0.789	0.019	1.522	0.117	0.583
Cardamine apennina	GLM	0.673	0.666	0.042	1.298	0.163	-0.365
Carduus affinis	GLM	0.910	0.926	0.004	1.608	0.094	0.669
Crocus biflorus	GLM	0.614	0.600	0.030	1.162	0.150	0.722
Crocus etruscus	GLM	0.959	0.946	0.022	1.904	0.450	0.885
Crocus imperati	GLM	0.858	0.879	0.015	1.698	0.103	0.464
Erysimum pseudorhaeticum	GLM	0.788	0.801	0.012	1.333	0.143	0.780
Galium magellense	GLM	0.986	0.988	0.002	1.935	0.100	0.948
Gypsophila arrostoi	GLM	0.657	0.607	0.064	1.305	0.264	0.211
Helleborus viridis	GLM	0.894	0.885	0.013	1.586	0.109	0.160
Klasea flavescens	GLM	0.586	0.494	0.100	1.207	0.330	0.218
Koeleria splendens	GLM	0.853	0.849	0.019	1.375	0.116	0.867
Leontodon anomalus	GLM	0.830	0.838	0.035	1.617	0.180	0.201
Linaria purpurea	GLM	0.753	0.758	0.013	1.224	0.085	0.813
Ornithogalum etruscum	GLM	0.686	0.631	0.089	1.306	0.227	0.537
Phyteuma ovatum	GLM	0.887	0.921	0.005	1.673	0.133	0.733

Polygala flavescens	GLM	0.717	0.720	0.012	1.239	0.112	-0.293
Ranunculus pollinensis	GLM	0.946	0.939	0.018	1.744	0.167	0.939
Sedum magellense	GLM	0.956	0.947	0.015	1.735	0.167	0.783
Siler montanum	GLM	0.866	0.872	0.017	1.387	0.156	0.810
Saxifraga porophylla	GLM	0.932	0.932	0.015	1.649	0.177	0.847
Viola eugeniae	GLM	0.957	0.956	0.009	1.782	0.118	0.767
Viola ferrarinii	GLM	0.959	0.964	0.017	1.874	0.140	0.819
Adenostyles australis	KDE	0.908	0.872	0.037	1.369	0.178	0.939
Aubrieta columnae	KDE	0.953	0.932	0.033	1.783	0.383	0.762
Bellevalia webbiana	KDE	0.822	0.789	0.054	1.484	0.200	-0.366
Cardamine apennina	KDE	0.780	0.739	0.102	1.314	0.213	-0.538
Carduus affinis	KDE	0.895	0.890	0.027	1.461	0.178	0.962
Crocus biflorus	KDE	0.671	0.643	0.036	1.148	0.136	0.792
Crocus etruscus	KDE	0.979	0.956	0.035	1.916	0.600	0.781
Crocus imperati	KDE	0.809	0.811	0.037	1.542	0.167	0.811
Erysimum pseudorhaeticum	KDE	0.724	0.645	0.078	1.231	0.214	0.975
Galium magellense	KDE	0.974	0.943	0.033	1.622	0.233	0.735
Gypsophila arrostoi	KDE	0.814	0.719	0.096	1.347	0.300	0.093
Helleborus viridis	KDE	0.881	0.795	0.090	1.314	0.309	0.847
Klasea flavescens	KDE	0.772	0.705	0.082	1.346	0.183	0.220
Koeleria splendens	KDE	0.833	0.783	0.055	1.218	0.196	0.992
Leontodon anomalus	KDE	0.928	0.851	0.084	1.574	0.320	0.893
Linaria purpurea	KDE	0.646	0.561	0.093	1.078	0.183	0.985
Ornithogalum etruscum	KDE	0.643	0.567	0.105	1.240	0.267	0.174
Phyteuma ovatum	KDE	0.869	0.867	0.023	1.539	0.089	0.897
Polygala flavescens	KDE	0.650	0.614	0.039	1.096	0.114	0.332
Ranunculus pollinensis	KDE	0.929	0.884	0.053	1.544	0.211	0.917
Sedum magellense	KDE	0.945	0.925	0.024	1.645	0.158	0.891
Siler montanum	KDE	0.760	0.755	0.027	1.176	0.156	0.984
Saxifraga porophylla	KDE	0.937	0.904	0.043	1.480	0.146	0.879
Viola eugeniae	KDE	0.921	0.872	0.049	1.522	0.273	0.963
Viola ferrarinii	KDE	0.943	0.916	0.038	1.721	0.200	0.236
Adenostyles australis	MARS	0.911	0.869	0.053	1.333	0.183	0.986
Aubrieta columnae	MARS	0.944	0.845	0.109	1.497	0.233	0.876
Bellevalia webbiana	MARS	0.809	0.625	0.185	1.149	0.267	0.327
Cardamine apennina	MARS	0.904	0.757	0.148	1.346	0.363	-0.312
Carduus affinis	MARS	0.928	0.865	0.068	1.316	0.244	0.937
Crocus biflorus	MARS	0.800	0.716	0.084	1.177	0.250	0.906
Crocus etruscus	MARS	0.983	0.905	0.089	1.818	0.500	0.478
Crocus imperati	MARS	0.873	0.708	0.174	1.264	0.293	0.740
Erysimum pseudorhaeticum	MARS	0.805	0.755	0.060	1.250	0.221	0.720
Galium magellense	MARS	0.990	0.946	0.046	1.694	0.258	0.892
Gypsophila arrostoi	MARS	0.824	0.666	0.158	1.256	0.271	0.511
Helleborus viridis	MARS	0.861	0.814	0.056	1.261	0.145	0.809
Klasea flavescens	MARS	0.638	0.539	0.109	1.389	0.293	-0.333
Koeleria splendens	MARS	0.895	0.864	0.040	1.296	0.140	0.980

Leontodon anomalus	MARS	0.897	0.876	0.063	1.583	0.240	0.970
Linaria purpurea	MARS	0.811	0.728	0.085	1.143	0.221	0.988
Ornithogalum etruscum	MARS	0.679	0.529	0.151	1.100	0.253	0.921
Phyteuma ovatum	MARS	0.910	0.839	0.080	1.390	0.233	0.967
Polygala flavescens	MARS	0.804	0.772	0.034	1.234	0.135	0.564
Ranunculus pollinensis	MARS	0.943	0.908	0.044	1.552	0.156	0.978
Sedum magellense	MARS	0.961	0.923	0.041	1.626	0.158	0.748
Siler montanum	MARS	0.890	0.842	0.052	1.274	0.178	0.969
Saxifraga porophylla	MARS	0.945	0.903	0.049	1.488	0.154	0.713
Viola eugeniae	MARS	0.951	0.875	0.087	1.500	0.273	0.968
Viola ferrarinii	MARS	0.952	0.907	0.063	1.671	0.300	0.845
Adenostyles australis	Maxent	0.919	0.919	0.012	1.566	0.111	0.928
Aubrieta columnae	Maxent	0.958	0.932	0.036	1.756	0.300	0.945
Bellevalia webbiana	Maxent	0.812	0.791	0.072	1.502	0.183	0.715
Cardamine apennina	Maxent	0.842	0.763	0.094	1.361	0.225	-0.442
Carduus affinis	Maxent	0.926	0.925	0.016	1.553	0.122	0.392
Crocus biflorus	Maxent	0.762	0.730	0.037	1.237	0.177	0.856
Crocus etruscus	Maxent	0.954	0.949	0.018	1.906	0.400	0.839
Crocus imperati	Maxent	0.873	0.834	0.052	1.616	0.317	0.890
Erysimum pseudorhaeticum	Maxent	0.839	0.831	0.025	1.419	0.171	-0.512
Galium magellense	Maxent	0.991	0.986	0.006	1.925	0.225	0.957
Gypsophila arrostoi	Maxent	0.791	0.731	0.078	1.438	0.326	0.542
Helleborus viridis	Maxent	0.905	0.872	0.037	1.527	0.245	0.962
Klasea flavescens	Maxent	0.747	0.603	0.149	1.266	0.342	-0.193
Koeleria splendens	Maxent	0.883	0.874	0.025	1.420	0.136	0.596
Leontodon anomalus	Maxent	0.914	0.932	0.024	1.798	0.180	0.816
Linaria purpurea	Maxent	0.818	0.797	0.033	1.303	0.182	0.987
Ornithogalum etruscum	Maxent	0.739	0.657	0.109	1.344	0.363	0.303
Phyteuma ovatum	Maxent	0.909	0.900	0.034	1.608	0.189	0.251
Polygala flavescens	Maxent	0.801	0.772	0.032	1.265	0.130	0.886
Ranunculus pollinensis	Maxent	0.958	0.954	0.015	1.794	0.133	0.980
Sedum magellense	Maxent	0.963	0.947	0.023	1.719	0.167	0.975
Siler montanum	Maxent	0.869	0.888	0.016	1.481	0.117	0.848
Saxifraga porophylla	Maxent	0.956	0.953	0.019	1.742	0.169	0.984
Viola eugeniae	Maxent	0.970	0.953	0.023	1.759	0.191	0.772
Viola ferrarinii	Maxent	0.976	0.949	0.036	1.812	0.280	0.651
Adenostyles australis	RF	1.000	0.959	0.041	1.458	0.372	0.978
Aubrieta columnae	RF	1.000	0.988	0.012	1.808	0.517	0.963
Bellevalia webbiana	RF	1.000	0.975	0.025	1.519	0.650	0.276
Cardamine apennina	RF	1.000	0.910	0.090	1.330	0.725	0.070
Carduus affinis	RF	1.000	0.939	0.061	1.377	0.417	0.929
Crocus biflorus	RF	1.000	0.908	0.092	1.120	0.768	0.889
Crocus etruscus	RF	1.000	0.971	0.029	1.820	0.650	0.969
Crocus imperati	RF	1.000	0.939	0.061	1.567	0.803	0.902
Erysimum pseudorhaeticum	RF	1.000	0.940	0.060	1.324	0.729	0.926

Galium magellense	RF	1.000	0.999	0.001	1.921	0.292	0.982
Gypsophila arrostoi	RF	1.000	0.938	0.062	1.431	0.848	0.870
Helleborus viridis	RF	1.000	0.973	0.027	1.523	0.564	0.667
Klasea flavescens	RF	1.000	0.899	0.101	1.238	0.890	0.813
Koeleria splendens	RF	1.000	0.973	0.027	1.408	0.488	0.991
Leontodon anomalus	RF	1.000	0.958	0.042	1.675	0.420	0.892
Linaria purpurea	RF	1.000	0.962	0.038	1.262	0.654	0.996
Ornithogalum etruscum	RF	1.000	0.963	0.037	1.337	0.783	0.810
Phyteuma ovatum	RF	1.000	0.954	0.046	1.517	0.422	0.837
Polygala flavescens	RF	1.000	0.946	0.054	1.231	0.707	0.319
Ranunculus pollinensis	RF	1.000	0.988	0.012	1.743	0.400	0.974
Sedum magellense	RF	1.000	0.991	0.009	1.759	0.350	0.958
Siler montanum	RF	1.000	0.969	0.031	1.393	0.494	0.967
Saxifraga porophylla	RF	1.000	0.981	0.019	1.633	0.346	0.949
Viola eugeniae	RF	1.000	0.997	0.003	1.816	0.400	0.543
Viola ferrarinii	RF	1.000	0.989	0.011	1.866	0.420	0.097
Adenostyles australis	SVM	0.919	0.915	0.014	1.471	0.117	0.993
Aubrieta columnae	SVM	0.974	0.980	0.003	1.855	0.200	0.943
Bellevalia webbiana	SVM	0.895	0.825	0.080	1.471	0.300	0.367
Cardamine apennina	SVM	0.877	0.806	0.076	1.343	0.263	-0.178
Carduus affinis	SVM	0.935	0.925	0.021	1.494	0.194	0.925
Crocus biflorus	SVM	0.817	0.763	0.057	1.157	0.195	0.717
Crocus etruscus	SVM	1.000	1.000	0.000	1.976	0.300	0.961
Crocus imperati	SVM	0.919	0.880	0.047	1.605	0.377	0.893
Erysimum pseudorhaeticum	SVM	0.869	0.818	0.059	1.294	0.250	0.825
Galium magellense	SVM	0.990	0.991	0.002	1.934	0.125	0.957
Gypsophila arrostoi	SVM	0.916	0.873	0.052	1.552	0.248	0.757
Helleborus viridis	SVM	0.936	0.893	0.046	1.476	0.227	0.823
Klasea flavescens	SVM	0.877	0.776	0.104	1.340	0.363	0.634
Koeleria splendens	SVM	0.894	0.886	0.022	1.355	0.120	0.951
Leontodon anomalus	SVM	0.977	0.949	0.031	1.736	0.320	0.947
Linaria purpurea	SVM	0.851	0.812	0.040	1.219	0.144	0.980
Ornithogalum etruscum	SVM	0.876	0.708	0.167	1.212	0.467	0.593
Phyteuma ovatum	SVM	0.936	0.909	0.038	1.520	0.189	0.900
Polygala flavescens	SVM	0.810	0.788	0.025	1.198	0.123	0.006
Ranunculus pollinensis	SVM	0.967	0.951	0.019	1.756	0.133	0.976
Sedum magellense	SVM	0.967	0.958	0.013	1.742	0.175	0.916
Siler montanum	SVM	0.921	0.889	0.033	1.372	0.200	0.986
Saxifraga porophylla	SVM	0.969	0.937	0.036	1.573	0.138	0.968
Viola eugeniae	SVM	0.976	0.980	0.002	1.843	0.164	0.483
Viola ferrarinii	SVM	0.988	0.960	0.032	1.768	0.380	0.769

Table S.7.B. Complete list of evaluation metrics calculated for all procedures based on 35 environmental variables (plus dimensionality reduction via PCA) and modern data.

Species	Method	AUC_train (present)	AUC_test (present)	AUC_diff (present)	pROC (present)	OR_p10 (present)	CBI (present)
Adenostyles australis	Bioclim	0.866	0.799	0.040	1.157	0.172	0.302
Aubrieta columnae	Bioclim	0.952	0.775	0.269	1.302	0.367	0.586
Bellevalia webbiana	Bioclim	0.902	0.761	0.083	1.292	0.317	0.453
Cardamine apennina	Bioclim	0.777	0.727	0.095	1.279	0.163	-0.506
Carduus affinis	Bioclim	0.923	0.828	0.051	1.162	0.306	0.492
Crocus biflorus	Bioclim	0.648	0.617	0.023	1.071	0.229	0.429
Crocus etruscus	Bioclim	0.985	0.567	0.373	1.142	0.850	-0.256
Crocus imperati	Bioclim	0.857	0.797	0.151	1.391	0.213	0.733
Erysimum pseudorhaeticum	Bioclim	0.799	0.731	0.067	1.166	0.219	0.023
Galium magellense	Bioclim	0.988	0.907	0.102	1.432	0.167	0.435
Gypsophila arrostoi	Bioclim	0.859	0.739	0.122	1.235	0.271	0.549
Helleborus viridis	Bioclim	0.774	0.694	0.068	1.141	0.218	0.212
Klasea flavescens	Bioclim	0.758	0.644	0.089	1.179	0.300	0.855
Koeleria splendens	Bioclim	0.859	0.824	0.041	1.154	0.192	0.640
Leontodon anomalus	Bioclim	0.880	0.742	0.164	1.316	0.300	0.507
Linaria purpurea	Bioclim	0.744	0.674	0.049	1.060	0.235	0.852
Ornithogalum etruscum	Bioclim	0.714	0.615	0.131	1.169	0.300	0.401
Phyteuma ovatum	Bioclim	0.870	0.787	0.090	1.245	0.189	0.854
Polygala flavescens	Bioclim	0.756	0.727	0.035	1.177	0.123	0.827
Ranunculus pollinensis	Bioclim	0.951	0.790	0.103	1.192	0.322	0.471
Saxifraga porophylla	Bioclim	0.925	0.813	0.082	1.192	0.238	0.257
Sedum magellense	Bioclim	0.943	0.873	0.061	1.359	0.150	0.460
Siler montanum	Bioclim	0.871	0.830	0.064	1.277	0.128	0.856
Viola eugeniae	Bioclim	0.921	0.823	0.103	1.205	0.236	0.771
Viola ferrarinii	Bioclim	0.981	0.785	0.271	1.324	0.400	0.478
Adenostyles australis	Biomod2	0.979	0.942	0.080	1.664	0.361	0.988
Aubrieta columnae	Biomod2	0.997	0.969	0.041	1.868	0.683	0.934
Bellevalia webbiana	Biomod2	1.000	0.921	0.222	1.750	0.983	0.961
Cardamine apennina	Biomod2	0.998	0.918	0.068	1.676	0.650	0.933
Carduus affinis	Biomod2	0.991	0.946	0.078	1.731	0.528	0.964
Crocus biflorus	Biomod2	0.961	0.671	0.181	1.174	0.786	0.992
Crocus etruscus	Biomod2	1.000	0.994	0.071	1.970	0.950	0.853
Crocus imperati	Biomod2	0.999	0.915	0.121	1.744	0.813	0.907
Erysimum pseudorhaeticum	Biomod2	0.982	0.853	0.172	1.507	0.807	0.957
Galium magellense	Biomod2	0.999	0.994	0.006	1.948	0.242	0.922
Gypsophila arrostoi	Biomod2	0.999	0.886	0.199	1.658	1.000	0.973
Helleborus viridis	Biomod2	0.979	0.763	0.102	1.385	0.891	0.963
Klasea flavescens	Biomod2	0.998	0.601	0.415	1.196	0.833	0.974
Koeleria splendens	Biomod2	0.956	0.877	0.083	1.324	0.240	0.960
Leontodon anomalus	Biomod2	0.995	0.923	0.130	1.791	0.800	0.910
Linaria purpurea	Biomod2	0.955	0.788	0.165	1.210	0.543	0.983
Ornithogalum etruscum	Biomod2	0.999	0.749	0.359	1.403	1.000	0.958
Phyteuma ovatum	Biomod2	0.995	0.984	0.244	1.892	0.289	0.689
Polygala flavescens	Biomod2	0.946	0.780	0.122	1.277	0.347	0.994

Ranunculus pollinensis	Biomod2	0.993	0.980	0.013	1.908	0.600	0.989
Saxifraga porophylla	Biomod2	0.989	0.958	0.018	1.768	0.454	0.918
Sedum magellense	Biomod2	0.994	0.971	0.053	1.814	0.392	0.936
Siler montanum	Biomod2	0.987	0.890	0.112	1.477	0.678	0.991
Viola eugeniae	Biomod2	0.990	0.960	0.019	1.762	0.382	0.963
Viola ferrarinii	Biomod2	1.000	0.996	0.014	1.966	0.600	0.917
Adenostyles australis	CTA	0.955	0.908	0.035	1.309	0.206	0.945
Aubrieta columnae	CTA	0.954	0.913	0.029	1.313	0.133	0.861
Bellevalia webbiana	CTA	0.933	0.793	0.127	1.274	0.317	0.956
Cardamine apennina	CTA	0.945	0.853	0.145	1.222	0.238	0.972
Carduus affinis	CTA	0.977	0.956	0.036	1.371	0.133	0.802
Crocus biflorus	CTA	0.906	0.771	0.152	1.064	0.275	0.972
Crocus etruscus	CTA	0.914	0.889	0.000	1.000	0.050	0.733
Crocus imperati	CTA	0.930	0.852	0.089	1.236	0.197	0.935
Erysimum pseudorhaeticum	CTA	0.925	0.823	0.159	1.190	0.278	0.985
Galium magellense	CTA	0.995	0.975	0.021	1.409	0.100	0.764
Gypsophila arrostoi	CTA	0.909	0.804	0.204	1.197	0.229	0.949
Helleborus viridis	CTA	0.923	0.774	0.123	1.129	0.345	0.955
Klasea flavescens	CTA	0.903	0.721	0.154	1.164	0.367	0.964
Koeleria splendens	CTA	0.948	0.876	0.072	1.152	0.200	0.958
Leontodon anomalus	CTA	0.937	0.844	0.159	1.204	0.260	0.956
Linaria purpurea	CTA	0.943	0.801	0.134	1.099	0.304	0.993
Ornithogalum etruscum	CTA	0.886	0.718	0.171	1.156	0.367	0.940
Phyteuma ovatum	CTA	0.977	0.960	0.068	1.384	0.144	0.832
Polygala flavescens	CTA	0.896	0.786	0.075	1.121	0.273	0.963
Ranunculus pollinensis	CTA	0.982	0.898	0.062	1.318	0.211	0.909
Saxifraga porophylla	CTA	0.977	0.930	0.045	1.337	0.177	0.952
Sedum magellense	CTA	0.977	0.932	0.034	1.320	0.200	0.861
Siler montanum	CTA	0.958	0.888	0.069	1.220	0.233	0.975
Viola eugeniae	CTA	0.956	0.931	0.032	1.410	0.118	0.860
Viola ferrarinii	CTA	0.994	0.934	0.013	1.176	0.200	0.942
Adenostyles australis	Domain	0.845	0.822	0.052	1.342	0.150	0.946
Aubrieta columnae	Domain	0.939	0.934	0.023	1.792	0.150	0.948
Bellevalia webbiana	Domain	0.888	0.822	0.072	1.570	0.350	0.898
Cardamine apennina	Domain	0.757	0.713	0.076	1.405	0.200	0.652
Carduus affinis	Domain	0.916	0.907	0.034	1.554	0.133	0.953
Crocus biflorus	Domain	0.563	0.544	0.034	1.086	0.133	0.742
Crocus etruscus	Domain	0.982	0.788	0.033	1.615	0.650	0.813
Crocus imperati	Domain	0.809	0.885	0.084	1.657	0.053	0.480
Erysimum pseudorhaeticum	Domain	0.724	0.676	0.039	1.172	0.187	0.975
Galium magellense	Domain	0.987	0.985	0.007	1.942	0.167	0.982
Gypsophila arrostoi	Domain	0.843	0.818	0.111	1.542	0.171	0.835
Helleborus viridis	Domain	0.747	0.702	0.066	1.297	0.264	0.917
Klasea flavescens	Domain	0.727	0.664	0.092	1.366	0.300	-0.164
Koeleria splendens	Domain	0.847	0.837	0.010	1.304	0.120	0.838
Leontodon anomalus	Domain	0.888	0.842	0.038	1.607	0.380	0.913
Linaria purpurea	Domain	0.737	0.738	0.033	1.266	0.113	0.997
Ornithogalum etruscum	Domain	0.697	0.648	0.145	1.323	0.250	0.092

Phyteuma ovatum	Domain	0.874	0.856	0.044	1.524	0.200	0.799
Polygala flavescens	Domain	0.726	0.712	0.026	1.187	0.120	0.916
Ranunculus pollinensis	Domain	0.949	0.923	0.006	1.730	0.267	0.992
Saxifraga porophylla	Domain	0.929	0.907	0.019	1.652	0.177	0.961
Sedum magellense	Domain	0.948	0.951	0.017	1.816	0.108	0.993
Siler montanum	Domain	0.869	0.856	0.023	1.502	0.128	0.996
Viola eugeniae	Domain	0.920	0.911	0.025	1.681	0.182	0.968
Viola ferrarinii	Domain	0.986	0.980	0.068	1.935	0.360	0.918
Adenostyles australis	FDA	0.922	0.916	0.023	1.565	0.156	0.916
Aubrieta columnae	FDA	0.942	0.900	0.027	1.713	0.350	0.794
Bellevalia webbiana	FDA	0.801	0.747	0.039	1.435	0.333	0.699
Cardamine apennina	FDA	0.706	0.692	0.070	1.317	0.138	0.593
Carduus affinis	FDA	0.959	0.954	0.013	1.726	0.167	0.892
Crocus biflorus	FDA	0.586	0.593	0.048	1.095	0.130	0.660
Crocus etruscus	FDA	0.989	0.936	0.011	1.550	0.450	0.821
Crocus imperati	FDA	0.910	0.884	0.018	1.612	0.240	0.833
Erysimum pseudorhaeticum	FDA	0.812	0.826	0.020	1.433	0.155	0.992
Galium magellense	FDA	0.989	0.992	0.002	1.793	0.125	0.642
Gypsophila arrostoi	FDA	0.663	0.598	0.014	1.173	0.271	0.177
Helleborus viridis	FDA	0.759	0.753	0.042	1.344	0.136	0.660
Klasea flavescens	FDA	0.643	0.562	0.078	1.209	0.317	-0.099
Koeleria splendens	FDA	0.873	0.855	0.023	1.310	0.128	0.990
Leontodon anomalus	FDA	0.916	0.862	0.007	1.581	0.360	0.853
Linaria purpurea	FDA	0.831	0.855	0.024	1.318	0.100	0.993
Ornithogalum etruscum	FDA	0.713	0.596	0.103	1.210	0.400	0.628
Phyteuma ovatum	FDA	0.942	0.884	0.031	1.521	0.278	0.520
Polygala flavescens	FDA	0.695	0.702	0.014	1.166	0.100	0.954
Ranunculus pollinensis	FDA	0.973	0.953	0.015	1.735	0.200	0.908
Saxifraga porophylla	FDA	0.949	0.948	0.018	1.684	0.123	0.897
Sedum magellense	FDA	0.958	0.967	0.009	1.798	0.100	0.897
Siler montanum	FDA	0.929	0.910	0.024	1.534	0.167	0.954
Viola eugeniae	FDA	0.949	0.945	0.004	1.711	0.182	0.897
Viola ferrarinii	FDA	0.980	0.976	0.025	1.770	0.280	0.897
Adenostyles australis	GAM	0.936	0.928	0.046	1.608	0.150	0.966
Aubrieta columnae	GAM	0.933	0.910	0.118	1.721	0.217	0.858
Bellevalia webbiana	GAM	0.855	0.806	0.042	1.563	0.333	0.847
Cardamine apennina	GAM	0.860	0.754	0.099	1.362	0.275	0.866
Carduus affinis	GAM	0.957	0.947	0.054	1.673	0.161	0.921
Crocus biflorus	GAM	0.719	0.636	0.055	1.139	0.204	0.944
Crocus etruscus	GAM	0.980	0.951	0.022	1.905	0.500	0.981
Crocus imperati	GAM	0.912	0.796	0.083	1.477	0.307	0.863
Erysimum pseudorhaeticum	GAM	0.836	0.787	0.079	1.439	0.282	0.795
Galium magellense	GAM	0.985	0.985	0.003	1.904	0.100	0.989
Gypsophila arrostoi	GAM	0.835	0.758	0.182	1.447	0.257	0.789
Helleborus viridis	GAM	0.791	0.778	0.054	1.413	0.136	0.549
Klasea flavescens	GAM	0.827	0.550	0.219	1.203	0.517	0.820
Koeleria splendens	GAM	0.884	0.830	0.027	1.186	0.184	0.934
Leontodon anomalus	GAM	0.899	0.878	0.023	1.672	0.280	0.975

Linaria purpurea	GAM	0.844	0.809	0.053	1.257	0.178	0.909
Ornithogalum etruscum	GAM	0.839	0.773	0.118	1.490	0.367	0.858
Phyteuma ovatum	GAM	0.952	0.932	0.046	1.684	0.189	0.899
Polygala flavescens	GAM	0.790	0.791	0.033	1.325	0.108	0.817
Ranunculus pollinensis	GAM	0.954	0.949	0.035	1.775	0.200	0.921
Saxifraga porophylla	GAM	0.952	0.937	0.052	1.681	0.192	0.901
Sedum magellense	GAM	0.963	0.950	0.024	1.740	0.142	0.972
Siler montanum	GAM	0.934	0.919	0.027	1.553	0.167	0.867
Viola eugeniae	GAM	0.940	0.915	0.010	1.606	0.155	0.979
Viola ferrarinii	GAM	0.986	0.987	0.047	1.955	0.240	0.966
Adenostyles australis	GLM	0.923	0.920	0.045	1.557	0.133	0.778
Aubrieta columnae	GLM	0.929	0.945	0.025	1.815	0.133	0.989
Bellevalia webbiana	GLM	0.760	0.761	0.019	1.493	0.133	0.703
Cardamine apennina	GLM	0.661	0.621	0.042	1.260	0.213	0.731
Carduus affinis	GLM	0.956	0.941	0.004	1.656	0.194	0.920
Crocus biflorus	GLM	0.587	0.554	0.030	1.092	0.143	0.674
Crocus etruscus	GLM	0.935	0.931	0.022	1.876	0.300	0.486
Crocus imperati	GLM	0.889	0.844	0.015	1.614	0.293	0.839
Erysimum pseudorhaeticum	GLM	0.813	0.819	0.012	1.464	0.158	0.918
Galium magellense	GLM	0.990	0.991	0.002	1.940	0.125	0.994
Gypsophila arrostoi	GLM	0.599	0.518	0.064	1.192	0.314	0.305
Helleborus viridis	GLM	0.766	0.750	0.013	1.379	0.182	0.319
Klasea flavescens	GLM	0.589	0.382	0.100	1.129	0.400	0.385
Koeleria splendens	GLM	0.867	0.847	0.019	1.297	0.144	0.850
Leontodon anomalus	GLM	0.870	0.867	0.035	1.656	0.300	0.987
Linaria purpurea	GLM	0.821	0.819	0.013	1.272	0.152	0.925
Ornithogalum etruscum	GLM	0.651	0.582	0.089	1.275	0.350	0.665
Phyteuma ovatum	GLM	0.913	0.908	0.005	1.607	0.167	0.946
Polygala flavescens	GLM	0.691	0.692	0.012	1.170	0.080	0.941
Ranunculus pollinensis	GLM	0.953	0.970	0.018	1.864	0.044	0.980
Saxifraga porophylla	GLM	0.945	0.938	0.015	1.662	0.146	0.984
Sedum magellense	GLM	0.959	0.960	0.015	1.777	0.125	0.973
Siler montanum	GLM	0.921	0.917	0.017	1.568	0.156	0.866
Viola eugeniae	GLM	0.939	0.947	0.009	1.751	0.118	0.995
Viola ferrarinii	GLM	0.989	0.985	0.017	1.946	0.320	0.989
Adenostyles australis	KDE	0.886	0.862	0.034	1.397	0.150	0.931
Aubrieta columnae	KDE	0.940	0.884	0.058	1.655	0.500	0.953
Bellevalia webbiana	KDE	0.873	0.780	0.093	1.490	0.500	0.823
Cardamine apennina	KDE	0.866	0.808	0.072	1.468	0.175	0.880
Carduus affinis	KDE	0.928	0.913	0.019	1.531	0.189	0.975
Crocus biflorus	KDE	0.624	0.620	0.026	1.078	0.145	0.939
Crocus etruscus	KDE	0.976	0.960	0.020	1.926	0.550	0.622
Crocus imperati	KDE	0.854	0.826	0.073	1.544	0.250	0.889
Erysimum pseudorhaeticum	KDE	0.712	0.654	0.058	1.219	0.193	0.903
Galium magellense	KDE	0.991	0.966	0.025	1.774	0.283	0.938
Gypsophila arrostoi	KDE	0.839	0.791	0.056	1.428	0.243	0.939
Helleborus viridis	KDE	0.785	0.733	0.059	1.277	0.191	0.966
Klasea flavescens	KDE	0.726	0.660	0.098	1.304	0.250	0.899

Koeleria splendens	KDE	0.864	0.841	0.030	1.269	0.096	0.968
Leontodon anomalus	KDE	0.920	0.853	0.075	1.592	0.340	0.995
Linaria purpurea	KDE	0.787	0.762	0.035	1.176	0.126	0.898
Ornithogalum etruscum	KDE	0.722	0.570	0.155	1.241	0.417	0.021
Phyteuma ovatum	KDE	0.953	0.938	0.021	1.713	0.178	0.994
Polygala flavescens	KDE	0.704	0.657	0.049	1.120	0.156	0.803
Ranunculus pollinensis	KDE	0.941	0.875	0.071	1.487	0.256	0.958
Saxifraga porophylla	KDE	0.956	0.923	0.033	1.632	0.223	0.966
Sedum magellense	KDE	0.961	0.948	0.016	1.751	0.167	0.833
Siler montanum	KDE	0.883	0.838	0.046	1.344	0.222	0.979
Viola eugeniae	KDE	0.934	0.893	0.048	1.541	0.209	0.980
Viola ferrarinii	KDE	0.975	0.897	0.083	1.659	0.280	0.974
Adenostyles australis	MARS	0.915	0.865	0.053	1.304	0.178	0.802
Aubrieta columnae	MARS	0.939	0.821	0.109	1.509	0.433	0.896
Bellevalia webbiana	MARS	0.856	0.771	0.185	1.456	0.433	0.754
Cardamine apennina	MARS	0.874	0.797	0.148	1.455	0.175	0.851
Carduus affinis	MARS	0.972	0.909	0.068	1.442	0.272	0.917
Crocus biflorus	MARS	0.702	0.605	0.084	1.094	0.207	0.807
Crocus etruscus	MARS	0.945	0.711	0.089	1.596	0.750	0.657
Crocus imperati	MARS	0.918	0.697	0.174	1.268	0.457	0.829
Erysimum pseudorhaeticum	MARS	0.833	0.721	0.060	1.231	0.328	0.744
Galium magellense	MARS	0.991	0.971	0.046	1.804	0.192	0.899
Gypsophila arrostoi	MARS	0.764	0.702	0.158	1.379	0.294	0.869
Helleborus viridis	MARS	0.822	0.757	0.056	1.327	0.173	0.928
Klasea flavescens	MARS	0.764	0.577	0.109	1.168	0.400	0.742
Koeleria splendens	MARS	0.884	0.834	0.040	1.159	0.132	0.863
Leontodon anomalus	MARS	0.916	0.790	0.063	1.426	0.480	0.933
Linaria purpurea	MARS	0.846	0.820	0.085	1.223	0.130	0.817
Ornithogalum etruscum	MARS	0.777	0.534	0.151	1.111	0.400	0.774
Phyteuma ovatum	MARS	0.912	0.766	0.080	1.274	0.356	0.620
Polygala flavescens	MARS	0.800	0.742	0.034	1.222	0.148	0.893
Ranunculus pollinensis	MARS	0.950	0.874	0.044	1.485	0.189	0.968
Saxifraga porophylla	MARS	0.934	0.865	0.049	1.414	0.115	0.938
Sedum magellense	MARS	0.954	0.917	0.041	1.463	0.225	0.922
Siler montanum	MARS	0.918	0.911	0.052	1.434	0.117	0.666
Viola eugeniae	MARS	0.938	0.825	0.087	1.289	0.300	0.967
Viola ferrarinii	MARS	0.971	0.899	0.063	1.582	0.400	0.738
Adenostyles australis	Maxent	0.932	0.923	0.012	1.593	0.128	0.967
Aubrieta columnae	Maxent	0.953	0.941	0.036	1.810	0.300	0.897
Bellevalia webbiana	Maxent	0.852	0.818	0.072	1.563	0.217	0.795
Cardamine apennina	Maxent	0.817	0.780	0.094	1.463	0.150	0.944
Carduus affinis	Maxent	0.965	0.956	0.016	1.707	0.167	0.916
Crocus biflorus	Maxent	0.723	0.686	0.037	1.165	0.179	0.959
Crocus etruscus	Maxent	0.931	0.909	0.018	1.837	0.450	0.610
Crocus imperati	Maxent	0.879	0.873	0.052	1.647	0.177	0.920
Erysimum pseudorhaeticum	Maxent	0.840	0.820	0.025	1.456	0.271	0.995
Galium magellense	Maxent	0.992	0.991	0.006	1.939	0.175	0.914
Gypsophila arrostoi	Maxent	0.840	0.799	0.078	1.508	0.114	0.789

Helleborus viridis	Maxent	0.807	0.737	0.037	1.347	0.255	0.958
Klasea flavescens	Maxent	0.750	0.625	0.149	1.253	0.283	0.934
Koeleria splendens	Maxent	0.894	0.879	0.025	1.406	0.132	0.977
Leontodon anomalus	Maxent	0.894	0.880	0.024	1.688	0.320	0.918
Linaria purpurea	Maxent	0.851	0.840	0.033	1.315	0.122	0.973
Ornithogalum etruscum	Maxent	0.740	0.626	0.109	1.285	0.367	0.875
Phyteuma ovatum	Maxent	0.942	0.919	0.034	1.678	0.211	0.940
Polygala flavescens	Maxent	0.795	0.760	0.032	1.293	0.152	0.986
Ranunculus pollinensis	Maxent	0.971	0.962	0.015	1.818	0.189	0.983
Saxifraga porophylla	Maxent	0.958	0.953	0.019	1.772	0.138	0.895
Sedum magellense	Maxent	0.968	0.961	0.023	1.801	0.175	0.863
Siler montanum	Maxent	0.935	0.929	0.016	1.603	0.128	0.954
Viola eugeniae	Maxent	0.954	0.937	0.023	1.688	0.209	0.979
Viola ferrarinii	Maxent	0.989	0.988	0.036	1.931	0.260	0.600
Adenostyles australis	RF	1.000	0.982	0.041	1.626	0.394	0.974
Aubrieta columnae	RF	1.000	0.981	0.012	1.747	0.517	0.959
Bellevalia webbiana	RF	1.000	0.973	0.025	1.615	0.683	0.968
Cardamine apennina	RF	1.000	0.929	0.090	1.409	0.613	0.986
Carduus affinis	RF	1.000	0.985	0.061	1.677	0.333	0.916
Crocus biflorus	RF	1.000	0.925	0.092	1.134	0.863	0.955
Crocus etruscus	RF	1.000	1.000	0.029	1.794	0.700	0.895
Crocus imperati	RF	1.000	0.967	0.061	1.624	0.547	0.938
Erysimum pseudorhaeticum	RF	1.000	0.980	0.060	1.432	0.686	0.993
Galium magellense	RF	1.000	0.998	0.001	1.927	0.300	0.951
Gypsophila arrostoi	RF	1.000	0.973	0.062	1.445	0.843	0.970
Helleborus viridis	RF	1.000	0.938	0.027	1.302	0.764	0.991
Klasea flavescens	RF	1.000	0.933	0.101	1.384	0.767	0.965
Koeleria splendens	RF	1.000	0.973	0.027	1.446	0.544	0.994
Leontodon anomalus	RF	1.000	0.981	0.042	1.662	0.540	0.985
Linaria purpurea	RF	1.000	0.940	0.038	1.261	0.609	0.990
Ornithogalum etruscum	RF	1.000	0.960	0.037	1.353	0.900	0.954
Phyteuma ovatum	RF	1.000	0.993	0.046	1.790	0.444	0.933
Polygala flavescens	RF	1.000	0.962	0.054	1.281	0.717	0.988
Ranunculus pollinensis	RF	1.000	0.977	0.012	1.747	0.356	0.976
Saxifraga porophylla	RF	1.000	0.991	0.019	1.736	0.377	0.966
Sedum magellense	RF	1.000	0.990	0.009	1.763	0.392	0.903
Siler montanum	RF	1.000	0.965	0.031	1.458	0.406	0.963
Viola eugeniae	RF	1.000	0.960	0.003	1.568	0.455	0.946
Viola ferrarinii	RF	1.000	1.000	0.011	1.933	0.540	0.989
Adenostyles australis	SVM	0.956	0.942	0.014	1.615	0.206	0.961
Aubrieta columnae	SVM	0.969	0.949	0.003	1.788	0.267	0.922
Bellevalia webbiana	SVM	0.918	0.865	0.080	1.591	0.500	0.870
Cardamine apennina	SVM	0.916	0.862	0.076	1.476	0.263	0.950
Carduus affinis	SVM	0.958	0.958	0.021	1.744	0.128	0.936
Crocus biflorus	SVM	0.787	0.720	0.057	1.141	0.198	0.911
Crocus etruscus	SVM	0.997	0.986	0.000	1.921	0.750	0.951
Crocus imperati	SVM	0.941	0.866	0.047	1.542	0.303	0.892
Erysimum pseudorhaeticum	SVM	0.868	0.826	0.059	1.356	0.176	0.986

Galium magellense	SVM	0.989	0.989	0.002	1.952	0.158	0.979
Gypsophila arrostoi	SVM	0.906	0.796	0.052	1.340	0.343	0.903
Helleborus viridis	SVM	0.883	0.833	0.046	1.357	0.164	0.945
Klasea flavescens	SVM	0.899	0.817	0.104	1.359	0.333	0.939
Koeleria splendens	SVM	0.904	0.885	0.022	1.302	0.128	0.984
Leontodon anomalus	SVM	0.968	0.932	0.031	1.704	0.300	0.962
Linaria purpurea	SVM	0.863	0.842	0.040	1.245	0.130	0.946
Ornithogalum etruscum	SVM	0.891	0.774	0.167	1.290	0.400	0.855
Phyteuma ovatum	SVM	0.967	0.947	0.038	1.735	0.256	0.906
Polygala flavescens	SVM	0.786	0.766	0.025	1.210	0.138	0.970
Ranunculus pollinensis	SVM	0.967	0.948	0.019	1.747	0.211	0.985
Saxifraga porophylla	SVM	0.966	0.950	0.036	1.727	0.169	0.920
Sedum magellense	SVM	0.971	0.969	0.013	1.823	0.125	0.869
Siler montanum	SVM	0.944	0.936	0.033	1.549	0.150	0.977
Viola eugeniae	SVM	0.935	0.943	0.002	1.785	0.136	0.942
Viola ferrarinii	SVM	0.990	0.984	0.032	1.922	0.320	0.995
