# Supplementary material for: Assessing the Effectiveness of Correlative Ecological Niche Model Temporal Projection through Floristic Data
Source: Biology (Basel). 2022 Aug 14;11(8):1219. doi: 10.3390/biology11081219 (PMC9405103; doi:10.3390/biology11081219)
Supplement: Supplementary file 1 [file biology-11-01219-s001.zip › Dolci & Peruzzi Supplementary Material S.1-9.zip!/Supplementary material S.8.pdf]

Supplementary material S.8

Results of MOP analysis conducted on each set of environmental variables. Species were modeld with study area fitted on their distribution. Thus, multiple MOP analysis were required to check the transferability of each model when transferred to historical times to modern times. Table S.8.A reports summary values of environmental similarity for all study areas when only 3 environmental layers were used. Table S.8.B reports summary values of environmental similarity for all study areas when 35 environmental layers and dimensionality reduction via PCA were used. Below the tables all single results are described. Table S.8.C reported a summary of the differences in extrapolation risks for the study area of each species.

Table. S.8.A. Summary values of environmental similarity obtained through MOP analysis. All results are referred to the cases in which only 3 environmental variables were used.

**Species**	**Range of Environmental Similarity**									
	**0.0-0.1**	**0.1-0.2**	**0.2-0.3**	**0.3-0.4**	**0.4-0.5**	**0.5-0.6**	**0.6-0.7**	**0.7-0.8**	**0.8-0.9**	**0.9-1.0**
Adenostyles australis	0.39	0.01	0.03	0.06	0.11	0.27	1.35	5.35	31.72	60.69
Aubrieta columnae	0.75	0.01	0.03	0.05	0.12	0.2	1.29	5.05	49.2	43.3
Bellevalia webbiana	3.13	0.04	0.05	0.06	0.08	0.13	0.36	1.13	7.44	87.59
Cardamine apennina	2.22	0.02	0.04	0.08	0.2	0.64	1.58	4.05	26.18	64.99
Carduus affinis	0.85	0.01	0.02	0.05	0.11	0.18	0.72	5.01	48.71	44.34
Crocus biflorus	0.7	0	0.01	0.03	0.07	0.12	0.23	0.56	2.15	96.14
Crocus etruscus	3.4	0.03	0.08	0.14	0.22	0.48	1.01	2.93	19.93	71.78
Crocus imperati	0.8	0.01	0.02	0.05	0.11	0.19	0.87	4.83	46.01	47.11
Erysimum pseudorhaeticum	1.75	0.01	0.02	0.05	0.09	0.3	0.98	2.73	17.1	76.97
Galium magellense	0	0.01	0.04	0.08	0.17	0.28	0.57	2.74	18.1	78
Gypsophila arrostoi	2.79	0.01	0.04	0.09	0.24	0.61	2.61	15.32	50.49	27.81
Helleborus viridis	0.94	0.01	0.02	0.05	0.09	0.16	0.34	0.94	12.14	85.31
Klasea flavescens	1.43	0.01	0.02	0.04	0.09	0.16	0.57	5.46	43.13	49.1
Koeleria splendens	0.99	0.01	0.03	0.05	0.11	0.23	0.72	5.59	36	56.26
Leontodon anomalus	0.61	0	0	0.01	0.04	0.12	0.33	1.65	14.04	83.19
Linaria purpurea	1.11	0.01	0.03	0.05	0.1	0.2	0.43	2.98	29.53	65.56
Ornithogalum etruscum	1.2	0.02	0.04	0.06	0.13	0.29	0.71	4.92	34.82	57.79
Phyteuma ovatum	2.52	0.03	0.06	0.1	0.18	0.41	0.88	2.66	18.01	75.14
Polygala flavescens	1.27	0.02	0.03	0.06	0.11	0.23	0.52	3.5	30.5	63.76
Ranunculus pollinensis	0.47	0.01	0.04	0.06	0.12	0.31	1.09	4.03	26.59	67.26
Sedum magellense	0.73	0.01	0.03	0.05	0.11	0.21	1.27	4.96	47.74	44.9
Siler montanum	1.15	0.02	0.03	0.06	0.13	0.26	0.59	3.91	32.01	61.84
Saxifraga porophylla	0.73	0.01	0.03	0.05	0.11	0.21	1.27	4.96	47.74	44.9
Viola eugeniae	0.49	0.01	0.02	0.04	0.08	0.2	1.18	4.62	36.28	57.08
Viola ferrarinii	0.8	0.03	0.07	0.12	0.18	0.46	1.19	3.42	21.16	72.57

Table. S.8.B. Summary values of environmental similarity obtained through MOP analysis. All results are referred to the cases in which 35 environmental variables and dimensionality reduction via PCA were used.

Species	Range of Environmental Similarity									
	0.95	0	0.02	0.1	0.2	0.64	2.99	15.21	70.29	9.6
Adenostyles australis	0.17	0.02	0.04	0.21	0.61	2.05	10.09	45.84	40.97	0
Aubrieta columnae	0.31	0.01	0.06	0.29	0.34	0.61	2.41	12.78	68.81	14.38
Bellevalia webbiana	7.06	0.02	0.02	0.12	0.38	1.17	7.78	46.62	36.67	0.15
Cardamine apennina	0	0.02	0.03	0.2	0.58	2.08	9.09	41.74	46.25	0
Carduus affinis	0.03	0	0.01	0.04	0.09	0.25	1.26	9.29	51.53	37.49
Crocus biflorus	0.22	0	0.05	0.3	0.55	1.4	4.06	17.69	59.85	15.88
Crocus etruscus	0.1	0.02	0.03	0.18	0.53	1.71	8.41	42.5	46.52	0
Crocus imperati	6.97	0.02	0.03	0.1	0.34	1.01	4.24	32.65	53.6	1.06
Erysimum pseudorhaeticum	0.01	0.02	0.05	0.22	0.63	2.32	6.09	21.12	69.55	0
Galium magellense	0.08	0.01	0.01	0.01	0.03	0.13	1.13	9	83.84	5.76
Gypsophila arrostoi	0.21	0.01	0.04	0.08	0.14	0.29	1.82	12.4	61.38	23.62
Helleborus viridis	0.01	0	0.02	0.04	0.16	0.71	5.1	24.81	69.02	0.12
Klasea flavescens	1.01	0	0.03	0.08	0.19	0.61	4.63	22.87	65.96	4.61
Koeleria splendens	0.04	0	0	0.03	0.29	1.12	5.08	12.52	35.25	45.66
Leontodon anomalus	1.06	0	0.01	0.06	0.31	1.55	7.7	31.75	57.54	0
Linaria purpurea	0.87	0.01	0.03	0.15	0.83	2.56	6.55	20.73	62.61	5.66
Ornithogalum etruscum	8.21	0	0.05	0.19	0.44	1.01	2.8	28.71	56.07	2.53
Phyteuma ovatum	4.72	0	0.02	0.2	0.65	1.6	8.44	33.1	51.15	0.11
Polygala flavescens	0.09	0.01	0.02	0.17	0.7	2.84	11.65	30.18	53.89	0.45
Ranunculus pollinensis	0.18	0.02	0.04	0.2	0.62	2.03	8.5	45.99	42.42	0
Sedum magellense	0	0	0.01	0.02	0.21	1.32	5.57	29.04	60.8	3.03
Siler montanum	0.18	0.02	0.04	0.2	0.62	2.03	8.5	45.99	42.42	0
Saxifraga porophylla	8.14	0.01	0.02	0.08	0.23	0.73	8.3	41.74	40.71	0.03
Viola eugeniae	0.05	0.16	0.2	0.18	0.5	1.61	6.95	18.69	55.99	15.68
Viola ferrarinii	0.95	0	0.02	0.1	0.2	0.64	2.99	15.21	70.29	9.6

Table. S.8.C. Summary of differences in extrapolation risks when different sets of environmental variables are applied. The values reported are referred to the percentage of cells associated to a high risk of strict extrapolation (environmental similarity equal to 0-0.1). Differences were observed between the use of a low number of a priori selected environmental variables and the use of dimensionality reduction techniques (PCA) starting from a high number of environmental variables. Petitpierre et al. [53] favoured the use of a PCA calibrated on multiple predictor variables to increase the transferability of models. The MOP analysis performed on the two selected sets of environmental variables confirmed the usefulness of this approach. For most of the study areas, a reduction of the strict extrapolation risk was observed when the PCA was performed. However, in 5 cases a worse transferability was obtained (*Saxifraga porophylla* subsp. *porophylla, Ornithogalum etruscum* subsp. *etruscum, Crocus imperati, Bellevalia webbiana,* and *Phyteuma ovatum* subsp. *pseudospicatum*). Indeed, no prior assumption related to the transferability of models should be done without verification of the results, as demonstrated by the increase of strict extrapolation risk in 5 cases.

Species	Set I: 3 variables	Set II: first 3 axes of PCA	Diff.
- *Adenostyles australis* (Ten.) Iamonico & Pignatti	0.39	0.17	0.22
- *Aubrieta columnae* Guss. subsp. *columnae*	0.75	0.31	0.44
- *Bellevalia webbiana* Parl.	3.13	7.06	-3.93
- *Cardamine apennina* Lihová & Marhold	2.22	0	2.22
- *Carduus affinis* Guss. subsp. *affinis*	0.85	0.03	0.82
- *Crocus biflorus* Mill.	0.7	0.22	0.48
- *Crocus etruscus* Parl.	3.4	0.1	3.3
- *Crocus imperati* Ten.	0.8	6.97	-6.17
- *Erysimum pseudorhaeticum* Polatschek	1.75	0.01	1.74
- *Galium magellense Ten.*	0	0.08	-0.08
- *Gypsophila arrostoi* Guss. subsp. *arrostoi*	2.79	0.21	2.58
- *Helleborus viridis* L. subsp. *bocconei* (Ten.) Peruzzi	0.94	0.01	0.93
- *Klasea flavescens* (L.) Holub subsp. *cichoracea* (L.) Greuter & Wagenitz	1.43	1.01	0.42
- *Koeleria splendens* C.Presl	0.99	0.04	0.95
- *Leontodon anomalus* Ball	0.61	1.06	-0.45
- *Linaria purpurea* (L.) Mill.	1.11	0.87	0.24
- *Ornithogalum etruscum* Parl. subsp. *etruscum*	1.2	8.21	-7.01
- *Phyteuma ovatum* Honck. subsp. *pseudospicatum*	2.52	4.72	-2.2
- *Polygala flavescens* DC. subsp. *flavescens*	1.27	0.09	1.18
- *Ranunculus pollinensis* (N.Terracc.) Chiov.	0.47	0.18	0.29
- *Sedum magellense* Ten. subsp. *magellense*	0.73	0	0.73
- *Siler montanum* Crantz subsp. *siculum*	1.15	0.18	0.97
- *Saxifraga porophylla* Bertol. subsp. *porophylla*	0.73	8.14	-7.41
- *Viola eugeniae* Parl. subsp. *eugeniae*	0.49	0.05	0.44
- *Viola ferrarinii* Moraldo & Ricceri	0.8	0.95	-0.15

Set I: 3 environmental variables

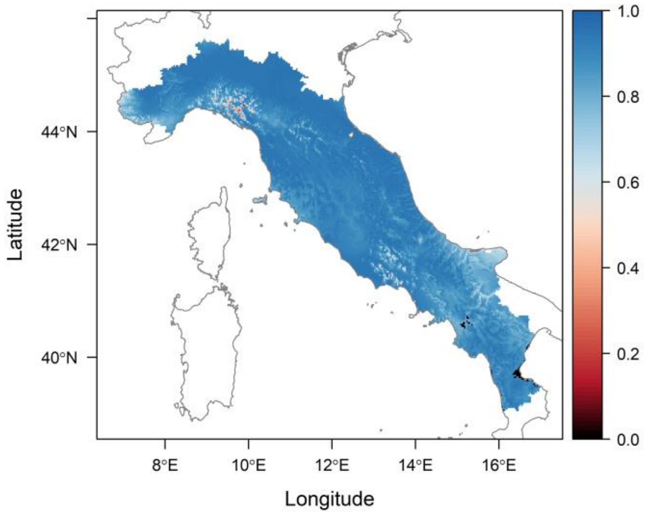

Adenostyles australis (Ten.) Iamonico & Pignatti		
**Range of Values**	**Number of Cells**	**Perc (%)**
0.0-0.1	969	0.39
0.1-0.2	34	0.01
0.2-0.3	86	0.03
0.3-0.4	146	0.06
0.4-0.5	277	0.11
0.5-0.6	679	0.27
0.6-0.7	3331	1.35
0.7-0.8	13230	5.35
0.8-0.9	78413	31.72
0.9-1.0	150001	60.69

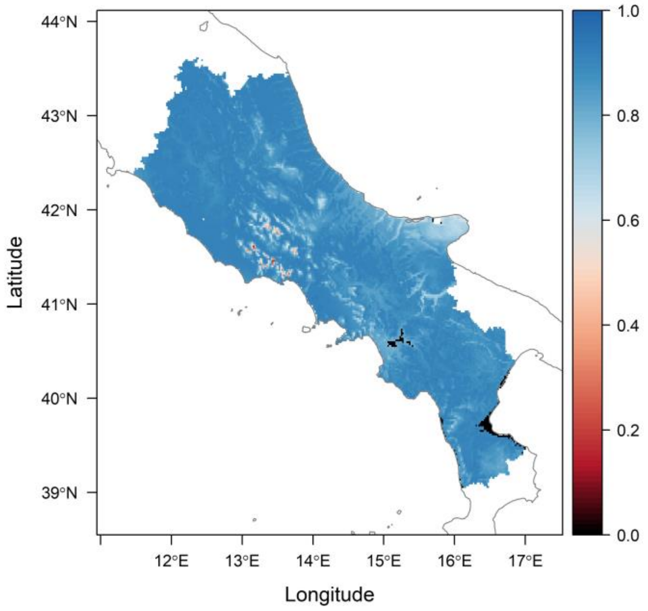

Aubrieta columnae Guss. subsp. *columnae*		
**Range of Values**	**Number of Cells**	**Perc (%)**
0.0-0.1	963	0.75
0.1-0.2	10	0.01
0.2-0.3	36	0.03
0.3-0.4	61	0.05
0.4-0.5	149	0.12
0.5-0.6	264	0.2
0.6-0.7	1668	1.29
0.7-0.8	6507	5.05
0.8-0.9	63378	49.2
0.9-1.0	55769	43.3

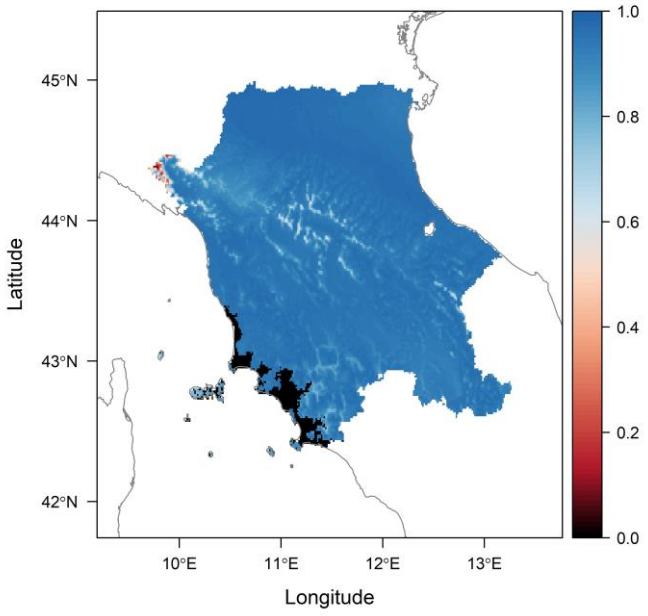

Bellevalia webbiana Parl.		
**Range of Values**	**Number of Cells**	**Perc (%)**
0.0-0.1	2430	3.13
0.1-0.2	29	0.04
0.2-0.3	38	0.05
0.3-0.4	43	0.06
0.4-0.5	62	0.08
0.5-0.6	98	0.13
0.6-0.7	281	0.36
0.7-0.8	880	1.13
0.8-0.9	5776	7.44
0.9-1.0	68025	87.59

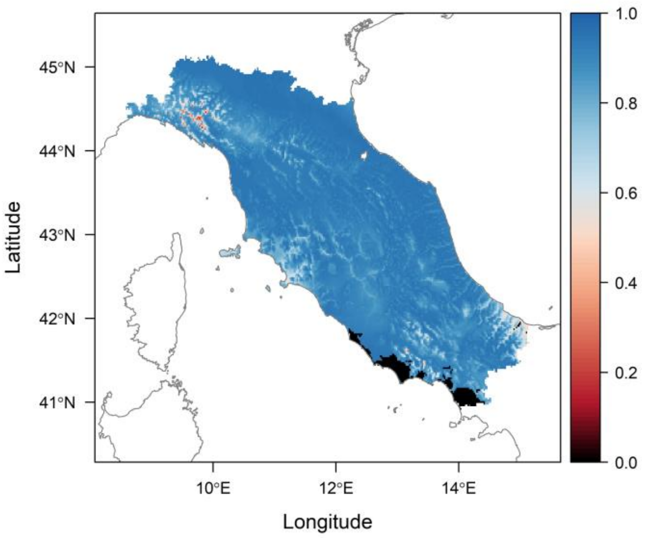

Cardamine apennina Lihová & Marhold		
**Range of Values**	**Number of Cells**	**Perc (%)**
0.0-0.1	3474	2.22
0.1-0.2	33	0.02
0.2-0.3	70	0.04
0.3-0.4	126	0.08
0.4-0.5	310	0.2
0.5-0.6	1006	0.64
0.6-0.7	2474	1.58
0.7-0.8	6347	4.05
0.8-0.9	41055	26.18
0.9-1.0	101898	64.99

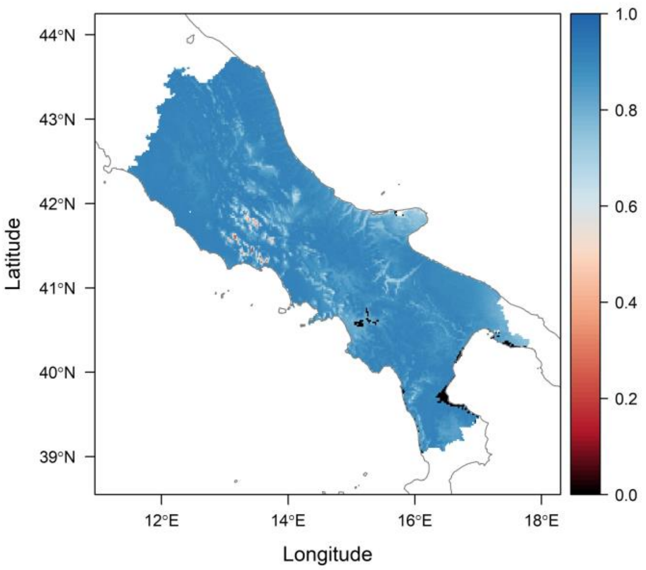

Carduus affinis Guss. subsp. *affinis*		
**Range of Values**	**Number of Cells**	**Perc (%)**
0.0-0.1	1217	0.85
0.1-0.2	12	0.01
0.2-0.3	35	0.02
0.3-0.4	71	0.05
0.4-0.5	156	0.11
0.5-0.6	266	0.18
0.6-0.7	1042	0.72
0.7-0.8	7204	5.01
0.8-0.9	70092	48.71
0.9-1.0	63815	44.34

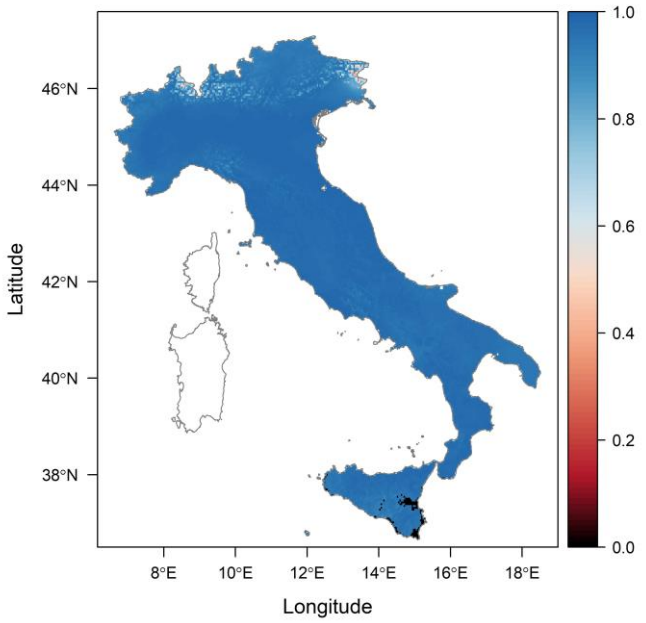

Crocus biflorus Mill.		
**Range of Values**	**Number of Cells**	**Perc (%)**
0.0-0.1	3077	0.7
0.1-0.2	4	0
0.2-0.3	30	0.01
0.3-0.4	115	0.03
0.4-0.5	325	0.07
0.5-0.6	541	0.12
0.6-0.7	1012	0.23
0.7-0.8	2450	0.56
0.8-0.9	9441	2.15
0.9-1.0	423082	96.14

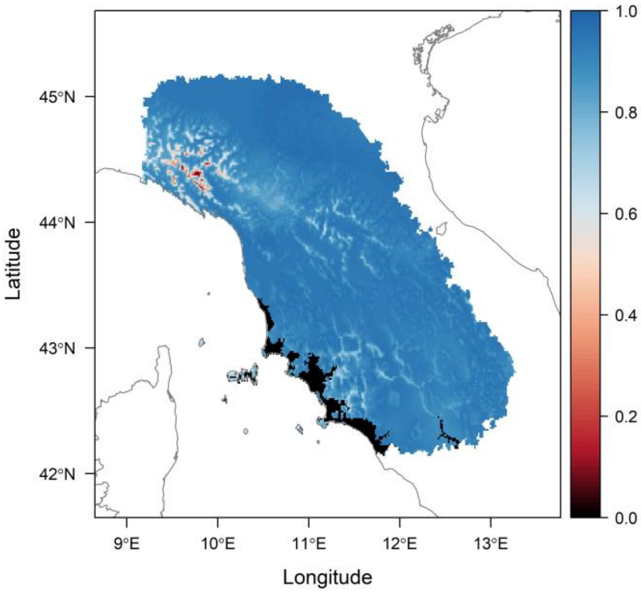

Crocus etruscus Parl.		
**Range of Values**	**Number of Cells**	**Perc (%)**
0.0-0.1	3242	3.4
0.1-0.2	33	0.03
0.2-0.3	72	0.08
0.3-0.4	129	0.14
0.4-0.5	209	0.22
0.5-0.6	459	0.48
0.6-0.7	962	1.01
0.7-0.8	2793	2.93
0.8-0.9	18981	19.93
0.9-1.0	68373	71.78

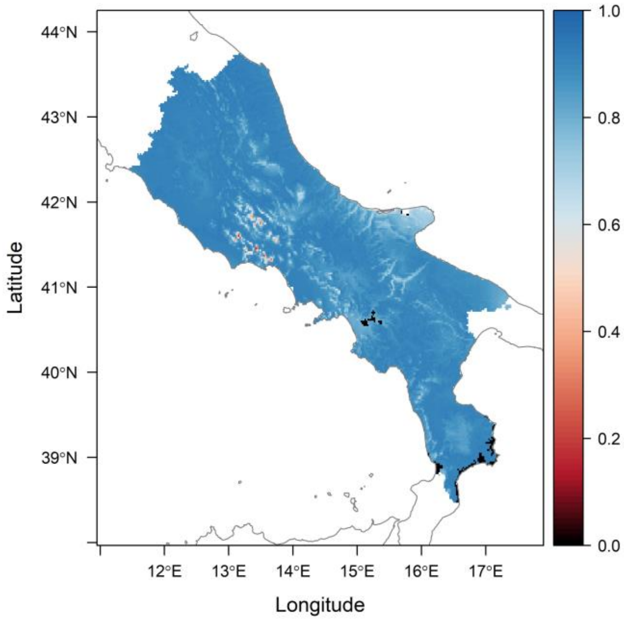

Crocus imperati Ten.		
**Range of Values**	**Number of Cells**	**Perc (%)**
0.0-0.1	1168	0.8
0.1-0.2	12	0.01
0.2-0.3	35	0.02
0.3-0.4	71	0.05
0.4-0.5	156	0.11
0.5-0.6	277	0.19
0.6-0.7	1271	0.87
0.7-0.8	7073	4.83
0.8-0.9	67323	46.01
0.9-1.0	68936	47.11

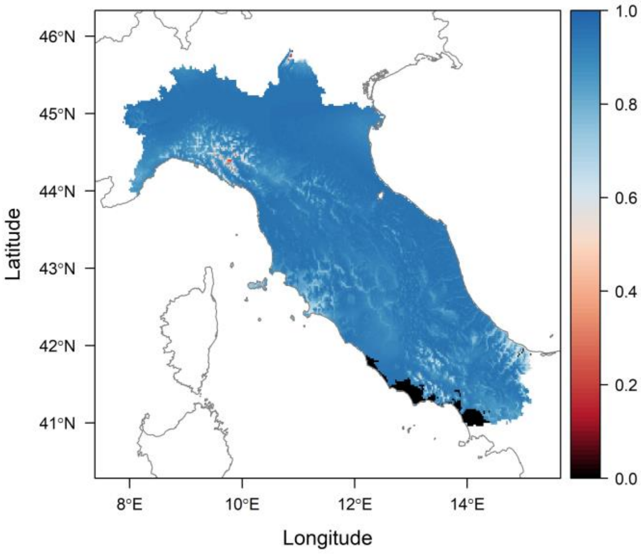

Erysimum pseudorhaeticum Polatschek		
**Range of Values**	**Number of Cells**	**Perc (%)**
0.0-0.1	3473	1.75
0.1-0.2	14	0.01
0.2-0.3	46	0.02
0.3-0.4	103	0.05
0.4-0.5	181	0.09
0.5-0.6	593	0.3
0.6-0.7	1950	0.98
0.7-0.8	5418	2.73
0.8-0.9	33976	17.1
0.9-1.0	152902	76.97

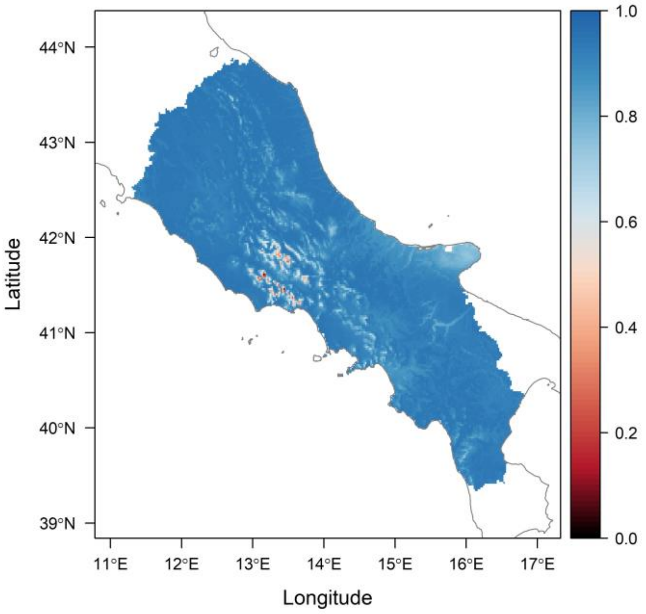

Galium magellense Ten.		
**Range of Values**	**Number of Cells**	**Perc (%)**
0.0-0.1	5	0
0.1-0.2	17	0.01
0.2-0.3	48	0.04
0.3-0.4	112	0.08
0.4-0.5	236	0.17
0.5-0.6	382	0.28
0.6-0.7	774	0.57
0.7-0.8	3736	2.74
0.8-0.9	24657	18.1
0.9-1.0	106260	78

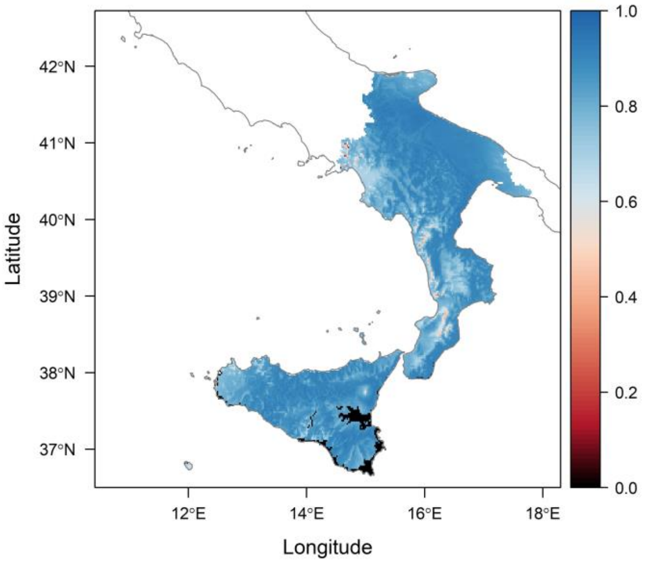

Gypsophila arrostoi Guss. subsp. *arrostoi*		
**Range of Values**	**Number of Cells**	**Perc (%)**
0.0-0.1	3077	2.79
0.1-0.2	10	0.01
0.2-0.3	39	0.04
0.3-0.4	96	0.09
0.4-0.5	264	0.24
0.5-0.6	671	0.61
0.6-0.7	2877	2.61
0.7-0.8	16870	15.32
0.8-0.9	55611	50.49
0.9-1.0	30633	27.81

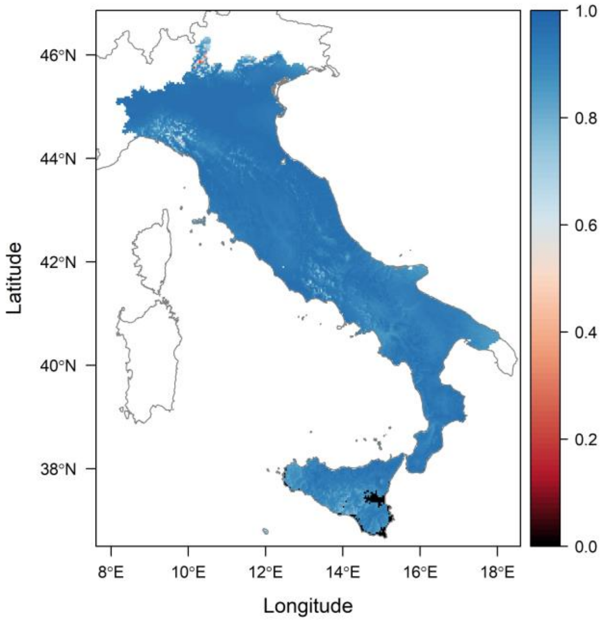

Helleborus viridis L. subsp. *bocconei* (Ten.) Peruzzi		
**Range of Values**	**Number of Cells**	**Perc (%)**
0.0-0.1	3080	0.94
0.1-0.2	39	0.01
0.2-0.3	80	0.02
0.3-0.4	168	0.05
0.4-0.5	311	0.09
0.5-0.6	543	0.16
0.6-0.7	1120	0.34
0.7-0.8	3085	0.94
0.8-0.9	39968	12.14
0.9-1.0	280956	85.31

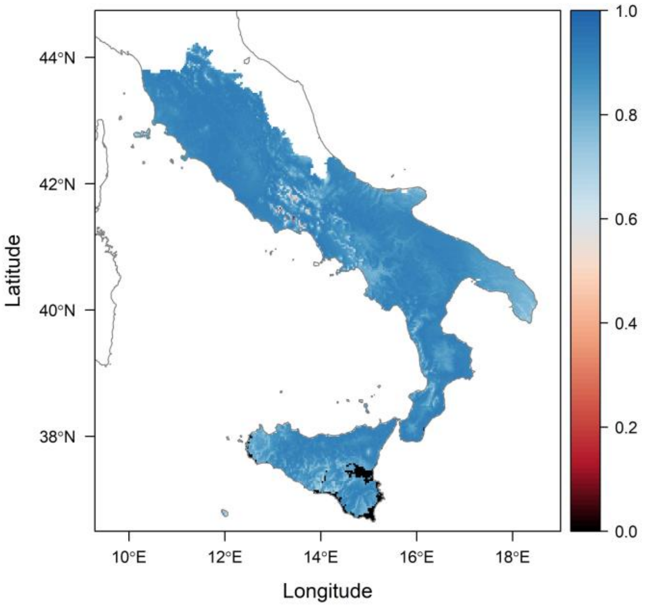

Klasea flavescens (L.) Holub subsp. *cichoracea* (L.) Greuter & Wagenitz		
**Range of Values**	**Number of Cells**	**Perc (%)**
0.0-0.1	3081	1.43
0.1-0.2	16	0.01
0.2-0.3	42	0.02
0.3-0.4	94	0.04
0.4-0.5	187	0.09
0.5-0.6	346	0.16
0.6-0.7	1225	0.57
0.7-0.8	11749	5.46
0.8-0.9	92868	43.13
0.9-1.0	105711	49.1

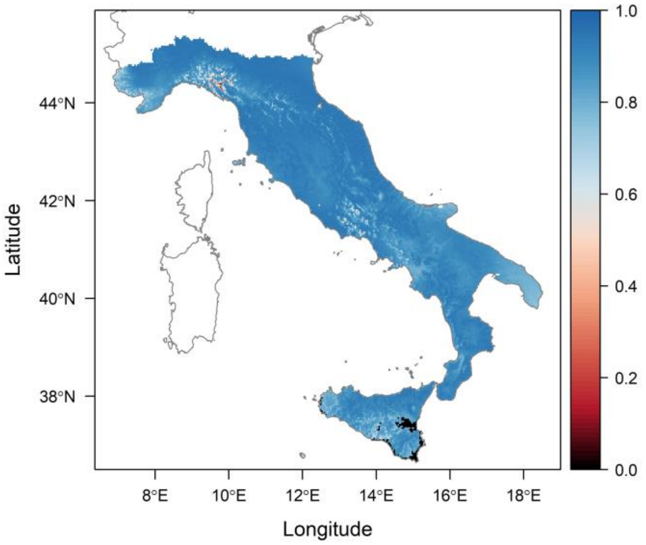

Koeleria splendens C.Presl		
**Range of Values**	**Number of Cells**	**Perc (%)**
0.0-0.1	3087	0.99
0.1-0.2	45	0.01
0.2-0.3	94	0.03
0.3-0.4	164	0.05
0.4-0.5	351	0.11
0.5-0.6	733	0.23
0.6-0.7	2251	0.72
0.7-0.8	17440	5.59
0.8-0.9	112289	36
0.9-1.0	175502	56.26

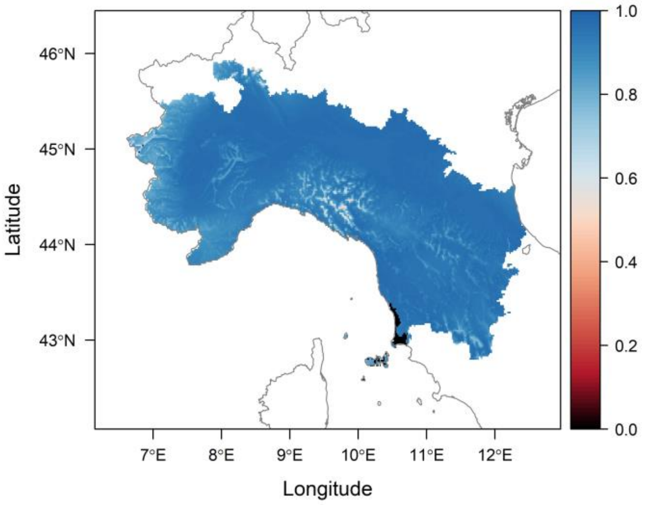

Leontodon anomalus Ball		
**Range of Values**	**Number of Cells**	**Perc (%)**
0.0-0.1	741	0.61
0.1-0.2	3	0
0.2-0.3	3	0
0.3-0.4	9	0.01
0.4-0.5	48	0.04
0.5-0.6	151	0.12
0.6-0.7	400	0.33
0.7-0.8	2004	1.65
0.8-0.9	17029	14.04
0.9-1.0	100903	83.19

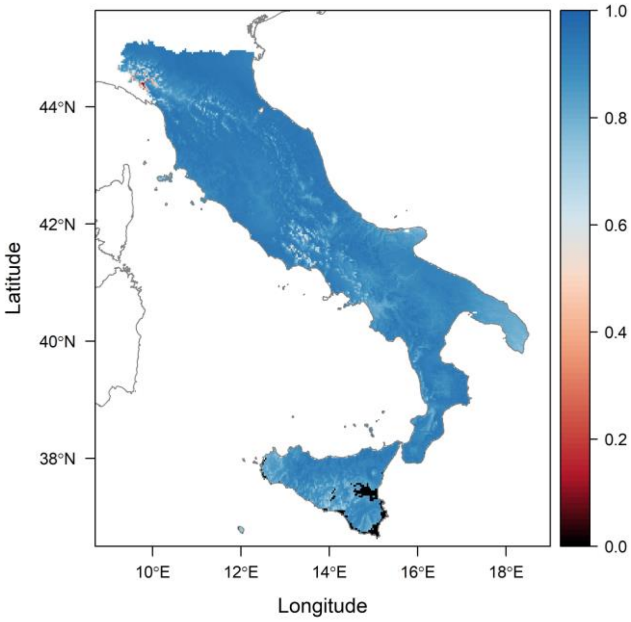

Linaria purpurea (L.) Mill.		
**Range of Values**	**Number of Cells**	**Perc (%)**
0.0-0.1	3088	1.11
0.1-0.2	37	0.01
0.2-0.3	78	0.03
0.3-0.4	137	0.05
0.4-0.5	286	0.1
0.5-0.6	548	0.2
0.6-0.7	1200	0.43
0.7-0.8	8317	2.98
0.8-0.9	82319	29.53
0.9-1.0	182733	65.56

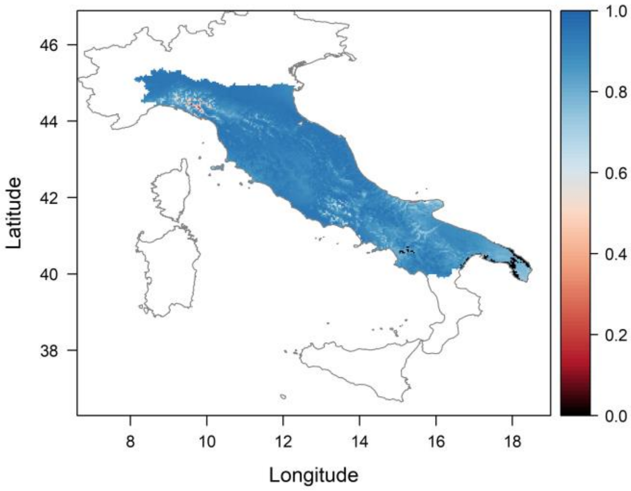

Ornithogalum etruscum Parl. subsp. *etruscum*		
**Range of Values**	**Number of Cells**	**Perc (%)**
0.0-0.1	2024	0.87
0.1-0.2	30	0.01
0.2-0.3	61	0.03
0.3-0.4	359	0.15
0.4-0.5	1937	0.83
0.5-0.6	5986	2.56
0.6-0.7	15300	6.55
0.7-0.8	48428	20.73
0.8-0.9	146225	62.61
0.9-1.0	13212	5.66

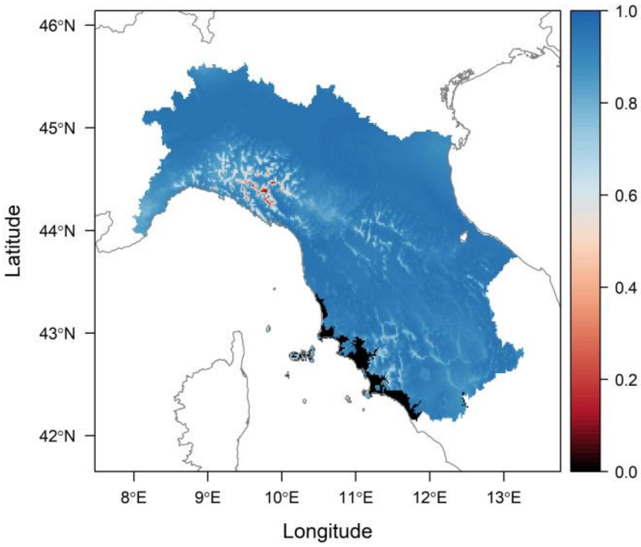

Phyteuma ovatum Honck. subsp. *pseudospicatum* Pignatti		
**Range of Values**	**Number of Cells**	**Perc (%)**
0.0-0.1	3144	2.52
0.1-0.2	34	0.03
0.2-0.3	79	0.06
0.3-0.4	131	0.1
0.4-0.5	230	0.18
0.5-0.6	511	0.41
0.6-0.7	1106	0.88
0.7-0.8	3330	2.66
0.8-0.9	22507	18.01
0.9-1.0	93920	75.14

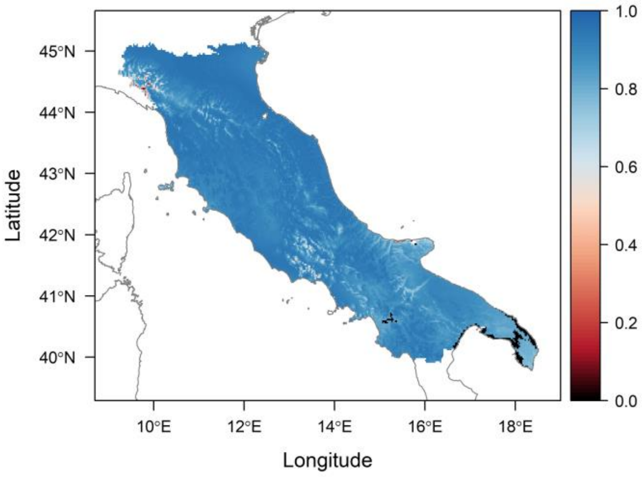

Polygala flavescens DC. subsp. *flavescens*		
**Range of Values**	**Number of Cells**	**Perc (%)**
0.0-0.1	2813	1.27
0.1-0.2	35	0.02
0.2-0.3	68	0.03
0.3-0.4	126	0.06
0.4-0.5	247	0.11
0.5-0.6	503	0.23
0.6-0.7	1154	0.52
0.7-0.8	7746	3.5
0.8-0.9	67461	30.5
0.9-1.0	141048	63.76

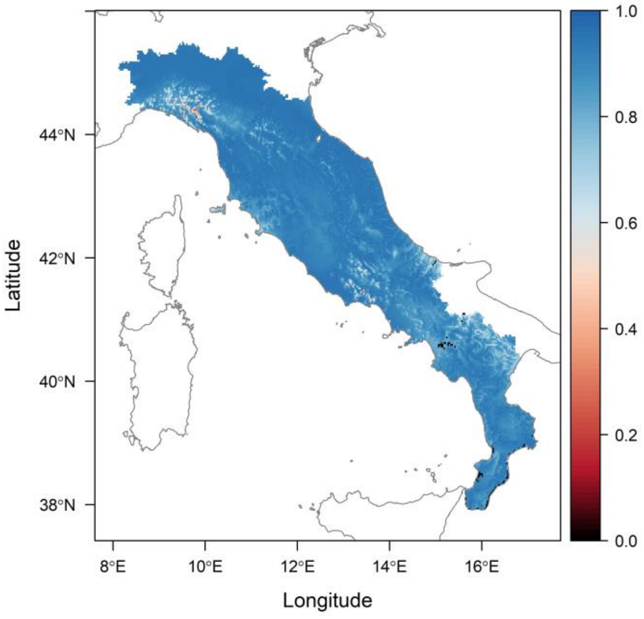

Ranunculus pollinensis (N.Terracc.) Chiov.		
**Range of Values**	**Number of Cells**	**Perc (%)**
0.0-0.1	1085	0.47
0.1-0.2	34	0.01
0.2-0.3	86	0.04
0.3-0.4	145	0.06
0.4-0.5	280	0.12
0.5-0.6	720	0.31
0.6-0.7	2519	1.09
0.7-0.8	9275	4.03
0.8-0.9	61245	26.59
0.9-1.0	154909	67.26

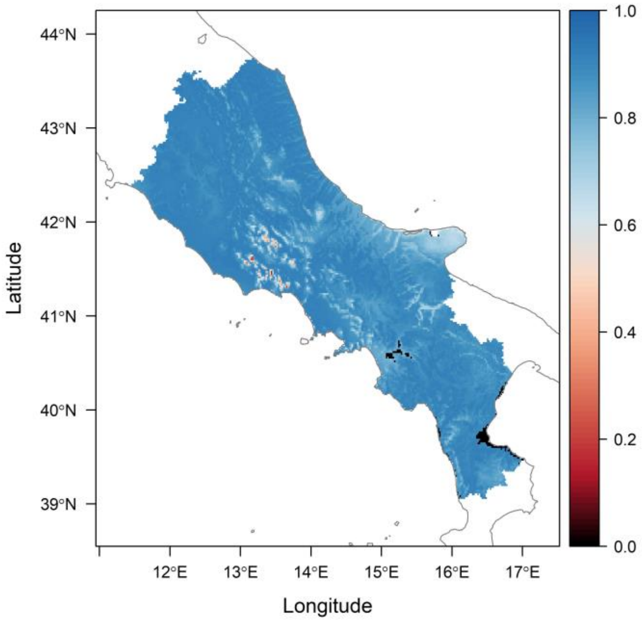

Saxifraga porophylla Bertol. subsp. *porophylla*		
**Range of Values**	**Number of Cells**	**Perc (%)**
0.0-0.1	963	0.73
0.1-0.2	10	0.01
0.2-0.3	36	0.03
0.3-0.4	63	0.05
0.4-0.5	148	0.11
0.5-0.6	273	0.21
0.6-0.7	1674	1.27
0.7-0.8	6541	4.96
0.8-0.9	62994	47.74
0.9-1.0	59248	44.9

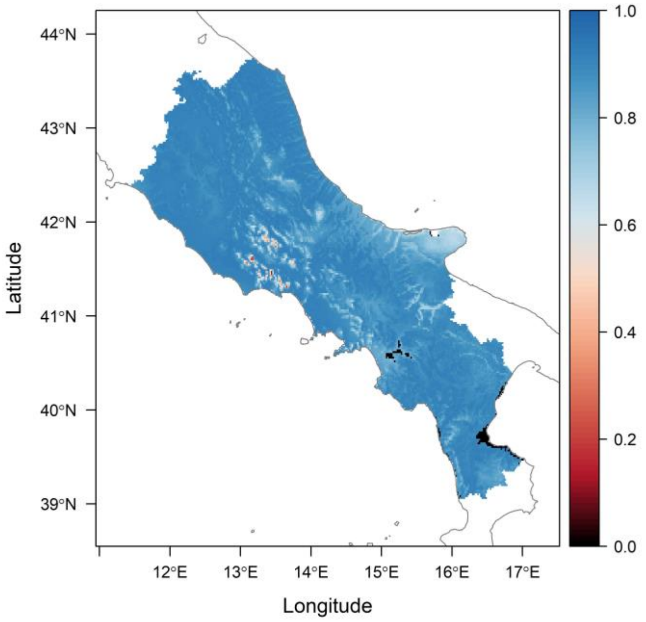

Sedum magellense Ten. subsp. *magellense*		
**Range of Values**	**Number of Cells**	**Perc (%)**
0.0-0.1	963	0.73
0.1-0.2	10	0.01
0.2-0.3	36	0.03
0.3-0.4	63	0.05
0.4-0.5	148	0.11
0.5-0.6	273	0.21
0.6-0.7	1674	1.27
0.7-0.8	6541	4.96
0.8-0.9	62994	47.74
0.9-1.0	59248	44.9

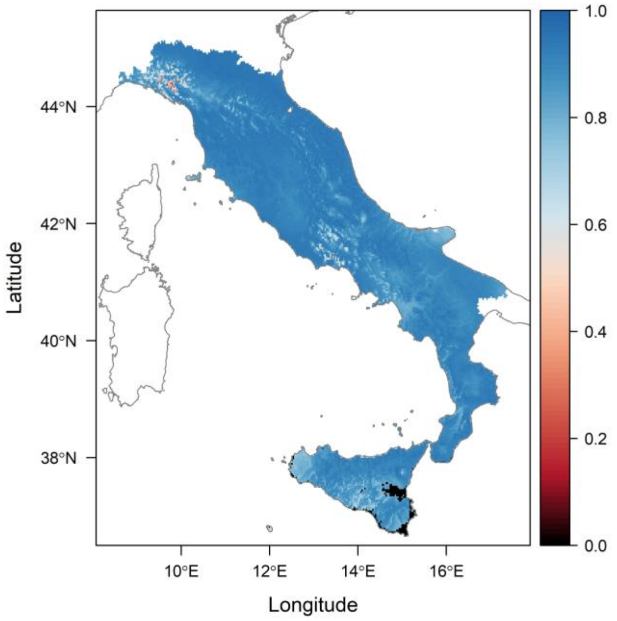

Siler montanum Crantz subsp. *siculum* (Spreng.) Iamonico, Bartolucci & F.Conti		
**Range of Values**	**Number of Cells**	**Perc (%)**
0.0-0.1	3087	1.15
0.1-0.2	46	0.02
0.2-0.3	93	0.03
0.3-0.4	165	0.06
0.4-0.5	353	0.13
0.5-0.6	693	0.26
0.6-0.7	1588	0.59
0.7-0.8	10472	3.91
0.8-0.9	85845	32.01
0.9-1.0	165821	61.84

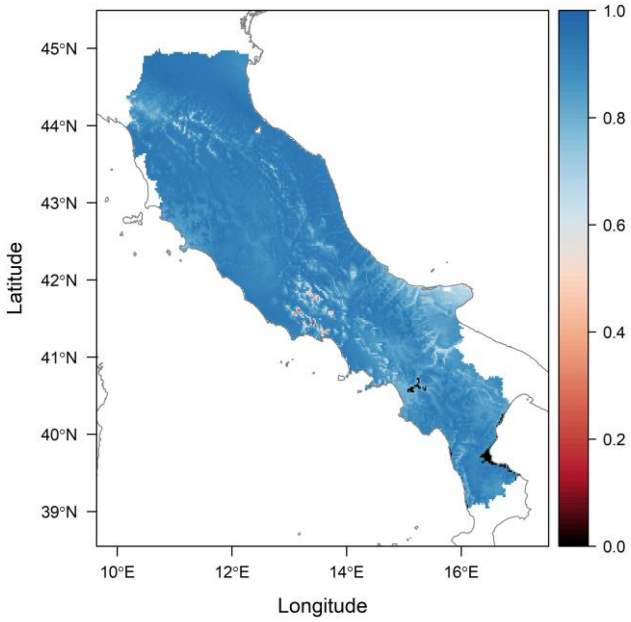

Viola eugeniae Parl. subsp. *eugeniae*		
**Range of Values**	**Number of Cells**	**Perc (%)**
0.0-0.1	963	0.49
0.1-0.2	14	0.01
0.2-0.3	36	0.02
0.3-0.4	86	0.04
0.4-0.5	158	0.08
0.5-0.6	387	0.2
0.6-0.7	2309	1.18
0.7-0.8	9043	4.62
0.8-0.9	71017	36.28
0.9-1.0	111749	57.08

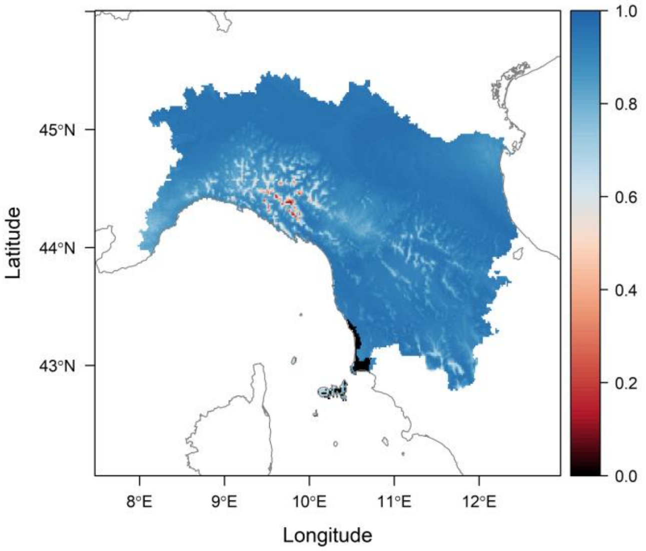

Viola ferrarinii Moraldo & Ricceri		
**Range of Values**	**Number of Cells**	**Perc (%)**
0.0-0.1	725	0.8
0.1-0.2	29	0.03
0.2-0.3	64	0.07
0.3-0.4	111	0.12
0.4-0.5	160	0.18
0.5-0.6	413	0.46
0.6-0.7	1076	1.19
0.7-0.8	3101	3.42
0.8-0.9	19171	21.16
0.9-1.0	65758	72.57

Set II: 35 environmental variables plus dimensionality reduction via PCA

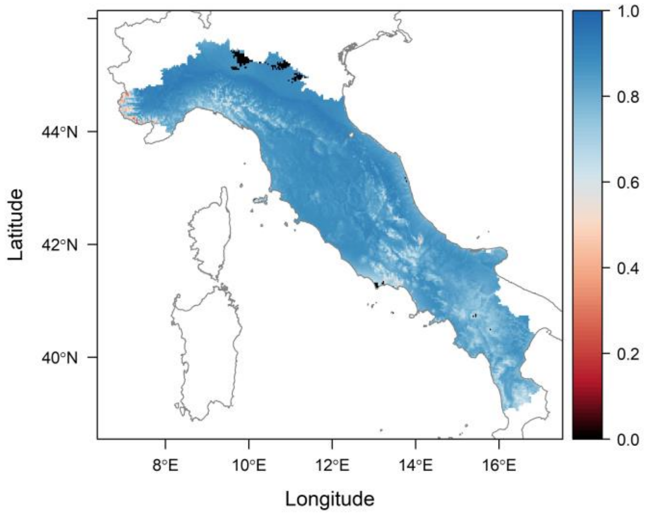

Adenostyles australis (Ten.) Iamonico & Pignatti		
**Range of Values**	**Number of Cells**	**Perc (%)**
0.0-0.1	2342	0.95
0.1-0.2	6	0
0.2-0.3	56	0.02
0.3-0.4	237	0.1
0.4-0.5	505	0.2
0.5-0.6	1587	0.64
0.6-0.7	7382	2.99
0.7-0.8	37586	15.21
0.8-0.9	173732	70.29
0.9-1.0	23733	9.6

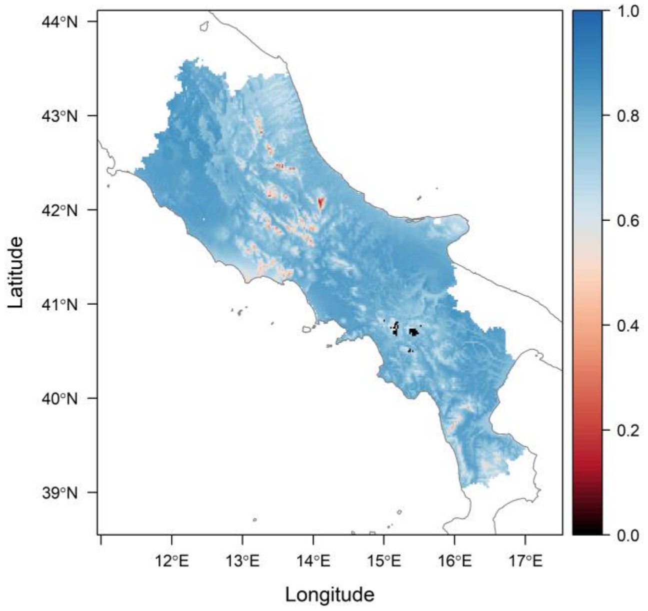

Aubrieta columnae Guss. subsp. *columnae*		
**Range of Values**	**Number of Cells**	**Perc (%)**
0.0-0.1	221	0.17
0.1-0.2	26	0.02
0.2-0.3	52	0.04
0.3-0.4	265	0.21
0.4-0.5	789	0.61
0.5-0.6	2639	2.05
0.6-0.7	12992	10.09
0.7-0.8	59049	45.84
0.8-0.9	52772	40.97
0.9-1.0	0	0

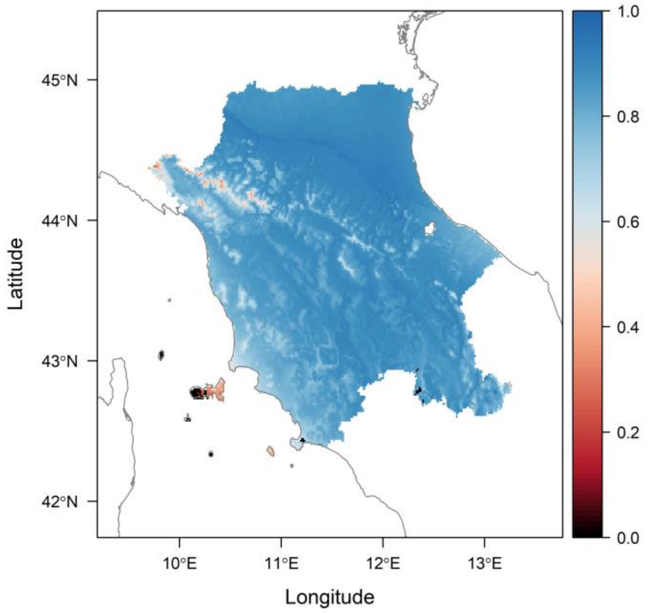

Bellevalia webbiana Parl.		
**Range of Values**	**Number of Cells**	**Perc (%)**
0.0-0.1	242	0.31
0.1-0.2	9	0.01
0.2-0.3	48	0.06
0.3-0.4	224	0.29
0.4-0.5	264	0.34
0.5-0.6	477	0.61
0.6-0.7	1869	2.41
0.7-0.8	9924	12.78
0.8-0.9	53438	68.81
0.9-1.0	11167	14.38

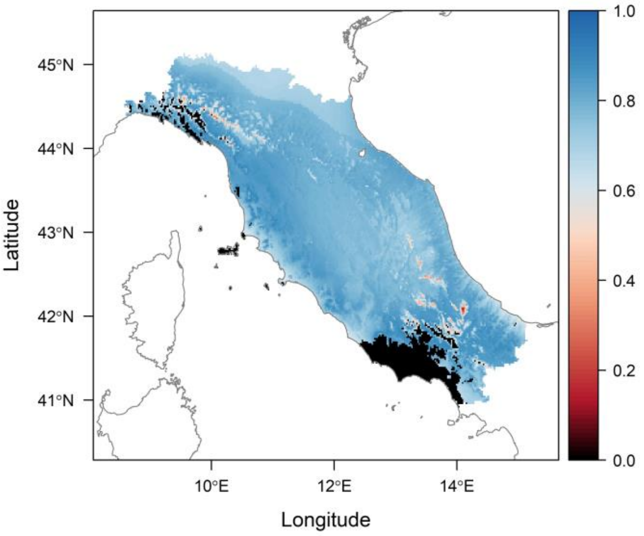

Cardamine apennina Lihová & Marhold		
**Range of Values**	**Number of Cells**	**Perc (%)**
0.0-0.1	11071	7.06
0.1-0.2	26	0.02
0.2-0.3	37	0.02
0.3-0.4	187	0.12
0.4-0.5	596	0.38
0.5-0.6	1831	1.17
0.6-0.7	12205	7.78
0.7-0.8	73104	46.62
0.8-0.9	57495	36.67
0.9-1.0	241	0.15

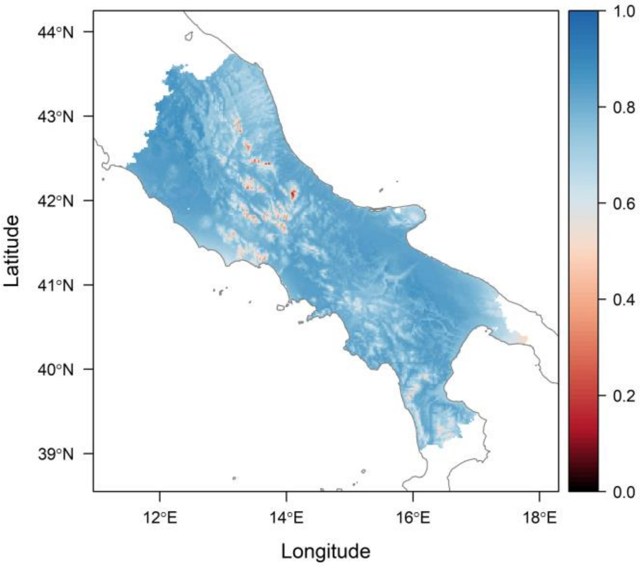

Carduus affinis Guss. subsp. *affinis*		
**Range of Values**	**Number of Cells**	**Perc (%)**
0.0-0.1	1217	0.85
0.1-0.2	12	0.01
0.2-0.3	35	0.02
0.3-0.4	71	0.05
0.4-0.5	156	0.11
0.5-0.6	266	0.18
0.6-0.7	1042	0.72
0.7-0.8	7204	5.01
0.8-0.9	70092	48.71
0.9-1.0	63815	44.34

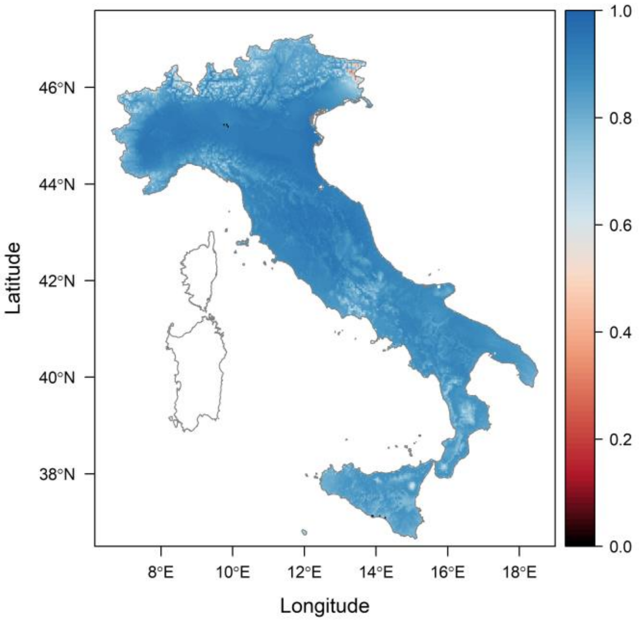

Crocus biflorus Mill.		
**Range of Values**	**Number of Cells**	**Perc (%)**
0.0-0.1	128	0.03
0.1-0.2	6	0
0.2-0.3	46	0.01
0.3-0.4	162	0.04
0.4-0.5	410	0.09
0.5-0.6	1118	0.25
0.6-0.7	5546	1.26
0.7-0.8	40888	9.29
0.8-0.9	226777	51.53
0.9-1.0	164996	37.49

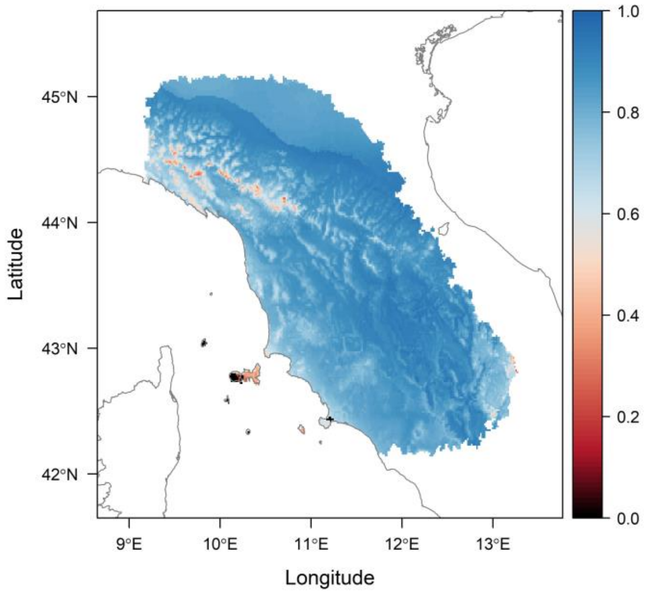

Crocus etruscus Parl.		
**Range of Values**	**Number of Cells**	**Perc (%)**
0.0-0.1	206	0.22
0.1-0.2	0	0
0.2-0.3	46	0.05
0.3-0.4	282	0.3
0.4-0.5	523	0.55
0.5-0.6	1335	1.4
0.6-0.7	3872	4.06
0.7-0.8	16852	17.69
0.8-0.9	57012	59.85
0.9-1.0	15125	15.88

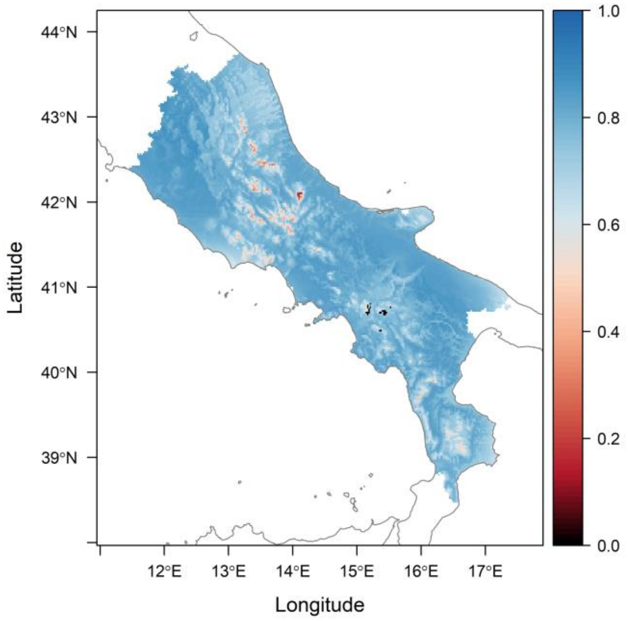

Crocus imperati Ten.		
**Range of Values**	**Number of Cells**	**Perc (%)**
0.0-0.1	144	0.1
0.1-0.2	28	0.02
0.2-0.3	46	0.03
0.3-0.4	259	0.18
0.4-0.5	771	0.53
0.5-0.6	2506	1.71
0.6-0.7	12310	8.41
0.7-0.8	62189	42.5
0.8-0.9	68069	46.52
0.9-1.0	0	0

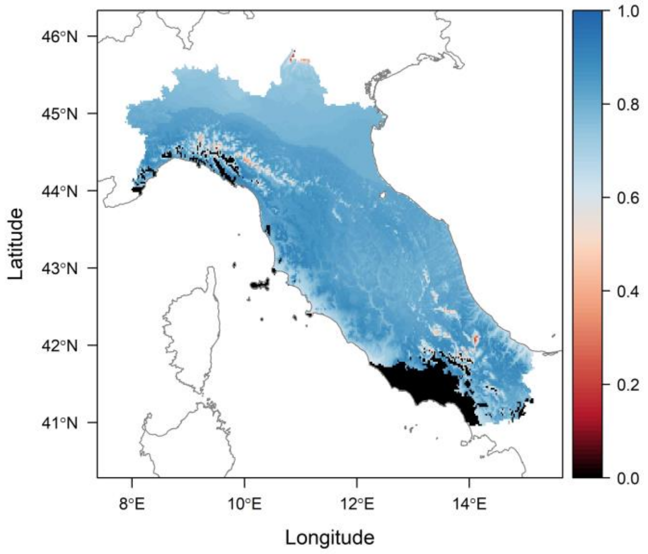

Erysimum pseudorhaeticum Polatschek		
**Range of Values**	**Number of Cells**	**Perc (%)**
0.0-0.1	13842	6.97
0.1-0.2	32	0.02
0.2-0.3	53	0.03
0.3-0.4	199	0.1
0.4-0.5	666	0.34
0.5-0.6	2003	1.01
0.6-0.7	8420	4.24
0.7-0.8	64855	32.65
0.8-0.9	106487	53.6
0.9-1.0	2099	1.06

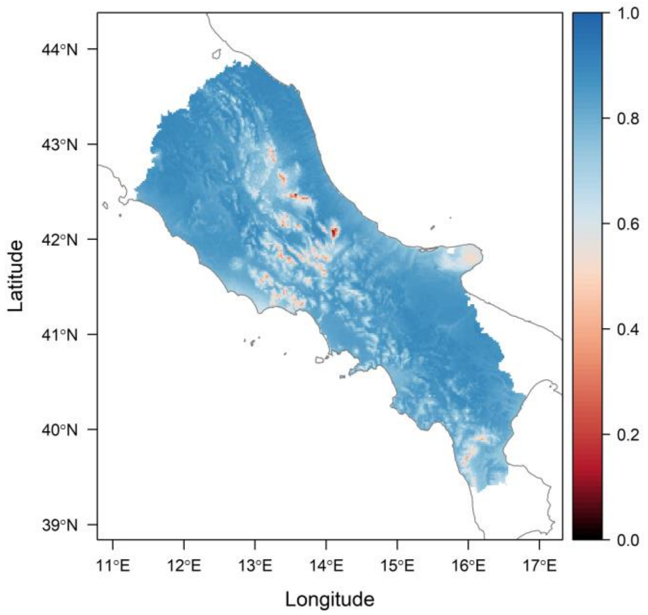

Galium magellense Ten.		
**Range of Values**	**Number of Cells**	**Perc (%)**
0.0-0.1	11	0.01
0.1-0.2	29	0.02
0.2-0.3	62	0.05
0.3-0.4	297	0.22
0.4-0.5	864	0.63
0.5-0.6	3162	2.32
0.6-0.7	8297	6.09
0.7-0.8	28765	21.12
0.8-0.9	94740	69.55
0.9-1.0	0	0

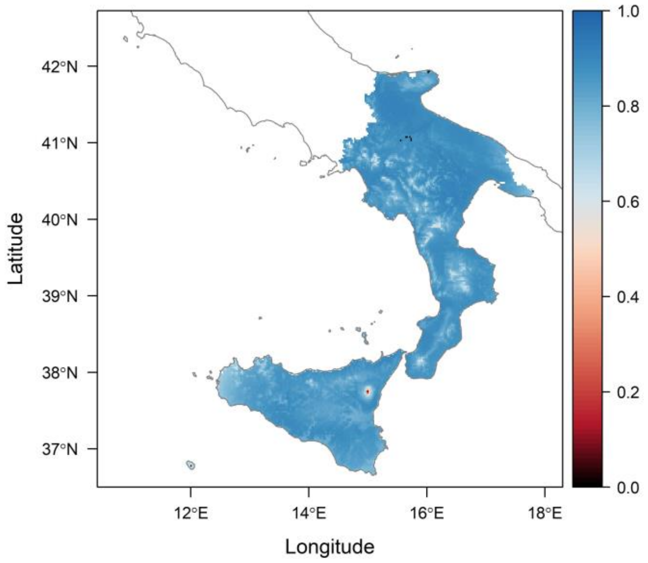

Gypsophila arrostoi Guss. subsp. *arrostoi*		
**Range of Values**	**Number of Cells**	**Perc (%)**
0.0-0.1	93	0.08
0.1-0.2	8	0.01
0.2-0.3	10	0.01
0.3-0.4	13	0.01
0.4-0.5	37	0.03
0.5-0.6	145	0.13
0.6-0.7	1241	1.13
0.7-0.8	9908	9
0.8-0.9	92353	83.84
0.9-1.0	6340	5.76

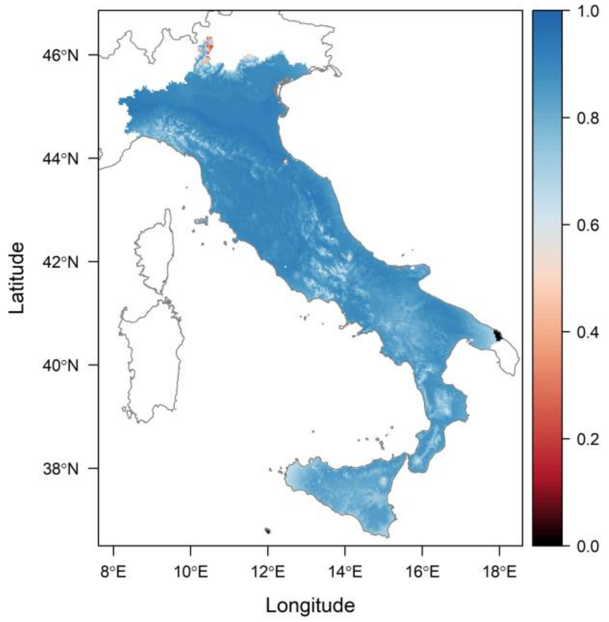

Helleborus viridis L. subsp. *bocconei* (Ten.) Peruzzi		
**Range of Values**	**Number of Cells**	**Perc (%)**
0.0-0.1	694	0.21
0.1-0.2	43	0.01
0.2-0.3	142	0.04
0.3-0.4	275	0.08
0.4-0.5	447	0.14
0.5-0.6	969	0.29
0.6-0.7	6006	1.82
0.7-0.8	40841	12.4
0.8-0.9	202146	61.38
0.9-1.0	77787	23.62

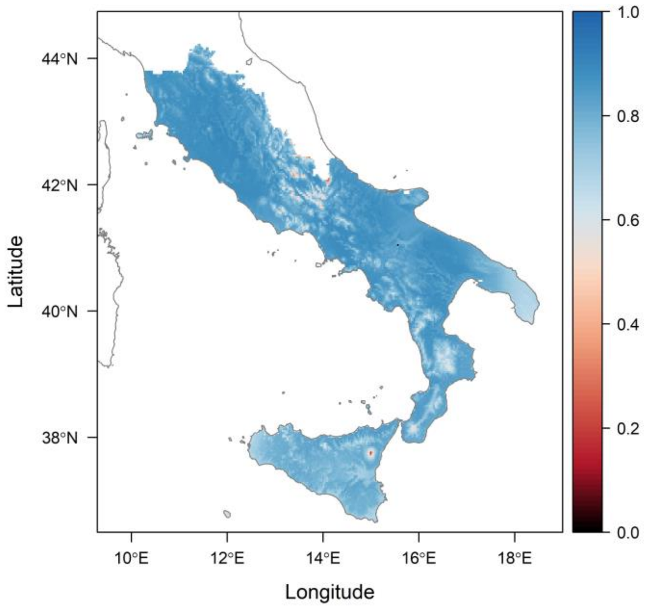

Klasea flavescens (L.) Holub subsp. *cichoracea* (L.) Greuter & Wagenitz		
**Range of Values**	**Number of Cells**	**Perc (%)**
0.0-0.1	32	0.01
0.1-0.2	9	0
0.2-0.3	37	0.02
0.3-0.4	83	0.04
0.4-0.5	354	0.16
0.5-0.6	1522	0.71
0.6-0.7	10990	5.1
0.7-0.8	53411	24.81
0.8-0.9	148623	69.02
0.9-1.0	258	0.12

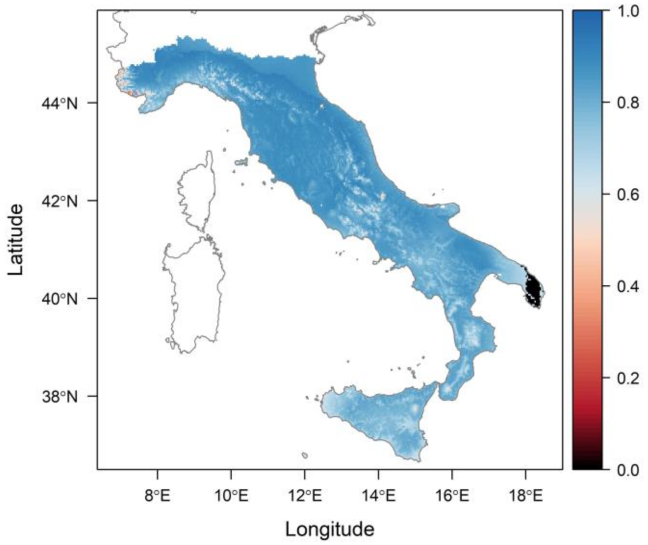

Koeleria splendens C.Presl		
**Range of Values**	**Number of Cells**	**Perc (%)**
0.0-0.1	3159	1.01
0.1-0.2	14	0
0.2-0.3	79	0.03
0.3-0.4	252	0.08
0.4-0.5	607	0.19
0.5-0.6	1916	0.61
0.6-0.7	14448	4.63
0.7-0.8	71339	22.87
0.8-0.9	205773	65.96
0.9-1.0	14369	4.61

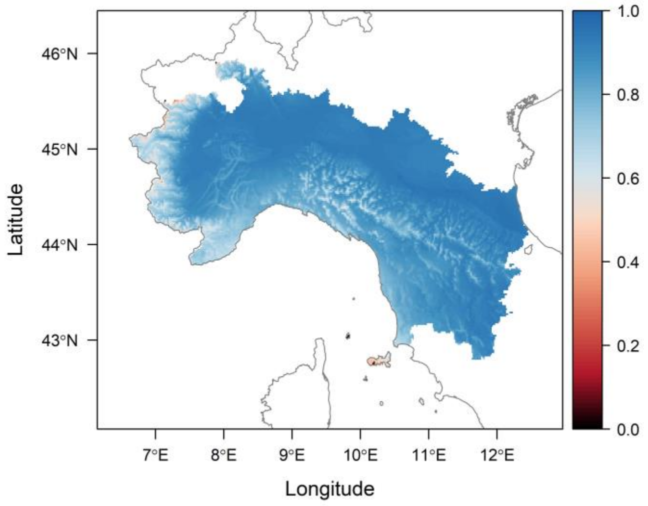

Leontodon anomalus Ball		
**Range of Values**	**Number of Cells**	**Perc (%)**
0.0-0.1	46	0.04
0.1-0.2	0	0
0.2-0.3	3	0
0.3-0.4	41	0.03
0.4-0.5	355	0.29
0.5-0.6	1356	1.12
0.6-0.7	6165	5.08
0.7-0.8	15186	12.52
0.8-0.9	42755	35.25
0.9-1.0	55384	45.66

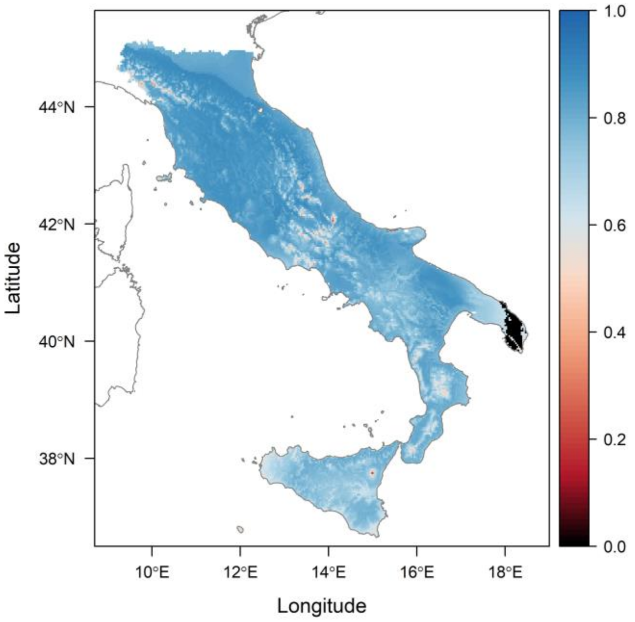

Linaria purpurea (L.) Mill.		
**Range of Values**	**Number of Cells**	**Perc (%)**
0.0-0.1	2966	1.06
0.1-0.2	11	0
0.2-0.3	37	0.01
0.3-0.4	165	0.06
0.4-0.5	865	0.31
0.5-0.6	4323	1.55
0.6-0.7	21477	7.7
0.7-0.8	88494	31.75
0.8-0.9	160399	57.54
0.9-1.0	6	0

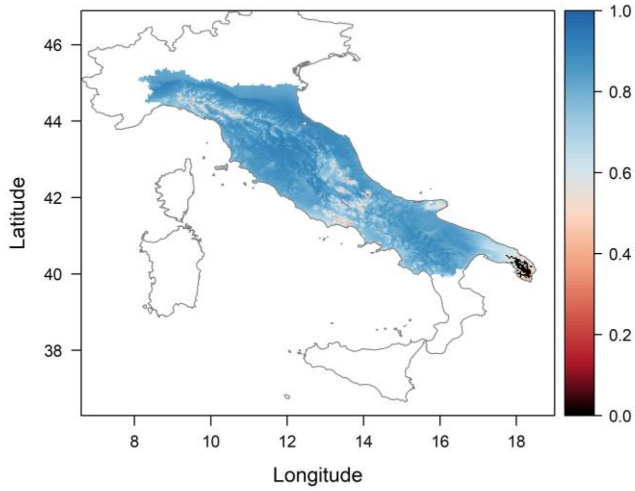

Ornithogalum etruscum Parl. subsp. *etruscum*		
**Range of Values**	**Number of Cells**	**Perc (%)**
0.0-0.1	2024	0.87
0.1-0.2	30	0.01
0.2-0.3	61	0.03
0.3-0.4	359	0.15
0.4-0.5	1937	0.83
0.5-0.6	5986	2.56
0.6-0.7	15300	6.55
0.7-0.8	48428	20.73
0.8-0.9	146225	62.61
0.9-1.0	13212	5.66

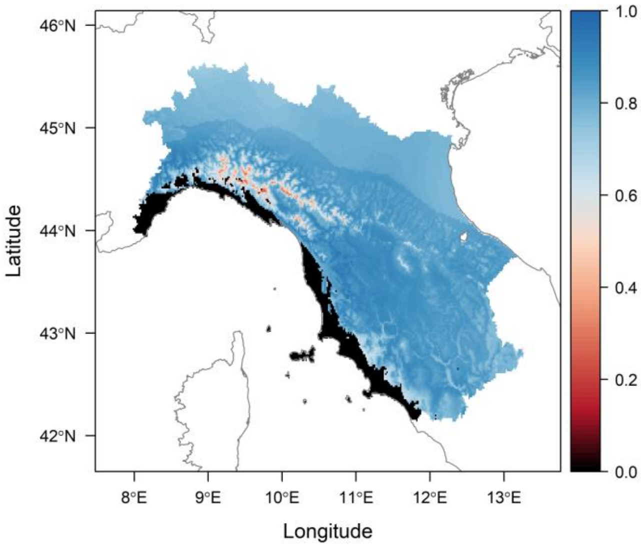

Phyteuma ovatum Honck. subsp. *pseudospicatum* Pignatti		
**Range of Values**	**Number of Cells**	**Perc (%)**
0.0-0.1	10259	8.21
0.1-0.2	0	0
0.2-0.3	64	0.05
0.3-0.4	238	0.19
0.4-0.5	544	0.44
0.5-0.6	1264	1.01
0.6-0.7	3501	2.8
0.7-0.8	35880	28.71
0.8-0.9	70083	56.07
0.9-1.0	3159	2.53

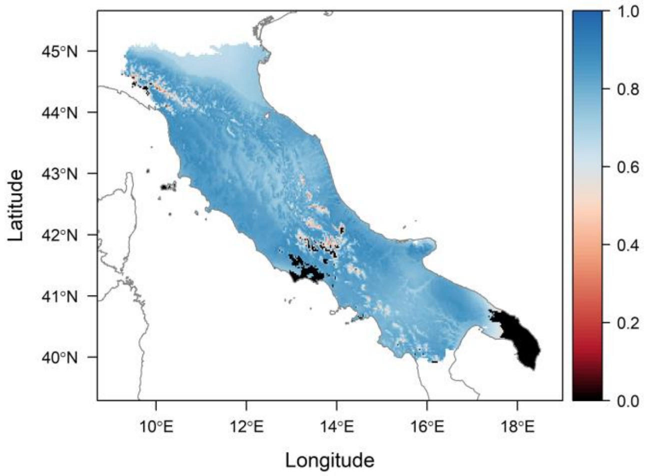

Polygala flavescens DC. subsp. *flavescens*		
**Range of Values**	**Number of Cells**	**Perc (%)**
0.0-0.1	10447	4.72
0.1-0.2	2	0
0.2-0.3	55	0.02
0.3-0.4	437	0.2
0.4-0.5	1433	0.65
0.5-0.6	3532	1.6
0.6-0.7	18680	8.44
0.7-0.8	73227	33.1
0.8-0.9	113155	51.15
0.9-1.0	233	0.11

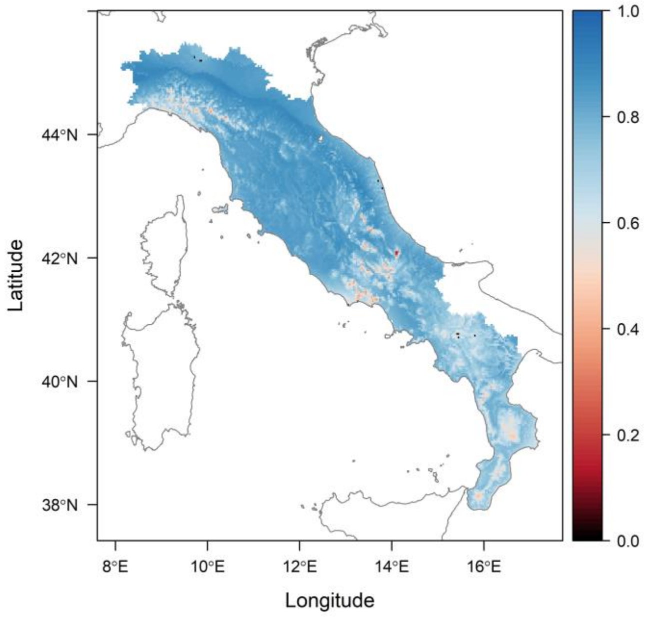

Ranunculus pollinensis (N.Terracc.) Chiov.		
**Range of Values**	**Number of Cells**	**Perc (%)**
0.0-0.1	205	0.09
0.1-0.2	26	0.01
0.2-0.3	43	0.02
0.3-0.4	403	0.17
0.4-0.5	1602	0.7
0.5-0.6	6538	2.84
0.6-0.7	26827	11.65
0.7-0.8	69507	30.18
0.8-0.9	124110	53.89
0.9-1.0	1037	0.45

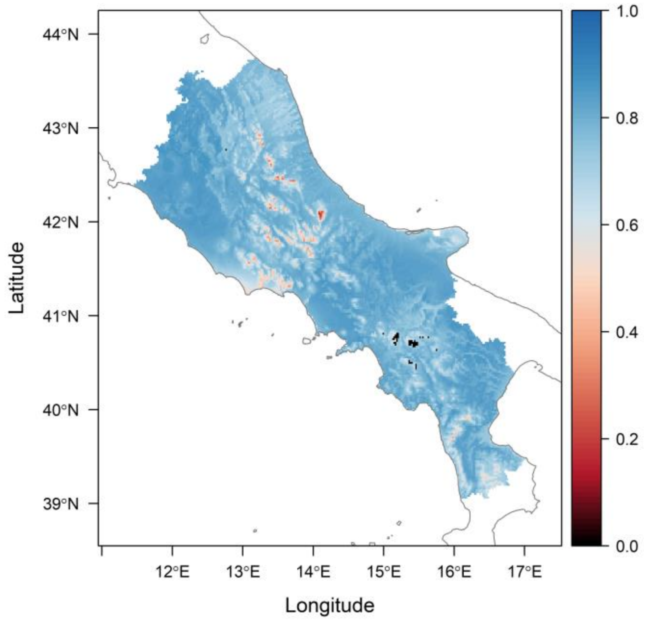

Saxifraga porophylla Bertol. subsp. *porophylla*		
**Range of Values**	**Number of Cells**	**Perc (%)**
0.0-0.1	234	0.18
0.1-0.2	26	0.02
0.2-0.3	50	0.04
0.3-0.4	266	0.2
0.4-0.5	822	0.62
0.5-0.6	2676	2.03
0.6-0.7	11216	8.5
0.7-0.8	60683	45.99
0.8-0.9	55977	42.42
0.9-1.0	0	0

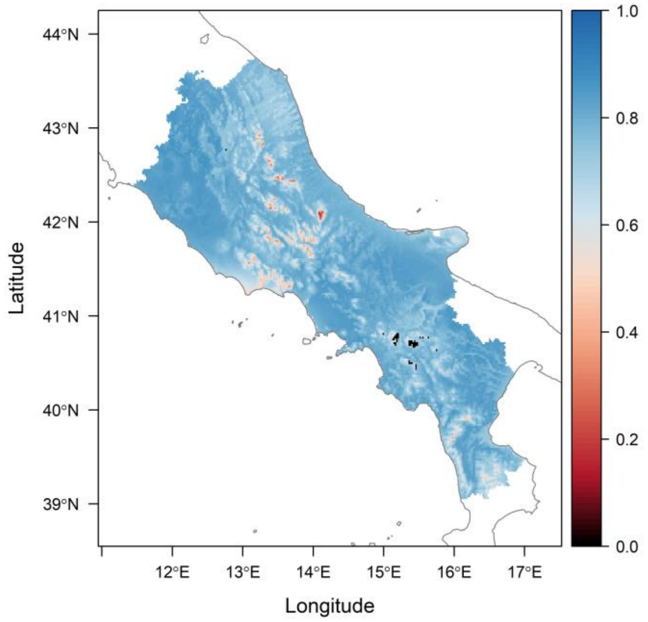

Sedum magellense Ten. subsp. *magellense*		
**Range of Values**	**Number of Cells**	**Perc (%)**
0.0-0.1	234	0.18
0.1-0.2	26	0.02
0.2-0.3	50	0.04
0.3-0.4	266	0.2
0.4-0.5	822	0.62
0.5-0.6	2676	2.03
0.6-0.7	11216	8.5
0.7-0.8	60683	45.99
0.8-0.9	55977	42.42
0.9-1.0	0	0

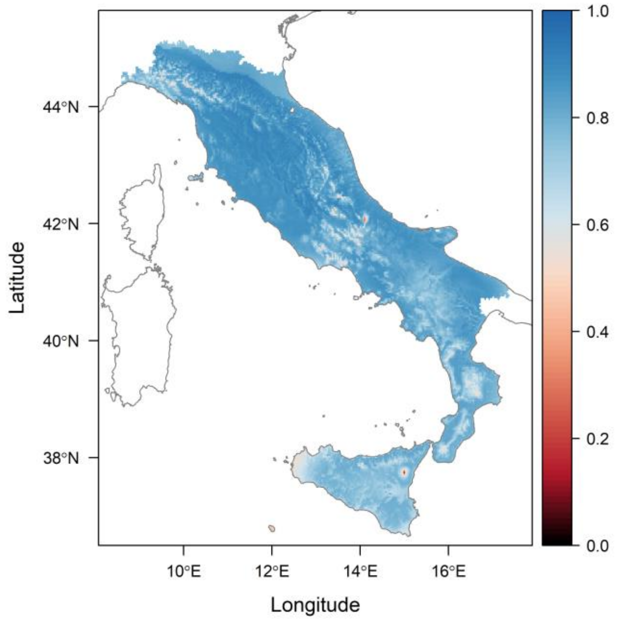

Siler montanum Crantz subsp. *siculum* (Spreng.) Iamonico, Bartolucci & F.Conti		
**Range of Values**	**Number of Cells**	**Perc (%)**
0.0-0.1	2	0
0.1-0.2	8	0
0.2-0.3	28	0.01
0.3-0.4	55	0.02
0.4-0.5	564	0.21
0.5-0.6	3534	1.32
0.6-0.7	14935	5.57
0.7-0.8	77864	29.04
0.8-0.9	163037	60.8
0.9-1.0	8136	3.03

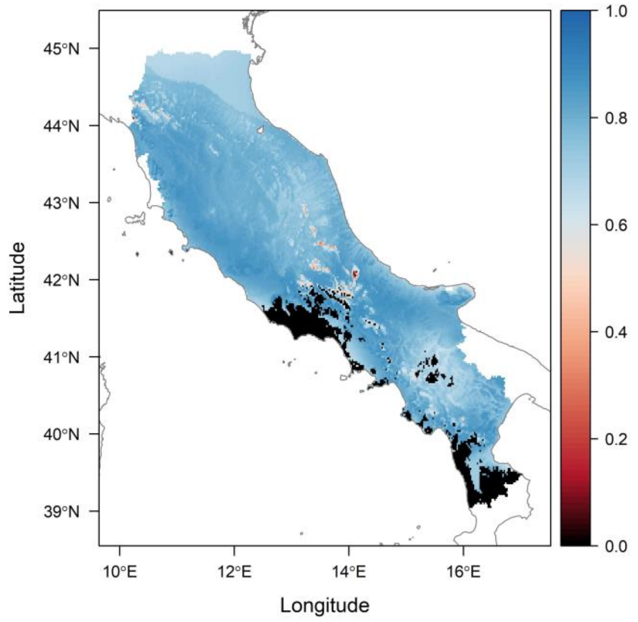

Viola eugeniae Parl. subsp. *eugeniae*		
**Range of Values**	**Number of Cells**	**Perc (%)**
0.0-0.1	15939	8.14
0.1-0.2	18	0.01
0.2-0.3	42	0.02
0.3-0.4	151	0.08
0.4-0.5	458	0.23
0.5-0.6	1437	0.73
0.6-0.7	16251	8.3
0.7-0.8	81715	41.74
0.8-0.9	79694	40.71
0.9-1.0	57	0.03

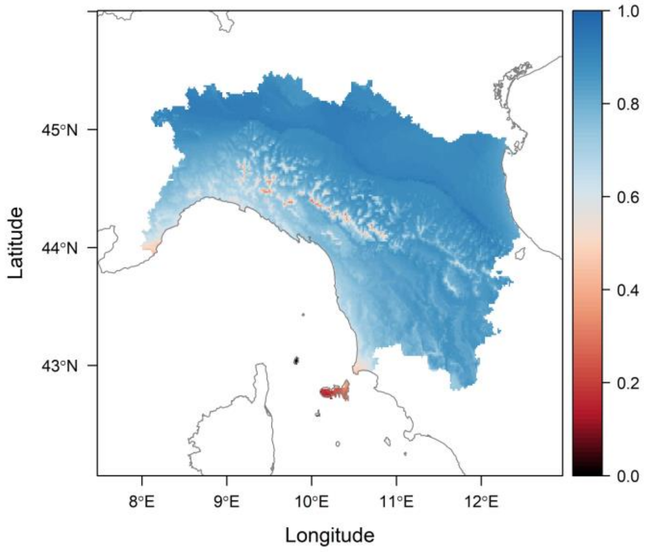

Viola ferrarinii Moraldo & Ricceri		
**Range of Values**	**Number of Cells**	**Perc (%)**
0.0-0.1	45	0.05
0.1-0.2	143	0.16
0.2-0.3	177	0.2
0.3-0.4	160	0.18
0.4-0.5	449	0.5
0.5-0.6	1455	1.61
0.6-0.7	6300	6.95
0.7-0.8	16938	18.69
0.8-0.9	50734	55.99
0.9-1.0	14207	15.68
