# Supplementary material for: Assessing the Effectiveness of Correlative Ecological Niche Model Temporal Projection through Floristic Data
Source: Biology (Basel). 2022 Aug 14;11(8):1219. doi: 10.3390/biology11081219 (PMC9405103; doi:10.3390/biology11081219)
Supplement: Supplementary file 1 [file biology-11-01219-s001.zip › Dolci & Peruzzi Supplementary Material S.1-9.zip!/Supplementary material S.9.pdf]

Supplementary material S.9

Summary results of all modeling procedures obtained by using 3 selected bioclimatic variables and the first three axes of a principal component analysis (PCA) of the 35 bioclimatic variables.

Bioclim

Statistic	Var.	Mean	Median	SD
Distance	3 var.	68.870	74.265	16.861
Range Size variation	3 var.	-0.211	-0.201	1.555
Stability	3 var.	31.147	25.737	16.857
D-Index	3 var.	0.705	0.756	0.175
CBI Projected	3 var.	0.550	0.623	0.341
CBI Present	3 var.	0.502	0.549	0.318
Distance	35 var.	75.490	77.544	13.909
Range Size variation	35 var.	0.663	0.326	1.980
Stability	35 var.	24.536	22.456	13.892
D-Index	35 var.	0.619	0.664	0.163
CBI Projected	35 var.	0.533	0.553	0.240
CBI Present	35 var.	0.467	0.478	0.339

Biomod2

Statistic	Var.	Mean	Median	SD
Distance	3 var.	69.383	71.118	19.937
Range Size variation	3 var.	-0.390	-0.186	1.740
Stability	3 var.	30.640	28.955	19.936
D-Index	3 var.	0.703	0.719	0.097
CBI Projected	3 var.	0.804	0.871	0.197
CBI Present	3 var.	0.928	0.931	0.074
Distance	35 var.	68.399	64.441	20.478
Range Size variation	35 var.	0.216	0.085	1.776
Stability	35 var.	31.623	35.587	20.475
D-Index	35 var.	0.714	0.712	0.077
CBI Projected	35 var.	0.708	0.783	0.311
CBI Present	35 var.	0.941	0.960	0.063

CTA

Statistic	Var.	Mean	Median	SD
Distance	3 var.	67.202	65.540	22.402
Range Size variation	3 var.	-0.810	-0.657	2.230
Stability	3 var.	32.841	34.480	22.408
D-Index	3 var.	0.712	0.726	0.110
CBI Projected	3 var.	0.740	0.827	0.258
CBI Present	3 var.	0.898	0.928	0.092
Distance	35 var.	65.614	61.724	21.634
Range Size variation	35 var.	-0.516	-0.289	1.982
Stability	35 var.	34.413	38.277	21.620
D-Index	35 var.	0.734	0.747	0.091
CBI Projected	35 var.	0.704	0.826	0.292
CBI Present	35 var.	0.917	0.949	0.071

Domain

Statistic	Var.	Mean	Median	SD
Distance	3 var.	75.659	77.094	14.750
Range Size variation	3 var.	0.585	0.172	1.869
Stability	3 var.	24.363	22.922	14.736
D-Index	3 var.	0.884	0.925	0.131
CBI Projected	3 var.	0.743	0.868	0.315
CBI Present	3 var.	0.831	0.908	0.204
Distance	35 var.	77.425	78.611	13.406
Range Size variation	35 var.	-0.004	0.011	3.042
Stability	35 var.	22.626	21.390	13.366
D-Index	35 var.	0.835	0.875	0.142
CBI Projected	35 var.	0.637	0.839	0.442
CBI Present	35 var.	0.814	0.917	0.285

FDA

Statistic	Var.	Mean	Median	SD
Distance	3 var.	53.905	50.456	24.825
Range Size variation	3 var.	-0.324	-0.172	1.371
Stability	3 var.	46.116	49.546	24.832
D-Index	3 var.	0.851	0.855	0.081
CBI Projected	3 var.	0.786	0.874	0.212
CBI Present	3 var.	0.806	0.873	0.150
Distance	35 var.	55.009	53.780	23.327
Range Size variation	35 var.	0.227	0.193	1.177
Stability	35 var.	45.007	46.247	23.333
D-Index	35 var.	0.833	0.839	0.081
CBI Projected	35 var.	0.646	0.847	0.437
CBI Present	35 var.	0.759	0.853	0.259

GAM

Statistic	Var.	Mean	Median	SD
Distance	3 var.	82.741	82.857	7.096
Range Size variation	3 var.	0.842	0.427	2.041
Stability	3 var.	17.287	17.196	7.084
D-Index	3 var.	0.751	0.764	0.079
CBI Projected	3 var.	0.796	0.848	0.174
CBI Present	3 var.	0.867	0.873	0.096
Distance	35 var.	79.260	78.428	12.315
Range Size variation	35 var.	0.221	0.323	2.141
Stability	35 var.	20.770	21.573	12.323
D-Index	35 var.	0.741	0.755	0.086
CBI Projected	35 var.	0.706	0.818	0.314
CBI Present	35 var.	0.887	0.901	0.094

GLM

Statistic	Var.	Mean	Median	SD
Distance	3 var.	64.354	57.943	14.149
Range Size variation	3 var.	0.409	0.354	1.301
Stability	3 var.	35.660	42.086	14.148
D-Index	3 var.	0.861	0.867	0.068
CBI Projected	3 var.	0.773	0.873	0.248
CBI Present	3 var.	0.823	0.865	0.149
Distance	35 var.	63.287	60.449	15.569
Range Size variation	35 var.	-0.222	0.348	1.769
Stability	35 var.	36.739	39.553	15.576
D-Index	35 var.	0.863	0.883	0.080
CBI Projected	35 var.	0.634	0.902	0.445
CBI Present	35 var.	0.806	0.918	0.221

KDE

Statistic	Var.	Mean	Median	SD
Distance	3 var.	84.198	85.567	12.323
Range Size variation	3 var.	0.271	-0.079	2.243
Stability	3 var.	15.829	14.436	12.306
D-Index	3 var.	0.829	0.887	0.167
CBI Projected	3 var.	0.682	0.762	0.318
CBI Present	3 var.	0.883	0.911	0.140
Distance	35 var.	82.073	82.944	12.126
Range Size variation	35 var.	0.383	-0.051	2.503
Stability	35 var.	17.963	17.056	12.114
D-Index	35 var.	0.786	0.817	0.123
CBI Projected	35 var.	0.736	0.832	0.320
CBI Present	35 var.	0.881	0.939	0.197

MARS

Statistic	Var.	Mean	Median	SD
Distance	3 var.	91.862	93.028	4.114
Range Size variation	3 var.	-0.543	-0.427	1.654
Stability	3 var.	8.154	6.972	4.114
D-Index	3 var.	0.691	0.693	0.110
CBI Projected	3 var.	0.634	0.734	0.399
CBI Present	3 var.	0.844	0.836	0.094
Distance	35 var.	92.396	93.068	3.918
Range Size variation	35 var.	-0.873	-0.409	2.316
Stability	35 var.	7.636	6.976	3.893
D-Index	35 var.	0.649	0.662	0.121
CBI Projected	35 var.	0.578	0.733	0.362
CBI Present	35 var.	0.832	0.851	0.099

Maxent

Statistic	Var.	Mean	Median	SD
Distance	3 var.	60.808	59.378	17.459
Range Size variation	3 var.	0.162	0.068	1.395
Stability	3 var.	39.209	40.623	17.462
D-Index	3 var.	0.841	0.845	0.076
CBI Projected	3 var.	0.827	0.918	0.195
CBI Present	3 var.	0.902	0.949	0.088
Distance	35 var.	65.317	70.709	20.517
Range Size variation	35 var.	-0.164	-0.019	1.916
Stability	35 var.	34.708	29.302	20.509
D-Index	35 var.	0.778	0.813	0.097
CBI Projected	35 var.	0.726	0.876	0.364
CBI Present	35 var.	0.902	0.934	0.104

RF

Statistic	Var.	Mean	Median	SD
Distance	3 var.	64.108	63.931	23.342
Range Size variation	3 var.	0.130	-0.033	1.577
Stability	3 var.	35.914	36.074	23.349
D-Index	3 var.	0.790	0.790	0.050
CBI Projected	3 var.	0.853	0.928	0.144
CBI Present	3 var.	0.954	0.966	0.045
Distance	35 var.	68.619	75.846	18.946
Range Size variation	35 var.	0.100	-0.273	1.952
Stability	35 var.	31.404	24.156	18.929
D-Index	35 var.	0.783	0.797	0.078
CBI Projected	35 var.	0.783	0.902	0.287
CBI Present	35 var.	0.962	0.966	0.028

SVM

Statistic	Var.	Mean	Median	SD
Distance	3 var.	56.245	52.537	25.126
Range Size variation	3 var.	0.168	-0.215	1.436
Stability	3 var.	43.775	47.471	25.131
D-Index	3 var.	0.790	0.809	0.101
CBI Projected	3 var.	0.834	0.900	0.163
CBI Present	3 var.	0.897	0.903	0.048
Distance	35 var.	63.981	67.216	26.011
Range Size variation	35 var.	-0.488	-0.406	2.115
Stability	35 var.	36.049	32.786	25.996
D-Index	35 var.	0.754	0.788	0.135
CBI Projected	35 var.	0.764	0.900	0.306
CBI Present	35 var.	0.938	0.945	0.040
